# Expression of the RNA-binding protein RBP10 promotes the bloodstream-form differentiation state in *Trypanosoma brucei*

**DOI:** 10.1371/journal.ppat.1006560

**Published:** 2017-08-11

**Authors:** Elisha Mugo, Christine Clayton

**Affiliations:** DKFZ-ZMBH Alliance, Zentrum für Molekulare Biologie der Universität Heidelberg, University of Heidelberg, Heidelberg, Germany; University at Buffalo, UNITED STATES

## Abstract

In nearly all eukaryotes, cellular differentiation is governed by changes in transcription, and stabilized by chromatin and DNA modification. Gene expression control in the pathogen *Trypanosoma brucei*, in contrast, relies almost exclusively on post-transcriptional mechanisms, so RNA binding proteins must assume the burden that is usually borne by transcription factors. *T*. *brucei* multiply in the blood of mammals as bloodstream forms, and in the midgut of Tsetse flies as procyclic forms. We show here that a single RNA-binding protein, RBP10, promotes the bloodstream-form trypanosome differentiation state. Depletion of RBP10 from bloodstream-form trypanosomes gives cells that can grow only as procyclic forms; conversely, expression of RBP10 in procyclic forms converts them to bloodstream forms. RBP10 binds to procyclic-specific mRNAs containing an UAUUUUUU motif, targeting them for translation repression and destruction. Products of RBP10 target mRNAs include not only the major procyclic surface protein and enzymes of energy metabolism, but also protein kinases and stage-specific RNA-binding proteins: this suggests that alterations in RBP10 trigger a regulatory cascade.

## Author summary

African trypanosomes cause major economic losses in Africa and other tropical countries through infection of cattle and other domestic animals. In addition, infection of humans results in sleeping sickness, which is usually lethal. Within mammals, the parasites multiply as "bloodstream forms" in the blood and tissue fluids. The trypanosomes also infect African Tsetse flies, multiplying in the midgut as "procyclic forms". Adaptation to these two different environments requires that different RNAs and proteins are made. In this paper we show that one particular protein, RBP10, binds to RNAs that are needed only in the procyclic form. RBP10 is made exclusively in bloodstream form parasites; when it binds to its target RNAs, it prevents them being translated into protein and also causes destruction of the attached RNA. When we drastically reduced the amount of RBP10 in bloodstream forms, they died unless they were placed under conditions suitable for procyclic forms—in which case they turned into procyclic forms and started to grow again. Conversely, when we artificially turned on synthesis of RBP10 in procyclic forms, the parasites could only survive and grow if they were placed in bloodstream form conditions. The presence and absence of RBP10 therefore acts as a regulatory switch.

## Introduction

In multicellular organisms, cell differentiation is usually unidirectional, with stabilisation of differentiated states by epigenetic mechanisms, and reversal only when healthy gene regulation is disrupted. In contrast, differentiation in unicellular organisms can be reversible, or involve cycles in which the direction of each transition is fixed. In each case differentiation is initiated by environmental stimuli, which activate signal transduction cascades; these in turn result in post-translational modifications that modify transcription factor activity. The consequent changes in mRNA synthesis, and hence protein synthesis, trigger a cascade of downstream regulatory events, affecting additional signalling molecules, transcription factors, and post-transcriptional modulators. Differentiation states are then stabilised by modification of DNA and chromatin.

Kinetoplastids are unicellular flagellates, and include many pathogens of mammals and plants, including the leishmanias and trypanosomes that cause human disease. Their life cycles involve a series of unidirectional transitions, which are driven by environmental changes as they move between mammalian and invertebrate hosts. The kinetoplastid studied in this paper, *Trypanosoma brucei*, causes human sleeping sickness in Africa [1] and diseases of ruminants and ungulates throughout the tropics [2–4]. *T*. *brucei* multiply as bloodstream form trypomastigotes in the blood and tissue fluids of mammals, escaping immunity by antigenic variation of Variant Surface Glycoprotein (VSG) [5]. High cell density triggers growth arrest and differentiation to stumpy forms [6], which, upon uptake by a Tsetse fly, convert to proliferative procyclic forms in the midgut. Prolonged growth in the laboratory diminishes this capability, giving rise to "monomorphic" lines with defective differentiation [6]. Procyclic forms express EP and GPEET procyclin surface proteins [7], and have numerous metabolic adaptations including increased mitochondrial metabolism. Stage-specific expression of metabolic enzymes has been shown to be essential for survival of both bloodstream and procyclic forms [8, 9]. In the following 2 weeks, trypanosomes lose GPEET expression [10], and migrate towards the salivary glands [11]. Epimastigotes, many of which express the surface protein BARP [12], are found attached to the salivary gland epithelia; here, a subset of parasites undergoes meiosis and mating [13, 14]. Finally, growth-arrested, VSG-expressing metacyclic forms are formed; these can infect of a new mammal. Trypanosome developmental transitions are marked by many changes in mRNA [15–19] and protein [20–23] abundance.

Despite extensive changes in gene expression through the *T*. *brucei* life cycle, and the existence of stable differentiation states, the normal paradigm for regulation does not apply. This is because—as in other Kinetoplastid protists—transcription by *T*. *brucei* RNA polymerase II is polycistronic [24], with no evidence for regulation at the level of individual mRNAs. Monocistronic mRNAs are excised by 5' *trans* splicing of a 39nt capped leader sequence, and by 3' polyadenylation [25, 26]. Differential regulation is thus exclusively at the levels of mRNA processing, degradation, and translation [17–19, 27–30]. The sequences required for regulation of translation and mRNA decay often lie in the 3'-untranslated regions (3'-UTRs) of the mRNAs. Many groups of trypanosome mRNAs are coordinately regulated [31] and in a few cases, co-regulated genes have been shown to share regulatory elements that are bound by specific RNA-binding proteins [32, 33]. RNA-binding proteins thus assume the burden that is carried by transcription factors in other eukaryotes, by regulating distinct mRNA subsets [27, 34]. Although an RNAi machinery exists, it is not essential for cell proliferation or differentiation [35], and microRNAs have not been found [36].

The very abundant *VSG* and procyclin mRNAs are made, exceptionally, by RNA polymerase I [37], and their transcription is developmentally regulated by epigenetic mechanisms [5]. Procyclin is not subject to antigenic variation, and its presence on the bloodstream-form surface might enable the development of host immunity, or impair the integrity of the VSG coat. Suppression of procyclin expression in bloodstream forms is therefore thought to be important for trypanosome pathogenicity. However, procyclin gene transcription is only 10-fold less active in bloodstream forms than in procyclic forms [38]. This means that additional suppressive mechanisms are required: the mRNAs are both extremely rapidly degraded and very poorly translated [39–43], and trafficking of procyclin proteins to the surface is suppressed [44]. A 26nt motif within the 3'-UTR of procyclin mRNAs is required for their degradation and translational suppression [41, 42]. However, until now, the protein(s) responsible for preventing procyclin expression in bloodstream forms were unknown.

RBP10 (Tb927.8.2780) is a cytosolic protein with a single RNA recognition motif. Proteome analyses [23] and Western blot results [45] indicate that RBP10 is expressed in proliferating bloodstream forms, but not in stumpy forms or procyclics. *RBP10* mRNA has correspondingly low abundance in procyclic forms [45] and possibly also in salivary gland trypanosomes [46]. We previously found that both depletion of RBP10 by RNA interference (RNAi) in the bloodstream form, and forced RBP10 expression in procyclics, caused strong growth inhibition [45]. Microarray analyses on the growth-inhibited cells suggested that RBP10 expression correlated with expression of enzymes of bloodstream-form-type energy metabolism, and repression of mitochondrial pathways. Unfortunately, however, it was not clear whether the gene expression changes were due to RBP10 itself, or to growth arrest, since many of the repressed mRNAs were implicated in cell proliferation. To find out whether RBP10 can post-transcriptionally repress, or increase gene expression, we expressed a fusion of RBP10 with the lambdaN peptide, in cells expressing a reporter mRNA with five boxB motifs. The boxB motif binds to the lambda N peptide with high affinity, thus "tethering" any lambdaN fusion protein to the reporter mRNA. Tethering of RBP10 resulted in strong repression of reporter mRNA and protein [45, 47, 48]. The biological relevance of this was however questionable since we could find no evidence that RBP10 could bind to mRNAs *in vivo* [45].

Recently, Kolev et al. [49] showed that procyclic forms can be pushed, by an unknown mechanism, towards epimastigote, then metacyclic form differentiation through expression of an RRM-domain protein called RBP6. This, together with the numerous doubts left by our previous investigation, prompted us to re-visit both the role of RBP10 in differentiation, and its mode of action. We here show that the presence and absence of RBP10 acts as an on/off switch for the ability of trypanosomes to grow as long slender bloodstream-forms; results of transcriptome analyses suggest that RBP10 may be at the apex of a post-transcriptional regulatory cascade.

## Results

### Attachment of the RBP10 C-terminal domain to a reporter mRNA causes translation inhibition and mRNA degradation

**Fig 1:**
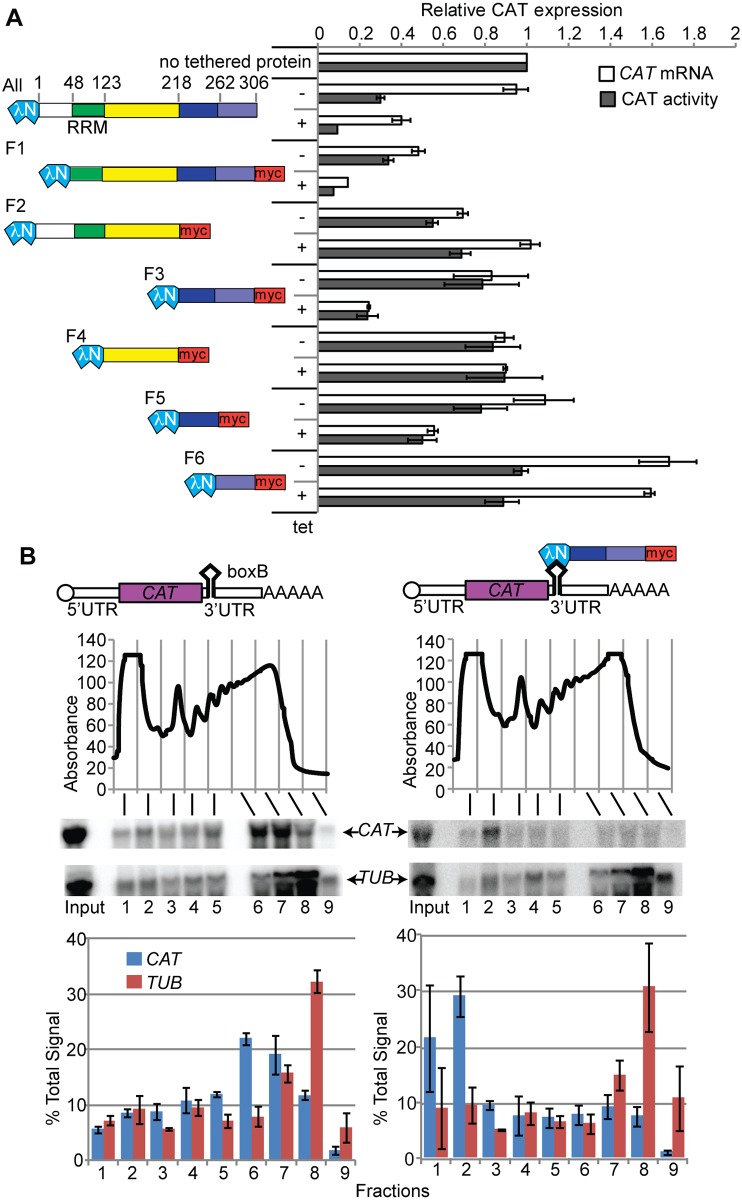
Tethering of the C-terminal portion of RBP10 to a reporter mRNA inhibits its translation and causes its destruction. A. Various fragments of RBP10 were inducibly expressed with an N-terminal lambdaN peptide and a C-terminal myc tag, in a cell line expressing a *CAT* reporter mRNA with 5 boxB sequences in the 3'-UTR. Cartoons showing the fragments are on the left. The numbers above the full-length protein are amino acid residues. The effects on the amounts *CAT* mRNA and CAT protein were measured by Northern blot and enzyme assay, respectively. Levels are expressed as mean ± standard deviation for at least 3 replicates, relative to a line with no lambdaN peptide protein (top bars). B. Bloodstream-form trypanosomes expressing the reporter in (A), and with tetracycline-inducible expression of lamdaN-RBP10-F3, were used. The reporter with or without tethered protein is shown at the top. Lysates from cells grown without (left) or with (right) tetracycline were subjected to sucrose gradient centrifugation. The panel below the cartoons show the absorption profiles at 254 nm as the fractions were collected. The migration of *CAT* and beta-tubulin (*TUB*) mRNAs on the gradient was detected by Northern blotting; representative blots are shown. The graphs show Northern signals, expressed as the percentage of the total signal, with arithmetic mean and standard deviation for three independent biological replicates.

In our initial experiments we set out to try to understand the mechanism by which RBP10 can influence gene expression. These experiments were done with monomorphic Lister 427 bloodstream forms. We had previously used cells expressing a chloramphenicol acetyltransferase (*CAT*) mRNA with boxB motifs. We found that after 24h lambdaN-RBP10 induction, there was a decrease in *CAT*-boxB reporter expression [45, 47, 48]. RBP10 is a 306-residue protein, and the RRM domain is towards the N-terminus (Fig 1A). Deletion analyses (Fig 1) revealed that the C-terminal 88 amino acids of lambdaN-RBP10 were sufficient for the repressive effect (Fig 1A, fragment F3) whereas N-terminal fragments, including the RRM, had no activity. Smaller C-terminal fragments were less effective than the 88-residue portion, but this could have been due to lower expression; all were expressed (S1A Fig) but levels were not strictly compared. As seen previously, expression of lambdaN-RBP10 decreased expression of endogenous RBP10 ([45, 47, 48]; S1A Fig); we did not investigate this further.

In our previous tethering experiments, CAT activity was decreased much more than *CAT* mRNA. We therefore suggested that RBP10 might suppress translation [45, 47, 48]. To investigate this in more detail we examined where the *CAT-boxB* mRNA co-migrated with polysomes in sucrose gradients. In the absence of tethered protein, the *CAT-boxB* reporter migrated in the dense sucrose fraction with large polysomes (Fig 1B). With lambdaN-RBP10-F3 expression, *CAT-boxB* mRNA was reduced in abundance and the remainder migrated towards the top of the gradient. The control mRNA (encoding alpha-tubulin) was unaffected (Fig 1B). The RBP10 C-terminus thus prevents translation initiation and promotes mRNA destruction.

To see whether the function of RBP10 might be conserved in other kinetoplastid organisms, we compared the sequences (S2 Fig). The RRM domain is conserved in all species examined. There are 7 mapped phosphorylation sites immediately downstream of the RRM; these are with one exception conserved only within *Trypanosoma*. The *Crithidia*, *Leptomonas*, *Endotrypanum*, and *Leishmania* sequences are extended at both the N- and C- termini, where sequence similarity to the *Trypanosoma* sequences is much lower (S2 Fig). There are however some small partially conserved patches within the C-termini, which might result in similar chemical properties. We did not find a homologue of RBP10 in *Bodo caudatus* in Genbank, but the result is not definitive since the *Bodo* genome is presumably not complete.

To investigate in more detail how RBP10 suppresses translation, we made Lister 427 bloodstream forms in which one *RBP10* allele had been knocked out and the other had been modified so that the expressed RBP10 had an N-terminal tandem affinity purification (TAP) tag. The TAP-RBP10 was functional since the cells grew normally (S1B Fig). We subjected six affinity-purified preparations to mass spectrometry—three with RNase, and three without. TAP-tagged GFP served as a negative control. Since the numbers of peptides found in the different experiments were quite variable, to select interaction partners we chose only proteins in which lowest obtained peptide count from the RBP10 purifications was at least twice the highest count from the GFP controls. We also excluded proteins that had been found in more than 6 other previous purifications. By these criteria, 16 proteins (apart from RBP10) were selectively enriched in all purifications (S1 Table, sheet 1). They included six proteasome regulator subunits, of which three were recently also found to be specifically modified by BirA*-tagged RBP10 in procyclic forms [50]. Three additional proteasome regulatory subunits were enriched only in the samples with RNase. The RNA-binding proteins ALBA1 and UBP2 were in both our pull-downs and the BirA* dataset, as was an armadillo repeat protein, Tb927.1.1670. CAF40, a NOT-complex subunit, was enriched in our pull-downs; it was modified by BirA*-RBP10, but also by the BirA*-GFP control [50]. We also detected NOT1 and DHH1 but in our experience these latter proteins are frequent contaminants. The remaining NOT complex proteins, including CAF1, were absent although usually, the components reproducibly co-purify [51, 52]. The RNA-binding proteins DRBD18 (also found using BirA*-RBP10) and DRBD4 were found only in the absence of RNase.

To find direct binding partners we also did a two-hybrid screen with a bait library of trypanosome protein fragments. This yielded nearly 100 potential partners (S1 Table, sheet 2), suggesting rather promiscuous interactions, but none of the 16 most reliably associated proteins from the tandem affinity purification was included. Since some of the two-hybrid interactions could have been affected by poor folding of bait fragments we also did some pair-wise assays with complete open reading frames. We found no interaction between RBP10 bait and various translation factors as prey: EF1alpha, EF2, eRF1,eRF3, eIF5a and eIF2B (S3A and S3B Fig). We also followed up some of the 2-hybrid fragment screening results. RBP10 as prey had interactions with baits ZC3H22, DRBD12 and Tb927.10.10050, but we discovered that all three of these were auto-activating in the bait configuration (S3C and S3D Fig); unsurprisingly, DRBD12 and Tb927.10.10050 we also detected in screens with CFB1 [53] and MKT1 [54]. However, RBP10 and RBP26 (2 positive fragments) interacted in both configurations without auto-activation (S3 Fig, section B). We therefore tested this interaction in trypanosomes, by expressing RBP10-myc in a cell line expressing endogenously V5-tagged RBP26. The results (S3E and S3F Fig) suggested that there might be *in vivo* interactions between the two proteins but if so, less than 1% of each was involved, which could explain why RBP26 was not detected by mass spectrometry. In bloodstream forms, some RBP26 is associated with mRNA [55], and RNAi targeting RBP26 caused a slight growth defect [56]; however tethering of RBP26 had no effect on the attached reporter [55].

The major conclusion from these studies is that RBP10 does not act as part of a stable complex and probably does not recruit the deadenylation machinery. It is possible that RBP10 recruits the proteasome. However, it is also possible that some of the recombinant RBP10 fusion protein is poorly folded, ubiquitinated and targeted to the proteasome.

### RBP10-bound mRNAs share a UAUUUUUU motif

**Fig 2:**
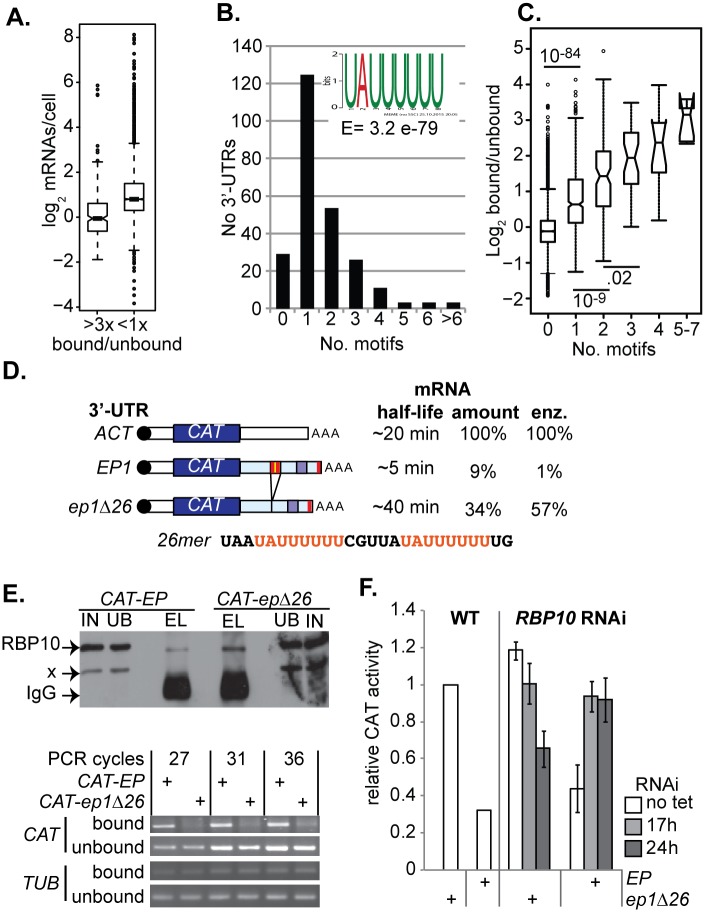
RBP10 targets a UAUUUUUU motif. A) Abundances of mRNAs that were at least 3x enriched in both RBP10-bound fraction, relative to those that were less than 1x enriched. B) The 3'-UTRs of 188 mRNAs that were at least 3x enriched in both RBP10-bound samples (Supplementary S2 Table) were compared with those of mRNAs that did not bind (<0.7x enrichment in both experiments) using DREME. Only 3'-UTRs annotated in tritrypDB were used (S1 and S2 Text) and some were not mapped with sufficient confidence to be included. The best-scoring motif found is shown in the inset. The graph shows numbers of UA(U)_6_ motifs in 255 manually annotated bound 3'-UTRs (S2 Table). One mRNA each had 7, 8 and 9 motifs. The numbers of motifs shown here are sometimes higher than with automatically annotated 3'-UTRs from the genome database, because some of the automatic 3'-UTRs are truncated. C) Effect of the UA(U)_6_ motif on RBP10 binding: median ± 25th percentiles, with 95% confidence limits and outliers. Database 3'-UTRs were analyzed. Since these are often truncated, and are missing for 30% of genes, the numbers of motifs are under-estimated. Significant differences (Student t-test) are shown. D) Reporters used in (E) and (F) [42]. The red bars are UAUUUUUU elements, which are highlighted in the 26mer sequence. E) RBP10 binding to the *EP* 3'-UTR requires the 26mer. Cells were UV-irradiated, and RBP10 was pulled down with anti-RBP10 (upper panel, Western blot). RNAs were detected by reverse transcription and PCR using gene-specific primers (lower panel). If reverse transcriptase was omitted, no PCR band was obtained. F) *rbp10* RNAi specifically affects expression of a reporter containing the 26mer. CAT activity was measured 17h and 24h after RNAi induction.

The action of lambdaN-RBP10 in a tethering assay does not necessarily reflect its function when it is bound to mRNAs via its own RNA recognition motif. To investigate this further we needed to know which mRNAs were targeted and regulated by RBP10. We were previously unable to identify any RBP10-bound mRNAs [45]. However, a subsequent proteomic study showed that RBP10 is indeed bound to mRNA [48]. We used the cell line expressing TAP-RBP10 (S1B Fig) in order to purify bound mRNAs, then compared bound and unbound mRNAs by RNASeq (S2 Table). A simple scatter plot shows that a subset of mRNAs is in the bound fraction (S1C Fig). The RBP10 mRNA was among the enriched RNAs; this could be genuine binding, but could also be an artifact because the encoding polysomes are pulled down via the nascent polypeptide (S2 Table). In addition, 260 mRNAs were at least three-fold enriched in both pull-downs (S1D Fig and S2 Table). The bound mRNAs were significantly less abundant than unbound mRNAs (Fig 2A), with a median of 0.96 copies per cell, as opposed to 1.74 copies per cell for the unbound fraction. This was consistent with the tethering result, which had suggested that RBP10 suppresses expression. Although we cannot completely exclude the possibility that RBP10 associates with RNAs via a different RNA-binding protein partner, no evidence for such a partner had been obtained by mass spectrometry, so in our discussion we will assume that RBP10 binds the mRNAs directly. In contrast to the suggestion of de Pablos et al [50] we found no relationship between the mRNA targets of RBP10, and the proteins with which it may interact. Since RBP10 suppresses expression of target mRNAs, their suggestion that BioD tagging was due to proximity of RBP10 to nascent polypeptides is unlikely.

A comparison of the sequences of the most reliably RBP10-bound mRNAs with those of unbound mRNAs (S2 Table and S1 & S2 Text) revealed that the motif UA(U)_6_ is highly enriched in the 3'-UTRs of RBP10 targets (Fig 2B). It is present 1–9 times in 225 out of 255 annotated and/or map-able 3'-UTRs (Fig 2B, S2 Table, sheet 2, and S1 Text). Transcriptome-wide, the number of UA(U)_6_ motifs was strongly associated with RBP10 binding (Fig 2C). There are however clearly some mRNAs that contain UA(U)_6_ but were not pulled down with RBP10. Although there are some exceptions [57, 58], RRM domains usually bind to single-stranded RNA [59–61]. It is possible that in some cases, UA(U)_6_ motifs are in double-stranded structures; alternatively, binding by competing proteins might prevent RBP10 binding. The *RBP10* 3'-UTR has a single UA(U)_6_ motif, so it is conceivable that RBP10 binds its own RNA, resulting in feedback control. This might explain the loss of endogenous RBP10 that we saw upon expression of the lambda-N-tagged version.

The UA(U)_6_ motif was previously identified in several mRNAs encoding procyclic-specific proteins [62], many of which are included in the "bound" mRNA list. These include the mRNA encoding EP procyclin. Notably, the 26mer 3'-UTR element that is required for *EP1* procyclin mRNA instability [41, 42] contains two UA(U)_6_ motifs (Fig 2D), which are single-stranded *in vivo* [63]. To test whether the UA(U)_6_-containing 26mer was required for binding of the *EP1* 3'-UTR to RBP10, we used cell lines that expressed *CAT* reporter mRNAs with either the intact wild-type *EP1* 3'-UTR, or a version without the 26mer (*ep1Δ26*) (Fig 2D) [42]. Co-immunoprecipitation of the *CAT* mRNA with RBP10 depended on the presence of the 26mer (Fig 2E). Depletion of RBP10 by RNAi also increased expression from the *CAT-EP* reporter but not from *CAT-ep1Δ26* (Fig 2F). We do not know why *CAT-ep1Δ26* decreased at 24h; one possibility is that expression was suppressed as a secondary effect of RNAi-induced growth inhibition. These results demonstrated both that RBP10 binds to the *EP* 3'-UTR via the 26mer sequence, and that the 26mer is necessary for RBP10-mediated regulation of an mRNA containing the *EP1* 3'-UTR. Overall, the results support UA(U)_6_ as the main binding motif for RBP10.

The *PGKB and PGKC* mRNAs have completely different 3'-UTRs, which determine developmental regulation [64]. The UA(U)_6_ motif is present twice within a sequence that suppresses PGKB expression in bloodstream forms [65] (S1 Text). Targeted searches in the sequence reads from RBP10 pull-downs revealed that the *PGKB* mRNA was almost exclusively in the RBP10-bound fraction, whereas most *PGKC* mRNA was unbound. It is not yet clear whether the *GPEET* mRNA, which has only UA(U)_5_ repeats, is bound by RBP10, since targeted searches suggested a bound:unbound ratio of only 1.3:1.

### Many RBP10-bound mRNAs have higher expression in procyclic forms than in bloodstream forms

To assess the effect of RBP10 binding to its endogenous target mRNAs *in vivo* we first compared them with published proteomes. The proteins encoded by 112 RBP10-bound mRNAs have been quantitated during differentiation [23]. 56 of these were more abundant in procyclic than in bloodstream forms, while only 5 showed the reverse tendency (S2 Table, sheet 2). There was clear enrichment for proteins that increased 2h and12h after the initiation of stumpy form differentiation (Fisher P-values <10^−4^ and <10^−7^, respectively).

**Fig 3:**
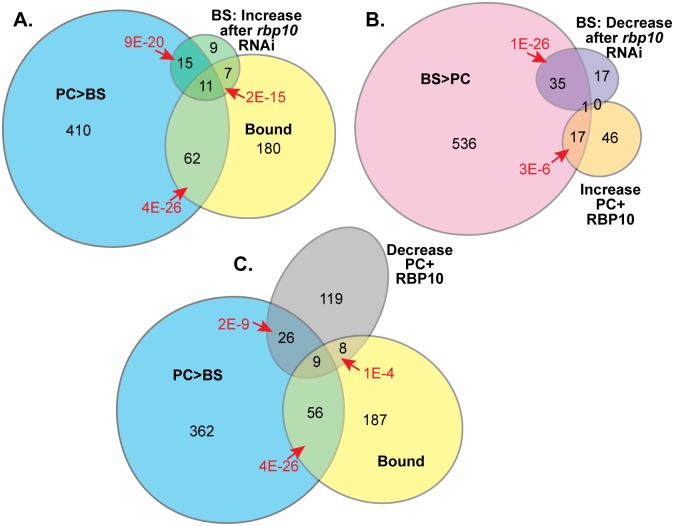
mRNAs bound to and/or regulated by RBP10. Results for mRNA levels are displayed as proportional Venn diagrams made using http://www.eulerdiagrams.org/eulerAPE/. BS = Bloodstream form, PC = procyclic form. Categories were: Bound: S2 Table sheet 2. PC>BS and BS>PC: at least 2x significantly regulated in polysomal RNA. Values for increases after *rbp10* RNAi in bloodstream forms (at least 2x significantly regulated), or RBP10-myc expression in procyclic forms (at least 1.5x significantly regulated), are from S3 Table. All data are from Lister 427 strain parasites. A) RBP10-bound mRNAs relative to those that are more abundant in procyclic forms and/or increase after *rbp10* RNAi. B) Overlaps between mRNAs that are more abundant in bloodstream forms, and those that decreased after *rbp10* RNAi in bloodstream forms or increased after RBP10 expression in procyclic forms. C) Overlaps between bound RNAs, procyclic-specific mRNAs, and the mRNAs that decreased after RBP10 expression in procyclic forms.

We next wanted to assess developmental regulation of RBP10 targets at the mRNA level. In order to assess translation as well as mRNA abundance, using Lister 427 cells, we separated polysomal mRNA from RNA that was free or co-migrated with monosomes (S3 Table). We defined regulated mRNAs as those showed at least a two-fold change, with an adjusted P-value of less than 0.05 [66]; not surprisingly, slightly more regulation was seen for polysomal RNA than for total RNA. Comparisons with these and other datasets indicated that the RBP10-bound mRNAs were strongly enriched in mRNAs that are more abundant in procyclic form than bloodstream form total or polysomal RNA (henceforth, for simplicity, described as "procyclic-form specific"). For the polysomal RNA, 73 of the 260 RBP10 targets were classified as procyclic-specific (Fig 3A). Some additional mRNAs are increased in stumpy forms (I. Roditi, personal communication). 20 mRNAs showed the reverse tendency, with higher abundance in bloodstream forms (shortened below to "bloodstream-form specific").

We had previously analysed the transcriptomes of bloodstream forms with *rbp10* RNAi and of procyclic forms expressing RBP10-myc using microarrays [45]. However, the data obtained were problematic. In addition to the very low sensitivity of the microarrays, the cells with RNAi or over-expression were already growth-inhibited at the time of harvest, and showed numerous mRNA changes related to cell proliferation [45]. It was therefore impossible to distinguish between primary and secondary effects. To find direct effects of RBP10, we needed to repeat the experiments using RNASeq. The new experiments were done after 15h *rbp10* RNAi induction in Lister 427 bloodstream forms (S1E Fig), or 6h induction of RBP10-myc expression in Lister 427 procyclic forms (S1F Fig). Both cell numbers and polysome profiles were similar to those in uninduced control cultures (S4 and S5 Figs). We again characterised polysomal, monosomal and total mRNA. Strikingly, 90% of the mRNAs that showed microarray decreases [45], including nearly all of those implicated in cell proliferation, were not decreased in the new RNASeq experiments, showing that many of the previous observations had indeed been due to growth arrest. Despite this, many other effects were seen due to the increased sensitivity of the RNASeq approach. The data from monosomes and polysomes also enabled us to calculate the percent of each mRNA that was on polysomes.

RBP10 depletion by 15h RNAi induction increased 42 mRNAs, of which more than half were procyclic-specific (Fig 3A). 52 were decreased, of which 2/3 were bloodstream-form specific (Fig 3B). Functional enrichment analysis showed significant increases for RNAs encoding mitochondrial functions and stage-specific membrane proteins, and decreases for glucose metabolism (S3 Table sheet 4). With only a single exception, RBP10-bound mRNAs were either unchanged, or increased after RNAi (Fig 3A), indicating that RBP10 suppresses expression of a subset of its bound mRNAs. 30 mRNAs moved towards polysomes (S3 Table sheet 1 and S6A and S6B Fig); and 19 of these are known to have higher ribosome densities in procyclic forms than in bloodstream forms [18]. An example is the *COXVI* mRNA (S3 Table sheet 1, S6 Fig), which is developmentally regulated mainly at the level of translation [62]. The mRNAs encoding three proteins required for stumpy formation: Tb927.11.14220 (*REG9*.*1*), *ZFP3*, and *DRBD5* [67], were not significantly affected by *rbp10* RNAi, although *DRBD5* mRNA showed a roughly 2-fold decrease that was below our significance cut-offs.

Overall the RNAi caused two effects: increases in some RBP10 targets, and a shift towards the procyclic transcriptome pattern. If RBP10 depletion initiates changes similar to bloodstream-to-procyclic form differentiation, expression of RBP10 in procyclic forms might do the reverse. Our induction period for RBP10 expression in procyclic forms was only half of a single cell division time. As a consequence, most changes were rather small, so we analysed mRNAs that showed 1.5-fold changes. The polysomal mRNA changes after RBP10 induction indeed suggested conversion to bloodstream forms (Fig 3B and 3C) (S3 Table sheets 3 and 4). We also saw decreases in a subset of the bound mRNAs. Pablos et al also recently published transcriptomes of procyclic forms expressing RBP10 for either 5h or 10h ([50], S3 Table sheet 3). The results were weakly correlated with ours, with correlation coefficients (R) for total RNA of 0.34 for 5h, and 0.41 for 10h. Correlations with our polysomal RNA results were lower. Some of the discrepancies between our results and theirs are probably due to random variation, since de Pablos et al [50] did not analyse replicates.

Since most of the mRNAs that were affected by changes in RBP10 expression were not detected in the RBP10-bound fraction, many of the changes that we saw, even at short induction times, must have been secondary to changes in the primary target mRNAs. Selected RBP10 target mRNAs that show increased abundance or translation in bloodstream forms relative to procyclic forms, and were affected by changes in RBP10 expression, are listed in Table 1. In addition to mRNAs encoding known regulated metabolic proteins and surface proteins, there are two protein kinases, a protein phosphatase, and the procyclic-specific zinc finger protein ZC3H22. Changes in such regulators might initiate a regulatory cascade.

**Table 1:** 31 selected RBP10 targets. These genes encode mRNAs that were bound to RBP10, and were significantly more abundant in procyclic than in bloodstream-form fractions for either total RNA or polysomal RNA (PC/BS; the results for polysomal RNA are shown). Some of these are already elevated in stumpy forms (I. Roditi, Universität Bern, personal communication). The polysomal mRNAs were also at least 1.5 x and significantly (Padj <0.05) increased after *rbp10* RNAi in bloodstream forms (BS RNAi). The effect on polysomal RNA for expression of RBP10 in procyclic forms (PC+RBP10) is also shown. Apart from two genes which were affected by RBP10 expression in procyclic forms, we selected genes with annotated functions. For details see S2 Table sheet 2.

Gene ID	Annotation	BS RNAi	PC+RBP10	PC/BS	UAU6
**Membrane**					
Tb927.10.10250	EP2 procyclin (EP2)	5.9	n/a	199.5	3
Tb927.7.6850	trans-sialidase (TbTS)	2.4	0.51	4.0	4
Tb927.8.7340	trans-sialidase	1.9	0.34	5.9	3
Tb927.4.3500	Amastin-like protein	2	n/a	10.4	1
**Mitochondrion**					
Tb927.11.5050	fumarate hydratase	1.5	n/a	2.0	1
Tb927.9.5900	glutamate dehydrogenase	1.8	n/a	2.5	4
Tb927.10.2350	pyruvate dehydrogenase complex	2	n/a	2.3	1
Tb927.10.4280	Complex III cytochrome b/c	4.1	n/a	19	2
Tb927.5.3040	Cytochrome c oxidase complex	2.5	n/a	4.0	1
Tb927.4.4990	ubiquinol-cytochrome c reductase	1.7	n/a	3.2	1
Tb927.9.4310	tricarboxylate carrier, putative	2	n/a	2.2	1
**Regulation**					
Tb927.11.15010	NEK21 protein kinase	2.5	n/a	3.1	4
Tb927.2.4200	protein kinase	1.5	0.63	1.7	4
Tb927.10.8050	Protein phosphatase	2.1	n/a	1.8	2
Tb927.7.2680	ZC3H22	1.9	0.57	1.8	7
**Other**					
Tb927.8.7730	Dihydroceramide synthase	1.7	n/a	2.1	2
Tb927.9.7470	purine nucleoside transporter NT10	1.9	n/a	12	1
Tb11.02.5400	cystathionine beta-synthase	2	0.58	2.3	1
Tb927.7.1320	Heat shock protein HSP10	2.1	n/a	2.2	1
Tb927.10.7700	ABC transporter, putative	1.5	n/a	2.7	2
Tb927.11.2410	Flagellar membrane protein	2.7	n/a	11	1
Tb927.8.4050	Flagellar membrane protein	2	n/a	5.9	1
Tb927.9.13200	Unknown function	4	0.46	15.8	2
Tb927.9.13570	Unknown function	1.8	0.62	2.1	2

UAU6: number of UA(U)6 motifs in manually annotated 3'-UTR.

n/a = not significantly regulated.

### Repression of RBP10 expression primes bloodstream forms for differentiation to procyclic forms

**Fig 4:**
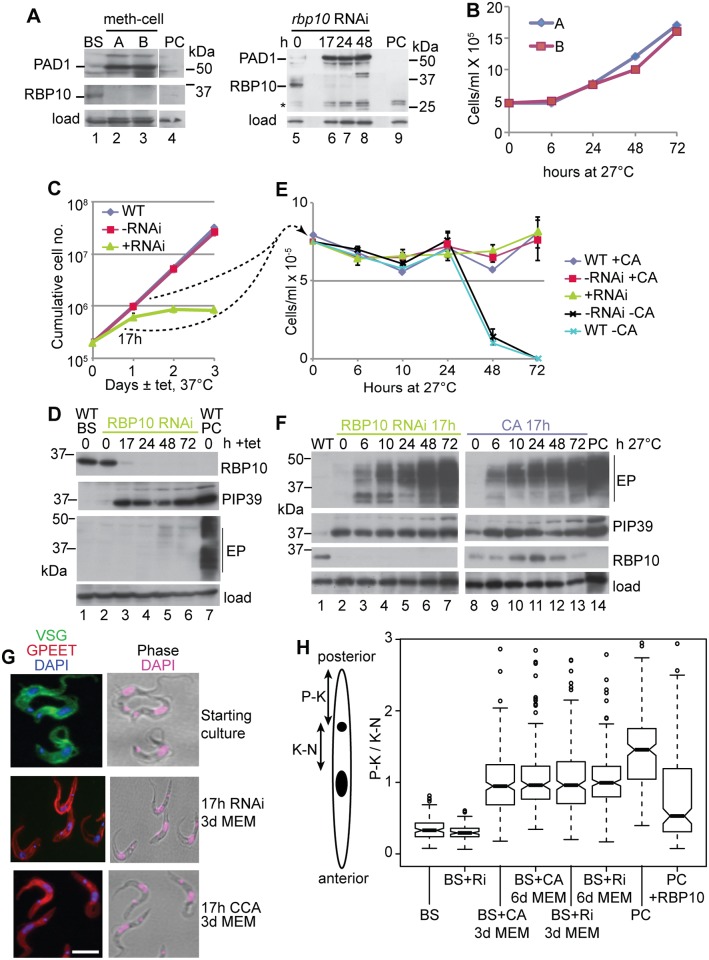
RBP10 depletion primes bloodstream forms for differentiation to procyclic forms. A) Expression of PAD1 and RBP10 in trypanosomes incubated at maximal density in methyl cellulose-containing medium for 3 days (left panel), or after *rbp10* RNAi (right panel). * = nonspecific band. B) Growth of trypanosomes incubated at maximal density in methyl cellulose-containing medium for 3 days, followed by cis-aconitate treatment for 17h at 27°C. Time 0 is the time of transfer to procyclic conditions. C) Cumulative growth curve of bloodstream-form (BS) trypanosomes with and without RNAi. WT = wild-type (*tet* repressor only); -RNAi: RNAi cell line with no tetracyline: +RNAi: RNAi cell line with tetracycline. D) Expression of RBP10, PIP39, and EP procyclin were measured by Western blotting up to 3d after tetracycline induction of RNAi. E) 17h after RNAi induction at 37°C in bloodstream form medium (+RNAi), or incubation at high density in procylic medium with 6mM cis-aconitate at 27°C (+CA), trypanosomes were placed in procyclic medium (MEM-pros) at 27°C with neither tetracycline nor cis-aconitate. The cell densities of the cultures were monitored. Negative controls were either wild-type or uninduced RNAi lines, without cis-aconitate. Cell densities are shown; they started to increase at 72h. F) Protein expression after transfer to procyclic medium at 27°C after 17h pre-treatment as in (E). G) Immunofluorescence: Cell morphology and expression of GPEET procyclin after 3d cultivation in procyclic medium. DNA is stained with DAPI. H) The cartoon shows the way in which the position of the kinetoplast, relative to the nucleus (P-K/K-N), was measured. The box plot shows the median with 25th percentiles and 95% confidence limits. Some outliers have been deleted. The full results, together with the numbers of trypanosomes measured, are in S8A Fig. BS—normal bloodstream forms; PC- normal procyclic forms; CA - 17h cis-aconitate pretreatment; RNAi or Ri; 17h *rbp10* RNAi induction; +RBP10—induced expression of RBP10 for 2 days. There were no significant differences between the 3-day and 6-day differentiating populations. All other pairs were significantly different (Student t-test, p<0.05).

To find out whether changes in RBP10 really can initiate differentiation, we used differentiation-competent trypanosomes (EATRO 1125). To confirm their differentiation competence, we grew the cells at high density in methyl cellulose for three days. This resulted in expression of the stumpy-form marker PAD1 [68] and stumpy morphology [7]. RBP10 was also reduced (Fig 4A, lanes 2 & 3), consistent with its absence in the stumpy-form proteome [23]. The arrested cells were treated with the differentiation inducer cis-aconitate in bloodstream-form medium at 27°C for 17h. Upon transfer to procyclic medium without cis-aconitate, the cells started to grow as procyclics within 24h (Fig 4B).

Induction of *rbp10* RNAi in the differentiation-competent cells in log phase resulted in growth inhibition (Fig 4C), PAD1 expression (Fig 4A, lanes 6–8) and, within 17h, expression of the differentiation-regulating phosphatase PIP39 [69] (Fig 4D). Most interestingly, after 17h RNAi, when cell numbers were still unaffected, transfer of the cells to procyclic-form medium at 27°C—without cis-aconitate—allowed survival and growth (Fig 4E), with expression of EP procyclin (Fig 4F, lanes 1–7) and GPEET procyclin (Fig 4G). As expected, cells with neither RNAi induction nor *cis* aconitate pre-treatment died under procyclic culture conditions (Fig 4E). These results suggest that *rbp10* RNAi can substitute for 27°C, cis-aconitate and high density as an initial inducer of differentiation, although the subsequent conversion to growing procyclic forms is either slower, or less efficient, than when stumpy cells are used. Our RNAi experiments were done in log phase and without methyl cellulose for two reasons. Firstly, induction of RNAi requires active RNA polymerase I transcription, but transcription is suppressed in stumpy forms [70] and probably also in cells at high density [71]. Secondly, RBP10 decreases anyway in stationary phase cells (Fig 4A). As the nearest possible control, we therefore treated high density, but non-stumpy, differentiation-competent cells in liquid medium with cis-aconitate for 17h at 27°C [72] before transfer to procyclic medium without cis-aconitate at 27°C. In these cells, weak RBP10 expression persisted for 2 days (Fig 4F, lanes 8–13) but growth and procyclin expression were similar to those seen after *rbp10* RNAi (Fig 4E, 4F and 4G). The slow kinetics were due to heterogeneity in the cultures, which were mixtures of normal-looking, proliferating, and dying or abnormal forms. Even after 3 days in procyclic culture, about 9% of the cells in both cultures retained bloodstream-form morphology.

Phosphoglycerate kinase is cytosolic in procyclic forms due to expression of the *PGKB* gene (S7A Fig) whereas in bloodstream forms, a glycosomally targeted isoform, PGKC, is expressed (S7B and S7C Fig) [73, 74]. The differentiating cultures showed a mixture of the two localisations (S7D Fig). In bloodstream forms, the kinetoplast is at the posterior end of the cell (Fig 4G), which means that the distance between the posterior and the kinetoplast (P-K) is much less than the distance from the kinetoplast to the nucleus (K-N) (Fig 4H and S8A and S8B Fig; "BS"). Procyclic forms are longer and the kinetoplast is nearer to the nucleus (Fig 4G), giving a much higher P-K/K-N ratio (Fig 4H and S8A and S8B Fig; "PC"). The differentiating cultures showed heterogeneous P-K/K-N ratios, with medians that were intermediate between those of bloodstream and procyclic forms (Fig 4H, S8A–S8C Fig). As expected, cells with the low ratio typical of bloodstream forms were preferentially GPEET negative (S8D and S8E Fig).

### Expression of RBP10 in procyclic forms induces expression of variant surface glycoprotein and loss of procyclin

**Fig 5:**
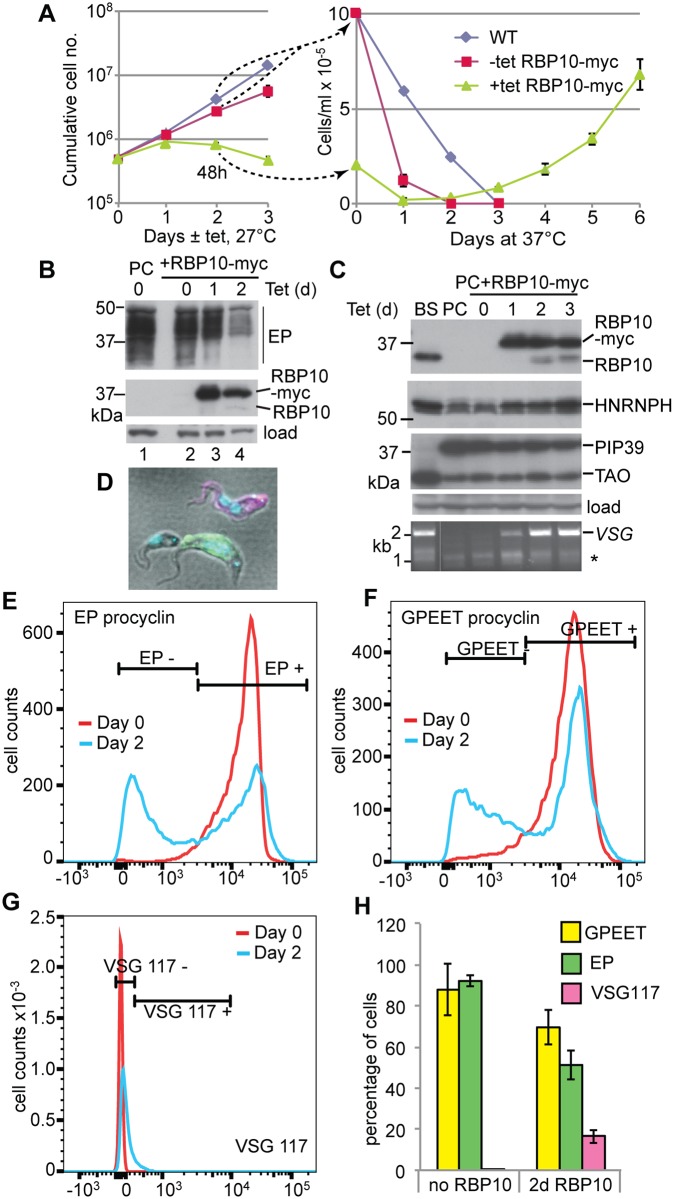
RBP10 expression in procyclic forms causes differentiation to bloodstream forms. A) Cell counts in a typical experiment. Expression of RBP10-myc was induced using tetracycline at 27°C in procyclic-form medium. The left-hand panel is a cumulative growth curve. After 48h cells were transferred to bloodstream-form medium at 37°C; in the right-hand panel cell densities are shown. B) Expression of EP procyclin and RBP10 after induction of RBP10-myc expression. Cells were cultured in procyclic medium at 27°C. Expression of EP procyclin protein decreased after 48h of RBP10-myc induction, while untagged RBP10 becomes detectable. C) As in (B) except that culture continued for 3 days. Proteins that are expressed more in bloodstream forms than in procyclic forms were detected by Western blotting. TAO is trypanosome alternative oxidase; HNRNPH is an RNA-binding protein. *VSG* mRNA was detected by RT-PCR using a spliced leader primer and a primer that hybridizes to a conserved region within the *VSG* 3'-UTR. D) Trypanosomes were taken after 48h RBP10-myc induction. They were stained for EP procyclin and phosphorylated GPEET procyclin (green), VSG117 (magenta) and DNA (cyan). The stain is overlaid with differential interference contrast (grey). The panel shows a typical green procyclic form, a bloodstream form with two kinetoplasts, and a third cell which resembles a bloodstream form (terminal kinetoplast) but was not stained by anti-VSG117. This cell may express a VSG that does not react with the anti-VSG117 antibodies. E) Quantitation of surface EP procyclin by flow cytometry, comparing cells with (day 2) and without (day 0) induced RBP10-myc expression. F) Quantitation of surface phospho-GPEET procyclin by flow cytometry, comparing cells with (day 2) and without (day 0) induced RBP10-myc expression. G) Quantitation of staining with anti-VSG117 by flow cytometry, comparing cells with (day 2) and without (day 0) induced RBP10-myc expression. H) Measurement of surface protein expression by flow cytometry. Values for the different windows are mean and standard deviation from 4 replicates. Similar results were obtained by manual counting of stained smears (S8 Fig).

The results so far showed that the absence of RBP10 allowed trypanosomes to grow as procyclic forms. We next asked whether the presence of RBP10 would force cells to become bloodstream forms. To find out, we made differentiation-competent EATRO1125 bloodstream forms with inducible RBP10-myc, differentiated them into procyclic forms, grew these for more than 3 months, and then induced RBP10 expression. After RBP10-myc induction, cell growth was inhibited, as expected [45] (Fig 5A). After 48h, expression of procyclin was strongly reduced, alternative oxidase increased, and untagged RBP10 was detected (Fig 5B and 5C). At this point, some cells had normal procyclic morphology and expressed EP and GPEET procyclin; some were clearly abnormal; while another subset had bloodstream-form morphology (Fig 5D). This was reflected in the P-K/K-N ratios, which ranged from procyclic to bloodstream-form patterns (Fig 4H, S8F Fig). Flow cytometry analysis revealed that about 50% of cells had decreased, or absent, procyclin expression (Fig 5E and 5F). Cells with bloodstream-form morphology were usually (but not always) procyclin negative (S8G Fig). Notably, *VSG* mRNA was present (Fig 5C). PGK staining in the bloodstream-form-like cells was more punctate than in procyclic forms, but had not assumed a purely glycosomal pattern (S7E Fig).

**Fig 6:**
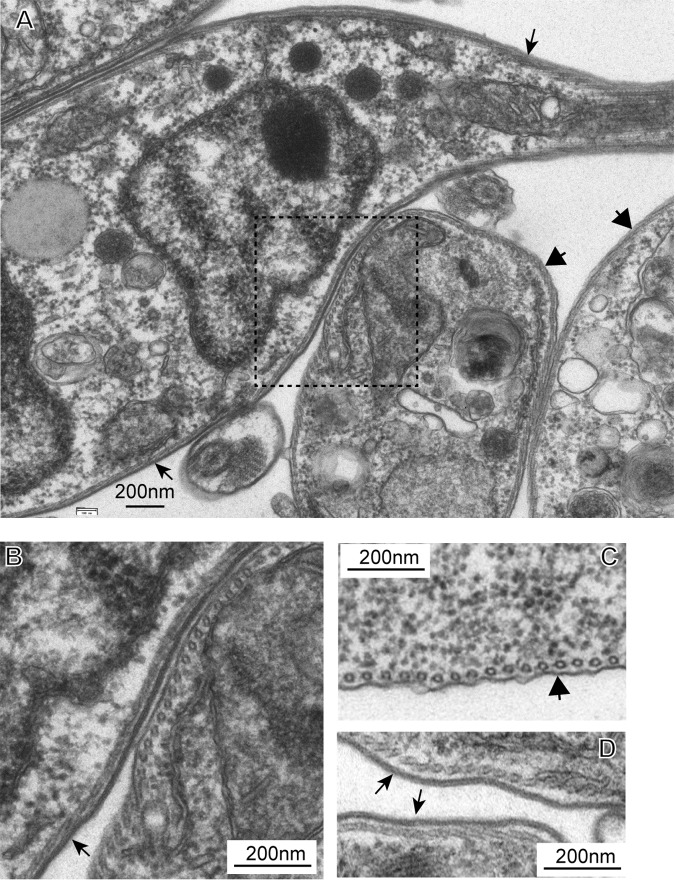
VSG expression after RBP10 induction in procyclic forms: Electron microscopy. A) A sample field from procyclic-form cultures after 48h induced expression of RBP10. The thin arrows point to VSG coats and the thick ones to procyclic surfaces. B) Close-up of the boxed area in (A). C) Typical field showing the surface of a procyclic form. D) Typical field showing the surfaces of bloodstream forms.

We do not have antibodies to all VSGs that are expressed in the bloodstream-form-morphology cells, but a subset of them stained with a polyclonal antibody against VSG117 (Fig 5D, 5G and 5H). These cells all had small P-K/K-N ratios typical of bloodstream forms (S8G Fig). To estimate the number of bloodstream forms induced by RBP10 expression without relying on the antibody, we examined the 48-hour induced procyclic forms by transmission electron microscopy. A complete field is shown in S9A Fig, and more highly magnified examples are in Fig 6A and 6B and S9B Fig. Control procyclic and bloodstream forms are in Fig 6 and S9 Fig, panels C and D respectively. The differentiating cultures contained both intact and abnormal cells (S9A Fig). To distinguish bloodstream forms from procyclic forms, we examined cross-sections in which the cytoskeletal microtubules were clearly distinct from the plasma membrane. In such sections, the plasma membrane of procyclic forms appears naked and is often rather wavy (large arrows, Fig 6C and S9C Fig), whereas bloodstream forms have a dense coat with a smoother appearance (small arrows, Fig 6D and S9D Fig). Fig 6A and S9B Fig are sections in which a newly-developed bloodstream form is next to a procyclic form; Fig 6B is shown at higher magnification to illustrate the difference in parasite surfaces. The bloodstream form in Fig 6A may be dividing since it has two nuclei. Counting of over 100 trypanosome profiles from 25 EM images suggested that about 15% of the cells had VSG coats, but this could be an under-estimate since any membranes that were difficult to classify were counted as procyclic.

### RBP10 expression primes procyclic forms for growth as bloodstream forms

To find out whether RBP10 induction in procyclic forms was indeed causing differentiation towards the bloodstream form, we transferred the cells to bloodstream form medium at 37°C. Procyclic forms without RBP10 expression died (Fig 5A), but after a short lag, the RBP10-induced cells proliferated (Fig 5A); after 6 days all survivors had bloodstream-form morphology and 60% of them cross-reacted with the anti-VSG117 antibody (107 out of 187 counted cells). RBP10 induction at 27°C for two days was required; this was also the time needed to detect native RBP10 expression by Western blotting. Results from serial dilution of the 48h-induced cultures suggested that approximately 1 in 10,000 cells was surviving, but this may be an underestimate. Procyclic forms do not survive at low cell densities in culture, possibly because they require a degree of inter-cellular communication [75]. It is possible that despite their VSG expression, the induced cells retain this characteristic.

### Does RBP10 expression in procyclic forms cause formation of epimastigotes?

Within the Tsetse fly, procyclic forms differentiate to epimastigote forms before forming non-dividing, VSG-expressing metacyclic forms in the salivary glands. This process takes 2 weeks. After induced expression of RBP6, epimastigotes were clearly seen [49] and *VSG* mRNA and protein were first detected after 3 days [76]. Induction of VSG expression by RBP10 was faster, since the mRNA was detectable after only 24h, and VSG protein within 2 days. Is RBP10-mediated induction an accelerated version of the normal pathway ("forward" differentiation)? Or is RBP10 forcing the procyclic trypanosomes to revert back to bloodstream forms, essentially driving differentiation in reverse? To try to answer this, we first looked for epimastigotes in the cultures. Epimastigotes are clearly distinguishable from the other forms because the kinetoplast is anterior to the nucleus. The only available protein marker for epimastigotes is the surface protein BARP, but it is not certain that all epimastigotes express it. Epimastigotes are thought to be the only cell type that adheres to the salivary gland epithelium. The BARP surface protein is expressed by many trypanosomes that are attached to the salivary glands, and all BARP-positive cells have epimastigote morphology [12]. However, some attached trypanosomes are BARP-negative [12]. Cells with an epimastigote-like kinetoplast-nucleus arrangement were never seen after induction of RBP10-myc expression in our differentiation-competent cells (less than 1 in 500 cells) and BARP was not detected by Western blotting or immunofluorescence.

**Fig 7:**
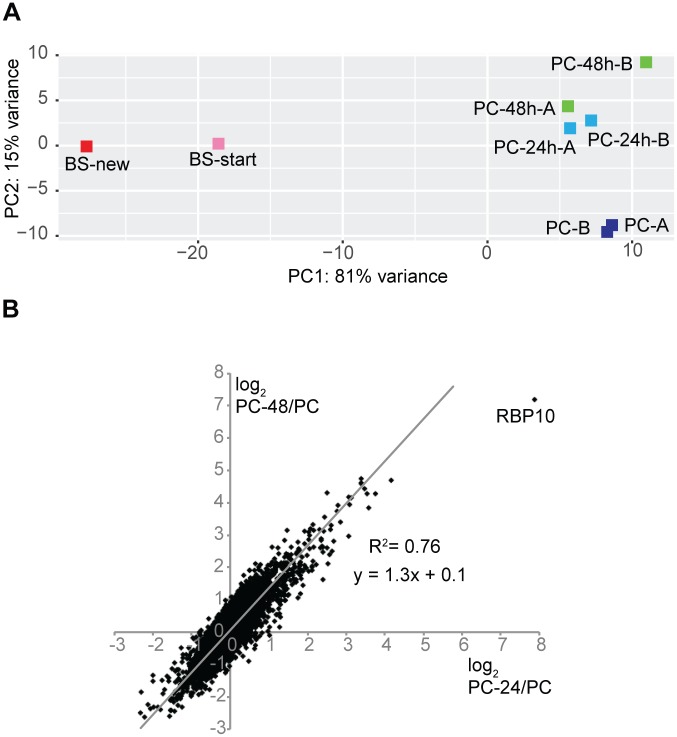
Analysis of transcriptomes of EATRO1125 with forced RBP10 expression. A) Principal component analysis showing the clustering of procyclic forms, bloodstream forms and cells with induced RBP10 expression. B) Correlation between the effects of 24h and 48h RBP10 expression for 7000 unique genes. For each gene, the log_2_ of the ratio between the induced and uninduced cultures is shown.

The morphological analysis did not resolve the "direction" of RBP10-mediated differentiation. As an alternative, we compared the transcriptomes of the differentiating cells with those of starting and final populations (S4 Table). Principal component analysis showed that the transcriptome of the initial bloodstream forms clustered with that of re-differentiated forms (Fig 7A). The 24h and 48h induced populations clustered as well, with changes at 48h being similar to, but stronger than, those seen at 24h (Fig 7B). The transcriptome changes after 24h or 48h RBP10 expression were weakly positively correlated with those seen when parasites move from the proventriculus or midgut to the salivary glands, but not at all with those from the midgut to the proventriculus (S4 Table, sheet 1). These results would be consistent with RBP10 expression causing a jump from the procyclic to the bloodstream or metacyclic form. *RBP10* mRNA itself is not regulated in the Tsetse dataset, but the reasons could be technical: for example, we have found that for unknown reasons, using the Cufflinks tool eliminates most *RBP10* read counts.

The mRNA encoding BARP is 100-200-fold more abundant in salivary gland and proventricular trypanosomes than in midgut trypanosomes [77]. Since salivary gland and proventricular trypanosomes are a mixture of epimastigotes and metacyclic forms, the level of *BARP* mRNA in pure epimastigotes is likely to be even higher. In contrast, we observed no change in *BARP* mRNA after 24h RBP10-myc expression in procyclic forms, and a mere 2-fold increase (Padj 0.005) after 48h (S4 Table, sheet 3). This may mean that 1–2% of the cells express a normal epimastigote level of *BARP* mRNA; alternatively many cells may have a much smaller increase. However in either case, the mRNA is not translated sufficiently to give detectable protein. The mRNAs encoding meiosis-specific proteins MND1 and SPO11 were not changed in either our, or the Tsetse datasets. Our results thus yielded no evidence that RBP10 expression results in creation of epimastigotes. Instead they suggested an abnormal differentiation pathway.

In a separate manuscript, we have recently shown that after induction RBP10 expression in procyclic forms, and culture for two days in procyclic conditions, the cells are not infective for mice. However, after transient transfection of an RBP10 expression plasmid, growth for two days in procyclic medium, and culture for 3 weeks in bloodstream-form culture conditions, the resulting trypanosomes were indeed infective and showed stumpy form development at peak parasitaemia [78]. These results indicate that the presence of RBP10 primes procyclic forms for growth as bloodstream forms, but the temperature and medium shift is required to complete the differentiation process.

### VSG expression during and after RBP10-induced differentiation

To pursue the question of the "direction" of RBP10-induced differentiation, we investigated whether it was inducing metacyclic forms. The fact that after two days of RBP10 expression, some of the VSG-expressing cells were dividing (Figs 5D and 6A), argued against their being metacyclic forms. However we do not know whether the final viable bloodstream forms could arise from the cells that were dividing at day 2. To find out whether RBP10 expression causes cells to become metacyclic forms, we examined the *VSG* mRNAs in our cells. Active *VSG* genes are always telomeric. The promoters that drive their expression in established bloodstream-form trypanosomes are up to 60 kb upstream of the gene itself, with several intervening open reading frames called "expression-site associated genes" (*ESAG*s). In contrast, in metacyclic trypanosomes the active VSG is in a monocistronic transcription unit, with a promoter about 3 kb upstream of the *VSG* gene [79]. Metacyclic promoter sequences also differ from those that drive transcription of bloodstream form expression sites [79]. Use of metacyclic VSG expression sites is currently the only known marker for metacyclic forms, although metacyclic VSGs continue to be used in the first wave of parasitaemia after a Tsetse bite [80].

To find the *VSGs* expressed in our different cell populations we assembled their transcriptomes *de novo*. The resulting contiguous sequences were then searched for a motif that is conserved in the 3'-UTR of all *VSG* mRNAs. 47 contigs ranging 709–4788 bp were found (S5 Table Sheet 1). Most of the contigs (41 out of the 47) were identified from the previously procyclic cells that had been expressing RBP10 for 1–2 days; the other six contigs were from established bloodstream form cultures. Some contigs included precursor RNA sequence segments with low coverage. Consistent with the RT-PCR (Fig 5C) result, no contigs containing the *VSG* 3'-UTR motif were found in uninduced procyclic cells.

To determine whether the *VSG* contigs contained partial or full length *VSG* coding sequences (CDS), the sequences were subjected to a BLASTn analysis against the *T*. *brucei* genome at NCBI. The results were comparable between the replicates, with multiple contigs having the same *VSG* hit. Out of the 47 contigs, 20 unique *VSG* hits were present (S5 Table sheet 2, S3 Text); half had complete *VSG* CDSs, while most of the remainder were near complete (S5 Table sheet 2). To estimate *VSG* transcript levels, we added up all CDS reads from the 20 different *VSG* genes (S5 Table Sheet 2). The starting bloodstream form cells either expressed AnTat1.1 (also designated as EATRO1125.4156 at NCBI) or EATRO 1125.211, together accounting for ~7% of the total mRNA (S5 Table Sheet 2). The newly-differentiated growing bloodstream forms mainly expressed other VSGs, some of which were not present in any database so far; this made it difficult to calculate the total number of *VSG* reads for this population. The dominant identified VSG was however EATRO1125.224, and less than 0.1% of the VSG reads were the same as those of the starting cells, suggesting that VSG expression had been completely "re-set" in the new bloodstream forms.

After two days RBP10 induction, cells that had previously been procyclic showed >100x increased expression of eight different unique *VSG* transcripts, adding up to a surprising 5% of total mRNA (S5 Table Sheet 2). This result—compared with the electron microscopy—suggests that many of the cells were expressing *VSG* mRNA, but did not yet have visible surface coats. Interestingly, a known metacyclic VSG sequence, *MVSG5* [81] was present among the eight unique *VSG* transcripts (S5 Table Sheet 2). The *MVSG5* expression site was originally described for a *T*. *rhodesiense* stock that was isolated from a Kenyan patient in 1975 [82], but had already been identified in the EATRO1125 genome as well [82]; EATRO1125 was taken from an antelope in Uganda in 1966. The remaining seven VSGs came from unknown expression sites. To find out whether they had metacyclic promoters, we assembled shotgun reads from the EATRO1125 genome (~30x coverage) [83]. 18 contigs of 4–17 kb had the conserved *VSG* 3'-UTR motif. Of these, 3 included complete *VSG* CDSs from 48h-induced procyclic forms (S5 Table Sheet 2), and all of these contained a metacyclic promoter (S4 Text). In two expression site contigs, there was also a putative *ESAG1* gene further upstream (S4 Text); one of these was 99.4% identical to the previously reported metacyclic *MVSG5* expression site [81, 82] while the other resembled the ILTat 1.22 metacyclic expression site [84] but had a different *VSG* CDS. This third expression site had a putative *ESAG9* gene upstream of *ESAG1* (S4 Text). After 2 days of RBP10 expression, the three metacyclic sites accounted for about 86% of all *VSG* reads. The bloodstream-form types AnTat1.1, EATRO 1125.211 and EATRO1125.224 were present but accounted for less than 0.2% of *VSG* reads; moreover we do not know whether they were transcribed from bloodstream-form expression sites, although this seems possible. The origins of the remaining VSGs are unknown.

We conclude that RBP10 expression in procyclic forms at 27°C causes the trypanosomes to switch towards the formation of metacyclic trypanosomes, which can divide when placed in culture conditions appropriate for bloodstream forms.

## Discussion

Our results indicate that the presence of RBP10 promotes trypanosomes to become, and remain as, bloodstream forms; and further, that RBP10 binds to mRNAs with a UA(U)_6_ motif in the 3'-UTR, suppressing translation and causing mRNA destruction. The proteins encoded by the direct targets of RBP10 include EP procyclin, various regulators, and enzymes of procyclic-form energy metabolism. The UA(U)_6_ motif in some of these mRNAs had already been identified as a potential destabilizing element, and its function had been demonstrated for the EP procyclin and *PGKB* [65] mRNAs. Our results now show that RBP10 is the protein that binds to the *EP* 26mer element, inhibiting translation and initiating mRNA destruction. In contrast, deletion of the region containing UA(U)_6_ from the *COXV* 3'-UTR increased protein expression without much effect on the mRNA level [62], consistent with translation control.

Many RBP10-bound mRNAs were not affected by *rbp10* RNAi. This has however been seen for other RNA-binding proteins. Every mRNA can bind many proteins, and it is the combination of bound proteins that governs mRNA behaviour [85]. Thus in bloodstream forms, loss of RBP10 may not affect an mRNA that is bound by several other proteins causing degradation or stability. In procyclic forms, expression of RBP10 will not immediately affect a target mRNA if the UA(U)_6_ motifs in that RNA are bound by other—perhaps procyclic-specific—proteins which can compete with RBP10. Even if RBP10 binds, it may be unable to destablize the target mRNA if other proteins elsewhere on the mRNA are strongly stabilizing. There is, for example, evidence that the zinc finger protein ZFP3, which is implicated in promotion of bloodstream-to-procyclic differentiation, can bind to *EP* mRNA [86] and promote its translation. The precise target sequence of ZFP3 has not been mapped, and is unlikely to be the same as that of RBP10. First, ZFP3 does not bind to the *EP2* and *EP3* mRNAs, which have the UA(U)_6_ motif [87]; and second, of the 411 unique mRNAs with at least 3-fold enrichment in the ZFP3 pull-down [88], only 31 were 3-fold enriched in at least one of our RBP10 pull-downs.

Most of the mRNAs that were affected by artificial changes in RBP10 levels were not bound to RBP10. Moreover, decreases in RBP10 caused many non-target mRNAs to decrease; and expression of RBP10 in procyclics caused expression of some mRNAs to increase. Many of the affected mRNAs were, however, developmentally regulated. This suggests that changes in RBP10 trigger a regulatory cascade. Possible culprits are not difficult to find among the direct RBP10 targets: mRNAs encoding proteins with domains for mRNA binding or signal transduction. RBP10 binds to 12 mRNAs encoding potential RNA-binding proteins. Most of these were unaffected by RNAi or RBP10 expression, but total and polysomal mRNA encoding ZC3H22 was significantly decreased by the presence of RBP10 (Table 1 and S2 Table, sheet 2). ZC3H20 and ZC3H22 are normally expressed only in procyclic forms [22, 89]. (Dejung et al [23] confirm this for ZC3H22 but have a contradictory result for ZC3H20.) ZC3H20 binds to, and stabilizes, at least two procyclic-specific mRNAs [89], of which one, *MCP12*, is not bound by RBP10 but was increased after *rbp10* RNAi. ZC3H22 suppresses expression in the tethering assay [47], and is required for proliferation of procyclic forms [22, 90]. Suppression of ZC3H21 and ZC3H22 expression is clearly very important to bloodstream-form trypanosome survival: their mRNA 3-UTRs contain, respectively, no fewer than 5 and 7 RBP10 binding motifs (S1 Text).

RBP10 binds to mRNAs encoding 13 protein kinases and two protein phosphatases (S2 Table, sheet 2). The mRNAs encoding two of the kinases and one of the phosphatases increase after RNAi (Table 1). NRKA is up-regulated after the onset of stumpy form differentiation [22], and its mRNA was in the bound fraction but slightly less than 3-fold enriched in one replicate; it was not affected by the RNAi. RDK1, another product of a bound mRNA, is a differentiation repressor since its depletion primes bloodstream forms to differentiate to procyclic forms [91] and it is more abundant in bloodstream forms, making regulation by RBP10 unlikely.

Overall, the changes in direct RBP10 target mRNAs, and their encoded proteins, can be predicted to have numerous downstream effects on gene expression. In turn, some of theses secondary changes will trigger further events. These include the increase in PIP39, a key regulator of differentiation [69] (Fig 4), and decreases in mRNAs encoding the RNA-binding proteins PUF11, RBP9, ZC3H31, ZC3H46, DRBD5 and HNRNPH (S3 Table). These changes in turn will trigger further changes in gene expression. Finally, RBP10 regulates expression of proteins implicated in procyclic energy metabolism. Changes in RBP10 expression will affect metabolite levels, and such changes are known to influence signaling pathways (e.g. [92]).

In other organisms, several proteins that decrease the translation and/or stability of bound target mRNAs have been shown to do this by recruiting proteins that are directly implicated in such processes, such as the CAF1/NOT complex and eIF4E-interacting proteins (e.g. [93–97]). Unfortunately, none of the proteins that we identified by affinity purification was found in the yeast two-hybrid analysis, although nearly 100 potential 2-hybrid interaction partners were identified. We thus have no evidence for direct recruitment of the degradation or translation machineries by RBP10. There was, however, a limited overlap between the proteins that co-purified in our affinity purifications from bloodstream forms, and the proteins that were specifically modified by BirA*-RBP10 expressed in procyclic forms [50]. Most interestingly, both sets of results suggested that RBP10 interacts with the regulatory portion of the proteasome—in total, we identified 9 of the 16 subunits. Recruitment of the proteasome to an mRNA could result in destruction of proteins that protect the mRNA, such as the eIF4F complex and poly(A) binding protein, and could also inhibit translation by destroying factors involved in elongation. However it is also possible that the proteasome interaction is a consequence of tagging RBP10. Interactions with the RNA-binding proteins UBP2 [98] and ALBA1 [99] were also found; these two proteins are however both very abundant and quite commonly found in RNA-related purifications.

The large number of two-hybrid partners suggests that RBP10 might conceivably act via aggregation or hydrogel formation, rather than via highly specific protein-protein interactions. Purified full-length RBP10 shows a strong tendency to aggregate in solution; this can be abrogated by removing the 88-residue repressive C-terminus (Bin Liu and Igor Minia, ZMBH, preliminary results). The C-terminus is rich in polar (31%) and hydrophobic (34%) amino acids with 10% glycine and17% proline; but it is not predicted to have prion-like properties [100]. In mammalian cells and yeast, some enzymes involved in mRNA degradation are concentrated in microscopically visible structures called P-bodies. Exponentially growing unstressed trypanosomes lack microscopically detectable P-bodies (see e.g. [101]) and we have no evidence for aggregation of RBP10 when it is expressed at normal levels in bloodstream forms, RBP10 sediments at the top of sucrose gradients and TAP-tagged RBP10 is distributed throughout the cytosol [45]. However in procyclic forms, inducibly expressed YFP-tagged RBP10 was located in granules containing the P-body markers DHH1 and SCD6 [50], and the presence, in bloodstream forms, of RBP10-containing granules that are smaller than stress granules has not been ruled out.

Previously, the only condition known to allow differentiation of cultured *T*. *brucei* procyclic forms to bloodstream forms *in vitro* was induced expression of the putative RNA-binding protein RBP6. This mimics the natural differentiation pathway, with formation of epimastigotes within 24h and appearance of VSG-expressing metacyclic forms 4–5 days later [49]. *RBP6* mRNA is maximal in salivary gland parasites, and low in both procyclic and bloodstream forms [46]. RBP6 action is therefore transient; its mechanism of action is unknown. Depletion of the RNA-binding protein DRBD18 from procyclic forms caused a partial switch to a bloodstream-form or epimastigote expression pattern; the mRNAs that increased included those encoding RBP6 and RBP10, but the ability to differentiate further was not tested [102]. Heat shock can also cause small increases in epimastigote-specific transcripts [103]. The rapid conversion of procyclic forms to bloodstream forms by RBP10 expression seems to be different from all of these effects. The mRNA encoding the meiosis protein MND1, which is specific to a pre-metacyclic intermediate form, is bound by RBP10 and was moderately *decreased* upon RBP10-myc induction. Although a moderate increase in BARP mRNA was detected, no epimastigotes were found either by BARP protein expression or by morphological criteria. Thus instead of the normal life-cycle progression, the RBP10-induced conversion seems to resemble *trans*-differentiation, or at least a "jump" from the procyclic to the metacyclic form. In animal cells, *trans*-differentiation can be achieved through changes in expression of transcription factors, either by direct manipulation or by changing expression of microRNAs that control transcription factor expression [104–106]. However, these changes have so far always been unidirectional. *Trans* differentiation that works in both directions according to the presence or absence of a single protein has, to our knowledge, not previously been observed.

It has long been known that stumpy forms cannot revert to long-slender bloodstream forms, and recently the differentiation of stumpy forms to procyclics was shown to resemble an irreversible bi-stable switch [22]. We suggest that expression of RBP10 governs a similar bi-stable switch during the transition from bloodstream forms to stumpy forms, and from procyclic forms to bloodstream forms. The bi-stable character might be explained by the fact that RBP10 directly suppresses procyclic-specific signalling and post-transcriptional regulators. While RBP10 is present, the bloodstream-form state is self-reinforcing. Loss of RBP10 enables procyclic regulators to be expressed, while expression of RBP10 in procyclic forms suppresses them and thus tips the balance towards a bloodstream-form expression pattern. Results so far suggest that this system is likely to be redundant; if so it is quite possible that additional proteins will be discovered that are capable of initiating the switch in at least one direction.

We do not know whether RBP10 has any role in regulation of gene expression in specific life-cycle stages of other kinetoplastid species. Indeed, beyond *T*. *brucei*, so far there is no information concerning regulation of RBP10 protein levels. In *Leishmania*, *RBP10* RNA is more abundant in mammal-infective amastigotes than in sandfly-infective promastigotes [107–109]. For *T*. *cruzi*, results from a microarray analysis suggested that *RBP10* mRNA is less abundant in amastigotes and epimastigotes than in non-dividing forms [110], but by RNASeq, very few reads were obtained for any studied stage [111]. RBP10 did not evolve to allow survival in mammals, since RBP10 genes are present in the monoxenous species *Crithidia* and *Blechomonas* [112]. Perhaps, in the common ancestor of Kinetoplastids, RBP10 had a constitutive or housekeeping function, which it retains in monoxenous species. In trypanosomes, perhaps some other protein took over the housekeeping function, allowing RBP10 to evolve a species-specific role. Alternatively, RBP10 may be involved in stage differentiation in all species: there are 3 morphological variants of *Crithidia fasciculata* in mosquitos [113]. Studies of RBP10 expression and function in other Kinetoplastids would be needed to resolve this issue.

## Materials and methods

### Parasite culture and plasmid constructs

*T*. *brucei* Lister 427 [114] and pleomorphic EATRO 1125 [72] cells expressing the Tet-repressor were used. Lister 427 were used for all experiments except those shown in Figs 4–7, S7–S9 Figs and S4 Table. Bloodstream form parasites were cultured in HMI-9 medium supplemented with 10% fetal bovine serum at 37°C with 5% CO2. To maintain pleomorphic morphology between manipulations, the EATRO 1125 cells were maintained in medium containing 1.1%methyl cellulose [115]. The procyclic forms were grown at 27°C in MEM-Pros medium supplemented with 10% heat inactivated fetal bovine serum. Stable cell lines were generated and maintained as described in [72, 114] except that selection of the pleomorphic cells was done with 8μg/ml hygromycin and 2μg/ml blasticidin.

The tetracycline inducible constructs for *RBP10* RNAi and over expression were described in [45]. All new constructs and oligonucleotides are in S6 Table. A bloodstream-form cell line expressing all RBP10 with a tandem affinity purification tag at the N-terminus was generated by inserting the tag at the 5' end of one endogenous *RBP10* open reading frame (pHD2506) and deletion of the second copy (pHD2061). Plasmids for the tethering assays are in S6 Table. The tethering constructs were separately transfected in a cell line constitutively expressing the *CAT* reporter with 5 copies of *boxB* preceding the *ACT* 3’ UTR. Expression of the fusion protein was induced for 24h using tetracycline (100ng/ml) and the CAT assays carried out as described in [33].

### Protein-protein interactions

To study interactions of RBP10 we used bloodstream-form trypanosomes in which one *RBP10* allele was knocked out and the other bore a sequence encoding an N-terminal TAP tag. TAP-RBP10 was purified on an IgG column, bound protein was eluted with Tobacco Etch Virus (TEV) protease, bound to a calmodulin column and eluted with EGTA [116, 117]. Proteins were subjected to denaturing SDS-PAGE and analysed by mass spectrometry [53].

The yeast two-hybrid screen was done as previously described [54]. Very briefly, the two-hybrid prey library consisted of yeast expressing random genomic DNA fragments cloned into the yeast 2-hybrid prey plasmid pGAD7. The library was screened using RBP10 cloned into pGBKT7 as bait. After stringent selection DNA was made from surviving cells, then library inserts were amplified and sequenced in bulk. The inserts were then mapped to the *T*. *brucei* genome and fragments that were in frame, and began downstream of position -36 of open reading frames, were selected for further analysis [54]. Proteins were scored as being potential interactors only if at least two different interacting fragments gave more than ten read counts. Proteins that have shown interactions in several other screens [53] were excluded.

### Cross-linking and RNA immunoprecipitation

3x10^9^ bloodstream form cells expressing TAP-RBP10 were irradiated using UV (400 mj/cm2), washed in cold PBS and the cell pellet snap frozen in liquid nitrogen. The RNA immunoprecipitation was done as described in [32]. Briefly, the extracts were incubated with the beads, and the unbound fraction was collected. After washing, bound RBP10 was eluted using TEV protease. After digestion with 50μg proteinase K at 42°C for 15 minutes to reduce cross-linked protein, RNA was isolated from both the bound and unbound fractions using Trifast reagent (Peqlab, GMBH). To assess the quality of the purified RNA, an aliquot of the sample was analysed by Northern blotting and the blot hybridized with a splice leader probe. Total RNA from the unbound fraction was depleted of ribosomal RNA (rRNA) using RNAse H as described in [118] except that a cocktail of 50-base DNA oligos complementary to trypanosome rRNAs was used [103]. The recovered RNA from both bound and unbound samples was then analysed by RNA-Seq.

### Polysome fractionation

Approximately 3-5x10^8^ cells were collected by centrifugation (850 x g, 10 minutes, 21°C), and then treated in serum free media with 100μg/ml cycloheximide for 7 minutes at room temperature. The pellet was washed in 1 ml ice cold PBS, lysed in 350μl lysis buffer (20mM Tris pH7.5, 20mM KCl, 2mM MgCl2, 1mM DTT, 1000u RNasin (Promega), 10μg/ml leupeptin, 100μg/ml cycloheximide, 1× complete protease Inhibitor without EDTA (Roche), 0.2% (vol/vol) IGEPAL) by passing 15–30 times through a 21G needle, followed by centrifugation (15000 x g, 10 minutes, 4°C) to clear the lysate. KCl was adjusted to 120 mM and the clarified lysate loaded on top of a 4 ml continuous linear 15–50% sucrose (w/v) gradient in polysome buffer (20mM Tris pH7.5, 120mM KCl, 2mM MgCl2, 1mM DTT, 10μg/ml leupeptin, 100μg/ml cycloheximide). After 2 hours of ultracentrifugation (40000 rpm, 4°C; Beckman SW60 rotor), 400μl fractions were collected using Teledyne Isco Foxy Jr. gradient fractionator system and RNA was isolated using Trifast reagent (Peqlab, GMBH). In the case of RBP10 tethering, lambdaN-RBP10 was induced (24h) and the distribution of the *CAT* reporter and *alpha-tubulin* mRNAs in the collected fractions detected by Northern blotting as described in [119].

For RNASeq, bloodstream form cells plus or minus *RBP10* RNAi for 15h and, the procyclic form cells with or without RBP10-myc over-expression for 6h were used. In this case 250μl fractions were collected from each gradient and RNA was isolated after pooling the fractions into two groups; i) lighter fractions including monosomes, ii) denser fractions with at least two ribosomes. The amount of mRNA in the pooled samples was assessed by Northern blotting using splice leader RNA as probe. Also, total RNA was prepared from the input samples (~10% of total cell lysate) to quantify changes in the steady state mRNAs levels. All samples were depleted of rRNAs (as above) prior to analysis by RNA-seq.

### High throughput RNA sequencing and bioinformatic analysis

RNA-seq was done at the CellNetworks Deep Sequencing Core Facility at the University of Heidelberg. For library preparation, NEBNext Ultra RNA Library Prep Kit for Illumina (New England BioLabs Inc.) was used. The libraries were multiplexed (6 samples per lane) and sequenced with a HiSeq 2000 system, generating 50 bp single-end sequencing reads.

The quality of the raw sequencing data was checked using FastQC (http://www.bioinformatics.babraham.ac.uk/projects/fastqc), and the sequencing primers removed using Cutadapt [120]. The data was aligned to the *T*. *brucei* TREU 927 reference genome using Bowtie [121], then sorted and indexed using SAMtools [122]. Reads aligning to open reading frames of the TREU 927 genome were counted using custom python scripts. This was done in individual steps for the Lister 427 data; for EATRO1125, a custom pipeline was used [123]. Analysis for differentially expressed genes was done in R using the DESeq2 package [124], using a custom tool for trypanosome transcriptomes [55] which also yields principal component analysis plots. Comparative analysis was limited to a list of unique genes modified from [16]. Gene annotations are manually updated versions of those in TritrypDB (http://tritrypdb.org/tritrypdb/), and categories were assigned manually. Other statistical analysis was done in R. The 3’ UTR motif enrichment search was done using DREME [125]; annotated 3’-UTR sequences were downloaded from tritrypDB and we considered only the mRNAs with 3’ UTRs >20 nt. Manual 3'-UTR annotation was done using the RNASeq reads and poly(A) site data [16, 18, 126] in TritrypDB.

For *de novo* assembly, the SPAdes [127] genome assembler was used. Contigs having the ‘TGATATATTTTAAC’ motif that is present in the 3’ UTR of all *VSG* mRNAs were identified and analysed using a VSG gene identification tool (https://github.com/klprint/IdentifyVSGs) (Kevin Leiss and others, in preparation) The random shotgun reads from the EATRO1125 genome were obtained from [83]; only contigs of 4 kb and longer were considered for metacyclic promoter identification.

### Analysis of 3'-UTR reporters

Bloodstream form cells expressing the *CAT* reporter with either full length *EP1* 3’ UTR (pHD1610) or a mutant version (pHD1611) lacking the 26mer (*EP1Δ26*) instability element [42] were used for RNA immunoprecipitation. For the pull down, 4x10^8^ cells (without the stem-loop) were irradiated using UV (400 mj/cm^2^), washed in cold PBS and the cell pellet lysed in 350μl lysis buffer (10mM Tris pH 7.5, 10mM NaCl, 1000u RNasin (Promega), 1× complete protease Inhibitor without EDTA (Roche), 0.1% IGEPAL) by passing 15 times through a 21G needle. The lysate was cleared by centrifugation at 15000g for 10 minutes at 4°C, NaCl was adjusted to 150mM followed by incubation with 50μl anti-RBP10 [45] coupled agarose beads for 2 hours at 4°C. After washing the beads 5 times with IPP150 buffer (10mM Tris pH 7.5, 150mM NaCl, 0.1% IGEPAL), the beads were treated with 20μg proteinase K at 42°C for 15 minutes and RNA was isolated from both bound and unbound fractions using Trifast reagent (peqlab, GMBH). Equal amounts of the recovered RNA (eluate and flow through fractions) were reverse-transcribed using RevertAid First Strand cDNA Synthesis Kit (Thermal Scientific) according to the manufacturer’s instructions. 2μl of the cDNA was used as template in a 50μl PCR reaction to detect the *CAT* and *alpha-tubulin* genes. PCR was done using Q5 DNA polymerase and buffer (NEB) with 0.5μm of the following primers, for *CAT* (CZ5725; CZ689) and *alpha-tubulin* (CZ5725; CZ6168); the forward primer is the same for both since it anneals to the splice leader. Aliquots (10μl) were removed after 27, 31 and 36 cycles and analysed by agarose gel electrophoresis.

To determine if the regulation of the *EP* mRNA by RBP10 depends on the 26mer instability element, a stem-loop construct (pHD1984) targeting *RBP10* was transfected into the two cell lines [45]. *RBP10* RNAi was induced using 100ng/ml tetracycline for 17h or 24h and CAT activity was measured.

### RBP10 and trypanosome differentiation

For bloodstream-procyclic form conversion, pleomorphic AnTat 1.1 bloodstream form cells with stem-loop RNAI (pHD1984) targeting *RBP10* were used. 17h after RNAi induction, the cells were pelleted, resuspended (~8x10^5^ cells/ml) in procyclic form (MEM-pros) medium and incubated at 27°C. As positive controls, wild type or uninduced cells (2x10^6^ cells/ml) were treated with 6mM cis-aconitate (Sigma) at 27°C; after 17h the cells were transferred into procyclic form media (~8x10^5^ cells/ml) and maintained at 27°C. Samples were taken at different times for Western blotting (~5x10^6^) and for morphological analysis.

To convert procyclic to bloodstream forms, AnTat 1.1 bloodstream form cells with an inducible RBP10-myc construct (pHD2098) were differentiated to procyclic forms using cis-aconitate as described above. The cells were cultured in presence of hygromycin (8μg/ml) and phleomycin (0.2μg/ml) for more than 3 months to generate well-established procyclic forms. RBP10-myc was induced using 100ng/ml tetracycline. Marker proteins were detected by Western blot. VSG transcripts were detected by semi-quantitative RT-PCR as previously described [49] using primers CZ6308/CZ6309. To obtain growing bloodstream forms, RBP10-myc was induced for 48 hours, the cells were pelleted, resuspended (2x10^5^ cells/ml) in HMI-9 medium and then incubated at 37°C with 5% CO_2_. The cell density was monitored for 6 days or more; wild type or uninduced cells served as control.

### Morphological analysis

To stain surface proteins, dried smears were fixed with 100% methanol at -20°C for 15 minutes, rehydrated in 1x PBS for 15 minutes, blocked for 20 minutes with 20% FCS, then labeled with mouse anti-EP (1:500; Cedarlane, Canada), mouse anti-phospho-GPEET (1:500; Cedarlane, Canada) and rabbit anti-VSG-117 (1:500, from either G. Cross or P. Overath). Anti-BARP staining was done as described in [12], using a procyclic cell line with the *BARP* open reading frame in the procyclin locus as a positive control. For PGK staining, 2x10^6^ cells were collected by centrifugation, re-suspended, fixed in 4% paraformadehyde (in 1x PBS) for 18 minutes, sedimented again for 2 minutes, re-suspended in PBS and allowed to settle on poly-L-lysine coated slides for 30 minutes. Before staining, slides were blocked with 20% fetal calf serum (1x PBS) for 20 minutes. Cells were permeabilised with 0.2% (v/v) Triton-X 100 (in 1xPBS) for 15 minutes at room temperature, washed twice then incubated for one hour with rabbit anti PGK antibody (1:1500, [128]). The cells were washed 3 times before being stained with fluor-conjugated secondary antibody (1:500; mouse-Cy3 or rabbit-Alexa-488; Molecular probes, Eugene). Cellular DNA was stained with 100ng/ml DAPI in 1x PBS for 15 minutes. Images were taken using Olympus Cell-R microscope. Random fields were photographed (by E.M.) and analysed (by C.C.) using Fiji [129]. We attempted blind analysis but this was not possible because the differences between samples were too obvious.

For transmission electron microscopy, samples were prepared as described in[130]. Blind analysis was again attempted but was not possible due to the obvious abundant disintegrating cells in the 48h RBP10 sample. Since we had no specific expectation of the percentage of cells that might be expressing VSG, the results are probably not markedly distorted.

### Flow cytometry and Western blotting

Approximately 5x10^6^ cells were fixed with 2% formaldehyde/0.05% glutaraldehyde at 4°C for at least 1 hour. The cells were pelleted, washed twice with PBS then incubated with 200μl (2% BSA in PBS) mouse anti-EP (Cedarlane, Canada; 1:500), anti phospho-GPEET (Cedarlane, Canada; 1:500), or rabbit anti-VSG-117 (1:500) for one hour on ice. After washing twice, the cells were stained with the secondary antibody (1:500; mouse-Cy5 or rabbit-Alexa-488; Molecular probes, Eugene) for one hour. Cells stained only with the secondary antibody and the unstained cells served as the negative controls. Flow cytometry was performed with BD FACSCanto II flow cytometer and the data analysed using FlowJo software (TreeStar Inc.).

Western blotting was done as previously described [119]; Antibodies were: anti-RBP10 (rat, 1:2000, [45]), anti-HNRNPH (rabbit,1:5000, [28]), anti-TAO (rabbit,1:100, [128]), anti-EP (mouse,1:2000, Cedarlane, Canada), anti-phospho-GPEET (mouse,1:2000, Cedarlane, Canada), anti-BARP (rabbit,1:2500, [12]), and anti-PIP39 (rabbit,1:1000, [69]) and anti-PAD1 (rabbit,1:1000, [68] with electrophoresis under non-denaturing conditions).

### Ethical approval

The research did not involve humans.

## Supporting information

S1 Text 3'-untranslated regions of mRNAs that were at least 3x enriched in both RBP10 pull-downs. (DOCX)

S2 Text 3'-untranslated regions of mRNAs that were less than 0.7x enriched in both RBP10 pull-downs. (DOC)

S3 Text RNASeq contigs containing the *VSG* 3'-UTR motif. The motif is in red, non-coding regions in lower case and *VSG* coding regions in upper case. Translations are below.
(DOCX)

S4 Text Three contigs from the EATRO1125 genome that had a metacyclic promoter and complete VSG CDS. (TXT)

S1 Fig Cell lines and RNA binding. A. LambdaN-RBP10 expression in bloodstream forms: Western blot showing inducible (24h) expression of full length lambdaN-RBP10 or six different fragments (F1-6) of RBP10 fused with lambdaN peptide on the N-terminus and a myc tag on the C-terminus. Samples from two independent clones are shown. B. Characterisation of bloodstream-form trypanosomes with a sequence encoding an N-terminal tandem affinity purification tag integrated in frame with the *RBP10* coding region. The upper panel is a western blot showing the presence or absence of TAP-RBP10 and native RBP10, with genotypes above the lanes. The lower panel is a cumulative growth plot for the cell line used in the interactome experiments. C. Scatter plot showing average reads per million for bound vs. unbound RNAs. The most prominent enriched spots are labelled, with functional designations where available. "AT" = putative adenosine transporter. This plot does not allow for variations between samples, so some labelled mRNAs may not be in the final enriched list. One spot that was clearly less in the bound fraction is also labelled. The plot shows that many mRNAs were enriched in the bound fraction but only one (labelled) was more than 4-fold depleted. D. Analysis of binding of individual mRNAs to RBP10. The transcripts were split into groups based on the ratios of bound/unbound reads per million reads; the number of different open reading frames in each class is on the y axis. E. RNAi cell line: Western blots showing the time course of RBP10 decrease in Lister 427 bloodstream forms after tetracycline addition F. RBP10-myc expression in procyclic forms: Western blots showing the time course of expression of RBP10-myc in Lister 427 procyclic forms after tetracycline addition.
(PDF)

S2 Fig Alignment of RBP10 sequences from different species. Sequence alignment was done with the Megalign Pro application of DNAStar. using MUSCLE [131], except that a single gap was introduced to make the last four amino acids from *T*. *brucei* and *T*. *congolense* match *T*. *vivax*. The sequence IDs and colour code are given at the top of page 1. The RNP1 and RNP2 motifs from the RRM are indicated (red), as are mapped phosphorylation sites (outlined text). A purple bar show the position of the C-terminal region characterised in Fig 1. An evolutionary tree with uncorrected pairwise distances is at the bottom of page 2.
(PDF)

S3 Fig Pairwise interaction experiments. **A, B**: Pairwise yeast two-hybrid screen between RBP10 and translation factors. **A)** Plates showing interactions between RBP10 as bait and the translation factors EF1 α, EF2, eRF1 eRF3, eIF5A, eIF2B as the prey. Cells diluted 1:10 or 1:100 were grown on medium and high stringency nutrient selection media. P53 and SV40 T-antigen interaction served as positive control. **B)** Bait and prey proteins expression were detected using anti-myc and anti-HA antibodies respectively. **C, D**: Confirmation of the genome wide yeast two-hybrid data using full-length prey inserts. **C)** Plates showing pairwise interactions of RBP10 as prey and RBP26, ZC3H22, Tb927.10.10050, DRBD12 as baits. As a negative control SV40 T-antigen (T) was used as prey (lanes 2 & 4); the asterisk (*) highlights the auto activating baits. P53/T interaction served as positive control. The cells were selected on quadruple dropout medium (QDO); blue colonies highlight addition confirmation by alpha-galactosidase assay. **D)** Reciprocal interaction. As in (A), but using RBP10 as bait. Screening using Lamin (lanes 2 & 4) as bait served as a negative control. **E, F**: Co-immunoprecipitation of RBP26 with RBP10 (and vice-versa). **E)** Co-immunoprecipitation with α-myc beads using extracts from cells expressing V5-RBP26, with (lanes 4–6) or without (lanes 1–3) additional expression of RBP10 myc. The precipitated proteins were detected by Western blotting using anti-myc, and anti-V5 antibodies. Ponceau staining served as a loading control. I: input, Un: unbound (both 3% of the lysate) and E: eluate (50% of the boiled beads in sample buffer). **F)** Reciprocal co-immunoprecipitation using α-V5 beads.
(PDF)

S4 Fig RNAi targeting *RBP10* in bloodstream forms: Samples used for RNASeq. A. Typical sucrose gradient profiles of samples. B. Total mRNA in the sucrose gradient fractions, detected using the spliced leader probe. C. Average quantitation of the spliced leader signal.
(PDF)

S5 Fig Expression of RBP10 in procyclic forms: Samples used for RNASeq. A. Typical Sucrose gradient profiles of samples. B. Total mRNA in the sucrose gradient fractions, detected using the spliced leader probe, C. Average quantitation of the spliced leader signal.
(PDF)

S6 Fig Effects of RNAi on translation. A. Scatter plot comparing the effect of *rbp10* RNAi on total RNA (x axis) with the effect on polysomal RNA (y axis) for mRNAs that were at least 3x enriched according to DeSeq; all P-values were less than 8E-5. The black line is the regression line and the red and green lines show the 95% confidence limits for the data. The blue shadow encloses total mRNAs that were less than 1.5x affected, and the pink shadow encloses polysomal RNAs that were less than 1.5x affected. The cyan line is perfect correlation. The box beneath the graph lists relevant TritrypDB accession numbers. The gene numbers on the plot are also accession numbers, with "Tb927." removed. B. As (B), but here the y axis shows the effect of RNAi on the percentage of the mRNA in polysomes.
(PDF)

S7 Fig Relocation of phosphoglycerate kinase during differentiation. Cells were fixed with fomaldehyde, permeabilized with triton x-100, and stained using a polyclonal antibody to phosphglycerate kinase (PGK). The grey-scale panels show PGK alone and the differential interference contrast panels show DNA in cyan and PGK in magenta. A. Procyclic forms. B. Bloodstream forms. C. Bloodstream forms after incubation with cis aconitate for 17h at 27°C. D. Bloodstream forms with 17h *rbp10* RNAi followed by culture for 3 days under procyclic-form culture conditions. The selection shows one procyclic-like trypanosome (left) and one which still has bloodstream-form morphology (right). E. Procyclic forms after 2 days induction of expression of RBP10-myc. Cells with terminal kinetoplasts are labelled "b" and cells with more procyclic morphology are labelled "p". Cells that appear to be dividing are indicated with asterisks. A very abnormal cell is indicated with "?". Cells if unclear status are not labelled.
(PDF)

S8 Fig Morphological analysis of differentiation. Cells were treated as indicated, fixed, and stained for DNA (cyan), EP-procyclin, phopho-GPEET procyclin, or VSG. (magenta). The posterior-kinetoplast and kinetoplast-nucleus distances were measured in Image J and the ratios calculated. A. Summary of all results. This is the same as Fig 5H, but all of the outliers are included, and the numbers of cells analysed are below each lane. BS—normal bloodstream forms; PC- normal procyclic forms; CA - 17h cis-aconitate pretreatment; RNAi or Ri; 17h *rbp10* induction; +RBP10—induced expression or RBP10 for 2 days. B. Cell distributions after cis-aconitate-induced differentiation. The x axis shows the P-K/K-N ration, and y-axis shows the percentage of cells with that ratio. Controls are bloodstream forms (magenta) and established procyclic forms (green). The other lines are for 3 and 6 days after transfer to procyclic medium (MEM). C. As B, but with transfer after 17h *rbp10* RNAi. D. Phospho-GPEET expression 3 days after cis-aconitate-stimulated differentiation. The cells indicated with the cyan line (BS_CA_3d MEM) were subdivided into GPEET positive and GPEET negative. E. Phospho-GPEET expression 3 days after RNAi-stimulated differentiation; other details as in D. F. Cell distributions in procyclic forms after induced expression of RBP10. G. As (F), but also showing staining with anti-phospho-GPEET and anti-VSG117. Cells with no GPEET have bloodstream-form kinetoplast positions, and a subset of them cross-reacts with anti-VSG117 antibodies. The remainder may express VSGs that do not cross react with the anti-117 antibody. (Note that the plotted "GPEET negative" parasites will include VSG117-positive cells, because the counts were derived from different images. Counting of images of double-stained smears was avoided because of potential bleed from very high signals).
(PDF)

S9 Fig Additional electron microscopy images. A. A procyclic trypanosome culture 48h after induction of expression of RBP10 expression. VSG coats are quite difficult to distinguish at this magnification but possible candidates are indicated by thin arrows. The asterisks indicate degenerating cells. In some cases plasma membranes look slightly thickened, these could be cells that are in the process of acquiring a coat. Higher-resolution images were used for quantitation; the images here have reduced resolution in order to decrease the file size. B. Close-up of cells with (thin arrows) and without (thick arrows) VSG coats from a different field. C. Procyclic forms from a normally-growing culture. These cells seem to have membrane blebs. D. A bloodstream-form trypanosome from a normal culture.
(PDF)

S1 Table Protein-protein interactions of RBP10. For legend see the top sheet 0.
(XLSX)

S2 Table RNASeq results for RNA that was bound to RBP10. For legend see the top sheet 0.
(XLS)

S3 Table RNASeq results for Lister 427 with altered RBP10 expression. For legend see the top sheet 0.
(XLS)

S4 Table RNASeq results for EATRO1125 procyclic forms with induced RBP10 expression. For legend see the top sheet 0.
(XLSX)

S5 Table VSGs expressed by EATRO1125 bloodstream forms and RBP10-expressing procyclic forms. For legend see the top sheet 0.
(XLS)

S6 Table Plasmids and oligonucleotides used for this work. (XLS)
