# Supplementary material for: Expression of the RNA-binding protein RBP10 promotes the bloodstream-form differentiation state in Trypanosoma brucei
Source: PLoS Pathog. 2017 Aug 11;13(8):e1006560. doi: 10.1371/journal.ppat.1006560 (PMC5568443; doi:10.1371/journal.ppat.1006560)
Supplement: S1 Text — (DOCX) [file ppat.1006560.s001.docx]

**Supplementary Text S1: 3' untranslated regions.**

**This document has 3 sections.**

**Section 1 includes all procyclin 3'-UTRs, the 3'-UTR of PGKB, and the section of the THT2 3'-UTR which is absent from other (bloodstream-form-specific) THT mRNAs. The TATTTTTT element is in red and a truncated version, TATTTTT, is in orange.**

**Section 2 is the 3'-UTRs from Table 3, sheet 2, which were used to look for the conserved motif, which is in red.**

**Section 3 are 3'-UTRs that were manually corrected on the basis of the densities of RNASeq reads in tritrypDB.**

**SECTION 1: PROCYCLINS, PGKB, THT2**

Characterised elements are highlighted

>GPEET (427 strain)

Glycerol-induced expression in procyclic forms

GCGGATGCAAGCGTGTAAAGCGCCTCGGAGGAACGAAACCCCTTTGAAAAGGTGGCTTTCGTTTATATATTCTCCATCTGGTGCATCGTGTTTATTTCGTATTATTCCCTGTCAAAAGCTGCGGATATTCATTTAATATTCTTTTCGTTATATATTTTTAGTTCTATCCTTTATTTTATCCGTTTTAATCGTCTTCTGAGACCCACAGCCCTGTAGATTTCTGTGATGTTTCGGTTGCGTATTCCATAATTTTAAGCGTTTCACTTCTATTTTTTTTCATTCCTTTG

GPEET (927 strain)

GCGGATGCAAGCGTGTGAAGCGCCTCGGAGGAACGAAACCCCTTTGAAAAGGTGGCTTTCGTTTATATATTCTCCATCTGGTGCATCGTGTTTATTTCGTATTATTCCCTGTCAAAAGCTGCGGATATTCATTTAATATTCTTTTCGTTATATTTTTTTAGTTCTATCCTTTATTTTATCCGTTTTAATCGTCTTCTGAGACCCACAGCCCTGTAGATTTCTGTGATGTTTCGGTTGCGTATTCCATAATTTTAAGCGTTTCACTTCTATTTTTTTTCATTCCTTTGAATTTGGATCTT

>EP3

CTGATGTAAAGAGAAATCTTTTTGGAGGGTCAAAACTTTTTAACAAGGGTGACCCCAATTGTTTATTTAACCCTTCCTTTGGCGCGATGCGCCGCTTCATGGCGTTCCTTTGTGACAGGACACTGACACGCATAATATTTTTTTCAACCCATTTTTTTGAGTTCTATTTTTTGTTTTATGTCTTGGAACCGTCGGCGGTAGCACGCAGCCCTGTAGACTTCTGCGTGTGCCTGTCAATGTGTTCCTTGGCTCTTTTCAAACGGCTTCTTTTATCTTACCGTTCATA

>EP1 26mer deletion (nt 134-159 inclusive) increases expression in bloodstream forms

(Partial deletions or mutations affecting only 1 element also have almost the same effect as deleting both. Element does not function in a different context.)

GCGGATGCAAGCGTGTAAAGCGCCTCGGAGGAACGAAACCCTTTGAAAAGGTTCCTTTCATTTATATCGCCTCCATATGGTGCATCGTGTTTGTTTCCTGCTGTTTCTTGTAAAACAAGTGTGGACATTCATTTAATATTTTTTCGTTATATTTTTTTGGTGACATCCTTTCTAATGCCTTATTAACCATCGCCTGAGACCCACAGCCCTGTAGATTTCTGTGATGTTTCGGTTGCGTATTCCATAATTTTAAGCGTTTCACTTCTATTTTTTTTCATTCCTTTGAATTTGGATCTT

Mutations that also increase expression in bloodstream forms:

Deletion from the 5'-end to nt164 gave 13x increase. An internal deletion (101-173) gave 27x (schurch et al).

Hotz et al measured as % of actin, so numbers are different. Deletion to nt 197 fully relieved repression at the level of CAT activity so maybe the final element is not suppressing.

Mutation of one element increased CAT a lot (background to low to make the calculation) but RNA increase was only 2x. The two elements may work together for optimal efect.

>EP2

GCTCATGTAAACAGAAAATTCTCTGGAGGGTCAAAACTTTTTAACAAGGGTGATCCCAGTTATTTCTTTGACCTTTCCTTTGGCGCGATGCGCGCCTTATGACGTCCCTTGGCGACAGGACACTTGCATATACGTAATATTTTTTCAACCTATTTTTTTGAGTTCTATTTTTTGTTTTATGCCTCGGAACCGTCGGCGGTAGCACGCAGCCCTGTAGACTTCTGCGTGTGCCTGTCAATGTGTTCCTTCGCTTTTACCAAACGGTTTCTTCTGTAAACCATTCGTATGGTTTTGC

Deletions were done on this too by Hotz et al. Deletion to 159 gave 85% CAt and 64% mRNA, deletion to 200 gave a (probably non-significant) increased CAT to 106% but mRNA stayed at 64%.

>PGKB deletion increases expression in bloodstream forms

(The sequence shown in the paper by Quilada et al (2002) suggests that the deleted region did not include the second UAUUUUUU element. However, our sequence records show that both elements were deleted.)

TGTACATCAGGCGAAGGGTTTGTTTTATGGAATTGTGTTTTTTAGTCCTTTTTATTTGTTGGTTAGGTATTGGTTCGTACGTGACTATTATTTTTTTTTTAGGATAACATTTATGTTTTTTCTCTACTCATTTTATTTTTTGGTACTATGTATCGATTGCACAGTAATATTTCAATCGCTATGATATTTCTATCTTTTTGATCGTTCTACTGTGTAACTTTGTGTTTATCTTTGTTACTTCACTCTTTTTTCACTCAAATCGTTTGGGCTGCAGGCTCAGCTCTTTGGTGATATCAAAGCATAATTGCTGCGGAGATACGTTTTTCCACCTAATAAGTAATTGTGATACAAGA

>THT2A: 3'-UTR sequence that is found in the THT2 mRNA, but not in THT1 mRNA.

CCGATATTGTTAGAGGGCGGGAATTCCCCTCGATGTTTACGTTGTGAGGTAAATAAAACAGGATTTTCTGTTGGGCCTTTGGATTTGACATTTTAAGGTTATCTCCCATGTCCCTATTTTAACGAGGCAGCAATCGCTATAGTGTATCTGATGTACCAATTGATGAGGCTGACCGTTTGTTTTTCCAGACTGTGGTTAGAATTGTAACTTGATTCCATATTGTTTTAATGAGCGTACGGATTATGCCTTAGCGGATATTACTGTTATTTTTCTTTTAACTGAGCGAGTTAATTTACTGATTCTATTTCTTTGTAATTTAAAAGTTGCTTAATACCACGCTCTTTTCGATTATATTATCGATCACTTTGTTCTCTTTCATTTTGTCGACCTATGAATATTCTTGGTTTTCTGTCTTGTTGCACGGCAATA

**SECTION 2: DATASET USED FOR SEARCHING**

>Tb927.10.10250 | Trypanosoma brucei TREU927 | EP2 procyclin (EP2) | genomic | Tb927_10_v5.1 reverse | (geneCodeEnd+0 to geneEnd+0) | length=301

AGCTCATGTAAACAGAAAATTCTCTGGGGGGTCAAAACTTTTTAACAAGGGTGGCCCCAG

TTATTTCTTTGACCTTTCCTTTGGCGCGATGCGCCCGCTTTATGACGTCCCTTGGCGACA

GGACACTTGCATATACGTAATATTTTTTTTCAACCTATTTTTTTGAGTTCTATTTTTTTT

TTTATGCCTCGGAACCGTCGGCGGTAGCACGCAGCCCTGTAGACTTCTGCGTGTGCCTGT

CAATGTGTTCCTTGGCTTCTACCAAACGGTTTCTTCTGTAAACCATTCGTATGGTTTTGT

C

>Tb927.10.760 | Trypanosoma brucei TREU927 | hypothetical protein, conserved | genomic | Tb927_10_v5.1 forward | (geneCodeEnd+0 to geneEnd+0) | length=495

AATCATGTTTTTTTATTTTATTTTTTATTTTATTTTTATTTTGGGGGGGGGACTCTATGG

AGTGGGAATGGATGGGTTTTCTTCACCATGTAGTTTAAACGCGATGCAACTTTGGCTTGC

GTTACTCAAGGAGACGTCTCGTATGTTTTACAGGCAATATATGCGTGACTGCCTCGTCTA

GATTGTTTTTGGTGTTTCTTTATATATATATATATATATATTTTGGTGGAAGGTGGGGGA

GGAAATCATTGTTGGGAAGAAGGGAGGTTAATGGAAGTTCCACCACGTCTCGTCATTTTT

GGAAATTTTTTTTTCTTATCGATGCGAAATACCACCGCACTATTTGCCTACGGTGATGGT

GTAGTTGTATATTTTCCCCTACTTCCAGATATGCTTGTTTTATTTTACATTTGTGTAGGT

AGATAATGATGAGGCTGCTGGACGCAGCAGTGATGTGAAGCAGAGTAAGCGTAAGATAAG

GTTAATACGAATTAT

>Tb927.11.15410 | Trypanosoma brucei TREU927 | hypothetical protein, conserved | genomic | Tb927_11_v5.1 reverse | (geneCodeEnd+0 to geneEnd+0) | length=993

AAGATTTTTATTCTATGTTGTTTTTGCACCCGTATTTTTTATTCTTATTTTTATTTTGAA

TACCTCCCTTTCCTTTCGCCTTAATTTTCAATATCGTTAAACGAAAAAATAGGGGAACGC

GGCAAACGGGCGTAACAGGGCGTGGGGATGGATTGTGACTTGGTTGTATTGAATTCAGAA

CTCAGTCACTTACGCTTTTGTTTTATTTCATACGCGCTGGTTGTTTCTAGTGGCGAAGTT

CGTATTGATATATACAAGGCTGAGACGTTCCTCCACGTTGTCATAGTTTTTATTCGTCAT

TAAAAAGTTTTGTTTTATGCCGCCGTTGCTCATTTACTATTATTATTATTATTTATGCCG

TGTTTCATTAATTTAGTTGTTTATCTTTTATTTATTTTTTGCTCGACCCATAAGCGTCTG

CACGAATGCCTCCGCTGTTTACGTGGAGTTCGGATCAAGTGGACCAAGACATTCCACTCC

GTTTGATAACCGGGGAAGGGGAAATTGGATCGGTGAGCTTCCGTATATTACGCCTTTCAT

TGCATCTCTGCTCTCTCTCTCTCTTGGAATCAACCACTAGCCCAAACCATAAAGCTCATA

TGAACTACCAATATATTTTCGGTGCCCACGAATAAATCTTATGCGCACACGTACGATTTC

GACTTGCGCCACAGCAGCGGGATTAGAAAAAGGCCACGGAACGGTGCTCTGAGTTACATT

CCGTGCCACGATGAGTTGAGTTAAGGACCGAGACTAACTTACCTTGTAAAAACTAGAAAC

TTTTTCTAGGGTGCCCGGCGCGGCTGCGGATGAGTATTTATTTTTTCCTTCCAGTTGTTA

TATTCCTTCCGTTTAAACGAGAGAACGCTAAATATTTTTCCATGCTACTGCCATCCAATC

ACATTTCACGGACAGTGACGTATGTTTCCCCCCTTCTGACAACGGTCTCGCCCCACCCAC

GTATTTTCACTTGTATGTGGATTTTTTTTCGGT

>Tb927.11.5090 | Trypanosoma brucei TREU927 | aspartate aminotransferase, mitochondrial | genomic | Tb927_11_v5.1 forward | (geneCodeEnd+0 to geneEnd+0) | length=2226

ATAGGAGAATGTTGCATATATATATATATATCTGTTACATGCCGTGACATTCTTCTCCGT

TCTTAACGCCTTCCCCTTCTTCGTAGCGTTTGACTTACTTTTTTCCGTTCTTAGTGTTAT

CAACTTTATATATATAATTTTTCAATGCTGCTGATGACTTGCAGTGTGTTTTTTACATTT

TCCTCCTCCTTTTTTGTCCTTTCCGTTACTCTTCTTTTTTTTTCCTTTTGGAGACTAAGT

GAGTTCATTTGAACTTTCTTGGATGTAAAATATATTTGTTTTTGTTTAAATATATTTGTT

GCGGTGTTTTGACACTCGTTATTTTTTTTTGATTATTTTCTACTACTTTGCCTATAAATG

TGCTTTTCGTCCGATACTTAACGGGCACGGTGCGGAGAGAATGAAGAATAAAAGAAATGA

TTAAAACCCCACTCCCGCTGCGGGAATGTTGGGTCCTATGCATTTCAAAAATAATAAATG

ATAACCACAAGCGTGGAGAGGTGGGAGTAAAGAAGAGCGAATGAGAACAAGTATGTTTTA

ATCTGTCATGTGCATAAAAACAAGTAGATATACATTTATGTATTTATGCATACATACAAA

TATATCTGTAAATAAAAACATATATGTTAAAAGCAGTAGAAGGGCAGAGGGTTACTTCGC

CTCTATTAGGCGGTAGGGCAACTTTATTTCGCTGATCCCGCAGGGGTTTTCTCTTTTTCT

CAATTTCCTACGGTATTGTCAGCACTTCCTTTTTTTTTTGCTGTTATCGACATTTTTCGT

TATGAAATATATATATATATATATTCGTCTTCTTGATTTAGTTTTCATTTGGTTGCTTGG

GGAGGGGGAAAAAAGACAATTAATTGATGTGTGTTGCACCACCTGTCGCCATTTTTTTAA

ATTGTTTCATTGCATTGATCATCCCACGGAAACCTCCTTACATTTTCATGTACGTGAAGC

AAAAGAGAAATAAACGCATTAACCTTACTCACTTCCATTAAGTGAAAGTACTGTGTATTT

CAATTTCATCTTCCTTTACGGGCTGGTACCGATCTCTTTTTAGTAAAAAAAACAGGGGAG

GCGATTACATTTCTTAAACTGCAGAGGGATGTGCTGCTGAAGAAGGGGGGAAGGGGAAGG

AGATGAGAGAGAGAGAACATGATAAAGTGGATGAGGTGATATCGCGGCAAAAACAACAAC

AACAACAACAACAGCAGCAGCAGCGGCAGCAAAGTCTGGGTTAAGACGTGTAGTTATGTG

GAATGAAATTGTTAAATGGTTGTAATTCTTGGGTGTGCACAAGGGACACCTGTGATTGGT

AACTGGACAGCTGGAGGTCGTTTTTACATCCTTGTTAATTTGTGTGTGTGTGTGTGTGTA

TGTGTGAAAGTAAAAGTAAAAAATAAAAAATAAATAATGAAAAGCATATGCATATACATA

TATGTAAACACGAGTGCATGTACATACATGTGTTTTGCTTATTTTCTTTTTCTTTTTGTC

GTTGTCATTGACAACCATTTCCATCCGTACCAAATCTACCTTTTTTTAAATTTTTTTCCC

GCTTCGCGTGGGTGTTTTATTTGGGTTGCCATGGAGTAAGGGTCACGAAAGGAATAACTG

TGCCTTTTAACGGGGTTAAATGCATACATGTAAACAACACGGGTTGGTTAAAACTATGAA

CGTTTGAGGGGTAAAATAACACTAATAACATGTGACGGAACTTTCTCTCTGCTCCTATGT

ATTAACTCACGGGTACGTAATCTCTACTTTTTGTTTTATAGTGGCCTTTTTTTCCCTCTC

CCCTCCTCTCCCTCTTCGCACATTTCTTTTTTTTAGGTATTGGTTGGGTGCATACCCAAG

AAAGTAGAGACACCTTCTAACGTTCAGTTTGATATTCTCCCCCGTGGCACACAGTGGGGG

ACCCACTTGAAAAGTTTGCGATGGTAACAAGGGGAGGATGAAATGAGGAGGTAAAGAAAC

TTGTGTGGGTTTCCGTAATGCCTGTAACAGAAGGTCAAGGAACTGTAAATACAAACACAC

ACAAAAAAAGAGGAAGATTCAGTTAGACCTAAAGTCTGCCGTTAACACCTTTCCATCCCC

CCCGTGAAGCGGCATTTATGTGTGTATTTGTGTGAGGAAGGGAAGTGTTGGGAGCTAAAC

GGTGAGGCAGCGGCCCGCTTATGTTTAGTAGAACTTATCATCTCATACATCCTCCTATAA

GAACAT

>Tb927.2.2140 | Trypanosoma brucei TREU927 | hypothetical protein, conserved | genomic | Tb927_02_v5.1 reverse | (geneCodeEnd+0 to geneEnd+0) | length=224

AACGGAAATGTTTGCGTGTCTTTATTTATTTATTTATTTATGTTTTTTTCCCCCATCCCC

CAATTTTATATTATTGTTATTGTTATTATTTTCTTGTTGTTGTCGTTTTATTGAGGGGGG

GGAGGGAGGAGGCAAGTGGATGGCGCTCAGTTAATGGAAATGCGTGAGTAAATTAATTCA

AGTACCGTGTCGTCCTTTTAAACTCACATGAATACTTATTTTGC

>Tb927.3.5410 | Trypanosoma brucei TREU927 | hypothetical protein, conserved | genomic | Tb927_03_v5.1 forward | (geneCodeEnd+0 to geneEnd+0) | length=173

ATTAACCTTTTTTTTTGTTATTTTTTTAATCATCACTATTAATATCATCATAATTATTAA

TATACATATATATTTTTTCTCTTCTGCCTGAGGGCTACATCATGTGTTTTGCGTAAAAGT

AAGATTCCCCTTAACCTGTTGTTGGAAATCTGTCTCCATTGCGAATGCTGGCC

>Tb927.5.1050 | Trypanosoma brucei TREU927 | hypothetical protein | genomic | Tb927_05_v5.1 forward | (geneCodeEnd+0 to geneEnd+0) | length=2232

ATCGGTGACACTCTCTGCCGTGGGTCTGTCCAATATATATATATATATATATATATATGT

ATATGTGTTGAAAAATTACTTATTCGCACAGAAACATTCCTCACAATCCCGTCCTCGCGG

CGACGTGATGCTCTTGGAAACTCCAGGAGAAAGAACAGGTGCCGTGCTTTTGGATTTCTC

TGAAACAAGTGCCGACTTTAGGCAAGTACATGTGGTTCTTTTTCGTTGGCCGCTTCGTAG

CGAATCGTATTGTCCTTTTCAGTAGATGTGTAACGCAATTGTATGCTACTAGCAATTACT

TATTATTGTTATTGATTTTTTTAAAAATTTTGTTGGGTCATTTTTGGCAGTATTGGTCTC

TTACTTTTCAAATGGTGCAGTAATGGGGAGGAGCGCGATGTGTAACATGCGTGTGTATTG

TACACTCATGGAAAAGGAAAGGTGGGGGCTTCGAACGTATAATGTGGATATGAAGAGGAG

GAGAGGAGGAGGGGGAAGGGAACATTAGTTTTGATGGAAGTTAAGATGAGAGGACCACAA

CAATGATGAAAGTGCTGCCGGTGTTTAAAGAAGGGGGTTTGGGACACTTCTTTCCAACCC

TTTTTTGATCCGTGCTTTTGTTTCGTCTCCTGGTCCCTCCATCCTCGACTCCGAACACAT

CCGGCACTGTTTGTCACTTATATGCGTTTGCGTTTAACGGCTCACCTCTTTTTTTTTTTT

TGAATCCCTGTGGTGCTAAAACAATGTTGAGCTGCCCTTCTCTTGCGCGTTGCGTGCGTG

TCACCACAGTGCTATACATCACTTTATTTTATCTTATTTTATTGTATTTGTTTTGTTTCC

AACGAGAGTTATCGGGGAAGCGGGGGGGGGAAGAGACGGCTGAGCGCAATGTTATAAATT

GCCACAGTCGCTTCATTTCCCCATTTTTCTTATTTTTTCTCTCTTATTTTTTTTTTAAAC

AAGTTTTCTGAATGATGAAGATACCACTGTTGAATAAAATGTACGCATGAAAGTATTTTC

TGTGTGGTTATGTATTTAATTTACTAGTCTACTTTCCCCATCTGTCCAGTCGTCTCAGCA

CCGTTCTTTGCTGTTCACGGCAGCCGCGGAAGCTACGCTTTCGTATTTTGTTTTCACTTT

TGTTTGCTGTGATATTTATTTGTTTAAGCATTTATATGAAAACCTTTTTTTTCTTTAAAA

AAATTGAAAACTTGACTTATTCCCTACAACCCGGGTTTAAATGTCTCAGGAAAAAAATTT

CTCGCCTTCTTTGCCCCCGTAGATAAATATATATATATATATATATATATATATATATAT

GAATACAGGAGGTGTTGAAGTGCTTTCATGTTCCCCTTACTTTCGGTTGTTTTGTGTTCG

CTGCAATCGTACTGTTGGTGGTGAAGTTGCCGCTGTTTATATCTTTTCCTAAATTTCCGT

TTCATTTATTTTATCTTCGCTAACTTATATGCCTCTACGTGTCGTTTTTTCTTTCCCTGA

TCCTCACACATATTTTAAGTATTTGTCTTGCGGGAAGTCTAAATGATCATTTATACATTT

GCATATTTGCAATTCATATATATATATATATATATATATATATGTGTGTGTGTGTATATA

TCAACAGAAATGTATTGTTGCGTTTATGTTTGCTGTGTCTGTTTACCACACTGGAAACTC

ACGTATATATGCACTTCCTCATTTATTTTCGTCTACCGTACACATGCGTTTGCGCGACTA

AAGGTAGGAAGTCAAGAGTTCATTACAAACGGCACCAAAAGAAAGAAATTAAGAAATAGA

GAGTAAACAAAAATCAGGAAAGTGTTGCCAAGGAAAAGAAAAAAGGGAGAAGTGTAAATA

AGGCAGTAAACAAATAGTGTGAAAGGGTAGTGCATTCCAAAAGTAACAGGAAAATAAGTA

AATAAAAACATTGAAGGGTGCAATCACTACGAGTGAAAACAACATTAATACAAATAACGA

CAGTAACAATACAGCCCATACATATATATAAGAGGAAGCTGGAGAGGGGGAAAAAGCACA

GCAATAGCAACTAAAAACACAAAACCGAATCAAAAACAATAAAGCGGAAGTGAATCACAG

CGTAAGGTAGAAGTAACGTGAGAAAACACAAACAGGGTGAGTACGCCGTAAAAGTAGCAG

CGGCATAACCGGCAGCAGTATCAGTGACAGCTTTAGTATCACAAAGGGAATTTATTTATC

GTGAAACCGGCC

>Tb927.5.2350 | Trypanosoma brucei TREU927 | glycosyltransferase (GlcNAc), putative | genomic | Tb927_05_v5.1 forward | (geneCodeEnd+0 to geneEnd+0) | length=1090

GGCTCTCCCGCCTCCTCATGTACATGATTGTGGTGAGTTGAGGAGTGAAAATCCAAAATA

CGAAATTAGTGCAAATTCCATTATATTCTGTTTTTTTTTTCTGTTGTTGTTCTTGTGTGT

GTGTGTGGAGGCAGCAAAAATAAGGTAGCGAGGTTGAGAAGGCAAATGATGTTACTGACG

AAATTCAATTGGTCGCGTTTATTTGGACTTTGGCAACCTCGACTTTCTCTTCTTATAAGT

TTTGTTGGCGTCTACATTTTATTTCTACAGTGCCGGTTGCAGAAACTTGCTCATTATTTT

CCTTCCATCTTATTTTTCTTTTTCGTGTGTACATATTTTTACAAAACTGTATTCCACGCC

GTTTTCGGTGCCATTTATTCTTTCGATTCCGTGTACTTCCTCCTAGGTGGCAAAGCATCT

TTTTTTTTGTTCGTGTCATTGTTAAAATAATGATAATAGTTATTATTATTATTATATATA

TTTTTTGTGTGCGTGTGGACACTAGTGGATGGCTGTAACCCTTCCTTTATGTTTTCTTGA

ATCCTTTCTTTTTAATTCTCTATTGTTGTTTGTTTCACCGCATGATTTTACCTGGTGAGC

GCCAGCCATTGGCGGTGGGTTCCTTTTTTTTCTTTGTTGTACTTGAGAAGGTACCTGTTT

GGTTATTGCAAAGTCGTTACTGACATCGATGGGGGGACCACTGGGAAGCAATGGGATGCG

GAAGCAGATGAAGGAGAGTTTCATCTGCACTTTTTCAAGTAGTTATATTGCAGAGTGCCT

TTGTTTATATTTATTTACCTATTGTTTGTTTGTTTTTGTAGAAACAAAACCTCTTCTTCC

TCTCTAGCACACGGTAGTGTTCGCTTTGTCAGTGAATGAGGAAAATTCCCCTAGTCATGT

GAGTTTATAACAGCGCATATATATTTTTTTCATTATTTTACACAAGTTCATACACAAAAG

TATCATCTATCTATATTTGTTTATGTCCGTTTGTCTACCCATCGCACGAAACAGCTCTGT

AAGTTATGCTATAATGTTACGGTGGCCGTAAGCTGTCGGGTTGTCGGACAACTTGAAAAA

TTCCTTTTTT

>Tb927.6.860 | Trypanosoma brucei TREU927 | hypothetical protein | genomic | Tb927_06_v5.1 reverse | (geneCodeEnd+0 to geneEnd+0) | length=3199

AAGGAATGACGACTCTTCTTTTCCTTTATTCAAGTGCCGTATACAAGTGTACAATACGAA

GGGAGTTGGCGCGCGTATGTAAATATAAATATATATATATATATTTATTTATTTATGCAC

GTATGTACAATATTCATGTACCGCCATTTAACTTTATCTGGTTGTTTTGTTAGTTGCTCT

TCCCCTCGTATTTGTTTTTTTTCTTTTTGCTCTTTTTTTTGTTTTGTTTATTTGTCGTTT

AATAACTTTCTCTCAACCCCTTTCGCCCCTTGTTTTTATATTACACCACCGTAACGAGAA

GTGATGTGTCCAATTGGCTGCCAAGAAATAATGAAAGAAAACGGTATTCCTCCTTTTATT

TTTGTGTGTGTGTGTGTGTTATTTTATTTTGTCGTTTGAACTTTTTTTTGCCCTATTTTT

AATCGATATGTTGTTTTGCTTTGTGTTGAAAAGGTGGTGTTTAAAGGGTTTATTAAATGC

AAGCCTCTCTCTCTCTCTCTCACACACACACACAAACATGAAAAGAAAAGAGAAGAGAAG

AGGAACAAAAGAAGAAGAAATTAACAAAATGAATGAATGAATCAATCAAACGACATAAAA

TTATATACTTATACATAAACGTGCATTGCTTCCATTGCGGTTGGGATGATTGTTCAGACT

CCTTCATACCATCATTTCACATGGATGTTGTAACTGCACATTCATCTCAACAACCCACAC

GCAAACGTCAAATGTCATTCAAATCCAATCATTCCTCAGCCCTGTTTTCTTTTTTTTTTT

TTCTTTCCTTTAATTCTTTTATTTCTTTAGTTGTTTTTTTTTGTTTTCGTTTGTGTGTTT

GTATGTATGCATGTGTGTATTACTTTTATTGTTGTTCATATGTTATCGATATTGATGCTT

TTGTTGTTTCTGCTGTAACGCAACGATGTACACAAATATGTCTATTTGTGAGTTTTCATT

TCATTTCGTTTCGTTTCTTTTTTTTTTTGTGTGTGGGGGGGGGGAGGAGGGGGGCTTATT

AATTGTTCGATAAGTGTATGGGAGACGGTAAAGGAGATTTCTCGTTTTGTTTTGTTTCCT

CCTCATTTTTGTCGGTTGTTTTGCTTTGTTTTTTTTTTTGTTTTTAGTAAATGTGAGTCA

TATTCACTTGTGTGTACGTGTGGACAGCCTTCCTTTCCTCCTCTCCGCTAACTTTTAAAA

AAAAATGTAAAAAAAAAAAAAGACGAACTTTCTATCACGCCTTTTTCCATACCTTTTTAT

GTGCCGCACGCAAAATGTTTTTTTTTGTGTTATATGCTTATTTGTGTTAGTTTGCATGAT

TTTGTTTATGTTTATTTATTTAAGAATCTGTTTTTTTTTTTCTTTTAATGAATGCGATGT

TGGGTCATATATTCGCTCTAACTATTATTAGAGTTGTTGTTGTTGTTGTTTATTTTTCAT

GCTTTAGTACTTTAAATAACCTCCATAGAACATGATTGCGGATCCACGTACATCACATTT

ACTTTATATTTATATCGTTCGTCACGGGTTTAACCTTACATGCGTTTCGTCACGTGTGTG

GAGATTAAAAAATACATAAATATAAATAAAATAAATATAACAAATAGAAACGCATGAATG

TAACGGGAGGGTTTAGTATATATATATATATATATATATATATGTTTAATATTTATAGTG

AAAAAGAGGACGCAGCAGCAGCTGATATGTTGATTTAATGTGAGCTCTCCTACATTTTCT

TTTCTTTTACCACCCCTTTTTTGGGGGTTTGTTTTTTCCTTTTTTTTTTTTTTTGACTTC

AATATTTATTTTGTTCATATGATCCTCTCAGTTCCTCATATGCACGCAAAAAAGTACCGC

AACCTCTTTTTTTTTTATTTTTTCAATACCTCGAAGTGGTGCCGCACAAAAATTTACGTT

TATGTTTTTTGTTTACATATATGTATGTATATATATATATATATATATATATATATTGTA

AATGTTATTCGCACAGGCCGTATACAATTCATCATGTTATATTTGGAATGTTGTAATGCA

CATATGTGTTATTTTAGCGTCAATGTTTCACATATTTTGTTTTCATTTATGTGTTGTATG

CGCGTGTTGCTGTTTTTGTTTTTTACTATGTCGTATTTTATTTATTTATTTATTTTTCCA

TAAACCTGTTGTAGTAGTATCTTACTATTTTTTATCGTTGTTATAGCTGGCGTTCTCTTC

AACAGTTTCTTACTGTAGCCGCAAAGGGGGGGGGAAGGAAAAAAGAAAAAAGAAAAGTAA

GAAGAGAGAAATAAATAAATAAATACATACATAAATACATAAATAAATAAATAAATATAT

ATATATATATATATATGCGCTTCCGTATGTGAGTGTTTTGTTGTTTTTGTTTTAATCCTT

TTTATGTGTTTTACGTCCCGTCCTGTCCCGTCTCAGCAGGAGATTTTTCTTTCTTTCTTC

CTTTTTTTTCCCATCATCTTCCTCCTCTCCTCTCACTTATGTTTTCCATCACCTCCCTTG

AGAGGAAACGGGAAGCATCGCTAGTGATAATGTTGATAACGGTGGTAGTAATGACGATTA

TGAAAATGTTAAGGACGGGGAAAAAAGAAGAAAAGAATGAAGGGGGAGAAAATAAACATG

AAATATCCCCAATTGTTATTCCCAACGTTAGTGTTTACTCCTCCACCGCAACTCTTGTTG

TTGTTGTTTGTTTGTTTATTTTTTTTTTTGAGTTTTGATATGACGCAAAACGTATATGTG

CTTTTTATCGTTGGCTTTTCACCAAATTCATCCGCTTCGTTCTTTTGTTCTTCATCTATA

TTCTTTAGATTCTTCTGTTATTATCATAGTCCCAATAATAAGATAGGTGTATATATGTGT

TTGGTTCTGTACAGAAATTCACTTGGGTATGATTAAACGCAGCTGCTACTTTCATTAGGT

GCATTACGAGAGATGAGAAATAAATCAATTCAAACAATAATAATAATAATAAACAAAAAG

GAAGGACAGTAAATGAAGAAGTGTGCAATTACTTGCACATCAATATATATTTATATATGC

GTGTGTGGTTCCGTACCTTCCAGCCACGCCACCACCCCTCCGGTTTGACGAGCCATCGGT

CGCGCATCACATGAATTTTTATTATTACTTTATAGTTCATTCTATCATTTAGTTCAGTGC

TGTCGCGAGTGTAAAGTGT

>Tb927.7.1210 | Trypanosoma brucei TREU927 | regulator of chromosome condensation, putative | genomic | Tb927_07_v5.1 forward | (geneCodeEnd+0 to geneEnd+0) | length=1700

GTCCTCCTTTCAGCGTTTTTTTCATCGTTTACAGTATTGTCGTCATTTTTATTTCTTTAC

TGTTGTAACTTCCGTTATCGTGTTGTTGTTTTAATCCTTTCTTGACGCTGTTTTATTTTT

TGCCTCCTTAATTTGCCTGCAAATTGACTTCTTTGGCCCCATTCTGATGCAGAACTTACG

CGCATGTATGCGAATGTGGCCCGACAGTGAAGTGGCGGGAGCAGTCGCTCTGGTGTGTTG

TTTTACACGCGTTCGTGTGCATTTATTTTCTTTGATCTTTTGTTTAACTTTATCCCCTCT

TTTTTTTTGTATTGTCCATCCCGCCTACCAGCGGCAATCGCGTATCATGTGTTGGAGGTT

GAGGGGGTGATTGTGGAGGGGGTAAAATAATTTAAATGAAAAACAGACATAATAGAAACG

AAGCTTTTAAAAAAAATTATGTGTCCAATGAAGGAAGAAGGGGAAGTGGATGTGTTAATG

TTATTTTGTGCGTTGAAGTTTTTAGTCGTCCATCAAGATTGAGTTTTTCTTTACGTTTTT

TTCCCTTGCTGGTGGATTCCTCCGATCCACCCCCCTCCTATTTAACCCTTAACTTGTGAA

GTGTGGGAATTGGTGTAATAAATGCAGAGGGGAGGCGGACGGAGAGTGAAGGAGGGGAGG

GGAGAAAAAAGGATATCAGGGGAGGTGCTTTTAATAAAAAAAAAAGTATGCATATATATA

TATATATATATATGTGTGTGTGTGTGTATGTGTATGTGTATGTCTGTGTTTATTCGTATA

TCTACGCTTTATTTGTTTAGTTAGTTGCCTTTTCCTTATTGTTACAAGTTATACTGTTGA

TATTGTTGATATTTTTCATCTACTCGCACATCTCACTTTGCCCTGCAACGCAACAGGCTT

ATCGCATTACCGTATCGCAGAATACCGTTCTTCAACCATCGAAGCCACATTTACTCGAAA

AATATCGTGCCTGTCATTTACCTATTTATTTATTCATTTTCCTTCTTAATGATTACTGGT

TCCTTCATCTTAAACCATTACAAGGGGAGCCATCGCTGGTATATGTATTGCTTCACTGTG

TCTACTTATGTATGTATCAGCGTAAAGAAGAATATTGAATACGCTCCGTCACTTTCCGCC

TTGCCTTTTTTTCTTTAGTTTCAGGAATTTTTACAGTTCTGTAAGCATCCGACGAGCCGT

CTTGTATTTCTGTGCAAATGGTCGTGTGGATGCTGTAGAGAGAAATGCGTTGAGTTGTTG

TATGTAGTGAAATAGAGGTGACGGGTATGAACTTCTTCTACAAAGAAAAATCGCAGAAGG

AAAGCACGCACTTAATGACTAGCATCCATTGTAAGCGCCACTGTCACGCCGTATGAGTGC

ACTTTATCAGTTCTAATTTCTTAATTCATTATTTTTTGCGCTGGTACTCTTAAGGGTGTA

ACACGATGTGCTTGTTTGCGTGTTGAGGACGGCGTGTAGGGAGTTTTAGCATAAAACAAA

AAAGGCGACAGGCACCAACCTGGGTCATGATCATTCTCTTCACCTATTTTTTTCCCCTTC

ATTATTGTTATTTTTCCTGCGCTATATCGTTTGCGAACTGCGGCAAATAGATGAGTAAAT

AAATATTTAGAAACGTAAAAGGTAGTGACTGTACAAGAACTCAAGTAAACAAAGTACGGT

TTCCTCTGTGTGTTTGCGCG

>Tb927.7.5680 | Trypanosoma brucei TREU927 | deoxyribose-phosphate aldolase, putative | genomic | Tb927_07_v5.1 forward | (geneCodeEnd+0 to geneEnd+0) | length=1274

ACGGATGTTTGTTGCTGAGTATGTGAAGGCTGTGACATATTTGATTTTGAATACAATAGT

GGTTACAGGTGCGCGAATTGGGGTGAGTGGGCGTCCGGGAGTGGTTTCAAGTTAATATTT

ATGTTTCCTTCTTTTTTTTTTCACTTTGGTGGTGTCAGGCCATTGTTATCACCTACACGC

CGTGTTCTGGTGGAACAGTAATGGTGGTGTTTGGGAGTGTTGTTTCCTATTTTTTTAATT

CTGTTTTGATCATTCTGTGTAACCATTCACGGAGGTTCTGGTGCTCTGTTTGATCTATTT

TTTTTGTTTCGTTTTTATGTGATAACCATCAGGACTGTGTGCAGCGGTTACTCGGTTCTA

ACCTTTTATGTCACCTATTATTTTTGTTATTTATTTAAGTGGGGGGAAAACGTTCTAACT

GAAGGAATGGCAAATGTTGGGAACAATCAGGCACTGGTAAATTTACGTTTCTGAATTTCC

ATATTTCTTTGAAATAAATAATGTATTCTGCGTACCTGCTTTTTGGTTATGTGGCTGTTG

AATTTTTTTTTCCTTGTAAAGACGGTCATTTCACTTGTATGAATCACTTTCGCCTTTTGT

GCAGCCTCGTAGAAAAGTTAAGAAAAAAAACGCTTATCTCTATACGGATACGCCGTTGGA

CGTCACGGAACTGTGCTTGGCGCAATGCAGCTATGGTTATAGGAAAGATACATTTTGTTT

AAGAAGAATTATGAATATGTGTCATGGCTGTGGACAAAAGAGAAAAAGAAGGGCATCTCC

GGTGACCGGAGGTGACGGATGGCCTACCGCCGCGGTTTAAAACTTCAAATTTGAAGTATT

TCTTCAGTCTGAATATTTCGCCACCCAGGACCACTTAGACTAGTTACTCTACCCAATATT

CATGGGTATTCTGTGAATGATGAACTTAAACATACTTTTTTCCTAACAAAACGCCTGAAT

GTGTTGAATGATGCACAACCAATACGTGTGTTCTGATAATTGGTATTTACCACCACCACC

ACCCTTAGTTAAACATATAAAAACATCATCCGCCGTAACGAACAGTTTTGGGACTTCACA

TTTAGTTGGAGTTGCCCCAGCACTCTCGTGACACAAAATGTTAATACATCATTTCCCCCT

AACTTGCAATCAATCCCCTATTTGTTTCGAAAGAGTATAAATATTTTTTGAATGTCTGCG

ACGAGTGTATTAAATTTTTATTTATTTGCTCTCATGCGTTACGTATAGTACTTCGGCGAT

CGTCTTTTTGTTGC

>Tb927.7.690 | Trypanosoma brucei TREU927 | zinc finger protein, putative | genomic | Tb927_07_v5.1 reverse | (geneCodeEnd+0 to geneEnd+0) | length=1921

ACGGAGGGAGGGGGGAGTATGTTAGACTTAAGGTTAGGAAAATAGAGATAATACCAAATG

AAATCCCTTCCCTCCTCCTCCTCTCCCAGTCCCTCCCGCATGCACAAATAAATAAATAAA

TAAATAAAAGAAACAAACGAACGAACGAACGCGAAAAGAAAAGAAAAGAAAAGGGGGAAG

AAACAAAAAAAATTGGAAGGCTTAAATCATTGACACGTATATATCAATCGAAGTGATAGC

CGAGTTGCAAAGAAGCGGCGGAAAGGCCCCTACCTCTCCCTCAAAAAAAAAAAAAAATAA

TAATAATAAATAGTACGAACAAATTGTGGGAAAACTTTTGCCGTGTAACCACCGCTGCGC

TCCATTGGTGTAAACTTCAGGGTCAGCATCACCTCACGAAGGTTATGTCCCACTGGATAT

GTCTTTTCTATCATTACTTCTTACTTTTATATTGTTTTTTTTTAAATATTTGTGCTATTA

TTTCTGTGTATTAAACCGGTTACAAAGTGTAAGAAGAACATCCTCCTTTTTTTTCTTTGC

TGGCGTGTTGTTGTTGTTTTTTTTTTTGTGGTTTCCTTTTTTCCACTGTTATTTTCGTGG

TAGTTCGACTTGTTTTACTTTTTCGTGCAAGGAGTCATGTTTATTTAACATGGTTTCTTC

ACGGGATTCCTTTACTCATTTCTACTTACTTTCTTTTTGTTAGTGTTTAGCCACTGTTTT

TTTTTGTTTTCTTCTTTCATTTGTTTCTTTTTCGTGTCTTATTTGTTCATATTTTCTTAA

AATTATCTTTGTATTACTTGTCCTCCCCTTTAAACGTTCTGCACCCTCTTTTTTTTTGTG

TTTGTTTGTTTGTTTGATGGGAAAGTCACTACTGACCTGATGTGATCCCCCTTAAATGCC

TCCTTTCGTCCCACTTCCGGGGTTTCATCGTCGTTACTTTTGTTTTTTTTTAATTTTTAA

AAAAATCAGTGGTCTATGAATTTGAGCGGTAGTTTGTGTTTTTTATTTATTTAATTTATT

CCTTTTGACTGCTTCTTTGGCATTTTACACGCGCCCTGTATTTTTCTCTTCCCTTCTATT

TATTGAGCGTGCGACTATTTTTCTTTTTTTTTGTTCGCGTTTGTGTGTGTATATGTGTCT

GGATCATCGAACATATGAATTGCCTTTATATTTTCCCCCCTCTTTTTTTCCCCCTGTATC

TTTGTAAGTCAATAATATATGTATATATATATTTTTATTTATTAAGTGTGTGAATGTATT

GCGGTTGCTAAACTGTTTGTTTGTTTGTTTAGCACATTCAGTTCAGTGCCTTTCAAAACT

TCGTTAGCGGCCGTCCAGGTATGTTTTTTTCCCCCTTTCCGTTCATTTTCCTTTTGTTTT

GTTTTAATTATTATTATTATTATTATTATACTCTTCCTCTTTTGCCTTGGATTCTGGTTT

GGACATCTGAATACAACTTGTGGACCATACAATCCTTCGAGCAACTACAAACTCATCATC

TTTGCTTTTTTTTTTTGCTTTTTGCTGTTTCGTTTTTGATTTTGTTTGTCTTTTTGTGCC

ATCATTAGTTTCCCAACTGCACTACCCTCTTGCTTGTGAAAAGCTGTTTCCACCCTTTTA

CTGCTTTGTGACCATCATTTTCAATTTTTTTTTTTGCTTTTACGTTGCGTTGCGTTGAAC

TAATATTATTACCATTATTATTATTATTATTATTATCATTACAATTGTCGCTGTGAATAC

AACTTATTTCCTTTCTATGTGAACACCCTCTCCAGTGAACTTTTCATATTAATTGACATT

TCTGCAGTGTATGAAGGCGTTTGCCGCGATATTTTTGGTTCATTGTTTTTTTGTTTTCTT

TTTATTTCAAAGCAGCGAATTCTCCACCTTTGTTTTCTTATCATCACTCACTGTTTTGGT

C

>Tb927.8.6570 | Trypanosoma brucei TREU927 | hypothetical protein, conserved | genomic | Tb927_08_v5.1 reverse | (geneCodeEnd+0 to geneEnd+0) | length=1069

AAATGTTCTGCATTTTGTATACGCACACGCACGTGTGCTTCCCTCCCCTTCTCTTCACTT

TAGTTCATATATAATATGCGTTTATTATTATCATTATTATTATTATTATTCTAGTATGTT

AGTGTATACGTTTGGGAGTTTCAAATTCTAGTTGTTATTTCGTCTATTTGTATTACTCTT

ACCGACCCTTTCCCTTTCCCTTTTTGTTTCTATTTTTTGATTTTCTAAATTTTTGCTCTT

TTTTTTTTCTTTTCCTTCATTGATATATAAATATAAATATATTTATTGATTTGTATTTAT

TTATTTATTTCTCGTATTATTGGTTCTCCGCTTATGCTTCAGTATGGCGACTGTTGTGAT

GACATGCAGCTGTATATAAAAGCAACTGACTGATAGTTCCAAAGGCGGAAAGAAGGGTCG

AAAGCAAAAGCACAAATTCCTTCATTGTTTTATTTAAACTCCCCTCTGCAATTTTTCTCG

TTTTCTCTCATTTACATTCGGAAGCCACCGTGTGATGTGAGACCCCGTCCCTTAAAACGA

AAGAAAAATTTAAAAAATGCACATGTGTGCGCGTGTCCATATGAGAGGGGAAAAAAGAAA

AAGAAACGTATGATGGTGGTGCGGCTTCGTTGTATTCGCCGCTAAAAAAATTTTGTTTTT

TTTTTTGTTACTGTTGCCGGAACCGTCATCCCACACACCAAAGTTGGTTCTGAAGAGGAA

ATGAAATGACCCGCCGGGCTGCCATTCGTACGGTGCGTTTAATTAAAAGAAGTCGAACAG

ATGAAGTGTGATTTTCTGAATTTCTATTAATAAATAATATATATTTATATATATATAACT

TCTAATTTATTTGTTTATACGCTTTGAAGAAGAATACGTGAAAACGAAACGAGGGGTTGA

TGGAGTTTTCCCTTTCTTCCTTCCATTCTTTTTTCCCCCGTTGAGGTGCGTATGCCTTCT

TCTTTTTATTATTTTTTGTACCGTTGTTATTTTTCTTGTTTTACTCTCACTGGGTACGCG

TGAAAACCTTTCGCGGATCCATTTCCCTTCTCTTGCTTGCGCCCCCTTT

>Tb927.9.13200 | Trypanosoma brucei TREU927 | hypothetical protein | genomic | Tb927_09_v5.1 reverse | (geneCodeEnd+0 to geneEnd+0) | length=352

ACGGTGCGTGTTGGATATGTAAATGAGGACTATTTTCTGATCCTTTTACTATTATTGTTA

TTATTTTTTTTTTTTTCCAAAGCACTAGTGTGCACTTAACTTCTTTATTTTTTTTTATTT

ATTTTCAAAAGACATACATCCCATCGCCGCGTATTCGCTCCTTCTAACCAAAATATTCGT

TTGAGGGTTTGGAATGCCGGCAGAGGGACGACACGCCCGTAAAACAGCTTGTTTACCTTC

TTAGCAGTAGTTGTGTTATTTTTGCAAAAAAGAAAATGTTTTTATTTTTAAATAGTAAAC

GTATCAGCAAGCAACTGTTTTGCCGTACTTGAACACGATGACAAGGAGCCTT

>Tb927.9.1520 | Trypanosoma brucei TREU927 | hypothetical protein, conserved | genomic | Tb927_09_v5.1 reverse | (geneCodeEnd+0 to geneEnd+0) | length=284

AACATGTATTGGGGTTTTACCATTCCTACTGCAATTTTCTTTTTTTTTCTTTTTTTTACT

CTTTTTATTTTTCCTTCGCTGAGAGCGTGCATCACTATTATGAACACTATTACTTGTAAC

CAGTGAACGTATTTATTTTTTTATTTTTCGAGCATATAAAGCTTCAAACGAGTGCCGTCG

TGGGAAAGAGGTTTCCCCTCTTTTTGCTACCTTTTTTGTAAAAAGATTATTATTATTAAT

TATTTATTTTTATCATGCTCTCTGTTTATTTGTCGTTTTTTTTG

>Tb927.9.8260 | Trypanosoma brucei TREU927 | rhomboid-like protein, serine peptidase, Clan S- , family S54, putative | genomic | Tb927_09_v5.1 reverse | (geneCodeEnd+0 to geneEnd+0) | length=528

ACCACTTGGAGGGGGTTGCTAAGGTTTTCGTAATTAACAACTTTTCGTCGGGTGTTCTCG

TCAGGTGGGAATGAGCCCAAACTCTGCAGAAAGATATATACACTTATAACAGCACGAGTA

ACGTATTCAATCTGCTGTAACCTTAACACCCATTGCGACTCTTCATCTTGTTTAGTTCTT

GTTTTCACTTGTATGCTTCGTGTCCTTGGCGGGTGTCGCCCGTATTATTATTATTATTAT

TTTTTCACAGAATAAATGTTGACGGATGGAGTCGGTGAAGGTGGTGTGAGGACCCAGTAA

AGGGGGCTGAGACGAACATGTCGGAGAGGAGGGGGGGGGCGGAAAGTGGAGGGCGGCGTG

TTAATCGGGCACAAACTGTGATGCACGCGTAGGGAAAGATGTGACAGCGTAGGTTCTGGC

ACATTATTTAAAGGCTTAACGGACCTGCGAAGGGTGTGAAATGGAGGGAGTTGGTGGTGA

TGGCGAGTTCCTCGACCGGATGATACGGACATATGTTCCGAATTCCTT

>Tb927.8.7340 | Trypanosoma brucei TREU927 | trans-sialidase, putative, neuraminidase, putative | genomic | Tb927_08_v5.1 reverse | (geneCodeEnd+0 to geneEnd+0) | length=1037

GGTTATATATTTTTTAAATTTCACTAATTGTCGTAGAGTAATGGGCAAACATATAAACAT

TTATTTACTTATTTTTTTGAGTGTGAGCGATCATTAGCGAGCTATATTATTTTATTATTT

AACATGCAATATCATCATGGCTATACATGTATATGCCGTTTTTGAAATTCACACCGTTGA

AGTGGATGTCATGTCACGTCATATGATGTCCAGGGATGTATTTTTTCTGTTAGTGATCTA

AAGGAAGTATGAAACAAGTGGTTTTTGTGTCTGTACCATCTTCGTTTGATTCATTTAGAT

CTCAAGTGGTTAATCTTGTTTGCATGTTGTAACGATACGTGTCCGCTACAGCTGCTTGTT

GGGGTGATTGTGTGCCCCGATTTTCGTTTAAAAGGAGTATATCCTCCGTTGTGTCATCGA

CGTTGGCGCCGTGTTAATATGAGGGTAACTGCTGCGAGGTCTGTTTCGTGCTCCATTGTC

TTTTGTGCTTTTTCTATCTTAGGGCGGTACTTCTCTTCTGTATCATTTTGTATTCTTTTA

GGGAGGTCATCTACATGGTTTAAACAAATTTGTGTTGGTTATTGGAATGTAGGCAGTCCA

CTTCCGGATTCCCTCGAGGAGGGTCTGGTGGACATGGTAGCTTATTTAAGTAGATGCGAA

GCGCAGCAAGATGTTTTTTCCCCTTTGAGAAAGCAGAGCGGCCACCCCGTTAGATTTTAT

AGGGGTTTGATTGACGCCCACGACGTCGCCAAAAAACGCAGTGAGCGGAACGGTTGCTTG

TGGAATGCTTGGAAATCAAAATGCGGACAGTAGAGCAGCTGATGCAGGAGGAAACAAGAT

GCTGTGCTATTCTGTCGGCTGCCCCCTAACCGGAAAGCAAGTTCCTGGTTCCAATTCCTC

GACCTGTGCGAGTAGTGACGGGGCTGGAGCGCGGCCCTGTTGGCCAACAAAGGTCCCAAG

CGTTGGCACCACCCATTTACTATTCATTACCTTCGATGATACTCCTTCTTTAAAACCACA

AGGGTATCGTTCAATGT

>Tb927.3.590 | Trypanosoma brucei TREU927 | adenosine transporter, putative | genomic | Tb927_03_v5.1 forward | (geneCodeEnd+0 to geneEnd+0) | length=118

GCACCGCCCTTCATGGGAACCTCATTGGGCTTTTGGAGCCATATCCGTAAATGAGAAAAA

TTGCAAGCAAGTACACAAGAGGTGCTTGCTAGCGAATATAAACTTGTGGTAACCGAAT

>Tb927.6.3880 | Trypanosoma brucei TREU927 | hypothetical protein, conserved | genomic | Tb927_06_v5.1 reverse | (geneCodeEnd+0 to geneEnd+0) | length=1652

AGCTGTGTGCCGCATGTTATACTTGTGTTTGTGTGTTAATATTGTCATTTTTTTTTCTTC

ATTGAAATTTTATGTTGTGGAAAGAAGAGATGCGGTTTTCCTGAGGACCATTTTGCGAAA

AAAAACCCTTTTTTGTTTTTCTGTAGTGATTTTAGTGATACTGTTCGCTTACGGACTGTC

TCCCAGACTTTGTGCACACACATATATAAATAAATATAAATATGTGTGTGGACGGGCGTT

TGCGTTTTATCTTATTATTTTTTTTTAAAACCTTTCTATTGGTGCTTTTCGATACTGGTT

TTGTTATTTTTACCTTTTCTTCTCCAGCTTATAAATCACTCTCAGTTTCAACCTTCTGAT

GCTTTTGTATCGGAAAAAGTGGCGGGATGCAGTGTGTACTACCTTACTTTTTTTTCTTTT

GGTTTAATCGTTACGTTGCTCGTACCATCATTCTACCATAATAACAATAATAATAATTTT

ATTCCGTTCCCCCGTCGTTTGATCCTTTGGTACGTTTTATATCTTTTTTTTCCCTCTTCC

CATTTTTGTTGTTTGTGTTTTTTTTTGTCAATATACGTTTTTTAAATCCTTTTTGAAAGT

GCTGCATAACACGTTTTTTTTTGTTTTCTTGCTGCTGCGCATTCAATTCCACTTATCACA

TGCACGTGAATCCAGTAAATAAAGTAAAATGTGGGATATTTTTCTTGTTTTTATGCTCTT

GTATTGTGTGTTATTTTTAGTATCACTATTTTTATTAATTTTTTTCCACCACTTTTGTTT

TTTACTATAACATTATTCTATTTTATTTTTTTACTTTTTGATTCTATGAGTGATCGGATA

GATGTGAACCATTCGGTTCAGGGAGCTGTCGAGGTGAAACGTTTAACGCAGCACCGTTTG

ATTTATCGCGTCTTATTTTTTTTTTACGTCTTTTTATTTTTACTCACTCGGGCATTTCCG

TGTGCATTTTCTTTTTTTTTGTTGTTGTTGTACGTGTTATTATCATCATCATTATTATGT

CGTGGTTTGTGATATTATGCCTTTAATATATATTTCTGTTTTTTTTCTTATATTTTTTTT

TGTTATTCTTATTTCCCCTCCATTTGTATTATTGTGTTCTTCGTTGCTCTCTTTGGCTCT

CTGTGTCTTTCTTTTTTTTTTTTTTGTCAAACTCTTCAGTGGTTTTGAATTATAAATTCT

TATTTTTTTCCCTCTATTTTTCATAGCTGCGGGTGTCTCATATGCAACAAAATGCGTATT

TTTGCAGCATTTTAATCCCTCATTTTGTTTTTCCTTTTGTCGTCTGCAGTGGTTTGAACA

AAACACAGCCGTGAGAGTATTTTAATGGAAAACTGTGAGGCAGCAGCGAAGAAAATGAGG

GAAACTTAAAAAAAAAAAGAAATGAATAGTGTTCGTGTTTTTATTTATTTTGTTACTGGT

TTGTGGTATGAAAGAGAGTTAATTCTTTTTTTTTTTAGTTTTTTTTGTTGCGTTTCCTCA

CTTTTTTTTTTCTTGTTGTTTGGTCTACAGCACCACCAAACTTCCTCCGTTTTTTTTATA

TTTTTTTTCCTCGTAGAAACTCGCTAATGCGATATATAAACAACTCAAAGCCAGAAGAAA

GGCAGTGTGGAAGAGACTGTAAAGGTAAAAGT

>Tb11.02.5400 | Trypanosoma brucei TREU927 | cystathionine beta-synthase, putative | genomic | Tb927_11_v5.1 forward | (geneCodeEnd+0 to geneEnd+0) | length=892

ATATTCTGGCAGTGAAGCGCAGGAAGGCACTGCGAAAAAAAAGGGGGGAAAAGCGGAAAA

GACTCAACCAGCGAGTAGCCTGTACTTGAGGAGGTGATACGTGCTTTATTTTATTTCTTA

ATTTCATTCTATATTTATATCGATTTCGTCGTTGTTATTTTATTCCAGGACATGTGCGGG

GGACATCTGGCTTAAGGTGGTCGTTTAACTATAGGGGTTGTCTGCCGCAACTTTCCCCAT

ATTTTGGATGTTTTCATTTTTCGCGTTCGTTATATTATCTAATTACCCGCCGGACCGCTT

CTGTGTGTGTGATAAAAGGCTTTACAACGGTATTCTGGTGAGCGGAGGCACCTCGCGCAT

CGTATCTATCTATAAATAATAATATATATATATATATATATATATTCTTCGCATTTGGGA

AGTGGGTAGCTCGGATGCGAGGCGCTGTTCTTTGTTGTAAATCCAGTGTGCTCAGCCCCC

GCCGTTTTTATTAGTCATCGACTGTTACTATTATTATCATTTATTTATTTTTTTCATCCT

GGGCACTCTATCAAGCTCAGACGCACGCATATTCACGCTCATCTAAAATAATTTTGTCCA

ACAAAAAAAGTGAGGATGGTTGGGGAGGAGTAAAATCTCTCCTTCCTGTTAAAACATAAT

TTTCTGACAGCGACATTTGGTACTCTGTTAGTAAGCCCGACGGTGTTTTGTCCATCTTCC

CCATATATTCATGGACTTGGACGCGTCGCTGGACGTTTTTCCACTCATCTCCATCAAAAA

TTCCCGTGGTTTCCGCCACTTTATTTCCTTTCCCACTTCCCCTCGTTTCCCTCAATTAGA

GAACAAAACTAACCCTTTATCAGGGCTAAATATTCACTTAGTTACTTGCCGC

>Tb927.7.6850 | Trypanosoma brucei TREU927 | trans-sialidase (TS) | genomic | Tb927_07_v5.1 reverse | (geneCodeEnd+0 to geneEnd+0) | length=699

ACGTTCTCCAGTACGTATGTGTTTTTTCTTGTCCCTTGGGTGGGTCTACTCTCACCTGTA

GAGGCACCGCCTCTACTTGTTAACGTATTTTTTTTGTTTACTAGTGCAGACATTTTTAAT

TTTTATTTGGAGGATAATAGTTACCTAAGGGAGGAGGCGTACGGGGTGAGGAGGGCGAAA

CATGCATGTATGCGTGCTGTTCTGTGTTGATGCCCACTAGTTGAGGACGGGGATGGTAGC

ATTCGTGGCGCGATGGGACCCTTCTGCTCCCCTCACGTACGGTTCGTCGCCACCCTCCAT

TCGTGAAACTGAGTCAGCAACATTGCATGTGGTGTGTAATCTTTACATTCCATTTCCCCT

TTTATGCACAACCAAACTTCCGAGGTTTATTTTTCTCTATTTTTCTCTATTTTTATTTTT

ATTTTTTATTTATTTTTTGAGTGTTCCAAGAACACCTTGTTTGTACGCATGGTTCCTTCC

TGTTTTGTTTTTACATTTAAATTATGAACATCCATTATCGAAAAATATATGTTTCTCTTC

TTCCTATTAGACATAGCATGGGTTACTGTTTCCAAAAAAGGAATAACACACACTCACGCA

CACAGAGTGCGACGTTTCAAATAACTATTTTTTTCTCTTTTGTCAACGAACATTTCTGCG

TTGCGAACCTCATCGAGGTGAATTTTACCCCCGCTTCTT

>Tb927.7.2980 | Trypanosoma brucei TREU927 | Nitroreductase family, putative | genomic | Tb927_07_v5.1 forward | (geneCodeEnd+0 to geneEnd+0) | length=654

AAGTTCGTTGATATTTTTACCGTACTTGATCACACACATGTATTCCTCCATGGACTGATC

TTATTTACACTCTTGGTGTTATTCACCCTCCTTTAAGCACCAATTTCTTTGCAATACATG

CATATACATGTTGGTTTTGGGGCACCTTTATATTGTTATCCAACTGCTTTTTTTGTTCGC

CACGCTTTTCCACTTCTTACCTTTTACACACTAGGTTAATTTGGGCACCGCGTGAGGTTA

ATATGACCAAATGTATGTTTCTCATGTTTTCCCTTCGTTTAGCGAGGCAAATGTATTGAA

GAAAGTGTCGTGGTTGGGGTAAGTGGTGTTACACGCACGACGTGTTCGTTACCCACCCTT

TCCACCCTCTTCTCATGCTTCTGCACTTCTTTACTTTTTTGTTCATTCGTTTATATACAT

ATATACATATATATTTACGTGTTCTGGCTTCTACGATTTTTAAAAAACACGTATCGCTAT

AATTATAAGCGTGTACGCACTTATTTTTTTGTTATCGCTCTATATACATGTGGTGCATAA

TACAAGGTGTCTCATTATCACTAGAGGTTTCTTAGGTACCGTACCCCGGTCATGTAGCAT

GATGTAGAGGGATGAGAGTCGATATGAATATAATAAATGATGCTTGTAAGGATG

>Tb927.7.2180 | Trypanosoma brucei TREU927 | T. brucei spp.-specific protein, putative | genomic | Tb927_07_v5.1 reverse | (geneCodeEnd+0 to geneEnd+0) | length=464

ATATTGTTATTATGTCCCTCCCCCTCCCCCAACAGATGGTAACAACAGACGTTCATTTTA

TGTGTTGACTTGTTTGAGCTAAAACGACTTCAAATATTCACTTTTCTCTCCGCGTTTACG

CCATTAACGTTCGCTTTTACTGTTTTTCTTTTTTATTTTGATTCGTGTCCGTACCCTGTT

GCTAACGTGGGAAAATTTCCCTCCAATTCTAATGGCAAGAAACAGAGTGCCTGCCTAATG

GAAGGGGGAAGTTACGGAATGAACTCCCTTACCCTTTGCCGCTGTTTGACAGCGGGTGCT

ATAATAATTATTATAATGACATTATTATTATTATTATTATTATTATTATTTTCTATCCTT

TAACAATTTCTTTTTTTTTCGTTTGAGGGCATTGCCAACTGCAGTTGTGCTTACTATTGA

GGATTCACATGAGAGATATGTCAGCACTTGTGTGTATCTGTTCG

>Tb927.7.2680 | Trypanosoma brucei TREU927 | zinc finger protein family member, putative (ZC3H22) | genomic | Tb927_07_v5.1 reverse | (geneCodeEnd+0 to geneEnd+0) | length=4595

AGGTAAGAAAAGAAAAAAAGTAACAAGACAATGGCGATTGGAATAGGGATGGTTGCGTCG

CAGGGCTGTCGGTACTTTCTTTTTCTCTTCTCGTTTTCCTTTTCCTTTGTATTTCTTTTG

CTTGTGAACGCGACTGACCCTGTGGGGGGGGGGGGGCGGCCGTGAATGGAATATAGCTAA

TTGAGTTGTCCTTTCGTTGAGCTTTAGCCCTTAATGGTTTCCGCATATATATAACGGTTG

AGGCTCCATTGCCCGTTCTGAATAATGAAGTTTCCCCCTCTCTGCTGGCTTGTTTTTGCT

CATCAGAAGCTGCACCACATAAAATGCAGCGATGCTTGCGTGGGAATTGTGCTGGATTAG

TGAGGGAGAAGGGAATGAGAAATGAGCGGCGAAATGAAGGGTTTTGGTGGCTGTAAATGG

CAACGGAAGGCTCAAAGGCGCACTCCGTCGCTTGAACAAAGGTTAAATTTTTAGAAGCTG

AAAGAAAAAATCGGAAACAAAAGAAGGCAAAAGGAAAGTACGACAAAAAAAAAAGAGGAA

AATGAGAAACATAGGCTATTTAAACCGAAGTAAAAGCTATTGAGCGATCGAACTTTTAAA

AATTTGGAGGAAGGGACACCCAGAAGCAGCTTACTCTCTTTTTTTCTCTCTTCGCTGCCA

CGGGTGCAGTTTACAAATTTTGGTCACCCCCTTTAAGCAATGATTTACTTGACTACATAC

TTGTATATTTTTTGTTTGGTTTTATTTTCCATTTAAGCATTCTTCCTCCATAAATATTGA

TTATTTGTTTATTTTTTGAATTTATTTTTGGCCGCCTCCATCCTCCCTTTCATTTCCTCA

TTCCTCCCACTTCGAAAATGATATGATGAGGACTCCTATCATCGGTATGTTTCATAACAG

CCGTCCAGGCCATGGGGGTGGGAGGAGGCTGAGGGGAATGGAAGTGGGATGATGGAGAGA

CTCGAGGTGAATTATCGGTGGTGAAGGAGGGGGTTTTACACACACATGCACACACACACA

CAAATAATAATAAAAATAATAACCGCTATCCAAACAGATGCTAGAAAAATTTACCACGTG

GTTGTTTGTGAGGCCTGCCCAGTGGGATTGATATATATTTTGCAAGCAAAGAAAGTGATA

TATATGCATGTGTGTGTGTGTGCATGGGGGAAAGGTGAAACAGAGAGCGTATGGATATTA

TTTTATTTAATCTTCCCGTTCTCCTCTCTGTCACGTTCCCCCTATTTATTTTGTTTTCAA

ACTCTTTTATTTTACTTCTTTTCCTCGACTTGTTTGTTACCATTTATTTTTCCTTCTCCT

TCTCCTCTTTTTCCATCTCCATTCCCAACCTTTTATTAATTTCCAAGCCCCTTCTACAAA

TATCAAATATAAATATATATTATTTATTATTATTATTATGAGCCCGCCCATTTGACACCC

TCAGCCACTCAGCGTTTTGTTATTTTTTATTTTACCCTTCCGTGGGCGTAACGGGAGGAG

CGGGAGGAGAATTAAAGAAAAGGGATCGGGCCGCGGATCGCGCAGCTTTTTCGTGATAAT

GATTTTTCTTTCCCCCTTTATTTTTCTTTGGTTGATAGTTTTCTCTTTGTTTTTTTGTTT

GGTTCTGCGTTTGTTCCACCTTTTCTTGTTTTATTCTTCCTTCCCACCACGTTAGTATCT

CGCTTCCTCGACAGATGTCCATGTAGAATAAAACCGTGATGCGAACGTAAGGAAAGACAA

ACGATTTCTACACACCTTATTATATATACATATATTTCAAAAAGAAAATTAACACAATAT

AAATATTTGTATCTTTGACTGTTCTTTGCCGCACCACCTTTTCGCCATTTGAGCGGAACA

AAAAGATAACGATAATAAAATAACCGCAACGATAACACCAGTGGAAAGGTCGAGGGGCCT

GCGGAGTGAACGGGAGCGTACGGGTGGAAGAAAAAAGAAGAAGAAAGAGATTTAAGGAAG

ATGAAGTCAGATCGAATATGCATACATAAATTTATGAAAGAACGCAATAACTACTTCATT

TCATTATTTATTTTTTCTTTCTCGTCATCGAATTCGTGGTTAACTTATGCTGTCTGAACA

CTTAGATGTAAGATAATTAACTCTCGAAAGGACCACTTTCCACTTTTTTTCTTCATATTT

TACCTTTTATTATTATTATTATTAAGAACAATGAAATGATGATAATAACAAGGAGATAAA

GAGTCAACGTGATGTCTTCTCAAATGGTGGATGCAATCTGTGCATTTATTTTCCTTTCCT

TTTATTTCTACTTTAAAATTTTCTTTTTTTTTTTCCACGCCGTTGAAAGCCATGAGTGTG

AGCACGGGAGAAATGGCCGGTGTGATGAAATCGGTGGTGTGTTATTGTTAGCACAATTAT

GATTATAATTATTTTCTTGTCTATTAACCCTCTTTTCATTTAACGTTTTTATTTATTTTG

ACCGCGAGTCATTCGTACGTTTGTTTGTCATCAAATTTCCATCAAATTCCCCTTACCCCC

TACGGGTGTTGGTGTCAAGTGACCGAATATATTCGTGTCTCTCCAATCTCATTACCCGTT

TCTCATTCCATTCCATTCTTTGTTTTGTTTGCTATTCTTTAATTTTTCTCTTTTGCTTTT

CTTATTTTTTATTTTTTTCCCGGTCATTGACCGAATAAGTCACCTCCTTTCCATCCTTTC

CCTCCTTTCCCCTGTTCTTGTTCTTGTTCCTTTTTTTTTTTGTGTTTCTTCCTGTTTTCT

TTCATTTTTCCATTTGTATTTCTTTTTCTTTTTTTTTGTTTTTTTAAAACCCTCCTTTTC

CCCGCTGGTTCCATGCTACTGTGCGTAACTGCTTTGGTCTGTTGTGAATGTTGTTTTGCT

TTGTTTTTCCTTCCCTCCTCCATAATATTCCATATCATGGACGAATACGGCCTTAGGCGC

ATAATGCAGGATTATGTTTATGTTTGTGTTTCTGTCTTACTCCTTTTTTTTTAAAAATGA

TTTTAACTTTAATTATCGGGGTCGCTCTCTCCCTTTTTCTTTTTTCGTGTTTTCTTTTTT

TTTTTGTTTTTTTTTTGTGTGTGGGATGCATTTTGTGGCAGGGTGGTGTGTGTGTGTGTG

TGTGTGTGTGGACGGGGGGATAAGACAAGAAAGAAAAGGATATAAAATATTAATTTCAAT

GGCAACAGCGGCATCCGGCGAAGCGAGCGATTTGGTGACGAGTACAGACAGGGAGAAGAG

TGAAAGTAATAAATTATATTGTGTCGAATTAAATCCACATACAGGTGCAAACTAATATAT

ATATATATGTATTGTGTATAGGTGGGTGTATGCAATGAAGAGAGCAAATAGTTTTTTTTT

GAAAAAAAAAAGTAAAGCAAATGGCAAAAACAAAACAAAAAAAAGAAGAAAGAAGAAGAG

TGTGTGGACAAAATATATACTAAGGCAACTGCGTATGTTCGTATATGTGCGTGAGTATGT

GGGAGACGGGCAGGCATTTGTTGGCCAAATTCGTTTTAAATCAGTAATTCATCTGGTATG

ACTGAACTCAAATAAGAAAATTTATTTATTTATTTACATTTTGCAGAGGTGAAACGAAAT

AAGTGGAGCAACTTTGGTTTCATGATGGTGAGTGAAGCAGAACAGCCAAACAAAGTACAG

AACAGAACAGAGCGTAACAGAAGAAAAAAAAAAGGAAACGAAAGGGAAAGCGAAAAAGGA

GGAGAAAAGGAAACAACATCAGTGGAAGAAACGGATGATAATGCGGATTGGAAAAATATG

GCTCCAACGCTGAAGATCAAACATGCAAGCACGTATGTGAATTAAAAATAAGTATAAATG

AATATATTTAAAATATGTACATACGTACATATGTACATTTTAACCATTATTATTATTATT

ATTTCAAATAGTAAACAACGGTGTAACAATGAATAGCGAACATAATATAAGACAAAAAAA

AAAGAGGAGAGGAACATGTGTGTGCGTGTGAGGGTGCGGGCCTGCAGCGTGAGTAAAAGT

GGTGGAGAGAAACATACAGGATAGGTGAAATGGAAAGTTCACAATGAAATAATGAGGAGG

AGTAGGGAGCATAAGATGGAAATGCGTGACTTTTATATGCGGAAAGTACGCAACAGTGGA

TTGTGCTGTAAAAAGAGGGATGAATATTGCGGAGGGATGGAGGAGGGAGGAAAAAAATAA

TAAAATATGCGTCCTTACTTTTTGAACACTTTCTTTTTTGTTTTGCTCCACTCTCGCCCG

TTCTATTCAATTCCATTTTCTTTTCGTTGCCTTTCATTCATACCATTTCTTCAACTCATC

GTCGCGTTTTATTTTATTTTATTTTTTACATTTGAGAATGTAGGACAGTGTGGAACGTGC

CATTTGTATCACACTCTGAGCGCACATTTCTTTCAGAAATTACTGTATGCGTTTGTGAAG

TTTTTGCGTCCTCGGCTCCCCTTAATTCTTTTTTGTTTTTATTTAATGATTGAAGGAAAA

AACCAGTAGAGTGATTTGAAGGTAATGAAGTGAAGATAAACTCACAAGGATGGAAAAAAA

TATGGAAAAGTCCCACACATTATTTTTTCCTTTAC

>Tb927.9.7470 | Trypanosoma brucei TREU927 | purine nucleoside transporter (NT10) | genomic | Tb927_09_v5.1 reverse | (geneCodeEnd+0 to geneEnd+0) | length=494

ACTGTCCCTAAGAGGAGGTAATAAATGAATAAATGAAAGAATGAATGAATGAATGAAAAA

GAAAAAAAAAGGCATAAAATAAAAAATAAATGAGAGCAAAACGTTTATTGAATTGGTTAA

AAGGTTTTTCTTATTTTTTTTTTTTAAAAAGAAAAAATAGAAAAAAAGGAAGACGGATTA

AGAAGTTGTATAGGGTAGCGGCTAACAAAGGGAAACGACAAGAAGAAAGAATGAAAGAAG

GAGAAAGAAACTGGAAGAGAGTTTTTTTTTTTAAAAAAGAAAAAGGGTAGGAAAAAAAAA

TTTAATCGGTGTAACGCGAGTATTTGAGGAGCGGAAGGAAGAAAACGGTGGAGTAAAATT

AACGATGAGGGGACGGGTCACTTTTTGTTTTTTTCTTTACGTTTTTGTTTCTTTTTTTTT

TTGTGTGTGTGTGTGTGCAGAGAGAGAGAGAGATAGAGAGAGTGGGGGAAGAAGACAAAG

AATGAATAATAAAT

>Tb927.9.13570 | Trypanosoma brucei TREU927 | hypothetical protein, conserved | genomic | Tb927_09_v5.1 reverse | (geneCodeEnd+0 to geneEnd+0) | length=552

GGAGGGCGTATGCATGAATCGGTTCTGCACGGCTGCAGGAGCGGTAATGATAGCAACATA

TCCTTTTCAACAGAAAGTAAGGTGTAATTATTACATCACACACAGACACACGTATGTATA

TACGTAACTATTTATTTTGTTTTCTTGTGGCGTGCGTACGCGTATACATACATTTATGGT

TTGGTGTTCGTTCTTTATTTTTATTTTTTTGCTGCATTATTATATATATTTTCAAAGCAG

TCGTTTAAAAGTTGTTTCGTTATTTTTTTAAAAGCTCTTTTCTCTAATTGAAGGCTGAGG

GGAGTTCACGCGTTGAAACGCTTGTTCCTTTTCATTGTTTTAACATGACTGTTTGATATC

ATTTGCTTTTTAAACTTGTCTAGACAGATTTTAGGTTGGTTGAATGCTTTTGTACCCCTT

CATGTCGACAAGAATGAACAGTAGATACAGAGGAACTTATTTTCACTTTTTCTGGTGGTT

TGTAAGTAAAAAAGGAAAAAAAAATGGAAAGATACAAGGGGGTAGGAAATTATCGGGCCT

TTTCGGCTATGG

>Tb927.8.7730 | Trypanosoma brucei TREU927 | Sphingosine N-acyltransferase, putative, Ceramide synthase component Lag1/Lac1, putative, dihydroceramide synthase, putative (DHCS) | genomic | Tb927_08_v5.1 forward | (geneCodeEnd+0 to geneEnd+0) | length=1056

GAGGGTAATAAAATGGCTGGGAGAGCAGTGGGAAGATGATGACTCTATTTACAAAATGTT

ATTTTTTTTTCTTTTTTCGTTATTTTTTTGACCTCCAGTGCCTCTGAGACACAGTGCTCT

CCTGAGGAAACGTAAGATAATTCTGGAAAAAAAAGGGTAAACCCCCTTTAGAGTTAGCGC

AGGTTGGGTGCCCAAACTCATGACCTTCAGAGGTAATGACAATCTTTTCATAAGCAGCGG

TGTTAGCCATGTTGTATTAGCTCAATTTCACTCCTTTCCCCGTAGGCACACATCATTTGT

AGAGACCGCTGCCGCTGCAAACATGTCCACCCCTATATATATATATATATATATATACGT

ATCGTTGTGGATGCTTATTCCCCCCCCCCTCCCTTCTATCTTGACAAGTCTTCCCTGGAT

TTTCTTTTTATTTTTCTTAGACGCACATGGGTGAATATGCTCATATTTTTATATGTAGGG

TGTTAATCTGACATTCCTAGTTTATTTGAGCCGCGAACATGTGCTTGGTAACACTTACGG

GGATGGGGCCGGGAGCGGAGTTATTTCTGCGGACTGTTCAGACAACCTGCAGCAGTTTGT

AGAAAGTTATGCGTTTTTGGATGATGGATAAAACTCAGGTTTTATCCATGTGTTTCGCTA

GGATTCCTCTTCCATTCAAAGTTCGACAAGATAAGTTAGTGGAAGGAAAGGCGAGCGATA

TGTTGGCGCACTGAAATTGGGGCTAACTAAACCAACTGTGAAGGAGTTATCGGGAAGGTG

TTGGAAATTGCGTGGGGCCCCGAAGCCCCAAGTGGCCATTGGAAATTTTTTTTTTTGGTT

CAGAGAGGATCGTCGTGTTTTACATCGTGTTGTGGTGCGGTTGAAAGTATCATTACATTC

GGGGCGAAACACTGTGGAAGGTAAAGAGACAGTATGCACCTCAGGTAAATAAAGCCTGTT

TTGACCGTTCTCTATAATTAACCTCCCTTCCCTTTGGTTTCTCCGCCCAACTATCTCCTC

CCCATGCATAATTACGCGCAACGGTAGTGCCTCATT

>Tb927.11.1560 | Trypanosoma brucei TREU927 | 1,2-Dihydroxy-3-keto-5-methylthiopentene dioxygenase, putative | genomic | Tb927_11_v5.1 reverse | (geneCodeEnd+0 to geneEnd+0) | length=142

GTTGTAATTCAGGTGATGCCTTTCCACGCTTCCTTTTTTATCTTCATTTTTATCTTTATT

TTTATTTATTTATTTTTTCATGTTTTACGTCTTGTTATTTTACGCTAATAACAATTATCA

TCACCACATGGACAAAGGCATG

>Tb927.5.740 | Trypanosoma brucei TREU927 | hypothetical protein, conserved | genomic | Tb927_05_v5.1 forward | (geneCodeEnd+0 to geneEnd+0) | length=376

ACGGTAGGAAACCATATACCCATGCATGACCGTTCTTCATTTTTTTATGATAAGGGAGGA

TGCGTTGGCGAGGGCTCTATAGTTCCTTTATTTTTTCATTTGGTATGAGATAGAGGATCG

TGGAGATGATTGGGCGCGTTCTGAGGCCCATTTTCTTTTTTTTTCAGTGCTTCGTCTCGC

CGTTTTCCATATATGGCTTCCTCCGAACCTCAGCAGTTAGTGTTGCGCCGTGAGGTTTCT

ATTCAATGTACATACAAACGTTATCATCATTACCTATTTATTTGTCTACCGGTTGTTGTT

TTTAAGAGTTGGCTTAAGCGCAGTGACTGTAGGGGACCCATATAGGGAGGCCCCGATAAA

TTGACGAATAAACGTT

>Tb927.7.3600 | Trypanosoma brucei TREU927 | emp24/gp25L/p24 family/GOLD, putative | genomic | Tb927_07_v5.1 reverse | (geneCodeEnd+0 to geneEnd+0) | length=657

GTGGGTGTATAAATGTTGATGCGGAACAAGAGACTTGCGGTCATTTGTGGGATGGCCCAC

CGCGCAATAATAACAACTACTGTTATTTTTTCCTTTGTGTATACACGTATAGTGGCGCTA

TCAAATGCACCGCTTATATACCATTGTGATGTGCTTATTTATATACATTGTTTACTTCCT

TCCGCCATTATTATTTTGTTGTTGTTTTACTGTGCGGTATATACTATACACACACTCAAC

TCTTTGTGCTTCTGCACACTGCGTGATGAGCAGGGGAGGAAGAAAGAAGGCGTGAACACC

AAAAGAAAACACCACAGATGAAGCTGTCCGTACATGCGTATATACATATAACTGTGAATG

TTTATTTGTAGGCGCATTTGTTTGGTAGTTCTTTCTTTTGCTTTGTTTTTTTTATATATT

GATTGACAGCTATTTCTTTTCACTGACTTTCCCGCGCTGCAACACTTTCAAACAGTATTT

GACCTCGAGACACTTTGTCTCACATCCGATTTTTTTTTTTACCGCATAAGAGGCTTCCAT

CATTGTGCTTATTTACTATTTCTTTTATTACCTCTGTTATCATCTCGTCTGTGTGACGCC

CGCCTCCACCACATTGCCTGCGGGGACAGTTTTTCGATACTACACTCGGCATAGCTC

>Tb927.10.4280 | Trypanosoma brucei TREU927 | ubiquinol-cytochrome c reductase complex 14kD subunit, putative | genomic | Tb927_10_v5.1 forward | (geneCodeEnd+0 to geneEnd+0) | length=345

ATAGCAGCTCCATTGGTGGTATATAGGGGTGGGGCATGCCGATGGTGGTAGAAGTGTGGG

CGAGAAGAAAAATAATGAGGGTCACTGGTGACGGTGGTGTTGGATTACTTCTTCCCCCCT

CCCCCCTCAATTGCCCTTTTCCCCTACTGCATCGGTGATTACTGATGAAAGTTAGGAATT

TAGGAGGTCCACGTATTGTGAACCGAAATGTGGGTTGATTGATGTTATGGTATGTTATGT

TATTTTTTTCGTTACTTACTCACATGTAACTATCGCTATCGATATTAATACACATGCATA

TTATTTTTTTTGTTATATTGAATGGAGTTGGGAGTTTGGGTGGGT

>Tb927.10.5220 | Trypanosoma brucei TREU927 | hypothetical protein, conserved | genomic | Tb927_10_v5.1 forward | (geneCodeEnd+0 to geneEnd+0) | length=631

GTTTGTGCAGAAATTGATTTGTGTTTTGCGTCTTTTTCCGTTTGGATAAGGGAATATGAA

AAGTGGAAGAGGGCGCAACGGACGGTGAAATTGTTAGTCACCGTGTTAATGTGAGGTGAC

CATCCTCCCTTTCCTTTTATTTTTTGTATTTTTTTTTTTCGTCATCATCTCTTTTGAGCA

ACCTGACACGACAGATGTGTGTGTGTGGGAACACGAACAGGAGCGAAACAAAAGGAATAA

TAGAGGAATAAAAATATGATGAATTGCATTTATTCCATGATAGTCAACAACAGACAAGAT

AATTCTTAAACGTCTTGATTTCGATATTCCTGCATCTTGAAGTTCCCGTGTATTTTCTGA

GGGCGAGACATTTATAACGTAAAAGTGGTATTATTTTCAGATACATCGCCTCTTTCCCTC

TATCTCTCTCTTTCTCTGGCGGACGTTTATTTTTGTTTTATACACACCTCCAGTGGTAAT

TTGTTTTTCTTGTATTCTCTGCGTGCTGAAGTTCCACTTTGTTCCGTTCCATGTTATAGA

GTAGTGTGAACTCAAATGTGTGAGGGAAATAAGAGCTGTCTGATTATCTTCTGTGTGTGT

TTGTGTGTTTGTGTGTTTGTGTTTGTTCGGG

>Tb927.11.1340 | Trypanosoma brucei TREU927 | hypothetical protein, conserved | genomic | Tb927_11_v5.1 forward | (geneCodeEnd+0 to geneEnd+0) | length=1583

AGCATTGGTGTGATTGTTGCCGCTTGGGCGTAGATTTTTCTTTTCCTTTTTCCTTTTTTT

TGTTTTTGAAGAGTATGAATCACTTCCTTCTTTTCGTCGATGATTTCTATTATTCCCCAT

CCCTCCCTCGGTTGATTTTTTTTTTTACACCTGTACGTGTATAGTTTTTTTTTTAATTAA

CTTGTGTTACCGCAGTGAATGTTGTCAAGAATGGGAAATATTATGAAGGGGAAAATGAAT

AAGCAACCTTCACTGTTTGGCTGGAGCAGTAAGGTGTACAACAAACATTTCCCAGTCTTT

TTTTTTTCATCGGATCCCCCCCCCATTTTTTGTGCCCGTTTGTGTCCGCCTTTCATGCAG

AAACGAATTGAATAGCGGTAACTGTGCTTCACGGGAAAACAAGTAATGAAAGCTGAATAA

AATGAATATTTCTCGTTTTGAGTTGTTTTTATGCTTTTATTTATCTCTTCGGGCAGGCAT

CCATTTTATTTCCTGCGTTTGTTTCCTTTTTTTGTCCGTGCCATTTCCCCCGTTCAGTCC

CGAGGCTCCTCCGCGCTATCGCAGTAGATTTTCGTAGATCCACGTATTAATTTTCTTTCT

GAATAATTATTTTAACTATTTCCTCCTCATCTGCGCTACTCTAACACAAAAGGGCTTTCC

ACAAGTGAGTTGGTGCAGGAAATTCTCCTACAAATTATATATAATTGTATATAGTAGTTT

CATTTCTACTATTTATTTTTTTTCCTATCGAAGTTTATTTTTTTTTTTGTTTCTGATGTG

CATATCGCAGACATCAAACCCTTGTCTTGTATTTGAAATTATGCATGGGGATCTATGTAT

CGGCGCACGCACGCACGCAAACACATGCACAAATACTATTTCCATTATTTGTTCTTATAG

AGAGAAAGAAAGAGATACGAATGTTTTTGACATTATGATAATATGAGATAACAAAAGAAA

ATAATAATCAGTAAAGGCAATAAAGGCAGTGATGCGTGCTGGTGGTTTGTGAAGGATGCG

GCGTACACCGGTGTTCCTGTTTTTTTTTTTCCTGTTTCAACATTGACACACCCGCACTGT

TGTAATAGGAAATATAGATTTCTCTCGTAAATATGACCAAACCATGTAAACGCATACACC

CACATGTATATATTCTAAGTGAGGAACACTTTAATCGCTAAAGAGAATAAAACAACGGAA

GAAAGAAAGAAAGATGGGGGAGAAAAGATAAATTGTGGTGACTACCTGAACGCAGCGAAT

TATATTTTATTTATTTATTTCTGATTATAATAAATTACCTTTATTATTCTTATTTCTTGA

GGAGGTGGCACCCCTCTCATGTGGCGAGTGAGGTAGAAAAGGAGGAGCAGACTGATATAC

ATATATATATTTATTTAAAAAGGGACAAATGCAGCGGGTATGGGGAAAAGGAAAAGCAAC

CTAAGCTGAAGAAAAGAAAAAGGTAAGGGAAACTTGATTGTTGAAGCGCGGTAAGGGGAC

ACATTCAAGCATTGATGAGTATCTTTGTGCACTTAGTAGCCATGTATCTCATCGTATATT

TACACGATTCGCCACATACTTAT

>Tb927.10.7700 | Trypanosoma brucei TREU927 | ABC transporter, putative | genomic | Tb927_10_v5.1 reverse | (geneCodeEnd+0 to geneEnd+0) | length=52

AACAAAATCGGGTAATGGACCTGAGAAATGCAACATGGGGATAGAATTTTTT

>Tb927.11.2410 | Trypanosoma brucei TREU927 | hypothetical protein, conserved | genomic | Tb927_11_v5.1 reverse | (geneCodeEnd+0 to geneEnd+0) | length=160

ACGCATGGGCGCGTCAACGAAAAAGAGGTAGGAACCCCACAACGTGCGGAACGAGGAATG

TGAAATTAAAATATTTCAAACTGATCTGTAGTTGCAGGTTTCCTTGGCGAGGCGGGGAGA

AAAGAAAGAAAGTGGAGCGTAAATAAATATAAGTAGGGAG

>Tb927.10.10000 | Trypanosoma brucei TREU927 | hypothetical protein, conserved | genomic | Tb927_10_v5.1 forward | (geneCodeEnd+0 to geneEnd+0) | length=979

AGATGCTGCTTGTGTTAGTGGAAAAGTTCGAACGTAAAACCCACCGTTTTATCCGTCTTC

TTCCTTTTATTTTAAATTCGAATTGAACTAGAAGGAAGGAGCTCAAGAAAGAAAAATGGT

AATTTGTTATAGTTTGTGTTTGTGTGTGTGTGTGTGTTTATTCTCATGTTAATGTTCATT

CTTGTTTTAACCTGTTAACAACAACTGTAATTAGTACTGTTGTGACAAGTATTTTATTAT

TTTTTTTCGGTGTGGCGGTAGCGAGAATAACAAATTTTCCTCTCCCTTTCCTCCCTCAGT

TTGTCCCCCTTTTTTTAATTTCACCCGTTAAGTTAGACGTAGCAAATGTGTAGGGCATGT

TGTAAAATCATTATTATACGAGCGGGTTTTAAGACATGTGGTCGTTGTATTAACCTACGG

TTTCGTTCCCTTGATTGAGAATGAAGCTGTAGAGATGGGAGTTAATGTTAATGAAAACGT

CAGAACGGGAGTAACCTCACGGTAACACAAATCACGTTGCGAGTGGGCTGCCGGAAAGAA

AAAAGGAAAAGTATAAAATAAAACAATACAAAACGAAAAAAAGAAACGGGGATGGGAGCT

GAGGAAACGCGCTAATCTTCCTTCCTGCCTGCGGGAGGGGTGTTGGTAATCATGATGATA

ATAGTAATAACCGTGAAGATTGTAATGGTAGAGCTTGGGGACGCGTAACGAGCGGTATTG

GTTTCCCCTTTTGTTGTTTTTGTTATTACTACTTATTTGTTGGAGTACCTACCGTGGTTG

TGTTTTTTTTTGTTATTTCTTCGGTTGTGTTTTCTCCACTTAGGTCGCTTCTTTACTTCG

CGTGCCAAGGAACAACTGAGAGACTTGCCACCACTTTCGCCACGAGTGAATATGGTGTTC

ATGATCGAATGAAGGGAAGCATGTGAGATTGTAAGGAAGCGATCAAAGCAAACGTGGGGT

AATCTTATCTGTTTTCATT

>Tb927.8.4050 | Trypanosoma brucei TREU927 | hypothetical protein, conserved | genomic | Tb927_08_v5.1 reverse | (geneCodeEnd+0 to geneEnd+0) | length=676

AATTTCCTAGACTATCACACAAACATGCGCAATGTCTTACTGTCCTTCCACTCCCATGCA

ACACGTGCTTGCAGTAACTGAGTGAGAGCAGGGTGGAAAGGTCTCAGTAACTCAACTGAT

CGCTATTTTCGCTATTCACTAGTTATTCATGTGCTCCCGTACATGTACATGTATTTCCAC

CATTATCATTCTTATCGTTATTGATCGGTCGTTATTTCTTTTTTTTGTTGTTGTTGGCAT

CGGCTCTGGGGTTACCCTACGCCGGCCTCAGTGCAGCCGGCATGTTTATACATAGACGCC

GGCTCTATCTAGAGATCTCTCCATCAAAATTATATTTTCAGCTGCTGGAGGTTCCTGGGT

TGCCGCCTCCTACCACTACGGTTCCGCATGCCACTGGTGCGGTGTCATCTGAGTGTGTAG

GGGACAAGGTTGTAGGCAACATATATTGGGGTATGAACTATCATGTTACAGTGAAGCAGT

GCACAATACAAGACTGTGCGGTCGCTACAGCGAGCACGCCTGTACAGCTTCAGGTACCCC

GACGCTTGCCGTCGTTGTGCCACAGGGGAACGTAATTTTATTTTTTCTATTCTTTTCCCT

TATTTGTCTTCCTTTGAGGTGAGTCCATGCTGACTCCAACTGTGAAACCCTCCGAAGGTC

CCCTATCTTGTTGGTG

>Tb927.8.1620 | Trypanosoma brucei TREU927 | MSP-B, putative | genomic | Tb927_08_v5.1 forward | (geneCodeEnd+0 to geneEnd+0) | length=402

AATGGCGTGACTATAGGTGTGGGGTTAAGCATCCGTTACTGTCCTTGGATGTTGCGTCAG

AAATGGTTACTACATGCGATACGCAGGTTTTCCTTTTATACGAGTATTTTCCATTACCAC

TTTGCAAGGTTGTTGTATTCCTTTCTTTTGTCTTTGTACACGTTTTTTTTAAATACTTTG

TTTATTATACGCACAGAACCCTATGACTCCAGAGGTTGTATGGGGAGAATGTGTGTGCGA

GCGTTATCGTCTGTTATTTCTTAGTCCAGTTGTCTGCGTTCTCACGGCAGAATCGTGGCA

GCAGCGGGCACCTAAACGATTTTAGGCCGCCACGTCGCGGTTTTCTGTATTGTTTTGTTG

ATTTCCTATTTAGAAACTCCCTGTAGATAATGTTTACCTTTT

>Tb927.4.2410 | Trypanosoma brucei TREU927 | Glycosyl hydrolase family 65 central catalytic domain/Haloacid dehalogenase-like hydrolase, putative | genomic | Tb927_04_v5.1 forward | (geneCodeEnd+0 to geneEnd+0) | length=609

AGGAAAGAAAGAAAAAGAAGGAGGAGGGGAAAAAAACGCAAGGATGGAAGGGGCTTTGCT

TTCCTTTTTGCCTTCCGACTAAACCCTCTATCCTTGCGTTTTTTCTCCCCTTCCTTCTTT

TTCTTTCTTCTCTCTGTATACTCGCTTTTCCCTCGTCACACACGCCTTACACATGCAGGA

TACGTCAGTCGCAGACGGTCAAACAGCACCGGCAAGATTAGAGAGTGTCTTCGGTGATTG

ACCGAAAGGACGAAAGAAAAAAACGCAAAAAAAATCCACATTAAAGAGACGGAAGAATGG

AGAGAGAGAGAGAGAAAAGGGGCGAGTGAATATATCAATAAATGAAGTAGAGTGGGATTG

AGAAATAATATTGCCTCGGCTGGTCAATGTGCATAATAAGGAGCGTAAATGAATGAAGAA

AGGAATGGGAAACGGGATGTGTTAACTCCTAAAGGTGTAGATAAGAAAGGTGTGAAGTGT

GTCTGTGTGTGTTTGTGTACGTGGGAAGGATGCACGAGAAAATATATGTGAATTGATTCT

CCCCTCCTTTTCTCCCATTTGGCCGTAACGAAAGGAAACGCAAGACGAAACAAATAAAGG

GTGGAGAAC

>Tb927.8.6580 | Trypanosoma brucei TREU927 | succinate dehydrogenase flavoprotein, putative | genomic | Tb927_08_v5.1 reverse | (geneCodeEnd+0 to geneEnd+0) | length=1216

GGAAAGTTATTTTTTATTTTTCTTTTTGTTGACGGCGGCATTTAGTGAGAGATGTGCGGA

TGTGCGTTAATAAGGAAGAAGCGTACGAATATTTCATAGTTACATTTCATGTCAGGGCAG

ACATACTGTGGCCGAGCCAATAAGGAGGATACAGATGAACCAGTAGAGAGTAGCGAATGT

TAGAAGAATCTAGGCAAGGTATAAACGTATGATGGAGGAGGAAAGAAAGTTACGCACCAG

TGGTATGACACGACGTGTTTTGTTTTGTTTTGTTTTGTTTCTCCTCCTTTCAAATTTGCG

CCCCGTAGTTACATTGTGGTGGGCTGAAAGGACAACGACTGGTTCATGTTCGTGTGTGGG

GGGTAACAATGCGGATGCCAACGGGGTGACTTCAAGTCAAGTGTGAGGGAAGCTCGTGAA

GCATGTGTTGAAAATACCTCATGTTTCCACTAGTGAGGTTTGAAGCCAACCAAATTACAA

ACAAGCAACAAAAACGGTAGTAGGGAAAGGGAAAAGAGAAAAGAGAAAAGGGGATGTTTA

CTAATATTCAAGTGTAATGTTCCAAAAAGTAATGAAGTGACTGAAACAAGTTGATGTTGT

TGTTGTTATTATTAAGGTGTGGAGGGCACACGAGTGGCAAGGGATGAAGTTATGCCACGG

GACGGGTCCTACTCAATGGAATTCATGCATATCATTGGGGTCGAATGAAATACAATAATA

AAGGATCCAGTGAGGGAGTAGAAAGAAGAAAAAGAAAAGGATTTAGGCGGGGCGAAAGCG

AAATGGGAGGGGGGGGGAGGGGGAAGAGGAAGACGAAACGAAATAAAGAAGAAAACAGAA

TTATGTATTTTACCGATACTCCATTGGCTTTGCTTCGTGCTCCTCGAAACCTTTTTTTTC

TTCTCTCTTCTCTTCTCTTCTTGTTTTTGTTTTTGTTTTTGTTTTTGTTTTAATATTTAT

TTCAGTGTCTATTTGATGTTCACAACATACATTCATACATATGTGTTTGTTTGTTTGGTA

ATATTCATTTCTCGTCCCTTAGGTCTCTATGTTCGAGGGTATTAACGGCAGTAGTGGCTT

GGCGTATGATTGGCTGTGGGTGAAGAAAACTTAATTTAATTTAAATGAAGGGGAGTGAAA

GAGAATAGCCCACCACCGGAGTTATTTCATGTGAAGGAAATAAGGAAGGAACAAAACATT

TGAAATGGCAAACTTT

>Tb927.6.690 | Trypanosoma brucei TREU927 | hypothetical protein, conserved | genomic | Tb927_06_v5.1 reverse | (geneCodeEnd+0 to geneEnd+0) | length=2094

AATGACTTGGATTGAGTACCATGCGTCTGTTCGTTTAAGTGCTATGCGATTTCTACGAAA

TATGAGGAATGAATAATAAAAGGAAGGTGGAGGCGCCAGAGGGAAAGGAACCGAAGAAAT

GAGATACGCTGCTCCTTTTAACCTCAAGTTTTGTTTACTATTATTATTATTATTATTACT

GTCGTGGCCTACTTCTGTAAGCTTGGGATACGCAAAGATGAAGGAAAAGAGAGAGAGAGA

GAGAGAGGAAGAATGGAGTAGAAGAGACGAGAAGTGTACAGTAGAAAGCAATACAGTGAA

GGCTAAGTGATGCTACGGCAGTAGTAAAAGAGGAGCAAACAAGCAAAATAACAGAAAGGA

ATGAAAAATAATAAAAAAGTAAACAAACAAACAACTGGTGTGATCATGATGTATGGTGAA

AAACCAAAACCAAAAACAAACAAAAAGCAAAATGAATGAATAGAGTAAAACACAAACACA

CGCATGCGCACGCGTATATATATATATATATATAAGTGTATACATAAATATGTTTATGAG

ATTTTAGGATGAAACAGGACAAACCAAAGGAGGGGGAAACAAATCAAGTTAAATCAGCAG

GAAAGAAAAAACGAACATGCTCCACCACTCCGATGGTGAGAATTTTTTTTCTCTTTCCCA

ACTTCCATCTCTTTTTTTTTGCTTTTTTTTCCTCCAACAAAGACAAGTAATGTGCTTACG

GCCTGTTTTTTTCTTTTTTCTCTTTTCAATTGTTTTTTTTTTTCATTTATTGCTATTCTC

TCATCCCGGCCGTATATTTCCCCCCTCCATTCGTCGTTATGCGACACCGAACACATCACT

CCTTTTATTCCTTTTCTTTTTTTTAGATAGTTTTCTTGTTTTTATTGTTTTGTTTTGGTT

TCGATGTCTGATGTTTTGTGTTCATTTGTTTTGTTCCTTCCATTTGTTACGTTTCATATA

TATATATGTACATGTAACTGCGTGTGTTTGTGTGTTTGTGATTATTACGCCTGCTTTTGT

TGGTATTAAAGTTTCTAATTTTTTTCTTTTTAATCCACTAACTTTTTCAATAATTCACAG

AAGGAGGAAAAACAGAAGCAGAAAATAAAAAAGAAAATAGATGGCGACCTGATAATAGTA

ATAAAAGTAATGAATGGTGTTAAAAAGAGAATGAATTAAAAAACGGGGCTAAACAGACAC

GAATATTTCAACAAGAAAAATGAAGGAAATATATCATTATATGTGTCCAATATTTTCGCA

CATATGTTTGTGTTTTCTTTTATATTTTTGTGTTTATATCTGCTGTTTTTGTGTGCCTTT

GTTGATTGGAAGATCAAAAGGAAAAAAAGGAGGAAGGGAGGAGAATTGGGGTTGTTTGGG

GGGGGGCATCTTCAGGAAAGAATATATATATATTAATATTAATATTAATATGTGTGTGTA

TTTGTGCGTTGGGGTGTTTGTATTGGAAGAGGCATGCAAAAGCAGCGCAAGTCCTTCCAC

ACTCATTCGTCCAAATTTTCCTTTTTCTTTTTCTCTTCCTTTTAAATTGTTTGTTCCGGT

TTATATTTCTTTAAATATATAAAAATTAGGGTTTTTTTTGGGGTTGGTGTTTGGTTCTTT

CTTATTTATTAATATTTCTCCTGAGCGCCTCATCACTCTCTGCGGTGGCGCGCCCCTCTT

CCTTTTCCCTTTTTTTTTCCTTTCCTTTTCCTTTTTTTTGTTTTGTTTTGTTGTTTCGTT

TTGTTTTTGCTTTTGTTTTGTTTTCATTTTTATGCATATGTGTGTTCCTATGGGGTGTGT

GTGTGTGTGTTTCCCGCTTCATGTGTGCTCACTCATGCATTTGATCTTTTGTTGTTGTTG

TTGTTGTTGCCATTGCCGTTTTTTTTTTAAAGAAAAAAAAGAAAAATTTAATATTACTAT

CATTATTTCTTTGAATTGCCATGTGTGTATTGTTTTATTTCATTCACGGTGTTTTCCGTC

TTCTCTCTCATTTTTTTTTCCTTGTTTACTGCTTTGATGCGTTGGCATCCGTTTATTACT

TCTGATATTTTATTGTTCTTTTTGTTTTGTTTTGTTGTGTGTGTGTGTGTGTAC

>Tb927.10.13410 | Trypanosoma brucei TREU927 | hypothetical protein | genomic | Tb927_10_v5.1 reverse | (geneCodeEnd+0 to geneEnd+0) | length=1

A

>Tb927.8.2780 | Trypanosoma brucei TREU927 | RNA-binding protein RBP10, putative (RBP10) | genomic | Tb927_08_v5.1 forward | (geneCodeEnd+0 to geneEnd+0) | length=4116

ATGGCACAGAGGGTAACGAAGTAGGAATTTTTGCCGCCAGCTGAGGTCGTTTACCTTGGG

TTGGCTGTCATGGAGATAGGGAAGAGAGAAGCAACATCGCGTGCAAGGAAAAACGAACAG

AAGAATCGATCCCTCCCCCCTCCATGAGTCCTTCTTCGTTTATAATTTCGGCTGTTGTTT

TTGCTGTCACCTTTTTTTTTCTTCCTTCTCGTTCGCCACACCCTCTCCTCAACCTCCTTT

ACCTCCATGGATCTCTCGGCAGCAGTCCCCCTCCCCTCATTCCCCTCATGTTTTGGTGGT

CGCCTTATTGGCACCTCTTTTCGTCATTTTTTCCCCCCACTTGCTGCAGTTCAAGGCGTA

TTTGTGGGGAGAGTAAAGAACATAAGTGAAATCTGAAAACAATAGAAAAGAGAGATGAAT

TAATTAATGAATGAATGGGAGAAATAAGGAGGAGAGGTGGTGGAGAGCTGGGGAACTGTA

ATGAAGAAGAAAAAAAAGAAGAACGAGGGAAAGGTAGATTCAGTGGGGGAGACAAAACAG

TGAAGAAGAATCTGGCGGAAAGCAACAATAAGTGTGAAAATGAGAACGAAGGTGTGAATG

ATTCTTCAGTTTAAGAAACCACACGTGAAGAAGGACAGAATATATAAATAAATATATCTA

TTTATATGTTGATGTCTCAGAAGAAAAAAAGCAAAGGAGGGGAGATAGAAGAGGTTTAAA

GGGAGGAAGGAAGACAGGCCCTGAAACCAATAAAACAAAATAAATAAATAAAGAAACGCG

AAATCAAAACTGAAAATGCGAAAAAAAAAACGAAGAACAAGAAGGGTGCAATGAAGGAAT

GAACTCCACAGAGAGAATACCCGTTCAAACATTCTTTTCTTCGTTTTTCCCCTCCTCCTC

TCCCCGACGCAACCGCCCCCCTTTTTTTTCTATAATTGGCTCTTCTGACCCACACCCCCT

CACTTTCCTTCTCAAAGAACGGCATCTTGTTTTCCCACCCTTTATTATTGTTAATTTCTT

TTTTCGCTTTACTATTATTATTATTATTTACCACCCCATCACCGTTACTTTTAGTTGTTT

TTTTTTTTTTTATGCCTCTCTCCTTTCATTTTTTTGCGCTTTTGCTCCTCCTTATTGGTT

ATGTAGAGCGATTTATATAAATCTAATATATATATATATATATATATTTGTATTCATATA

TTTTATGTCTTATATACATAACTACTTTCGCGCAGAAAAGGGAAAGAGGAAAGGAGAAGA

AGGGGGAAAATAGAAAGCCCATGCACATATATATATATATATATTTATTTATTTGTTTAT

TTATTTATATATATATATATATGTATTCTTTGTACCTCCCTTTCGTTTTTACTTTGTTTT

TTGTTTTACCTTTTTTTTGTTTCTACTTTGTTTCTTGTTTTGTTTACTGTTAGTCACTCC

TTACTATTTTTACCGCCTCTATTATTATCACTATTATTATTATTATTATTACTATTATTA

TTCCTATTATTATTATTATTATTCCTATTATTATTATTATTATTATTCCTATTATTATTA

TTATTCCTATTACTACTGCTATTATTATTGTTATTTCTGGGGCTACTGCAGTGCTTTCCC

TTTCTTTTTCTTTGGGTGTTCGCGTGGTTGAGATTGTGATGTGACTTCTGTTTGCTGACG

ATTGTTTGTTTCTATTTGATTGTGCCGGTGTCTGTTTTCCCCCACCCCCGTGATGTCACG

AAGAAAAACAAAAATTAAATTGAAAAGTTTTTACTTCTCCTCGTCCAAAGCAATTGCTCT

CCTCCTTCCTTTTCTCCACACGCGCGCACGTACGTAGTGAACTAATCAAAGAGTGAAGAA

ATAAAAATAAAAACACGCGTCGTGTTGTGGATCCCTTTTTCGGATCCAATTTGCGCTATT

GCTTTTTTTTGTTAGTATTATTGTTTTGTGCTTTTTTTTTTCTTTTTCTGTGTGTGTAAT

ATGATTTTGTCGCTTTTCATTCAGCGGAAGCAGACAGTAAAGTTATGGGCTCATATGCTT

GCATGTGCATTCCATCCGATATTGCGCGGGAGTTGTTGTTTTGTTTTGTTTTTCACACCT

TCGTCCCTTTTTTTTTTCTTTTTGGTTTTTATTGTTTGTATATATTTTCATATTTATGTA

CATATATTAGGAGCAAATGCATGCGTGTTTGTGCTTTAACGCCCATGTGTGCGCCCCTTG

GCTTGCTTTCCCACAAATACCCTTTCGAAACGCCTTACGGAAGCGGGGACAATATAATTA

AGAAGAAAGGGAGATGGGGGGGAAAAAAAAAAGAGAAAGAGAAAGAGAAACACCGGCTCG

TAGACATGAGGAGAGGAATCAAAAAAAAGACAATGAAATGAAATAAAGTTAAGTGAAGTG

AATTGAAACGAGGTAAGAATTTAAGAAGTTAAGAAAGCTGGTGACCCGCCGTCGCTTTTC

TACTTCATTCCACTCTTTTTTTTTCTTCTTTTCTTTTTTTTCCCATCCTTTTCCTTTTTT

TTTGCTAGTTTTTGATCCGCTTTGCCTTTGCATCTTATCTTTCCATTGCTCCCACTTTTT

TTTTATTGTTTCTTTTTTTTTCCCCCTGTTGTTGTCACCATTATTATGTCATGTTATGTC

ATCGGTACTACGACAGTTGCATTATGATAGTTATTATTTTTGTTCTTTCTTTTTTGTTCT

CGTTTTGTGACGTTGATGTTTGGTTTCATTTTTATTTTTGCGGTTACCTTTTTGTTTTTA

TTCGCTTTTTTTTTCCTTTTTGGTTTATTTATTTGTACTTGGTGAAAAGGAAAAAAAACA

AAAAATATATATATATATAGATACAGAAGCAGAGGAAGAGAGAAAGAGAGAATGAATGAA

TGAATGAATACGATGAAGAAGATATTGGAATAGAGGTGGAAAGGGAGGGGGAAAAAAAAA

AAAGAGGAAAGGGTGACGCGGTTGAGTTGACGGTAAACAAAACAGAAACGATAAGAAAAA

TAATGCACAAAATTCTTCCCCTTCTGTTCCTGTTTTTCGTTTCTTTGTTTGTTTTGTTTT

GTTTCGTCTTCTGTTTGGTACCGCATCACCCGTTACCATGGCCCTCAATATGTCTTTATT

ATCATTATTATTATTATTATTATTATTACTATCCGTTACTGTTATCCTTCATTGGCATGA

TGTTTTTCCGCCGATATTTCACATCTTTTCATTGACTCTTATTATTTTTCACTTCATCCA

TGCCGACTCTGCAGTACTTGTAGAAATTTCATTGAAACAGTATTTTGACGAAAAGGAAGC

AAGAAAGTTAAAAATTAATGTAATGTAATGCAATGCAATGTAATGTAATGTAATAAAAAA

GTAATAACAATGACAAACCTAATAACTATCATAATTATAAGAGAAATTGAAAGAAAGGAC

AAGGTAGGGTGGAAAAGGAAGCAAAAGAGGGAGAAGGGGGTGAAAAAAAAAATTAATTGT

TTAAGGCTTGAGAAGGGAAAACGGCACTGTAGTAGAGAATGAGAAGGAATAAAAATAAGT

GCGTGAGTAAATGGATGAATCAACAACTAAGTGAATGAGCATTTTCATGTACACAAAAAA

AAAAATGAAAAGACGTGTTTGACTCACAAAGGGGAGGAAGAATAGCATGAAAGGTAAATA

TTTGTGTCAGAATAAAAAAGAAACTAAGAATAAAAAAGAAATAATAACAATAATAAAGTG

AGGCAGAAAATGATGTTTCCACACCATTGGGATTGTTAAATGTTGCGATTTGGAGAGGAG

GGAACGCGTGTTGACTGACGTGGTGATGAAAATTTTTTTGTTTTGTTTTGTTTTGTTTTG

TTTTGTTTTGTTTGAGGGTCACACGTGTTCCACAACTCCTCCTTTTGTTTTATTTTGTTT

CCGCCCTCCCTCGTTCCCCCTTTCTGCGTTTCCCCTTTTTTTTTCTTTTTTTTTCTTTTT

TTTTTTCTGTTTTCTGTTTTCTGTTTTGTTTTGTTTTTTTTTTTTGCATCCCATCGATTT

GAGAGTTATAAAAGACGAGGAAAAGCGGAATGTTTCTCGTGCGACGAGAGCTGGACATGT

AAAACACAAAGGGAAATTAAGGAAGTAAATAAAAGT

>Tb927.8.6490 | Trypanosoma brucei TREU927 | protein kinase, putative | genomic | Tb927_08_v5.1 forward | (geneCodeEnd+0 to geneEnd+0) | length=3946

ATGGTCCGTATTCGTTTCGATAATATGCGGTGCCAAAGAGGGTAATGTAAAGAGAGAGAG

GAGGGGAAACAAAAAAGCAACAAAGAGAAAAAATAATAATGATAATAACAATAAAAATGC

GCGGTTTATTCAAGTGCAGTTTGCAAAACGGTTGCGGTCGTATTGCTTTTCAGTTACCGT

GGATGTGGAAGGGTTCCGCATCCGCATTCCCACTTTGTATGTGTATTTCTCAATGTGTAT

TTATGTGTTCATGTGTACGTTTATGTCTGTAGTCGCAGTTGATATGCAAGTTGGGAGCCA

TTTGGTTCTTGTGACTGCCGTTGGTCACCTTTATTTGTTTGTTTTTGCGTAATGTGCCTG

TTGTTTTTTGTTTTGTTTTTGTTTTTGTTTTGTATCTTTTCCCTTCCACCTCCACCTGTC

GCACGCCTCTCTTTTATTTATTTACCTATTTTTGTTTGTATCGAGTGATTTGTGACGAAA

CGCTGTCCTTTATCGTGAGGGGTTCTTGTTTTGTTTTTTTTTTGCATTTACTATGGAGAA

GTGAATCAAAAGGAAAATAAGAGCAAAGGTAAAGAAAAGTGTATCGAGGAACAAATACTG

CATGTACACCTGTTGTATTTGTGTTTATTTTACTTATTATTTTTTTTTGGCATTTTATCA

TTTTAGTGCGGAGGGCACGGTGCGGTGTGGTGTGATGTGTCCCACGAGCCTGGGTAACAT

ATATATATTTATATATATTATTGTCATTAAAAATACAACTATATGTTACACATGTATTCC

AGAAAGAAATTGAAATGAAAGGAAAAAGAGGGGAAGAATAAGTGCATGTATTGCACAATA

CCACATCCATTTATTTGTTTGGTTTATTTGCCTTGAGCGTCACGGAGAATAAATGTTTAA

AGTGTTAGTGTGTGTGTGGGGGGGGGGGTATTGTTATTATTGTGTGAAGAGGACATATTT

GTGTAATGACGTCTAGTGAGATGATGTAAGTCCTTTTATGTATTTAATTTAATTTAATTT

ATCTTCCCTTGAACCACGATGTGTTTCTCTCTGCTCTTCACTTCACGTTTACAATTGGCG

AGTGCTTTCCTTTTTCTTTGCTCGTATGCGCTACTGTGTGCGTGTGCGTAATATGTATGT

CCCCCGTTTTTTTCTTTCGTTAGTTTTATTTATTTGTTTTTCCCTTTGAGCGTTTACGAA

AACAAAAGAAAATAAGTAAGTAATCGTGTCGGCGCGCACTTGGTTTCGATCGGACGATGT

TAAAAGAAAGTGAAAGGATTTTTTATTTGTTGCTCACGTCTGTGTTCGTGTGAATTCTAG

ACAATTTTTTTTTTATTTTTTTGCTTGAATGAAAGAGAGAATAGTAGAGCGCGACAGGAA

CGAATCGAGATAAAGGGTTGTTGTTGTTGTTTTTTTCCAAGTGTGAGTTAATAAAATACA

AACACGACTCGTTACTTTATTATAATTTCCCTTCCCTCTTCATCCCTTCCTCCTCAGTGT

TGATACACGTGTGTTTTATCATATTTTTTTTATTAATATTATATTTATTTACTTCGATTA

CTTTAATTTTTTAAAGTCACGCATGCGTGACGCGGTGCACTACGACTTATTTGCATAATA

TATATATTATGTGTTATATCTTGATATGAATATTTATGTGCGCGTACGTTGAATTATTAT

TATTATACGAGTGTGACGAACATATAACGGAGTGAAGTGAAACAAGCCCGCATGTGTCAA

CCCAAATTGCAGCACTTTAACACGACTAATATAATGATAATAATAAATATTATTATCATT

ATTGTTATTAACGCCGGGGCTAACTGTTGCTGCAGCCCCGGATCCTATCTTATCGTCGTA

TATGTTTATCTTTTTACTTTTCTTTTTTTTAAAAAAAAAGATAATAAAGCAGTATTTATA

TATAAGTTCTTCCCTCCAGATTATTTTTTGTTACCTTCTTTTTAATTATTATTATTATTA

TATATATTATCTCCTCTTTTCCCTTTCATTCAATCTCATTATCAACATCATTGCTGTTAA

CAACATTTCTTTTATGTGTTTATGTTTACTTCTTACGTATTCGTTTCGTGCTCACTTGAG

TTTATGGGTGAATTAACCAGACTGGTGTTTTGTATTTGTTTATGTAAGTGTGTATAATTT

AAAGAAGAAAATGGTTGAAATATGAATAAAGATATAAATATTTGAATGAATAAATAAATA

AATATATGCGTGTGTATGTGTATGTGTATGTGTCATCCATTACTTTTTGGTCTCCGCGTG

GTGGTTGAGGAATGAGAGAAAAATTAAAAAATTAATAGAGAGAAAAGTGTACAGGTATCT

GAGAGAATGCATTGATTTGATCCTATGAGGTGCATTTTGTTATATAAACTCAATTGCGTA

TGTATGGGAACGGGAGGGAGTGAAAAGCCTGATTTCTTTCAATAAATAAATATATAATAT

GATATGAAAGTTATTTGTTTCTTTTTTTTTCTATTATTATTATTTTGCAATACTTACATC

GTTTCGTTATATGTGTTTGATCCCTTCACTATCCCTCTCATTCAAACTCCCACAACTTTT

CTTCGGGCCATCAACCCTCCACTTAAATATGTCACTTCTTCGTTTTATTTTTGTTTTGCT

CATAACCTGCTGCTGTTTTGGGGTTTTTATTCATTTTATCTGCTCAATTTATTCATATTC

GCTTCTCTTCTATTCTATTTTTGTTTCTTTCTTTTCTTTTTTTTTTGTCGTTTGACATTT

CTTTTTCGTTCTTATTGCTGTTAGGTACCCGCAATTGTTTTAGTTTGTATTTCACTCACC

TCGCCAGAGCGGCATCTTCTTATTTTGTTTTATTAGTTCGCTCCCCCTCATTGTCTCTCC

TTGGCTTTTCAGCTACTCCAAGAGACAAACAAGAAACAAAAAAAAAAGAACAGAACAGAA

CAGAAAAAAATATATTATTAAAAACAAAAACAAAAGCAAAAAAAGAAAGTAAAAGGGTCA

CTTTATCTTTTTGTAAACTTCTTCACTTTGATTTCCACTTGTAATTAATTGACCTTTCTG

TTTCATAGTTCCTGCTCCCTTGTACCTGTTTATTATTATCATTATTACTAGTAGTGTTTG

TATTGTTGCGGCTTATTTGCCTTCACGCTAGTAACTTCGAACCGTTGACGCACGGATCTC

ATTGATTGATTGGGGAGATATATATATATATATATATATTCTTCCCTATCAAAAATTAAA

AAAGAAGGAAGAGAAAAGAGTATATATTGTTATATAAAGGATTTTAACAACTAAAAGTAA

ACAAAAGGAGGAAGGGAACCCCGGCGTTTGTTATCAACATACGACGACACGTGAATTCAA

GCAAACACCACATATATATTCCCTCCGTTTTTTTGTAAGTTTTTTTTTTTGCCTTTTGGT

TTTGGTTTTTTTCTTAAAACAAATCAGAAGAAAAGGCAAGGAGGCTAATTGAAAAGAAAA

TACCAAATCGGAATAAATAGGTAAAGGGAGGGTCAAAAAAAAAAAAAAGAACAGGAGAAC

AAAAAAAAGGGAAATACCCTTAGTTTTTGGTGAAGTGAGAGAGCCCTGTAGTTTCATTTT

TTTTTTTCAAATATATATCACGAAGCGGTACAGCGAAAGGGAAAGGAGAAGAAGAAGAAA

GACTTTATCGAGTTTTTTTTTTCGTTTGCTTTATATCACTTTTGTCGTTGTTTTGTCCTG

AGGTGCTTGGACGCGTAGTGCGAAAGAAATTAATTTCAGAAGGGAAACCGAATCGACAGG

TAGCAGATAAGCCCATTTGCTGTTGTTTTACATCTTTTTAATTCACTCCGTTACTTGTAG

AAGCAGAGGGTCGTGGTTGACTTGTTTGTGGTTTTTTTTGTTTGTCTGTTTTCTTGTTAT

TGTGTGTGAGTGCATTTGTGCGTTGTAAACTGTTGGCGGGTGAACG

>Tb927.10.8050 | Trypanosoma brucei TREU927 | TFIIF-stimulated CTD phosphatase, putative | genomic | Tb927_10_v5.1 reverse | (geneCodeEnd+0 to geneEnd+0) | length=3243

ATATTGGCAAATGAGGGAATTTAAGAGATGTTTTCCAGGAAGAAGGGCGAAGGACTTAAA

GTGGAAATAAAGCGCATTGAGGTGGATGCTTTTATCACCCCCCCCCCGCCGCCGCGCCAC

CATTGCTGCCCGGTTTCCTTCTTGGTTCTCTCCTTTCGTTGCTCTTTTTTTTTTTTTTGT

CAATCTGCTTATATGTTGATTTATATTCCTTGTTGTACACTGTTTCGCCACCGCGCCCAA

CACCGAGGAGTTATTTATTGTGCGTTGTTCCAGACACACGCACACAAACACACAATTTAT

GGGCACGGGGCTACGGCACGGAAACCCCATTGCTGTTGTTGTTGTTGATTTGTTATTATT

ATCTGTCTGCTAGTTTCACACTCCCCTCTTCTTCCCGTAACAGTCGAGTGCGGCTCAGTG

CATGGGCCGACCCGTGAGGAAAAGAAAAAAAATGTGAAGGGATGAGAGGAGGGTAATTTA

TTTTTATTTTATTTTCGCCCCCTCACATATACGTGTCCACACGCTCTTTCTGTGTTTTTT

TTGTTTTGTTATGGCCGTTCTTTGGTTTTGTTTGTTTCAGGATTATTACTTTTGTTATTA

TTCCTTTTATTACTATTATTATTTTAGTTGTTTTCACATGTGCGGAAAAGCTTCGCTCAT

TCCCACCGATATGACACTTTTTTTTAGATTTTATTAGCCCGCAGTTGTTAAAATTCCGTT

TTCACTTCTCTTTTTATTGTTTCGTTTTGATTCTTAATGCTGCCATTCGTTTCTTAAGTC

CTACTTTATGTTCCTTTTATTTCCCCTCCCTTCCCCTCCTTTTCGATATTTTGTTTCTAT

AAAAAGAAGAAAGATAAATATACAAATGTAATATATATATATATATATATATATATGCTT

TTCTATATTTTCGTTTATTTTATATAATTACTTTTATTATTTGTATGGTCGTATTCCATT

TTGGGAGTCTGGTGTTCTCGTTTTGTTTTATTTATATTTATGTTTGTAACTATCTACCGT

GTGGGCCGTGTCGTCAGTCTTCCATCCAGTCATTTTATCTTAATTTATTTCTTTTAAATT

TACGGGAGAGCCGTTTGTGCACGTGAGGGAAAAAAGGGACTTCAGCCATACCTCTTTTTT

TTTCCTTTAAGACGAAACGGGTTTATATATATATATATATATAATTACAATTATAAAGAG

AGGAAATGGTAAACAATCATAATGATATCATTTTCAATATTACCACAGGCAAATACATGT

TGCTTCACTTTGTTTGTATGCTTTTGCATGTTTATATTCGTTGCTGTCGTTCTGTTGATT

TTATGTTATCTTTCTCCCCTCATACACCAGTGAATGTATAAGTAAGTAACTGAAGAAAGG

CAAAAAAAAAAAAGAAGCAAATAATTAAAAGTGATCGCTCTTGGTGCGTCGTTGCTTCTG

TGTCTTTCTTTTTTGATTATTATTATTATTATATATATATATTTTTACTTACTCTCCCTC

ATTATTGCGCTGTCCGTTTTTCCAAAACGCATCGTCGTACACAAAGGGAAAATAAGATAA

TATGTAAATGTACAAATGTGGCCGTGTGTGTTATTTCCCATTAAATATCAATATCAAATA

AACTAACGGCTGAAAGTGCTCATACCCATTGGTATATTTATGAATGTTTTATTTGTTTGT

TCGTTCGTTCGTTCGTTTTTGCTTCTGTGTTTTATTATTATTATCATGTTTTTCTTTGTG

CACCTTGTGTTTAATAATTTTAGTTATCGCAAGTGGCTGCACCTCATTTATTTATTTGTT

TGTTTCATTCTACACCAACTCCGTTGTCAACGCTTCACTTTATTTCACGTTTTTTTTTCT

TTTTCTATCGTTTGATTTCTTTTTTTTTTTTTTTTGAATGTTGTCTGAAGGTATTTCAAG

GCAGCGTGAAAACAGGGGTGAAAAGGGATGATTTAAGCGGAGAGTGAAAAGGAGGAGGAG

GAAAAGGACAAATACATTTTGTTATTGTTGTCTTATTTTCCTTTAACTCATCCGCATTTA

GTTTACTACTCATTCATTTCCATTCCTCTCATCTTGCAGCGTGTCAGTGGTTTCCTTGAT

TTGTTTGTCTTATCATCATATTATATTGTTGCTATTATTGTTATTCTGATTGGTAGTAGC

GATCATAAGTGCTGTCAAGTTATGCGGTCTGTTGTTGCTTTCACAACTTCGCCATTCTTT

TTTTTTTCTCTCTCACACAGTCATGTGTAAATATAACTGAAATAAAGCGAAAATGAATAT

ATTTGTGTTTATGTGTTTATGTGGGTCAGCGGTGAAGATGTTAATTACGGTGATGATGTT

GTCGTTATTGTTTTTATTGTATTTTTTTTTTTTTTGCCGCTGCCGCCGTGACGGAACCGT

TTTTCATACGTCACTTTTTAGTTATGCGCACCTGTACCAAATGGCACAAAAATAATGAAG

CATAACGTAATATGATGTGGCATGTCATGATTTAGCGAATTCAAGTGATCGATCGGAAGA

GTAAAGAAAAGAAAAAAAGAGAAAGAAAGAAAGAAAAAGGTAATTGATGCAGAGGGAACT

TCACTTTATTTTATTATTTACTCACTTATCTCTGCATATATATTTTTATGCATCTTGTTG

TGATATCATCCCTCCCTCTCACTTATATTTTCATTTTAAAGTGCGTGCGCATGCGTTTTA

TTTGTTTTGATTATTTATTTATTTATTTTTGGTTGTTTCCCCCTCGGATTTCATGAATTT

TCCTTTCTTTATTTTTTTTTCTCCGTTTTACTTCTTACCTTTTATCTTAATTAAGATTTA

TTACTTGAGAGTGTAATCGATCGTTTTGAGGAAAGCGGAGCTCAGGCACAAAAAGTGGCA

TATGCGTGCATTGCTCAGTGGGAAAAGTTTTTAAGGAAAGGGACCGTTGTTTTTCTTTTC

TTCTTTTTTCCCATTCTTTGTTTTCATCTTTTATGTTGTTTCACTTTCATTTCGCTTCAC

CTTATCTAATTTTATATCTTATTTTATCTTCTTCTCATTGCGTATTTCAATGATAAGTCT

TCTGCTCGTTTGTTACATTTGCTCCAAATTCTCATGCCTCATTTAGAGTTATTAAGAAAA

CAAAAAAAAAAAGAAAATGATTGAATCAGCGAATTCGCCCTTGACTGAGTGTTTGTAAGT

TCTCCAAGAGAGGTCCAAATGTGGTCAATACCCACTTTTCATGACTGTTAATTATTTTGT

GGG

>Tb927.9.4550 | Trypanosoma brucei TREU927 | hypothetical protein, conserved | genomic | Tb927_09_v5.1 forward | (geneCodeEnd+0 to geneEnd+0) | length=811

ATTCTTGAAGCTCCGACGAGTCACGTTCCTCCTAAAATTTACTGTTTCGTTTTTTAAATC

TATTTTATTTATTTATTTTTGTAGGTGCCAATTTTTATTCAATTGCCTATCGTTGTGACG

AATGCGATATATATATATATATATATATATATATATATATATATTTCTCTTTCTCCCCTG

TAAAAGACCGGCATGACATGACCGCAAGTAACGAGATGACAGTTGTGGTGTCATCATTTG

TTACAACGAACGAGGGGAGCCACATATAAGTCTTTTTCCCATTGAACCTTCTCCGTGATG

TAAAGTGTTGATGTGCAACACAGTAAAGGAGGACTGGTGTTGGGGTTGAGGGTACCGAGG

CAGTTATTTTTTTCTTTTATATATATATTTTTTACTTTTCTTCCGTTGTTTACCTAATGC

GTTTATGGGGTGTACACTCTGTCTCCAACAACATTACTAATATTATCACCGTCATTATTA

TCTCGCTTTCAATATTTCCTCTGTATTTTATTTGCTTTCCAGTAACTTACCTCAGTTGCA

TGTGGGCTCCATCACGTAGCCGAGGATAGTCACAAGGAGGCGTGCCTTCCTTCCTTTTTC

CTTTTTTCCTTTTTTTTTTCGTAATGTGCGCGTGTGTGTCTCTCTATGTTCCCTTTCCCT

TTCGCCTTCAGGCTGTGAATATATGATAACGCGCTCCAAGCTGTCTTGGGATTGGAGAGG

AGCAGTGGTGTCGTGTGTGACTGTATTGGCGGTAGAGAAGTGTTAAGAGCGGTATTCTCT

GAACCTCTTCAGGCGCCATAGTGTAGACATG

>Tb927.11.15010 | Trypanosoma brucei TREU927 | serine/threonine-protein kinase, putative | genomic | Tb927_11_v5.1 reverse | (geneCodeEnd+0 to geneEnd+0) | length=2214

GTTTTGTGACGGAGGCTATTTTATTACTCTTTTTTTTTCTCCCTGTAACGCTCCCATATT

TTAGTTTTCCTATTTTTTAACCCTTTCCCTTTTCGGTTCTTAAAGCAAGGGAGGAAAGAG

AAAGAAAGGAACAGGTGGTGCAGATATTGGATGAGACGCAATTAAATGTATTCCGAGAGC

TTATCCACATCAACATATGTTTATCGTTATTTATTATTATAATTATTTATTTAATTAACT

TTACTAGCAAAAATTTCCGTAGCATATAAATAAACTCGTCCCTTTAACGTAGCATACTTC

TTTTTCGATTGTTTTTTTTTTTTAAAGATACCCCGTTCGACATTCAGGTTTGTAATTTTT

TTCAGGCTCTTTTTCTTTTTAATTATGTTAAGGCACCTCATATTATTTTAGTTGCCCCAA

TTACATGTTTGGTTGTTATCACCTCCTGTTCTTTTTTTTTTTGGTGGCAACTAAATCTTT

GTTTACTCCCTTTACTCCCGTTTCCTACTTTATTTTATTTATCATCCCTATTTTTCACTT

TCCTCTAACATGGGGATTGTTAGTCTGTGGCCGTTATCATAATTATTCAAAAAAGGAAAA

ACCTGTTGCCTCCGTGCTTTATGGCGGGAATTTGTTTATTGATAGTTTAATTTACCCTTT

TCTTTTTCTTTTTTCTTCCTTCTCTTTTCGCTTTGACCTTATTTATTTAGTTGCCTGGTG

TTGCTGTTGTATATCATGGCTTTCCATGAAGCTCGTTTTATTTATTTTTGATTTGATTTG

ATTTGTTTCATCTCTCCATCTCAGTGCGGTGTTATTTTCGTTTTCTCTCTCCCCTTCCTT

CCAGCCCCGGAAGTGTGACTGTTTTATTTACTTTTTCCATTCCGTTTCCCTCCCCCAATT

CACACATAATCCGTAATTATTATTATTATTATTTATTGTTATTGTTTACATATAATTACT

ATTTTTTTATTTTTTATGTTTGTTAGTGTGTGATGGAGTTATGTGCGAAGGACAACGACG

CAAAGGCATTGCTCTTTCCTTCCCCCCCCTCGTGAGCGTTCCCCATCCTTCTTTTTCTTA

TGTTTTATCTTGTATATATCTCACTCTTTACGCAATGTTTGTGTGGTTGAATAAATAAAA

ATGAAGGTGTACATGGCCAACACGTTGAGTGTTTCTCCTTTTGTTGCTGCTCTTGTTGTA

TTTTATTCATTTGTTTTACCCTTCGTTCATTTTCTCCCCCATTTTTTATTGTGCGTAATG

CGGAGTGTGAGGGGTACAGAAGGTGGTGTTTGTTTGTTCCATTGGTTTCATGCAATAGTT

ACTATCCCCTTCATCTTCTTTTTCTCCTTCCTTTTTCTTTTTACACGCTTTTTACTATTA

CCCCGTGTGTTTATTTATTTACTTACTTCCATATTTTTGAATTTTTTTTCCTTCCCATTT

TTCCTTATTGTTTCTAATTTTGATTATTATCATTATTATTTTACTCTCTTTCTCTACCAT

GCATTTTTTTTCTTTTCATTGCTGTTTCTTTGTTATGACATTGTCACATAAATAGGTATG

CGGAAGTTTACATGAGTTGGTTACAGCCTCTCCTCTGTAGCGAAACAGATCTTGACAATT

AAGACGTTCCTAAGAGAAATAATATTCAAAGAAAACGCGCGAAATAACAATGCATATTTT

TTCGGTTTGTTTTTTTTTCCAAGAGTTGTGCAAATAGGAATAAAAGATGAGGAACTTTTG

ACACTCACCACGTAAACCTCTGTGTCATATGAGAATACTCGATGGACTATTAAACCATGA

GCGAGGGATGATAACATTTATTCACTCCGGGTTGTGTTTACCTTCCATTCAGTGGTCGTG

CGCTTCTTTTTTTTTTCTTTTTTTGTTTCCACTCGCCAGTCCCTTCCTTCCTCTTTAATA

CAAATAGTTCCTCTCACCTAATGTGGATTCTCAGTGGTAATGCTTTTTTGTCACTTTCGC

TCATGCACCGCAACAAATGTTACGTGCGTGTGTGGTTGTGTTGTGATGAAACCTTTGAAA

TCACTTGAATCAACTGTGTGAAGTTGTCTCTATGCGTATGGCGGGTGAAGGGGAAAGCAA

AAAAAAAGAAGAGTAGGAGCTTGTGTGTGATGTAAGTGAGTGTGTGGATTGTTCCCTCTC

TGTAGTTTGTGGTTACATAATTAATCTTTCTGTCTGTATTGTGCTTCTCTGTTT

>Tb927.10.2210 | Trypanosoma brucei TREU927 | Peptidase C19, ubiquitin carboxyl-terminal hydrolase, putative | genomic | Tb927_10_v5.1 forward | (geneCodeEnd+0 to geneEnd+0) | length=851

AATATATATACATTTTTTCTTGTGGGTAGTGCCGCCCCATCCGCCCATGCACATATACAC

ACACACACACATATATATATATATATATATATATGTACGTACATGCAGATAAAGGAGTGT

TAGTGTGGAGTAATTGGTGTAACATGGGTTGCTCAACAAAAGAATAATAGTGAGAGAATA

AGTAAAGCAAGCGGGAAGTTGTATTATTAGTTTTTTGCAGTTGTCGCTCGATGTCTTCTT

ATTTTCTTTTCATTTACCTCCATCGCTTATCCTTTTTCGTTTCCATGAGTACTTTCCGGT

TTTGTTGTTGTTCTTGAGTGTGTCAGTGCACGGGTACTTTCATGACATTTTGTAGCCTCA

TTCTTTTCTTCTCTTCATTTCTCGCCATCCGTTAGAATCGTCCGAAAATCTGCTGCGCCT

ACTGGTCGTATTTTTTCTTTTTTCTTTTTTATAGAGTTAAGGCCATCAGTGCTGCTGCTC

CGACGCACCTTTACGCAGTAATTAAGTAAGTAATGAACATAAGGGCGAGGAAATGGGTGG

GGATTTGCGTCAGTGCTTTTGTGCTGCTGCCGAGCGTTTCCTCACGCGAGTGAAGTTCTA

CGCATTCATGTGATATTTAAATTTTTCCCTTATTTATTTATTTCTCTATCTGATTTCGAC

GCACGGCATGCAGCAGTTGTGTGTGTGTTTCGCCGTTGACGGAAGGGGACGATGTTGTGG

TAAGTGACGGAGTGAAGCGGGGAGAAAATGAAAGTAAGAGTCAAACTGATGTTGGAGGAT

ATTAGCTAAAGAGAAGAGGTGAAAAAGGATGGGACAACGGGTTATTTTCTTTTAAACAAC

AATCTACACAC

>Tb927.4.4940 | Trypanosoma brucei TREU927 | hypothetical protein, conserved | genomic | Tb927_04_v5.1 reverse | (geneCodeEnd+0 to geneEnd+0) | length=2858

GCAGTTTCCAAGTGCTTGAATAAACAGGAGCTGTGTTAACAATTAAAAACGAAAAAAAAA

AGGAAGAGAGAAAAAAAAAAAAGCATATCTAAAGATGGGGAAATAACAACATAAACACAT

GCAAGACCGCACACAAGCAAACAACATACATATGGACATATACATACATATATATGTACT

TTTGTATTTGAAAGAGTGGACCCTCAGTGGTTTATCTCAGCACTGTGTTCAAACGCTTCC

CAACGTATGGCCCAAAACGAGTTTCTTTTTTTTTTGTTTTGTTTTGCTTCCCTAAACGAT

TTTCCTTTGGTTTGATTGTGTGCTTTAGCTCGTGCTGTATTTCACGTTCGTTTCGTTGTG

GCATGGCGGTACATCCCCAAATATAAAAGAAAGGAAAAAAAACAAAGAGGGAAAACATTC

GGGGAAGCGCCTTCCATTTCTTTTATTTATTTTCGTTCGTATGTCAAAAGCAACAACAAC

AACAACAACTGATTTTTGGCCTGTTGGTGTCGCACACGTATACATTTCACGTGTACTTCT

TCTTAAATTCCCTCACCTTCATTTGTTATTATCATTATTTTAATTGTTATTGTTATTATC

TGCTTTTTTCTTTGGTTTTGCTTTGTTTCATGTTGTTGCCTCTTTTTGTTGTTCTTTTTT

TTTTTTATTGTTGTTGTTTCTACGTTTACCTGATTCAATTTATGGTTCCCTTCCAATATG

ATTTGCGACTATTCGGATGAACTATAGAAAAGATAATTTAAAACAAAAAAAAAAAGACAA

AAGAAGAAACTAAAAGAGGAAGGAGGAGACGGGGGAAGATATCGGCTGTTCTCTCATCAA

GATAGAAAAAATAAAGCAGAAAAATAAACAAATCAAACACAAAACTTTGTAAAAATAAGA

GAGATTCATATGATTCACGCAAGTCAATTTGTATAAATAAAGCATATATATATACTTATA

TGTTTCATATTTGTACGGCTTTGTGTGCACGTGTGTGTATATGCCGGTCTTGTTCAATCC

CTCACTTCGCAGCGCATAAACTCACTTTTATGCTTTCCATGTGTTTGCGTGCATTTTTTA

TTTTATTTTTATTTTTGTTTATTATAAATGCCGGTGTCACCAGTCACCAATTTGATTCAA

CTTTGCCCAGCACTTAAACGCCTTAACACGCATACTTTTTTCCTTGTTTTATTTTGTGTG

TTCCTCTTGCTCTCTATTTTTGATTTTGATTGTTTTCCCGTCCGTGTCTTTCGATGCGTG

ACGCGGTGCCTGTCGTGAGACATTACTTTTTCATTCATAATTCTTTTCTTTTCCCTCTTT

TATTTCTATCGCTTCCGTTTTTTTTTCTTTTTGTTTGTTTGTTTATGGTTATGGTTATGG

TTATGATATCGTTTTTGATTCGATTGCTTGACATTCCTGAGTGAATGAGTGAAGAAAAAC

TTAACAAATGAAACATGAAAAAAAAATGACAAATATTTACATTTATATTATAATTATATC

GTCATTATCATTATTTATATTTTCATGAACAAGGGAAGGAAAGGTTTTTGTTCTCAATTA

CGTGTACGGTACATCTCCTTCTTAATGTTTACCGCTGCTTTCTTCTTATTTTTCTCAAAT

ATACGCTAACCGGCACTGAACTTGACGCCTCCTCCATATTTTTTCGTGTTGTCGGTTATT

TTCTTTTTTCTCTTTTGAACTTCGTTTTGTTGTTTTATTTTTTCCAATTTTTTCCCCTCC

CCTTTCTCTTCTGTTTTATTAGGTTCACTGCCTTTTATCTCCCGTATTGGTTTCTTTTTC

TTTTGAATGCGCACATTTTTTTTTTGTTGTTGTTTTTAAAAGAAAGGGGGGGTATACTTT

GTTTAGCCGCATATGGTTTTTGCCACTTGACGGCGGCTCCCCATCCACCCCCTAAAAAAA

TACTAATAATAATAAATAAACGAATAAATAAATGAAAAAAAAATCAAATTGTGAAAACTT

TAAATATATATATATATATATATATATATATTTATTTATTTATTTATTTATTTACAAGTC

ATATAAAATATAACAATTTTGGGGGGCTTGTGGCTATTTGAAAGGAAAAAAGGAGGAAAA

ACTGAGTTTTGTCCAAGAGAGACGGGAAACATTGGGTGGAGTGATGCTAAAAACAAAAAA

AACAAAAAAAAAATGACGCATCACCCTGTCAGTCCCTGTGACATTGAGGGAGGAAGAGGA

AATAAAACAATTTTATGAATGAATGAGTGGAAGCCAACACAAAGACTCATTTATTCATTT

ATACGCAAACACATAGAAATATGAGTGAAACATAAAACAGATACAAAAAAGGGATGAAAG

AAAAGGATGAACAGGATAAACGAGCAGGAATTTGCGCCTCTCCCGCATCGACCGCCCTCG

GGGGAGGTCACATGTTTATACTTTTTTTTTCGTTTCGTTTTGTATGGGAAACAATAAGAA

GCTAAAGAGAGGAATAATGAATTACTTTGTCATTTCATACAGCAACTATACGGTAACTCA

TCTCTTCTGTTTTGTTATTTTTTCTTTTCTTTTTTTTTTCGCTTTCTGATACAGTTCCTC

ATTTGTTTTCTTTTGGTTGTTCATTTCTTGCAGTTGCCGTGTTTGTTACGTTATTTTTCT

CTATGTTTCTGGTTTCTGATTTGTATGAATATGTCCCACGTTTTCATTATCCAATCTTTT

TTTTGTCTCTTTCCAGCCGTTCGTGGAAACTTTGTGCATACGAAAGAAGAGGAATAAACG

GAATGGAACGAAGTGGAGTGGAGTGGAGTGGAATGATGACATCCTTCCATTATGTGACTA

TTCGGTTTGAGTAAATGAAGTAGTAAAGAGGGACAGAC

>Tb927.10.2360 | Trypanosoma brucei TREU927 | hypothetical protein | genomic | Tb927_10_v5.1 forward | (geneCodeEnd+0 to geneEnd+0) | length=514

ACGGGAAATTGATTGACAAAAAAAAATAATAATCAGAAAGTCAAGAAAGGAAGAAAGAAA

GAGGGAGGAGGGAAGGAAGAAAGGAAGGGGGGAAGCACGCACCAGAAGTGTAGGAGAGAA

GGAGTAAGTAATAATAAAAATTTTAAAAATAATTATAATTATAATAACAGTGCGAACATA

TTTTATGGGGTTAATAACACAGGGTATGGGAAAACTAACACATCTGTGTTCTCACGTATG

TGTGGTGAAGTAACAAAGCAGGAATAACAACAACCGAAGGGAGGAGCGTTTAATTTCAGA

TGCAAATGTGTTAATGTGATGGAGTTGGCGGAGGGAAATAAGGTTAACGCTTTTTTCCAC

CACCCCCCTCCTCCGCTACATTCCTCTTTCTTAACATATGGCGTGTTGTTTTTTTTTTTT

GGTGAATCAATTCGAATGAGAAATGTCGTTTCGCTCTGGTACGCCGCTGGAGCCTGAAAC

TCTTCATGTGTATTGCCTCGACAACAACTTCTAT

>Tb927.11.5050 | Trypanosoma brucei TREU927 | fumarate hydratase, class I (FHm) | genomic | Tb927_11_v5.1 forward | (geneCodeEnd+0 to geneEnd+0) | length=265

GCGGATGGATTTGTTGTTGTTGTTATAAAGAAGAGGCGCTGAGAAAAGGAAAGGGAAACG

AGGAGGAAGAGAGAGAGAGAGGCAACCGTTGGGCATAATGTGCTCGGCCAAATGAATATA

CGCGTATATATATATATATATATATATATAGTTTAAGCTCTACATGCGGTGGAAATGGAA

TGTTTCCGCGGGTGAAGGGAGAGGCACGGAGGGCGCAAGGAAAGAGGAGAGGGATATTCA

AATAAAACGAGAGTATTCGACAAAG

>Tb927.7.210 | Trypanosoma brucei TREU927 | proline dehydrogenase | genomic | Tb927_07_v5.1 forward | (geneCodeEnd+0 to geneEnd+0) | length=414

AAGTAAATTGACGCGATTTAGGGATATTGCTCCGTTTAAGTGTATGATTACCGCACCCCG

ATTATGGGAAGGTATCAACTTGGTGGCGAGTGTAAGATTGCGCAACTGCACCATCTTTAT

GCTGTAAAGGGTTATTGACTTAAAGGTTCTTTATGTGTGCGTAGATAGTTGTTTTTAAAT

GATTTTTTTTTGTTAACGAACGTACCCTATCTAGTTTAAAAACGGCGCAATTTTTTTTTT

ACTCCTTTATTTTTTTTTTTTTGTACTTCCACGTTTTCGTAAGACGCATTTCTCCTCTAT

TTTTCTATTATTATTATTATTTTTTACCCTTAACCTTGTTGGGAGCGTAGATAGCTATTT

GGTTGATTAAAGACGCATTAGCGTTTTCCTCATATATATTTTTTATTGGGCCAG

>Tb927.5.3040 | Trypanosoma brucei TREU927 | MIX protein | genomic | Tb927_05_v5.1 forward | (geneCodeEnd+0 to geneEnd+0) | length=16

AGCGCGGAGTGCAAAC

>Tb927.11.15550 | Trypanosoma brucei TREU927 | NADH-cytochrome b5 reductase, putative (B5R) | genomic | Tb927_11_v5.1 reverse | (geneCodeEnd+0 to geneEnd+0) | length=2176

ACGCGAAAATAAGTACAACCGAAAAAATATATATATATATATATAAAGGAGGTAAGAGAA

TGGGAATGTGAGGATAGTGGACCATTGCAACGGACGATGCTCAGCAGGCCACAAAATGTA

AGCAGTAATCGTCAGCGACGCATTAGCGAAAAGGCGAAGGGATCATATTTTTATAACGAC

AAAAAGAATAACAGATGCAGGTTGAAATCAGTGCAACAAATGCGGATGCAAGGAATACAG

TGAAGAAAACAAAAAGGTACTGGGGGATCGATTACCATGGGGCATCCCCTCTTTCTTGTT

TCTTAGGAAAGTACAATCACTCTCATCACGTTAGTTTGCATACTGCGAGCACAAGAAGCA

CTTATTACTTTTCCTTATTTTATTTATTTTATTTTATTTTTTTGAAATATAGTACATCTT

CTTGTGTCAGATGCTACACATCATCATGTTCTAAAACATATTTACTTAACGGAAGCAGCT

GCTTTTAGGGAGAAGTGATCCGTTCGCAAGTGGAGAGAAAGTTGTTTACTGTATTTCCTT

TCCAAGCTATCATTACTTAAAAGTAGCGTTGCTGTTTACAGCGGTCATTTCCCTCTTTTT

TTTTCTTTTTCTTGCCTTCTCCTCTGTAGTTGCGGTGTTGTCTAACCGCTACTATTATGT

GGTTGCGGCTCCAGGAGGGGGGAGGAGGGAAGGGGACAATGTTTCGTGCGTCCCTAAGGG

GAACCGACTAATTGTTTCCAAGAATTGTTTGCAAGCGGCCATTTTCAAGCGGCTGACACA

TTTGCCCAACTGGATCACTTGTTAATACATCTGTCTTGTTGTTTTCAGCGTTACTGTCCT

TCCGTTTTTTTTTCTCACTATTTTGCACTCATAGGAACACTGTGTAACAAATCTTATATG

TTGTTGGCGTCTACTTTCTGTACTATATTTAATATATAACTCGGTCACTGCCCGAGTTAT

ATATTAAATATGCTTGATTATGTTAGACACTTTATCGCATCGGTGTCGCAACCACTGGCG

CTGTGGTGACTGTTTTACTCACTCTGAAAATGTTCCATCGCCTTATCCCTCTTGGGGACT

CCAATGACAAATAGATGGAGGATTAGTACGCTCTTCATCAACCAACGTAAACACAAATAC

ATAATTTGAGCGGGTCTTTAAAAAGGTGGAAGTCCTCGAAATCCCGCTCCACATATCGCT

GCTGTCTTTTTTTTTTTACTTTTTTCTTTCCCCCCCCCTCCTCCTTTCCTGAATTCGTGT

GGAAGGTATTACAGGTTCTATAGCCAGTATGTACATGGGCAATGGGAAAGGACGGTAGAT

CTTTAGGTTCAGGCGTAATTTTGATGGACCCATGCCTCTGTGGGCGTGGTTGAACGAGAT

TCTTTTACTTCTAACAGGACATTTTTTTTAATTTCTCATATGAGAATACTCAATGGACTA

TTAAACCATGAGCGAGGGATGATAACATTTATTCACTCCGGGTTGTGTTTGCCTTCCATT

CAGTGGTCGTGCGCTTCTTTTTTTTTTTCTTTTTTTGTTTCCACTCGCCAGTCCCTTCCT

TCCTCTTTAATACAAATAGTTCCTCTCACTTAATGTGGGTTCTCAGTGGTAATGCTTTTT

TGTCACTTTCGCTCATGCACCGCAACAAATGTTACGTGCGTGTGTGGTTGTGTTGTGATG

AAACCTTTGAAATCACTTGAATCAACTGTGTGAAGTTGTCTCTATGCGTATGGCGGGTGA

AGGGGAAAGCAAAAAAAAAGAAGAGTAGGAGCTTGTGTGATGTAAGTGAGTGTGTGAATT

GTTAAACTGGAAACTGTCAACCGAGTATCCGCATCGCAAATATATATATATATAAGGCAA

TATGGAAAAAATCCCGTCGTTTGGGAGAATATTAACTTGGATTCCTCCCTACTCGCCGGC

TGCATGTCGTCCTTACGTCTGATCTCTCCATAAGTCGTCTTCGGTGGCTGTCTTTTGAAT

TATTATTATTATTATTTTGTCGCCTGTGTTTTACCCAGTTTCTACGATGGATGCACGTTA

ACTTGCATTAGGCTCATTTACCTCCGTGCTGTTCATTGAGGCTCCCATCACATGCGCGCG

TGCACGTATATTCTTCGACACTGCTAAATGCATCTGTTGTGGTGGCGTTAGCCGAAAACT

GGTTCGTAAAGCTTTG

>Tb927.11.16300 | Trypanosoma brucei TREU927 | hypothetical protein, conserved | genomic | Tb927_11_v5.1 reverse | (geneCodeEnd+0 to geneEnd+0) | length=221

AGTCGGAAAAAAATAAAATCGCCTGTGTCTGTTCTGACGCGGCAAAAATTATCCTTCCAT

TCCATTCCATTATTTTTTTTTTTTGAGGAAGGCGGTGCAGCAGTCACTCCGCTTGCTTCT

ACTCTCTGCGACAACTCTGTTTTTATCGGTTACTCTTTTCTCTCGGTTCACACTCCCCTA

AATAAATTTTTCATCTGCTTAATCTCAAGCGTTGTGAACCC

>Tb927.10.6200 | Trypanosoma brucei TREU927 | hypothetical protein, conserved | genomic | Tb927_10_v5.1 reverse | (geneCodeEnd+0 to geneEnd+0) | length=313

ACACCGGCAAAAGTTTGTTGCGTATTCCACAGCGAAGGCACAGGTACGCATTTTAGTCTC

CTTTGTTATTTTTTGTTTTTTCTTTTAAAAGAAAAAAGAAAAAAAACCGTTTATCGCTAT

TTCTTTTCATCTGTTTCCATTGCAGACGCTCTCAGTATAATATATATATATATATATATA

TATATATATATATGTAATTTAAAAAAAAAGGAAATGGCATTCCAGCCCTCTCGCAAGAGG

TGCGGACTGGGGATTTGAACTACCGTAGTGTAATAGAAGATATGATTGAGGGTTAGGGGA

AGTGGTAAATTAG

>Tb927.4.3500 | Trypanosoma brucei TREU927 | Amastin surface glycoprotein, putative | genomic | Tb927_04_v5.1 reverse | (geneCodeEnd+0 to geneEnd+0) | length=582

GTAGCCACATATACGGTTGCATTACGGCAGCTCGAACGAACTACATTTATTTATTTATTT

TTTTCTGCACGGCGTCGCGTGGAGATGCGATGAAGTGGTGCTTACTTTCCAGTTTCACCT

TGTGCAACAGCGGACGTGAATATGTTATTTCTTATTTTGGTCAGTTCACACCACTCTGGT

GTTTTCTTTAAATGTTCTATTCCTTCGTATTTATATACTACACTTTTAGCGACTACCTTT

CCCCCTGTCATTAGTGGCTGATTTAAGCAACTTTCGTTTCTTCAAGTTGATCAGCCAAGG

TTGGTTATTACTTTTCCGTATTTAATTTCGTTCGTGTCTATTTCTTTATGTTCAGTTATT

TTTCACTTTATGAAGTGCATTCTATTTTTGCGGCCACTCGTGCCTGACCTGACTGGCGTG

TTGCGATGGTTTTGTGAGAATAAATGTCCGTACGGCCGTTTCTGATTTTTTTTTTTTCGA

CTATTAGGGAGTGCGTGCGGTGGTAGCGTAGAGGGGGAGGGATTTCCTCCCTCCCCCCAC

CCACCCAGTCAAGTGGGTGTGTGTATCGGGTGCGACAAAATT

>Tb927.9.4310 | Trypanosoma brucei TREU927 | tricarboxylate carrier, putative | genomic | Tb927_09_v5.1 forward | (geneCodeEnd+0 to geneEnd+0) | length=1

G

>Tb927.1.1470 | Trypanosoma brucei TREU927 | conserved protein, unknown function | genomic | Tb927_01_v5.1 forward | (geneCodeEnd+0 to geneEnd+0) | length=1

A

>Tb927.7.1320 | Trypanosoma brucei TREU927 | 10 kDa heat shock protein, putative (HSP10) | genomic | Tb927_07_v5.1 forward | (geneCodeEnd+0 to geneEnd+0) | length=253

AAGGGTGGACAGTAACCGCTAACGTGCATCACGTCATTCTCTGGTGGCACTTTGTTCCCC

TCTCGTGTCCTACACGCTCCGCTTCCAGCCTGGTCTGTTTCATTGCTGCGTAAAGGGTGA

GAAGCCGTGATAGCGCGAGGAGTGGGGAATGGAGTGCATGCCACACTTCTATTTCTATTT

ACACTTTTATTTTTTTTATTTTAATATTACATCATCGAATTTGTCGGACACCTTCCTTGA

TTTAGTTTGTGTG

>Tb927.9.6960 | Trypanosoma brucei TREU927 | hypothetical protein, conserved | genomic | Tb927_09_v5.1 forward | (geneCodeEnd+0 to geneEnd+0) | length=1

G

>Tb927.7.6200 | Trypanosoma brucei TREU927 | chaperone protein DNAj, putative | genomic | Tb927_07_v5.1 reverse | (geneCodeEnd+0 to geneEnd+0) | length=890

ATTGAATGCAACGTGAGTCATGATTTATATCGCATGCTGGTGTTATTACTTTTCTCTTGT

TAGTTGGAGTTATTTGGTTATGAACTCTCAGTCTTTATATATATATATATTATACATATG

TGCGCATAATGTGTGCTTGTATGTGAGCGGTGTTGTTGATCTTACCGTTTGTGTTATTGT

CCTCTTTATTTTTTTTTTTTTCGATGTGGTTAGTTATGTAAGCTTCTTACTCGATTTATG

CCACTTTTTACCTCAGAAGAACCAATCATTGGCGAGCAGATGGTCTGTGTGTCAATGTGT

CACCTGGTACAGTTTTTACAATAATAAATGATAATTACTTTTATCATTACCATCATTACA

TCATCTGTTGGTTGCTCCACGTTAACGGGGTTATTTCTCTCCATATCTTTCCTGTACATT

CACGTGTATTCATCGATATGCGCTTAAAGTTGCACTGTTTCCACTTCTCAACCGTTTCTC

TCTTTTTCTTTTTCTACTTTTATTTTGTCTCTTTCTTCTGTTCACCCGCCTGTTTGTGTT

TTTGTGGGTGCTGAAATTCAGATCACGGCGGGGTGCACTGCTGCCGTGCGCCGTCGCTCA

GGTTTGTGATTTCTTTTCTTTTCTTTTGTTGAGTTGGTTTGGGTTTTATTTTACCCTCCC

CGCTCCCACAGAAGGTAGGAACTGACAGGTGAGTGGTGTAATTAAGGTAACGCAGTAGGG

CAAGTGTACTTATATACACGCATACACATACATAAACATAAACATACACATACATATAGG

TACAAGTGTTTGCATATATATTTCTCTGCTCATATATTTCTAGTGATTATTAGGGAAAGA

GAGGGGTGTCAACAAAGGGAAAAAGAAATATAGGAAAGAAACTTAAAAAG

>Tb927.2.4200 | Trypanosoma brucei TREU927 | protein kinase, putative | genomic | Tb927_02_v5.1 reverse | (geneCodeEnd+0 to geneEnd+0) | length=3274

ATTAGTGTGTGTTTATTTCCCTTTAGGTAAAGCTAAACTCTCTGGTGAATGGATGTGAAC

GTTTCGGTATCGGCGTCCTTGAAGGGAACAAAGGAATGGATATCAAATGAGAACATTGTG

TTCGTGTTTCCCGTTTGGTTAAAAAATTAATATATAAGTATGGCTGGTTATTTGCGCATT

TTTATCGTGTGCTTCATGCGGGGGCTTATCAGTGACAGTAAAGAAAAGGAAAGAAAAAAA

CGCACATACAAACACATACATTAGCAGTGCTGACATGAAGCAAATGAAGGGAAAAGAAAA

AGAGAGAAGGAACATTTGAAGCTTCACCGCAACGAGGAGAGGAAGCGGATGAGGATCTGG

TGGAGATAGACGTTTTACAAACAATTGTGTTATTACATACTCCATCAACGCGCTTTTTCC

TTGATGCTTATGTCCTCTCTTTGTTGAAACGGCTCATTATTATTATTATTATTGTTATCA

TTATTATTACTATAGGTAAGTATAAATAAACATAAGTAAATAAATATTTATTTTATTTTC

CAATGAGGAAAGGATACTATTTTGCTGTCCTCCATTATTTTATTTACGTTATTTTGTTTC

TGTTTTGGTATATGAGTTATATACGCTTGCGGCCGTGGTAGAGTAATGAATTTAAACTAA

TATGGATTCTTGTTTTATCGTAATAAATATTATATATATGTTTATATATCATTTATTTAT

TTCTTTCATACATTGATTTTTTCTATCAGTTTCCAGTTTCGAGTCGCTGCATGACCGTTT

TATCTAAAGGTGACAAACAGTAAATTAAAGAAGAAGAAGCAAATCACCCCCGAAGGAAAA

AAAAAGAGGAAAAATGATGATGTTTGAAGTTCACAATAGGAAAGAGTGGAGGAATGGGAA

GAGGAAAAGACCGTGGTTGGCGACGCAGCGATGAGTGAGTGAAACTCTTTTGTTTTGTTT

TCTCAAAGTCAAAATCAAAATACCAAAACATTTTTCTCAACATACTTGAGCAATTATAAT

ATATTTAACTCTACTTGTGTAGGCATTTATTTTTACATGTTTTTCGTACCTTGTCAGTAT

GGGCGTTAATTATTATTACTTTTTTCTTTCCCTTCCCCCTATTTTAAAGAAAATTCCTGA

AGGTTTTCCGTAGCGCATGAAAGTAGTAAGCTGCTGGCGTACGGTTCCGAGTTTTATCAC

TACTTTGCTTCAGTAATACTGAGCCAAAGGTTATTTTTTATAATGAACGGAGCCGTCATT

TTCCACCATCAGTTTTATTGCATTCCCATCCGAACGCGTTTCCTTTTTCTCTCGACTCAA

CTCATTCCTTCACTTATTTTTTTTGTCGGTTCAATTTTCCCGATTTTTGGAGTTTTTTTT

GTTTCTTTGTTTGTGCGAAACCCATTTCTACCGTCTCATTCCGTTTATTTTCTTTTTTAC

TTTTATTATTTTGGTCTGGTTGTTGCCGTATATTTCCGTTTCGCTACATCAAATATGTGG

TGACGTGTTAGCGCCGGCAGGTTTTTTTTGTGTTTCTTGTGAGGTTGCGCGTATTTTCTA

TTTTCGTTTTTCTCAAGAAAGTGAACGGCTCGATGTTTTTTCTTGCGCCGCCACTTTGCA

GCAGTGTCCATTCAATTATTTTTTTTAAAAAAGAGATAGAGAGAGATTTTTTTAAAAAAT

CCCCTTCCTTCCCACAGACATCTCGCTCATTTTTTGATTTATTCTTTATTTTTACGCTAG

TGTATGCAGACATTCCCTTATTTATTCATATTTGTTGGTTTTTGTTGAGTATAAATTAAA

AAAAAGAGGGAAAAGATGGATAAGTAAAAATAGATAAACACAGGAACGTAAATATACATA

TATATTTATTTATTTGTTTATTTTGTATTGAACGCGCGTATTCTCCCCCCTTTTTTATTG

TTTGGTGTATGTGTGTAGGGAGGAAGGTGGTTCAATTACTTCGTATGGGTGAATTTGTTA

CTTTTCTCCCTTTTCCTCACAGTCACCTTTACAGACAAATTCAAACACAAAATTTCTGTT

ATGATGAATGAATGGACGGATAGATGGATAGAAGCAGCTTCTTTTTCTTCGCTTGCTTTT

CCTTTTTCCTTTTTCCTTTTCCCCTTTAACGGTTTCTGCGCTTCTATTTCTATTTCTATT

TTTCCTTTTTATTTTTTTACAACGGCATCACTCTTCGCTTTCATTTTTTGGGTGTTTTGT

TTGTAGAGAAAGATTTTTTTAAAAAAAAACTTTTTTCTTACAACAGGTACGAGTTGAGTA

AGGTGTGCTTAGGGAGGAAGGGGATACATATATATATATATATATATATATATATATATA

TGCGAAGAAGTGTAATTTCTCTTCTTTTTCTTTTTTTTTTTAGTGCTGTCATGTGTTTTC

GTGCTCATTTCTCCATCCTCCTCTTTTTTCTTTTTGATTTGGCCCTGTATTATTATATCT

TTTTGTTTTCTTCTTGATTTCATGCCGTTGTTTTTGTCGTTATTTTCTTCCTTTTCAGGT

TTGTATTCGCGTGCATTGAAGCACTGCCACCAAAACAACTCGTTCACACGTACATATGTA

TGAATATATCAAACCCCAACCATTTCCCTATTCCTTCTTATATATTTCTTTCATTATTTA

TTCCTCTCCTTATTTCAAAATTTTTGTTTTGTTTTGTTTTGTCATATTTTCACTTTGTTT

TATTTCTTCTTAGTGTGCCTCGTACAATTCTTGATTTGTGTTCCTTTTATTTTATATTTC

TTCCATATTTGTTAAGATTCCTTCATGTGTAGATGTTGTGGTACGTTACGAAAATAAATA

ATATATAACCATATAAATTTTGGAGGAAAGAAAAGGAGGTACAAATTTCACAACAGTGAC

AATATAAAATTTAATAAACTCAGGAAAGGAGATGAACTGAAGTGAAGTGAGAGAGGGGGG

AAAGACGAAAAGGGAAGCTCCAGAAGCATCTGTTTGTTATTGAAGTCAAAAAAAGTAATA

ATAATAATTAAAAAAAAACTTTGCATTGTGATAGTTGACGGCACAGAGAAGAGGTAAGAC

AGACGGGCGAGGATCTGATGATTTAATTTTTTTGTGTGTGTTTTCCCTTTTCTCTCTCTC

TCTTTCCAACCGTGTTCTACTCTTTCTCTGTGTGTGTGGATGTGTGGATACGAATACCAC

ATTGGATGTGACAGTTATGCCTAATTCTCTTCATCTTCCGTACGGCTTTCTCTTCCGTTA

GGGAAACCCGCTTCCTCCTTTTCGACATTGTATG

>Tb927.11.4760 | Trypanosoma brucei TREU927 | hypothetical protein | genomic | Tb927_11_v5.1 forward | (geneCodeEnd+0 to geneEnd+0) | length=658

ACGATATGCCCTCACACGCATGCAGAGGGAATAAATCTGCAGGCGTAGCACCGTCCATGG

TGCAGCGACTTGAGTATAAATGAAGTATGAGCGTGTTGTTTTACGTGCGTAACTTTATGT

GAAAAATGTGCACGTGGGGCTGAGGCATGAATGCACGCATGTTTGCCTCGCGTTCACGCA

GTAATGATAGTTTTCGACGTGTGTTGACGACACATTGCCCCTTACCTCCTTTTTTATTTT

TTATTTCACTTGTTAGCGTCATTACATTCGCAAAAACAGATGTGCAAAGTGTTTTTCATG

TAAAATCTGTCTGCCTCCCCTTTACTGCCGTAGATACTGCACGCTGTGCAATCGTGTTAA

CACGTGTATGTTCATTTAATTGCTCCCCTTAGCAGTGAATCTACGAAGTCCTCTTTCGCA

CATTCCACTGATGTGAACCACACCCTCTCGCACATAATTAGTTATTGATTTAATACTGCA

TATAAATCTATTTTATATATATTTTAAATATTTACTGATGCTTTCTACCAGCCTTGAGGC

GCATGCAGTCGCCCAAAAATAAGTGGACATCGCAGTAGATTTATTGTGGAGTGATATCAA

TAAGCTTGCGCGTGTGTATATGAGACCCGTTTCCTCTCTTCTTTTTAGTGGTGCTCCT

>Tb927.11.5940 | Trypanosoma brucei TREU927 | receptor-type adenylate cyclase GRESAG 4, putative | genomic | Tb927_11_v5.1 reverse | (geneCodeEnd+0 to geneEnd+0) | length=1308

AGAGTTGCGTAGCTATTTTCCCCCTTACCAACCCACACCTTATGCGAGAATCGTTTTTGT

TTTTTTTTAAAGTTATTGTTTTGTGTTTTAAATCAGATGCACTTCCTTCTACTACCTCGC

ACTAAGCGACTTATTTCTTAGCGTTATTTAACCAGCAACGGCTTACTAGGGTGCCTTGCC

GGTGCCTTTGCGCCCGACAAATATATATATATATATATATTTTTAAATCTCATCCCCCAC

ACCCTTGCAGGCAGACGTGGTGCTGATACAGAATCACTCATCTGGATGTTAATGTTGATG

TTCGCCACTGTATCCCTACTCTTTATTCGTTTACATTCCATTCCCTTCCATGTGTTTATG

GTGTGTCTTTGGCATACACTTAATAACCTTACTGTTTAATTATTATTATTATTTTGATAG

CGAATGCTGGCGCCAACCCCTGTTGTAGGTGCTGGCTAAATGCCCTCTTTCCATGTCACA

TACGGTAACGTGACAGGTGCTTCCGTGACGAAGTTATTTTTTTAAATTTTGTAATTTAAC

CCCTCTTCCATTAACACGCTCCCTTTTTTTTTCGTTTCCATACGTTTCGTCTCCTTCACA

TGGGACTGCAGTATGGTGCCTGGCGCTATTTGCGCGCCCTCTCCTTCTCTTTTTAACATT

GACAGCGGATTCATACAGTGGCAATGTGAGAACTATGCTTCCGCCACTTATGAGATTGTA

ATCTTAGCGTGTGCCTCCGCAGGGGTCGTTATATATGTGGTTCCCCCGCAGGCCTCTTAA

CTTTTAAAACATTCCAGCCACCAACAGATCGCGTATACCGCTTTCATTGTTTCTGCCTCC

ATTATGTTGTTATTGTTTTGATCCCTTTCTGCTATATTCTTCCACACACACACACACACA

CACACTGAACTGCATATTGTTTGAAGGAAGAAGTCATGCCGGCGCGGTGCCCCTTAACCG

CTCCCGCTTAGCGGTTGTGCAAACATGATGAGGAGTTGGTGGTTTTTGTTCCATTGAGGG

GGCGTTGGGGTCGTGGTGAGAAGAGGAGGGAAGTCGGGAGAAGTTTGCGCATCTTCTCCA

AAATGATTGTTTCTGTACGCGTGTATTTTCGGACTCTCTAAAGGCGGCGGAGAATAGAAT

GGGGGTTATTGTTACAGTGGAAGGTAACAAGCCCCCTTCTTTTCCCCCTTCTCTCTTTCT

TCATGAGATATCCAAACATGAAGAAGATACTTAGAGGGGATCATTCAAAGGCACCAGGTA

GTGTTCGTTATCGAGTACGATGTTTCAATCGCTTTAATTGTTCCTTTT

>Tb927.7.2660 | Trypanosoma brucei TREU927 | zinc finger protein family member, putative (ZC3H20) | genomic | Tb927_07_v5.1 reverse | (geneCodeEnd+0 to geneEnd+0) | length=5151

ATTTGTATTTGTATGTGTATGTGTACGCGCACACGCAAGCAGTAGGGAGCTTGTATTATG

ATTTAGCGTGCGGAGCTACATCGCAGAGGAAAACAAGGGAAAATGTGAAGATAATTACTG

GAACTGTGCGATAAACAGTAGCGTAACAGAGTTTAACTAACGCGGAGAAGGGTGAAATAA

CCCTTTTTTTTAAAAAAAAGAAAGGGAGCGAAGAAGAAAGGAGGAAAAGAGAAGTAAGGT

GAGGAAACGAAGTGAAACAGAATGTAACGGATCAAGTGGAAAATTCGATACATGTGCTGA

AGGAAAAGGAGTAACACAGACTGCGTTCTGAGCAAATAAAAAAAGGAAAGAAAAAGGGAG

GGACAGAACAGTGAAAGAAAGAAAATGCACCTTTGGCGCCCTAAGCGTGACAGGAAAAAA

TTAAAGGAAAATGGACTGAAATCTATTTTATTGTTCGTGGTAATGAAGGAAGGCGAGATA

AGAAAAAAAAAAGAAAAAAAGCAAGAAGCAGTGTAACCGTTTGTGAGGCGGATTTTTTTT

CTTTTAGCTTACATGTTCATATTACCTTACGTCATTAATTATAACATTTTTCTTTGAAAT

GAAAAAGGGATAGGGGCGGCACAGAGCCAAAAATAAATAATAATAATAATGATAATAACA

GTGATAAGACAAGAGACAAAACAAAATAATAAAAGGGAAACAAATAAATGAGAGGGTGAG

AGAAAAGGAAAGGGTGGTGAGAGGTTATAAAATGGGATTTATCAAGAGGAGACAATTGGG

AGAAGGAAAGAAGATGAAGAGGGGAGGGACTGAAGGTTTTTTTTTCTCCTCTCTCTCTTT

TCTTTTCTTTTCTTTTCTTCTCTTCTCTTTCTCTCATTATTATTATTGAAAATATTTTTT

TTCTTTTTTTATTTTCTATTTTTGTTATAATGCACTTCCCCCCATCCCCAAAATGAAAGT

TATTTTATTTTTACTTTTGCTTTTTTTTTTGTTTAGTTTTATTTATTTCCACCTCTCGTA

TATATTTTTTTGTGTTTCGTTTTTATGATCACCTTCCCAATCAGAATGCTCTCTCCTCTT

GTTTACCCTTTTGTTTTGTTTTTTGTTTGCTTGGTGTTATTTTCATTTGGAGTCATCTCT

TATTTTATTTTACTGCTTTGCATCATCTTCCTTCCGGTAAATGTCTTAGAGCTTTGAAAA

GGATCAGGGAAGACCAAAAAAAAAAAGTGATAAAAATACAAAAAAAAAGAAAGGAGGGGG

AAAAAACAAAACGAAACAAAAACAAAAACACAACATACTGTCATTGCGGTGATAGTTTTA

TTTTAAGCTGTTTTTTAAGGGGGGGGGGAAAAATAAAATAAAAAGAGGTGCATTTTCGCC

GTTTTTGCACCCCCCTTTTCCTTTTCCCTTTTCTCCTTTCCTTCACGCTTCTCCTCTTAC

CTTCCTTTTATTTTTCTTTTTACCTTTTTTCCTTTTGCTTCTTCGTTTTTGTTTTTTATT

TGTGCTTTCGTTCTCCCCTCAACTTCACTTAAATTTATTGTTATTATCATTATTTTTGTT

TTTATTGTTTTATTTACTTGCTAACTCACTCTCTTAGTTTTGCTTTTTATTTTTATTTGT

GTTATGGTTAATGTACATCTGGTATGGATAAATTTATATGAAGGTGCGTTTTTTAGTTTT

TAGTTTATTTTTATTTTAGTTGATGTTTTCGTTGCGGTAAAGGTGAAGGTAAGCGGTGTT

TGTCAAGAAAAAAAAAGAAAAACGGGAACAAGAAGAACTTTTGAAGTATTTCGGAATTGT

TATTCATTTTTTTATCTTTATTTTATGTGTGTGTGTGTGTTTTTTTTTTTACCGGTTATT

TTATTATTTTTGTTGTTATTGTTATTGTTATTATTATCTTTATTTTTGTTACGATTGTAA

TTGTTACATTATTCTATATCCCAATGTGTGCTTTCACCCCCTCTTTTTCGTTTTTTTTTC

TACGATGGGGACTTTATTTATATATTTTGTAGCATTCTCGATTTATCACTTGAAATATTT

AATTCGTTGTATGAATTGTTATCATTTTTTTCATTATTATGAGTTGTTTTTTTTCTTTTT

GGAGGTTATTATTATCATTATTATTGTTATTATTGTCAGTAGTTTGTTACATGAGCGACG

GAAGGGAGAGTATGAACAAAAAGGACTGGAAAAGGAATGAGATAATTGAAAAAAAAAAGA

ATGAATAAGAAAACGGAAGGGAAAGTTAACTGTCATGAATATGAGTATGAATGTGGTGAT

TTTATTACCATTATTGTTATTATGAATTGTTTTAATTTATATTTCAGTTGTTATTTTTAT

TATCTTTATTATCTTTATTATTTTAATCATTTTAACTATTTTAACTATTTTTGTTATTTC

TCCCTTTAACAAGAAAAAAACAACAACCTCCGAACACCTTCTCATCTAATTACTTTATTG

ATGCTATTTTTCTTTTTCCCTCTTCTTTGTTTTGTTTACCTTCCGTGTGTTTATAAATGT

ATTCCTTCTTGAAAAGAGGGAGGGACGGGAGGAGGAGGGGGAAAAAAATGTCTTTTTTGT

TTTCCCCCTTCCTTTCCCCCTTTCCCCCTTTCCCCCTTTTCATTCTCTTTCTTTCTTTTT

TTTTTTATATTCCTTACGGCGTTAGACACGCAACGCCTTTTTATTTTTGCTTACCTATTT

ATGTTAGTATTATTGTTATGGGTACCAATCATCATCGCCACACACGAATGTATGAATGTG

TTTATATATATATATATATTATATTATATTATATTTCCATGAATACGTATGCGTATACGT

GTGTACGTGTATGGGAGTAAATGCAGGGAAAAAAGTAAAGGAAACAAAAACAAGAAGAGA

GAAATAAACACAAAAGGGGAAATACATGCAATATCACTTATTCTTGTTGTTATTTTTGTT

TATGCGGTTTTTGAGGAATTTTGCTTTTGTTTTATTATGTTTTATTATGTTTTGTTTTGT

TGCGTATTTTTGTGTGTGTTTGTGTGTGGTTTCCCCTTCTCCTTCTTTTGTTTCGTTTAA

TTTGATATGTTCCTACCACTTTTGTTTGTGCTGACTAAATATATTTTTATGCTGATGCAA

AGGGAAGACGAAAATAAGTAAATAAATAAATTTAATTAATGATATATCTTTTGCGCCACC

GTGGAGGGGAAAGAAAATAGGAATAAAAATAAAAATAAAGGGTTGAATCTGAAGGAACCA

AAGGAAAAGAAAAAAAACTTAAAGGAGATGAAAACAAAGGCGCACCGGAAGGAAAAAGGA

AAATAAATTGAGTGGACGGCACTAATGCAAATGCAAGGCAACGAAAGGTATTTAAAAAAA

AATATGTAATGATATCGTTTTTTTTTTTTAAAATGCTGAGGCAGAGGATGGATGACATAT

TTTTGAAGGAAATGTAAAACAGTTGAAGCAGCCCGTTCTCTACGGCTATCTTCCTTTTCC

CCCTTTTTTTATTGGTTGTTTTCATAACTTCCGTTTCTCAGCCGCTACAGCGCTTTTACC

ACCCCCACCACACTCCATTAACACTTTTGCTGATTTGATTATCATCCCGTAATGTGTGGT

TGAGGGCCCCCTGTTTTTTTTCCCCCTTCTTTAGCCGAAATTGTTACAGAACGTTAAGGT

TGTGCTGAGGAAGGAATAACAAGGGGGAAAACGAAAAGAAAAAAAAAATGAAAAAAACAG

CTAATAAATAAATGAAAGGGCGCAAAAAGGGATAACAAACGATGCAGAACACCTCAGAGT

TGAATAAAGTACAATGAGGAAATATATATATATATATATATATATAATAAAAGATAAAAG

ATAAAAGATAAAAGATGAATGCGAGCAACAACAATAACATATGCGCATACGCGTGGAATT

AGGAGAAAATATTGTTTTGTTTACTAAGGAAGAGATGCGAGAGAAGGGAAAAAGGAGAAA

AGATCGTCTGTCACTACGTATGGCTTTCTGTTGTGGAAGTGGTAAGGTAAATAATAAAAT

TGTGTATGTATGAGTATAAATGTGCTTGTGCTTGTGCTTGTGTGTGTGTGTGTGAAAGAA

ATGAAAGAATAAATAAATAAATAAATGAATATATATATATATATATATTCACACGCAAAC

GCACGTGTGCACTAACAATGATGATAAGAGGAGTACAAGGAGTTCTTAAGGAAGAGGGGA

GGGGGGAAATTGTGGGAAAAGAGAATGAGAGGACGCGACTATAGTAATGCATTTTCCTCC

CTCTGCAGTCCCTCATTTCCCCCCTTTCTTTTCTTTCATTTGCTTCCTTTGAAACTTCTA

AGGGGGAGGGGAAAAAATGAAATGAAAACCTTGGTTCCCTTCGCCCCTTCACTCCTTCAC

CCCCTCATCCCTTCACCCCTTCACTCCTTCACACACACACACACATATATATACTCGGAC

ATACGGTAGTAAAATGACTGAGGAAAAAGAAAACGAAAGGAGAGAGAAAATAAGAAAAAG

AGAAAAAAAAACATGCTAGGAATAAGGATTTTTTTTTTAACTGACACTTTTTTGTCGTTT

TATCCCTCCACCCCTGTCCACCGCCGTTGTTGTTAATGCCGTCATAAAAAAACTACCATG

TCTCTTCTTTTCCCTGCTTATCACGCTTCAATCAAGGAGGATGGGAATGAGCAATTGATG

CACGTTAATAATGAGGGAAGTGGCGATGACTCAATGGAAGAATGATTTATGTGAATGAAT

AAAGTAAAGGTGAAGTTGAGAGGGCATAGGAGAGAAAAAAAAAAGGGTGACAACGAGAAA

CGCACTCATACACGCGGTGGACGGCGGTAATAATGACAACAGAGAGGAAAACAAAGAACC

ACATGGGAGAAAAAATGTAAAGAGGAATTACGAGTAGTGCCGAATGAAAAAGAAAAAGGT

TCCGTTTTCTCTCTCTCTCTTCACTTTTCTTTCTTTTTTCTTTTTTCTTACTTTGCCTTT

TACTTTACCTTTCTTTTTTGTTTTGTTGTTTTGATTTCATTTCATATTGACAATTTCAGA

TGACACTGCACTTCTTGTGTTTTCCTTTCCTTTATACCCCTGGAAAAAGCACAAAACGAA

TTGAGCGTGATAAAGTCGAAGAGGAAAAATGTGTGTAAGTGATCCATGTTG

>Tb927.10.7290 | Trypanosoma brucei TREU927 | Phytochelatin synthase, putative | genomic | Tb927_10_v5.1 reverse | (geneCodeEnd+0 to geneEnd+0) | length=1305

ATGAGTAGAGTTTTGTGTTGACAAGAGTGTGATTTGTCGAGTAATGTTTTGTATGAATGA

AAAATACATGCCCAGAAATGTTCGCTGCGAGGAATTTCAGATGTGGTTTACCCGTGCGTG

AATGTGACTGGGGGCGTAGGTAACGTAGAAAGGGGCACCCAGGGGTAAGAGGATATCTTC

TTTCCTCCTTTTCGTTTTTTTTTGTACTCTTCTCTGTAATCCCATTTCACTATTCCAGTA

CCACTCCCTATTTAATTTTTCTTTTTCTTTTTTCTTTTCTTTTTTACTCATCCTTCAACT

TTTCCAGTGTTGTTATTTTTTTTTCATGTTTTAATATATATATGTCTTATACCAGCACTC

GTGTGCACAGTACATCATCTGAGTGAAAAGGGATTATGACAGCAGCGGTGGCAGTACCCA

CGCTTGCTGTGGAGGGAATTGAGCGGGAGAAAAGAAACCGAGGGGAAGTAGCTCCATCAG

TTTGAATTAAATGCATAGTGAGCGTCGTGTGCGTAAAGTTTCCTGTCTTTCAATATATAA

AGAAGTGCAAGTAAATATATGTTTGTTTATTTGTTTACTTATTTTGATTTCGAGACTTTT

AATTCAGTACTACGTGTATGTTTTTCTCTACATAGGTTTGTCGGTGGAGGAGCGGTAATT

TATGTTTTTTTTTGTTTTACACTTTAGTGGCTTGGACGCTCGGTTTGATTTCACAAGCGT

TAGAACTTGTGCAGATGCAAAAAAAAAAGGGGGACTTGTTAAACAGTGTGCGTGTGTGTG

GTTGTGTGGATACAATTGATCAACAACGGAAAAGAAGAAAAGGATGGATAATGTTTTTTT

TTTCCTGCCACTTCTTCATTTAGTGCTAATAAAGGTAGCAACTCACTCCATTGCGCCTAC

ATTGTACTGCGATGGGACGAAGTGACTAGAGTTGTTGCGTGCGTTTGCGGTTATTTATAA

TTTCCTTCTCTTCAAGGTGTTTTGACAACGTTAAGCGATGATAGACTCCCTTCTCATCTT

TTCCCCCCTTTTTTTACCTCATCGCTGGTTTATTGCGTGTGTTTCTTTCCGGACGTGACC

CACTTCCCTTCACACCTGATCGTTAACTTCATTTTATTTTTTTGAAAGTTTCTTGTTCGT

ACCGCTGTTTTTTTTTTCCCCATTGTCTCTTCTTTTGGCTCTATCTTAAATTCAACCGCC

CTCTGTTAAGCCTGGGATTTCCAATTTCCCTTTACGCAGTGTCCCTCTTCATTGGGAGGC

CATGCTTTTACTTCCCTACGAGTATTTTTGGTTCATACCTTACAT

>Tb927.4.4990 | Trypanosoma brucei TREU927 | ubiquinol-cytochrome c reductase, putative | genomic | Tb927_04_v5.1 reverse | (geneCodeEnd+0 to geneEnd+0) | length=16

ACGTCAACGGGACTTT

>Tb927.11.10140 | Trypanosoma brucei TREU927 | hypothetical protein, conserved | genomic | Tb927_11_v5.1 forward | (geneCodeEnd+0 to geneEnd+0) | length=453

GGGCAGGCCCAATAGGTTAGACTGTCTAGACGTCGCTGCCTGTGTTGGGCGGGTGCTGCG

TTTCTGCTGATGGTCAGTTTGTGAGAGGGGAAACCATATGTTGGGTTTATTAGCAAAGCG

GTGTATGCGGACTTTGCAGGACGTTAGTACTATCATTTACAACATTACTCTAGCTGTAGT

TTTTAACTTTCTCTCACACGTTGTTAGTCTATTACCGTTATTATTATTTTTTTTGTGGTG

GTGTTAGTTTATATTTGTTCTTAACGAGTGCTGTTTAACTGTTTTTTTTTTTAAAACCAA

CGAAGTGGTGTGTTTGTTGCGCACATTTGTGTGTATATATATATATATGTTTGTTCACTG

TTAGCTCTGCGGGGCAGCCAAACGGAGGGTGCGGTATAGTTACTTGCCTTTCTTTGTTCC

TTGTATGGGTTGACGTTGACCCGGAGCTATTAC

>Tb927.7.1700 | Trypanosoma brucei TREU927 | hypothetical protein, conserved | genomic | Tb927_07_v5.1 forward | (geneCodeEnd+0 to geneEnd+0) | length=800

AATTTTATTATTATTATTGTTGTTGTTGCTGTTGTTGTTTTAGTATATTCACTCGCACGC

TGCTATTTTTTATTACTTATTCCATCTCTGGCTTTTTCCAAGTGTACATGTGTTTTTCCA

TTGTAATGGCACGTCGCGTGTTACTCACATATGCTCTGAAAACCAATTTAAAGTTGAGTT

TCTTTTATTTTTTATTTTTTAAATTGTTCTGCCTACTAGCGTCATTCCCTCCATACATGT

GTGTGTATATGTATTTCGTTTTTCCCACGTTGTTTCCCACTCTTGCGTTTGAGAGAACCA

CTGGTAACTTCAAATATGTAACGGATTACGTTTCCCCCCTCCAAAAAAAAAAAATAATAA

TAATAATAATCGCGTACGTTAGTTCCTGCAATTGTTTACATGTGTTTACTTAATGAAGTA

CCACTGTACATGCGTGGGATTCTCTATATACTTTCCTGCGCGTGTGCGCAAATTTATACT

GACGAACTCGATAACGTTTTCTCTTAGAAGTTTAAGTGAGTGTGAGTGTGTGTGTGTGTG

TGTGTGTCTGCAAGGATAAAAGAAGGTGTAGGGTGGAAACAAAAAGGAACAGCTAAAAAA

AGAAAAGGTGAATAATGCATAGAGTGAGACGAGGGGTGTCGGAGGAATCGCTAAGCTGGG

TCCCTGCCACACGTTTCCTATATAACATTTATTTCACTGATACCGACTTTCCCCCTTACT

CAGACTACATATGTCTGTTTTTTGTGTGCGTTTTCTATTTATTTATTTCCCCCTCTCTTT

CCATTACAGCTTTACGGGTG

>Tb927.7.6340 | Trypanosoma brucei TREU927 | hypothetical protein, conserved | genomic | Tb927_07_v5.1 reverse | (geneCodeEnd+0 to geneEnd+0) | length=1860

AGTTAAGCAAGGGTTTGGATAAGGGGATAACGCGCGGTTACGTGAATTCATAAAGTCACA

AAGTAATTGGATTGTAAGGGCCATTGGGAGTGATTTTAAAAAAAAAAAAGGAAAAAGAGA

AGAAGGCAAAGGCCCCATCAAATAGTGAAGGAAAGGAGGAACTAAAGTATGCGTACGTGT

ACATGTCACTATTCCTAATGGCTTTTGGACCAGAATGTGTTTTTTCGTCAAGAGCAGAAG

TGAAACGATTCCCACCGGCACAACACGACGAGGAAAAGGATATGAAACCGTGCCAACGAA

GTGAAATGATCATGAGGATTTGTGATTTGTACGATTGTTTACGTGTTTGTGTTTGTGTAT

GTGTGAACAACAAGTCGAGAGGAACGCAGAACTAAAGAGTTTCAAAAAAAAAAAAAGGGG

GTGTCTGTGGTGTAACAAACAAACAAAGTGAAATGGAAGCTAAAATAAAGCAAAAGTTAA

AATGAGGTAAAAAAGAGGGAAACATATGTGCGGATGATGCGGATATGTTAGGAGCGCAAC

AATAGCAAGAACAGTATGAAAAAAATATATATATTTATTTCATAGAGAGAGAGAAAGAGA

GGAAACGAAGGGAAGTGTACGAGTGAAGGGATATAGCGCTTCTGGGAAGGTGAATTTGAG

GAACTAAGTAGCGAGGGAGATAAAAAAAAATGGTGATGGAAGTGAAAAAAAAAATGAGCG

GAGATGCGTGAAGTGTTGCGACCTGACGTGTGGTGATGAGTTGCCACTAACAACAATAAA

TAATAAATAATAAATAGTAATAATAATAATAATTATTATTATTGTTATTTTGATTTTCCA

ACTCTCACACATGTGGCGTCACTCCACCAGCGTTGCGTCTCCACGGCGATGAATTTAATG

CCGTCATGCACGGCAGGTTCTGCCTCTTTTCCTTCTTCGATCCATTTTTTTTTCCCTTTC

CGTTCCTTCCTTCTTTCTTTCCTTCCGTTTGTTTGTTTTGTTTGTTTGTTTTTCCTGAAA

AAAACTAAAGTTACGGGGCTAAATAAACCATGGATGAACGAGAAAAGAACGAAAAGGTAA

AACACACCATTATCGTCGAAACGAGGGGGGAAAAAAAAATAGGAGAAAAAGTGTTTATTT

TTATTTAAGAAAAAAAAATAATTGGTTGTCCATTTGTTGTTTTGTTTCGTTTCTGTTTTC

AGTTCCTTAGAAACGTCTTGTATTTTACTTTACCAGCGTGGAAAAAGTGTTAGCTTCGGT

TTGTTATTGTTATTTTTATAGTGATAATAATAAAGGAAAAGAAAATGAGAAGAAAAGTGT

GAATAAGTGACGTTGCATGATCAAATATATGTTGATTTACAGCACCGTATGCATTTTTTT

GATTTTTATTTATTTTTCTTGGGGCTTCTTTCCTTTTTTTGTCTTTCTGCGTTGTTTTTC

TCTTTCCTTTTTTTCTTCTTTTCAGTTTTGTCTCACGTGTCGTGTTTTAGTGCTCTTAAT

TTGTTCCTCTTCCGTTTTTTTTTCAATTATGAAATAAATCCCCAACAGAAGGGGTTTAAA

GGAGTTTTTATTTTTTTATTTTTGAGAAATGCTGCTGAGAAGCAGCGCCACCTCGTGATT

CCTGAAGTGAGTGAACTGCTTTATGTTGTAGTAAAACTTGTTTTATTGTGGTCGTTTTTT

TTTTTACGTGTGCTTCTTTTTTTTTCTTTTTTTTCTTTTTTTTGTTTTTTGTTCTCCCCT

CTCTCTGCATTTGTTTGGAAAAAAAGAAAAATAAAAAAGAGAGAAAAAAGGTAGAAGATA

TGTGTTTGCGGTTGTTGCTGATATTTTGCATATTCGAAGCGTAAGTACGTTAAAAAACAA

>Tb927.3.940 | Trypanosoma brucei TREU927 | Complex 1 protein (LYR family), putative | genomic | Tb927_03_v5.1 forward | (geneCodeEnd+0 to geneEnd+0) | length=137

GCGGAGGGAGCACGTGGTGGAGAAAAGAGAAGCGGGTGCTTCACGTGGGGAAAACTCAGA

CACTCATCAGGAAACCAGAGAGCGGTCACTGTTGGGTGAAGGGTTGGCGGTATCATCGGG

AAAATGACTTTTCCGTT

>Tb927.9.11820 | Trypanosoma brucei TREU927 | CRAL/TRIO domain containing protein, putative | genomic | Tb927_09_v5.1 forward | (geneCodeEnd+0 to geneEnd+0) | length=663

ATACCACCACGGACCTTTTTCAATGTTTTAAGCATTTTATTAAAGTTTCTTGTTTTTATC

GTGAACTAGCACACACATATATATGTACATATTGTATTCCTTTGGCTTCGATATTGTTGT

CACAATCATCATCATCATCATCATCACTATAATTATAATTATCGTTATTATCACTGTTAT

TTCTACCCTCTCCTCACGTTACCATTTCATGGTTTTAGATCTTCTCTACCACTATTTTGA

ATCGTGCGGCCGTCTTTTACCCGTGCGCATAAATAAATGGACGACCATAAATATATAGAT

TTGTGTTGTGCCATCTTTGTTTGTTTTTGTGTGTGCCTCCGTACAATTGAACCGTAGCCA

CCTGCGCCATTATTTGTTTAGCCTTGCCGCAAACAGGATCGGCTGTAAGTTCGTTTTGTC

TACTCTATGTTCGCATACGCGCTTTTCTTTGTTTTATCGTTTTCATTAATTATTATTATT

TTTTTTTTCGGTGCGTTTTATTTTATTTAGTCTTTCGGATATTACTTTTAATCGGTCGAG

GGATTGGCGGGAAAATATGTATGCTATACATATATATTTTCGTTCGCGGTGGGTGATAAG

TGTGCCAGTTGGATAAAGCATGTACGTGCGTATTAGAAACCCGCTATGATGCGGTGGCAC

CAC

>Tb927.11.16200 | Trypanosoma brucei TREU927 | cytoskeleton-associated protein 17, corset-associated protein 17 (CAP17) | genomic | Tb927_11_v5.1 reverse | (geneCodeEnd+0 to geneEnd+0) | length=758

ATGTAATCTTTTAAAATGCTGAAGGGAAAAAGAAAGGGGGAAAGTAAGAGGGAGTTATGA

AACGAAGTGAGGGGATGAATGTAGAAAATGTTAGTGAGAGTGACCCCTTGGAAATTGTGC

GAGAGACGATACAAGCGTTTTCTCTTTTTTTCTTTTTCTGTTAGCGCCATGATCCCTTTT

TTTTCCTTTTTTTTTTGAAATGTACCTTAAATCGAGCGTCTCGGATTTGTTTATTTATTT

ATTTATTTATTTTCCACTTGTATATCACTCACTTTTGCGTGATATATTATTACTATTATT

ATTTGTAAAGGGAGAAGGAAATGAAAAGGAATATGGGACGACCTAATGAACAACAGAGGG

ACTTTCCAAGTGGGAAGAAAAAAGTGAATTGCGTTTCAACATTTACTCGTTTATGTCCGC

TATGTCTCCTTCACTTTACTTTACATCGACTTTCATGTTCCCAATGAAGGAGGATAAAAA

GGTGTAGTGAGTCCCATAATCGAATGAAACACAAAGCGGTAATGATGATAAATGTGTGCT

GCATTTCAACATGAGCCCCTCCCTTTCCTATCCTTCTCTTCATGTATTTATATTTTGTCA

AGGGACATTCCTTAAACTATTGTTGATGTCATTATTATTATTATTATTATTATTATTATT

ATTTTCGAATGTAAACCATCATAAAAAGGGGAAAAATGAATAACATTTTGGGGGGAATGA

AACTTCAAGAAAACGCGTCTCCTCCCATTCGTCATGCG

>Tb927.7.6300 | Trypanosoma brucei TREU927 | WD domain, G-beta repeat, putative | genomic | Tb927_07_v5.1 reverse | (geneCodeEnd+0 to geneEnd+0) | length=2492

GCGTGCCCCTTCCTGCCGGTGTTTCCAGTGAGCATATAAATATTATATATGTGTCTGTCT

GTTTGTTTGTTTGTTTTATTATTTGTTTATTTAACCCCATTGCCTCGTTTTTGTTCTTTT

TTTTTTACTTTTTGTTGCCTTCCCGGACAGCAGCTTCTGTTTGTGACCTTGGCTGATGAT

TCTTTAGATAACAAAACCGGCTTTGTGAAGGAGCGGTAATTGAAGGTCGAACGGAAAAAT

TTTCTTTTGGATTGATTTGCAGTATTTTTCCTTTTTATTTTCTTGGTGTTTATATGCCTA

TTTACAATATATTTATCTGTTTGTGTTAATATGTATATATTTGATTTCCCATGTTCCATC

TGTCCTCATACGCCAATATAGATGTACCGATGAGTACGGCGTAGTGGTAACGAAGCATGT

GCCGCAGCCAGCATAACTCGTTTGGTTCCTTTTCACCTACGATAGTTTGCTGCTGTTATG

TCATTTTTAATATATATATATTTCCCCCTCTTCTCGTCTGTTTGTTTAACAGCAGCATGG

AAACCGCCGTACTTTTCGCTCTCAGAGCATTTTGTTCGTGCGTTTCTATTTGTTTAGGTT

GAGCGTTTTCCTTTTTCAAAAAGAATTTGATTTTGTCATTCCTCTTGTGTGTACACTCTC

TCTTTTTTTTTTTTTAAATTTAAATTTTATTTTATATTGCTGCACTCATGTGCTTTACGC

GCGCTTGTGGCATCTGACGACTTCACTTCAATCACGGAGGCGATTAGTTAGCCGTTACTC

GTTCTTATATTATTTTTACCTCTTATTATTATTATTATTATTTTTGTTTGTGCGTCCAGC

ATTATTTAGTACGGCTTCTTTCCCTGGGATGGAACTCACTGCCGTTTCCAGCACTTCTTT

TACTTCTTTTCTTTTTTACTTTTTGTTTTTTTTGTTTTTTCCCCTTATCTTCTTTAATGT

GTGATAATACCCGTCCTGAGGCGGTATGTGATGTTGAATTTTTATTATATCATATAATAT

AATATGTTTTATATGATAGGTGTTATATAAACGAGTATTTTTTTTTTTTTATGTTTGTAC

TAATATTTATTTGATTATTTTTTTTTGTGCAGATGGACAATGTCAAAGAATGTGGATGTA

GCATTTTTGTTTCTTTTTTTATTAATTTTGCGCGCTCGCTTTATTTTTATTATAACCATG

TTTTTTTTTTTTTTTTGTAAAAAAAAATATTTTGTTCTCTACCGTTTCCCAAGTTGTATT

TTTCTATTTTCGTGAATTTATTTCTTTCGGTCCCTTTGTCTACTGCCATTGCAACACGAA

AAAAAAATCTAAATAAATAAATAAATATATATATTTATTTATATTATTTGTGTTCATTTG

CATGACAACTACGAGGGTTATGGAAGATAAAACAAAACAAAACAAAACAAAACAAAACAG

GAAAGGGGAATGTAAAATTTGATATTGGAAATGTGTGAGGGATGTGACGGCTGGACACTG

CAAAGCAAAATTTCAGTACCGTCTCCATTCACATGCGAAACTGAGAGTATGAAGGGTGAG

GATGAGGGTAAATTAGTTTTAATTATTTTTCTTTTTGTGTTGATGTTGCCATTGATCCGC

TAATTATTTTAATTTATTTGTAGTTAATAATAATAATAATTATTATTATTTTACTTTAAA

ATTAATTACCTACCTTTGGAATGATTTAGTTTTCTTTTTTGTCGCTGTGTTTTGTTTTGT

TTTATGTATTTGACAGCGGGTGTGTTGTGGCGTAAACGCTCGTGTTGTGACTATCTTTAA

CCTTCCCTTCTCTTTCTCTACTTCCGTATTTTCTCGTCACTTGTGTTTTTTACACCTGCC

ATTTCCCTTGTCTTTCTTTTCTTTTTTCTTTGTTTTTTTGTGTTTTTTTTTTCACAGTTG

GAGCAAGTGCCTTTGATTGTTATTTTTTTTATTTTTGTTTTTCCTTTACTGAAGAGGAAG

TGCAAGTATATATAAATGAATATATATTTAATTAATTTAATTAATGTATATATTTGCATG

TGAAACCCCTCTTCCCTAACCCCCCCTCGACAAATGAATGAATAAATAAATGATTTCCCC

CCCTTATTCCCGATATCGGGTATATCATTTGACCTGAGGAGCCGCAAAAGAAACGCGCTG

AATGCGTTGCGGTTTATTTATTTCCCTCTCTCCCACCTAATGAATTTTTTTTTTCTCCAT

CCATCCTCCCTTTTATCCCTTTTTAGTTGCCCCATTTCTCAGATTTGCCAATGCATTTTT

TTTTTGGGGGACATTATGTTGTGCCGTCTTTTTACGGGTAGTTTCTTAGCCTCAAAATTA

ATTCTGCCTTCTTTGCCCCTACTTACCTATCAACTTTCCATTTATTTATTTATTTACTTT

TTTTTTTTAACACCTCTGATCCATTTAGTGGGGAAGTGGCAGACGCATCGACAAGGAGGA

GAGGGAGCGCATTAGGGGAGAAAGAAAGAAAG

>Tb927.8.4700 | Trypanosoma brucei TREU927 | amino acid transporter, putative (AATP6) | genomic | Tb927_08_v5.1 reverse | (geneCodeEnd+0 to geneEnd+0) | length=3034

ATCAACATTGGCCATTTTTTAATTTTTTTGATAGGCTATATATTTACATTGTTCTTTTCG

TTTCGTTAGATTTTTTTATTTTGTAGTTGTGATTAGTGTGTTTTTTTTTCTTCACAAACT

TGAGCTGTGTTGAGATTTTTTTTTTGCTGAATGGTAGGTAGTGTCTTGATTTAAGTGGTA

TCCTTCTCGTCTCTTTTTTATGTGTTGTAGGTAGGGGTGTTATTTTTATGTGTAATGCCT

CGTGTTTTTGTTGTATTTTGTTTTTTAACTCTTTTTTGTTTTGTGTCTGCCTTTTTGTTG

TCGGAATAAGTAGCTTGTCGGTTTATATTGTGCAGGTTTTAGTTATTTGTGGGCGTCGTT

TGTGGGACACGTAACAGACTGTGAGGAAGATATATTTGAGTGTCTGTTTATATGATTTTT

TGGTTGGTGTAGTTGTTGTCTTATATATTTATATATTTATATATTTATATATATATGTGT

TTGTTTGTTTTTGTGCGTGCGTGCTTGTGTATTGGGTATCGTTTTGTGTTGCGTTGTAGT

TTTTGTTCCATTATCCGAAAGTTTATACAAATATATTCCACTCTGCTTGCGTGCTAGGTG

CTGTCCTGGATGACTTGCAATATCAGTTGTCTATTGCCGTTGGCGCACGCTGAAACCGCC

CGGTTTTGACTACTGTTGCAAATGTTCCTCTCCGTTGCTCCTTTGCCCTCTTCTCCAGTT

GCTTTTGCATTTACGTGCTCTTTTTGTTGGCACTGGGAGGATGAGGGAACGTTCGGTGGG

GCGCCATGACACCACTAACAACTGTTAGAAGAATTAAAGTGACCAGAGGAGGGAGGAAGT

TTGGATGAGGAAATGGATGGGTGAGGGACTCTGGGTGGAGCCTGTTATACTAATGGATAT

TTTAAAAAGGAAAAAGAAAACGTTTCTTTTGTTTTGTTTATTTATCTTGATTCCCCGCTG

CTTAGTGTATCTGTGTGATGTTTGCGCCACATTTTTACGCGCATCATCTTTAGAGTACCT

CTGCGGAGAGTGACCCGCCTATTGGTTTTCAGTTTTTGATCTCTTAACCTTCCCTTTTCT

TCTATTCACCCTTTTTTTGGGTTTCTGTCGACGCTTGCGTGCGCTCCGATTTCTTATCGG

GTGCTGCGGTGATAACGCTGTGGAGCTTGGTTGAGTGCGTGGCAAACTCTGATTGTTAGG

TGTGTCCCCCCTAGTTTTCTTCCTTTTATGTGTGTGTGTGTGCGGTTCATAATTGAATGA

ATGAATGGATTTAATGAGGCTGCCGATATGTCGTGTGGTGCCGTATTCTTCTTTTTATTT

GCCACTTTCCAGGTTCGTTGAACGTAAATGCAGGGGTCCACTTCATCTGTAGTGAGTGCG

TGAGTTTCCTTTTTTTTTTCTTTCGTTGTCTTATTTGTTACCTCGAATCTCTTTCCCATT

CCCGCTTGCTGCGTCTTCCGCCTTTTCTTCTTTTTTGGGTCGTCTTTTAGCGGTGTCGCT

CCGATGGGGTTCATATGGGTAATTTGTGAGTTTTCAGGTGTTATCACCGATATTTGTTTG

CGGCTCGATGGTTGAAGAAGTTAGTGAACCTGCAACGGCCAACTGCAACGATCAAAAATA

AGAAAGGTGTATAAGGAAGGGGATGGGGTGTGTGTGTGTGTGAGTTGAGTGGAAGAAAAG

GAGTTGGTTGATAAGAGTGTGGAAGCGGAGATAAGGAGGAAACGTAAAGGTAAAAAAAAT

GCGATTAAAAAAAATATTTATATATAGATGGAGCAAGTTAACTGGGATGGTTCCTATTTT

CTTCTTTCGTGTGGCTCATGGATAACAATGTTGCGGGTACGGTAGCAAACACCCCTGAGT

GCGCCCAAAATCGACATAAATGGTAATATCGCGCCTTGTGGTGGTGTTTCTTTATTATTT

TTCTAAGGAAAATTATCCACCTCATTTCTAAAGCGTTTTCCAGCCTACTTGCTGCGTCTC

GTGCTAGTTGTGTGAGATAGATTATTGTTTATTTCCCCACACACGCACTGACACTTCTAT

TTTTTCTCTGCTAACTTTCTTTCTGTTTGTTCCTTATCTTCCCCTTCTCAATGTCGTTGC

TTTAGTTGCTGTGTCATATGTAGTACTTGCTGTTTTGCAGTGTTTCCTAAGTGGCGTGTC

CAGTAAATTTCCACACACGCACAAACACATCGTGGAAGCATTTGAGTTGGGTATCCCTTC

TTCGATGGTATTATTACAATTAGCCGCCTCCTTTTTTTCTTCACAAGAGAAAAAGAGCAG

AGAAGACAGCAGTTGGTCTCTATTGTTACGCACACTTTTGGTAACTGTCTGTTAAGTTCG

CGATGTCACGGGCCGTGCTAATGGAAGTCAATCATTGTTATCATTTTTGCTTACTCATTA

TGTGAAGACATCAAGGCTTCGCGGAGTACATGTGATGTTCGTTTTGCTTTGCTCTGTCGT

CTCCTGGGAGATTTGTTGTGAATAATCCGTTTGTTTGCCTCTATTTTGTTTCCAATTGCC

CAACACACATTTCCTTTCTTCGTATTTTCCCCGTTTTACCCTCCACGCACCACGCATTGT

GTGGCGCTGATGATAAGTTGTGCCGCTGTTGTGTGCTCACCGCGAGCATCTTTGCGGGAT

TGCGGGAAGTTGGGCGTGTGGGTTACCACGAGTGGTCATGCGGTTTTCTCGGAGGTTATT

TCACCCGTATCGTATTGATGTGAGCAGTCTGGAACGCGCCAAGAGGCAGCAAGGAGCACA

AGGGATACGCACGTCACCGAGAGTTTCGTTGATCCATTGCGCAAAGCGCGCTTGGAGAAA

GGCGATGTCTGGTTACGAATTCTTCTTTCCCTCTAGTGGGAAAGGCGGGCCGGCGAAGTG

ACGCACTCCAGATGCAAACTGAGCATCTTTGTCTTTATAGGTGCTGCGGGCTCCTCACGG

GTCCCGTTATGTGTGGCCGCACCTGTGAACACTATGTCAGCATCGTTCCGCTAAGGGTCC

TCTTCCCTCCGTTGCGTTTCTGTTCCACTCACCT

>Tb927.1.2310 | Trypanosoma brucei TREU927 | hypothetical protein | genomic | Tb927_01_v5.1 forward | (geneCodeEnd+0 to geneEnd+0) | length=411

AGTTAAATATACCGCAGCTACACCGATCTGCGTGCCCACACTTGTGTAAGCCCGCGCATG

TACATGAATGATTGGGGTGAATAAATGAATAAGTAAGTTTATTTATTTTTATCTTATTTG

TTTTCCTTTTTTTTTGTGTGTGTGTGTAGGGAATCGTATCAGCGTTTTCTCTTTTCTTTT

CTCGAGGTACAACCCTGAGAAAAGAATAGCAAAATCCCTAACGGCTACGCGCTGCGTGCC

ATAATGAACCGGACTTCTTTTTTTTTTCTCTCTTCTTCCTCTTCTGTCATTACACAACAT

GAGATATTTGTATCACCCCCCCCCTCCTTAAACCCCATGATGTGATTCCCCTCTTCCCCG

ACTACATCCCATCGTGCCACCCCTTGATATTTCTTTTTTGTCCATTTATTG

>Tb927.2.2920 | Trypanosoma brucei TREU927 | UAA transporter family, putative | genomic | Tb927_02_v5.1 forward | (geneCodeEnd+0 to geneEnd+0) | length=1096

ATAACAAATAAAAAAATAAAATAAAAAAAATGATAAAAAAACAATAAAGGCACAAAAAAA

AACATCGTTAGTGAGCTTCAGCTGCGGGGTTTCATAAATGGTATGAGTTTTATAACATTG

CATTTCGCTCCCAGAGAGGAAGAGATTGGAGACAACAGTTAATGCATATTTTCCGTGCTT

TTTGTCCACTTTTCTTTTTCTTTTTCTTTTCCTTTTCCCCCCCGTGGACGGGTGTGTTTG

GGTGCTGATTTGCCGCGTAAATTAAACATGCAGGATGAGAAGGGAGCGCAGAGAGAAGGT

GAGGGAAAATGGAAATGGAAATGGGATTGGGGGAAAACCAAAAAGAATAGTGGAAGCAAT

TTAAAAATTTATTTAGCTATTCGCATATGTATTTGTGACCCTTTACAGGAGAGGATAAGT

GGGAAGATGAGGAAGAACGTGTTGTGTGAGTCCGAGGAAATATAAATTGTGGGAAAGAAC

GAAAGGGTAGTGAATTCCATGAAATGGTTCTTGCACCAAATCTTTTTTTCTTTTAAATCC

CCTTTCCTTCCGTGTGTTGAAGGCAGAAGTGCACTGCGCAACCATATATATATATATATA

TACATATATATTCCCTGTTACTGTTTCTGTTATTATTATTATTATTTTCCGAAGGTTGAG

AGGAGTCTCCACCTCTTTTCCTTTTGGATGTATATCCGTTTATATCTAAGCGTGGCGTGT

TTTGTTTGTTTGTGTGTGTATGTGTGTGTGTGGTAACATGGGGGTGGATATGTACAATGT

ATTTGTATGTGAGGTATTCATCGTTTATCATTCGTACGACTCCCGCTGCCTATTTTTTTT

ACCCTTATTTGTATCGTCTTATTATTTTTTTATTTTGTTTTTACACGAACTTTTTACTTC

ATTTCCTCTTCGCGCGATTCCTCCTCCCCCCATCTCACGTAGTGAGCGTGTATATCTTTG

GTTGGTTGGTGACCAAACGGGTGCTATGGGAGTTTCCATACACACACACATACAGTCATA

TATTGGAACATCAAGGCGTGTAATGTTAGAAGTCGCGCTTATCCCTTTAACGTTGTAAAT

GTTTCTCAGCTTTTGT

>Tb927.7.5780 | Trypanosoma brucei TREU927 | thioredoxin, putative | genomic | Tb927_07_v5.1 forward | (geneCodeEnd+0 to geneEnd+0) | length=831

AAATTGTAAGAGGGGATGGAGGAGGAAAAGAGTTACCATTTGAAATCAATGAATCAGTTA

AAGTTAGCGAAGGTAAGAGAAGGACTGTGAAGAATTTCAAAAAAACACACACACACATAA

CTATAAATAAATAAAATAATAATAATAATAAGCTTTACGGAAGTACCGAGTGAGCATCGT

AAGTTGAGGAAACCGTAAAACACTCCTACGTAATCAAACTCTTATATATATATATATATA

TGTGGGTGGGTCCGTCCGTTTTTCATAGTGTGTACCGTATGAGTGCCCTTTTTGTGTTTA

GTTGATCAATTTTTTTATTTTTTATTTTGATTCCATGCAAAACTGTATGCGGAGCAATTT

CATCGTTAAAATATTCACCACGGAGCAAACAAACGGATTTATACTTATGTACATGTTAGT

GTTCTTAATTCGTATTTGTATTACGTGCTTCACCGTGTTTATTTCTTTATTTCTTTATTT

CTTTATTTCTTTTACTCTTTTGGGGCGGCATTCAACACTTCTTCCTTTCTTATGTTCCTT

TAACTCCTTTCCGAATATTAACGTCTGAAAGTGTGGAAGTGGAAGGAGGGACAATGACAT

CAAAAATACATGAAAATAAACAATATATTTATATTTATTTACAAGTGGGGAAGCGTCTAT

TGCGAAGGGGAAAAAATAAATAAATAAAATAAATTGCTTCATTTGAAGCAACGGGGGGGG

GAAAAAAACGATAAAAATGTGTTAGTGCCGTCACCCGGACGGTTTCACTATTTTTTTTTG

TTATGTGCTTCTGTTTCGGTAATAATGAGTTGTATATTTTATTTTAAATTT

>Tb927.11.180 | Trypanosoma brucei TREU927 | electron transfer flavoprotein, putative | genomic | Tb927_11_v5.1 reverse | (geneCodeEnd+0 to geneEnd+0) | length=301

ATGTTCTGCGGAGGGTGAGAAACCAATTTCTTGACGGCAGTGAGGGAAAATCAAATTGGG

TGCGTGCACACGTGTGTTTTCGTGCATGTGTAAATGTGGAAGCAATGGGATATGAAAGGT

ACAAGGAGGGAAAATTTGCGTTCGAATGCCTCCTTTTTTTGTGTTATCGTTCTATGGATC

CTTTGTAGTGTCCATAAACATAGTTCATTATATAAAATACCGGTGGGGACACCATTTCTT

CTTTATTTTTTCTTTTATTTCTCTCCTTCTCGTCATGCTTCTACGATTTTCGTCACCTTT

T

>Tb927.3.3890 | Trypanosoma brucei TREU927 | hypothetical protein, conserved | genomic | Tb927_03_v5.1 forward | (geneCodeEnd+0 to geneEnd+0) | length=1

A

>Tb927.11.4870 | Trypanosoma brucei TREU927 | hypothetical protein, conserved | genomic | Tb927_11_v5.1 forward | (geneCodeEnd+0 to geneEnd+0) | length=255

GGGTTGACTCCTTTCTTGTTTCCATTAGTCTCCAGGGAGAAGGTATGGCTGATGATGATG

GAACGGCCGCAGGAGAGGGGTGTGCGGAGTAGATGTGGAATAACGGTCTCTCTTTATATA

ATATTTTTTTTTCGTAGCTGTGACACTTTTACCTGTTTGTTATTCTAGCAGAGGGTGCCA

AGTTAGCGCTACACATCTTATCGTTACTTTAGGAGTTGTTGTGGCGTTCGCATTTTTTCT

GACTCCATTTCTTGT

>Tb927.11.1570 | Trypanosoma brucei TREU927 | hypothetical protein, conserved | genomic | Tb927_11_v5.1 reverse | (geneCodeEnd+0 to geneEnd+0) | length=1097

ACGTCGGCGCAAGTCGCGTCAGTACTTTATGTCGTGCCATCTTCCCTTCTCCCCCTCTTT

GTGCATTACTCCTGTTTAATATTTTTCGTACCGTTCCATAACTTCTGCATAGCTGGGCCA

CTTTAATTCTATTTGTTTTTTTTTTTCGCTCCGGACCTTTTTCCTTTTTTTTTTCTTGTT

TCACTGTTATTATTGTTCCTTTCCCCTCCCTCTCCACGCACACCGCAAGCTTATTTCGAT

CCCCCTGTCCACTTCCGTAACGTATATAAAAGTTGTTTTATATTTTTCTTTTCTAAATTA

TTTTTGACTGTTATATATGTTGTTTAGTCGACGTTTATTTGGTGTTAAGAATGGTAATAA

ATTGGGTATAACACTTAAATGTGGCCGCAATTACAAAAAAAAATTGGCATCTTCACATAT

TAAATCAACCCATAACTGTACGGGGGCTGGTTGGACGGCTTATGTCCAGATCATCATAAC

ATTTTAAGTTTCGCCTTCTCACGCGTAGTTGGTATTTATTTTTTGTTTGTTGTTGTTATT

ATTTTTTTTCTTTTTGTTTTTTTGTTTTTTGTTTTACTGCCTTTCCCTTTTTCCCTTAAC

ATCACCCGGATGAGAGGCATGTCAAAATACATTTCAAGTGACGGCAAACATTCGGGTCCT

GTTGAAATGATGCGACACGACATTTAACTGTGGTTCGGTTTCGATCCCCCCTCCCCCAAA

ATATGGAGATGTGTGAAACCACAAATCTGCGCGGTATACTTCAGCTCATTCATGTGGACT

CCAAAAGTGGGGAAAACTTTGTGAGAGTTAATAACATCATTTTGTTTTGTGAGCTCTTCC

CAATCACTGTTCAATACGTTTTTTAACCATTCTTGAAATATATTTTAAAAATCTTTTTAT

CCTTTCCTTTCCTTTTTTTTCTTTCCTTGGTTGTGTGTGTGTGTGTGTGTGTGTGTGTGT

GTGTGTGTGTGTGTGTGTGTGTGTGCTCTCCTCTTCCTTCCCGTTCCCGATATCGTGGAC

TTTAATTAGGGGCCCATCGAGGTTACAGACATCGTCAACTTTTGCGAAACCCGTGCAGGT

AAGTTAATTAAAGGTGC

>Tb927.9.2320 | Trypanosoma brucei TREU927 | methyltransferase domain containing protein, putative (POMP1) | genomic | Tb927_09_v5.1 reverse | (geneCodeEnd+0 to geneEnd+0) | length=1

A

>Tb927.4.3920 | Trypanosoma brucei TREU927 | CRAL/TRIO domain containing protein, putative | genomic | Tb927_04_v5.1 reverse | (geneCodeEnd+0 to geneEnd+0) | length=539

GCAGCAAGACACACATATGGATCATCGGTCGTACGAGACGTGGTTTGTTTATTTTTTGGG

GGGGTCATTTCTTTGTTCGATTACCTGCGCGTTCGTTTCGATTTTTTTTTGTTTTTATCC

GTCTCTACTTATTTTTTTTTCGTTATTTTACCGCCTTCACATTTCTTTAAAAACCCTTCA

ATCGCACTTGTGTTGGGGTGGTCCAGGTCTACATGAATTGACAATAATTACGGTGCCGTT

GGCAATGATAGATGTGTATTGCAAAAGCATTCCGAAATGATGAGATGGAGTTGAAGGAGC

GTTGTATATTGTTTATCGGGCGTTGCGATTTTATTTTATTTGTTTCTCTATTTGGCGTAT

AGATTTCATGTGAGGAGGTAAACGGGAGTAACAAGAATAAATAAAAAGATTATCGTCATC

ACTCTTTTTTTTCGTGGGTTATTTGTTATTATTGTTCGCCTGAAGGCATTGACACGCAAC

CGCACGCTCGACAAGACAATTTCTGATTGGTAAGAAAGGGTGGAACCTTTCGTATACAT

>Tb927.4.1910 | Trypanosoma brucei TREU927 | hypothetical protein, conserved | genomic | Tb927_04_v5.1 reverse | (geneCodeEnd+0 to geneEnd+0) | length=3368

AGGAAATGGAGACACGAGTATTTGGGTATGCAAGCCTTTTTTTTTTGTTTCCTCTCCATT

TCTCTTGAGACCTGTGAGTTTCCTCTTTTTTTTTTTTTTCGTTTCTCTTTTTTTTTTTTA

TTTGTCGGAGAGGGAGGCGAAATCGTGCATGTCAGTTCCTTCTCCGTTCCGACTTCTTTT

CTTTGTTTGACCATACACGTATTTGGTTGTTTGTGTCCTGAAGAGCTTATAAGTAGGATT

GTGAGAATGGGGAGACAACAGCCGCTGGAAGGGTGGAGGGGAATGGAACTGAACACATGT

GATTTCGAAAAGGAAACCATCAATAAATAATATATAAATACATATATATAAATAAATATA

TATATATATATATATTCCCCTTCCATTTCCTGCTTCCAGTTTTTCCGTTTTGTTTTTAAT

TTCCTCTTCTTCGTTTTGTTTTTGTGAGAAACATATTAGTTTCTGTGTTTTTTTTATATC

TTTATCTGTTTTTTTTTATAATTTAGTGCTTCAGATATGCTTTGTGTTTTCCTTTTTGTT

TTTTTTCTGTTTTACTTTTCCTTTGTTTGACTGTTTTATTATGTGTTTTGGTTACTTTCG

ATTACTTACGCACAATTCACCGGCACTTGTGCGGATATCATTGATATTATTATTATTATT

ACTCTCCCCCGCCTCATGGGGGTGGTGGGCGACAGCACGAGGGGGAAGTGAAAAGGCGGT

CATGAGGGAAAAAGAGAAGAAAAATAAGAAAAAAAGAACAATAATGAGAAAAGCAAAGGA

AAATGAGTGAGGAATTGCCAGACCAAATATATTATTTCTACACACACAAACACACGTATA

TATATATATATATATATATGAAAAGCTTGAAAGGTTTAATTTCTTTGTTTTGTAACAAAA

AGGAGGTAAAGGAGATGAATAAAAGAAAGAAGAAGAGGAGGAGGAGATGGTTTTGGCATG

CTTCTTTTCTCAACCTTTGTTTACTTCAATTTGTTTTCGTCTGATTTACTTCCCGTTTCC

GCTATATTCCTTCCACTCTATTGCTGCACCTTCCACCCACCCACACACACACACATACAT

ACATACGTACACATATATATATATATATATGCACAAATATGCATGTAAATAAATAAAACT

ATTTATTTATTTTTGATAATAATGATAATAATATTATTAATTGTCACCATTGATGTTATT

ATTATTATTATTACTATTTCACGGGAACACAGTGTCTCACCCTTCAACACATCACAACCT

CTCCGACCTCTCACAAGAGGGGATGTTCCGCCAGTGTTGTTTACATAAGCCGGAAACCAA

TGGAGAAAAAAAAAGGTGGAAAAATGTGGGAATATTTATATTTATGAGCATTTCGTTCAA

TTTTCAAATTTCATATCCCTTGACATTTATCCATTTCATTATTTTGGTGTGTTTGTATGC

ACGTATTATATGCGTAAAATGAATGTTATATGCTCATGTGAGTGTTGTTGAGTGGCATGT

GTTCCTGCTGCTGCCTTTTCCTTTTTTTTTTTCTTACGTTTGTGTGTATTATTTTTGTGT

GCTCGTCAAGCCCCTTCAACGCTTTCTTCAAAGTGGAGGAGGGGCTTGTCGCCATGACGA

AGGGGATGAGTGTTCGGGAAACCAAATAATTCTTTTTTTTTCTTTTCTAAAAACAAACAA

AAAAAAAGAAAAGGAGGTGCAAAACACGAGGAAAAATAAAATAGAGAAACAATATGAAGG

ATCGAGTGAGTGATGGTGGTTGATGTAAAGAGCGAAGGAAGAGGGAAAAAAAAAGAAAGG

AAGAAAATGTTAAAGGAAAGATGAAAATGACACAACCAAATAAATGCATGACATTACTCA

ACATCCTCTCTTTCATTTTGTTTTTTGTTTATATTCACTGTTTTGTATTTGTAATATCCC

CAAACCTCCCCAAAGTTCTCCTACACTTCTTTTCCTTTTGTTTGTTTTTTCCCCTTTCCA

ATTTTTTTTCCCCATTCTCTCTCTCCCTTTGTTCCTTAACCTGTTGTTTTTGTCTTTTTT

GTTTGTTTGTTTGTTTAACCTCAACGCGCAGAGAAGGAGCAAAAGTTAAGGAAAAGCAAA

AAAAAACAGAAAACGATTAAAAGAGAGGGGGAAAATGAACTTTTTGTTTAAAAAAAAAAA

AGTAAAACAAAAACAAAAAAAAAAGGACTCGTGCGTGTTATTTTGTTCAGAGTAATCCGT

AGCTTCTCCCTTTCTTCCTTTTTTCTATCTTTCTTTGTGATTTATTTACTTTTAATATAA

TTTTTCTTCTTAAAGTAAAGAAGACTGTTAACTCTCAAAGAGGCGTAGGGGGTCATTTTC

CAACCGTCTTTTTTTTTCCTTTTCATTTTCTTTTAGGTTTATTTTATGATTATGATTATT

ATTGTTGTTGTTGTCATTTTAGTGGAGAAAGGAGATGTAGCTCATGTTTATTTGTTTATT

TGTGTGTTTATGTGTGTGTGTGTGTCTATTTGTGTGAGAGTGTTTTTTATTTCCTTTCTT

ATTCCTCTATTTGTTTTACTTTCGCTCAATTTGTTCAACCGCGTACCGCATGCATTTTTC

CAACTGCTTCTGCTGTCGGTATTTGTTGTTGTTGTTATTGTTGTTGTTGTCACTGTTTTT

GTTTTTGTTTATTGTTTTTGTTTTAAAACTTTTATTTATTTCCTTCATTTTATATTCATG

CGTGGAATTTTTTAAAAACTTTTTCCGCGTGTCTGTGTTGTTTCACAACGCCACCGCTTC

CACCGCGATTTAGCCCCTTTTTTTTCTTCTTCTTTTTCTTTTTAAAATAAATAAATTCCC

CTTTCGTCTCACCTTTCCTTTCCTTTCTTTCTTCTCTTTCTTCTCTTTGTCCTTCTTTTT

TTTCTCTTCTTTTTATTTTTCTCTTGTTTTTTTTTCTTCAATTACTTTTCCTATTGCTTT

TTGCATACGCCATTCCCTTTTCTTTTAAAAAAAATAAATAAATATAATAATAATAATACA

CTGGTGTCGTTGATGTTGTTGTTGTTCTTTTGTCGTTTGTTGATTCTTTTTTTAAAAAAA

ATAAATATTATTTTAACATTCTTCACTTTCATTTTTTTTTTTAATTTTTTTTTTTACAAG

TTTGTTTATTCCCCGCAATTTCCATCATTACCACCGACTGATTGAAAGACGACAACACGC

GCCATGTTGTGATTATTATTATTATTGTTATTATTATTATTATCTTCAAAGGGAAAAATA

AAAATAAAAAAATGAAGAAAGGATGTGTGAGTGAGAGAAAGAGAGAGAGAGAGAAATGAA

GGGGAGGGGAGATGTGGAGGTTACCGTTCACAAAACTTTTAAAAAATATATTAAAAAAAT

ATAAAAAC

>Tb927.7.5380 | Trypanosoma brucei TREU927 | RNA-binding protein, putative | genomic | Tb927_07_v5.1 forward | (geneCodeEnd+0 to geneEnd+0) | length=1

A

>Tb927.8.6600 | Trypanosoma brucei TREU927 | Glutathione S-transferase, C-terminal domain containing protein, putative | genomic | Tb927_08_v5.1 reverse | (geneCodeEnd+0 to geneEnd+0) | length=1

A

>Tb927.10.5420 | Trypanosoma brucei TREU927 | hypothetical protein, conserved | genomic | Tb927_10_v5.1 forward | (geneCodeEnd+0 to geneEnd+0) | length=485

ACGGTAATATCGACTTTTGTATAAATATGAAATGAATCGTATCGACATCTGCTCCCTTTT

ATTTTTTTCATACATGCATAATCCGGATAAATATTCCGTAACCTAGCTTTTAGGTGGTTA

TTGTCTTGTTTATCGTTCTTAATTATTTTTTTCGTGACAGCGTGTTGACGGACTAAAGCG

CTTGCGTACGTCTGCCGTTGCAACTGTTCAAACTAGGACTATTGTCCCTCTTTGTGATAA

ATACACCGGTACGTGAGAAAAAACAAAAAAAGAGGAGGGGCTTGGGGCGGTTGTGAGATG

GTAAATTTGCCAGCAGTCGTTTGGCAACAATTATTTTACCATGATGTAAATCTTTACGTC

CTCACTTGCCTCTTCATCTATGTTTTGGGTGCGCGCTGTGTGTTTGTGCGCGTACGTGGC

ACCACAACCCTCGGTTTGAGATACTCACATTGTTTTCTACTCTTTGTTGGTATATTTGCG

TACAT

>Tb927.10.7250 | Trypanosoma brucei TREU927 | hypothetical protein, conserved | genomic | Tb927_10_v5.1 reverse | (geneCodeEnd+0 to geneEnd+0) | length=181

AGGCGCGTGCGCGATCGTTTGATTTTGAGCTAAGTGCGAATTGGATTTGGATCACTGAAA

CTTCGTCATCCTGAATGTGGTAGTAAAGAGACTTTTACATATATTTATTTGTGTATATAC

ACGTACGTACTCGTTATTTTTTGTGTTTCCATATACCTCTGGACCGTTCGATTTGTTTTT

T

>Tb927.10.9080 | Trypanosoma brucei TREU927 | pteridine transporter, putative | genomic | Tb927_10_v5.1 forward | (geneCodeEnd+0 to geneEnd+0) | length=1442

ATATCATTAGGGCGCAGCGACGCTTCGTTTACAAGCTTTTCAAGGCGAGGAGGAGCTCGG

TGTAAACACGTTTTTTTCTTCCCTCCTCTCGGAGAGATTGCAACTGCATTTTCCCTTATT

AATTATTTTCGTTCTTTTCCTCAGGTGCGGCACATTCTTCCGGGACTAAAGCGAACGCGC

TTTTTCTTGCGTTGCGTCGTGAACACCCGTTAGCCGCCACGACGAAAATTCTCTTACCTT

GTTGTTTGTTTTCCCATTTTTAAAAAGATTACGGAGGCTGTGCGTAGAAGTTGTTGCGAG

CAAAAACACATCTACAAACAGCTAAATCAAGTCAAAGAACTTACGGATAATTCCAAACTT

ACGTTTTACTACTGTAATCCCTTCCCTTTTTTAATTGTTGTTTCCTCTTCTTTTTTTTTC

CGTTTCCTTTCCTGTTCGCTTATATGTCTGCCTTGTCCGTAGACGATTGTTGTACTTCTT

TGAGTTCTTTAGCAATTTGTTAAATCAGGGGTGACCAGATCCCCCTTTCCCCCTTTTTTT

TTCAGCAACCGGTGTTGTAAAAAATTTTCGTGTGTTATTGCCGTTCTTAATCTGTTCCCT

TTTTTCTTTTTCCCTTTCCTTTTTTTCTTAACTACTTTTCATCTTTTTTTATTTTTGTTT

CTCTTGTTTTCACTGCTGATTAACGGCTGGCAGTGCAGTATGCGATCGTTATATATTTTC

TTTTTTTTGTTGTTGTTTCACTTCGGTTCAGGGGAGCACGTGCGGATATTTAAAAATTCC

GACACGCGCGTTTGTTTATATTTAGATGATGAAAGTTTCATCCATTGTTGTCACTCAAAA

CTTATTTTTTTCTGTTTTGTTTTTAGAACAACTCGTCTTCTTTTTATCGGTATTTTCGGA

AGTTATGCTTCTTTTTTTGTTTGTTTTTAAATGCTAGCGTCGCTGTCATTTGCACAACGG

TATATATGCATATAAGCTGTTCCTACACCTGAACTTAGTCTGTATAACCTTTTTGAAGCG

CTCCCATCACTGACTTAACCGTGAATTTCGCTCCTTTTTAAAATTTTTTTTTGGTTTAGT

TTTCTTGACCTCTTACTTGCCTACACTACAAACAAACAAGAAAAAAAAAAAGAAAGGTAA

TATGACGATGCAAGGCTTTGTTTCAATGAGTTTCGAATTGCTGACGCCAACCCCCTGGCC

TCCCGGATGAGAAAATCACATATATCTGTTTCCTCCATGCTTTCGTTTATCTCCTTCTTT

CGCTTCATCATCAGCTGTGCCTTTCTCAACAAAAATGTTCTTTTTTTTTGGGGGGGGGGA

GGGGGTAACAATTCGGTGTATACCAAAAACATACGAAAGAACCGAGTAACTTGTCACAGT

GATTTTGCACCATTTGTTAGTGGTTGACTAAAAGGTGAATGGTACCACTAAGGGAGAGGA

GG

>Tb927.7.4730 | Trypanosoma brucei TREU927 | pumilio/PUF RNA binding protein 5 | genomic | Tb927_07_v5.1 reverse | (geneCodeEnd+0 to geneEnd+0) | length=2432

AGGCACATTTGCATATTTTTTTCTTTTTTTTTTTGTGTGTGTGTGCGTACGTGCGAGGGG

ATCATTGCGATGACGGTGATGAATGTGTGAGGTTGAGGAAGCGATCGCGAATGAAAGAGT

GAAGAAAAAAAAACGAAGCAGTGAAAAGTACGAGAAGTTGAGACAATTAACGGTTGATCA

TGAAAGGAGCGACGGGACATGTGCCCCTCTTTTCGTGTGAATGCGTGTTAGGGGAAGCCG

TAATTGTTTTATACTTTCTCAACAATAGCGATTGGAAGCACTACAAGAATAACAACAAAA

GCAATACCGATAGCAGAAGGAAAACGAAGGCAAGCGATAAGACGCCACATTTCCTCTATT

GCATAACTCCCTGGCATTTCATATCCCCCGCGGTGCCTCCACTGAGCGAAAATGGGAGAA

ATGTAAAAGCGTGAAGAACACAATGGTGATTGTGGCATGTGCGGGATGGAGGGGAAAAGG

GAAGGGAACAAGCGAAGGGGAATAAAATGATATAAATGAAAAAAAAAGAAATATCCAAAT

AAATATATTAAACGAGGAAAAAATATATACGAGAAAAAAAATTTAAAGTCACATAACTAT

ATAGAGTATTTATTCACTCCGGCGTATTCTATTTCTTTTTTATTTCGTTTTCCCTTAAGG

GAAGAAGTTGTTGTTGTTTTTGTTATTAAAATTATCCGCTTGTTTTGTTTTGTTTTGCTT

TAATTTATTTATTTTTATTTTTATTTTTATTTTACCGTTTGTTAGAAATATATATACAAA

TCGGATGAAGTGCGAAGAGTAGAGAAAATAAAAATATTATGAAAAACGGACGACATGAAA

CAAAATGTTTGGGTGATGTTTCCTTTTTTTTTTATTTTATTTTTGTTCTCACCGTTTCCG

ATTCCGTGGTGCCTGCAGTATTAGAACGTTAGAACTGTCGCCCACAATTATTTTATTTTT

CGGCATGCCTCAGTTTATTTCTCCTTTTTGTCTCCACGTGCGTGTGTTTGTTTTCCTTTT

GTTTTATTTTATACGTACACAATTGCGTTTGCTTTTTATTTTTATATTTATTTATTTATT

ACTTGTTACAACCTGCCTATGTTTGTTTTATTTGTTTATTTACTTATTTGTTTTGTTTTG

TTTTGGAAAGAACTCATGTCATCATTCAACATATAAGGAAGAAGTGTCGAGAAAAGGGTT

GAAGAGTCCGAATGACATATTAAATGTTTAAAAATAAAAAATAGAGAAGTAAAACCAAGT

GACAAAGAGGAAGAAGAACAAGAAGAGAGAAAGGGAAAGGGAAACGGGGAAAATGTCGCC

ATGGAGGGAAGCACTAAAAAATGACGGGCTTTGAAGCCGATGAAAGATTGCATCGGCGAA

GGGAACTGTAAATAATGTTGATAAATTATAACGTCCGTTATTGTCACAATTGCCACTCTT

GTTGCTGTTGGTGTTGAAATAAAATAAATGAATAGATAATTAAAAGCATGAAAAGGGAAG

TGGTTGCGATGCGGTGAAATGAAAAAGGAAATATGAAATGAAGTGAAGTGAAGTTGAGAA

AAAGAAGAGATGGGAGGTTTCATGTGTGCGCGTATGTCTGTGTAATCACGTTGGCATTAC

TTTAAATATAAATAAATAAATAAATAAATGTATATATATATATATATATATATATATATA

TATATATAGTATTTAATTTACTTCTGCGAGCAGTTTGGTTTCATTCCTATGCGCTAGGCG

AGCACCTGTACACACAACTAGAAGATGTATATGTTCGCCAAGATTCACGTGAGGTTTGTG

TTCATTCTCGTTCCTTTGTCTTTTTTGTAAAGTGAGTACGGACGCTCATATATATATTTA

TATATATATATATATATATATATTTATTTATTTATTTATTTATATTAATTTATATGATGT

GTACTTGCATTAGATACATTATATATGCTACACCATGTTATATATATTATTTATGTGTGT

GTATGTTTCTATCTATATATGCATATATATATATATATATACAACGCACATGTGTTTGCA

TGGGGTTCCTCGTGGGTGTTCATGTTTTATACGTGAACATATAAAATACCTGAAAAGGGG

GAAAGGGGGGATTTATATGCGGAAATATATACATATGTGTGTGTAAGTGTGGGTTTCATA

CTTTTCCCCGTTTACTTCTTTTCCAATGCTTTTCCCTTTCTTTTCTTTTTACTTTTTTCC

TTACCCAACTGCCCCGCGCTTCGGTCAGTTGCTTTATTTGTTTAGTTCAAATGTTGTTGT

TGTTTTTGTTCTTGCTTTTTTTTTTTGGTTTCCATTCCCACTGTTAATGCTACTGTCATT

ACTTTTATCGTTGGCCGCAGAGGGGCGTCACACAAGACTAGCGGACCGGAACTTCGCATG

TGACAATTTAATCAAATCATCATTTGTGCTTT

>Tb927.8.6080 | Trypanosoma brucei TREU927 | Glycerophosphoryl diester phosphodiesterase family, putative (POMP42) | genomic | Tb927_08_v5.1 forward | (geneCodeEnd+0 to geneEnd+0) | length=901

AAAACCCCTCCAAAATAATAATAATAATAAAGGAAAACTGAAAAAGAAGGGAGAAAAGGG

AAGGGACAGGACGCGGTTAGTGTGAACGAAGGAAAAGAAAGAAGTGAGAGATCATGAGGA

AATGTATATGTGCATGCAGTATGTTTTGAATACAAATGTATTTTTTTTTTTGGTGTGTGT

GTGTCTGTGTTTGTGTGCAGCTGTGTACACATATATATATATATTTGTGTGTGTGTGTGT

ATTTGTCCGTGGAGTGTATGCTGGAAGAGAATGCGAGCACGTGCGTATTATATTCCCTTT

TGAAGGAAAACGCATGGAAGAGGAGTCACTGGAGGAAAAGGAAGTGTAAAAGGGACACAT

GCAGGCGTGTTTGTTTATTCGACCTGAGGAAAGTTTTGGGAAAAGGGCGCGAAAACATAA

CACTGAAAATTTGTGGTTATTCATCATAGAGTCTGAACCGTGTTGTCATCTTTCTTTGTT

CCTAATTTTCTTCCTTTTGTTGTTGTTTTGTATTCCCCATTCCCTGCTTCCTTTCTCCCG

TGTGTATGTACCCATTTACTTAATGCACAATTTTGTCGCTCCGTAACGCCATTTGTAGGG

AGCCGTGCGTGTTTGTGTTTTTTTTTTTTCTTGTTTCGATTTGTTCCCCGATTGGTTTTG

TTTTGTCATTATCATAATTGTCATTATTTTTTTTTTCTTCAGTTTTCTTGGTCACCTCCC

GTCTCTTTGTTTCTTCGTTCCCTTTGTTTTTCTTTCTCAAAAGTATGGGTCCATATTCCC

TCATTCCTTCGTTCCCTTTGTTTTTTTGTACAAGTTAAATGATTCTTGTAATAAAGGTGT

GAAGGGAGTTTGAGGAAAGGTGAAAAGGAAGAAGTCACTGCTTTCCCTTTTTGACTTCTG

C

>Tb927.7.2670 | Trypanosoma brucei TREU927 | zinc finger protein family member, putative (ZC3H21) | genomic | Tb927_07_v5.1 reverse | (geneCodeEnd+0 to geneEnd+0) | length=3589

AGCATTGTGAAGAAGTTCCCTTCCATACCACCACTTTCGTTATTATTTGGTTATAACTTT

TGTAGTGCATCTTTCTCCGTTTTATTTTATTTTGTTATTATAATTTTTCCATGTTTCTCT

TATAGCTCTTGTATATTTCCTTCTCATTTTTTTTTGTGGGAACACGTTGTTTCTATTTTT

TTTTTGTTATTTTTGTTGTCACTCGGCTATATGCGTTCGTTACTCCCTTCTTGCGGCAAA

TAACTTATGTTAGCATCGCTGTCATCTTTTTTTTTGTTAATACAACTGTTGTATTTGTAC

ATATGCTGACATTACTGGATGATGGTAAGGGGTGAGGAGGGGAGGGGAGGGGTGTTGAAG

GAGCGTAAATCTCCCTTTTTATGTTTGGATTATATTATTGTTATTATGCAATAATATATA

TATATATGTATTTTTTTAATTTTTGGGTAAAGGATACTGTCGTAGAGAATAATACGAAAT

AAGAAAAAAAGAAATGTTTCATTTTTGTGAAGCGGGTGTGAACTCACCTTTTGTGCTTCC

TACTCTGCCTCTTCTATCGCTTCATTTGTGCTTTCTCTCATTTCGGGTCCTGTGGGGTTG

TACTTGGTAGATGTCTTCGTTGATATCGGAGTGAACCGAAGAAAAGAAGAGAGGAGAGAA

GCAGCGAAACATATGAATGAGATGCAAAGAGAAGTGAAGGAAAGAAGAAAGTATTTAAGC

GTTTCACTGTACATAAAAGGGTATGGAAGAGAAATATATAAGAAAAGGAGAAACTTACTG

ACGTGTGAAAGGGCATGTGTCATTTGTTTCTATGTGTAAGAAAGGAATGTGTAGGTAACT

TAACATTTGATAGGAATGAGTGAGGGAGAAACAGGTGGGAAGAAACAGCAGTATAATGGT

TATTATTATTATTATTATTAGAGTTGTGTGATGCTTAACTGTGCTGCATTTCTATTTATT

TTATTTTGCTCTTCCCCATTGTGTTGTATCCTGTCGTTTATCTGTTTCCCCTTCAGGTGA

GGCATCTTAATATAAGAACGAAACTACTGCAAAAGTGAAGAAGAAGAATTGGCTGACAAG

CATACGCGTGTCTTTGCCTTTTAATCTTCTCGTTCGGTATCTGTGGACTTGAGGGCGCGG

TTTTATTTGTTTAATTTATTATTGTTTGCACCTGCTCATCTTTTTCTCCCCATCTGTGTG

CTTCTGAATTGATCGAATTGTACCCCGTATGGCGAGCTGGGAAGGGGCGGGACAAAAAAT

AAAGAGAGGTGAAAAACATATTTTTTGTATAACGTCAAACAGTGCCACCAGGCACTGAAG

GAAAGGGAGAGGGGTGAGGTAGCGCATGGTTTCTCCTCCTTGTTTTTCTTTTACCCTTTT

GATAATTAAATATAAAAAATAAATGATATCAAGAGATATTAAAGAAAATAAATATCTATT

GGAGTCGGGGAAAAAATGGGAAGTAACAGGATCACGGTGTGTGTTCCTGTTTTTTTTTTC

CGGACGAACTGAATTTCTTTTTTTTCTTCTTTATTTCCTATGTGTGGGCGCGTGTTTTTA

CGCGTGTTTGTTTGAAGCTGTTGAGTAAATAAAGGAATGATTGGCTAACTGACTGACTGA

CTGACTGAGTGAATGAGTGAGATGGATATAAGTTTGTCGGCTGTGACAATAAAGTGGTGA

GATGCCTTTACATGTGTGCATATGTGATGTTATCCCGTTCCTTGTTTAATACGGTAGAGA

GGCGTTTCTTTTTCTTTTTTTTTTTGTTGAAAACCATTCCCTTTCCCTGTAATATTAATG

TACCATTTGTGGGGGATTTCGATTTCCATATAAATATTATCAGTTATTATGTTTTAACTG

ATTTTTTAAAATTTCCTCATTAGGTACAACTTCAGCTTTTTTCTTCTTCTTTACCTTTGT

AGTTTTTTTTGTATGTGCGGTTAGGGGAGTCGTCGGTGCGTTGTGTTTGTTTCATTCATG

TTTTTTTTTCTAAACCTCGGTTATTCATTCACTCAGTCATTACGACGACTTCCTCCATTA

CACCTTCTTCCTTTTTCATCTTTTTCTTAAGTTTTATTTTGTACCTATCGTGTTGTTGCT

GTTTTAAATTTTTTTTAACTAGTGAATGGGCATTTGTTTCGACAATGCACCGGTTATTTT

TTTATTTTTTAATATATATATATATATGAATGTATGTATATATTTACCAGGTCTCTTTTA

TTGCGCTTGCCTTTCTCTTTTCTTTTCCTCTCTCTCAAATTCATTCTTCCATTTCCCTTT

CTTCCTGCCCAACTTCGTTGCTGCCTTCATTTCAATATAATTATTGTTATTATTTTTATT

ACAGCAGCAGTCGGAGGTATTAATGATCTATCATTATAGTTACTATAGTTATAATTGTTA

TATATATATATATATATATATCTTTTTTGTTTCCCTCAACCCAGCCGAAGTGTTTTCGTC

ATTTTTTTTTTGGACAATCCACACACATGAACACGCGTTCATTTTTTTTTTCTGCACTCT

TTTCCCCACCTGTTCGTAATTCCGTTTTATCTCTACTCGGGGACCGTTTTTCGTTTTCAT

ATTGCTTCTGATAGCACTTTTGGGCCAATTTCATTCGCACATTCTTGCATTTGCATACGT

GTCGATTGAAAAAGAAATGAGGAAAGGAGGTAAAACCCGACTCTCTTTAACCGGTTGTTT

CTTTATTTTTATCTTTTTTATTTTTATTTATTTGTTTGTGCTGCCCTTTTTGTTTTTTTC

TTCCCATTCCCATTCCCGTTTTCATTTTCATTTTCATTTCCTTTCTCTATCTTAGTTCCC

GTCCCCCGTCTACCGCCTTTTGGTTCTTTTACATCTTACTTTGACTTGGTTGTAGCATCG

GGTTGCTTTGAAGGAAATTTAAGGCATTATGTATCTGCGTAAGCGAAATAGAGATTAAGA

AAGGAAGAAATGTTAGGGAACCAAAAGGAAAGGAAAGGAATGGAAGTGTGAGGGAAAGTC

CTTTTCTTTTTTTCTTACGCTCTTGCGCGGTAACGGAAGTAATGATGACTTACTGACTAC

GAGAGAATAATCACCAAGTGGTTGCGCGGTGCCCGTGGGGTAACAGAAAGGTGAGTAATA

GAATTCTGAGGAGGTTACGATGGTAAAAGGTAAGTAAACACACACAAACGCCACCACAAA

TACTTATAGGTGCGCGTTTCCCCTTTTTTTTTCGAAAAAAAATAAGTATATATATATTAC

TGTGTGCAGCTTCATTCAGATGCATGTGTGTGTTCGGATCTTTGTGCACAGTTTTCCTGA

AACATTTCGCGTGGGTGGCTGTTGCTGGTGAAGTGGTGATAGACTAGGTACTATGACCTC

CTTATATTATTATTATTAAACTATGTGTATTTCTTTATACATATATTTATGTATTTGTGG

CGTTTCAAATGTTTTACTCTTTGCCTTTTTATCATTGTTATTTGTGCCGCCTTGTCCCCA

CTTTCTCTTCATTATCTGTTTCCCTCTTCTTCTTCTTTCCTCATCATCCGTGAAATGAGC

TGTGCGATGGGTTGTGTAGGTGTTGGGTGCTGGATTCCTAACCCGCTGG

>Tb927.4.3520 | Trypanosoma brucei TREU927 | Amastin surface glycoprotein, putative | genomic | Tb927_04_v5.1 reverse | (geneCodeEnd+0 to geneEnd+0) | length=457

GCGGACATTTAAGCAGCTGCGTTGACATTCTCGAGCGGCCTCATTCCTTCCCATGACACT

GAAGGTTCTGCAGGTACGCGGCGTGGTAGTGACTATTTTCTTATTTTTTCCTATACAATT

GCGGTCGTGAACGAACGTTTTTTATTTTTATTTTTCTATCTGTACGTATTTCCTATTCCC

AGGTTTTTTGTTCATCTTTCCTAAGCTGCAACACGAGAATATACAGATATCGATTTTACT

TATTTCATTTATTTATTTTACGTAGATGTTTCCGAAAACAGCTGTTAAACTTCGTTGTCA

CTCTCCTCCGTTTCGTGTTCCCTCACATTTCTTTTACGCTGTGGGCAAGATAAGTTTCTG

GTTCATCCCTTTCTGTTAGAATTACCACTTTGTACGCTATGTGGTGACTTAGGGTTATTC

TTACCTGGTATATTTATATAATAAGGCGTGGTGCGAC

>Tb927.5.2560 | Trypanosoma brucei TREU927 | hypothetical protein, conserved | genomic | Tb927_05_v5.1 forward | (geneCodeEnd+0 to geneEnd+0) | length=464

ATGTGACCACTAAGTTGCAATGTATGATGCAACTTATCTTGTCACAGCGTGAAGCGGCAT

AACGCGTGGAATGTATACATGTACATGCATGTTGCCTTGTTTACTAATATGACTGTTGAA

CCACCTTTAAAAATGTGTCAAGGTGGAGGGGGTGGGCTGGGTATTATAGTTGTCTCTGTG

AGCTGTTTAATAGTAGGTTAGAAAAGAGCCGGAAGTCGGTCTAATGGCCCGAGCATCTGT

ATGCGCATCGCGCAACTGACGCTTCATTACCATTATTATTATTATTGTTATTTTTTTCTT

AGGAAGTACCGTCAGGTGGATATATATATACATGCGTATCCCTGTGTGTCGGTAACGGAA

GGAAGAAGGGAAATCAAGTCAGATTCCTGTTGGCTTAACCCAACTTATTTTCGAACAAGG

TGGTCCTTTTCGCTTGTATGTGTTTTTGTTGTCTCTTTTCCTGT

>Tb927.5.3130 | Trypanosoma brucei TREU927 | hypothetical protein | genomic | Tb927_05_v5.1 reverse | (geneCodeEnd+0 to geneEnd+0) | length=1910

ATCGACTGTGTGTGTGTGTCTGGTAGTCTCTCAGTGTTCTTCTGGTTTTTGTTTGTTTGT

TTTTCTTTTCTATCTTGTCTCCATGTTTTTAATGTGGTTGGATGCACGGCGTGTGATGTA

CGGTGTTTTGTGGGTGATGTGTGTATGGGGGGGGGGGAGGGAGGAGGCACAAGCGAAAAA

ACAAACAAGGAATCGGTCGAATGATCTACCGGTACGTTCGCTTAAGTTAGCGGATGAAAG

GAAAGAAAATAAGGTGTATGTTGTACCCTTATCGCTCTATCCCTCCTTCCTTATCATTAT

TTTCTTTATATATTTCTTGTTTTTATTCTCCTACCCGTATTTGGTTACATAAAAACACGG

GTGTCGGAAGAGAAATCAGACAAAACGTGACAACCCGAAAGAAATCAATACGTATTGTTC

AATTTTTTCTGTTTTAAATTCCTTTTTTGTCTGTATTTTTCCTTTGTTTTGCTTTTGCTT

TCCCCCTTCGCTCCTTTATTAAAATATACATTTATTCCTTTCTACCATTCCCTACGCAAA

AGTTATGAGTGAAACAATAACACGAAGGTGTTGTTGTTGTTTTTCTTTTCCTTTTCCCTC

CATGCTCCGCTTGACTCTATTTTGTTTTTGATGCCAGCGGAGTGTCTCCCCTAACCAGGC

GGTTTAGGAAGAAGGGAAAGGAGGATAGATATATACGCAAAGAAATAAAGAGAATAAAAC

GGCAATAGGCGGACAACACCAAAGCCGCACAAAGAAAACAAATAGTGGAATACAGGGTGG

GGAAGGGGAAAAAAAAGTCAAAGGGAACAGGTTTCTATCATTTTTTTTTATCTCTGCAAA

TATTTATCACGCATTTGTTTCAATACTTCTCCCCAATCCGGTGCATCACGTTTGCGACAC

ATTCATTTTATTTTTAGCGTGTTTTTAAACGTCTTCCATTCGAGAGGAGGAAATTATTAT

CGTACGCTTGTGTGTGAGACGGAGAGAATGAGAACTTTTAATGTGTCAGCAGCCGCAAAA

TTTAAAAAAAACGCTTTTTATTACCCCCATTTGTTTCACTCATTTCTTCTCTTTTTATTT

TTTTATTGTATTTTTGGTTTGTGGTGTTGTTCATTCCTCTCGCGTGTTTTCTCTTTTGTC

TTTGCTATTTTACCATTCACTTAAACTTTTTGATTTCTGTCCTAAGTTTTGGTTCAACTT

CTACCGTCCATTTCCATTGATTTATCTGTCATTACAGTACAGTTACGAATGCAGGTGTGT

GGTTGTGTTTGGTTACGCTTTTAAAGATAGTTTTTTTTGTTATCTATCGTTATAGTGATG

ATAACTTTTGCTATTATTTTATCCCTCCCCTTATCCATATATATATATATATATATATAT

ATATATATATATATATATATATATATATATATATATATGCATATCTCATTTCGTGTTCAT

TGATGGAAGTGAAAGCAGAAAAAAAACATATATATATATATATATATATATATATATATA

CACATATATATGTGGATTTTTTTTTTCGAGTCGGCACACGAAATTGAAATAAAAAAAGAG

AAGAGTGAAACGCAGAGGTCCACCTTTATATTTCCGTGCTGCAGTTTCTAATTCATTAAT

TCACTAATTTGTTTTTTTATTTTTATTTTTACCAACAGCCAAGCAATTGACTAAGCAATT

TTACTAAAGTGCAAAGGACAGATAAAAGTTTGTTTTCTTTACGTATATTATTTTGTTATA

TCTCTTGTAAAAATCCGAATTGCAACACGATTCCTTGATTCTTGTCTTCTTTTATTTTTG

ATTTTTTTTCTGTTTTGTTTTCCTTTGGTGTAAATTTGAATGTGTTGCTACGTTATGTTT

TGACTTATTTGTTCCTCCTATTAACCCGTGTGTCACGACTCTTAAAATAT

>Tb927.4.5060 | Trypanosoma brucei TREU927 | hypothetical protein | genomic | Tb927_04_v5.1 reverse | (geneCodeEnd+0 to geneEnd+0) | length=1

A

>Tb927.11.5330 | Trypanosoma brucei TREU927 | hypothetical protein, conserved | genomic | Tb927_11_v5.1 forward | (geneCodeEnd+0 to geneEnd+0) | length=387

AAGTAGTGAAGCCTACTGTAGCAACTGTTGCAAACAATAGCGATGGGTAGACGTTTCTGA

AACTTTGTCTGATGTTTTGCGAAACATTATATTATTATTTTTTTTTTTTAAATGGTGCCT

GCGCCGCTGCACATCATCAACTGTAACGTTCGTTGTCTGTTTTTGAGTGAAAAGTATTCC

TACGTTTACTTGTTTATTTTTTTAAAAATTTTCTCCGTTCCCTTTTCCTTATTACAGGAG

GTTGCTCTGCCACGAGTGCGCAGCAGTGGCACTCCGTCCACTTTGTCGTTGTGTTTAGTT

TGGCGAAGTGATGAGATACTCTAACGAGTAATTCTTTTGGTATGCGGGAATTTAAAAAAA

AATTACCCGCTTGTTTGTGCGATATCG

>Tb927.11.15660 | Trypanosoma brucei TREU927 | hypothetical protein, conserved | genomic | Tb927_11_v5.1 reverse | (geneCodeEnd+0 to geneEnd+0) | length=308

GAATAGTAGTACCCCAACCTTTGCACTATAATTCACACCGGATCCTTCTACGGGTTGTGG

TTGTTCTTCTTGTCTCTTGTGGATAGAGGCACTACCGCACAGAGAGGCCTTTACTTTTTT

GTAGTTTAACTGCTATTACGGTCGTTGGGCCTATAAATTTGATTTCATTTTTGGTTTGTT

ATTATTTCCTTTCCTTTTTTAGCTATTTTTTCTCATTTTTATTTTCTGAACTTGAAATAA

CTTTCTGTGTGGGTGACCCCTCCCCAAATCCATACACATTGCTTTAGGTATTTCATATTT

TCACCTGG

>Tb927.9.3170 | Trypanosoma brucei TREU927 | cytochrome oxidase subunit V (COXV) | genomic | Tb927_09_v5.1 reverse | (geneCodeEnd+0 to geneEnd+0) | length=203

GTAGCTTTAGTATCTTCACGGATGGGGTTATTACTTCCATTTTTTATTTTTTTGATTGTT

TTAAAGTAGGACCATAGCCAATACTAGTATAACACCCAATGGGTTGAGTTGATGTAAAGG

ACTGTATTCCGTCGTTTTGTTAGCCTGTTTTGTTGACACATACATATATATCTTTTACCC

CTTACCGTAGCGGAGGGTTGTGG

>Tb927.10.3850 | Trypanosoma brucei TREU927 | hypothetical protein, conserved | genomic | Tb927_10_v5.1 reverse | (geneCodeEnd+0 to geneEnd+0) | length=1

A

>Tb927.10.5190 | Trypanosoma brucei TREU927 | Kinesin associated protein, putative | genomic | Tb927_10_v5.1 forward | (geneCodeEnd+0 to geneEnd+0) | length=373

AATAAATGATACTCTGCTGCTGGAACTTACCAAATGGGATTTCTATTGATACAATCTTAG

TTTAAGGCAGCCCTTTGAATCAAATATTTCTGCAGCTTGAAGTTCCCGTGTATTTTCTGA

GGGCGAGACATTTATAACGTAAAAGTGGTATTATTTTCAGATACATCGCCTCTTTCCCTC

TATCTCTCTTTCTCTGGCGGACGTTTATTTTTGTTTTATACACACCTCCAGTGGTAATTT

TTTTTTCTTGTATTCTCTGCGTGCTGAAGTTCCACTTTGTTCCGTTCCATGTTATAGAGT

AGTGTGAACTCAAATGTGTGAGGGAAATAAGAGCTGTCTGATTATCTTCTGTGTGTGTTT

GTGTTTGTTCGGG

>Tb927.10.480 | Trypanosoma brucei TREU927 | hypothetical protein, conserved | genomic | Tb927_10_v5.1 forward | (geneCodeEnd+0 to geneEnd+0) | length=1

A

>Tb927.11.12320 | Trypanosoma brucei TREU927 | hypothetical protein, conserved | genomic | Tb927_11_v5.1 forward | (geneCodeEnd+0 to geneEnd+0) | length=325

AGGTGGCAGATGCGCACGCAATGAGCGTCTTTGTCTCTCTTGGGTTTGTCTTTGTTTGAA

ACATTGTAATCTTCTTATCTGTTGTTCCCCGGGCCCTTCTGAGAGGGGTGTTTATGTGAA

TCGGTATGATTTCTTTTAGTCTGCGGTTACTTCACCGCGTCCACCATTATATATTTTGTT

ATTATTTTTTTTTAAACGCAGGTCGCGTACAGCTCCTTCTATTTCCTTCACTCTTCACCA

ATAAGTCGTTGTGCCATATTTTCTGTGATTGAAAACTTTCAGAAAAACACTTCGGAAGTG

AAAGTTCCGCAGTATAGTATTGTTG

>Tb927.2.4700 | Trypanosoma brucei TREU927 | hypothetical protein, conserved | genomic | Tb927_02_v5.1 reverse | (geneCodeEnd+0 to geneEnd+0) | length=1867

ATTTGTTCATTTTTTTTATTTTTATTTTTATATTCATTTTCATTTTAGGTTTTTTTTCTT

TTTTTTTCCCCTCCCTCTCTCCCTCTCTCTCTCTCTCTCTTTCTCTTTCTTTCTTTTTTT

TGTTTTTTGGGATGAACCTCAATTTTCCCCTCCTCTCCTACAGTTTTTATGAAGGTGTGA

GTGTGAAGGATTTTACTTACTGGGTGTGATCATTAAAAAAAAAACAAAATAAAAAAAAAT

ACAATTATAAAGTAGTGAAGGGGGAATGCGAAATAAAAGAGAAGATGAGGAATCGGGCCG

TACAACACTCAGAAGAAAGTGTTTTTTTTTTTTTGAGTATTGGGATTCATTTGGCGCCCT

TTTTTTTTTGTTGTTTTGTTTTGTTGTTTTGTTTTGTGCAACACTCTGAATAATAGGCTG

GCGCAACGTAGATTAATAATGTTTTGCTTCCACCATGGAAACAACAACAACAACAACAAC

AACAACAACACACAATAAAAAAAAAAAGAAACAAAAATAACAACATCGAGGAGGTGAAGT

AACAGTGGTTAGAAATCCCATGTGACTCCCTTGATTTGTTTTTTTTGTCTTAGTTTCGCC

TCGACGTATTCAATTTCTCTTTTATTTTATTTTTTTTTTTCTTCTGAAGCATATGTCCAC

TCGTTGCTTCATCTCATCTTTTAGCATGGTGAACCGCAGCCGTTTTCGAACAGTGTATCA

GAACAAAGCATCGTATGCAGCCCGCTAAACTACCTTGCAGTATCGAGTGTTTAACTCCTC

TACTTCATAGTTATTATTATTATTATCATTATTATCATTATCATCATCATCATCATCATC

TTGTGTAGTGAACGCATTAAAGGAATTTCCCTCCTTTTTATTAAAAAAAAAAAAGAAAAA

ACAGAGGGGGTGGGGAAAAAACAACAACAACAAATGATCGGTAGTATTTGCTTCGTATTC

ATTGGTGATGTGGCGGTTCTTTTTCGTTCCTCCTTTTTTTTTACTTTCCTTGTGCAAGAG

GCACTTAAGAGCCGACGTTTTTACTCATCGGCTGATTCGTTGATTATGCACATACCTCTT

TTTTTTTTTCTTCTTTTCCTATTGTATTTTCTTTTCCCAATACCTTGTTAAGGTTTGTGT

GTGCTTCTGTGTGTTGTTTCCTCCTTTTTCCTCTGTTTCGCGATTCTTCTTCTTCTCCTC

CGCTTTCCAGTAACTTCTACGGTTTATTTACATTTGAAGTCTTCCTCACATATAATGAGG

TTTATTATTATATCGCAAGCAAGTGGGAGGATGAGAGATAGGTTTTGATTCCCTGAAGTG

AGGTATTTTCTATCATTATTATTATTATTATTATTTTGTCATTACCGAAAGAGCATATTA

AAATGTAGAAAAAAACCAAACCAAACCAAACCAAACAAACAAACAAACGAAAACGAGAAA

ACGTTGTAAACTAGTGGTTCGTGTTCTTCAGTTGGTAAGTTGTGACTTTTTTTTTTCTTT

TCATCTTTTTTTCCCTCTATTTCACAGTTCGTCGATGCACTTTACTTTTCCCCCTCCTCC

TGCCCTCACCTTTATCGTGTGGTTTTCTGAAGGAAAGAGGTGGAGGAAAGTAAAGTGAGA

CTCACACTTCATCTTTATATTGAGACAATATTGTGGAGGAAAAAGGGGGGAAAGAGGCAG

AAAGAAAGAAAGAGAAAGAGAGAGAGAGAGATTGAGAGATTGAGAGATAGCGGCTCCCTA

TAAATGTGACTGTGTCCATCTGTATTGTTTTCATAACCCTTGTTGAAGGGGAAGAAAAAG

AATAAGGATAAAGATAAAGAAGATGTTTTGAGTTGATCCGTTTTGTTCGTTTAGTTCCTC

ATGTTTC

>Tb927.8.2420 | Trypanosoma brucei TREU927 | hypothetical protein, conserved | genomic | Tb927_08_v5.1 forward | (geneCodeEnd+0 to geneEnd+0) | length=489

AAGAATTAGCGTGGCGCTCCTTTGACATGTTCCTGTGGGCGTGTAGCTCCAACAGTGTGA

CCTTCTTCTTTTTTTTTTCGTGATTGCTTAACAAACACACACAGGTGTCCTCATCTAAAT

GGATAGGTACCGTGTATGTGGGGGAGAAGGGGCTGGGTTATGGCCTTCCGTGATCCTAAG

GAACAACCGAAATCTGATCCCACTTTACCTCCTTTTCACTTCTTCACATGTGACTGTCCG

GCATGTGCCGTTTTTGACATTGTGATTACGGTGTGCTCATCAACACCATTTGGTTTTCGT

AACCGTTGTTATTTTTTTTCGATATACCAGTTGATTTGTGCCACATGAGAATTAGCCTTA

ACAACAACGCTATAACCATGTTGAGCGCATTTGCAGTAGACACATATTTTCTTATCTTTC

CTACTTTTATCATCTGCTTGTGTGCGGCCTGAATAACAGTTGGGGAGAGGGGAACAGTGC

CAGAGAGTT

>Tb927.2.5530 | Trypanosoma brucei TREU927 | hypothetical protein, conserved (POMP22A) | genomic | Tb927_02_v5.1 reverse | (geneCodeEnd+0 to geneEnd+0) | length=1131

ATCTTCATGCGTGTGGGTTAGGAAGCATATTATCATTGTACACGACTGGACACGCTTTTC

TGGGGGTGGCATCCGTTGACCCAGCAGAAAAAGTGGTGTCTGTCAATTGCATCGTTAGAA

AGTATGTTTGTACTTGCGCTATTATTATTATTATTATTTTGTGATTAGTACTAATTATCC

TGAAGTGTGTAAAGAAAGGCTATTAAATTTCAGAAGACACCAATTCTCGATACTGTGCAG

AAGTGCGAGTCGTGCTGAAATCTGCACTTGTTGGTGTGGCGAAAGTAGTGGTGCCTGTTG

AGCGGCGTTACACTCCGTTCAGACGCATCGCCAACCTACTTTTTCCTTTCTGTTCGTCAC

GTATGTTTTTCCACCTTTCCTTTTTCTTTGCACGTGGGGGGAGCGGATTAAACACGTGTC

AATATTAAAATAACTCCATGAATGAAGCGGTGAGGTTGGGGGTTGTATCCGTGCGTCTGT

GAGGGGTGTAATCAAAAGTGTCACAAGACTCTTTTCCCACTGGTCTACTGCATTTGGCAG

AATAATTCTTGTTTCTAACAAAGTAGGCAGATGCGATCAAAGATCAGCCCTCTGTCCGCT

TTAGTTATTTGTTACTTTTTCTCTTTGTGTTTCATTTATCGCTTTGTTATTTTTATTCTC

CTGCGGAGGCTCCTCCTCATGTGTCACGTCACATTGATTCTTTTCGTGAGGGTCGAAGCG

GACGCGGCAGGCAGGTTGGGCGCAAGCCCCTCATCCTTTCGTCTGAGCAGCACCATGCGA

CTGTTTAATATGAAACAGTAAGTGATGAAAACGACGAAGAAGTTCCACCGAGTATGACAA

TTGTAGCAGCAGTCCTTCCCAATACCGAGTTTTATTTTTCACGGAATTTCGCGTCCGTTG

TTTTACCAGTTATTTTATTATTCCTCCCTCTGCTGTACTGTACATTCCCTGCTGTTTTTT

TTTCCTTACTTCATTATCCTCTTTCTTTTTGCCCTGTAATATGTGTAGACAGTGGGTCTG

CCGTCGGCATGCTGATGGGAGACCCGCTCTGCACTCGTATTATTTGTTTATTTTTTCGTT

GGGAGTTACACAATTATCCTCAGTGTGTTGTTTTTGATTATTTGTTTTTAC

>Tb927.3.700 | Trypanosoma brucei TREU927 | hypothetical protein, conserved | genomic | Tb927_03_v5.1 reverse | (geneCodeEnd+0 to geneEnd+0) | length=531

AAACTATGGAGTGCATTTGATTTATTATTATTATTATTTAGATTAGTAGGAGGATCGATA

CGTAGGTGGTTGAATTCGGTGTTGTTTGTTGTTTGTTTGTTTGTTTTTTCAGTGACGATG

ATGATGATTATTATTATTATTATCTTTATTTACCTGGTTATTTTTTTCCAATTCTCATAC

ATATATATATATATATATATATATATATTGTTCGTATGTGCAAGTGTGTTTAGTTTCAGA

AACATTTATTAAGCTACAGGGTTGCATGTACATCTATAGGAATGATTGTGTGTGTGTGTG

TGTGTCCCTCCTCCTCTTTCCTCTTTTCTCTTCTCTTCTTTGTTTCTACTTGGGTGAAAT

TCTTATCTTTTCTGTGTGGCGTATTCATGAGGAAATGTTTTATTTTTAGAGCCAAACAGA

ATATAGTGGAAAAAAAATTAATTCAAAACATAAACAAACATATAAATGCTAACAAAATAT

ACATATATAATTATGTATATGGTTGAGAACCTATTGTTTTGTGTGATAGTG

>Tb927.10.2340 | Trypanosoma brucei TREU927 | SNARE domain containing protein, putative | genomic | Tb927_10_v5.1 forward | (geneCodeEnd+0 to geneEnd+0) | length=985

GGACACATACAAACAAACAGATACACACCTTCCCTTCCATCTATTTGGTGTGTTTGTATG

GTGTGTGTGTGTGTGTGTGGAGGGGGGGGGGAGAGGGGGCTTTGTTTTCACCGTAGCCTC

TTAACGTGTTGTGCAATTCATTTTTCTCTTTTCGCGTTTTGCTGTCGTCGTTGTTGCTGT

TGTTGATGATTATGATGATGATGATTATTATTATTATTATTATCCGCATGCTTGTTTGTG

GAGAAGGGTGGTTGCTCTTTTCCATTTATTTATTTATTTTTTGTGCCCACCGCGACGCTT

TCCCCCCCCCCTCCCCCAACAACAACAACAACAACAACCTCAAACAAAATCCTATTGATA

AATGCACTCATTTCATTGTTCACTCCTAACGTTTGTAGTGTATGGAAGAGTGGAGAAAAA

AAGGTAGTGGGGTTAAATGACGTTGTACCTTTTCCCGTGTGATTTTGTTTTTGTTGTGTG

TGTACTGTCGCTGATGCAGTAACTTGTGGCCACTTTTTCCAGTTTGTTATTATTGTTTCA

TATGTTCGGCGCTGGTCATCTCTTTTATATTTTGCCACATTGACCCCCACCCACCACCAC

GCACCAAAAAAAAAAAAAGGAAATGAAATGAAAAGAAAAGGGGAAAAGGAGAAAATGGAG

GAGGGGGAAATACAAATACACACGGATTTTGTGTGAGGGCAAAAGAAACTCTTTCAGTTA

TTCATTCGCGAACTCCGCGGGTGTTTTCACCATCGGTCCCTTCCATTATGATTGTTTCGA

AAGACTGTGGCTGCTTGACTGGTGTTTGATAATTCTATTATTAATCACATCAATAGGAAT

AATAATAATAATTATTATTATTTTAAATTCCTTCGTTGTTTTTATCGTTATTTAAACTAC

TACAAACTTTCACTGCGTGAACAAAATAAACTGATGTGACCAAGCAGAAGTAATTGGAGG

ACGCCACGGGTCCTTAAGTGCTTAG

>Tb927.4.520 | Trypanosoma brucei TREU927 | hypothetical protein, conserved | genomic | Tb927_04_v5.1 reverse | (geneCodeEnd+0 to geneEnd+0) | length=265

GGGATAATGACGGGATACTCGTGTGCACGAAAACTAATAAATAGCGCTTATGAATATATG

TGTTTTTGTGAATGTGCGTATTTTTTGTGAAATTGTAGGGGGGTTTCTCTTATGATTTCT

TTATCTCATACTTCGTGTAGAGGATTATTCCTGATGTTCGAACGTTTCATTTTTTTTATT

TTTACGCCTTTTCCAGATGGTATCTGCTCTTGATGTTTTTGCTCACCTTCCTATTTTGTG

CGTTGGTTTCGCTTGTTTCTGGATG

>Tb927.1.4300 | Trypanosoma brucei TREU927 | chaperone protein DNAj, putative | genomic | Tb927_01_v5.1 forward | (geneCodeEnd+0 to geneEnd+0) | length=2749

ATTGATATGCGTTTTTGGTTTTGAAAATTGCGTTTTTTTTTTTCGGGTGTTGGAAATTTA

GAGGGCATTTCTCGCAGTGGGGGAGGCGCTGCGTACATTTATGACTTCTCTTTTTCTTTT

TTTTTTTTGAAAAAAAAGAGAACACAAACAAAAAGGAAAAGGAAAAGGAAAAGGAAAAGG

AAAAGGAAAAGGAAAAGGGAAAGAGAGGTAGGGCAGAGGAGAAAAAGGTTGTGGTGGTGG

AGGATGAGTTTAGATGCGAAAACAATAAATATTAGGATTGAGAAGTGAGATGCGAGTAGA

GTTTGGATTATTGCGTCAATTAGGTGATAAAACATGTGGGGTGAGGGACGCTGAGGTGTA

ACATGAAACTAAGGGAAGGGGAACTACACAAATACAAGCAATGACATGTAGGCATCTGCA

TTTGCGCATGAATGTAAAAATAAATATGTTCAAACGTGAATGCATGAGGGGGGGGGAGGA

TGTGTTGTGGGGAGTTGAAAGGATGGGATTACTGATAATTTTATTACTCGATGAGCATTA

CAAAAAAAAAAAAGAGAAAAAAGGGGATTTCTTGTATCTTTTTTTTTTCTCCTCATTTCC

ACACCAAAAGGTAGTCAAAAACGTTGGGACGGGATTATGTGCCGGTGGGCTCCAATGCGG

TGCATCATTTTTTTTTCTTTTTTTTCTTTTTAGTTACTTTTATTATTATTATTATAATTA

TATGGCCACAGCCACGGCTATGGTCATCATCATCATCATCACTACCATTGTGATCCTTGT

CGTTTCCCGGATTCCTTTTAAGTGTTTGAACCTTTTCACTCCCCTGTGATGCACTTCTGT

TGTTTGAGTGTGCAGAGGGGGCATGATTAAATAGTTAAAGAATTTTTTTTTATTCTAAAA

AAAAAAGTAGATCAAAGAAACAAACAAACAAACAAACAGAGAAGCGCAAAAGGGATTCAC

CTGTAAGCACCACTGCGGTTCTCCGGGATTTCACATTCAGGAATCGAAACTTCACGTCTT

TTTCCCTTTTCCCCTCTTCTCTTCTCTGTACCACCCAATTAAGGCAAACACATATGGTGT

GTATATTTGCCCATGGTTACATTGGGGGTTGAATGGATGTGGTGTGAGGGAAATGTTATG

AATGAGTAAAGATAAAAGTGTCAGTGCGTGCGTGTGTGTGTGTGTGGCGAGACCATATTT

TCGGAATCACGGTTGGTGGTTTTTGATTTTCTTTTTTTGATTTTTCTTTTTCTTTTTTTG

TTTTTGTTTCTTTCCCTCCTTTTCTCACTCTTTCTTTTTCCCCTGTTTTCGTTATTTGTC

GTTCATTGCTATTTATCATCACCGTTATTATTGCTATTATTTTTCTTATTTATTTATTTT

CAAATGATAGCCTTAAATTGTTTTGGTGCCCCTTTTGTTTGTTCCCGCTAATCCCTCAGC

CCTTTTCCCAAACCATTTATCCCCCTGTGAAATTTCTCACGAAATGACATTAGACACTTA

AAATGTACGTGTGGAAGTTGTCTGTCATTTGTCATTTGTCATTTGTCATTTGTCATTTGT

TGTTTGTATATTTTCACATTGATATGAGAGCAAAAAACACCAACAAAAAAAACAAAAAAG

AAGGACGATGATGAAATTAAGTGAATACGTCACTTTAATGGAGGGATGAGTCATGAAAGT

TTCCCTTTTCCTGTTACTCATTTTTTTTTTTTTCAGTTTTCACCTTCTCCCCGAAAGTTT

TATTCCCGCTTGCGACTGTCGCCTTCCTTTTCTTCTTTTCTTCTTTCTTTTCCTGTTGTT

ATTGTTGTTACGGCATCCAATCCATGTACTAATACTACAACTCGCCGTTTTCTCGATTCT

TTTTTCTTTTTTTTTTTTACTGTTTCGTTCTGCTCTTTTTTTTAAAATTATTTGAAATGT

GCTAATCCTTTTTTTTCTTTTCTTTATTTGTTTGTGAGTTTCTCCCCCCCTGTATTTTAG

CCCCTCCTCACATATATATATATATATTGTTTTACATGCGGTCACAATCAGCTTCACTTT

TACTTCCCTTTCTCTTTTATATTCCTGTAGTTGCACGTTCAATAATATTATGATTATTGA

AATGATTGTTGCTACTATTGTTCTTTCCGTGCTGTTAAAGGCTACGAGGGTTATGGACAA

TTTATTTTTTTTAGTCATTACTGATGTTTAAACAATTTCACTACATAAAATAATAATAAT

AATATAATATTATTATGTTTGTCATTTATTATTTCTCATCATTACGTTTCTCCCTCTTTC

TCTCTCTCTTTCTCGTGTTTCTTTCCTTTCATAAAAGGTAGAATATTCATTGCTGAATCC

AAACCGTCCGTCAGTGTAAAGTATTTGGACATAAACATAAAAAAAAAAAGACACACGCGC

ACACGTACACACGCAGCTGTGTACAGTAAAAGCCGAATCAAACGTGCTCTCGTTTTTTTT

TCCACCCCGTCCTGACTTTTTTTTTTTCGTTGTCATTTTTTCCTCAGCGATCGGAATAAC

AACAACAACACCAACAAAAAAGAAAACAAAAGGAGGGGAGGATTTCTTGAGCAGAGTTGT

GTGACTGCTCAGTGAGATTTTTGAGGTGAACAGCCATCAAAGTTTCAGATACGGGTGTGA

TAAACTCATACGGAAGGAAAGCCTGAAAGAGAAAGAAAAGGAAGGGAATTGACGTTGGTA

TTTGTGTTTGAACAAAGGATAAGGGTGATTTAGAAGAGGGAAAGAAGGG

>Tb927.8.1240 | Trypanosoma brucei TREU927 | electron transfer flavoprotein-ubiquinone oxidoreductase, putative | genomic | Tb927_08_v5.1 reverse | (geneCodeEnd+0 to geneEnd+0) | length=1

A

>Tb927.6.3830 | Trypanosoma brucei TREU927 | hypothetical protein, conserved | genomic | Tb927_06_v5.1 reverse | (geneCodeEnd+0 to geneEnd+0) | length=762

ATGGCAACAGTTCTCCATTTGCGCTTCTTTTGTGGTGTGTCGTGCATGAACGAAACTTTA

TGGAAGTGCGGTGTATTTGTATGAAGATGTCAAAAAAACAGATTCCTTTTCGAACTTAAC

GGGCATTAAGCCATTGAAGTCTATATATATATATATATATGTAACTATTTATGACATCAC

TGTTTTTTTGTCAGTGTTACAGCTGTTGTTATTTTTGCTACCTGAGTGAAAACGGTTGAT

TCTTTCTTTCTTTTTATTGCACACTTTTGTAATAATCCTCGTTTGTTTTATTTATTGGTT

ATTCCTTACACAAAGAGAGAGAGAGAGAGAGATCGTTAAATGTTGGTTAACTTTTGTTGT

CACCTTCAGGTCCATCATATAGATGCACATAAAGTGCTTACGCATTCGAATGGCTAATTG

TAATCGTTTCAGCGTGGTTTCATGCAAAAGGGTGTGTCCAGAATCTAATATCTGTGGATT

TGCCTCTTTTTTTTTTCTTCGGTACAAACGATCCATAGGTGCAAAAAATAAAATAAAATA

AAAAGAGCGGTCGGGTGTATTTTTTTCTTTTTTAAGAAAAAAAATATTAGTGGGAAAGGG

TTTAACTATGCCGTTCTTTATTAGTGACTCGTTTTGACGTGAGTTCTTTATTTATTCTTT

CTGTAAGCGAAACATATGCTTCATTGAGTGTATATATGATGTTGCATAATGACAAATAAC

GCTTCTTTTGAAATAATATTAAAATGACAACCCATCGCTTAT

>Tb927.11.520 | Trypanosoma brucei TREU927 | hypothetical protein, conserved | genomic | Tb927_11_v5.1 reverse | (geneCodeEnd+0 to geneEnd+0) | length=925

AAATGCCTCGCAGTCAATCGCACAAAATGTCCTTTTTTAATCTTAATTTTTTTTTCAAAT

ATCATTCTTTTTACCGTTTGTACGAATGGTAAAATACCGTTTTGTGTTAGTGGCGAAGGC

TGAAGGAGAAGAGAAGGAGGGAGGGGCTGGATAATTTTTTATTGTTACAGAAAATACAGA

TGCTTTTTTTTTTGTTATTAGTTTTTTTCACTGGGGAGTGGATGAAAAGGTAGGAGGAGG

AGGAGGAGGAGGAGGCAAAATGAATCTGATGACTGGAGGAGTCCACCTTTAAAAATGTGT

GGAACTATTTAACCATCATTGTTTCTTTTTTTTTTTTTTGCATCTCTTTTTATATTAGAT

ATACCCTCATAGCTGTTGTGTCCTGTTCGCTTCATCAATATATATATATATGCATATATA

TTATACATCTCGGGTTGTTTGGCGTATTTCTCGTCTCCCATTTTATTTGAGCGCACGTAA

TGTCTCGGAAGTAGCGGGTTGTGTTGCGGCGCGAAGGTCCGTTTTATTATTTTTTTTGTG

AATTAAAAAAAAAAAGATATAAAAAATATTTATGAGTTCAGTTTAACATCTCGATTCGTT

ATTTTTCTTTTTTGAATATCATCTTTTTGTGATTTGCTTCGGCTGGGGTGATTGTTAGGC

ATTGCTTTTAGTTCTGTTTTCCATTAATTGCACCAGTGTAAGGTTGATATTTTACCTTCA

TATACATATATCTGAACATATATTATTTCTTTCATTCGTCTCTCTGCTGCCGTCGGAACT

TCCACATTTCCACGTTAACACTCAACCAAAAACGAAAACAACAACAACAACGCAACACAA

TAATAAGAAACCGTGTTTTACTAGTGATTAATAAACAAAGAGAAATAATTAGGACAGTTA

CAGAAAGCTAACAAGGTATTAAATT

>Tb927.11.2020 | Trypanosoma brucei TREU927 | hypothetical protein, conserved | genomic | Tb927_11_v5.1 forward | (geneCodeEnd+0 to geneEnd+0) | length=372

GTGGTTGTTCCTTAGCTGCTCACTTTTATTCGTTTACCCAACCCCTTGTTATTTTTTTTT

GGTTACATCACGCATTGTAAACATGCACATATATATGTATGTGCTTGTGTGTGGTTTTCG

AATTGCCACCACACACTCGCTGTACCTGGAATAGCTGCTGTGCGGAGAGTTACAGGGTTT

CCAGTCTTACCGAGAGGAACGGGACGGGCGCTCGACAGTAGGAGCGGATATTGGAAAAGA

ATTTATATACCTACTCGAGGCTTGAAGTTGAAAAGTAATCTTGTTTTTTCTTTTTCGTCG

TACTGTGGTTGAGCCCACCGGGGTCAGGGGAGCGCGATTTCTGGCATTCTCAAAATATAT

CTGTTCTCACAC

>Tb927.8.5110 | Trypanosoma brucei TREU927 | hypothetical protein, conserved | genomic | Tb927_08_v5.1 forward | (geneCodeEnd+0 to geneEnd+0) | length=2659

ACCCTACAGTGAGAAGAGATGCCTTGCGCTTCGTTTGTTCACATGACGCATGATCGGCGT

GCTTTTACACTTATATCTGCGAAGCTCGTGTTTCTTCCTTTTTATTTTTTTAAAAAAAGC

TTCAATGTTTTAATGGGGCGGCGATGTGAAACGTGGGGCTACGAGCGTGAGGTGAACTCG

GCTAATTATTATGAGCAGGAGAAGAGAGAAGTGGTGAGGGCGGTACTGCGCGCCGTGAAG

ACATGTGTAATATTTGCACTGCGTGAAATTGAATCATGAAGAGGAATGGAGATGACACGG

GTTGATGTAGAAGAGAGGAAAAAATTTCTAGTAGTAGCGGTTTCGTAGTGACGAGTATAG

TTAATTTGTGTGTGTATATATATATATATATATATATATGTTTGATAATACGGTTGTGAG

GATATGCAGTGACCAGCAAAGTAATAGAGGTATAAAGTGAGGCATAAGGAGGGCAATGAA

TGACTGTCCGATGGTGGCCAAACAACAACGACTGCAACAATAAAATGGAAGAGAGGATTT

GGTGGGGAGTCTCACGTGTCATAATGACAATTGGGTACGTGATTGTTTACTTGTGTTACA

TCACTTAAACCCACACACCCACTCTTTCTACCTTTCTGTCTGTTTTTCTAGTTCCCAAGG

AACTAACTTACCCTTTTGTTCTTTTTTTTCTTTTCCTCCTTTTTTTTATTTTGGCCATGC

ATGTGGTTAAATGCACTGCTCCACATGCAAAAGGAGCCAATAGCCGCCGTTGTAACGTTG

TAGTTACCATTCTTGTCGTTATCATTATTACCAAGGCTTCGACCGTCGGGTTTCTCTTTC

TCTCTTTTTTTCTTTTTGATCTTGCCCTTGCTCTTGCTTTTGCCTTTGTTTCTGTTTCTG

TCCTTTGTTTTATGTTTATCTGTTTGCATTCCTACGAACACAAATAAATTGCATGCACAA

ACGCATTATATATATTTATGCACATATATATATGTATATATTTGCGTGCTTGTGTGTGAA

TTTTATTTCTATATTTATTCGTTTACCTTTTTTTATATTTATATTTCATGTGTTCACCGT

GTGATTTTTACCTGTCGGAATATGCAACTGGATGAATGTTGTTACAATTATTATTGTTAT

CATTATCAATGCTATTATTAATAATATGACCACTACTTACTTTTCCACTTACTTATATTG

CCACGAACTTTTTGTTTTTTTTTTTGGTACGCTTAACCGTGACTCTGCACCACGAATAAA

ATGAAGAGGTGAAGGAGGGAAGGAAGGAAGGAAAAAAAAAAAGAAAAAAGAAAATAAAAC

GAAAAGAAGGGGAGACGGCCGTGAGGTGTTTTTTTTTTTTGGGGAGAAAAAAAAACAATA

ATAAAGAGGAAAGAGGAGAAAAGGCATTTCCTTTTTGCATTTGTGGTTTCGTTACAATGG

CGTTCGTCCCTTCTTTTTCTTTTCTTTTCTTTTTTTTCTTAAATGTTCCTTTTGTTGTTA

CTTATTTTTGTATTTGTGTGTGTCAGTGTAAATATATATATATATATATATATATATGCG

TGGGTGAAGGAAATGGGAACAATTTTCACATGAAGCAAAAAAATAAAATAAAAAGGAAAA

AAAAAGGAAAAAAAAAGAAGAAAATTGGATGAGGAATGAAGGGGAGGAAAGAAGAAGAAA

AAGAAGAGGAGGTAAAATAATAATTAAAAAAAAAAAAGAGCACAAAAGACATAATGAAGC

AAAAAAAAAAGGAAAAAGGAAAAAGGAAAGGAAAGGAAAAAGTGAAAATGAATATATGCA

TGTGTGTGTATATATATATATATATATATATATATATATATGTAAATATATATATATATG

TTTATACGCTGAGACATGTTATCAAAACGAAACGAAACAAAACAAAACAAAAAACCAAAA

AAAACAACAACAACAACAACAACGAGTACACCTTATGGATAGCAAAAGGGGGTTATTATG

ATCATATAAATATGTGGTCTTTACATACGTGTGATTATTATATACAATTATATTCAGAAT

TGTTCATTTATTCGCATCACACATTCATTCATTCATTCAAAAACATATACACATATGAAT

TTTGTATGAATAAGCACACTTATGTACTCATATAACTTTTCCTCTTTGATTTTGTTTTAT

TTTCTGTCTTATCATTTAGGTTGCTTCCGTTTGGTATTCTCTGTGTTATATTCCATTTAT

TTTTCTAAAAAAAAAGGAAATAATAATTTTTTTTGTTTGTCTCACTGGTCTCTATTACTT

CCTTCCATTTTTATGCTTTCACAAATAAGGCGGCTCTGACTATTATTATTGTTATTATTA

TTTGGTGGAAAAAAAGGCAAAACAAAAAAAAAAAAAGAACAGGAGTAAACACCAAATGTA

AGCTACCAGGTGCGAAGGCACAAAAAAACAAAAAAGGAAAAATAACAGAGGGAAGGAGAC

AAACAACCGGGGACACATACAAAAAGGAGAAAAGAAATGAAAAAAAAAGAAGAGAAATTA

TTACTTACACATGGCTTCACATGAAAACATGAGAATTGTATACATGATCATATATTTTTG

AAAAAAAAAATGCATGAAAAACACTTACACTTATATGTTTACAAATTTATGCTAATGTGT

TATTGCGGCCACTAAAAAG

>Tb927.11.14240 | Trypanosoma brucei TREU927 | WD domain, G-beta repeat, putative | genomic | Tb927_11_v5.1 forward | (geneCodeEnd+0 to geneEnd+0) | length=87

AAGTTTTCATTTGGTTACTTCTTAACACTGCATAAGCGCGTTGTGGGGAACGTCACAACG

CCGAGATAAGCACGCGTGCGATGTGTG

>Tb927.10.5310 | Trypanosoma brucei TREU927 | SNF1-related protein kinases, putative | genomic | Tb927_10_v5.1 forward | (geneCodeEnd+0 to geneEnd+0) | length=1

A

>Tb927.9.12680 | Trypanosoma brucei TREU927 | hypothetical protein, conserved | genomic | Tb927_09_v5.1 forward | (geneCodeEnd+0 to geneEnd+0) | length=16

AGAGAATGAAAGAAAC

>Tb927.11.8170 | Trypanosoma brucei TREU927 | Mitogen-activated protein kinase 8, putative (MPK8) | genomic | Tb927_11_v5.1 forward | (geneCodeEnd+0 to geneEnd+0) | length=1889

ACAAGAGTGGACTATACGGGCGGAAGGAGGCAACGACTTGAATGTTTTTGCTTGCTTTCT

TCCACTCCCCGTTTTTGTTTTTACCGGTCGGTATACCCATTGTTGTACCGGTGTTAAACT

TTGCAGTGTCTTCTTATGACTGCAGAATCGAACGAAAAAGCGAAGCAATGGAAGAAATAA

CTAAAGGAAAAGGGAACCCTGTTGTGATAAACATGAGCGCGCCTGTGGCGGAACACCGTT

TCGTTTCCTCTTCATGGCTGGTTGACTTTTCCGCTCCCCCCTCCGCCCATTTTTACCCCC

TGATCGTGGACTCACCGAAAACAGGAAAGGAGAGTAAATCTCTTCCTCCTCTCTCAAATA

TGTTTATGTATATTTGTTGGTGTGCCGACTTCTGTTCTTATTTCGCTGTTGATTTCTTTC

CTACGTAAGTAATATCTGATCTGTGCATGAGACAATAGCGTTTGATGGATTAAAGAGGCC

ACATTTTTTTTTTCGGTTTCGCAAGCCCACACATCTGATCCCAGCGCCTCTGTTCTTGAG

GCACCTCCCCGCTGCATATATTCATTACACGGGACAGTTGAACGCTACCTGGTTTGCGTG

ACATTTGTGATTTTAACGAAAATCTCCTCGTGTGCATTTCCATATTGTGCGGGGTGCGTG

TGTGTGTTTGTGTGTTTGCTTCCCTTCTCGTGCAACCGTCGATTAAACTCGCATGATGGT

TGTTAAAAGGAGGGAGCGAGAGAGAACGAAAGCAAGAAGTAGAAGTGGTTGCTTTTCTCT

CGATCAACGCAACAGGACGGTCCTCTGCCTCTCCATAAAGCGGTACCCACTTGTTGTACC

ACATCCTTTATTTTTTTTTTTCCTTTCTCAAAGGGAAAGAACTCCAGCCTCCTACCTTTT

TTTTTCTTTTTTCGCAACTGATATCCCTCTTTGGTGGATGCGGCCGGGCTAAAGCGATCC

CCTCATTGGTTCGTTCTAGTGTGTATGCGCATACGAACGTGTATATGTCTCTTTTTTTTC

CCCCTCTGCGTTTGCCATTTGTTCTGTTTGACTTCACCTCCATTACTCCATCTATATTTT

GGTTGAAATGCGTCCAACGATGGGTAAAAGAGGAGGGAAGGAAATGCTATTCTTTATGAC

AACCCCTATTTCTTCTTTTGGTGTTTCTGTTGTGTCATCTTTTTTTACCCCGTCCTACTT

GCTCCACCCGGCAAGTCGGCAAGAAGTGTATGCGTTTTTGTGTGTTTGTGCTTAATGAGG

CAGATCTGATCTTTCTCTTCTACTTTGCCTTCACCTTTACTTTTGCTTTGTTTCGTTTTT

GTTATTCTTGACTTTTATTCTTCCGTCTACACACCCCTCACACGTTGACAATCGTCTTAC

CGCAAAATGTAGCCGGTAAAACAACAAGTAACATTTGTGTTACAGTCACTTTCCATTTTC

TTTTCTTCCCGTTCTTTCATTTTTTCCTTTTTGTTGGCGTTTCTTTTTCTCCTTTACAAG

CCCCTCCTTCTTCCCTGATTGGTGTTATATATTCACAAATATTGTTATTATTATTATCAT

CTTTTTAACCAATGTTTTTGTGGCTTCCCACTTCCATCATTGAGAAATTATATACCTTTA

AGTTACTCGAAGCAGCTTCCTGTGTGGGATCTAGCGATGGTGAAAAGCCCAGAAGGGGGA

AAAAGTGAAAGTAAGAAGAAAAGAGGAAAAAGCCGTATTTTAAAGGGTGTGACACAGGTA

TTCTTGACTTCCTGCTTCCATTTATTTAAGTGTTTGTGTCCCCAAAACTTCGGCTGGAAG

GTGTTTGACAGTGGCGATACTTTCAGTCCATCCTCCCGATCTCACATATCGAAAGACATG

TGGGGAAGGGGGGACTAATGATGAAAATG

>Tb927.11.3050 | Trypanosoma brucei TREU927 | hypothetical protein | genomic | Tb927_11_v5.1 reverse | (geneCodeEnd+0 to geneEnd+0) | length=497

AACCCTTTAGTGACGACATCATTCTAATGTGTCGTAATCTGACCTCATTGCCATTAGTGT

TGGTGCGCATCCACGAGATTGGGAATGCGAAGGGAACTGTCACCTTCCAGCAAAATGTGA

TGGTGGTTGGTAACTGTCGTGCAAGCGGGCAACTTACCCGCCGTCTTTTCGTCAAGACGT

TCAAAATCTTTTTTATCTCTCGGTTTCATTGTGTGTATGCATTGATCGTATATTTTTTCA

GTTAGTTTACCTTGTTTTGCCCTAGGAGGGTAACTCAGGAGGGAAACGAAATTCTCTCTC

TTCATTCAGTCATTTATTCATTCTTTTAGTTGTCCGCATGCTTAGCGAAATAAGTAAGCA

TCCCCTTACACATTGTTTTTTTGTTTTGTTTTGTTTATCGAGAGGGTGCCGCCATACGGT

CTTTACTCTTTCTGTTATTGTGTGAGCTGGAGGCGAATTCATCTTTGGAGTTCACATCCT

TTAAGTACCCCGTCTAC

>Tb927.11.6890 | Trypanosoma brucei TREU927 | DNA repair and recombination helicase protein PIF1 (PIF1) | genomic | Tb927_11_v5.1 reverse | (geneCodeEnd+0 to geneEnd+0) | length=963

GAATCGAATTAAAAACTTTGTTTCTTTGTTGTTGTTTTTTTTTTAATTTATTGTTTGTTA

CAGTTCTTCCTTACCCTTGTATTTTTATCGTGTTTTGTCTAATGTTTTTTTATTATTATT

ATTTTGTTTTAAAGGATAAGTTTCTCCCTTCCCTTTGTACATTTCCCCACCCTTTCTTGT

TTTTTGTTTTACTACACCTCTTCTTCCCTTCCCCTTACAATTTAGATGACGGACTGTTTC

TTTGGTGGAAGTCCAACATATATTTCCTTCCCCTCTTACGAATCACACACGCACATATAT

ATTTATATATTTATATATGAATATGCAAACACAAGTATTCCCCTCTTCGTTTTTTTTATG

TTTTCTTTTATTTTATGTTTTATGTTCCACTTCGGAGCCGTTGGTCGTATATTTCTTCGT

TCATTATACATTTCAGAAGGACGAGAGAACAAAAGAAGAAGAGGGAAGGAAAAAATATAT

ATATATATACGCCTCAGGGATCCGAAATATAGAGCCTCTACATATTCTGCTGCATGAAAC

GGCCGAATTATCTTTTTTATTTTTTTTTATTTTTATCTTTTTTTTAATTTTTTATTTTTG

GTCTTGTGTTTGCGCGTGATTCAGTTGATTCCTCTTTATTTTTATTTTGTTTTGTAGATT

GGTAAATGGAACAGCCAGAGGATGTGTAAAAGGACCCCATAGACACATACACATACACAT

ATATATATACATCCACATTCATTCATTCACTCACTCACTTTTACACACACAATTTAATCG

CAAAAAAAAAATAATAAATAAATAAACACAAACACTTTCAATTGTTTTTTTTTCAATTTT

TCTCCCGCGCGGCGCGAACCGTTTTTCAATACGATGAGCGCTGTGGCACCATAGACGCAA

AAAAAAAAAGAAGTTAAAATACATCAGTGAGGTTCTACAATTGGCGGAGAGGAAACACAA

AAA

>Tb927.11.15720 | Trypanosoma brucei TREU927 | hypothetical protein | genomic | Tb927_11_v5.1 reverse | (geneCodeEnd+0 to geneEnd+0) | length=2235

ATAAAAGGCACCAATGATAATGAATAATAATTTCCATGAAGAAGGAGCTACAGAGAAGGG

GGAAATATAATAAATAAAGATTAAATTAGTCACTTTATGATTGCAGGAAAGAAAGAGAGT

CTTGTGTTTTTTTTTTTTTTGATTGTCGCGGGAAACTTCATAAATCAAAATAAATGGAGG

AAATTAATTAACTTTGTTTCGTTTCAGCGGGAACCAAAACGGAAGAACAATTGAAATAAC

AACTATTATGTTGGTAGCAATTAGGAGAAAGGAGAGAAGGTAAAAGGAGGTGCTTTGCAA

AGGATGAAGCGGCGACATGTATAAATAAATATAAAACATCGCATCAACTTCTCTATATCA

AAGGAAATCATTTACTCAATGGATTCTCAACGAAATCATGATATTGACCTGATCGTTTAT

ATATATATATATATATATATATATAAATATATAGTAAAATTCCTTGTTATTTCACTCTGT

TTTTTATTCTTGAGATCAGATATTTAAATTGATTATATGTATACGTATATATTTATGCAT

TGTTACAAGGGTTAGCGAAATAGACGAACTGACGCACGGTAATGACAACGTTTTCCGTAC

GAGTTAGCCATTGGTTGAAATGATACTTGAACAGAAAAAAAATACATATATATATTATTA

TTATAATAATAGAAAAAAACAATAGAAGCAAAATAAAGGGTTGAAACAGTTACTTTCTGC

GAAAATAAATTTATATGATGATTCGTGACGGAACGGTTGAGGGCCAGTCGAAAGAAAGGA

AAGGAAGGAACGAAGGGGACTAATTGATTAAACCTTTATGTTTTTGTTTATATAGTTACT

TATTTGTTAGTAAAGCACCCAGTGAGACGTCTATCATCAGTTGCAGGTTATTATTTTTGT

TCTTATTATTCTTATTTTTGTTTGTTTTACGTCCTTTTTGAATCTTTTATTTTTCCATCT

GGTGCTCTCCTTACCTCCTGCGCTCCCCCACCCCCTTGATTTTGTTTCTTTCTTTCTTGG

CTTTCTTTTTTTTTTCTTTATTCTCCTTGTATTTTCCTTTAGTGTGGTCTTCTCAATCTC

GGCTAAATATGTGCAGGAGATCCGACCTTTCGAGAAAAGCAAAGGAGGTAAAAATTGAGG

AAATCCCCCCCAAAAAAAAAAAAAAAACTGTTGCTACGGCACCTGTTGAGCTTTCTTTAT

TTTATTTTGGGTTATTTTACTTTTCTCCTCCGGCAACACACACGCGTTTTGTTTGTGCTG

GTACGTGCACCGTCATTTTTCCGCACTTTATTTTCCGTTAAAAAAAAAAAAAACTCAATT

GTATCGGTGCAAAGCGGCTGCCCTTTTTTTTTTTTAATCCCATTCGCCTATTTCCCCCTT

CACCTCACTTCCTTGCCAAATCGCGTGGCAAATCATTATGTGGTATACGGTAACGGCATT

TAAAGGTGTAAATAGGGTGGGAAATCAGTGAATGAAAATAATGAATGGAGAAAAAAAGAA

AATAAAATAAAAGGACAAAACAAACTTTTAATGTGCGCATAGGAGTGAGTTAGATGTCCT

GAGGGTCGGTGGAAGGTAAAGAAAACGTATAATACAACAAGTTTGTTCCATTTTTTTGTT

TGTTTGTTTGTTTGTCCTGTTTATTTTTTCTTTTTTATCTTTTAGTTGTTGTACCTTCAC

TGAGTTAGGTGAAAGTGAATTCCTCATCCTTCCTCATAGGAAAATTTCCGTCTTATCTTC

GTTTTGTTGCTGCAGTTGAACACAATGATACGAGCCCTACGTCACCCATCTCCTAATATT

CACTTCATATTTCGCAGTGCCCCCCTACCGTTGCTGACTATTTCTTTTTTTTTTTTTGAA

CAGTTGTACTCCCCATAAATGTAAAGGAGGGGAAAAAAAGAAAGATAAATGAGAAACATA

GAACATACTATATATATATATATATATGTAACCGCCCAATTTTCCATTTTTTTCCTTTTT

TATTTTGTTTGAAAAAAAAGTTATTATCTCTTTTTCCTTTTGTGGGCTTGTTGTGGATTA

GTTGGAAGGGGGATGGGCAAAGTTGGACACTTTTCGCCAGAAGTTTTACGAAGTGCTAAG

AAAAAAAAGGGGTGAGAGAAGCAGGCGCCGTTGCGAAGCATAATTTTATACACCGATTTG

TAAAGGGTTGATTCCAGTGGGCTGAAGGAAAAAGCTTTCGTTGTACTAACAGGCTAATTT

TTCATATATATTTTT

>Tb927.10.14260 | Trypanosoma brucei TREU927 | AP-4 complex subunit sigma-1, putative | genomic | Tb927_10_v5.1 reverse | (geneCodeEnd+0 to geneEnd+0) | length=670

GGAAAGGGGAGCGACAAAACACGAGACTCGGGCTGCCTGTGTAGCATTTAATGCAAGTTT

TCCCGTATCATCTGTTCAGCTTTGTACTCTTGCCCAAAGATGTGTCTTCGTGGCTACGCA

CAACTTGCTGAGCCGGAGCCCACTACTCGCGTATTTTCCTACTTGTGTCACATGGTACTG

TACTGGCGTGCAATTATTCTGTCGGTATGCACTACTTCCTAAGGCTTATTTTCCCTCTCT

AGCCCCTTTTCAAGCCGGGAGGCTAGAAAGTTGCAGCAGGGGGTAGTAAGGTTGCGAGAC

TAAGAGAAAGCGAGGACGAGGGCTATCACTGTTTACTCGCCTGGAACGCGCTTTCACGTC

GCCGAAGTTGTTGAATCACAACTCTTATTATTATTATTTTTTTCGGTTTTATTTTCCTGT

ACATTTACGGGCACACGTACGTTGCAACGCTCCTCCACCCTGAACCCGGAGTCCGATGCC

TGCACTGTTAATCTTCAAGGATGAGAAATGCCTTAACTTCTGTTCCGCTCTCTTCGATTC

CAAGGCGGGGTGACACGAGGAGGTTGGTTAGTGCTTGCCGACGCAAACCGTTGCGTTAAA

TGCAAATAGTTTTGTTGTCACCGAGAAAGTGAGTGAAATATAATAGAGGCACAGAGGCAG

CAACAACAAC

>Tb927.1.1840 | Trypanosoma brucei TREU927 | conserved protein, unknown function | genomic | Tb927_01_v5.1 forward | (geneCodeEnd+0 to geneEnd+0) | length=1991

GAAACGACAGGTGAAGGAACAGTATTAAAATAGAACGAACGAAAAAAAAAAACAAAAAAG

GAACAGAGGGAAGATGTGTCTGGGGGGGAGAGGGGGAAAAAAAAAAAGAAAAAAAAACTG

TGATGTCAACCGCTGGTATTCCCAGTTTTGCTCACAGGATGGTATCAAAAAAAATCACAC

ACGTATGTGTGGCAGCAGAACCTCCATGACTTTCCTTTCCTTCCTATTCCATATCGTATA

TATATATATATATATTCTTCTTTCTTTTTGTCAGCCTTCCCGCCGGCATACAACTGGAGC

AAGTTTACTTCCAGACCACTTATTATATAATCGACAGTAACATCCCTGTATCCGTTGGGC

ACGTGCGTGCCTGTTTTTCATGGCGGGAACAGAGGGGGGAAACAAAGAAAATAGTGGTAA

TAATAATAATAACAATTATTATTATTACTATTACTTTTCTTTTTCATTAAGGAGAACCAG

TGGGAGATGTGTACAATATTGGGGAAAGTTTGTGGTTTGTTGTGAGCGCTGTGGATGGCG

GGAAAGTTTTGAAGTCCAACAAGCGGGAGGAAAGTAATGGGTTGTTGAATGTGACTTGCA

GAGGAAAGAGGAAACTAAATAAAATAAAAGATATAAAATGAATTAAAGTGAAGTGATGCC

ATGTGATGTGATGTAAAAATGAAGAAGAGTAGAAGTTAAACAGGAGAAAGGTAAAGAAAG

TTTCTTTTTTTTTTCCATTGGTTTGGTTTGTTCGTTTTGGAGGGTTAAGTGCCAGCAAAA

GGAAAAGTAAAAGAAAACAAAAGAGGGAGAACGGTGACTTTTAGGATAGGGGTGATGGGG

AATTACATTCAGCAAAAAATTAAAAAAAACAGAAATAAAAAAAAAAGTAAAAGTAAAGGT

GAATGTCTGACATTACTCCGACAGCAGGTTCCGCTTTCTGTTCCCACACTACACCGTCTT

ATAAAAAAGAAAAAATATTTTTATACTCAACACAATGCAGTCATCGGATTTTCCGTTATT

CTTATTCTCGTTTTTTTGACTTTTTTTTTCTTTACCCTTTTTTATTTATCTATTTGACTT

CTCTTCGTTCTCATGTGGCTTGTGTATTTAAATTTCGGCCTGACCAGGTCTAAAAAAAAG

AAGAAAAAAGAAAGAGTAAAGGATTAATTTCTCATGTCTGTAAGTGTGTGTTTGTGTGCC

TGAGCCGATGTCTTTTTAATTTTTATTTTTCGATTTGGCATTCGTCTTGAAACTATTGGC

TACTGCACGTGACCAACGCGCAAAACTCTCTCTCTCTCACACGCACACACACACACACAC

AAAAGAAAAAAAAGGTTCAATGGTAAAAAAAAAAAGAAAAAGAGAGAAAAAAATAGGAGA

TGAGAAAAAATAACAATGAACAATAATAAACGTGCATTGCATCATTATTTTGTACGTCTC

GCTTTTTTTTTTTGTTTTTGTGCGTTCGGTCATCCTCCTGTTGTTGTTATTCGAGAGGTC

AAATGATAGGAGTTGGTGATGGTAACAACAATAATATAAATAGTAATAATAATAATAATA

AAAATAATAGTAACGATAATAATAAAATTGATGATGTTGAGAATTTGATTGCGCTGGTAC

TTGTAGCTTATTTATTGTTATAACCGGTTCTGATCTTCAACCAACCACCCGCTCGTGGGA

GGAATCTCACACGTCAAATATTTAAATGTTAGTACAAGTATATTTAAAAATACAAAATAT

ATTGAATGTATAATATATTCTTTCTCTCTTTTTTTTCTTTTTGTTATTTTGTTTTGTGGG

AAAGGGTGTGATATTCTGCACAGGGATGAGGATCGTCTCCTCCTCTAGTGCTTTGTTTGT

TTGTTTGTTCGTTATCTTCTGTTTTTAAACTTCCTTTTTCTTCAAAAAAAAAAAAAAGAA

AACCCCTTCACGGTCCACTATTTCTTTCATATGTTGTTTTCTAAGACTCTTTCCTTACCT

CTTGCTTCTTC

>Tb927.3.850 | Trypanosoma brucei TREU927 | hypothetical protein, conserved | genomic | Tb927_03_v5.1 reverse | (geneCodeEnd+0 to geneEnd+0) | length=1410

ACCATTGTTGGTTCCCTTCGACTGTCGTCGATTCAATATGGAAAAAGTCAAGACACGGGG

GAAAAAACTGTAATTCACTTATCGCTGTGAATGACTGATGGGGAGGTTGATTGCAAGGTG

GAGTACAGGAGAACTTTCTTTTCTTGCTGAAGCTTGTTCGGACGTGCGAGGCGACAGCCA

ACGTATAGGCGATGTAATCACAGCAATCTCTTAAATTCGGAAAGGCGGCATTAGCGCACA

TTGTCAACGTAAGTGTTATCCCTATACTCAGCAATTGTATTTGCTTATTTTATTTTTTGT

ACATGATATTGGTATCGTTAAGCAACAGAAGGTTGTTCTTCAATAACTTTTCTAAATACG

TATGGGCACTGTATTCCCATGTTCTTCATGTTTCCAGGTGTTGGCCGACAGTTTTTTTTA

ATGCTATTTTTTTTTTTAAAGGGGGTTTAGCTAATTCTATCCCCTGCATCATATGTGATT

ATTTGTAAAAAAAAAAAATGGGTTTTGCTGTCATAATTATTTTTTGCTTTAACTCAGTCC

GGAGAAAAGGTGGTGGTTATTGCTGTGTGTCACGGACACGATGCCTAGGGTGCGAAAGAG

GAATGGGTAAGTTGGGGAAATTGGGTCATGCAACGAGAAAAGTTGTTGCATTGGGTAACA

CAGAACGACCCGTATGCTAATAGTAAAAATCATCTTGTAAATGTACGATGTTCGATATCC

ATGCCGTTTCCTGCTTTTGTGTTAGTGATCATATTAAAAAAAAAAAGAACAAACAAAGAA

GTAAATAACCCCAAGTTTGTACCAAACTCCAGTAGTAGGACAGGAGTTAAAGGGCACCGA

AGATCGCAAGTACTTTCCACAGAAAGTGAATCTACTTACATTAGTCCTTCATATTAGTCA

GGTGGCAAATGTATGTAAGTTGCTCGTTCACCCGTTACTCTTCCCATTTGTTTGACCCGC

CTTGGTCGTTACTTTAGCCTCTGTGTGCAACCCTCTGTTCTTTTAGTGCTATTTTTGTAT

TCTTCCGCAGTACCCGTCTATTATTTTTATTATTATCATTTGTTGCGACCTTCATTATTT

GTGACGGACCGTCATTGCATTCTCCCGCGGTCCACATTTTGAAAGCGACCCGCTTCAGCG

AATGGCTTGTATGGCCACTGTATAATGGCGGGTGTAGGAAAGTGGCTGGAGCTGTAACAG

CACACTCCACCCACTCTGGGAGGGGAAAAGAGAGGAAAGAACTGTCTATACACTTAGAAA

TATGTGCATCGAATGAAGTTGTGGCGAAGTAACCATTCTACCTGGAGTGCTGCCAAGTAC

TTCGGCTGTGTGTTGAGATCATTGTTTTTATTCATTTAAAGGCTACCGCTCGTTGCCTTA

ATAGCTTGTGGTGCAACTGTAACCAACTTT

>Tb927.6.3530 | Trypanosoma brucei TREU927 | hypothetical protein, conserved | genomic | Tb927_06_v5.1 reverse | (geneCodeEnd+0 to geneEnd+0) | length=583

AAAAATGCGTGGGTCCGGGAAGATTAGTACTAGTCTTTGCTTCTGTAACGTTTATGGATG

TGCTTTCATTTATATGCTTAGCGCCACCTTGAGCTTAATATAACCTGCCATTCCGCTGAA

ATACCCAGCATGTTTTCACAATATTTGTGCTATACCTACACAGTGTAGGGGAAGCTACGC

GTGACATAAATATTAGCTGCATCTGAGGACTGAAAATTGCGCATCATCTAGTCACCATGA

TTGTGAATAAGTGGAATAACAACGAGTGTTAGGTGAACTCTTGATTACTGCAACTGATAC

CGTACATTGTTTCAGTTGTATTCTCACAGTACCACGCTGTGCTTCAGTTTCTGTTATTGG

TATGAGACCTATTATTATTATTTTTTTTTTTTTGGGGGGGGGGTAGCATGTCGCTGTTCG

AATGCCGCGCCTCCTCGTGTTTAACTGCGGACGATGTTATGAAATTGAAATTCTCTTTTT

GTGGGGAGTGTGGGTGTTCGGTGCATCATCAATAGATTAACACTATCGAAGTAGCGGAGG

GGTGATGGTTCTCCTTTTACTTATGATGTCTGTACTGACGAAG

>Tb927.10.12760 | Trypanosoma brucei TREU927 | zinc finger protein family member, putative (ZC3H36) | genomic | Tb927_10_v5.1 reverse | (geneCodeEnd+0 to geneEnd+0) | length=3004

AAGATGAATATTTCAATCCATACAGCAATGCATACATATACACACAAGCACACAAACATT

TATATATATATATATATATGTTTGTATATGTGTGCCTTTTTATTTTCTCCTTCTCTATTC

CTCTCTCTTTCTCTTTCTTTCTCGTTACTCGTCTCTCTGCCTTTTGACTTCCCCTTTGTG

TATAACCTATGTGCGTATAAACAAACGTACAAGCATATCTTTATATATATGGTTATGAGT

AAGTACAATAAAATATACGAGTGCACGTATGAATTGAAAGAGAAATACGAAGCAAGAGGA

TAACTGATGAAGGAAATATGACAACGGAAAGGGGGACATTGCGTGGGTTTTGGAGTGGAG

AGGGGAGACAAGACAGTGGGGGAAAAAAAGGGGGTGGAGGAAATAATATAAGATAATAAG

TATATATTTTTTTATTATTTTATTTATTTTTATTGGGATTGTCTTTAAGTTTTTTTTTGT

CTTTGGTTTGTCTTTCTACTTATTTATTTGAGCGGATTCATGGGATGAAGAAAGAACTAA

ATTTTCTTTTTTAAAAAAAAAAGGGGGGGAGGGGTAAAGAGGGCTCTTCATCGGCGTGTA

TGTGCATACATTTGTGGTTTATGTGAAAAGGAAAAAAAAGATGGAGAAACTTGAATATAA

GGCAAAGAAAATAGCCACGAACTATACGACTGTGGGGAATTGGTTTTTTTTATATGTTGT

AGAAGGGTACCGATTATGAGACATATTTGACTTTTTGAGGAGCGTTTTATTTGTTGAATT

ATTACTGATATATTGTTTTGTTTTTCTCCCTCCATCCTCCTTCTTTTTTTTTCTGATTTT

TGTCTTTTTTTCCTATTGGTTTTAACTCCTTTCACATTATACAACCATAATTATTGGCGT

TGTCGTTTCGGCATTTATAACACAAAAATTTTAAGAAAAATAACGTGCCTCGAGGTCAAA

TATGAAATGTAGGCCAATGACGAGAGCTTTTCCGCATTTCCCTCCATTTTTCTATTATTT

AATTAACTTATTTTCCTTTAAACATTTCGTCGCTTCCATTTCCGAATGATAATACGCATA

ACTAGTTTCAACCTTACGCCCCCTCTTTTATTTATTTATTTTGTTTTGGTGTGTTGAAAA

CAAAAGAAAAAACATATACGTAACATTACTTGTTCTGTGTCGTGTTGTTTCTTTTTCTTT

TCTTTTTTTTTCTTATCGTTATTTTTATTTCTCCACTTCTACACGTATGACCGGGGTCAA

ACAAGGCAAAACACGACTGACATGAGTTTCCCCTTATGTACACCTCATCTCATCATGTTG

TGATTGCTTTTTTTGTTTATTTACTTATTTTCTCTCATTTTAGTGTTTAATTATTATCAA

TATAATTTCCTTCGACGCTTCTTTTCACTACCCCATTTTTGAAAGAAAAAAAAGGTAAAG

AAAAAGAGGAGGAAAGGAAAGAAAAAAAAGGTGGTATTTTTTAAAAAAAGAAAAAAAACA

AAACAAAAGAAATGAAATGAAATGAAATAATGTGACACTTGAGCTTTGTGTTTGAATATT

TTTTTTTGTTTCCTTATCGTCTTTGTCTTACTCCTCCCTAAACTATTTTTGTTATTTTTT

AAAAAAAAAATATGTATAAGTAAATTAATGTATATAAATTTATATATGTGTTTGTTTAAG

TCATTATATGACCTGACACTTATATATGTTTGCATTTTTATATGATCTGACACTTGCATA

TATGTTTATATATTTGCATATCCATTTATATGTTTCCCCGGGCATGCATCATCCCTTTTT

CTCTTTTCTTTTTCCCCTTCCCTTCCCTTCCCTTCCTTTTTTGTTTCTGTTTTTCTTTTT

GTTTTTACCCAATTCCTTTCTTTTAAACTTTATTTTGTGCGTATCATCTTCCGCTAACGC

ATACGTGTGGTTTTAAAAACGCTCATATCCCTCCGCCAACTCCGAAAAGCAGTGTTTTTG

TGTGAATGTATGAATATATAAATGTTTGCATTTATTTTTGTGGGATTATTACCTTTCATT

ATATACTATAGCAACATTTACAAGAAACCTTTAAAATGAAAAAAAAAAGAAAAAAAAGAG

AACAAAACACGATGCTGATGGTTCTAAAGAGGGTGGGGAGGCGGAGCAACTATTCCCTAT

GCCCCAACATAATTTCTCTCCTATTTTTGCATCGTTCAACTTGCCAATAAATACGCAGCG

AACAAATAAACATTACTTTACTCTTTTTTTCCCTCTTTCTTTAAGTTTTTAAATGGGCCA

CTAATTTACACACGTTCTCATTTTTGTTGTGTCGACTTTTTACCCTTTACTTTCATATTT

TTATTACTTTAACTTAGTTTCCCCTCTTGTTTACCTTTTCCCATTGTCACTCCCTTCCCT

TTATTATTATTATTATTATCCTCGTTTCTCCCCTCCCCACATAATTTTCCAATCCTTCTT

TGATGCCAAAGCAAAAGTCAAGTAAGATAAAAAGAAAAATAAGACAAAACAAGAAAAAAG

AAAAAATAAATACGAACTAACGCAACCACTGACACGCCTGATTTCACTGTCGCGTATTTT

TATGCGCGTATTTCTGCGGCGAATATGTGGGAAGCGAGTAGAATTATGTGAGTTTTCATT

ATGCCAATTTATTGTCTCTTCACCTACCACCCGCACATTTTTCATTACTTATTAATAATA

TTAATATATATTTTTGTTTTCCTTCAACGTGAATACGGGCCACGGAATTTATAAGTTGGT

ACCTTTTTATACTCTCTAATCTACGGGAAGTGTTTGTTTGTGTGGCTGTTGTTTGTCTGT

CTATTTTTATTTTTATTTATTTTTTTCTTTTTGCGTGTGTGGTGTGTGGTGTGTGTGTGA

AATCAGCAGCACTGCCGGTAATTTTCATTCCGTTCATTCGGGGCCTCAACGCGTTGCGGT

CGCTTCCACCATTTTTATATAACTCGAACCCCTTACTCATTTTATCTTTATATCATAATT

TTTT

>Tb927.5.3760 | Trypanosoma brucei TREU927 | meiotic recombination protein SPO11, putative | genomic | Tb927_05_v5.1 forward | (geneCodeEnd+0 to geneEnd+0) | length=639

GCTCTGAGGAAATAAAATAATATATATATATATATATATATATACGCATCACCTCTTGGA

CACGGTGGAAGCTAAATGTTTTTCCCCTCCCACTCCTCCTTGCTGAGAATGCTTCTGTTG

GAACCGTGTGGGCCATAGGGAACGGCCTGTGACGCTTTGCGTCCACCTCTTTTTTTTAAA

AAAAAACATAGCTTGTATTGTTGTTCTCGTTTATTTATTTATTGTGTTTTATTTCGTTTG

CTGTTCCTGCATGTGTGCAGTTTGTTCGTTACGTGTTTGGTTGCTTTATGACTCTCTAAT

ATAGTTTTTTTGCGCATGAATATATTTTATTGTTCTTCTTTAGTAAGTAAATGCATATTC

ATATGCGTGTAAATAATTCACATGATTTATCTGACGTGTTGGTTTCAAGGCATTGTTGTC

CCTTTCCGCCGTCGGTTCGCTCAATCTGTGCGACTCTTGTTCTGGTTATTATTTTTATTG

TTATTATTATTATTAATATTATTTTTTACGGTGGTGTGAATATTTTTCCTTATACTCGAT

ACTTCGTTGCCCTCATGTGATGAGTTGTTGGCAGTGCTGCATCGGAAATTATTTTTATAG

TGAGCATGTTCTCATTCCTTATGGGCTTTCATAGTGATT

>Tb927.4.3340 | Trypanosoma brucei TREU927 | hypothetical protein, conserved | genomic | Tb927_04_v5.1 reverse | (geneCodeEnd+0 to geneEnd+0) | length=486

AAATGAGTTTAGTACACATATGTACATTTGTTTGTGTGTACGAGTGACCAATTTCAGTGC

GGGCGTTTGTTCTGTGTGTTGGATCTTTTTTTTTTTAAAGTTTTCCCCCTTCGTTTTGTT

TTGCGGACATAGTTTTCTTCTTTTCCTTTGGTTGACTTCCCTGTTGCCTTTTCTCGAAGG

CAACTGTTCTGCATCAAAGGGTGTCTGCATTTTGTGTTTCTCTGTCTTCTGCAGCTACGG

TGTGTCACTCATCACTGGAATGTGTTCTGCGCGATATCTGTGAAACTTTGTTAACTTCTC

TTCACATTGCAGTTCATTTTTTGTTTTGTCGGTGCGCGCGGATGTTTCTGTCATTATTGT

TATCATTCTTATTACTCATGTTCTTATTATTTATTTGTTTTTCGTTTCTAGTATTTTTTC

TATTTATAGTTTTATTGCGTTCCATCGTTTTGCCATTCGTTTTTGATTTTTCTCTTAATT

ATTTTT

>Tb927.1.2700 | Trypanosoma brucei TREU927 | hypothetical protein, conserved | genomic | Tb927_01_v5.1 forward | (geneCodeEnd+0 to geneEnd+0) | length=257

ATGTTAAAGCGAAGGAACTTCCACTACACCAAGTTTGATACAACTGCGGTGTGGTGGTTT

TATCTCTTGAATTTACTTTACACGGGAAATGAGGGGACGGGGGGGAACCTTCCGATACAT

TTGCACTATTTGGATATCCCACCACCACCCACGCGTGTTTTCCACACAGAAGAAAATTTT

ACATCTCTCTCTATTATTATTTTTTTTTCACCGTTTGCCTCCTCCTCCTCCCTGCCTGCC

TGCCTGCCTGCACATAT

>Tb927.7.6680 | Trypanosoma brucei TREU927 | protein kinase, putative | genomic | Tb927_07_v5.1 reverse | (geneCodeEnd+0 to geneEnd+0) | length=3276

AGCAAAGGTGTACATGAACGGGTATATATATATATATATATATATTTGTGCATGTTTTCC

CTTTCCATTTTTTTCTTTTTTTTTTTTTGCATTCAGCGTAGCTAAAATACAAGTAGATAC

AGCAGAGAAGCGTGTGTGTGTGTGTGTGTGTGCATGGGGAAGAGGAGCGAAAGTAAGTGC

AGAAATGAGTGAGTTGTTGGATTTACTCCACGAATATATATATATATATATATATGTATA

CTTAAAAGAAGAAGAGAAGGGGGTTGTAGAAAAAATAAAAATAAAAAGGAAAGGAAAGGA

AAAGAGGCTCACGGATCGTCGACAGTATGTTTTCTTCGCAGTTGAGGTGATATGTGGTAG

CATAATGAACCTATTGGTGATTGCATATGTTTTCTTAGCTCTGCATGTTTGGCAGATTTG

GTTACTCCATCATTCCACCTTTGATAAAATGAAGAAAATAAAAAAAAAAAAAGAAAGCAC

AAGTGGGGTAAAAAGAAGAAGAGAGAGAGGAAAAAAAAATAAAAGAAAGACCCAATGAAA

CAACAACAATAACATCATCGATGGCTGCGAGTGCACGGGATGGGGGCGAAAAAAAAAAAA

GAAAGTCGAGTTTTTAACTTTATCATACGTGCGTTTTGCATTCACTGAATATAGTTGGTA

GTATTTATATATATCTTTTACATCTTTTATATCTTTTATATGCTTCTTCCTCTTCTTCTC

TTTTTATTCCCGTTTTCTTTAGGCATATGTGAGGAGGCGTATTACAATTATAAGTATTTT

TGTTTATTTCGCCGATGGACTCATTGCTCGACTTACAACGGGAGATGAGCAGTGAAGGAG

GAGTGGGAGGATACGCTTTGATAATTAATTAAATTTTTTTTTTTACGTTTTTACGTTCTG

CTGTTCCTTCTTTTTCTGCTTTATTTATATTCCTGTGTATATTTATCCTTTCCACTCCTT

TTTTGTTTTTTTAGAGATCTGCTTCATCTTTTCTCTTTAGTCTTAAATGACCTCTTTTCT

TCCACTTTTCGTCTCGATTTATTTAGGTTTGCCTTTTTCCTCCTTTTCCTTTCCTTTCTT

TTCTTCTCCCTTTTTTTTTGCATTTGGTAGTTTTATTCTGTCGAATGGTATTTCTGGTGT

GGGTATTCACTCATTCACTCATTTATTCATCCACATATAAACACAAATATATGTTTATAT

TTATATTTGTGTGTATGTTTCTTTCTTTCTTTCTTTTTCTTTTGTGTTGTACCCAATTAA

GGAGTGACGGGCGGTAAATGTTGATGTATAAAGGTGTTTGGAAGTGGTCATGTCGTGGTG

GAATTTTATTAGTTGAGGAAAAAAGAAAAGGGGAAAACCAAAAGGAGTGAATGATCAAAG

AAAAAAAGAGGAAAGATGAAAAAAAAAAAAGAAAAAAGAAAAAATAAATAAAATGAAATA

AATGGCTAACCAAATAGGAGAAAGATTGAGGGAAGAAAGGAACCAGCTGCTTTGAGAAAT

AAAAAGAAAAAAGTGCACCCGTTCGTGTTTACATGTGCCGTACAACATCACTGTGTACAG

GCGTGCATTTACTTTTTTATTTTTAAAAATCTTGTGTTTTGCATGTGTGTTTATGTGCTT

GTATGTGCCTTTTTTTCTCTCTCTCTCTCTCTCTTTTTTTTTTTTCGTCTTCTCTCTCCC

TGCTTTCCATCTCTATTTTTATTTTTTATTTTTAATTTTTTAAAAAGACATAGCGCTGGT

GATGCGTTTATATGAATTTGGAACATGGTGATGAAACCCTAAAGGATGATGTGAACGTCG

CAAATGGCGGCTACGCCCCTCATGTGTTTCTTTTTCTTTTTTTTTTCCATCTTTCCTCTT

TTTTTGTTTTAAGCGCTTCAGATGTTACAAATCTGTCGTTCAATCGGCCGGGTTCCTTTC

GTCTTCCTCCCGTTTCTTCATGATTTATTTATTTGTTTTGTTTATTTGCGCTTTCGTCAT

TTTTTTTCCTTTTTTTTCTTTTTTGGTTGTCTTTGTTTTATTTTGGCGTTTTGGTTGTTA

TAACTCTTTTCTATGCTCCTCTTTTTATTTTTTCTTGTCTTCGTGCATATGTTACGGCAA

CGAAATATATATAAAAAGCGCAACGAAAAGAAACAAACAGATAAAACAAAATAAAGAATT

AAGCACAAAAGGTAGTCGTATCACCTAAGCCAAAATTAAACAGAAACAACAAAAAAAACG

AAGAGAAAAAACTAAAAAAAAATATATATATATTTTGCATGTGGGATGTGTATTTCAAGT

GATTAGTCACGGGTTGAAGAGGTGTTGACAACTGTGGTGCCACATCATGCTTCAGGGTTA

AGTAATGTTACGCTTCCTTTTTTTTTTAAAATTTTTCTACTTCTTTTTCTTCCCCCTTTT

ATGGTAACAAATAGAAAAAAAATATATGAATTACGTTCACGTTTACGTTTATGTTTTCTT

CCTTTTCTTTTCTTTTCTTTTGTTCCTTTTTCTTTTTTCTTTTTTTGACTTCCTCCACCC

TCATTTGGTGGCTGTTGTTTGATTCCGCCTAACGTGCCACCGGCACCTTTTTCCCACTCA

AAAAAAAAAAAAAAAAGAGAGAGAGAGGGAAAGTGAAAAATAAATAAATAAATAATAGTC

ACAAAAGGAAGAGCAACTTATTGCTTCGTAGCGAAAACACTTCTATTCTTAATTTAATCC

CTTTTTCTTCTTTCCTCTTTTCCTTCCCCCTTTTATAAATATGTTCACCCGTCTATTCAT

TCATTTGTTCATTTTTTTGTTTGAAGAATGATTTTTCACTTTTTTTTTAAATTACTTAGT

TCTACCTCTACTTTTGTCTTTCTCTCTCTCTTTATTTCTCTTCTATTTGTGTTGTCGGTA

ATCTTCTGCTTGGGATTGTTTGCATTGCGGTATGGCGTTGAGTTAACTTCGCACTCATAA

GTAAATGCATGTAAGACGGAAACAACAAAAACAACAAAGCAATCGTAAAATAATAACAAT

AATAATAACAATAATAATAAAGGAAAACATGTGCTTTGGTGGCGGGGGTGGGACAAAAAA

AAAAAGGAAAACCACCATCCCGCCGACAAATGAGGGCTGAGAAATCCGAGGATACGCCCC

CCCCCTCCTCAGGCAAATATGCGGTTATCTTCCTTTTATTTTGCTCACTAAATTCCCATG

AGCCGATTCCGTAACTTCTAATCCATATGCACCAACACACACACACACACACGCGTGGCA

AAAAAAAAAACAACAACAAACAAAAAGGAAGGGTTT

>Tb927.10.10050 | Trypanosoma brucei TREU927 | hypothetical protein, conserved | genomic | Tb927_10_v5.1 forward | (geneCodeEnd+0 to geneEnd+0) | length=673

GCGCGGTCCGTGCATCTAATATTTTAGATTGAAGTTGGACGGGGGTAAGCAGTGTCCACG

AAGTACATTTTTGCGTACTTGCATATGTATGGAAGGGAAGTTGAGTGAGGGAGGGAGGGA

AAACAAAAAGGAAAAGCACAGGTGGCGATGTCCTTGGATCTGTATTTGTCTCTGCAGTGT

ATCATTTGTTGTGTTAACTCACCCGTCTCTGCTACCGCTGCTGGTTTCGTGGACGTTCCG

CTCGTGGGAAGATGTACAGCCCGATGTGTCGAACCGAGCATCTACCTCAATGGCAGGTGT

GTGTGCGATTTCAAAGGGATTATTACTTACCTCTCCGAGATGGTATTAAAAGGAGGAGTC

CTTTGCGTTGAGGAAGGACGGGGTTAGTCTGACGGGCTGGCGCATTGTTAATTCTTCTTC

GCTGCACTGTGGCACGAGGGTCTTTGCAAATTGCATGTGTGCGTTTGCGTTTGCGCTCAC

GTGTGTGTGTGTGTATGTGTGTGTTTGTTATGTAAATATGTAAGCACTCGGACCTTTGAA

GGAGAGGAGCAGGTGTACTGGGAGGTTACGTATATGTGCACATATATATTACTCATGTAT

ACGTTACGAAGGTATTACTTGGACAGGTTTATGCGGACGGCAACAAGAGTAAATGAGCAT

AAGAAGGAAAAGT

>Tb927.11.10340 | Trypanosoma brucei TREU927 | Serine/threonine-protein phosphatase PGAM5, mitochondrial, putative (pgam5) | genomic | Tb927_11_v5.1 forward | (geneCodeEnd+0 to geneEnd+0) | length=277

ATTTGTTTTAAAAGCGGTGAAAGGGGGAGGGGTCGCCGCGGAAATAATACTAAGGGCGGG

ACCTTTTCTGTTTTCCTTCTTTTTTCCTTACTTGGGTGTGCGTCAGATGAAATAAGGAAA

AAAAACTGAAGGAGAAGGACGGTCAGGTCGGTGTCGTTCAGCTTTGCGCGAAGGCTTGTG

CCGGCAGTTTATGTCTCTAAGACTCAGGTGATGTGTTTTCCTGCATCTCTCACCCAAAGC

ACACACACACTTACGTGCGCATGGTTTTCCTCTCTTT

>Tb927.7.4510 | Trypanosoma brucei TREU927 | Domain of unknown function (DUF4201), putative | genomic | Tb927_07_v5.1 reverse | (geneCodeEnd+0 to geneEnd+0) | length=354

AGGCCCACTGTTTTTTTTTTTTGACTTGGGATACAGTGGACGGTCACGCGTTGCTGCGGT

GACATTACACAAAACTGTCACGTCCTGTTGGGGATGGGCGGAGAAAAGGGGATGTAGAGG

AGTATGGAGCGTGTGAAAAACTGGTATCAGAATGATGATGTCCGCAAATTTAGCAAGGTG

TAGAGGTGAGCTATGTTTACTGACAAGCTTCGGTAGGTCGCCCTGTGCTTGTCCGCTTTT

TTTTTATTTTATATTTATATGCTAACTTTCCCTACCCTCATTTTCTGTTCGTGTTTTAAC

TTATCATCGCCTTGCGCACCATGTAATCTCCTTTTCTCTGGGTGCAATCCTATG

>Tb927.7.7100 | Trypanosoma brucei TREU927 | hypothetical protein | genomic | Tb927_07_v5.1 reverse | (geneCodeEnd+0 to geneEnd+0) | length=2959

AGAGTAAATGAATAAATGAATGGATGAATGGTGGAGGTGGAGGTGGAACGAACTGAAGTG

AAGCGGATTGATATGAATATTAATATATATATATATATATATATATATATATATGCATAC

AAGAAGTCATAAAGTATAGCGTAGGGAATAAAACTGTTACTATTTTCTTTCTTTCTTTTT

TTTCGAATTTGGTGTTGTCTCTATCTCTCCTTTCTGACGTGACATGCGCATGTGTGTGTG

TGTGTGTGTGTGTGTGTTTTCCTGTAGGGGATAAATAAAGAAAGAATAAAGGGAGAAAGG

TATACCTGCTTGGTTGATTGTGTGCTTGAAATATGTGAAGGAACGAAGCACGAATAAAGT

TATTTAAAAAATGAGAAGGAAGAAATTAACATATGATCGAATAGATGCGTAATTAGAAAA

CAAGAATTGTTCTAGAGAAAGAGGGAGGAAAAAAAAAACAAACCTAATTGTTAATAGTTT

TCTTTGCTTTTCGTTCTTTTTCTTTTTTTCTTTGTACTTTTGTAGAAAAAAACTGTTGAC

GATACAAAATGATTTGGTTTGATTTGTAGTGCACAAAATAATAATAATAATAACTGAAAT

GACACATTTTATTTACCGCAGTAGGGACGGAAGGGGTGGAGAAGAGACACGAAATATTTT

CGAAACGGTACCTAAGTGCAAGGAATATATTTATATTTATATATATATATATGTTTGAAA

TATTTTTCAACCATTTAAACCAGGGGAATGTGGGCAAGCCGTATTGCTGTGATCGGGAGG

GAAATAACAGGGGAAAAAAGGCAAGAGGAATTTTAGAAGAAAAGAAACGGAAGTGCAAGA

AATATATTTATTTATATATAATTGGGAAGGGGTGAACCAAATGCGCAAAACTGTTTCACA

AAATGAACCAAGTTGCGGCAGCAAACAAAAAAAAATAAAAAAGAAAATTATGAAAACGGC

AATGGCACGAACCGCAATGAAGTTTAACTAAAAGCGGAGGCCAACCCAAGCGGCTTTTGA

ATGCCAAAGTGATTTTATGAAATATAGTGGAAAAAAAGGGAGGGAGGAAAACAAAAAAAA

ACAGAAAAAATAAATTATTATTATTATGTTGTGAGCTTCAATCATAAATAGTGGTCAATG

TGAGCATTGTTTTGCATCTTCTCTTCATACTATTTGAAAATGCCTTTTGTTTTTCTCTCT

TTTTTTTTCTTTCTTTTGTTCTTTTTTACATGTTTTCACATTTTCTTATCTACTTATTTG

TATATTTATGTGTAAAACGATGTTATTGATACTGACACTATTGTTATATTCATGTTTTCT

ATAATAACGTGTGGCTCGCCGTTTTGCTGTTGTGTTCCTTTCGCTCTTTAGCTTTCTCTT

TTTTTTTCTTTGTTTTTTTCTTGCTTTTGTTTTCCTTTCCTCCGTTCTTTTTGAGTGAAA

TGAAAAAAATAAAATAAAATACCCGAAACTGTCACTTCATCGCCGTTGCCGTAATGACTG

CTGTTGGTGTTGGTGTTTGCATTTGTGTGCCCTTTAATAAAATGTTTGATGTCTCTTCAA

TTTTTTTTTTTAAATGGGATTCCGTCATTGTTGTTGTTGTTGTTGTTGTTTTTAATTTTA

TGGTTTATTGCAAGCCTTTTCACAATCTGTGATGCACATCAAAAAAAATATATATATATA

CAGATAGATAAGTAAATTTAGTTTTCAGAAGAAAAGAAAAGGAAAAAGAAGGAAAAAAAA

AAACTCTTGCAGTTCCCTACACGCGTACCTCATTTCTTTTGGATTTAATTTACTTCGCTT

CCTCTAAAAACACGAAAGAAAAAAAAGAGAAGAAAAAAGGTGTGACGTGATGTAAATCCT

TCCACCTTTTTTTTTCTCCATTTATACTGCTGTTTATTATTATATTATTATTTTATTATT

TTAGGCCACCGCTGCAAAACCGTAGCGAGTTAATGAAGCGGAAGTAGTGAAAGGAAAAGG

CGTGTCTTTTCTTCTTTTTTTTTTTCTTTTGTGTTTTTTTTCTCCTCCTCTTCATTTGTT

TGGTTTCTTTTCTTCCTTGGCGTCGTTGTTATTGTTATTATTATTATTATTATTATTATT

ATTATTATTAGAGTGATCCGCTGCTTCATTTTCCCTTTTGATTACGTGCAGACAAATGTA

TATATTTGTATATTTATTTCTAACATATTTCCCCATTGTGTGAAAAAGTGCAGTTGAGGA

ATATATATATATATATATTATGTGTGTGGTGAGAAGAAGCCGATGCAATAAAAAATGTCT

GAAGCATTCTCCCCTTGCTTTTTCATTTTTTTCTTTTCACTTTTTTTTTTGTGTGTGCGT

GAGAATTCGTTTTAGATCGCCCATCCATTCCATTTCATACTTACTTCTTGTTATAATATA

TATATATTTATTTATTTATTTATATTATTCCCATCCTTTTTGTATGCGCTTATATTAATT

TATGTATTTGCGTTTGTTCGTTGTTGTTTTTTTTTTAAATTTCCGCAGCCGTGGCGGAAT

ATCATCAAGTGAGTGATATCAAGTTATAAGAGACGACAATAACAATAATAAAAATAGTAG

ACGAAACACAAAAAACAAAACAGTTGTCAAGACAGTGAGGACAACGACAATATTGAAGGA

AGAAGATGTAGCTTTGAGCGTTCAGAAAGTGAAATAATAAAAAAAACAAATTAAAAAAAA

TGAAAAGAAGATAAAATGAACTCAACAAAATGTAAATATATTTATGCGTTTGTGTATGAT

CAAACCCATCGATACAAGTGTATTATCGCAGTGTTTCAACTTCTTCTGTCCTTTTTTTTT

GTTGTTGTTGTTGTTATCTTTTCCTGTCTTTTTTACACTCACTCAAACATTGAAAATAAC

AATAACATTAATAATAATAATAATAATTTGCGTATCCCTTGTAATAACAAAGAATCATTT

GTCTAAGAAGGGATTATTT

>Tb927.11.11360 | Trypanosoma brucei TREU927 | receptor for activated C kinase 1 (RACK1) | genomic | Tb927_11_v5.1 forward | (geneCodeEnd+0 to geneEnd+0) | length=163

AGGCAGCTAAAGGGCATGTGGTATAAGTGGTTTAATTTTGTGCTTTCTGCGCCTTTTCCC

GGGCGTGCATACAGTTGCACGTAACCTAGAAGGGTGTCGCTTGCAATTTTCATTTGGTCA

GCAGTTGGAGCTGTCTTTATTTTTTCTTTTTCACCTAACATTT

>Tb927.8.6650 | Trypanosoma brucei TREU927 | RNA-binding protein, putative (DRBD12) | genomic | Tb927_08_v5.1 reverse | (geneCodeEnd+0 to geneEnd+0) | length=2800

AGAATTGACGAAACAAGCAAAAGAAGTAAGTTGTGGCACTGAGATGAGTTAGCAGTGGGT

AAGGAAAAAGACAAGGAAGAGGGCAGTGGCAACGATAATAATATTATTGTTATTATTGTT

ATAATTATGGTAGCTGTTGTTGTTGCTATCACGCAGGGAAAACATGGTAATATATATATA

TATATATATATATATATATTTATAGGTTTATTTACGCATTCGAATATGCATCTGTGCATG

TATATTGAAGAGAGAAAAGTCTTACGGGTGCGTTCGAAAATTCAAAGAAAAAAACGCATG

ATGGTACATTTACCTATCTGATGAAGCAGTTTATCAAAGGAAAGATGACCTGAAGACGAA

AACAGCAACAACAGCAGAAGAGAAAAAAAAAGGAAGGGAAAAGCATCATTTACCGGGAGG

AAAAGAGAGAGGAGTTCGGCTAGTTAGTCTTTCATATACGTCGTATACTATCGCAAAACA

GAGAAAATGTGTGAGGGAATAAGGGAGCGAGCGCACACGAATGCATTTAAAATATGTAAA

TGTGAATGCTTATAATATTTCTACAATATATTCCTGCAGTGCTCCCGTGCTTTCCCCTCC

CTTTTCTCTCTTGCCGCGTATTCAAACAGGGGAAAAAACATTCACTCCATTCGTTATTAT

TATTATTATTATTATTTTTATTTACATTTCTCCCTCCACACTATCAGTTCCCATACATCC

ACGCTGATAAATAAACAAACGTGCATATATATATATATATATATATATTTATTTATTTAT

TTTTGCCTTTGCCTTTATTTATTTGTTTGTTTTTGTGTGTTGTATCCCTCTTCCTCATCC

TCCCGCTTTCTATATTGTAGCCATCTCACATTCGCTCATTCAGTTATGACTTTTTTGTTT

TTTTTTACTTTAACCTTTAATTTGTACGAATGACGGACAACTGAAACCGCACACCGTGGC

GGTAAAAAAAAAAAAGGAGGAATTTGAGGAATGATGTGGAAATTTTGTGTTTGTATGCTT

TTGAGGAGGTGTGGGATTCACCGGTATTACTATGATTATGATTATTATTATTATTATTGG

TATATTTCTTTTTTTTTTCCTGTTAGTACCTCCATCCTTTTTTGTTGCTGCTTTGGTTTG

CTTTTTTTTTTTTTGACTTTTACTTTTAAACTTTGTTACTTTTATTCTATCGAGAAGTAA

AATGCTTTCCCTCCCTCCCTTTCTCGCTTATTTTGTATTTATTTGTGTTTGTGTTTGTAT

TTGACTTTACACTTTTCCTACTCTTTCTTCCAGTCATGAGCCATCCGTGTTTATTATTAT

TATTATTATTTTTTGCAACTGGACTGACAACGTTACATTACATTACATTCCCTTCATCCT

CTTTTTTTTTTTTACTGTCATTATTATTATTATTATTATTACTACTACTACTACTACTAT

CATCATCATCATCATCATCACCATTTTTGATTTTTTTTGTTTATTTTATTCGACTCAAAC

ATTTGTCTTTTTTTTTTCTTTTTGCCTTTTTATTTTGTTTGTGTTTCATGTGTGTCTGCA

TGAAGCTCTCCTTAAAACTTTTTCTTCCCCAAAACAGTGTAATGCAGCACTGTTCTCCTT

CTTTATCTATTTCTTTTATTTCTTTTAGTTTAAAAATCACTTTTTTCCCTCTCCTTTGTT

TTACATATTTGCTCCTCTTCTTCTTATCTCTGTGTTTTTGTTTTTGTTTTGTTTTTGTTA

TTTGGTTTCACTTCTTTTTTCTCCTTTCCCCCATCCTCCATGGCATTACACCAAATAATT

AACGTAACATTTACGCGTATTACTGGAGGGGAGGGGAATATGTGATTGACAAAATAAATA

ATCACGGGAGTGGGATGTGCCGTGTGTGAAAATTAAGAAGGAATAAAATAAAATAAAATC

TTCCAGCATTAAATATAACAATAAAGAGAAATGAAAAGTAAATATTGACGAATAATGCAC

GGTAATCACGATAATAATAGCAATGATGATGACAACGACTTTATTTAATACGGCTGGGCC

ATTCGGGACGAATATTTTGTTGTATTTATATTATATGTATATTCATGAATATTTCTTTGC

TATATATATATATATATATATATATATTGTTTTTTACCTTTTGTCCCATTTATTTTTCTT

TACGATTCCTTTCATTTATTTGCAATGCAGCGCTTTCCTCCACCATTATTATTATTATCA

TTATTATCATTATTTTAGAAAATAAAAATCCTTTTCCTTTCCTTTTCCTTTGTATTTCCC

CTTTCTCTTTTTAGTTTTCTTTCCTTTCTTCCGCACCATTTCGTTACTGGCGCCTTTTTT

TTTTTCTCTTTCTTTTCCTTTTTTTTTTTCCTTCTGCTCCTCCTCCTCCCCCTCCTTTTT

TTTTGTTCTTCCCTCCCCCTCCCCCTTTCCCCTTTTGTTTTGTTTTTGTTTTTTGTGTCA

ATAAAAAGATGGCAACAGGAAAAGAGGAAGGGGGGAAACGAAAAGTAGAAAAATATTTTA

TTTTGTATTATTTATTTATTTATTATTTTTTATCTTGTGAGTGCATATGAGAGATGATAA

TATTTTTAAAAAATATATATATGAGAAGAATAAGTATATGAATGCACATACTCCCATACT

TACAAGTAAAGAAAAAATCATTAACAGAAAAATATTTGCCATTGACTGCCGAGGCAACAC

AAAAAAGTAATGAAAATAAAACGGAAAGAAGAAAAAAAAAAAAGAACAAATGATGGAATG

AAATATAAAAATTAAAAGTGAACGAAAAGAAAAGTAAAGT

>Tb927.9.2250 | Trypanosoma brucei TREU927 | hypothetical protein, conserved | genomic | Tb927_09_v5.1 reverse | (geneCodeEnd+0 to geneEnd+0) | length=853

AATGCAGTAGCTTGTAGCAACACTTAACATAGAAAATGTGGTACATGAAGTGAGGGCGAG

ATATGATGCGACACGGTTGCATTCGTGGTCACACTTATCCCTTTCTTGGCTAAAGCACTG

ACTATCGCACGTCACCACTAGTCGTGTCATTTGTTCTATTGATTTTCTGTTATTTTTTTT

TGTGTGTGAAATTCGTTTCCTTGTTTATTATTATTATTAACATTTACTTACGTTGCTTTC

CTTGCATCATTTAGGCATTCAGATGCATTTACAGACTTCTTTGTGGTGGAAGCTCCTTTG

CACTGTTAGAGTATTTCTCACCTTTTTACTTTTCATTATTTTATTTTCACATACCTGAAT

GCTTTAATTTCCATTCTTTTATACCCTGTTTGTCCGGTGTGCATCTCGACATGTAATTTG

TACGGGCAGGACGCTTGGGGCAGTGGAGGAGAGGACAAAGAAAGTTCTCATTCATCCAAA

TATTTGCTGGATTTTCTTCTTCACCTCAGTTACGGTTTTATTCGTTTTGGCCCTGGGCTC

GATGGTGCTTAAAAATATTACCAGTCTCCGCTCATTTACGTAATTTTCTTACCATTTCCA

CTCTTTTCTTGATTTAATCTCTTGCCTCGTACCTGGTTGCCCAATTAAAAGTAACTTCGG

AGTCGCAGAAGGGGCCACGTGAGGTAAACTATTAAAGTCGTGGTTGGGAACTTACCCAAC

AGAAAAAAAAAATTCCCCGGCGCGGAAGGAATACCGCGAGGCAGTAACCGTGAACCCCTT

TCAACCATACGCGAGTGGGCAGAAGGGAGCGGATTGTTCTGTGACCACTCTGCGGCCCCA

CGAAGGTACCAGG

>Tb927.5.2360 | Trypanosoma brucei TREU927 | hypothetical protein, conserved | genomic | Tb927_05_v5.1 forward | (geneCodeEnd+0 to geneEnd+0) | length=3160

AGAGGCACAGTTAGTATGTTTGCATCGTCTAAACTTATCCTTTCTTTGTTTATGTGTTAA

GTTGCACTTTACTCAAACTCTCCAGATATATTTACATACGAGTGTGCGTTTCTCCGGTTG

TTTATTGTATCAGAGGGCCACTTTTTAAAAATTTCTCAGATAGCGGACGCATTTCTATTC

AACGGTTAACATCCTTTTGTTTACCCTTTCATTATATATTTATTTGTTTTTATTTTCTTT

ATATGATAAAGTATGTGTTTATATATAATAATGGGAGTGGACCTTAAGAGGCCGTTCCAC

CTTTCCTGAAGTTAACTTAAGTGCGAGGAGGAGGAGGAGAAGTCCGATTGGTGCCTTAAC

TTTTCATTGTTACTTATCTATTAAGTTGTTACACACTTCTTAAAATGAGGATGATGACTA

TCACCTCATTTTATTTGTTTGTAATAACCGGTGATGGGAACACTAAAAGTGCATCAAACA

GCAGCAAGAAAGGGCTTTTACCTGTGTGATTGGATGGATGAAAGGAACATCTTTTTTTTT

TTTTTTGGGGGGGGGAGGTGGTGATGGGAAGTAGAAGTGACAGACGCATTTATTTATTTA

TTTTTTATTTCGTGTGTTTCGGCAACATTTACTCGGAAATAGATTGTTTCGAATCACTTT

CCGCCTTTGCTCACATCTTGTTTTAAGTAAATGTGACACTAACCCGCGTTGTTTTGTCTA

TTGGCTCGTATATTTGTGTTTGTGATTTTTTCCGGTTTATCCTTATGCTTGTTATCCGTA

GTGGGTGCTGCGCCCTAAATTTTTAATTTCCGTTTCACAACCTTGTTACCTTTTCCCCCA

GTTCTAATTTACACTTCCGCCTTTCTCATGAATCCCTGTCGTCTATATATATATATATAT

ATATATATAACACCGTTTTATTTTGTTCTTATTTGCTGGGAATTTCACGTAAATAGCAAC

ACAGTGTGAGCCATATTTGTTTTCCTCTGTATACTTACCTTTTTCTCCCCCTCCCAGATA

TTTACATCTCTACTTATCATCATTTTCTTTGCTGTATTTTGTCATTGCTCAATGCGGGTG

TTTTTTTTTTTTGCTTTTAGGGAGTATCTTCCGTCAGATCCTTTATTGTGTTGTTACCCT

TTTCACCCTCTACTCCCTTCACATTATTTTTTTTTTATTATCAAAAATAATAATAATAAT

AATAATTACCATTATTATTATTATTGTTGTTTTATTGATTCTTCATTCCCGTTACTAGCG

CTTATAATGTTATTGTTGTTTTTGTTTGTTAGTGTCTTTGTGATGATGCACTTACATGTA

GGTGAACAAAGCGTCTGTGTATGGGCGGGAGGGAAATGTTCAATGTATTTTTGAGTATTT

TTTTCCTTTTGCCAGTTTCCCACCTGTTAGTTTAGTTCGCTAATTCTGTTTTTTTTTTTT

TGCACCTAATAGTACAGCATAAAGAAAGAACCTCTCAATGTCTGTACGTTTGAGTGTTTG

TAAGTGTCCGTTCATCCCTTTATCTCTTCTTCCTTTTTATGCAATGGGTGTGCGAGTAGG

CAGATAAATAAATAAATATATATATATATATTATATGCAGGAAAAAAAAACAATAACAAT

AATAATAATAATAAAGAAGGGAATATATGGGATATGGGATGAAAAGAAAGAAAGAAAGAA

AGGGAGAAATAATGAGAAAGAGATAGATGGTAAGAGTGAGAGGGAGTGACATTTTATTGT

GGCGTCACTTGATATAATAAAGGGAAAAGTACGTAAGAGGTAAAAGGGAGTAATATATTA

TTGTGCATGAAAGTGAAGGTCAAGAGATAAAAAAGCAATTTGCAGAATAATTCACGCGCA

TTAAAAAAAAAAAAACGCAATGAGACGTTGAGTTTCTGTTTCTGTTTTTATGTGCGTTCC

AACGGATTCATTCGTTTCGCCTATTTGGTTCTTTTTTTTTTTATAGATTTAACAACTTCG

TGTCCTTTGTGTGCATCTGCTCTTTTTTTTTTAAATGTCTTTTCTATTTTTCCCCCGTCT

TTCCTTTCGCTCTCTTCGCATGTTTCTTCTTATTTCAGCACCCCATTTTCGTTGGTACTC

GTGTTTTGACGGTGGGAAAAGTTTTACTTAATTTTCCGTACGTGTTTTGTTATTTGCGGG

TGTTTTGCAGCTCGTTTCCATATTTCAGAGTACAAATTCGTTCTTATCGTTAATATTAAA

CAAAAAGAAAAATTCCTTTCTCTTTTAGTGGACTGAAGTTGTCGTCATTGTTAAATCTCG

TTTTCTTTATTAAAATTGTTACGCGCGGTCCTTCTCTTACCTTTCCCAACTCCATTTCCT

TTTCTCTTATTTTTTTTAAATTGTAATTTCCATTCACTCTATACTTATTATTGAATTGTT

GGCGCGCTATGATTTATTATTATTATTATGATTTATTATTATTATTATGATTTATTATTA

TTATTATTATTGCTACTACCTTTTCTCTATAGAAGAGGGACAAATATTTTGTTACTTTAT

GTTATGTTTGTCCATTTGTCCGCTTCGCGCGTTTGCATTGTTTGAATGAATGATATAAAA

GTTATATATATATATATATATATATATGAAATGGGAGCTAGTGGAATAAAGCAAAATAAG

GAAGATGTTTTTTCCTTGGGGGAGAAAAGACGTTGTAGTCGTGTTTTTGTTCTAATATGT

TTATTTCCCCCCCTTTCCACTCCCTTTTTTCTTCACTGTTTTACCCTTTAATCTATTTCC

CCCTTCTTTCTTCGGAATATATATATATTATACCAATTATAAGTGAACGAAACGATTTTC

CATCATCATTTCTTCTCCTCTTGTCATTTTTATGTTATTCCTTATCATTGGCATCGGTTA

CTTTATTATTTCTTCTTTTTTCCTTGTATGATTTACTTCCACCGTTTCTATTTACATCAT

TGATCGATCCATCTTTTTTTTTTTTTTACTTTCCTTCCTAATCCCAAGCATTCTCGATAG

ATTTTTTTTTTTCGTTAATACGGTATGCAGGTAAGCAACCGAATACCAGTGTGCATTTTT

GGGCATGTCTGAGCAAAGTTTCCTCTCTTTTTTTCTTTTTGTATATGTTTATGGAAATGT

ATTCGGTGGAATGTGAGCCTGCGGTACGATAAGAAAAGGT

>Tb927.11.6990 | Trypanosoma brucei TREU927 | serine/threonine protein kinase, putative | genomic | Tb927_11_v5.1 reverse | (geneCodeEnd+0 to geneEnd+0) | length=1723

AGCTTGGCATAAAAATATATCACCACAGTGGTGGAATAGTGGTATTGGTGACATCAATGC

AAGTGGCTTCTGTGCTTGTGTGTGGAGGGAGTCCACAGTAGTGGGGCGCAGAAAATATGT

AACGCGGCAATATTCCATCGTTGTCGTTGGTGTAAAATTAAAGCATTACATTGCAGCAGC

GATGTGTCAGGAAAAGAGGAAGGGGAAAGGTGCCATATGGAAAACCAGAAGATGGGATGG

TTGATGCTGCAGCAAAGATGTGTAATTATCGACTCCGTCTGTCTGTGTCTGTGTGTGTGT

GTGTGTTGGCGGGAAAAGTGATGAGGTGCAGCTACCTGCGGCGCGACAGCCGCCATCACT

GTACCGTTTGCCTTTTCTGTTTTTTTTTTATACTCACTGCCAAAATTCAAACCGTTCATT

ATCTATTGTTATTTTTATTTTATTTTATTTTTTTTGCTCCTACACTTCCTCCACGCCAGT

TATTTCGTGAGCGGTGTATTTTTTCTTCTTTTAGAGTCGCTTGCTCCGCGGCCAATTACC

CATTTGCTGCGCCGCGGAAAAGAAAAATGGGGGTTACTAACCGCGCGCCAACTCGCTACG

GAGGTTTCGTTCATAATTTTATTTTATTTTTTTCATAAGCATCCATCTTTTTCACGTTTT

GTAACATGCAAGTGTGGGCACATATTTATGTTTTTCCCCTGCGTTCTGTAGCGTGTGTGA

TCACGTCTCTCTGATGCAGTGGCGACGTGTTAAGAAGGATTCAGGTCTTTGGTGTTACAT

TAGACAAATAATAACAAGAAAGAGGGGAAAGAAGCCGGATTAATATATTTACCTTGAAAA

GAAAAGAAAAGGAGATAAAAGGGAAGAAGATAGAGGTGTGCACGACTTTATGATGCCTTT

GTTTTTTTTTTTTTAACATTTACTCATATAACTTTTCTAAGCGTGCCTGAGGGGAGGATA

TCGGGGAGAGGGCATGCAAATTGCTCCCCGGAATTCTTTCATACTGCTGAGTGCGTGCGC

GATAACATTTTCTTACATTGAAAAGATGATGCGCCTCTTGAGGAAAGAAAGTGAAGATGA

ACGCACAGAGTAACCGAACGCGTTTATTCTCATCTCGCTTCATCACCACTAACGGGGGGG

GGAAAAAAAATCATCCCATACTTCTGTGTTTGTCTTTTCCGCCCCGTATAACCACATGCA

ATGTTGTGAGTTGCGGGTGGCTTCCATTTTCCTTCTCCTTTACCCTTCTCTACACAGAGG

AATAAGGTGTCGATGTTCGTAGTGTCATGCCGTTTTTATTTCTTCTTGTCTTATTTTGTT

TTGCTTTTGAGAGTAAAATTTTTTTTCCAACCTAAGTCCGAAATAACCCCTGGTTAACTC

CGTGTAGGACAGCGTACGCAGCTCAACTCTAGAAACGCATACTTTTGGTTGTGCACTTTA

TGGTCTCTAACTGTCAAACGGCGCGAATGTTTTTGTGGGTGGATGACGAGTTAACTTCCT

TACAAATCAGTGTTTTGTCTGTTTTTTTTTCGTATTTATTTTTTTTATTTTAATCCTTCT

CTTGCGGTTAATGGGCGCTCTTTAATGTCTTCCTAGGAACAGGCGCAAAACGTGACCAAA

CAGTGGGATGCGCCTCGCATTTCATCACGCATTACAGACCCATGCAGGAGCATCTACAGC

TGTTTAATTGTCACTGGTGGAGCAATAACTACTTTGGCCTCAC

>Tb927.10.8430 | Trypanosoma brucei TREU927 | 40S ribosomal protein S12, putative | genomic | Tb927_10_v5.1 forward | (geneCodeEnd+0 to geneEnd+0) | length=105

GCCAGTGCAGGAGCTCACTCACGATAACACAAGCAGTTGTGGGGTGGTCGTGCGCCCTGT

ATTCTTTTTTTTTATTTTTTTCTATAAACTTTTCCCCTGACTTTC

>Tb927.10.14900 | Trypanosoma brucei TREU927 | hypothetical protein, conserved | genomic | Tb927_10_v5.1 forward | (geneCodeEnd+0 to geneEnd+0) | length=1785

ATAGGTCTTGTTGCTTTTCTCCCTTCCTCACCTCCATCGTGGTGGATCCCACTTCCAATG

GGGGGGGGGCAAGGTTGAGAGGTCGATCGATAAAAATGAATCAAACAAATAAAGCGGTAT

GTACATACTTATTAGATTCTTATACATAAAGACAGCCGTCACCTACCGGTGTACGTGAGT

GACAGTTTGCTTTTACCTTGGCTTTCACTGCGTTCCTATATGTAATCACACTGTGGGTGC

CCTGAGTTTCAAAAAGATATATATGAAAGACGATTTGTTGCGCCTCATCCCCCTCTGCTT

TAGCTTTTGTTATTATCATTATTTTTTTTGTTGAAGTTGTTGAGATGGCGGTCGATACCG

GCACCAGACTCATTGCATCCCCTTCAAACCTCCTGATGTCATGAATTAAAACGGAGAATG

AAAGAAGAATGGTGTGTTTCCCTTTTTTTTTTCACTGCATTTTTGCTTTTTTTTTGGTTA

TTTTCTTTTCCCGACGTCCTCCCCAGTGACGCGACACTATTCCGGCTCGCGGGGAGAAGT

GTTTGAGAACAGTCATGGCTTCTTTTCCCCCTTCCCCCTTTCCTCCCTTTATCACCATAT

ATATATATATATATATATGCTTTAAGTATTGTGTGCATACGGATGCACGCAAAGTTATAC

ACGCACGCGCCTTTTTTTTTTTCCACCTGATCGCGTTCAGTAGGCCGTGGGGATATCTTC

AGAGGTGTGCTTATTATCGTTTTTATTATGATTATTTATTTAATTTGTTAAATAGTGTTA

ATACGGAGTTGTGGTGGGCGTCTCCGCCTTGCCTGCCGATTTTAATTATGTTTTCCGTTA

TTTTTACGTTTGTTATTTTGTCGCTGTTGTTGCTTACGGTTTCCCGTATTTAAAGTTTCC

CCTTCTCTCTTTTTTCTTGTGCGTTGCCGTTCCAGCCCCCTTTTCTTTCAGCGTGGGTAT

TTTCCCACTATCCAAAGGTCCTTTTCCTTTTTTGGTTTCCTTTTTTTCTACTTATGTGTG

CAACCTCTCTTTTCTATTTCTCCACGCAGCCATTTGGGTCCAGTGTCCCGTAACGTCCCG

CTGTGTGGCGCGGCAATTTTATTTTTACTGGGATGAATTTAACAGTTTTCCACCACATTT

TTTTCCTCTTCTTTTTATTTTGCTTTCCCAGATTGCTTGCTGTTTATTGTTATTGTTATT

ATTTTCTGTTACCTGGTTCCCCTTGTTTTGTACTCGCCAAATTAGCCTTTTCATTCAATA

ATAATAATAATAAAGTACCTGCTGACTTTTTTTGTGGGCCTGTTTTTAATCTCTTTTTTT

TAAACTTCATGTGGTACTGGAGAGAGGAATGCAAAAAAAAAAAAGAAAGAGAGAAGGAAA

TGAAAAGCTGAGGAAGGAAACAAAACAAAGAATAGAGAGTTTCACTGATTGACAAGTGTC

TTGCCATCGCATTTTCACACATTCTTCAGGCTTGAGGAGAGCAGCGCTCATGGCTTCGGG

TGTGTGGAGTGGCGTTACACCTTAACCCAACGAAAAAGACTATACGTGCATGTATACATA

TATACATATACATATATACAAATGGTATTTTTTTTAAAAATAAATAAATATACATATATA

TATATATATATATATACATATATTAATTGAAAGCACTAAACGTAATAAAATGAAACAGTG

CAAATAAATCATGTGAGACGACAAGGGAATGTTTTGCACATGTAATTATTTATCTTCTTA

TATAACCCAAATATATCCATATATATGTGCTTAAAAATATTTTTT

>Tb927.6.2270 | Trypanosoma brucei TREU927 | AdoMet dependent proline di-methyltransferase, putative | genomic | Tb927_06_v5.1 forward | (geneCodeEnd+0 to geneEnd+0) | length=96

AGCAGTTGGTCCTCCCACCGGTTGTTGATGTGTTCTCACTTCTTGTTGGCGCATTGCTGT

TGTCGTCACTTTGTTTTGTTGTTCTTTGCATGGTTT

>Tb927.3.3340 | Trypanosoma brucei TREU927 | 3', 5'-cyclic nucleotide phosphodiesterase, putative (PDED) | genomic | Tb927_03_v5.1 reverse | (geneCodeEnd+0 to geneEnd+0) | length=1625

GTGAGAAAGAAAAAGGTAGATAAGTCTCCTACGGGATGCCTATGGGTGGTATGGATGTTT

GTTTTTTGTTCGACGCGTTGGGTGGGGGTAATATTTATTTCTTTAGGTTATTTAACGCCC

ATCTTTTTATTCTTTCATTCCATTGACCTAATCAAGAAAGGAACGAAGAGAATGAAGAAA

CTGAAGGGTGAGGGGTAAACTGAGGGTGCAAGTAATAGTAAATATTGATAACAACAACTA

TTTACGTGCCTAAGCGACTTACTGGTCATTTTTTTTTGCCATCTGTTCTCTCTATTTTTT

TTTCTTTCCGCCGTGGTATGATTTTTTGTATCTTTACGATGAAACTGATCATCATCGTTA

TGGGCGCTGTCATCACCGCTATCACTATCATTACCTTATTTTTTGAGTGGGGGCTATTTG

TGTGCTTGAATTTTCTTGTCAAGCAATTCACATCGGAAGACAATACCTGTGCTGATGCGG

AATAACACCAGTTACTTAAGTTGTAAAGCTGCGTACTTGTTACATGGAATGTAAATAGAA

TTCAGTCACCACACACCTCTACACCGTTCTCACCCTGTTTCGATCTTACTCTTACTCTTC

CTTATTTGATGCGTATTTTTGTTTATTACCTCTTTGTTACCGTTTTCTCCGATTGTTCTT

TTCCCTTTTCCTTCTCTGATTCTTTACGCCGTTGTCCATTTACACATGATAATTTACTGA

CACACACACACTCTCTCTCTTTCTCTCTCTCTTTCGTTTTGTTACGACACCTCAGTTTAA

CTCTTCTATAAAAAGGGGAGGTAAGGCGCGCGCGTGTATGTGTATGTATGTGTATTGGGG

CGAAATTGTTTGATTGGATCGCTTCCAGAATGTTGTAATATGTTTCTTTTTTTTTCTCTC

TCCCTCTACTGGCATCAGGCGGTGTTATGAAGCGGCCATGTAATGACAAGTTTTTCCTTG

CCTTGCTAATTTAGTCTTTTCCCTATTTTTTTACTTACTTGGTTTTCTGCGCTAATGTTC

CTCTCCTGTGTTACTTTTCCTTTCCATGTTGAGGATGCACTGCTCCTCTCCATGCCCTGC

TGCAAGTATAAGTGAAATGTAAAAAAATAAAATAAAAAAAATTAAGGTAATATGGGGTAG

GGGATTGAAAGGACTAAGACTCAGAGAGAAAAGAAAAAGCAAAAAAAAAAAAAGGCGCCC

TCTGGTCCTTGTCCTCACTGCGGTATGAGACAGCAGCACATTTACCTGTAATAACGGTCA

AAAACATGAATTCAGCGTGAGATGTGCCTGCAGTTCTCCACAAGGCTGCTCAAACATTTT

GGAAGTATCTGGAAAGAGCCAACAGTGTTTTACGACCGTATAAATTGTCCCAGTTGTTCA

TTCCTTTTCCCTTTTTTGCGTGTCCCTTGTTTATTTATTTATTTATTGTATCTTCTCGCT

ACTGTTTCCAGTCGATGGACAATAGAAGCATTGCCCTATTTCCACTATTTGTGTTCTTAT

AACTTCTTTCATGTGTTCTTTCAATTTCTTTATTTGTTCACGTTGTTTACGCTAACGTAG

GACAACAAACAAACGAGGCAGCACGGTGAACATCGTGGAAGCCAAGGAAGGGAAAGAAGG

AGAAG

>Tb927.6.5020 | Trypanosoma brucei TREU927 | cyclin 7, putative, CYC2-like cyclin, putative (CYC7) | genomic | Tb927_06_v5.1 forward | (geneCodeEnd+0 to geneEnd+0) | length=494

ACAATGTAATCATTTCAGCATATGAACCCATACATGATGGTAGTGTAAATAAATATGTAG

GCAAATTTATGCAAATGTTTGTGTGCCTTTATGTATGGGTGTAATTCTCATTGTGTGTCT

CCAGTCCCTTTCAATTTGTTTTTTATTTTTAGTTTATTTTTGTTCTGTGATTTTATTCCT

TCCTTTTCCCTTCCCGTATTGTTCGCGCTATTAGTGTTGCACGTCTTCAAGTATTGGTAA

ATAAATAAATAAATATATATATATTTCTTTGTTATCATTATCATTGTTGTTATTATCATT

TACTCACTGAGTTTATGTTTACTTTATTGTAGTGTTACCATTCATTAATTGTTCTCTCTA

ACATTTATTTACTTTCACTATTTTCATTATTTTAATTTTATCTTCCCAGGAGGTGGAGAT

CGGGGGTTGAATAGGAGGAGGAAAGTTCTGCAATAAACCTGAACTTTCTTTGTTGTTTCT

CTTTTTTATTTAAG

>Tb927.5.3690 | Trypanosoma brucei TREU927 | hypothetical protein, conserved | genomic | Tb927_05_v5.1 forward | (geneCodeEnd+0 to geneEnd+0) | length=800

ATATCTAGCTTCTGCAATGAACTGTTCAGGAAGACTTCGAGGTATTATCATTGATATATA

TATATATATATATATTAAATGTTATCATTGTTGGATGCGCATGTCTTTCATTTGGTGATT

TGCAAGCACTCATCCAAGCATATTTTATAAGACATGTTGGGTCACAACCCGGGGCTTCGA

GGCATGAAGGTTGTTCAGCCAACGAGAAGGCTCAAACTTTACTACTGCTGTTAGTAATCA

GAGGGGTGGCCACGACATGTTTGATAAAGATATCAATCGAAAAGAAAGAGTATTGAGGTG

GAGGTAACATGTTCGCCTTCAGCAAGCATCCTTTTAAACGTGTGAGGGGAGCTGAGAAGT

GACGGATTAGTCGCCTCCTCGATTTTGTATTCTTTATGTTGAAAATAGGGGTGTGAGAAG

AGTTGTGTGTAGGACATGTGCTGATTTTGGAACCCCATGAGGAGTTGTGTTTGCTCCGAA

TAATGGCCATTTATCGTTTAGTGGGGGAGCGTACGTGTCGTTGGTTGTGTGAGTGTCTGG

GAAGTTTGTGTGTGTGTGGGGGGGGGGGACTTTACTGAGGTTTTGTGAAGGGGAATATCG

CTTAGATGCGTTTTACTGATTTAAATTCTCATCCACAGCTCTTTCCTAAAGCTGATGTGG

TGTGGCACTGCGTCCTCTGATTACTCGCGAACCTTTTTATTTTTTTTGTTTCCTAAACTT

ACAAACATCAAGGTGCTCCCAAAACCTGTAACGTGGAGGGCGCTTCCTCAACTTACTGTC

ACAGTGTTTGATTTTGTATT

>Tb927.5.3540 | Trypanosoma brucei TREU927 | Meckelin (Transmembrane protein 67), putative | genomic | Tb927_05_v5.1 forward | (geneCodeEnd+0 to geneEnd+0) | length=256

ATGCTTAACCGTTTTCTTGCGTTGCCATCTCCATTGTTATTTTTTATTAGTTATTTTTTT

TTTAATTTTCCTACCTCGGAGCAGCAGGATGAACAGGCCCCGTGTCTTCTCACGTCGCCA

CACATAAGTAAGGAAACTTATTTGTCTCTTGTTTTCAGGGATGTCCAGGTGGCAGAGTTG

GAGTGTATGTCTGTTTACGCGTGGTTCTTTTTTAGTTGGAGTGCAAAATGGCTAAGGAGA

GGGACAGTGAGTAATG

>Tb927.9.9450 | Trypanosoma brucei TREU927 | zinc finger protein family member, putative (ZC3H28) | genomic | Tb927_09_v5.1 reverse | (geneCodeEnd+0 to geneEnd+0) | length=110

ATTAACAATGGAGTAAGCATTGACATCTAGGGGGTAGGCAATGATACAAATAAGCGAATA

CCGAAAAGGACTAGGAAAAGAAGAAGATAATATAATATAATCATTCGAAG

>Tb927.3.3310 | Trypanosoma brucei TREU927 | 60S ribosomal protein L13, putative | genomic | Tb927_03_v5.1 reverse | (geneCodeEnd+0 to geneEnd+0) | length=41

AGGCAGAAAACTCATGTTCCTTTCCTTTATTTTTCTAAGTG

>Tb927.8.6610 | Trypanosoma brucei TREU927 | hypothetical protein, conserved | genomic | Tb927_08_v5.1 reverse | (geneCodeEnd+0 to geneEnd+0) | length=290

GCAAAGGTGTGTGTTTGTCTTGCTTCTAATATGATGAGACCCCGTGGGTATATATATATA

TATATATATATATATTTATTTATTTATTTATGTATTTCCCCCTCTCCTAATAACCCTGTT

TTCTTTATTTTCTTCATTCTTGGTATATGTATATATTTTTTACATTTCAGGTTGCCTCAT

TATTGTTTGTGCCCCTGATGGTTGCGAGTCGTGATTTATTATGTTTTTTATTTGCGTTTC

GTATTTTTAGATTTGCACCAGAGTAACTGCAAACCCTTCCAACGTGTATT

>Tb927.3.1570 | Trypanosoma brucei TREU927 | protein kinase, putative | genomic | Tb927_03_v5.1 reverse | (geneCodeEnd+0 to geneEnd+0) | length=1340

AAAGGGAAACAAAAGGGAGAATCCAATTGGATTGTTATTACGGAAGTTAATTTCCATTCC

CAGTTTAATGAATTTTGTTGTTGTTGTTGTTTTCTAAAGTGTGTACAAACATACGATACC

TCTTTTGTGCCACTGGACACAACCAACTCATGGCGCTCTCCTCAGCGCATTATGTCGCAG

CCAGGATGCTGCTGCACCACATATCATTCCAGAACCTTGGAAGGGAAGGAAGGTGTATGT

AAGTGCGGTATATTTTGTAGTGTGGTTGTGACGGTGATGTTTTGTTGTTTGTTTTTTATT

TCCCTTTCTACTCTGTGAAACACTAAACACTTATTTTAAAAGAATGAGTTGAAAAGATTC

CCATAACTGTATGGTACACACCTCTGATTATTGTTCTGGAGTGTGTTGATAGGCTGACCC

AAAGTAGATATGTGTGAGTGGCGAAGGGAAGGGAATGGAGTGGAGTGAGATAAATAAATA

AAAAAGAAGTGCGCTGAATGGATAAGCCAAAAGTTAGTCTATCCGTTTCTTGTTTTGTTT

TCCCACCCTTTATACTATTTTGATGAATGCTTTACTTCACATACTTTTTTTAAAGAAAGA

CCTCACAAACTCAACAGAGCGCAGTAGTGAAGTGCGTCACTTTTTCGTTGTTGTTATTGT

TGTTGAGGGTCCATGTACGATTGACCCTTATATTTCCTTTGTGGTGTTTACTTTCGTACT

TTTTATTGTATATTATGCAAATACGGGGAATTCATCAGTGGATTGGGAGGAAAACGTCAC

GCGTTCACACACATTCTTTCCATCTTTTTTCTTTTTTTACATTCCATTTCAAACGCATGT

GTTATTATTAGTTCGCGGCATCTTTTTCTCGTTTGTGAGTTCCTCCTTTTTTTTTTTTGC

TTTTCCTTTTTAAATATTTGCGCTCTCTCTCTTTTCCAGAACGGTTCTCCCTTATTTATT

TTTTTGTTTTTTTTTGGTGTATCAAAACCACCACACATATATATATATATATATATATAT

ATTCCTTCCTGAAGTTGTTAGTTGCGTGTGTGGTGACTGGTAGTAAAAGCTCAGTGACGT

TACGCCTAGAACACAAATATGCCACACCTTCCTACTATGATAATAATGATAATAATTATT

ATTTTAATTTCTTTCTCCTACCTTTACCTTTACCTTTCTATTTTATTTTTTTTATTTTTT

ATTAAAACATGTACCTTTTGTCGCTGTGTTGATGCCCATGTAAGACTGCAACAGGTACAC

ATCTCTGAAGCACCCACTCACATAAGGTAAGCCAAAGTGTATTTATTTGTTGTTTTCTCC

TTTATTTACCATTTAAGTTT

>Tb927.8.880 | Trypanosoma brucei TREU927 | hypothetical protein | genomic | Tb927_08_v5.1 reverse | (geneCodeEnd+0 to geneEnd+0) | length=1713

ATGGCAATTTTAATTTATGTTTTGTTGTTCTACTCTGGAGATATTTCAGCGCCGACATTT

GGCCTCGTGTTTGTTTTTCCATTCGCCCGCCTTTCTTTCTCCCCCTCTTTTGCTTACTGA

GTGAAACAAAGCAAATGGTATCTGCAAACCCCTGATGATAATAATGGTAATAATAATAAT

AATATTAATTACTTTTAGTATTGTTTGGAGCAGAATACTAAAAGAAATGAAAAGGAAAGG

AGGGGAGATGGAGGAGGAGAGAAGGTTTCTGAAAAAAGAATATTGAAGATAGGAAAGAAG

GGGAGTCAGCGAAAAGTATAGGAAACAAATGTGACTTTTTGCTTTCCATAATCGTTGTTA

TTGTTGCTGGTGTTAGGAAACGGTGTATCTCATTTAAAGGAAATTAATGTGAAGAAGCAG

AAGCTGAAACGATAGATGGGATTCGGGGAGATTTCTCCATATTTGTAAACTTGTGAGCCA

CTTTTACTTCTTTGTGCGTGTGTGTCATTCCTGTTTTTTTTTTTTGAGGGAGGAAAGGTG

ACTTTTCTGTATTTTTATGCGTGCATGGCGTCGTTTGCCTGTTCACTTTTTTTTTATTTT

TCATTTTTATTTTTCCATATTATAGTTTTGTTTTTTTTCCCCCTATTGTTTTTTTTGGTT

ATATTTACTTACTGTTTTGTTTTGTGGTTCACTCCATGTTTCGTTCCACCTTGTTAGTTT

TTTTTTGTCGGTACGAAAGAGAGAAAAGGGAAGGGGGGGGGGAAAAATGGAACAAAATAA

AATCAAAGAAGCAGTTGAAATGGTGAAGCAAAGAGGTTAATTATATGAATTTGCGTGCGT

TGCTACAAATGAAAGGGGTAAATTAATGGAACGTGTATTTATTGGTGCACTTCCGTGTCC

GTAAATAAGTAAGAATGAAAATGAAAATAAACATATACATACACATACAGATACATATAT

ATACACACACACACACATATATATATATATATATATATATTCATGTATATTTAGTTGTAT

GTGAGAGCGAATATATTTATACATATATCTGTACGCATTTTATATTTTTGTGTTTATATA

AATCTTTGCGTGTGAGGTGCTGGCGGCATTGTTCTTTTCTTACCCTTTTTTTTTATTATT

TAGAAAACCATCAACTGGAGTAATTCCGGCGTCCTCGGGGGTTTCATATTTGCGTATGGG

TATTTGTTGACGTTATTGTTATTATTCATACGTTCCTTGTTTGTTAAATATTTAGTAGTA

CTAGTAGTAGTGCCGCTATCGTTGTATCTATAAATAATAAAAATAATAATAATAATAATA

ATATATATATACATACACATAAGGTAAACGCATGAATGAATGAATGCGTCTATGTTTACT

TCTTTATTGTTTGTGTTTCACAAGTTGATTACATGTGTTTCTCTTTCTTCACTTCAATTC

ACTTCTCCTTTTATTTGTTTCTTTTTCTTTTTCTTTTTATTTGTTTCCTATAGTTGCCGG

GCGTCCACTCGTTTAGTGGCAAGTCCTCCAACGCCGTGAGCCACCGAGGCGAAGGGTGTA

AACGAGGTTTAGACAGGCGGACGCAAAGGAAAAAAACAACAAAAACAACGAAAAGGAAAA

GGAAAAGGAAGGGAAAAAAGAAAGCACTTTTTTTTTTTAAAAAAAAGCTGGAAAACAGAA

GGGAAAGTTCAGAGGCGAGAACAATAACAACAG

>Tb927.10.8580 | Trypanosoma brucei TREU927 | ADP-ribosylation factor-like protein | genomic | Tb927_10_v5.1 forward | (geneCodeEnd+0 to geneEnd+0) | length=984

ATTGGAAGCTCCACCTCTTCAGGGCTTGCTATTTTTTTATTTTATTTTATTTTGCGTGCC

TCCCTCACAACTGGCCATGGGGCGGTCTGGAGCGTGTGGATCATAGGGGAACCCCCACGA

GCAATTGTCGCAACAGTAAGGCAAATCTGTGTAAAATGTTCTATTTGTTTATTCTATTTC

CCCCCTATCAGCTGTTTCAAAAGGCTTCAGATTTCCTAAAGGAAACACGTTTGCCTAATT

CGCGTGTGTCGTACTTTCAACCCAGCTCTTTTCTTCCGCTGTGCGCGAGACACACGTTAG

GCCCGCTCCATTTCCTACATTTTTTCCGCGTTCTCTTACTTTGTTTCATTTAAGGCCCCA

AAATGGAATATAGGATTAAAGTGCGGAACGAAATTCTGGTGAGTACGCACAACGGGCCCA

CGTTTGTTGGCTGTGGTTCTTTCCAGCCACTTTCTGTGCTCACATCACGTGCCGATGTGG

TCTGCGGAAGCAACTCTCGCCTCAGCGCACCACAGTACAGCAATTCGGTTGGTAATTGAA

GGAATTCAACTTGTGGCTGAGAAGGTGATGACAAAATGTTTTCTGCACTGTCCTCGCCGG

TTTTGTTTCTGACGGGTTTGACGGCGTGTGCCGTGCTCCTCCTCCCTTTTGTGCCGATTT

CGCTTGCAGGTGGTGAAGTGATTCGATGGGCGAACGCGTGGGGAGTGAATGCGGGGACAC

CTTGTAGATTTTTTTTTACTTCTCTAGCTTCTTTCTACTAATGGAAGTCCGCCTAATTGG

GGTTGGATACACGCCTTATGGTGTTGTTTTGATTATTATTCATAGTTGCTACTGGAGGCC

GGTGGTTGCTTTAAAAAAATTTTCCTGCGGTCGGCGCAGCCAACAGTTATTCCGGTCTTA

ACGACTTGTTCTACACCATCACATGAGTGGAAGCTTTTCTCATGGTGTTCACGCCTCCTT

TGTAGCGTTGTGAGGGAAGTTGAC

>Tb927.7.2420 | Trypanosoma brucei TREU927 | glycogen synthase kinase-3 alpha, putative | genomic | Tb927_07_v5.1 reverse | (geneCodeEnd+0 to geneEnd+0) | length=4079

ATCTGCTGGGCGGATTTGTTTTTTTTTGTTTTTTTTGTTTTATTTTCCCTTTTTGTGGCA

TGTTTGCGTTTTGGTGTGGGGATGGAATGGGGTGGGTAATGTGCACATGCTGTGTTTGTT

CCCCTATATATATATATATATATATATATGTTTATGTGTGTTTATGCTTCTGTGAGGACT

GAAAGAGGAAATGGTAATAATAATATTAATAGAAATAATAATGGCTGTGAAGAATATTCT

TGGGAGGATGAAAGAAAAGGGGAAAATAAGACACAAAAGTGAGTAATTAACACAATGAGA

TGGTCGTACATATGTCAGTTTTTTTAGCGTGTTGAGGGTGTTGCGTCCATAAAAAGTGTT

TCGTTTCTTTTGGTCGTTTTAACTCTTAGCGCAATGGCGTGTGATAGTTTTGCAGATTCC

TTCTTTTTTTTCTTCTATTTTTGTTACGTAAACGAGAGACATTTCTCTTCAGCACGGCGA

TTCATTTCTGCTAAGTTTATGTGCGTATGTACTTATGGGTGCAAAGCAAGAGAAGATATA

TGTGGCAGCCCCTTCCGTTTCTTTTTTTTTTCTTATTGTTGTTATTGTTGGGGGTGCAAA

GGACTTCGTTTGGAAGGAGGGGGAAAAAAAAAGGCTTGAGCAGATTTTCGTTTTTTTTCT

TTTTTAATTTTTTGTGTTTCACCTTCACGTTGAGTATAACGTCACACATATTTACGCATT

TGTATTTTTGCGTGTATATATGTATATATTTATATACATATATGTACTTATATATTTATG

TTTACGTGCATGCATGCGTGGGCGAGTGCGCGAGTCACTCGACTGCATCGTAATGGAAGA

AACAAAAAGTGTAAAAAGCAAAAAGAAAAAGAAAACGAGGGGAGTAAAGGGATTTTATAT

TGAGGGCGAGGAATCCAATGAGTGTAATTAACATTGAAATAATGTTCCTTTTCATCTTTT

TTTTTTTACTTTATGTCTCTCGTTGTACATCTTTTTTTTTGTATTTGGAGAGAGGAGTGT

TTTTTTACTTTTACTTTTTTTTTTTGATTTGTTATCACCTTTCCTCGCCCAGTTGGAGCT

CCCTCCTTTTTACTTTTACTTCCTTTCTTCCGCAATATATATATATATATATATATATAT

TTACTTTACCAACATATTTACATGTTCCAAAAAATATATATATATAAATATGTGTTTTTT

TTACTGTTGTTATTTTTTACTTTTTCACCTTTTCACCTTGTTAGATTGATGATTATGTCG

TTGTTGTTGTTGTTTTATACCTTTTGAACGCTCCTCATGTTTTTCCCCCTTTTTTCCTTT

TTTTTATATGTTCGTGTGTTTGTGTTTGATTTCAGTAATCTCTTGTCAAAACCACTTTCA

GCAAAACAAAACCAAACGAAAAAAAATAATAAATAATTAAAAAAAAAAAGAATACCATCA

CTACCACTGTGCAGTTGCCGCTACCGCCATCCCTTTTCCATTAAATTAAAGGGGGGGGGG

AGAAAAAAAAAAGAAGAAACTAACAAAAAGTTCATAAAGCAAAAGAAAAAATATATATTT

ATATATTTATATTAATGGTGTTAAGGCGAACACAAATCATCATTTAAATTTATTTATCAC

TACTACCGTCATCTTTTTTTTCTTTTTTAAAAATTAATTGTTATTCCCTCAACCCCCTTT

TGTTTCTTTTTTTTTTCGTGTGATTAATTTGTGTTCGTGTGTTTTTACACATGTATACAT

GTCTGCCGGTGGTTTCGCCTTTGGTTTTTCATTCGACTTTTTTAAAAAAAAAAGCAATTG

TGTTTTCTGGTTTTCGGTTGATGTTGATGTTGATGTTGTTGTTATTTTTTTTTTCCCCTC

CCCCTTTTTGTGCCTATTTAATAAATAAAAAAATAATTTAAGAAATTAAATAAAAAAGAA

AAAAAAACCTATCCGCATTCCCGTCATATGTTTATTAATCGTTGTATATGTGTCATGAGT

GTCATGTGTTTGTATGTTAATGTCACAAAAGTTGTATGTAAAATATGTAAATTTAGTATC

CATTATGCAAAAAAAAGATTTTTGTACAGTAGTTAAATGTTTTGTTTTATTTTTATTTCA

CGGACAAGGTTATGTTGTTTTTGATGTTATTGCAATAGAGAAAGAAAAATATACATAAAT

ATAAACACACATATAAAAATATTAAAATATAAATATCTAAATAATATATATATATATATA

TAAGAAGTGAAGGGGGAAAAAAAGGAAATGTTATGATTGTGTATTATAATGTTATTTTTT

TTTCCTCTTTTCTTCATTTCATCGTGTCGTTATGATTCGTCTCTCTTTTTTTTTTTTTTG

TCTTTGTCTTTTGATGAGTTGACTGCAGAAACGAGGAATGCGAAATTGCATGCTTTTTTT

TATTTTTATAGCTTTGTTAGGGGGAGGGAGGAGATGCAGCGTCACACTCCGATAATAATA

ATAATAATAATATTGATTATGGTTTGTTACTTTTATATTGTTAATAAGATATATAATATG

AAAAACAATATATATATATATATATATATATATATTAAACAAATATCACCAACATATACG

TACAAATGAATGCAAAGGGGGGGGGGAGAAATACCGAAAGAGGAAAAGAGCTAAAATAAT

TTTTAAAAAATAAGTAAAGGAATTCAACGGGAGGGAGGGAGAGACAGTTTTGAAGAGGGA

AAATGTTAAGAAGATGAAAATGGTGAATGGCGTCCATTCCTCGCATTATATATGAGAAGT

TGCGTCTTTCCCTTTTTTTTTAAATTAAAATTTTTTTTTCTCTTTTAGTTTTTTTTTTGT

TTTTATTTACCCCCTTCCGTTCGGTCCTCTTTTGATTGTTTGTTTTATCCTTCCCAATTG

CTGTTGCTTCTGATTTCGAATATTTTCCTTGATTGTCTTTCTTTTTGTTTCCTGCTATAG

TAAACAGTTTTTTTTGTTTTTAATTATTAATTGGCGGTTTCTTGTTTATTTTTATCCCTT

AAATGCTGCTGGAGGTGTTGCTTTGATGGGAAAGAGAATGAAGTAAGAGGTGTTTAGCGT

GATATATACTCAATGATGTTACATCTACCTTTGTTGTTTTTTCTTTTAATTTTTCAGTTT

TCAGTTTTTTAGTTTTTAGTTTTCATCTTTTTTTTTTCTTCTGATTTTTTTTTTTTAAAA

AGGGGGTTTTACTTTTATTATTTAGGAATGGGAAGAGGGAAATGAGGAGTGCATATGATT

GTAATTATATTATTAACATTACGGAGTGGAATTCAACTCCTTCTAATGTTTATATCGTGC

CTTACGTTTATGAATATGAATTGAGAAAATGGTTGTTTGTATTATAAATTTCCCATGTTG

CTTCTTTTACCCTTCATCTTCTTTTTTTTTTCTTGTTGTTGTTGTTGTTCATTTAATTTT

TTGTTGCTACTATACGCGACATGTGATGTCCATGTCCTTGAGAAAATGAGCGCGTGTTGC

ATTGCGTGTTTTTTTTTTTCCCCCTTCTTGTTTGTGTGTTTGTGTGCAGGGGGTGGGGAG

TTAATGTCGGCAAAAGAAAAAAAAAAGATGAAAGATATGTACCGAACAGGAAATATTTTA

GGAATACAAAATGATGGCGAGATTAAAGGAAGGAAAGTACACGTGTGAAGTTTTACCCCT

CCTCGACTCACCGGTATTACTGAGCAATTAAAAAAAAAAATGAATTTTGCGGTCCTAAAA

CGCTATATGATGCGTATTAGTCGGTCTGATTTGTTTGTTTGTTTTTTCCTTTTCCGCCAT

TTTTGCCTGTAAAATATTTCATATTACTCTTTCAGGTATTTTTTTTTTTTAACTCCTGTT

CCTGTTCTTGTTCTTGTTGCTTTTGTTTTGTTTTTATTTTTATTTGTTCCCCGTCCCTCA

CCGTAATGTTTACCAACTTTATTTTCGCTGAGGATCAGTGAAAATGTTATTTTACCGTAT

GGTTTCCTTTTTTCTTCACGTGCTGATTATCATATGGTGATGAAAAAAAAAAAGGGCGAA

GACAAAAAGTGAGAGTAAAGAGGCGAGGCGAAACAAACTGGACAGAGTAGCTGGTAAAC

>Tb927.10.5880 | Trypanosoma brucei TREU927 | Proteophosphoglycan, putative | genomic | Tb927_10_v5.1 reverse | (geneCodeEnd+0 to geneEnd+0) | length=704

GAATTGACTTTTGCACTGTTTCTAACATTATTGTAGCGTGGAAATACGTTTTCACTTTTT

GTTGGTGTAGATGGTGAGTCACAAGTGAAGAAACATTTTTTTTTCCTTCTTAAGGTGGGG

TATTGGGAGTGACGCGCGGAGGATTTCGCCACCATGAGGGCTGTGAATCGCAATAAAGGG

GTAAGGGTTGCAGTGGAGAATGAAAAAGGAGAAACCACATGCAACTACGCTGATCCAGAC

GTGAGGATACTAGTGCGTTGGTTTTTTTTCTTTTTTTTTTCTTTTTGCGTGTGTTTCGAG

GAAAGTCGAGAAAGTCAGATCAGGACAGCGGGACGCATAAGCGGAAATAGGCGTCGAGCA

ATATATGTTAAGGGACTTTGCCCACATTATCTCTTGCTTTTCTCCACTTTATTTCGTGTG

TGGTTTGGACCCCTCTCGCGTTTCCATGCGAACGTTTTGTGTTTTTACCTAACGCAGCAA

GCAACGCTAAAAGAAGCAAGTGAAATGGAAAAGAGGGGAGGAAAGGAGGACGTACCCGCC

CGTAGTCCTTCTCTTTTTTTCCCCCTTCCCCTTTTCGCTTTCTTGTCATTAAAAACTGGG

GGAAATAAACATTATGTACACATGACCGTGCGTTGCGAAACACATTGAAGGAAATGAATG

ACGAATGAACTTGAGGGAGGTAAGAAGGGAACGGGAGGGTTGGC

>Tb927.10.7040 | Trypanosoma brucei TREU927 | hypothetical protein, conserved | genomic | Tb927_10_v5.1 forward | (geneCodeEnd+0 to geneEnd+0) | length=940

AAACAGGGGGAGCGAAGTTGGGCAGAGAAAGTGGAAAAGTCGACAATGCCACGTGGATCT

ACTTCACATCCCAACGTTGCCCGTGTTTATAAGTTGGCCCTCAACTTATTATCGACCTTC

TTTTCGCGCACAGGTGTAACTTACTGGTGCGATATCGGTGTCTCTCTACTTCCCTTCTCT

CCGTCTAACATATATATTAAGTGATTAGGTCCATTTTATTTTATTTTATTGCTCATCTAT

TTGTTCTGTTCCCCTCGCTCATTTGGTTCGTTACGAATGTCGTGATCTTTATTTTTTTTT

TCAGAATATATGATCGTCAATTGAAACACACAAACAACGATGACAACAGTGAGACAGAGC

ACATGATTGTTGCTTGGTTTCTTCACTCCCATAATTTGTGGACCCACATCATATACGTAT

GTGGGGAACCATATGTATATCTATTTGCTCCTTTACACATGTGTATATCTGTGTGTGTGT

GTATTTTTATTTTATTCTACCTACCTACTTACTTTAAACAATCCACACACTCCCAAGAAA

AAAAAGGGGGCCTTGCGATGGTGTGTGCATATGTTTGTGCGTAACATCCTCTGCGAATGT

GGACTCTCAACCCTGTGATCAAGAAGAAAGGAGAAAAAAGAATTGAAGTGTTGGGTAATA

GCTCCAAAAGGGGGGAAAGTAGGTAAGACTTTCGACCCGCGCGAAAAAGTGGATCTCCAT

CGTTTTGCTTTTTTTTTTGTTTTTTTTTTTGCAGTTGCCATTGCATTTTGTTGTTATACC

CACTATCTCTTTCCTTCAAGCGCACTCCGGCATCGCGGTGTGTCTTACTCTAAAGGCATA

CCCCAACCCCCCCCCAAAAAAAAAGATACAAAGCGGATTGACACTAACGTCTCGCAGCAA

TCATTTTCTTCGTTGGTGTGTAAAAGAAAGAGAGAGGAAG

>Tb927.10.2970 | Trypanosoma brucei TREU927 | hypothetical protein, conserved | genomic | Tb927_10_v5.1 forward | (geneCodeEnd+0 to geneEnd+0) | length=1585

GGAAGGAGCAGGCGTCATTCGCAAGAGCTCTACCACAATGTTAACAAACATGTAAAACCG

CCATTATATATATATATATATATATAAGCAACGAGCGTAACGTTTTTTCTTTAGCTAAAA

TGGTCTCAATTTCCTATAAAACGTTTCTTTTTTTTTTTTCACCTCCAAAATATTATCACT

ACTGTGACAAATCATGAAAACTGCTATTATTGTTATTTTTTATTTATTACTTCTTCATTT

CCACTCAAAATAATATTTTTTCATGCACTTTCCATCAGTTGCAACCCTTCCTCACCATGT

CACACCAAAACGGCCACCACTCCAACAAGGTTTTCTATTCGGGTCACTGTGGAACTACGA

ATCCGACACAACTGGGGCAGCACGTGGCAGGACCAAGTGAAAGGCAACGCGTGCATTGAA

ACAGTGCTTGATGTGGGCCTCTTGATATTTGTCTGCAGAGTTTTGTCTTTGTTCACACTT

CGGTGGTGTTGCCTTCATGTGCATGTTCCCCGCATTTGTTACTTACTCTCGTCTATTGCC

TCTCTTTGGTTTTTAACAATAAGGCAATATCATCAAATACTGTGCAGGGATGGACATAAA

AAGGAAACTGAATTTGACCTCGTTGGGGTGTGGGTTTCATTTGAGAGGGACTGTAGAAGT

CTCTCGGTAAGTCAAGGTTGGATCGCACTGCTACGGTACTACCTGCAAGTAACGGAACTC

ATCCTGTGCAACCTGCAGAAGGATAAAGAAATTTGGGATAATGCCCACACGTTGTTAAAT

ACTGCATTTACTTTTGTGCAGCTGCGGGAGTACTATGAGTCGAGACATAGAAGTGACGAA

GGTGCACTCGCTGCTGATATACGAAAGTTCTGCCGGAACAACCTAAACAATTTGGTTCCT

AATATACGACGGCTCTCACAGTCCTTTCCATCCGCACAGGATAGCACGGAGGTTAGGAAG

CTGTCCCAACGAGTTGAGTGGAGAGACCTTCTACACAAACTCCCAGAGTCAATTAATTCT

GTGTATTTTGAAGCAGAAGTGAGGGAAGCCAAGGAGTTTGCCAAAAGGGGCGTCGAGGGC

AGTGAAAAGGCTCGGGCAGAGTCATTGAAGAAGCATCTCGAACAAATTGACAATATGTCG

ACCGAGGACACTTTGAGGAATATCAATAAAACCATCAACCTCTCTGGTGGGTGCTGGTAT

AAATGCCCTAAAAGACACCTTTATGTTGTCGGAGAGTGTGGCAGCCCTGCAGGGGAAGGA

ATAGGTGGGGTGGGCCACATCTCATTCCCTGGCAATACATCTGTTGGGAGGTTTTAGGAA

CCGGGAGGGTGTCATAGTGGACGAACTATTTCCTGTTGTAATGATAAGTGGAATGGAATA

TAATGTAATATGTGATGATGTGAAGTATGGAGCATAATTTGAATGGAACTAATGATGGAA

TGGGGTTTTTGTGGATAGGAGTGCATCTCCTGTTGATCATGCAATCCATATCCCTTAATG

AGGGCACAGCACTCCTTATTAGTACTTTTACTTTTACTTTGCTTTTGCTTTTTCTTTTTT

TGCTTTTGCTTTTGCTTGTTCTGTG

>Tb927.3.3960 | Trypanosoma brucei TREU927 | RNA-binding protein (DRBD6A) | genomic | Tb927_03_v5.1 forward | (geneCodeEnd+0 to geneEnd+0) | length=2218

AGCGACGCCTTGCCTACCCTTTGCCTTGCCTTTCTTTGCCTACCCTTTGCCTTGCCTTCC

TTCTTTCCTTCTTTCCTTTATTTCGTGTTGTTTTTTTTTGTTTCTTTTCAAAAAAAAAAC

AAAGGAAAGTTATGTGGTGATATGATTTGCATTTTTTTTTTCATATATATATATATATAT

ATTGAATAAGTTGTGCCGTTTCTCTGGCATGCTAGCTCTTTAGTTTATTATTATTATTAT

TACTGCACCTTCAGCAGCAGTGGATGAAATGAAGGGGAGAGACGGGAGTATTTGCTGATC

CGTTCTTTTTTTTTCATTTTATTATTTTCATTAGTTTTATTATGTTTATTTGGACCATGT

GCGTGGGTCTTTATTTATTTCAACGTCGTCTCCTCTCATGATTTATTCACCACATTTTGT

TTGTTTATTTTTATTTTCCCCTCCTCTCCTCCCCTCTCCCGTATTTATTTTATTTGCTTC

CAGTATTTCCCTTGAGTCAAGGGTGAGGTGACAGTCATTAATGACATCAATAATTTCCTC

CCACTTGCCTAATGACGTTGAGACGATTCTTTTCGAGTCTCTTTTATTTATTTATTTATT

TATTTTTTCTCTCTTATTTTTATTTATTTAACTTTATTTATTTAGTTTTATTGCTCTCCG

AGTGTTTATTATTGTTAATATCATTTATGACGTTTGATTTATCCTTTTTATTTTTTATTA

TTTTTGAATAGAAGGCGACTGAAAATAGGAGGGGGGGGAATTACGAAGTTGAGCGGGGTT

TTGTTATATTTGGCCCCTGCAGTGCTTGAAAAAAGCTCAAAGACAATAACTTCGGCTCCT

CCTAATCAAATGAAATGACGCTCTGAATAAATTCCCGTTTATTTTTTGTGTTTATTTATT

TTTTTTATTTGCTTATTTTGTTTGTTTTCTTCAAGGTTATTATTATTATTACTGTTACTA

TATATATATATATATATATATGATATTTATTTTACGTTTATTTTTACGTTATTAAAAAAA

AAAATATAATGATTCATTCATTTTATTTTTATTTATTTCGTTTAGTTCTGCTTGTGAGTG

ATTTGTGTATCTGTTAGAGAGGATAAATAAAATTAGAGAGAAGAAAAAATAAAATAAAAT

GAAAAAGAAAATGAAAGGAAAGAGGGAAGAGGAGGAAAATAATAATAATAATTATTATTA

TTATATACAAGTGATGGGACTGGCATATTTTACATAAACCAGTGTGTGTGTATGTATTTT

TATCTTGTTAATATTTTAATACGTACGAATTGATTGACAATGATGATGAAGGAGGGAAAT

AAATAAATAAATAAATAATTGAAACAAAATCGAGCCGAAAGGGAAAATGCAGAGGTGACA

ACAACAATAACAAAAACTGGTAGCAAAGCTAATATGAATGTCAATGAAAATAATGACAAT

AATAATAGTAATAATGACAATTATATAATATTAATAATGACAATACCGAAAAAGGTATTA

CTTTTGTTTTTGTTTCTATTTACCCTTTCCTCCCTCCTCTTCCCTCCTCTTTTCTTTTAC

CCATCAGCCGAAGTGTTTGTATTTTTTTTTCCTGTTTATTATTATTATTTATTATTATTG

TTGTTATTATTGTTGCGGGTTTTTGTTACTGTTTTTGCTACTAATGTTGAGCTGTTCCTC

ATTTCCTTTCCTTCTTTCTTGTTTGTTTGTTTAAGTTTCATTATTATTATTATTTATTTA

TTTTTTTAAAGGAAAACTCTCTTGTTTTAATGAGAAGTGAAATGCGATCACATAAATCAA

TAAATGGATGAATTAAAAGAAAAGAAAAGAAAAAAAATGCCTGATTTTGCGTGAACTTAA

TCTCTCCACATTTATTTATTTCTTGTGTTGTTTATCTTATTCTTTACGTAATGCAGTTTT

AATGGAGCAATGGTGAGGAGATGACACACAAATATATACAGAATCAAAGTCCCGAAGCAA

AAGAGAAGGAGAGAGAGGAAAAAAAAACGGTAAGATATTTAAGATGACGGTAGATTTTTT

TTTTAAAAAAAAGGAGAGATAAAAGGAAATAACAAAAGAAGTGAAGTGTCAACATAAAAA

AAAAAAAGAAAAGAGAAAGGAAGAAAGAGAACAGCAAAGAAATGAGGAAAAAGAAATATA

TAAACACATAAATGGTGAAAATGACATTTCATCCCTTTTTTTGGTGATGCTCATATTT

>Tb927.11.11110 | Trypanosoma brucei TREU927 | serine/threonine kinase, putative | genomic | Tb927_11_v5.1 forward | (geneCodeEnd+0 to geneEnd+0) | length=389

ATATTTATCCTCTTGCTTCATTTTCTGGTGATATAGTGATGGTACATTCAATGTATTATC

CCTCATGTTATTTTTTAAAACTTCCTTTCCTCAACTATTTGTTTTACATCTCTAAGTAAA

TTGTTGGTTTATTTTGTTCTTCTTAGCTTTCATTGAACTTGTTATTATTTTATCCTGGAG

AGTTGACACAGTTCTGTTGGTAACAATATTTCGTTGTAGTGTCTCTTCACTAGCGGCTGC

GACATGCTTTCTTCTCCCCACCTGTTCTGTGGTCAGGGAAGAGACTGTATGAATCGGGTC

GAGAAGACATTCGGCGGAAATAGTTTTTTTTTCTTTTTAAAGCTCTGCTTTTTATCGCAG

GCGCAGTGCTTCCGATCTTCTTGTTAGAG

>Tb927.7.6220 | Trypanosoma brucei TREU927 | protein kinase, putative | genomic | Tb927_07_v5.1 reverse | (geneCodeEnd+0 to geneEnd+0) | length=50

AATATTAAAACAACACTTGCAGATGATTGTAGTGAAATGAGATGAAGAGG

>Tb927.3.5080 | Trypanosoma brucei TREU927 | PSP1 C-terminal conserved region, putative | genomic | Tb927_03_v5.1 forward | (geneCodeEnd+0 to geneEnd+0) | length=2875

AGCATCTCTGACGCTCATTTCTCCCTTTTGAATTGTGGAGCGAGGGAGATGGTTGCGGCG

ATGTGCAAACGGTGAATGCACAAGAGAGAAAAAAGCGAAAAAATAATAATAATTCTAAAG

TGTCTGTAGACATGACTAAGGAGAGAAAACCTCTCCCTCTACATTTCACCTTATTGTATT

CCATTTTATTTCTTTACTTAACAAAAAAAGAAAATAACTGTAAGTGACTTAACTATATTC

CCTTCCCTTTAAACCTGTTTTCATCCCCTTTCATTCCCTTTCCTTATTTTTCTCGTTATT

TTTAAAAAAAAAGCTGTGTGTGCTGCTGGGTGTGTTTTTATACATTAATTTATTTTCATG

TTGCAATATTTCCTCCCTCTCATCTCTTTTCTGCATTCCACGGATTCACGTGCGTTGGGG

TAATGAAAGAAGAAAATCTCAATTTCTGATGGTGGAGTCAAATCCTCCACTTTTTTATGT

TTTATGGTATGTTTTATGGTATGTTTTATTGTTTTTTTTAATTGTTTTATTATTTATTAT

TTTTTTTCCCCCATTTCAAAATTCGTTCGACCCACATTTGTGTTGATGCTTCGTTTCATT

GAACCCTTTTCTCCCTACTCTCACTCTCACTCTCACACGCACACGCACACGCACATGCAC

GATTATACGAAAATATGTGAAAGTTATATATTTATTTATGAATGTGCTTATTTTTTGGTT

AATGCAACTTCTCAACCCCCCTTTTCCCCGTTATTATTTCACATGTACCGTTTTTTAAGC

GATGCGACGCACTTACACTCTCTGCATGATGAAACGGAGTTTCGCGACACCCCATTTTAA

AATTTTTATTGTTTATCTTTTTTCTTTTTTTTGCTCCTATAAATGTAGTTTTTACACAAT

ATCACATAATATAATTTAATATGACTTCACATTATTTAATACAATCTCATTATTTTTCTT

TTTTTTTTTGTGTGTGTGTGTATGTGCGGTTAAACGACCCTCTATGAGGCAGTTGCTTGT

TGTCAATATCGTTATTTGTATTTTAAAACGAAAACAATAAGGGAACTGCAAAGGAACAGA

CAAAAAGGAAAAAAATAAAATAAACAACAAGAGGACACTCACAGAATAAACCGAATAAAA

TCCAGCATTCAATAATAAAACAATGACAACATCAACAGAAAACAGAACACAGAAGGAAAC

ACGAACAGAAAATAGGAGGAAAACAGAACCCGTTGACAAAAAATAAAAACAGAAGAAAAA

AAACACAGAGAGAAAAAGAATTAGTCAATATACCTCAGTATAAAATGAAATATTAACGGA

AAGAATATGTTATGATTTTGCCCGTTTGTTCTTTCTTCGATGCCGTTGCAATAATGGTAA

AAATGATAATAATAATAATTAAAAAAAACGTTTTCCCCTCCGATATTTTTATTTTATATC

CTTCCTCTTTTTTTTTTTTGCTTTGCATTTTGGTTTGCACATAACTTTGTGCAGTTCACG

TTCACACGATGGCGCTGCTGCTCACAGGCACTCAACGCATCCACTTCAAGAAGTGAGAAG

ATAAAAAAAGAAAATAAAAATGAAGCTATCAAATCTGCTCCACCCATACAATCCACGACT

ATTCTTACATACTTACATTTTTTTCTTTTAAATAAATAAATAAATATATATATATATATT

CTTTTCTTGTTGTTGTTTTTGTTCACATAAATATCAAATAACAAATAATAAATACATTCG

TTTTACTATTTATCCAATCACTTCCGATTTTCACAAATGACTTTGTTTCGCTTCGATACC

CTTCAAAACACCGCTGCGTGGGACGGATTCAAAAGAATAACTTGCAGACGACTAGCGGCA

ATGGGAGGAGGAAAAAAAAAAGACAAAAAGAACTGACGCACACGAGTTAGAAGTTGATAA

CTGTTGTGAGAAGGAAGAAGAAGAAGCAAAGGGGCGGAAAAAAAAAAGGAGACAAACAGA

GCAACAACAACAACAACAGCTAATTTTCTCATTATTATTATTATTGCTATTATCACTATT

GTTATTTTTGTTTTGCCGGTCACTAGAAGAATATACACCCGTATTGAAATAAATAAAATA

AGTAAATTTTAATTTGTTTTGTAACTATTATTTCCCATATTTTAAGCGACCAAATATTTA

CATCAAGGCAGAACACTTTGGATGATATGATCTCCAAAAGAAGGAAGATAATGCAATTGT

ATATGTATATGCATATGTATGAGTGTGTGGGTTTTATAAAAGAAAGCACGTGACACAATT

AATGGCAGAAAAGAGGGAGAAAATGCATAAAACGAAAGTCGCTCCAATGAACAATAATGA

AATATAGGACACAAAATATTAAAGATGGGGGATCAAATCGAAGGGCAGAGGGGCGGGGAG

GGAGGGCGGAGGAAAGAAAAAAAAAAGATAATAGCCTGATAAAAAGAAAATACGAATATA

TATATATATATATAACTATTTATATTTTATATGTTTTTTTTTCTTTTTATAACGTTATCG

CGTGATGACATGAATTATTGGGAGGGTGTGGGTTTTTATCGTTTCCTTATTTTCTTTTAA

TTTCAGAAAAAAGAAGTTGTTTTTGTTTTTTTATTTTTTAATTTTACTTTTTAAATTATC

AGGTATGTTCTCATTACCTCTCCTGCGATGTGACTACACGCCTTTATGTTTCACTGAATC

CAAATTCTTTCGTGTCTTTACTTCCATTTTTTTTTCTCTTATCCCCCCATATATTTACTT

TAATTTTTATTATTTATCGGAACATGACTTAAGCGATTACCTGACTTGGATATCAGTCAA

CTAAGCACGCACACACACACACACACACATAATCAAGTGAATTAGAAAGAAGAAC

>Tb927.5.3470 | Trypanosoma brucei TREU927 | hypothetical protein, conserved | genomic | Tb927_05_v5.1 reverse | (geneCodeEnd+0 to geneEnd+0) | length=182

ACACAATAGTGCGGGATAGTGCCGTTATTTTCAACAGATTATATGTGGTGATGTCTCATC

TGTTATGAGTAATTTTTTCTTGTGAACTGTCTTTTTTTTTCACATTTTTTTCCCAACTAC

CTCATTATTTTTTACTTCACAGTTTGCTCTCCGTTTTCCTTTTTGATACACGTTATACTT

TT

>Tb927.8.690 | Trypanosoma brucei TREU927 | PPIase, putative, peptidyl-prolyl cis-trans isomerase/rotamase, putative (PIN1) | genomic | Tb927_08_v5.1 reverse | (geneCodeEnd+0 to geneEnd+0) | length=1022

AGGTGATGTCCATTAAAATTATAGCCAGTGCACATTTATTTATTTTTTTTTATTATTTTG

CTTGAAATGATATCATTGAACGATAGATAATAGACAAGACTAACCGTCGGGAAGATGGAT

GTGTAGGGGCAGATTGATTATGTCGGTGGTTGAAGATATTGTTCAGAAGTGAGGGAAGGT

GAAGTTCAATTTGTGTATGGTAGCAGAAAAGAGGAACAAAGAAGTAAGAAGTGCCAGGAG

ACAAACTGATGCTGCCATCAGACATGTAATCACAAAGGTACATCAACAGCATTCTCTGTT

AGCCTCACGCTGACGTGGTTATCTGCCGTTATTCTCCTGAATTGTTAATTATTGTGCGGA

TGTATCTTTATGGAGGGGAGCGTTAGTCAGGCCTTTGTTGTTAATGTCGCTGAAGTTCCC

TTTCCCCGATTTTTGGTTTTCTTTTTGGTAAGTTTCTTACTCTAAATGTGCGTGCGTGTT

TGCAGGGTTCCAGTTACGACTATGCCTGGTTAACATTATATCTTCTTCCTCTCCTAAATC

ATCATCCATGCCCTTATCTGCCTCCTGTATTATTATTATTATTATTATTTCCCCCCCTAA

AGGATCAGTTCTATTGAAGAAATGAACCCGCGTGTAATAGCACCTGCAACACCACATGGA

AGTGGCGCGTAGGAAAAGTGGGTGAGTGATAACAACATCTGATGTTTGGTGCAGAGTAGT

GGTTGCAGCGACTATCCTTGTCCGTAATCTTTTCGGAAAATTTGTTGCCAGTGCGTGCGT

TCGAGGGTTTTTACGAGGGGGGGGGAAGCAGGAAAGGTGAAAATTGTTTTCCTTTTTTTT

TCCTCCTTTTGTGTGTGTGTGGTAAATAGGGTACTGCTGCGTTCGATGTATTGCAAGTGC

GTTTTTGCACACACTCTTTTTTAATAAAAGAGTGTGTGGAAAACTAAATCATGAAGATTC

GACGAATCACGGAGCTTTGATGCAGATAAACATCGGTTACTGCGTGTGTATGAATCTAAA

GG

>Tb927.1.2150 | Trypanosoma brucei TREU927 | calpain-like cysteine peptidase, Clan CA, family C2, calpain-like protein fragment, putative | genomic | Tb927_01_v5.1 forward | (geneCodeEnd+0 to geneEnd+0) | length=1798

ATTTTATGTGTTATTTTGTTTTTGTTTTTATTTTTTTTTTCCCCCATTACTTTACTTTAC

TTTATGCCCCTCTTTCTTTACGTGTTTCGTTGTGTTGTTACACTCATTCCGCGGGTACGG

TTGCGCGTTTATGCATAAGGAGGGTTCAGCGGAAATCGAGCACAAAAAGAAAAAAGAAAA

AAGAAAAGAAAAGAAAAGAGAAAAAAAAACGAAGTGAAGATGAAAATGAAAATGAAAACA

AAAAATATGAAAAGATGAGGGAAGAGAGAGTGAGGTAAGGGGCAGAGTGAAGGAGTTAAA

TGTGTGAATGGTGGCTTGTGTGGTTATTGTTAAGTGTTGTTCTCATGAAAAGAAAAGGAA

GGTACTTTTTTTTTTTAAAAAAAAAGAAAGAATAATAATAATAAGTCGCACGCACTCGCA

CATTCCTCCACGTCGCCTTTTTTTTTTCTTTGCTCTTTTTAAAGACCATCTCCCACTTCT

GCTACCGTCTCTCTCTCTCTTCTTTCCCATAGTTTAACTGCGCAAACAAATATTTGTTTA

TTCGAATATTTATATGTATTCCTTTCTTGTTTCTCTGTGTGTCTGCTCATTCAGGTGTGG

AACATTGTCTAGTGACATTCTTCTCGGCATCTGCCTCTAGTTATTTATTTAATGATATGT

ATTCTCAGCTTTTTTTTGTGTGTGTGTGTGTTTATTTGCTTCTTTACTTTTCCCTCTCAT

ATATATATATATATATATATATATTTATATTTATATATTTATATTTATATTTAAATATAC

TTTTTTGTTGTATGTGTATTTTTTTTTGTTGATTTATTTGAGTTTCCGCCTCCGTCATTG

TTTTGTTTTGTTTTTATCCTCAAATTTTACACACAGTTATTCAGCTCATTCACAATACAA

TACAGTACAATACAGTGGAAAGGCGGAGTGGAGAAAGAAAGAAAGAAAGAAAATCTGTGT

AACGTACGTAACTTCCCGCTGCTGCAACTGAGCGCTATTTGATTCTTTTCTTTTTTTTCT

TTTCCGTTTTTACTACTATTTTTTTTTAAATTTTTACACCGAGTTACAAATAAATAAATA

TATATATATATATACGTATAAATGTACAAGTAACATATGTTGGCATATGTTCATTTGTCC

ATTTATTTATTTTGTACGGGACTTAAAAAGAAGAAAAGGGAAATGAACTGAAGTGAAGTG

AAAGGGTGGAAAGAAGAAGAGAAGAGAAGAGAAGAGAGTTGAGAGCAAAAAAAAAGGGGG

AGAGAAAAAAAAAGAACTTCATCAAAGGCGGTGTTAATTAATTTGAAGTGAAAAGAAATA

TCGTATGAGTTGGTGTTTGGCATTTTTATCTGCCTCTTAAGTCTTTGCTTTCCATTAAAT

CTCTCTTTTTTTTTTCTCCTTTTCTTTCATTTACTCTATCTCTCCCTACTTATATATATA

TATATGTTTATTTGTTTATACGTATTTATTTCAATTTTATTTTTGTTTAGAGTTATTGGT

GTTTTTATTTTATTTTATTTTTTGGATTTATACGCGTTCCTGAGCCTCTTTTTATGTTGT

TGCTTTGTTTCAGTTATTTATTTGTACCTCCCTCGCGTCCGATAATAAATGATGACGATG

ACGATATGCTGTTTTCTTTATTTTTATTTTTTGTTTTACGCCTTCAGTTCCTCTTTTTCT

GTTGTTTTTGTTTTTTTTGTTTTTTGTTTTTTAATTCCGTTTCGGTCGAGTACTTTTAAT

GTGTAAATCTCTGTTTTGTTGTCCTGTCATGTCACCTCTGTCATGACTTTGTTTTTAT

>Tb927.7.4570 | Trypanosoma brucei TREU927 | inosine-guanine nucleoside hydrolase (IG-NH) | genomic | Tb927_07_v5.1 reverse | (geneCodeEnd+0 to geneEnd+0) | length=84

AGGAGGACGAGAAAGGGCGTTTCACCGCCCTCTTTCCAGCGACAAATCGCATGTCTCCGG

TATTCGACGAGAATCCTTCTTTTT

>Tb927.10.7320 | Trypanosoma brucei TREU927 | hypothetical protein, conserved | genomic | Tb927_10_v5.1 reverse | (geneCodeEnd+0 to geneEnd+0) | length=709

AAGGAAAAGTTCCCTCTGTTTTTGCGAAATCGACCATGTGTGCGGAAGGAGGGAGGATGG

AGAGGTGTTTGTGCGTGGGTTGTAGCTGGTGGAAGGGGAAAGTGGGGACGCATGGCATTT

CAACAACAACTTCCGTTGACCTTTGTTGTGCGCTTTGTTTTCACTTCAGTGCTCACATTT

TCCCGTTTCCCTGTCTTTTATTATTTTCCTGGTTATTTTGTCTTTAGCCTTCGCGATTTT

GTTTCTTTGTTCCACATTCTATTTTTTTCTGAAGTGTTTTAACCTATTTTTTCCCCCTTC

TTTTCTCATTTTCCAGGGTCTTGTTTTTTTTTTCCATGACCGCTACTAACGGTACCACTT

CTGGCGCTGCTACTGTTTACTACTAATTTTTCTCCATACTGTGGATATTCCTTTATTCCG

TGTAGTTTTGTGCGATCGTGCATCATTGAATCTCATTATGCAAATGTATGTATGTATGTG

TGTATATGTGTGTGATATGTGACTGTACATGAGAAGGGAGGTGATCCGCAGCCACTTATT

GCTGTGATCCCTAAAGGGGTGCTGAGTAGGGCACAGGGCAAGTGGATTATAATAAGATGG

AGGTGGTTTACTGTTGCCTCTTTGTTTTGCCTTTTGGTGGTGGCAAGCAAGGAAGAAATG

ATTTTAAGTGGGAAAGATAGGTATATGTACCACCTTTGTATCTGTACTC

>Tb927.11.12620 | Trypanosoma brucei TREU927 | Glucosidase II beta subunit-like, putative | genomic | Tb927_11_v5.1 forward | (geneCodeEnd+0 to geneEnd+0) | length=1278

ACCGATACATGAGATTGCAGGTAAGATATATAGCCGTGATAAACGAAACAACACCATGGG

GAGTTTGTGTTGGTGTGTCATTAATGTATATATATATATATATATTAGATAGGTGCGGTC

ATCGTGCGCACGTGAACGGCACAACCCTGAGGGATTATATGAAAGGGGAAAATGCCTTGA

ATATCACTAACCCTTGAGGGTAACATGACGGCTTATGTTGTACTTGCGAGAGAGTGGGTA

CATGCGGTCACAAGGCAGCACGCACGTTGGGGTCACAAAAAACACATATACTTGCATCTG

TATCGGTGTGTATGCATTTATTTATTTACTTGGTACTGCTGTTATTTAAGTATTTTTTTT

GTTTTTTTATTCAACTTCTAAACTATGGCCACTAGGCATACTTCACGAGGTTGCGCCCTC

GAACCCATCATTGTCACATCTTGAGGGTAGAAGCTTCAAGAACATGGGTAAAGGAAGAAA

AAGAGAAAAAATCTCGCGGCGCAGCAGCGCCCAAAGTTTTCACCTCCCCACCTTTTTGTT

ATCCCTTTCTAATTTACACACAAGACAAAACGTCTGATATACTCACCCATCTCCTGATAA

TTCCGGCCTCTTTGTTACACCTACAAGGCTTACATCCTCGTTTTTCTTTTCTTGTTGTGT

AGTTCTATTTTTTTTTTTTAATGGCTCGTATCCCGAAAATCATAACTTTGTAATAGCTCA

ACCTTGATTTATTTTTGTTTTGTTGCGTACACGTGTATCTTGGATATTGTACTGTTAAGA

GAGAAAATAAGGAGCTTTATATGGAATTGTTTACTTCATTGTGTATCTTACTTTTTTTTC

TGACTTTCCATTGTGTAATTCGCGGCAGCAGTGCAGTTCTATGGTCACATAATTGAGGAT

ATCAGCTCCCCGATTGTGATGCGTCTTGGGTGATTTTCCTAAAAGAAAAGAGAGCAGCTG

AAGAAGCGAAATGAAGTTCTAAGCGTCATGATTAGTAGTAGTACTAGTGGGGATAGAGTT

AGATGATTTACTCCACTTATCTTATTTTTTGTCTTATTTTCTGTTTGCCGAGTGTCTCGT

TCGAACAAAATGAAAAAGAGAGAGGATAACATGTTTGTGAACATTTGTAGTTGACGTCTC

TACACACTGCACCGCCTCCTTCACCCACACCTACATACTTCTTTTTTCTTATGTGACAAT

TTTGTTGAAGGGTTTCGCATCTTTTGAAGGAAGCGAGCAGAAGCAGAGGTGGGATAAGCT

TGCTGCAGGTTAAGAAAG

>Tb927.9.13610 | Trypanosoma brucei TREU927 | helicase, putative | genomic | Tb927_09_v5.1 reverse | (geneCodeEnd+0 to geneEnd+0) | length=472

GAGAGAAATGTGTTGGGTGAATGTGGGGAACATTTTTCTTTTTATTGGAATGAAACAAAA

GAACGCTCATCCTCTATTAAAGGACCTCCTCCACAAAAAACGAGGGGAGCGAGGGGAAAG

CAAATAAGGAAGGAAGGCGAAAATGAACGTGAACATGCTAATAGAAGGAAATAACGAAAA

CTTTCCAGTAACTAAAAATGAAAAGATAGAGATTTTCACCCTTCTTGTTGTTGTTATTTT

CAGTGGTGTTGCCATTACTACAGTCGCCGTAGGTTTCTTCAAATAAGTGTGGGAAAAGAT

CTATATATATATATTTCTGTGCGTGCCGACTACTCCTTTTTTTTTTTCCACGCCAATACT

CCTCGAGCCTGTCCAATTTATAAAACGGTAGCAGGAAAGGAAAAAAGGATAGGGACGGTA

TGAGTGTACGGAAGTGAAGATAGTTTAAGACAGCTAAATCAATGAATGGGTG

>Tb927.4.4220 | Trypanosoma brucei TREU927 | small GTP-binding rab protein, putative (TbRX2) | genomic | Tb927_04_v5.1 reverse | (geneCodeEnd+0 to geneEnd+0) | length=1708

AGATTTTTTGAGTGAGCAGCCGGCGTCTACTCGCAAAAATAATAATAACAATAATAATGA

CCGCACATAGATGTGTGTGGTGACTTACCGCCCCGTCTACGTTTCTGTTATGTGGTTCGT

TGTTGTTGTTGTTGTTGTTTTGAGCTGTGGTTTGCCTTCGCGTCATTGTCGCGCCAAGTG

TTTGCGACCATCGGCCCTAATGTGTAGCTCTCATAGCTTATGAAGTAGTTGAGGGAAGCG

CATTAAGAAAGACAGTAATTAATAATGAGGAAATGAGGGATGGTCCAGCTGGCAGCTCCA

CTGCCTCTTCGCAGCTGTGCGCATCTCCTCTCTTTTTTTTTTTGTTTTGTTTCCGCGACT

TTGTGGTTGGCAAAGACGTTTGGTCCGATCGAACTCGATTTTTTATTTATTTATTTATTT

TGCTTTTCTTCTCTTTTTTGCTCGGCATGAGCTACACTACCGCACATCTGTGTCTTCATT

ATCGAAACAGTGTTTGTGGCGGAAAAAGTGGAGGGGAATCGGTGGCTCATGTAATTAAAT

CCTGAATTTTGTCGTTTCTTTCCCTTCACTCAGAGCCTCTCTCCCTTACTTTCACTACTT

TGTCATGTTCATGTATATTTTTCCGCTTTTTTTTCCTTGTTTTTTTTTCTTTTCACGCTC

GGCCTCTGCAGTTTTCCTTTCCTTTTTCCCCCCTCCTGATGAACTCGCGATTCGTGTTTG

TAACGTTCAGGAACTGTCACCATAATAATACAATCATTTGTGGAAGGTTACGGCGTGACA

AAAGCAGATGGTAGTCCTTTAATTGTTCTTGTGGCTACTGTTATGCAGTTACATATGGAT

GGGATTGTGTTAAACATAGCATGAGAAGGAGGTTGTTGTTTGTTTGTTTTTGCATACGCA

AGGAAAAGGTGGTATATTCCATGTAATGCCTTTCATTTAAATTGGCTTTTTTTTTTCTCC

CAGATTCCCTGGACGGTGATGCTTTGAACACGCGTTGTTATGGGCAGTGCTTTTGGATCT

CACTAAACCCACCTTCGGCAAGTAATAATTTAAAAAAAACTGGAGCAAGCGCCTGCTGTG

CAAGCCAGTCGATATATTATGCAGGTAATCACATCATGAACAAGATTACACATATTTATT

TATTTTATTTGCGTAGAGTGAAGAGAACAAAAGGGAGTGGTCTGCCGTAAGAGCATAACA

TCTTCGAGGTAAAACTTCTTCGATATTCCCTTGTGGTCCTTCATTGACTATCAATGTTTT

GACGTGGGGAAGGAATAGTTATAGAAATTTCTTTTTCTTTTTTTTCCTTTTGAACAGAGG

ATTATTATGCGTTGCGCTTCCTCCGTTGTGACCAGTTTCATGACTGACTGCTTCGGCTGG

GAATAGACGTTTCTTTTTTACAACACCGTGTCGTGTTTCGCATCTGCGTCTCGGGTAATT

ATCACCTCGCGTTTAGATTATTATTCTTATCGGTGCACGTGTACGTATTTATTTGTCTTT

TTATCGACGCGTATTTGCATTTCTTTTTTTTCTTTTTTTCTTTTTTTTTACCTATCTGCA

CACCCGCCGCCACACCATTGGCTGCCCTTCAAACGCTCTCGAAATCAACTTCCCTACCAC

CACCACCAACCGAACATATGTCAACACATATAAACTACAGTGACAAAGGCAGTAATTAGA

TGTGGCGCATATAAAAAGCAACAAAACG

>Tb927.3.5320 | Trypanosoma brucei TREU927 | UBX domain-containing protein | genomic | Tb927_03_v5.1 forward | (geneCodeEnd+0 to geneEnd+0) | length=305

ATGTTAGCTGGAAGATGGCAATGCTAGTGTTAGCGATAGGCAACACAATTTTTGAGGTTA

ACCATCATTATTATTTCTATGTGGTAGCTGTTGCAAACGCTTATGTTTTTGTTTGAGAGG

GGGGGGAGGCCCAACTCTCATTCATTTGAGCAACCTTCTTCTCTGTGCGTGGGTATTTTG

CATCTGGAAGGTCGTGAGAATTTTTTTCCGCTCACGTTCACGGGTGGTTTCACATCGTGG

CCAACACCCCTCCCCTCCCTCCCCTACGCGCGCTCACTGACTGTAATGTTATCATCTTTC

CGTCG

>Tb927.2.5540 | Trypanosoma brucei TREU927 | hypothetical protein, conserved | genomic | Tb927_02_v5.1 reverse | (geneCodeEnd+0 to geneEnd+0) | length=1627

AGGCTGGTGTGCCTCCATTCTGACGTTTATTACTCCAAGCCTCATCTACTTGTCACTTTG

TGTGCTAGCATGAAGGTACTTGTGCACTACTGCCGAACGCTCGCACCCCTATGGGTCCGG

TTGAGCTGTGGAAGTGGGTACACACTTCTAGTATGTTGGGTTTCCCCACCTTAACACTAC

CTCTGGTTGCGTTGCCCATTCGGTGGCTCGGATACGCCAAAATTTGTGTGTGCGTCGTTA

TATAAATGTCATACACACGCGCGTATATATATATATATATATGTACCCCATAAGTGTTCC

CTTCTCCCTGCTGGTGCACGCTGCTGCTACCAAGATACCGCTTGTTTACTTCGATAAATT

TGCTTCTCCCACGTGCCGTTGAAGGGCGGTAAGGGTGAAAAGCGATACCTTTGTCTCGCT

GCCGCGGGGGAGCTAGGGCGGGACTTCGCTTTACCTCTTTCTGAGGGTTTCTGTAAGCCG

ATCTCTCTTCCGCAGAGAGTGGTAAGTATAATGTGTGTGTGTGTGTTTAGTGTATTCCCT

TCCTGCGCATCGTCTTAAGTGCTTAGTACATTTTTTAGTTTTTTTTCGTGTTGCCGTAAA

TTTATCTCTTTCTTTCATCGCCTTTGTTATCTGCAATGCGTCCGCATTTGGGAAAAGGTA

GGAAGGAGTGTTCCCTTCGTGAACGTAGTGAGTGGTTGGAAATAAAGTGAGCCGTGTGTG

CTGACCGTCACGCGCGTTTTTTTTTTTGTGTGTGTGTGTGTTTATTTCAATTTTGCTGTG

TTTTTGCGGTTATTCGATGGGAACCCACCCTTCCCCCCTCCCCTAGAGTTTTTTACACGT

GTTGGGCGGAAGGTCATAGTAGCGACCCGAGGAAGTACCGACACAGCCTCTTTTTATTTT

TTATATACCATTTACTTACGTGTTCTCCCCCTTTGTACCACTGCTACTGTTTTGGGGTCG

CGCTCCGTTGACGCGGAGTCACTTACATCGATGAATAGCATGTGTGCCCATTGTATTTTT

TTCCAGGTATCCTCTGCTTACTACTAATAATAATGAATGCACCGCGGAAACTAAATAGCG

ATCGCTTCATACGACTTGTTGTTGCGGTGACTAGGGCGCCCTTCCCAAGGGCAGCACGGA

AGGTTTTGTCGGGATAACATCATGTCTTCATTCTTTCATTCCTTTTACTCTTGCACACCG

ATAACGTAGCAAGTCGCGAATAGGATCGGCAGCTTCACTGAGAAGGTCTTTGCAATGCAC

TGAGCAAAACCCCTGTGAGGGCACGCCGCTGAAACTGGTAATTATATTTCAGAGTGAGAA

GATTCAACTGCCGAATCCACGGCTGCACAAGTCTCCGTATTGCATCTCGGTAAAATCTGA

GCTGGCGCGGAGAATTCTGTGTGGGCTTGGGGAGCAGTTACATAGCATCTTTGGACGGGA

ATAATACCCCTTGTACCCAAGAAGGGATCCTGTCGGATGTGCTCCGACTTACTTTGTAGT

CACGCACACACACATGCTCGGCAATCTTCCTCATCTGTGTTGTCAGCACGGTTCCCAGAC

CTCTACGATACTTGTTTTTACCGTCGACGGCGTGGCGCCATGCCTCGGAGGTTACACGTT

TCATGCG

>Tb927.7.6920 | Trypanosoma brucei TREU927 | hypothetical protein, conserved | genomic | Tb927_07_v5.1 reverse | (geneCodeEnd+0 to geneEnd+0) | length=1264

ATAATATTGATAATAATAATAATAATGACAATATTAACATGAGTCGCAACAGCACCAAAT

TAGAGGGTTAATACCTGGCAGCACGCGGTAGTTACCGTTGCTGGAACCGAATGTTGTGTG

TCATTTCCTTGACAATGGCTTCGATGGAAAGCAACGTCGGCATTTGTTGATGTGGGCGGT

GGCTAGCGTGGGGGATGGTAAGTCACGTGACGCTGTGGCATGTGTCCACCTTTCTCTTCA

AATTTCCGTGACAAGCCAAATTATCTATTATTATATTTTTTTTATACTATTATTTTGGTT

TGATTTGATTTGGTTTCTTGTTGTTGTTGTTGATTATATGCTGCAACTGAGTACGCGCAC

CTAAATAGTTATCTTGATGAGTGTTTGTGTCGATTATTTTCTACTTTCACCAGTATATTT

CTTTGGTTATGTCATTACCACAGGTCTTGAGTAGTCCCTCAGCAACGTGGGGGGAGGGAC

AGATGCGGCATAATCACGGAGAACTGGGCCACATCACCTACATTGTCATTATTGTTATAT

TGTGTGGGCAGTCAAGTCTGTTCGAGCTACTCGAAGGTGATCGTCTACAGTTGCAAGCTG

ATTCGGTTGCTCTGCACACAGTACCATAGCTTTTTATTATTATTATTATTATTATTATTA

TTTTCCTTTGTGACTAGTGGCTTCAATGGTTGATTTACTCGTCTTTTCTTTCTGTCGTGG

GGATCTCTTTATTGTGTAAAAGTATGGATGTTTCTTCCATGCATTGGCATCCATACATCT

CTGTTTATATATTTATGAACTGTGTATTGCGCCGTTTGCTTCATTTTGTTTCCCCTTTCG

TGCAAATCTGATGAGGGAACATAAGGCGAAATGCGGCAAGGTGCAAAGGCAAGGCGAAGG

TGACGTATGAAATTGTAACGGTTATTTTTTATTGTTTATTTTTATTTTTTTGTAGGGGGG

TGTACATGTTTACTCTAGACATATGCGCTTGTATGCATTTCAAGCAGCATGTAGCGCTGG

GGCAATTGCTGTTGATGTTGTTATTCTTTCAGCTCATTGTGTCTCAGTTTTGAAGAGGGA

AAACAATTGAAATGGAGAAGGGAAACTGGATGCTATGAATTCCATGGTGTTCATGCGTTG

GGTGAACTGCCACAGGCTGTTTTTTCGTATTAGAGTTGTCATTGCCTTTAAGACGCACGT

TCAATAACCACATCGCTGCGAAGACGGGTTGTGGATGGAAGGGGAAAGTGGCTAGCTTAC

GAAG

>Tb927.11.6020 | Trypanosoma brucei TREU927 | hypothetical protein, conserved | genomic | Tb927_11_v5.1 reverse | (geneCodeEnd+0 to geneEnd+0) | length=163

ATTTTCTGTTTTTTTCTTTTGACTGAACCTAACTTAACGTGTTTATTTTTTATATATAAA

TCCCTACACTTGCTATGTCAACGGCGGGGTGGCGAAATAGAGTTGGGGATGTAGAATAGT

TAGATTAGCTCTCCAAATTCGGAAGAATAGTGTTGTAACGACG

>Tb927.5.2870 | Trypanosoma brucei TREU927 | hypothetical protein | genomic | Tb927_05_v5.1 reverse | (geneCodeEnd+0 to geneEnd+0) | length=1730

AGGCATCCGGAACACTGTTAATCAGGTACTAACCGACACTACTTGCACCAATGCGGATTC

TTCCGTAACCATTCTGAGGCACAGAAGGCGCATTACGGGTCCCCTGTGAGATGTGTGTTA

CTTATGTGATTGACGGGGATGCTGCTGAAAAAAGAAAAATTGCCGGACACTTCACATGGC

AGTGGGCGCTTCTCTTCTGCATCGCTGGAGGGAACATCGATAAAGGACGTGTTGCTAATA

TTGCTCGTTTTTTTCTTTTTTGCTACTTACTATTGAGCGTTCATACTGCAAACGCACGAA

TCTGCGGAAGTAATTGACAAGAGTTGGGATTAAAAAACAACAACAACCTTACATGCATTG

TTTGCTCAGCGGACGCTATCTCTAGCCGCGCATTACTGCCATTGTTATTGCTTTTTGCAC

CGAGTTCTAACTCCAGTAGGAGTGTGCTGTATGTTATTAATATTATTATGTTACGTTGCG

TGTACTAGTATTTTTTTTGTGCATATATGTATTCATTATGCCCCATTGTTTGCTGGCCTT

CCGATTCACTAGAGCTTTTCTTTTTTGCTCCGCTCAGTGCCTTTGTTTCACTACTGGTGA

AAAAGTGAATTTTGGTGCGCGGGGGAGAGGGAGGGAGATAAATTGGAAGAAGATTTCCAA

CAGTGATTCTACAAATATAAAAATGTGTAGTTTACTTTCTTTTTAAAGACTTTTTTCTAT

TTTTTTTTCTTTGTGTATATTTCCATTTATTTTTATTTTTTAAATTTTTGTGTGTATTGA

GCAGCCCCGTAGTGCCCGCAGTTACTGCTTGCACAATTTTGTATTTTCTTTTGATTTCCC

CTTTCTTACTCTGTAGTGGGCAACCGTCTAACATCGTTCTTTCACTTATTGGTGCCCTTA

GTTCCTCTCCTTTAACTTGCTTATTTTCGTCACCCAATGGCAGAAATGCGTAATTGTAGC

CCGCGCACAACTCCCCATTACATTTCCCTCATACCATATTATTATCTATTATTTTGTCCT

TATTTTTTATTCCCTCGTAACTCAGGGTGTTTGGCTGCCGCTGCAGCCTTTTTTTTTGTT

TTTACTGTTCGTTACGACGTATGAGGTGAGGGAAAGTAGAGGGACGTACCTCGTCTGTCA

ATATCGTCGCATATGTACGAATGCGTGTAAACACGTAATTATAAAACTATGTGCTGAACC

AATCACGCCTTTTATAGCCTCTAGAGGATGTGCCGGGCAGTTTTCGCACCATTTTTCCGG

AGGTGCAGGCAATTTATTTATTTTATTTTTGTGTGTGTTTTGTTTCAGTTTCCCTCTTTT

TGATCCGCTGGCACATGTCCGGAGTGCCTACATTACGTTTAACCAGTGGCGCCTGAAAGG

AGATGTGTGAGAACAGAATGATTTTCAACGTGTGTGTGAATAGGTAAAAAATCGTTGGTA

GCTCAGATATGCACACGACTGTATTTAATTGTTTAGATTTTGTGTTTCTGTTTTTGTTAT

TATTACAGCATGGTGGTGGCAATAGAGGCGGAGGACATCCGTATTTTCATACGTAACTTT

TCACTGACCCTCCTCCCCTACCTTACTCTCCGTATTATCCACTAACAGTATTACATGAGT

GGGAACGTGAAGGGGCGGATCGGTATTAACGCGCTTCAGCAGGGAGGTGAGGGCATCAAG

AAGTAGCCTGATTTCTTTCCTTGACGTTATCCTCGTACGAGGCTGCAGTC

>Tb927.3.2930 | Trypanosoma brucei TREU927 | RNA-binding protein RBP6, putative (RBP6) | genomic | Tb927_03_v5.1 reverse | (geneCodeEnd+0 to geneEnd+0) | length=593

AAGTGTCACATTATTATTATTATTATTTTTTTGTTTTATTTTATTTGCTTGCTTTATATA

TTTGTTTGTTTTATTATTTTTGTATATATATATATATATAAATATAAATCAATAAGTCTA

TACATTTGTGTGCGTGTTTGTGTTTGGCCCGACGTTTTCTTTTTCTTTAAAAAAAAAAAG

CAAAAAGCAAAAAGCAAAACGTAACAAAAATTGATGGGAGAGATGCACACGAGGAAAAGC

AAAAAAAAAAAAAGGAAGAAAAGAAACGAATAACGAAGGAACGGGTTGGGGGTTTCGGTG

GAGAGGAGGGGGAGAGGGAGAGGGAGAGGGGAGGAGAAATTAAGAAAGAAAGGAAAAAAA

AATAAAATGACGTTTTGTGATGACTAAAATAAAATTAGGAGAAGAGGAAATAAATAAGTA

AATAAATAACTAAATAAATGAAAAAAAAACATAAAAGGGACTAATGACTACAAGTGATGG

GGAGGTGAATGGATCAAATAAATTATAAGAAAGCGAACGAATGAAAGAAAGATATTAACA

ATAAAATTCGAAAAAAAAAAGAAGAAAAAGAAAATAAGAGGGCAGGGAAGAGG

>Tb927.11.15160 | Trypanosoma brucei TREU927 | hypothetical protein, conserved | genomic | Tb927_11_v5.1 reverse | (geneCodeEnd+0 to geneEnd+0) | length=2977

ATTGTAATGCCAGCATACACAAAGGGATGGGTGTGAGGTGTGGGGTGGAAGCGTTTCCTA

TGCATTTTGTGTGGATGTGACGGCATGTCTCCCGGAACCCCCCTCCCCCAATAAATTAGT

TGCATATCCCTTTCTTTCACTGTGTTCATTTTTTTTTTTTTGAGCTTGCTCACACTTATC

ACAAAATCTCTCAAGCAGTTTCATCAGCAGAGGTGTCCTGGTGAGTGAAGGGGGAGGGGG

GGGGGTGTTATTTTCCACCTGTAGGTAATCTTTTTCGATTATATTGTTATTGTTATTATT

CATCGGTACGCATTGCAAGTTGTGTAAATAAATAAAAATATATATATATATTTCAGGGAT

TGTATGACTTATGAATTTTTTTTGTTCTCTTCGTATCCTCATTCCTTTTCCTTATTTCCC

TCTACCATTTTTCCTTCCGTTGGGCATCATATCCTTCATACGCTCAGTCATTCACTTCTT

TTTAGTTAATTTTTTTTCTTCATCTACCATATATTTTATTTCTTTTTTTTAAAAATATAT

GTGTATATGTTTCGTAAACCAATTGTTTTGTATATATATAATAATATATATTATAATAAT

AATAGGAACAACAACAACAGTAACACTGATTTTTTTGTCACAAAATTTGTATTATTACAA

AGGAAAAAGAACAGTCTCGAATCTCAACAAAAGCAACATAAATGTCGGCCGCCAGTGGAT

ATATGAATAAAATGTGTAAATATACAAAATAATACCAAATAAAAAATAATCAGAGAAAAC

TATGTTAAAGTATAAATAAAATGAATGACTGAGATAAAGAAAAGAAAAGAAAAGAAAGAG

AAAACGATGAAAAAAGAATTGAAAGGAGGAATGATGTGGCGTGTTATTATTATTATTGTT

TTTTTCCCTTCTAAAGTTTGCTGCACTCCACAGTCAGTTGATTGTAAGGAAGGGGTGGAG

ACAAGAGAGGTGGTGGAGAAATGCCTGTGGATGTTGAAGAAATTTAGAGATAATAAGAGA

AAATGGAGAGAAAAGGGGAGGCAAGAGAAAAAAGAAAAATAAAAAAGGAAAAAGAAAAAA

AAAGAGAGAAGCAAAATGCAAAATAACATATAAATAAATGAATATCTGCAAAACGTGAGA

TAAATGAAACATGACAAATCACAACATCGTTGCTAAAATATCGCGAAAGCATAAATGGAT

GAGAAGGTGTAAGTGAGGTTGTGACCATTTTGGTTTATATCTATTTACTTATTGTTTTCT

TGTTTCTGTTATATATATATATATATCTTTACACGTAAATAAACAAATACTAACATGAAG

ACAAATATATATTTCTGATCGTATGAGCAAACGGTTATGATCTATGAATGTATTTACATT

TATGTACGCGTATGTGTTATGTACTTGTCTGCATATAAGAGGAAAGGAAAAGAAGAAGAA

AAAGAAGAAGAGTGATTGAACAATTGGATAGATGTTTTTCAGTTCATCTCTAATAGAAAT

TTCATTGTCACTTGCATAGATGTGTCTTGCGAGTGCGTGCGTTTTGTTTTTCGTTGTTAG

CTTTTTCCCTCTTTTTTCCCCCTATTATTTAATAATAATAATAATAATTATTATTGTTGT

TGTTGTGTATGTTTTCCTCGTTTCCGGGGCGGGTGATTCGACAATCGATGAGAGAGAGGA

AAGAAAAAAAACGGAAGAAAAAAAAAGAAAGAAGACGTAACGGTGGTGCCAGCGACAAGA

AGTAAGACGCAAGTTCATATTTACGTTTGTTTTTTTCGAACGCTTGTGACTTGCATAATT

TGCTTCTTCGTTTTTTTTTTGATGGTGCTTTTGTTTTGTTTTGTTTTTCTTTCTTAAAGC

GATTGACCCTCTGTTGCTGAGGGAGCAATGACGAATGATGGGAAGAGGGGGGAGGGGGAG

AGGAGTGTGTGTATGTGTGGGGAAAGTGATGAAACAACTTCATATTTATATAAATATATA

AATATATAAGTGTATGTGTATGTATGTATGTATGTATGTATATATATATATATATATGCC

CCCTTTTTCATTTTTTTCTTTTTTTTTAAAAAAAACTTAACGTTGTTGTGATACAAACCA

GGTGTGGGGGTGTTTTTTTCTTTTTTTCTTTTTTTATATATCTCTTCTCCCCCCTCACAC

ACACACACACCTCTCCACTCACTCACCCGCTCGCTTTGACCTTCTTCAAATTATGCCTCA

CAAAACGAATTCCCACAACTATAAATCAGAAACAGGTACGTGATTTATATGAAACAAATC

AGCGTCAAATCCTTTCGTTTCAGCCTTACTTTTTTCTAGTTTCGATCTTGTTTTTTTTTT

CTCCCTCCCGTTCTCTCCTCGTCTTTTTTTTACTTTTTTTCCTTCTATTTACGCGCGCAT

CGCGTTTCCGAAATATAAAAAGTATTTAAATATATATATATATATATACATATATTTTGT

ACGTTTTTTCTTATACACCTCTAGCAGATGCTTATGAGCACTTATTATTATTTTTTTTTT

CATTTTTGATCAAAGAAGACAGAGCTGTAAACGACAACAACAACAGTAAAGAAGAAGAAA

ACAAAAAAGGAAGCAGTCATGAGACAAACAAACAAACGAACAAACAAAAAAGAAATAATT

CGTCATTACCCCCATTTGTTTTTTTCTTTTCCTTTCGAAAACTTTTTTGTGTATGTAGTA

GTCATACTTTGCAGCGTCATGAAATGTATTTATTTCTCCCCATATTACCTTCCTTTCTTG

GTGCTTTTACCCTTTGTCCAAAACCCTGCGCTTTTATATCCTTTTTTTTTCTTTCTTTTT

TTTTTAAAAAAAAAAGTGATTTCATTCTGCTTTTTTTGTCCCTCTCGGAATATTTCTTTC

GTACTTTCTTCCATTTTATGAACTTCACAAGAAAACAAAAAATCAAATCAGTTCATTAAA

GGTAAGACACAACACAAACATACGGAAGAAACAAAGG

>Tb927.3.4000 | Trypanosoma brucei TREU927 | clathrin coat assembly protein ap19, putative | genomic | Tb927_03_v5.1 forward | (geneCodeEnd+0 to geneEnd+0) | length=57

AAACTCCCCTTCTTTTCCCTTCCCTTCCCTTTCCTTCCCTATCCAAAGGCGGCTAAC

>Tb927.9.15480 | Trypanosoma brucei TREU927 | hypothetical protein | genomic | Tb927_09_v5.1 forward | (geneCodeEnd+0 to geneEnd+0) | length=1381

AATACACAATCCTCAGTTTTTAACTGCTACACGTTTTTTTTGGGGGTTTTCATGCACATT

TGCGGCAGACACACACACACACACACACATATATATAAATAAATAAATATATATATATAT

ATCATACCTGTGCTCCCACCGCACAACATGGATTCCTTATGATATAATTTTTTTTTTAAA

AATTCGTTCGGTTGTTCTTTTTATTATTTATTTGTTTATTTATTTTCATTGATTCCCCTC

TCTTTCTTTTTTTCCTGTGTCTGTGTATGTCTCTGTGTATGTGTTTCTCCCGGCTTGTAC

ATTTATTATAGTCGGATGCAGCGGAGGGGGAGGAGGAAAAAAAAACAACAACAACAAAAA

AATAAGTTTAACGTGTGAACTGCGTGTTTTTGCGAGTATATTTTTGAAATAAAGCGGTGA

GGCAACTTGTGGGAATAACACACAGAAAAATAATAGCACAGTAGGAAGTTGGGGTATTGT

TGTACTTTCATACTTTTGTGATTGTGATTGTTTTTTTTTCTTTTTCTTTTTTTTTTTGTT

GTTGTTTGATGTTTGTTTGTTTGTTTTTTTTGTTTTTTAAAAAAAGAAAAAAATTTATAT

CTTCCCCCCCCCTTTCATGAGAGTTTTTCCTCATCTCTTATTTCCTGTTATGATGTGATG

TGTGAAGATCTTACATCATATGGTCATGAAACTTCCGCTTGTTAGCGCAATAACGGTAAC

AACAATCATGATTTTTTTAAAAAAAAAAGAAAAAAAAAAGTAATTGTGATAATGATGATA

ATAATAATAGTAATAATAATAATAACAGTAGCGATTCTTTTTTTTTTTGAAAAAAAAATC

GAATTGGGGAGGATGTTGTTTTGCTCTATGCGTTTATTGAGGTGAATGCAAGTTTATTTA

TTTTTTTTTTTACTTTTCTCTTTTTCGTTCTTTCTCTTTTTCCCCTTACCTCCGTTTATT

TCTGTATGCTTATGACACACGTGGTAGCACCTTGAGGGCAAAGAAAAAATAATAAATAAA

AAAAATAAATAAAAATAAATTAAAAAAAGGTATCTCGTTTTGTTGTTTTTATCATCATCA

TTATTATTATTATTATTGTTGTTGTTGTTTCTACTTTGCTTCATTTCATTCTGTGCATCC

ACTTGTGCAGGTTATATTAGGTCATTTATATAGGGCCCATACTGAAGTAAAGTGAATAAA

CAAACACATAAACACAAACAGAGGAAGAGGAAAAGCACATATATATATATATATATCATC

ATATATATATATCATTATTTATATCTTTATTTATTTATTTGTATATATAAGTTTGAGTGT

AAGTAGACGCTGAAGAGCGAGTAGCACGGCACAAGTATTCGGTCAAAAAAAAAAGGGGGG

G

>Tb927.11.13300 | Trypanosoma brucei TREU927 | hypothetical protein, conserved | genomic | Tb927_11_v5.1 reverse | (geneCodeEnd+0 to geneEnd+0) | length=615

ACATTATGAAACGTGCACACTTGTCGGGAACAAAATGGTCGTCGGGGAATGATGCAATAT

ATATATATATATATATATATATTCATTTACTTATTTTTTGCTCTTTATCGGGCTCGCTGC

CGTTGCCGATGCTTTTTGTTTTCTTTTTTTGTTTTTTTCCTTTCATGTTGTTACGAGAGC

AGCAGATAGAAGGAGGGAGTTAGACACAGGTGGCTCTCAATTAACTGAAGGCAGGACAGA

AAGTTGGGGGGAGGGGGTTGCGAACCCATTCGTAACAGGCACGCTACACCAGTGGTTTGT

GATCAATATCATTTTTTTTTTCTTTTGGGTGAGGCCCCACTTCAATTTGTTGCCCGCCAT

ATATTTATGTGCTTGCTCTATGCAGGTACTATAAACCATAATACGTTTTTTTTTCCTGTT

TGGATTTGTTGTGAAGAGGGAATGCGGTTGAAAAAAGGAGCAAGAGGAAATTGCTTGGAG

GCGATGGGTGGTGTTTATTGTGTACGCCGGGAGGACGTGCGCTGCGGGCGAAGGGTGGAC

TGAAACGGCAACAGAGTTGCGGCGGGTTTCTACTTATAAGAATGGTGCTGATTCTTGATT

ATGTGTAGACATGGT

>Tb927.8.1150 | Trypanosoma brucei TREU927 | kinetoplastid kinetochore protein 9 (kkt9) | genomic | Tb927_08_v5.1 reverse | (geneCodeEnd+0 to geneEnd+0) | length=1022

AACCTTCTCGCCGGCATTTCAGATTATCGAACGGCAGCTACTGTTTTGTCTACATGCTAC

ATTTTCTTGGGGACACGGCACTTAGAGATGAGATGACCACACATTATTATTTTTTTTCAC

CGCTCCCTCCCTCCTTCCCCCCCTTTTTTCAAACTTAGATTGGAGGGAGCGTACATTTTG

TGTGCCTTCTTTTACACCCCGCCCTTCTTCCATTTTAACTGTTTATCGTTGCTTTCAACT

CGGTGGCGTGACGGCGCGGATTGTCCGGCAGTGACAGATGTGTCTGTGGGGATGGTCCTT

CGTCACGGGGGTGCATTTACGGGCATGCTTTTTTTTTTTTATACACTTCTCTTTCTAGAT

CCTTTCTTGTTTTTTTATTTTTTATATTTTGGTGATTACCGAATTGTGTGTGTGTGTGTG

TTTAAGTGTTAGCGGTTCATACTCCACTCATGCAAAGACGGGCATGTACTCCCGCCATCC

TGTCACTCACGCGACACAACAAAGGAGGTAAAACATCTTCACCGTCCCGAGCATTTTCTC

TCGGGTAAACAGTGCATATATTTGCTTTGGAGTGGAAGGTTTTAGAATTGGGAACCGGGA

TGGGACGTGGACGTATGTGGGGAAGGTTTTCGCCCCCTTTTTTTTAATTTTCTTTAAAAA

AGGGGGAAATAACATTTTTTTTTTTTTGATCCCCCCTCCCTACCAAATTCCTGGCGTTGG

TGGATGAGTGATAAGACATGAGGAGTGGAGAGAAGGGTACTAAAGTTAAATTAGATTGGA

AGGGAGTAAATTACAGGAAAAAGGAGCGGTAGTATCAATAAGAAATGTTAGAGAAGGGGG

ACACGGGGTTGAAGTGTGGTGCGATAGTTGTTTCCAAAAGTGGAAGGGAGGAAGGTATGG

GTTATGTTCTATATTCCGTTTGTTTCCCCTATTCGTTCATTTCTTTCCATTCTGCTTTTT

TCAGTTCATGATAAGGGACTGTTCTGCGTTTTTCTGTTGTTACCCGCTGTCTTCATTGCT

CG

>Tb927.6.2870 | Trypanosoma brucei TREU927 | hypothetical protein, conserved | genomic | Tb927_06_v5.1 forward | (geneCodeEnd+0 to geneEnd+0) | length=217

AATAACAGATAATATTTGCCGTGCGAGAATCATCACAATACCTCAAAGTTTTTATGAAGT

TTTTTTTGTCTCCGTCAAGAGTCGTTTATATTTATTCGTGATTATACTGTTCCATTTTAT

TTTTTTTTTTAAAGGCCTCTACTGCTATTATTATTTTTTTTTTACAAGTTTCTGTGCATC

TTTTTCTATTTCTTTTGTTGCTGCATGGTTACTTGCT

>Tb927.7.4110 | Trypanosoma brucei TREU927 | kinesin, putative | genomic | Tb927_07_v5.1 forward | (geneCodeEnd+0 to geneEnd+0) | length=2102

ACATTTTGTTTGAATACAGTTTTTATTTCTTTTCTTTGATTCGTTTATTACTGAATGTGC

GTACACGTATATGTGTATATATACATATATATGTAACTTTCTCATGTCATTTTAGCGTCA

TCGAAGAAGGGAAGGAAATGAAGAAAGGAAAGAATCGAGCATCTATTTTCGCTACTGTCT

GTCTATTTGTTTTTCCCATTCTTTCCCCTTTTGACCCTTTTTGTTAGTTTTTTTGAGTTT

ATACTCTGCAGACTCCAGCAGCACGGCAGAGAGAGGTGAAGACGAGACAAGAATAGAAGC

TGTTACAATGGTAGAAAAGGGGCAAAAGAAACGTAACCAACTGCAACTACTCTCAGCCTT

GAATGTTTTTTTTTTTTACTACCCGTAAAGGAGAAGCGAGGGTTCACCATGTTGTATTTG

AAATTTATAAATTCTTTCTATAGCTTTATTACTTGTCTCTATGTGGTGAATGATGTGGTT

GTTTCTTTTTTTTTTCCTCATGTCAATATATTGTTGTTACTGTCGTTACAAGTGTGTTTT

TGTCCGCACCGTGCTAAGAATCTTCCTCCTGTTAATTGTGCCATCTGCGTGATGTTTCTG

AAACGATATATATACATATATATATGAATTTAAAATCTATTGCCTTTCTGCTTTCCCTTC

CCACATAATCTCTGAGTCTGCGTTTGTGTGTGCCCTTTCCCCTCTTGTTTCCCATTCCAA

CTCATTTTTTTTTATTTGTTGCCTCTCCAAAATATTTCATGCATGCATATCATTTCTGCA

CCACAGATTCCATGGGGATGGGCACCGGTGAGTCCCTTTTCTCCCCACCAGGGTTTCCTG

TACGTTTTTTTGTTTTTTTGTTTTTTGATCTTTATTTATTTACGTTCTTGTCTCGTATTT

TCCTCTCGACTCTTAGTTTATGAATGCAGAGCGTGTATGTATATGTATTTGTATGTATGT

GTGTGTGTGTGTGTGTGTTTGTATGTATGGTAAATCTTTCATTCCTTCTCTTGCATTTTT

CTTTTCTCTCAATATTCCTTCCACCTCCGTTTTTTTTTAACTTCTACCTTTCTTTTATTA

TTGATCGTGCCCCTTTGTTTTGAATTTGAGTGCAGATGTACTGGTGTGCATGGCAGCTGT

AAACTACCTAAAGTATGAAAACAAAACGAGAGGTGAGAAAATATTTCTTCCACCCTTTCT

TTTATTACTTAACTTCTCCCCTTCTTCTCAATATAAACGATTTTTTTTTCCTTCAGCGTC

TGCTATGGGTTGGTACCGAACTTGACAGCACATTCATTTATCTATTTGTTGTTGTTGTTG

CGATTATTAATTAATTTTCTTCTGCTTTATTTATAATTCTTGAGTTTTTACCTCTTATTA

ACGAGTGAAGTGACGATTGTTGAGAATTAACGGTACTCCTTTTGGACCGATGACCGAGTG

TTTTATTCACCTATGTATGCGTATGCGTATGCGGAAGAGAGGCGTTTCGACTTCCCCTTC

TCTTTCTTTTTTTTTCCTTTTTCTTCCTCCTCTTACCGACTTGTTGACTGACTGACAGTA

AAGTGACAAAAATTGTGGGGTACGTACGGATATATTAGTAAACAAATAAATAAATTAGTA

GGTGAATAAATAAATAAATAAATAAAAATATATATATATATATATTTCCCCTGTGTTTTC

CCTGTTTCTATCTTATTTCAAGGTGATTAACATATTGTCGTCTCCCTCTCCTTCTTTTTC

TCCTCTTTAACACACATGCTCACACACGCCAAAGGTGTATAATTCAAGTCGCACGCATTC

ATTTACTCCTATGTCTTTTCCCCGTTTCTTCGTTTTTTTTATTTTTTATTTTTTATTTTT

GAAAAAAAAAACTTGTAGCGTATGAGCGCATTTACCTACTTTTCAAAACAATCTTTAACC

ATGAAGGCCCAGCGACCCGATTCGTAATTGTCCTCTTTTTACCCGTAATGAGGCTGATGT

TATAATGCCTTCTGAAGCATTTTCAATCCTTTTTTTTTTCGTTCTTATTCTCCTGTTTCT

CTCCTCCCCTCCTCCGGACTCGTCATCTTATCTTTTCCTTGTGCTACCTTTTCAGATTGT

TT

>Tb927.6.2850 | Trypanosoma brucei TREU927 | ESAG8-associated protein, putative (PIE8) | genomic | Tb927_06_v5.1 forward | (geneCodeEnd+0 to geneEnd+0) | length=322

ATGTGCGGCGTCACTTCTTTTGCAGTTACGCATATTGGGAGGGAGAGGGAGAGGGAGAGG

GAGAGAGAAGGTTTCAATGACTGCAAAAGTCCGTTAGTTTATAATTATTGTTGGTGGTGG

TGGCTTTTTTTTTTTTTTTTTGCTGTTGTTGGTATTGTTGATGTTGCTTTTATTTTGGTT

TGATAAAAATTTTTTTTTCTTTGAACGTCATAAACAGTACCCAGGACGAAGGAAGTAAAT

GCTTAGAAGGGAAATGAAAATAGATAACAATGAAGTGGAAGAGGCAATCACGAAATGAAA

AATTGACGTTGAAGAAGATTCT

>Tb927.6.3270 | Trypanosoma brucei TREU927 | hypothetical protein, conserved | genomic | Tb927_06_v5.1 forward | (geneCodeEnd+0 to geneEnd+0) | length=658

GTTGTTTGTTTGTTTTTTTGCGGAGATAAAGTAGAAAAGTTAGAGCAACTTCGCGTGTAT

ATATCCCTGTGAATTGCTGGTTGTGTTTGTTTTCCGTGAACATCTTATATATATTAAATA

TGTATATTTTATTCCTCTTATAAACAAAAAATATTGCACCTGCACATGTAAACAACCGCA

CAACATGCAGATAACTAGATACATATACATATATTTACGTTTTTTTGTCCTTTAATTAAT

TATGTTTGATTGTTCCACTACTGTTGGTATTTATCTCCTTTACAAGTAACTGCTCTTTAC

TTCCCCAGTTCGACTGGGTGCATACTTACGGATGAAAGTTATTTATTCGCTACTTATTTT

GTCATTTAGGACACACGAAAAAAGGGTTGTCGACCTCATCGGGGACTGCTGAGCTGCATC

CGATTGCTTTATTATTTTTTATTTGTTGCTGTTGTTTTATTTTTCTTTCACCCTTTTCAT

AAGAAGTCGGAGGAGGAGGAGACGACTGTTGGGGTGTTTTTTGTGAAAATCATGGGAAAT

AATTGTCAAAGGGTATTGGTGGGGATACTGGTTACTTACATGAATAGCTCCTCAGATTTT

TTTTTAAAACCTAAAGTAAAATAACAACGACACCGAAGTAGGTGTGAGAAAGAAAAAC

>Tb927.9.2840 | Trypanosoma brucei TREU927 | hypothetical protein, conserved | genomic | Tb927_09_v5.1 reverse | (geneCodeEnd+0 to geneEnd+0) | length=6993

AGTGTATACTTTTTGTTTAATGATGTGCATGAGAATTTATGGAGGTGTGTGCAGTGATGT

GCTGTTGTGTGTCATGTTTCCCTATTGTCACATTGTCTTTTTCTGCTACTGTATTTCTTC

GCAATGTCTTTAAGTACCTTCCTTTGTACCGTAATATTTTTGTATTTAATGTTTTTATAA

TCTTTGTGAAATGTTTCTGTTTGCATGGAGTAACTACAGTTACTCTCTTGAAATAATCAA

CTATTTATTTTGAAAATTGTTGAACCACTTAATGAGTAGTATTTGGTTTCATTTCCATTA

ATTTTGAATATATATTACCTTGTATTGTTCTATACACTTCCATTTCTTATTCTCTCATTA

ATAATACTTACCTGCTGAACTTTCATATATAATATACTTGAGTTATGTGTTAAGGCTTTC

TCTCTGCTACCTTTCTCTAATTATATGGAGTTTGTATCCTTATATGTTTTTCCCCCTTAG

CTGTATGCTTATATGCGTTGTGTATGTGTTTATGTTGTGCATTATTGTGAAGTGTGTAGA

TCCTTCTTGTGCATATATTCTATATTGTCAGCTTTGTAATCACTTTATATATCCTTTCTT

TTTATTTTTTGATTAATAACCTTTTCCTTTTCTGTAACATGGCACGGACGGTAAGTGATG

ATTCGCATATCAGTAGTTATGTGAAGTGTCAATTTTTTCTTTGTTCGAGTATATGTATAT

GTATATATCGTGCAGACGTAGCGTATTATGTGTTGATATGTTGTATTTGGATAAATCAGG

CAGGTGTTACGGCCTCCATGTGTTTGTTTTATAATATGTTTTTGATAATATTGATTGTAA

GGGGATGTAGCGATACGATGTTACTTTCGTGTTGCTGTGTTATTATACTGAGGGTTACTG

TGTCAACCCCTTCATCACCTGTATATGAATAAATAAATAAATATATGTTCGCTTGTCAAA

TTCACTATTTCTCATTTTTTTCTTATTAGTGTGTTAGACAAAAGGAAGGAATATATTTAT

GTTGTTACTTGTGTGTTGTGTATGTGTGTGCTGACTAACAATAGTGTTGCACTATATAAT

TCGTTTTGTCGTCGAGGATGTTTTGTACAAAAAAATAGTTTCATTTCAGTAATATGATGG

TGTGATGATATTATTAATTTCACAAATGAAGGTAATCCCCCTTAGCGTATTGCGTTACAT

TTTAACCCGAACATGGGAGATGTTTGTATTCATTTGAAGGAGTGGTATGAGGTGCCGTAT

GAAATGTAGAGTTATTGGAATTATCAATGCACTGAGTGTAAGTCATATGCCATTGCATTT

TCTATTTCCAACTATTAACTGTCATGAATATATCATTAGTGGTGTTCATAAACTCCATAT

GAAGTGTGAATGGGGTGTTGAGCCGTATTATTGAAGGACAGGGTGGTATCCCAAGGAGAA

TATAGTACTGCATTAATTTGTAGTTTTCCTTTAATTTTTTTGTTGTGCATGAGACACCTT

TTTTTCCTCCATAGACTCGTGATTCACTCCTATACTGTTATTGTTTTTGACAATTATTTG

CTTCTTAATTACTAGTTTCTAATATGAGTGATGAACTATTTTGATGTTGAGTTTTCGCAA

TGTGTGTGCGCGGCACTTTTATGATTGTACAACCATTACTACTTTCTTCACTTCCCGGTC

CCTTGACTTTCCCCTTTAACTAACCCTTTCATATCTCTTTGATTTCCCAACATTAACTAC

AGACTCATAAGCAAGCAAGTTTAACAGTAAACATGGAAATCCGCATGGTGCCATCAAACT

TCTCTCGGATTACAACTCTGTGTGGTATACTGATGTTATTGCAATGTTTTTTACGTCCAC

TGCAGGCTGCCAGCTCTGGAGGGGGTCCTGTGACTACAGCAGCTAAGAGTTACGCCGTAT

ATCCTCATGAAGTGTTTGAAAATAATGGTGTATATAATGTGGATTACGTTAATTCTGAGC

GTGTGTGCCGTGCTGAAGGTATGAACCTCGCTACTGACCACTCTGAGGCCACAAACAGTT

TAATATATAAACTATTAAAACCGAAAAATAAATTGGGGTATTTATATGCTTACCTTGGTG

GTGATGCCAAGTACAGTGCGAGTTCAGTACATGAGGAAAAGGACAGATGCAAAGTAGGTG

ACCTCGCTTCATCATTAAATTGTGTCTATCGATGGAACACAGGATTGTTTGCTCCGGCAA

CTCCTGACGATAATGGTGTTGCATTCTGGCGTGGGTCTTACTATGAAGTAACAGGAGCTG

GTAGTATGAATGACTACCCATCCTTTTTCGAAAATTATCCTGCGTACGGGAGATTAAACG

TAATTGCTAAACTGGATTCCTCTGGTCGCTTCACATGGTTTGATGATGATGACGACTTTG

GAAATATATCTATGTTTACGAACACTAGGGGTAGAAGTACACGCTTTTTCATGGTTCTCT

GTGAAGCCTCTGCAGTGCCAACTCCGCTTCCACCTGCTTCCCCTCACAGCGAAAATACAA

CAGCTGATGAAAATACAACAGCTGATGAAAATACAACAGTTGATGAAAATGCAACAGCTG

ATGGGAATACAAATGCTGATGAAAATACAACAGCTGATGAAAATACAACAGTTGATGAAA

ATACAAATGCTGATGAAAATACAACAGCTGATGAAAATACAAATGTTGATGAAAATACAA

CTGCTGATGAAAATACAATAGCTGATGAAAATACAACTGCTGATGAAAATACAATAGCTG

ATGAAATATCTAATGGAAGTAATGAAGCATCAGATAAGACTGTGCCATCTACAGCTTCCG

ATGGTGAGGGAAGTGGCGGTGGTGCCGGTGCTGCTGTTGTTATAATATTTATTCTTCTTG

CTGTATTGCTTATTCTTTTATACTTTTGCTGTTTTGCAGGTCATGAGAAGTATATTACTG

TAATGTCTCTCCGTGAAAAGGTTACCTCACCCGTATCTAATGTGGAGGCAGCAGAAGTGG

CAGCGGTACCATCAAATGGTGAGGAGCATTTAAGCATTACCAGCCATCAGCAGGAAACTC

CAGCAGTTGATGAGTGAGTGTATACTTTTTGTTTAATGATGTGCATGAGAATTTATGGAA

ATGTGCAGTGATGTGCTGTTGTGTGTCATGTTTCCCTATTGTCACATTGTCTTTTTCTGC

TACTGTATTTCTTCGCAATGTCTTTAAGTACCTTCCTTTGTACCGTAATATTTTTGTATT

TAATGTTTTTATAATCTTTGTGAAATGTTTCTGTTTGCATGAAGCAACTACAGTTACTCT

CTTGAAATAATCAACTATTTATTTTGAAAATTGTTGAACCACTTAATGAGTAGTATTTGG

TTTCATTTCCATTAATTTTGAATATATATTACCTTGTATTGCTCTATACACTTCCATTTC

TTATTCTCTCATTAATAATACTTACCTGCTGAACTTTCATATATAATATACTTGAGTTAT

GTGTTAAGGCTTTCTCTCTGCTACCTTTCTCTAATTATATGGAGTTTGTATCCTTATATG

TTTTTCCCCCTTAGCTGTATGCTTATATGCGTTGTGTATGTGTTTATGTTGTGCATTATT

GTGAAGTGTGTAGATCCTTCTTGTGCATATATTCTATATTGTCAGCTTTGTAATCACTTT

ATATATCCTTTCTTTTTATTTTTTGATTAATAACCTTTTCCTTTTCTGTAACATGGTACG

GACGGTAAGTGATGATTCGCATATCAGTAGTTATGTGAAGTGTCAATTTTTTCTTTGTTC

GAGTATATGTATATGTATATATCGTGCAGACGTAGCGTATTATGTGTTGATATGTTGTAT

TTGGATAAATCAAGCAGGTGTTACGGCCTCCATGTGTTTGTTTTCTAATATGTTTTTGTT

AATATTGATCGTAAGGGAATATGGCGATACGATGTTACTTTCGTGTTGCTGTGTTATTAT

GCTGAGGGTTACTGTGTCAATCCCTTCATCACCTGTATATGAATAAATAAATAAATAAAT

AAATGTATATATACATGTTCTCTTGTCAAATTCACTATTTCTCATATTTTTCTTCTTATT

AGTGTGTTAGACAAAAGGAAGGAATATATTTATGTTGTTACTTGTGTGTTGTGTATGTGT

GTGCTGACTAACAATAGTGTTGCACTATATAATTCGTTTTGTCGTTGAGGATGTTTTGTA

CAAAAAAATAGTTTCATTTCAGTAATATGATGGTGTGATGATATTATTAGTTTCACAAAT

GAAGGTAATCCCCCTTAGCGTATTGCGTTACATTTTAACCCGAACATGGGAGATGTTTGT

ATTCATTTGAAGGAGTGGTATGAGGTGCCGTATGAAATGTAGAGTTATTGGAATTATCAA

TGCACTGAGTGTAAGTCATATGCCATTGCATTTTCTATTTCCAACTATTAACTGTCATGA

ATATATCATTAGTGGTGTTCATAAACTCCATATGAAGTGTGAATGGGGTGTTGAGCCGTA

TTATTGAAGGACAGGGTGGTATCCCAAGGAGAATATGGTACTGCATTAATTTGTAGTTTT

CCTTCAAATTTTTTTGTTGTGCATGAGACACCTTTTTTCCTCCATAGACTCGTGATTCAC

TCCTATACTGTTATTGTTTTTGACAATTATTTGCTTCTTAATTACTAGTTTCTAATATGA

GTGATGAACTATTTTGATGTTGAGTTTTCGCAATGTGTGTGCGCACCACTTTTATGATTG

TACAACCATTACTACTTTCTTCACTTCCCGGTCCCTTGACTTTCCCCTTTAACTAACCCT

TTCATATCTCTTTGATTTCCCAACATTAACTACAGACTCATAAGCAAGCAAGTTTAACAG

TAAACATGGAAATCCGCATGGTGCCATCAAACTTCTCTCGGATTACAACTCTGTGTGGTA

TACTGATGTTATTGCAATGTATTTTACGTCCACTGCAGGCTGCCAGCTCTGGAGGGGGTC

CTGTGACTACAGCAGCTAAGAGTTACGCCGTATATCCTCATGAAGTGTTTAAAAATAATG

GTGTATATAATGTGGATTACGTTAATTCTGAGCGTGTGTGCCGTGCTGAAGGTATGAACC

TCGCTACTGACCACTCTGAGGCCACAAACAGTTTAATATATAAACTATTAAAACCGAAAA

ATAAATTGGGGTACTTATATGCTTACCTTGGTGGTGATGCCAAGTACAGTGCGAGTTCAG

TACATGAGGAAAAGGACAGATGCAAAGTAGGTGACCTCGCTTCATCATTAAATTGTGTCT

ATCGATGGAACACAGGATTGTTTGCTCCAGCAACTCCTGACGATAATGGTGTTGCATTCT

GGCGTGGGTCTTACTATGAAGTAACAGGAGCTGGTAGTATGAATGACTACCCATCCTTTT

TCGAAAATTATCCTGCGTACGGGAGATTAAACGTAATTGCTAAACTGGATTCCTCTGGTC

GCTTCACATGGTTTGATGATGATGACGACTTTGGAAATATATCTATGTTTACGAACACTA

GGGGTAGAAGTACACGCTTTTTCATGGTTCTCTGTGAAGCCTCTGCAGTACCAACTCCGC

TTCCACCTGCTTCCCCTCACAGCGAAAATACAACAGCTGATGAAAATACAACAGCTGATG

AAAATACAACAGCTGATGAAAATACAACAGTTGATGGGAATACAAATGCTGATGAAAATA

CAACAGCTGATGAAAATACAACAGCTGATGAAAATACAACAGCTGATGAAAATACAACTG

CTGATGAAAATACAAATGCTGATGAAATATCCAATGGAAGTAATGAAGCGTCAGATAAGA

CTGTGCCATCTACAGCTTCCGATGGTGAGGGAAGTGGCGGTGGTGCCGGTGCTGCTGTTG

TTATAATATTTATTCTTCTTGCTGTATTGCTTATTCTTTTATACTTTTGCTGTTTTGCAG

GTCATGAGAAGTATATTACTGTAATGTCTCTCCGTGAAAAGGTTACCTCACCCGTATCTA

ATGTGGAGGCAGCAGAAGTGGCAGCGGTACCATCAAATGGTGAGGAGCATTTAAGCATTA

CCAGCCATCAGCAGGAAACTCCAGCAGCTGATGAGTGAGTGTATACTTTTTGTTTAATGA

TGTGCATGAGAATTTATGGAGGTGTGTGCAGTGATGTGCTGTTGTGTGTCATGTTTCCCT

ATTGTCACATTGTCTTTTTCTGCTACTGTATTTCTTCGCAATGTCTTTAAGTACCTTCCT

TTGTACCGTAATATTTTTGTATTTAATGTTTTTATAATCTTTGTGAAATGTTTCTGTTTG

CATGAAGCAACTACAGTTACTCTCTTGAAATAATCAACTATTTATTTTGAAAATTGTTGA

ACCACTTAATGAGTAGTATTTGGTTTCATTTCCATTAATTTTGAATATATATTACCTTGT

ATTGCTCTATACACTTCCATTTCTTATTCTCTCATTAATAATACTTACCTGCTGAACTTT

CATATATAATATACTTGAGTTATGTGTTAAGGCTTTCTCTCTGCTACCTTTCTCTAATTA

TATGGAGTTTGTATCCTTATATGTTTTTCCCCCTTAGCTGTATGCTTATATGCGTTGTGT

ATGTGTTTATGTTGTGCATTATTGTGAAGTGTGTAGATCCTTCTTGTGCATATATTCTAT

ATTGTCAGCTTTGTAATCACTTTATATATCCTTTCTTTTTATTTTTTGATTAATAACCTT

TTTCTTTTCTGTAACATGGTACGGACGGTAAGTGATGATTCGCATATCAGTAGTTATGTG

AAGTGTCAATTTTTTCTTTGTTCGAGTATATGTATATGTATATATCGTGCAGACGTAGCG

TATTATGTGTTGATATGTTGTATTTGGATAAATCAGGCAGGTGTTACGGCCTCCATGTGT

TTGTTTTATAATATGTTTTTGTTAATATTGATCGTAAGGGAATATGGCGATACGATGTTA

CTTTCGTGTTGCTGTGTTATTATACTGAGGGTT

>Tb927.3.5660 | Trypanosoma brucei TREU927 | UDP-GlcNAc:alpha3-D-mannoside beta-1,2-N-acetylglucosaminyltransferase I (GnTI) | genomic | Tb927_03_v5.1 forward | (geneCodeEnd+0 to geneEnd+0) | length=1386

ACTTAAGTGCAACAATAACTTTCATCATCATCATTATCTCCTTATAAGTTCCTTTTCCTT

ATTTTCTTGTTATTGCTATTGTTGTGCCGGTTCTTCTTTGTTTTCGTTTTAGTTTTTTTT

TAAAAAAAACACTCCTCACACCTGTTTTGTCCGTATAAATGCGAATATATAAATAAAGAA

GGCAATTTATCCAATTTGCATTTTTGAAACGGTGATGGTGGGCGTAAATTCCGATTTTTT

TTATGATTTCCCTCACGCACACGCACACACACACACTTCCAAAAAAAAAAATGCGTCCCT

GTTACTTTAATAAATAAATATATATATATATCCTCATTTAGAACTGTGTGGTCGAAGAAC

CAAAAAAAAAAAATGAAAATTTTTGATTGCTGCACTTCACTTGATTTTTTTTTAAAATAG

TTTTCATTTTGAGTCTCAGTTTCTCCCTTTGGTTGATCTCAGCTAACAGTTTGTTCACTA

CTCTCCTTCTGAACATGTTAGGCTCAGTATGTTATTTTCTTTTTTTTTGTTTTGTACTGT

AAATGCTTTTGAAAAAAAAAGGGGGTGAAGGAAATGTGTGCGTCAATTCAAGTGTCATCA

TTTTATATTTAAAAAAAAATGTCAATTGGAGGTGTAAAAAAAAAAACATTTTTTGTTTAG

CGAGTTCATTTCCATGTATTTTCGTTCCATTCATCATCTAAATAGTTGTTTGTTATTTAT

TTTTCACTCTTTGCTTTGACTGAATTTTACTTAGGTTGGAAGTGTGTTGTAGTTTTTAGT

TTACAGTTTTGTTGCTGCCGTTTTTTAATTTTTTTTCTATAAAAAAAAGGAGCTTTGAGA

AGAAAGGGAAACGGCGCATTTAATTTTCACACACTTTTCTCTTTCAGTCCTGAAAGTTGA

GAAGTGGATGGGCACATGTTAACGGTATTTAACATTGATATGATTTGCAGGTGGATGTCG

TTTGGAAATTAGAGGGAGAGGAACTGGAAATATAATCGAGTGATGATGCGCAGCAGGAAA

TGCGTGCCACAAATTTGAAATTAAGAAATGATATCAGTTGTTGAAACATGGAGGCGAACA

GGAGAAAATATGAAATAACGAATATGACAAGAAGTAAGCGTTACTCGCATAAACAACCCG

CTGCGCAGCAGAACAGAGAAAGCGAGAGAGGAAGAAGATGATGGTTTAATTATAGCAGAG

AAACTAATATTTGGATGACTCCAAACTAAATTGCTGAAATGTTGGGAATGATGACATTAA

ACACGTTTTTTTTAATACTTGCCGTTTTGGTGGATTGCAAAATTTCGAAGATATTTATAT

TTGGTTGATGAATGTAATGCTTACGTAGTGAAGGGTACATGTTCTAATAGAGGAGACGCG

CGCCTG

>Tb927.8.7710 | Trypanosoma brucei TREU927 | hypothetical protein, conserved | genomic | Tb927_08_v5.1 forward | (geneCodeEnd+0 to geneEnd+0) | length=138

AAGCGGTGGCAGTGTTTATATGAAAAAAGTAGAAAGTGGGGGAGCGACGGCGCGCCTCTA

ATCATTTCGAGTGTATGTGTTCCGGCACAATTCAAGGGAATGTGTAAAAGGGACATGTGA

CTTTAATCCTCCGTTAGG

>Tb927.7.6310 | Trypanosoma brucei TREU927 | polo-like protein kinase, protein kinase (PLK) | genomic | Tb927_07_v5.1 reverse | (geneCodeEnd+0 to geneEnd+0) | length=1442

GAAGGAAAAGAAATGAAAAATAAAAAAAAAGAAAAGAGAAAAACGTGTGATAGCGTTTCG

ACAGATGAACTGTGTAGGCAAAAGAAAAAAAAGGACGAGTATATATATATATATATATAT

ATATATATGAATATATATGAATACCCGAAGGGACATTATTCTCTCGTTTCTTCTTGTTAC

CGTTCTATCTACGTTTTACGTCTCTTTCTCTACAGGGAAGGAGAACAGAAGTTGTGTTTT

TGTTTGTTTATTTTGGTGTTTACAGGTGGGCCCTAGTATTATGTGACACCCTTTTTCTTT

GTTATTCCTCCCCTTTATCTCAAAAAAAAAAAAAAAATAAAAAAAATAAATTAAATAAAA

ACAAAAGAGAAAAACAGGATGAGGGAGTGAAGCATTTTACTTCACTGCTGCACCGTATAA

GTCGATATGCAAGCCATATGAGGCCCTTTCTTTCTTTATTTTTATTTATTTATTTCCGCC

ACTTGTTTGGGTATATGAGGCTTCAGGCGGGGCTAGATTCGAAAATGAGGCCGGGGAGAA

GGGGCGAAGGGAAGAGAAGAGAAGAGATGAAATGAAATGAAGGTGTTGAAAAGAGAGGAG

AGAGAGAAATAGAAAAAAAATATTAATGAAACCCACATCTACGCATTGTTATTTTGCGTT

ATAAGACCCATAAAAAGCACTAGATCACTTCCCTCCCTTTTTTGTTATTTTTTTTGTCCC

CCCCTCTCTCTTTCCACGTACTCGTCGCTATTACGTTTTTGTGATGTGATGTGTTTTCTT

GTTTTTTTTTTCCATATGTACTGAATTTCCTCTCTTTGTTGCTCTCTCTCTCTCTCTTTA

TCTTCCTCTCTTTCTATTGCTTCCATCTGGAGGGAAATGATACACTGTATTCGCTTTCAA

CTCCTTTTTGTTCTTTCATTTCCATTTCCATCCTTTTTTTGTTTTTGTTTCACCTTCATT

CATTAATTCTTCCTTTCTTTTGTATTTGATGCCACGATCCTTTTCAACATCGTCGGCAAG

CTATAGAAAAATATATCATTAAATACACAACTACGATGGTGGAACCGCTGAAGAGGAAGT

AATACACGAGCAAACGTTCATTTGCAACTACATATATATATATATATATATATCTGCGTA

TGTGAATGTATATGAAACAGGTCACGAATTTGCTACGCGCCTGCAGGTGAGGCAAGGTGA

TTTCCTTTTTGTGTTCAGATGTTTGACAGTTTGCTCTTTTTTTTGTTGTTTTATGTTTCA

TCTCTTCGGCTCGTTCTCACGTCGTGTTTTTTTTCTTTTTTTGCCTCTCTTTGTTCCTTC

CCCTCTTTTCCCCTCTTTTTTATTTTCGTATCTTCAGATCCAACGAGGTATGATTATATA

TCAGCGACAACACAAAGCTCCCGCCTCGTTGCAGCGTGGTGTTTCTGTCATTGTTTCTGT

TT

>Tb927.9.3100 | Trypanosoma brucei TREU927 | hypothetical protein, conserved | genomic | Tb927_09_v5.1 reverse | (geneCodeEnd+0 to geneEnd+0) | length=1

A

>Tb927.6.3850 | Trypanosoma brucei TREU927 | chaperone protein DNAj, putative | genomic | Tb927_06_v5.1 reverse | (geneCodeEnd+0 to geneEnd+0) | length=953

AAAATATTAGTGTAAATACATGGAAAAAAAATAAATAATAGTGAGAATATATTTGTTCAG

GTTGTTATTCATCGAATCTCCCAAATTTGAGATTAGATGAGACACTAACGTACTGTTGCA

TGAGTTTCCGTTATCCTTTCTCCCTTTTAGTCCTCGCGAACACACGCATTAAAGAAAAAA

AAAGGAAGAAAGGCAGCGCAAATCGTCTGTGTTATCGACATCCTTCTGAGGACCGCAGAA

TTGATTAGCGCGCATGCGGACATTGCTATATACATATATATATTTTTTTACAAGTGGTTA

TTGTTTGTATCTATATACAACTGAATATTTGTTGCTTATATATTTTTGTATATATATATA

TATATATACGTGTGTGTGTAATTAGCGGGTTACCGACAAACGGAACAAAAAAGCTTTGAC

AGAGAACTGTTACAGCATGTTTTTGAACTCATGTCCAGTGAATCATAGTTGTGAATGCTG

ATGTGCTTCAATGCACTTCAAATGAAGTAAGTAGGCTCATTTCGCCTACCATGTCAACTT

TTTCTTTATCTATTCACCCCTTCCGTATCTTTCTTTCTCTCTCTCTCTCTCTCCATATAT

ATATATATATATAATTTATTTTTTTATTCCTAAGTTATGGATGAGTCGAAGAGGGGCGTT

TGAAACAACTATGCAATACATTGCTTTTTTTTTTTCTTCGCTTCGGTAATACTTGTCATC

TCGTGTTTCGTTTCCATTGAACCGCTTGCGTTTTCTCTCCTTTCGTTAAACTGTTCTAAA

CACGCTCAACCACGCAACATACTGGGGTTTAGGTTCTCAAGCATCCAAAGAAAAAGGAAA

AAGAATTAAAGGTAAGCACGCGGAATACGGAGCTTTATTTAAAAAAATAAAAAGGTTTCC

TTTTAAAGGTGAAGAACAAGAGCGTCTGAACAGCAGATTAAAGAAGGAAAAGG

>Tb927.11.16400 | Trypanosoma brucei TREU927 | kinetoplast-associated protein 3, putative (KAP3) | genomic | Tb927_11_v5.1 reverse | (geneCodeEnd+0 to geneEnd+0) | length=629

GAGTATAGAAAAACATGCAAACAACAACAAGGAACAAACAAGAAACGAAAGTACTGAACC

TAAATCCAGGCAAAGTCAAACCGAAAGAGGCACTAGATCGTGGGGGAAATGAGGGTAAAT

AGTGAAGTTGCTAAACATAGAAAAGAGAATGCAACAGCTCTTCCAGTGCATACTACGGTG

GAACCTAGTAGCGGAAGTGATCATTGCTGGAGCGCAAAATGTCTCGGTCTTTAGAAGTCG

GCATTGGGGAATATAAAAGGAAGTTCCTCCTTCACGCTCTCTTCAGACCTCTTGTTCCCC

ATATTTCTCCCTTACACACTTCATTCCTTTCTTCACTTTGATAAAAGATTCATTTGTTTA

CTTATTTTTTCGTATTTTGTTTATCACAATCCCTTATTTATTTATTTTTTTGCCCTTTAG

TTTTGCGCTCTGCCCCCCCCCCCCACACTCTCTCTCTTTTCCCCACCTCTCCACTGCCAC

GTTTGGATTCCTGCGGGATGCAACCTGGTGGACACCATTATGTGTGAAGCAGCAAACTAC

ACACACACGAAAAAAAAAGAAAAAGAAAATACAGAAAAGGCGTGTTGGAGTTGAAAACAA

AGTAATAGAAGTAATGGAACAACAACGAC

>Tb927.8.4200 | Trypanosoma brucei TREU927 | hypothetical protein, conserved | genomic | Tb927_08_v5.1 reverse | (geneCodeEnd+0 to geneEnd+0) | length=4630

AATGCAAAAGATAAGGCTTAAAATGAAAGAAAAAAAACAAGAGAAACTGAGAAGTTACGG

AACATGGGTGTGGGCCCAGTGGCATAAGGGCGAACAACATATATATATATATATATATGT

TTGTTTGTTTGTTTGTTCATTCACTCATTTATTCTTGCTTTCCTGTGGGTAGATTTTTTT

TTTTGAAAATGGAGAGGAAAGCAGGATGTGAAATAAGAGAGAGGTGCTACTGGTGCAATA

AGAAATATGATTCAAAATGCACCAAAACAAAAAAATAATAAACCAAAAACCAAGAAAAAA

TAAAAGAAGAGAGACACGAGTCAAAGGAAGAAAAAAAAAAGAGGAAAAATATCCCTAGCC

CACATGGCACGGAGCATATTTCTTTTTCGTGATTTTTTTTTGTGCGTTTTCTTACTTGGG

TTACTTTTTTTGGACCATCAGTTTTTTTACCCTTTCCCCACTTTCCCCCATCGAAACAAC

CCGTGTTGAAGTTTGTCGCTTGGCGGAGTTCAAAAAAAAAATAAAAAGAGGAAGAAAAAA

ACGACTCTTTCATTCACTTAGTTGAGTTTTCTTTTTTTTAAAAAAAAAAACAGTAACAAA

TATTATTACTACTATTAAATTGTCTCTTTCGTGCTTTTCTTCTTTTTTTTCTGTGATTTC

TTGCAGGAAGTTGCAGAGATGAAAGAGAAAGGGAGGGTATGACAGAAGTGGCTGTCTTTG

TGTGTGTATATATGTATGTGTGGGAAGACAAGTAAAAAAGAAAAGGAAAATAATAGTGGT

AGTAGTAGTGATAATAACAGTAATAACGAAATATAAGTTATATGCCGTTATTATTTTTAT

TACTACTACCATCATCACCACTATTATTATTATTGGGGGTTTCATCTTTTTGTTTCCTCT

TGGTCCTCTTTCTTGTTTGTTTGTCTTACCTTTCCATATCCATATCACACCCTCTGGTGT

GAGAGCGAAGTTAAATACAAAAGAAAAGAAAAAGAAGGGAGCAAACAAATATATTTTTAT

AGGGTTGGGGTCAATGGGGAACTGACATCAAAGAAAATATATATATATATATATATAATA

TAATACAAAGAAGAAGAAATGAAGAATGAGTGATGAGGCAGTGCAGAGAGAAGGGGCAGT

TGTATTGACAAACACGGCACATTTGGGGGAAATGAAATAACAATATGAAAATTACTTTTT

AGTTCTTTTTGTTGTTGGTTTCTTTATTATTGTTTTTTGTCTTTCCTTTTTTTTGTCGCG

TCCGCATGTGTTGTTATTATTATGTTGGAAATGGGAACGGAAGGGGATTTGAAAGGTCAT

TTTGACACCATCTTGTGCTTCTTTAGAGCAATCACTTCTTTTTCTTTTCTTTTTTTTTTT

CCATTCCAAAGTGTTCCCCTTGAGTGTTCGATCATTTCACGCCCATTTCAAAATAACAAT

TTTTTTTCTTTTTTTAATCCCTCCCTTTTTTTTGTTGCTTAGTTTTTGGGTTCTGATTTT

ACCGGATCTTCGCTTTTCGGTTTTGAACACGTGCAATTTTTCAAATCCCCCGCCACCTCA

GTTTTGTTGTTGGAGACGTGTGAAAACAAGGAGGAGGAGGAGGAGGAAGAAAATAAAGAA

ATATAAATCGAAAATAAATTTTAAAAAAAGTTAATAAAAACGGAATACGGTAAGGAAGGT

CGGGCAGGCTTTTTTTTTAAAAGAAAAAAAATATGTTTTGTTTCTCATTTCCCTTTCTGC

TGCTCTGTCCTTTCTTGCTTTTGTTTTGTTTTTGTGTGTGCCTGAAAGGTGTCCCCAACA

TTTTTCCTATCCTTCTCTTTACCTCTCTTTCTCTCTCGCTGTCTTTTCCTTTCTTCATCT

TTTCTTCTTCTTGCTTTTGTTTTTCCTTTGATTATTATTTTATTTTATTGTATTCGTGTT

TGTATTTGTGTTTGTGTTGCCTCCGCTTTGTAGAACAGGGGGGAGGGAAGAAAAATAAAA

ATACAAAAGGCCGTGGGGTATTGAATTTCGAGTTAAATAATTGAAGTGTGAGCGACTTCC

TACCGTCATTTTAATGCAGATCGCCCAGTTCCTTTCAATCATTTCTTCCGTTTTATTATC

TTTTGTTTTGTTTTGGTTTTGTTTTATAGTGATATTGTTTCGCCTCTCAGTTTCTTCCTT

TTTACGTTGTTTGGTTTTCTATATTCATATTGACGGGGTTTCTTCTTTTTCCTTTTTTCT

TTACGCACTTCATTTTACATATGGTTGGTTTTCCTTTTTCTATCCTTTCTTTTGTTTATT

ATTGACCTTCTTTTTATTTTAATTTTTTTTTTTACTGCTCGTGTGAGTGTTTTTCCATTT

TTTTTTTTTGTTGGGTCGTTTTTTTTTTTCTTGTGCGAGTCTCCGCGCTTCACGATATCA

TTCACACATTTTTTTCTGATGGGGGTGGGTGGTGGCGTTGTTTTTTGTATTGTTTTTTTT

TTTAATTTAGCTGATCTTCATGATGATGTTGCTTTTTGGTGCCTTCTCGCGGCGTCAAAT

TATTTAGTGTGGGAAAATAATGCTTATGTTTCCTCTCCCGCTTTTGCTGTTGTTATTGTT

GCTGTTTTTGTTTTTTTTTCCTCTTTCCTCTTCTCTTCTTTACCTTCTCCTTTTTTTTCT

TTGTTTTTGTCTTTTTTTTTTTTTTACGGTATATAATTATTTATTCATATCACTTTTCTT

TTTTATTTTTCATTTTTTCCCCATGATATTTTGGGCATTTCATCGCATATGACATGATAA

ATGAATGAAAATACGGTGTATTATATGAAGAACAAAAATATAATACGAACAAATAATTAA

AACAGTTGATCAGCTAATGGTTAATATTTATATATTTGAGAAGGATGTGAAAGCAAAGCA

TTAGAACGCCACAACTTTTGTAATATATATATATATATATATATATATTACATTATATTA

CACAGAGATGTATTTTTCTTTTAAATAAGGAAGGGAAAAAATGAAATAATCTACAAATCA

ACGATATATTCCCCTTTTGCTGAAGTTTCTTTCGTTTCATTCCATTTTGTTTCGTTTCCC

CTTCCTGTGAAACGGGTTTGTGTATTTGACTGCTTATATTTCCCCCATTTTTTTTGTATT

TATTTTCATACGTTATCTTTTTTTATTTGGTTCTTTCAATTACTTCCTTGCCTTTAGGCT

AACAATATATTTGGTCATATCAATGCCAATATATATGTGTTTTTTTTTTGGCCTATTGTT

TGTGTGTTTATGTGTTTGTGTGTTTGTGTGTTTGTGTGTGTTTTTTTTCTTTTTTTTCAG

TTTTAGTTTAGTTTTTTTTTTGTTCCCCCCCTTTTTTTTCTTGTTTCTCTTATATTTGTG

TACTTCACACTTTCAGAGGAGGGTATATCCATCATATTTGTCACAACATAATTTCTGCTC

CTTGTGAGCAAAAAAAAACAAGGTGGAGTGTCGTAATCTTTTTCTTTTTTTTTTCCTGTC

GCGGCATTCGTAAAGCACAAATAATTTCGCAAATAGCATTAAATAAAGCGGAATATAAAT

GCCTCTGAAGAAGTTGTGATGGATGCAATCATCACCAATGAGCGAAGAATGTATGCAGAA

GGGGGAAAAAAAATAAAAACTGAATTTCGTTCTTTTCTTTTTTGTTTTTATTCTTTTCAT

TTCCTCTTTCCATTCATATGTTTTGTTTATCATCCATTCTTCTTGTATTTATTTAATTTT

AAAATATGTGCCTTTAGTGTATTGATGAAATGTGCGTGTGAGTTTCTCCTTATTCCGCTA

TTGCTTACGTGCTTTTGTATATATTTTGTTCCCCCTCCCACACACACACACACACACACA

CACGCACCGTTCCTGAAACCAGTTATATTTGACTTAACGATTTATATATTACGTCATCTC

CCGTACACCGCAGGAGATATAACTGGTGGGAAGGGGAAAAAAAAACACAAACAAACAAAC

AAACAAAAAGAAAGAAAGCTTTAAAAGAAAGAATAAGATGATGATGACATGACTTCATTT

TTGTTGTTTTTATTTTTATTTTTGTTATGCTCTTAAGTCTTTAGTATCAAAAGCAGTGAG

GCTTGCGGGCGAATGTATTCATGAAGAGCGAAATGACTGAAGCAATTGATTATATCTTGT

AATAAATTTACCAACTCCTTGCTTACATCGTATAAGCTGCAGGGGATACGGTAATATTTT

ATTTAATATTTATATTTATTTGCATTTATTTATAATATGTGTGTAAATGTATGGGGTGGA

GATGGAGATGGAGGTGGAGGTGGACACGTCAGAACTTTTTCCTTTTCTTTTCTTTTCTTT

TCTTTTTTTTTCTTTTTTTCTCTCTCTCTCTTTTTGGTGTTTTTGTTTGTTTGTGTGTAT

ATGTTTTTGCTCCCTTCTTTTTTCTTTTTTTTTTCTTCCCCCTCTCTCCTCCCTCCCCCT

CCCCTCTCCACCCTTCTGTTATGTTTAGTGTTTGGTTTTATATTAACAGTAAATAGTTAC

TACTGCCACGGTCACACACAAGTGGTAAATAATAATTATCCGATACCAATGATTTTGTTT

TATGGTAATATTTGTTTGTTTGAATGGTCGGTCCCACATTTATATTTTTCGAAAAGGAAA

GTGAAAAAAT

>Tb927.5.1550 | Trypanosoma brucei TREU927 | mitochondrial carrier protein (MCP23) | genomic | Tb927_05_v5.1 reverse | (geneCodeEnd+0 to geneEnd+0) | length=772

ATGCAGGAGCTTTCATAGAAAAAAGGAGAGAACCTGAAAGCAAAAGCATTAAAAACTACG

TAACACTCGCCTCTATGGTCCATCAGGGACTCAACGGGACGGGTGAATCACAGGGGCTCC

TTTTTTTTTCTTTCGCTTGTTTTTTTTTGTTTGTGTTGCACCTTCGGTCGGTGCTACACC

CACTAAAGGAAGCGTTCTCGCGCTGACGCTATGAAAATGCAGCGATGAGCGCGCCACTTT

GTTTTGGCGTGGGACGTCCTTCCTTTAAATTGCATACGTTGTCTGCAACGTATGCAGTTG

CCACATAACAGGGCGTTTTCTCGTGCCTCATCCCTATGTATCTTATCCCTCCTCATTTTT

CTAGATGGTTTCAGCTTTTTTTTTCCCCCCCCCTACACCTCACAAATATCAGTAGCACAC

TCTAAACGAGGAAATCTCTAATTCTAATTGTGTGTCTGCTGCACTGTGCCGCCGCTAACC

TACTACATCCCTCTCCCGTATTATTTTTTTATTTTTAGATTCGTTGCGGCGCATCTTTGT

TTTGTGTTGTGGTTTATGCTGCGCATTTCTGTTGTCCACTATTTTTAGATACTGGGTGGT

TGAAGGTTTCGCAAAAAGAAAAAAAATGTGGCAACGTGGGGTGATTCATAGATTTTGAGA

GGGAAGCAGAAGCGAATGTACGCGATTCTACAGCGTGAAGTGTTTGTGGTGTAGAGTGGG

TGCTAGTTCCTTCTTATGCGAAGAGGGAACGGAAAGAGACAAGGGAAAAATC

>Tb927.4.1000 | Trypanosoma brucei TREU927 | hypothetical protein, conserved | genomic | Tb927_04_v5.1 reverse | (geneCodeEnd+0 to geneEnd+0) | length=3313

GGGCACCCCATTTTTATTTACTATTTTTTTCCCCCTTGCCGTAACCGCGGCCGCCGCCAT

TTTTTTTTTTTCCCCTCCCCTTCTCTTTCCCTTTCAGCCAGTTTTATTAACCTTGAGGCA

GTTTTTTTTTTTTTTGTGTGTGTTTGTGTCTGTGTATGTGTCCCTGTGTATGTGATGTTT

CAGAAGTATATACGTCAGTGCGGCACATATTTTCCCTTTCTCTTTTTTCCCTTGAGTGTT

TCCTCCTCTCCCCTTAACAGGCACGGGGGCGCATATTTTAATATATATATTTACATGCGC

ATGTCGTGCCTGCGTTTGTTGCCGCACTTCAGTGAGGCAAATTTCCCTTCAAAAAAAAAA

TAATTACTTTTCAAAATTCACTTATTTTTCTTCCCCGTGCCTCTGTTTTTCTTGATATGC

TTCAAGCAACTGGCTTTTTTCTTTTCTCCTTGTTTTCGTTTGCTTTTTTTTTTTTTGCGG

CTTTTCCCTTTTAATGCTATTTTATGTTTTTATTTGTTTGTTTGCCCCTCCCTTTTCCCC

CTTTGTTTATCTCCTCCTGGCTAATATAAAACAGAATGTGTTTGCGCAGTGGATTGCGTG

ACGCGCGTATGTGCGTGTTTCTCTCGGGCCATTCATCTTTTTATTTATTTATTTATTAAC

CTTCTTTCTTCTTTTCCCCTTCTGTCAGTTAATGAATGAATGAAAGAATGAAGGAAAGAG

TGAGTGATCGAGTGGAGGAGAAATCATGAACGTTTTCTTTTTAAAAAAATTGTTTACGGA

AGGGTGCTGCACTTTGTGGTGGATTTGTCTGCCCTCTTTCTAAAGTTTTCCCACCTTTTC

ATCATTTCCATCACTTATTATTATTATTGTGCACCATTTCTTCAAATTCTTCCATGATCA

ATAATCCTTCGGCCTCGGTGGAGAAAACTCCACAGAATAATAGCATTTTTAAAAAAAAAA

TGTGAGGAGTTATGTTTTTGTCAAATGGGACGCACCTCCGCTTTTTTTTTTAAAAAATTC

CATCACTTCAAACTGCATGATTTCCTCTTCTCCATGAACGCGCAGTTGTCACCAAAAGTC

AAATAAATAAATAAATTGAGTAAGTGGATGAATGAGACCAATGTACCGATCTTGTTTATC

TTTATATTATTTCTGTATGAATATCCGTATGGGTATCCGCACTCCTTTATGTGTCTATAC

ATGTATTTATATTTACATTACCGCACATGTAAAGTGAGTAAGCCAGTGATGAAGTGAAGT

AAATGGGAATGGGAGTTTCTTTTCTTTTCTTTTTCCTTCCCTTCTCCTTCTCCTTCCCTT

TTCTCTTTTCTCTCTTTTTTTTTTCCCCCTTTCTCTCCCTCATTTATTTATTTATTTCCC

TTCCTTCCTTTCCCCCTCTCTCTTTTTTTTTCTTTTTTGAAAGTTATCTTAATTTGATCC

CAACTTTTTCTTCCGCCAGCTGATCAAACCTCCTCTCCCTCTTTTTGTTGTTGTTGTTGA

CGTTGTTGTTGTTTTATTTTTTTTTTTTTCTACTTCTACTCTCACTTTTTTTTCTTTTTT

ATCACCACGGCGTGCTTTTTCTTATTGATAACAATAACAGCAGCGGTAAAAAAAAACAAT

AATAATAATAAAGATAATTAAAATGAAGACGGGGTCCGATGATTTATTTTACCAACATTT

GTTTTCACTTTTTCATTTTCATTTTCATTTTCTTTTTTTTTTGTTTTTTTTTTTTCATGC

ACGTCACATTTTTGTTTCCCCGTCAGGCAAATGAGAGGACTGAGTTTAGCGGCTGTTTGA

CATTCCCTCGTAGATTTTTCACAGCGTCAACTCGTCATTTTTTTTCTTTTTTTTTTCTTC

ATATAATTATATCGTTAACTTAATTTTATTTCATTATTTGTCGTGTCATGTTTTCCTCTG

CCGTGGTTTGAAGAAAGGAATAAGTAGAGGTGATATTGTGAGAGTACAAAAGGAAAGAAA

AGAAAAGGTGTGGTAGAGTGTACAAAAGAGGTAAAAATGAAATGAAATAAAATGTAATAA

AATAAAAAGCCAAGAAATCGTAAGAAAACGGAAGTAGTTTAAAAAGAAAAAAAATGTGAA

ATAATAGTAATAACAACAATAATAATATAGCAACAACAACAGCAACAACAATAACTCAGG

GAAAACAAAAGGAGTGTGTGGAGATGTGGCGGAATGAACAACAAGATGTGATGTGCGGAA

AGACGGGACAACCAAGAGAGGAAAAAAAAAAGAATAAATAAATGAAGAAACAACAACCAA

CGATAAGTGGCAAACTTTCAAAAAAAGCAAGAAAAGAAAAAAAAGTCCATAAAGAACACG

TGTCGTTTTATCAAAACCGTTGCAGTTGTTTATTTATCATCCCTAGTCATCCGTGTGTCA

ACTCATTTCAGTCCATATCTTTTCTCTTTTCTTTTCTTTTCTTTTCTTTTCTTTTTTCGT

TTTGCCATACCTTTTCAATGCGCATTTGTAACGAAACCCCGCTGGTTCGGGGTTTTGTTG

CAAGCGCAATTGTTTTATTTGAACTCACATCTTTCAGATGTGAGGCTCATGTTTTCTCGT

GGATTGCCAGTTTTGCTTTCGTGTGGAGGAAAAGTGAGGATGTTTTTTTTTCTTTTTTCT

TTTTTCTTTTCCTTCCTTATTCCTTATTCCTTCTTCCTTCTTCTTTCTTCTTCTTTTTCC

CTTTTTTTTCCTTTACTGCCACACATGTGGAGGCAGTAAACAACAACCACAATAACCACC

ACAAAACAAATGCCATATTGCCGGCTCTTGTGTGTCTGTGACTCCCCCTATCGAAATGGC

GGAATGATGGAAGAAAAAAAAAACAACAACACTTTTTTGTTTAATTTTTACTTTGGAAGG

AAATGGTGACAACACAATCGAGTGGTGGAGATTCCGAAACTCAAAATTGTTTTGCCTACC

GGCCACGCCATTGCCACTCACTCGGCTGTCACTTCCCTCACTGCTTTCTTTTCTTTTTAA

ATAATAATAATAATAATAATAATTATTATTATTACCACTTCCATCCGTTATTTTGTTCCC

ACGTTGTGGGGGTGTTCGCAACATTAATGCAGATGCATGAAAGAGCCTTCAATGGCGCAA

ACAATTGTGTAAATGTGTGGAGAAACTAAAGCTATGTGTCTGTGTGTGGTATTGTTTAGA

TGCGCTGAGCAACTGAAGGGAAAGGGGGGAGGGAGGAAAAAAAAAAGAAAGAAAGGAAAA

GGTATGTGGGAGGGGAGGGGGGAGGACAGTGAAACGACTACATGTCAGAGTAAAATGAAA

TTAGGAGGAAAGC

>Tb927.10.6770 | Trypanosoma brucei TREU927 | BRCA1 C Terminus (BRCT) domain containing protein, putative | genomic | Tb927_10_v5.1 forward | (geneCodeEnd+0 to geneEnd+0) | length=707

ATGGTGCCTAGGGCCATTATTTATTATTTTTTTTACATTAGATATTACCATAGATCTTTT

AATTGTGCTTTCTGTTTCGTACGTTTTATTAAGTTTCTTTTTCATTTCGTAAGCTGGTGT

TCTGTTTTGAACATGTACTTTGCGGCACAAAGATTATCGTTGAAGAAAGTGTGTTTTGTA

CTTTTCGTTAAGTTATTATTCTTGTTGAGAGCTTAGCGACGCGTATCGTACAGAAAGTTT

GCTAACTTTTCCACCACTGGTGTTGATGAATGGAGCAGCCGGGCTTGTTTGCACAGGGGG

TGTGATGTTGACGTGTGCCTAGATATTAGATTGTGGATTTTCCTAGAAAAGAGAAAGGTT

TGTATGCGGTGGAAGTGTCTGATGCGCGGTTTCGACCAGCGGGCCGACATTTGACAGAAG

AGATTCTTCAGAGCTATTTATTCCCGATTGAAGAGTTTCAGTTGTTTTATTTCTTTTTTA

AAGTTTCATCTCTCTTTTTCTTCAGTGTGCGGTTCCCTTCTTCTCAGTTGACTTTCGGTG

ACTCTGTTTCATTGCGCCCTTCTCGGTTATGTTGCTTCGTGCATATCCGTTGTTTCTTTT

TTCTCCCCTTCTCTTGTCATCTTGCTTTGACTAGTTTCATTCACAGGAAGGTTGATAATC

GTCTCCACCCCGTTTTTTTTTTTGTGTTACTCATCATTTCGGAGGGG

>Tb927.11.15200 | Trypanosoma brucei TREU927 | hypothetical protein, conserved | genomic | Tb927_11_v5.1 reverse | (geneCodeEnd+0 to geneEnd+0) | manual length=720

AGATGAGATGAGGGAGGGAGGAGGGGAGTATTATGTGTATGTGTATGTTGTTTCTGCTTTTTTTCTCTTCTCTTTTCTTTTCTGTTTTGGAGTAGTGAACTTATACCATGACCAAGTAGGAGAGAAAAAAAGGGGGGGACACAAAGGAGAGAGACGATAGATAATGTGTAGGAGGTGGTGGGAAGAGAGGGATATTTCATTTATTATTGTTATTCTTTCTTTTGCTCACCCACCTCTCGGTTTGTACCGTCCAAATACTTGTAAGATTTACAACACCTGAAGTAAGTGAAGGGCTAATGAGCGTTACCGCTGTTTCCTGCTGCGACTTGGTGAAATTACACTGGAGTTGTTTTTTTCTTTGTGTGTGTGTATGTTTGTGTCTTTGTGTGTAAAGCTCCTATTTACTCCCTACAGCTTCGTTTTTAGTGCAAAGGGATAGGGGAACTGTCAGTGCTGTCAGTTTTATCATTCCCTTTTTTCCGTTTACCCGTTTCTCCCTTTCATCCCCTTACTGCCAACCGTTAGAGGGCCTCTTTTTCACTATTAAACATTATTTGCCTTTTTTTTCCATTTTTTCACCACTTCTCCGTTTAACTTCTTTTTTTATATATATTGCCGGCACGAATTCTTGCATTTTTTGTTGTCGTGCTGTCAAAATGTGAGTGTGTGGTGTGCTCACCCGTGCAGTGCTGATAACGTCGTGAAGTGTTTTCTTTTAAA>Tb927.11.1480 | Trypanosoma brucei TREU927 | receptor-type adenylate cyclase GRESAG 4, putative | genomic | Tb927_11_v5.1 forward | (geneCodeEnd+0 to geneEnd+0) | length=892

ACGTCACGCCTGCACGTGTTTATGCGGACATATTAGCAGTTAAATATGTACGATCTTCCT

CTGTATAGTCTAGTTATTAACCATTGAGTTAGCCAAGACCTGTTAACTTTCCGGTGTGAG

CGTTCTTTTTTTCTTTTCTTTTTTTGTTTTCATTTTTTACTTTGAGTACTTTACAAATCA

ATAAGGCTTCGAAAATGTTGTTTTCCTTTCTTTTTCCTTACTGCGTTACGGTAGCGGCGT

TTTTTTTATCTTTTTTAATAACCTCAGGGACCTCTATGTAATATGCCAATTAGTTACATC

TCTCTAGGATAGGCATAATTTCTCATGATGTATAAATATAAATATCTTTTTTTATTTTTA

TTTTTATGTATATGCTTATCTAATCTATGTATTTTATTTTCTTGTTTCATTTCCTCTTAT

GACTATTAATGTTTTCATCTTACACTAATTTAAATTTTTTCCACCCCGTATTACCGAACA

CATTTGTCACATTGGGCTCCGGTTCGGGATTTTGAAGACGCCTGGCTCATGCCTTCCTCC

ACTGCATAACACTGCCCATAACCGTTTAGCGGCACATAATAATATAAATAGTAATAACAA

TGACAATAATGATAAATGTTTTTTTCTCATGAGCGTTTGCGGTATTACCCATGTATTTTC

TTTGTTTTTTTTTTCGTCATTATTGCCGTTATTTTCATCACCAATTGTTTACTCTCAACC

TCCCAATGACACTTCCCTCAGCCCAACTGAATGCGCCCATAGAGCGCTTACCGTAGTGTT

TATTATTATTTTTTCTTCCGAATCAAGTTAGTATAAATTTACTCGTGTGTTTTAATTTGA

TTTAACCTTATGCGTAGCACAAATGCATAATATCTTAGCAGGTACTGTCTTG

>Tb927.10.2420 | Trypanosoma brucei TREU927 | hypothetical protein | genomic | Tb927_10_v5.1 reverse | (geneCodeEnd+0 to geneEnd+0) | length=504

GTTGCTTCTTTGCTTAATGCGACTTTTGTTTCAGGAAAATGTTTTCAGGGTTCCTCTTCA

TTACTTTGGCTGTGTCCGACCACACATATGTTTTTATTTCTGCTAACGAAGGGGGAAATA

AAGCCACTCAACCAGCGAATGGAAAGCTGTGGCTGTCATGTTTCCTACCCGATACGTGCC

CTTTCTCTTTCACTAGGCCGTCATGAGGTATGTGCATACACCGTAAGGTATAACACTGAT

TGTTTTTGATTATATAGGGGCGAGTAGTACTCTGCATTTATGTGTATACATGTGTGTTGT

TGTTGGTTTTTTTCTTATGCCCTAATCCAATTTATTTGGGCCTCCCCACACTCTTATATT

TGGCTAGTTTCTTGTGCATGTGCCCTGAACGATGTCTCGGCGCTGGTGCCGATCCCTCCC

CCTTTTGGTTTCCTCATTAAAAGCTTTTTGTCGAGCTCCCGCAAGCATGAGTTCGTTTAT

GTCTATTTGGTGTAGGCTGCGTCG

**SECTIOn #: Manually corrected estimated 3'-UTRs**

>Tb927.9.6960

GGAGCCCCATTAGCATTCAAACGGAAAACTGTGCACGTGTATTAGGGGAGACGTTCCTTTTCTTGTTGGTGCTTGAATTTGTATCCTTTGGGATGAGCAGCGTCTGCTTCATGTTGTTCTTGTTCCTTTTCATGGTTCCAGAGGCCGACATTTCCCCACGAAACAAAGAGATCATTGAACAGCATCCCCGAGTCCTGCCACTCTTACTTTTTAAATCATTTGCCGCGTTTTGCGCCGATGGAGGGCACATGACCGCCCCCTTAATTATGTGAGGTTCTGTACCTTATTATTATTTTTTTTTGATTTTTAACCTTCGGCTTTATTTATCGTTATTACTGGCTTTCTTTTTTTGTTGTGTGTGTGTGTGACAGCTGCCACTGTCCTGAATGTAATCGCTTCAACTGTGCCTTACTTACTGATCCTCCCACCACCACGTCCTTTTTCTCTGTTGTTTTGGACGGAGAAAATATTTGCGTGTGTGAAAGGAGGTTCGTAGTCATCGGGCAACAGCTCCCTTCAGTGATGGTGAACCAGGCATGCTTTTACTTACTTTTTTTTCAAGTTGCGTTAACAACGCATTTAAGTTGACGTCTGTGCGTCTATTAGTTGCTGTACATTTTTATCCCTTTCGGTTTTGGTCTTGCTGGTGGGTGTTGTTTGAGCAACTAAGTGGGAATGCACCTTGTCGGAATTTTGTTGTTATTTTATGCGCGAAACAGGCACCGCTCAATAGCTTTAATGTAAATATGGCTTCTTTCTCTTATTCTTTGTGCCCCCTGGCGGCGTGCCTATTCTTCTTTTGTTGTTCTGTTTTTCGGTATCCTTTCGACGCTTTTCACTTTATTGAGCAAGTCGCTTTCAGCGTCTGTTTCCATTTCCACTTCGAACGGCGTACTATTGTGTTTTACTTTTCCTTGGGTACTTGTTTTCTTGGCGTGTGCTGTTACACCCTGCCATATCTTTTTTAACACACTCTTCATTATTCTACTTTTTTGCCCCCTTCCACTTCTTAAATGTTTGTTATTTATTTGCTATTATTATTATTTTTTTTTTTGCACCTCTCATGTAAACTCGTGCGAAGTACGGGGTGGAAAGAACGGTCTACACTTCCCACCCTCTTATTGTCCGTCTTTAATCCAGACGTGGCATTTACCAATATCAACAATGACAATGGGCGTTATCACTGCTTACTTCAGTGGTCTAATTAATCTTGTTCGTTTAACTCCGTACTCGCTGTTGAGACAATGCAGAGTGTGTGCTACTTTTCTTTTATTTCCCTTGCGCCCCGTGCAAGTCGTTGAGACACATGGTTTCCCTGCTCTCACCGACGTTGGTGAAGGTGCACACACATCGCGGCGGTGGTACCCGCCGCAAAACATGGCGCTTGGAAGCATATTTTTGAATAGGAGCGGATTACCGCTTGCTTTACTTCCTGCGCTCCGCTCCTGCATACGCTAACCATTATCATTAATATAACTGCACGATAA

>Tb927.10.10770 beginning

AATGCAACCATCAACTCGTTTGCCAGATGGTTATTTGACTGTTTGTATTAATGCATTCGTGTCGATTCTTACGCCCATCCTTATTTATTTTATCATCCTCTCTCTCCTTTCGACTACAGCATTTCTTGTACGTCGTTACTGTTTCTTTACTTTTTGGTTTAACGCCGTGTCGTTCTTAATTTACTTGACGAGGATTGTTTTTATTTCCCTCTTTTGCTAGTTTTATTTATATAAAATATATTTTTTTGTTTTCTCTCCCTCTCGCATACTATTTTTTTTTCTCTTGTTTGTTTACACTTCCTCGCCTCGCCTCGTTCCCTTTCTCCTTTACCACAGGAAGGAGGGTTTTATGCGTGGGTTGGTTGGCCGACTCTCTTTCCACGAGAGCGAAAAGTGCTCCTGCTCTTTATTCGTTTTGTTTATTTTATCGAGGTGTCCTTCTTTTTCCCACGGTAGCTCCCTTGTTTACAATAGCGGAGGGCAAGCACTGGGGGTTGGTTCCTTGTCAATGCTCGAGAAGGATGCTTACAACACTCATTTCTTATATATTCCTTGTACATCTCTCACATTTATCATCACTCTTCTCTTTTACGTGACGCGCGCCTTAGTACAACATCATTGCCGCTTCTGCCTTGTCTTCCCTTTGCCCTGTAGTCCTTCAGACGAACTCTATTATCCTGTTGGATGCGTATCTGAGGCGTTTCATGCTGTAGTCTGAAGCCATCGCCTTAAGTTTATCGTATGTAACCTGCTGCCATTGAGGCGGGAGAGCACCAGCGGGCATTGCTGGAGAGGGAAACCAAAAAACCGCTTGTTGGGGTTGTTTTTTGTGGTTGGCTGCTCAGTGGGGAAGAAGAGAGAGGACTGCAATCCGTTACTTAACGTGAGTGATATGTATATGTCTGCTTGTGTTTGGTTACATGCAACGTGAGGCAGACACATGTGGTAATATTTCTAAGCACAAACACGGGTATG

> Tb927.10.3300

AAAAAGTCTTGATGCCGATGCTGATGCGGGCGATTTCTGCCACCTGACTATGCCACCTCGCGCAACGCGGTGTGAAAGCAAGGGGGATGCTGTGGTGGCTTCTGCTATTTGTTTATTTGTATCAACTGTATTTTGTTTATTCTTTATTATTTGTTTTATTATATATATATATATATTTATTTATTTATTTAAAAATTGCACGATTGTTATTTTACAATTATCATTGCGAGAGAGGAACTGAGGTGTGTGTGTGTGTGTGTGCCATAGGGAAGGAATGGCCGTGGTAGTTGTGAGCGTCGATTCTGTGGTGACCCCGTGGGCCACGAAAGCGTTACATTTGAAAGCTTTTTATTTTTTTTTCTTCGTCCCTACTCTCACCATGTATTTTGTGGGAATACTGTTGGGTCCATGAAGAAACATGTGCATTGTTAACGTTGATGTGAGTGGAAATATTGAGATTTGGCAATGACAATCATTATTGCTGTTACTTTCTTGTCTTATTCTTTGTTGTTACTGCAGCCATTACCGTTACCTTCCACGGTGAAGGAATTATCTTTAATGGTGCTTCGTGCTAATTCGATTGCCACGGCGGGTCATTTCACCCTGCCGATCTTCCTTTGATGTAGCGGTCGTTTACTGCCATAGTTTCTGTTCGTGTGAGGACCGCTGTTGTGCATCCAGTGGGTGTTCACTCAGTAACCCCTTTTGTGACCCTCGATTATTTGTGTCACGCAGTTGCGTGACACCCCCTCCGCCCTCAATTAAATTGTTACCTAATGCGGCACCAGTTAAA

Tb927.9.4310

GTTGGTGGGGATAATTTTTCCCGTCGCGTTTGCATGTGCATGTGTGTATATGTGTGTGTGCGTGTGCGTGAAGGGACGCTGCTGGCGTGCCACTGTGGCATTATAATTTTGTTCTGTGCTGGGAATGCGCCGAATGCGTTAGAAGATGACTCAGTTAAAGCAGAATGGGGGGAAAATTTAAAAAAATGAGGAGGGAAAGAAAGAAAGAGAAGAGAATCATTATCGTACGTGGAGGTTTGAGTTTTTTTTCTTTATTGTTCCTTATCATTCCATGAAAACGAAATCATGAGGTTTAGCGGTTTTACCCCCTACCCCTACTCCCATTCTCATCTCTTTCTCTTTGCCTCCCCTTTTCCTTTTTTCCTTTTACCTATTTTTTTTTTGTATAGGCATGTTTCGGTAAAAGCAGGGGGATGGTGGCACTTTTACTTTCCTTCGACGCGAGGCCGACTGAACTTTCCACTATGATGAAGCGGGAAGATGCGGTAAAGGGTTAAAAAACGGAGTGAACGATTTTTATTAATTTGTTTAAAAAGAATGAAAAGAGGAGGTATTAACGAATTGTGTGGATAACACGAAGCGTTTTTTTAATAAAAGGAAGAAAGAAAGGGAAAGCACCTTGTTGTGCTGTATCCTCTTCTCCACATTTTTAATGATGACTGCGTGGTTTTTCTTTCTTCCTTCTCTCTTTTTATTCCTCTCAGAAGTATGTCTACCCCCGAAAATATATAAATATCCAAATATGCAGGTGTGTCTGTATTTATGTTTATGTTTGTATATATATATATATATATATATATA

>Tb927.9.5900

AATGTTTCACCAATTCGATGAAATTAGTGAGTTAACAAACCAATCATACGGGAGGAAACTGCAGCAGTATCTTTATACACGCACATAGTTATGTGCATGTATATTTGTATATGCGTGTGTGTGGGAGACATTTGAGGGAAGAAGATACAAATTTGAAGATGGGAAGGAAACAAAATTCAAGAAGAAAAACGAGTGAAAGGGCATTAGCTAAAGAAAAAGACGATAAACGCACACAAAAAAAAATACACCAGTAGATGGAGAAAACGTTTATTTATTTTTTTTTACTTTTTTTTACTTGTTTGAAGTGTGAACGGGTGGCACGTAACCAGTGGGGCAGGGGGGGGGAAACAAATGGAAATGTTTAAAATTTAAAAAAAAACTGAAACCCGCTAAAGTTTAGGTGGTGAATTTGTGCTTGCGTTTGTAACCAACTGTAAATATGTATAGAAAATATCCATGCATATTTATTTTTTTAATTAAATTATGGTTTATGCATGTAATAATGCAGTGAAACATGAAGAGATGAAATTGTATTGGTATATGGGCCCTGGTTTTCAGGTAACAGAATCTGTACGCGTGTTTAGTGGGTTACTTTTTCTTCTTTTCTCCCGTTTCCCTTTCTCTTTTAGTGATGGTGCTAACAATAATTATAAATATATTTATATTTCTTTACTTTATTCCGTCTTCCACCTGATCATTATGTTCAGGTGTAGGCATTAGGGAGGGGCGGGTGATGTGATGTGTGTGGTGTGTATGTGGGGAAGACAACACATAAATTTCATTTGTGCATTTCCCCCTCCCCCCCCTCTCCTTCTTCCTCCTTTCTTTCCTTCGAAATCTGCAGAAACAAAAAATAATGGTTATGATGACGATACGGAAGAAGACGGAAGGGTGGATGCACTGGTAGAAGTAAAAGTAGTGGGAAAGGGGGAGAACGTTAAGTTAAGGTAACGTGAGACAGGGTAAAGAAGAATATATATATATATATAATTTGATAAACAAATGTTGAAAAAAAAACGCAACGACGCCATGCAATTTGAGCGTGTACCAGTTCTCCATTGGAGTGATAAACGGCATCAGGTCTAGTTTACAGCTTTAACTTTTTTTTACTTTTTGTGTGCTATTTTCTTTCTCTTTTTCGTTCTCTTTTTACTATTATTATTATTTTGTTTTGAGGGTAGAACTTCTCCGACTTTATCGTTTCTTCCTTTGGTAGAAGGTTATCCACCATTTATTATTATTTTTTTGTTTTCCCTCTTTTTTCTTTATTTTCTCCTTATTTTTCATGTGGAGTACCGTTTCATTGAGTTTTTATGCAAAGAAAATGAAACTGGCTAAAGGGAAGAGAGAAAGCAACAAACAGTGAAGGAAAAGAAGAGGAGGGAATCGAATGAATGAATGAATGAATAAACAGGCATTGGCATATAACTTATAACCGCAATAGAAGTATTCCCAATTTTTATGCCACTTGCCTTCTGTTTTGACTATTAAAGTAATTTTGTTTTTATTTTACTCTTTTTATTTTGTTTTAATTCTTCATTCATTCTTATTCTGACTTTCCATCCCCTCTCCTGCTTTCGTTTTCATTTTATATCAGCTTTACACACTCAGCAGCCACTCAATAAAATTAGCGAAAAAATGTCGATTACTGTGATGACACCGAAAGAGTTGTAATGATCATTTGTCTATGAAATATATATTTGGGGCCGCGCGTGTTTATGTATGCTGAGTGTGTGGATATTTTTTTCTTTTCCTCCCCCATCTAAAGCGTTGTGGGGGATAAATAATTAGTTTCAGGGATTGAAGGAACGGTGATTGTGTGTGAGAGGAATTGTTTTGGTGTTGTGACAAATTCCGCTGCTATTCAAATTCATTTATTGCGTTTCGGAGATTGACAGTTGAATCGGCGTTTTGGTGCCGGTGAAACTGCATGCGCAAATAGGAGGACTGTTCTTTTATACCTTTTGGTTTTATTATTTTATTTTCCTCACCCATACCCTA

>Tb927.10.2350

AACACATTAGCTACATGCCTGAAAGCAGTTACCAATGCAATTATAAGATGTGGGTAATACAAACGGTCGCGTGGGTGTTGTTAACGTGTGTGTAAGTGTGTGTTTCCCCCGTTACTGCATGCGTTTGATGGTTTTGTTATCCATTTACAAAAGAAATGGTACAAAAGGGTGAGGTAGTGTGAAGAAAGAGCGGAAAGTTTTCCGCGATGTGAACACAATGTCAAAAAGGAGGGAAGGTGGAAAATATAATGTGGTGATGAAAGAGCCGCTCCATCATTCCTTCCTCTTTGTTATGCAGTAACGGTTGAGTTTTTATTTTATTATTTTCGTATTATCATTATTTTTTATTTTTTTATTTCGCTATACTTTTCTTCCTTTTTTAACGTGTACTTCATTACTGCGTGCGTGTAAGTGTTATGTATTTTGGTTTCGCTCATTTGCCTATGTCTGCCTCTTTGTGGCTGAGTTTGTTCCCTCACATTCACTCGCCTAGCACATACAAATGGAAAGAAGAAGGCAAAAGAAAAAAGAAAGAAAGAAACACAGTAAATGTGGCAGAAATGCAAAATGAATATTCTCCTCCGCACCACCACCAAGAGGAGGAAAAAAGAAAAAGGAAAAAGGAAAAGGCAAGTGAGCTGGGAGATGAGAGGAGATGTAAGTATCCTACGTCACGTGTTGAAAGTAGTAAACGTTACAATATTTTTGCTCCTTGCTGGTTATTTGCGCAGCGTCTCCCCTCTTTTAGCGTGCCTTGCGATCATGTGTTTTTGTTTCGTTTGTTACGTGAGTTCATCTCCTTTTTTTTTGTGTGTGTGTGCTTTATATATAAATTCCTTTATGTCAGTACACGCCGTGCCACATCTCATACCGTTACTTCTGGTGGATGGGGTGGGAATTGTAAAGGTATGTGTGTCAGAGGAGGGGTGTGGGAGATCAAAAGAAAATCAAAAAAAAAAGGAAAGCGCCTCCACCACATAAAGTAACGGGAAATTGATTGACAAAAAAAAATAATAATCAGAAAGTCAAGAAAGGAAGAAAGAAAGAGGGAGGAGGGAAGGAAGAAAGGAAGGGGGGAAGCACGCACCAGAAGTGTAGGAGAGAAGGAGTAAGTAATAATAAAAATTTTAAAAATAATTATAATTATAATAACAGTGCGAACATATTTTATGGGGTTAATAACACAGGGTATGGGAAAACTAACACATCTGTGTTCTCACGTATGTGTGGTGAAGTAACAAAGCAGGAATAACAACAACCGAAGGGAGGAGCGTTTAATTTCAGATGCAAATGTGTTAATGTGATGGAGTTGGCGGAGGGAAATAAGGTTAACGCTTTTTTCCACCACCCCCCTCCTCCGCTACATTCCTCTTTCTTAACATATGGCGTGTTGTTTTTTTTTTTTGGTGAATCAATTCGAATGAGAAATGTCGTTTCGCTCTGGTACGCCGCTGGAGCCTGAAACTCTTCATGTGTATTGCCTCGACAACAACTTCTATACTCTCATTGCTTTGTTTCTGTTTCTTAACCCCTCGCTTGTACGCATCTTTCTGCTGTTCTGGCCTTCTGATTTCCCCAATACATCTTTTGATGTCAATTGTTAATCTTGTGCTGTGCACATACATTCACAAAGGGAAAGGAAAAAAAAACAAGAAGAATAAACAACTATGCCACTTTCCTCCGAGAACAAGCAGAAGTTGC

>Tb927.4.5190

ATTAAACGACCACGACATGATTTATGATTTCCCTTATCCCCCGTCTCCAGATAGGAGAAGGACCTTTCTATCCACAGGATAGGCAAACGCTCTGACGGAGTATATAGGTCTGTTAGTCAATTGCACAGAAGGTTTTTTATCGTTTTACTATAATACGACTTATTTTTTTTTCCACCGAACATACCTTTTCCCATAGTTTACACTTGTGCAAAAATACATATATCATCGACAACTAAATCTGTAGCTGATGTAACCAGGGTAAACTGCTTTACCTCTGTTACAACCCCA

>Tb927.7.5550

ACCAAAGCTATCACTTTGAGAGGTGTCTTTTCTTTCGTTTTTGTATATGAGGCGTCTCTGGGTACGTGACAATTTTTTTTTGTTTTGCTTTGTTTTGTTTTTATTTTTTTTTTGTTGCCTTGCGCTGCTTTATGTTACGTTTGTCCTTTATTTTATTTTTTTTTGTTATTTTTATTATTATTATTATTTAATGTCCCCCTTACTCTTGGGCTTTCGCCCGACTTGATACAGTAGTTATATTACTCCATATATAAGCAGACGAATGCGTATATCTACACATGTATATAGATATGTTATAGCACAGTACACTTATTTGTGTATGTGTGCTGTTTTCTTCTTTGTTCTGTTTGCTTTTTTGATGTTGTTGTTCC

>Tb927.11.1830 (more than one copy)

GAGTCACAGGGCGCCTCGCTTCCCTTTTCGTCAATTAATTGAGTTCTTCATTTACTTATCTATTTCTGTTTTGTCTTTCCACTAGACTGAATCGTTTATTCTCCTTACATCAGATTTATAATGCACACAATATTGTGGGATAGATGACAATGAGGAGTGGGGAATGGAGGGAAATGAGTGAAAAGAGAAACATTACAAAAATTGTACCTTTTTTTCTTTTCCCCTCAGTGTCGTTTATACATGTGACAGTATGTAAATTGGCCCTCTTCTTGTTCCTTTCGCTTTCGTTGTGACGCGCCTTAATGGGACGGGTTGTGTATCGTTTGACAATCATTTAGTTCTTTCTCATCCCTCTTACTCTTTTTAGTATTTTTGTTGTGGTCCCTTCATTTAACTGATCTAGCGGAGGAGGAGGCCGTCCCCCTATTTATCTCGTGAATAAGCGTTGGGTCTTCCTCCCCACTTCTCTACCGGGTGAATCAGTACCGCTCGGTCGAACGTTATCTTAATTTTTTTCTTTTGAACGGCACTTGTCTCCTGTATTTGTTGCATTCTGATCTTGTTAGTGTTTTTGTTGGCGTTATATTTTTCTGATCTCTCTTTCCTGACACAATTTTCCGATCGTTTTTTTTTCCGTTTTCCCACATGCGCATGAACGCGTGGGAAAGGGAGGGGGTGTGGGAGTGTATTGAGGAGATAAGTCGGCGCGAGTGTATCCAACATTAGGGATATTCTTCGTAAACCATTATGCAATATCGCACATGTTTGTATATATTTATTTATGTTTATGCGTATTTTTTGTTTTTGTTGTTCGGGTTTGGCGTTTATTTTTTAAATATTATTGGTAATTCAGCGAACAATTTAATGGGGTACGGAAGAGAAAAGGTAGAGTGTAAAGGTGGCAGTATGCCAGGACCCTTGCACGCACATATACAGTCACATATGTTCTCAACACCTCTTTTGCGCGTAGCATTTACGTTTGGTGCCGTCAAAAGTTATCGGGAGGCAAAAAAGAAAAGAAAACAAACGGTTGGTGGATTGTGGAGTTGTCCACATCTCTCATTACTGGTGAGGTGAAACATGTATGAGAGATTGCTACGGTTGTGCTCCGTGCGCTTTTTCTTCCACACCCGCATAATAAATGGAAAGAAATATGTTTAATTTGGTGGAGGCCGTCGAACAATTGAAAGACGGATTTTCTTGTTGCGATGAGCAGCAGGGGTTAGGAGAGGGGTAGCGAGTGACGCGGATAAGTAGGATCCAAAATAAATGCAGCCAACGACCACTTGTGCTTCGTCAAGGGCACTGATGTTTGCTCGAATGTTTGTTAACGTGAACGGTCAATTGAGCACCGCAATTAACTGTGCATTGCAGGGGAAGGGGTCCATCTTGTGCTTTCAA

>Tb927.4.5060

AAACACTGGTGGTGTTGATAAATTCAGTGGTAACGAGAGAAAGCATGGGGGCCTGCAAAACGTTACTGCGTTGACCAACCTCATACGTGCACAGGGGAAGTTTCCTATGCAAAGCACACCGGATGTAAGCTGCGGGGGTTACCGTCCACAAACACTCACTTATTTTTCGTATATGTATTTTTTGCTCTCCCTTGTTTGAGCCAAGTTTCTCAGTTTAATTGTTCTTCCGTGGTTTTATCTTTAATGCCACCGTGTTTACTTTAGTTGTGGTTTTCTCACCTCCCTTATTTTTATTACATCCCAAATGGGACCCCGTTTTCACAAATAAGTGATTATTTTTTCATCTCTTTAAGTTTGCCTCCCATTTTCGTTTTTTAGCGGGTTGTTTAATGCTTCTCCATATATGTATGTATGTTTCCGTCAGCGGTAGGATTACCTGAGTCACCTTGGACGACTCCCATTTTTGTTTTATTTCATGTGTAACCACGTAGTGCACGATGTGAATATGCGACACATAGCTTTGTTCATCGGGGTATGGTGAAGCCGAAATAGTCATATTTATGCATTACGCTTGTGGTAGTAGCTCGCCCCGCGCACGATGTTGCTGTCGGCCATCTACCCACGGCACCTTTAGTACTTGTTTTTCATTGGGGTCTAAGTAGGTGAGTCCTAACACCTTCTTTTAATTCTACCTCTCCATTTTCCACAATTTTTTGCAAGTGTTTGTGTTTTATGTGCTTGTTACTTTTACTGCATAATGGAGAAAGAAAAATGCCGTCAGATGTCTTTGTTTCCCCACGACTCCCCTCCTGTTCTTGTTGTCCCCCTCATTGTTGATTCCCTTGGTTGCAAGTATTCTGACACATTAATGCAGATAGAAGCAATACTGGGGGGGGGGCTCAGGGTTGAGCGTCGCTCAATTTTTTTATTTTTGTTTTCCGAGAACTAAATGAGCGTCAAACATGTGCATATGCATTATTTCTAACTTTAACGTCTACATG

>Tb927.3.3890

ATATGACGAGGGCTGCGGTTGTTGCCTTTGCATGTTGACAAGTGAGTGAGCCAGTAAGGAGTGTGGTAAAAGCTGTAGGGGTTTGGACGCTGGGAGTCGCAGGTGGTGTAATGTTTTCCATTTTTCCTTTTTTATGTCAGCTCTTTTGTTTGTTTTGTTTTTGTTCTGTGTGTTGTTGTCGAGCGCCCTCAGTGCACTTTGGTAATGAGGGCTTACGTATAAAAGAAGATTGTCGTTTGGTTCTGTTTTACATTTTGTGTTTTCCCTATTTTGTTTGGTTGTCATGGAGGGGATGATGAGCGGTGTGTCGACGGAGGTATCGTGAACTCACTCCCGTTAGTGTGGTATTATTTTCATTATTATTATTATTATTATTTTTCTCCTACACTAAACATTAAAGTTGGTAACGCGATGTATTTTTTTATTTTTTCCACACCAAAGACATGTATACATGAATTACATTTATTACTATTATTGTCATTATTATTATTTTCCACCCTTTAACACATGCCCGGGCACCGAGCACTTGGCCCTTGTACGTTGCCATCTGGGCCTGCGAGTTGTTCCAGTGCTGTGATTATGAGCAACAACCTCCATCACGTATAAATTCAAATTACATTAATGAGGGAATGGAAAAGGTAAACGGACTCTGAGCATACACATTACTAACGAGGCACGCGATGTCGTGTTATTTTCCATATCCGGAGAGCTGAGGAAAAGCAAGAAAGAAAGAAAGAAAGGAGAGGGAGATTGTTTCACACGTTACACCATATCCTCCTCCCCTTTCCGATATTTGTTGGTGGTTGCAGTCGCCGCCGCCGCTCCTCTTCCCCCCTCCACTGGATACGAGT

>Tb927.9.2320

AGGGGTGAAGCACTTTGGCTAACAAACGTGGAGATTTGCTCTTGAATGATGTTTCGGCTTATAAAAAATAGTCAGAAGTACCGTCTTGTGTCAGTCAGCTCCGCTGTATCGTCTTTACGTGTACTCATCAACGAATAGGTACTTGGATTTGTTTATTTGTTAATACGTGTTAAACCTCACCACATGGTTGTGTGAACTCCCGCGCATGACTTCCAGTTGCGGATAAGCGACCAAATACATGTGCGTTATTTTTTGTTAAACTTCTTTTGGGGCGCTATTTCCAACAAAGTTTCCCCCACGCTGCCTTCATTTTCCCAGGGCCTCCGACCAACCGAGTCGCTTATTCATACTGTGCTATATAAGACTTTTTTTAAAAAAAAGGAATGTGGCGGGAGTTGGAAGTACCGCTAGAAAACGTATCGGTAAAAATAAACGAAAATAGAAGGCTATTTTTTCTTTTTGTTTTTTGTCTTCCTAAAGCATTTTTTTCTCTCTCTTTCTATCTTCGTTACCGATCAATGCTTCTTCTCCGGGACTTCATGTTGGATTTTCTTCCACTTATTTTACTGATTTACGCCTCCAGCGTTGCTGAGGGTTTTAGCGTACGCTCGACTAACATGACGGATGTGGTAGGGAGCGAAACCACAACACCACACTGAATGTTTTATAATGCCTGCAGAACTTGTGTTTCAAATCCGCATTTGCTTCGTCTTTTCGAGAGTTTTTACCCCTTTTGTTTGTTCAACAGAGTGCAAATCCGATACACATGTATGCACACTTAAGCGTATTTGAACAGCCTTCTCTTAAATGCTGAGTTTAGGTGCTCCGCTAACCAAAAATGCAAATGTAGACTGTATGTAAGGATGAAAACCCCGTGTGGCCACTCATTACTGTGGAGTACCAAATAATTTCTTCTTAACAACTTAAGCAATGCTGTGCGATGGAGATATTTTTGTGCGGTGGCTGCACCACGGTGGAGGAGTGTTTTTAATCCCCTTTTCCTTTTTTTTTGGGGGGGGGGAGAAACCCGTGGAAAAAGATAATTATATTAATAAAACTTCAATAGGGCGGTGAAGAGAAAGCGAAGCAAACAATTTGTTA

>Tb927.4.4990

ACGTCAACGGGACTTTTAAAAAAATGGGGGAGAGGATGTGATGCTATGGGAGGAATCAAATCAAAGCAAGAGGGAGCAATGCAGTTCTCACTGGTTGTGACTGGGTTCTTGTCCACTTATTCGCTTATTTACTTTAACTATCGGAATTGAAGGAAAAGGGTAGGGAAATATATGCGACTGTCACTTTAAGATCCGTGCGTAGGAGTGAGCAACCCCTTGCCACAATTGTGGTTTGATACTCGGCAAGCGATTGGAAAAAGTTTGTTAGGTGTTGTTTTGTTCGTTCGATTGTTATTTTTTTTCTTTTTGTTTAAGAATGGAGGAGATGCCTGACTCACCCCGCCGCGGTTGCAAGGTGAGAGGGGCGTCAATTCGACGTGTGGAAGTCTGTTCTACATATTGATGCACCGCATACGACGGGGGGTATTTATTTTCTGGTTGTTGTTATTTTCAAGTAAAGGAATTTTAGCATTATATTACGTTTGGGGTGGTGAAACGGGTGGAGATTGGTCCACAGAGGATGTTTAGGCTCGTGAAGGGACATGAGGGGATTAATGGGGCGCATTTGTAGAGAATTTCAAATGCATTCGTTTGCGAGAAC

>Tb927.11.5050

GCGGATGGATTTGTTGTTGTTGTTATAAAGAAGAGGCGCTGAGAAAAGGAAAGGGAAACGAGGAGGAAGAGAGAGAGAGAGGCAACCGTTGGGCATAATGTGCTCGGCCAAATGAATATACGCGTATATATATATATATATATATATATAGTTTAAGCTCTACATGCGGTGGAAATGGAATGTTTCCGCGGGTGAAGGGAGAGGCACGGAGGGCGCAAGGAAAGAGGAGAGGGATATTCAAATAAAACGAGAGTATTCGACAAAGAAAAAAAAACATACTTACTACTTAGACGAAATTAAATAAAGGTGAGTAAAAGAAATACAAACATTGGGAGGGAAGAATGCGTGGAGGATGACACTGAGGATGTAGTTTCCACGTCACCGCACCATACTTGCAAGCAAGCAGAATACGCCAGAACAGTTCTTTGTTTGTAGTATATATATATTATCGTTTTCGACATGAGATTAAACGATGCATATTGCTTTTGTCATTATTGCCATGTTGTTCGTTTATTTATTTATTTATTTTTTTGCAGGGTCCCGTTGCATAAAGGGGTTAGGGGGGAGATTGTTTGTTTGGTTCGTCTTCTTAAGGAAAAGAATTGTACGGCCATGCAGTTACTTTTGTATATCTTTTTCTTCCTACGAATTCGTAGAAAATGTGACAGCGAGCGATTTTTTCCCCCTCCGTTTCAAGCGGA

>Tb927.5.3040 (could be too long)

AGCGCGGAGTGCAAACAAAAAAAAAACAGCAACACAAAGTGAGAGTTACGTAGTGTTTTTCTCTTTTTTTTTTTTCGGTTCCTTGTCACTGAAACTTATACATGTTACTTTCCCCCCTTTTTGACTTTAATGCCGGACGTCTTGTGGTCGAGTGCCATTTTTTTTTTCTTTTCGTGATTACATGACCATGTTTTGTAGCTTGACCCCTCTTTATCTATCTCGATGTGGATGTGTTGGACTCTATCAAAACTGGGTGTGGTGAAGATCACTGATTATGGAGTGTGAATGGCGATTTTACCTTGAAGGCTCAAACACGGTGCTCATTTCCATCGGTTGGGTTCCGCTGCCCAGTGGGCACATGTGCTGCTGCGCAGGTGGGTGGAGCCACGGGACACCGACGATATGATGATAAATGTTGATAAGAAGAAGAGGAGGGAGCGGGGCACAGTTGGAGGCACGACTTTTTTTGTTTTGCAGTTCGAAACTCGATCATTTTGGTAAAGAGCCCGCAGCTGAAAAGCTAAATGGGCTGAGTGACCGGTATCAAGTCTAGTTAATTATTACTGTTGCCACTTTTTGGGGAATAAATCACGCGATTTCGGTGCTACCGTCGTTGAGCGCCTAAGAGCAGGCGAGATGTGGGGAAAGGTGTGCACGGGGTTATTTTTTTCGAA

>Tb927.4.2410

AGGAAAGAAAGAAAAAGAAGGAGGAGGGGAAAAAAACGCAAGGATGGAAGGGGCTTTGCTTTCCTTTTTGCCTTCCGACTAAACCCTCTATCCTTGCGTTTTTTCTCCCCTTCCTTCTTTTTCTTTCTTCTCTCTGTATACTCGCTTTTCCCTCGTCACACACGCCTTACACATGCAGGATACGTCAGTCGCAGACGGTCAAACAGCACCGGCAAGATTAGAGAGTGTCTTCGGTGATTGACCGAAAGGACGAAAGAAAAAAACGCAAAAAAAATCCACATTAAAGAGACGGAAGAATGGAGAGAGAGAGAGAGAAAAGGGGCGAGTGAATATATCAATAAATGAAGTAGAGTGGGATTGAGAAATAATATTGCCTCGGCTGGTCAATGTGCATAATAAGGAGCGTAAATGAATGAAGAAAGGAATGGGAAACGGGATGTGTTAACTCCTAAAGGTGTAGATAAGAAAGGTGTGAAGTGTGTCTGTGTGTGTTTGTGTACGTGGGAAGGATGCACGAGAAAATATATGTGAATTGATTCTCCCCTCCTTTTCTCCCATTTGGCCGTAACGAAAGGAAACGCAAGACGAAACAAATAAAGGGTGGAGAACAAAAAAAACCGAAAGCAACATTAAAGACAACAATAACGATGGCGAAGTGTATCCTTCACAACTGTCTTCCATTTTAAATTCACCTATTTTTGTTTTATGTCAAGGTAAATTATAAACATGAGAGGGGAAAAAACAATGAGAACGTGCGTATTTTTATTGGTTTCTTTTACCGTTTCAAGTGTCAGCAAGCGGATGACACGACTCCCAAGTTGCAAAGTAGAGGGGAATGGTAATAAGCGCATTACACTTTACAGCGCGTGTAAGAAACGGGAAAGCAGAAGAGGAAGTCTTTTTTTTTTTTAAAGAAAAAACAAAACAGAAAACAACGGGAAGCAAAACCAAAAGTTATTGCGTCCGAATATGTGTTAATTAGTTGAATGGGAAACCCCGCTTTTGTGTTTGGCCGTATTTCAAAATGAAGAACGGCGTACCTCACACCATGAGGGTTTTTTTCTTCTTTTTTTGTTTAAATTTTTTAAAAAAAGGTTCCGCAAAACGAAGTTATTTACGTATTATTCAACGGGTAAAACGGTGAAGGGGAAATTAATTTTAAACAATAAAAAGATTTCATGAAGAAGTGAAAGGAAAGAGGGAAAAGTAGAAGAGGCGGTAAGATAAAGTCATTAGTTTGTGTGTGCGTGTTTTTTTTTTCCAATTTTTCGCTCTTGATTCATTTGACTTTTTCGCGCTTCACCGTGGCTTTTATCATTATTAATTGATTAACCTTCCTTTCGGAAAATAACATGTGCCCCCCCCCCCCTACGGGTCTTAACGGGACGGTCACCGAAAAAAAAAAAGAAGGGAGGAATGATGCCGAAAACGAAGGGGTATGGCGCGATTCCATAGACCCCAGTTTGCCGAGAGAAGTTTCACTTCGCATCATATTTCAGGTTGTGTGTTTTTTTTTTAATTTTCGCTTCGGATCGTCTTTTTTTTTTCCCCCTCCCCTTCCACAACTCTTTTTCACCTTCACACCTTTCATTCTCCCTTAAAGCTCTTGTCCTTATCAACATCTAGTGCTACACATTTGATGGTGTGCTCTAACCATACTCCATTTATTTATTTTTGATTTACTTCTTCATTTTCGTTTACGTGCTTAATTTCACTTTTTTTTCCTCATTCCATTCCATTCCATTTTTTGTTTTGTTTTTGATTGTTTGATACGTGATTTTGAGTTGTTGAGAAGAAGAAGTGATGGTGAGTTGAAGCGACGCCAGTGTGGTGGCATCCGCTTTTTTTCTACACGTTTGTGCCTTCGTCTAAAAAAAGAAAAATGTGTTTGATTATCGGTTCACTTATTCCAGCATTTGACATCTTATGTATGCCTTATTTGTTGTTGTTTTTTCTTTTATTATTTTCCTTTCGATTTGAAAACTGCTATTTTATTTTTCTATTTTATTTTTTTTATTTTGCATTTTCACTTGTACTGCTATTTCCCCTTCCCTTCCTCTAACTTGAGTGCTGGCCTTTCAGCTACCCCTGGGGGAAAAAAAAAGAAATAAACGGTGAAGTTTGGGAAGGGGGTGATGAGAACCAACCTTCAGTATATATATATATATATATCTTTTTTTCTATGTTAGTGTTTACTTTTTTATTTCTAGTTGATGTGCATGTCCTCTCTTTTATCTGTTTTTTTTTTATTTACCATAAAATCTCCAAAGTCAGCTGCTACCGTCAATCTTCCCTTATCTCGAGTAGTTTGAGGAAGAATAGAAATGCCCTTCTTAAAGCAATTGAGTGGGATTTCTGAAAAGTGAAATTAGCGTGAGACAGCTCCAGTAATTATTATTATTATTTTCTTCTTCAATCCAA

>Tb927.10.7700

AACAAAATCGGGTAATGGACCTGAGAAATGCAACATGGGGATAGAATTTTTTTAAAAAAAAAGGGAAAGACGGCAATCGTGCGAATATTTGGAAGGAGGGTTTGGCAGGGCTTACAAAACAAATGGGCTGGTCCCGTGCACATTCCGCACGTTCCGTAGCGTGGGGACACGAGCAGGTACACGTGCGGACAATAAGAGTTGAAAGAGAAAAAAAACATACATGCATAAGATGTACATGCACATAATAATAATAATAATAATTTGAGAAGGAGAAAGGAAAAAGAAGGTAGAAAAAAATGCGGGATGAAGGGTTTCTTTGGGTTGGGGGCGCGAGATCCTATTCTGCCTTTTATGCCCCTCCCTCTGGCACGCTTTACTTCCTACTTTTCAAGAACAGGTTATGTTTTCTCCTTGTTTGCGGGAGTATGCAAATATGTGGCAAGTTCGAGAGGACCAGTGTGACAGGGTGTGTCTAAGAGGAGGGTACATAAGCGTATTGTTAGTTTGGTTTTGTTCTATTATATTTGAGTTCGTTTGTTCGTTATTATTTTATTTTATTTTTTGACGTAAGTATTACTCGATGGCGTATAAACGTCACGCTGCGGTATATGGAGAGGCAGGGGGGACGCAATCAATGGGGCATTTCCTTCTGAAGGAGCCAACAGATTTATTCTCCGGGAACGTTACAATACAATATGGTGAGAGTTCCACTCCTTTATCATTTACTTTGGTGGCTTGCAAAGGACTTACGTGTTTTGTCATATTTCTTTTCCTTATTGATTCGGATGCTAAGTTAATTTGGTTTCCGCAGAGCCCGTGGAGCACAACGGGGGGGTCCCTTTTTTTTTTTCCGTTCTCCACCCCAACATCCCGTAAGCGGTGTTCGTGTGAGTGGTGTGCACCTCAGTGGCGCGTCAATTCGCAACTTGTGTTCGGGATGCGGACAGTGAGGCCGCAATTCGAACGTTCACGAACGCGCATGGGCACGTATACATATCTATTTATTTTTTTTTATTTATTTACTTGTTCTACTTATTTATTCATTGGTGCGACCGGGATGGGGCGGGCAATTTTCCGCTGTGTCACTCGTTCCTCTTTTTCTACTTTGGGTGTTTGTGACCTTAAGAATTAGGGGAGGTAGCACCAGTTCGTCCTTACTTTTATTCTGGGAGCCATTATATTGAGGTACAGTACCATTTATTTATTTGAGAAGCGGCTTCACCCCCTCCAAAGGACACGCGGCTACATATGTATATTGCAATTATCGTTTATTTCAATTTATTATTACTATTATTATTTGTCAGATTGCGGTCCCGTTCCCCCCCCCCCCCCCCAAACCCCGATCGCTTCCTCTTCTTTTTTTTTTTCCACCCCCGGCATTCACAGCTGAGGCTTCGGAAATGAGGCACAAGTGTTCTTATATTCCCACACCTGTCCAATCGTGAGTCAACCTGTGGGGTGCGTCTGTTTTAACGCAGGCGCGCTTAACGTACGACACACA

>Tb927.9.12680

AGAGAATGAAAGAAACAAAAGAAAAAGAATGCTGGAACACTATCGCATATACATATATATATACCTATATGTATGTGTGTGTGTGATCCTGCTGTTAAGTAGTAGTACTGATCTCTTTTCAACTCTCACTACCTAGATGTTTATTTGGGTTTGTTGGCTCACTCTCTCTCTCTCTCTCATCTAGTTAGCTCCTTTTATGTTTAATCATGTTGATTTGCTAATTTATTTGGCGGAAGGTTTTTATTTTATTTTTTTGAGGGAGGGGGGAGGGTGGTGAGGGGAGAGCGTGACGAATGTGAAAGGATGGCGGAGGTTAACGATGTGACGGAACAGTGTTAGGACAGACTAGAAAATAAAAAAGGAAGAGGAAAAACGTGCTTGAGAAGAAAACCTCAAGACAATTATTACACGTGTGTTGACCTGAACAACTTTGCTTTGGTTCAGTGGGTAGTCCTTTCCTTCATCAGGTAACGAAGTAACCCCAAGGGGTAAGGGAGGGGGGAAAGGAAACCTTCGTAAAGTGGTTGTAGCTGTTTTTTTTTTAAATATCATCGTGCTGCTATATATATATATATAAGAGAACCATCCTGCACTGGATGTCTGCCGATCGCTTCGGTCAGATAAGCGGCTACAAGGGCGAGTAGCGAAAAGTAGTGGTGTTTCAATGAGTAGGTTTTCGTAGTATATTACTATTAGCGTTTAGAAAAATTTTGTTGACACGGACGAAGGATATTTAGTTCATTGTACATAACCTATGACTTTCCGGGAGTATTCAAAAA

RBP10 extended 3'-UTR

ATGGCACAGAGGGTAACGAAGTAGGAATTTTTGCCGCCAGCTGAGGTCGTTTACCTTGGGTTGGCTGTCATGGAGATAGGGAAGAGAGAAGCAACATCGCGTGCAAGGAAAAACGAACAGAAGAATCGATCCCTCCCCCCTCCATGAGTCCTTCTTCGTTTATAATTTCGGCTGTTGTTTTTGCTGTCACCTTTTTTTTTCTTCCTTCTCGTTCGCCACACCCTCTCCTCAACCTCCTTTACCTCCATGGATCTCTCGGCAGCAGTCCCCCTCCCCTCATTCCCCTCATGTTTTGGTGGTCGCCTTATTGGCACCTCTTTTCGTCATTTTTTCCCCCCACTTGCTGCAGTTCAAGGCGTATTTGTGGGGAGAGTAAAGAACATAAGTGAAATCTGAAAACAATAGAAAAGAGAGATGAATTAATTAATGAATGAATGGGAGAAATAAGGAGGAGAGGTGGTGGAGAGCTGGGGAACTGTAATGAAGAAGAAAAAAAAGAAGAACGAGGGAAAGGTAGATTCAGTGGGGGAGACAAAACAGTGAAGAAGAATCTGGCGGAAAGCAACAATAAGTGTGAAAATGAGAACGAAGGTGTGAATGATTCTTCAGTTTAAGAAACCACACGTGAAGAAGGACAGAATATATAAATAAATATATCTATTTATATGTTGATGTCTCAGAAGAAAAAAAGCAAAGGAGGGGAGATAGAAGAGGTTTAAAGGGAGGAAGGAAGACAGGCCCTGAAACCAATAAAACAAAATAAATAAATAAAGAAACGCGAAATCAAAACTGAAAATGCGAAAAAAAAAACGAAGAACAAGAAGGGTGCAATGAAGGAATGAACTCCACAGAGAGAATACCCGTTCAAACATTCTTTTCTTCGTTTTTCCCCTCCTCCTCTCCCCGACGCAACCGCCCCCCTTTTTTTTCTATAATTGGCTCTTCTGACCCACACCCCCTCACTTTCCTTCTCAAAGAACGGCATCTTGTTTTCCCACCCTTTATTATTGTTAATTTCTTTTTTCGCTTTACTATTATTATTATTATTTACCACCCCATCACCGTTACTTTTAGTTGTTTTTTTTTTTTTTATGCCTCTCTCCTTTCATTTTTTTGCGCTTTTGCTCCTCCTTATTGGTTATGTAGAGCGATTTATATAAATCTAATATATATATATATATATATATTTGTATTCATATATTTTATGTCTTATATACATAACTACTTTCGCGCAGAAAAGGGAAAGAGGAAAGGAGAAGAAGGGGGAAAATAGAAAGCCCATGCACATATATATATATATATATTTATTTATTTGTTTATTTATTTATATATATATATATATGTATTCTTTGTACCTCCCTTTCGTTTTTACTTTGTTTTTTGTTTTACCTTTTTTTTGTTTCTACTTTGTTTCTTGTTTTGTTTACTGTTAGTCACTCCTTACTATTTTTACCGCCTCTATTATTATCACTATTATTATTATTATTATTACTATTATTATTCCTATTATTATTATTATTATTCCTATTATTATTATTATTATTATTCCTATTATTATTATTATTCCTATTACTACTGCTATTATTATTGTTATTTCTGGGGCTACTGCAGTGCTTTCCCTTTCTTTTTCTTTGGGTGTTCGCGTGGTTGAGATTGTGATGTGACTTCTGTTTGCTGACGATTGTTTGTTTCTATTTGATTGTGCCGGTGTCTGTTTTCCCCCACCCCCGTGATGTCACGAAGAAAAACAAAAATTAAATTGAAAAGTTTTTACTTCTCCTCGTCCAAAGCAATTGCTCTCCTCCTTCCTTTTCTCCACACGCGCGCACGTACGTAGTGAACTAATCAAAGAGTGAAGAAATAAAAATAAAAACACGCGTCGTGTTGTGGATCCCTTTTTCGGATCCAATTTGCGCTATTGCTTTTTTTTGTTAGTATTATTGTTTTGTGCTTTTTTTTTTCTTTTTCTGTGTGTGTAATATGATTTTGTCGCTTTTCATTCAGCGGAAGCAGACAGTAAAGTTATGGGCTCATATGCTTGCATGTGCATTCCATCCGATATTGCGCGGGAGTTGTTGTTTTGTTTTGTTTTTCACACCTTCGTCCCTTTTTTTTTTCTTTTTGGTTTTTATTGTTTGTATATATTTTCATATTTATGTACATATATTAGGAGCAAATGCATGCGTGTTTGTGCTTTAACGCCCATGTGTGCGCCCCTTGGCTTGCTTTCCCACAAATACCCTTTCGAAACGCCTTACGGAAGCGGGGACAATATAATTAAGAAGAAAGGGAGATGGGGGGGAAAAAAAAAAGAGAAAGAGAAAGAGAAACACCGGCTCGTAGACATGAGGAGAGGAATCAAAAAAAAGACAATGAAATGAAATAAAGTTAAGTGAAGTGAATTGAAACGAGGTAAGAATTTAAGAAGTTAAGAAAGCTGGTGACCCGCCGTCGCTTTTCTACTTCATTCCACTCTTTTTTTTTCTTCTTTTCTTTTTTTTCCCATCCTTTTCCTTTTTTTTTGCTAGTTTTTGATCCGCTTTGCCTTTGCATCTTATCTTTCCATTGCTCCCACTTTTTTTTTATTGTTTCTTTTTTTTTCCCCCTGTTGTTGTCACCATTATTATGTCATGTTATGTCATCGGTACTACGACAGTTGCATTATGATAGTTATTATTTTTGTTCTTTCTTTTTTGTTCTCGTTTTGTGACGTTGATGTTTGGTTTCATTTTTATTTTTGCGGTTACCTTTTTGTTTTTATTCGCTTTTTTTTTCCTTTTTGGTTTATTTATTTGTACTTGGTGAAAAGGAAAAAAAACAAAAAATATATATATATATAGATACAGAAGCAGAGGAAGAGAGAAAGAGAGAATGAATGAATGAATGAATACGATGAAGAAGATATTGGAATAGAGGTGGAAAGGGAGGGGGAAAAAAAAAAAAGAGGAAAGGGTGACGCGGTTGAGTTGACGGTAAACAAAACAGAAACGATAAGAAAAATAATGCACAAAATTCTTCCCCTTCTGTTCCTGTTTTTCGTTTCTTTGTTTGTTTTGTTTTGTTTCGTCTTCTGTTTGGTACCGCATCACCCGTTACCATGGCCCTCAATATGTCTTTATTATCATTATTATTATTATTATTATTATTACTATCCGTTACTGTTATCCTTCATTGGCATGATGTTTTTCCGCCGATATTTCACATCTTTTCATTGACTCTTATTATTTTTCACTTCATCCATGCCGACTCTGCAGTACTTGTAGAAATTTCATTGAAACAGTATTTTGACGAAAAGGAAGCAAGAAAGTTAAAAATTAATGTAATGTAATGCAATGCAATGTAATGTAATGTAATAAAAAAGTAATAACAATGACAAACCTAATAACTATCATAATTATAAGAGAAATTGAAAGAAAGGACAAGGTAGGGTGGAAAAGGAAGCAAAAGAGGGAGAAGGGGGTGAAAAAAAAAATTAATTGTTTAAGGCTTGAGAAGGGAAAACGGCACTGTAGTAGAGAATGAGAAGGAATAAAAATAAGTGCGTGAGTAAATGGATGAATCAACAACTAAGTGAATGAGCATTTTCATGTACACAAAAAAAAAAATGAAAAGACGTGTTTGACTCACAAAGGGGAGGAAGAATAGCATGAAAGGTAAATATTTGTGTCAGAATAAAAAAGAAACTAAGAATAAAAAAGAAATAATAACAATAATAAAGTGAGGCAGAAAATGATGTTTCCACACCATTGGGATTGTTAAATGTTGCGATTTGGAGAGGAGGGAACGCGTGTTGACTGACGTGGTGATGAAAATTTTTTTGTTTTGTTTTGTTTTGTTTTGTTTTGTTTTGTTTGAGGGTCACACGTGTTCCACAACTCCTCCTTTTGTTTTATTTTGTTTCCGCCCTCCCTCGTTCCCCCTTTCTGCGTTTCCCCTTTTTTTTTCTTTTTTTTTCTTTTTTTTTTTCTGTTTTCTGTTTTCTGTTTTGTTTTGTTTTTTTTTTTTGCATCCCATCGATTTGAGAGTTATAAAAGACGAGGAAAAGCGGAATGTTTCTCGTGCGACGAGAGCTGGACATGTAAAACACAAAGGGAAATTAAGGAAGTAAATAAAAGTAAAAAAAAAAGAAAAGAAGAAAAACGAAAAAGAAAAAAGGAAAAAGGAAAAAAAAAAGCACCCGAGTGGGTAGAGGATATGCTGGCAATAGTGTGGTCAGTTTATTTTAAAGAGGGAATATGTGGAGGGAAGGGAAATTTTTTGAATATAAATATTTACTCCCACAACATGCCGGAAATATATATTAATATTTGAAAAAAAAAAGAGAGAGAGAGAGAGAAAGAAAGAGATGGATTGGAAGGTAAGGTAAGGTAAAGAAGTAAAGTGAATGAAGCGCGTTGATAATAAATTAAGAGGAAATAAAAAATGTGAAGGATTTGAAGAAGTTTTGTTGGTGCTACTTTCAAGTGAAACTAAACAAAATATGTGAAGGACGTAATCAATATTTATTTGTTTGTTTGATGACGCTTCTATCCATCTGTCTTCCTTTTGCTTGTAATTTCACTTTTGTATTTTCCCTCCTTCCCCTCATCGTTTGTTTTTTCATACTTTTTTTACTTTTTTTTTGTGTTTGTATGGTTGGTTGTTCCGCGTATTGCGTTTAAAAAAAAAAAGTATTGAATAGCATTGTCGTTCGTGGACCTGGCGCCCTATTTTTTTTTGTTTTTTTTTGTTTTGTTTTACTTTATTTTCACCTTGGATATGGGCATATGATACAAATAAAATAATAATTAAAAAAGGGGAAGTGAGGCTTAGTGAACAAACGAAAAGGAAGTCAAATGAAATAATAATTTCTTTATTAAAGTGGTAAAAGAACAAGAATGACGTTAACGATGAAATGAGCTCCACATATGTCAGGTACTTGAAATCACGTAAAAGATAAAGAATGATTTAAAGGAAGATTAGAGAATATGAGGAGTTAGAAAAGAGCAAGTAAGTAAGGGTAAGTATATTCTAAGGGAAATGCTACTTGTACTTATAATTAATGACAATAATAGGGAGGGCAAACCAAATATATATAAATATAAATAAAGATATATATATATATATATATACATATATTTGATTAAAGGAGCGAGAGAAAAGGGGGGGCAGAAACGAACAAAATAAAGTTAGTGAAGGAAAAAAGAACTGAATAATAAGGTGCTTTCCTTACCGCATTTGTGAGCCACCCCTCATCCCCACATGTACGAGAGCATTTTTCATCGTTTTCGTTCCTCAACAAACTTTTGTGCGATGAGGTGGTGGATGAGGAGTGGCAGTGCCAAAAGTAAACAAATGGATCTATATTTCCATTTTTGTTACTTATGAATGTTCACACCTTATCTTGTTTTTTTTTAAGTTTTCAGCCTTTTTTTTTCGTTTTTTTCTTTTAAATTGTGTGTGTGAGCTCTTTTTTTTTTTTACGCTTCCTCCCACCCCCATTGCGGGATCCGCTTTGCAAAGAGGAAATATTTTGAGGTATGTGGAGGGCTCCCGTGTCCTCATATATGTAAGCAGCGCTTTTTTTTCGTTATGCTAATTTGAAAAGGAATTTTCATGTATGTACATAGTTAATATAAAGAAATATATTTATATATTTATAATATACAATATATACAAGAAATATACATACATGTTTCTGTGACCGTCTATCTAACTGTCTGACCATCTAATTGACTGACCGACTGGCTGTTTGTTTGTTGTTTTTTTTTTTTCGTTTGCGTGTAAACATAGATACATCATAATGGAGTGGCCTCTCATGTGTATGTGCGGGTGCATCCATGTGTGCATGTCTCGTGTTTCCGTTTATTGTCATTATTATTGTTTGTATTCCTTGTTTCGCACCTTGTGTGTGTTTGCTTGTTCGTGTTCGCCTACTTTTTTTTTTGCTTTTTTTTTGTTTTTGTTTTTCCTCCTTCCCTTTTATGTGAACCATGGCTATCATTTCTTATTATTACTGACATTATAGCTATTGTTATTGTTTACTGTTGCTACTGACATTCCTCCCTGGGTGAATTTCAATTATTTGTGATCTCCTTCTCGTGTTTTTCCCCCATCTTCTTCAGTCTTTCCTTTTTCTTTTTTTTTGTCTGCTTCGTGTTGACGTTAGTTTTCTGTTTTTGATGTGCGTGCAGTTTTTGTTGTTTCAACTAAATTAATTTTTCTGTTTTTTCCTTGAGGTCCTGCGCGCGGTGGCGAGAAAAAACAAAAGTAAATGTCAAATAATGGCTGGTTTTTCGACAAGGTTAACAGAATTCAAAAGGAATAAACAGTTTTTAAAAGAGAAAAAAACGGTGAAATATATATATATATATATATATTTAAAGCTGACAGATATACAAATACGTGTGTGATCCTGTGTGATTGTGTGTTTGTGCTCACATTAGAAAGTAAATAAACAAATTTAATGTGCTTTTCTGCCGTCGAAACTTCCCTCCGCTCCCGTGTTTTGTCTTTGACGTGATATGCAGTGTGCGGATGAGGTTGTTACCACTTTTCTTTTTGTTGTTGTTGTCGTTTCTTCGCTTTTTTTTGGGGGGGGGTTTCAGTTTTTGGCTTTTGGCTTTATTATTGCCGCTTAATGGATGGAGAAGGGAGAGGGAGGCGATAGAAGCAAAGAAGAGTTCTGTTTGTTTGCTCGCTGCGGACTTCTGTTCACTTTGATTTAATATCCGGAAAGGAGCAAAAAAAGAAAAGTATAATTCATTTGACATTTCTTGAGCAAGTCGTACAATGAGTGAGCATACAAATACTTGCAGCATCCGTTCTCTTCCCTCCTTAATCTTTCTCAGACTCTTCCCCTGTCATCTACTTTATTGTTTATTGTACTGTTCCCTTTCTATTTTGTTTCTGTTTTCGTTTGTGTTTGTGTTAAATCCGATAATCATCAACAGCATCTAACCGGTATTTTGTTTTATCCGTGGTAACGAAAAGAAAAAGAAAAAAGAGAGACAATAATATATCTCTGTTAATGCCCACGTTAAACTATATATTTTTGTAGGAAGGAGAGAAGAGGGTATGGACCTGCTAAGGGAATTACATATTTGTTCCCTTCTGTATGAACCACACTTACCTACGGTTTTTGTTGTTTTTTTTTTGTCTTTGTTCCTCTTGTTTTGGTTTGGTTTTTTTTTTTGGTTTCGTTTCGTTTCTATTTCCTTTTTTTCTATTTGTTTTACGTCTTTGATTGTCTCGTTGCGCGGGAAGAGGAATACGAGTAAGGGGAGAGAACAGAAGAAAAGAAAAGGAGACAAGAAGAAAAAGGAAATTTTGTTTATTCCTTATGCTTTCAAAACTCTTTTCCCGTATCAGTGTGCGTGTACGTGTGTGACTGCCTCGATACAACTGGTTTCACTTTCGTGCCCTATCATCTGCTCCTTCTCTTTACCTCTTTTTTTTTCTTTTTTCTGTTCCTTTCATTCTGCTCTCACGTTCAACAACCACACAGTGAATTTTATTATTTATTAACTTTATTATTACGACAATATATATATATACGGTCGCTACTGTTAGCTTACCGTCAATATCAGTAACCATGTCACGCGGTTCACAAATGTTAAATTTAATAACAGTAATAAAACGAATATACAATTGAATAGACGTGCAGAAGTTTCGAAGTTAAGTCGAAAGAGCAAAAAAGTTAAAGAAAAAAATATGTAGAGGGTGGAGCCAACAATAACGTATGGGAGGAATGAATGTGCTTTATGTCCGTGACGGAGGCGTAAAATGATGATGAACTGAAGTTATGGAAACTAACAAAATAAGGGAAAGGACATATATATATATATATATATGCGTGCATATATTGTGCCGGAAGGAAGCGGAAATATACACTGAAGCGTACCGGTCAAAAATTGAAAGTGCGGAACCCCGCGTCTCCGCGTAGTGTCACCTGTTATCCGCGCATTTTCTTAGTGTCTCTGCTTTTAGCGTTGCACATCACCTTTGCAACAACCGAGTTATCGCCGTTTCACACCTGTGGCACAGATTTTCGTGGCTCCTTCATGACACTTATGCTCACTTTACATCTCACCTGCCGTTTTTCATCAAAAGTAAAGACCTTCCGTAGGGATGTTGAGGAACGAAATGACGAAGTATTAAGTCTGTCGTCACTGCGGACAGCTTCTGGCGTGGCACCAAAACACTCCTACTGAAAGAGCTCAGTGACGCCGCGCTCGCCATTTGTGAGGCAGAGGGCCACAACGTCCAGTGCCTCGTGTACGATCGGTCCAAACGGGAAAATGTTCCCATGACAGAGGGCCGCGATGCGTGGTACAGCGACGTTGTAGAGACCCTCACGGCAAGTGAGCTCGCGGATTGCCCCGTTGTATGGATTGGGGCGGAGGATCCCCTTTTCCTCTTGTACACCTCTGGCAGCACTGGTAAACCCAAGGCAATCTTGCATACGCTGGGAGGTTACAATTCACTTTCTGTAATATGGTGCGCACACAGAATGAACTGCAGTTTCACCGCAGGGGGGCTGCTGTATTTTCCTGGTCTTGAACCAAATTTGCGCTCTCCACGGCGGAGACACTAGACACACCCAACCCGGTCTGCGTTCGTCTTTGACCTCCCGTTTCAAGGGTGGCACGGCATTGATTATTTACCTCTGCCATTTGGAA

>Tb927.10.11630

AATGATACAAATGCGTCTGTGAGGGCTGCTGGATTATAAGCGAATCGTGGTGAGGAAAGGTGGAGATTGAGGAAAAGCATATATTATAGGTGAACGGGCTCGTGCACGTGCTTGCAGTGCCAGTCAACTCCGGTATTACGTGGCGAGAACACGGGTGTGGTATTGATGCACTACCTCGTAGTGCCTAGGATGCTATTTCACAATCTATTCAGTTCTTTTTTCCCTCTTGGGTCTTCCCTTATCCTTCTTAAAAGGTATTGTGGCATTGCTTTAAGGTTTATTTACCTTCGCTGCCATTTCCATTTTGCCCGCTGCGCTCGATAACGTTCGATTGTTATGATCACACAACTTTTTTGGGACCGTATTTTATACAGATCTGATTCTGCATGGTTTGGCCGCTGCAGCTTTTGATCCCCCATTAGTCCTTTTGTTTTTGTTTCCGTTCCATTATTTTATTTGACTTTGGGTGGACTTTCCTCCCTTCCCCTCTCTTATTGATACGCTCTCCCGTTCGTTCCACATATGTTTGTGTTACTTTTAGCCGTGGTGATGACATGTATTTGTGCTGTGACTAATTATTTCCGGAAAGATATTCATGTGATGGATTAGTAGATAAATTTAAATCTTCACCTGGTGAGTACCCCCTTTTTTTGTTTATTTCTTTTGATCGTGTACTTCCCTCCCCCATACACAATACGCTCTTTTTTTTTTTTTACTTTTGTTGGTTTGTTCGTGGCATGAGTTTTATATCGATTTATCAGAGAATCAACCAATAACAAATACTTGTATGTATATGTTTACATGTATGTGCTTCCCTTTTATATGCATTTGTTTTACTTTGGAGAAGCTGGGACTCGCTTTCCGCTTCTTTTCTTTTAAAAGAAAAAAAAAGTAATTTCCTTGTACAGATGTTTTATTTTTGTCATGTTTGTTTTGTTTATTGTTATTTATTTTATTTTACAGTTTAGCGTGATTTCGTTTTTCTGACACGTGAAGGCCACCAACCCCTCCAAGAAGAAAAGGCACAGAAGATCAGAGAAACCAAAAAGAGAAAAAGGAAATAATAATAATAAAACAAAATAAAGGTGAATGTAAAGCGGAAAGATAATTTTTGGGGAGTTAATGTGAGGAACTGAAGTGAGCAGACGCGGGACCTATTTATTCGTTCCTTTCATTATTATTTTCGTTTCTTACATTTCTGCGGTACTTCCCTTCATGTTTCACGTCCGCTCGTCTCCATTCCCTTATTTATTTATTTCTTTTTGGTCTTTTCACTCCCTTTCCCTTTTCTATTACAAAAACAAATAACAAAAAACAATAAAAAAATTATGTTCATCGCCGCGTCGTAGTGTTGTTTACCATAACTTCCCTCTCCTTCTCCCCCTCCAAAAATATGAATGAATTACCTCTTTTTTTTTTTCCTGACGTCATGATGGGCCTTTCCATTTGTTACTTCCATTTTTATTTTATATATTTCTGTTGTTTACTTCATTTCACCATACTTATTTTCTACATTTACATGTTTTATTTTCGTGTATTTATGGTATATTCCACATTGCTATCTTATTACTTCATTTCTAACTGTGTTATTATTATTATTATTAAAGTTGTTGTTATTATTATTATTATTATTCAATGTATGTATCAAAGAAATAAATTATCAAACCAATAAGTGAATAGTTTTAATAGCTACAGCTTTGATACGCATTTTCGTGTGTCTATGAAAGGAAGCGAAGCAACATAAAGATAAAAAGCAAAAGCAAAAGCAAAAGCAAAAGCAAAAGAAAAAAAAACAACAACAACAACAACACATATATGTTTCTTTAATTCGTTTACATCTTCTTTTTTACGTGCGCATTCCATCACCTTTCATTCATTCAACTTTTTTTTCCATTTATTTACTTATTTTTATTTTATTTATTTTATTTGGTCACCCTCTCAGTCACTTGCTTTTTATTTCTATCATCATTATTTACATGTATGTGCATGTGAAGATGTGCTGTTCACAAATGTGCGTGTGTGTTTAAATAAATATATAAATATATAAATGTATGTATTTGTATGTGTTGTGATGAATTTGAAATGTTGCGCCTTCGCAATTTAGTGTGGGGTCACTGCTGAGGAGTTGGGGAATTCTGTATGCAATTTGTGTTTTTGTTTGTTTAATTTATATTTTTACGCCTCTAATTTTATTTAAGTAATGATATAAGGGATATGTTTAAAAATATCTTTAGAAAACGCCAAACGAATGAATAAATGAATGAATGAGATAATTAAAATGTACGTACAATAGTGTATTTAATGAGCGAAGGCACGTTACTGTGTGGCATTAAGATGGCACGTAAAATCAAAACAAACAAACAAACAAATAATAATAATGATATGTATATATGTATTTGTATATTTAAAAGGGGTAAATGCGATTCGTGTGAGGGGTGGTGGTGGGAAATGTTTGGTAACGACAAACAAAAAGAGGGATACCAAGAATAGGTAAGCAAATAAATAAATAAATTAAAATAATAAAGGTAACTCTCTGGGGCCCGTGTCGGCAACCCAGGAAATAAAAAGTTAGTTTTATTCTTGTTATTTTATTTTCTCTTTTTATGCACTGTCACCGCTTAGCAGAGGCAAGCCCTTGTGCCGTTGCCCATTATTATTATTATTATTATTATTATATTTGCTGCTGACGTGCCGTTGTTTTTTTTTCTTCTTCTTTTTTTAATATATATTTGTTTATTTATTTATTTATTTATTTGTGTGTATGCGTATGTATATATATGTGACGTTTTGGCTTCGTTGTAACGCTTCGACTTTTTCGTGTAAGATTGAGAGATTTATGTATTCATTTTTTTTTGTTTTGAGTTTGTGCACTTCTTAATCCGTCATAGCTTTATGAGTTATTGATTTAGTTGTTTTCTTTTCTTTTTTTTTCTTTTCCTTCCTTCTCCTGCTTCATGCATGTAGGATATGAGGGAATAAATGAAGAAGATAGATGAAATAAAAGTGAAGAAGTGAAAGGGAGGTGGAGGGGGAGGGGAAAAAAAAATAAAAATGAAATAAATACAAACAAACAAACATAAAAAGAAGAAAAAAAGAGAAAGAGAGAAGAATATATGAGACAATTAAAAGGCCGCCACCACCATTAGAAAATATAAAAATAAAAATAAAATAAAATAAACACGCAGGCGCGCGACCAGAAAAAAAAAAAAAGAGGAAACAGAGGGAGAACAGGCGAATAGGAGAATAATGACGGCACGGATGCGGATTGCCGAGGTTTTCTCAATCCATCAGTGGATGTTTTTTTTTCTTTTCCTTTTTTTTTCTTTAAGTTTTATTTCCTTTTTATGCGTTTCCACACCGACATGTGTTCGGTCTTACGTGCATAACAGATGTGCGTTACTGCGCAAATACATTCAGACATTTTCGTTTCCTTTAGTTAAGAGAGGATCAAAAAAGGAAAAAAAAAGAGAAAAAGAGAAGAATTTAGAATAATAATTAAAAAAAAATCATAGCGGTACTTGAACCTTATATTCTTTTTCTTTCTTTTCTTTCTTTTTTTTTTTTTTGGAGGAAGTGACACAAAGAGTAACGGTTTTTTTTTTTGTGGGGGGATTTCCTCCATTCTCTTGAATGATGGTGTTAAGATGAACTGCCATGTCTACACCTATACAAGTATTAATTTTTTTCTTTTTTTTCTTTTTAAAATTAGTACTATTACCGTTACTATTGTTATTACCGGATTTCATTATTATTATTATGATATATTTATTATTATTATTATTTTTTATGGGTTGTGGAGACTCGAATTTTGCGCTTATTACTACTCGTCAAAACGGGTTTCTTCCACAGTTTTTATCCCAAAACGATGATTTTTTTTTAAAAAAAAGATGAGGAAGAGAAAAGGAGAGAAAGAAAATAATAAAGTTTTATTTTTATTTTTATTTTTATTTTTAAAAAAAATGATAACGGAACTAAAGAGAAGGAAACACACACGTACTCATATTCATATATTCATACATACACACACACACACACATGAAAAGGGGAAAAGGCGGGGCCTCTTTTTTTTTTTTCTTTCTCTCCTTTTCTTTGGTGGTTCTTGTTTCAGTGAGCTGTTGGAAGTCTTTTTTTTTTTTCTTTGCAGGAATTTATTTGTGTCCATGGTTTTGTGCTTTAGGGAAACCCGCCACGTCACTTTTATTTCAACTATTATTTATATTCATTCTCTCCTTGTGTTGTCTTTTGCCTTTCATTAGAGCTAAATATATATATTTTTTTTTTAGAAAAAGAGAAAAGAAAAGAAAAGGAAAACAAAAGAAGAAAATACATTTGTCACCTATTTTTGTCTAGTGGCTATTGGCATTATTTACAGCAGATATCAGCTGGCACCTGACGGGTGAGTTTTGTTTGGGTGACTGAAAACCAAACAAAAGAATTAAGTTAGGGGACGTGACGGTTGATTTTCTTTTCTTTTCTTTTTTTAAAAAGTTTTCTCCCTATTTTGTTAGTGAGATTTTCATTACCAACTACTGTACTTACATTCAGGAAACCCGTCGGTGTGTTTGTGTTGTAGCGAAAATATTTCGTGCGTTACTGAATTCACCTCCTTCATATATTTCCATACAACAGTGCTGAGCATATATATATATATATATATACGCATACGCACCCGCATATACATTTATATACATATATATATATATATATATATATATATATTAATATTAATATTAATATATATATTTATTTAATTAAATCCCTGTTTGGTGAGGGATGCATGACCAACGAAAGGTACGACCACACAACATCGGGGAGAAGGCGCGGGAGCAACAACTGGAAAAAATACGGCAATTTACAACCTTGTATGACTCATTACTTGAGCGGCGAAGCAGGCGTGTATATGAGGAATCGATTCTTCCACAACTAGCGGAGTTGTTGACTAAAAATGCAGAAGCATACACGATGTTTAATTATAGGCGTGAAGTATTATTGGATTTGTGGAGGAAGATGCCTGAAGCTGCTGCTTGTGAGACCGTTGCACCACCTGAAGCGGTAAAAGGCGAGGAGAAGCGGCAACAATCGGTAGTAAAAACACAACTGGATTGGTTAAGCGAGGAACTGAAACTCAGTTCGAGTATAATTCAGAGCGATTACAAAGTGTATGCGGCATTTGTGCACAGACGCTGGGTATTTATGCAACTGCGCCGCCTCGCAGAATCCGCACTGGGAAACGTCGGGAAGCGTTCAAAACCTGCCGCCCCTGCGGGATGTCAGTTGGGGGAATGTGCGGCTGCGGGTGAATTCGATCTTCCGGAGGAGGTTCTTTTTTGGGCGAAAGCGCTGCTTAAGGAAAAACGGCAAGGTGACGCGTTACTCGCAATGGATGAGCGGAATTTTCATGCATGGGAGTTCCGGCGGTGGGTAATGTATCAATTGGGACAAATGGAAGATCTGTTCGTACAGAGTTCCATTCAATTCGGACCTGCAGTTGTGGGGATTAAAGAAAGGGAGTTCGCATCTTACATGAATGGCAGTGCCAAGTCTGATCGACCGCGTGATTTGTTCTTTACCCCAACAGAAGTAAAAGAATTGAACTTTACGTCGGCAGCTGTTCGGCGAAACTTTTCCAATTACAGCGCCTGGCACCAGCGAGGATTTATTATGCAGGGTGCACTTCGGCGTTTGCAACAGCGTCAGTGGCGGGAAGAGGAGGCAGACAACAATCTCCGTGACGGGATGCTTTCGCAGGCTTGGGGGCAATTGGAGGAGGATTTGACACTTCTCACTACCGCCATTTATTGTGACCCATTGGATCAATCAGCGTGGTATTACGCGAAATTTCTCATACACGCCTCTAAGCAGTTGACAGCGTTGCCTTTTGCTTCAACAGCAGTTCCTATTGATATCACAGCGAAACTGGATGAGGTTTGTCTGGATCTTGTAGGGGAGGAGCGGCGTTTAGGGGAGGATATGGAAACGTATTGGCCTTATTTACATCTTGCCACGTCTCTACTCACAAGTATCAAAAAAAGCAACGACGGTAACAGCAGTGATGTGATTAGCGATCGGCAGTCGGCGTTAAAACTGGCACGTGAAGTATGGGCTGCCCTACAACCACAAGGAAGGCACGTTAATGGTGATGATGATTGCATTAAATGCTTGCAAGAATTATGTGCTCACTTAACTACGGCTGACCCTTTACGTGCGGGCATGTACAAATACTTCCTTTCTGAGGTTACCACAGCGTAAGGGATGTGATGCAACTTCAACTTGCATTCGGCTCCTTACTTCTTGTCACGCATTCCGTAAGGTTTAGCGAACCAGGAATAGGGCTGGTATAAGAGTAAAGTGAAATTGTTTTTTTTTTTCCTTGTTTGCATAGTGTTATCAAATCATATCATGAAAAGAAACAAAAGGAAAGCAAAGAAAAAGGATGAAGTATTTGTATGTGACTGTGCACGTGTAGATATATGGAAAGTCTTCAGAGATGTTGGGTGAAGCGATTTAAAATATGTTTCTCTTCATACGAGTTCACAGTTCTGCGCGTTTAAAAACATACATTCAATCCTTTTTTTCTTTGCCACTACATCTGCTTATCGCTACTCGCTTTACTGTAGCTTTAA

>Tb927.9.10280

ATGTGGAAAAAAAAAAAGTAAAAAAAAAAGAAAGAAAGAAGAAGAAGAAGGAAAAAAAAACATTTCTTCACATGAAGGCCCTTGGCAAAGAGAAAGAGAAAGAGACAATGCAAAAAAAAAAAGGCAAAATGGTTCATGCTTCAAGTACATATGATGATGTGAAAGTCAGTTCATTCTTTTTTTACTTTTTTTTTTCGTTACTTCACTATTTTCACTTTTTTTGTTTTGTTTTGTTCCATTTATGAAGATGGTATAGTAAAAAGGAGAGAATCACAATGATAATCATAGCCGTTTGAGAGGTGTGGCATGTTAGAAATAAAAAAAGAAAAAAGGGAGGAGGAAAAGTAAAAAAAAAAGGAGTTAAATGGAAAGAAGAGCGAAAAAAAAAATTGACGAACTGAGACGAGCAGTGTGAGAGAATTTAAAAAAAAAAGAAACGGGAAAGGAAAAAATGAAGGAAAATTATGTGAATATATTTTAAAATACTGTGGCGTGCCCCTGTTGTTAATTTTTTTTCCTTGCTGGTTTTTTAGTGGCATTTTTTTGATAGTGCGAAGTAATGGGCTCACCATCGAACGATGATCTATTCAGGTGGTAAAAAAAAAAAACAACAACAACAACAAGCAAACAGGATTAGTTTAAAGGGGAAAATATATAAAGGAAAATGTGCAGTAATGCCACAAATAGGGAAGGATTAAAAACAAATGTATAGAAGAAGAAGAAAGGAGGGGAAAAAATTAGAAACTGAGAGGAAATAAAATTTTTTAAAAAAAACGCGAGAAAGCTAAAAGCAGATGACCCTTTTTTTTAAAAAAAAGGAGGGGCAAAGGTACTATGAAATAAAATACAGACTGGAAAAATAGCGAGTGAAGCGAAGTGGGGGTAGAGTAAATTCAGCAGCATAATTATACGCAAAATAAACATATATTCCGCACGTATTTATTTATCCTACACAAAGACTTAATTGAAAAGAATACACAAGAGTAATTTTTTTTAAAAAAAAAGTCACCGTTTATTAGTGTGTAGAGATGAAGCATTGAGGCGGTCTCCTTCCGTCATTCCTGCTTCTTTTTTTTTTTCGCTGCTGAAGATTTTGGAACTTTATTCCCAGAGAACTCTTGGCTCCTTTTTCTTTTTTCCATTTCGTCTGTTTTGTTTTCGCGCGTGCACGCATTAAAATTTTTTTTTCTTTTATATTATTTTTCTGCTGTTTTCTTTTCTTCCCCTCTCCTTTTTGGTCATATGTATATTTGTATATACCTATATATATATATATATTCACTATATAGTTTTATATCTTTGTGCTTATTTATTGACAGTGGTGTTAGTGACGTCTCTTTTAGTACGTGGTTTCCTTTTTTTTTTACTTTCTTTGTTACGAATTTATTTATTTTGTTGCATTTCTTTTCCTCTCCTCTCCTTTCCTTTCCTTTCCCCTTCTTTGTTTTTTTTTAATCTCTTGTTTCTTATTTATTTATTTTTTTTTTACGCTTTTGTATTCATTAAACTTTCATTAATGAATACAAATGATGAAAAGAAGGAAATGGGAGAAGGAAAGTAAAATGAGGAATTGCGTGTGGCAAAACAAAATCAAATAAAGAAATAAAGAAAAGGGGGAAAAAAAAGGAATCATGGGGAAGTTATATTAAATTTGCTTTTTTGTTTTGTTTTGTTTTCTTCTTTTCTTTTCCTTTTCTTTTTACTGCAGCCCCTTTTATTACCATCCATTCCATTATGGATGAACTGTGGGGCGATTTTGTTTTTTTTTTTCTTCTTTCTTCTCGTTACAAAGTGAGAAGAAAATTATTGTTTATTTTTGATTATTATGAGAATAAAAAAAAGGAAAAATGAATAGTGCTACGTATTTTATATATTTTTTATTCTATACTTCCAGTGCTAGTGGTACCTATCTTTTTTTTAAAAAAAAAAAGTTTCATTTTTTTTTCTTTGGTTATAAATGTCTTGACTTCGTTTGCGGTTTCTTTCAACTTTTTTTTTTTTGATTTCTTTTCCTCTTTTTAAAGGTAACGGACACGGTAACCGTTAGAGGGAGAAAAGAAAAAGAATGTAACAAAGAAATGTAAAACAAAGCAACGAATAAAATGGAGGAAAAACAAAAAAAAGAGAAGAACGTAATAATTTTGCTGAAGTCACAACTGAATATGATGGTGATTGCTTTTATTGCTACAGTTATTTTTGTCATTATGTTTATTTTTGTACTGGTTTTATATTTATGTATGCTGTGTATAAATTCTACACGCGTATTGCTCGGTCCTCTTTTCCCTCTTTTTTTTTTTGTCTTTCAGCATGAATCAACAATTTATTTCTGTTTAATTTATACTCCCTCGTCCCTTTTTATTCTCCTCTTCTTCCACTAGTTCTCATTTTTTTAAAAAAAAAATTAATCTGTTTCTTTTCTTTTTTTTTTGTTTTTCTTTTTGCTTCTCAGCGCACACGAGCACGCATTTAAATTTTCAAGTGAATGATTTTTAAAGCAGGAGGAAAAAAAAAACAATCTCCATTTATCTATTTATTTATTGCGGTTTCTTCCTTTTTAAAAAAAAAAATTTTTTTTTCCTCCCCTCAATGCGTTCCAACTTTTCCTTAGTTGTAACATTAGCGCTGTTTTGTCCACTGCACGCGGTATCCAAATGCTCAAGCACATATTTACACACACACATATATATATATATATATATATATGTTTGACAAAAGAAAGAGAAAAGAAAAATATGTCTGGGAAAAATATTGCATGAGGGAACTGGAACTTACACAAACTGGTGGAAACAAAAAAAAAGGGGGAAAAAAAGAAGGAAAAAAAAAAGAAGGGTGGTAGTGGTGGTGGTGTTGGCTACTTGCAATTACAAATGCACCACAAAGAGGAGGCAAATAATAATAATAATAATAATACTAATTATTATAATAAGGGGGAGGTAATAAAAATGAGTGAATGAATATAAAGAAGTAGTGGGGAATATAAACCAAAATAATAATAATAATTATTATTATTATTAGAAAAGAGCAAACACCAATAGTTCTTTTTTTTTTTTAAAAGGCGTGACGAGAAGAAGAAGAAAGTAAAGAAAACAGAGTGGGAGAATTTTTTTTTTTAATTTTCATCGCATTTTTAACCGATCACAATTTTCCTCCTTTGGGTGTCTGTTCGTGATGTTGCCGTTGTCATTTTGCATTTGTACTGATGGAAATTTTTTTCTATAAAAAAAAAAGGGAACTTTGACAATAACAACATTTTAATAGAATATAATTGAAAAGGAGAAGAAAGGAAAAAAAAAAACAAACAGTTATTTTCCCAGTTTCTGTTCTCTTCTGACTTATACAAAAACGCTTTTTGGATTATTGGATGAATGCTGCTTTTTCTACTTTTCGTTTTTGTTTCTCCTTCAAACACTTTGCTTCAATCGAATTATTTATTTTTGACGTTCCGTATTTATATTTTCACACAAAAGAGAAACCCCTCTTTCTTTTTTTTTTGTTTATACTTTCGGTGTAACAACCTCTCATGTAAGAGGAACGAAAATTGTAAAAAAGAAAAATAAGAGGAGGTGGATGATACGGCAGCAGGTAATAATGAAATAAAACATTCTTTTTTTTTCTTTTTTACTTTTCTTTGGTGCTGTTTGGCTGCTTTATTTGATCCGTTATATATATATTTCCTCTTTTTTTCTTTTTTTTTTTAATTTTCACTTGTGTGTGGATGTATTGGAGGAGGGGGAAGAAATGAGTGAAAGTTTTGAGTGAAATGAAGAAAAGTGGTTAAGAGGTCACACGTTGCCATTAATTTTTCTTCGGTTTTTGTTTTTCATTATAAATATATATATATATATATATTCACTTTTTGAAATGATACAGCACTCGCTTTGTACCATTATATCATATTCCGCTGCTATTACATATATTGATGAGAAAACTTATTACTATTATTAGTATTATTCTCATCGATAATGCTGCCCAAAAGGGTTTATCTCTTTATTGTTTGTTTGTTTTTGCAAATTAAATTAAATTAAATTAAGTTTTCATTTTCGAACCAAATTTTTTTTTTTGTTTTTCTTTTTTGTTTTTAGTTTTGCTACGAAGAAGGTGAGGGGCGGTGTGAGGACCAAAATATGGAGTGAAGAGAGGACGAGAAAAAAAAAAAGAAGATATTGGCACATATAAACGAGTGTTCAAATGGAGTTATATAAATAAATATAACAGACGGGGGAAAGTGTGTGTGTGTGTGTGTTTTATCTATTACGGGGGGGGGGGAACAAAACGATGAAAAATGTGGAAGTCACCTCAACAACATTTTTTTTCCGGCGTTTTTTTTTTTTAAAAGAAAAGAAAAAAGAAGAAAAAACAAAAAGAATGCACTGAAGCTTTACTACCTGGTGACTCTTGTATAGGTTCTTTCTTCCTCTTTTTCCCCTTCTTTTTTTTCTTTTTGCTTTCATCAATTTTATTTGTACACAAATATTATTTGAGAAGAGATATTGATTCACTTATCATTATCATGTTTACTTGTATTGTTATTGTTATCGTTATTACAAACTGTGATGAGAGGAACAATGAAGGGAATAAATAGCAGGAAGGGGTACAGTAAAGTAAAACACCGTCGCCCACCTCCCCAGTGTGGTTCTTTTTCTTCTTTTTCCTTTTTTTTTTTACAAAAAAAAGGAAAGAAAACTCTTTCAACTTCTCTTCCGTCTTTTTATTTATTTATTTAACTTTCAAATTCTATAAAAGCAACATTAATAACACCGTATGGACACCGTTCAAACTTCCCTTTGGTTCGTTCTGTTTTATTGGAATTACACCTCTTACAATCTCCATTGCGGAACCGCTTTCAACTCGTGATATTTACATTTACATTTTTTTTTTTGTGTGTGTGTGGTGTATTGGGGGGGGGGAGTGTGTTCCTCATGTGAATTCCTTTCCGCTTGTTGTAAACCTAAAGAAGATCAAAATTAGATAGATAAGTGAAAAAGGAAAAAAGAAAAACAGTGGAAGGAAGGAAATTAAAAATGGGGAACACGAAAAAGGTCATTTTAAAGGAAAAAAAAAGAAAATGAACGAACAACTCTTTCGAATTTATTTCCAACTGAAATGTTTTTGTTCAAAAATATCTCACCGTTGAGTTTCTCAATTATTATTTTTCTTTAAAAATAAAAAAATAAATTCAATTATCTGCGTGCTCTTACTTTCGTCCTGTACTGCTGAACTCACATTCTTTCTTTTTCATTTTCTTTTTTCTGAAGGAGTCGAATGCGCCTGAAagGGAATAATATCACCTTATGATAAGCAACAACTTAAAAATAATTAAATAAATAGGATAGGATAGGAAAGGAAAAATCATGGCATAATGTTTTTAAAAAAAACAATAGGAGTTTTGCGAGCATCGCAACAACATGAAAAGGTGTCACAACTACGTAATGTCGTACAATATTTTACGTATTGTTATGTTATGTGTTAGTTGGGATAAAATGTGAGAGAAAGAGAGAGAGGGAGGAATGTTTTTGATGTGGTTTCGTCCGTTAGGAGAATTGGTATTGTGTACCTTTTATTCTGATACGCATTCTTCCCTTGTGGAGCTATTCTTTTTAATTTAATTTTTTAAATATCTTTTCTTTACATCTGTCCCAAA

>Tb927.11.2860

GCGCAGGGCAATGCGCCCTCCCATTGTTTTTTTTTCTTTATAACTTTTTTTGTCTTCGTTTTCTTTTTTCTCTCCCTGTTTTCCATCTTTGCTTTTCCCCCCCCCTTCCCTTTTGTGTGGTATGTTCATTAATAACTCGGTCATTGTCGTGGACGGAGGCAGTGTCACATTCGGCCCACACCATTTGCCCACTTCGCTTTTTCGCCCCTGTTTTCACCCATTCCCTTCCCTCTTCTTCCCATCCGGTTCTCTCTCTCTCTTCGCTCGCTTTTATCTTTTGTTTTTTTTTATCATTTCCCTCACCGGTTTCCTTGTTTTCTTACGCATCTGTTCCTTTTTTTTCTTTTTGCTCTAGCTCTTTCCACTTTCCAAAGCATGTAATTCAACTGGGTTCTTTTCGTTTTTTTTTTTACTCCTTCCCCATCTAACCCTGTCGCTCTTAACTTGCATGCAAGGTTGTGCTTCCCCACTCAAATTTTCTTCACTAAATTTGGCTTCTACGTCCTACCTATTGTTTCACCATCTTTTTTTCCTTTGTTGTTTTTTTTGTTTTTTGTTGTTTTTGCTTTTGCTTTATGCTTTTGTCTTTTGTTATTCAACTTTTTCATCTTTTTGCGTTTCTCACATTTTAATTCATCGTCAACAGATGCCTGCAATGGTAACAAGAGGTGAAGAAGAGAGGGAGAGAGAGAGGAAGGACGTATAAGCGGAGAAAAATGTGTGATGCGAGATAAATGAAGGAGATGAATGAGAAAAGAATAAAACGGAGCCAAATCAAAAAGAAAGAGCAGCAACAACAGTAACAATACCTTGGAAGAAAAAAGAAAACTGTAACAACAACAACGCCAGTATCAACAAGAAACGGTTTTTTTTTTGCAGTTGATTTATTTTTTTTTTAAAATTTCCCTGCGCTGTGACTGTTAAGATTATAATGATTGAAGGAGGGGAACTTAACGTGAGGAATTGGGATCGATGGGTATTCAGTAGTGTGTTGGTGTTTTTTTTATTTTTGTTTTATGTTACGTGTGTCAATGAGCGGTTGAAGGGAGTAGATGAATAAATCAAAGTATCACGTTTATTATATGAATATTTATATTTATATCTACATTCATTTATATTTATTTACTTACTCATTTTTTTTTTTGGCGTTTTTGGTTGCGTGTGTACGCTTTTTTTTTTCCGGATGGGTGTGAGGGAAGGTGCATGAGGTAAAAGAAAACTAAATGTTTGATTGGGTGCGGGCGAGGTTTTTTTTTGTTTTGTTTTGTTTGGAGGAAAACAAAGAAAAAGAAGAGAAGTGATGGGACGAGAAAAAGTAAAAGAAAAAAAAGCGGCAAGTGGTGTGGAAGAGGGACGTGTGTGACCAAATTTGTGTGACCTCTTTGAAAACTCTTTTTTTTCTTTATTATTATTATTACTTTTATTGTTATTGATGTTGTTTTTCTTTCGTTATTTTTACCTTCTTTTTTTTATTTTAACTGTTTCTCTTCTATCCCTCTTTTTTTTTTTACCTTAATGATGTATGCGCAGAAGGAAGTCAAAGAGTGCGCTTTTGTTTTCTTTTGTTTTTTTTTTTTGCTGTCACGATTTTTTTTTTTTTGTGCACATCACTTACCCTTTTCTCATTTTTTCCCTTTTTCCGTTTTCATGATTTTCTTTCGTTTTGTTTTGTTTTTCTTTTATTTTCGATTATGTCGCGGATGTGATGTGTGAAATGAGCGCGACGTATATTCCGCTTCTTTTCTCCATTACCGAGTAGCTCACTTTTATGTCTTTTTTTTTCTGTTTTACTTATATCTATATCTATATATATTTATATATATATTTATATATTTATTTATACACCACTTTATTATTATTATTATAATTATAATAGTTGTAATAATTATTATAATTACAGTTATTATTATTATTATTATTATTATTATTATTATTATTATTATTATTATTATCATCATCATTTTTATTAGTTCAATCGCTTTTAGTCTCTCCCACGGATTCCCCGTTCCTTTTTTTTTTTAAAAAAAAGTATTTTTCTCTCCAATCGTCACACTTTTAAGTAGTGAATGGAGGGAAAAGGGAAAAGGGAGAATTCCGGAGTGATATAAGGAACGAAGAAAAAGAGTAAAAGAAACGGGGGGAAAAAAATAAAGAAATCAAAAAACAACAACAACAACAACAACAAAGAAACGAGTGAACAAGTGGATGAGTAAATGAGTGAAAAAGTGTAAAGGAGTCGTTATTTAAATAAATTAAAACAAAAGTGATGTGATACTTCAATTGTGTGAAGTGTGGCTGTGTATATAAACTTTCCTTTTAAATACTTTAAAAACTATTTTGCTGCTGTATGATAGTGTGGTGATGTGTGTCTGTGTTTCTGTGGAGAAAGCGAATACGTTGACGTGTATGTTTACATTTATATATGCATCCATGCACACACACACACACACGCACATATAAATATATATATATATTAAATTTATTTATATATTTATATATTTATTTATATATGAATGGATATGCTTGTCTCTGGAGAGGTGGTGGAGAAATTAACTTCATGAACGGGGAGATGATTGAGCGATGGGTATAATTAACGATGTGTCGCGACATTATATACATACATATATATATATATATTTACATGTTATATATGTGTGTATTGCTTTTGGTTTTTGATTTTGTTTTTGAGGAAAAGAATAAATAAAGTAGTGGGAAACGTTGAGGAGAGGGAAATAAATACTCACAGGACACGCGCATATCCGCACACTTATACAAAATAGGTGGTTCTTTTTTTTTTTGTTTGTCTGTTTCCAGTTATTTCCCCCCCCCATACAATAAAGGTGTGGATCATATAAAAGAAAGAGGTGAAATAAAACGAAACAAAACGGCGCCAACATGTTATTTTGTTTTTAAATATAAAAGAGGAGAAAAGGAGAAAAAAAGAGAGAGAGAGTGTGTGACGGAAGGAAGGAAGGAAGGCGTGTAAATAAAGTCACGTAAATGATACCTCTAGTGGACACATGTATAGAGGCAAATTTTGTAGAATTCAGCCTTTCCGTCAAAAGCAGGAAGAAAAGAAGAAATAAAATTAAAACGGTGAGATCAAACAGTAAAAAAAAAAGGAAGATAGAAGGAAGAAACAAATAATGATAATTCTTTTTTTTTTTCTGTAAAGACAGAAGATCAGGAATTACAAATTGATAAAGTACAAGTATGAGGGTTTGGAGGGGCGACGCGCTTATTTTCCTTTTTTTTTTGGTTAAAAAAAGTTCAAAGCAACCGCCGGTACGTCAGTTGAAGTGAGTTATTATTATTATTATTATTATTGTTTTTGTGTTATTTTTGTTTTGATTAGGGGGAACCCTCTCCATTTAAGTTCTTAAAGGGTTGCTCATGGCATTTTAAAAAAATTTTATTTAATTTGTAGATGTACATATATGTTTCAAAAGTATGTAGACATATACGTATAAAATATTTATTTATTTTCCCCAGATCTTTTTACCACCTATCTGTAACATCTTTCTTCTTTTTTCCCCCCTTTTTATACCTTCTCAAACAAATCTCCTTTCTTCAGTAAATGGCGGTAACTGTACGGGTGTTTTTATATTTATTTATTTATTTGTTTCCTTTTTTTTTTTCTTTTGTCATTGGGCGTATGTATCGCGAAAAGGACAAGGAGAAGGAGGAGGAGGAAAAGATGCGGTAATAAATGAGATATGAGACAAAATATTACGGTCTTAAAAAAAAAAAAAAGAGATAAAAGGAGGATAAATAATATACATTAAATCATATAATATAAAGAAAGGTAACCACAGCACTTTGATATCAAAGGGGTCGCCCCGTAAGGAATGGGACGTCCTTAATGCATAAATAATAATAATAATAATAATGGTGTAAAGAAGGTCGAAAGTAACAATGGGTGATATCAAAAATAAATAAATAAGAAAAAAAAGTAGCTATTGTTATGTTTCTGAACTATTTTGCTTCGTTTTGTTTTTTTAATTTTTTAATTTTAATGAATAAATAAATTGATAATTAAATGATTAATTTATTCATTATTTTTAAAGCACTCCTTTTTCTCTTTCTCTTCTTCTGTTGTTGTGGTGTTTACCGCAGTACACTCTCCCAAGTGAATATATGATGTGAATTTTTTTTTTATCGTGATATGTCAAACCCGCATTGTGCTCTCATTCGCTTATTTGTTTATCTGTTTTTTTTTCTATCTTTCAATTCCACTTTTGTATTTACTTGTGCTTGTTTTCTTTTTTTTTTTAATTATTTGGGTGTTTGTGTTTGTGTTTGTGTTTGTGATCGTGCCTCGACAAGAAGAAAGAAAAAAGAAAGAATAATTAGATTAACATGCAACAGTAACAGCAACTAAAAGGGAAGAAAACTAAACTGTTATTTTTGGTGTTTGTTTATGTGAGTGTTTCATGAGCACGTTCACTGTGTGTAAATGCGTATATATATATATATATATATATATATATGATTTCTTATGCTTACATGTACAGCTTTAAAAAAAAAAAAATTGTGTCTGTCTGTCTGTATGTTTTTTTTTCCTGTGGTCTTTCTATCTATTTTTCGATCTGTTTCGTTGTCTTTGTGTGTGTGTGTGTGTTTGTGTGTTTGTGTGTGGGTAAATTTTCCTTTATTTTTTCCCTGCACAAAAAAAAAAAATACTTTTTCTTTACTTTACCTGCGGAGTCTGGCCTCTTCCATCCGCCTTCAAGACGTTTTCTTTTTGTATTCGGGTTTTGATCAGTATTTTCTCCTTTCCTTTTCTTTCCTTCTATTCTATTTTATTTTGTTGTGTTGTGTTGTGTTTCCTTTTTCCCGCCCTTTTCCACACACGTGTGCAAATAAAAAAAAAAGGGGGGGAGGGAAGAAATTACAAAATCAGACTAATGACTGGTAAGGGAGGGGAGGGGAGGGGGGTAAGTGGTCTTATTACATCTTTTCTTTTCTTTCTTGTTATTATTATTAATATTAATATTGGTGATGTTTAACACTGTTGTAAATATTCAAAATGTTTTTTTTTTTTAAAATTGATATTAACCAATTACAATCATGTTTAGATATCCAGGGGCTGTTGATCGCTCCGGTGCCATCGTCAGTCAAGTCAGCTGCAAAACTGCGCACTTTCTGGCGAGAAAACAACTTACAAAGAAGAATTAAAATAAACAAAAATAAAATATTGGGCAAAGTATGATGGGATTGTATACGAAAGTGGGTGAAACAAAGCAAATAAAAGGAAGGGAAGAGGGTTTGGAAAAAAAAAAGGAAGAAACGACAACACTAATAAGCCAAGACAAAGGCGATCAGTACAGTTTTTTTTAAAAAAAAGAAAAGAAAACAAGAACATGAAAAATATATATATGGGAACACAAGAAAAATGAATGCTACAACCAATGAAGAAAAGAAAAATAACAACTGAAAGAATAGTTGCTACTTTTTATATCCCTGAATTAGTGGTTTCTCCCCCCTTACCTTATTATTATTATTACCATCACTATTATCATTACTGTTATCATTTTTACCACCCTTGCAATCACTACTATCACCATTTTATTATTATGTGTTAGACA

>Tb927.6.690

AATGACTTGGATTGAGTACCATGCGTCTGTTCGTTTAAGTGCTATGCGATTTCTACGAAATATGAGGAATGAATAATAAAAGGAAGGTGGAGGCGCCAGAGGGAAAGGAACCGAAGAAATGAGATACGCTGCTCCTTTTAACCTCAAGTTTTGTTTACTATTATTATTATTATTATTACTGTCGTGGCCTACTTCTGTAAGCTTGGGATACGCAAAGATGAAGGAAAAGAGAGAGAGAGAGAGAGAGGAAGAATGGAGTAGAAGAGACGAGAAGTGTACAGTAGAAAGCAATACAGTGAAGGCTAAGTGATGCTACGGCAGTAGTAAAAGAGGAGCAAACAAGCAAAATAACAGAAAGGAATGAAAAATAATAAAAAAGTAAACAAACAAACAACTGGTGTGATCATGATGTATGGTGAAAAACCAAAACCAAAAACAAACAAAAAGCAAAATGAATGAATAGAGTAAAACACAAACACACGCATGCGCACGCGTATATATATATATATATATAAGTGTATACATAAATATGTTTATGAGATTTTAGGATGAAACAGGACAAACCAAAGGAGGGGGAAACAAATCAAGTTAAATCAGCAGGAAAGAAAAAACGAACATGCTCCACCACTCCGATGGTGAGAATTTTTTTTCTCTTTCCCAACTTCCATCTCTTTTTTTTTGCTTTTTTTTCCTCCAACAAAGACAAGTAATGTGCTTACGGCCTGTTTTTTTCTTTTTTCTCTTTTCAATTGTTTTTTTTTTTCATTTATTGCTATTCTCTCATCCCGGCCGTATATTTCCCCCCTCCATTCGTCGTTATGCGACACCGAACACATCACTCCTTTTATTCCTTTTCTTTTTTTTAGATAGTTTTCTTGTTTTTATTGTTTTGTTTTGGTTTCGATGTCTGATGTTTTGTGTTCATTTGTTTTGTTCCTTCCATTTGTTACGTTTCATATATATATATGTACATGTAACTGCGTGTGTTTGTGTGTTTGTGATTATTACGCCTGCTTTTGTTGGTATTAAAGTTTCTAATTTTTTTCTTTTTAATCCACTAACTTTTTCAATAATTCACAGAAGGAGGAAAAACAGAAGCAGAAAATAAAAAAGAAAATAGATGGCGACCTGATAATAGTAATAAAAGTAATGAATGGTGTTAAAAAGAGAATGAATTAAAAAACGGGGCTAAACAGACACGAATATTTCAACAAGAAAAATGAAGGAAATATATCATTATATGTGTCCAATATTTTCGCACATATGTTTGTGTTTTCTTTTATATTTTTGTGTTTATATCTGCTGTTTTTGTGTGCCTTTGTTGATTGGAAGATCAAAAGGAAAAAAAGGAGGAAGGGAGGAGAATTGGGGTTGTTTGGGGGGGGGCATCTTCAGGAAAGAATATATATATATTAATATTAATATTAATATGTGTGTGTATTTGTGCGTTGGGGTGTTTGTATTGGAAGAGGCATGCAAAAGCAGCGCAAGTCCTTCCACACTCATTCGTCCAAATTTTCCTTTTTCTTTTTCTCTTCCTTTTAAATTGTTTGTTCCGGTTTATATTTCTTTAAATATATAAAAATTAGGGTTTTTTTTGGGGTTGGTGTTTGGTTCTTTCTTATTTATTAATATTTCTCCTGAGCGCCTCATCACTCTCTGCGGTGGCGCGCCCCTCTTCCTTTTCCCTTTTTTTTTCCTTTCCTTTTCCTTTTTTTTGTTTTGTTTTGTTGTTTCGTTTTGTTTTTGCTTTTGTTTTGTTTTCATTTTTATGCATATGTGTGTTCCTATGGGGTGTGTGTGTGTGTGTTTCCCGCTTCATGTGTGCTCACTCATGCATTTGATCTTTTGTTGTTGTTGTTGTTGTTGCCATTGCCGTTTTTTTTTTAAAGAAAAAAAAGAAAAATTTAATATTACTATCATTATTTCTTTGAATTGCCATGTGTGTATTGTTTTATTTCATTCACGGTGTTTTCCGTCTTCTCTCTCATTTTTTTTTCCTTGTTTACTGCTTTGATGCGTTGGCATCCGTTTATTACTTCTGATATTTTATTGTTCTTTTTGTTTTGTTTTGTTGTGTGTGTGTGTGTGTACGTGAAGGGAGGAAAGATTAGAAGAATGAGAATACCTATGTATAAAGAAAAAAAAGAAAATAAAGAATATATATATATATATATATGTTAGAGGGGAAAAAATACTAAATAATAATTGTTATTATTGTTATTATTATTATTTTAAGTTAAAAGGAAAAGAAAACCTTCCTTCTCACGATGTTGTTCTTTTTACCTTTTTTTAAATCTTTTTTTTTGTAAAGAAAAAATATTTTCTTTAGTCTTGTAGCACTTCCTTTTTTTTTTCCTTTTTCTCTCTCTCTCCTTCTTGTTACTTTCTACTTCCCTTCCCTTTAATTTGACCCGCTTATTCATGCGCTTTTTTTTATATGTTCATCACTTATATTTATGTATATATTTAACCTTCTTTTTTTTTTCTAAATCTTTAGTTGGTGCTGTTTTTTTTTCTTTCTTCGTTGATTTTTTTTCTGTCTTTTTGTGTGTGTGTGTGTGTTTGTGTTTTATTTGCTTTCGTTAAAATGATAATGATAATAATAATACTTTAACTTTCTTTGTATGAGATTTTTTAACTCAAATGAAAGAAAAGAAGAGGTGGAATAAAAAAAAAAAAGAAAAAGAAAAGAAAAACGCCTGCGGTCCTGTTAATGGCACTGAACGCAAATAAATACGCACAAGGAGGGAAACCAAAGCCAATGAAGTAAAATGATGAATAAATTAATCAGTCAATTAGTAAGAGAAGGGGAAAGGAAACAAAGAAAAAAAGAAATATATTTTAAAATATATATATATATATTTGAAAAAAAAATACTTGACCGTACAGAGGGGATGTGATTCCAAAGCAAAACGGAATGAAATAAATAAATAAATAACGAGTAAGACTGAGTTTGTTGTTGATGATTGATTGTTTGTTTGTTTTTATCTTTTAAGTATGGAAAGACAATTATAAATAAAGTGAACATGAAAGTGTGTGAAAAGGGATGAACAGAAGAGAAAGCGAGAAAAATAAAAATAAAGTAAAAGTAAAGGAATGAGAATGAGAATGAGAATGACACCACAATTTGTTGAATAAATGAGTAAAAAAGAATTGGTGGTATTGGTAGTAATAACGATTAATGGAAAAAAAAAAAAAGAGTGCCTGAGCGACACGAAAGGGGTGTAATCCCCCTACAAAAAAAAAAAGGAAAGAAAAAAAAGATGAAACGGAAAGTAGGGGAGCAAAATCGAGCAACGCAATAAAATAAAATAAAATAAAAGTTGTTTAGAATCAAGAGGAGGACAAATACTCAAGAGGGGGAAAAGAAAAGAAAGAAAAGTAAGATCTGAGAGTGTACAACCGATTTTATTTATTTATTTATATTCGCTTGTGTGTGTTTTTGTGTTTGTTATTCTTTCTTCCCAGTATTATCTTCCACTTGGTTGTTTTGTCGTTGTTTTCTCCAACTTTTTACACGAGTGATTTGGTGGGTCCCATGTGATTATTTGCATTGATGCGTCTGCCGGAAGTTCGTGTTTTCGTTTCAGTTTTTTTCGCTTTTTTTTTCTTTTTTCTTTGTTCTTTCCTCCAATTGTTATTATTACTGAGAGTTGATGTACAATTTATGCAAGTTGTTGTGCGTTCCCATACGCATTTAATTTCTTCTGTTGTGTTTCCCCCCTCCTCTCCTCCTCCTCCTCCTCCTCTCCACCGTCATTCAGTTGTTTTAGTTCATCAGAAGTAAACAAATAAATAAACAAACAAATAAATCACACCCACTCACACATGAACGTACAAACGGAAGAACGAATGAAATAAAAATAAAGCCATGTATGTATAGATGTGTATGTATGCTATTGTTGTTGCTGTTATTAATATTTATTGTTATTGTTACCATCGCCATCTCACCTTTCAACTTATGACTATGACTGAATGTGTTTCCACACATGAGCGCTTGTGTTTTTTTTTTTCTTTTTGAGTTGATTTGCTTTGCTTTATTTTTAATTAAATTCAACTTTTTTATATTATTATATGGTTCCTTTGCGTTACAGTTTCTCTTTTTTTTTTTCTGTCGGCCATTTGTTTGTTTATGTGTGTGTGTGTGTGTGTGTGTGTGTGTGTGTGATGAGTTTGTGTGTGTGTTTGTGTGTTCCATTATGATATGTTTCATTACATATTTTATATATTCAATTATGTGCTTATATTTATGTTTGTGTTTATATGTGTGAAAAGGAAGGGGAATAAATGAATTAAGGAAGTGGGGGGGGCCATTTGGGGTTTTGCTTTTGTTCCATTCCTTTTGCACTATAATTTCATTCCACGTGAAACGAAAAATAAAAAGAAATGAATTTAATAAAATGGATGAATGGATGGATGGATAAATAAATAAATAAAAGGAAAACGAATAGAATAGAAGAGAGGAGAGAAGAGAAGATAAGAGGAAATATAATCGCTCGAAATATCATGGCATTTGAATGAATGTTTTAAGTCACTTGACGGAAGCTTGAAGCTTTTCATTTTGTCACGCAGCCGTTACGCGAAAAATGCAGAAAATATGGGAGATGGTGGTAGTGGTGGTAATTTTCTACCGCTATAATTACTGTTACTATCACTATTATTTTTATTATTATTATTATTATTTCTTCTTTTGTTGTTGTTGTTGTTATTGTGTGTACTTGTGTGTATTTATGTGATAAGAGAGATGGGAACAAAGTTATAGATGGATTTTAAGTTTATTGTTATTTTCATCATTATTTGTATTGGTCAAACCTTCCTTACTCCTCCTGTTATGTTTGTTCACTGTTGTTGGTTTTTGTTTTTTGTTTTTTTGTTTTTTGTTTTTTACGTGTGATTCTCCCTGCTGGGGCATTGGAGACACTTATTACTTAACAGTGACCAAATATATATATATATATATATAAAGTTGTCTATATTTTGAAACTTGTAAGAAAAGTACTAATAAAAAGGGGGATATATTACAAATACACAAAAATGTATAAAAATAAAAATAGAAAAAGAGATGAAAAGGTGGAGTCAGTATTTATATAAATTCGTGATGATGGACATAACAAACTCGTGGTGCGCAAACAAACAAATTCTCCTACATAATTTCATCTTCAAATATTTTTATTTATATGGTGATATACCCATTTATTTTATGAATGTTTACGCGTCGTGGGACCGTTTCTTTTTTCTTTTTTCTTTTGTTTTCGTTTTTGTTGTTCTTTACTATTTTTCTTTTATATAACTGTTATTTTACTTTCACGTGGTGACGTTGTCATTCGAGAATTCCCCTTATTTCCTTTTTTTTCTTTCTCTCTCTTTTTTTAATTCTCCCTTTTTGTTGGTGTTGTTGTTGTTGCTTTTTTTTTTTTAATTTTAATCTCCATATAGTCATTCTATTTAGTTTTGTTTCAAAGGGGAGGCAAAAAAAAAAAAAGACGAAAGGCAACCGAAAGATTTTTTTTGCTCGCGGATATTTTCCCTCATTTCGTTCGGTTTTTTTTTAATTTTTTTTACAGTTTTTTTTTTCCGGCAAAAACACCAAAAATAAATCTCGACATATATATATATATATTTATTTATTTATTACAACGAGAAAAAAGGTGTTGACAACCGAAAAGTATTTTAAATTATTTCAGAACAATAACAACAATAAAAGAAAACGAATGCTAATTGCTAATAATAGCAATAATATGGGCACCACGAAAAAGAAAAAAAAAGACGTGAAGAATTCTGTTAAAAAAAGGCAAAACGACAAAATGATAAATCAAAACGGATTCGTTTCATTTACTTTTGTTGTTTGTTAATTTGTGTTTTGCTTTTATTTTTGATGCCGGTCTTCCCCATTGTTTTCCTCTTCTTTCTTCTTTCATGATTTATCTCCAGATTTGTTGATGTTGTTGTTTTTTTTTTTTCCTTTTAAATGGTTGTCGCGATTGTGGGAAATAAAAATTAAAAAAGAACTTCTCTCCCCATCATACCACTACGCTGCTGATATGATGTTTTTTGTGAATAAGTATGCAAATGTTTTCGTATTGATTTTAATTTTTGTTTTACCTTTTTTTTTTACAAATATTATTGTTTCATTTGTATTTATTTATATATTTATATTTATTTATATGATATAATATGGGATTTTTTTTCATGTTTTACTCATTTATTTGTTTGTTAATTTGTTCTTCAAGGCTTAGATGTACAAATAAATAAATATGAAGCCAATAATTGGATGTTGATGAGGGGGAAAAAAAGGGGGGGAGGTGAAGGGAGAGTTTAAACACGTGATGTTGACATTACAGTTTTTAATGTTGTTTTGTTGTTTTTTGTTGTTTTTTACTTTTAAGACATGTGGACAGTTTTTCAACAGACCTCCCTTTTCCCCCAATCCCCTGCCCTTCTTTTGTTTTATGTTTTCCTTCTTTTGGTTCCTTTTTTATTTTCTATTTGTTGTTTATTTATTTATTTACAGGTTTTGTGCACATAATTCGTGTGAATATTCATGCACCGAAATATATATATATATATATATATTTATTTATTTATTTATATGAATGAATAAATGAAAGAAAGAAAAAGGAAGAATAGAAATAGAAATACAAGATATGTTATGTGAAAAAGAGCATTAAAAGCGACTCACCGAAACAAGAAAAAAGTTGGGTGATGACCAAGTGATGTGAAAAAAAAAAAACATTCGAAGAGAGTTTCCTAAAAATGTGTTGGAAAAGAAAAAGGTCGAGTGAGTGAATGAATGAAATAATAATAAAACAACAATAATAATAATAACAACAAATTGCTATCGCTTCACTGTTTTTTATTCTGAAATAAAGAAGCAGTCTCAGTAACCTCAAGTGGGTGGAA

>Tb927.11.14860

ATTGACAAACCCATCTTTGTATATAAAAATACACATACTGACACATACACAAATTTGTGCACATCCATAGATAACGGTATAGAATCATCGAAATATTTTGTCTCCTTTTTTATATATAAAGTTACATCCATAGACGTATCTTTGCTGTTTCTCACGAGAAGGCCTATTTATTTATTTTTTGCAAATCTCATCCCCCTTGTACTTCTCGGTTCCAGGTTGTAGTATATACACATGTGAATATGTGTTTGTGACTGTGGGTTCTACAACGTTTTTCATACCGTTTTCTCTTCTCTCTTTTCCAACCAGCTGCGCGCGTAGGTCGTACGAAGACCACGATTTGCACGCATCCCCTTAGCTGTAACTAATGCTTAAAAAAAGTAAATCAGGACCACAACCGTCACATACATTCGCTCCCTCCTCAGAACGCTTTCTTTTTTTTTTGTGTTGTTCCCCTGTCATTCACACTTTCTTCAACGTTTTCTTTCTTTTGAACTTGCCTAGAAAGCAACATTTGTTTATCTTTAGTTTAATCGTTGATTATTCCTTTCTTCCCCCTTTCCAGTTTTGTCTCCTTTTACAAGGACTCCGCGCTGGTTGAACGTCATCGCGTTCCAAAAGGGGGGAACTTGTTTTTTGGTCGCGAAGCTTATTTCATCGAATTCCCAGCGTGATCATGTCTGATCAGGACGGCAACCACTCGCCCTCACCTTCCCCCTCGCCATGCTGTTCTATGGAAAGTAGTGCAGAGACATTATCCTGCCGTGGCTCGCGGGCGGAAAGTGAAGTGCCTCACCGCTCGCA

>Tb927.1.3560

AGGTTGTTCGGTGAGAGCGGGCGGAGGATCTCGGGTGGAGTATACTTTCGTGGGCGCATACGTCTGCACATGCACGCACACACGCACACACATACACACACACACACTTGTGATTATTTTTTTTTAAACTTTGTTTTTCTTTGAAACCCTAACCCACTCTAATCAACACGTATCGTTCCAACGTTGTGTTACTACTATTTCCTCGAAAAAGGGGTATCAGCGGTTGTTTCCCCTCAGAGGAACTAGAACAGTAAGGGCGACGCTGACGGAGAAAGAGTAAAAGCGGGACATGTAGTGATATCATAGAGTTTTTTTTCCATAAGAGATTGAAGGAGGGCAGCTGTGGCACTTTTGTTTCCACAGTCTTACGGTGAGAGGAAGGCACTTATTCCAGGTAGTACCCCGTAGACATTCGAAATGGGTCACGCGGAGATGTTTCCTAAATTGCATGGGCTCTCTGCATTCTCTGCCCCGAGAATTGCCATCTTTTTTTTTTTTTTCATTCTCCAGTAACCTCATATAGTCGATGTGGTCTCAGTGTGCCCTTATTTTTCTTCTCCTACTGGAGAAATCCACTTTTTATGTTTATTTTTTTGCTTATTTATTCGAA

>Tb927.11.15510

AAGGTATTTTAATCCATCCTCATTCGTTTCAATTTTTACAGTTACGCAGCTGAAGAGGCGTCAGCATTGGGATGGAACCCTTGCATCTATTGCAGTGGCGCTGCTGATGGTGCGCTTCCTTGTCACAACGTTGCCTTTTTGCTTCTGTGTCTCACAAAAGTGGGCAAAAGAGAAAAGAGGTTCTCATGGTCAGCTTTCTTTGTTTCACTGATTATTATTTTTTTTCATAGCTATTCTAGTTTGGGCTTTTTTGTTGCTAAACCACTTCCCTCTTACGTCCGCAAGAAGCTTTTTTTTTCTCCCTGTCTTGTTCACTATTGGTTTACTTTTTTAAAAAAAAAACTTTTTTTGTTTTTTTTTCTATGAATCGGCTTGTTAATGCAGCTACCAAA

>Tb927.6.3470 NON_UNIQUE

GCTTTTTTTATTTTTTAATTTTTTAATTTTTTTCAGTTTTGAACTCCTGAGTAATGTGTTAGTTTGTGTTTTGATGTGTATTCAATCTTGAACGTGTATCACCACCACTTACAATTTTTATGGTGGTACTCTCAAAAACGTTTCAAGGTATCATAGATGTCGGAGGTTTAGAAACGTGGTAAGAAGGAAGTAGGATGATATTGACACTATAGAAACAATTAGATAACGTCAATACATATATATATATATATATATATATTAATTTATATACGTGTATTCATACATTCGTGTTTATATATACGTATGTATGCATAAATATTTATACATTTATTTTTCTATTCCATGAAACAAGGACAAAAAGCGAGGAACTACTTACGAAATAGAGAGTAATATGTTTCCTTTACTTTTTATTGCTGATTGTGTTGTAGCAGTGTAATTCTACGCTCCTTTTTTTACTTTTTTTTTGTTGCCCCTCTTCTTAAA

>Tb927.9.3100

AAACTTCGGGGAGTCACGTGCTCGTTTTCTGCTTTTGGCAGGGCACTGGGTAGCAAGTATGAGGCTCAGACGCTCTGGGTGAAGGATTGGCCCTCACTAGTGCTTTTCAGCTGCTACCCGGTTTAGATCCACGAGATATGCCCGCCCTATTTTCAATTTTTTGCATTCACTTTATCACATTATTTCATACAGTTCTTTTTTCGTTTGTTTGTTCTCGTTTTGTTATTTTCGTCCGTGTGCTTTCCTACTTTGCTTCACTTTCACCATTGTTATTTTTTTTATTTTTCGAGTCCTCTGCACTGCTAATTAAATATATTTCCCGCCTTGTTTTTTGTTGTCAGCAATGTTGTTTTCGGTTGTGCATTTCCGCTGTCTCCCGCGGCAGGCTCTCTCCTTTGAGCTTGTTGGGTATTCTATGAACTTCAACTCTTCTTGTAAAAAAAA

>Tb927.8.7710

AAGCGGTGGCAGTGTTTATATGAAAAAAGTAGAAAGTGGGGGAGCGACGGCGCGCCTCTAATCATTTCGAGTGTATGTGTTCCGGCACAATTCAAGGGAATGTGTAAAAGGGACATGTGACTTTAATCCTCCGTTAGGAAAAAAAAAGTTGAGGAGCAAGGAAATAGGCTTCGGGTGCCGTATTTCAATTGGTTATCTCCTTAGTTGTTGTCACACATTATTTTTTGGAGCTTTCTCTGGAGGTGACTACTTCGTTGTTGTTTTACTGAAACTTCTACCCATCTTTTTTTTGTCTCCATTGATTTACCCTCACATGTCTTTGTTCCCCGTAGCGATAGTTGCTCCTTGTTATTTATTTTACTGTTTCTACCGCCCGGTTTTGCTCAAATTTGTCATGTGATTATTGTGCAATCGTGTCATTAATAACGTCATTCTCAGCTTCTTTTCGCCTTTTAAAGTAGAAGGAGTGCAGTGCTTCGGGGGCTATAAATTGGGCTATGAATATTTCCTTATTTGTTTGATTGTTACCACCACTTTTTTATTTTATTTTACTTTCTCCTCTCTGCGTTCATATTTTTGTGTTTTTTAGACGTTATTGTCTTTTCTTTTTTCTCCTTTAATTATACACGAAAAGTGTGAAGAA

>Tb927.5.3440

GAGTTTGCACTTGGGTGTGTTATTTTTTTTTGCCACCGTTACATGATCATTTCTTGCGGCAAACGTTTTCGGATATGTCCTCCTTTTTTTTTTGTCCCTTAATTTGGAATGGTTAGGACGGAAACAGCATATGGTTATAATTTATCACAGGTAATGTGTTTGTAACTTTGATGTCGCAGGCTGTCACAATATGATACACAATTTCTTGTATTCGTATTTGTATGTGTTTTCGTGTTTCTTTCTTTCTTTGTTTGTGTGTTAAGGTGGTAAGTTGCCATGCAGCATTTGTGCGGCTCAACAATTTTAGTTTCATATTTACTTTCTTTCGACTGGGTGTTGTTACCTATTGATTATAGTTGTTTTTTAATTTTTTGCTTTTTGTTGATTGCAATACTACTTTACATACTTTTTTTTTTTAATTATCTGCATCTTCTTATTTTTGTGCTATTTTTTTTTGTTCCGCGCTTTTATGTTTATTCTGAGTGATGTATTTTTTTCTGTTACTTCTTTTTCACTGTTTATTTTCTGTAGCCTGTGCGGGTTGTGCTCCTTCACTGAA

>Tb927.3.3320 (identical ORF to 3.3310)

AATTTGTTTTTTTCTTTTAGGTTCACGTCGGCGGCATTTGCATGTTCTCCGAAACCACTACTTTCTTATTTTTTTTGCTCTATGCAATATCCGAAAGGGGATGTTTAGACGCATTACTTTCCTGTAAA

>Tb927.10.3360 PTP1-interacting protein

GTGTGGTTGGATTGTTGTTATCATTCACTTATTTTAAAGGGGGAAATATGCTCAAAACTAATCTCATTAATACTACCACCACTGCCTCCCTGCGGCTATAGCGTTTTAAGTGCATTGGGGGGTGGTACATAGACAAAACGTTTTCTCGTCGTTGTAGTTCGAAAATCTTCCCCCTTCTTATTTTATTTTCCCTTATCGCGCTTATCACTTCGTTCTTAACAGCGCTGATTACTTTCTCTAGCGGGTGTTGTGAAACTGCATGGAAGACTTTTCTTCTTTCTTTTTCTCCATACGATTATTTCTATCATTAAATATTTCGTTCTTTCTCTTAGTTATTTATACTGTGTTTATCTTCCCTCGTTTTCTAAAAACAAAAATGCCTTCTCTGTGTTTAAGTTATTTTTTTTCTTTAAAAAATAAATTATTGTTATTATTATTTGTTGGTTACCGTGTATTCAGCCGTAGAGGCACTTTAGTTCCCGCGTTTGAGAGTGAAACCAGAATATTTGTTTAATCTTCACATGCGTAACAAACAGAAACCCCTCGTTACGAGAAGGGATATGAATGCTCCATGCGAGAAGAGTAAGCACAAGATTTTATCCCCTGCCACTTTTTTTCTTTTTACACTCCAGTTTCACAGAGTCATAGCACCTACTCCCGGAAGCGCCTCACGTGTTCTGATATGGGAGTCACTTTTTCATTTCATTTCATTTTTAATTGCTATCTCTTTTATAAGTGATCCCTCTGTTTCATTAATTTCAAAATCGTTATATTAGCCTTTAAAAACTTTCAGTGTGATCTTTCCGTTGTTGTTTTACCATCCCTTATTCTTCCCACTTTTTTTTTGCTTTTTGATCATATGATCAATTTTTAAAAACAATATATTATTTCGATTCTTTACTTTATTGATTATTTTTGTTTCTTTTATTGTTTTCTTTACTGTTTGTTTGTTTGTTTTTTTCAGTTGATGAGATTGTGATTTTGACAGGTTTTGTTTTTTGTTTTGTTCCTCTCCTCCACACCAACGAAAACAGAATGGAATAAATATTCAACAAGGTGAGGAAAGGGAGAGAATGAGAGATGAGGTGAGGTGAAACGATGTGACATTGCCACTCTGCAGGAAAGATGGTAAAA

>Tb927.11.2450 - this is probably part of the 3'-UTR of Tb927.11.2460 This is the "ORF" sequence.

ATGCGCGTAGCTGATCGGATGAATGGATACACTAAACACTGTTTTTATTTTTATTTTTTCCCCCTTTTTATACTTTCCTCTTCCCAATTTCTGTCCTCTGTATCACTAACATTGTTGATGGTATTTCTTCTGTTTCCGTTTCTCCATTTCATCTCCCTCTCACACAAACTCGTACATACTTTAAAAAAAAAAAGAGAAGAAACTGTCCCTCAACAGGTTTCTTTGTGTTGCTCTACTTATCGTCGAAAACTCTTCTTTCTTTTTTTCTTTTTCTTGTTTCTACTGAAAAGCAGACATTTATATAGAGGGAGAGGAGCTGCGTATTTGCATTGCCTCCACCACCAAAAAGGGAAGGGTTGTAAGAAAATTATTTATCATCTCACATATACAAAAGCTGCTATTGTAGTTTTTGAGAAGTTATTTTTCCAAAAGAAGAAAAGCAAAAAAGGAAATAGATTATCTGGAAAGCACGTAGAAGAGCAGGAAACAAAGCGGAGAGCACTAGGTTAG

>Tb927.10.3420 ORF sequence - probably not an ORF.

ATGAACTTAAATTGTGAAGGACGTGACGTGGGCAGGAATTTATTAATGGAGGGAAAGGGTAAAAAAAAAAAGATAGCACATTTATTTATTTATATTTATTTATACATATGCGCCACAAACCGATGGGGAGAAATGAAAATATCCTTGATTTTGTTTGTGGTTTTCGTCCATTTACATTCATTTTTTTTTCTAATTTATGGTCTCATTAACTCTTTCGTTTCTGTGACTTGTTTTTTTTTTTCAGTTGTCGTGGAAGAACCTGCATTATTTTGGTGTATTCAATTAGATATGACAATCCTCCCCTTTTATGCAATTAATTTCTTTATTATTGTTATTGTTTTGGTCCCGTTTGAAAATGTGGCACGCACCTTACTTTCGCTTCAAATTATAGCGCGACTAAATACTTTTTTCTTTTTCTTTTTTTTGCTTTCCTTTTTTTTAAAAAAAAATATTTATATATATTTATAA

>Tb927.10.15040: The 3'-UTR probably includes 927.10.15050, 15060 and 15070 (shown in lower case)

GGCAAAAGAGTAGCAGAGGTGATTAATTTCTCCTACTTGGGTTCCATTTTATTACCATTATTAATTTAGGAACAACTAAATATTAACGAAATAAAGGAAGAAAACAAAAAAGAGAACGAAAAAAAAGAAAAAGAAAGAAAGAAGATAAATAAAGGGGACGTGGGAGAGCAGAAGAAAAAAAAAGGAAAAGCAAAAGGAAAAGAAGCTAGAAAAGTAATGGTGCCCATCACATGATGGCAAAAAGGGAAGTGTGTACGTGCCAATGTGGAGGTGAGGGAAGTGAACTGATCCTTTACTTTTGTCGCGCAGTTGTTATTATTGTAAGGGTTGACTATCATTATTTTTATTGGTGTTATTGTTACTATTATCATTTCAACGAGTAAAGTGAACGATCAGCAAGTGAATAAGCACGAGCAAGTAACCATTAATAATTATATTGCATACAGTTGTACAATTATGTGTGTAAATGTACCTGTGTGTACGCGTTTTTTTTCCCCCTTTATGCTTGTATGTTCGAATCCGGGGATTTAAGTTGTTGTTTTTGTTGTTGTTGTTGGGGTTTTCAAATGTTGTGGGGGATTTCTTCTTCACTCCTCTTCTTCTTTTTTTTTCTACTTTTACGTTTGGAAGCTCGGCGATACAGAACGCAGAACTTTTCCTTTTTTTTTTTGGTTTAatgcttattattggtactgctgctgctgttgtcgctgttgttgttcattctagtcaacatcctctctcaatcggccctttcccgtcctttccatgttattttcattatttccaccgcagtcgccaccatcgtgttttcgattccccatccctctttctctctttctttcctttcattttttgttattattactgttttcattgttattgtctctgttgctggcccttattaatcgcaattgttactcttcttttcacctccatccccctctttttgtgtgttgtgtgtttgtatgtgtttggctgttgtaatttgtttgtttgttcttttttcacctccctccttccaaccccgcatcttatatttcgggtgtcgggactgcaacttcttgaaaatacctttttttttaattttttacgtttgtcgtcgttcttttgcctcccttttttttttcctcacttcggtgatacttcctcccttccttccctttctttttccctttcttttttgtttttgtatgtgtcttatatcccactttttttcttttttgagcagcaaaataaaaaaagaaacacataaACACACACACACACACACATATATATATATATATATATATATAATGATAATAATAACAACGGAAACGATGATGGTGATAATATGTATAGGTGTGGATGTATATGAGGATGTATGCGTACATATATATATATATATATATATGCACGGGGGGTTCTGAAAAGGATATAAAAAAAAACGTAGAGGCGTCATAATTTGAGTTTACTTTTTTCTGTTTTTTGAAAGGGTTTCGTCTTATTGTTTCGTACTTTTTAAAATATCATGGTAATATGGGTAAGGAGGAAGTTTTTTTTTTTGTTCAAAAATTAACATTATGTCCGACGGTGTGTGGTTTGTGGTGCGATTGTTGTCTGTTTCGTCCGCGGTGTGCGATTTGGGGTTTGAGGGAAGCAAAAAAAGAAAAGATTTGAAATAAAGGAGGGTGTAATTTCCCACTATGGTGACGTTATTGTTGTGGGTTTTATCAGATGATTGTTTATTATTACCATTATTAGTATTACTATTACTACTACTTTCCACTGTTTCGCTTTTCTTTTTTTTCCTTTTTCCTTTTTTTTAAAAATTCTATTCCCCTCCCTTGCATCAGTTTCGatgattaaacacttacgttttgatcacaaattttgtgtgaagcttcctctctttgactttagtttttttttgtttttggcgctcccatttgatttgatctctctttttaagtttcggtttcccactctgtcaccttctcccctccctttaaatcctcactttagtcttttgttgtattcgggtatccatgttgcgctacgcccagctgtttggaatattacttcggaagatgaaaagttcggtttatttatttatttattatttttttttacgttctttctttctttcttcgacatgtttgttttttcctattctcgaaatcaaaggcatcaccaacaccaccaccaccagcagaattaaGTGAACACATCCGCGAGTTACTGCCATGTGTTAATCCCTTTGCCTTTCTACCCTTCGATGATTTTTAAAAAAAAAATTTCCCTTTCGTTTCATGTTTTTCATCTATATTTTCTTATTCATCCCCTTTCTTTCTTTCTTTTTTCTTTCTTTTTTTCATCCCTTTACATATATAAATATATAAATATATATATATATATATATATATATGTTGAGATGTCTTCTTCCCTCGTATCCTCTGTTGCCCTCATTTTACGCGTCCAGAAACTTCCATCATGAGGTGTGACTGCTTTCTTTCTCCTTTTTGGTGTTAATATGTCTTCAATTCCCTTATAACTTATTGTAATGTTAATTTACCATTCAAAAGGGTTGAAAGATAGAGGGAATGTGTTTTGTTTCTGTGATATCCTGCTTTAAATTTTTGTCTTTTAATATCTTATTAAGCTTTCTACTCTCTCTCTTTTTCTTTTTTTTCTTTTTGTCCCCCTCCTTTAAAGTTTTTTCTCGCATATTTTCTCTTGTCCCGTCATCTTTTTGTGCTGTGGTTGTTGGGTGCCGCACATTTTTATGCATACCTCGTTGATGCGGTGATGAAATGCTGACTTTTTTTTTTTAAAAAAAAGAATCTATCGCGAAGGAGGCTCGATATGTTTGTTGGTTCATCTGTGTGTAAACACATAAATATGTCTTTTTATGCatgattgccatcaatttgttcgtttctttgtttgctcggtttgttttcgttactttttgttgttcctcatcaacccgtgttccctccttccgtgaaggtggttttaatctgtttgtgaatccgttttgtttctgtgtgtttgtgtgtctttgttgttgttgttgttgttgttgttgttttgttttgttttgtttttttgtgtcatctcacttcatctccttccccattttttgtctttccattatgcatgtgtacttacattgcgtattcttttttttttccccctcttccttcttctcttcagctctcgtactttttgtcttatttcctctttccttcgctacattctgttttggggtctgcagctgccaaacatatatatatatatatgttttatttatttaaATGGTGTTACATTTCCCGCTGCTTCTTTACGAGCATTCAAAACTCTTCTCTTTTTCTTCTCCCTCTCTTCTCCACTTAATCCGTCTATAAATCTGCTTCTTCATGTATTTAGGCGTTTACCGGCTTCCCTTCAGTGGTTGCACCTCCTTACCCCACCAAAAAAAAAAAATGATTTTGGGATCGGAGAGGTTTAAAAGGTTGAAGGGAGACGGTGGGAAGAGGAAAGAGGAAGACAAACAGACAAACAAACAAATGCTCAAATATATAAAGAAAAGTACAAGGGACAAAATGTTAGTAGCGGTGGTGGCACGAGGAGATAATAAAAGAGGGGAAACCCTTCCGAATCGGAGATACGAGATGGAAATTAGGAAGTGCCACACACGCCGGAGAGCATCAGCGTAACAAAACGTGATGTCACTAATGCAGTAAGCAGGAATTAACCAGCCAAGCAGCCAGGACTGAATTACCAGAGGAAGTCCCAATAACAACAAAAGGAAATTAAAACATTTGGTCATATATATACGCTTGTTTGTTGTATCTACTTCCCTCAGCTGGCGCTTCCTTTTTTTTTTTGGTGCGTGTGTGTGTTTTTGGATAGTCTCGTGAGCAGGTTTGTGAAA

>Tb927.11.14070

ATGGTCAACATTAATAGGAGCTTGCAACATTATTCCCATGTGTAGAACCACTATTTTTTTCCTTTTTTTTTTTGGACGTTACCTTTTCACTTTAGAGAGTTGTTTTTCCCCTTTTTGGGAAACCGTACTGTTATTTATAATGACGTGTTCGGATATTTCATTAGGATGTTACGATGTTATTGTGTGTTGGTTTTTCACGTAGTAATTATGATAATCGGTGGTCTGCTGTTCGTCGGCGGCGTTTGTGAAGGTGACTTTCTTATCCTTATTCCTCTCTTTTTTTTCTTTTTTTAAAAAACCTTTTTTTACATTCCGACGTTCGTTCGTTTGCTTGGTTGAGTTTTTTTTTTTAAATTGTTATTCCCCTCCCCTCCTCTCCACACTTGTTGTTGTTGTTGTTGTTGTTCTTTTAGCGTCAGTGGTGCTTTGGTCCTTTACTTTATGTGAAGACGTTTTCTTTTCTTTTTTTTTTTTCGTGCTCATATAAATAAACAAATATTTGCTCTGTTTTACGTATTTCATTCCGTAATTGACTGCAACGATTTTTTCTTTTTTTTTTCTTTTTTACCCTCTCTCCCTCCTTTTACTTCTAAAGTTCAAATGTAGAGATGGAATGTATGCCGCTACGCCCGCTTTCCCTTTTTCTTTTTTTCTCATTTCTCTCTGCATGGCAAAACTCTTTTCCCACGTTTGTTTTCGTTTTTTTTTTTTCCTGGCCCCATTTGGATTTATACGAAATTAAATAAATAAATGTAAAAGATCATGCATTATAACTTATTTTTCTGAGCACTTTTTATGTGGTGTTTTGTTTTGTTTTGTTTTTTTTTTTCAGTTGAAAATAACAAAAGAGGGAAATAAAGGGAGCCACCGCGCGCGCGGGTTGCGGTATTTTAGGTATTAGTATTGTGTTGTGCGGAGGGGGAGGGGTGAGAGGGAAATAATAACTTTTATCATATGACTGTACATAAATGCGTGTTTGTATTTTATGTTTTATATTTAAGTTTTCAAATGTTTTATCGTTGCGCCCACTGGTCCTGTACGTGGGCTCACACGCCATAACCCTTCCGTTGTTTGTTGTTTGTTTTTTTTTTTTTACATTTTTAGTTGTTTTGTTTGGACCAAATATGTAAATATATTTGTCCTCTATATTTTTCACGGTTCCTGTCCGCGCCACCAGTTGCTTTTTGTACGGTTAATCCACATTTGTGTAAAAGCACTGCAATCCCTCTCTTTTTGTTTATTATTATTATTATTTTTTTTTTTTCAAAGAGGAAAAGCGCGCGTGACGGGTTAAATATCGGAACGTCTATTATATATATTTATATATATTCCCGCTTTTTTTTCTTTTCACAATCTTGTTGTCTCCTTGCATATTGAATAGGTATTTTCCCTCGTGTATAACTGCACTTATATATGCTTTAGAGGGACAGGTACAGGGACAGAAGGGAAAGAAAAAAAAAAAAGAGAACGAAAGAAAGAATAACTCATTGCTATTTAAAAAAAAAAAA

>Tb927.9.13430

AAGAATGATAATGATGGAGGGGTTGGGAAGAAGGGGGAGACATTTGTGTGTGGTTGGGTGAATTGTGTTTTTGTATTTAATGTTTCTGTTACTGTTTGTGTTAGTTTGCGTTACTTTGCTGCTTCCCCCCTCCCTTAAATGTCGTTGTCGGCTATTTCAATTTGTTAAGAAGAGGTTTGGTGGGTGTTTTGTATGCGTATGTTTATGTTTGTGTTTTCAATTCTTCGTTTTCATATCACACGTTTGTCTGATTTCGTGTCGTAGTGGAAGAGAAAGTGATTTAACGTCCGTCTTGGCTATTTTGTGCGACGTGTACGCATTTTACCTTCTTTAACTTTTTCTCTTTATCTTTTTTTTCTCTTTTCCGTTGTAGCAGTTTCTTTTTTTTTTTTTGGCTTCATGATGTTCTTTTTTTTTTTTGTTAAAAAATTTTTTTATCTTTCGTTTTATCTTTTGGTAAAACAAAAAAAAATCGTTTCTAAAGAGAGAAACAATAAAAGCAAGACATTAATATCCTCTGTTTGTTTATTCCTGTGTCTTACATGCCACCGAAAAAAAAAAAATAAAAAATAAAATGGGATTAGTGGAGTATCGAACCAATGAATAGTGTTAGATTCATTTCACTTTTTTTTCTTCTCTTTTTCTTTAAGAGGAGAAATTAGGGGGAATAAAAATCTAAAAGCGGAGAAATGGAATATGAATAAATGAAACAAATGAAGAGGTACAAAAAAAAGAAAAAAAATTATAAGGATGAAGTTGTTGGAGTGGAAGAGGGGATTTAAAGGTAGATGAGATGGTTTTTATCCGCTTGAGTTTGTGTGTTCTTTTTTTTAAAAAAAATAAAATAAAATAATAAAATGAGAGATAAAATCAAATAAGGAGGAGGGGGAAAGAATTTTTTTTGTTGATTGATTTCTATTTTGGTGCTAGTGCTGCTGCTGATTCTGAAGATGATGTGTTTTTTTGTGTGTGCGTTTGTGTGTGCGGGAAATGAAGCAGCAGAAGTGAAGGAAGAAGCAAAAAGAAAAACAAAAGTGAGGAAGCAAAGAGAAATGAGTGAAGAAAAGGTAAGTAATAAAGAAAAGAAGAAAATTGTGATTCTTTTTTTTTCCTTCCCTTTTCTTTAATTGATTATTTATTAGTGCGTTTATTAATATTAATATTTTGTTTTCTTATTTTATTTTATTTTATTTTTTTTGCATAGAATAGCACGGAGTTATTCATTTTTTATTCTTTCTTTCTTTTTCATCTTCCCTCGCGTATGCTCTTTGTTGTCCTTTCATTATTGTCTCTTTTTCTTTTTCTTCCAATAGTAATAATAATAATAATAATTATTATTATTACTATTGTATTGTTATTACCGCTCCTTTTTCTATCCCCCTCCCATTACTCGTCACTCATTCATTTTTTTCTTTTTTTTTTACTCAGTTGTTTTCGCTCATTGATTTTTGCACATATATACACAGTCACATGCCATTACTACCCGTTCATTCGCATTATATATTATATATTATATTCCATATGTTATATGTAATTGGGTTATCCTTTTGCCACTGAATGAATGAATGAATTGGGTGGTTTATTTGTTTAATTTCTTTTTTTTTTTTAAAAAAAGAATTAAGCTTCACTTTTATTTTTATTTTTGCTTTCACTTTTTGTTTATCTTTTTTTTTTCTTTCTTCCCCCTCTCTCCTGCTTGACGGTATGTATATATATATATATATATATATATATGTGTGTGTGTGTGTGTGTATGTGTGAGAAAGGGAGAGAAAAAGAACAACTGGGTATAAATGGAAGAAATGGGGATGACGTTAACGCGTTTTCATATGAGGGCTTTTTTTTTGTTATAACTTTATGAAAATAAGTAAATAAATAATAATTTAATATGTAAATATATATGTTTATATATTTACATGTTTGTGTACGAAATGAATTGAGCTGAGGCAAATGAATGAGAAGGAGGCGATTTTTTTTTTTTTTGTGGGGGGTTGGTGTGTGGGCGTGTGTGTATGGTGGGGGTGAGGAAGCATAGGAAAAGCTAAGGAAAAAAATATATAGCAAAGGTTTGTAAAAAGAGAATAAACTTACATACATACAAGCACAAACACACACACATAAACACACATAAATAAAAAGTGCTCCTGCACGAGTTAGTTTGCCCTTTGTGGACGGAATGAATTTTACGTTCCTTTTTTCTTCTTCTGTTTTTATGTTTCAACTTTTTAATTTCTTCCTTTCTCGGTACCACATTCATATTCATATTCACATTCACATTTATATGCCTCTGTTTATTGTTATGTATAATTACCTTTCTCTTTCCCCCATTTTTTAAATATCATATCATATTTTGCCACTTTTTGCTTTAACATTTATTCCCCCCTCCCCTCCCCTCCTCCTCATTCTTCTTTTTTTTCCTTTTAAAAACTCCGTCTCTTTCAACAAATTTTCTCACGTTTGTGACTTCATTTTTGTTTTTGTTTTGTCTTCCTCTTCTCGCGTTGGGCATTTCCATTTTTTCTTTTCTTCGTATTTTGGTCCGTTTTGTTTTGTTTTGTTCTGCTGTGATGTGCTGTGCTTTGTTCTGTTCTGGCTGTGTTTTGTTGTTGTTATTTGTGTGTGTGTATATATATGTGTGGGAAGAGAAAAAAAAAAAACGGTTTCTCCCTTCTCTTAACTTCTACGTATTTCCCTTCAGAGAGATAGGGATACAAAATGATAATAAGTAATAAGTAATAATAATAATAATATTAAAAAAAAAACAATATTATACGGTAAAAAGTATAAAATATAAATATAATGAACAGAACAAATCAGCATATAAGGGGGAGTGCGGGACAGAGAAGAAGAGAGGAGTGGAGGTGTGGCTTAACTTCACATTTCTCCCCCTCTTCCCCCCTTCATGAAGAGTAAGTAACACAAACATATATATATATATATATATATATATTTATATTTATATTTAAATAAATATATAAATATATATAAAAAAATATATATGTACATTTGTATATTTTTATTTGAGAATATTGTATATAAGTGTGGTTGCAACCCGTTTTTCTTTTGTTTTTTTTCTACCTCTACTTTCTCTTCTCTTCAGTATTTTAGATATTTCATTTTGTTTGACTGATGGATGTAAAATTGTTGTTCCTATACTTCTATATTTATTCGGAGGCGTAATTTCGTTTTTGTTATGATATACAGTAATTAGTAAAAAAAAGGCGCTTCTGATAAATAATTAATAATTGTGTTCTGTTGTACTTATGTTTACGCGACTCCTTGCGGGGTGTAGAATTTTCTTTTTTTCCCCCTCCGATCAGTATTATTGTTTCACAAAAAAATATATATATATATCTTCTTCTTTTTATCAATTCCCGTTGTTTCATTGATGATTATGTTTTGATGATATTCTTGTTTGCCCTGGTGATTTGTCGTTTTCGTTGGCTATTGACGGTGATAACAAAATTCTTATCTATTGTTGTTTCGTTTTATCAAATAATTAATTTTTTTTATAAAAAAAA

>Tb927.7.5380

AAGCCACCGTTCCTTTTGATTCCTTTTATTATATTATATTTCGTTTTGTTTTTGTTTTTCACATCAGTGCCTTTGCTTACTATCTTCGGTATTTTCCTCTCAGCTCCGCCTCTTTTTTTCTTTTTTTCCTTTTTTTCCCCGTTTCTGTTTCTGTTTCGGCTCTTTCTTTTATGTTTAAAAAAAAAAAGACCATTTGCTCTTAATAATGACAATTGTTGTTATTATTTGTCTTTATTTCCGTTGTATCGCCTTTTGTATTCCTGTGTTTATGACAACCGAAACCGTTTCAGCGGAAGGGTGAGATAAAAAAAAAGGGAGGGGAAATATGCGGGATCCCTCAATAGAAAACAGAAAAAAGGAAAGGAAAGGAAAGGAAGCAGATAAGAATGTGGAAGATTAAGGAAGGAGGGGGGGGAGATATAATGGAGGTGTGGGGGAACAAATGGAAGGGTTTTCTTTACTTTTAAAAAACAAAAGAAAAGAAAACAAAAAAAGATGGAAAAGGGACAAAAAAAGAAAAAAAGAAATACTGCTCGAGAGGAAGAGGAGAAGAACTAACACACACACACACACACACATACATATATACATACATACATATATATATATATATAGATTACACGGAAAAAAAAAGAAGAAAGGAGCGGCATATTTGGTTTTTATCAATCAGTTGCCGTGTGCTGTGGATTAATAAGCGGGTGCGATCGCGTGTGTCGGAAAAAAAATGACAATGCATTAGTCCTTCCGTTGCAATATATAAATATGTATATATGTATATATGTATATGTGTATATTTATGTATATATATACATATTTATTTGGATGTTTTACATATTTGTATGTTCCGCTTATATAAATAAATATATATATATACACATATTTAATTATTTTATTTTATTGTTTGTTCATTGACTTACCTGCGCGCACATGTCCCCGAGTGTCCGTTTGCCTGTCTCCATTTACATGTGTACTTTTATGTGTTTAAATATATATATATATATATATATATATATATATATGTATATTTGTGTGTATGTGTATGTGTATGTGTGTGTGTGTGTGTATGGCTTCCCTTCTTTCTTTTATTATTGTTATTGTCAAATGTGCAATCTGGTACCTATCTTCTTCCTGTTTTTATTCCCCGATGGGTTGCCGTTGTTTTCTTTTTTCTTTTTCTTTTTTTTACTCCACTTGACATTATTTTATTTATTATTTTATGTCCTGCTCCTTTTTTTGTTTGTTTTCCTCTTATTTCTCCTCCATCCCTTCTTTTTTTTATTTTACTGCATTTTCTTTTCTAATTTATTTTATTATATTAAAAATACTTTCGCCATGTCCCTCTATTTCGTATATTACCCGACTCTTTCTTTCATGTGGTTGTATCGCAATATATATTGCTATTGCTATTACTATTCCTCCTACTATTGTTATTGTTATCTTTTATTATTTTCAAAAAAAGAATTTTTCTGTTCAATTTCTAGTTTATGTATCACTTTTTATTTGTTTGTTTATCGCCTTTGCGTTAAATAATAAATTTTATGAAAACAAAGAAGAAAGGGAAACAATCCGAAACAAAGTCTCGTTCCACACATTTTGATTCTTTTTTTTAATTGTTCCTTCTTTTTTTTTTTTTAGATCTTCCTGTTTCAAACCCTTCATTTTCCTTTTTGTTTTATTTTTATTCTCACTTCTTTACTGCAACTTTTATTTATTTATTTTGTCCTCATTTTTTTTTTTATTTCCCCCCCTCTTCCTCACTAATTTCTTCTCACCACAAAATACACGTCAGTAATCGTTGTGTTTGAAATCATATACTTAAAATATATATATATATATATATTTTTTTTTCTCTCCATATATTTCTCCTTCATTCAACTTTTTTTTGTCCGCTTACCTTTTTTATCCCTTCCTTTCTTCTTTAAAAAAAATAATAATTAATTATTTTAATTTAAATAACTTTTTAAAGGGGTAGAAACCTTCGCAGTGGTCCTTTTTTTTGTTTTTAATTTTTATTTCTTTCCACCTGAATTGTCTATTTTCCTTTTTGAGTTTAACGTTTATTATTATCATTATTATTTCCTTCTGTTTTTCATTGTTATTATTTTTTTTAATTTTTATTTACTTCGTAACAAAATAAATAAAAATCAGAGAGGAAAAAAAGGAAGTCCTCCCTCTTTCTTTTCTTTTCTTTTTTTTCCCCCCTCTTCCCCCTTCAACTCATCACTCTGCTTTCTCTTCAACAACCACATAAGTAGAAATTTTTTTTAAAATAAAAAGCTGAAGGATCAAAGTTTATATATTCCTTTCGGTTTTATTTTTCCTATTTTTCCAATTTGTATTGATTATTAAATTCTTAAATTATTACTGTTATTATTTGTTGTTATTATTATTATTTTATTTGTTATTTCTCCTTATTTATTGTGTTATTTTAAAATTTTCTTCATAATTTTTGTGTTTTTCATATTCCTTCCTTCCTTCCATTCTTTCTTTCTTTCTTTAATACCTTTATTTGTAATCATATTATTATATTATTATTATTTTGATCAACTCCATTATTTGTTTACTGCGGCTACCACACTTTCTGCTAAAAGTTTATTACAATAATAGTAATAAATGTTGTTATTTTTATTTGTTGTTGTGATTTTACGTCTCTTGGTTCAAGAGGAGCTTAAATTTTTTTTGTCGCATGATTGTGAACAAATATATGCAATTGATATAAATAAATATATATTTTTTTACATGTATGTATTTATATATGCATTTCCTGATGTGCTTATATCATTTATTCTATTGTCATGATTTCATCACTATTAAAATTGTGTGCCTGTGCCTTTCTCTCTCTCTGTCTGTCTGTGTGTTATGGTTATGGTTAGGGGGAGGCACCTTTCTTTCTTTCTTATCTTTTTTTTTTTAACCTCCCCCTTTTTAAAATTAATTAATTAATTAATTAATTTTTATTTAGTTATTTATATGTCTTTTTCTTCTCTTCCACCACCTGCTCCTCCTCCTCTATTTTTTTTAATAAAAATATATATATTTACTATCTTTTTTTATTAATTGTTATTCTTCCATTAAATATATAAATAAATATTTATGTATCTCTTTTTTATGTATTAAATTTTCTTTACTTCCATTTTCGTTCCAGTCAGTTAAACTTGTTCCCGTTTATTTAACTAAATCCCCCCCCCCCACAAAAAAAAAAGAAAAAGAGGGAATATACAACATTAATATATAAATGCGTGCGATATAGGTGGATCTTATTAGCCTTTTGTTTTTTTTTAAAAAATCCGTCTCTTGTATTTCATTACTTTTGTTGGTATATATTTTAACCAATTGAGGACACAACAGCTATGATAATGATGGTAATAATAAAAATATGTTGTTGTTACGATGATAATAATAATAATAATAAATATTATCATTATTATTATTGCTATTGTGTCATATCATATCAGGGTTTCATTTATTTATAAATAAATATATATTGTTATTATATGATCGTGTGTGTTTGTGTTTTGACGTGGCTGTAGAGAAGGGGAGATTTATATTCAACTTTTTATTTGTTTTACATTTTCTTCGTTTTGTATGTATTTAAAAGAAGTAAAAATAAATAAATTAATATATATATATATATATATATTAAAAATCTTAAGTGTCCTTCTGAGCTAAAGCTGTTGCGTGTTTATAAGAAAATGAAAAGAAAAAAAAGAAAAACGAAAGGGATAAATAGATAAAGGATCGGTGTAATAATTTTTGTTATTTGTTTATTTATATTTCCTCCCTCTTCCCTTTTATCTGTTTTTTTTCCGCATTTTCTTCGGGATTCGCTTCATCATTTATTTTGTTTATATATTTCTTTTCTTTTTTCTTTATCTTTTTCATTCGTAATTGTTATATTTATTCCCTTTTTTCCTTTTCTATATTTTCACCCCACTTTTATTATGATGTACATTTATATGCGTATCAAATCTGTCGATTTTATTTTATTTTATTGTTTTACTATTTTCTTTTTCTTTTGTTTCTTTTTGTTTTTTATTTTTATTTTATTATTATTATCATCATGTTGTGATGACTGTTTTTCTGTCCTCTCCTTTCAATTTACCATTTCTGTCTTTTTTTTATATGTTGTTCCTATTATTTATGTCTTTTTTAAAAAAAAAAATAATTTTGGAGGAGGTCGTTGTTATTATTGTTAATACTACTTTTATTATTGTTGGTAACTATGATAATGAGGATTGTATTATTATTATTATTATCGTTGTTATTTTCCAGCTACAATTGGGGTTGAAACGTGAGAGATGGAAAAAGAAACAATAGAAACAAAAGAGTCAAAACGAAAAAGAGTAGAAAAGAAAAAAAGAAAAGAAATAATAATAATAATAATACAATATTGATGACAATAAAATATATAATGAATAGAAAATAAGGGAGATATGAGGCTAAAAACCCGTAGAAAAGTCACTTGGAAATATAAAACAATGAATATTTTCCAGTATGTTAAGCGAACCCGTGAACAAAATGAAAGTCAAATATCGGTAGCTATTTAAAAAGGGGGAAAAACAACAACGGCCGGAACAAATACGTAAAGAAGAGTTTATCAATCACACTTTGCACGTGTAGTGAAACTCGTTCAAATTGATTAATAGACTAATTAATTGATTAAATAATTAAAAAATCATTCAAATCATTCTTACCAACGCATCTTCCCACATTTATATAACTATATATTTTTCAGTATTGATGTATTTTTTTCTTTTATATTTCGTAACTGTCTTTTTTTTTCCGCTTTTTTTAAAAAGAAAGAAAAATGCCTACTATGCTGCTTCGTATTTTATATTAGGCTTTTTTGACCATTTCAACCCCGACATTTACCATCAATAAAGATGGTAATAATGAAATATATATATATACATATATAACTACGGGAGGCCTTTTATTAAATTACTATTATCGTTTTTGTTTTTGTATTGTAGACCTTCTGCCTTGAAGAAAACAAGGCATGGGTGTAGAGAGGGGAAAAAAAACTAGATGTACGAAGATAAAAAGCTTTCTCATTTACTCTCTTCTTTTTTGTGTGTTTTATTAACGTCTGTTCTTAATTTTTTTTTCTTCTTATTTCTTAATTTTATTTGTTTATATTTTACTGATGTTGTGGGAAATGCGGAAAGGAGCTACTCACTTTTTTTCTTCCACTGAAGGGGAAAAGGAAGAAAATTTAACATATATATTTTTCATAACTAAATGATAAAAGGGATTCGAGCCGGAGAAATATAAAAAAAAATGGGGAAAGAAGAAAAGAGAGAGATATGGAGTTAAATAGGTTTGTTACGTCTGAAAAACCTTTTGTGTATAAGTAAATAATATTAATATATATATTAATATTATATTATATATATGTTTGGTATTTAAATTTCTTATAAGGGGAAAACGATTATACGTAATGCACATATAAGTATTTACAATTATATATATTTACATATTCATACGCCTATACATTCACGTATACACACTTTTTTGATCACTTATATTTATATTCACTTATATTAATTTGATTAGCATTGGTATATATGTATGACTGTACTTGTAGACCTCTGTTTGATACATTTCATATGGAAGAAAGGAGAAGAAGGGGGAGGGAAATGAAAAAAAGAAAATGAAAGAGAAAATGGCTCTCTACTGGGATAAAAACAGGAAAATAAAATTAGGGGGGAGAAGGTGCTTGCAAAGGGAAAACGACTCTTTTTTTGTTTTGTTTTTTTATTTTTATTTTTTGTTATTTATTTGTACGCGTCTGTTTCCGGCCTAAGTTGCGCACGGGGAGTTCGGGGGGCTGTTTATTATTATTATTATTATTTTCCCGGTTTCCACCGCTTTTTGTCGTTAGTGTTTATGCTTTTGTGTTTCGGGGGAGCAACGCACAACTGTCACTTTGTCTTCGACGTGTCCTTTTGTGCATGCTTTAAAAAAATTTTTTATTTACTTGTTGTGAGCCTTGGAGGGAGGGAAGTTGCTTCACTGTTCCTTTTCTATTATTTTTTCCTTATTTTTATTTTGGTCTCTCATCATTTTTAATGGGGGTTTTGTTTTAACTGACTGTTGTTATTTTTTTTCCCTAACTGTCTTCAATGTTTGAGCGACTGTTATAAATCTTGACTTTTGATTATTTTTATCTTTTATTATTTTATTATTTTGTTTGTTTTCCTTTTTTTTTAAAAAAACCTTTTCTCTTTCTGCGTGCGTGAGTATGTGTCTGTTTCCAGACTGACCACTTTCAAAAA

Tb927.11.1580

AGAGGGCGTATACATATATATATATATATATATATATTTGTCAAATGGAAGCGCTAGTGGTGCATTTTTTTTTTTAAAGATGCCATAGATGGTGACTTATTCTTTTATAACATTGCTAGTACTTCAGCTGAGTATTTGGTTGTTGTTTATTTACTTATTTTTTGCTTTTTTTTTTGATGTTGCGGCGGTGAAGTGTGTATGCCGTGTGTTTTAGGCGCACTCATTTTTTTTTTACCTTTTAAATGTCATTGGGTGCACTCTATCCTCGTGACCTCACGCATTACTTTGTTTTATCTTTTTGGTTTTATTGAAGTGTGTGGGAGGAGGGTAATTTTCTCATACAGTGTGATAGTTGAACAATGGGTCTTTTTTGTGTAAAA

>Tb927.10.1110

GGGGGAAAAGACGGAAATCGTTTCATAATTGGGCTGATAGTCTTTTCCCTTTTGCTTTTATTTTTTTGAGTTATTTGTTTTTTATTAAATTTAATCTCAATTTGTCTAGTATATATATATATATATATATATATATTGTTGTTGTTTTTTTCTTTAGTTCTATTCCTTGCTTTGCTTTTCCGTGGAACTGCACATATCATCCCTCGCTGGTTTTCCACTGGAAAATCCAAGAGGAAGACGTTACAGCGGTTGCGGGTGCCCGCCGCACATTTCGTTGTACAGCGAGTGGTGACATCTGTGATGTAGTCACTTTTTACGTCACGAAAAAAAGCTGACGATTGAGTGCATGATATTTGTAGTGACTACCGGGAAAAGTGTATAGCGGCAGGAGAATTAAAAAAAAAAAGCGTGGATGTATGTTAAGCGACATGCGGACCTTTGCGCAAGCTCAACTCTACGGCTGTCATTGTTTTGTTACTATCACTCTCGGTAAAAAAAAATTTAAGTGTTTAGGTTAATGTGTGTATGTGTTTGGGGAGCGGGGTTAGGAGAGGTCAATTGCTTCTCTGTTTGATCCTGGCATGACACACTGCTTCAAGTGTGTCGTTGCTCTTTCTTGCCTTTTTGTGGGGGTAAATAAATTTTATTCCTTTTTTTCCCTCTCAAATGTTGTTATTGTGGGGAGAAATATTTTTTGTGAGTTTGTCCCTCCCTGTTTCTTTATCATTCCGACTGCGATGTCCCGCGTTGCATCGTCTTGTTTTGACTTTTCTCACCTTTCTGGGATGCATCCGACCTTTTTTTTTTCTACTGGATATTCCTCTATTGTATTAATAATGCCATTAAAGGTTCGCTTTCCCTATCCCACGAGGAAAGGAAGGGAAAAAAGTGTGTGTGGGGGGGGGAGGGGAAAATCTTGAGATGGAAGTAACAATGCGTGGAGGAGCGAACCGAGTACACCTATCGAGCGAAGCGCTTTGGCAACTGTTACTTTCCTCCATATTTCTACTTCATAAGGTACTGCAGTAGTGTGTAGTTAAGGGTTGTAATGGTGTTATCGTTTTTTATTGCATTTTTAGATGTCACGCACGTGTGAAGGGGAAAACGAAAGGGGATGCATGGGTTGCTCTAGTTTATTCTTTTGATCAGTTCCATTTCTATATTTGTTCGCTTGTTTTCCTTATTATTATTTTTAAAA

>Tb927.9.4560

ACCTGGCACGTCTCTTCTATGCTCCCCCCTCATCTGTTATTCATTCACGCGCTAATGATCAATTGCGCATCTCACCTTCTTATCACTTTATGACTACCAGTGACACACGACTTTTGACTTTTTTGCAGGAATCGGGTAAACGCTTTGGTCGGATTGCGTAGTCTCCGTTCCGCTTGGCAGAGCCTTAGATGAGGAGCGGGACAGAAAACAGAAATGGAAGATAAGACAGATAAAGCTTCTCAAGTTGAAGCACGGGATTATTTTATTTTATTATTTTTTTTTTAATCCACTTATTATGATTGTTATTTATTTTTGTTTTTCGCTTTGACTTTCACCCTGCACCAATTTCCCCCTTCCGTTGTTTATCCGAACGATGGGTTTCATGGGACTATTTTGCATCTATTTATTTTTTGATTCCCTCTCTCGTTTATGACACATTTTTTTCTTTATTTAGCCCCTACCCCGAGCATTCTCTCTATTTAATTTCTGCTTTTAAACTTTTGGTTTCAGTAAACGTAACACCATTATGGTGGACGCTATTTGATTTGTTTCAACTGCGTCCTATCCTCTCCTCTCCCAATTTTTATTTTTTATATGTTTTAAAGATATTACGCCCTTACTTATTTTTGTTTCTTTTATTTGTTTCTTTGCTTTAACCCGAAGCACAAACAGCTCTGCGTAAATGACGCTTTGACTGTTGTCACTTGCAGAAAGCAGTAACGACGTCAAACGTGCGGGTTGGCAAGCAGAAAAGGATTTTTCGACCTCCAGAAGGCGAAGTGCCCGCAACAATACGAAGCTGTCTCATTCATTTTTTCTTTGGGTTTTATTAATTGGGTGCCTCACCTGGTATCCCCTTTCTGCAACATATACAGATGAATATAAAGATAAATAAATAAA

>Tb927.8.7840

ACCGCATCAAACGGTGAAACGGGGTTGAAACCAGCAGCAATGGAAGGGAAGTTCCATATACTCTGGAGGTTTATAGTTGATATCCTTGTGTGTACAAAAGCTGACAAAATTGAAGGAAGGGGGAAAGATGTAAAAAGAAAAAAAAAAGGAAAAGAAGGGAGCTCATAATTCTGTTGCATGCGTTTTTGCAGCTGCATGACTCGGTGGCAGGAATAACGTCACAAATTATGTTACGGGAGTGGGAGGCGGCCACGACAGAAACCGTAAGTTGCCGACACACATTCACACATTTAGTGGTCACGTGTGTCCGCGCGGCTGTTCCTTCCTTCCTTCCTTTCCTTTCTTTTCTCTTTTTTGTTTTTCCTTCCCCATTGGTGGTCTCATTCTTTTTTACTTTTCTTTCTAATATATATATATATATATATATATATTTATTTATTTTTTTGTTTTTGTCTTTTCATCATTCTATCGTGAACAGTTCCATAAGCAAACCAGCAGAGTGAAGTTCTTTTTTTTTTTTCTGTGGGAGTACATTGCCAGTGAGCTTGCGCGTGTAGGGGGAGGAGGGGGGCGCTATGAGCCGTACGACATCTGTGGAAATACCGCTTGAGGAATAACATATGCTCACGTGTACGCATGTGTGGGATTCATTGAAGACAATTCTAATGTTTCTTTCACTTTTTTTTTCTTCTTTCTTTCTTTCTCAGAAAATTCTTTTTAGACCCTCCATTTTTGTTTTGTTTTGTTTTTACCTACCTACCTACCTACATACCGCTTCGCGCACGTATTCATATAACATTTGCCGTGTCGTTGATGTTTTGATTTCTTAACCCTTTTCATAGTTAACACCATTGTTATTGTTATTGTTATTATTATTTATTTTTATTTTTTTGACGCTCTTACTGTTGTTATTGCTGACTTTGCCTGTGGTTCTGTTGCCACCGCTCCTTTTCCTTAATATTTTTTTTTCCTCTTGTTTTTGAATCCACCGTTTAACATCAGTGTACAGGTGGAGAGGTGCGAAGGGGCCTTCTGTTACATTTCCCCACTTTTTTTTTCCCGTTTCAAAGCATGGTGGATGTAACAGGTTCCTTCTTACATCTTCTTTTTTTTTTTTGAGAAAAAACAATCAACTAAACAAGTTAAATTGTAATAATGATAATAATAATAATAGTAACAATAATATTTATTTATTTTCCCCCTTTTCCCCTTTCCTCCCCCTCTTGTATTATTTTGCGCGTTTCATGCGTTTGCTTAACTTTCATTGAAGGGGAATTTCTGCACATCTGCGGAGGTGGGTCAGTGAATAGTTAATTAGATATTTCAAAGGGAAACTACGGGAAATAAGCGAATGAACCAAAGAAAGAAAAAGAAGGGGAAAAGGTGAGGGGGAGGGAGCAGTGGTAAAATAATAATAATAATAATATATGTACTTAATAAAAGGCAATGAGAAAGCGGAATCATGTCTGATATTCATTTGGGAAAGAAGGTGCGCGTGCATGTGTGCGTATAAATATTAATCTAAATAATAATGATGATAATAATGATAATAATTGTTATTAATGTCATTTTTTTTATGCTTTACTTCAGTGTATCGTCCCCACATCATTAATAGTAACAAATTATCACCGTTGTATTTGCTATTTTTTTTTTAAACTTAAAGCAGCTGAAGCACGATGCCGGTCTTACGGTAAAACTGTACATGCTATATTTTTAAGAAACGAAGAGAGAGAGAGAGAGAAAAAAAA

>Tb927.1.2160

AAATGAAAAAATATATATATAAAGAATATTTAAAAAAAAAAATAATAATGATAATATAATGATGATGATGATGATGATGATGATGATAAAGAGAAAGAGAAAGAGAAAGGGAAAAGAAAAAGAGAGATTAAAAAAAAAAAAAGAACATCCAATGAAATGTGTGTAAGAAGAAGAGGAAAAGGGGAGGAAAACAATGACAAACAAACAAACAAAAAAGGATGGGTTTTATTTTTTCAAAAAAAAATCAACACATATATGTATCTATATCTATATCTATATATATATATATATATATAAAGTGGTGTAATGCATACGTTTATATGATACAATGTGTGATATATTTGTTTTATTTATTGATATGTTTATTTGTGCATAGGGAAAGAAAGGGAGGGAGTATGTACGTTGACAAAATAGGTAGAGTTATGGTGTATCCATTTATGTATCTACGTATTTATTTATTTTTTCCCCCCTTTCTCTCTGTAGTTTGGAGGGAACAAAATAAAACAAATTTAAGAGCAAAGCAAAGCAAAGCAAAATAATATAATATAATATAGAGAGAGAAAGAAAGCAAATGAGGGAGGGATGAAGTTTCCCTGATGCAAGGTAATACAGCTTTGGATTTGTTATTTATTATCTGATTTATTTATTTATTCATGTTGTTTATTTGTTTATTTGTTTGTTTGTTTTCTTACGTATTTTGTTTGCAAGTTTGATTTATTTAGTGATTTATTGATTTATTTTATTCTGCTGGAGTTTGACCACATGATTATATGTGTCATTAATCTCCCCTCCCCCCCCCCCAAAAAAAAAAAAGAAAGAAAATAACTATTGTTATCCATTGTTTGTTTGTTTGTTTGTTTGTGCGGACGTATGTGGTATGTGTATGAGTGTGTGTAAAAGGGTATTGACGGAGTGTAAATAAATGAGAAAAAAAAAAGAAAAGAAAAGAAAAGGAAAGGAGTGAAATAATGACGGCATTATTGTCGTAGTAGGAGTGGTAGTAATAATAATAATGATAATAATAACAAATAATAATGATGATAATAATATTATTATTATTAATAGCAATCCCCATACTAATACCAATGCCAATACCAATTATTGTTACCATTATTGCTATTGCCGTCGTTTTTGTTACAATTATTATCACCGCCATGAAACAGCTCCCAAAAAAAAAAACGAGGAGGAGGGGGAAAAAAAAAAGAAAGGGAAGATATTAGTTTTATGGCAGTTGTGTCTTTCATTTTTTCTGCTTCTTTAGATGCCTTACTGAAATGAGTTGCATGCATGTGTTATCGCTATTATTATTTGTTATTTGTTATTTATTATTGCCATCGTTACCGGTCCACAGCGTTTCTCTGCAAACTTCAAACGCAAACACGCCAAACTTTGCATTGACGTGGTCACTCCTTACTTGTGCTTCTTTATTTTATTTTTCTCATTATACCTCCACTTATCTACATCTATATCTATATCTAAGTGTATATATATATATATATATATGTGTGTGTGTGTGTGTGTGTGCGTTTCTGTATTATGTAACTCCATACGTAGTTTTTGTTTCTGTTTTTTTTTTGTTTTTTGAGGGTGTAACTCATCCCCGTACACTTATACACGTTTCCTTTCTATTATATATAAATGAATATATATATATATATATATATGTGGGTAATATACGAAACCTTTCTCTCGACTTATTTCTTTCTACTGTCATTGTTGTCTATTTACGCGAACGCCTATTTAGCCTCCCTATTTCCATTAAATAAGTATTTATATTTATAGTATTTATTTACGTTTTGTTTGTTTCTATTCCTTTCCTTTCTTTTTTCTTTTTTAACTGTTATTATTATTGTTATTTTTATTTTTCCATTCTTGTATCTCGTGTGAGTGGCTTAACTTCGTATGGGTACTTGCAAAAAGAAAGGTATCGCCTCCATACAAACGCAAACGCAAACGCACGTCTTCCTTTTCTTTCTCATTTATTTACTTTTATTTTTATTTTTTGCCCCCCCCCGTGCAGTTTTGTTTTGTGTGTTTGTGCCTGCATTTTGTTACAGCATGATGTCACCATTATTTCCTCAGTGCATGAGATACTGAAGCGAAACAAAAACAAAAGAAAAAGAAAAAAGTGAACTGAAGAAAAAGAAAATAAAATAAGAGATAAAAGAAAAGAAGAAGGATATATATATATGTGTGTGTGTGTATGTGTGTGTCTGTTTTTGTTTGGAGGTGATACTTTTCACTCATTAAATGTATTTAAGTTTGTATGGGATTTAGTTACCGTAGGAGAAAGTGTGGTTTCCAGCTCGTAATTTCGACTCATATGATTACCACCATTGTTATTAATACTCTTATCATTATTATAACCTACCCATCGTGGCGTATTGTTTGCGCTTTCTCGGTACTGTGTTGGTGAATTTATTGTTTCTGTGTTACTTAACCTCAACTTTTCTTTTTTCTTCCTTCTCTTTCTTTCTTTCTCTTTCTCTCTCTCTCTCTTACCTCCTTTTTTTTTTTTTTGGAAGGTCTCTCTTTCACTTCCCTCCTTCAAGTTTCCCCTCTCTTCCCTTAAATTCCACACGTCCGTGTGTTCATTTATTTCTCTCGCGGAGCATTGGCGCCTCGCAGTGCGTCCGTAACTTTGCGTGGTGCTGCGTGTAAAACGCAATCGCTCACCCTCAAGGAGAGTAATAATAATAATAATTAAAATAATTAAAAAAAAAGAAAAAGAACAACAGGAACAGGAGCAAGAGCAAGAGCAATAAGATCAACAACAATAGGAAAGAGAGGGAGAGGAAACATCATACGAGGAAGAGAAGAAGGGGGAACTAACCAAAGCGAAACAAAAAGATAAAAAAAAGAAGAGAGAAGAGAAGAGAAGAGAAACAAAACCAAACAAAACAAAACAAAACAAAACAAGTAAGAAAGTAAGAACGGTTGTGAGCCTCAGTGAAGAAGGAATTATCACATCACTTTGAAGTGCATTAGGGGTATACGTTTAATAAGAATATAAGTATTAATTTATTTATATTTTGGTGCGGCGGAGAGATTAAACGGGGAGGGAAAGTAAAGTGCACTTCTCGTTTGCGTCTCTGTGTTTGAGCGGAAATAGGAGGCAAAAGGCAGACAGAAGCAAAGAAAGAAAAAAAAAAGAACAACAAAACAACAAACCTAAAACAGTCTGCGAGCCAGAGAAAATTTACGTAGCTTCGTTATTGTTGGGTTTCCATAGGGAAGACAGAAAGAAAGAGCTTCAAGAAAGCTAATAGGAAACAAAAAGTGAAGGGGTGGAAACTGTTCACGTATACAGAAATAAATAAATAAATATATATATAAGGCGCGGTGACCTTTTTTTTTCTTTTTTGAAGAAAGAGGAAGCGCGACGCGAGGTGTCTGTGACGAGGGATCGAAAACGTGGCGAGGGGAATTATTGAGACGCGTGCCGCAAGTGGACGGGAAAATATAGCGGCGGTTGAAAAAGAAGTAAAAAAAAAAGAAAAGAGAGGAAAATAGACGGGGAAAGGAGAGAGCAACAACCATTCGGCCTCTGTCGTAAGCCGCTTTGCGTGTGCGGTGGAAGGGAGAGAATTTCCTTTTTCTCTTACCTTGCCAACCCACACGCTGCGTGTATCGAATCTGAATCTTCTGTTTGGCGAAATCATTGTTGTTTTTTGTTTTTAGCATTTGCTGCTTTTGTTTCCTTTTCTCTTGACAGATTTGACTGGCGCGGACGCTGACGTCCACACGGTTGAGAGGGGGGAAAAAAA

>Tb927.11.2460 It looks from the RNASeq and ribosome profiling results as if this finishes in the middle of an unlikely annotated ORF, 2450. This is shown in loer case.

AGACGGGAGGGTGCAGGAGAGGTTCGTAAAATGCGTCACAAACGGGAATTATTTGTGGTGTAATAATGAAATAATATGATCCACTAACCTTTTCTCGGGGGGGGGGGGGAGAAGTGGTTAGAGTTAGTCATTTCTCCAGTTATTGTATGAATTAGCCGCAGTTGTACCGACCCCGGTGATATGTGGGCGATACTTTGAAAAGGTGGGGAATGGCAAGGAAGGTAAAGGTGAAAGTAACCACTCTATTCTCTTCCTTATTTTATTTGGCTCTTTCGTCACAGCGACGAACTATCTTTTAAGTGGTTCCCGTGCTAATTTATTCTTTTTTTTTTTGCTATCATCGTTAACAGTGGCTGAGTTTTTATTTGTTTTATCTTTTTTAACTGTTTTCCGTGTTATACTTGTTTTTGTCGATTGTTATTGATGTGTATTCACAAGGTTTTTATCACGTACTCCCTTAATCACTTATTTGTTTATTTTTAAAACATGGAAGTCCGCTGCTGTGCGGTACCCCATCTGTTCTCCTTATTTCATTACACCCCGCTACTTTTTCATCCCGTTTCTCCTCTTCTCTTTTCCGCTGTTTGGAAGGGACGGGATGAATTGATATGCTCGATGGGATCAGCAACAAAATAAATACGTTAACAAATACTTTTACGCTCGTGCACTGCACGTGCGTGTTGAatgcgcgtagctgatcggatgaatggatacactaaacactgtttttatttttattttttccccctttttatactttcctcttcccaatttctgtcctctgtatcactaacattgttgatggtatttcttctgtttccgtttctccatttcatctccctctcacacaaactcgtacatactttaaaaaaaaaaa

>Tb927.11.3120

ACATAATTATGGACTGGTGTCCGTGAGAAAGGTGTAGGTTGTGCCGTTTCCCGTGTTTGCGTCTGGCGGAAGAGAGTGCTTGCGTGTTCCTTTTTGTTACTCACACACACCTCGTTCCCCCTTTTCATTCGAAGTGTGAGGTTGAATATGTGAAGAGGGAGGTGAAAAGGAGCAAGAAGGACAATTGTCTAACTGGCGCAAATTCAGGTAGACATCTCAGGAGTGAACAAATATGGGGTTCAAAGGCGCGCGCGCTGGGGGAGGGGAGGGAGCGGTTCCCACTTCCTAACACAATGAGACCAACGTACTTTTCTTACTTTATTATTATTAATTTATTTTCTTAGTCTTACGTAAGTCAAGGGCATCCAGTGGTTGTGCTGTATTTGTTGACTGCGCGAGTGTTTGAGTGGATGGAAGAGAGGAAAGAGGTGCTGGTTCGCTAATGAAGAGTTAGTGGAAGGGAAATATTGTAACATCGCGTTTTCATCTTGTTGTACACCTATTGGAGGAGATGCGAGTAAGCTTGTGGTAATATTTAAAAGAAAGTTATTCCAAGGAAGTTAAGTTATAAATTTATTTATTCGTGTATGTAGTTTCCTTTTCTATTTCCCTTATTCCTTTTAGCAGATCGAAGGAGTGCATACTGCAGTAGCACAGCATATAGAATTTTCTTCTCTGCCATTGTCTAATACTGCTATTTTTTTTTCTTTTGTTGGCTCATCTATTTTTAAA

>Tb927.11.14790

AGATATCGTTTTTCGTGCCCCTCCCTTCCATTCATCCTCTGTGTTTGTTTTTAGTTGTGATTCTCTTCTTGTTATTTTTTTGAATATATATATATATATATATATATATATTGTAATTACCCAATTATTTGCACTCTTTTTTATTTGCGCAAGCGTTTTGTTGTATTCATCTGTCTTAACCAGTTAATTTTTTTTTGCTAAACTTCCCCTCCTTGGCAGTTTCCACTGTCGTGTTACCATGTTTTTTTTATTTTTATTCTCACAGGTCCTACAGTCTCGAATTTATTTTTTATTTTTTAAATGGCCTGAACTGCGGTTGCCTTACCTTGTCTTCCATCTCTTTTTTTTTTTTGTGCATGTGTCATCTAATATATATAGTCGTTTGTACTTTTGTTTTCTTTTAAGCGGGACTCCCTTGATAGAGGTCGCCACCATCATATCTGTTATTGGCTTTCTAAAA

>Tb927.1.1850

AGCGTGCTGCGGCGCAACTTCATGAATCAAGTCACGGGAGCAACGAAATAAGAATATCAAAAGTCTTAAAAATGAGGAAAACAAGAAGTGATCAAACGACGTTTACGATTGCTTATGCTAATTGGCTTTGAAAGGAAGAATTATCGCCATAGAAGAGAGAGAGAGAGAGAGAGAGTAAGGATGTCACTAACCCTATTTTCCTTTACAGTCGCTCAGTTTTGCTGTTTCTTCTTATATTCCCTTACAAATTTCCCTAGCTTTTTTTTATTTCCTTGTTCGCACGTATTCAGATTTGTTCTTTTCTTCTTTACTTCGTTTGTTGCTTGTGTTTTATTTTTTTTTCGTCATTTACAAGTTCTGTACTTTTTATTGTCGTTGTTGCCATTTGCTCGTTCCCACATGAGCGCTGCTAAAATCCCTTCTCCGCTTCTTCACTCCGGACTTACTTTTAGCTGCCATCCTTTTCATTGTTTGTTTGATGTTCCCCCCCCCCCCACGTGTTATCGCTCCGCATTTCATTTATTTATTTATTACAAAAATATATGAACAAACAAAGATATGCGCACGCATGTGTTTGAGGGCATTTGTATATTTGTGAATACGTCCCTTGAAATGTGCCACTGAACCGCCCTATTTTCAAAAACAGATCGGTCGTCGTATTATTGCTATTTATTTCCATTTTTTTTTTTCATAAAACCAGGTACACCAAAAAAAAAATTGAAAGAATTGTTGGACGAACTTGCGCTTATGAGCTCACTTAGTTAGCGGGTACAAAATGAATAAAATAAATAAATAACTTTTTTTTTTAAAGTGCGGGGATAAATAAAAAATAACACAAACAGCTGAACTTCTAGAAGAGGTGTCTTGACCACTTTTTTTTTTATTCACTTCGTTTTGTTCCCATACCTTAATCTCGTTCGCCTTTTTCAATCATTAAATAAGCTTCCATTATTACGATTGAGAGTTTTCGCTTTATTTATTTTCCCGTAAATATATATATATATATATATTAGAAGTGAAAGAACGAATAAATAAATAGTGAGGTGCTCCCTTAACGGAGTGAATTCTGTTATGTTATGTTTTTTGTTAACTCTTCTCTTCATTTTTTTTTCGCGCGGTTAAGAATGGAGCTCCGCTCGAATTGCATTTGAGTACTGAGCTTTCCTGCTGAAAGTGAGGGAACTGCTTTCGAAGTAACATACGGATAAATTTGCCGTTTGTTTGAAAATTTACTAATTTATTTGTATTTTAATTTTTTATCTATTTCCTTTGTGTCCTCATTCCTGCTACATTTTTTTTTATAAGTATTGAGTTGCAGAGCTTAGGAGGAATAAACACGTAGTTTTTTTTTTTACTGCATCATTTTTGTTTGTTTGAGACCTCCGGAAGTTCCTGGTTTTCTCCCTTTTTATTTATTTTGTTTCATTTGTGTTTGATCATATGTATTCGCTTATTAATATTTTTGATTCATCTGCCTGTGTGCATTACTAGTGATTTTATAGTGGGTGGGCATCGGAGTAGTGAATTGTTTGTTTGTTTTACCCTCAATTCCGCACTATATATATATATATATTTATTTATTTATTTACTGTGTTGTTTGTGTTGTTAACATTGCGATCGCCGTTTGCTACTGTTTGCTCGCAGTTTTAACACATAGTTTATTTAATTATGTGTTTGAGTTTATTGATGTTTATGATATTTGTGTTTCGGATGTATTTTTGTTCGCAGTGGGGCACCGCTGGACGCTTTCGCGCAAAAAATAAATATACGTTAATTTTGAAAGCGCTGGGAGGTTAGGTGTTGCGGAGAAGCCGCATAATTTTTTTGATACCTTTTCGCGTGTTGGTGTTGTTGTTTTTTGACGTGCCGCAGCCGCAGCTTTAATTTCTAAGGAACTCAATGAGTTTACAAAAAAA

>Tb927.10.15880

ACCGATCGGCATTTAGGGGCCTGGCGGGTGTCTCTTGTTTCACGCGTTCACCATCCGATGCGGTGGTCTCTAAATTTCCTCACAATATTCATTACCTTTACTTACTTTCTCTGCCACACGGCGCAAGCGGCTCCTGCTTGCGGGCGGGTTTGCTTAACGTGGGTTGTGCTTTTTTGCCGCTTGCTCTCATTGTCCGAGTGGTCACTTCTATTTTTTTTTCTTGTTGACGTTTGACTGTGGTCAGGGGACATCTCATTGTTGTTATTTTTGCTTTTAATTGTTACCTAAACATGTTTCTTCGCCTAAACGGAGGCGGGTGGGGCCTCGCGCACTTACAAGAAACCTCTGTAGAGGTGGTCATTTGGGTCAGGTGGAGAGGGAAAGCTTGTTTGCTTGTTCAACTTTACGCTGTGAGTAGTCCTCCCTTCCGTGCATCATTCTTTGGACTTTTTTTTTCTTTTACCGCCCATCCGTTGGGCTGTTCGTTTATTTTTTCTATCAGGCTATGTCAAA

>Tb927.10.15880 includes two mis-annotated ORFs, here in lower case.

ACATTTGTTTTTGTGAAAGTGGCGTGCATGCGGATGAAAAAATGAAGCGCAAAGCAGTAAAAAGAAAAACAAAAAATAAAATAAAATAAGAAACAAAAAGGAAGAAAGGAAACCTTCTCCTTCACATTCCTTCCCTCTCCCCCGTTGACCAGCCTGGTCAGAAAAAAAAAAAGGAAAGATCATGAAGATGTGCAAATATGCCGGTTTGGGGTTTCGTTAGAAATCAGATTGAAAAGTGAGTTATCCCCTTTTTATTTTTTATTTTATGCATTTTTATCCGTTGATTTGTACTTTATTTATTGCCCACATGAGTTAGCGGTTGTGATTTAGAAGGAGAAATGTCTTCTCCCTTTCTCTTTTCTTCTGGTTTTATCATATTTATTTTTTTTTCTCTCTCTCTTTTACTACCGTTATTGTATTTGTCTTTCTTTTTTTTTATTTTTTATCTGGTTACGCGTCGAAAGGAGTGTGGTCCAAGCGCTGGGAACATTTGCTCAGGTGCGCGTCTGCCTCCATatggggccctttaagaaactaatgagaaaaaaaaaggaaaaaaaaatgaaacaaatgttttattgcagcaaaggaaacagtcgctgcttttctctttttttgtttccctttgatcccatcagagtgcatcctgtaaagtttctctttttttttcccccttttccacgtgcatttgtatatattttctatcgggtttTATTTTTTttttgtgtgtttgtttttttcttccgttgtgtgacagcatccgtatatataaatatatatattatttattttcctttttacgtattttatttaattacaacctcgagaaatcatcattaccacacgtgcgcagtcgtaactgcctctttttttttctttttctttttaacatttcttcactcaaatatgtacttttgtttccatgtgagtgaatgaATGTGCGACATTCCGTGACAATTCTTCCGTCACCCATAGTAATCCTCATCGTATCTCTGATTTATTTTCATTAATGTTATTCGCATTATTTTTTTCTTCATTTTTTATTATTATTTTTAAATTTAATCTTTCCTTCCTTATATTTATTACTGTTACCTTCCACTTTTACAGTTATTTTATTTTTCTTTGTTTGGGGTAATTTAATTTAATTTAATTCGTGTCGTCGTCGTTGTTGTTGTTTTGTTATTATTTTCCTTTTATTGAAAGAAAAGGGGATTGAAAAAAAAAAGAAAAAAAGAAAAAAAGTGGAAATGAGAGTTGCGTTAACTGACGCAACTCCGTCGCTATGTTTTAATGAGTGACACGCGTGTCTTTTTTTTTAAAAAAAAAACCTCTCTTTCTTTCTTTTACTATTTTATTATTTTATTTCTCTCTTAAACACACGTACAAATTGCAAAAGCAAATACACCAAAGAAAAACACATTTCATCATTCTCATTATTATTATTTTTATTATTGTTGGTGGTGCTGTTTTTTTTTCCCCCCTCCTCCTCTTCCTTCTTCTGATTTATGTTATCGGCAATCTTATGTTTGCTCTCTGatgcatttatgtttttcctttctttctttcttatttatttatttctactccctaacatcactcatgtcttcgatattttactacctttttatgagctatttatttatgtttgtgtttatgtttatgtgtgtgccggcagcgacttcctgtccttattattcttcattattatttgtatttattttctgtttgatccgttttttcccccccttttactctgccttgttatgtcatgtctgtccttctttttctttttttttttggtttttactgtttttttttttactttttgtatatcttcatggtaatttatttcccccttttgtttttttctttctattatatatacgcaatgtgtaaCAAATATCCCTTTAAATATAATATCACTGTGTATGTGTGTGTGTCGCGCACCATTTGGCTCATAAATGTCATGTACCTCATTTCCCTTTCTTTCTTTCTGTTTAATTCATTCCTTCCTTTTATTCATTTATTTACTTATTTACTTAATCTGCTTTTTATTTTATAAAAAAAATATATATATATATATATATCCATCCATCCATTCATCCATATATATATTAATATATTAATATATTTAATTATATTTATTTATATAAAAGAGTGCTGATATCAAATCGTTTCGAATTTTAACTCGTGTTTGCTTCGGGTTTTTTTTTACCCCTCCTCCTCTGCTGAATTGACAGGCGATTATCCAATGGAGATGGTTGATATTATTGTTATTATTAGTTTAACATATGCGTGTGTTATTTTTTATTCTAACGCAAAAGTGATGAAGAAAAAATAAAAAGAGATTATCTGCAACGTTTTATAAAAATAAAATAAAATAATTACTGTTGTTATTGTTGTTGTTGTTGTTGCTACTGCTATTATTATTATTATTTTTAAAAATATTATGAATATTTATTTCCTTTCTTTGATGTGTGATCGCATCTACTCCTCATTTTTCTCGAGTACATGTACCGAAAATGCCATAAATTCCGCTATTTTTTTTTTTTGCGTTTGTGTGGTGACAGAATTGTGAAGAAATTATTCAAAAAAAAATAATAATTAAAAATGAACGAAAAAAAAAAGACGAAATAAAATAAATAATTATATATATATAAACAAATATTGATAAATTCAAGACGGTTATCTGACATGAGTGTTGTACAAAAAAAAAAAGAAGCAAAGAGTCGAGAAATTATAAAAGTGGTGGAAAAGTGCCTGAGAAAGAGTGACGGAGGGAGAAAATATGTTAAAATATATAAATATATGTATTGTATTTTGTTTTTTTTTCTACCTCCCATTCCTTCTATGTTTTTCTATAGATTTATAAATAAATTAATAAAAATATACGCAAGAAAGCGTGTCTGAGTTTACATGTGGTCACTGCATCAATTTCATTACGAGAAGGACCGGCAAGTGACGAACCGCAATGTATAATAAATATATATATTTTTTTCTTTTTTTTTTTTTAGCATATTTATATTTATATCTATCGGCATGTTCATATTCGTTTCTTTTTTTTTTTAATTTGGTAGAATTCTTTTTGTTTAACTCTTTGGCATTCCATTTCCCATTCGTCCTCTTTTGCACGCAAACGTGTGGCTGAGTGGTGGAATCTGCCGGTAATGAAGAAATAAAACAATAAATGACAATTAAAAAAGAAGAAAAGAGAAACAATAATGATAAAACAAACTCACATTTCTTTTTTTTTTTTAAAGAAAAAGGTGCGAAATTATTAACGTAATGAATTAAACAGGTGACTATTTGACAGAAAGATGTGAATAACTACAGAAATTTGAGAGAAAGAGAAAAGAAACGAAAAAAAAAAGGACAAAAAAAAGAAGGAATAAATCAACAAAGTAATATGAAAAAAAAATGTGATTCACGTATGTATTAATGTATGAAACACATTTATGTCTCAATGGATTTTGCTGCTTTTTCTCATTATATTTATGTTTGTCTCTCGATGTATTTATTTCTTTATTTATTTTTTAAATATGTGAAAGGAATTTATTTGTGACGCGTCAGGAACTCCTCGCGCCTGTTATCATTTCACGTAATGGAGAATAAATAAATAAATATTTATATTTACGCGCTCGTTTTGTTGAATTCTTATCCTTCATCATATCCATTGTACCATTTTTATATTTATAATTCATCTATTCAATTTTATTCTGTTTCTTGTTTTACCTATTTATTTTTCATCTGTACACGAATTAACAGATTGCGTTTAGCAGTTATCAATTCAGTTCCAAAAAAAAAAAAGAGAGAAGGCATATTCTTTCTGTGTATGAGTTCACAAAAAAAAAAGAAAATGTTATTAAATGTATTCACGTGTCGTTTTGCTTTCTTTTTTTTTTGTTTCCCCTTCACTCTGCTCAGACACTCTACAAGGAGAAAAAAGATGTTTTCTTGTTGTTGTTATTATTATTATTATTTCGTTCATGTTGTTTTGTTCTATTCTGTTTTTTTTCCTTTTCCTTTTGCTACCCGGTGCGAAAACGGTATCTTCTTTAATATGGTGAAGTGAGCGAGTAATAGCTATTTTTAAAAAAAATAATCAAACGATAAAAGTTAAAAA

>Tb927.3.2940

AGGTTTATATTATTATTATTTTTTTTGAAAAATTTTTTCCCCTTCATGGAGCTCTGAATTTTGATTATTTTTTTTTTGTTGCCTCCGCTCGTTCTGTACGATTATAATTTCACTTACCCTATCCATAGTAGCTTCATGTGACAGATTATTGTTTTTTCTTTTTTCATTGTCACCTTCCACGACCCGCGTAATTATGTGCATGTATTTTACTACTACGTTCTGTATTTTCACAAATGCCATGTTGGATATGTGTTCTGCCGTTTTGACGTTTTATTATGTTGCAAAATGTTTTGTTTATCTTTGGCATTAACATGATGGCTACTCTTTAAAAAATTTCCGTTTAGTTCCATCGTGGCCGATAAATTGTTCATTTGTACGTTATTTCCCTCCTGCTTCGCGCATTTTCTTTTTTTTTTTCGCTTTCGCTTTCGCTTTCCTTTTAAATCATCACATAAACCTGACAGTGCAACCCCTCAGCGAAAACCCCCCTCTTTTGTTTATATTTTTTAAATAATAATAATAATAATAATATGGGGTGTGGGGGGGGGGTGGGGAGGCGAAGTTGTGTCAGTTCTTCCTTCAACTTCCGCGCCGCGATTGAGAGCTTTCATAGTTTCGGCATGTTTCGTGCGAGTGTATTTTTATTTACGTATTTAATTTCATTTGTTTATTTTTATATTCTTCAGAATATATACACAATTTAAAGCTAAAATAAATGAATTTACATAATCACAAA

>Tb927.3.2920

ATAACAGTTTGTTGCATGTCACTACTCATGCGTCTCTTTGATGCTAACAGTAGCACCAGTGGCGCCAAACATGTTATTGTTAGTATCGTTACTATTATCGTCATTATTATTGACGTCACCATTAGCCCCAAAGTTTTTGCTCCAGTTACTTGTATTTGTGTTCTGCAGGAGGTTATGTTAACTTTCCCGTTTGCCATTTTTATTGTATTTTACATTGTGTACCTTAACTAGCTGCACTTGTGCAGTAAAGTGCGGATGTGAGCGTGCAAATTATGAAAGAGTGCGGTAAAAGTTGTTTCTTTTACTGAGGGGTTTGGTGGAGTATGGGTTCTAGTCCTTGTTTACACTATTACCATTACCATTACCATTACCATTTCCATTCATTATTGTTTGTTATTGTTCGTCATTTAGTTTTTTTAAAAATTTCTTCTTTTACCATACCGCGCTATCGTTGCCTGTTTTTTTTCTTTCTTTTCTTTTCTTTTTTTTAAATACACTTTATATTTTGGGTTTCTTTTTATTTTTATTTTCCTTGAATTTAACTCACTTGCTTATTTTTATCTGTTGGTTTTATTCCCTTCTCTA

>Tb927.11.2410 annotated pA site length=741

ACGCATGGGCGCGTCAACGAAAAAGAGGTAGGAACCCCACAACGTGCGGAACGAGGAATGTGAAATTAAAATATTTCAAACTGATCTGTAGTTGCAGGTTTCCTTGGCGAGGCGGGGAGAAAAGAAAGAAAGTGGAGCGTAAATAAATATAAGTAGGGAGAAAAAAAAATACCGCAAATAGGGAGGATTGACCAATATCCCTCAGGAGTTTCTTTCTTCAGTTTTGTTAGCGTTAGAAAGCTTTTTTTGTCTTCACTGTTTTGTTTTAAAATGTATCTTTTACTCTTTTACTTTTTTTGTGTGTGATACTGTTTGTTCTTTACTCGAATTGTTTCAGCAGGGGTAAAGCAACTTCGACGCAGAAATCGAAGATAAGAAATGGGGAAAGGGGAACTGTTTGCGGAGGAGGCATACGCATATGTATCGTGAGAATATTCACTCCTCTGTCGTCGTTCCTTTCTAAACCCATGCATGAACTTAAATGCATTAGAGGCAGAAGGTGCACATATATATATGCTTATATGAATTTGTATACATATATAGTCGCCCGTGCATTCGCTCTGATACCTTTTTCCCTCCCACGATCGATACTATTTTCTGTTTACTTGATTATTATTATTATTATTATTTTTTTAAAGGCCGCAGCACAAAGTGCGCACCACATTTCCAGTAAGTACAAACGGTGGCATTAAATTTTGTTTCGTATTTCTGTAATGAGCGTCAGAATTCTTTTTTACTTTT

>Tb927.9.13610

GAGAGAAATGTGTTGGGTGAATGTGGGGAACATTTTTCTTTTTATTGGAATGAAACAAAAGAACGCTCATCCTCTATTAAAGGACCTCCTCCACAAAAAACGAGGGGAGCGAGGGGAAAGCAAATAAGGAAGGAAGGCGAAAATGAACGTGAACATGCTAATAGAAGGAAATAACGAAAACTTTCCAGTAACTAAAAATGAAAAGATAGAGATTTTCACCCTTCTTGTTGTTGTTATTTTCAGTGGTGTTGCCATTACTACAGTCGCCGTAGGTTTCTTCAAATAAGTGTGGGAAAAGATCTATATATATATATTTCTGTGCGTGCCGACTACTCCTTTTTTTTTTTCCACGCCAATACTCCTCGAGCCTGTCCAATTTATAAAACGGTAGCAGGAAAGGAAAAAAGGATAGGGACGGTATGAGTGTACGGAAGTGAAGATAGTTTAAGACAGCTAAATCAATGAATGGGTGAAAAAAAAAGGGCGGAAATGGTGAGTGCCAGGTGAGTTGGGCTAAGTCAGGGGACAACCATATCAGCAGCTGAAACAGCTAAGTAAATTAATATGATTATACTTATTATGAAGAGATAATACCACCCAAGGGGAAAATGTTGGATGGATGGGAGAGTGTGCGGTTAAAGCACTTTTGAGTAATAACGGTAGAATAAAAGGTGAGAGAGGAGGCGCAACAAATACGGGTAATTAATGGCTTAAATAGCGTGAGAAGGATCACAGTTTCTTGATAATAGCAATGACTGTTGTTCCAGTCACTTTTACTTTTGTTATGATTATTGCTGTCATTGTTATTCACTCCATTCTTTTTCCTTCGTTTTGTTTCAAATATACCAGTATTTGCTTCTCTTTTGCCACGTCCACATGAGTTTGTTGTTGCGGAGTTTTGGAACTCATATACACTTATACATATTTATTGTGAAATGTTTGGAGGAAATAGGGATAGTTTATCCTTCCGTTTCTCTGTTCGATCTTATTTTTATGTTGTTTTGTTGCCAACGATGCTGCTTCCACGCACCTTTCTTTTTTATTGGTTGCTGCATTTGGAAAATGCAGACAGATGAGGGGGGCCCTATGCTGCAATTTTTTTCTTTTTGTGTTTCTTCCGTTTATCACATTACTTTTTTTTTTTCGTTTTTATTTTTTAGCGCACCGTCGTGGCAAGTTTTCGAGGTTTTCTAAAAAGGAGATTTAATTACCGGTGATTTGTGTTAGCCGATGCATGTGGCATTATATTCAGTGAGTGTTGCATTGCACCGGTAAATCAAAGGACCTCGGTAGATCATCTCAGCTTCTTACTTTGCTTCGACACCTGTCGCTTTGGTTTCTTTTTGTTTTTTGTTTTTTTCCTTCCCTTTATTTCTTGTTGTCGTCCTTGTCGTCATTGTCAGAGGTAAACAGGTAAGTAAATAAATATAGATGTACAAATTATGTATGGATTTGCGTCTCTGTGTGCCCCTTATCTGTCTTGCTTTTCCCTGTCTCTACTTTTTCCATCTGAGTTTTTATTTCATGGGCTGCTGAGAGGTGAGGCACGTGATATGGTGTTGACTGCCACTCGAATGATCCCAATCACATGCTTCACTTAATGTTGTAAATACCAAATTATCAGCATACCTTTTTTTCCTGGTTTCGGTGTGTCGTTGTGTTGGTGGTGTCGTTTCCCCCCCCCTCTCTCTCTCTCCTCTTCTCTACCTTATTTCCTACTTACCCTTCCTCCCCTAATTCTTCCTTGTAACAATTATTGATGCCGAGTCTAAGCATGTCGTATTAATTGAATGAGCAAATATGTTATTATCTATCGATATGCCTATCTATATATGGATAGATCCCTTTATTTATTTGCTAAAAGTTAGTAATGCTCGCTTCTTCACTAATTGGGAAGCTATCGGCTGCAAAAAACAAAAAA

>Tb927.7.690

ACGGAGGGAGGGGGGAGTATGTTAGACTTAAGGTTAGGAAAATAGAGATAATACCAAATGAAATCCCTTCCCTCCTCCTCCTCTCCCAGTCCCTCCCGCATGCACAAATAAATAAATAAATAAATAAAAGAAACAAACGAACGAACGAACGCGAAAAGAAAAGAAAAGAAAAGGGGGAAGAAACAAAAAAAATTGGAAGGCTTAAATCATTGACACGTATATATCAATCGAAGTGATAGCCGAGTTGCAAAGAAGCGGCGGAAAGGCCCCTACCTCTCCCTCAAAAAAAAAAAAAAATAATAATAATAAATAGTACGAACAAATTGTGGGAAAACTTTTGCCGTGTAACCACCGCTGCGCTCCATTGGTGTAAACTTCAGGGTCAGCATCACCTCACGAAGGTTATGTCCCACTGGATATGTCTTTTCTATCATTACTTCTTACTTTTATATTGTTTTTTTTTAAATATTTGTGCTATTATTTCTGTGTATTAAACCGGTTACAAAGTGTAAGAAGAACATCCTCCTTTTTTTTCTTTGCTGGCGTGTTGTTGTTGTTTTTTTTTTTGTGGTTTCCTTTTTTCCACTGTTATTTTCGTGGTAGTTCGACTTGTTTTACTTTTTCGTGCAAGGAGTCATGTTTATTTAACATGGTTTCTTCACGGGATTCCTTTACTCATTTCTACTTACTTTCTTTTTGTTAGTGTTTAGCCACTGTTTTTTTTTGTTTTCTTCTTTCATTTGTTTCTTTTTCGTGTCTTATTTGTTCATATTTTCTTAAAATTATCTTTGTATTACTTGTCCTCCCCTTTAAACGTTCTGCACCCTCTTTTTTTTTGTGTTTGTTTGTTTGTTTGATGGGAAAGTCACTACTGACCTGATGTGATCCCCCTTAAATGCCTCCTTTCGTCCCACTTCCGGGGTTTCATCGTCGTTACTTTTGTTTTTTTTTAATTTTTAAAAAAATCAGTGGTCTATGAATTTGAGCGGTAGTTTGTGTTTTTTATTTATTTAATTTATTCCTTTTGACTGCTTCTTTGGCATTTTACACGCGCCCTGTATTTTTCTCTTCCCTTCTATTTATTGAGCGTGCGACTATTTTTCTTTTTTTTTGTTCGCGTTTGTGTGTGTATATGTGTCTGGATCATCGAACATATGAATTGCCTTTATATTTTCCCCCCTCTTTTTTTCCCCCTGTATCTTTGTAAGTCAATAATATATGTATATATATATTTTTATTTATTAAGTGTGTGAATGTATTGCGGTTGCTAAACTGTTTGTTTGTTTGTTTAGCACATTCAGTTCAGTGCCTTTCAAAACTTCGTTAGCGGCCGTCCAGGTATGTTTTTTTCCCCCTTTCCGTTCATTTTCCTTTTGTTTTGTTTTAATTATTATTATTATTATTATTATACTCTTCCTCTTTTGCCTTGGATTCTGGTTTGGACATCTGAATACAACTTGTGGACCATACAATCCTTCGAGCAACTACAAACTCATCATCTTTGCTTTTTTTTTTTGCTTTTTGCTGTTTCGTTTTTGATTTTGTTTGTCTTTTTGTGCCATCATTAGTTTCCCAACTGCACTACCCTCTTGCTTGTGAAAAGCTGTTTCCACCCTTTTACTGCTTTGTGACCATCATTTTCAATTTTTTTTTTTGCTTTTACGTTGCGTTGCGTTGAACTAATATTATTACCATTATTATTATTATTATTATTATCATTACAATTGTCGCTGTGAATACAACTTATTTCCTTTCTATGTGAACACCCTCTCCAGTGAACTTTTCATATTAATTGACATTTCTGCAGTGTATGAAGGCGTTTGCCGCGATATTTTTGGTTCATTGTTTTTTTGTTTTCTTTTTATTTCAAAGCAGCGAATTCTCCACCTTTGTTTTCTTATCATCACTCACTGTTTTGGTCAGTTTTTTCGGTCCTCTTTCTGTTTTTACATGCACTTCAGGAGTAGTTCACTTTTTTAAAAAAAAAAACACTCTCTTCTCTTTTCTTCTCTTCTTTTCGCTTCAGCGTGACTTTTTTTTTTCCATCCCTTTAGTTTTAAGTTATGAATGAGTTCTTATGCGGTGTGTGCCAAATGAAATGTAGGTTTCAAAAAGAACATCGTCATATGCTTTAGCGTACTTTTCATTTTGTCCGTAACATCCGCCCCCTCAGCGTGTTTTGTGGGCATGTCATGTAAGTTTATTTACTTTTATTGGTAGTTCATAGACTCTTGAGAGTTAATGGAAGTGCTGGATATCTATATCTATATCAATATCAATATCAATATATATGTATATATGTATTTATAATAACTAAGTTTTCCTCCGGCTACATGTTGTGTATTTCTTTGCTTTTACTCGTTACTAACCTTGAGAGTATTCCTTTTTTTTTTATTTATTCCACATATATGTGGCAGCCACCACCTCGTTTCTCGTGTAGCGGTTGATTAAATTTGTTTTGTGTAAAGAGGTATCGTGTGAAGCAGCTCGACTCCCATTTGTAGTGTTTGTGTTACTACGCTTCATCACCGTTGGTAATTACATTTCGGGTGATATATTGAATCTGGAAGAAGAATACTGATAAGAGAGGGAGGCGCATTTTCTAATGTCTTTTGTTGGTTTGGTGGTGGTATGAGAAAATGAACAAAGGTTAATGGTTATGTGGCGCATTGCAATATCTCACTGTTTGACTAGCGGCTTCGCAGTTGGAAAGATGTCGATGGCGTAATGTCAATAACCTTTGAATCTTATCGCATTCTGTTACACTGAAGGGTTGTAGTGTTTTGGCCTCATCTATCTCTGCATAACCTCCAACCACTTGTCGATGGAAGTGTTGGCTCCGGCTGGTTCATGTTTAACAGTTATCATTGATATGTTACTTACTTACTTACTAGTTGGCTGATGACTGTTCTGCAGTACTCATGTGATAATTTCATATTAACGCTGAGTAAAAGCAGGATAAGAAAAA

>Tb927.10.10050

GCGCGGTCCGTGCATCTAATATTTTAGATTGAAGTTGGACGGGGGTAAGCAGTGTCCACGAAGTACATTTTTGCGTACTTGCATATGTATGGAAGGGAAGTTGAGTGAGGGAGGGAGGGAAAACAAAAAGGAAAAGCACAGGTGGCGATGTCCTTGGATCTGTATTTGTCTCTGCAGTGTATCATTTGTTGTGTTAACTCACCCGTCTCTGCTACCGCTGCTGGTTTCGTGGACGTTCCGCTCGTGGGAAGATGTACAGCCCGATGTGTCGAACCGAGCATCTACCTCAATGGCAGGTGTGTGTGCGATTTCAAAGGGATTATTACTTACCTCTCCGAGATGGTATTAAAAGGAGGAGTCCTTTGCGTTGAGGAAGGACGGGGTTAGTCTGACGGGCTGGCGCATTGTTAATTCTTCTTCGCTGCACTGTGGCACGAGGGTCTTTGCAAATTGCATGTGTGCGTTTGCGTTTGCGCTCACGTGTGTGTGTGTGTATGTGTGTGTTTGTTATGTAAATATGTAAGCACTCGGACCTTTGAAGGAGAGGAGCAGGTGTACTGGGAGGTTACGTATATGTGCACATATATATTACTCATGTATACGTTACGAAGGTATTACTTGGACAGGTTTATGCGGACGGCAACAAGAGTAAATGAGCATAAGAAGGAAAAGTTAAAAAAAAAACAAAGAAAATAAAAAACAAAGGGAATCTGAACGCAACTTTTTGTACGTGAAGGGTAGTGAGGAAGGAGGTGCCTATGTAAACATAGAGAATTTTTTTTCTGAGGAAAAGCCCTTCATTGTCACTTTTTTTTTTGCTTTTCGGTCCCTACTTTCCTCTTCCATCTTTTTATTTTTGTTCACCGGTGATCGTAACCTAATGTGTTATTGCTCTCCTCTACGTTGTGATTACACTTTCACGGATGTGTGATGGAGGTGGTGGTGGTAATCACTCTTTTTGCTTCCTCATGGTTTGTAAACTCGCGACGTTTCGAACACCTCACCCCATTTTCTGTTTTTTTACAGCGGTTTGTGGCTGTCATTGACATCCGGTACTGCTGCCACGTGCCCAAGCTGGCCACAGTGGTGCCCACACAACAAACCGGATGGTGTGTTCTCTCGTCTGCGTGTGAAGGAAAAGGGAATGTAAAGAGTGAGGGGGATTCCACGCAAAAATTGTGTGAAAGGGATGAGATTGCTACGGGGTGGAGGGGCGGTTGCAGGAACGAAGGGAAGGAGTGGAATGCGATAAACCACCAAAAAAAAGAAAAAAAATAGGAGTTTTCTTTTTTTTTTTAATTTTCCCTTTCGTTACTACTTCTCGCGTATTGAGCAATTCTAAAGTACACTCGGTTGATGGTGGGATAGTTTTGTTGATGGTTACTTGGAGGAGGAGAGAAATCTGTAGGGAAAGCGTGGGTATGTGTGTGCATGAAAGCTTGAAGTATTTTGAAGAGTGATACAATGGTGCCCATGTACTTCGTAGCTAACAATATACATATGTACGCACATGCGGAAGATATATCAACACTGGTGAAGCGAAGGGCGGCGAAGAAGCTGCAATCCACACGCCAATCACTTTGTTGGTATCCCTTTTCTACTGCTATCCCGCGAGAAGAGGAGGAAAAAAAAAGAAAACAAAAAATGGAAAAGGAGACGGGGGAGTCACACGGCATGCTTTTCGCGTACCGCACAGATGTTTTCTCATTTTTTTTCTTTTTCGGAGCAGTTGTTGACTCTTCAATGACTTTACGTACACATGCAAGATTAACACTCTCGCACATATCACGTGCGATACCTCATGCTCAATTCCGCATGTGTGATGTTTGACCAAACCTCGTCTCGTCTTGATCGAGTATGTTCGGCTTTACGCATTGCATCGGAGCATGCGGGTGGGTTGCTATGCGGCCTGTTGGCATGGTTATTGTGGTGTGCAGGCGGTGAGAGTGAAAGTAAAGAAATTTATATAATGTCTGTATAGTGGTATATATATGTATATATATATATATATGCACATAACAGGGAGTAAGTTTCGACTATATACATTGATTGATTGGGAGGGGAAAGGTACAATGGAGTAAAAGGTGGAAGGAGAAAGGAGGGGAACGTGTGGAAGATTGTTATCCGAAAAGACACCAATTTACGCTCAAACGTACGTGTCGTCTTTGTTTCATTTTCCTTTAGCTGGTGTTGTCCATTTTTTTTTACCTTATTTTTCTTTCTTGTGCACATTACTTTTGTTGTCGACTACCATCCCTTTCTTATTATTTTTTTTTGATAATTCTTTCTTTTTTTTTTGGGGGGGGGAGGGTCTTTGTTTAAACGGTATTAATAACGACGAACAGAGAAA

>Tb927.4.1910

AGGAAATGGAGACACGAGTATTTGGGTATGCAAGCCTTTTTTTTTTGTTTCCTCTCCATTTCTCTTGAGACCTGTGAGTTTCCTCTTTTTTTTTTTTTTCGTTTCTCTTTTTTTTTTTTATTTGTCGGAGAGGGAGGCGAAATCGTGCATGTCAGTTCCTTCTCCGTTCCGACTTCTTTTCTTTGTTTGACCATACACGTATTTGGTTGTTTGTGTCCTGAAGAGCTTATAAGTAGGATTGTGAGAATGGGGAGACAACAGCCGCTGGAAGGGTGGAGGGGAATGGAACTGAACACATGTGATTTCGAAAAGGAAACCATCAATAAATAATATATAAATACATATATATAAATAAATATATATATATATATATATTCCCCTTCCATTTCCTGCTTCCAGTTTTTCCGTTTTGTTTTTAATTTCCTCTTCTTCGTTTTGTTTTTGTGAGAAACATATTAGTTTCTGTGTTTTTTTTATATCTTTATCTGTTTTTTTTTATAATTTAGTGCTTCAGATATGCTTTGTGTTTTCCTTTTTGTTTTTTTTCTGTTTTACTTTTCCTTTGTTTGACTGTTTTATTATGTGTTTTGGTTACTTTCGATTACTTACGCACAATTCACCGGCACTTGTGCGGATATCATTGATATTATTATTATTATTACTCTCCCCCGCCTCATGGGGGTGGTGGGCGACAGCACGAGGGGGAAGTGAAAAGGCGGTCATGAGGGAAAAAGAGAAGAAAAATAAGAAAAAAAGAACAATAATGAGAAAAGCAAAGGAAAATGAGTGAGGAATTGCCAGACCAAATATATTATTTCTACACACACAAACACACGTATATATATATATATATATATATGAAAAGCTTGAAAGGTTTAATTTCTTTGTTTTGTAACAAAAAGGAGGTAAAGGAGATGAATAAAAGAAAGAAGAAGAGGAGGAGGAGATGGTTTTGGCATGCTTCTTTTCTCAACCTTTGTTTACTTCAATTTGTTTTCGTCTGATTTACTTCCCGTTTCCGCTATATTCCTTCCACTCTATTGCTGCACCTTCCACCCACCCACACACACACACATACATACATACGTACACATATATATATATATATATGCACAAATATGCATGTAAATAAATAAAACTATTTATTTATTTTTGATAATAATGATAATAATATTATTAATTGTCACCATTGATGTTATTATTATTATTATTACTATTTCACGGGAACACAGTGTCTCACCCTTCAACACATCACAACCTCTCCGACCTCTCACAAGAGGGGATGTTCCGCCAGTGTTGTTTACATAAGCCGGAAACCAATGGAGAAAAAAAAAGGTGGAAAAATGTGGGAATATTTATATTTATGAGCATTTCGTTCAATTTTCAAATTTCATATCCCTTGACATTTATCCATTTCATTATTTTGGTGTGTTTGTATGCACGTATTATATGCGTAAAATGAATGTTATATGCTCATGTGAGTGTTGTTGAGTGGCATGTGTTCCTGCTGCTGCCTTTTCCTTTTTTTTTTTCTTACGTTTGTGTGTATTATTTTTGTGTGCTCGTCAAGCCCCTTCAACGCTTTCTTCAAAGTGGAGGAGGGGCTTGTCGCCATGACGAAGGGGATGAGTGTTCGGGAAACCAAATAATTCTTTTTTTTTCTTTTCTAAAAACAAACAAAAAAAAAGAAAAGGAGGTGCAAAACACGAGGAAAAATAAAATAGAGAAACAATATGAAGGATCGAGTGAGTGATGGTGGTTGATGTAAAGAGCGAAGGAAGAGGGAAAAAAAAAGAAAGGAAGAAAATGTTAAAGGAAAGATGAAAATGACACAACCAAATAAATGCATGACATTACTCAACATCCTCTCTTTCATTTTGTTTTTTGTTTATATTCACTGTTTTGTATTTGTAATATCCCCAAACCTCCCCAAAGTTCTCCTACACTTCTTTTCCTTTTGTTTGTTTTTTCCCCTTTCCAATTTTTTTTCCCCATTCTCTCTCTCCCTTTGTTCCTTAACCTGTTGTTTTTGTCTTTTTTGTTTGTTTGTTTGTTTAACCTCAACGCGCAGAGAAGGAGCAAAAGTTAAGGAAAAGCAAAAAAAAACAGAAAACGATTAAAAGAGAGGGGGAAAATGAACTTTTTGTTTAAAAAAAAAAAAGTAAAACAAAAACAAAAAAAAAAGGACTCGTGCGTGTTATTTTGTTCAGAGTAATCCGTAGCTTCTCCCTTTCTTCCTTTTTTCTATCTTTCTTTGTGATTTATTTACTTTTAATATAATTTTTCTTCTTAAAGTAAAGAAGACTGTTAACTCTCAAAGAGGCGTAGGGGGTCATTTTCCAACCGTCTTTTTTTTTCCTTTTCATTTTCTTTTAGGTTTATTTTATGATTATGATTATTATTGTTGTTGTTGTCATTTTAGTGGAGAAAGGAGATGTAGCTCATGTTTATTTGTTTATTTGTGTGTTTATGTGTGTGTGTGTGTCTATTTGTGTGAGAGTGTTTTTTATTTCCTTTCTTATTCCTCTATTTGTTTTACTTTCGCTCAATTTGTTCAACCGCGTACCGCATGCATTTTTCCAACTGCTTCTGCTGTCGGTATTTGTTGTTGTTGTTATTGTTGTTGTTGTCACTGTTTTTGTTTTTGTTTATTGTTTTTGTTTTAAAACTTTTATTTATTTCCTTCATTTTATATTCATGCGTGGAATTTTTTAAAAACTTTTTCCGCGTGTCTGTGTTGTTTCACAACGCCACCGCTTCCACCGCGATTTAGCCCCTTTTTTTTCTTCTTCTTTTTCTTTTTAAAATAAATAAATTCCCCTTTCGTCTCACCTTTCCTTTCCTTTCTTTCTTCTCTTTCTTCTCTTTGTCCTTCTTTTTTTTCTCTTCTTTTTATTTTTCTCTTGTTTTTTTTTCTTCAATTACTTTTCCTATTGCTTTTTGCATACGCCATTCCCTTTTCTTTTAAAAAAAATAAATAAATATAATAATAATAATACACTGGTGTCGTTGATGTTGTTGTTGTTCTTTTGTCGTTTGTTGATTCTTTTTTTAAAAAAAATAAATATTATTTTAACATTCTTCACTTTCATTTTTTTTTTTAATTTTTTTTTTTACAAGTTTGTTTATTCCCCGCAATTTCCATCATTACCACCGACTGATTGAAAGACGACAACACGCGCCATGTTGTGATTATTATTATTATTGTTATTATTATTATTATCTTCAAAGGGAAAAATAAAAATAAAAAAATGAAGAAAGGATGTGTGAGTGAGAGAAAGAGAGAGAGAGAGAAATGAAGGGGAGGGGAGATGTGGAGGTTACCGTTCACAAAACTTTTAAAAAATATATTAAAAAAATATAAAAACAAAAAAACAAAAACACAAACGAGACAACAATCGATAGTGACAACAACTCCTGTACAAAATGAGAAAATGAACAACTGAACAAACAAAATAAAGGAGAAAGAATATCGAGAGTTGAGAGTGGAAAAAAAAAAGAAATAAAAGAAAAGACACAGAGATGTGAGTAGCAAAGGCTCATATATTTTATCATATTAAAATATCTGTTTGTTTTTGTTTTCATTTTCCCGTTTTTTTTTGTTTGTTACGTTTTCTTCTTTTTTTTTCTTTTTCTTCCATCATTTTACGTGTTTTTATTTATTTTTGTGTGTTCGCGCGTCAGTTATTGGCGAAGCTGTTATTATTATTATTATTATTATTATTATTATTATTATTATTATTATCATTATTGTTATTATTTTCCCATGGGCGCATTTGCACATGAAACTACCAAGGAATGGATATTTGTGTGTGTGTGTGTGGGGGGGGGGGAATGTCATAATAATAATAATAATGCAAATAAAAAGGAAAAGGAAAATAAATAAAAAAAAACTAAAGTTAAATGAAGTAAAACGCGTGGCTATGCGTGCATGGGATAAAATGAAACCACAAACAAAGATGAAAAAAAAAAAGAGATTCTAGTGCAAATATGATAATAATAATAATAACAATAATAGCAATAAAAATAACAGATGGATGGATGGATGGATGCGTGAACTTTTCAAAAATAATAAAAAAAGGAAAAGGAAAAGGAAAAGGAAAGAAGAGAAGAGAAGTATTTTTCCCCCCACCTTCGTTACACATTTCTCCCATTTTTAATCTTACTCCTGCAAACACATTTTTTTTAAATTCCAAAAACGAATTTTTTGGAGAAAAGTTATGTTTGTTTGTTTGTTTGTCTTCCCCATTTCCCTTTTTGTTTTTCCTCCTCCACTTCCTCTCAACTGTACGGTGCAGTGCAAAACAACAACAAAACCAATTAATACAAATAAATAAATAAATATATAAATATATATATGTATGTATTATTTTATACATTTACGCATCATCGTCAAAACATATACACAACAGCAACACAAAATACAAATTCTTCCTTATAAAATCAAAGAAGGAATTTTTGTTTTTTTGTTTTAAAAATATATGAATATGTCCCTTTTTTATTTGTGCTTGTTTGGTCCCTCATGAGTTGGTTTTGACTTCCGGTAGCATCGGTTGGGTTTTTGTGTTGTGTTGTGAAAATTAAAATAAAATTTAAAAAAAGGAGGAGGAGCGGGGAAAAAAAAATCAGTCGCCTTTTGGTATATATTTTTTCCCCCCTCATTCTTGCCTTCTTTTTTTTTCTTTTTCTTTTTCTTTAAAAAAATTTTCTCCCCATAAATAAATAAATATAAATATATATATATATATATTATATATATTATATATTATTATTTTTACATCTTTTCGTTGGGTTTTTTTCCTCTCCGTTTACTCTTCTTCATCAACCCTTTCTGTTTTTTTCTTTTCCTTTGAAAAAGCACAGATTTTATTATTTATTTTTATTTTTTTTTTGAAAGGGAAAGAAAATAAAACGAGACAATTTGTGGTCATTTGTTTTACCGTTATTTTATATTCGGTCCGCGATTCGCGCCTTTCATTGCTTTTCCCCTTTTTTGTTTCACTCATTTGTTTGTTTTATTATATCGTTATTTCCACTTAACTGTGTTTGCTTTTATTATATATATATATATATATATATACTAGGGACTAAAATAAATAAATAACAAATAAAGCACAGGAAAGAAATGAAAACAAATGAAAAGAAAACGATGGGGAACTTTCGTTTTGTGTTTGGGTATGCCGAAGGATCATCATTTTTTAATTTTTATTATTCGTTGTTATTATTGTTTTAGGGCGGTGGTGATCTGTATGAAATATGAAATATATATTTATATTTATATATTTATTTATACTTAAATTTATTTAAATGTTGCACAGATTGCACGTACATTTCCGTTGAGAGGAAAAAAAAGGAAAGGAAAAAAGAAAAAAAAAGAGAAAAGAAAAGCGAAGGGAAGGGAAGGGAAAAAGATATTCGGGAGGGGATGAGGGAGTTGTGTGAGCGATTTGTCATGATGTTAGTTTTCCTTATTTTTATTGTTTTTATTTTGTTTCTGTAATAATAATAATAATTATTATTTTTTGTTTTGTTTTGTTTTGTTGTTTTCAAGTGAAATTTACATATCCATAAAGAAAATGAAACAAGTGTGTTTTGAGATTTGTTTCTTTGTTTCTGCTTTAAACCATAGAAGTACATGACAAGAGAAATAAAATGACTTAAAGAGCCAAAATACAATAAAAAAAAAGAGAGAGAGAGAGAAAGAGGAAAAAAAAAGATTTAACAGCAACAAAAATAATAAAAAGAGAATCAAAAAAAGAAAAAAAGAAAGAAAGAAAAAAAGAAAAAAAGACGTAATCGATTGTTGTTTTTTTTTTTTTTACAGAAGTGATAAAATGAAGAAGAGAAAGAGGTTGTGTAACATTGGCTGAAGGGAGATGCATGAAGATTCCTCCAGACGTGTTTTCTTTTTTTCTTTTCTTTTCTTTTCTTTTATTGTTTTGTTTTTTAAGTTTTTATGAGGAAATTGGTGTAAACCATCGGTAAATTTTTTTTGTTTGTATTTATTTTTACAATTTCCTAAACCTTTGTTTACGGCTCTTTTCCTTTTACATCATGGAGGAAAAGTTGTTGTTTCCTTCATTTTCATTTTTCACTTTTTTTTTCTTTTTTTTTTTTGTCTTGCGTTGGTGAGAAACTCACTTATTTCAGGAGAGAGAGAGATGGGGGAAAACAACAACAACAACAACAATAATAATAACAATAATAACTGTATGTTGTTGCAGAGGGATATTAGAACCAAACCAACAAAAAAGGCAAGAAAAAAAAGAAAAGAAAAAAGAAAAGGGCGAAGCTTATGAGGACTTAAAATAAAAAGACTACTGAAAATGGCAGCCGGAGAAATGAATTAGAAGTGAAACATTAGCAAAATGATGAACAAATGAAAGAACATCAACGGAAGTTGTGTTTGTTTGTTTGTTTGTTTTTTTTTTAATTTTTACCTGCTTCCTTTACCCTTACGGTTACACACGCTTCATTATTATCATTTAGAGCCTGTTGTTAATATTCTCCACCACGTGGCAGCAAGTTGGCCGCGGTTATGATGATAGCGATGGTAACTGTTGTTTTGCTTTTTTTTTTTGTGATGGTGACAAAGCAAAGCAAAGGAAAAAAAAGAAAAAATAATTAAAAAAAAAAGAAAGAAAAAAAGAAAGAAAAAAAGAAAGAAAAAAAGAAGTGAAAACAACTCTCTTTTTTCTCTTTTTTTTTTTTTATTCCTTCCTTCTTTGCTGCTGTTGTGTTGTTTTGTTTTAATTATTATTTATCTTTTATCTTTTATTTTCTTTTTGTGGGATATGCAAAGGGTGTGTAAAGGGAAAAGGAGGGGAAATTTTTTTTTTCTTAAAAACAAAAAAGGAAAAATAGGGTTTTATACTTGAAAGGAAATGTGTCAAGTTTAGGGATAGTGATAGGAAGAAAAGGAAGATTGTGAATAAAAAAAAGAGGGGGAAAGAAGAAAAAAGAAATTTTCAAAATAATAATAATAATTATATATATATATATATATATATATATATATATATATATATATGGATATATGGATATATGAATAGAAAAAAGAAAAAGAAAAAACAAAAAGGAGAAAAGAAAAGAAAGGGATGTGTTACACGCCGGTAGCAGCAGTGATGATGATGATGATGATGTTGATGATATTGTTGTTGTTGATTATATTAATAATAATAATAATTATTATTATTATTAGTTGTGTTGCTAATTATTGTGTTGGTGATGGTGCTGGACTTTTAAAAGTAAAAAAAAAGAAAATGAAATATAAGGAGGTGGAAAAGCAGCCGTCATGAAGGACATTTCTCTCATGACGCAAAGGCACATACGTATGAAAAAAGAAAAAAAGGGAAAGTCAAAACAAAAGAGAGAAAATAGACAAAATAAAAAAAAAAAAGAAACTTCAAAGGAAAAGAAGAAGAGAGAGAGAGAGAAAAAAAAAGGAAAGTTGGGGTGAGAAGATTATTACAACAATAACAAAAATAGTAGCAGTGAGGGAAGGGGGGGGAGTGTGCGAGGAAAAGATTTAAAAAGAAAAGGAGGAACTGATATTAGTTTTGCTTCTTGTTGTTTTGATGTTGTTGCTTCTGCTGTGCTTTCGTTATTACTTTTACGCGTTTTGTTTTATGTTTTACCGCCGTTTACCGATTCCAGTACGCAACACTCTCTTTTTTGTTCCAGTTTCTCTTTTCTTATTCGGTTCTTCGTTGTTCCACTTCTTTTCCCATTTCATCTTTTTTATTAGTTGTTGTACCTTTATTTCGATTCGTTCCCCAGATCCTTTTTTTTTTCTTTCCCTCTCTACAGTTTCTTTATTTACGTGTGTGTTTGGGGTTTGTTTACTCATTTCTTTTTTTTTTTGCTTTTCCCTTTTGATTTACACGGTTTTTGTTTTCCTTCTTTCCTCTCGTGTTTCGACGATCCCGTGAGGCGCGACCTCGAAATGTGTTCTTTTTTTCTTTTTCGCTTACAAACAAAAAGGGAGACGACGGAGCATGAAATAAAAGTAAAACAGTAAAACAAAAA

>Tb927.9.9450 - includes and annotated ORF, shown in lower case.

ATTAACAATGGAGTAAGCATTGACATCTAGGGGGTAGGCAATGATACAAATAAGCGAATACCGAAAAGGACTAGGAAAAGAAGAAGATAATATAATATAATCATTCGAAGAAAAAAAAAAGAAAAAAGAAGGAAAATTGGGGGGGCAGGGGGAAACCAGTGAAGTAACTCCTCGACGCTAGCACAACCAAGAAGAGAATGGAGGACCGACGTCAATAATGATCAAAAGGGAAGAAAAAAGGAGATGGCTGGTGCGAGGAGCGCCACCACAGAAAATTTATACGTATATGTTTGTGTGAAAAAGGGCCAGAGGAATGAAGTAAGGAGGAAGAAACAAAAACAAAGGTATGAGAGTGTCTGAACCTCGTAAATTTTAGAGTAAACGGGATATATATATATATATATATGAAACAATTTTTAATAATAATAATAGTAATTTAATCATAAACGGACTATATGTAGGTGGGGCGCGTTTGCTTGGTTTGTTTCTTTCTCGTCCATGTCATCTCATTTCTTTTTCTTCTGTTCACCCTTCAGCTCATTTTCTGATCCCAGTAACAGCGGGATGTTATTATTTTTTTTTTGTATTACAGCAACTCCACCGCGCGATACGACGGGTGCAAAATACAAAGGAAATACAAGAAGGGGAAAATAAGGAGGGAGAAGAGGCAGAAGGGAGGTGTGAATTTGTATTTATCCCCCAGTAGTCCTTTTTAGGAGTAACCTGGGTGGCATCTGTTATAAGACGGCGAAATGAAGTGGATAGGGAGATGTGACAAGGGAATATAAGAGGTATAGTTCTGTTGCGTATATTTGTGTGTGTATGTGCTGGACCCAATGGCATGGGCGATGGTGGGCTCGTTTACATGACCTTGTTTCTTTTCTACCTGTCCGTCTTCTTTGTTTATTTATTTTTTCTTGTTGTATGTTTTGTATGTGTGTGTGTGTGTGTGTGTTTGTTTGTGTTGGAGACTTGCGTACAACTGCTGTTTCTTTCTTTCGGTTTGGatgtgtgtttgtgttcttggcttttgtgtggatgattgcgtgcatgggtgcctgtggtttctccttctgatcatcttcctttccttcccagtctcattcctccccctcactaccatctttactgttgttgTATTTTTTttttctttcttgtaccgcctgaagtttcaagtgggcatgttggctcggtatcgtcacggcaggtggtgtttccgttctccggtggttgtagggggataaGAGAAAGGTTGAGGGGAAGGGGTAGAAAGGGAAGGAAAAGGAGAAAAAAAAGCTGTAAGGAGAAGGTATGCAAGTAATATTAATATACATATATATATATATATATATATGTATGTATATGTAACATTTAAATGTTTTGTTCTGTTTTCTTCTTGGGCAAAGCGGTGGAGGGGGAGGTAGGTAGGTGTAAAGACAAGGAAAGCAACCACATCAAGGAAAAGTGAGTCGGTGTGTGTGATGGAGGGAAGAGAAGGGTAAAAATACACCCCCTCTCCCACAGAGAGGTTGTCGAGGGGGGAAGTGAGATACATTAAGTTTTTTTAATTTATTGTTACGTAATACGAGAGTGGTTTTAA

>Tb927.7.2180

ATATTGTTATTATGTCCCTCCCCCTCCCCCAACAGATGGTAACAACAGACGTTCATTTTATGTGTTGACTTGTTTGAGCTAAAACGACTTCAAATATTCACTTTTCTCTCCGCGTTTACGCCATTAACGTTCGCTTTTACTGTTTTTCTTTTTTATTTTGATTCGTGTCCGTACCCTGTTGCTAACGTGGGAAAATTTCCCTCCAATTCTAATGGCAAGAAACAGAGTGCCTGCCTAATGGAAGGGGGAAGTTACGGAATGAACTCCCTTACCCTTTGCCGCTGTTTGACAGCGGGTGCTATAATAATTATTATAATGACATTATTATTATTATTATTATTATTATTATTTTCTATCCTTTAACAATTTCTTTTTTTTTCGTTTGAGGGCATTGCCAACTGCAGTTGTGCTTACTATTGAGGATTCACATGAGAGATATGTCAGCACTTGTGTGTATCTGTTCGAAACACTTTTGCTTAAGACATCCTCATCTCGCCCATTTGTATTTTCTCGTTCCTTCACAACGCCTTGCCATGTTTTATTTTTACTCTATTTTTGGTTTTACCCACCCTTTATCTTAAGTTATTACATCTCTGTCTTAA

>Tb927.11.10920

AGGGAAAGGGGGAAAACAAAAAGATGAGAGCAGGTAAAGAAATACAATGGACCCAGTGTTTATACAGGCATAAAAACATATGAAGAAACGTCTTTGTGTGTTGTTCTGTTTTCTATTTTATTTTTGTTTTTAAACGAATTAATAAATATATTAAAACGTAATCACCAAGGGGGATCGTTTTTGCAGGGTGGCGTTTTTTTTCTTACCACACACCATTGAACGGAGGGTGATTTGGTGGGTCTCGCCCGCGCCTTGCCTTTTTAAAAAAAATTAAATATTTCCTCTAATTTACCACACTAATCCCACCTTTTTTTTTTATTTCCCTCTGTCGTTGTTTCTAGTGAAAACTTGAAGCAGGGGAATTTCCCTTTCTTTCACTTCCGTTTATCTTTTTCACTTTTACTTAATAAATAAAAAGACTTTATTGTTTTGATTGGAGCTCCTCTTTGAGAGGTTAAATCGGCCTTCCCCACATTGTGCCTAGCTGTGTTACTCACTTTACCTGGTGATATTATTTGTTTGTTATTTACTTAGAAACGTCTTTCATGCGGTATGAACCTTGTGTGAGCTTACATATGTGTATTACACAGGGTTGTATCTGCTCATTTAACATAGATTAAATAATAATGAAATAATATGGTGCATTAGTGGGTATTGAAGGTCGAAAAGTGGGAGGGGGAGGGGGAGGAGGGGCAAAAACTAATGTGGGATCGACTACATTTAATTGAAAGAAAAAAGCAATTGCCTTGGCAATTCAAATCAGCAACAGGCATTATAGTTTGTAAACGTAGACACGTTCCCATGTGTGGCTCAATATATCCACTGTCACTGATACATGCGGGGACGTGTGATTTTTTTTTGAAAATTTGTTTATATTTTGTTTACTTTGATCTCGTCACTGCCTACCGTTGTTAGTGAAGAGAAGAAAGGAAACAGAGAGTGTCTATACAAAGCATTGAACAGGTATGGATGAAAGGTGAGGCGAAGGGAACACAGAGCGGAGTTTGTTGGTACATCACGCTGTGCATGCGCAAAGCATCAAGTAAAATACGTTGGCTTCATATATCCGCGAGCCGCTTGACAAGAGGATGTCCAGAAA

>Tb927.6.2850

ATGTGCGGCGTCACTTCTTTTGCAGTTACGCATATTGGGAGGGAGAGGGAGAGGGAGAGGGAGAGAGAAGGTTTCAATGACTGCAAAAGTCCGTTAGTTTATAATTATTGTTGGTGGTGGTGGCTTTTTTTTTTTTTTTTTGCTGTTGTTGGTATTGTTGATGTTGCTTTTATTTTGGTTTGATAAAAATTTTTTTTTCTTTGAACGTCATAAACAGTACCCAGGACGAAGGAAGTAAATGCTTAGAAGGGAAATGAAAATAGATAACAATGAAGTGGAAGAGGCAATCACGAAATGAAAAATTGACGTTGAAGAAGATTCTAAAAAAAAAAAAGAGAAAAGGGGAAATGAAGGAGGTAAAATGTGAAAGAAAGAAGGAAGGACATTAAAATAAAAAAATAGCAGCGGCTCTGAAACTCACGATTTTGAAGTCGTTTTACCCTTGTGGAATGCACACACACACACACACACACAGAAAATATTCCAGCAGGAACTCTGAGAGTTGTGAGAGGAATATGTTTATATTTGTTTATTTGTTTTTTTTTTGGAGGGGGGTTGGAGGCAGAAGAAAGGATGGAAGTTCTTAAATTGTTCTTCTCTTCTCTCTTGTACTTCCTCAATATATACATATGACTATCAGATTCTCACATGCATATTAATGGAAGTCGTGAACACATTAGAAATTTAGCTTCATACTTCTTTATGCGCATTTTTTTTTTTTGGGGGGGGAGGGGAGGGGCGTTGTTGTTTTATTGGCGTGCACTGTAATAATTTAAATATAATCCAGTTACCGAAATGACAACCACTTTTTCTCCTTCTTTTCTGCTATTAGTTTGGTTCATCTTGGGTACATTTTTTTTTTGTCCTGTTTTACTTATTTACTTTTTACTATTTCCGCTCTTCTTCTTTTTTTTTCTAAATCATTTCTCCTTGCTGCCATTTTCTTTGGACTTACCCGTCTGTCCATATTTATATTATTTATATATTTGTTTGTATTTGCACTTTAAAAAAATTTTAAATGTTTTTTCTCCCTGTGCAGCAGCACCTTTTCCTTTTATGATTTTTTTTCTTCCTTCTTTTTTTTTTTTAAAAATTTTTACCTGTTTGTGGTTGTCATAATTAACCATTAACGCCATAGAGCCGTCGCGGCGCTCATGAGATCTTTCGTTTTTCTCCCTTTTTTTTGTTTAAATGAAGTTTGTGGAGAGCTTTAAAAAAAAATTGTAGGGGATTTGAGCACCGCGGCAGGCCAAATTAAAAGGAGGGACAAGACAAAAAAGAAAAAAGAAAAAAACATAGAATTGTAATATTAAAAGAGTGACGGCGAGCCATGAATAAATAATAATAACTAAAAAGAAGGAGATGATTTTTTTTTTATTTAGAAAAAAAATGAAAACAAAAAAGATAAAACAAAACTTCTTTTTTACGACCAGCGGATACGAGGATTAATACAAGTGAAGAAGGAAAGAGAAAGAAAACTGGGGAAAGAAGGTAAAATTACAAAGAAAGGGAAAAGGAAAGGGAAGACAGAAGAAGAAAAGAGAAGAAATGTGCTACAGCAAACATAAACAACCCTCCAGGTAATTCGAAATTTATATGCATATTTGTCTTTTCTTTAAGTTTTTCTTCATAAATTATTATTATTATTATTATTTTTTTCATACACGAAGAGGAATTGTAATTGTTCTTATAGAGGGAAAACAACGCCACCACTTCCATGAAACTTAAGAAAAGATAAAAACAAGTAGCAATTTTTTTTCTTTTTTTAAATTCTTCTTCCCACATTTTTTCTCCCTATATTCTTATTTTCTTTACACTGTTTTTATGAGGGAACTCGTAACTTGTCGCTGTGTTTAGTTGTTTTCATTTGTTTCGTTCTGTATTAAAAGTTGTTGATTTTTTTTTGTTTACTCTTCTCCTCTTGCAATTAGTGATGTTGTTATTTTTGATAGCCCCGTCTTTGTTTTGTTTTGTGTTTCTTTTTTTTTTTCCTCCTCTTTCGTGTTTCATTTTTGTATGACAACTCGCTCACATTCGCCGCGCCGCTCTTCAGAGAAACCTCTAGTGGTGGCATGTGCAATGTTAATGCCGAAGAAAATAATATATAAATATTATATATATTATTTATATATTTGTATATGTAAAAGGAAAATTAAATATGTGAGATAAAATCAAAGATGAAAGACGAGGTGAAATTAAATAGGACAGAAGAAAAAAGGAAAAAAAAAGTGTCATCGTCGTGATCACAAGAATCATCTTTGATTAATGAGTAAGTAAGCAAGTGCGTGTTAATTAAATTAAATTTTGTAACTAATATATAACAAGAAAATGAAAAGAGAATACTTGAGAGATGCCAGTTCATTAGATTTCACACCATTTTTTTATTTTTATTTTTATTTTTTCATTAGATTTTTTTTTCATTTTAGATGCAAATGTGGAGAAAAGCGGAAGGAGTCGTACTGCCGAGTTGAAGGTTTTTTGTAGTACCTTTTTTCTTGTTTCGTTTTTATTTGAAAGTAAATTTGTGGTTCTTTCCTCTTATGCTCTTGTGCTCATTTACAAATCCTGTTACGGTATAAATTTTTTTTTTTAAAAAAAGAAAGTGAAAAAAGCAAACGGAGCAAATCAAATTGACGAATGAAAGAACGAATAAATGACAACGAAGAGAAGAAAAAATAAAAAGGAAATTCGTGAACATATGATAAAAAAAACTAAAAACAGGGGGACGAAAAGCAAAAAGAAAAAAACACGTCAACATAGACAAAAAATACATAGGCAAACATACACGTCGATATTATATAAATATAAATATATTTATTTATTTATTTATATTTATATTTATAGTATTGATGGTTGTATGTTTGTGTGCGTTGAAAGGGAGTGAGCGCCGATAAACTCCCTTTTGTGCGCCTTTTTGTTGTTGTTTTTTTTTTCCACATGTTTGTGTTTTCGTTCTCTCACAGTTCTGTTTTACCTATTACTCGTTATCAGTTCACTCCGTTAGATGTTATACCGCTTATTTATTAATATAAATTTATTTCCCTTGCATTTCCCTCATACCTCATTTCTTTTTTTCTTTTGAGCTCGGTCGCATGTGTGAGTTTATTTTTTTTTTGTCGGAGCTCAATGAATGAAAAGTGAGGAGGAAAAGGCAAAAAAAAACATATATATATATACTTATTTAATTTATATATGTTTTTTTCCCCTCTTCTGCTTGGGGGTATGTGAGGGAATTTCACCTTCCTGTTTCTGCAGGAAGTAAACCGCATTTAGATAAATTAAAAAGAAGGAAGAATAAATGTTTGAATCGGTGAATGAACGAAAGAGTGAAGAAATGGAGAAGTTATGGATACGGAACAACACTGCGCATAGAAATTTTTTCAATAAAGAAAAACTTTTCCACCTCCCCACCCCTTTCCATCATTTATTTCCTCTTTACTTTTTCTTCTTCAACGAAATTTGTTAAATGTGTTATTGTTGATATTATTACTATTACTATTACTATTATTGTCATTATTGCTATTATTATTGTTGTTGTTATTACTGTTAATGCTGTTACCACTTATGTTGTCGTTATTATTATGTTTTTTTTTTTTCGTTTGACCGCTCATTTATTACTGTTGAGGTATTTCCACCTTATGTGAGCGAACGGCTTTGATAAAGTGGTGAAAGTGCCAAGAACTGTAATATAAGTTTTATTATGACAGTATTGACAATAATGATGACAACAACAACAACAATAATAATAATAATTCATATGATAGTTGTAACAGCAATAATTTGTAACAACAGTTACAATATTGACAAATAAGTTGATTTCTTCCATGGGACACACACACACACACACCTCTGTTTTTTTTTCATTTATTTAGGCAATGAAGAGACTGGTGGTATGAAAAAAAACAAAGCTGGAAGGAATGAAGAAGGAGATAAAAGAAAAAGAAGAAATTAAACTGGAAAAAAGGGGTTTAGATAATAAACAGTGAAAGTTCCACCTCCATAAACGCACCCACTTCCTTTTCGTATTTGTACGTTTCCTCCTCCCTCTCATTTTTTTGTTTTAAATGTTGTCGTCGGTTCTTTTCTTCTTCTTTTTTTTTCTCCCCCTTTTTTTTCCCCACTCCTATTCTCTTTCTTGTTTTTTTTTGTATACTTCAACTTAAGCAAAGTAAACGAAAAGAAACATTTCTCCATTTTTACTTCCTCGTGAGTCGTCTTTTTTTTTTCTTTTGTGTTGTTTTATTCGAAACCTTTCTTCCTTCAATTCCAATGATTTCCTCACTTTAAAAGTGAAAGAACAAAAAAAAAAAGAGGAAAACGTTACCAAATTAAGCATGATCAAAACAAAAAAAGAAAAGATACCAAATATATAAACATATACGTATACGTTGAAATCATGCCTGCGTAAAGAAGAAAAGGAGGAGGAAGAAAGGGGAGAATAATTAAAGGGGGAAAAAAATAAGAAAAGAAAATAATAAATGGGGTGGAAAAAAAAATTAAAAAGAGACACATGCAGTTGAAGGAAACACAGAAATGTGAGCTGGAAAAAAGGAAAAAAAAAAGGAATCAAAAGAGAAAGAAAGAGAAAAAAAAAAGAATATGTAACGACCGTATAGAAGCACCGTGACGATGAAGATGATGACAATAATAATACACATCTTTGTCCCCCTACCACCCATACAAAACACTTTAAAACTTTTGCCAGAATTTTTTTACTTTTGTTTTTTTTTCTTACATTCATTTATCTATTCTGCAGAGAAGAATCTAACTATTTATTGAGTAGATGAATAAATTATAAGAAAAATGACAGAAATGATGGGAAGAAATTCGTTTAAAAAAAATTGGCGATTGTTTTTGTGTTTGTCTATTTACTTCTTTTTTCGTTATGTTGGTGGCGTTTTTGGTGCAATTTTTTTGTTTCATATAAATATGTTTTTTTTTGGTTACTGTCGTTGTTATTGTTGCAAAGGGTGTCGCGTGAGTCAGTATATATATATATATATATTTTCTTAGGGGTTCTCACTGTGCGCGCCTCGTATAAAATATAACGTAAAGTTAAAAAAAAAAAA

>Tb927.11.14240

AAGTTTTCATTTGGTTACTTCTTAACACTGCATAAGCGCGTTGTGGGGAACGTCACAACGCCGAGATAAGCACGCGTGCGATGTGTGAAAAAAAAATTGTCTGTTTGATTTGTTGTGCGCTATCACTCACTGCAAGCACTGCTGCAGTCACGTGCATTTTATTTTTACCCTTGACCGTAGGGGGATGTTATTTTTTTTTTCTTGCTGTTGTGTCGTAGAAGAAGAGAGGTATGTATAGAGCGGTGGAGGAGACTGCTTTTCATTTTATCGGATCCATGGGTTTGTTGGGATAAACATTAACTTATTTTTTTTTATTCTTATGCCCTTCACCAA

>Tb927.1.1840

GAAACGACAGGTGAAGGAACAGTATTAAAATAGAACGAACGAAAAAAAAAAACAAAAAAGGAACAGAGGGAAGATGTGTCTGGGGGGGAGAGGGGGAAAAAAAAAAAGAAAAAAAAACTGTGATGTCAACCGCTGGTATTCCCAGTTTTGCTCACAGGATGGTATCAAAAAAAATCACACACGTATGTGTGGCAGCAGAACCTCCATGACTTTCCTTTCCTTCCTATTCCATATCGTATATATATATATATATATTCTTCTTTCTTTTTGTCAGCCTTCCCGCCGGCATACAACTGGAGCAAGTTTACTTCCAGACCACTTATTATATAATCGACAGTAACATCCCTGTATCCGTTGGGCACGTGCGTGCCTGTTTTTCATGGCGGGAACAGAGGGGGGAAACAAAGAAAATAGTGGTAATAATAATAATAACAATTATTATTATTACTATTACTTTTCTTTTTCATTAAGGAGAACCAGTGGGAGATGTGTACAATATTGGGGAAAGTTTGTGGTTTGTTGTGAGCGCTGTGGATGGCGGGAAAGTTTTGAAGTCCAACAAGCGGGAGGAAAGTAATGGGTTGTTGAATGTGACTTGCAGAGGAAAGAGGAAACTAAATAAAATAAAAGATATAAAATGAATTAAAGTGAAGTGATGCCATGTGATGTGATGTAAAAATGAAGAAGAGTAGAAGTTAAACAGGAGAAAGGTAAAGAAAGTTTCTTTTTTTTTTCCATTGGTTTGGTTTGTTCGTTTTGGAGGGTTAAGTGCCAGCAAAAGGAAAAGTAAAAGAAAACAAAAGAGGGAGAACGGTGACTTTTAGGATAGGGGTGATGGGGAATTACATTCAGCAAAAAATTAAAAAAAACAGAAATAAAAAAAAAAGTAAAAGTAAAGGTGAATGTCTGACATTACTCCGACAGCAGGTTCCGCTTTCTGTTCCCACACTACACCGTCTTATAAAAAAGAAAAAATATTTTTATACTCAACACAATGCAGTCATCGGATTTTCCGTTATTCTTATTCTCGTTTTTTTGACTTTTTTTTTCTTTACCCTTTTTTATTTATCTATTTGACTTCTCTTCGTTCTCATGTGGCTTGTGTATTTAAATTTCGGCCTGACCAGGTCTAAAAAAAAGAAGAAAAAAGAAAGAGTAAAGGATTAATTTCTCATGTCTGTAAGTGTGTGTTTGTGTGCCTGAGCCGATGTCTTTTTAATTTTTATTTTTCGATTTGGCATTCGTCTTGAAACTATTGGCTACTGCACGTGACCAACGCGCAAAACTCTCTCTCTCTCACACGCACACACACACACACACAAAAGAAAAAAAAGGTTCAATGGTAAAAAAAAAAAGAAAAAGAGAGAAAAAAATAGGAGATGAGAAAAAATAACAATGAACAATAATAAACGTGCATTGCATCATTATTTTGTACGTCTCGCTTTTTTTTTTTGTTTTTGTGCGTTCGGTCATCCTCCTGTTGTTGTTATTCGAGAGGTCAAATGATAGGAGTTGGTGATGGTAACAACAATAATATAAATAGTAATAATAATAATAATAAAAATAATAGTAACGATAATAATAAAATTGATGATGTTGAGAATTTGATTGCGCTGGTACTTGTAGCTTATTTATTGTTATAACCGGTTCTGATCTTCAACCAACCACCCGCTCGTGGGAGGAATCTCACACGTCAAATATTTAAATGTTAGTACAAGTATATTTAAAAATACAAAATATATTGAATGTATAATATATTCTTTCTCTCTTTTTTTTCTTTTTGTTATTTTGTTTTGTGGGAAAGGGTGTGATATTCTGCACAGGGATGAGGATCGTCTCCTCCTCTAGTGCTTTGTTTGTTTGTTTGTTCGTTATCTTCTGTTTTTAAACTTCCTTTTTCTTCAAAAAAAAAAAAAAGAAAACCCCTTCACGGTCCACTATTTCTTTCATATGTTGTTTTCTAAGACTCTTTCCTTACCTCTTGCTTCTTCACTGTCGTTCTTGCCGTTGTCATTACTCCGGTTTGTATTTTTTTTTATGTTTTTTTTTAAAAGAAAAAAGAGGAAAAAAAAAAAGAGACGGCGAGACAAGTACGTGTGGAGATGTCGCGAGGTTAACTGACAGTGTTGCATTATTATCTCTCTTTTTGTTTTTGTTGTTGCTGTTGTTCATTTGTGTGTCTTTATGTGTGCCTTCGGTTTGCAAAGGGGAGGGGGGAATGATCGCTTTATTTTATTTTATTTCGAATAGATGAACATATATATATATATTTTTTTAAAAAAAGAGAGTAGGTTTTGTGTTGTCTGGGTGGTGGTTCTCCGTGTCGTTCTCTATCTTTCTTTCTCCCTCTCTTACCTTTCTGTATTCTGTCGACCTCTTGTTTGTTTTTGTTTTTATTTCTTTCCTACGGGTGAACGATTGTTTATTTTATTATTCAAATTTAACGGACTTTGAGTATGTCTTATGTGTTAATCTTTTTAGTTTCGTTTGTTGTTGCTGTTGTTGTTGTTTTGTCTTTTGCTTTACTTTATGTTGTTGTTATTGGTTGCTGCTATTTATTAACCTACGCAAACGCACAGGTGGTCTCGTTTTCTCTTCACAAGGGTTGGGCCAACTTATTTGATTTTTCTTTTCTGCATCATCGTCATTATTAGCACTGCAAATGCAAAAAAAAAAATCGAAAAGTAGGAAACGGAACAATATATTGATTATTTTATTTAATATTATGATAAAAAATAGGAGAAACCGTCAGACGCATTGGGTGGTAGCGGTGTGCGCAGCACTTTTTTTTTATTTTCAATTACCCTCCTCCCTTCCTCTCCCCTCCCTTCCCCTTCAATCTTTTTAATCTTTATTTTTAATAATAATAATTATTATTATTATTTTACCCCTTTCGCCACATTGGCCCCTGCACACACACACACACACACATCCACCCTTTTAGTTCCCTCATACAAAAATAAATGTGTCTGACTTGTGTTTTTTTTTGTTGTATTCCACACTTTTTTGAAGAGGATGAAAGGAAGAAGAAGGAGGAGGGGTAATGCAAACCAGTGTGTTAACACAATTCACAGAATCGGAGACGAGTGTTTGTGCGTTGTAAAATATTCCAGTAAATAAATGAATAATGCTTAAATATGTATATATACCTGTTTATTTTATTTATATGTGGATCGTTGTGTGTCATGATCCGTGAAGCAGAGCGTAAAAAATTCGGTCGTGTGTGATTCCAGTTAAAGGGTGACCCTTGCAATGATCAAAAATAACAATAACACTAAAAAGGGTAATAATTAACAACGACAAGAGCAACAAGTGTGCATGCGGGTGGGGAGAAAGAGAAGAAGCAAAAGAAGGAAAAGAAAAGAAAAGAATGCGTTGACATGCATACACACACGCATATATTCATATATATATATATATATATATATATACATATATAACTTCAATGATGAGGTATTTGCGTGTGTATATGCGTGTGAAGAGGGGAAAATATAGTTATGATTATTATGCATTGTACTTATTTGCGGCCTAATTTCCCTTTCTCGAAACACAGTTCGCTGAATTTGGCAGCTAAAA

>Tb927.4.3320

AGCGAGCGCAAATGGGAAGCGAAGTGAAAAAAAAAAATTGAAGTGGAGGTCAATAAATAAAAAATAAATTAAATAAATAAATATATATATATATATATATAAAGAAGAAAGATATGGCATATTGCCTCTGTACATAGAAAAAAAGAGTTTTTGTTAGGAAAGAGAGGAAATTAAGAAAAAGAAAAAGAATACGAAAAAAGAAAAAATAGAATTAAAAAAAAATGGAAAGAATGAAAACAAAAACGAAACCCCCCCCCCCAAAAAAAAAACAACAACAAAAAGAAACATGATAAGTCAAGTCAAGTCAAGACAAGACAAGATAACAAGATAACAAAACGACAATGAAGGAAACAACAAAAGAAGGAAGGGGAACGAAAAAGGGGGAAAAAAAAGAGAATGGTGGGAATTTTGACGTGTGAGTGGAAGTGATTACAGCACGCAGTTGCACACATGTGAGATTTAACATACATATATATATATATATTCATATATGTTTATTTATATATGTGTGTATTTGTAGAGGGAGGTGGTAGGCATTTATGTGTCTGTGTTTTAAACAAAAAAAAAAGTGTGTTCAACGAGTGGCCAAAACGTAGAGATGGATATAAGTTTCACTTCACTCCATTCCTCCTCTTTATTTTTCTTCTTTATTGAAGAGAAAAAAGTTTATCTTGTTTTTATTGTTTTTTTTTCTTTTTATTTGGCCGTGACTACGGTGAGGCCCGACATGACCGTTAGCCAAAGTTTTTTTTTTAAAAAAAATTACGCGCTACCACTCCTTCCCCCCCTTTTTTTTGTTTTTCCCTCTTTTTCCTTTTTTCTTCTCTTCTCTTTTCGTTTTTTTTAAAGTTTTGTTTACGTGAATAAAACAAAATAAAAAGAAACAAGAAAGAAGTGGTGATGACGTTTAGTTGTGCGAAAGGTTCAACGAGCGACTGTTTATTATTATTATTATTAATTTCCATTCTCCGTCTCTTTCTTTCCCCTTCCTTACGTTTATGTCTCCTGTTACCGCGCGCCAGTTCTTTTTGCCCCCGTTTTTCTTTTTTTCTTTTTTTAAATTCCTTTTCATAATTTATTTCCCCCTCAACATCATTACGGGAAACTGTACAACAACGGACAAGCAACAACAGGACACAAAAAATAAAAAAAAAAGAAAAAGAAAAGAAAAAAATGCACACAAGCACACACACGCACTTACATATATACTTACATAAATATATACGCATAGTGGAAGGTCATAATACTCATAAGCAGAACCTGCTTCTTTTCTGACATGTTTCTTCTTTTTTCCTTATTTTTCTTTTTCTTTTTCTTTCTTTGTTTTAATTTCCTTTAATTTGTATCGCCGACGCCGCTGAGCAACCGAATGATCAACTACGAACTTCTTATGGTGTGGGGCCCCCGGGAAATGGATTGTGATATGAATTGTGCTCCTCTTTTTTTTTTTCTCTTTTAAAAGGGGGAAAAAAAAGATATAAAAAATATATACATTAAAAAAAGAATGAATGGTGGAGACATCATCACTCCTCCCTTTATTCTTTCGGCTTTCAGCACTTCGTAGTCTGCAGTAGTTCCATTTCCATTTCTATTTCCATTTCCATCTTTATTTTATTTTATTTCATCTCTGTGCGTGTGAGGCCACGTACGGGGAGAAGTGAAAAGGCTTGCGTGTGCGGGTTTTTCATTCCCCATAAATTTTTTTACTCTTTTTTTTTTGTTTCGTTTTCCCTTTTCAAAGGAAAAAAAAAGGTTCTGAGTGGGGTCGTGCCACATTTAAAACGTATACGTATCTGTGCGTGCGCGCGTATATGTGTAAGTGTGTGTGTGTGTGTGTATGTTTTGTATGTGTAATGTACGCGTTGTTATTATTTTCCGTTAAAA

>Tb927.6.2750

ATGCGGAAATGAAGAAATAAATGCGGCAGTGGAACCGTGCGTTTCGGTTAGATTGGAGTGGTGCTAGCTGCGTTTTCACTCATCGGCGCTCATTATTATTATTGTATTTTTAATTTGAACGGATGCACTCTTCCTGTACTCTTTATTTGTGCGCATCAGCATAATCTGTCGATGGGTCTGATATTCGGTTTATTTTTTTTTAAAATGTAAAA

>Tb927.10.9270

AGCATGGCTCCAACTCTCCACCCTGTGGATGAGTATTTAACGTTGAGATAACCAATTGAGGGGCAGTAGAAAGCGCTTTAATACCACTCACCACCAATTAAGATGACGAAACCCCTGTGAAGCAAAATAGGGCAGGGTGGCCAAAAGAAGTTATATTCAACAAACAGGCAGGGTGGAAATGCAATGTGGTTGCAGTAGAAATGCGGAACGTTAATGGGAAAGACGAAAGGGATGAGTACTCGCAAGTTGCGGCGGTGAGTTTGTGGTCTCTGGGGCTCATGCTGTGTGTGATTATTTTTTCTGTGAGCTTGTTATTCTCTTCGGTGTAGTCAAGTTGTGCTGTAAGTGCTAAGATTTGTGTGCCTATGAACTCTTTTTATTTTCGTTTTGATATATATATATATATATATATGTTGTTATTGTCATTGTTTGTTATTTTGCTGTACTTCTGGTTCCCTTGGCTCACGTAACGGTGGGATTCAAGGGGATAAGATGTGGCTGCAGCGTATGTGCCCAACTGTTCACTGATCGGCTGCGGCACTAACGGAACTCGATTATGAAATTTTATGTGCGTTGCGTCTGCAATTTATGCATTTTTTTTTCATCGTATTGCTGCGTGCAAAACAACAACAACAAGGGATCAGAGAGTGTTTTGCACGTGTGTATTAGTGTGAGTGGGCGCCTCTGTAATAAAAAGAAGTGACGAGTTTATACCGGGTGTCACTGCCATGTTTTCCTCGTCTTGTATGCAGAGCATGCAAACACAATACATATGCCCACATGCGTAAGTAAATACAAAAATGCATGTGCTTATTGTTGGTTTTACGGTGTGGTATATATGGGAGGTTGTGCGTTTATATATTTCTGTGTGTGTGTCTTTTCTTTGTTATTATTATTTATTGTTATTTTTTTTTTGTTTATTCCCTTGCATGTCAGTTGGGTCGCAT

>Tb927.9.10630

AGTATTGAATGGATTTGATTGTGTTCGAATCCGGCGACGTGTGTGTTTGAGAGAGAGAGAGTGACAGACAAAGAAAAAGGTAAAAATGAAGTAAAGGGAAGGAAAGGACACTGGTGGCACGTCATTTTAGTGCTGCAGTTCCTTTTTTTTTTTGTCGTGGAAAATGACTGCGGTGAGTCGATTAATGACAGTTACTTCTCCTTCCTGCTGCAGCTCCGTACTATCGCCATTTGTGTGTGCATGTGTTTTTTGTGCGTGCTTATACTTATAGACGTTGTCACAATAACAACGATGGCGAAAACAATAACAAAAAAAACAAAAAAAGAAGCAAGCAAACAATGTCGCAACAACGTTTTTAAATAACAGGTACGCAACGGCTCTGTGAATACAGACTCGAACATCGTAAGTCAACAATGTTTTTTTTCTTGTAGTTCTCTTTGGTTGTGCGTGTGTACCCGGTCAGGGATTCGTGGCCCTTTTGCGTACTTTAGATTTAAGGCGTTCCCCAATACTTATCAACGACATGCCATTTTTCTATCTTTAAATATTTTTTTACCTTTTTTATATATTGAATGTACAGTTAGACGCGCTGCAACGGCAACTGAGTCCGTGCGTAGCTACTGTATAAAAGTCACTGTTACTATTATTATCATTATGCATACAGATCATATATATATATATGTATATATGTATATGTACTCGTTTTTATTGTCCGTTTGCGCTTACGCTTATGTTTGTTTATATTTGGGAGTGGTCGTCTTTCGGCTCTTGCAGTGAGTAACAATTTATGTACGGTTTCGAATTTTGTTTGTTTTCTCAGTTACATTTTTGTGTGAAAACAAACGTAAACTTTCATACATGAGTGTATGTTTGTGAAGGTCGGTGCGTCATTAAGCGAAGTAAGCTCTATGATCAATTAAATTACCATCGGTATTATATCTTATTTCTGCGCTTTTTGTTGAAAAATTAGAAAGGTGTGTGTTTACTAATACTACTTGCTCAAGTAATTATTTTTCCAGTTAGTAATGTGAAAGGGGGGATTCTTGTGTATATATTTATATTTCTTCCGTTAAACGGAAAGAAGTAATAAAAGAGAAATTAATTAATAAACAACAGCAACAAAAAAAGTAAATGGAGAAACGAGCAGTGCGCATGAATTGTTTTCTCCCTCTTTTCTTCCTTCACTTAAAAAATTCCCCTTCCATTTATCTTCGTTGTTCGTTACATCTTCTCTGCCCACCTTTCCTATTCGCTCCAAACTTGACTTATTGTACCACACGAATGCGTGCTTTTGTGAATGTACATAAGTTTTAAAACTATAAGGAGTGGAATACAGCGGCTTTACCCCATCATATTTTCCCTTTCCCTTCCCTCCTGTGCATGGGTGTACTTAATTAATATAATAATAATAATAATTATTATTGTTTGTTTGTTTGTTTTGTTTTCTACTCTCTCTCTCTTTTTTTCTCCCCTTTCCTCTCCTTCTTGTTCTTTCCCCTTTTCGTTACATTTTCATCCTCATTTTTTTTTCACTTCTCTCAATCTTTTGTTACCTCTTTACCTCCTTAAGCGGTGCCGGTTACTTCATCATCCCGAATATCTCATTTTTTTTTTCCAAACCTTCCTTATGGCATGTTCTTACGGATGACATTAAATATTTTAAATATTTTTACTCATTCTTTTTTTTCTTTCCTCCCCTCCCCTTTATTTTCTGGACACTTTTGGTGTTGTTGTTATTTTCTTCTTTTGTTTCTTTGTTTGTTTTTTTTTTCGTCGCTACCTGGTGGCTGCGATGATAATTCTTTTCCATTGTTAGAGAAAAAAAAGGATAAGGAAAAGAAAAAAAGAGTGTGGGATCAAAGAAACTATTGGGAATATATATATATATATATATATATGTATACGGTGAAGGGAAACTCGGAGGACATAAGGTGGAAAAAGGCGTGGTAATAAATAAATAGATATATAAACAAAAGGAGAAGTGGTTAGAGAGGGGGGAAATGATACGTGTGAATTTGCCTCGTGAGAAGGATTGTTGTAAAACGTTTCTTTTCCTTTTTTTCTTTTCGTACATTTGGCAGAAGGTAACGGAAGGCGAGTGGAATCGAAGGCTACGAGAGTAGAAGCAAAATAAAACAAATAAAACAGACAGACAACAAAAACAGTAGCAGCAGCAGAAGCAGGGGAAAAAAAAGAAAAATGGAGGCGATACGACGGTATACGATACGATACGTTGTGCTTGTTTTGTCTTGTTGCGTATATATATATATATATATGTCCATGCATTTGTATGAAAACGGATAAAAGTAAAAAATTCGAGTGTGATGAGTTGCTTCTTTTTATACCTCCCCTTCCCATCCTCTCGAGCGGTGTTTTATTATTTTTCAATACCCCCGAAGGAGTCGATTAAAAGCATCTAGTGTGTTTACTCTTTCGTTCTTTTAGTTTCTTTTTTTCTTTTTTTTCTTTGAAAATGAAAAGAAAAAACCTTGACCACTTACTATCGTCTATTACTTGATTTTATTTGTATTTTTCTTGTTTGTCATTTCGGTAATGGTGTTACCATAACTTTCGCCGCTACCGCTTCACATTTGGATTTTTAAACTTTCACGTAGAGGAAAACCCCCCTCTGAATGGGATACCGATTAAAGGGCGCGAAGACAAAAGGAAGGGGAAAAAGTAATAATAGCAATAAAACAAACAAACAAACAAACACACAGAGAGAGAGAGAGAGAGATACACAAGAAACTAATTTCTCACACACGCGCCCACAAATACGAAGGGTGAAGAAAATTTTTGAAAAGAAACGATAAAA

>Tb927.10.5880

GAATTGACTTTTGCACTGTTTCTAACATTATTGTAGCGTGGAAATACGTTTTCACTTTTTGTTGGTGTAGATGGTGAGTCACAAGTGAAGAAACATTTTTTTTTCCTTCTTAAGGTGGGGTATTGGGAGTGACGCGCGGAGGATTTCGCCACCATGAGGGCTGTGAATCGCAATAAAGGGGTAAGGGTTGCAGTGGAGAATGAAAAAGGAGAAACCACATGCAACTACGCTGATCCAGACGTGAGGATACTAGTGCGTTGGTTTTTTTTCTTTTTTTTTTCTTTTTGCGTGTGTTTCGAGGAAAGTCGAGAAAGTCAGATCAGGACAGCGGGACGCATAAGCGGAAATAGGCGTCGAGCAATATATGTTAAGGGACTTTGCCCACATTATCTCTTGCTTTTCTCCACTTTATTTCGTGTGTGGTTTGGACCCCTCTCGCGTTTCCATGCGAACGTTTTGTGTTTTTACCTAACGCAGCAAGCAACGCTAAAAGAAGCAAGTGAAATGGAAAAGAGGGGAGGAAAGGAGGACGTACCCGCCCGTAGTCCTTCTCTTTTTTTCCCCCTTCCCCTTTTCGCTTTCTTGTCATTAAAAACTGGGGGAAATAAACATTATGTACACATGACCGTGCGTTGCGAAACACATTGAAGGAAATGAATGACGAATGAACTTGAGGGAGGTAAGAAGGGAACGGGAGGGTTGGCAAGAAAAGAAAGAAAGAAAAACGAAAATGAGAAAGAAAAAAGAGAAAGAGAGAAGTAAAGGGAATGCTGTCATTTTTTTTTTTAAAGAAAAAATATATGGAAGGTTTGCAGCTCTTTTTTTTTTTTGTGTGTGACGTTGGCAGCCATGCCCATGCCAAGCGAAGTCAAGGGTGTGTGGGACGAACGTGACTGAATGTAAATAACAAAACAAAAAGAGGAGGAAAAACACTAATATCATAGCAACTGAAAGCGTGTGAAGAGGCGTAGGGGTGCTCTTGAAGGTTGTGTGTCTGTTTTCTTTTTTTTTTTTTCGTTAAAAAGAAGTGAAAAAAAATGATAGGAAAATATAGGGGACAATAGTGCGTTTTTGGAGTTATGTACTAAAATAACATACAAAAAAAATTTTCCCCTATCCCGCCTCTTTCCAACATACCAGCAGGAGGAAGCTTTAAAGTTTAGTGAGAAAAACATCATGATCATATGTAGGAGCGATGCGATGGCGTTGCGCTCCAGCAAAGGTGTTAAGTAGTGGTGGCGATGGAGAAATATATATTTATTTATACCTCTCTTTTCTATATTTCTATATTTATGTATATATTTTACCCCTGTACTTTTCAATTTTATTTTTATTATTTTTTAATTTTTCTTGATTTTATTTGTCGTTCTTTTCTACCCCTATCTTTTGTTTTTCTTCTACAGCACCACACAGCCATTGACAACCCTAAAAGTGGCGTCCGTTTTTGGACACGCCAACAACAAGAACTAAAACCAAAACGAAACTGTTTCCACTACCATCATTATCATTACTGTTATTACGAACGAGTTTGCATCACCCGAAGTCGCAGTTCAGGTTTGCAAATTTTCCTGATGGTGTTGAGTCCGAAACCGTAAAGAAATTCCATAAAATTTTGGTGAAGGAAGGGAAAAGGGAATTAATAAGTGAAGAGATGTCGACTTTTTAATTACATTTGTGACCCTATCTCAATGCTGTAAAGGGTTAATCAGTTACGGTGCTCTTATCACGTTTTCTTTTCTTCTTTCTTTTCGTGTGATGATGCTATTAGCGTTGTTGTTTGTCCTCTTCAATGTCCTCTGTGTAGTAGATATTATTGTTGCCCCTCGCATTTTTTCTTACGCTGATTCTCATATTGTTTTTTTTTTCCACCTTGATAGCAGAAGAAATAACTTTTTTTTTGTCTCCATATACCTTTTATTTCATACAGCTGTTTACCGAATATCTTAGAGAATCAGGTGCGATTGCCTTTGTGGGTTGTTCACAGTGGCTGTTTTCTTTTTCTTTTTTTCATTTCGTGTTTTATTTCTTACATCTCCCTTCCCTCTTCCTCTCTGATGTATTCCTGTTTATCTCATTCGTTGGTTAACTGCCGGTAATGGCAGGCGAATATAAATAAATAAATAAACATACATACATATCTATATCTATATATTTATATACTTATATAAA

>Tb927.7.4570

AGGAGGACGAGAAAGGGCGTTTCACCGCCCTCTTTCCAGCGACAAATCGCATGTCTCCGGTATTCGACGAGAATCCTTCTTTTTTAAAAAAGAAACACATCAAAATATATCTGTCATTTGGACCTGCGTGGTTCTGTGCGTGGGGCCGAGGTTACCGCAGGTAACGTGTGAACAGCTTATGTAGCTAAGTATTGGTGACGAGGTAAGGGAAAGGTGCGGGTATGTGTGTGGTTGAGGGAGGAGGAAGTCGCAAACGTTTATTTGTGTGTTTCTTTTGTTTTTGTTTTTGTTTTGGAAGGTCGATATCGAGTTTCCTTCTTAAAATGAAAAAAAGATATATATATATATATAATTTTATTTATTATTATTATTATTTTTTTTTTGGTTGAGAGAGCGCAGCGGCTCCTGGTAGTGCAGGCTGGTGGTCGAGCGTGGAAAAGGGAAAATAAAACTAAGTTTTAAAAAAGATGAAATGGTTGTAGGCACGGATGAGAGGTACATAAGTTCTGAGGCTCTGCTTTTGTCTTGAAAAACGGAAGGAGCAGAATTTAAAAATATATGTATACATATGAATATAAGAATGAGGGTCAAAGCGCTGCAGTGCACACATAAATGGGTAATTCGTAGGAAAGTTTTAGGTTTTTAAGGCGAAGCGAGGAGGCGGAAGGTGAGGTTCTGTTCGCGTGGGAGAATGAAGTAGAACCGGTAATGAATTCAACAAAGAAGAAAAAAAGAAAGAAAAAGGGTGCGGGAGGTTACTTCACCTTTGTCGCGATGTATGTGTATGCATGATTTTTGTATTTGTTTGTAAGTTTCGTGTGCGTATATCCACGCGTGCCTTCAAGTTTGCATACATCCCTTCCCCCATGATCAACTGAACTATATTTTTCTCAAGAGCACTTCCGTCGCTACGGCTACTGTTTTTGCTGTTGCTTCATTTGTTTTTTTTTCATTTTTCGCCGACTACTCTCCCCATTTAATGTTACAATGTATGTAGACCATTCAACCCCGTTCCTTTCCCTTGCGCATGTCACAATCGCGGTTTTACTCTTCGAACAAAGATTATCTCGGACTAACCGGGTGTGGGGCTTGTTTTTTCTTTTTTTCATTTTATATTGTGCTGTTTCCTTTCTGTAGAATCTGTCCCATTCCTTTTCTTCCTTCCTTGACTCTCCTTTTTTCTTTTTTTCCTTTTGTCGTGTGTTCGTTTGATGGTGACATTTCGCAGTATTTGCTGTTTTTGTACGGAATGCCGTTTGGTACTGTAAGGTATCCGCGGGTGTTTATTCATTTCGGTTCACACAACGGTTTTATGGTTTTGTTATGTTATGTTTTGTGAAAAGGATGAGTAAGAGGGAGAGAAAGGGGGAGGAAAGGAAAAGAAAGCAGAAGGTGAACTGACAGAAGCGAAGGGAATGCAACGAGAAATTGAACGAGTCCGAGAATTTAAATACTGGTAAGGACATAAAGCAGAAACGTTTACTTGTCTTTTGTAATGATTTAATATTGGCGAAGGAAAAGGATGGGGAAAAATCCTTGTAAGAATATATATATATATATATATATATATATACCAACCTTCTTATTTTCACGCTCTTTATCTTATCTTATTTGTCATTTCATTATCATTAATAGTGTGGGCGTGAGTTAATATTACAATTGGGTCCTTTGTTTACTTCTTTTTTTTACTTTATTTGGCTTCTCTGCCACGTGTATGACTTCTAAATAGAAAAACAATGCCTCGGAAAGTACATTCTCCTTGCTTCTGCGAAGTGATGTGCACAAAAACAATATGTATGTCGAATACAAATAAATTAAAGAGAAGTTAAAAGTTGAACTCAAACATATGCGCACACTCGGGTACAGAAATACACATATTGTATCTCCCTTTATTCCTCGCGAAAGATCGCGTGGGTAAATGGGAGAGGGATTTCTTTATAGAGAAATTTGAAGGAAAATAATCGTATGAACTATTAAAAGCGCGATGTGAGGTGGGTGGAAATGGTGTAATATATATATATATATGTGTGTGTGTGTGTATGTCTGTATGTGGAGGGGAATTAAACAGAATATGAGTTAAACACAACGTTACCCGATTGGATGTGTGAAGGTTGGGGAGCTCAGGCTATTAAAGGTCGGCATTGGTATAAATAATCAATAAACAAATAGGAGAAATATTAAATTTTGGACCCCATCACTACCTTGGGGCGGTGCATCGTGCATACATAGATGATGTACTACCTCTGGTGTTGTGATGCAGTCCTATCAACCGTCACTGTTACCTCGCCTTGTTCCTGCGACGTTAGGACTTAAAAAAAAAAAA

Tb927.6.5020

ACAATGTAATCATTTCAGCATATGAACCCATACATGATGGTAGTGTAAATAAATATGTAGGCAAATTTATGCAAATGTTTGTGTGCCTTTATGTATGGGTGTAATTCTCATTGTGTGTCTCCAGTCCCTTTCAATTTGTTTTTTATTTTTAGTTTATTTTTGTTCTGTGATTTTATTCCTTCCTTTTCCCTTCCCGTATTGTTCGCGCTATTAGTGTTGCACGTCTTCAAGTATTGGTAAATAAATAAATAAATATATATATATTTCTTTGTTATCATTATCATTGTTGTTATTATCATTTACTCACTGAGTTTATGTTTACTTTATTGTAGTGTTACCATTCATTAATTGTTCTCTCTAACATTTATTTACTTTCACTATTTTCATTATTTTAATTTTATCTTCCCAGGAGGTGGAGATCGGGGGTTGAATAGGAGGAGGAAAGTTCTGCAATAAACCTGAACTTTCTTTGTTGTTTCTCTTTTTTATTTAAGAAAAAAAAACTTTCCCCTCCCGTTCACAAACACTGTAGGGAGCGATGAAACCACTTTTGGCATTTAACAGAAATAGATTTATATATATATATATTTATATTTTTTAAAAATTTTGACCACCCTCCCTTACACTTATTTATCATCATCGGGGTTTTAATATATATTTTTTATTATCACAATGTAATTGTACCGTGAGGGGACTTACTATTATTTTTGAAATGCAGCTACTAAGATCGGATGCTGTGTAGAATATATATATTTTTTGGTTTTTTTTTTGAGAGTTACTCAGCCACCGCGTAGCATTGGTTTCCTTTTGTCTCTTTTTATTGTACCTCATATATATATATATATATATGTATTCGTGTATGTCCAAAATTAACAAACGAAATAAATGCATAAACAAA

>Tb927.11.1560

GTTGTAATTCAGGTGATGCCTTTCCACGCTTCCTTTTTTATCTTCATTTTTATCTTTATTTTTATTTATTTATTTTTTCATGTTTTACGTCTTGTTATTTTACGCTAATAACAATTATCATCACCACATGGACAAAGGCATGAAAAGAAAAAGAAAAAGTATGTCTGGAAGGGATGTGGTTTGCTCGAGTGGGGAAGGATGGAGTGGTTGTGGAGGAAATAAAAAACAAAGTGGGCAAGGTTGAGCTGTTTTTTTTTTTTAGATGGGGGAAAGTGGACAGATGCCGTACGGACATACGCGCACAACCGTCAAAATAATAATAATAATAATTCAACACTTTCATCACTTTCGGTAATGTTGAAGAGTAAAGGTATACAAGGTATTCATTTCTGTTGAGTCAAAACCGAAAAGAAAAAAAATACCATAAAGAAGGGGAGAAATGTGTGTGCATACGTTTAGCTCGCCTAACGGTGCATTAATACAGTAAAGCAGGTGCTGATGATTGTAAATCCAGAGCTGGTGAAAATCATTAAGGTTGAATCTGCTTGCGGTGGTGCGAGCACAAATGTCGTGGTGTTACTTCATCCGTACACCCATAAAAACATATAAATATATATATATTTATATGTTCATATGTTTTTATGTCTTAATGACTCATTACATTCAGAAATGTTTCATGCAATTTGGTGCTACTGTAGGCGTGTGTGCGTGTGTGTGTGGAGGCATCTTCGCAAAACATGGAGCGACTTTACTACCGAGAATCCAGTAAATAAGTGATTAAGGTGAGAGATGTACATGAAATGATGTTTAATGCGGCTCAATCAGTTTTCATATTGTCAGAGGAGCAACGTGTGAATGTGATGACCGTTTATAGAACTTTTCGGAATGAGAATTTTATAAATTGTCGTGTTGACATTCAACTTCACCGTGTCTCCCACTAGGTGGTGCGCGCTTGTGGCGGAACCACGGAAAACATTGCCTTATCATAATCTGTGTGCCATTTCCTCCGTGAAATAACCGCCTCTTTTCGATGTTCTGCGGTTTTACTGATTGCCCGAATTTTTACTCAGATGCTGTAATTTACATTCCGGTTCCTAAAGAAA

>Tb927.2.2140

AACGGAAATGTTTGCGTGTCTTTATTTATTTATTTATTTATGTTTTTTTCCCCCATCCCCCAATTTTATATTATTGTTATTGTTATTATTTTCTTGTTGTTGTCGTTTTATTGAGGGGGGGGAGGGAGGAGGCAAGTGGATGGCGCTCAGTTAATGGAAATGCGTGAGTAAATTAATTCAAGTACCGTGTCGTCCTTTTAAACTCACATGAATACTTATTTTGCAAAAAAAAAAACAAAAAACATGTTTATTTGGGTACATTTTGTTTGTATTATTTCACAACAGTAATGAAATGTACTTGCAGCAGTCATCGGTTCGCCGCAAAATAAAACAATAAAATAAAAAGAATTACACAAAACAAAGTGCAAGCGGGAGTGGGAGTATTTCATTTACATTT

>Tb927.10.5190
[truncated: 42,042 more chars]
