# Supplementary material for: Expression of the RNA-binding protein RBP10 promotes the bloodstream-form differentiation state in Trypanosoma brucei
Source: PLoS Pathog. 2017 Aug 11;13(8):e1006560. doi: 10.1371/journal.ppat.1006560 (PMC5568443; doi:10.1371/journal.ppat.1006560)
Supplement: S2 Text — (DOC) [file ppat.1006560.s002.doc]

## S2 Text

3'-untranslated regions of mRNAs that were less than 0.7x enriched in both RBP10 pull-downs.

>Tb927.9.3340 | Trypanosoma brucei TREU927 | hypothetical protein, conserved | genomic | Tb927_09_v5.1 forward | (geneCodeEnd+0 to geneEnd+0) | length=31

ACAGGTGAAAAAGTGCGCTTCGCTTTCAAAG

>Tb927.11.16160 | Trypanosoma brucei TREU927 | ATP binding protein-like protein | genomic | Tb927_11_v5.1 reverse | (geneCodeEnd+0 to geneEnd+0) | length=31

ATCTCTCCTAAGATCATGTGGTTGTTTTTCT

>Tb927.11.5470 | Trypanosoma brucei TREU927 | hypothetical protein, conserved | genomic | Tb927_11_v5.1 forward | (geneCodeEnd+0 to geneEnd+0) | length=31

GTTGTTCATACATCACGAATGAACAGGTGGT

>Tb927.8.7000 | Trypanosoma brucei TREU927 | E1-like ubiquitin-activating enzyme, putative (ATG10) | genomic | Tb927_08_v5.1 forward | (geneCodeEnd+0 to geneEnd+0) | length=32

GTTTTTTCGAGGCTGTTACCTCCTCGCTAGCG

>Tb927.11.7090 | Trypanosoma brucei TREU927 | hypothetical protein, conserved | genomic | Tb927_11_v5.1 reverse | (geneCodeEnd+0 to geneEnd+0) | length=33

AGTGTCTAAGCTGCGAGTATTTTGGAGTGGAAG

>Tb927.10.4970 | Trypanosoma brucei TREU927 | hypothetical protein, conserved | genomic | Tb927_10_v5.1 forward | (geneCodeEnd+0 to geneEnd+0) | length=33

GCGCGGTGTTAAAACGTGCCAGGGAACATGTTT

>Tb927.10.7670 | Trypanosoma brucei TREU927 | Protein of unknown function (DUF2962), putative | genomic | Tb927_10_v5.1 reverse | (geneCodeEnd+0 to geneEnd+0) | length=33

AGTGCAATGACGGAGCAAAATCATCAGCAGAGT

>Tb927.2.3420 | Trypanosoma brucei TREU927 | hypothetical protein, conserved | genomic | Tb927_02_v5.1 reverse | (geneCodeEnd+0 to geneEnd+0) | length=33

AGTACGTCGAAACAACATCGGCAAAAGGTATTT

>Tb927.8.8310 | Trypanosoma brucei TREU927 | chaperone protein DNAj, putative | genomic | Tb927_08_v5.1 forward | (geneCodeEnd+0 to geneEnd+0) | length=34

ATATTCCTTATGAAATCAAAAACCAAAAAAGAGT

>Tb927.7.6990 | Trypanosoma brucei TREU927 | hypothetical protein, conserved | genomic | Tb927_07_v5.1 reverse | (geneCodeEnd+0 to geneEnd+0) | length=34

AAGTAAAGGATAAAGCAGAAGCGACATCAAAAGC

>Tb927.3.4410 | Trypanosoma brucei TREU927 | pre-RNA processing PIH1/Nop17, putative | genomic | Tb927_03_v5.1 reverse | (geneCodeEnd+0 to geneEnd+0) | length=35

AGTGTACATGATTTCTATCACTGGGAGAGGTGTGG

>Tb927.11.6290 | Trypanosoma brucei TREU927 | HIT zinc finger, putative | genomic | Tb927_11_v5.1 reverse | (geneCodeEnd+0 to geneEnd+0) | length=38

ACGACTCATGGAGGAGCGCATGTGTTTGCTGGCGTTTG

>Tb927.9.4940 | Trypanosoma brucei TREU927 | presenilin-like aspartic peptidase, putative, presenilin-like aspartic peptidase, clan AD, family A22A, putative | genomic | Tb927_09_v5.1 forward | (geneCodeEnd+0 to geneEnd+0) | length=38

GATGAGTAGCATAGAACAGGTGATTCATTTTTTTTTCC

>Tb927.4.4630 | Trypanosoma brucei TREU927 | hypothetical protein, conserved | genomic | Tb927_04_v5.1 reverse | (geneCodeEnd+0 to geneEnd+0) | length=39

AGTACCGTAATGGGCTGTCGAGTGAAATGGAGCGCTAGT

>Tb927.10.7070 | Trypanosoma brucei TREU927 | small nuclear RNA-activating protein (SNAP3) | genomic | Tb927_10_v5.1 forward | (geneCodeEnd+0 to geneEnd+0) | length=39

AGTGTAATGCTTCGGTGCTGTTTGCAGTTGCAGTTGCAG

>Tb927.10.3980 | Trypanosoma brucei TREU927 | unspecified product | genomic | Tb927_10_v5.1 reverse | (geneCodeEnd+0 to geneEnd+0) | length=39

ATGAAGATGAAATGAGGGAGGTTGGTTTACTTAATACTT

>Tb927.4.3660 | Trypanosoma brucei TREU927 | hypothetical protein, conserved | genomic | Tb927_04_v5.1 reverse | (geneCodeEnd+0 to geneEnd+0) | length=40

AGGAAGACACTGCATGGACTACTATTGAGAAGCAACGACT

>Tb927.8.7170 | Trypanosoma brucei TREU927 | inositol polyphosphate 1-phosphatase, putative | genomic | Tb927_08_v5.1 forward | (geneCodeEnd+0 to geneEnd+0) | length=40

GTCGCTACAGGAAATCAATAAATCGTTATTGCCCCTTTTT

>Tb927.11.7190 | Trypanosoma brucei TREU927 | hypothetical protein | genomic | Tb927_11_v5.1 reverse | (geneCodeEnd+0 to geneEnd+0) | length=41

AGAGTTTTGGCGTAGGCTGTACGACGACTCTAAGTATAATT

>Tb927.10.8380 | Trypanosoma brucei TREU927 | hypothetical protein, conserved | genomic | Tb927_10_v5.1 forward | (geneCodeEnd+0 to geneEnd+0) | length=41

GCATGCTTTGTGCTGCTGCTACTGTTGTTGTTGTTTATTAC

>Tb927.4.4650 | Trypanosoma brucei TREU927 | diphthine synthase, putative | genomic | Tb927_04_v5.1 reverse | (geneCodeEnd+0 to geneEnd+0) | length=41

ACAGCAATTACTAATATCGAAAGTGGGTAACATACTAACGG

>Tb927.10.14140 | Trypanosoma brucei TREU927 | pyruvate kinase 1 (PYK1) | genomic | Tb927_10_v5.1 reverse | (geneCodeEnd+0 to geneEnd+0) | length=42

AGCATTGGTGTGTGGGTGAGCAATGTAATGAGGGGTTAATGA

>Tb927.3.2380 | Trypanosoma brucei TREU927 | hypothetical protein, conserved | genomic | Tb927_03_v5.1 forward | (geneCodeEnd+0 to geneEnd+0) | length=43

AGGCAATTAAATCATAACTAATAAGAGACACCGGCTGAATGTC

>Tb927.6.2680 | Trypanosoma brucei TREU927 | transmembrane protein, putative | genomic | Tb927_06_v5.1 forward | (geneCodeEnd+0 to geneEnd+0) | length=44

AAAATTTGGTTTTGTGCTTTATTGCTGCAGTTGTTGTTTTGGTT

>Tb927.5.3520 | Trypanosoma brucei TREU927 | queuine tRNA-ribosyltransferase, putative | genomic | Tb927_05_v5.1 forward | (geneCodeEnd+0 to geneEnd+0) | length=45

AGAACATTCAACTTAATGAACGAATAATGATAATGATGATCGCAT

>Tb927.11.13860 | Trypanosoma brucei TREU927 | hypothetical protein, conserved | genomic | Tb927_11_v5.1 forward | (geneCodeEnd+0 to geneEnd+0) | length=45

ATCTCCCGGAGGGTTAAGTTTAACGGTGAATGGACTGATTGCCTT

>Tb927.9.8720 | Trypanosoma brucei TREU927 | fructose-1,6-bisphosphatase (FBPase) | genomic | Tb927_09_v5.1 reverse | (geneCodeEnd+0 to geneEnd+0) | length=45

AGCCAATGGTTGCTTCGATGCGTGTGGAAAGGGTACAGGTTTTTT

>Tb927.8.1750 | Trypanosoma brucei TREU927 | Cytokine-induced anti-apoptosis inhibitor 1, Fe-S biogenesis, putative | genomic | Tb927_08_v5.1 reverse | (geneCodeEnd+0 to geneEnd+0) | length=46

AATGAGATTTGGAGTATGTGTGGCTGAGGGAGAGGGGAAAGATAAG

>Tb927.11.12630 | Trypanosoma brucei TREU927 | hypothetical protein, conserved | genomic | Tb927_11_v5.1 forward | (geneCodeEnd+0 to geneEnd+0) | length=46

AAGTGATACTGGTGCCTATCGCCGTGTTCTGCGGGTCATTGTTACC

>Tb927.6.750 | Trypanosoma brucei TREU927 | kinetoplastid-specific phospho-protein phosphatase, putative | genomic | Tb927_06_v5.1 reverse | (geneCodeEnd+0 to geneEnd+0) | length=46

AGTGTTTCTCGTGTGTATCGAGCGCTAATTGTGTAGATGTTGTGAT

>Tb927.4.680 | Trypanosoma brucei TREU927 | hypothetical protein, conserved | genomic | Tb927_04_v5.1 reverse | (geneCodeEnd+0 to geneEnd+0) | length=46

GCTGAGAGTTGAAACATCGACTATTGAGTCATTTGCTGTGTTTTCG

>Tb927.3.2960 | Trypanosoma brucei TREU927 | inosine-adenosine-guanosine-nucleosidehydrolase, IAG-nucleoside hydrolase (IAGNH) | genomic | Tb927_03_v5.1 reverse | (geneCodeEnd+0 to geneEnd+0) | length=46

AGACCCCGCGCAATGCGCAAATTCGAAAGTTAAAAGGAGAGAGAGG

>Tb927.11.13710 | Trypanosoma brucei TREU927 | hypothetical protein, conserved | genomic | Tb927_11_v5.1 reverse | (geneCodeEnd+0 to geneEnd+0) | length=47

ACTCTGCTAACGCAAATTTTTTCTCTTGTTTTGTCTCTTTCCCGTTC

>Tb927.11.11210 | Trypanosoma brucei TREU927 | paraflagellar rod component, putative (PFC17) | genomic | Tb927_11_v5.1 forward | (geneCodeEnd+0 to geneEnd+0) | length=47

ACCTGTGAACCCACTAATTTTTCCCCTCCACGAAAGAAAGCTAGAAC

>Tb927.11.6610 | Trypanosoma brucei TREU927 | hypothetical protein, conserved | genomic | Tb927_11_v5.1 reverse | (geneCodeEnd+0 to geneEnd+0) | length=47

ACAAGCTAGGCGTCATCAGCCTTCCATGTTCGTTCGCTCCTCCTCTT

>Tb927.6.4560 | Trypanosoma brucei TREU927 | hypothetical protein, conserved | genomic | Tb927_06_v5.1 reverse | (geneCodeEnd+0 to geneEnd+0) | length=48

ACTTATGGTGAACAAGCGTCAAGAAACCGTCACATATCGCTTTCGGTT

>Tb927.9.3360 | Trypanosoma brucei TREU927 | N-acetyltransferase, putative | genomic | Tb927_09_v5.1 forward | (geneCodeEnd+0 to geneEnd+0) | length=49

AGGTGGACAACAAGGAATTTGTCCACGCGGCGAAACTATCAACTCATAT

>Tb927.2.4870 | Trypanosoma brucei TREU927 | kinetoplastid-specific dual specificity phosphatase, putative | genomic | Tb927_02_v5.1 reverse | (geneCodeEnd+0 to geneEnd+0) | length=49

ACCAATTTGTTGCGCAGATTTTTTTAAGAAAAGATTTAAAATACAACTT

>Tb927.7.4670 | Trypanosoma brucei TREU927 | ribosomal RNA processing protein 4, exosome complex exonuclease (RRP4) | genomic | Tb927_07_v5.1 reverse | (geneCodeEnd+0 to geneEnd+0) | length=50

AATAATACACTTATGCTGCCAGCGAGTTGTGCTACGTAATGATATTTGTG

>Tb927.10.13110 | Trypanosoma brucei TREU927 | Dynein light chain roadblock-type protein, putative | genomic | Tb927_10_v5.1 reverse | (geneCodeEnd+0 to geneEnd+0) | length=51

GAGTAACTGAGTTGCGTTATGAGGTTTTATTAGTTGAGGAATGAGGCGAGG

>Tb927.2.1720 | Trypanosoma brucei TREU927 | hypothetical protein, conserved | genomic | Tb927_02_v5.1 forward | (geneCodeEnd+0 to geneEnd+0) | length=51

GTACCAATACGTTGCCTCCTTTTGAGACTTTTAAATTCCCTTATACACAAC

>Tb927.10.3080 | Trypanosoma brucei TREU927 | methionine biosynthetic protein, putative | genomic | Tb927_10_v5.1 forward | (geneCodeEnd+0 to geneEnd+0) | length=51

AGTCGCGGTCGTTTGGACGGTTGAGGAAATGGTATGTTGTGTGCTAGAGAG

>Tb927.9.3920 | Trypanosoma brucei TREU927 | ribosomal protein S7, putative | genomic | Tb927_09_v5.1 forward | (geneCodeEnd+0 to geneEnd+0) | length=52

GATGAGTTTCGCCCAGCTACTTTGAAGGTGCCATTATCAGTTTTTTGTTTTG

>Tb927.4.3710 | Trypanosoma brucei TREU927 | Predicted membrane protein, putative | genomic | Tb927_04_v5.1 reverse | (geneCodeEnd+0 to geneEnd+0) | length=52

AGTCCAGTTGGTGTCAAGAGCCGCTGTCTGATTTTGCCATCGTCACATACAT

>Tb927.9.10830 | Trypanosoma brucei TREU927 | HD domain containing protein, putative | genomic | Tb927_09_v5.1 reverse | (geneCodeEnd+0 to geneEnd+0) | length=52

AGACACCAACTGACGGGAGGGGAGGTATGATGGTTCTGTCGAGGTATGGGAG

>Tb927.9.7070 | Trypanosoma brucei TREU927 | exosome complex exonuclease RRP40 (RRP40) | genomic | Tb927_09_v5.1 forward | (geneCodeEnd+0 to geneEnd+0) | length=52

AATATAAGTTAAGGATGTTAGCGACGGTGCGCTTGCCGCTGTAAGTTTCTTT

>Tb927.4.2000 | Trypanosoma brucei TREU927 | ruvB-like DNA helicase, putative (RUVBL) | genomic | Tb927_04_v5.1 reverse | (geneCodeEnd+0 to geneEnd+0) | length=53

ATGTTTCCTTTTCTCCAGGGAAAGAGGGTGGCGTGAGTAGAATGGCAGCACAT

>Tb927.10.11260 | Trypanosoma brucei TREU927 | hypothetical protein, conserved | genomic | Tb927_10_v5.1 reverse | (geneCodeEnd+0 to geneEnd+0) | length=54

AGCAATATGGGCTTTGGGGTTCCCCTCTGACCACTTTCCTTCGCTAGGTCGTGC

>Tb927.9.8810 | Trypanosoma brucei TREU927 | hypothetical protein, conserved | genomic | Tb927_09_v5.1 reverse | (geneCodeEnd+0 to geneEnd+0) | length=54

GAGGACACCCATGTATGTGACTTCACTCACCGAATGAATGGTGCTGCGAAATTC

>Tb927.8.680 | Trypanosoma brucei TREU927 | KREPA5 (KREPA5) | genomic | Tb927_08_v5.1 reverse | (geneCodeEnd+0 to geneEnd+0) | length=54

AAGGGTGTAATTCATAAGACGTGTAATGTTTTTAGTCGTGTTGTTGCCATTGTT

>Tb927.9.8070 | Trypanosoma brucei TREU927 | 60S ribosomal protein L10, putative, QM-like protein (QM) | genomic | Tb927_09_v5.1 reverse | (geneCodeEnd+0 to geneEnd+0) | length=55

AACGACCACGGCTCTTTTGCTCGATTGAGCGTTCATTTCTACTTTTCTGCTGCTT

>Tb927.9.7110 | Trypanosoma brucei TREU927 | GRAM domain containing protein, putative | genomic | Tb927_09_v5.1 forward | (geneCodeEnd+0 to geneEnd+0) | length=55

AATATGTGAACTTAGGAAGGACAAGGATGGTCGTTGAATGGGAAGAACAACATGG

>Tb927.5.970 | Trypanosoma brucei TREU927 | class I transcription factor A, subunit 6 (CITFA-6) | genomic | Tb927_05_v5.1 forward | (geneCodeEnd+0 to geneEnd+0) | length=55

GGTGACCTAAAGTGGATCCAGTGCATGGGTGTTGGGCTGCTAACAACGAACACTG

>Tb927.8.4920 | Trypanosoma brucei TREU927 | hypothetical protein, conserved | genomic | Tb927_08_v5.1 forward | (geneCodeEnd+0 to geneEnd+0) | length=55

ATGGAATTCGTGCGTCATGTCGTTGTGCCCTTCGACAGGCGCATACCACTCGTTT

>Tb927.10.7970 | Trypanosoma brucei TREU927 | Organic solute transporter Ostalpha, putative | genomic | Tb927_10_v5.1 reverse | (geneCodeEnd+0 to geneEnd+0) | length=55

GGCTCCCGGCGTGGGGGTGGAGGAGACCTGGTGGGGTATGCAAAAAGAGGGGTAG

>Tb927.10.7750 | Trypanosoma brucei TREU927 | hypothetical protein, conserved | genomic | Tb927_10_v5.1 reverse | (geneCodeEnd+0 to geneEnd+0) | length=56

ACGTGGAAGTGATTTAAAAAAACTCCCGTGTTTGTCGCTGTGGTTCATGGATGGAT

>Tb927.11.11130 | Trypanosoma brucei TREU927 | hypothetical protein, conserved | genomic | Tb927_11_v5.1 forward | (geneCodeEnd+0 to geneEnd+0) | length=56

AGAAAGAAAGGGTGTCGCTGACGCAACCTTCAGTTGCGTGGTACTGATGATGGTTT

>Tb927.11.11100 | Trypanosoma brucei TREU927 | glucosamine 6-phosphate n-acetyltransferase (GNA1) | genomic | Tb927_11_v5.1 forward | (geneCodeEnd+0 to geneEnd+0) | length=56

GTTTTTTTTGTCTGCATTTTCGATTTAACATGAGTATTCACTTATCTACTTTCAGC

>Tb927.9.7820 | Trypanosoma brucei TREU927 | hypothetical protein, conserved | genomic | Tb927_09_v5.1 reverse | (geneCodeEnd+0 to geneEnd+0) | length=56

ATTAAAGAGACGTATGGAAGAGTAAGAGGTGAAGGGGTGCATTTCCGTGTTATTAC

>Tb927.11.12090 | Trypanosoma brucei TREU927 | endonuclease III, putative | genomic | Tb927_11_v5.1 forward | (geneCodeEnd+0 to geneEnd+0) | length=56

GTAATATACATGCATATTTCCCCTCTGTATCTCTTTACCACTTGTACTATTATGTC

>Tb927.8.1430 | Trypanosoma brucei TREU927 | hypothetical protein, conserved | genomic | Tb927_08_v5.1 forward | (geneCodeEnd+0 to geneEnd+0) | length=57

GGTTAAGTATCGATGGTAGCATGCGGCGGCTGTCGATGCTTGTGAAGAGTTAATATT

>Tb927.11.6980 | Trypanosoma brucei TREU927 | hypothetical protein, conserved | genomic | Tb927_11_v5.1 reverse | (geneCodeEnd+0 to geneEnd+0) | length=57

AGTAGTAATAGTGATGATGATATTGGTAGTATTGGTAGAAATGCAGGAAAAGCGATC

>Tb927.11.11350 | Trypanosoma brucei TREU927 | hypothetical protein, conserved | genomic | Tb927_11_v5.1 forward | (geneCodeEnd+0 to geneEnd+0) | length=57

ACGCTATTCGTTGTTATGCAGAACAACTGTGTTTTCGTTTCATTTAAGGTCGATGTT

>Tb927.8.1960 | Trypanosoma brucei TREU927 | hypothetical protein, conserved | genomic | Tb927_08_v5.1 forward | (geneCodeEnd+0 to geneEnd+0) | length=58

ACGAATACTTTCTTTGGTAAACAAACGATGAGTACAGTTCGATAAGGAGGCTGAGATG

>Tb927.7.4350 | Trypanosoma brucei TREU927 | hypothetical protein, conserved | genomic | Tb927_07_v5.1 reverse | (geneCodeEnd+0 to geneEnd+0) | length=58

GGGTACGCGGAATGAAGGTAAGTCGTTTTTGCTAGGTTGAAAAGGGTGTGGGACCACG

>Tb927.11.13780 | Trypanosoma brucei TREU927 | profilin | genomic | Tb927_11_v5.1 forward | (geneCodeEnd+0 to geneEnd+0) | length=58

AAGTCTGGTTCCATAATAGTGTTGCTTCGCTTTCTTTTTATTCGAACCTATCAATTAG

>Tb927.10.14030 | Trypanosoma brucei TREU927 | hypothetical protein, conserved | genomic | Tb927_10_v5.1 reverse | (geneCodeEnd+0 to geneEnd+0) | length=58

GTTTTATTATTATTCCTTTTTTTTTAAAAAAAATATCAGGGTTCGGTTCCGTATTTTC

>Tb927.10.1310 | Trypanosoma brucei TREU927 | Domain of unknown function (DUF4042), putative | genomic | Tb927_10_v5.1 reverse | (geneCodeEnd+0 to geneEnd+0) | length=58

AGGTGAAAGATCGGGGTTGGGAAGCGCACGGCGTACACTGGGGACAAGTTATCGAGAG

>Tb927.7.2800 | Trypanosoma brucei TREU927 | Prp18 domain containing protein, putative | genomic | Tb927_07_v5.1 forward | (geneCodeEnd+0 to geneEnd+0) | length=58

ACTACTAGCAGTCAGTTGCCACCTTCCTGCTGGTACTGCCAAATAAACATTTCTTTTT

>Tb927.11.7960 | Trypanosoma brucei TREU927 | kinetoplast poly(A) polymerase 1 | genomic | Tb927_11_v5.1 reverse | (geneCodeEnd+0 to geneEnd+0) | length=59

GTACATATGGACGTGGCATTGGGTGACAAAGTATCTAGTGCCGCCTTAAAGCTGTTTTT

>Tb927.9.13460 | Trypanosoma brucei TREU927 | Spt4/RpoE2 zinc finger, putative | genomic | Tb927_09_v5.1 reverse | (geneCodeEnd+0 to geneEnd+0) | length=59

ATCGAGTACACAGGGGTTAGGGCAGGAAAGATGCCTTAGAGTGGGAAGATGGTAAAAAC

>Tb927.9.3770 | Trypanosoma brucei TREU927 | hypothetical protein, conserved | genomic | Tb927_09_v5.1 forward | (geneCodeEnd+0 to geneEnd+0) | length=60

ACACCGCAGATGCCTTCTAAACAATACAAAAACCGACTGCATCATATCTACGTTCCTTCT

>Tb927.11.16410 | Trypanosoma brucei TREU927 | enolase, putative | genomic | Tb927_11_v5.1 reverse | (geneCodeEnd+0 to geneEnd+0) | length=60

AGGGATGACACGACCTGGCTGTGTAACGCGGAGTATGTTAGCATTTCCCTACGAGCATGT

>Tb927.4.2120 | Trypanosoma brucei TREU927 | hypothetical protein, conserved | genomic | Tb927_04_v5.1 forward | (geneCodeEnd+0 to geneEnd+0) | length=60

GTTGCGTTTACGTGATACGCAGGAGACAATGCAAGTCCATGAGGTGCCGGCGAAGTTCCC

>Tb927.7.5000 | Trypanosoma brucei TREU927 | 60S ribosomal protein L19, putative | genomic | Tb927_07_v5.1 forward | (geneCodeEnd+0 to geneEnd+0) | length=61

AAGGTTGCACCACTGCTTCTAACTGATTTTGGTACTTTTCTTTTTATTGTGTGTATCTGC

T

>Tb927.11.6320 | Trypanosoma brucei TREU927 | MRB1-associated protein | genomic | Tb927_11_v5.1 reverse | (geneCodeEnd+0 to geneEnd+0) | length=61

ATCTACTTTTCTACTAATCACAACAACACTTCTCTGTTCTTACTTCAATATTTTTTGTCA

C

>Tb927.9.1880 | Trypanosoma brucei TREU927 | WD domain, G-beta repeat, putative | genomic | Tb927_09_v5.1 reverse | (geneCodeEnd+0 to geneEnd+0) | length=62

GGTTCATGTGTTTTTCTGTATATTTGCCCGCTCATCTACCACTTACCACTTCTATGTAAA

TG

>Tb927.8.5880 | Trypanosoma brucei TREU927 | eukaryotic translation initiation factor 1A, putative | genomic | Tb927_08_v5.1 forward | (geneCodeEnd+0 to geneEnd+0) | length=63

ACAACCGTGGGGAAGGAGGGGAAGAAAAGAAAAAGAAAAAAAAGAAGTAATAAAAACAAC

AAC

>Tb927.11.8020 | Trypanosoma brucei TREU927 | hypothetical protein, conserved | genomic | Tb927_11_v5.1 forward | (geneCodeEnd+0 to geneEnd+0) | length=63

GGCGAACAATTAAGTTTGGGGACGCGTAGTTGGTATTGTTTTCCGAGTATTTTCTTCGTC

CTG

>Tb927.10.210 | Trypanosoma brucei TREU927 | GPI transamidase component GAA1 (TbGAA1) | genomic | Tb927_10_v5.1 forward | (geneCodeEnd+0 to geneEnd+0) | length=63

GATGGGTTTAACTGTAACAAAAAGTCAGGGCGCATTCTGGTGGTATGTTGTCTTCGTTTT

CCC

>Tb927.11.15850 | Trypanosoma brucei TREU927 | kinteoplast poly(A) polymerase complex 1 subunit | genomic | Tb927_11_v5.1 reverse | (geneCodeEnd+0 to geneEnd+0) | length=63

AGGTTGTGGGAAGCGTTGCGTGAAGATGCGTGGATAATGCTCCTGTTTGAGTTCTATGTC

CTT

>Tb927.11.2420 | Trypanosoma brucei TREU927 | Tubulin-tyrosine ligase family, putative | genomic | Tb927_11_v5.1 reverse | (geneCodeEnd+0 to geneEnd+0) | length=63

AGCTATTTCATTGGAAACATTTTTCTTCCATCATCATTTATGTTGCATGTGGATTTCCAT

CAG

>Tb927.9.11490 | Trypanosoma brucei TREU927 | 60S ribosomal protein L27a, 60S ribosomal protein L28, 60S ribosomal protein L29 (RPL27A) | genomic | Tb927_09_v5.1 forward | (geneCodeEnd+0 to geneEnd+0) | length=64

ATGTGGACAGACGGTCCACTGAGGCAATCTTTTTTTCATTTCTGTGTCTGTGTTGTACTA

ACTG

>Tb927.6.1990 | Trypanosoma brucei TREU927 | hypothetical protein, conserved | genomic | Tb927_06_v5.1 reverse | (geneCodeEnd+0 to geneEnd+0) | length=64

AAGTAATGATAAACCATACCTTTATTAATTATCTTTATCAGTTTTTACCCATCCCACCTT

AAAA

>Tb927.8.1930 | Trypanosoma brucei TREU927 | Isy1-like splicing family, putative | genomic | Tb927_08_v5.1 reverse | (geneCodeEnd+0 to geneEnd+0) | length=64

GTGCGTGTGTCCACTAAGATTCTTTTGTTTGGGCGATTACCGGATTTTCCTGTATAATTG

TTGC

>Tb927.11.4860 | Trypanosoma brucei TREU927 | Ring finger domain containing protein, putative | genomic | Tb927_11_v5.1 forward | (geneCodeEnd+0 to geneEnd+0) | length=65

AACAGGGTTCTTCCCATGTTACTTTTGATCTATTTATCATTTGCTTGTGTTTAAGGTTGG

TTATG

>Tb927.9.11560 | Trypanosoma brucei TREU927 | hypothetical protein, conserved | genomic | Tb927_09_v5.1 forward | (geneCodeEnd+0 to geneEnd+0) | length=65

AGGAAAGTTTATTGGTATTGCGTGTACGATACTAACGAACAAGAAACTTTTCTCTTTTGC

TCTGC

>Tb927.2.5070 | Trypanosoma brucei TREU927 | hypothetical protein, conserved | genomic | Tb927_02_v5.1 reverse | (geneCodeEnd+0 to geneEnd+0) | length=65

ACATAAATGGCGGCATAACGTTCGTTTACCAATGTTAGCCATCCATATAACCCAAAGGCT

TATCG

>Tb927.9.2110 | Trypanosoma brucei TREU927 | hypothetical protein, conserved | genomic | Tb927_09_v5.1 reverse | (geneCodeEnd+0 to geneEnd+0) | length=65

AAATAATGAGTCAGTGGAGATGTTTTTCTCAGCGAACGAACTGTAACGTTAACTCACAAC

ATCCG

>Tb927.3.2300 | Trypanosoma brucei TREU927 | DNL zinc finger, putative | genomic | Tb927_03_v5.1 forward | (geneCodeEnd+0 to geneEnd+0) | length=66

AGTTTTCCTCCGTTTTCCCTTCCCACTCCCCTCTTTGGTTGTCAAACTACCCGGTGAGGA

TACCAG

>Tb927.4.1470 | Trypanosoma brucei TREU927 | Domain of unknown function (DUF4379), putative | genomic | Tb927_04_v5.1 reverse | (geneCodeEnd+0 to geneEnd+0) | length=66

AGTCTAATAGGTGAGGTCACAATATTCGCAAAGTTTTCACCGACGCATCGGTCTGTCATA

CACAAG

>Tb927.6.2500 | Trypanosoma brucei TREU927 | RF-1 domain containing protein, putative | genomic | Tb927_06_v5.1 forward | (geneCodeEnd+0 to geneEnd+0) | length=66

ACGTTTCTGCAGAGGAATTGTGTACACTCAAATGGTTATGAGTTCAACAGTTTTTTAGTT

GTGTTT

>Tb927.10.1660 | Trypanosoma brucei TREU927 | hypothetical protein, conserved | genomic | Tb927_10_v5.1 reverse | (geneCodeEnd+0 to geneEnd+0) | length=66

GTTTATATTCATGTGTTAGCAATGCAGTACCGTGTTGCTTTGTAGAAGAGACATATCGTT

TTCACT

>Tb927.10.11900 | Trypanosoma brucei TREU927 | thioredoxin, putative | genomic | Tb927_10_v5.1 forward | (geneCodeEnd+0 to geneEnd+0) | length=66

GGTTGAATGGAGGAGACCACTCTTCCCCATGTCTTTTCCTTACGGAAAGGCTCTGATCTT

CACACG

>Tb927.2.5120 | Trypanosoma brucei TREU927 | vesicle-associated membrane protein | genomic | Tb927_02_v5.1 forward | (geneCodeEnd+0 to geneEnd+0) | length=67

GGAAACACGCTATCACGTAGTCGGACATTTTTTCATAGTGTAGTTTTTTATAGTTATTTC

CACTTTG

>Tb927.10.7340 | Trypanosoma brucei TREU927 | 40S ribosomal protein S24E, putative | genomic | Tb927_10_v5.1 reverse | (geneCodeEnd+0 to geneEnd+0) | length=67

AGTGACGATGTGGCGGACAGACCATACACTCCTTGGATGCAAGGCTTTTTTCGTATATTT

TTCTTTT

>Tb927.5.2910 | Trypanosoma brucei TREU927 | hypothetical protein, conserved | genomic | Tb927_05_v5.1 forward | (geneCodeEnd+0 to geneEnd+0) | length=67

AAGTCAGTGAAGTATTTATCTTGCGCTCCAGTAATATTGTATTACCTTACGTTACGGTTC

TTCTGTG

>Tb927.11.12080 | Trypanosoma brucei TREU927 | N-acetyl-D-glucosaminylphosphatidylinositol de-N-acetylase (GPI12) | genomic | Tb927_11_v5.1 forward | (geneCodeEnd+0 to geneEnd+0) | length=67

AATCGAGGAAATTTAGCCCCCGCGCACTTTTTCTACTGCTTACTGGTGTACATTAAGATG

ATGAATG

>Tb927.11.8040 | Trypanosoma brucei TREU927 | hypothetical protein, conserved | genomic | Tb927_11_v5.1 forward | (geneCodeEnd+0 to geneEnd+0) | length=68

GTGTTTCAACGTGGAATGTGTCTGTGTTAGGAAGGAGGAGGTCCGTTTGTTGAATCCCCT

ATATCTTT

>Tb927.11.7020 | Trypanosoma brucei TREU927 | proteasome alpha 7 subunit, putative (PSA4) | genomic | Tb927_11_v5.1 reverse | (geneCodeEnd+0 to geneEnd+0) | length=68

AGGCGCGTGCACCTGTGGGAAAAAGAATGTGGACAGAATTTGGTGAGGAGGAAAACAAAC

AAAGGAAG

>Tb927.3.4140 | Trypanosoma brucei TREU927 | hypothetical protein, conserved | genomic | Tb927_03_v5.1 reverse | (geneCodeEnd+0 to geneEnd+0) | length=68

ACACAAGGTTTCATGGAGTGGCCGTTACAATGGAATTGGTGTGGGCCGCGTAAGGATAAC

AATTTGCG

>Tb927.9.13970 | Trypanosoma brucei TREU927 | hypothetical protein, conserved | genomic | Tb927_09_v5.1 reverse | (geneCodeEnd+0 to geneEnd+0) | length=69

ACCAGGACGGGGAGCGCGACAAGGTTAAGTGATGGTTTCTTCCACCATGTCACCACTGGT

GCGGGAGCG

>Tb927.9.15020 | Trypanosoma brucei TREU927 | hypothetical protein, conserved | genomic | Tb927_09_v5.1 forward | (geneCodeEnd+0 to geneEnd+0) | length=70

AGGTGGACGATATGCTCCCCCCCTGTGGCAAGCACCTGGTTAACTCCCTGTGGACTTATG

TACACCAAAT

>Tb927.7.3390 | Trypanosoma brucei TREU927 | hypothetical protein, conserved | genomic | Tb927_07_v5.1 reverse | (geneCodeEnd+0 to geneEnd+0) | length=70

ATAAGGGAATCATTCAAATGACTTATCCCCATCTAGAGGCTAGGAAAGTGCCTTGACAGG

TTTTACTATC

>Tb927.7.6380 | Trypanosoma brucei TREU927 | ssm4 protein, putative (TbSsm4) | genomic | Tb927_07_v5.1 reverse | (geneCodeEnd+0 to geneEnd+0) | length=71

ACGCAGCTCATAACTTCAGTTGAATTCTCTCACAACTGTTCTGCGGGGTTTGTCGTGATG

TTCACGTTTAC

>Tb927.8.2720 | Trypanosoma brucei TREU927 | Putative serine esterase (DUF676), putative | genomic | Tb927_08_v5.1 forward | (geneCodeEnd+0 to geneEnd+0) | length=71

ACGTATACGTTAGGGCATTGGGTACTACACTATGAACTATTTTGCATCGTATTTCTTGCA

CTCTCGTTTGG

>Tb927.8.6920 | Trypanosoma brucei TREU927 | hypothetical protein, conserved | genomic | Tb927_08_v5.1 reverse | (geneCodeEnd+0 to geneEnd+0) | length=72

ACTGCATACGTGTAGCAGGCGTGGCATACTCATTTATACTTACTTCCTGGTCCTTCGCTT

TCTTTTTTCTGG

>Tb927.8.3860 | Trypanosoma brucei TREU927 | WD-repeat containing protein | genomic | Tb927_08_v5.1 reverse | (geneCodeEnd+0 to geneEnd+0) | length=72

AAGAGAGAGGGTGTGTGTCTGGGATCGGCAGTGCGCAGGTGTTAAATCGGCGCCCAATTT

TCTTTAGCTTTT

>Tb927.9.9020 | Trypanosoma brucei TREU927 | ribosome-interacting GTPase 2, putative (RBG2) | genomic | Tb927_09_v5.1 reverse | (geneCodeEnd+0 to geneEnd+0) | length=72

AACTCGAGCTCATTTGCCAGGTCACGATTTAACTTGCATCCCGCAGGAGGCGGTGCTACA

ATTGTCAAGCGC

>Tb927.7.2720 | Trypanosoma brucei TREU927 | translation initiation factor SUI1, putative | genomic | Tb927_07_v5.1 reverse | (geneCodeEnd+0 to geneEnd+0) | length=72

AGGTAACCGATTGAGCCAGCTTTTTTGTTTTAGCATACAAGGACGACGTAACTCGAAGAA

AAATTGGTGGTG

>Tb927.10.11400 | Trypanosoma brucei TREU927 | WD domain, G-beta repeat, putative | genomic | Tb927_10_v5.1 forward | (geneCodeEnd+0 to geneEnd+0) | length=72

GAGTTTTGGATTGTTGGTGAAAGTGTCACTCCAGTTGGGATGTCTGTTGGTTTGCTGTCA

CTTCGCCTCTTT

>Tb927.7.3240 | Trypanosoma brucei TREU927 | hypothetical protein, conserved | genomic | Tb927_07_v5.1 forward | (geneCodeEnd+0 to geneEnd+0) | length=73

GTCGAAAGGCATGTTGTATACGGAAGGACGGAGGGAGGGAGTTACAGTGGAAGAACGTCT

TTTAAGAAGTGTG

>Tb927.8.6280 | Trypanosoma brucei TREU927 | hypothetical protein, conserved | genomic | Tb927_08_v5.1 forward | (geneCodeEnd+0 to geneEnd+0) | length=73

AGGTTGCGTATGGACATGCCACATTACACTTTGTGCATGTGTACAGTTTGTGCATATTTG

TTGTGATTCTCTT

>Tb927.9.3280 | Trypanosoma brucei TREU927 | acidocalcisomal exopolyphosphatase | genomic | Tb927_09_v5.1 forward | (geneCodeEnd+0 to geneEnd+0) | length=73

AAGATAAAAGAAAAAGAAAAATGGACCATTTTGTTATGTTGATCTGTCTTGTTGTGACCG

GCACTATTACTTT

>Tb927.5.580 | Trypanosoma brucei TREU927 | prefoldin subunit, putative | genomic | Tb927_05_v5.1 reverse | (geneCodeEnd+0 to geneEnd+0) | length=74

ATGGATGGCAAAGGTTAAGAAAATTTAACGTCCGCGTTTATGTCTTTTTAACTCAGTTAT

TATGCACTATTTTC

>Tb927.5.600 | Trypanosoma brucei TREU927 | hypothetical protein, conserved | genomic | Tb927_05_v5.1 reverse | (geneCodeEnd+0 to geneEnd+0) | length=75

GTGCGCTCGAGGACGTTCGAGACGCGCTATTGGTTGTATGAACGGGAGGTCAAGAGGCTT

GTGATCTACAATAAT

>Tb927.11.1970 | Trypanosoma brucei TREU927 | hypothetical protein, conserved | genomic | Tb927_11_v5.1 forward | (geneCodeEnd+0 to geneEnd+0) | length=75

AAGTTTCAGCGATGCTTCAACTGTTCGTGTGTTGTGAACATGGTCACTGCTATCATTCGT

GCGCATTCGCATTGT

>Tb927.10.10600 | Trypanosoma brucei TREU927 | ARP2/3 complex 16kDa subunit, putative | genomic | Tb927_10_v5.1 reverse | (geneCodeEnd+0 to geneEnd+0) | length=75

ATCAAACAGTGTGTATGTATCGGTGTGGCCGCAGGCGCCATGTACTGCTATGCAAGGCAG

CGTGTCATGGCAAAG

>Tb927.6.1410 | Trypanosoma brucei TREU927 | hypothetical protein, conserved | genomic | Tb927_06_v5.1 reverse | (geneCodeEnd+0 to geneEnd+0) | length=75

ACTGATCTTCTTTCTCCTCCTTTATCTTCCCTTACCCCCACCCAAGAGGCTTCAGGGAAT

CATTGTTTTCACATC

>Tb927.8.4910 | Trypanosoma brucei TREU927 | hypothetical protein, conserved | genomic | Tb927_08_v5.1 forward | (geneCodeEnd+0 to geneEnd+0) | length=76

GAGATGAAAGGGATACCCAATCTTTTTGCGTTTCTTCCTTTTTTGTAGCTCAACCAACAG

CATATATAGTATGTAC

>Tb927.9.8100 | Trypanosoma brucei TREU927 | nascent polypeptide associated complex subunit, putative | genomic | Tb927_09_v5.1 reverse | (geneCodeEnd+0 to geneEnd+0) | length=76

ATCGAGAGTGCTTTTCGTGAGCGATTACCGAGGTGAAAAGTTTTTTCTTAATTTCCTTTT

TAAAAATAATGGCCTG

>Tb927.4.4610 | Trypanosoma brucei TREU927 | hypothetical protein, conserved | genomic | Tb927_04_v5.1 reverse | (geneCodeEnd+0 to geneEnd+0) | length=76

AATGATTGTCACTTTGTACGTCGTGCTATATAATTCACACAAGTACTGTCTTGTATCTCC

TTCGCCCTCTTTGCTT

>Tb927.10.10640 | Trypanosoma brucei TREU927 | hypothetical protein, conserved | genomic | Tb927_10_v5.1 reverse | (geneCodeEnd+0 to geneEnd+0) | length=77

AGGTGAAGTTAGTGTCGTGGTGCCGTATACATGGAGATAACTCTTTATTTGGAGTAGTGC

GGTCTGTGTTGGTTTCT

>Tb927.9.5150 | Trypanosoma brucei TREU927 | ribosomal protein S6, putative, NHP2/RS6-like protein (NHP2) | genomic | Tb927_09_v5.1 forward | (geneCodeEnd+0 to geneEnd+0) | length=77

GTGATGCGTCTTCACTGTAAAGGGGATGCAGACTGAAAAAAGGAAAGCAAAGTACAACGG

AGGCTGAAACTTAAACC

>Tb927.11.15920 | Trypanosoma brucei TREU927 | glutathione peroxidase, putative | genomic | Tb927_11_v5.1 reverse | (geneCodeEnd+0 to geneEnd+0) | length=77

ACGGGTGTACATTCAGCGTATAATTTGTAGCGTACGGGCGTATGTAGGGGCATGGTATGT

TTTATACCCTCATGTTT

>Tb927.11.12370 | Trypanosoma brucei TREU927 | hypothetical protein, conserved | genomic | Tb927_11_v5.1 forward | (geneCodeEnd+0 to geneEnd+0) | length=77

GGAAAGTTGGGAGCACGAGAACTAGCCGAAGCTTATAATTTGTAGCATGATACAACAATT

GAGGTTATGTTTTTCTT

>Tb927.6.3020 | Trypanosoma brucei TREU927 | hypothetical protein, conserved | genomic | Tb927_06_v5.1 forward | (geneCodeEnd+0 to geneEnd+0) | length=77

AACACTCATTCTTCTCTATATGTTTCGTACTGTTACGCTTTTGCATATGTTTTTACCTTT

TCACAACGATAATAAAG

>Tb927.3.3450 | Trypanosoma brucei TREU927 | ADP-ribosylation factor-like protein 3, putative (arl3) | genomic | Tb927_03_v5.1 forward | (geneCodeEnd+0 to geneEnd+0) | length=78

GCTTCGTCGTAGTGAACTAGAGACACTGGATCGTAAGGGCTAAGAACAAAAAGAATCAAG

CTGAGGTATGTGTTCTTG

>Tb927.8.6900 | Trypanosoma brucei TREU927 | transport protein particle (TRAPP) component, putative | genomic | Tb927_08_v5.1 reverse | (geneCodeEnd+0 to geneEnd+0) | length=79

ATTTTTTGACTTGTACGTGCTATACTAATTTCTCCTTCTTGTGAATCATATATCGAACAT

TTTTGACCCACTGTTTGGT

>Tb927.7.3910 | Trypanosoma brucei TREU927 | hypothetical protein, conserved | genomic | Tb927_07_v5.1 forward | (geneCodeEnd+0 to geneEnd+0) | length=79

ACTAATGGTGCTTTGGAGGTTATGTTATCGGCTCGTAACGTTTTGGCTACTTGACACGTA

ATCGTTTATTGTCATCACC

>Tb927.10.10880 | Trypanosoma brucei TREU927 | ATP-binding cassette sub-family F member 1, putative (ABCF1) | genomic | Tb927_10_v5.1 reverse | (geneCodeEnd+0 to geneEnd+0) | length=80

AAAGGCGAAGGAAGGTCCGTGTGGTTCTGGTAAAGAAAGGAAAAGGGAAATCAAACTGAG

CCAACGTAACTAACGAAATG

>Tb927.3.5050 | Trypanosoma brucei TREU927 | 60S ribosomal protein L4 | genomic | Tb927_03_v5.1 forward | (geneCodeEnd+0 to geneEnd+0) | length=80

AGCGTTAAGAGAGGAGAAAACCAATAATGCCCATTATGTGGTGAAGGAATAATTTTATTT

TACTTTATTTTTCTTTTCAT

>Tb927.8.1370 | Trypanosoma brucei TREU927 | hypothetical protein, conserved | genomic | Tb927_08_v5.1 reverse | (geneCodeEnd+0 to geneEnd+0) | length=81

ATGTTTCTTTTACTCTCTTTTGTTATACGACAAATCTCAACTGCGTTCTGACCGTTGGTT

TAGACTTGTATGTTCGTTCAT

>Tb927.10.1730 | Trypanosoma brucei TREU927 | hypothetical protein, conserved | genomic | Tb927_10_v5.1 forward | (geneCodeEnd+0 to geneEnd+0) | length=81

ACCTTGGGGAGCGAGCCGTCGTGCTTCAACTTGCCGCACTGGCTTGTCTGTTTACTGTGG

CGGTACGTTGCAGAGCTTGTG

>Tb927.7.2740 | Trypanosoma brucei TREU927 | hypothetical protein, conserved | genomic | Tb927_07_v5.1 forward | (geneCodeEnd+0 to geneEnd+0) | length=81

GTGATTAGGTAAGGGAAGATGTAGCGTTTGTCTTCTAGATACTCCTGTGCTTGCGTAGGT

GTTTTAAACGAAAACACACAG

>Tb927.6.1240 | Trypanosoma brucei TREU927 | hypothetical protein, conserved | genomic | Tb927_06_v5.1 reverse | (geneCodeEnd+0 to geneEnd+0) | length=81

ATGACAGCTAGGAAGCCGGCTGAGGGAGTACACCAACCACCTCCTCCGTGTACGTGCATA

CTCGGATATGCAACAAATTTT

>Tb927.8.5520 | Trypanosoma brucei TREU927 | hypothetical protein, conserved | genomic | Tb927_08_v5.1 forward | (geneCodeEnd+0 to geneEnd+0) | length=82

ATGAACGACAACGAACTAAAGAAAAAAAACTTCTACTGCGTGTAACTAGCAATATCTATT

GCGCATATTGCTGGCAAGGGAG

>Tb927.4.3490 | Trypanosoma brucei TREU927 | DNA-directed RNA polymerases II and III subunit RPB6, putative (RPB6) | genomic | Tb927_04_v5.1 reverse | (geneCodeEnd+0 to geneEnd+0) | length=82

ACTGGCCACTTTTGGTATTGCCTCGTTGGCTTTCATGCATGTTTCTCTACATGTTTCACT

TCTTCTGTTGTTGCCGTTACCC

>Tb927.8.2010 | Trypanosoma brucei TREU927 | hypothetical protein, conserved | genomic | Tb927_08_v5.1 forward | (geneCodeEnd+0 to geneEnd+0) | length=82

AGGTTGCATTTGTCAAAGGGGAGAGAAACCACGGTCTGCCCCACGCTACTTGTAGCACTT

TTTTCTTCTTCCACACTTTGTT

>Tb927.11.16150 | Trypanosoma brucei TREU927 | tubulin binding cofactor A, putative | genomic | Tb927_11_v5.1 reverse | (geneCodeEnd+0 to geneEnd+0) | length=82

ATACAATCGGTAGTACGAAACCTTAGCGGTGGTGTTGCCTTTTTAATTGAAGTGAGGCAT

GTGTTCTCAGATTAATTTACTT

>Tb927.8.6910 | Trypanosoma brucei TREU927 | cyclophilin, putative | genomic | Tb927_08_v5.1 reverse | (geneCodeEnd+0 to geneEnd+0) | length=83

ATTTCCCCCCCTTTTCAGGTAAAGACTACTCCAAACAAAACGGTGCTCTCAGTGTGTTTA

TACAACGATGTTTGTGATACCGC

>Tb927.9.7710 | Trypanosoma brucei TREU927 | hypothetical protein, conserved | genomic | Tb927_09_v5.1 reverse | (geneCodeEnd+0 to geneEnd+0) | length=83

GTAAAGTTGTTTACTGGGGGCTAGGCGTAATGTGGGATGGGAAGGCGGGGCATCAAGTTT

TGTTGTGATATTTGTTTACCTCT

>Tb927.8.6040 | Trypanosoma brucei TREU927 | hypothetical protein, conserved | genomic | Tb927_08_v5.1 forward | (geneCodeEnd+0 to geneEnd+0) | length=83

ACATTTCGATGGGCAGCGAGGGATGGAGGAGGGGGGAACAGCAGCGAAATATGCTCTCAG

CGCTGTTGTTCGTGTTCGAATGG

>Tb927.9.4910 | Trypanosoma brucei TREU927 | protein kinase, putative | genomic | Tb927_09_v5.1 forward | (geneCodeEnd+0 to geneEnd+0) | length=83

AGCGGAAGCAGGAGGTCGCGTTTAACGTTTCGGAAGTTTCTTTATTAACTGTGTCGTTAG

CAATAGTATTCACGGAACGCAGC

>Tb927.3.4800 | Trypanosoma brucei TREU927 | hypothetical protein, conserved | genomic | Tb927_03_v5.1 reverse | (geneCodeEnd+0 to geneEnd+0) | length=83

GGCCCACATTTTGTTAACAACAGCTACAAATCTTCAACAACACCACTCCAAAACGAGTTC

AACTCTATTGCTCTACACCGTGT

>Tb927.11.3230 | Trypanosoma brucei TREU927 | 60S ribosomal protein L44 | genomic | Tb927_11_v5.1 forward | (geneCodeEnd+0 to geneEnd+0) | length=84

GGTTGTGCGGCGAGACTGATGACATCCGGTTTGAGCAGTATGAACTTTCTTTTTTTCTTT

TCATCTTCTGTGTTCTAACTGTGT

>Tb927.10.230 | Trypanosoma brucei TREU927 | proteasome subunit alpha type-5, putative | genomic | Tb927_10_v5.1 forward | (geneCodeEnd+0 to geneEnd+0) | length=85

ACATCGCGCGTCGTCCAAACTCCCTGCGACGTGCCCTTTTCTTTTGTTTTTGATATGTTT

CTGTAGGACTCAGTGCTCGCGTGTT

>Tb927.11.8850 | Trypanosoma brucei TREU927 | Uncharacterised protein family (UPF0220), putative | genomic | Tb927_11_v5.1 forward | (geneCodeEnd+0 to geneEnd+0) | length=85

AGTGAATTTCGAAGCAATACTTGTCGTCATATTTGTTTCAATTTCCCCCATCCACCTCAT

TTTCCTTGTGTTTTGTGTCAGCGCG

>Tb927.4.1220 | Trypanosoma brucei TREU927 | leucine-rich repeat protein (LRRP), putative | genomic | Tb927_04_v5.1 reverse | (geneCodeEnd+0 to geneEnd+0) | length=85

AATGCTAATTGGAATATTGTAATTTTTTAACACCAGGAGGGGACGGGAGAAGAGGGAGGG

AAAAGGTAAGGCTTAACTTTGAAAA

>Tb927.10.2580 | Trypanosoma brucei TREU927 | Nucleolar protein 12 (25kDa), putative | genomic | Tb927_10_v5.1 forward | (geneCodeEnd+0 to geneEnd+0) | length=85

ATTCGGGGTCCATTTTTCTCTTCCTTGTTTTTTTAGCATATGTCTCTACTCTTCTTCTTT

TTTCTTGGTTTTCCTGCGGTTAATG

>Tb927.4.1330 | Trypanosoma brucei TREU927 | DNA topoisomerase IB, large subunit | genomic | Tb927_04_v5.1 reverse | (geneCodeEnd+0 to geneEnd+0) | length=85

ATAACACGCCGTTGCAGTGACGTATGTTCGTTGGAGAGTGGGGATAACTGAATAAAAGAA

AAGAGCAAGTCGAAACAAACAAACG

>Tb927.9.7590 | Trypanosoma brucei TREU927 | 60S ribosomal protein L11, putative | genomic | Tb927_09_v5.1 reverse | (geneCodeEnd+0 to geneEnd+0) | length=85

AGACCTTTAAGGGCTGCCTCCACCTATTGGAGGGGCCTTTTCCCTGAGTCATACTGTGCC

CTTTATCATTTCTATTTTCTTTTGT

>Tb927.8.2310 | Trypanosoma brucei TREU927 | (H+)-ATPase G subunit, putative | genomic | Tb927_08_v5.1 forward | (geneCodeEnd+0 to geneEnd+0) | length=85

ATGGGTGTAGGAGCAAGATAAAAGGGAAAGGCAGTTGAATGAACGGATTTGTGGTGTCGG

TTAGGCTCAGGGAGGGGGAAGAAGT

>Tb927.6.4980 | Trypanosoma brucei TREU927 | 40S ribosomal protein S14 (RPS14) | genomic | Tb927_06_v5.1 forward | (geneCodeEnd+0 to geneEnd+0) | length=86

GACATACGGAACACAAAACCACACCAGTGGCTATTTTTGTTTTGTTTTAGTGGTAATTTG

CCGCGAACTGATAATTTTATAGTGAG

>Tb927.10.4790 | Trypanosoma brucei TREU927 | hypothetical protein, conserved | genomic | Tb927_10_v5.1 reverse | (geneCodeEnd+0 to geneEnd+0) | length=86

ATACATGATGTGGTGTACCTCTTTTTGGTTTGTGCACATGGATTGCAGGCGGTTTTAAGG

ATGGATGGTTGTGGTTGTTTTGCGAT

>Tb927.8.3040 | Trypanosoma brucei TREU927 | hypothetical protein, conserved | genomic | Tb927_08_v5.1 forward | (geneCodeEnd+0 to geneEnd+0) | length=86

AGAGACGCTTTGGGAGGTAACTGCAAGAGCGGCACCCGTCTAATTTTGGAGTGGAAAGGC

TTGCAAGAGCTGTGTAGTTGTCTTAC

>Tb927.3.3710 | Trypanosoma brucei TREU927 | Scavenger mRNA decapping enzyme C-term binding, putative | genomic | Tb927_03_v5.1 forward | (geneCodeEnd+0 to geneEnd+0) | length=86

GGCAATGTTGTAAACACAGGGGTATGCGTGTATGTATGTACGTGCTTGTGTGTGCGCGTT

TGGCGCCGTTTTACAATTTTCATTGG

>Tb927.8.1870 | Trypanosoma brucei TREU927 | Golgi/lysosome glycoprotein 1 (GLP1) | genomic | Tb927_08_v5.1 reverse | (geneCodeEnd+0 to geneEnd+0) | length=87

GTTGAACGGTTGACGAGAGTACCAGGTAGCAGTGGGTGTTTGAAGAACATGGACCCTTCC

GGAACGAGGAAAAAATGTTTAAGGGAG

>Tb927.9.5210 | Trypanosoma brucei TREU927 | glutaminyl-tRNA synthetase, putative | genomic | Tb927_09_v5.1 forward | (geneCodeEnd+0 to geneEnd+0) | length=87

AGGAAGGGTAATCTGCGCAGAAGTGTCTTGTGTTCCATGCGCTTATTTATTACGAAAAAC

GCGAAGGGAGAAGCTTTAGCACGTCTC

>Tb927.11.14020 | Trypanosoma brucei TREU927 | RNA-binding protein (NRBD2) | genomic | Tb927_11_v5.1 forward | (geneCodeEnd+0 to geneEnd+0) | length=87

AAAAAGATATGCTTGCTTTTTCCTTTTTTCTTTTCTGTTCTTAAATATAAAAAAATATTT

GTTAAGTTAATACTTTTTTACGTATTG

>Tb927.4.1850 | Trypanosoma brucei TREU927 | hypothetical protein, conserved | genomic | Tb927_04_v5.1 reverse | (geneCodeEnd+0 to geneEnd+0) | length=87

AGTATGTCTATTGGTGTTTGTTTCCATTCGCTGTCCTTTCTTCCCAATCCTCATGCTTCC

CTTCTCTGCACGACGCTGGGTTAATTT

>Tb927.11.4270 | Trypanosoma brucei TREU927 | hypothetical protein, conserved | genomic | Tb927_11_v5.1 reverse | (geneCodeEnd+0 to geneEnd+0) | length=87

AGGGGCATAAACACATCATTCCCATCCTTTGCTCTTCTGTTCCATTGTTCATGAGGGCGC

AACTTTTGTTGTTTCTATGGAGTCTTC

>Tb927.10.10110 | Trypanosoma brucei TREU927 | PIG-P, putative | genomic | Tb927_10_v5.1 forward | (geneCodeEnd+0 to geneEnd+0) | length=88

ATAAGGCAGGGGGTGGTCAGCGAGTATGCGTGAGCATGCACGACTGAAAGTTGGGGAATA

CGGCAGAAAAACTGTCTTTTCCATATAC

>Tb927.11.9840 | Trypanosoma brucei TREU927 | hypothetical protein, conserved | genomic | Tb927_11_v5.1 forward | (geneCodeEnd+0 to geneEnd+0) | length=88

ACAGGTTTGCGATGTTCTGACCTACCATTTCCGTGAATTTGTTTGTGGTGAAGTAATGGG

CGACGAAGGAGCATGTATATCACGGAAC

>Tb927.10.12990 | Trypanosoma brucei TREU927 | predicted ankyrin repeat family protein | genomic | Tb927_10_v5.1 reverse | (geneCodeEnd+0 to geneEnd+0) | length=88

ACCCTCAAGTGCTTCTTACAGCGAGAAACACTTTCAGCCACGGGTGCTTACACCGTTAAG

TCTCTTTTGTGTCATTTATTTCCCCTCC

>Tb927.5.4050 | Trypanosoma brucei TREU927 | hypothetical protein, conserved | genomic | Tb927_05_v5.1 reverse | (geneCodeEnd+0 to geneEnd+0) | length=90

ACATATATCACAAATTACTTTTCAAAGCGGTTGGGATACAGTCACTGGAAAACTGGGTTC

GTTGCATGTTTCAATCTGTTGTTGCTGCTC

>Tb927.11.1890 | Trypanosoma brucei TREU927 | WD domain, G-beta repeat, putative | genomic | Tb927_11_v5.1 reverse | (geneCodeEnd+0 to geneEnd+0) | length=90

AACAAAGCAACAGTCAGAGGACACTTTTGGTAATTTTCCGTCGACCACAAATTTAGGCAC

CTCACCTGATTGTCCCGGAAGATCACTACC

>Tb927.9.3480 | Trypanosoma brucei TREU927 | U5Cwc21 small nuclear ribonucleoprotein (CWC21) | genomic | Tb927_09_v5.1 forward | (geneCodeEnd+0 to geneEnd+0) | length=90

AGGGGGTGGGGGAGGAATCGCACCAAGAATGTAGTGCGTGACTTTCTGGTTCTACTCAGT

TCTTCACAAATATGTTGCATTAGCTCCTTG

>Tb927.8.1120 | Trypanosoma brucei TREU927 | hypothetical protein, conserved | genomic | Tb927_08_v5.1 reverse | (geneCodeEnd+0 to geneEnd+0) | length=90

GCTCGCCGGAGAGGCTGATCAGTGAAGGTATTGGGTACATAACCTCGTGTAAAGGAGGTA

GGGACAAACAAGTGGGAAAGGTAGAGAAAG

>Tb927.7.3870 | Trypanosoma brucei TREU927 | hypothetical protein, conserved | genomic | Tb927_07_v5.1 reverse | (geneCodeEnd+0 to geneEnd+0) | length=90

ATTAACCACTGTGACTAAAGGTGGAGGGTATATCATTTGTATTGTCCTGGCTCGTTTCCT

CTTGACTCCGCTCTCGTAGTAGGCTTCTTC

>Tb927.3.4380 | Trypanosoma brucei TREU927 | Tob55, putative | genomic | Tb927_03_v5.1 reverse | (geneCodeEnd+0 to geneEnd+0) | length=90

ATTTCGTTGCACAAGTGTTGAGTCATCCATCATTGTGTGTTTATTACTTTCCTCATTTGT

ACAAGAGGAGTTGAGATATATTAATCCATG

>Tb927.10.8290 | Trypanosoma brucei TREU927 | eukaryotic translation initiation factor 3 subunit 8, putative | genomic | Tb927_10_v5.1 reverse | (geneCodeEnd+0 to geneEnd+0) | length=90

AGTAAAGCAATCACTTAAGTTGTTACAGCTCTACTTTAAATATTAAACGTAATTACGCTA

ACCGACTGCCAATCATATGTTTATGACCGT

>Tb927.3.670 | Trypanosoma brucei TREU927 | hypothetical protein | genomic | Tb927_03_v5.1 reverse | (geneCodeEnd+0 to geneEnd+0) | length=90

ACGATGTTTCCCTGCTGTATAATTTTCGTTGCACATCACAGTCTCTGTTGATTTGAAGTA

ATGTCTTGCGCTGGAGTAGTTAATTTTGTG

>Tb927.11.9700 | Trypanosoma brucei TREU927 | nascent polypeptide associated complex alpha subunit, putative | genomic | Tb927_11_v5.1 reverse | (geneCodeEnd+0 to geneEnd+0) | length=91

GGGGTGCTATGTGAACGGACTTAAGTTCCTTTGTCTTTCTTTATGTTTCGTTTTCTCGTT

TCTTTTATTCCCGTGGTTCGTCACTTTTCCT

>Tb927.9.1810 | Trypanosoma brucei TREU927 | 60S ribosomal protein L35, putative | genomic | Tb927_09_v5.1 reverse | (geneCodeEnd+0 to geneEnd+0) | length=91

ATGCGACATATTGTGAGATACCGGTTCATTTATTTAACCTTTTCTTTTGGGTGTTCTTTA

ATGGCCCCTAACACGGTTATTTTCGCCTATT

>Tb927.8.5840 | Trypanosoma brucei TREU927 | hypothetical protein, conserved | genomic | Tb927_08_v5.1 forward | (geneCodeEnd+0 to geneEnd+0) | length=92

AAAGGAAGTTCTTTCCTGTACCTGGTTCCAAGATTGATGGGGTAACAGCGTTGTTATTGT

ACCTTTGGAGCAGTGGTGGATCAGAGAATAAG

>Tb927.11.3590 | Trypanosoma brucei TREU927 | 40S ribosomal protein S4, putative | genomic | Tb927_11_v5.1 forward | (geneCodeEnd+0 to geneEnd+0) | length=93

AGTTGGTCTCTTTCTGCTGACTTTTTTTTTTACGAAGAAGCAAGGATTTTGGTTCCATTC

ATACTAATTTATTTTACGTTTTATAATCTATTG

>Tb927.9.11280 | Trypanosoma brucei TREU927 | unspecified product | genomic | Tb927_09_v5.1 forward | (geneCodeEnd+0 to geneEnd+0) | length=93

GTCGCTGCTTCTTTCCTTGACTTTATCTAGTTGATTTCGCCTCTTTCAGCTATCAGTACA

TGCGGTGGGCTTTGGGCCTGCTAAAGGCAAATG

>Tb927.11.230 | Trypanosoma brucei TREU927 | cleavage and polyadenylation specificity factor, putative | genomic | Tb927_11_v5.1 reverse | (geneCodeEnd+0 to geneEnd+0) | length=93

ACGGAGACGAAGGTGATGAGGGAGTGTTACCACCATCGATTCACCACTTTCGTTTAGGGA

ACAACTCGGTCACTCGTCGTACTTTCTGCCAAT

>Tb927.11.590 | Trypanosoma brucei TREU927 | hypothetical protein, conserved | genomic | Tb927_11_v5.1 reverse | (geneCodeEnd+0 to geneEnd+0) | length=93

AACATGGGGACTGTTTCACAGCAGGTTGCGTCGGTTTTGGCTTGTCTCGATTTGTGGAAA

CCGCTCCCTTGCCCATTTTAATCTTTTGTTATC

>Tb927.7.3770 | Trypanosoma brucei TREU927 | YjeF family N-terminal domain/YjeF family C-terminal domain containing protein, putative | genomic | Tb927_07_v5.1 reverse | (geneCodeEnd+0 to geneEnd+0) | length=93

GTGGCATTGAGCTTCCTTTACTCGTTATTGGCTGCGGAGGTAATGAAGATACCGAACTGG

GATTGTGTCTGCGTAGGCATGACCGGTAGATTG

>Tb927.9.11250 | Trypanosoma brucei TREU927 | predicted WD40 repeat protein | genomic | Tb927_09_v5.1 forward | (geneCodeEnd+0 to geneEnd+0) | length=94

GGGATCTCGTCCGTAAGCGCGTCAGTTGCCTGTTAAAATGTTGCGATATGTTAGTACGTA

TGAAAGAAGTAACTATAGGTTTGTGTATAAGCGG

>Tb927.10.4550 | Trypanosoma brucei TREU927 | hypothetical protein, conserved | genomic | Tb927_10_v5.1 reverse | (geneCodeEnd+0 to geneEnd+0) | length=94

AAGTGCCTGATTCTTCACTACTCGTGTGTCTATCTTTGTCATTATGCGTGAAAGGGCTGC

AAGACCTTTTTAAAAAATTTGCTGTCGTTGCTGC

>Tb927.10.3380 | Trypanosoma brucei TREU927 | 60S acidic ribosomal protein P2, putative | genomic | Tb927_10_v5.1 forward | (geneCodeEnd+0 to geneEnd+0) | length=95

AGCACCCGTTTTGGCGGCTTCGGTCGCCGTTGGTGTAGCTGGACACGTGGACGGCAACTT

TTATTTCTTCCCCCTTTTTTCGCTTTCATCATTTT

>Tb927.6.3210 | Trypanosoma brucei TREU927 | AAA domain containing protein, putative | genomic | Tb927_06_v5.1 forward | (geneCodeEnd+0 to geneEnd+0) | length=95

GTGACTAGCAGTAGTTTGGCATCCGTGTAAAGCTTTTGTTCGTGAGTAACGAGACTGTTA

AGGTAAGAGTGTAACATGAAATAAGGGAACTAGCG

>Tb927.10.1690 | Trypanosoma brucei TREU927 | nucleotide binding protein, putative (NBP 1) | genomic | Tb927_10_v5.1 forward | (geneCodeEnd+0 to geneEnd+0) | length=96

GTAGTATCAATATGCGGTTTTAACGTTGTTTCTGCAGTATTTTTGGAGAAGTTTTTTGCG

CAGCAGCAATGTTGTGAATTTTTATCGCAGTCTCTT

>Tb927.7.230 | Trypanosoma brucei TREU927 | 40S ribosomal protein S33, putative | genomic | Tb927_07_v5.1 forward | (geneCodeEnd+0 to geneEnd+0) | length=96

AGTAATGGTTTAATTCTACATCTCTTTAATGTGACTCGTTTTTCCCTTACTGAGCATATT

TTAGGTTATTTGTGAGTACTTTCCATTATATTGAGT

>Tb927.6.1270 | Trypanosoma brucei TREU927 | PITH domain-containing protein | genomic | Tb927_06_v5.1 reverse | (geneCodeEnd+0 to geneEnd+0) | length=96

GTAAGTACCTCTTGTCCTCCAGTTGTTCTTAACACGGAGAAAAAGAAGAACCAAAAGGGG

GGAGAGTCCAATGCCGATAGTGGAGCTTAACTTGTC

>Tb927.8.5570 | Trypanosoma brucei TREU927 | transporter, putative | genomic | Tb927_08_v5.1 forward | (geneCodeEnd+0 to geneEnd+0) | length=96

ACTTACCTGCGAATTAAGATATACAGGAATACTTATGTGCTAACTATATGTAAAGAAAAA

GAGCTTCGTATCGTTTCGTTTTCTTTCAATTTGTTT

>Tb927.11.2060 | Trypanosoma brucei TREU927 | 60S acidic ribosomal protein P0, putative | genomic | Tb927_11_v5.1 forward | (geneCodeEnd+0 to geneEnd+0) | length=96

AGCACGATGTTTTACCTGATGGTCTCCGGCATCCATTCTTTTTTACTTTGTTCTTTTTTT

TTTTGCCTTGGTGGTGATCAATACCTTGGTAGACTG

>Tb927.10.4250 | Trypanosoma brucei TREU927 | ADP-ribosylation factor-like 2, arl2 (ARL2) | genomic | Tb927_10_v5.1 forward | (geneCodeEnd+0 to geneEnd+0) | length=97

GGAATCCACAAATACTTGATGCAGGTATAAGTACGCGCGGTTGGTTTATTCAGGGTTTTT

CTCCTTTCCCTTCTCACACCCCGTGTTTTTGTTGTTC

>Tb927.1.560 | Trypanosoma brucei TREU927 | WD domain, G-beta repeat, putative | genomic | Tb927_01_v5.1 reverse | (geneCodeEnd+0 to geneEnd+0) | length=97

AATCGCTCCGCATGACGTCATGTGTTTCCGTGCGGTCATTTAGCTAAGGGAGTGTTACCC

ATGAAGTGCATCTGCGTTAATCGAACATTTCTCGTTT

>Tb927.8.5820 | Trypanosoma brucei TREU927 | Las1-like, putative | genomic | Tb927_08_v5.1 forward | (geneCodeEnd+0 to geneEnd+0) | length=97

GTTGAATAGTTGAATCTAACCGGAGACATGACGCACTGTGTGGGGTTTATATTTCGTTTA

AGCGCTCCTTCACTGTGCAGCACCGAACACACGGTGG

>Tb927.11.6710 | Trypanosoma brucei TREU927 | predicted tetratricopeptide repeat (TPR) protein | genomic | Tb927_11_v5.1 reverse | (geneCodeEnd+0 to geneEnd+0) | length=97

ACAGGTGCTGCAGAGGGATTTCTTGTTGCACGAAAATGCCTATGGAGAGCTTTGTAACAG

CTTGCGGGGGTCGTACCATGTATAGGATCACAGAGAC

>Tb927.8.3090 | Trypanosoma brucei TREU927 | hypothetical protein, conserved | genomic | Tb927_08_v5.1 forward | (geneCodeEnd+0 to geneEnd+0) | length=98

GGTGTAGTGTAGCACATGCTTTGAATTGACGCGAGCGGACTTTGATACGTTTGTTTGGTG

GGCTTCATCTGTGCTCTGGATGGGCGTGTAGGATGTTG

>Tb927.10.12600 | Trypanosoma brucei TREU927 | methyltransferase domain containing protein, putative | genomic | Tb927_10_v5.1 reverse | (geneCodeEnd+0 to geneEnd+0) | length=98

GCGTATGAGATCATTTAACTGCTGTGTGGTGCAAAGTGAATAACCGCGGGGGAAGGGCGT

AACCCTTGGCTTGCTTGCTGCGCTTTTTTACGGATTTT

>Tb927.10.11920 | Trypanosoma brucei TREU927 | hypothetical protein, conserved | genomic | Tb927_10_v5.1 forward | (geneCodeEnd+0 to geneEnd+0) | length=99

ACAATACCGAAGATTTTTGAATAAGAGAACATGTACGATGTCCCTCATGCCAACGAGCAA

GGTGTCGGACGTCGGGTTGTGACTACGCCTGCAGGGTGG

>Tb927.8.1920 | Trypanosoma brucei TREU927 | histone-lysine n-methyltransferase (DOT1A) | genomic | Tb927_08_v5.1 reverse | (geneCodeEnd+0 to geneEnd+0) | length=99

AACCACACGTCTGACATATATCTTTTTGGATAGCTCACTTCAGCTATTCAACTTATCAAG

TGGTATGTTACCTGCTACGTGTTTTTGTATCCATTGTGT

>Tb927.10.170 | Trypanosoma brucei TREU927 | pseudouridine synthase, Cbf5p | genomic | Tb927_10_v5.1 forward | (geneCodeEnd+0 to geneEnd+0) | length=100

ATAAGTAAGTTTTTTTTGTTAAATCGTTAAGAGCACAAGGGGGATAGGGAGAGAGGGTGA

TCGATGATTTATTTTCGTTTTGTTTGTGTCCGCGGTTATC

>Tb927.5.4410 | Trypanosoma brucei TREU927 | hypothetical protein, conserved | genomic | Tb927_05_v5.1 reverse | (geneCodeEnd+0 to geneEnd+0) | length=100

GTGCCTCATTGGTGGATTTCAATTTTTTTTTTTAAAGCCGCTGTTGCAGTCTCGTTCGGT

GTGGTGCATTTCTGTTGTTGTTTCCCTTTCTACTACTCTT

>Tb927.11.740 | Trypanosoma brucei TREU927 | eukaryotic translation initiation factor 5A, putative (eIF-5A) | genomic | Tb927_11_v5.1 forward | (geneCodeEnd+0 to geneEnd+0) | length=100

AGGCCGTAAGTTCTAGGTTAATATATTTTTTTCTTTATCTTTTTCTATACAATGTCTTAA

AAGAAGAGTATGAGACACCCATGTTCTAAATTGTTAGCTG

>Tb927.10.2540 | Trypanosoma brucei TREU927 | adenylate kinase, putative | genomic | Tb927_10_v5.1 forward | (geneCodeEnd+0 to geneEnd+0) | length=100

AACCTTCCTTATCTACCATTGCTTTAAGGGCACTGTGCGCTGCATTGCTGTATCGCTGCT

ATAAGGGTTGCTCAGCTTTTAATCTTTGCTGGTTCACCAC

>Tb927.4.3730 | Trypanosoma brucei TREU927 | hypothetical protein, conserved | genomic | Tb927_04_v5.1 reverse | (geneCodeEnd+0 to geneEnd+0) | length=101

GCATATGGGAGCAACGTGCATTATACAGTGCTAGCGTTGCTAAGGTGACTTGCATGTACT

TGCCCTCTTCCTTCTCCTTTCTTGTGTTTATGCCTGTTAAG

>Tb927.11.14300 | Trypanosoma brucei TREU927 | dynein intermediate chain IC70, putative | genomic | Tb927_11_v5.1 forward | (geneCodeEnd+0 to geneEnd+0) | length=101

AGGGAGCACTCATTAATGTTGTATCCCACCCTCTAGGATATTTATGTAGATCACTGTTGG

TGCCGTAGTTACGTTGTTATAACCGTTCAGTGGCGTCGTTG

>Tb927.11.13560 | Trypanosoma brucei TREU927 | SET domain containing protein, putative | genomic | Tb927_11_v5.1 reverse | (geneCodeEnd+0 to geneEnd+0) | length=101

AAGTTGTGAAGGACCGCCATATCACTGTATATTATTATTTTATAATAGTTGGAAGTGAGA

ACACTCCAGGTGGTGGCAGGGACTTCTTGGGAAACCAGAAG

>Tb927.7.3290 | Trypanosoma brucei TREU927 | WD domain, G-beta repeat, putative | genomic | Tb927_07_v5.1 reverse | (geneCodeEnd+0 to geneEnd+0) | length=101

ACGACACGGTGTGTGGGTTGAAGGTCACTGCTGTGTCTAATGACGCCGTCGTTGTGACGG

TTAATTTGGCGCGCGTAATGGGCCTCCCTCCTGTGCTTTGT

>Tb927.9.7160 | Trypanosoma brucei TREU927 | hypothetical protein, conserved | genomic | Tb927_09_v5.1 forward | (geneCodeEnd+0 to geneEnd+0) | length=102

GTGTAGCGCACAAACTCGTGTCTGCGTGCCCTTTACATTTCAAGAGTTTCTTGTATGCGT

TTTTCATTGAATGTGGTCCAGGACACAATCTTCCACAGCTTT

>Tb927.8.4650 | Trypanosoma brucei TREU927 | hypothetical protein, conserved | genomic | Tb927_08_v5.1 reverse | (geneCodeEnd+0 to geneEnd+0) | length=102

AGCTACAACGTGAGCGCGGCTGACAGTGAGTTTACATCCGTACACGAACGTGCTTTCGAA

TATCTGCTCACGGTGATGAGTGAGGTATTCTGTCTTGTAAGT

>Tb927.11.13800 | Trypanosoma brucei TREU927 | hypothetical protein, conserved | genomic | Tb927_11_v5.1 forward | (geneCodeEnd+0 to geneEnd+0) | length=102

GGCATTCCCCTGTTTCAATTTGAAGGGATATTGAGGTCGAATTTACTTCGCTACTTGGTA

GTACTTTGTTTTCGAAAGATTACTTGTTTCCCTACGCCTCTT

>Tb927.11.8230 | Trypanosoma brucei TREU927 | hypothetical protein, conserved | genomic | Tb927_11_v5.1 forward | (geneCodeEnd+0 to geneEnd+0) | length=102

ACGAACATCTGATATATCCACATGCGATGAAACCATCCGGGTCTGCGTGTCAAAATGGCT

GAGGTAAGAAATTACAGACCTAGAAAGGAGGTACAGAGTTAT

>Tb927.11.12070 | Trypanosoma brucei TREU927 | hypothetical protein, conserved | genomic | Tb927_11_v5.1 forward | (geneCodeEnd+0 to geneEnd+0) | length=102

GATTGAATTTTGTTTTGTTCCCATGAGTAGATGCTAGATTTTAAAAATTATATTCAAACC

CAGTTTGACTTAGTAGTCTTAACCAAATCGTGCTATTAAAGG

>Tb927.10.8310 | Trypanosoma brucei TREU927 | acetyltransferase, putative | genomic | Tb927_10_v5.1 reverse | (geneCodeEnd+0 to geneEnd+0) | length=102

ACGTAACCAGTGTTTTTCACCTGTTTCTTACTTGGCATATCATCTTCTAACATATTAAAC

GTAATTACGCTAACCGACTGCTAATCATATGTTTATGACCGT

>Tb927.10.11390 | Trypanosoma brucei TREU927 | 60S ribosomal protein L6, putative | genomic | Tb927_10_v5.1 forward | (geneCodeEnd+0 to geneEnd+0) | length=103

ATGTGATGGGGAGTTGAGCTTTTCGATCAGCACTACGGTGCACTCATTTTTGTTTTTGTT

TAATTTTTTTTAAAAAAAACTGTACTCTTAAACAAGGGGAAAG

>Tb927.4.3690 | Trypanosoma brucei TREU927 | Iron/manganese superoxide dismutases, C-terminal domain containing protein, putative | genomic | Tb927_04_v5.1 reverse | (geneCodeEnd+0 to geneEnd+0) | length=103

ACCTGTTTCTATCTTCACATATGGGGGCACTTATCGTTGTTTATCTATATGCACGTGCGC

ATGTACATGGGTGGGGTCCACCGATCTGTTTTTCCCCTTGTTG

>Tb927.10.3580 | Trypanosoma brucei TREU927 | hypothetical protein, conserved | genomic | Tb927_10_v5.1 forward | (geneCodeEnd+0 to geneEnd+0) | length=103

GGCCTCACGACTCCGTACATGCGCATGTTTGTCTCTTAAAGGGAAACTTTTGACTAATGA

CTTGCATGTTGCAATGTGAGGAAAAGGTCCCTTTCGCACACGT

>Tb927.7.4590 | Trypanosoma brucei TREU927 | chaperone protein DNAj, putative | genomic | Tb927_07_v5.1 reverse | (geneCodeEnd+0 to geneEnd+0) | length=103

ACCCTATAGCACGCGTAGCAGGATTCGTTTGCGCTAACAACACACAATATCGATATGCTT

AATTACTTGCGCAGCTTGTACTCGGTATTGTTTCCATCTGATC

>Tb927.10.2130 | Trypanosoma brucei TREU927 | Vps51/Vps67, putative | genomic | Tb927_10_v5.1 forward | (geneCodeEnd+0 to geneEnd+0) | length=103

ACTTTCCTTTGTGACCGCTTCTTCATGCTATTTGATTTTCTGCTTTTACTCATTTACGTA

GTGGTATTCTGTCATCTAATCTGCAGAGGGCAGAATATCATTC

>Tb927.6.1860 | Trypanosoma brucei TREU927 | conserved protein | genomic | Tb927_06_v5.1 reverse | (geneCodeEnd+0 to geneEnd+0) | length=104

GGAACAATTCAGCCAAGCATCGTCCATCACTGACTAATTTTACAAAACCGCGACAGCGCA

TGAATCAACGCGTTGGTGAAAAATGGGTGCTTTATCGTTACAAT

>Tb927.11.6310 | Trypanosoma brucei TREU927 | hypothetical protein, conserved | genomic | Tb927_11_v5.1 reverse | (geneCodeEnd+0 to geneEnd+0) | length=104

AGTGCGTTTGTGTTCAAGGGCCTCACCTTTACGAGGCAGCTCCCCTACCCATCAAACAAC

CCTCAAGCATTTGCGTACAAATCGTTTATATGCAGATAACCGAT

>Tb927.10.4620 | Trypanosoma brucei TREU927 | peptidyl-prolyl cis-trans isomerase, putative (PPIase) | genomic | Tb927_10_v5.1 reverse | (geneCodeEnd+0 to geneEnd+0) | length=105

ATACCAGCGAAGGTATTTTTAGTGGTGTAGGAAGGTGGATGATAAAGGTTTCTTTTGAAG

GGGAGAAAGACAGAACTCTATTGTTGTAAAATAAGGGACCAGGAG

>Tb927.6.2990 | Trypanosoma brucei TREU927 | Putative papain-like cysteine peptidase (DUF1796), putative | genomic | Tb927_06_v5.1 forward | (geneCodeEnd+0 to geneEnd+0) | length=105

AGGTACACATACCAAGGATTGAATGTTTACTACCCCTTCCGGCGTCTTTCTTTTAACATA

TGGAATACCAATCAATGCAGTCTGAATTGTTGAAAGGAGGTAGTG

>Tb927.11.920 | Trypanosoma brucei TREU927 | hypothetical protein, conserved | genomic | Tb927_11_v5.1 forward | (geneCodeEnd+0 to geneEnd+0) | length=105

GGGCACTGGCCGGATGATGGGATGCCCGTTCGGATGCATAGTGCTTTCTGTAGCCTTTGC

TACGTGTGCGTGGTGGAATTCTAGTTTTCTTTCGTGTTTTGCCGT

>Tb927.10.1580 | Trypanosoma brucei TREU927 | Domain of unknown function (DUF543), putative | genomic | Tb927_10_v5.1 reverse | (geneCodeEnd+0 to geneEnd+0) | length=106

GGTTGGAGGTGTTGCGAGAGCCCTGTAGCATGTGACTTCCTTCTCCTTTTATTTTGTATG

GAGTTTGGGCAGTGTATCGCTTGTTGCTCTTCCTCTGTCATCAGTG

>Tb927.7.5870 | Trypanosoma brucei TREU927 | hypothetical protein, conserved | genomic | Tb927_07_v5.1 forward | (geneCodeEnd+0 to geneEnd+0) | length=107

AACGTTTCTTGTTGTTGTTTTTTACTGCCGAACATCTGAACGTTAATCGTGTTTTCACCC

ACTCAGTGCGGGCGTATTGACCCGGATTCTCTACGTTGCGTTGCCTG

>Tb927.5.3550 | Trypanosoma brucei TREU927 | hypothetical protein, conserved | genomic | Tb927_05_v5.1 forward | (geneCodeEnd+0 to geneEnd+0) | length=107

GCTTCACCCTGTGCCGGATTTGACGAACTTTGTGCGCACGTTTTTTCCCCCGCCAACCCT

TGCTCTCTTTCTCCCTCGGACGTGTTCTGTTTCATTCGTCTTTATAG

>Tb927.11.7216 | Trypanosoma brucei TREU927 | origin recognition complex subunit 1 (ORC1), putative (orc1) | genomic | Tb927_11_v5.1 forward | (geneCodeEnd+0 to geneEnd+0) | length=107

GTTGTAAAATACCGTCTTAGGACGTGTGTGCTTTAAACGGATTAAAAAAAAAAAAAGCAA

AACAAACCAACAGATCACGCAAAAAAGATGACTCTGTATGTCACTTG

>Tb927.10.3940 | Trypanosoma brucei TREU927 | 40S ribosomal protein S3A, putative | genomic | Tb927_10_v5.1 reverse | (geneCodeEnd+0 to geneEnd+0) | length=108

ATGCTTGGCTGCCGGTATGCAGTTCCAGCGTTGGCACTGGTATCCATATGTTTTCTCTCA

TTGGCTTTTTTGTGCCATGTGTTGTTCTTTTTCATTTTCTTCTCTACC

>Tb927.10.1320 | Trypanosoma brucei TREU927 | hypothetical protein, conserved | genomic | Tb927_10_v5.1 reverse | (geneCodeEnd+0 to geneEnd+0) | length=108

GTATTTACAGGCTGTTTCTTCTTTCTCTTTCCCGTGGTGTAGTTACGATTAGACGCTGTA

AGGTTGGCTGCACTGTTAGGGTCCATTGCCTTTATCCATGCTGTAACG

>Tb927.7.470 | Trypanosoma brucei TREU927 | hypothetical protein | genomic | Tb927_07_v5.1 reverse | (geneCodeEnd+0 to geneEnd+0) | length=108

AGTAAATGCGTCAGGAATGTTACAATGATATGAAATAACAAAACAACTTCAGTGTAATGA

GAGGAGGGATGGGATACGATAGCAGGGCAGAGAAAAAGAAAAGCGAAG

>Tb927.8.4150 | Trypanosoma brucei TREU927 | hypothetical protein, conserved | genomic | Tb927_08_v5.1 reverse | (geneCodeEnd+0 to geneEnd+0) | length=109

GCGGAGTGCAAAAAACAACAACAACAACAAACAAATGATGCGATTGTTTCGTTTCCCTCC

CTCACCGTCTCTTATTTTGAAGATTTTGTCTCATCTTATCATTTCACTC

>Tb927.11.6180 | Trypanosoma brucei TREU927 | 60S ribosomal protein L28, putative | genomic | Tb927_11_v5.1 reverse | (geneCodeEnd+0 to geneEnd+0) | length=111

AGGTCGAGGGAAGGGATTGCCATCTGCTTGTGCGTACTGATGGTGTGATCAGTCGTTTCC

ATACCTTTTTTTGATTTACAGTTCTCCTTTTCGTTTACATTGAGAAGCTTT

>Tb927.6.1700 | Trypanosoma brucei TREU927 | hypothetical protein, conserved | genomic | Tb927_06_v5.1 reverse | (geneCodeEnd+0 to geneEnd+0) | length=111

GTCGATTTGGGTACGGCTACATGTGAACGAAATCTGTGCTTTCCGTTCTTTCTGTGTGTG

GGTACGAGGTTGGGGGCCCTTATCGTGTTGTCGCTGACGTGCGCGCGAACG

>Tb927.10.8160 | Trypanosoma brucei TREU927 | hypothetical protein, conserved | genomic | Tb927_10_v5.1 reverse | (geneCodeEnd+0 to geneEnd+0) | length=111

GTGCGCTGGAATCACATGAGCACGGTCTCGCGGGAAGGTAAAGCGAAAGATTGGTCGTTG

TCGTTTCTCCCCTTCTCCTGTTCTTTTATCCCCACCTCCGTTGACATGCAG

>Tb927.3.4050 | Trypanosoma brucei TREU927 | hypothetical protein, conserved | genomic | Tb927_03_v5.1 reverse | (geneCodeEnd+0 to geneEnd+0) | length=111

ATATCCGTCGGTGCGAACTTTGTTTGCCGGGTGTAGTGAACGTGAGACAGTGCAGCAACT

CCCTGGTGGACTGATTCCAATTCTCTTTTCCGTGTGTGAGGTGACTGTGTG

>Tb927.1.2430 | Trypanosoma brucei TREU927 | histone H3, putative | genomic | Tb927_01_v5.1 forward | (geneCodeEnd+0 to geneEnd+0) | length=112

AGAAGGTACAGAAATGCGTAATTGAGTGAGTGAGTATGAATATGAGTAGGTGTGTGTGTA

TGTATGTATGTATGCGTGAGGGTCAGGGTTGAGACAAATATTCAGATTAGAG

>Tb927.9.12650 | Trypanosoma brucei TREU927 | ubiquitin-activating enzyme E1, putative (UBA2) | genomic | Tb927_09_v5.1 forward | (geneCodeEnd+0 to geneEnd+0) | length=112

AAGCTTCATATTGAATGGCGGAAAGCGGAGAAGGCACTTGGGTGAAGGAGGAAACGCAGT

TACAAGGGAAAGAAGAGAGAAATTATGATGTGGAAAATGGAATAAGGAAGAG

>Tb927.2.4970 | Trypanosoma brucei TREU927 | UV radiation resistance protein and autophagy-related subunit 14, putative | genomic | Tb927_02_v5.1 reverse | (geneCodeEnd+0 to geneEnd+0) | length=112

ACAGCGCGCACGATAACGGAGTAAAGTTGCTTTAAGGGGACTCGGCCCCCACTAGTGGAA

ACGAATGCCGTTCCTGGCCTATTCGAAGATGGTACACGTAACATCCCATCAC

>Tb927.11.9940 | Trypanosoma brucei TREU927 | hypothetical protein, conserved | genomic | Tb927_11_v5.1 forward | (geneCodeEnd+0 to geneEnd+0) | length=112

GCTTTCTGTGCTTTGCTCACAAGGGTGCTGAGTTGGCACTTGACGAGCAGTATAGTAGTT

TCACAGTTGCCCTTTATACCCCCCTTTTAACTTCGTTTGTGCCTATGTCCTT

>Tb927.11.15760 | Trypanosoma brucei TREU927 | GPI transamidase subunit Tta1 (TTA1) | genomic | Tb927_11_v5.1 reverse | (geneCodeEnd+0 to geneEnd+0) | length=113

GGAGCGGATAAGAACGTTAGGTTAAGCCGGTGGTGTTGCTTTTTGTTTGCCATTGGTATT

TTCCCGTCTCGTACGCTCTTGTTTCATTCCTGTTTTCTGTAGTGTAATTGGCT

>Tb927.10.1810 | Trypanosoma brucei TREU927 | RING-H2 zinc finger, putative | genomic | Tb927_10_v5.1 forward | (geneCodeEnd+0 to geneEnd+0) | length=113

GCGGACATCTGTCTCAGGTCATAAAAAGGTTTCTTATTCTCCTCGTTACCTTCAGGTAGA

TACACTTCCACTTCTTTTAGCCCGATCGGGGAAGGTTATGATATGCTAGGTTG

>Tb927.9.3470 | Trypanosoma brucei TREU927 | low molecular weight protein tyrosine phosphatase, putative | genomic | Tb927_09_v5.1 forward | (geneCodeEnd+0 to geneEnd+0) | length=113

GCGCCTCAGCCACGTGTACACACGTATATACATATTTATCACACACAAGTGCGTCTAGGC

GTATCGAGCCGATATCGTTTTGCTTGAGTGGCAATCCTATTACTTCTTTCAGT

>Tb927.10.8200 | Trypanosoma brucei TREU927 | Ribosomal protein L1p/L10e family, putative | genomic | Tb927_10_v5.1 reverse | (geneCodeEnd+0 to geneEnd+0) | length=114

GGGTGGATTGACTGGAACCGCGTGTCCATACATTCACACCCAATGCAGCGACGAACACTA

GTGAGGATAATAAGAAGGGGGTACGCTTGGGTAAATTTAAAGTACCAATTAACG

>Tb927.4.2150 | Trypanosoma brucei TREU927 | hypothetical protein, conserved | genomic | Tb927_04_v5.1 forward | (geneCodeEnd+0 to geneEnd+0) | length=114

ACATGTATATGCGAGTTTTGTGCCCATGACCCAGAACATGGCATGTAGTGAGGATAAAGG

GGGGAAATCGACTATCTTGAAAATATTTTCCCTTTGTATATGTACGTGCACAAC

>Tb927.9.8710 | Trypanosoma brucei TREU927 | hypothetical protein, conserved | genomic | Tb927_09_v5.1 reverse | (geneCodeEnd+0 to geneEnd+0) | length=114

GGCCCATTGTACAACTTCGCTCGCTGTGAGGCGATTTTTGTTGTTTTTTTTTTTTGCTGA

GGGCTGGGTTATTGTTGGTTGTGTGGCATAGTGAAGTGACGGAAGGGGTTAAAG

>Tb927.10.14840 | Trypanosoma brucei TREU927 | Mitochondrial ADP/ATP carrier protein 5a, putative (MCP5a) | genomic | Tb927_10_v5.1 reverse | (geneCodeEnd+0 to geneEnd+0) | length=115

GTGATTTCATTGAGGTACGTTGTACCGTCATCTTCTGTTTTCAGTAGGGTGCGGGATCAC

GGACGCAGTGCGCGCGGGGGAACCTGCCTTTGATGAGATATATGTTTGTTTTAAG

>Tb927.11.13910 | Trypanosoma brucei TREU927 | hypothetical protein, conserved | genomic | Tb927_11_v5.1 forward | (geneCodeEnd+0 to geneEnd+0) | length=115

AAAAACACAAAGAATCCCCTCTTAGTGCGGTCGTGCCTCATTTCTCTAGTTTCTGTCGCG

CTGGCGTCGCGACGCTTCGGGTGCATGGTTGGGTAAGATCATTTTTTTTCTTTCC

>Tb927.10.240 | Trypanosoma brucei TREU927 | peroxin 14, putative (PEX14) | genomic | Tb927_10_v5.1 forward | (geneCodeEnd+0 to geneEnd+0) | length=115

ACGCGGAGGCGTGTAGAGTGGCTGAAAGCTGCAATCTTGTTAGTATATCAATGGAGTGGG

TGAATTTAATAAGAGTGTGCACTTGCAGAGTGCATTCACCCTCTTAAAATTCTTT

>Tb927.10.1760 | Trypanosoma brucei TREU927 | hypothetical protein | genomic | Tb927_10_v5.1 forward | (geneCodeEnd+0 to geneEnd+0) | length=115

GGGCAAACCTCCACAGCATTTCTCACTAAGAACAAAAACCCGAGAAAGGGGAGAAGAGGT

AGCGCAACACAACCGGGCAGCCCAGGGGGTTGCACCAACCTTTGAAGTTCCCATT

>Tb927.10.8170 | Trypanosoma brucei TREU927 | nuclear pore complex protein (NUP155), putative, nucleoporin, putative | genomic | Tb927_10_v5.1 reverse | (geneCodeEnd+0 to geneEnd+0) | length=116

GGTGTACGGTGATAGATTATTTCACTGCGTTGACCTGTCTGCGTCGACAGCGAATATTAA

TTGACTAAAACTGCACAGAAGATGAGGCGTTCCCTTTATTCTTCGCACTACCATCG

>Tb927.8.5320 | Trypanosoma brucei TREU927 | hypothetical protein, conserved | genomic | Tb927_08_v5.1 forward | (geneCodeEnd+0 to geneEnd+0) | length=118

AGTGGGTGAGGACGCTGCTTATGTGCGCCCTCCCTTCCAGCATACGGATAGGACGAAAAG

AAAAAAGACGCCACCTTGAGGAGGACCCTTTCTCCCTTTTATTGCGGTTCATATTACC

>Tb927.1.3810 | Trypanosoma brucei TREU927 | hypothetical protein, conserved | genomic | Tb927_01_v5.1 forward | (geneCodeEnd+0 to geneEnd+0) | length=118

GCAACTCTCTAATCTTTTCCCTCCTTGCACATATGTACTAGGAGCAAACATACCTTTGGT

AGTTTGTGATTATTGCCGCTGCTGTCGTTTTGGTTGTTGCGGTAGTCGAGCCAATGTG

>Tb927.5.2110 | Trypanosoma brucei TREU927 | G-patch domain containing protein, putative | genomic | Tb927_05_v5.1 reverse | (geneCodeEnd+0 to geneEnd+0) | length=118

AGGTCATGACTTTGTTCGTTTTGTCAGCAGCAGGGCTAGTTTCGCTGCGTAATTACTGAG

GATTGTTGTGAAAGCAAATGCTAAAGGTTGTTGAATGTTGCGAAGCAACCCGAACCAC

>Tb927.9.13350 | Trypanosoma brucei TREU927 | hypothetical protein, conserved | genomic | Tb927_09_v5.1 reverse | (geneCodeEnd+0 to geneEnd+0) | length=118

GGTGATATGTTATTTAAGGTTATTTCGCGGGTAACGTTCGTGTTCTCTTTGCTTGCATGA

GCGCGCATGCGTGTTGTGAAGCAGGGGGCTTCCGGGTAAATGCTGGTGTCCGGTGTTT

>Tb927.10.13990 | Trypanosoma brucei TREU927 | hypothetical protein, conserved | genomic | Tb927_10_v5.1 reverse | (geneCodeEnd+0 to geneEnd+0) | length=118

GGTTTCACGTTGCTGCGACTCGTAGTTGCGTGAATGCCCGGTATCGGGGTCATATCGGTC

TATGTTTAGTTACAGGGGCCACCTTGTCTTGTGTCGTGTCACGGTGCGGTAGCGTTTT

>Tb927.10.5360 | Trypanosoma brucei TREU927 | 40S ribosomal protein S10, putative | genomic | Tb927_10_v5.1 forward | (geneCodeEnd+0 to geneEnd+0) | length=119

GGAACGGTTTGGGTTACTGATGGCATTGTGTTTGTGTGGTGTTTATTTTACTAATTTATG

CTTAACTGCGTCTCGTCTTGGTCATCTTACGGTGGTCATTGTTGGTCGTTGGTTTCTAC

>Tb927.7.1040 | Trypanosoma brucei TREU927 | 40S ribosomal protein S16, putative | genomic | Tb927_07_v5.1 forward | (geneCodeEnd+0 to geneEnd+0) | length=119

ATCGCCTGCTGTATGACAGCGTATTGATGAGGTGGCGGCACGATATACACCCGGGTTGCG

GAGTTGCCTTTATCAGTTGCATGTTTTTAATTTCTCTTTTAACTGTTTGTTTCACTATT

>Tb927.1.2340 | Trypanosoma brucei TREU927 | alpha tubulin | genomic | Tb927_01_v5.1 forward | (geneCodeEnd+0 to geneEnd+0) | length=119

GAAAGTGTGACAACGTCGCACCATGTGTAGGTTTTCATTTACGTTCTTTCTTTCTTTTGT

GAATTTGTTTTCTGTCTCAAATGTTTTTAATTCGCTTGGGACCTATGTTTTTCTTGTTT

>Tb927.8.4470 | Trypanosoma brucei TREU927 | chaperone protein DNAj, putative | genomic | Tb927_08_v5.1 reverse | (geneCodeEnd+0 to geneEnd+0) | length=119

AGATGAGCCCCAGAGGTGATATGAAGAGAAGGAGTAAAAGCGCGACGAGGCACATACTCG

TTTTTACGCCATTCTTCGTCCACTTGAGAAATTTGTGCGCTGCTTGTGACGACTGATTT

>Tb927.10.9010 | Trypanosoma brucei TREU927 | leucine-rich repeat protein (LRRP), putative | genomic | Tb927_10_v5.1 forward | (geneCodeEnd+0 to geneEnd+0) | length=119

AGACATGTTCAGTACATTGGTTATCTATTAGGAGTGGGATAGGCATTCTGAACTGGAACT

CTTTACCTGAATCAACTGGGAGAGTTGCTGCAGCTTCATCACACTGCTGTTCTTTGCTT

>Tb927.10.7470 | Trypanosoma brucei TREU927 | RNA-binding protein, putative (RBP15) | genomic | Tb927_10_v5.1 reverse | (geneCodeEnd+0 to geneEnd+0) | length=119

ATGTGACCGCTTCCAAAGGGGGTGGGAGTTTGTTCCGTCGCTAGGGCGGTTATTCTTTTC

TCCCTTTTGTTTGTTCCCTATCTCGTTCCTTTCTTACACTCAACTTTTGGTAACTAAAT

>Tb927.10.14110 | Trypanosoma brucei TREU927 | sjogren s syndrome nuclear autoantigen 1, putative | genomic | Tb927_10_v5.1 reverse | (geneCodeEnd+0 to geneEnd+0) | length=119

AAACATTTCAACCCTCTTGTCGTGGTTTTCCCGCACAGCTGGACGAGCGAACCACAAGTT

AAACCTGCTGCTCTCGTAAAGAGTGGTATCCCTAAAGTTGTCTGCTTTTCCCCACTATT

>Tb927.6.2170 | Trypanosoma brucei TREU927 | co-chaperone GrpE, putative | genomic | Tb927_06_v5.1 forward | (geneCodeEnd+0 to geneEnd+0) | length=120

GCGTAGTTAAATGTGCTTCCGACATAGTACGATATTAAGAAGAAGAGGGAAAGTAAAAAA

GGAGGGCACCAAGATATAATAACCACCATTGACTTAAATAAATGAGAGAGCAGATGATTG

>Tb927.11.14490 | Trypanosoma brucei TREU927 | RNA polymerase subunit, putative (RPB7) | genomic | Tb927_11_v5.1 forward | (geneCodeEnd+0 to geneEnd+0) | length=120

GTGTTGATTGTAATGAAAGTGGCAAACTGAGAGAAATGTGGATGAGGAAATGCAAGAACA

GCGGAGAGGGACTGAATCACAGTTTTAAATGATTATAAATGCATATATGCATCTTTCAGT

>Tb927.6.4950 | Trypanosoma brucei TREU927 | mago nashi-like protein, putative | genomic | Tb927_06_v5.1 forward | (geneCodeEnd+0 to geneEnd+0) | length=121

GTCTGATGAGACGGAGTCTGATAACAGGTGTGGCTGTGAGTCTGAATCACTCGCTGATTC

TTTGCCTTTTTTTCCAGGGCCGGAGGAACGAAAAGGGGTGAATGCGACTTGTTGCGGATC

T

>Tb927.11.9860 | Trypanosoma brucei TREU927 | EF-hand domain pair, putative | genomic | Tb927_11_v5.1 forward | (geneCodeEnd+0 to geneEnd+0) | length=121

ACTGAAATGAAGTCAGCGAAGATATTCAACTTCTTTACCTGTTCAGGGGAGTTTCCAGGG

TATGTATACAGGGGAAAAATATACGGCAAAGTGAAGATGAACATATCTAAGCAAATGAAA

C

>Tb927.8.2090 | Trypanosoma brucei TREU927 | cyclophilin type peptidyl-prolyl cis-trans isomerase, putative (PPIase) | genomic | Tb927_08_v5.1 forward | (geneCodeEnd+0 to geneEnd+0) | length=122

ATGAGGACGAATATGCGGGTTTTTACGATCTGGTGAGCCTTTAGAGTCCCTCACCTTTGT

TTTGTTCCCTTTAAGATTGTCTTGGGTAAATTTAAATGGACACCATGTGACTGCCTTGGT

TG

>Tb927.3.2330 | Trypanosoma brucei TREU927 | kinetoplastid kinetochore protein 17 (kkt17) | genomic | Tb927_03_v5.1 forward | (geneCodeEnd+0 to geneEnd+0) | length=122

GACTGCCAATTCGTATTTACTGGCATGTTTTACTCCGACTTGTCCTTCCACGCTTGTATA

TAAATCACTTCTCGCGTGTATGCGGCAAACCATGGATCGTTCGTTGCCATGGCTGAGTAA

TG

>Tb927.9.11220 | Trypanosoma brucei TREU927 | hypothetical protein, conserved | genomic | Tb927_09_v5.1 forward | (geneCodeEnd+0 to geneEnd+0) | length=123

ACTGATTTTGTGGGCGTTTGTTTTACAGAGATAACTAGCATGCCGTCTCCCGAGTGTCAT

ATCACTTGTAAGATAGGCGGAGGCATTGGTATGCTAATATCGTGGGTGAATTTATGCGAT

GTT

>Tb927.10.10130 | Trypanosoma brucei TREU927 | mitochondrial RNA binding complex 1 subunit (MRB10130) | genomic | Tb927_10_v5.1 forward | (geneCodeEnd+0 to geneEnd+0) | length=123

ACAAGGCATATGAACTGCATCTCCGTCGATGCTGTCACTTCGTTACAAAGAGAGGAGAGT

TCTTGGAGGGAGGGCCATTTCCTACACGTACCTCTTAGGATATTTTATGTGTGCGGGTAT

GCG

>Tb927.9.5440 | Trypanosoma brucei TREU927 | actin, putative | genomic | Tb927_09_v5.1 forward | (geneCodeEnd+0 to geneEnd+0) | length=123

AGGCATGATTCCACAGGAGGGTTGAGTGAAGTGATGTGTAGAGTGAAAGCATGAACGATA

TGTGCTGCTTGATCACATGTTCATGGATGTGCACGTATTGGGTTTGTGCATCGCGACATA

TAG

>Tb927.4.1380 | Trypanosoma brucei TREU927 | D-Tyr-tRNA(Tyr) deacylase, putative | genomic | Tb927_04_v5.1 reverse | (geneCodeEnd+0 to geneEnd+0) | length=124

AGTTCTTTTTTGTCATGGGTTTGTGAAGATGTTTCGCAGCTGGCAGCAGGGTCATCGCCT

CGGGTTTACCGATGGAGTGATGTGCGCCGGTTTGTCGGAATCGTTTGTTGTTCCAATGAA

ATAC

>Tb927.10.15580 | Trypanosoma brucei TREU927 | dynein light chain, putative | genomic | Tb927_10_v5.1 forward | (geneCodeEnd+0 to geneEnd+0) | length=124

AAAAACTTCCAAGTACAGCAACCACCAGCGTAACACCCCTACAAATGAAATCGGTCGGCT

GTGCAATGTCATTTGTTTGTTCTCTTGCCCTTGCATTCACGGAGCTACTTATATGATGCA

CTAG

>Tb927.2.1560 | Trypanosoma brucei TREU927 | cyclophilin-type peptidyl-prolyl cis-trans isomerase, putative | genomic | Tb927_02_v5.1 reverse | (geneCodeEnd+0 to geneEnd+0) | length=125

AGGGCATTCTCAGGAAGTTAACAACGGCTGTTACGGTTGTGGTAACTGTGTTAAAAAGTA

CGCGAGCTTTTTTGTTTTTTTTTCACTTAGGACTGTGATCAAGTTCGATTGTGTGTTGCC

TCGTT

>Tb927.11.8280 | Trypanosoma brucei TREU927 | hypothetical protein, conserved | genomic | Tb927_11_v5.1 forward | (geneCodeEnd+0 to geneEnd+0) | length=125

GGTTAATACTTGGTGTTTTGATGCCTTCCCCATTGTGTATGTTTTGTTTGAGTTTCAGGT

GAAGTCCGACTGCACATAACGTCTCCCATACCCCTGTTTAATTATGCCACTGCATAGCAA

GAGTG

>Tb927.4.3670 | Trypanosoma brucei TREU927 | CBF/Mak21 family, putative | genomic | Tb927_04_v5.1 reverse | (geneCodeEnd+0 to geneEnd+0) | length=125

ACCCAGAACCGTTCGATGTGTGTGTGTGTGGACTCGATAATCATAACGCAGTACCGATCG

TATGGAGGTATACGTAGGCTGTGCGAGACGTCGTTTTGGCGCGTAAGACTAATATTCGGG

GTGTC

>Tb927.11.7240 | Trypanosoma brucei TREU927 | dynein regulatory complex subunit (CMF70) | genomic | Tb927_11_v5.1 forward | (geneCodeEnd+0 to geneEnd+0) | length=126

GGCAATTCCTGTTGGGGTATGAGCACACTTTTCATCGTTTCCTCCTTTAAGCAATATGGG

GAAATGGCAATGTAGAGTTTACGACTAGAGCAACAGTGGTAAAGACCAAATCCGAACAAG

CGGTGT

>Tb927.9.8580 | Trypanosoma brucei TREU927 | hypothetical protein, conserved | genomic | Tb927_09_v5.1 reverse | (geneCodeEnd+0 to geneEnd+0) | length=127

AGCAATTTTCTCTAGGGGTGGTAGCCTCAGTGAAAGGCTTTTGCCGTATATCCCTACATA

TATACTCCGGTGGCCGGTCGATACTCTTCCCAGCCTTCTATTTCTGTTATCAGTTTGTTG

TTTCTTT

>Tb927.10.10380 | Trypanosoma brucei TREU927 | ATP-dependent DEAD/H RNA helicase, putative | genomic | Tb927_10_v5.1 reverse | (geneCodeEnd+0 to geneEnd+0) | length=128

AAGTAAGGACAGAAAGGCTGTGAACCAAAAAAGGGATAAACTTTTCTCCGCCCTTGCGAC

ACCCAAGATAAGGGTTAGGGTAAAAACGAAAGTCTCCCTATAACTTGTGCATTAGTACTC

AAGTGTGT

>Tb927.10.12420 | Trypanosoma brucei TREU927 | predicted S. cerevisiae Got1 homologue | genomic | Tb927_10_v5.1 reverse | (geneCodeEnd+0 to geneEnd+0) | length=129

ACCCCAAGGCGCCTTTGAGAGTTAATTAAAGGATGATGGAATTAAGCAGAAACTGAGGTG

CAAACGATCATTGACCTGCTCCTCGTTCTCTCTGTGTGTGCTTCGGCATATATCTGCACG

GCTACCATC

>Tb927.10.8090 | Trypanosoma brucei TREU927 | hypothetical protein, conserved | genomic | Tb927_10_v5.1 reverse | (geneCodeEnd+0 to geneEnd+0) | length=129

GCATTTCTCTAATGAGATGATTATACTCCTTGTGCTAAACGGCAAGAGCGTTACCCTCCC

CCGTCTCTCACGCATGAGCTTTCTCTTACTAGCGCAGACGAAAACGAAAAGTCACTTGCG

ACAGCCCTG

>Tb927.4.1720 | Trypanosoma brucei TREU927 | nucleoside diphosphate kinase, putative | genomic | Tb927_04_v5.1 reverse | (geneCodeEnd+0 to geneEnd+0) | length=129

GATAGCAACAAAGGGGTTCATCGACTTTTAGTTTTTTTTTCCACCCCTGCCTGACTTTCG

GTGACAAGTGAGTGGAGGAAAACTCGAAAGTCGTTGCCTCTTGGTTTGCTGGGTTCCGTC

TAGTCATGT

>Tb927.6.3340 | Trypanosoma brucei TREU927 | Domain of unknown function (DUF814), putative | genomic | Tb927_06_v5.1 forward | (geneCodeEnd+0 to geneEnd+0) | length=130

AACTCTGGAGATCTTTCCACGAAGTAGCAACTTCTTTCCCCCCTCCCCCTTCTAGTTGGT

GTATCACTTCGCCAGTATACATCTCAAGGGTGTCACGTTTAAGTGCTGCTGACGTTTCCT

TGGAAAAGAG

>Tb927.9.9720 | Trypanosoma brucei TREU927 | glucosidase II beta subunit-like protein, putative | genomic | Tb927_09_v5.1 reverse | (geneCodeEnd+0 to geneEnd+0) | length=130

ATATGGGAAATCTGCGGTGTGCAAACAGGACCCTTGCATGGAAGCGGCTTGAACCGCCGG

TGGATGTGTGGTTCGTTCCATTTGGTGTCGGTTGCTGAACTGATAATATCTCCCTCTTTT

CTTTGCCCGT

>Tb927.3.970 | Trypanosoma brucei TREU927 | NAD binding domain of 6-phosphogluconate dehydrogenase/NAD-binding of NADP-dependent 3-hydroxyisobutyrate dehydrogenase, putative | genomic | Tb927_03_v5.1 forward | (geneCodeEnd+0 to geneEnd+0) | length=130

GTAATACCCACATCAGGGAGATGCAGTCTTTCTCACTTCCTTGGAGGGTACTAAAAGTTA

CGGTTGTTCACAGTTTTTCGTAGGTTGTTGTTTTTGTGTAACGTGCTGCCGCAGCGTGCT

TTACCTGTTG

>Tb927.4.1570 | Trypanosoma brucei TREU927 | hypothetical protein, conserved | genomic | Tb927_04_v5.1 reverse | (geneCodeEnd+0 to geneEnd+0) | length=130

ATCGGTTGCTGCAGCCTCATTGCCGTTTGGTAAAGTGGTGGCGTGTCTTTGTGCACGCGT

AGGTGTACAGTGTTGGGGTGAGTTTCCAGTGGGTTACCATTGATGCGGGAGGGGGATGTT

GAGCAATTGC

>Tb927.10.5570 | Trypanosoma brucei TREU927 | hypothetical protein, conserved | genomic | Tb927_10_v5.1 forward | (geneCodeEnd+0 to geneEnd+0) | length=130

AGGACCACGAGGTGTCGTAGGACGCGGGCGTAGACTTGCGTGCGATTGGCGCATTTTGGA

GAAGCGGCAACTTACAAACTGAGTACAAACTTTCTGAGCATCTTTACCTTCCCCTGTTGC

AGCAATGCTT

>Tb927.11.9100 | Trypanosoma brucei TREU927 | Domain of unknown function (DUF4586), putative | genomic | Tb927_11_v5.1 forward | (geneCodeEnd+0 to geneEnd+0) | length=131

ATGGTGTGCTCTGGTTTCGAAGGGAAAAAGAAGGTGTTTCAAATGATGACAAACAATAGC

GAAACAACAAGGGGGTGAGCGCGATGCTTGATGATAATGATGATGATGTGTGAGCTAGAA

TGAACAAGAAG

>Tb927.1.890 | Trypanosoma brucei TREU927 | hypothetical protein, conserved | genomic | Tb927_01_v5.1 reverse | (geneCodeEnd+0 to geneEnd+0) | length=132

GATGCCCTTTCGCACATGTTGCCAATAGTATATGTTTTTTTTTGATGTGTGGACATGCCA

AATGCAGGAGCTGAGATCCAGTATTTCTGAATATCATGTTCTGTCTAACTCCGTTCTATT

CCTCCCCCTTTT

>Tb927.6.4160 | Trypanosoma brucei TREU927 | hypothetical protein, conserved | genomic | Tb927_06_v5.1 reverse | (geneCodeEnd+0 to geneEnd+0) | length=133

GTTGGGCCCAGTTTCTTTTTTTTTTTAATAAAGTTGATTATCATTTTGCGGTTTGGGCAG

TTTGCGAAGCATGATAGGTGTGTTATAGTCGTGTGTTCCTGTACGGGGGATGCTGTGTCG

TAGCGCTCTTCTC

>Tb927.4.430 | Trypanosoma brucei TREU927 | proteasome beta 7 subunit | genomic | Tb927_04_v5.1 reverse | (geneCodeEnd+0 to geneEnd+0) | length=133

AGTTCGGTAATTTGCGCAGGAAACAAGCAGGAGGGGTGGGTGGAGCTCGTAACTTTGTTT

CGTTTTGTTTTGTTTGTGTGCAATGTTAAGAGAAGTAAGTAGAGAACAAACATCATGATG

ATCAAACGGATGG

>Tb927.11.11030 | Trypanosoma brucei TREU927 | exosome-associated protein 4 (EAP4) | genomic | Tb927_11_v5.1 forward | (geneCodeEnd+0 to geneEnd+0) | length=133

GGAGATAAGTGTTTCGCTATAACGATAAAAATTTTCCATTCCACCTTTCATTTGCAGGAC

CAGTAAAAGCATGAGTGCGGTGCGGGGGGGAGAAGTTGCGCCCGCACGAATAAATTTGGT

TGCAGACCTCAGC

>Tb927.10.13710 | Trypanosoma brucei TREU927 | Activator of Hsp90 ATPase, N-terminal/Activator of Hsp90 ATPase homolog 1-like protein, putative | genomic | Tb927_10_v5.1 reverse | (geneCodeEnd+0 to geneEnd+0) | length=133

AGTAATGCTGAAATCCTCCTTTTTTTTTTATCATCACTCGTTCCCCCTCCCTCTGTTGAC

ATATTATTCACTTATTATTATTATGAGTAACCAGGAAACTACTAAACACCTATTATTAAT

TAGTACTCTACTG

>Tb927.10.14040 | Trypanosoma brucei TREU927 | vacuolar ATP synthase subunit c, putative | genomic | Tb927_10_v5.1 reverse | (geneCodeEnd+0 to geneEnd+0) | length=133

GGAGGTACAGACATTACGAGTCGGGTTTTATTTATTGCGGTACATACGGCTCTTGCTCTC

TTTTTTTGTTTTTCGGAAACACGCATATATATGTGTGCGTATATATGCTGTTGGTAGTGC

TCCTCTTTCGGTG

>Tb927.6.4250 | Trypanosoma brucei TREU927 | hypothetical protein, conserved | genomic | Tb927_06_v5.1 reverse | (geneCodeEnd+0 to geneEnd+0) | length=134

GATGTTCGTGTGTCGGCGTGTAGCCATTTGCTCGGGAATGGCGGATGCTTTTCTTTTTCT

TCTTCCCCCCCTCATCGACTAGTTGGTGTTACGCCGCCGTAGCTCTGTGTCGAAGGAGGA

CCAAGCGTGAACAG

>Tb927.11.15880 | Trypanosoma brucei TREU927 | 60S ribosomal protein L27, putative | genomic | Tb927_11_v5.1 reverse | (geneCodeEnd+0 to geneEnd+0) | length=134

AGCTCGCACTGGCTCCTCTCTGGTACTTTTTGTTCTTTAGGATTTTTTTTTGGAGCACTT

CTTGCTGACAGTGGTAGGGTTCCCACCGCACGAGGTGGCAGGTGTGCTGCACTCCTAAAC

GTTTTATAATACAT

>Tb927.10.5300 | Trypanosoma brucei TREU927 | eukaryotic translation initiation factor 6 (eIF-6), putative | genomic | Tb927_10_v5.1 forward | (geneCodeEnd+0 to geneEnd+0) | length=134

ATGTGGTGTTAATCGTCGGGCGCATCCGAGACGAAGATAATGGACAATTCATCTTTCGAA

AGAACATTATCTGTTGTTCCGCTTCTCTTTTTAATAGTAATCAACACTAACGCGGGCTAA

AGTACCGAGTCTTT

>Tb927.11.7310 | Trypanosoma brucei TREU927 | RNA binding protein, putative | genomic | Tb927_11_v5.1 forward | (geneCodeEnd+0 to geneEnd+0) | length=134

GTTTGTCACCACGTGGCGGAAGTCGGTAGGGGTGATGACGTTACTGAACCGAAAACGAGG

ATAAGTTAACAGTGCTCTTTCCGTTTTGTTTTCTGACCTTACGCTCGTTATGTGTAAGAA

GGGATAATACGTGT

>Tb927.4.4980 | Trypanosoma brucei TREU927 | adrenodoxin precursor, putative | genomic | Tb927_04_v5.1 reverse | (geneCodeEnd+0 to geneEnd+0) | length=135

ATATCGACGAATATCTATTTCGGTTGACTTCTTCCGTCGTGCATACACACACAAACGCAC

ACACGTGCGCACGTGAAGGTGATGCGGAGAACACAAAGACGTACGGCAAATAATAATTGA

TCCTGCCTTTTTCTC

>Tb927.11.13890 | Trypanosoma brucei TREU927 | AKAP7 2'5' RNA ligase-like domain containing protein, putative | genomic | Tb927_11_v5.1 forward | (geneCodeEnd+0 to geneEnd+0) | length=135

GTTGGTGGTTTTGTGTGCGTGTACACACATTCCCATATATTTGTGGAAAGAATTGCCTGG

GACCGATGGACAAACTCATATCACGCGCTTCTCATTTGCTGCCACCATTGTCAGCGTTGT

TTCCACCTTTGTTCT

>Tb927.3.4910 | Trypanosoma brucei TREU927 | signal peptide peptidase, putative, aspartic peptidase, clan AD, family A22B, putative | genomic | Tb927_03_v5.1 forward | (geneCodeEnd+0 to geneEnd+0) | length=136

GAAGATATGTATTGTTCCCTTTTCACTTCTCTTGCATCAGGAAAGTCGTTCTAGAATCAA

TGAGACCACTTCGGTTTCATTCACTTAGCAGCCACAGAGGCGCGGTTGGAAACCTCTTTG

ATAGTTTGCAATCATT

>Tb927.10.14640 | Trypanosoma brucei TREU927 | Rtf2 RING-finger, putative | genomic | Tb927_10_v5.1 reverse | (geneCodeEnd+0 to geneEnd+0) | length=136

ACGTAGAAGTATGCTGAGTAACGGGAAAGGGTCCATATCGTCACCGAACATTACGAATGT

GTTGCAGACAATACATCGGTATGGCTCGGGGGTTTACCTATCGCCTCAGCTATTTCTTTT

ACATACTTCGGTTGCC

>Tb927.3.4480 | Trypanosoma brucei TREU927 | hypothetical protein, conserved | genomic | Tb927_03_v5.1 reverse | (geneCodeEnd+0 to geneEnd+0) | length=136

ATGGAATGTGTGCGATAACCTCTGGAGCGTGGGCTGAAATGTGGGTGAATGGAACCCCTC

CTAGGGGTGATGTTGGTTATTTCATGTGGATGACAAGGCGGGTGCGTTTTGGTATTCGCC

TCTCCCAGGGTTGTGC

>Tb927.10.13970 | Trypanosoma brucei TREU927 | uracil-DNA glycosylase, putative | genomic | Tb927_10_v5.1 reverse | (geneCodeEnd+0 to geneEnd+0) | length=136

GGTTTGGTCAGCTTACTTTCCCTTTTGTTGAGGGACTATACATTTTTGCTGCTTCCTTAC

TTACTCCACAGCTTTTAGTTCGTAATATTTTACTTTGTTCCAGGATTTAAAGCATAGCTT

AATACAACTGCTCCAG

>Tb927.11.5890 | Trypanosoma brucei TREU927 | Domain of unknown function (DUF4505), putative | genomic | Tb927_11_v5.1 forward | (geneCodeEnd+0 to geneEnd+0) | length=137

AATAGGACAATGAATTTGTCCGTGAGAAATGATGCCCACCTTCTACAGATAACTTGCTTG

TCCAAGCTGTGGAGTTTGTGACGTGTACACGCGCCTCTTAACTTCATGTATTTTATTTCA

AACGAAACCCTCGTATC

>Tb927.11.15230 | Trypanosoma brucei TREU927 | cytosolic coat protein, putative | genomic | Tb927_11_v5.1 reverse | (geneCodeEnd+0 to geneEnd+0) | length=137

GTGTAGGTGTATGGAATAAAGATGTTTAACTTTCCATTAAAGGGGAGTAGGAGCGTCAGG

GACGGTTGTTAAGACATCAGTGTTCATGTGTGAGCGTACTTAAGGTGCACGTGGGAAAAT

GTGTGTACCTGTTGCGG

>Tb927.11.11870 | Trypanosoma brucei TREU927 | ADP-ribosylglycohydrolase, putative | genomic | Tb927_11_v5.1 forward | (geneCodeEnd+0 to geneEnd+0) | length=137

AAAGGGTGGGATGGGACACCGCGTCTCGAGGGTTAGGGAAAGGGGGGAAATGGAAATAAG

AGGAACTGACCTTGACCTTCTCCCTTGTCCGTTACGGCTCTGCTTGCCATTGGGCTGCAT

TGTCTACTCAAAATGTG

>Tb927.7.4790 | Trypanosoma brucei TREU927 | proteasome beta 6 subunit, 20S proteasome beta 6 subunit, putative (BETA6) | genomic | Tb927_07_v5.1 reverse | (geneCodeEnd+0 to geneEnd+0) | length=138

AACGCGCTCCCGTGCGTGGCGTCGTATGTGGACGTTTGCAGAGTAGTATAAAGGCGAAGG

GAAAAGAAGAGGAGGGGAGGAAAGGAGGGAAGAACGCGACAGGCGTATGCTTCGGTCCGT

GCGAAGACGTCAAACGTG

>Tb927.8.3070 | Trypanosoma brucei TREU927 | hypothetical protein, conserved | genomic | Tb927_08_v5.1 forward | (geneCodeEnd+0 to geneEnd+0) | length=138

GCGGCGGTGCGTGGTTGCCGCTAGTGTGTCCGCTGCATCCCCGTGCAGATGCGCTGTTCG

TTCATTCAGGCAATTTTGTGTCATTTTTTTCACCTGCCGCCACATTGGTTTTCTCTGAGC

GGTCTCTCTCCTCTCGTT

>Tb927.11.14660 | Trypanosoma brucei TREU927 | hypothetical protein, conserved | genomic | Tb927_11_v5.1 reverse | (geneCodeEnd+0 to geneEnd+0) | length=138

ATAGGGCGCAGCCGCTCACGTTGTGGCGGTGGTCTTCCGGCATATACACCACGCGGCGAG

TATATAAATGACGGAAAAAAGACCGTTGCCATATTACTTATCCATGAATCGACAAGATGG

CATTTGTTACAGTGCTTT

>Tb927.10.6190 | Trypanosoma brucei TREU927 | aldehyde dehydrogenase, putative (ALDH) | genomic | Tb927_10_v5.1 reverse | (geneCodeEnd+0 to geneEnd+0) | length=138

GAAAGTTGCACTTCTTACCGTCACATGGCTGGTTGACATTGTTGTTATCGTTTGTTTCTG

TTAAAACAAAAAGTTCTATAATTATCTATGGTTTTTATGCGACTGTTGTCGGTTGCGTGT

GCGTGCGGTTGGACGTGT

>Tb927.9.3540 | Trypanosoma brucei TREU927 | hypothetical protein, conserved | genomic | Tb927_09_v5.1 forward | (geneCodeEnd+0 to geneEnd+0) | length=139

GGATACAGGAGGAAACGATCGGGCATCCGAATATTCTTGAGAGAAGTTGAGGGGTGAAGA

AACTCGTCGTGGGACGCTCGCACCGTTGAGTATACTTCACTAGTTTGCCACTGTGCGGCA

GGGCCCACTTGCGTACAGG

>Tb927.11.10510 | Trypanosoma brucei TREU927 | ubiquinone biosynthesis methyltransferase, putative | genomic | Tb927_11_v5.1 forward | (geneCodeEnd+0 to geneEnd+0) | length=139

GAAAACTCTCCCGATTGAGCTGTGTTGTGCAACATTTGTTGAAAACATACAAGCAGAATT

AAGTATAGTAGTCGCAGGGGAACTTTAATCATTCATATGCCACAAGGCGTATAATAGTGG

TGGGCTTATATATTTTCTT

>Tb927.6.570 | Trypanosoma brucei TREU927 | hypothetical protein, conserved | genomic | Tb927_06_v5.1 reverse | (geneCodeEnd+0 to geneEnd+0) | length=139

AAGCTCCACTGACCTCGCATTGAGCGCTGGGTGCGCTCTGGAACGCATCACCCCTCCTCT

CACCACATTTCCTCCTTATTAGATACGAACGCAGGTTTTCATGGACGAAACTGAGTATTG

GAGGCTATTTACCCGCATG

>Tb927.4.2770 | Trypanosoma brucei TREU927 | hypothetical protein, conserved | genomic | Tb927_04_v5.1 forward | (geneCodeEnd+0 to geneEnd+0) | length=139

AAGGCTATTATATAACGTCTGCGTTTCGCACAATTTGCCCTCTCGTTTGCGCAGGGAAAT

GTTTCCCAGTAGTATCGTTTCGTCTATTTAATGTGTGTATCTGTTGGATATCTTTCGTTC

TGTATGGGAGGAAGAAAGG

>Tb927.7.1740 | Trypanosoma brucei TREU927 | 60S ribosomal protein L7, putative | genomic | Tb927_07_v5.1 forward | (geneCodeEnd+0 to geneEnd+0) | length=140

AACTCAGCGGCGACGGTGGAGCACTGTGCATTATTACTTTGAAATAGTATTTTGTTTTTC

GTTTGATGTGGGTGTGTTGTGTGTACTATTTTTCCACGGCTACCTATTCATCTTTCTCGG

TTCTACTTAGGTGGAGCTTT

>Tb927.10.10370 | Trypanosoma brucei TREU927 | hypothetical protein, conserved | genomic | Tb927_10_v5.1 reverse | (geneCodeEnd+0 to geneEnd+0) | length=140

ACGACCAGCGGTTGCCAGTCAGTAGATGTAGAAAAGGAGGCAGTTTCTCAAACGCCTAAC

GTCACGTGTATTTGCATGAGTAACACCTTCCACAAGGAACTGTGATGACTGAACACCAAA

AGTATACCTTAATATCCGCT

>Tb927.10.1880 | Trypanosoma brucei TREU927 | hypothetical protein, conserved | genomic | Tb927_10_v5.1 forward | (geneCodeEnd+0 to geneEnd+0) | length=140

AATCTTGAAGTAGTTGTGATTGTATCGTGTTGCCGATGGTAGCTGTGTGGAGGACGTGGT

GGATTTCACGCATTTTATGGAGTTTTGTGTCCCCCTTCTCGTACTTGGTATCGTTAACTT

GTTTGCGTAACCGCAGAGTG

>Tb927.11.3540 | Trypanosoma brucei TREU927 | hypothetical protein, conserved | genomic | Tb927_11_v5.1 forward | (geneCodeEnd+0 to geneEnd+0) | length=140

AGGCTTAAGGTTGCGCTTCGCAAGAAGGTTCGTTTCGTTGCTTCCCACATCACGGCCCTT

CCTTACTACATGCGCTCTCATCAGGGTGTCCGGCATTTGGGTGGCATGCTCAGCGCCATC

TTCATTCCCTTCTTGTACTT

>Tb927.8.6150 | Trypanosoma brucei TREU927 | 40S ribosomal protein S8, putative | genomic | Tb927_08_v5.1 forward | (geneCodeEnd+0 to geneEnd+0) | length=141

GGAGGCGTTATGAAACCATGGTATCTTGTTTGGGCGCTCACCCGCTCGTATTTTTGTTTT

TTTCTCTTTTCGTAAATCATTTACGTCGGTACTTTTTATCTTTATCACTAATGACTGTTT

TAAACGTAACAGACATATGAT

>Tb927.11.6140 | Trypanosoma brucei TREU927 | 40S ribosomal protein S15A, putative | genomic | Tb927_11_v5.1 reverse | (geneCodeEnd+0 to geneEnd+0) | length=141

GGCGCCAATGATGGCAACAAGGGGAGATGCTTTTGGCGTTGGCACGCTAAGTCGTACCTC

GAAGTGCGTTACTCAGCTTCCATAATCGCTCCGTTCTGCTTTTCAATTTTCCTTTTCTTA

TTGCCTCAAAGAAAAATGAGT

>Tb927.11.14900 | Trypanosoma brucei TREU927 | coatomer subunit epsilon (COPE) | genomic | Tb927_11_v5.1 reverse | (geneCodeEnd+0 to geneEnd+0) | length=141

GGGGCGAAAGTAACAAAAAAATTTTACTTTCTTTGTTGTTGTTAGTTTTCTTGGTTATGA

GGGAAAGATAACATCTAAAAGAAAAACAGGAGGGGGGAGTGTTATTTCCTAATTCCGGTA

AAACCTCAACATCAAAACACT

>Tb927.7.220 | Trypanosoma brucei TREU927 | CDP-DAG synthase (CDS) | genomic | Tb927_07_v5.1 forward | (geneCodeEnd+0 to geneEnd+0) | length=141

GTCCATGTTGGAAGGGCACGAGTAATAAAACGGAGCACGGCGACCTGTCATGGTTAGCGT

TTAGAATTGCTGCGAGCGAGCATGTTACCATAGTTGTAAGAAGCTGCTACCTGTATTGGT

CTTTGCCGTTATATCGTATGC

>Tb927.9.2280 | Trypanosoma brucei TREU927 | hypothetical protein, conserved | genomic | Tb927_09_v5.1 reverse | (geneCodeEnd+0 to geneEnd+0) | length=141

AGTACTGGGAGGCTGGGAGTGGTCAGGCTCGAAAGGTTGGTGTTATGGCCGCTGGGAGTT

AGCGGTGGTGACCCCACGGATGGCTGCGTGCCGAGGTGCAGGGGATTCTGCATTGCCCCG

GTTTCTGAATTTGTGTTCTTT

>Tb927.10.14210 | Trypanosoma brucei TREU927 | TFIIH basal transcription factor subunit (TFB5) | genomic | Tb927_10_v5.1 reverse | (geneCodeEnd+0 to geneEnd+0) | length=142

AGGGTTTGGTGTGTCATCGTGTCCGTTTCTCCACTCACTACCATGTTGCCACAGCAAAAC

GATTCTGGCTGCAAATGTCAAACGATCACCAAAAAAACATATATCAGAGGTGGCGACGGC

GTTACGCAAATTTTCTTTGGGG

>Tb927.6.1260 | Trypanosoma brucei TREU927 | proteasome beta-1 subunit, putative (PSB1) | genomic | Tb927_06_v5.1 reverse | (geneCodeEnd+0 to geneEnd+0) | length=142

GTCACAACAAGGGTGCATATTGGTTATCACGCTCCGTGGTGACAGCTCTTTCTCTCGTTT

TACTCAAACCCCTAGTAATAAAGAAATGTGCGTGTGTGTATCGCCGTCTGTTGATGAGAA

AAGGGAGGAAGCTACGCTTTTG

>Tb927.11.11300 | Trypanosoma brucei TREU927 | hypothetical protein, conserved | genomic | Tb927_11_v5.1 forward | (geneCodeEnd+0 to geneEnd+0) | length=142

GTTTGGGTTCCTGTTTATCACCTGGATGTTCACACAGGATCAGTTAGGTGACGTATCCTT

CCTCCCAATACAGGCTAAGAAAAGAAGTCTGAAGTCCGGTGAAACTGACGGTGGAAATTT

CTTATTAACGCTTATTCGCGTG

>Tb927.11.11820 | Trypanosoma brucei TREU927 | 40S ribosomal protein S17, putative | genomic | Tb927_11_v5.1 forward | (geneCodeEnd+0 to geneEnd+0) | length=144

AAGCACTAGTGAATAGCCACTAGCTCAACCCGTGGGTTGCTCATTTTGACTTTTCCATTT

CAAAAAAGATATCATTTGTGAGGTTCTGTGTTTTCGTTTCCTTTCACAACAGTGCTCGGT

GGTTTAGCATGCCCTGTATTGCTG

>Tb927.3.1120 | Trypanosoma brucei TREU927 | GTP-binding nuclear protein rtb2, putative (rtb2) | genomic | Tb927_03_v5.1 forward | (geneCodeEnd+0 to geneEnd+0) | length=144

GAGACGGCACACCTCTCATCATTGAATTGAGTTAACTCGCGTGGAATAAATTTGTGTAAG

TCGATGTATGGAAGGAACGTGTTGGTGTAATGCAAGGTTGATGGATGAAGGAAACGGAAA

GCGATATGAAGTAAGCGCGCCATC

>Tb927.8.4970 | Trypanosoma brucei TREU927 | Paraflagellar rod protein 2 (PFR2) | genomic | Tb927_08_v5.1 forward | (geneCodeEnd+0 to geneEnd+0) | length=144

GCGCTGCGCTTAAATGTCTTTCATTATAATCAATGTATAACCTTTATGTAGTATTTCAAT

CTATGCCGCTGTGTACGTGCACTGCGGTGCCTATCCTTCGGCATTAGAGAGTCACTGTTT

GTGTAGATCGTAGCTGCATGTCTG

>Tb927.10.7510 | Trypanosoma brucei TREU927 | chromatin binding protein, putative | genomic | Tb927_10_v5.1 reverse | (geneCodeEnd+0 to geneEnd+0) | length=144

AAAGGAGAAAAATACACAGTTTCTGCCCCGATCTTCCTTGGCAGCCCTTGGTTTGCAGAT

GAGGAAACATGTAGAACATAACTTATCTATGCTCGTTGTTTGTGAGGCGGGATCGAGCCA

TCATCCCTTCGTATGTTGTTCTCC

>Tb927.10.5060 | Trypanosoma brucei TREU927 | Protein of unknown function (DUF3592), putative | genomic | Tb927_10_v5.1 forward | (geneCodeEnd+0 to geneEnd+0) | length=145

ACAGGAGGTAATTTGATATCAGGTTTCTCAGGGGGGAAACGTCTTTGCAATACAGTTGTG

CCAGGATGCACTTGAGTGGACATAGTTAGCTTGGTTCATACTACCCCGTGGCACCAACTT

GCTCATCTGTTTAGGTTCGCTTGTC

>Tb927.10.5480 | Trypanosoma brucei TREU927 | 60S ribosomal protein L24, putative | genomic | Tb927_10_v5.1 forward | (geneCodeEnd+0 to geneEnd+0) | length=145

GAGGCGCGCAGGATTCGTTTCTCACTACTCCAATTCCTTCAATGAGATTTGTCTTTGTTA

GTTTTTAGTTAGTGCTTTCCACGCCAACACCAATAGCGCTTTCTCATGTTCACTGTTGTG

AGGTGTGAACTGCTCAGCAGAGGCG

>Tb927.3.5560 | Trypanosoma brucei TREU927 | hypothetical protein, conserved | genomic | Tb927_03_v5.1 forward | (geneCodeEnd+0 to geneEnd+0) | length=146

ATGGTAAAGGTGAGATGAAGTTGTCAGGAAAATTTGAAAATTTACGGGTTTAAAGGCGTA

TGTGAGGGGGTTTTCGAATGTCAAATTCATTACTTCCGTTGTTTTCACATGAAAGGGGAG

GAGAAAATAATATGGCTATCACCATC

>Tb927.8.2200 | Trypanosoma brucei TREU927 | terbinafine resistance locus protein (yip1), putative | genomic | Tb927_08_v5.1 forward | (geneCodeEnd+0 to geneEnd+0) | length=146

GCTGGTGATTGTGTGCGTGTGTCTGTGTCTCTGTGTAACGAAGGTGATTGAATCACGTAG

GGGATCGAGACGAAAGAAGAAAACAAAGGGGATAGAAGCTGGGGAGAATGTGTAAGGATG

GAGGGGGAGAGGGGGGAAATGGTACC

>Tb927.7.3800 | Trypanosoma brucei TREU927 | hypothetical protein, conserved | genomic | Tb927_07_v5.1 reverse | (geneCodeEnd+0 to geneEnd+0) | length=146

GTTTTTTTCTAATTCCCCGCGCCTCGCCGGTTTTGTGTTTGTCATGATCTTTTTCATGAT

TCCACTATCTTCACCATATCGTTAATAATTTAGCGTCACAGTTTGGCTTTATCTCTTTGT

GTTTCTACGGGAAATGCTAGGATTGG

>Tb927.10.10400 | Trypanosoma brucei TREU927 | hypothetical protein, conserved | genomic | Tb927_10_v5.1 reverse | (geneCodeEnd+0 to geneEnd+0) | length=146

AGTCGTCGTCTCCCCCTTTCCTCTTTTGTTCGTTCGTTTATTTATCCAATAAACGTTTGA

GTTTTCAAACATCAAGTGTTGATGGAGAGATGGTTGGCAGCAACGGGGGAGGGACTAAAG

ATGCTCATCGGCGTACCCACCCCTTC

>Tb927.10.2300 | Trypanosoma brucei TREU927 | hypothetical protein, conserved | genomic | Tb927_10_v5.1 forward | (geneCodeEnd+0 to geneEnd+0) | length=146

AGAGCTTCATCCACTGGGATCTTTCTTTAAACCGTTGGGTCACTCGACCAGGTTACATAA

GTAAATGTGTTTGCGTATACGTATGTGCATAAAAGTCGACGGGTGTGGTCGCAATACCCA

ACGTATTTCTCCAAAAGGCACGAACG

>Tb927.11.16170 | Trypanosoma brucei TREU927 | hypothetical protein, conserved | genomic | Tb927_11_v5.1 reverse | (geneCodeEnd+0 to geneEnd+0) | length=146

GGATTGTTGAGCGTTTAAACTGCAGCCCGGAGAAAGTAACCCGGTGTGGATGTCGATGTA

GGCCGCCCTTCCAAGCGGTAGGAGGATAGATGTGTCGCGCAAGACTCAGCTTTGTTGGGG

ATTGAGCACTGAGGTTGCGAGTGCAG

>Tb927.3.2420 | Trypanosoma brucei TREU927 | Microsomal signal peptidase 12 kDa subunit (SPC12), putative | genomic | Tb927_03_v5.1 forward | (geneCodeEnd+0 to geneEnd+0) | length=147

AAGGAGGGACCTTGGCGTTATGCGAACCCACGACTTATGCATGGAAACGAGCTGTAAGTG

AGGTTGTAAAGATGATGCTTTCATTTTATCTAATGAGTGAACTTGCCCGTGCGCTAAGCT

TCCCTATTGTCATTATTATTATTATTA

>Tb927.3.5300 | Trypanosoma brucei TREU927 | hypothetical protein, conserved | genomic | Tb927_03_v5.1 forward | (geneCodeEnd+0 to geneEnd+0) | length=147

GTGCTATTGGTAGCATTTTCGTTGTTTCATTTCGTAACCTCCTTGAGGGGTGGTGTTGTA

CGCTCTAACTCGAGGGTGCTGGCCAGCGCTTTTGCATACCTTCTCTTGAGTGGAGGAAAC

AACCTCTTCAGCACTGCTGTGCTCAAC

>Tb927.10.8630 | Trypanosoma brucei TREU927 | Sucrase/ferredoxin-like, putative | genomic | Tb927_10_v5.1 forward | (geneCodeEnd+0 to geneEnd+0) | length=147

GAGGGGGTTCAGGGGAGACTAAACGAATGACGAAAGAATGTCTAATAAGTATTTTCTAGT

TTAAAGGTCAAGTTCTGCTGGACTGAGTGAAGTGAATGGAGTAAAATAAATGTTTCATTC

TTTTTTGGGTACTAGGAGGTCCTCTTT

>Tb927.10.160 | Trypanosoma brucei TREU927 | RNA polymerase II | genomic | Tb927_10_v5.1 forward | (geneCodeEnd+0 to geneEnd+0) | length=147

AAACACTAGCCGGTGCTGCGAATTGCAACTACGTCTTCCTATCAAGGAACAAAGAATTCT

AATAATAATATATTTTGCAAATGGATGCGCGCACACTGAACTAGCAGCTCTGCACTGACT

GTAGTGAGAAGGTGTTTTTTCTTGTTT

>Tb927.7.1900 | Trypanosoma brucei TREU927 | protein kinase, putative, cdc2-related related kinase, putative (CRK7) | genomic | Tb927_07_v5.1 forward | (geneCodeEnd+0 to geneEnd+0) | length=147

ATGTCTGTTGTTGAGGTGGTGGGGGGGGGGGTACCCAAACCAAATGGCAAGTTGCAGACT

GCTTCTGACTTTTGTGCGCTCACGCATTCATCACATGCAACCCCCTTGAATACCGTTTGC

TCCAATATGCGTTACGCTATGCAGGGC

>Tb927.6.1970 | Trypanosoma brucei TREU927 | nuclear cap binding protein, putative (CBC20) | genomic | Tb927_06_v5.1 reverse | (geneCodeEnd+0 to geneEnd+0) | length=147

ATGATGTGTGGAGTACCCGTTGGTGCAACTTTGACCTTTTTTCATTTGAATGCAGAGATG

CTTCTCTCCGTGGAAATTGAGAATCTGACGTAGTTTGCGAGAGCCCATTGTGAGGCTTGA

GGCAAGGCCCTTCCTTCGCTCACATGG

>Tb927.11.7920 | Trypanosoma brucei TREU927 | hypothetical protein, conserved | genomic | Tb927_11_v5.1 reverse | (geneCodeEnd+0 to geneEnd+0) | length=147

ATGGATTTATCCCATTTTGTTGCGGCTGAGTTCTGTAACAGAAGTTGTCTTACGGTCGGC

TGGGGTCGTTTGAAGTGCTTGTGCTTTACACTGTGCGATTGCAGTTAAGTTGAACGCAAA

AAGGATTTACATTTCCTTCTTCCGACC

>Tb927.2.5830 | Trypanosoma brucei TREU927 | hypothetical protein, conserved | genomic | Tb927_02_v5.1 forward | (geneCodeEnd+0 to geneEnd+0) | length=147

ATGGATTCATGGGGTTGTTTCACCCTTCATTTGTTCCCTCCTTTAACGCTCATCCTCGCA

GAGCCGCCCGTCCGTTTGCGTGCGTACCTCGCCCTTACGGATGTACGTGAGGCGGCACCG

TCGTCATATTGTTTTGTCTTCGTCTTG

>Tb927.10.16150 | Trypanosoma brucei TREU927 | ATP-dependent zinc metallopeptidase, putative | genomic | Tb927_10_v5.1 forward | (geneCodeEnd+0 to geneEnd+0) | length=147

AGGTGTCAAATGGTATATGTTGTTTGTTTGCGCAGTAGCGCACCTTAGTATTAGTGGAGG

ACTTGTTTATTTTGTTGGCACCGTTGCTATTATGGATGCCTTGCCTCTGCGACCTTATTC

CCCTACCTGTAACGCTGCGCGATACCG

>Tb927.8.4480 | Trypanosoma brucei TREU927 | hypothetical protein, conserved | genomic | Tb927_08_v5.1 reverse | (geneCodeEnd+0 to geneEnd+0) | length=148

ATATATTGGTAGGAAAGCTCGAGGGCCTCCCTAGTTTTGTGGGTGCAGCCACAGATGCCT

TCGCTGGGTCTGCGTTTGTATGTCTTTGCTTCTTGCACACAACGTTTCGGGTGCACGAGC

CGCGGTCTCTTCTACTTTGGTGCGAACG

>Tb927.9.11260 | Trypanosoma brucei TREU927 | nucleoside diphosphate kinase, putative | genomic | Tb927_09_v5.1 forward | (geneCodeEnd+0 to geneEnd+0) | length=148

GTTTGACATTTTCCCTCCCCTTGTTAGATGTTTTGTCGTTCAATGTTATCATACCAAACC

TTGTCAAGAGTCTGAAACTGTGCATGTAAGTGTCTTTTTGACGTGTTGATGTAGCACAAT

TGTGGCACGCAGAGCTTGCGTGGGGAAC

>Tb927.5.1340 | Trypanosoma brucei TREU927 | hypothetical protein, conserved | genomic | Tb927_05_v5.1 forward | (geneCodeEnd+0 to geneEnd+0) | length=148

ATTACGTCCCGGCGTGTTAACAGGTTCTGTTTTGGCGTTGTTTTTTTTTCGTTGCTGTGG

ATCCCTCCTCCCTCCAGCATATCGTACGTATGCACGTCAGTTTCTATGGTGAGAAGTGCT

TATGGCACTTTCCCTTTCGCTGTCGATC

>Tb927.10.14310 | Trypanosoma brucei TREU927 | hypothetical protein, conserved | genomic | Tb927_10_v5.1 reverse | (geneCodeEnd+0 to geneEnd+0) | length=148

ACGTGGTTTTGCACATACAGGTGCGTGTGTTGCATGCACCCACGTATGCATAAGTGTGCG

GACTTTTAATGGCTTTTGTGCGGGTCGTGTTGGGGTGGAGAATAGATGTTATGCTTGCGT

TTGTCAGTCCCAGTGCCTTACCTTCCTT

>Tb927.8.7010 | Trypanosoma brucei TREU927 | chaperone protein DNAj, putative | genomic | Tb927_08_v5.1 forward | (geneCodeEnd+0 to geneEnd+0) | length=149

GCTCCACCCCCTCTACAGCGAAGGCCCAGTGCATTGTAGCCAATAGCAGCTGCTAAGAGA

AGAGTTGCTATTAACCATCTCATTAGGGGGGCGCTGTAAGTACCGGCACGCATAATTTAC

CCTCCTTTGCCGCATTATTGCATACTTCT

>Tb927.4.3790 | Trypanosoma brucei TREU927 | ubiquitin carboxyl-terminal hydrolase, putative, cysteine peptidase, Clan CA, family C19, putative | genomic | Tb927_04_v5.1 reverse | (geneCodeEnd+0 to geneEnd+0) | length=149

GAGAAGGGAAGAAGAAGAGGAAGGAAAAAAAAAGAAAGAAAGCAGAAGTAAGGAAAGAAG

AAAGGAAACAAAGAAACACGAGAGCCGTGATGTCGCTACAGGGTAAACACGGGGGATAAC

GTAAAACTGTTAGCGCGCAAACAACTATT

>Tb927.6.1280 | Trypanosoma brucei TREU927 | translation initiation factor EIF-2b alpha subunit, putative, eIF-2B GDP-GTP exchange factor | genomic | Tb927_06_v5.1 reverse | (geneCodeEnd+0 to geneEnd+0) | length=149

GAGGGATGATTTCTTTCGTCGTGTGTGTGCATGGGGTATTTAATGAGAAGTAAGATTCAC

ATTAACGACGGTGTTCGCTGTGTGAGCGGAGTGAAGCCGTCTCCGTACTTTCATCGTGCT

TTTGTTATCATGATGTGCGTGCCTCCTTG

>Tb927.10.4240 | Trypanosoma brucei TREU927 | hypothetical protein, conserved | genomic | Tb927_10_v5.1 forward | (geneCodeEnd+0 to geneEnd+0) | length=150

AATGAGGAAAAAGCCCTCTATGTAGGAAATTAGGATCGGCGGCCGATGTTAGAAGGTAAT

GGGGTTACTATTAGGAACCACTTTTTTAGTTTTTATCGCACGCTAAAGGACGACAATCGT

TTTGTCTTATTGCTTACCCTCGTTTTGTTC

>Tb927.10.11270 | Trypanosoma brucei TREU927 | RNA-binding protein, putative (RBP23) | genomic | Tb927_10_v5.1 reverse | (geneCodeEnd+0 to geneEnd+0) | length=150

AAAGCTAGATAAATTAAGTAGTCGAATAAAAAGCGAAGCACGGTACCAGAGAAGTCGTCA

TGATCCTTTGACCTGTTTGCTTCCAACTCTCTAATATGAACTTGTGAAACGTCGGGTAAG

GGGAGCGGGTAATGTGTTGTGTGTTATGGT

>Tb927.2.4800 | Trypanosoma brucei TREU927 | hypothetical protein, conserved | genomic | Tb927_02_v5.1 reverse | (geneCodeEnd+0 to geneEnd+0) | length=150

GGGGAGTGGCCTCGCGCCTTATAGTGACTCACTGAGTACCGTCCCCATTACTGTTGGCAA

AACCTTTCTCTTTGTTATTTTCAAACCCCTCCCCCTCCGTTACCATTTCAGCTGGGAGTT

GGTACTTGTGCGTGTGTATCCGCTGCTACG

>Tb927.10.7530 | Trypanosoma brucei TREU927 | hypothetical protein | genomic | Tb927_10_v5.1 reverse | (geneCodeEnd+0 to geneEnd+0) | length=150

AATGTGCGACCTGCGGAGTTTTGAGGAATGGGGCGAGCCACTCTTTGACCCATGAGGGTT

TGGGGGTGAAGTTCGTGGTTTGGCGGCGTCGTGCAGCTGCGCGCAGGTTCACCTCGAGGG

CCAACTACTGATGACTGTAGCGATAATTTC

>Tb927.10.5000 | Trypanosoma brucei TREU927 | hypothetical protein, conserved | genomic | Tb927_10_v5.1 forward | (geneCodeEnd+0 to geneEnd+0) | length=151

ATTAGCGATTAGCGGTGCATTGATGTTCACTTGTTGCCGCGTTTCGATTTTTTTTTTGTG

TGGGTATGCAGGCATACATATAATTATTGCTTACGCTGTCAATGTACATCCATCTACGTG

GAGGTTCATCATCAGGGAGGGATTGCCCAGT

>Tb927.10.180 | Trypanosoma brucei TREU927 | ATP synthase F1 subunit gamma protein, putative | genomic | Tb927_10_v5.1 forward | (geneCodeEnd+0 to geneEnd+0) | length=151

GTATTTGTTCATAAATGCGGTAGGTATGCGTGCGACCGTGATGTTTGTGGTATAGGTAGG

CAACATGTTTTTTTTGTGGTTCGTAGGTGTAAGGCTCTTTCTATCACTTCTATTGGTTTT

TGGTGCAGATGTGCACTCCCTTTCCCTACTG

>Tb927.5.4390 | Trypanosoma brucei TREU927 | hypothetical protein, conserved | genomic | Tb927_05_v5.1 reverse | (geneCodeEnd+0 to geneEnd+0) | length=151

GAATATACATATCTGCATTGGAGAATAACAAGGGCGTTAGCGTATGGGGCGTAATTTATG

CCTTGTAGCGCCCTATGTTCATTTGGTGGGGATCGCTTTATGTGCCTGAATAAAGATGAT

GCATCCAGAAGAAGTGTGAGGGCGGCAAAAC

>Tb927.10.8080 | Trypanosoma brucei TREU927 | Uncharacterized conserved protein (DUF2042), putative | genomic | Tb927_10_v5.1 reverse | (geneCodeEnd+0 to geneEnd+0) | length=151

GGGTCGATTCCTTTCCTCCCCTAAGGGTTTTCACATTCCGGTGCCTCTTAGTGCTCTCAC

GGAAGGAGGAGGATGTTTCTTACGGTGCGGGTACAGATCTGACTTCTTGGTGAGAGCGCT

GTGCACTAGCTGAGTTGACGACTACGATCAT

>Tb927.8.3720 | Trypanosoma brucei TREU927 | hypothetical protein, conserved | genomic | Tb927_08_v5.1 reverse | (geneCodeEnd+0 to geneEnd+0) | length=151

ATCGGCCGAATGCTATATAAGGGCGGATAATTTCCGTTTTGCTTTATCCAAACAGCGTTT

TATAACTTTCTAAGTTTCTTCTTACAATTACAATCGTTTCCTTACCGCCTTCGTGGTTTT

TTCCCCCCTCCTTGAGAGAACTCTGAAGAAG

>Tb927.9.1470 | Trypanosoma brucei TREU927 | hypothetical protein, conserved | genomic | Tb927_09_v5.1 reverse | (geneCodeEnd+0 to geneEnd+0) | length=152

AACTGAATAATTGCAGTAATCTATAAAGCACCGGTGTGCAGTGTGTAATAGGTGTAACGA

ATAGGTGCTGTCTTGCACAGAGGATTGAATAACCCTTTTGTTCCCTTATCGTACCGAATT

AAGTGCCTGAATACCTTTGTAACTTTGTTCAT

>Tb927.11.16110 | Trypanosoma brucei TREU927 | integrin alpha chain protein, putative | genomic | Tb927_11_v5.1 reverse | (geneCodeEnd+0 to geneEnd+0) | length=152

AAATCATTATTCGAAGAGCTGTGAGATGTGAAGGGACCGATTTTAGTATGGTATCGGTAC

CAAATACCTTTCCTCGTGGGCTATGTCTCTTCACACACGGACGCGAATAGGGCAGACCGC

CCTGCTTCATTTCGTGCCTCAGCCTGGTTGTG

>Tb927.8.2510 | Trypanosoma brucei TREU927 | hypothetical protein, conserved | genomic | Tb927_08_v5.1 forward | (geneCodeEnd+0 to geneEnd+0) | length=153

GTTGTTGATTCAATCTCCAGTCAGAATAGGAAGCTGGGCAATTCACCACTCGGAAAAAAA

GTACGACGAACATGAAGAGTCGGTGGCCAAAAAAAAAAAAGTAGTTGGGGTGCGGTGTAT

TGGTGGCTGAAACATGTATTTCAAAATATACTT

>Tb927.2.1790 | Trypanosoma brucei TREU927 | hypothetical protein, conserved | genomic | Tb927_02_v5.1 forward | (geneCodeEnd+0 to geneEnd+0) | length=153

ATGTGGTGGGTTGCACCGTTTGGAGTTAACGGGACTACGTGTGATAGATGCTGCACATGC

ATCACAATTTCCCTGTTGACGTTTGTCTCATCACTCTTTTTTTTTCCTACTAAAGCTGCT

GCTATGTTTACGGTCGTATAGTGTTGTTTTCCT

>Tb927.10.14020 | Trypanosoma brucei TREU927 | hypothetical protein, conserved | genomic | Tb927_10_v5.1 reverse | (geneCodeEnd+0 to geneEnd+0) | length=153

AAGCAATTTAGGTGCTTTAACCGCGGAAGCCCGTTTCTGCGAGGTCAATCACGTTCGTAC

GTGCGTGTGACCAGGGTGCACTGTGGAATGTATGTTGTATTGCGCCATTTCCTAAGTGGT

GGGATTACAGAACAAGGAAGTAAAACTCGTCGT

>Tb927.1.610 | Trypanosoma brucei TREU927 | hypothetical protein, conserved | genomic | Tb927_01_v5.1 reverse | (geneCodeEnd+0 to geneEnd+0) | length=153

ATATAACTAACACAGGAGACAACGCCAAAATGAAAACAAGAGCACTCACCGCCATAGAAA

GATATATGAGGAAGTTAAGATCAATCAAACAGAACATACATTTTGCACCAATTTCCCCTC

TTAAGGCTCTTTGCTACTGTTCGCGACAAAAAC

>Tb927.11.9610 | Trypanosoma brucei TREU927 | eukaryotic translation initiation factor 3 subunit 2, putative (eIF-3 beta) | genomic | Tb927_11_v5.1 reverse | (geneCodeEnd+0 to geneEnd+0) | length=155

AATGAAGAGAGAGCGGTTAAGTTGCATAGCCACCAACGGATCCGATAAAAGAGAAAAGAG

CCGAGACTCAAAAAAAAAAAAGAAATAAATGAATGAAAAACAAAATGACTAAGACCAGTT

TAACCATCCGTGGCGGTCCGTGTGCGTGTTTTGTG

>Tb927.11.13970 | Trypanosoma brucei TREU927 | Pab1p-dependent poly(A) ribonuclease subunit, putative | genomic | Tb927_11_v5.1 forward | (geneCodeEnd+0 to geneEnd+0) | length=155

ATCAATGCGTTCATACGTTCGGGTTGTGCGGTGGACCAAAAGGTGCATGCGCGACAACAT

GAGGTCCAGGTATGAGAAGGGAGTATTTTTGTTCCTTTTCTGCCCCTTCTGCTTTTCCTC

CTTCCCCCCTTCCTTTGTTACGTGGGGTTAACATT

>Tb927.2.5230 | Trypanosoma brucei TREU927 | protein kinase, putative | genomic | Tb927_02_v5.1 forward | (geneCodeEnd+0 to geneEnd+0) | length=155

AGAAAGTTTGCGCACTGGACGTGCGGGAAAGGAATTACATGTGCAAATGTTTAGGAATTT

GCTTTCACCGAGGCTCCACCTAAGGGGAGGAGTGATGACGTGGAGACAGTATCTGAGTAG

GGGCATGCACCTCTCTTGTGTATAGTGACTAACGG

>Tb927.10.9400 | Trypanosoma brucei TREU927 | branch point binding protein, putative | genomic | Tb927_10_v5.1 forward | (geneCodeEnd+0 to geneEnd+0) | length=155

GCTGGTGGGAGATGTGGCGACAGAATAAAACTGAAACATTTGCTGACACCATGTTTTCGG

TGTTGGGGCGGAGCAACGTGTCTATCCTGTGGATATTGACTCGCTTATTGTACAGCAGTA

GCAGACTCGTGCCACGCTGTCTAACTTCACCGGTG

>Tb927.4.3700 | Trypanosoma brucei TREU927 | hypothetical protein, conserved | genomic | Tb927_04_v5.1 reverse | (geneCodeEnd+0 to geneEnd+0) | length=155

AACACTTTTCGAGATGTGCGTGTACCATTTTGTTTGTACTGACTGTTCTTACCGAGTGTC

CTGGGTCTGGTCCCATGTATTTTGTATGGATTTTTCCCTCCCAGCGGTTCGATCGTACGC

CTACCTTTCCTATTTCTCCATTGATCTGTATTGTT

>Tb927.6.4570 | Trypanosoma brucei TREU927 | hypothetical protein, conserved | genomic | Tb927_06_v5.1 reverse | (geneCodeEnd+0 to geneEnd+0) | length=156

ATTTTTTTTTAAAAAGTCGAGACAAGTTCATATGTTATGGAGGTGTGGGAAAGTAAGTTT

GGTGAAGGAAAATAGCGGAAGTGATTGTGGAGGTGACCTATTGTAGTGAATCTGTATCCT

TAACAGGAGATAGAAGTTTTGTTCTTTAGAAAGTTG

>Tb927.9.9840 | Trypanosoma brucei TREU927 | lipoic acid containing carrier protein, putative (GCVH) | genomic | Tb927_09_v5.1 reverse | (geneCodeEnd+0 to geneEnd+0) | length=157

GGGGGTGGTAAAACATCTGTTTTTCCCTAAACGCATTCACGTTGAAGTCACAGACATACA

AGCATTTGCGCAGCGAAAACGAAAAAGTGAAACTGTTTAACTTCTTTATTCCCAGTCCCT

ATTGGTGCACAGGAAGGCTCGTCCTGGTAGGATACAG

>Tb927.9.13650 | Trypanosoma brucei TREU927 | ADP-ribosylation factor, putative | genomic | Tb927_09_v5.1 reverse | (geneCodeEnd+0 to geneEnd+0) | length=157

GCGGTGTGTGATAGATGATTTAGTTCTTGAAGTAGGTTATTGTTAATACCTCTTGCTATG

GGTTCCTTTATATCTTTTTTGCTTTCTTTTTCCCTTTTTCGTAATTTCATTTGTTGCTGA

TCGCGGGGCACAAATGCGTTTATTGAGGAGTATACGG

>Tb927.11.7150 | Trypanosoma brucei TREU927 | NGG1 interacting factor 3-like | genomic | Tb927_11_v5.1 reverse | (geneCodeEnd+0 to geneEnd+0) | length=157

ACGTTCCGCTTCCGCGTGTGTGGTAGATTTACTGTAGCCTACACTTGATCCTCTCCCCTC

TCCGCGGCATGAACTCCTGCACTTCGAATGTGGTAGTAAAGAATGCGAGGTTTGAAGCAG

TGAGCGCATATGTAGCGATAGCTACCTTACGTTCTCC

>Tb927.8.2000 | Trypanosoma brucei TREU927 | cyclophilin, putative (NCP1) | genomic | Tb927_08_v5.1 forward | (geneCodeEnd+0 to geneEnd+0) | length=157

AGCGGGAAAAACCGACGTATGGTGGTGCTGTATGAAGGGCATTATCTTGAAATGAACACG

CCACGGGGAGGGGGATATCAAGATCACGTTTAACACTTATTTGATTACTATTTCACCAGG

CAACCTTCTTGACCCCTCCTTTTCACCTTAACAGTCT

>Tb927.3.3540 | Trypanosoma brucei TREU927 | Nucleoporin (TbNup53b) | genomic | Tb927_03_v5.1 forward | (geneCodeEnd+0 to geneEnd+0) | length=157

ATACCTCCGTCGCAAGGGTATTCGAACGGTGCATGTGTGCAGGGTTGCTTTGATTCCGCC

GCCGGCGTCCCCCTGCTGTAGCCGGGGTCTGACATATGTTGTCTTCAGGGTGTTGACCGC

TTCGGTAGCCACCCGCCTGCAGTTTTTACTTCATTTT

>Tb927.10.320 | Trypanosoma brucei TREU927 | hypothetical protein, conserved | genomic | Tb927_10_v5.1 forward | (geneCodeEnd+0 to geneEnd+0) | length=158

GGCGCGTCACAAAATCCCCACGTGTTAGATATTTCTTCTTTTCGTTGCTTCCGCCTTCAC

ATGTGGTAAACGAGGCGAACCCAAGACAAAAATGGAAGTGTGTGGCGTCTCATACGTACA

CGCATAATTCACCTGTATTTCACATTTAACATTTGCTT

>Tb927.9.8850 | Trypanosoma brucei TREU927 | actin A | genomic | Tb927_09_v5.1 reverse | (geneCodeEnd+0 to geneEnd+0) | length=158

AAAAAAAAATTTAGTATACCATAGGACACTTTGGTTACCTACTTTTTGCTTATTTGTTAT

TCTGATTATCTTTGTGTAATTTTTTTTTGTTTGATATAGAGGTTCGTGGGTGCCAGTTTT

TCAGTTACTTCCTCATTTCCCTCCTTTTTTGATCCCTG

>Tb927.9.8950 | Trypanosoma brucei TREU927 | CAAX prenyl protease 1, putative, metallo- peptidase, Clan M- Family M48 (FACE1) | genomic | Tb927_09_v5.1 reverse | (geneCodeEnd+0 to geneEnd+0) | length=159

AATAGCTCGATCGGTGTTTCACCGGGACTTGGCTGTTGCGATTCACCCCTACGTAAAGAA

AAGAAAGAAAGCGGTGTGGTGGATTGGGGAGGGGGGAAAGGGGAACAAGAATACTGGTCA

TTATGACCACGTCATCAACACAATACGTGAGGTTTTTGT

>Tb927.11.12850 | Trypanosoma brucei TREU927 | oligopeptidase b, serine peptidase, clan SC, family S9A-like protein (OPB) | genomic | Tb927_11_v5.1 reverse | (geneCodeEnd+0 to geneEnd+0) | length=159

GGTAGGTAAGGAATAGCTGAAACTTGTGGAGCTAAGGTAGGATGTGTTTCACAGAAAATG

AGGTAGAAGGGTAATAGTCAAGGGAGGCGAAACGCGAAAGAGGGTGAAGGAGCTGGCGTG

TGTGTTTGTGTGGGGGCTAACAGCGACAAGACGCATGGC

>Tb927.5.2380 | Trypanosoma brucei TREU927 | hydrolase, alpha/beta fold family, putative | genomic | Tb927_05_v5.1 forward | (geneCodeEnd+0 to geneEnd+0) | length=159

ACGCACGGAATATGCAACTGGAGAAGTAGCTATTATTGACGGCATTGCATTGTTTTCTTT

TTGTTTTTATTATTAACTCAATGTCTTTCCAGGTGCTAACTACGGATTTCATTGGGTTCT

GCTGTCCGTTTTCCAAAATTAGGGACTGGTTGTCAAAGC

>Tb927.10.12330 | Trypanosoma brucei TREU927 | zinc finger protein family member, putative (ZC3H34) | genomic | Tb927_10_v5.1 reverse | (geneCodeEnd+0 to geneEnd+0) | length=160

ATGAAAAGCAATAGCTAGGTAATTAAGAGTAATTTAGGAACAGAGAATGATTAATCAAAA

ATATTTTCTTGTTCCCACTTTCTTTAACTTGGTCTCGACCGTCCTCTTGCATGGTGGGTG

TCCGGGGCTTGTTGGGGTTTATCGCTCCCACTGCTATATT

>Tb927.9.2910 | Trypanosoma brucei TREU927 | histone acetyltransferase subunit NuA4, putative | genomic | Tb927_09_v5.1 reverse | (geneCodeEnd+0 to geneEnd+0) | length=160

ATATGGTCGTGGCGATGAAAGGACATGTCTCGGCGTGTTAACCGTTTCGCGTTCCTGGGA

ACCAGTTTGATGCGCACGTTGATGGGCATTTCCACCTCTTAACTGGGTCAGGGTAATGTT

TCTGACCGAGCTGACTAGTTTTCTTTGTGCCTTTCCACCC

>Tb927.11.9040 | Trypanosoma brucei TREU927 | conserved protein | genomic | Tb927_11_v5.1 forward | (geneCodeEnd+0 to geneEnd+0) | length=160

ATTGGTTGTTTGGTCGTTTGGTTGGTGGCGTACATTTCTGTTTTATTAGGATTTTAATTT

TGTAGTCTTTCTTAAGGTTTGTGTCCCCTATGTCTGGGTTTCATTTTTTTTTTTTACCCG

ACGAACTGTGCTGATGTCTCATGCTTTTGGGAATTCTTTT

>Tb927.11.14250 | Trypanosoma brucei TREU927 | T-complex protein 1, epsilon subunit, putative (TCP-1-epsilon) | genomic | Tb927_11_v5.1 forward | (geneCodeEnd+0 to geneEnd+0) | length=160

GATGTGCTGTTGCAGGAGGTACGGGATGGAACGCTATGTTGGAGCGGGAGGGGGTCATGC

GCACGAACATATACACTTCGACTACTGTAAGGGCTGAAGGGAAATCAGGGGATCGTGTGG

TTAGACCGTAGTCGTAGGGCACTTAAATGAATGATTAAGT

>Tb927.9.9780 | Trypanosoma brucei TREU927 | peptidyl-prolyl cis-trans isomerase (cyclophilin- 40), putative, cyclophilin-40, putative | genomic | Tb927_09_v5.1 reverse | (geneCodeEnd+0 to geneEnd+0) | length=160

ACGGTGCCTCGTGTGGCACATTTTTCTTCGACACCCTCCCTCGTACCTTCCGTTCTTTGT

GTTTATTTCCCTATTATATTATTACACCGTTATTATTTTAATTATTGTCAATATTATTGC

ATTGATGCTGGTTCTCCGAGTGTTATTGCAGGTGTCAGTG

>Tb927.5.590 | Trypanosoma brucei TREU927 | protein phosphatase 1, regulatory subunit, putative | genomic | Tb927_05_v5.1 reverse | (geneCodeEnd+0 to geneEnd+0) | length=161

ATGTTTATGAATACCGTCGACATACTTGGATATGTATTTACGAGGTTTCTTCAGCACTGT

TTGAGGTGCATTCCCGCGACACCACATAGTGGAGATCAACGGAAGTCAGACATGCGTACT

AGTGACGCATACGGCGGCATACGCAGTGGTTGTTTCCATCT

>Tb927.11.14520 | Trypanosoma brucei TREU927 | Polypeptide deformylase 1 | genomic | Tb927_11_v5.1 forward | (geneCodeEnd+0 to geneEnd+0) | length=161

ACAACTTGTGGGGGTACGTCCTGAAGCCGGCTTGCTGTGCGTTTCTGTGTGTGTTTTTTG

TTTTCGCTTTCGAATGGAAGTGGTGATAATGACGATCGAAATATGTTGTTGTTTGTATGG

TTACATATGTTGTTACCGCACACTTTGAATCGCTTTTTGGC

>Tb927.3.830 | Trypanosoma brucei TREU927 | flap endonuclease-1 (FEN-1), putative | genomic | Tb927_03_v5.1 reverse | (geneCodeEnd+0 to geneEnd+0) | length=161

GGATCTGGGGTGTCACACAGAGGGCGCATTGTACGCCTAACTTTCCCTCTCTTTCGTGTT

TTCTCTGATGTTTTTGTGGGGATCGAGACAAATCTTTTTGTGTGGCTATTTCTTTTGCGA

CACTACGAATGGAGGAAGACGGGGTTATTTACAAAACGTTT

>Tb927.11.1940 | Trypanosoma brucei TREU927 | hypothetical protein, conserved | genomic | Tb927_11_v5.1 forward | (geneCodeEnd+0 to geneEnd+0) | length=161

AGTTACTTCTCAATTGTCTTGTTCCAAATTTAACTTTCGTGGTGTTGTTATTGTACATTT

CCCCTTTGTTATCGTGCTTCTTTTCGGATGTTGTCACGCTATCTTCCATTAATATTCCAT

GGACGGGGCACCTTAACATTAAGCATGGCTTTTTTCCCTTG

>Tb927.1.3310 | Trypanosoma brucei TREU927 | hypothetical protein, conserved | genomic | Tb927_01_v5.1 forward | (geneCodeEnd+0 to geneEnd+0) | length=161

AGTTGTTGCTGGCGGTTGTTTTGTTTTTGTTTTTGTTTTTGTTTTGGTGTGTGTGTTGGT

GTTGGTGTTGGTGGTGTTTCTGTTGGTGGATGAAAGAGGAGAGGAAGAGGAACGTGTATT

ATCTTGTTTTTGAGCCGGTGTTCTCTTGTCTAATGGTAAGT

>Tb927.11.14430 | Trypanosoma brucei TREU927 | proteasome regulatory non-ATP-ase subunit | genomic | Tb927_11_v5.1 forward | (geneCodeEnd+0 to geneEnd+0) | length=162

GTCTCCCGGTGTGTAAATGCGTGAGGTAAGAAGGGAGAGTACCGAGGATGGTGTTAGATA

GCAAACTTGGTAAAGTGGACGTACCAGTTCCACTCATTTTTGCGCACAAATGAGTGTGTG

ACACAAATTTTTGGTACCGTGCCCCTTTTTCCCGTTTCTATT

>Tb927.11.670 | Trypanosoma brucei TREU927 | epsinR | genomic | Tb927_11_v5.1 reverse | (geneCodeEnd+0 to geneEnd+0) | length=162

GAGCACGCCAAGACCTTCAATTTTGCTTTTCTTCTCCCCCCCTTTCACTGCTTGTTTAGC

CTAGTACATGCTCCTAATGCTTGATTCGTGTGGTAAAAGAAAAACCATGTTCATGCTTAC

CTTTAAGTCCAGCCATAAAGTACGAAATGGCTTACTGTCTGC

>Tb927.9.10200 | Trypanosoma brucei TREU927 | hypothetical protein, conserved | genomic | Tb927_09_v5.1 reverse | (geneCodeEnd+0 to geneEnd+0) | length=162

AAAAGTGGTGTCGCGCGAGTGAGTATTGACTGATGCCATCACGTTAATGTTGCGTTGCCA

GTGACAACACCCGACTTTTTTGCCTCTTCCCTCCTCCTCCTCTTCCTCACCTTTCGTTGG

TACAGCATAAACGGTGCACCGAAAGCAAATCCAATCCTTGTG

>Tb927.5.4440 | Trypanosoma brucei TREU927 | dynein light chain, putative | genomic | Tb927_05_v5.1 reverse | (geneCodeEnd+0 to geneEnd+0) | length=162

ACCTTCGTCGTGCGGTTATTTTGGTGGCGGTGCAACCCTGGCACCTGTTCAGGTACGTTT

CCTCTCATTTTCGTTGTGGTTACTAGAATGTATAGAGATGATGAAAGGGTGCACAATGAC

TCTTTATGCACCTTTTGTGTAAATATACATATTCGTATATAT

>Tb927.7.890 | Trypanosoma brucei TREU927 | electron transfer protein, putative | genomic | Tb927_07_v5.1 reverse | (geneCodeEnd+0 to geneEnd+0) | length=163

AAGGTGGCTAGGAAGGCAGTTTGCACCACACACATGCTTCAATTTTTTTTCTTACTGGTT

GTATTGTTACCTTTTTTCCGCACCCGCCGTAGCGTGTTCATCGTTCTGTTTCCTTCCCGT

GGTGATTGCTATTCCCGCATTAACGTCTTCTGTCTTTTCCATC

>Tb927.9.1440 | Trypanosoma brucei TREU927 | Paraquat-inducible protein A, putative | genomic | Tb927_09_v5.1 reverse | (geneCodeEnd+0 to geneEnd+0) | length=163

ATGTCTTTCTTCGTTGCGCGCTTTAGTTTCGGGGACGGGGAATGTAGCATACATGTACAA

CTGGTATCTGTGGGGTAAGTGGTTGAGCAAGTTTCAACCTAAACCTCAAATGATTATTAC

CGCCGCGCACCCTGTGGCTAAAATTTTATGCTTTTGTTTTTGT

>Tb927.11.7100 | Trypanosoma brucei TREU927 | cytoplasmic translation machinery associated protein, putative | genomic | Tb927_11_v5.1 reverse | (geneCodeEnd+0 to geneEnd+0) | length=163

ATCAGCTGGGTGAGGTATTGGTGGTCATTTGGTCGTAGTTGCGAGGCGAAGGGATGTCCG

CTGCGCCCCTTTGACTCCCTCTGGATTCCACCTACTCTAGCGGGATAAGATAGTGAGCCT

CCTTCACGTGAGTAAAGGTAAAAGAATAATACTTAGTGTGTGT

>Tb927.5.2830 | Trypanosoma brucei TREU927 | hypothetical protein, conserved | genomic | Tb927_05_v5.1 reverse | (geneCodeEnd+0 to geneEnd+0) | length=163

GAACTCGGTCACCACTAAAAGTCACCTGAACATGCATATATTGATACCTATTTATTCCTG

CATCGCCGTTGAGTGCAGGTTCAAACTTAGTTGCGAAGAGGTGTATATTCGTGTGTGTGT

GCGCGGCCAAACGAGGCCGGGAATTCGTTGTTAACGGATGTGG

>Tb927.9.10160 | Trypanosoma brucei TREU927 | hypothetical protein, conserved | genomic | Tb927_09_v5.1 reverse | (geneCodeEnd+0 to geneEnd+0) | length=163

AGCGTATGTGAATCGAGGTGCTATATTATGGATGGATGAATGGATGGGGAAAGTGATGTT

AGTAACCGTAGGCATGAGCAAAAGTCGCATGGCTTTAGCGTGAATCATGTGACGTCATTG

TGTAGCGCCTTTGTACCAAAGTCTGCTGTTTATAAACTCCTCT

>Tb927.5.2590 | Trypanosoma brucei TREU927 | Macro domain containing protein, putative | genomic | Tb927_05_v5.1 forward | (geneCodeEnd+0 to geneEnd+0) | length=163

ATGCTTGCTTCCAACATGCAGGGAAGCAACTTACGGGCGTTCTTCTTGCTTATTCGCATG

CATTTTGTAGGTGATCCCTTCAAAGAGGTTTTAACAAGGAAAACGATGTAGGATTGTCCC

CTTTGTTTCCTCTTCTTTTTTGAAATTATGCATAGCATGTGTG

>Tb927.2.6070 | Trypanosoma brucei TREU927 | mitochondrial RNA binding complex 1 subunit (MRB6070) | genomic | Tb927_02_v5.1 forward | (geneCodeEnd+0 to geneEnd+0) | length=164

GCATGCCGCGCGTCGAATACCGTGCACATCCCTCGAGGCGTCTATTTTTCTTTCTCTTTT

TCTTCTTTACTTTACTTCTTCTTTAATTTTTGTCGTTTTGTTATTGTATGGGACTGAATA

TTGTTTTCCTCCTCCTCCTACTCCACTTTCTTGAGGAATTAACG

>Tb927.6.1090 | Trypanosoma brucei TREU927 | proteasome regulatory ATPase subunit 3 (RPT3) | genomic | Tb927_06_v5.1 reverse | (geneCodeEnd+0 to geneEnd+0) | length=164

GTTGGTGTGTATTCTGAAGTGAAGGTAAGAAAAAAAGAGATGGAGAAGGGAAGGACAAGC

AATATGTGGTTATTTTATATGCATCCTTGTGCGGGCTCTCAAAAATGATACTGGTTCTGG

AAGGAACAGGGGAGAGAAGCGAGGAGGAGGGGAAGAAAGTGGAG

>Tb927.11.10440 | Trypanosoma brucei TREU927 | hypothetical protein, conserved | genomic | Tb927_11_v5.1 forward | (geneCodeEnd+0 to geneEnd+0) | length=165

AGTGATGTACCAATGCTATGCATGGTATTTACAGTCACTTAGCTTTGGGGTGCCGGTGGC

TCGTGTAATCAGCACCTACTTGTAGCACTAATTAGTAATAACTTGCGCCCCCCCCCCTTG

TACACCTGGATAAGCTTTTACAAGGGTTTTCAGCCTTAAAATTTG

>Tb927.7.4610 | Trypanosoma brucei TREU927 | hypothetical protein, conserved | genomic | Tb927_07_v5.1 reverse | (geneCodeEnd+0 to geneEnd+0) | length=165

AATATTTCGGCAACCTTCTAGGTATGTCAGTTACTTACCAGAGCACCATAACTTTCATGT

TACATGTACCGGTGTATTCTCTGCGGGAACTTTTTACTTTTTATTATCCTATGACAGGGT

TCTTGCTCCAGCAATGAAGGAGCACCATTTCTCGTTCTTCCTTGC

>Tb927.7.3900 | Trypanosoma brucei TREU927 | vacuolar transporter chaperone, putative | genomic | Tb927_07_v5.1 forward | (geneCodeEnd+0 to geneEnd+0) | length=166

AGTGATGCTAATATTAAAAAGAAAGTGTAGTAGGCTATTGGAAGGGATCAGTTTTAAACT

TCTCAGATGATATCAGTAACATCAGTTGTTGGATGAGCCGAGTAGGACCGTTGGGAAATA

GTAGGTCATTGTTTTAACCTGGTAGGAAGATCCTTAATCTAATTCC

>Tb927.2.3960 | Trypanosoma brucei TREU927 | chaperone protein DNAj, putative | genomic | Tb927_02_v5.1 reverse | (geneCodeEnd+0 to geneEnd+0) | length=166

ACGCAACACGATGCCTTGTTTTTTTTGTTTTTCTTTCAAACTCTACCGTTGTTGAGATGT

GTGGGTGGTTGCTGTTTCTGAGTTCATGGATGTGGTGTACGGCTTGTCTTTCTCACCACC

CCTTACCTTTAGTTTCATCCTTCTATGGTTGTGTAAGAGTTTATTT

>Tb927.9.12300 | Trypanosoma brucei TREU927 | replication factor C, subunit 3, putative | genomic | Tb927_09_v5.1 forward | (geneCodeEnd+0 to geneEnd+0) | length=166

AGTTTTTGTAGCTATAAAGTTAAGCCGGGGTTATGAATTGTACCGCTGTACTTCGTGGAG

AGCCACATGATGGCAGAAAAAGAAGTAAACGGGGTATGGCTGCGCGGAAGAATAACTTTC

TTCACCAAATTGTGTCCCTTGCCTTTTTTGCCTCATGGTATATTTT

>Tb927.9.2160 | Trypanosoma brucei TREU927 | hypothetical protein, conserved | genomic | Tb927_09_v5.1 reverse | (geneCodeEnd+0 to geneEnd+0) | length=166

ATAAACATTTTTTGGGTAAAAGGAAGAAAAAAAAATAAGAGGTGAAATAAGTGGAACGGT

GGCGGGTGGCGGATTTGTTGAAGGGGAAAAAGGAAGTGGACCGCAAAAAAGGTGTGCACG

GGACGAGGCATGGCGGAGAGTTGAATGGGTGGAAGAGGGGAGTAAG

>Tb927.7.1430 | Trypanosoma brucei TREU927 | leucine-rich repeat protein (LRRP), putative | genomic | Tb927_07_v5.1 forward | (geneCodeEnd+0 to geneEnd+0) | length=166

ATATGATGTACCAACACCGTTCGCGTAACGTGGAGTTACATTGAGGGGAGCGTTCACCCT

GTTGTCTCTTTCAAATGTATAGTTTAGACCAGTGAGCCTGACACAAAACAGAATTTCAGA

TCTTTTGTTAGAAGGTACACCGCGGTGGAATATTCCTTTCGTCAGT

>Tb927.7.2340 | Trypanosoma brucei TREU927 | 40S ribosomal protein S15, putative | genomic | Tb927_07_v5.1 reverse | (geneCodeEnd+0 to geneEnd+0) | length=167

GTGAAGCATGGAGAGTTGAATGGGATGCAATCCATGACTTGTTTGGTCTTGTTTCACGCC

TGCCGATGCATCATTTTGTGGTATTGTGTTGCCTTCCCTTCGATCAAGAACACTAAGTTT

AGTTATGTTCTTTCTTTCTTTTTTTTTATTGCTTGTGAAAGCACTCC

>Tb927.10.9430 | Trypanosoma brucei TREU927 | phosphoribosylpyrophosphate synthetase, putative (PRS) | genomic | Tb927_10_v5.1 forward | (geneCodeEnd+0 to geneEnd+0) | length=167

AGGAAAAAAAAGGGCATCTCGGGATGCTGTGTGCGTTAAAGCCCCTTTCTCCTCGCACCG

AGACCGCGACCGACAACGATCAACGAGAGCAAAAGGGAAGGCAGGATTAAGATGGCGATC

GAAAGAAGGGAAAGGAGCGGGTCGCGATGATTTAATATATTAATATT

>Tb927.10.8650 | Trypanosoma brucei TREU927 | hypothetical protein, conserved | genomic | Tb927_10_v5.1 forward | (geneCodeEnd+0 to geneEnd+0) | length=167

ATTAGTGTGTAAGCAATTGGGGTTGTTCCAACGCGGATGCAAACAAGAGCGTGCACAACA

GAATGAATCAGGAGGCATATGTGGCTTTTTGGTTGAAGGGGAATCGTGAATGTACGTTTT

TTGTGATCGTGTATTGAATGCTAATGCGAACCTCGTCCAGTTTCGGT

>Tb927.7.3780 | Trypanosoma brucei TREU927 | poly(A) polymerase, putative | genomic | Tb927_07_v5.1 reverse | (geneCodeEnd+0 to geneEnd+0) | length=167

AAAGAGTTGGGCTACATTTTCTCTTTTTTATAATCGGCGAGTGTGTTTTCGTCGTAGTCC

AATATCTTTATCCTCCGTGCCGCTGTTTTTCTTTGTCAGTTCTTTTTGGCGGCAATTTGA

ATGCCAGGCATTTACCTTCTTGCACGCGTGCGTGTGAGACTGCACCG

>Tb927.6.3770 | Trypanosoma brucei TREU927 | hypothetical protein, conserved | genomic | Tb927_06_v5.1 reverse | (geneCodeEnd+0 to geneEnd+0) | length=169

ATAAGTAGCGAAGTATGTTGCTGGAAACTCTCCATTGGGGTGGTGTGAAGAGGCTCCTGG

CTTAAGAATAGTATGCCTGCGGTTGTGTTGAATGTCCGAGCCGAATGAGGACTATGTGAG

AGTATCATGTTCGTGCGTGTGTGCGAGTGCGTTTTCCGTTTCTTTCCTC

>Tb927.3.3260 | Trypanosoma brucei TREU927 | hypothetical protein, conserved | genomic | Tb927_03_v5.1 reverse | (geneCodeEnd+0 to geneEnd+0) | length=169

ACATGGCAGCTTGGCTGCTTTTTTTGTGTAGAACATGCATATTTCTGCCGAACATGCGCA

TGAGAGCTGATACGGGGCCTCACCTCGAGTTTGGTTACCCCGCTCCACTGCTGGTTTGCG

AACCATGCCGGTTAAAATGGGACCGTATGCCCACTGAACCTACTTACCC

>Tb927.10.3160 | Trypanosoma brucei TREU927 | hypothetical protein, conserved | genomic | Tb927_10_v5.1 forward | (geneCodeEnd+0 to geneEnd+0) | length=169

GAGTGGAGGGAGCAAGGCATGATTATCCTTTCCTTTGTTTGCTGTTTTCACTGGCCGTGC

CGGCGGGGGGTTGTGTGCCGTTTAGTATGTATATCAGCTGTGCTTCCCTTATGGACTGGC

CGCGGCGCGCTGCCACACGTGGGGTGACCCAGCAATCAAAGTCGGCAGC

>Tb927.3.5580 | Trypanosoma brucei TREU927 | tryptophanyl-tRNA synthetase | genomic | Tb927_03_v5.1 forward | (geneCodeEnd+0 to geneEnd+0) | length=169

AAGTTTGTGAAGGGTTGGATGGAATTAACTGGTTGTTGGTGTATTTGTTCGTCCTTTCTG

AGAATCCGACTATTGTGATTTCTGCAACCATTCTCTACCTGCACTAAATCTCTCGATAGT

ATGCGCCTTGCCGCCACCGGAGGTTGTTGGTGAGTAACGTGGCGTTTCT

>Tb927.9.12080 | Trypanosoma brucei TREU927 | hypothetical protein, conserved | genomic | Tb927_09_v5.1 forward | (geneCodeEnd+0 to geneEnd+0) | length=169

GTAATCGATTATTCTGTGTGAGTTTTACGCCGCGTGTGCTATGTACTACTATTTTGTTCT

TCTGTAGGTGATGTGATTTTGCATCCTTTTGTTTGCTTGCTCTTCTACGCCCAATTCCTG

CTTTCCTTATTCCACTCTTATATATGTGAGTGACGTTGCGGAATGCGCT

>Tb927.11.3500 | Trypanosoma brucei TREU927 | Dpy-30 motif containing protein, putative | genomic | Tb927_11_v5.1 forward | (geneCodeEnd+0 to geneEnd+0) | length=169

AGTGACCTGTTGTCTTTTCGTTTCTTACTTCGTCGGGTTGGGTTGTGAAGAGGGCCACAC

AGAGACTTAGAGTGCATTGGAACTCGTACAGCGTAGCCGCTATTGTCTATAACACCCCTC

CCTCCTATTTGACCCAGTAATAGGGGAAGTTTTGTTTATGTATGTTGTG

>Tb927.7.1380 | Trypanosoma brucei TREU927 | hypothetical protein, conserved | genomic | Tb927_07_v5.1 forward | (geneCodeEnd+0 to geneEnd+0) | length=170

AAGTATCTACTTCCAGTCGTTTACGTGTCAGAGGCCATATTCTCTTTTCTACATTTCCTT

TTATTTCCCTGATTGTCTTGAAGTCGCCATGCGGGGTCTATTTGTGACGTTTTGTTGGTC

ATCGCTATTTCTCATTAATTTATTTGTGTGCTCGAGCTCTCAGCATCCTG

>Tb927.11.5460 | Trypanosoma brucei TREU927 | hypothetical protein, conserved | genomic | Tb927_11_v5.1 forward | (geneCodeEnd+0 to geneEnd+0) | length=171

AAGTGGATGATGTGAGTGAAAGGAACCTAAAAGTCTGAGGTTCCCTCTTCTTCTTGTGTG

CGACAAACCGTTACGGACGATATACTGAAGAGAGAAATGTGTGTAATATTTGGGATCTTC

CAATTTAAGTGAAATAAATGTCCAAGTGAGGACGGAAGTGTGATTCTGTTC

>Tb927.11.13660 | Trypanosoma brucei TREU927 | hypothetical protein, conserved | genomic | Tb927_11_v5.1 reverse | (geneCodeEnd+0 to geneEnd+0) | length=171

AATGTGCGGAGGCACTGGCAATGTGCAAAGTTGAAGAGTGAGTGGTGCCGTGGTTTCTTC

CTACTTCCGGCTTGCCATCGTCATGGGGTTGACGTTACAGCTGTATCTCCCGAAGGATCG

TGGGGCAGGTGGTTAAAACTGCGGAGTGAAGCTTCGCACTGAACGTCAGGT

>Tb927.9.10310 | Trypanosoma brucei TREU927 | mitochondrial carrier protein (MCP11) | genomic | Tb927_09_v5.1 reverse | (geneCodeEnd+0 to geneEnd+0) | length=172

ATTTTGTTTGAGACGCGCCTTCCAGGGCCCTCCTTTTTTCTTTACTCGCTTTTCTTCTTT

ATCTCTCAGCGGTCGACCTCTCTCCTCAATTTCTCGACCATGTGCTCACTTGGTTTCCTC

ACTTTCTTTTTTGTTTTGTTTTGAGGTCTCTCCCTGTACGGATGAAAAGTTG

>Tb927.11.13280 | Trypanosoma brucei TREU927 | mitochondrial RNA binding protein 2 (GBP25) | genomic | Tb927_11_v5.1 reverse | (geneCodeEnd+0 to geneEnd+0) | length=172

GGATAATCTTGTTGGAACGTGATGAAAAAAAGGGAGGATAAAGAGAGGAGGATGTCGGGC

GTGTCGGGATAACGGGTGTGAATGTCAGCGTCATGTGCATTTATGACAAGTAGGATAAGA

GGAGTGGGCACGAAAGAAGTTTATTGGTGGAACAGGGGAAGGGAATGTTGTC

>Tb927.10.9680 | Trypanosoma brucei TREU927 | SET domain containing protein, putative | genomic | Tb927_10_v5.1 forward | (geneCodeEnd+0 to geneEnd+0) | length=173

AGATTTTTTTCTTGTAGTCGCACAAGGGTTTCGGACGTACCGCTTGTAAATATATAGGGG

GGGGGTGTCCAGTTCTTTTTTTTTTGAGGGTTCGGCGATTAAAGCTCTCAACGATCCCCT

TCCTCCCATTTCGTCATTCGCTTTAATTATTTGGGGGCCGCTGGCTACTCTAT

>Tb927.10.9420 | Trypanosoma brucei TREU927 | mitochondrial chaperone BCS1, putative | genomic | Tb927_10_v5.1 forward | (geneCodeEnd+0 to geneEnd+0) | length=173

AGTTTGTGTAGTTTTTCAAATGCGTCAAGACGCCGCCGGTAGTGCAACACACTCCCTCCC

CCCAAAACGGTTTGATTTGCTCCTGTTTTGAAAAGCGGGAGAAACAGTCATTAGCGGCAC

TTCTGGCGCACTGCACAGTTGTTCAACCCATTGCGTGTGGGGTGACTGACCAC

>Tb927.9.7770 | Trypanosoma brucei TREU927 | spermidine synthase (SpSyn) | genomic | Tb927_09_v5.1 reverse | (geneCodeEnd+0 to geneEnd+0) | length=174

AGTTTTCTTTTTGCCATTTTATTTTTCGTGGAAGCTCTGCGGTAAACGAACTGTCAGTGT

TAAAAATATATATATTTTGTAGGGGATAACATATTATGTGTCAGAACATTCTGTTGCTTC

CATATTTTTTTTACTTCATGTTCCTTGAGTACGTGATTTTGTCTCCTTGCTCCC

>Tb927.11.15640 | Trypanosoma brucei TREU927 | mitochondrial RNA binding complex 1 subunit (MERS1) | genomic | Tb927_11_v5.1 reverse | (geneCodeEnd+0 to geneEnd+0) | length=174

ATTAAAACGGTTGTTGGGCGGTGAATGTTGTGAATGGGTGGCTACTAGCTCTCTACATCC

TCTCTGAGTTAATAAACCGGCGAGGGGGGGAAGAGAGGTTGGACGGGGGGAAAGGGCGCA

GGAGCGGAGGGTTCTGTTCCTGGAAGCACGATGCAAACCGGCGGAGCAGACGGC

>Tb927.7.6090 | Trypanosoma brucei TREU927 | hypothetical protein, conserved | genomic | Tb927_07_v5.1 reverse | (geneCodeEnd+0 to geneEnd+0) | length=174

GGCTTCGAAGGGGGTCTTAATACGGTAGCTTAAGAGGTAATAAGTGAGTACCTGTTGAAG

GGGAATAGGTGGTATAAGTAGGAGAAAGAGAGGAGAGGAATCTACAACAAAGACACCATC

CACTTACAAACTTTCTTGTTCCCTCCCTGTTTATACAGAGTAACTTGTTGATTT

>Tb927.8.2440 | Trypanosoma brucei TREU927 | hypothetical protein, conserved | genomic | Tb927_08_v5.1 forward | (geneCodeEnd+0 to geneEnd+0) | length=174

GCAGTCTCCTACCGCGGGGCGCGGGACCTCTTCATGCCCATGTACAGTAAGAAACAACGT

CTGTGACACCAATACCACCAGCCGGGGATATGTGTTTTTTAGGCAAGAAGCTTCTACTGG

CACAGCACACTCTTTCCTCACTAGCCGTTACGGTAACCACGGGACTGAAGTCGC

>Tb927.9.10430 | Trypanosoma brucei TREU927 | Zinc-binding domain containing protein, putative | genomic | Tb927_09_v5.1 reverse | (geneCodeEnd+0 to geneEnd+0) | length=175

ACAGATGAAGGTGTACCAGGAACAGAAGTACGCCAACAGCCGACGCTTCCTGCAGGACCC

CCTCTTGATCGTTGACCTCTGTTGTGCAAAATCAGGAACCAACCCACACGAAGTTGTATT

CATCATGCTGAATCCTTATTGAGATGCGTTTTGTCTCCGTGCAAATCTGTCTGTG

>Tb927.10.12010 | Trypanosoma brucei TREU927 | hypothetical protein, conserved | genomic | Tb927_10_v5.1 forward | (geneCodeEnd+0 to geneEnd+0) | length=175

AATACAACAGATAAATACGCATTGCGCATAATTTGCTTAAAAGAAATAGCGGTAACAGTA

AAACGTACTTCTCTTTCATTTTAAAAAATAATAATGGGAAAGTCGGGAAGGGTGCGGTGG

GGCGCAAAATTACAGTGAGATTTTCCTGTCATAATATTGATCTTGCATTTACTTC

>Tb927.9.10000 | Trypanosoma brucei TREU927 | hypothetical protein, conserved | genomic | Tb927_09_v5.1 reverse | (geneCodeEnd+0 to geneEnd+0) | length=175

ATAGGAAGTGGTTTTGTAGGTTCATCGATCTACCGTTCTCGACAAGCACCCTCAGCTTCC

TACATTTCCATCCAAAGCACCCTCGTTCGTTTAAAAGCAATAAGGTCTCGTGTTACTATG

GCGCGTTGTTGGCACGAAATCATCCGACAAGCAAAATATCACTATACCTCCCAAG

>Tb927.7.5760 | Trypanosoma brucei TREU927 | nuclear transport factor 2 protein, putative, mRNA transport regulator MTR2, putative, nuclear transport factor, nuclear transport factor 2 protein, putative (MTR2) | genomic | Tb927_07_v5.1 forward | (geneCodeEnd+0 to geneEnd+0) | length=175

AAGACTGCAACGTTCATCTATTTGGTGGTGTAAGTGGCAAATCGCGGCCATCTGGCTCCT

CAATTTCTTTTTGTGTCCATATTATAAAAGGAGGTTTCACTCAGCTCTCTTTTTTTTTCT

TTTGTTACGCCCCATTTTTTGACAAGTGGGATACAAAAATACTAAAGAATGATGC

>Tb927.5.2930 | Trypanosoma brucei TREU927 | hypothetical protein, conserved | genomic | Tb927_05_v5.1 forward | (geneCodeEnd+0 to geneEnd+0) | length=176

ATGCTGGTGACCCGGTAATGTTTTGAAGGAAATTTTGGTTGTGCAACGGCCGCCCGACAC

TTTTATGTTTTGGTAGGAAGCGGGTTCGTAGGATTAGTAGAAAGTGGCATTATGCGTTTT

GACGATGCTTTCTTCATGAGTGTTTTCTGAGGAATAATAACTGTATGCGGTCGGAT

>Tb927.7.4680 | Trypanosoma brucei TREU927 | hypothetical protein, conserved | genomic | Tb927_07_v5.1 forward | (geneCodeEnd+0 to geneEnd+0) | length=176

AGGACCCACCCAACACACCTGGAGATAGCAGTAAAGTGTGTTTTCCCTGCAGTGTCAGAC

GTGCGCCTTCCACCGCTCACGGAAAGCGTTATGTTCCCCACGTAAGGGAAGAGGCCGCGT

ACGCGTACGTGGCTCAGGTGCCGTCACCTCGTCGGCCATACGCACAAAGCACCCGC

>Tb927.11.1440 | Trypanosoma brucei TREU927 | hypothetical protein, conserved | genomic | Tb927_11_v5.1 forward | (geneCodeEnd+0 to geneEnd+0) | length=176

ACATTCCGGAACATGCTGAAGGAATTGTGGTGCTGTTTTGTTGCCGTTACAGATGTTTGT

TGAGCGGTGGCGGTGGGGGGAAAGGTGCTATACGAAATGATGCAGCAGCGACCCGGACAT

ACCATTGTAATTTTGTCTCCTCTCCTCTTTCCTCTGACATGTGCCTCCTACTTGTG

>Tb927.10.10160 | Trypanosoma brucei TREU927 | pentatricopeptide repeat domain containing protein, putative | genomic | Tb927_10_v5.1 forward | (geneCodeEnd+0 to geneEnd+0) | length=176

AAGAAAAGCTGTCGATGCGCAACGAAGCTCGTCGGAAATTCTCAATTTCACATAACGCGT

CTTTCCCAGCATACGCTCGGATGTTAGCGCCTCTGCTATTCGAGCAGAGACCACCCGAAG

TCTCTAGTAAAACTATTTTCGGTGGCCGTTAGTCATTCCCGTCACCATCCCCACGC

>Tb927.6.4990 | Trypanosoma brucei TREU927 | ATP synthase, epsilon chain, putative | genomic | Tb927_06_v5.1 forward | (geneCodeEnd+0 to geneEnd+0) | length=177

GTAAAAGGCTGCAAACAGGAGGAGTGGGACAAGAAAAAGAAATGAGGGACTGACCGTAGT

TTGCGAGCTTTAGTTTATTGCATGTCTCTTGTTGTAGCGTTTCAGTAGCGGAGGAATTGG

AGATAGCGGTATGGGGTAAAGTACCACTTATCACTATCGTGAATTGTTTATATCTCT

>Tb927.11.13990 | Trypanosoma brucei TREU927 | GDP-L-fucose synthetase, putative | genomic | Tb927_11_v5.1 forward | (geneCodeEnd+0 to geneEnd+0) | length=177

AAGGCCGGTAGGGAGGACGTTTACAGCGCCTCACGAAACGCTTATATAACATCGTACTTG

CACACTTGCTTCAGTGCGTTGGTTGATACACACAAATCATTTATGCTGCCTCCCTTTCCT

TATCGTCTTTTGGTGAGAAACAACCTACTTGTGTCTAAACTTTCCACGGTGCCTTTT

>Tb927.10.1090 | Trypanosoma brucei TREU927 | 40S ribosomal protein S23, putative (RPS23) | genomic | Tb927_10_v5.1 reverse | (geneCodeEnd+0 to geneEnd+0) | length=178

GGAAAGTGCATCAATGGAATAACCCTGCAGGAAACTGTTTGGACGGTTCACTGTTCAACC

ACGGTGGTGTAGTGAAGAAGTTCTTGATGTCCTCCAGGGGTTTCATTTTTTGTTTTGTTT

CCCTTGCGTAACTTTTTTTTTCGTTGATTTCGTCATTTAAGCTTGAATTTTCTTTTTT

>Tb927.3.3410 | Trypanosoma brucei TREU927 | aspartyl aminopeptidase, putative, metallo-peptidase, Clan MH, Family M20 | genomic | Tb927_03_v5.1 reverse | (geneCodeEnd+0 to geneEnd+0) | length=178

AGTGCCAACTCCAGTTAGCGAAGTAGATATTGGGGTTATTTATAAATAATATCAAGTTTC

GCTGAGGCCAATAAGCAGTTGATGTCAGGGTAGGGGAGAGAAGACGAAGCATAAAGGGAG

ACAGGAACAATCACAGTGTCATTAGCATGGGAGAAACATCGAATACTTCCACACAATT

>Tb927.11.6470 | Trypanosoma brucei TREU927 | hypothetical protein, conserved | genomic | Tb927_11_v5.1 reverse | (geneCodeEnd+0 to geneEnd+0) | length=179

AGAGTTACGCCTCGCTTAGTGCCGTATATTTTTTCCCCCTTTTTACTCCAAACTTACTAC

TGTGTAGGGGGATGTATAAAGGGGAGAGGTGCACCGGTGGTGAGGGCTCTAGTAGAGAAG

ATAAATTGGATGAAAACAAGCCATGCTCCTCCTTTTCCCATTTGTTCTCCCGTGCACTG

>Tb927.5.4170 | Trypanosoma brucei TREU927 | histone H4, putative | genomic | Tb927_05_v5.1 reverse | (geneCodeEnd+0 to geneEnd+0) | length=179

ATGCACAAGACGTGTGTTCGTTGAGACGGCGTTATTGCCGCTCCGCACTTAGATAGCAGA

TGGCCAGGAGGTTGATACACCGTAATGGTAACATCCCTCCTCTGCCTCACCAAAGATATA

TCTTTTTTCTTTCCATTTTTCTCACTTTACTTTATTTTAACTTCCCTTCTGTGCCATAC

>Tb927.8.2990 | Trypanosoma brucei TREU927 | 5-formyltetrahydrofolate cyclo-ligase family, putative | genomic | Tb927_08_v5.1 forward | (geneCodeEnd+0 to geneEnd+0) | length=179

AGGCATTGGAAAATGTTACACAAGGTTCGCGTTGTCAGGGTTGAATTTCCTACCTTCTTT

TGTGAGCCGGTAGGAAGTTGTGTTCAAGTGAGACACAGATTATCTTTGCCATGCATTCGC

GGTACCTAACCCTCCTTTGTCCCACACATTTGTTTCGTCGTTGAACAGTTTTCAAAAAG

>Tb927.11.16880 | Trypanosoma brucei TREU927 | leucine-rich repeat protein (LRRP), putative | genomic | Tb927_11_v5.1 reverse | (geneCodeEnd+0 to geneEnd+0) | length=180

ATTAGCTCTCATGTATTTGTTCACAGCACGTTTTCAAAGCACGCGTAATACATACGTAAA

TGGCTATGTACATGTGTCATTGCAATGTGGGGCGGGGACGCCGGATCTCTGCCCAGGTTT

CGGCGTGTACTCACGCTGTCGGAGCGCGCCACTACAATGTGGCATTGTATTTTCTTGAGG

>Tb927.11.10400 | Trypanosoma brucei TREU927 | hypothetical protein, conserved | genomic | Tb927_11_v5.1 forward | (geneCodeEnd+0 to geneEnd+0) | length=181

ATGACTCTTCGATTGCTTTTATTTGTACTCGGGTTTAGCGAAGTGTGTGTGTGTGCTTGT

GTATGTGTGTGTGTGTGTGTTTAGCTTTCCTTTAAGGGATGAGAAGGAGGAGTTGCCATT

GGTCTATTGCGTGCCATCTACAGGTTCTAACCACCTGCGCATACTTTGAGGAAGTGTCTG

T

>Tb927.6.2060 | Trypanosoma brucei TREU927 | histidyl-tRNA synthetase | genomic | Tb927_06_v5.1 reverse | (geneCodeEnd+0 to geneEnd+0) | length=181

GTGTCGGTGAACAATGGAGTATACGATTCGGCACTGATTAAGTATTTTCAATGTTCTATG

AGTGTTGCCACATAATGGAGTATCCAAGGGTTGTGGCTTTAATTTATATCAACCCTTGGT

GTTCTGTGGGGGGGGGGGGGGGATGGCACGTTGAAGCGGAGGACAACTCTTTGTTCGGTT

C

>Tb927.11.4130 | Trypanosoma brucei TREU927 | ubiquitin-like protein, putative | genomic | Tb927_11_v5.1 reverse | (geneCodeEnd+0 to geneEnd+0) | length=181

AAGCGAGTGCGGAAGCGCGCGGAGATGGGCCTTACAGAGCCACATATTATTAACACATGC

TTTCTGGGTGCTGGGAGTGATGCCCGGTTAATCGCGTTTCCTTCACCATAGCGCTTTCCG

GGGGAAAAGCGTGTGCACTCTAGGGAGGGTCAGAAATGGGTATCTTTTTTTTCACTTTTC

C

>Tb927.10.8880 | Trypanosoma brucei TREU927 | hypothetical protein | genomic | Tb927_10_v5.1 forward | (geneCodeEnd+0 to geneEnd+0) | length=181

ACCTTCACATAGTTACGTTCCCTGTGGTCGCGCTGTGACCGCATTTATTATTATAATTTT

GTTCGCTTTCACACACCTCTTTTATTTCTCCCCGCGTTTTTACTAACCGTGAGGTGGAGC

AAAGGCATGTATTCGGCGAGACATTGATTACTTAAACTCGAGAAGAGCATAAATTAAACT

T

>Tb927.7.5180 | Trypanosoma brucei TREU927 | 60S ribosomal protein L23a, putative | genomic | Tb927_07_v5.1 forward | (geneCodeEnd+0 to geneEnd+0) | length=182

GTGTGGTGGATCTGTAGGGGTTTCTTACTTCCTCAAGCAACTTTGTATGGTACTGTGTGG

ATACGATTCCTTCAGTATTTTGCACCGTATGGTAGGAGGGGATGAAAGAGAAACAGCCAC

GAACTGTCGTTTATGTTTTTATTTGGTTTTTTTTGAATTCTCATCTGAAAATAAAAATAA

AT

>Tb927.10.4980 | Trypanosoma brucei TREU927 | ubiquitin-like protein DSK2, putative (DSK2) | genomic | Tb927_10_v5.1 forward | (geneCodeEnd+0 to geneEnd+0) | length=182

AGCAGCAGAGGCCACTAGGTATATTTTGCGATTAGCTGAAACCGAGGCATTTCTTATGTT

AAGTCTCTGTATTTAGCTATGATCTCACTATTTCCCTCTTTTCTTCGTTGGGTTGTGTAC

CTAGCGATGTTTTACCTCGACAATTCTCGGTCGAGGGGGCTAAGGCGGTTTCTACCCCTA

TC

>Tb927.5.1840 | Trypanosoma brucei TREU927 | hypothetical protein, conserved | genomic | Tb927_05_v5.1 forward | (geneCodeEnd+0 to geneEnd+0) | length=182

ATTAGCTAAAGAGGACTCGATGCAAACCAAGACTTCCAATGGATAATACTTTTCCGTTCT

TTCTTTCTGTCCATTCAATTTTGTCATTATCTTTTCTTGTGCCTTTCCCTTTTGTGTCAG

TCTGTTTTCGTGTTGGTTTGCGTGCGTTTTCTCTTCTTCTTTCGTTTCGCCTCTATCTTG

TT

>Tb927.10.1430 | Trypanosoma brucei TREU927 | hypothetical protein, conserved | genomic | Tb927_10_v5.1 reverse | (geneCodeEnd+0 to geneEnd+0) | length=182

AATAAAGTTCAATGAATTTTCCTTCTTGTACCATCAGTACTGTCTGGTATTGTTATTGCT

AGTTTTTTTTACTTGTTTCTTGTTGCTTTATTTATACCGGGGCTAAGCGAGATCATGAAG

AATGGGCAAGAGAAAAACAGGAAAGAAGGGGTGTCGTTAAAGACGCTGTGGAAAAGGTAA

AC

>Tb927.10.8640 | Trypanosoma brucei TREU927 | Galactose oxidase, central domain containing protein, putative | genomic | Tb927_10_v5.1 forward | (geneCodeEnd+0 to geneEnd+0) | length=183

GCGCAGATGAACAGGTTTCGTGGAGCGCTGCTCTTGTTGTAGAGTTGTGCTGATGACAAT

AAAGTTTTTGTTTTTTTTTTCGAATGTAGCTGTGCGCACCCTCACGCTTCCCCTCAGGGA

ATTCCTCAGTGCTTTCCGCCACTGCTGTGGCCCTATGTACCATTCATGGCGAATAATGAT

AGT

>Tb927.7.2310 | Trypanosoma brucei TREU927 | DNA primase small subunit, putative | genomic | Tb927_07_v5.1 reverse | (geneCodeEnd+0 to geneEnd+0) | length=183

GACGTATTGCGGGCTGATAGAACACACATACGCATAGGTGAGGGTTCACAAAGTAGCACA

CCGGCTGCGGCTGAAAATGTCACCACCAAAAGGTGGAATGATATGGGATATTTTTACCAC

CAATCGTTTAGTGTGTGTTAGATCTTCAATTCGAGCGCTCACTGTTCGAAGTTTCCCCGT

TTT

>Tb927.10.13930 | Trypanosoma brucei TREU927 | phosphatidic acid phosphatase, putative | genomic | Tb927_10_v5.1 reverse | (geneCodeEnd+0 to geneEnd+0) | length=183

AGCAGTTCTCTCCGGTTCATGGTACAATTTTTTTTTCTAGCAACTCTGTTTTGCTTAGAA

AATTTCGTGGGGAGTCCCTGAAAAAGGTGTTGGGATGTCCTCCCACACTTTAAAAAGAAA

AAAAACATGTATTTCTATCTTTTTCTTGTGGAATGTGTGTCTTGTCTATGTTAAATGACT

GGC

>Tb927.11.5200 | Trypanosoma brucei TREU927 | hypothetical protein, conserved | genomic | Tb927_11_v5.1 forward | (geneCodeEnd+0 to geneEnd+0) | length=184

AGTAGTTGAAGTACATGTGCTAAATAACAAAACACCAGGCATTGTCGGACAATGTTTTTG

GTTGTTATTGTTGTCGATTCACGGACCACATCTGTCAGTAGGTGGGAAAATAGGTGGAGT

TACCGAGAGGGGTTGGGGGTAGAGGGACGATGCGGATGATGGAATCGTACCTTAGTGTCG

CAAT

>Tb927.6.3060 | Trypanosoma brucei TREU927 | hypothetical protein, conserved | genomic | Tb927_06_v5.1 forward | (geneCodeEnd+0 to geneEnd+0) | length=185

ATGCACTGCAAGGAGTTGGTATTCTTCCAGTTTTTGTCACTTCAGTTATTACACCTAATT

TTACTTCTCAGTAAGGTGTCCATTTTCTTTTAGATTTGAGTATTCATATATGTGGTGATT

TGCTTTTGTAGGCGAGTTTCTTTCCGTTGGCCCACTTTCCATTCGTATTTCGGTTTACTG

ATGCT

>Tb927.11.7480 | Trypanosoma brucei TREU927 | vacuolar type h+ ATPase subunit, putative | genomic | Tb927_11_v5.1 reverse | (geneCodeEnd+0 to geneEnd+0) | length=186

ATACCACATGGAAAGGAAAAAGGACAGGGGGCCTTTGGGGGCAAATGTAGGGGGGGAGAG

AAGGGAGGGAAAAAGTGACTGCTGGTTGTGCCTCGGTCGACTTGGAGAGCAACCCGCATG

GCATTGGTGCTAAGGAAGGTAGCGTTTTCCCCCAAGGGAGGATTTTGCTCAGAGTCGGTT

TACTTT

>Tb927.11.6070 | Trypanosoma brucei TREU927 | hypothetical protein, conserved | genomic | Tb927_11_v5.1 reverse | (geneCodeEnd+0 to geneEnd+0) | length=186

ACTCTGTTTCCGGGGATTTTTTTCTTTTACCCACAGCGTCTCGTGCACGCTTGGCTTTGT

GTAGACTATGACATTCCTCCTTCCAAGCTTGATTCAAATGTAAATAGCCGTCGGTGGGCA

GTGGTAGTGATTCCAGAACAATAGGGGTGCCGAAACAAAGCGCCTTCACCATGGTGCGAC

TCATTT

>Tb927.11.4020 | Trypanosoma brucei TREU927 | Complex1_LYR-like, putative | genomic | Tb927_11_v5.1 forward | (geneCodeEnd+0 to geneEnd+0) | length=186

AAGGGATTTATTCTGTGAGTCCTGAGATGTACACACGGTGCGACGTATTCGTATGGATAG

TTTAGGCTTTGTCCGGCAAGTGGCGTCCTTTTTTTTTTTTTTTAATGCTTATATTCTAGC

AGGTGGTCCGATGCTTCCAGTAGTGTTAATCATATCGCCATCAGTTTAGAAAATCGTTAT

GTTGGT

>Tb927.8.1860 | Trypanosoma brucei TREU927 | pitrilysin-like metalloprotease, metallo-peptidase, Clan ME, Family M16C | genomic | Tb927_08_v5.1 reverse | (geneCodeEnd+0 to geneEnd+0) | length=186

GCGTTGCCCGTGCACCCGCTTTTGTGCATTGACTTCTTGTTTTTCTACATTTATGTTGTC

GCCAAGATTTTCCTTGGACGTTTGAACGTTACGTTGATGGCAGTTGGCAGTCTTAACGTC

TTATGACCTGTAGTATTGGGCACTATTGGTGGTAGGGGGAGGTGCGTATTTAACGGATTG

ATAACT

>Tb927.3.2140 | Trypanosoma brucei TREU927 | transcription activator, putative | genomic | Tb927_03_v5.1 reverse | (geneCodeEnd+0 to geneEnd+0) | length=186

GAGGTGAGGTGTGTGTGTGGGGGGACGATATTCGATGCAGGGGATTTGGATTACATAAAA

TTTATAGTTTCCCCCTCTTTCTCCTCCCGACCAGTCAAGCTCGTTTTCGTAGACGGTGCC

ACCAGGTTTCGCTAGAAGACCAATGTGGTCGGTTCTTCCCCCCTTTGGTCGATTTTTTTT

TATTTC

>Tb927.9.1380 | Trypanosoma brucei TREU927 | hypothetical protein, conserved | genomic | Tb927_09_v5.1 reverse | (geneCodeEnd+0 to geneEnd+0) | length=186

AGGGTACTCAAATTTCATGAGAGCAAGAAATGTTTCTTAGTTTCGTTAGTGGATGGAGTT

AAAGGTGAATGATGTGCATTGTGAGGTGCTTGCTTGTGCGTACCGTATGGCGAAAAGAAA

AAAGACGTTGACTAACCGTAATCGTAATTATAACTATTGTAAGCAAATTATTGAACCCTG

TTCTGC

>Tb927.11.14690 | Trypanosoma brucei TREU927 | Microtubule-binding protein MIP-T3, putative | genomic | Tb927_11_v5.1 reverse | (geneCodeEnd+0 to geneEnd+0) | length=187

AACCCTTTTTGCGTATGCACAGCCTCTGCGCTATACATTCTTCCGTATCCCTTTCGGTTG

CATGTCTACAGTGCGAAGAATGTGCAGCACCGTGATTGCGGTTGCGCGTTAGAGCATACA

AATACTAATGGTCACGGGAAGTTGCATATGAGGAAGGGGAGAGGACACCGCAGTTTTGGC

AAATATT

>Tb927.9.4500 | Trypanosoma brucei TREU927 | heat shock protein, putative, HSP70-like protein | genomic | Tb927_09_v5.1 forward | (geneCodeEnd+0 to geneEnd+0) | length=187

AGCCCCCTACACACACACACTCACTCACTCTGTTCACTTTTCAGGGACCTTTCGTGTGCC

AGCCTCAGTGACGTACTTACGAAAGAAGGGAAGTGTCGGACTTCATGCTGATATTATTGG

TAGAGCTTGCTAGCATGGTACATGACAGGACGGCATATGTCCCTTGCGATCGTTAGGTGA

GGGGAGG

>Tb927.5.2640 | Trypanosoma brucei TREU927 | cytidylyltransferase, putative | genomic | Tb927_05_v5.1 forward | (geneCodeEnd+0 to geneEnd+0) | length=187

ATGGCGATTTTTGGGGTAAATATTTAATCGAAAATTATACGCTTCACTTTTGTAGGCACT

GGATGTAAGGACTCCCAGTTCGCGATGTCTCAAGGACTTCTCGGTTATCGTGCATAAATC

AGGTTGTGTCCTTGCGCGAGTTGCTTGAATCCTTTCAAACATCTTCGCGTGTCTATCGCG

ATATTAG

>Tb927.10.11250 | Trypanosoma brucei TREU927 | BRE1 E3 ubiquitin ligase, putative | genomic | Tb927_10_v5.1 reverse | (geneCodeEnd+0 to geneEnd+0) | length=187

ATTATCAGTTTGAGAGTTGTGGGTAACACATGGGCAACTATGGGTGCGTGATGTATATTG

AAGTGATCTCCAGAGGATCCAACCAGCTATGATGTCTCAGTATTTTTTCTTTTACATTTC

TCTGAGGCAAAAACATAATATTTCAACTCGTCTGGCACTGTGGCTGCAACCAAAAATAGT

GCACGGT

>Tb927.11.15680 | Trypanosoma brucei TREU927 | hypothetical protein, conserved | genomic | Tb927_11_v5.1 reverse | (geneCodeEnd+0 to geneEnd+0) | length=188

AGAATAATCCACTCTACTGCGCGCGTTCTTGCCTTGATGCAAAATGAAGATACTGGCGAG

AGAACTCTCACTTACCAACACGTGAAGAATGTCGTCTTCGGTGGTGATAAAGAGGGAGGA

GGGGATGTTTGGGCATATGATTGCGTTTAGTCCAGCCGCACACTGGGGTTTGGAAGGAGG

TTATGAGC

>Tb927.11.11170 | Trypanosoma brucei TREU927 | hypothetical protein, conserved | genomic | Tb927_11_v5.1 forward | (geneCodeEnd+0 to geneEnd+0) | length=188

AAAATTCCTCTAATTGACTCCCTTTCCGCGGAAGTACCCGGCGGAAAGGCGGGAAATTAG

CTAAGCTACAAAGGGTGTGTTTAGATCGTAAAGTGTTGACAGCTACCGACACTGCGGACC

GCACGCCCCCGCCTTTGTCTGAGAGGAAGGAAGCCCGTCTTTCCCTTTTTTGGAATTACA

AGCTGACG

>Tb927.11.1740 | Trypanosoma brucei TREU927 | intraflagellar transport protein 88 (IFT88) | genomic | Tb927_11_v5.1 reverse | (geneCodeEnd+0 to geneEnd+0) | length=188

GAAAGGATATGCTCGTAGAGGAACAAAAAGGGGTTGGAAACACATACGAACTGGTGCCTG

CGTTGGCCAAGGGGGGATTTATTGAAGTGTTTGCATATACGCCTGTACATATATATGTAA

ATATATATATATATATATATATAGACGACAATGATACGTGTGCTCGCTTTAGTGGAGGAG

GGCAAAGG

>Tb927.11.12040 | Trypanosoma brucei TREU927 | hypothetical protein, conserved | genomic | Tb927_11_v5.1 forward | (geneCodeEnd+0 to geneEnd+0) | length=189

GGCATTTTTAGCGAATACATGGAAATTTTTTGTCTTCAAAAGTTTTTTTGTTTTGTTTTT

TGTTTTCTTTGTCTTTTGTTGTTAGGGCGTACCGGCAATACGGTAGGGGGAATTAGTATT

TATGTGTAGACTGCTCGTTATCTGATTTAGGGATTTTTGTAAAACCAATAAAGTACCATA

TAAGTACTG

>Tb927.6.4070 | Trypanosoma brucei TREU927 | hypothetical protein, conserved | genomic | Tb927_06_v5.1 reverse | (geneCodeEnd+0 to geneEnd+0) | length=189

AAGTGAGTAGCGCATTTGATCTATCAAGCTATGAGAATACATTTGAAATTGTTCATAAGT

TCCCCCTGATTATTTGGTGATCGGTCTCCTAGCGTGCCGACAGAAGTTTAGCTGGAATGA

AGAGGGATGGGTGGATGGCGGCTGATTTTTTGAAAGATGGAGCATGTCCATTTCACTTTT

TTCTATAGG

>Tb927.10.13600 | Trypanosoma brucei TREU927 | hypothetical protein, conserved | genomic | Tb927_10_v5.1 reverse | (geneCodeEnd+0 to geneEnd+0) | length=190

GTATCAAATAATCGAAATGTAAGATGAAGACATTCTTCAACGTGAACAAACCTTATCGAC

GACATTACCCATCCCTCTTATATTCAGATGAATGCAGTGAATTACTTACATATGCGTTCG

GAATGCGACCGTGTGAACAACTGCTCCATAGAGGTGGCAGTGGCGTGGTCGTTTGTGCGC

TCGGCTGTTT

>Tb927.10.3710 | Trypanosoma brucei TREU927 | proteasome activator protein PA26 (pa26) | genomic | Tb927_10_v5.1 reverse | (geneCodeEnd+0 to geneEnd+0) | length=190

ACGTGATGCTGGAAATTGAAACTATGTGCTGCAGCAGTGCATTCGCTGTTTTGAGCAGTG

GCTGGCGTTTATTCGATTTTCTCTTTGTCAAGGGTATGATGCGAATTCTTGCAGTCATAT

ATGCGTTCACACATATATGACTATTGGGTCACCTTCGTCTACCCTCTAACACTAGTAGTT

TCATCTTTTC

>Tb927.10.12410 | Trypanosoma brucei TREU927 | hypothetical protein, conserved | genomic | Tb927_10_v5.1 reverse | (geneCodeEnd+0 to geneEnd+0) | length=190

ATAATCCCCTGTTCACCGCTTGGCTTTTCTCATTCTTTTCACTTTAGAGCGCCATAAGAA

TATGAAGGTCAGCTCCACACCGGTGATTGAACCTGCACGTTTGCGCACGGCGAAAGGAAA

GAATGAAAGACTGGATGCAAAGAGTCTTTTGTCGATACCTAGACAGCACATGTAAAATGG

ATTATGTTTT

>Tb927.7.3200 | Trypanosoma brucei TREU927 | hypothetical protein, conserved | genomic | Tb927_07_v5.1 forward | (geneCodeEnd+0 to geneEnd+0) | length=190

GAAGCGCATATACGTCCATGAAATCCCCTCGCGCAGTACTGTCAAAAAAAAAAGTGGGTG

GACAGATGACATCCACTTCACTTACATACTTAACCACGGTTGAACGTGATAAACATACCG

CAACAATTCGTGGGTTACACTTGTTTTACGTAATAACACATATCTGGTAATAGCTGGGTT

TTGTATCGCC

>Tb927.10.4850 | Trypanosoma brucei TREU927 | hypothetical protein, conserved | genomic | Tb927_10_v5.1 reverse | (geneCodeEnd+0 to geneEnd+0) | length=190

ATCATGTGCGCTTGACTTAGGTTTGTAGGGGGTGCGGATGTCGACGAGTGTGCGTACTTA

CGTGGCGGCGAATGAAGGCGAGAAAAAAGGAGACAAGGATCGCAGAAGGGGATACGGTTC

GACTTGAGTGCTGTCTTTCCCTGTTGCTTTACCTCCCTCTTTCTTATGCACCGTGTCATT

TTCAGTCGAT

>Tb927.7.3410 | Trypanosoma brucei TREU927 | centrin-4 (Centrin4) | genomic | Tb927_07_v5.1 reverse | (geneCodeEnd+0 to geneEnd+0) | length=190

GATGGGTCTATGCTGTTTTAGGTATGTATGCGTGTTGGAGCTGACAGAAGAGCTTCCATT

CGATCTCCGTGCGCATCCGCTGTTGACCTTACAGCTTCCACCCCACTTTTACTGTTTTAT

AAACTATCTTTACTTCCTCCCTTTTTCCTATTTCCATACTTTCTCCCTCTTTGTGTAATC

GAAGACGTTG

>Tb927.11.3240 | Trypanosoma brucei TREU927 | T-complex protein 1, zeta subunit, putative (TCP-1-zeta) | genomic | Tb927_11_v5.1 forward | (geneCodeEnd+0 to geneEnd+0) | length=191

AAACAGTCGATCTATATTAAATTACCAGAAAAGAAAACAACACTGAGGAAAACAGCCTTT

CAGCTTAATAGACAAAAGAAATGAAAAAGAAAATTAAGGCATAATTTTTTTAAAAGTGTG

ATTAACGCATAGGGGAAAATGAAAGTACTTTCGGCATAAGTTGGTTCGTGTTTCCCTGTG

TGGATGGAGAG

>Tb927.8.7040 | Trypanosoma brucei TREU927 | hypothetical protein, conserved | genomic | Tb927_08_v5.1 forward | (geneCodeEnd+0 to geneEnd+0) | length=191

ACAGAAAAATAGATGAAGAGAGGGGGGACACTACGGCTGCTTCCCCCTGAGTTGCTCGTT

ATATATATATATAGTTCTCCAGTACGTAGTAGGGGGGCACTAGCGATGTATGTAGGTCAC

CATTTTTTGTTTGTTTGTTTAAATCCCCCTAGCTACTTTTTGTGAATGTGTCCTCCTCAT

GGCATCTGGGC

>Tb927.10.11530 | Trypanosoma brucei TREU927 | hypothetical protein, conserved | genomic | Tb927_10_v5.1 forward | (geneCodeEnd+0 to geneEnd+0) | length=191

AAGTGGCTTGACCCCGGTGACGGTACTTGTAGGCAGGTGTATGAGGCATGGCGTCACTGG

GCAGACAAACGGCTCGAACGTGGTGCGTCAACAACCGCTCCCCTTCCTTTTCTTTTCCTT

ATTTCCCTTCGTCACTTCCATCGGGCTGCCGATGACTGCATGTGGCTATGCTGCTGCTAT

CGTGGCATCTG

>Tb927.11.14050 | Trypanosoma brucei TREU927 | hypothetical protein, conserved | genomic | Tb927_11_v5.1 forward | (geneCodeEnd+0 to geneEnd+0) | length=191

ATACCTAAGATACCGCCTCGTATTGGGTGGGGAAATTCTCTTCAGCCGACGGAAACGATT

CAGTGGGGTCGGAGTCCTGTCGACTTCCACGATGTGTGTGTGGTTCGAGTGTTGTTTGTG

CTGCGTGCCGTCGATATATCAGAGAAGAGGGAATAATGACATGTTCGTGGTTGCATGAGG

TATGGTGCCTG

>Tb927.4.1370 | Trypanosoma brucei TREU927 | hypothetical protein, conserved | genomic | Tb927_04_v5.1 reverse | (geneCodeEnd+0 to geneEnd+0) | length=192

GTTCTTCCTTGTCTGAAGATTGAAGAGAACAAATGCAGTGACTGACGGTGTTGTAGTCAG

TTGGTACTGTCACCTGTGAGTAGGCGAGGAGCCGTGACCGCTTTAGTGTTGGGGGAACTG

GGTTTAATATATTAACGGTGGTTTATTTTATCCTGCGTACCTGCCTGTCGTTACCTTTGG

AGCGGGTCTCTT

>Tb927.10.7500 | Trypanosoma brucei TREU927 | fibrillarin (NOP1) | genomic | Tb927_10_v5.1 reverse | (geneCodeEnd+0 to geneEnd+0) | length=192

ATAATGTTGTGAATAAGAACGGAATGATATAATGTGCAATGTTGATCGAAAGTTATGGAT

GAGGAAAGGAAAGAAAATGGAGGCTAAATGAAACGGCCTACGACACAACATTTCGAGAAA

AAATTAAGTGGCGAACAGGTAATAATAAAAGGAGGGAACTAACTTATTCACGATATTTTA

AGGTGATCGACT

>Tb927.9.9060 | Trypanosoma brucei TREU927 | Lsm12 protein, putative | genomic | Tb927_09_v5.1 reverse | (geneCodeEnd+0 to geneEnd+0) | length=194

ATACTGAAAGAATACCAAGAAAAGGAAATGAGCAAGGAAGAATAGACTATGAATGTCCCC

CTCCCACATTATCATTATCACGATTGCCTCGCTCATGGACCGCAACGTGTGAGCTTGACT

GAGTTCTTTCTCATATCTGTTGCTGGTGTCATTTCCCGGTTTGTTCCTGCACACGTCCAT

TGTGCACAGTTGGT

>Tb927.10.10420 | Trypanosoma brucei TREU927 | monothiol glutaredoxin, putative | genomic | Tb927_10_v5.1 reverse | (geneCodeEnd+0 to geneEnd+0) | length=194

AGCTTTTAACGTCGCGCTTTGTGTGGTGGCCTGCAGTGTGTTTTGTGGCGGGTCTGGGTT

GCGTGAGAAGAGGCGTTTTGGGAGGGTTTTGTTTCCAATACCACTACTGCGTGGTACTGC

CAAAAAGCTAAAAGTTTCTTCATAATTGTTACGTGCCTACGCAGAGTTTGCGGCGTAGTG

TTGTACCGCTAGTT

>Tb927.10.4640 | Trypanosoma brucei TREU927 | eukaryotic translation initiation factor 3 subunit L, putative (EIF3L) | genomic | Tb927_10_v5.1 reverse | (geneCodeEnd+0 to geneEnd+0) | length=195

ACATTCGTGCGTTTCTCCCCTCTTTTAATATTGGAAGGAAGAAGAAGAGAAAAGGAGAGA

AACAATGGCGCCAAAATAGGATTATTCATACGCAAGTTCCTAATTAAGACATATAATGCG

AAGGTCACTAAGAGATCCAGATAAAATCAAAGTGTGGGGTAGCTACTTCCTCAAAACCCT

TTACGTTTTTGTCGC

>Tb927.6.2280 | Trypanosoma brucei TREU927 | prefoldin subunit, putative | genomic | Tb927_06_v5.1 forward | (geneCodeEnd+0 to geneEnd+0) | length=195

ACAGCTACGCTCTGCCCGCAATCGGCGTTCTAGGTATCTGCCGATATTGTGCATTTCTGT

TGTCTGAGGTGTGCGCTACAGAATGTGCTGCTTTGGAGTGCGGCACCCTATCAGCAAGGG

TCCGTTCCAAGTAGTTTTGGCGTGCGTGTGTGAGTTTGACTGTGATGCATGGGACACCGT

ATAATTTAACGTCTC

>Tb927.10.14530 | Trypanosoma brucei TREU927 | 19S proteasome non-atpase subunit 8 (Rpn8) | genomic | Tb927_10_v5.1 reverse | (geneCodeEnd+0 to geneEnd+0) | length=195

ACTGAATATGGATGAAGGCTGGGTAATGCACGCAGGTGGTGTGGGTCATGGGTTCGCGTT

TCCTTTACGCGCTGTGCATTTTGGCTGCACTTCGCTGCAACATGGGCGGTTTCCTCATAT

CTTTTTTTTCCTTTCGTGCTTGGCTACTCTGTGCCAAATCCGTTACCCTCTGTCTTCATT

CTGTTTCTTCAAGGT

>Tb927.6.2300 | Trypanosoma brucei TREU927 | adenosine kinase, putative | genomic | Tb927_06_v5.1 forward | (geneCodeEnd+0 to geneEnd+0) | length=195

ATGTTTTTTCACGTAAACACTTTGTTTAAAAATGTTATGTAACATAAAATATATGTTGGT

TTTTATCTTTTTTCTTTTCGGTTTCTGGTAAGTAAAGTTTAATGGGAAATATGGCGTAAG

CGATATCATATCACTTTGTCTGAGGGAAGAAAGAATGAGGTCATAAATGGCAGATGGTAA

CAGTGTAATATCTTG

>Tb927.11.880 | Trypanosoma brucei TREU927 | cyclophilin type peptidyl-prolyl cis-trans isomerase, cyclophilin a (CYPA) | genomic | Tb927_11_v5.1 forward | (geneCodeEnd+0 to geneEnd+0) | length=196

AGAAGAGCACGTAGAGGCGTGCACATGCAACATGAAATTACGCTCCGATCCCACTGCTTC

CCTCCCCTCCCCGTACTGATACACGCACAGACGAACACCGACTAATTTTTTTTTTCCCTC

AAGAAGTGCAAGTTGAAAAGGGGAAAAGCAAGCAGTAAAAGGGACAAAGGAGATTATTAA

AAGCAGAGGAGCAAAT

>Tb927.10.11560 | Trypanosoma brucei TREU927 | hypothetical protein, conserved | genomic | Tb927_10_v5.1 forward | (geneCodeEnd+0 to geneEnd+0) | length=196

ATACTTCGCTCGTGGAGGCTACTGCTTGTTTTTGCGCGCGTGCTTTTTTTTTTTGGCGTG

TGCGTGGTGCCAATGGGACATGTAGGTACGACGGCCTCTACGTTGTGTTTTTTTTTTTTT

TTACGCCACTTCTCCTTGTTTCAGTTTCTAGACGGGAAGAAACGGGTGTCGGAAACGGGA

TAGTGTCCTACGATTG

>Tb927.8.620 | Trypanosoma brucei TREU927 | KREPA3, RNA-editing complex protein MP42 (KREPA3) | genomic | Tb927_08_v5.1 reverse | (geneCodeEnd+0 to geneEnd+0) | length=196

AGAAGCAACATGGTGTTAATCTTTGCAGTTCTTAAGCACTATATGAACCATTAACTTCTA

AAAGGAGGCTGTGAAAGATTTGACAAAACCGGTCACTTAACAATGGGGTCAGAAAACTGA

GGACAGAACTCTACAGTTTGAAGAGTGTGAACTTACACGCCATCACGCTGCACTGTTGTT

TATTTGCCTTTCTTGT

>Tb927.9.3530 | Trypanosoma brucei TREU927 | U6 snRNA-associated Sm-like protein LSm6p, Sm- like protein (Lsm6) | genomic | Tb927_09_v5.1 forward | (geneCodeEnd+0 to geneEnd+0) | length=197

GTGGAGGTGCTAGCATCACGTTGCGCATGGTTAGGTTACGTTGTACCTGTCATGGCTTGC

GTCCTTGGACCATGAAGTAAAAGGAGGGGTTGTGGGTTTCCGTTTTGGCCGCTTACTTGG

ATGTTGCGTTTTACAGCTTAAAACCGTGGCTTTAGCTTTATCTGCCGCTCCTTGTCTTTC

GCCCGTTCTTTGCATTT

>Tb927.11.12610 | Trypanosoma brucei TREU927 | serine peptidase family S51, peptidase E, putative, cyclin 1 (CYC1) | genomic | Tb927_11_v5.1 forward | (geneCodeEnd+0 to geneEnd+0) | length=197

ATATTGGAAGAGCCAGCTGTTTCGTTAGGCCCCGGTGTACGGACATTTTGGTAGCAGTGT

CGTTTGAACGTGCGTTGGTGAAGAGGTAACGTTGGCGTTCCCAGATGCACGATAGTGTTG

TTTGGAATCGACACATGTGCGCGCGTTCGTCAACATGTGAATAGGAATTTTTTTTTGATC

CAACCCTTTGGAAAGCG

>Tb927.4.3010 | Trypanosoma brucei TREU927 | hypothetical protein, conserved | genomic | Tb927_04_v5.1 forward | (geneCodeEnd+0 to geneEnd+0) | length=197

AGATGGTGAGAGTGGAAAATCATCTCTTCCTCCAGACGTGAGGGTATTGACCGCGGCTCC

TGTGGGAAAGTTGTAGCAGTTTCTCTTTTTCAACCCAGTTCGTTAACTGGGTTTCTCCCC

CATCCTCCCACAGCGCCTTGTGCGTGCTGATGCTCTCTTCCCTTAATAAATACGATGCAG

TGACCTAGACACTCACG

>Tb927.3.4750 | Trypanosoma brucei TREU927 | metallo-peptidase, Clan MA(E) Family M1, Aminopeptidase M1, putative | genomic | Tb927_03_v5.1 reverse | (geneCodeEnd+0 to geneEnd+0) | length=197

AGCCAGTATATTTTGTGCATGGCTCCACTACGCATGACTGTGTGTGCAGGAAACACAGGT

GAATTTTATTTTTAGGGTTGGGGAGGGAGAAATGAGAAACATGAAGGAGGTAGTATTGGT

AAAGTTTATTGCTAGCGCATGAGTGGGTAACATTTATTTTACCATTCATCGGGAGACTTC

CCACCAGCTTGCTTGTG

>Tb927.7.2780 | Trypanosoma brucei TREU927 | hypothetical protein, conserved | genomic | Tb927_07_v5.1 forward | (geneCodeEnd+0 to geneEnd+0) | length=198

GCACGACCAATGGCTGCACATGGGATGTTCTTGTCGTCCAGACGAGCTTTTCGTGGTGTT

TTCACTATTGTGACCGGGTTTGATGCTCCACCTTAAGAGGAATAAAAGAGAAGATGAGGA

AAAGAAAGTGTACAGGAAATGAGTCATGAGGATTTTGGGGGAAGGTAGAAGAGAGAGCAC

ACATCAGGTGTTGTAATG

>Tb927.10.14720 | Trypanosoma brucei TREU927 | peroxin 13 (PEX13) | genomic | Tb927_10_v5.1 reverse | (geneCodeEnd+0 to geneEnd+0) | length=198

GGAACTCGTCATCTAAACCACTGTCCCCTGCTAATAATTCCTCTTTTGTCTACCGCCCTG

ATATACCCATCTGATGCAGCGATGTAGGGGTTAAATCCTTTACCGTGTCAGGGCATCAGA

TATTACTTTTCTCGCAGACAAACAACTTTTTGTCGTTAAATGACACTGCTTCGGCGGTGT

TTCTATTTGATCACCATG

>Tb927.8.5780 | Trypanosoma brucei TREU927 | phosphatase of regenerating liver-type phosphatase, putative | genomic | Tb927_08_v5.1 forward | (geneCodeEnd+0 to geneEnd+0) | length=198

AACCTCTGCTGAGAGACTAAGTGATGGTGTTCTAATCTGTTTATTATCAATCGATCCGCG

TACATATATATATATGTTGGTGTGTACATAAAGGTGAATAGGAAAATGGAAGTTAAGTAA

TTTATTACAAGTGATGGGTAGGGGACTGCATCCAATGCACGCGTAGGTACGCATACTCAC

GCTCACATATGTAGAGGG

>Tb927.6.4240 | Trypanosoma brucei TREU927 | hypothetical protein, conserved | genomic | Tb927_06_v5.1 reverse | (geneCodeEnd+0 to geneEnd+0) | length=198

AAGCGCTTCAAGTGCCATCGTTACGCTTCCGTATTGTCGTGTGTGTGGCTTGAGAGGGCG

CAGGTTACGCTCTAGTCATTGCTTGCCCTTGACAAATACTTGGTCTGGGTAGTGAGGGAA

GAACGCACATGATAGTGCAGCCTCCCGTCTCGTCTGGGGATGTCTTCGTTTCCCTCTATG

TGTTCTTTCCTTTCGTTG

>Tb927.9.15000 | Trypanosoma brucei TREU927 | proteasome complex subunit Rpn13 ubiquitin receptor, putative | genomic | Tb927_09_v5.1 forward | (geneCodeEnd+0 to geneEnd+0) | length=199

AGATGATTAAGACTCACGCAACCGCAAACGACTTCAAATTTCTATTCTCGCAATGTGGTG

TTTTGTTACCCGCACCGTTTCGTACAAATCGATGGGGATGGAAAGGGGGAAAAGTGTATG

TCACCGCTATGACGATAGCTGTTGTTGTACTTTCAGACACGTCCCCCCTCTTAACTTCTT

TTGAAAGAGTAGTACTGTT

>Tb927.9.14480 | Trypanosoma brucei TREU927 | dynein-associated protein, putative | genomic | Tb927_09_v5.1 reverse | (geneCodeEnd+0 to geneEnd+0) | length=200

AAGATGATCGTATATTTCGCCGAACTTCTGGTTTCTTTGGCTTTGCTTCCGATTTTGGGA

ATCATGTAGAGGCGGCATAACGGCGTTGTACCAACAAGAGACTAGTAAGGCAAAATAGAA

GAAAATAAAGATGTATCCATCCACGTATTTGGTCTGTTTTTAAATTTTTGTTTATGATAC

GGAAATTGGGGGTTCAGATG

>Tb927.11.10910 | Trypanosoma brucei TREU927 | 40S ribosomal protein SA, putative | genomic | Tb927_11_v5.1 forward | (geneCodeEnd+0 to geneEnd+0) | length=200

AGCGAATTGTTGCCTTGGGACAAGCACAGTTGTTTAACATATATGTCTTGGTTGTTACCT

CCGTATAATTTTTAATTTTTTTTCTTTTCGGTGACCGCGTGCGGCATCGCTAACTTTAGG

GAGGAATGAGGCGCTGCGCATTCGAAGGTGCTCTGCCGCTGAACAATGACTGCCTCATGA

CACTATACAACCAACACCTC

>Tb927.8.1600 | Trypanosoma brucei TREU927 | lysyl-tRNA synthetase, putative | genomic | Tb927_08_v5.1 forward | (geneCodeEnd+0 to geneEnd+0) | length=200

AAGGGTCTCAGGCACTACACGCGAGACAAATAGGCAGCTGAGCAACATGCAAAGGAAAAA

AAAAGGTAAGACAGAACGGAGGATCACAAATGAAAAAGCATTAATCCCTACGAAAAACAA

CACAGCATGATTTAAAAAAAAAACAGAATTCGGATGGCGTAATAAATTACACATTTCAGG

AGCAGTGTTAACATAATGTC

>Tb927.11.5520 | Trypanosoma brucei TREU927 | triosephosphate isomerase (TIM) | genomic | Tb927_11_v5.1 forward | (geneCodeEnd+0 to geneEnd+0) | length=200

ATTTTCCTTCATGTGTCAATGAGGTTTGGTGCTTTTGCCGTTGAGTGGGTGAAGATAGCA

GTATATATATATGCGCAAGTGAATATAAAAAAGATGTAAAGACAGGTAGCAGGGAGAAAG

CCTCGCATAACATTATAAAAGGGAGTGTAACTGGAGTGGGAAAACAAAGGAAAGGGGGAT

TCGTGTATTGAGCATATGAG

>Tb927.11.800 | Trypanosoma brucei TREU927 | prefoldin subunit, putative | genomic | Tb927_11_v5.1 forward | (geneCodeEnd+0 to geneEnd+0) | length=201

GAGCAGCTGAACACATTACAGGGGGAATTAAAAAGAAGGGAGAGTTAACAGCGTACGTCT

TGTACCCGCAGATGCCAGCCGCCCTTCAGCGGACATGCATGCAGAGCAAATAAACTAACA

ACCTTGTTGATTTCTCTCCCGCCACTGCCGCTAATTCCCTTTCTAACGGGTTTACCGTAT

GGAGAGATTTAATTACTGTTG

>Tb927.11.9790 | Trypanosoma brucei TREU927 | calmodulin, putative | genomic | Tb927_11_v5.1 reverse | (geneCodeEnd+0 to geneEnd+0) | length=201

ATTGGGTAGCGAAGATTGTTTTTGCTATAGAGGAACAGGGTATACATGTAGAGTTGAAAG

TGCGACTGGATACGAGAGTCCCCAGAGAGTTTCATTTTTCGTTACTCGCGCCTTTTTCAC

TACTATGTAGCATGGGTGCAACCTTCCTATGTTAAAACAGATGTTATATATACCTTTATG

CATATTTCTGTTAGGCAAATG

>Tb927.10.2640 | Trypanosoma brucei TREU927 | intraflagellar transport protein 81 (IFT81) | genomic | Tb927_10_v5.1 forward | (geneCodeEnd+0 to geneEnd+0) | length=201

GGGCTTTCACTGCGCGGCGGAAGGCAGCTGGTCATGGTTCCGCAGCTGGATAGAATGATA

AATGGAGTGAGTTTGTAGATACAGCAGCTTCAATGAGGTGTGCGGGTAGGAGCTGGAGTC

GAAGTAAATAGGTCGGAAGAAGGATGTGTTACATACAAAGGGAATTTGCAGATAATTGAG

CTCCAAGGGGATTGGTAAAAG

>Tb927.10.14690 | Trypanosoma brucei TREU927 | syntaxin, putative | genomic | Tb927_10_v5.1 reverse | (geneCodeEnd+0 to geneEnd+0) | length=202

AAAATGTTGTCCCCTGTGTATACGGAGGCACGTAGAGGGTGGTGATGCTTGCTACCGTAA

TAGTTGCACAACCCTGTTCGGTTTGTTCTTTGCTTCTCAGACCAGCTTTTTCTTCCTCTT

ACTGTTATGAGCGCAGTGCCTTTTTTAAAAGGAATAAAAAACTTTTTGCTCTGGGAGGAC

TGGTGATGAATATCGGCTTTTG

>Tb927.10.3030 | Trypanosoma brucei TREU927 | proteasome regulatory non-ATPase subunit 11 (RPN11) | genomic | Tb927_10_v5.1 forward | (geneCodeEnd+0 to geneEnd+0) | length=202

AGGAAACAGGGTACATTGCGTGTGCTCCAGCTATTACTGACCATACTTTTTATATGCGCT

ACCGTGTTGATTTTTTATCACCCTCTCTATAACGTTTACGGGGTGTTATGCTCCTTTTCT

GCACTCCCGTTTCTGTGACAATGTGGCGTGATTACAATCAGCGCAGGATGGCATTTGCTA

GTGATGTGGTGTCATCTTCGTG

>Tb927.11.320 | Trypanosoma brucei TREU927 | dynein light chain 1, putative | genomic | Tb927_11_v5.1 reverse | (geneCodeEnd+0 to geneEnd+0) | length=203

AGGCACCCACACAACCCGTGTTGCGGCATTAGAAAACAGGTCAGTTGTCGTCTTTTCTCG

TTTTTCGTCCTTTTCTCCCTCTTCAACCTCTCTCTGTTTCTGCCTGCGAGAATAAACAAA

AAAAGGAGAGAAAAAAAGGAAAAACATGTAGATACATACTTCCCTCTCAACTATTGAACT

GCGACTCCCGTGACGATTCGTCG

>Tb927.4.3080 | Trypanosoma brucei TREU927 | Uncharacterised ACR, YggU family COG1872, putative | genomic | Tb927_04_v5.1 forward | (geneCodeEnd+0 to geneEnd+0) | length=203

AGGGAGGGGGTGCCTCGTGTCTTTCTTCCCTTGCTTTGCGGCTTTATTTGCCATCGACTC

CGCATATATTTCTATATATATATATATATATATATATATACGTGTGTGTGTCAGCGCGTT

AATGTGAATGCAAGTTTACAGCAGAGGACGGTGGGACGAGTTGAATCCACCCCATAATTG

CTTCCATCGCCGTATGCTCGCTT

>Tb927.11.3900 | Trypanosoma brucei TREU927 | glycosyltransferase family-like protein, putative | genomic | Tb927_11_v5.1 forward | (geneCodeEnd+0 to geneEnd+0) | length=203

AGGGTCCAGCGTGATACGTAATTACACGTGTAGGTGCTGAAAACCCCGCGTGTCCGCGTT

CCTTCATGTTTGAATCAAGAAGGATGGGAACTCATTACGGCAATTGACGTAACGCATTGT

GGTAGTGGTACACCTCCATTGTTCTAGCAACACTCTCGATTGGCGTTCGTTATTACTCTT

GGTGTTTTCTCCCGAAACGTTTT

>Tb927.10.6120 | Trypanosoma brucei TREU927 | Peptidase M76 family, putative | genomic | Tb927_10_v5.1 reverse | (geneCodeEnd+0 to geneEnd+0) | length=203

GTTTTGTGGCGTCTTTCCGAGGACGTAAGAAAGACGCCGCAGCAGGGCTTGTCTGTTTCT

CACACGGACACGGGTTGGCAAGGAAATGGCGTCATAGCGGTTCTTTCGCTAATGTTGCTT

TTGGAAAAAAAAACATATATGGCGAAGGAGAAGAACTAAGACTTGAGGCCTTCTTCACAT

TACAATTACTCCACACTCTAACT

>Tb927.10.6440 | Trypanosoma brucei TREU927 | phosphomannomutase, putative | genomic | Tb927_10_v5.1 forward | (geneCodeEnd+0 to geneEnd+0) | length=204

AGCTACCAGGCTTTCTTCACAGTTTTTTCGAAGACAACGTACAAACACCCTTACTTACCG

TTGGTTTCTGATGGGATGGAACTCAATTGCTTTGGCACCGTTTTCTTTTAGCATCCAGGA

AGAACCATGGCACATTGAGTGTACCGAAGTTTGTGGATGCGGTTTACCTTTTTTGATTTT

ATATTTCCTAGTTCATGGAGACAC

>Tb927.11.4460 | Trypanosoma brucei TREU927 | ALBA-Domain Protein (ALBA1) | genomic | Tb927_11_v5.1 forward | (geneCodeEnd+0 to geneEnd+0) | length=204

ATGAAGAGCGAAACCGAAATGAAGAGAGGGAGAGAGAGAGAGAGAGAGAGAACGGAAAAA

ATGAAAATACACGAGGTACACGGGGAAATATGTTTTTCATTTGGGCCGGCTTTGATAACC

CATATCTTTGGAGAAGATGGAAAGAGAAAGTAAAAGGATAGTGGCTCTGGGACAAGAAAA

GGAAGAAATATACTAGGTATTGAG

>Tb927.10.10140 | Trypanosoma brucei TREU927 | paraflagellar rod component, putative (PFC19) | genomic | Tb927_10_v5.1 forward | (geneCodeEnd+0 to geneEnd+0) | length=204

AGGAACCTATGTATTTTTACTGCATGCAAAACAACTTATAAAAAGTACCGAAGGCAAGGA

AGAAAAACAAGGTGAAGAATGTTTCCAAGGGGCTCCCCGATGTATTTCGATGTTTAAAGA

GAGGAGGTTTTGATTCGTTTTGTCCTTCATGTATCTGGGCAACTAACACTCCGCTCCATT

TTTTGTCGCTATTGGCTTGTCCCT

>Tb927.5.1800 | Trypanosoma brucei TREU927 | hypothetical protein, conserved | genomic | Tb927_05_v5.1 forward | (geneCodeEnd+0 to geneEnd+0) | length=204

ACAGTCATGGAGGTGGACATTACATGTGTTAGTGGTGTGGCTTTCTTTTAGGTGATGCCC

TGCACCCTTTCCATTGCACAGTGTGCAAAAGAGTGATATTTTTCAGGTACTGCTGTTGTT

ATTGTTGTTCCGCTATGGATTGAGTGCTTGTGAGCAGCGGCACCTGGCTACGCAGGATTG

TGAGAGCAGTGTGCTTACCCTTAT

>Tb927.10.12960 | Trypanosoma brucei TREU927 | Ras-related protein Rab5A (RAB5A) | genomic | Tb927_10_v5.1 reverse | (geneCodeEnd+0 to geneEnd+0) | length=205

AGAGGGTTAGAGTATCAGCTTGCTCCGTTTTGTTCGCTCAGAAACTTTTGTTGCTTTCTC

CAAGGCATTACCCCCTCTCTCTTCCCCAATTTTTGTTTTAGAATATGTTTGAGGTACTAA

CCGTTGCGTGATAGCTTAGTTCAGTTTATTTTCCTGCCTCTATATATTCACTAACCATTT

TGCTGTTTTCTAAGTGTATAAGGGC

>Tb927.8.3120 | Trypanosoma brucei TREU927 | Uncharacterised protein family UPF0546, putative | genomic | Tb927_08_v5.1 forward | (geneCodeEnd+0 to geneEnd+0) | length=205

ATAATCGACATAGTTTTTGTGGCGGCTGCAATGCGATGGTGACCGCACTTCTCATTGTCG

GCAACATCGCAAAACGGAACGATTTAGCGAAGAGTGCTTTGACCGCGGCTTCACCTGGGA

TAAGGAACCCCAATAATTTAAAGAATTTTGGAACAGGGACATCGGAGTTCACTCGGTGTC

CTGCATTTGTATCTGTTTTCACTGG

>Tb927.1.1170 | Trypanosoma brucei TREU927 | DNA-directed RNA polymerase 2, RPB12 subunit (RPB12) | genomic | Tb927_01_v5.1 forward | (geneCodeEnd+0 to geneEnd+0) | length=205

ATAACACAAAGCCATTTAGGAGATGGTGCCCTTCCGGCTGACGCCTACATTGCCTCGGTT

GTGGCGGCCCCCTGTTGTGCGAGTACGGAAGAGGTGACTGAATTGATTCTCTTTCGCGCA

CTCCTCCGCGACGAACGCCATCAGTTGCGCACATACACACACACATACACAATTCTCTAT

GCCAAATTTCTGTCTGTGTGTACTG

>Tb927.3.2190 | Trypanosoma brucei TREU927 | RNA triphosphatase (TbCET1) | genomic | Tb927_03_v5.1 reverse | (geneCodeEnd+0 to geneEnd+0) | length=205

AGATACATGGACTCAGTACGTGTGCCCCTCCTTTTGTGGGTTGAATATGTTACTGACCTT

TTGAGGTGATAGGTATTTGGTTGGTGCAACTGGGCCATTGCGGAGCGGATACCAGATGCA

AGCGCAAAAATCTGTGACGCTACTCGCCTCTATATGCCTATCTCCTCCTCCTAAAACGTG

CGGTCTCTGTTTTTACTTTCCTTCG

>Tb927.3.4290 | Trypanosoma brucei TREU927 | 73 kDa paraflagellar rod protein (PFRC) | genomic | Tb927_03_v5.1 reverse | (geneCodeEnd+0 to geneEnd+0) | length=206

GTGTGGCCGCAATTATTATGTAGATTTTTTTGTTTGTAGATGTTTTTTTCTTATGAACCA

ACCCATTCTTCTAGAAGTTTTGTCATAGTTTTTACTTTTTGAGTTAGTGATCTTTGCGAG

ATTGTTACTTGTGGGTGGTCGTGCTGTTTGATGGAAGTGTAGAAAATGCTTCTTGTTCAC

TTCTTTCTGATGTATTTTTAGGTGAT

>Tb927.7.3420 | Trypanosoma brucei TREU927 | peptidyl-prolyl cis-trans isomerase, putative (PPIase) | genomic | Tb927_07_v5.1 reverse | (geneCodeEnd+0 to geneEnd+0) | length=206

AGAATTGTGGTAGCGCTGCGGCTGCGTATGTAAAATTATCTGAGAACTGGAGGGAAAAAA

TGCTCCTGTTATAATCAATACGTGCCTCGTGTCTTGCACTTTTGCTGAAGCACCCGAAAA

AGTAATCTGCATGACGTACGGGTATGTGCGGGGGTGTTGGTGAGTGTGTGAAATAAATGG

AGGTTGTCATTGTAAAGGCTGTGCTT

>Tb927.11.7880 | Trypanosoma brucei TREU927 | hypothetical protein, conserved | genomic | Tb927_11_v5.1 reverse | (geneCodeEnd+0 to geneEnd+0) | length=206

ATTCGCTCGCAGGGTTGACTTTTCCTTGCAACGCAAAACTACCACAGGTACGGGTGAAGG

TGTGAGAAAAGCATCGGTTCTCCCTTGCAGGCCGCTGGAGTTGGGCCTACAAAGTGCGAT

GTACACTTTTGTTCTCGGGAAACAATGCCGAAGCAACGCTACGTTGTTAAAGGTAGGGAT

CAGGCACTATCCGTTGCGCGATATTG

>Tb927.9.7800 | Trypanosoma brucei TREU927 | hypothetical protein, conserved | genomic | Tb927_09_v5.1 reverse | (geneCodeEnd+0 to geneEnd+0) | length=207

ATGTGTGGTCGTCTAGTTTGCGGTGCATACGGCAGATGTTTTGTTGCGGTAGGGGAGATA

AAGAGTCTTGTGGGGGGGGGGGGTGATAAAATGGATGGACGAAGCGATTAAATGGTTTCT

TTCGGTCGAGAGTTATAATCAGCGTGTGTGGGTTATCTTGTTTAACGTTTTTCGTCGTCA

GTGATGCTTTACACCGATGCAAGGCTG

>Tb927.7.3750 | Trypanosoma brucei TREU927 | TFIIF-stimulated CTD phosphatase, putative | genomic | Tb927_07_v5.1 reverse | (geneCodeEnd+0 to geneEnd+0) | length=207

AAAAGGGGGTTCAACCAAAAGTGTATGGTCGAAGCAATTCGCAAGGAAAAGTGCACAGGA

AGATATTTGTATGTGAATCGGTGCATTCACATTGTGATGAAGGAGAGGGAAGGACATGAG

ATGAATTGGGTGCTGTTACTCCCCCCTTTCCCGCCCCTTCTTGAGAGGACACCGTTTCAT

GGTGCCTGTATGGGTAAATGGCTAGCT

>Tb927.11.3690 | Trypanosoma brucei TREU927 | Alpha/beta hydrolase family, putative | genomic | Tb927_11_v5.1 forward | (geneCodeEnd+0 to geneEnd+0) | length=208

AACGGGGATCGGCTCGTATCAAAGCTCTTATTCGAGGGTTGTGGACATGTTTGGCTTTGC

TCCCGATTTGCAGAAAGAGAGGATGGGTTCATTTCTCTCGCAACTTGACGTGACCGATGC

CAAGAATATGAATGCGTGAGGAAAAAAAGAAAGGATAAGTGAGGGGCACCGGAGGGAAAA

GATCCCTTCTAAAGAGCTGAATATTAAG

>Tb927.7.1180 | Trypanosoma brucei TREU927 | RNA-binding protein, putative (RBP19) | genomic | Tb927_07_v5.1 forward | (geneCodeEnd+0 to geneEnd+0) | length=208

AAAAAATTGGGAGTGGAAAAAGGGGAATGGATGACAGTCTCCGTCACTCATGTTGCCATG

GTTGTGCTTCCTTTTTTTTTCGCGTGTCTGCCATTTTGCCGCGGTGGTTTCTGCCACCGA

AATGGGCGTGTTTCGTCTCTGCTCCGCAGGGGGAGGGAGCGAGGTGAACAGAAATTGGTG

CACCTACACCCTTGCGAAGAGGGTATTG

>Tb927.11.850 | Trypanosoma brucei TREU927 | protein kinase, putative | genomic | Tb927_11_v5.1 forward | (geneCodeEnd+0 to geneEnd+0) | length=208

GGCAATAATAGCATTTACTTGTCGTTGGCGGTGACACTCCCCTTTTTTCTCTTCACTATT

TTTGCGTTTCGTTTTTCCCACTTGGTTTACTTGTCAGGACCGATGGTGCCGTATGGGTCG

CCCTTTTTACTTGGGCGGCGTTCGCATCGATGTGTGCTTCCGTATTTGCACGATGTTGAA

GGGACGTGTCCGCTTCATATCTCTGCTT

>Tb927.11.12030 | Trypanosoma brucei TREU927 | hypothetical protein, conserved | genomic | Tb927_11_v5.1 forward | (geneCodeEnd+0 to geneEnd+0) | length=208

ACCCGTAAAACCCAGGAAAATGTTTGGGAATATGTATCTGTTTCTCTGATCGGGAAGGCA

GGTAATATGATACTGTACCAAGCGGAGGTAAGTTAAACATCGTTTAAACGTTTTTTTTTG

TATTTGGGTCGTTGCATCGTAAAGAGAATTTATTCCCTGCCGAGGGAAGTGGTAAATGGT

GCAGTTCACAGTTCTGTATACAATGCTT

>Tb927.8.5770 | Trypanosoma brucei TREU927 | Elongator-like Protein 3a | genomic | Tb927_08_v5.1 forward | (geneCodeEnd+0 to geneEnd+0) | length=208

ATATGTGTGACTCCTCCCCCCCCCCCCTATATGTTCATTGGGGATGCAGCAATATCCGCA

ACCAATCATATGGAGGGTGGAGCTGCGGGGGGAAGCGTATCTACAATAGCTAAAGTTCAA

CTCCTCTCTGCTTCAAGGAGTCCCAGATTTTGCCGTAAGACAATGAGTGGTGTTTTCACG

TACGAGTAAGGTTTTAACTAGTTTGATG

>Tb927.6.2410 | Trypanosoma brucei TREU927 | hypothetical protein, conserved | genomic | Tb927_06_v5.1 forward | (geneCodeEnd+0 to geneEnd+0) | length=209

GCCACGGTTACCAGCATAATTGCGGGTGCGTTTGCAAAGAGCAGCATCGTTCTCAAATGT

TTCTCTCTCACTTTTTTTAATCCCTCAGTTTCACGGTACAGCGGGAGGCGTGCACAAGCT

TTCGCTTAATAGTCGAAAGCTGCGTTGATTAAGCATGTAGGGGACATCCCTTCTCCGTAC

CGCTTGGATACGTATCTACTCATCTCTCT

>Tb927.9.14180 | Trypanosoma brucei TREU927 | chaperone protein DNAj, putative | genomic | Tb927_09_v5.1 reverse | (geneCodeEnd+0 to geneEnd+0) | length=209

AAGGAGCGCCCGGTATTCGCGTCAAGGCTCCCGTACAAGCCGAAGTTGTCGCGAACAATA

CCCCCCTTTCAACTCGCACTCCGTTCGTTTGTGAGTTTTTGCTCACTCGTAATGGGGAGT

GTGCGTTCCGTTATTGGAGGGAGGTTTCTTTGTTCTCCCTCCCGGTTTCTGGGCGTCTCC

CAACTCTCTTCTTTTTAATTTGCATTTCG

>Tb927.8.3140 | Trypanosoma brucei TREU927 | NAD dependent deacetylase, putative, SIR2-like protein 4, putative, NAD-dependent deacetylase sirtuin 4, putative | genomic | Tb927_08_v5.1 forward | (geneCodeEnd+0 to geneEnd+0) | length=209

AAGATGGTTGTTGGTGCCAAGTGACATGAAGGAGTCGCCCCTGCGGTGCCACCCTTGTTT

TGTCACTGTTGGTGGCGACGATTCACTTTTGCATGTGTTTAAAATCTCTTTTTCTGTATC

CTTCCCATGGAGAGAGACAACGATCGCAGATGGACGATGGTGAGATCTCTAGCGTTTGTC

CCTAGAAATCTTTTTTTGAATCCACCATT

>Tb927.11.580 | Trypanosoma brucei TREU927 | lipid-like atypical dual specificity phosphatase, putative | genomic | Tb927_11_v5.1 reverse | (geneCodeEnd+0 to geneEnd+0) | length=209

AGGTGGAGAGGGAAACAGCGCAGAAGAGAGGAGAAAAAAACACATCATGCTGTTGCACTA

GGTGAGACTGGCGTGTTGTGTCGCTCGCATGGTGCCCGGCATTTTTTAAAACTGTGGTCA

TTGGGTCTTTGTGGATTTTTTTTGCGTGTTCCCTTTTTTTCATCGCTTTGCTCCACCTCG

TGGTTTCTTCCCTCCTTTGCAGTTCAGTG

>Tb927.3.5340 | Trypanosoma brucei TREU927 | Hsc70-interacting protein (Hip), putative | genomic | Tb927_03_v5.1 forward | (geneCodeEnd+0 to geneEnd+0) | length=209

AGTTAATGGCAATATTGTGGGAACCCCACCCCCTCATTGATTACCGGCATATTTAACGTT

CAGAATGGTGAGTAGTTGTTCAGGTAAGTATAGCAACACACATACTAAAATATACTTTTT

ATTGTCATGAACAGCAGCTCCGTGGTTAGTAGCTCTTTATTATTTGATTGTCCACTCTAT

ACAAAACAAAGTGTACGGTGTTAAAAACG

>Tb927.10.2380 | Trypanosoma brucei TREU927 | Tetratricopeptide repeat, putative | genomic | Tb927_10_v5.1 forward | (geneCodeEnd+0 to geneEnd+0) | length=209

AGGGGTTAAGAAGTGCTGTTGCGCTCTGCTCAATTACATTTTCTCATATGAGCGGTATTA

GTTAATAATCAAACTCACACTGTCCATCGGGTTACCAGCGCGACCCCAGGGGGTTTCCCC

CTCTCTTCAGTTCGCACTTCTCTCTCAAAGTATATTTCCCTTTACTGAATTAGCGCATGT

AAAGAAGAAGAAAAAAAGGTTGGAAAAAC

>Tb927.10.12880 | Trypanosoma brucei TREU927 | SET domain containing protein, putative | genomic | Tb927_10_v5.1 reverse | (geneCodeEnd+0 to geneEnd+0) | length=210

GGTACGAGGGGATGGTACAGTTGTTTGGAACACAGCTACCCCTCTTTCGTTGCACTTTTC

GTACCGAGGAAGGCGCGTGCGTTATACTCGTTATCGCCTCAGACATACTCGCATGTGTGC

AAGTAGCTTTCAAGAAACGAACAGTTTACTTACTTTGAAAGGAGTCGTCTAACATTGTTT

CGCCGTTTACCTTCTTGCTTGCGTCTCATG

>Tb927.6.2240 | Trypanosoma brucei TREU927 | hypothetical protein, conserved | genomic | Tb927_06_v5.1 forward | (geneCodeEnd+0 to geneEnd+0) | length=210

ATACCAAAGAATAAAAAAGTAGATTGATACATATATAGATCTATGCAGAGATATGGATAC

GCACTCGAGTATGTGTGTGACTTCGTACCTGAATGTGAATGGAGATAGTGCTTTATTTCC

TTATATGCTTGGAAGGTGCCACCGTACAGAGCACATGCACTCTCAGCCACTGTTTGCCGC

TGCGGTGGGTGGTAAAATGTCATCGTTGCG

>Tb927.10.8040 | Trypanosoma brucei TREU927 | adaptin complex 1 subunit, putative, beta adaptin, putative (BAD1) | genomic | Tb927_10_v5.1 reverse | (geneCodeEnd+0 to geneEnd+0) | length=210

AAGGGAAGAAAAGGGTTAACAGCCAAATCCGCAACTGCTCTCCCACCGTTCCTCTTCGTT

GTACGTACCTTTTCCCCTGCTGGACTGTGTGGATGTGTGGATGGACTTTGCTTTTCACGC

CCCGGTAGTTTTGAATGTTTTCTGTTCTTCTACCCTTATTGTTGTTCGCTCTCTTTATTA

CTCTGAAGAGAGTGGAGGAGGAGAAGCGCC

>Tb927.10.8190 | Trypanosoma brucei TREU927 | T-complex protein 1, theta subunit, putative, CCT-theta, putative | genomic | Tb927_10_v5.1 reverse | (geneCodeEnd+0 to geneEnd+0) | length=211

AGAGGTGGCGTGCGGCACAGATACGGTGTAGTGGGAGGAGGTCGTCCTTACGCGTCAAGC

GCTGTTGCCTCGTAGTGGATAGATCTTGGTAGGCATATTCTGAACTCGTGCCTCTACGGG

ATACTTTTTTTTATTTTGTTTTTGTTTTATTTTGATCTTGTGAAGAGAGCGCTAAACCAC

TTAAGAGAAGAGGAAGGAAGAGGAGGATATT

>Tb927.8.7020 | Trypanosoma brucei TREU927 | peptidase, putative, metallo-peptidase, Clan ME, Family M16 | genomic | Tb927_08_v5.1 forward | (geneCodeEnd+0 to geneEnd+0) | length=211

GGTGTATTGAACGTGGTGGGTAGGTTTGTTTGTTGTTTTTTTTGTGACAGCGTTGCGGAT

ACCAAACTTCAGTGGAAAGGGTGCTTCGTGTAGGCGAATGTTTTTTTTTTCATTTTCGAG

AGGGGAGTGGTGTATCCGTGCCCGGTGTATACGGGTCGTCCAGGGAACCGCTTCATTTAT

CTAATGATCTAATTCTTCTCGGGATGTGTTG

>Tb927.10.13810 | Trypanosoma brucei TREU927 | hypothetical protein, conserved | genomic | Tb927_10_v5.1 reverse | (geneCodeEnd+0 to geneEnd+0) | length=212

ACTTCAGGGAAGTGTGGTGCCAAGTGAAGACGCCGTTGTGATTGTTATGTTAAACATGCA

CTTCACGAACGTTTTGTGGATGTTCGTGTGAAGGGGTTTCGAAGGCTACAGGTGTACTTT

GGGGGCGGAAGCCACAGGATTTGGTGGCTGTGTTGACCGAGGGATGCGTTCAAGGGTAAA

TTTTTGAACACTTGTTCTTTCCTTTGCTACTG

>Tb927.4.470 | Trypanosoma brucei TREU927 | snoRNP protein GAR1, putative | genomic | Tb927_04_v5.1 reverse | (geneCodeEnd+0 to geneEnd+0) | length=212

ATGCAGTTGGAGCTGATGGGTGTCTTTTAACCTATTTGCGCCACCCATGATCGGTAGTAA

CTTCGGCTAACGACGCGCAAGTAAACGGAGAGGAAAGGGTGTAACACAAGAAGAAAAGGA

AAATGAAGGAAACGAAGAGACATAAAAGGGAACGAGAAAAGCCGCTTAATGTTAAACGGC

GAGTGAGAATCTGTTCTAATAAGTTTCACCGT

>Tb927.11.3090 | Trypanosoma brucei TREU927 | LisH domain-containing protein FOPNL, putative (fopnl) | genomic | Tb927_11_v5.1 reverse | (geneCodeEnd+0 to geneEnd+0) | length=212

AATCATGCCTTTGCTGGGGGCTGTGTGGCATGTCGGAAGGGGGACGCTGTTTAGTGACGT

GATTTCTTTCCGACAAAAAAGTTTAAATTTGCGCAGCAAGTGTTGGAGCCAGTAATAGAA

GTTTTGGGGGGCTTCATTGCACACCGCTTCCTTCCCTACTCTCGGTGTAGCTCCCACCAT

CGTTTGTTCCCTACTTCTTTTTCCTCCTATCG

>Tb927.9.12710 | Trypanosoma brucei TREU927 | hypothetical protein, conserved | genomic | Tb927_09_v5.1 forward | (geneCodeEnd+0 to geneEnd+0) | length=212

AATATGATAGGTGTGTATCATCATCTCCCCTTTCCTTTGTGAACGTGTATGTGATCATCA

TTACGGTTGCTCTGTTTTTTGGAGGCACTCCGTCGCAGTGTTTGCTTGTTGGTATAGTTG

TTGTTTTTTGCAGAATTTCCCGATGCTGAATGGACAACTGAAATCGTACTATTCACATAG

GGAGTTGCTTGATCGCTTGATTGGTGAATGGG

>Tb927.9.4360 | Trypanosoma brucei TREU927 | KREL1 (KREL1) | genomic | Tb927_09_v5.1 forward | (geneCodeEnd+0 to geneEnd+0) | length=213

ATACTGCAAATTGTTCAATTCGTGGTATCCTGCGAGCGCTCATATGCCTCCTTCCCTACT

GCTTTTCAAGATGACGTCTACATCCTAATCATTGCGTGCGGTAGTGATTCACTACGCGTC

ACACAAACACATCTTTTTTGCATAAGGGTAATAAAGCCTTTCACATCGGTTGCTTGGTAC

TTCCGAGTCCGGGGTTTCCCCAGACCAGTGGAG

>Tb927.7.1390 | Trypanosoma brucei TREU927 | RNA-binding protein, putative (RBSR2) | genomic | Tb927_07_v5.1 forward | (geneCodeEnd+0 to geneEnd+0) | length=214

ATAGCATTAAGGTAACTGGGCTCTCATTTCAGTTGGGAGAAGTGAGGTGTTCTCAATCGA

GATTAACGAGTTGGCGCACGGATGTGTTGTGCGGAAAATGCGTGAGACGAACAAGGAGTT

GTTCCGTCGATATGTTTTTGTTCCCCTCCTTCTGGCAGATAGTTGTTTGTTCGTTGAAAT

TAATACTGGTTTAATGTCTCTTTCCCCTCAATTT

>Tb927.11.16440 | Trypanosoma brucei TREU927 | Transmembrane protein 18, putative | genomic | Tb927_11_v5.1 reverse | (geneCodeEnd+0 to geneEnd+0) | length=214

ACTTGGTTGTGGATGATCGTGTAGGGGGTTTGCACCCCTGTTAGCTCTTTCCTTTCCCCT

TTGTGACGGTGCTTGTCACTGCACGATTCCTTTTGTGCTCTCACTTTCCGGTTGCAAAAG

GGAGGTTATTGTACTGCAAACCTTTATCGGTGTGTCGTGCGCCCTTACATTTTCCTCGTT

TTTATGGCGTATGGGTGGCCGCTTATAGCCGTTT

>Tb927.10.5630 | Trypanosoma brucei TREU927 | hypothetical protein, conserved | genomic | Tb927_10_v5.1 forward | (geneCodeEnd+0 to geneEnd+0) | length=214

ATCACTGTGTATCATTTATCCGGGTGTAGTGACGGTGAGTTTCTCGTGGCTGAAGGGGGT

TCCTTGCACCATTTGTACTGTGCACTGCGTAACTAGCATTTTGCCTCTGTTGCTCCTTAA

GGGCATAGGTTCATGTAGATTAGCGTCCCGGAGGAATTAGTGTGGAATTGTGTTGGAGAA

GCTTCTTGCCTCCGTTTTAGACCACGATTGTGAT

>Tb927.8.3210 | Trypanosoma brucei TREU927 | hypothetical protein, conserved | genomic | Tb927_08_v5.1 forward | (geneCodeEnd+0 to geneEnd+0) | length=215

GTGTTGTATTACTTGTGTTTCACTGAGGGGTTTTATTGTATGTGCGCGCTCATGCTTGAG

CTGCGATAAAAACAGTTGAAGTGGCGGTGTGCGTGTTGGGGACGCGGTTAAATCCCTTCG

GGATGCGGTGAACATGGAGTTGCATGCGCGGTAGTTTTTTTAATTGCCGCTGTTTGGACC

CGCGCCGTGGTCAAGCGCAAAATGAACATTATTTT

>Tb927.11.15420 | Trypanosoma brucei TREU927 | COP9 signalosome, subunit CSN8, putative | genomic | Tb927_11_v5.1 reverse | (geneCodeEnd+0 to geneEnd+0) | length=216

GGTGTATGGCCGCGTTGGCGGTTCGTCACCCTCCGAAGGCGCTCATGTGCTTCACGCGCC

TTCCAGCCTGAGGAGGTGCAGGAGTGGACGCCCGTGGGGTTTAAGTGCATATATATATAT

ATATATATATGTGTGTGTGTGTGCATACCTCGTTGAGGCAACTTGTTGACTAAAAGGGGA

ATCTGTGCGAATAAACCTTCTCCTCCTCACAACCTT

>Tb927.11.10970 | Trypanosoma brucei TREU927 | RWD domain/Uncharacterized protein family UPF0029, putative | genomic | Tb927_11_v5.1 forward | (geneCodeEnd+0 to geneEnd+0) | length=216

ACTCAGGTGATCGTGAAAGGTCCGACGGAAGTGCAGCGCTATTGTTCTGTTCATTTAACA

TGGTGTGCTTCCTCACTCGATGGTGATACCACGGGGCCTTACTGGCGGTATCTCAAAGAT

CGCAAGTCGTCCAGGTTACTTGGAGGTCTGTGTTTGCAGGAGTACCTCTGAGACTGGAGG

TCAATTGCGTAAATCTAAACTTCTCGTGGTCCTTTG

>Tb927.5.3090 | Trypanosoma brucei TREU927 | hypothetical protein, conserved | genomic | Tb927_05_v5.1 forward | (geneCodeEnd+0 to geneEnd+0) | length=217

ACTGGTGTGCTTAACACGGGGAACGGGGCACCCCTTGGGAGTTGCTTGCTGTACGGTTCC

AAGGAATGGCGTAACGTTGATGTGTGTGTTTTTAGCGGATATATTTTATTGCAGTGGCGT

GTGTAGCAGTTGGTCGGGTAAGGAATTGAACGAAAATTTGTCTGTTTGTTGGCTGCTTTT

TGCAGCCTTAGGAGCATGCTGGAGAGTATTTACACCG

>Tb927.11.12750 | Trypanosoma brucei TREU927 | cleavage and polyadenylation specificity factor 30 kDa subunit, CPSF 30 kDa subunit, NS1 effector domain- binding protein 1 (CPSF30) | genomic | Tb927_11_v5.1 reverse | (geneCodeEnd+0 to geneEnd+0) | length=217

AGCTTTGGGAAATGGGTTCTCCCCCGTACGTGAAGGCACACGTCGCATCCACGAACTGTG

TCTGTATGCATCATTTTATTGCAACGGGTTCATGCAGGCACAGATACGAAGGTTCTTTCA

GTTCATGTCGGGAGTAAAGATCGCGCCCCCTTCTCTGCTACCACGTGCAGCGGTCTTCCC

GCGTTTTCGTCTGCAGGTTACTCTGATGCTCTCTGTC

>Tb927.9.5690 | Trypanosoma brucei TREU927 | 60S acidic ribosomal protein, putative | genomic | Tb927_09_v5.1 forward | (geneCodeEnd+0 to geneEnd+0) | length=217

AATCGGGCAGAGTATACTCCTGGTCTGTCGTTTCCGCTCGCTTTGCATCACGTTAGGACC

TTTGACATTTTGTGTGTTCGGTGAAGTCCGCTGGGTGGTCGGAGTATCCTGGGGCGAGAG

AGTGGAGGGAAGGGCGAGAGAATTCTACCCCGTTACGTTGTTTCTTCTTTTTCTTTTTTT

TATATCTTAAATAATTATCTTCTGATTTGTGCATTTG

>Tb927.11.3000 | Trypanosoma brucei TREU927 | 60S ribosomal protein L37, putative | genomic | Tb927_11_v5.1 reverse | (geneCodeEnd+0 to geneEnd+0) | length=217

AGCAGCCCAAGGTGGTGCAGTGTCTGGATATGATGCTGGGTGGGTGGGATGGAAAAAGCA

CACACCAATGAAAAGTGTTACCTTGAGACCTCAACGCTGTTCTCACTTGTCGATGGCTAG

CTGTATGCTGACAGTTTTCGTAGTCAGTGAGTTCTTTGTTAGCCTCAACATGGCTGCAGT

TTTTCTTTTTCCTTTCTGATTTGTCCCCCTAAGCCTT

>Tb927.7.630 | Trypanosoma brucei TREU927 | hypothetical protein, conserved | genomic | Tb927_07_v5.1 reverse | (geneCodeEnd+0 to geneEnd+0) | length=217

GCGCTCGTATATTACGTATACATGTTTGTGTGTTGTGGTTTGTATGAATACATCATTGCT

AGCGTATATTGGTTTCTGAAGCAATATATGGCAAGTGTTGTGGGGCTATCGACTTCAATA

ATTCTACAAATACATATATTTTTTTTTCGGGCCATTTTCGTTGTAGGTGTGTGATTTGTG

TTATATGCTCCGGGAAGCTGACAATGTTTAGTGCGTG

>Tb927.10.2620 | Trypanosoma brucei TREU927 | CS domain containing protein, putative | genomic | Tb927_10_v5.1 forward | (geneCodeEnd+0 to geneEnd+0) | length=218

AGAAAATTCCCATATTCAATCTTATTGGCGTATTACGCCTCCACAAACTATTCAAAGTAA

AATAACAAGCGGGCGAATTAGAAGCAACTCAGATGATCCTTTTGAAAGGGGGAGCAGAAA

GCACTTCCAAGGGGGATGTGGGACAATAATCTCCGCACGCTGTGAATCAAGTGAATTATT

TTTCCGTACGCTGGGTATTATACACTGAGTTGATATTT

>Tb927.7.1540 | Trypanosoma brucei TREU927 | Staphylococcal nuclease homologue, putative | genomic | Tb927_07_v5.1 forward | (geneCodeEnd+0 to geneEnd+0) | length=218

GTGAATGGGAGTAACTTTTGTTGTTGTTGTGGTTGGGCAATTCGGACTGCAACAGTTTCT

TGGTTCCCGCTTGTGGCACCGAAGTGTTTCATTTGCATATTGCTGTTGGGCAACAGTAAC

TCACGTTGCGTAATCAGATTTTTTTTTGTGGAGTCATTGTTGGGCCGGCCCTTTTTCTTC

TGTCTTACGTCTCCTTTTTGGCTACGCTATGCCATACT

>Tb927.7.5640 | Trypanosoma brucei TREU927 | G-patch domain containing protein, putative | genomic | Tb927_07_v5.1 forward | (geneCodeEnd+0 to geneEnd+0) | length=218

AGGGCAAAGCTACTACTGGTGGAATTTACATCAAGTGGGGCTTTAGCCGTCTCCACGTGA

GGAGACGGGGGAACAACAAAAGATGTTGCGTGAAAAGGTGGCCACGTGAAGTTATATGCT

TGTGAATGTCGGTGCATGCACAGCTGCATACATGCATTCTCTCTTTCGTAGCTGAGGCAC

TTGCATTAACTACTGCGAATAGCTATAAACCGTCGTCT

>Tb927.9.10850 | Trypanosoma brucei TREU927 | Splicing factor 3B subunit 10 (SF3b10), putative | genomic | Tb927_09_v5.1 reverse | (geneCodeEnd+0 to geneEnd+0) | length=219

ACAGCCCAGTCGATTATCCAAGGACTACCCACCGTGTGTGTTTTGGTGAAGATTTGGATC

ACCGAGCGGTACGGCACGCAAAAGGCGGAGAGGAAGTCAACATGTGCCCGGAGAGTGATT

TTCCATCTATATTCTCTCTCTCCAATCTCTTTACAGCTGTAAACTGGACCCACACGTTGC

GCTCCATCGTAGTTTGGCCCTTTACGCGAAGGCACGTAC

>Tb927.7.7440 | Trypanosoma brucei TREU927 | hypothetical protein, conserved | genomic | Tb927_07_v5.1 reverse | (geneCodeEnd+0 to geneEnd+0) | length=219

AATTGATGCTTGATGGACCAAACATTACGGGAACACGCATTTCTTAGCGTTTGTGGTGAC

TGCTGCCCTGTTGTGTGGTGGGGGTAAAAGTTTTGGAAGCAATAATACAAAGGCTTACGG

TTTGTGTAAATTGATGCAAGGCATTGAAAAACATATTTACAGAACAAAATGGAGCCGAAA

GTTAGAGAAAAAAGTATGACGTATCGTACACGCTGTATT

>Tb927.4.3900 | Trypanosoma brucei TREU927 | hypothetical protein, conserved | genomic | Tb927_04_v5.1 reverse | (geneCodeEnd+0 to geneEnd+0) | length=219

ACGGCATGCAAAAAGGGCTTAGGCGAGACGCTCTGTCGAGAGAAAGTCACAACGGGTAGA

AGCGACCATTTCCGCGGGGTCGCCACGCTTCCGCTCCCCATAATTGCCGACGGCTTCCTC

TCGTGCATTCAGTGTGCAAATTCATTTTCTGCCTGCTGTGTAGCACGTTTGGAAGCCGTA

AGTTGCACTCACTGTGTTGACACTCACCACGAAACCCGC

>Tb927.10.11380 | Trypanosoma brucei TREU927 | hypothetical protein, conserved | genomic | Tb927_10_v5.1 forward | (geneCodeEnd+0 to geneEnd+0) | length=219

GGTGTGGCGGTGGTGGAGTCGTGTTGTGCGAATACACTGCGTTCACGTTTGTGTCCTTTA

ACTTTCACCCTAAAAAATTGTTTTGGAACTTCATTCGAGTGTGGGTGGGCCACGGTGGAG

AGGGAAGAACGCAAAGGAGAACAACGGCTGTGTGTAGATGATATTGTCCCAGCCTTTCCT

GCTCGCGTATACGATCTAATAACGCCCATACTTTCGTGT

>Tb927.10.3280 | Trypanosoma brucei TREU927 | 60S ribosomal proteins L38, putative | genomic | Tb927_10_v5.1 forward | (geneCodeEnd+0 to geneEnd+0) | length=220

AGTTGTACGACTCCGTGCGCTGGGTGGAATTATAGGGGGATGAAGGAAGAGCCGAGCAAA

GTTTCACAATGGAGTGCTTTTGTAACCATAACCCATGGACGGTTGCTCTTTCCTTCGTCA

CCAAGAAAGTGGCACGCTTCTCAATCAATAGTTGCTTCGTTTTTTCCTTTTCCGTACTTT

TTTTTTGTTTCATTTGAAAAATGAGTACATTACTGTATTC

>Tb927.2.5260 | Trypanosoma brucei TREU927 | hypothetical protein, conserved | genomic | Tb927_02_v5.1 forward | (geneCodeEnd+0 to geneEnd+0) | length=220

AAAGCAGAAGATGCGGTAGCAGCAGCGACAGAAGCTCGAGACAAAAGGTGGAGGCACCGA

ACTGCTGGCCGTAAGGAAGGCGCCTTGGGGGCAGCGAAAGGCGCAAAAGTAAAAAAAACA

AAGAGGAGTGGGCATGAAATTGCTAGTGGTGGGGTTCTTTCCATACTAGTGGGCGGAGCT

GCGGGCTATTTTTCTTCTGCATGGGGTCGTCAGTTCTGCG

>Tb927.10.16120 | Trypanosoma brucei TREU927 | inosine-5'-monophosphate dehydrogenase | genomic | Tb927_10_v5.1 forward | (geneCodeEnd+0 to geneEnd+0) | length=220

AGGCAGAAGTGCCCGCACACCCACTTGTCCACTTCCTTTTTGGCACGCAGCAACTGCTAA

AGCTCCAAGGGGTTCCGGACGGGGCTGAAGACGTTCAAGTGGGGTTATATATTACCGTAG

TGATGTGCATTTATCAGTGGTGTTTGGGGGCTGTTTATATTGGTTTGGGGGCCGCACCCC

ATCTGTCACCGAGTGACGCTGCGGCAATACAAACGTAGTC

>Tb927.8.2800 | Trypanosoma brucei TREU927 | hypothetical protein, conserved | genomic | Tb927_08_v5.1 forward | (geneCodeEnd+0 to geneEnd+0) | length=221

AGCTATACACAGCATTAGTTTCCTAGAGTATATGCAATTACCGGAGATCTCCAATTACCC

TTACATATATATATATTATTTTTTCGACTCCTTTGTGAGTGGAAAAATTATGTGTAGGGT

AGCAGGAGCTGAACTAATGTATAGAACGTGGGTAAGAACATGGAAAGAAGGCAGTAACCG

TCGTGAAAATGTTGTGCCGATGTGCACATTTGTGAGGCCTG

>Tb927.10.12540 | Trypanosoma brucei TREU927 | NADH-ubiquinone oxidoreductase complex I subunit, putative (NDUFS1) | genomic | Tb927_10_v5.1 reverse | (geneCodeEnd+0 to geneEnd+0) | length=221

GTTCGTGAGGCGTTTTTGAGCGATCTGTGCCACAGACGCTCACAAAATATCGACTTACTT

TCTTTGGCTACCGTGCTATATAATGTGTTTCGGTGCTTTGTAAACAAATTTAGCAATAGG

GATGTGCTCCGGGCGAGGATAGGAACGCACCACCGTAGGCCATTTCCTTTAGGTGGCGTT

AGCAGTGGCTACGATACCGTACAGTTAGTAACAATGATTTT

>Tb927.7.4990 | Trypanosoma brucei TREU927 | hypothetical protein, conserved | genomic | Tb927_07_v5.1 forward | (geneCodeEnd+0 to geneEnd+0) | length=221

AATACACGTGGTTACGTTTGCGGGGCGGGTGAGTGTATTTACGTTGTTCCCCCAGCGAGT

TGTCCTAGTGGGCGAGAACCAGAGCCACCGGCCGCGTCAGACTACATTAATATTCAACTC

CATGCCATTATATTTCTTTATGGTTTTGTTGTCCTTTGCTTGCTAACTGATTTTAACGGA

TCATCCGGAGTCTTTTTAATGTGTGCGTACACGTGCTGCAC

>Tb927.10.4740 | Trypanosoma brucei TREU927 | nucleolar RNA-binding protein, putative | genomic | Tb927_10_v5.1 reverse | (geneCodeEnd+0 to geneEnd+0) | length=222

AGCAACACAGTATATTATTCAGTGGGAGGGACGTGATTTAATTATATTCTTTCTTCTCTT

TTCTTTTCTTCTTCCCCATTCGGCGTAGTTGTTGCTGGGGGAGAGGGAGAGTGAGAGAGA

ACGGAAGCCTCTTTGTCGAAGCTATTAAAACTACATGACGAAGGTTAAAAGTAAGGTTTT

TATTTTTCTACACTTCTCTCTTCTATTCCTTTTTCTCTCTTT

>Tb927.11.6190 | Trypanosoma brucei TREU927 | hypothetical protein, conserved | genomic | Tb927_11_v5.1 reverse | (geneCodeEnd+0 to geneEnd+0) | length=222

GTCCCCCTACTTTTCTCAGATTTTTTTTGCAACTGGTTTTCCCTTATGCTATTTGTTGAT

AAGAGGAATAACCTTTCGACCGCATCGTAAAAAAAATAAATAAATAAAATTTAAGATAAA

AAAGATATATAACCCCTTTTGGTGTTTGTATGGGAGTCGTGAGGTTGGGACATACACTAA

TGTTGCCACGGAATGGGTGCTACGTAATGATGGCACCTTCCG

>Tb927.9.11270 | Trypanosoma brucei TREU927 | T-complex protein 1, eta subunit, putative, t- complex protein 1 (eta subunit), putative (TCP-1-eta) | genomic | Tb927_09_v5.1 forward | (geneCodeEnd+0 to geneEnd+0) | length=223

ACGGAGAGTTCGTGGCGGGAATTTACTCGTGTTGTGGCTGCTCAACCATTTTAAGGACAA

TTAAGTGATCAAAAGGGAATGAAACGAAAGAGGAAAGGGTAGTGAACGAGAGGACTCGTG

TACAAGCGAAATTAAGAAGGAGAAACAAGGACCGAGAAAGATATAGATCTCTCTAAAAAA

TTTTCTAACACGAAATTGTGTGCAATTAAAATCTTTTTTACTG

>Tb927.11.13190 | Trypanosoma brucei TREU927 | elongation factor 1 gamma, putative | genomic | Tb927_11_v5.1 reverse | (geneCodeEnd+0 to geneEnd+0) | length=223

GTCTGGTGTCCAGTTGTATGCGGTGCGAAGGCGAGTTGACCGGGAACGGAGAATCTCCAC

AGTAATGTTACACCGTCGAACAGAGGATACAATGGCCCGAAGAACTGTAAGAGGTAATAA

TTTACTTTCCACGGAGTGCGTAGGTGAAATAATGAGGTCCTCTTTATGCTTTTTTAATTC

ATGCACAAAGAAGGAGATAAGTGTGACATAATACGCGTTGTTG

>Tb927.3.650 | Trypanosoma brucei TREU927 | hypothetical protein, conserved | genomic | Tb927_03_v5.1 reverse | (geneCodeEnd+0 to geneEnd+0) | length=223

ACAGTCGACATGGCTTTGTTTTTTCTTCTTTTCATTCAGTGCCACACAAGTTATGTGTGA

TGTGGAATGATTATGTGTGTGTGTATATGCCACAGTACTGTGAGGATATGAGGCGTCGCT

TAGCCTTTTCATTACACTTCTCCACACACTTTCTTCTTTGCTTGCTTGCGTTTCATCGAC

ATGTTATTTCAATTCATCCGTTTAATATACATCAGTCACATTT

>Tb927.9.8680 | Trypanosoma brucei TREU927 | cytochrome c oxidase assembly factor, putative | genomic | Tb927_09_v5.1 reverse | (geneCodeEnd+0 to geneEnd+0) | length=223

AGGCTAGGTGTTATTCACGTTCGATCTGAGAAACTTTATCGACACTGTAGTATATTAATG

GATGCAACTGTTCATCATTATCATACGCATATTACGTATGGGTGTTTACGCGTCACGTTT

ATCTCTTCCTACATGTCTGTTTGTTGTCCCTTTAGCTTTTTTTTTTGTTTGCCGCGCTTT

TGAGCGTAGGAATTTTGGGTGCCTTACGTGATGATGTATTTTT

>Tb927.11.12590 | Trypanosoma brucei TREU927 | Der1-like family, putative | genomic | Tb927_11_v5.1 forward | (geneCodeEnd+0 to geneEnd+0) | length=224

ACTGGACCCCCTCGCCTTCCCCCTCCTTTTTTCTTCCTCGCACGGGAACTGTGACTATTG

TACATCCGTGTGGTTAGTGTTGGGAATTTCCTGGGTATCCAAGCAACGCATGAGGAAAGT

GGTGTGGGAACAAAAATTTGTGGACAATTGCCCCAGCAACAGCCCACTCAACACATCTGT

TCCCTCTATGTTATCTTGCGCGGTTGCTTCGGGTCTTAAGTTGG

>Tb927.11.3790 | Trypanosoma brucei TREU927 | hypothetical protein, conserved | genomic | Tb927_11_v5.1 forward | (geneCodeEnd+0 to geneEnd+0) | length=224

ATCAAGGGTACAATATGAAAGGGTCTGGGGCCTGGCTGGAACAAACTACAACCAAGCGTG

TTGCATGAGTGTGCCTTAATCGTGTGTGATTCAATATGGCAATCAACGAAGGGTAGGGTA

GAAGAGGAATTGAAGTAATCATGCGCATTAATGTATTTTTAGTGCGCATTATGCGTGCTT

TTGTAGCCGCTATTCGATTCTCTCCGCTGTATGACAAACTTTTT

>Tb927.10.2630 | Trypanosoma brucei TREU927 | Regulator of Vps4 activity in the MVB pathway, putative | genomic | Tb927_10_v5.1 forward | (geneCodeEnd+0 to geneEnd+0) | length=224

AGCGATTGGTTGGGTGAGTTGACTTTTTTAGCTTCTGGGCACTCTTCTTCTCCTGCTACT

GGGAAGTGGAAAATAGCCAACCAACGTGGAGCGTTTTTGAAGAAAGTACCGTTGCGGTTA

AGGACAAGGGAGACCGACTGTTTTTAAATTTTGTCTCTTCTGCTCCGCCGAGTGTGGGCT

TGATTCCCTAGTGTAAGAGGAGACTGATGTGACGCCATTGCTGG

>Tb927.9.8190 | Trypanosoma brucei TREU927 | hypothetical protein, conserved | genomic | Tb927_09_v5.1 reverse | (geneCodeEnd+0 to geneEnd+0) | length=224

GTTTTTTTGTGGGTGCAGGTTGCATGGAGCGGGGGGGGGGGAGGGGCAGTTATTAGGGCT

TGATTCACTACCGGAATGTATAATATGCTGTTGGGATATATTTAGAATCGACACAGATTT

TTCGTGTGTAATTGAAGTTCACATTTAGTTGTGGGTTTTGTCGTCACGCCCAACCTTCAC

TCCCTCCCTCCATTTCATTTCCGTGCTCTCTTGGTCACGCGAAG

>Tb927.4.550 | Trypanosoma brucei TREU927 | Zinc knuckle, putative | genomic | Tb927_04_v5.1 reverse | (geneCodeEnd+0 to geneEnd+0) | length=225

AATAAACCGGCTGGCGTAATCGCATGTGCGCCAGGAACCGGACCCTCAAATGAAAAAAAA

GGAATTGTTTTGGTGCTTTGAATGGTTTTTTTTGTTTTCGTATAGTCGGACACTTTTTAT

TTGGCATTGTTGTTAGTGTGATTATCATTGGGCATATTTCACTCGTTGCTGCACGCACGT

TTCCTGTTATGCCTTCGGTTTTTTTCCCTATCCTTTTCTATTTTT

>Tb927.4.3370 | Trypanosoma brucei TREU927 | hypothetical protein, conserved | genomic | Tb927_04_v5.1 reverse | (geneCodeEnd+0 to geneEnd+0) | length=225

AGGAGGGCCTCTGCACTAGCGGCTTCCCTAGAGTTGGGAGTGGGGTGAGCGCCGCTCGCG

GATTGCCTGCACGATGGGAAGGAGAAAGGACACTATTATTATTATTATTCTTATCACTAT

CACCACTTTTATTTATTATTATTATCTTCAACAATTGACTCAGTTCGTGATTTGTGTGCG

TGTGCCTGAGGTGTGTAAGCCCCACTCTGTGGTGGCAAAGCGATT

>Tb927.11.6850 | Trypanosoma brucei TREU927 | Optic atrophy 3 protein (OPA3), putative | genomic | Tb927_11_v5.1 reverse | (geneCodeEnd+0 to geneEnd+0) | length=226

GTCGCTAGAAAAGGGGAGGATGTTCTGTTGTTTTTTTTTTCTTTTTCATTTCCCGTGTTT

CTCACACATTATTCCCTTTTGTTTCTCGGATCACTACCGGGGAAGGGGCGAAACTTACCT

GCTGGTGGAACTATTGGAAGTGTGCAAGTGAAAGCTGTGTATAACCTCATACATAGGGAG

GCAAGGATGTACCTTGCTCTCCTTCCTGTCTCACTGACCCCTCATG

>Tb927.9.6870 | Trypanosoma brucei TREU927 | RNA-binding protein, putative (RBSR1) | genomic | Tb927_09_v5.1 forward | (geneCodeEnd+0 to geneEnd+0) | length=227

AAATGTTGTACTTTCGGAAAAGCAATCCCAAAAGAAGTAACGATACGTGGCGAGATCCCC

CCATTTTTTTTCGTTGAAGAGTACTAACCGTATCAGTAAAGTCATGAATGAAAGGTGCGT

TGAAAATCCCTATTCTTCTTTGAGACATTCTGAGTTACCCTACTCCACCTCTGGTTTAAT

CATCAAACGTTGGCGGCTTATCGGTGGTTTTGTGTACGGGATTTCCC

>Tb927.7.7510 | Trypanosoma brucei TREU927 | hypothetical protein | genomic | Tb927_07_v5.1 forward | (geneCodeEnd+0 to geneEnd+0) | length=228

GTAATTTTTGCTACAATGCTTTTCCTTCACGGAAGATGTGGATGCTATATTATTAAAGAA

ACTTGTGGGGAGAGAATGTGGTATAGGTGTGTTTAATTCCTCTGCGTTTTTGGTGCTTCT

TTGCATGATCTTTTTTTTTTATTATCAAAGCGCCAACGTTGTAATAGGGGTTATAAAATA

AACTCCTCATCTTTATTACACCTCACAGAAATGCGATTTAATATTGTT

>Tb927.5.1170 | Trypanosoma brucei TREU927 | hypothetical protein, conserved | genomic | Tb927_05_v5.1 forward | (geneCodeEnd+0 to geneEnd+0) | length=228

GACGCGAACGAAAAAAAAAGGGGGAAGTGGAGTGTTACGGAGGGGAGGCTTTTTGCCTTT

GGTTAAGGTTTAGAACCTGAAGCCCTTCTACTGAGCTTGTTGATCCTTCTGTACTCGCGC

ACCCTTTATTGAGAGCTGCCGCAATTTCGTGTTGGTGCCTGTGCGTCTGCACACTGACTC

ACTACCCCTTTTGAGGTTGTTCAGTTCATTAGTCTTTTTCACTTTTGT

>Tb927.11.16450 | Trypanosoma brucei TREU927 | hypothetical protein, conserved | genomic | Tb927_11_v5.1 reverse | (geneCodeEnd+0 to geneEnd+0) | length=230

GCTTTGTCGCATGCGGAGCCGCACGGGGGCGCCACAGCCCCTGTGAAGCGCATTTGCAGA

AAGCCAAACGATGTAGTGACGATGACACATGCTCAAACATGTGCTTTGCGCTGTGCACTA

TAGTGTGCGCAGTTACGTGGGTGATGATTGTTAGATCTGGACTTGTGTTGGCGCTCGTGT

GTATGAAGACATCATTACCTAAAGTCGTATGCGGTCCGTTTAAATGTTTT

>Tb927.10.13140 | Trypanosoma brucei TREU927 | hypothetical protein, conserved | genomic | Tb927_10_v5.1 reverse | (geneCodeEnd+0 to geneEnd+0) | length=230

GCGATTATTCCTTTTTTTGTGTGTGTGTGTAGGGGGGGGGGTTAACGAACACACACTCAC

ACACATACACACACACAAGAAAAAAAAGGAATAATCGCTCGGTGCGTCAGTCCTGATGTG

GTGGCAGTTTCAAATACACAGTAGCAGGACCAGTTAAAGTCACTCTCGCGTTACCGTATG

TTGTTTCCTTTTAGAGGGAATAAATTTAATGCAGTATGATTTTCAACAGG

>Tb927.5.1740 | Trypanosoma brucei TREU927 | hypothetical protein | genomic | Tb927_05_v5.1 forward | (geneCodeEnd+0 to geneEnd+0) | length=230

ATGGCTCTGTATTGCAATTTGTTCTAGTGATGTCGTTTTGTTTTTTTTGTCTTTTAACTG

TTTTGGTTCTCCTTTCCATTTTTTCCATCTTACGGTATGGTTGGTGGTATGTAATTCTTT

TGAAAGGGCGCTCATTGATGTGTTATGAGTGTTGGCGTACAATTTGCCGTCTTGAGGTGT

GTTAAACCTCTTTATGTTTCTTTCCTCCTCCTTTCCCCGTCCTCCCGTTC

>Tb927.11.9450 | Trypanosoma brucei TREU927 | cyclophilin type peptidyl-prolyl cis-trans isomerase, putative (PPIase) | genomic | Tb927_11_v5.1 reverse | (geneCodeEnd+0 to geneEnd+0) | length=231

ATTATCGCGGTTTGTGTGCAACAGTTGAGTAATTGAGATAATATATTTTTTTCTCGCAGT

GAAGATCGTCGGGGGAACACACATACAGATGCTCTAATAAAAGGAAGAGGGATGAGTCTA

TGCTTTTCTCCCACCCCTCTAAGGGAAAGAAAGAGAAAAGAGCGAAAGGCAAAAGCGAAG

GCGTTTGTGTGCTACTTCATCTGGACTTGTTCCTCTACTTCTTTCGTTGTG

>Tb927.11.16860 | Trypanosoma brucei TREU927 | mitochondrial RNA binding protein (MRB8620) | genomic | Tb927_11_v5.1 reverse | (geneCodeEnd+0 to geneEnd+0) | length=231

GCGGACTACAGATTAGTATTGCAGTGTGAAACCGTTCTGGCTGTAGTGAGTTGCTGCACG

ACATGTTACGTCTCTTTTTTCCGTCTTTCGGATTGCGGCAAAACGCTTTTGTCTACTTCC

AAGATTAGTGCCCCGCGTGTGAGTGTTTACTTGCGACTGGAGTCGAAAGCATTCGTGGGT

GCGTCTGTGAGGAAGTGAGGAATGGGGTTGTGAGGAGGGGTGAAGAGTGAG

>Tb927.10.15510 | Trypanosoma brucei TREU927 | hypothetical protein, conserved | genomic | Tb927_10_v5.1 forward | (geneCodeEnd+0 to geneEnd+0) | length=232

ACTTCATCTGGTTCTTGTGGTGAGGGAAAGAAGTGTGGGCGCGATATGAAAGTAAACACA

TTTATATTTGCATAGGGAAGGTCGCATGGTATGGTGCGGATCCTCCTTTTGCTACTCGCA

CCCCCAACAAACTCCTAAATTTGGTTAAGGGTGCTCCCAAAACTTTCGTTATAAGCAAGG

AGTTCTAACAACATATTTAAGCTAATGGGTGTGGGGCTTTCTTGTACTCGTT

>Tb927.10.9050 | Trypanosoma brucei TREU927 | pseudouridine synthase TruD, putative, tRNA pseudouridine synthase TruD, putative (pus7) | genomic | Tb927_10_v5.1 forward | (geneCodeEnd+0 to geneEnd+0) | length=232

ATGACGCTGATGCGCATCGTATCCCTTTAGGGTAAAGCCTCTGGCTGCACCGCGGTTCTT

TAGGAATAAGGTAGCCACCAGTTGTTTCGGGTATATACACATGTTAAAAATTTTAAAAAA

GTTAAGCGCTGTGCGAGGTGCTGTAAGCACCCTTCGTCGGTTTCTTTACATCGTCTCCTG

CAGGAGTGGAAGCTCCTAAACTTTATGGAGTCAAGAGATCTCTTATCACACT

>Tb927.2.2590 | Trypanosoma brucei TREU927 | hypothetical protein, conserved | genomic | Tb927_02_v5.1 reverse | (geneCodeEnd+0 to geneEnd+0) | length=233

AATTACTGGACACTTGCTCATATGAAATATATGTTGGGTAGAAGAGCTGATGTGTTCAAC

CGTTCCAACTGTTCATTTGTGTTCCGTGTCATTGCTTGGAGGAGTTTTGAGAGACTAGTG

GTCAAACACCCGACCCTGTTGTTTCTACACAGGGTCAGGACATTGATGTCTCGTCACATC

AGATGGTCTGTGTTGTGACTTATTGTGGGTTTTTGGTCGTATCACCGCACTGG

>Tb927.11.9640 | Trypanosoma brucei TREU927 | glycyl-tRNA synthetase, putative | genomic | Tb927_11_v5.1 reverse | (geneCodeEnd+0 to geneEnd+0) | length=233

ACGTGTGTGTGTGGAGAGTTGTGTCACTTGCTGAGGTTTGATAATCGGGTGTATTTCTTA

TAATATGTTGTTTAAGAATTATTGGTGCGAGTGACTTGTCACTTTTAAGGTTAACGTTAT

TCCTAATGACACCCCGCCAACAGCTTGAAGAAGGTAATGTCTTTGCTCCAAAGTAGGAAG

TCACACAATATTAACAAAGACATGAATGGGCCACACTTACACCCTTCGCACCG

>Tb927.11.11270 | Trypanosoma brucei TREU927 | conserved protein | genomic | Tb927_11_v5.1 forward | (geneCodeEnd+0 to geneEnd+0) | length=233

AATGGGAGATGGGTATAAATTGGGTTAAAACGACACTGATGATGTCGAGGTTGGGCACAT

GCAACATATATACGCAGACGTTTATATCGTCCGATCCAGTCGTTTATGTGAGCACAGCTT

ACCAACTTTTTTTTAGCGAAAGTGTACCTTTTCTACCCGCTTTTGTGGTTAGAGTTAGTC

CCATATTGTTCTTGTGAGTGCATTTACTGTGTCGTTGTTGATCTGCTGGTTGT

>Tb927.10.2110 | Trypanosoma brucei TREU927 | elongation factor 1-alpha (TEF1) | genomic | Tb927_10_v5.1 forward | (geneCodeEnd+0 to geneEnd+0) | length=234

AACAATGGTCCCTACCGCGTCACAGCGTATGAAGGCCACATTGCAATTTAGCGTGGGACA

TTGCATAAATGACGGTGGTGGGGGTCACTGTTTGAGGGCGTGTCTTAAGCTGGATTGAAT

GATGTGGTGACGGATCCAAGGAGGTAGAAGAGGATGTAAGGGGTATGAAGGGAAGAGAAA

TGGGAAAAGAGATTAAATGATATTAATAGGAAAATGAAAATGATATTTTAGTGC

>Tb927.6.2610 | Trypanosoma brucei TREU927 | hypothetical protein, conserved | genomic | Tb927_06_v5.1 forward | (geneCodeEnd+0 to geneEnd+0) | length=234

ACAGCGGCATCCACATCCTGTTGTGGTCCCGCTCATATGATCTGTGTAACAAGGTGAAAA

CTTTTGGGGAAAGCTCCTTCAAAGGGAGGAACAGAGGGGCGTTTGCGTGTATATAAGACA

ATGAACTCAGCATTATCGTTACCGTTTTTCTTGCGTATCGTTCTTTCGCCAACTTAATGC

CTCATTATGTGACGCAGAAGATAGAATAAAGAAATTTGCGTTGCGAGACACCTG

>Tb927.4.1150 | Trypanosoma brucei TREU927 | hypothetical protein, conserved | genomic | Tb927_04_v5.1 reverse | (geneCodeEnd+0 to geneEnd+0) | length=234

ACTAACCGTTACTTCGCGTTGGCCGTTGGTAGCTTCCCCTTGTTTTATGAATTCACAGCG

TTAACAAGTCGTTATGGGTGGAGCTTGTATCACTGCTCACGGAACTCTTTCATTCTTTCT

AATGTTGTACATTGAATGGCGCGAACTACGATGCTTCCCGGTGGGGAAGACGCTGGGCCG

AACAGGTTATTTTTCTTCTTTCCTCCGTCGTGGTGATGTAAAACCTACATCTTT

>Tb927.8.5380 | Trypanosoma brucei TREU927 | ubiquitin fold modifier protein, putative (UFM1) | genomic | Tb927_08_v5.1 reverse | (geneCodeEnd+0 to geneEnd+0) | length=235

AGCCAATGCATAATCAAGATACAAAATAAATGCTATGAAGGAAAGGAGCCGGCCAAACAG

GCACGTATGCTCCCGGTGCGTCCAGCGTATTTCCACTGCTTATCGTGGGGAGGGGGCAAC

CCCTTTGGCCTTTCGTGTTCGTGCGTGTGGGTGTACGCTTGTTCCTTTTGCTGCGTAAAG

CAGTTGATGCGAGGTGGAAAAGCTTTTGTCCGTTTTTCTTATCTCGATCGAGCGG

>Tb927.6.2540 | Trypanosoma brucei TREU927 | DREV methyltransferase, putative | genomic | Tb927_06_v5.1 forward | (geneCodeEnd+0 to geneEnd+0) | length=235

AAGCTGCCGTAAAACACTATTATTCTGCATGTAACTGTGTTGCTTCTGATGGGGGCTCTG

ATGCGTTTCCCTCAAATCATATGGTGATCTAGCCTACAAAATGGGTGAAGTGTCTTGCCA

TCATCGTGTTCATCCTCTACCGCGTACGTTTACTTGTGTTGGGCATTTTTTCCCCCCCCC

CTTTGGTTACATTTGGTCTCTTTTTTTTTTAAGTTATTATTATAATTTTTTTTTT

>Tb927.7.190 | Trypanosoma brucei TREU927 | thimet oligopeptidase, putative (THOP1) | genomic | Tb927_07_v5.1 forward | (geneCodeEnd+0 to geneEnd+0) | length=236

AAACGTCGAAGCCTGATGGCAGACTATTTTTACATTTTTTTTTTCTTAATACCTGTCTAC

TTCCTTTTATTTTCAAGTATCAATCTGTCTGAATGTTGTAATGAACTTCTTTCTCCTCCT

CCCAAGCATGAAGATTATCGCTGGCATGTGTAGGATATATAAACTTCTATCGAACAGGAA

ATGTCCGTACACATATTTTTAATTTCCCCCGGAGTAACAAATACGTAATGACAGGG

>Tb927.8.1380 | Trypanosoma brucei TREU927 | hypothetical protein, conserved | genomic | Tb927_08_v5.1 forward | (geneCodeEnd+0 to geneEnd+0) | length=236

AAATATTGGTTCAATTTTGTTTCTTTTTTGACTTCACTAACTAACCCCTTTTCCACTTGA

TGAGGTGGGTTCAGGAGCTCAAACCAAGAAAGCCATCAACTGGAAACACGATTGGCGCAT

ATCATGAAGGTAATGTGTTTGAAAAAGATGGTAAATCGAAAAGCCATACACCTATCAAGT

TCGAGACGGACTGTCACCAGCTGGAGTTATAAATAACTCAATAGGCGCCAAAGTAG

>Tb927.8.3360 | Trypanosoma brucei TREU927 | hypothetical protein, conserved | genomic | Tb927_08_v5.1 forward | (geneCodeEnd+0 to geneEnd+0) | length=236

GCAGCAACACTTGAGGTAAGAATGAGAGGAACTTTCGCATAATTGCTGGTGGGTTTCCTT

TGCTTCGTGGGTATAACTGTGTGTATACAACTCTGTGCGTGTCCTTATCCTGGGCGATAA

TTCTTTCCGTTGTGTTTGCTCTCAAAATGTTGGAAAGGGTGCGTGGAATTGGGGAGTGGA

CGTTTTAATTTTGCTTCACATCTTTCAACCCCACCGAAGTGATTATTTAACACGTG

>Tb927.11.6500 | Trypanosoma brucei TREU927 | 40S ribosomal protein S21, putative | genomic | Tb927_11_v5.1 reverse | (geneCodeEnd+0 to geneEnd+0) | length=237

ATGCTTTTTAGGTTGGACCTGAACCATCATTTATTTGAGGTTTCCCAAAGGATCACCCCT

TTGTGCATATCTTCACATCTTTTCTGTGGCGCTCTCGCCATGTCTATTTGGCAAGGGCAA

AACAGCGGGATGCGAAACGCCGGGGGGAGACCAATAAGGAGAGCGATGCCCACCAAACAG

ATAAAATTTTTTCTCTCGGCTTAATTTCTTCTGGCAACAGCATTTTATTTTCACTTT

>Tb927.7.2620 | Trypanosoma brucei TREU927 | hypothetical protein, conserved | genomic | Tb927_07_v5.1 reverse | (geneCodeEnd+0 to geneEnd+0) | length=237

ATGTTGCCTTCCTTTTTTGTGCTTGATGCCTTCTTGCGTTGCAGCGTTCCGTGTCTCGAT

GACTTCTCCTTTGGCTTTTTTCTTTCACTTGTTACGCTAGTGTTCACTATCTACCGTCAT

CATCAAGTTGGGGCATGCTCGATTTCTCTCTCTAGACATATTGTTTACGAAGCAGAATAT

GTCCATTTACCGCTTGTCGTTTCGGCCGAATCGGCTATGTTTTGCTGCGTTTCAGTT

>Tb927.10.4760 | Trypanosoma brucei TREU927 | Integral membrane protein DUF106, putative | genomic | Tb927_10_v5.1 reverse | (geneCodeEnd+0 to geneEnd+0) | length=237

GTAGCAGCAGCAGTGTACTTCAATGGCTGTTTGAAGCCAAATTTCAGGAAAGGCGTATCG

CCCGCATTGTGGTTGTGTTGTTTATTTTCCTTGCTTTCATTGTTTTAATTTTGAACGATG

ATAGGAGATGAAGTAAAAGGGGATGAGAGCTGAGGGATAGGGTAGTCGAAGGAGGGAACG

GGTACTTCTAGTCCTGACAGAGGAAGGGCAGAAGGTTAATCAATACACATACTAAAG

>Tb927.11.570 | Trypanosoma brucei TREU927 | DnaJ domain containing protein, putative | genomic | Tb927_11_v5.1 reverse | (geneCodeEnd+0 to geneEnd+0) | length=238

ACTTTATGAGGGCAATGGGTTATCTTCCGGATGATTTGGTTTGTGGCGTATCCATTGCGG

CGGCTCACCGGGGATGAGACGTTTTTTTTTTGTGTACGTGTGTTCATGCATGGGTGGTGT

GGACCAGTTTCAACCGTGTCTGGGGTGCTAGGGTGGGGTGAAATCAGGCATATGCCAAAA

GAAATGACAAGAAATGATACTCCCCCCCCCCCAAACTATCGGTTGCCATGTGCTAGGG

>Tb927.10.15820 | Trypanosoma brucei TREU927 | hypothetical protein, conserved | genomic | Tb927_10_v5.1 forward | (geneCodeEnd+0 to geneEnd+0) | length=238

AATGAAGTGGAGGTGCAACCACTGCTAGCACTAAATGGGAGAGATGTCACACTGATGTGG

GGGACGTGGGGATGCCCACGGGGAAGAATAAGTTGCGTTGTTTGGTGGGGATGTTGGTCT

CTGATATCCTTATTGAGAGTTGCAACGCGGCCCAACCTGACAGCTAGAAGTACAGGGTCG

CGAGCGGCGTGTGTGTGTGTGTGTGTGTATGTGTATGCGTTGGGTGCTCATCACTGTG

>Tb927.4.4320 | Trypanosoma brucei TREU927 | divalent cation tolerance protein, putative | genomic | Tb927_04_v5.1 reverse | (geneCodeEnd+0 to geneEnd+0) | length=238

AATGTGCGTTGCAAAAAAAAAAAAACTGGAATGTGTAAACGCTGTTGTCTTTTGCACAAT

TACCGATGGGGTGTGTGCAGTCCTAGTCGTTCACGAACATTTAACTTCCGCTGTTCTCTG

CGGCAGAAGAAAAGAAGTTGGGAGAAGGGAATTGGTGAGTAAGTCCGGTGCGTCACATCC

TCAATTTGATATCATGTGTCAAGAGAAACTTATCTCGCCATTTTTTTTATTTGCGGTG

>Tb927.11.10080 | Trypanosoma brucei TREU927 | hypothetical protein, conserved | genomic | Tb927_11_v5.1 forward | (geneCodeEnd+0 to geneEnd+0) | length=239

GAAGGCTCCGTGGGTTTGTGTTATGTGTTTCTGTGCTTTCTTATCGTCGAGACAACTGTG

CGCTTGTATGTTTAGGTATATGTTTTCCGTGGCGTCTGCTAGTACACTTACACGCGTTCG

TACGTTGTTTTTAACGAGAGATGCGTTTATGGATGGGGTTGATGTTTAATTCCCTGGTTG

AATTGACGGAAGGAAAAGACGTTGACTCAGACGGCAATGCTTTTTACGTGCTCTTTTTT

>Tb927.8.7030 | Trypanosoma brucei TREU927 | hypothetical protein, conserved | genomic | Tb927_08_v5.1 forward | (geneCodeEnd+0 to geneEnd+0) | length=239

AGTGGTGCTACAGCGTCTATTTTTACAACTCTTCTAGCTTCGTCTCTTCTATATAGTGCC

GGCGGATATGTGTCTCAGTTCTTCCCTTCCTTCCTCACAACACCTTTCCCCTTTTTATAT

TTGCCTCTACACTGTTGCCATATGGCGAGTCTGTGGAGCTTACCGGGTTACTCTTGCTTA

TCTACGAAAAGTGGCATTTAGCTCTTTAACGTAAGTACCGCGCTTCCATGTAGTTGATT

>Tb927.11.7380 | Trypanosoma brucei TREU927 | glycerol-3-phosphate dehydrogenase (FAD-dependent), mitochondrial | genomic | Tb927_11_v5.1 forward | (geneCodeEnd+0 to geneEnd+0) | length=239

AAAGCCATGTAGGGGAAAGAGAAAAAAGGGAAAATTACCTTTCGGCATGATTGTGTGTGT

GTGTGTGTTATGGAGGAGGTGCGGGGGGCGATTTTGCGTGCGTGTGTGGGGGGTGATGGT

GGAATAATGAATAAATTGGTTAATTGTAAGAGGGGTTATCATACACTATCATCTTCACTC

TTTGTTTTTCGTTTGGTTGTTTCACTAACTCAATGGTGGAATGAGAAGGAAAAAGGGGG

>Tb927.11.12680 | Trypanosoma brucei TREU927 | prefoldin subunit 2, putative | genomic | Tb927_11_v5.1 forward | (geneCodeEnd+0 to geneEnd+0) | length=240

AGTGGTGTCCAGGGAAAGAGGTCGGCGGTGAGTCGTATAGAGTTGCGTCTGCATTTACGT

GACAAATGCATATATCATTCGAGAACCCACACATATACGCGTGCCGGCGAAGGGTTTGAG

CGAAAAGAGAGGGATGGGCTGCCGACGGAGGCCGGCGAGAAGGTGCCTGTGTGGCGACCC

CCTGACGGGTTCCCATCGCCGGTGACTTGGTCGGGGAGGAGCTCTCTTTCCTGTGGTTTG

>Tb927.7.2820 | Trypanosoma brucei TREU927 | histone H2A, putative | genomic | Tb927_07_v5.1 forward | (geneCodeEnd+0 to geneEnd+0) | length=240

GATAGAGTAACTCACAGTCGGTCTGGTGAATATCCCGCGACCTGGAAATAGTTAGATGCC

TATGCGGATCCCTCACTGCATCCCTTACGGGATTGTTGCGAGTGAGCCCGCAAACTGTTT

ACTTCACTCCCTTTTTTTTCTTGTTTTTTCTTGATTGTGTGTGTTGTTGGCACTTTTGTC

CACAAATCACATTCACCATTTTCTCAATAATTGTTGTGTGGCTCGGTTGTATTTATCGAC

>Tb927.10.8020 | Trypanosoma brucei TREU927 | serine peptidase clan SC, family S9A, putative, prolyl endopeptidase (POP) | genomic | Tb927_10_v5.1 reverse | (geneCodeEnd+0 to geneEnd+0) | length=240

ACGGTGGGGCGTGTTCACTCACCGAATGCGCTTCACATGTTGCTTTCTTGCTCATCATTA

CACCTCTCGATTTTGTTTTTATGTGAGTGTTGTGTTCAGCTCTCCTTGGTCTAAACTGAT

AGCGCATCTCTTTATGGTGGTTTCAACCACCTCTGATGTTGTAGTAAAGATTAAAAAAAG

CAACTGCTGTCACTGACCAAATGAATAGGGGAGGATGTGGAGACTGATTGTGGGTTATTC

>Tb927.10.12210 | Trypanosoma brucei TREU927 | ribulose-5-phosphate 3-epimerase, putative | genomic | Tb927_10_v5.1 forward | (geneCodeEnd+0 to geneEnd+0) | length=240

GTTGGCAGTGGGCGAGTAACAATCATGCATTTTAGGATAAAACGAAGCTCGCGGGGAGAT

TAGTGAGAGTAGGGACTACTTAACTACAGAGAGCGTTTACGTATTATCGGTGTCTAAGGC

GATACGTGTTTATTGATGAATTATTTATATGTATGAGCGAACGAATGTTAGCGTTTCTGT

AGATATATTAACACTTAACAACCAAACCGAAGCGCGTCCTTTGGGTAAAGTATCATCATG

>Tb927.1.1120 | Trypanosoma brucei TREU927 | Ribosomal RNA-processing protein 8, putative (RRP8) | genomic | Tb927_01_v5.1 forward | (geneCodeEnd+0 to geneEnd+0) | length=240

ATAGGTACCGGTTCGTTCGTGACAACTGCGGTTTTTGCCGTGCGGAAACAAATTAAGCGG

AAGATAAATAAGTATAGTACAGTGGGGGGGCAGCCAAAGAAAGCGTTTAAAAATCACTAC

ACTGGTGATTCGGAAGGGTTGGAGACGTATATATATATATATGTGTGTGTGTGTGTGTGT

CTGTATAAGTACCTCCTTTCCTCACCCTGCTTCTCTGAGTCCTACTTGCCCCCTATTTTT

>Tb927.7.1490 | Trypanosoma brucei TREU927 | hypothetical protein, conserved | genomic | Tb927_07_v5.1 forward | (geneCodeEnd+0 to geneEnd+0) | length=240

AAGGCGAAGGTAGTTGCGGAGGGGAGGGAAACATTGCGCAACCTCCTCATTTCTTCGGGC

AATGCGGCAGCATTGCGAGAGTTTGTGACAGGTGAAATTGCGCCTTACATTCATCTGGGC

GCGTGACTGGACGTGACACAGATAATTCCAAGGGGCAAACATTCGGAGGAACACGTTGTG

TGGGTGACGGGTGAAACGAGGCACTGGTTCTCATTTAATTTTTTTCGTTTTGTGTTGGAG

>Tb927.6.4010 | Trypanosoma brucei TREU927 | Translation machinery associated TMA7, putative | genomic | Tb927_06_v5.1 reverse | (geneCodeEnd+0 to geneEnd+0) | length=241

AACGGTCCGTTTAGGCCGGGGAAGGGGGCTGCCACTTCAGTTGTCGCTCAATCACTGAGA

GGGTGAGAGGAGGAAAAACAAAAAACAAAAACAACCCTCCCGTCGTCTTTTATCAGGAGT

GTGCAGCGGAATGTGTCAATGTTGAGTGACAAATGCAGCCACACTTTTTTTTTCCTTTTA

TTTTCTCCTCGTTTTCACTTGTTGTCAACTTATTGGTCATTTTGTTTAGAGGTCCGTGTT

T

>Tb927.10.750 | Trypanosoma brucei TREU927 | hypothetical protein, conserved | genomic | Tb927_10_v5.1 forward | (geneCodeEnd+0 to geneEnd+0) | length=241

AGGCAAAGCGAAGGAAGTGATGTGGTAGTGAATTTTATATTTTCCTGCATGGTTGGCTCC

TCCTTAACTGAACTTCTTCTTCTTCATATTTTTACACATTATGCAGTCATGTCTTATGTG

TAGTTCTTTCAGCTGCTTTTATATGGACTCAAATTCACAACCGCATCGCTTTGTTTCTCG

CTTTCCCCGTGCGTTTTGGTGGTCGCTGCTTGTAACTTATTTGATGTGTTTTGTTCCCTT

C

>Tb927.10.7850 | Trypanosoma brucei TREU927 | protein-L-isoaspartate, putative | genomic | Tb927_10_v5.1 reverse | (geneCodeEnd+0 to geneEnd+0) | length=242

GTTTGTTGGAACTGCGCTGTCGTCGTTTTTTGTTCTTTTCTTCTTTTTTTCGCTCATACT

TTCACTTAGTGTTGTTCTTCTTTCTTTCCAAAGTGACCCCCTCCCTCCTCCCTCCTTCTT

TTGATTTTTCTTACCTGTTGCTTACACAAACGACTACCCATATTGGCAAACATGATTAAG

GGGGGTCTGCAGATTTCCACGATTTGAGTTAAGTACAAGTTTTCAGTTTTGCGCAGTTGT

CG

>Tb927.11.2330 | Trypanosoma brucei TREU927 | molybdopterin synthase sulphurylase protein, putative | genomic | Tb927_11_v5.1 forward | (geneCodeEnd+0 to geneEnd+0) | length=242

AAATCCATTGATTTACCTATTAAGGTAATCGGACGTCGTGCCCCCACATCTGGTAACGCA

ACACATATCACATCTTTGACAAAAAAAAGAAGGGAACATAGCCCGCCAACAAGCATTTTT

GTCTCTCCCCTAAAATCAACCGAACTTGTTCTTTTCACTTGCTCCCTTCTGCATGGGATT

GCTTTCGTGCCTGGCGTGCACCCTGAAAATGGATGGGAAACGAAGTATAAAAGTAAGTTT

AT

>Tb927.8.4770 | Trypanosoma brucei TREU927 | small GTP-binding protein Rab18 (TbRAB18) | genomic | Tb927_08_v5.1 reverse | (geneCodeEnd+0 to geneEnd+0) | length=243

GGCGGCGCAGGCATGCTGCTTGGCGGATTGCCATCTCGTTTTCTCGCTGTTTCTTTCTTT

CGCGCGTGACATTTGCAATACAAACCAACTTTAGGGGGTTGTTGCGCGCTTGAACCCCAG

TTGGTAGTAATTTGTGGAGAATGTGAGATCAGTTTCTTTCTGTCGTTGCTTCGTCAAGAT

GTGGGTGTTGCTCCGTAACCGCTGTGTAAGCATGCGTTACGTGCATGTATGAAATGCTCC

CGC

>Tb927.9.1720 | Trypanosoma brucei TREU927 | peroxisomal membrane protein 4, putative | genomic | Tb927_09_v5.1 reverse | (geneCodeEnd+0 to geneEnd+0) | length=243

AACAGTGGCTGTGAGGAAGCAAAGGGAAAGAAAACAATGAAGCCACAGTCCACGTATAAT

ATTTGGTGCGGTTTATACCAGTTCTTTATGTGTGTATGTTTAAAGGCTACACAACTTTGG

TGTGTCATCGTGGTTTGCATCCATTCAGTGAGTAATATGGTCAGGGGTTGCCTCCCATTT

TGATGCTGTGCTAATGCGTGGGGAGTCACTACCAAGTTGACGCTCGCCGTGACAAGTTTC

CTG

>Tb927.9.12030 | Trypanosoma brucei TREU927 | hypothetical protein, conserved | genomic | Tb927_09_v5.1 forward | (geneCodeEnd+0 to geneEnd+0) | length=243

GTATTAACTTTTCATTATTTATTACGGGTCTTATCACTTATGTGACAGACGCTATTGTTA

GCGCATGTTGCGATTTCAGATGAGTGACGCTCCAGTTGGCGAAGAGGTGCGTGTTATTTA

CTCCAACGTATACTGTTACCGTTGTTTATTACTCGTTGAGCACATTTGACGCGAACTGTT

TCTGTGCAAAAATGCCTATCTCTCCTTTGTGCTCGACTGTTTTTTCTTCTTCCTTCTCAC

CGC

>Tb927.8.5230 | Trypanosoma brucei TREU927 | cyclophilin-type peptidyl-prolyl cis-trans isomerase, putative, PPIase, putative, rotamase, putative, cyclophilin, putative (PPIase) | genomic | Tb927_08_v5.1 forward | (geneCodeEnd+0 to geneEnd+0) | length=244

GTTCGGTTGAAAGGGATGCTCTGATTTTTCCATAATTGCAGGATATCACTTCCGTGTCTC

CCTTCGTCCTGACTGTGACACTGTGGCGCGTTGGCTTCACCGCTCAGGGTGCGGGGAAAA

TAGAATGTAACAAGCGGAAACATTGTGTATGTACTGTCGGTGTTTATCCACCATGGCTTG

ATTTCTAATGTACAGAGGCCCCTGTGGGTGGGTTGCAAAAGCAATTAACTATAGGCAAAG

CTTG

>Tb927.10.4500 | Trypanosoma brucei TREU927 | mRNA capping methyltransferase, putative | genomic | Tb927_10_v5.1 reverse | (geneCodeEnd+0 to geneEnd+0) | length=245

AGGAGTACTTATCTCCCCGTTTTCTTCCTGTGGCGGCACTTGCCTACTTAAGGTTGTCAA

CATTCGGTGGGCGCTCAAATACATACGGGGCCCTCGTGGAACCAACTACGTGCTTAAGAG

AGAAGGTTTATCCTCTGTTGTTTTCTTCACCCCCTTCCTTCTTTTCTTCTTTCCTTCTCT

GAGGGGACATTATGTAATGGAAGTTCGTCTTCTCTATCTGCTGATTATTGTGTTTCTGTC

TTTTC

>Tb927.10.11520 | Trypanosoma brucei TREU927 | hypothetical protein, conserved | genomic | Tb927_10_v5.1 forward | (geneCodeEnd+0 to geneEnd+0) | length=245

GTGCGAATGCCCCCATGAAGGACGTGTGCGCGTACGTTGCTGTCGCGGAGAAATAAGAGA

GCGATAGTGGCGAGGGGGCGTGTAGTTGTGTTGACGGTGTCTAAGCCGAAGGTGACGAAT

AGCTGTTTCCAATCAGTTGTTCGAACGGCCGTGCTTCGGTTCCCGTTGTTCTATGAAAAG

GTAATAGGTGGATTTTGATTCACTTTGACTAAACTCTTGGCTCGTTATTAACGTTTTCTC

TGTTC

>Tb927.2.5850 | Trypanosoma brucei TREU927 | small nuclear ribonucleoprotein SmD2 (Sm-D2) | genomic | Tb927_02_v5.1 forward | (geneCodeEnd+0 to geneEnd+0) | length=246

GTGGCACTGCGGCACTTGTGGGGGCGAAAATACAATACGCAGCAGCGCAGTTGCGGGAAA

ACGATTGTATTGAACATGGTTGTTTTTGAGTTTCTTGTTGTCGCTGCTTCGTTGTTCATT

TTTTCTTTTTCGGTGTGCTCGGCTATTCGCACCGCATTCCCCCCACAGTGTTAAGAAGCC

GTGGGGGCCGTTTAGGATGCGGTGTCAAAATTTATTGTAAAAGCGGTGCGTACCTGTGTA

AGAGAG

>Tb927.3.3570 | Trypanosoma brucei TREU927 | hypothetical protein, conserved | genomic | Tb927_03_v5.1 forward | (geneCodeEnd+0 to geneEnd+0) | length=246

AGGATGTAATGCGAATGTCGTTGTTAGTATCGTGGGCAGCGATGCCGATTCTTCGAGAAA

TTACCACGGTTGTGTGGCAGTGGGTACGTGGCCACAGTTTCTGTTTTGATTTGTACAGTG

GCAGCAGTTCATTCAGCGTCCCTGCAAAAGTAAGGTGTGCTCACATGCATGTCTGTATAT

ATATATATATATAGACACATATGTGTGTATGTGTGCGTGCGAGTGTGACTAAACGCAGTG

ACGGGG

>Tb927.9.10370 | Trypanosoma brucei TREU927 | TAX-1 | genomic | Tb927_09_v5.1 reverse | (geneCodeEnd+0 to geneEnd+0) | length=247

GGATTTCCTGCTGTGCGCACCCGACTAGCCACAGCAATGCCCACATGCAATTTAAGCGTT

CCTAAGAACCCTTTGGACGCTGGGGAAGTGCAGTGGGTGTCGAGGAGGGGCGGTGCACCC

CATGTTTATTAGAGTCGAGTGGTGATGAAGTACGAGAAGTAGGGGGAGAGGGGAGGAACA

ACATCTTTTCCCCTAAAATGTTGTAGACGCCTTTGTCATTTTTGTTTGTAATATATTGAT

TTTGGTC

>Tb927.1.3670 | Trypanosoma brucei TREU927 | expression site-associated gene 8 (ESAG8) protein, putative | genomic | Tb927_01_v5.1 forward | (geneCodeEnd+0 to geneEnd+0) | length=247

ACAAAACACCGAGCTGATCCTGTGACAATTTAACGATCATCAAAAGAGTCGAAAATAGGG

GGAGGGGCGCAAGCCGCCGTCTGCTTTTTTTAAAAAAAAACACACAACTTTGCTATCAAA

AGAGCGAATTCCGGTCGACATCATTCGCTGCTGCATTCGAACTAGAAAAAACGTCCTCAG

AACAAACGGCTCAGTCGAGGACACGCTACCTTGGCATGAACCTAATAAGTGATACCTTTA

TCAGTGT

>Tb927.10.1250 | Trypanosoma brucei TREU927 | Aminoacyl-tRNA editing domain containing protein, putative | genomic | Tb927_10_v5.1 reverse | (geneCodeEnd+0 to geneEnd+0) | length=248

AAACCAAGAAGTCGCTGCTTCTTTCAAAGCAGTAAACGGGCGTACACGAACGTCCGCCTA

CGGGCTACCTGATGGATGAGAGATGGTAGTGGGAAAATAGTGAAACATTGACAATGGGGA

AGGGTATGATGCCACTCCTCACTTCTCCCGTACACCAGTGTGGGCGCACAGCTCGGTTCA

CTTATTTGTTGTTGTGTGTTCCGTGGTTAGCGATTCCGGATGTGTAGCGATAATAATACA

TTCCTAAC

>Tb927.8.5490 | Trypanosoma brucei TREU927 | Nucleolar protein,Nop52, putative | genomic | Tb927_08_v5.1 forward | (geneCodeEnd+0 to geneEnd+0) | length=248

GCCAATAACTTGCCCGAGGGGGAGGGACACATTTTCCCGAGGTGGCAGGCTCAGTTCACT

GACCTCCACCAAGTCGGCTATTGTTCGCACTGCTGTCTGTGTGTGAGGGTGTTATTTATG

CTGTGATCGCGACTCGTTTAACGCCACCGGCGCTTTGCACTATCGTTTTAAACGATGAGG

TGCAATGTTTAGCTTAGTGAAAGAATAATGATAACGTCAAATCTGCTCTAAAAAAAGTAC

TAGGCTAT

>Tb927.9.2120 | Trypanosoma brucei TREU927 | hypothetical protein, conserved | genomic | Tb927_09_v5.1 reverse | (geneCodeEnd+0 to geneEnd+0) | length=248

GTCAGTGAGGAATCCAGAGGACTACGTTGTGGCGCGCAAAAATTGCGGTGTAGCGGCTGT

TATGTGTTTTCAGGTCCCTTTGGAGCACAACGCGGGGCCAGCTTGGGTGCTCCAAGCATG

CATTTCCGTTGTGTGTAAGAATTGGCGATTAAACCTAACGTAAGCAAGTAAACTCTCAAT

GAGAGGATGGTGAAGGGGGAAGCGAAAATAATGCCTTATGCTTTCTCGTACGTGATTCCC

TACTTCCT

>Tb927.8.4890 | Trypanosoma brucei TREU927 | endoplasmic reticulum oxidoreductin, putative, pol-associated gene 1 | genomic | Tb927_08_v5.1 reverse | (geneCodeEnd+0 to geneEnd+0) | length=249

ATTGTCTGCTTGCACAAAGTTGGTGGGGTAGGACGTTCAATTTAAAGCCTTATATAGTGA

CGAGGTGCGAAACGGGGGAGTAAATCTCTGGTGACTATTAAGAGAACACACTACCTGCAC

ATTGAAGGGAATGACTACACTAATATCCACTTACTGGGGAACAAATCAAAAAAAAAAAAG

AAAGTCAGATTTACTTTGTTAGTGATTACTTTGCGCAGTGGTGCTCTACATCGTATAAAG

AGATTTTAG

>Tb927.6.3300 | Trypanosoma brucei TREU927 | GPI alpha-mannosyltransferase I (GPI14) | genomic | Tb927_06_v5.1 forward | (geneCodeEnd+0 to geneEnd+0) | length=249

GCGCGGTGTTGTCGATGTCTTACAGTTGGCTTGCAAGTGGGTGTTTGCTTCGCGGTGGAT

TGGTTTAACCCGTAAGAGAGTACTCAACATCCCTTCCCCAACCTGCGTTTTCTCATTTCT

TTTTTTTTTTGGAGTTTTTTGTCACATTGAAATATAAGGAAAGCGTCGACCGTATTACAT

ACCCGTAATTCCTTTAACACCACATTCGTCCACGTGTCCACGTCACCCAAACCTTGTTAC

TTGAAACCG

>Tb927.9.13300 | Trypanosoma brucei TREU927 | Ring finger domain containing protein, putative | genomic | Tb927_09_v5.1 reverse | (geneCodeEnd+0 to geneEnd+0) | length=249

AAGTTGCCCCCCCCGTCGAGGAAAAAGGGTAGCGGCTGTGTGTCTTATTAGTTGAAATGT

TTTGCACTCGATTCTCATCTCTTCATTTTTACATGATGTTCAGTGAAGGAGTCGGACAAT

ACCAGCTCATTTTCACAATTCTCGTCTCCTTTTAATTCGTTCGTCCTCCCCTTAACATCC

ACCGGGCCACGTGCGTGTGCCCCGTGTCACGGTATGGGGATCCATCTGTACATATGCTTG

TCTGTTATT

>Tb927.4.2450 | Trypanosoma brucei TREU927 | thioredoxin, putative | genomic | Tb927_04_v5.1 forward | (geneCodeEnd+0 to geneEnd+0) | length=249

ACTTGTTGGAATGGAATGCATCGTAACGGTGCCACCGGATTTGAGTGACGGTGCCAGTTG

CTGCAAACTTTGGAACAGGTGCGGTTGTGGTGTATCTTCTTGTGCGCTAGACTGTCCATG

ATGGGCGCAAGAGAAATCGAACAGGGGACAGCCGTGGGGAACAATAAATCAAGACGAAAA

AAAAGGAACCGAGTCGAAAGCGTTGAACCTGCAACGATTACTTCTTGGGAGTAAACGAAG

CATGCGGTT

>Tb927.9.9630 | Trypanosoma brucei TREU927 | TPR repeat/Tetratricopeptide repeat, putative | genomic | Tb927_09_v5.1 reverse | (geneCodeEnd+0 to geneEnd+0) | length=250

ATGGCTTTGTGCACCATTTACTTTCTTTGTAGCCATCTCCTGACTTCTGCGTTCCGCTGC

TGTGGTTACATTTCATTTTCAAGTTATACCAGCATCTTGGTGTGTTATCAATGACTCCTG

CCAATGGGTAGTGAGGAAATTCCTGTGGCAGCACTGTCTTCCAAAGGTACGGGCACTGTC

TCATCGTCATAATTAGCGCCTTCACGAATGAGTACTACAGTTAGTCCCTCGGTTCTCCGT

TCTCCTCCAC

>Tb927.4.4350 | Trypanosoma brucei TREU927 | emp24/gp25L/p24 family/GOLD, putative | genomic | Tb927_04_v5.1 reverse | (geneCodeEnd+0 to geneEnd+0) | length=250

AATCATTTGTTGTTCGGTACGTAGCGGGACGAAATAAATGTTGCGCATTGTTTTGGGGGA

GCGAAGTAGGGGTTAGGGGGGGGGTGCTTTTCTCTTTTTCACTCTGCCGTGTCTGCAAGA

CCACTTACCGTTCATCTTCACCTGAAATGAGGGCGGGGAAGGGGTCGGCTTGAACCGTTA

ATAGCTGATTGAGTGAGCGTGAGCGAGTATAACATGTGGGAAGAGGCAAATTATAACGCA

CGACAAGCTG

>Tb927.3.5150 | Trypanosoma brucei TREU927 | exonuclease, putative | genomic | Tb927_03_v5.1 forward | (geneCodeEnd+0 to geneEnd+0) | length=251

ACGTGAAAAGAAGGGGAAGACACGGGGAAATGCATGAGATTGACCCTTCAGAAACAACAG

ACAGCAACAGATGACAACAGATGAAGGCGCCGGCAGCGAATGCAACGATCCCTACTCGTG

CGTTACTGTGGCGTAGGGGCTGGCGGGGGTAGGGTTCCTAACATTGGGTATTTGTTAATT

GTGTTCAGATATCACTGGTTTTCTTTTTTGTTTACACCAGAACCTTTTGCTGTTCATCAT

CGTTACAGTTC

>Tb927.3.3500 | Trypanosoma brucei TREU927 | hypothetical protein, conserved | genomic | Tb927_03_v5.1 forward | (geneCodeEnd+0 to geneEnd+0) | length=251

AGGTTTTTTTAATTCTTACAGGGTGTAATGGGGAATGGGGACAAGGATCGCAAGATGAGG

GAACGAGGGGAATTAGAGGCACCGCAGTAACAACGGGTTCCATAATGTTCGGAATGCGAG

GTACCTTGGATTCGCTTCACTGGTTTCATCTGAACAATGGAGGCGCTTCGAAGCCAGTGT

GGGTGTTGCGTTTTTCTTTTTTTTCCTTTGTGCCATGCCAAAGACCTGTGCTACATACCG

CTCTTTCATGC

>Tb927.11.13960 | Trypanosoma brucei TREU927 | U6 snRNA-associated Sm-like protein LSm4p (TbLSm4) | genomic | Tb927_11_v5.1 forward | (geneCodeEnd+0 to geneEnd+0) | length=252

AAGCATTTTCCCGTCGTGGTACTTACGGGATCGATCCCCAATCAACTTCATTAGCGTCCG

CCGTGCCCATGAAAACCCAAGCGGCGTCTCCCCATATCTTCAGCAGTCGCGTATTTTGAA

TTCTCAGTGCGCTCAGTGTGGTGAGGTATCGGTTGTGGAGAGACAGAAGCCATAGGAACG

AGAGGTATGCCTTCGTATTGTATTGTTCTTTTTCCCCCTTGAACATCCAGTGTGTACTAT

ACTGGGTGGTGC

>Tb927.5.2670 | Trypanosoma brucei TREU927 | hypothetical protein, conserved | genomic | Tb927_05_v5.1 forward | (geneCodeEnd+0 to geneEnd+0) | length=252

AGCACGTGAGGGCACATACATGGTGAGCACGTTACGAAGCAGTACGGGGCCGTACCGAAT

GTGATTTGGTGTTGTTATCTTTTTCTCAAGGTTGTTCATGCAAAGGGTAACGCGGGTTCG

CTCATAATACCGTTACCACACTGTTCCTGTTGTGGCTGACCAACCAGTGACGGCGTCCCT

TTTTTGCTTGGGATGTTTACATATCGTAAGGAGTGGGCATCTCCTCTTCGCTCGTTGCAC

GTGTTCGTTACG

>Tb927.8.4810 | Trypanosoma brucei TREU927 | prohibitin 1 (PHB1) | genomic | Tb927_08_v5.1 reverse | (geneCodeEnd+0 to geneEnd+0) | length=252

AACAGAAGGAAGGGATTAAATAATAAGATTGAACGGAAACAAGGTGTGTGCGTGTGGGTG

GGCGGGTGAAACGCCGTTCCCTTCGTCTGTGGTGGAGCTTACTGAGTAGTATTAAAGTAT

CGTCCGCATTTTCTCCATTTTCTTCGTTACTTAAGACTGTGCATATTTACACGCGCGACG

GAGGAGCGGGTACGCCCTCTCTTGTTGTGAACGGTGCTAAAGGGAAAGCGTCTGCGGTGC

TTGTGCTCGACT

>Tb927.7.4800 | Trypanosoma brucei TREU927 | hypothetical protein, conserved | genomic | Tb927_07_v5.1 reverse | (geneCodeEnd+0 to geneEnd+0) | length=255

GCCCGTTTGGTATATTTTGGTATTGTGTGTGCGAGTGTTGTGTGGCATTAAAAGTGTGCC

ATGTGAGGGTGTGTGTCGCCTCACTTGGTTCACCTATGCGTCTCTGAAGTTTCCATTGGC

AGTATCCGGTACATTTTCTGAGAGTTCGTGATGTATACGTGCTTGCGTGAATTTGTTATA

CAATACACCATCGTACTGGAAAAGCAGAGCGGTCGGGAAAGACCCGAATAAAGGTTCACA

TTGTATTTTTTTTTT

>Tb927.6.1420 | Trypanosoma brucei TREU927 | Uncharacterised protein family (UPF0220), putative | genomic | Tb927_06_v5.1 reverse | (geneCodeEnd+0 to geneEnd+0) | length=256

AAAGTGACACAACTGTTGAAAACATCAAGTGACCTCGGGTGCTAGTTAGTGTTTGTGGAT

TGGGTGTGGAGGTATTGACAAGCGGAAGGATGTTTTCCCCGCTCATATTGTTGCTGTTGC

GTAGCTGCTCCATTGGAGTGGAGAAAGGAGGGAATGAAGAAGGTGAAGACGATCAGTTGT

GACCGGTTGTGAACTCTCAACTTTTCCATGCAGGGGCATTTGTGCGCCCACACATGTGAA

CGGAGGGGTTTCTTTG

>Tb927.10.11300 | Trypanosoma brucei TREU927 | paraflagellar rod component, putative (PFC16) | genomic | Tb927_10_v5.1 forward | (geneCodeEnd+0 to geneEnd+0) | length=256

ATATTTTGCTCCTTTCGCTCCCCTATTTTTTACTCAAAGAAAATACGAGTCAAAAATGAT

GAAAGGGGAAAGGAAAAGCAACAAAGAGATTGAAGAAAAGTCAATGTATTTATACATGTT

ATGTGGGATATTATATGGTAGGGAAGTGATGTATTGAGGACGCTTTTTTTTTCTTTGTTT

TAATGAGGAGCTAAAAAGATAGAGAGAAAAGAAAAAAAGGAGGAATTGCCTTCGTTAGCT

CAATTGGGCATGATTT

>Tb927.10.13370 | Trypanosoma brucei TREU927 | Dynein light chain Tctex-type, putative | genomic | Tb927_10_v5.1 reverse | (geneCodeEnd+0 to geneEnd+0) | length=257

ACTGGGAATGAGGTGTTAACACGGTACGGAAGGCAACACAATCTACATGGGAGGGGAGCA

CGAGACGCAGGTTTCCTATGAAGTGGAAAGTGCTGTTATAGAGCTAGAGGTGTGCAACCA

CTGCGCTGCCGCTGTGCCGAAGGGGTTGCTCCGTGGGCCATACTGATGTAAACAAAAATG

AGTCAAACATAGAGGAGACACAAGTGGTTAGTCAACGTTCACTTTCCATACGTCACCTTC

GCGGATAGCGGTACCTC

>Tb927.11.12960 | Trypanosoma brucei TREU927 | hypothetical protein, conserved | genomic | Tb927_11_v5.1 reverse | (geneCodeEnd+0 to geneEnd+0) | length=257

ACGGGGAGGCCGGTGGTGTTGTTTGTTCGTTTGTTTGTTTTCTTTTTCTTTTTTCACCTG

GTCTGTCTTGTCATTGAGGAGCCGGAGCACACATCATTTGGAGGAACGGGGAGAGGGGAT

GAGCTTATGAAATAATACACCCTTTAAAGATAATTACCCTTCTCTTCCGGGTATTGCTGT

CAGAGGGGAAGTATCCGTGTTTTGGAAGTTGTATGTAGTCCGTCGCAAGCGTCCACCGAA

AGACATTATAAACCTTT

>Tb927.9.9750 | Trypanosoma brucei TREU927 | membrane- trafficking protein, putative | genomic | Tb927_09_v5.1 reverse | (geneCodeEnd+0 to geneEnd+0) | length=257

GAAGCGGAATGGAAGGTTGTGAATGGCGATCGAGTGGATTAAAAAAAAGTGAGAAACAGC

AGCGCCGGCCATGAAGTGACGTCATTTGCACGTTACCGGTATATTCATTGAGGCGGGGGG

GGAGGGGTATAGCATTATATTTTTGCTGAAGGAAGAATCATTTTATATCCCTATTACGTT

TGAATACCAACGAAGGGTAATTCAGTGGGTTTTTGTGCCTCAGTGAGTGGGCTTGGTTAC

AGTTACGTCCTATCCTT

>Tb927.3.1970 | Trypanosoma brucei TREU927 | DNA / pantothenate metabolism flavoprotein, putative | genomic | Tb927_03_v5.1 reverse | (geneCodeEnd+0 to geneEnd+0) | length=257

AGCTAACCGTACACGTGTATGTGTGTGTGTGTGTGTGTGTGATTGTATGTGCCGTAGTTT

TGTTGGCTAGAGATAAAGGAGGCAAGATATCTGTGGACCCCATATTTTGGGGTGTGTCGT

TTCGCAGAAAGTTGTTGATTTCTGCCACAATAACTCGACGTGAGTTGGTGATATCTTGCG

GGAGGTCTCCGACACATCGAGCGGTGTCATGGGGTGCTATGATTGTTATCGGTAATGCTC

ACTCTCTCTTGTTTTTC

>Tb927.11.5220 | Trypanosoma brucei TREU927 | chaperone protein DNAj, putative | genomic | Tb927_11_v5.1 forward | (geneCodeEnd+0 to geneEnd+0) | length=257

ACGAAACCAGTCATTTTTAGCGTACGTTTCTACGTCTTTTCCATTTTGTTGTAACCTCTT

CTTCTTTTCTTCACTTCCGAGGCTCGCGGTGGAATGAGGTACAGATACTTTTGACATTTT

TTAAAAAAAATGACAACACAGCGGATTAACGAGTGACCTTTCGGAATTCTTTGATATTGT

AAGTAATTTGTGTGCATAGATGGTATTTTGTATCGTTACCTCTCCCATGGTTCGTTTCCT

TCTCTTACAGTCGTGCG

>Tb927.9.15930 | Trypanosoma brucei TREU927 | small GTPase, putative, GTP-binding protein, putative (RAB1B) | genomic | Tb927_09_v5.1 forward | (geneCodeEnd+0 to geneEnd+0) | length=257

AGATGAGCTTACCATAGCCTTGTGTGAATGGTGCGTTAACAAAGGGTCTCGCCCCAATAT

CCAGTCAGTGAGTTAACTTAGGTGTAATGTGCATGTGCAAACGCAGTCTTTGATTTCTTT

TATTGATGTTCCTGTGCTTCTCCTCCCCTCAAAATAAAATAAAATAAAAGGAAAGCTGAC

GATGCCCTTAGGGAGTCAAGAAGATGGTTCGATGTCCCTTCTGTGCGTCCAACGAGGTGC

GTGCACTTTCCACCGCT

>Tb927.10.15140 | Trypanosoma brucei TREU927 | hypothetical protein, conserved | genomic | Tb927_10_v5.1 forward | (geneCodeEnd+0 to geneEnd+0) | length=258

GGATGCAGTGGAACCTGATCATTGGGTTTGCTTCGTCCTTCTATTTCGCAATGCATCCCA

CTCACGTGCTCCGTACCTGAAGCGCGAATGTAGCAAGAGATTCTTACGGTTTTATTCGCA

CCTGCACCGTTTGGGCAATTGCGTTTTAGTTCACCTCATCTTTGGTTGTCCAAACGCTTT

TATTTGATACTTCCCTCTTCTTTCCGCGTGGTCTATACTCGTTTCCTCTGCAGTCAGGAG

GGGTCACGTTTGGTCATT

>Tb927.11.3580 | Trypanosoma brucei TREU927 | Thiamin pyrophosphokinase, vitamin B1 binding domain containing protein, putative | genomic | Tb927_11_v5.1 forward | (geneCodeEnd+0 to geneEnd+0) | length=258

GGTGCGTGTTTTCTTTGAGCTCTATTCGTCCTTGTTTTATTTATTCATCGTTTTCCTTGT

GTTTGGTTACCCTGCTGTACACGAGAGTCCCACCAACATTGTACTGATATTTCCATCTTT

TTTAGCGTTTCATTCTTAAACGCACCGTTTCTGGAGTGCGTATCCACGGCAATATAAGAA

AACTTCACGAGTGCTAATGGGTGATCCTATCGATGATTTATTGCACCATTTTTAACGGCC

ATTTATTCGGGTATTCTG

>Tb927.9.9000 | Trypanosoma brucei TREU927 | isopentenyl-diphosphate delta-isomerase (type II) (idi1) | genomic | Tb927_09_v5.1 reverse | (geneCodeEnd+0 to geneEnd+0) | length=259

GTACGGATGTGTCTGTGTACCAAAGGGTGTGAATGGCGGAAGAGGGATACATGTGACGAG

AAATCTGGGCCTTCAGGTGAACAATTTTAATGTTTGTTTCACGCGTCTCAGAGGAACGCG

CTACCCACCCAACACTGGACCATTGTATGTCGGTTTCAAATATGTGTGTTTACGAGCGGG

AAGTTGCTTCTGGGATTTATTAGGGTTGCAGTTGAGGTGTATTCTTACGTGAAATTGCCC

AGTTTTCCCTCACATGTTC

>Tb927.8.1880 | Trypanosoma brucei TREU927 | hypothetical protein, conserved | genomic | Tb927_08_v5.1 reverse | (geneCodeEnd+0 to geneEnd+0) | length=260

AGAAAAAGGGGTGGACGTAATCACAAAGCATGCTGACAGGGGAGGGGATTAGGCGACTAG

TTTCCTGCATTTCGAGATCGTTGCTTCCCATTGGGTTGCTCTCATCAGTGCTGCCTGAAA

AGCGCCCCCACCACGCAATCTTTAAGCCCCTTCCCTCGAACGAGTCGGCGCGAGGAGTTT

TGTGGTATGTGTGTGTGTGACCACATGCTTGCTTTTACACACAAGCAAGTCCGTTCTTCA

CACACGCTTTGTGAGTAAAT

>Tb927.4.3840 | Trypanosoma brucei TREU927 | nucleolar protein, putative | genomic | Tb927_04_v5.1 reverse | (geneCodeEnd+0 to geneEnd+0) | length=260

AACGCAACACGGAGATGCGGACACATACAACCTTTTTTACTTGCCTGTTCGTGGAAGCCC

AGCGATATGTCTTGAGTCAGGGGGCATAGTGTGCGTTGCCTGGTATTTGAACGACTTCGT

GATAACCCACTCTGTCACGCATTGCAAACACAAACACACGCGTCGTGGTCACTTATAAAA

TATTTAAGAATAATCAACCTGTTAATTGTACCGCGCAAGGACGAAGCGAAGTAACATAAA

CCAGCGTTGGCACTTACAAC

>Tb927.10.3800 | Trypanosoma brucei TREU927 | predicted RING finger protein | genomic | Tb927_10_v5.1 reverse | (geneCodeEnd+0 to geneEnd+0) | length=260

ATTAGTCACAAAATGAAACTCAAAAAGAAAAAAGACGGGTTCATCGAAGCTGGAAGGTTT

TTCGAGTCACGTGACCCACAGCGAACTATGAAGTAATAATATATATATCTTTTTAAAGTC

GACAACTTTTAGGTGAAAAATTATCCATTCTTCTTAAGGTTTCTTATCTTTCATACTCTA

ACTTTTCTTTTGTTTTGCTTCCTCTCCTTCATTATGAGGTACTCGGTTCGTGTACATTGA

TTTGACTTGCTGCCTTTTCT

>Tb927.11.10980 | Trypanosoma brucei TREU927 | Got1/Sft2-like family, putative | genomic | Tb927_11_v5.1 forward | (geneCodeEnd+0 to geneEnd+0) | length=262

AGCAGGGCGTCAAGATTTGTGAGCTGAGGGGGGGGACTGGAGGTGCATCATCTTTCTTCC

CAACGTCGTGTATCTGAGGAGAATGGAGCAAATGAGGAACCACGACGGGGGGGGGCGGGC

ACCCGTGTCCAACTGCAGCGTTTGAAACTACTGTTAATAATCTGGTGGGGCAAGCGCTTC

TGGAAGCTCAGTGATGGTTGTGTGCTTCATTTTCTTTGTTTTTGCTTGCTTTGAGCGCGT

GGTGTGCGCTAATAATGTCGTC

>Tb927.10.290 | Trypanosoma brucei TREU927 | proteasome alpha 2 subunit, putative | genomic | Tb927_10_v5.1 forward | (geneCodeEnd+0 to geneEnd+0) | length=262

ATGTGGTAGAATGTTTTCTCCGCTGTGATTGCGAGTAGGGTGCTTCCTTATTTTTTTCCT

TTGTAGGCAACCCGCGGCGGAAAAGCAACGAATGAGACATTGACGCAATTTGAGAGGAAA

AAAGAGGGGCCACTAAAGAGTGCAGGGTAGTAAATGGTGGGTGCGTGATGGTTTCAACTG

CCCTTCTGGATTGTGTCCCACTATTATTGGGCACCATTCTATACGCCTGCCCGAAAAGGT

GTGTGCGTGTGTGGTTCAACTG

>Tb927.10.12460 | Trypanosoma brucei TREU927 | Zinc finger, C3HC4 type (RING finger), putative | genomic | Tb927_10_v5.1 reverse | (geneCodeEnd+0 to geneEnd+0) | length=262

AAAAGTCCCCCCTCCTCCCCCACACATCTTGCGTGTGCATGTTGACGAATATAGCAGGGT

TGTCGAGCTCAAAGAAAAAATTTTCTATATTTCCACCCCCATTTTGCCTTTTATTGTCTC

TAAAACAAGTTCTTCTTTTCTTTGTTTGTTTCTTAAGCTGCAAGGAGTGAAAAAATGTGC

ACGCTTATAAGTGCACGCATTCTCTGCTGCCTCTTCAACACGTTCTCCCATGTTCTCTTC

TTGTTGTTTTTTCTTGTAATTT

>Tb927.2.4980 | Trypanosoma brucei TREU927 | EMG1/NEP1 methyltransferase, putative | genomic | Tb927_02_v5.1 reverse | (geneCodeEnd+0 to geneEnd+0) | length=263

AGGGACAAGTTCGGCGAGGGTTTGAGTGTGAAGGACCTTGTTGAGAAGTAACGAAGGTGG

GGATAAAAGCTGGGAGCTAGTAGCGGGAACCAATAAGAGAGATAAGTGCGGAAATATATC

GACGGCAGTGACGGTGACGCTGAGTGGGGACTGAATGGCAAAGGGGTACTGTTCATGTGA

TGTTTATGAACGTACCACCTCTTCCGCTTTTGTGTCTCCTTCTCTCGCCTCACCAGATCA

CTCACCGTATTCATCTTCTGCTT

>Tb927.11.11790 | Trypanosoma brucei TREU927 | R3H domain containing protein, putative | genomic | Tb927_11_v5.1 forward | (geneCodeEnd+0 to geneEnd+0) | length=263

AGCACCGATGCAGTGCTTTCGCATCTTTTTTTTTGTACGGGACGTTCACGGATTTAGTAC

GTGTTTCACTAAGAGTACAGTGATTCCTCCCTGTTCACTTTTTCTTCTTTTATTATTCTC

TGTGTGCTGTGGAATATGCAAACGCGGATAGGTCTGCTTACACGAGCCTCTACGCGTTTA

CATTCTCAGTCAGTCTCAGCGATGAACCGTCGGTAATAAAAGCTTTACGGTTCTTCCCCC

AAGAGAAGAAAACAAAAAAAGAT

>Tb927.8.6250 | Trypanosoma brucei TREU927 | hypothetical protein, conserved | genomic | Tb927_08_v5.1 forward | (geneCodeEnd+0 to geneEnd+0) | length=263

AGCAGAATGGCGAAAAGTCCCCGATGCCGTTTTTACAAAACTAAGTGGGGTACGCTTCCC

TCCAGTGAACACCATTAAGAGGGGGTTGGTCAGACCGGGGGAAAATAAGAAATAAGGTTT

TGAGGTAAGATGGGCAGTCTTCCACGTGGCAGGCATATGCACAGGTACCGTGGACCGGCA

GCGATGTTGCAAAATATGTACAAACATATATACGCAATAAGTGCTCGCACAAGGGTCGAA

GGTGCGTAAACAAAAGAGCTATT

>Tb927.11.1750 | Trypanosoma brucei TREU927 | hypothetical protein, conserved | genomic | Tb927_11_v5.1 reverse | (geneCodeEnd+0 to geneEnd+0) | length=263

ATACATTAAAAGACCAACCTTAAATTAAGTGTGTGTGTGTGTGTGTGTGTGTGGGTTTGC

GTTTGTGTGGGGAGCGAGGGAGTGTTCGGAGACAGGGAAATTAAGATATGCGGGGAAAGT

AGTGCTGTTGAGTGTGGGGGTTGCTGGAGGAACATTACGCCGATAAAATATGTCGATACG

TGTTTGTCAGGTTATACGTGTCTTTTTTTTTTGCTCTCTGAGAATTTTTATGATGGTGCT

TTTCGTTTCCGACATGTACGAAC

>Tb927.8.1230 | Trypanosoma brucei TREU927 | hypothetical protein, conserved | genomic | Tb927_08_v5.1 reverse | (geneCodeEnd+0 to geneEnd+0) | length=264

ACCCGCGCTAATAGTCATGTGGGTATGTCGGATAGTGGAGCGCTGCTCCTCCACCGTGTT

CCCCCTTTCACTTCTCCCAGAGGTGCTGTGATTGACGGCTCCCGCTTCCTTTTCTTTTTT

TGCCCATACTGTGACGGATTAGGTTATTCTGAACGTTGGCTAACGCGGGTGGCATGTGTT

TATGTATGATGTGCCGTGGGGGAGGGATTCATGACGTGAAAATTTAAGTATAAGAGGTGC

GTTTTTGTCTTGGCTAAGTGAGGT

>Tb927.11.4450 | Trypanosoma brucei TREU927 | ALBA-Domain Protein (ALBA2) | genomic | Tb927_11_v5.1 forward | (geneCodeEnd+0 to geneEnd+0) | length=265

GCGGTGATTTAACAAAAAGAGAGAATGAACGGTAAATCCGAACTTACGTGTGCTTAGGGG

GGGAGAAATAGGCTGCTGAAGGGCATGTGTGGTATACATATACATCATATGTCCCGGTGT

AAAGGGGAGATAACTGGTGAAACGGAGGGTTAAGAAACAGTTCGAGAATCACACTCAGCT

ATGTTTTTTTTTTTAAAGGTGGGATCTCTCTTTTTCGTCTTCGATACAATTATTGATATC

GCCGTGTTTTACGGTAGCATCCAGG

>Tb927.6.2220 | Trypanosoma brucei TREU927 | hypothetical protein, conserved | genomic | Tb927_06_v5.1 forward | (geneCodeEnd+0 to geneEnd+0) | length=265

AACATAAGCAAGGGCAAGTACGCGAGATGTAGGGAGCGATCAAGCGAAGGGGAAGAAAAC

CAAAAAGGGAGAATAGTCAAGAAAGGACAGTCGAATCTTAGTTTCTGGAACTGCTCTCGC

TTTTTTAGCTTTAGTTCCTAACTTCTGAAGAATATCATATCCAAATACGAATATTATCAA

ATGGCTTGATGGTAGGTGGCCCGTATTTTTGCTTCTTTGTGTTTCTTGCCTTCTCAGGCT

TGGTTGTTAGACCTATGGTAGTAAC

>Tb11.02.5370 | Trypanosoma brucei TREU927 | hypothetical protein, conserved | genomic | Tb927_11_v5.1 forward | (geneCodeEnd+0 to geneEnd+0) | length=265

AACGAACAGTGCCTGTGACAGCTGCCACTGTCAGTCATTTCGGTATGTCGCGTAAATATC

CTTGATGTGTACATTTGCGCATTCACTGTGGTAGCCGTTGTTGTCGAGATTATTAACGAA

TTTCTTTGGTGTTGTCGTGCTGTTTCCTGCTGCTGATTCCATCCTAATCGTGTTCATCGG

GAGACTATGAAGAACTGTGTCTTTCCGACTTCATGTTATCTTATGATTCTTAACGATACA

CTTCGTTGTCGTTGATATCGTCCTT

>Tb927.9.10890 | Trypanosoma brucei TREU927 | Regulatory subunit of type II PKA R-subunit, putative | genomic | Tb927_09_v5.1 reverse | (geneCodeEnd+0 to geneEnd+0) | length=265

ACAAGGCAGAACTTCGCCTTCGCTGCAGTATTAGGGGCGGGCCAAGCATAAAACGTTTGC

ATCCGAAACACATGCAGAGAAGGAAGTGTTTTTGTGGTGCGCGTATAGTCGTAACATGTA

CCATCACTTCGGGCGCGCGCGTGTGGTGCATTAACACAGGATGCAGCAGGCTGGGTGCGT

ATATGTAACATGTATATATATACATGTGTAGATATGCATGTGTGTAGATACGTAAAGCTG

CAGGCGGGCTGCGCTTTGCAAAACG

>Tb927.4.390 | Trypanosoma brucei TREU927 | hypothetical protein, conserved | genomic | Tb927_04_v5.1 reverse | (geneCodeEnd+0 to geneEnd+0) | length=266

ATATGCTGAATACCCCTTTTACATTACTATTACCCTTCTTTTTTTTCTTTTCATTTTTAA

AGAAAAAGAAAAATAGTGAGGTGCATTGTACAAGCGTTACCAGTTCTTTTCTTTTTTTTA

ATTGTTATCGTATATCTGGACGGTGCGGAATAGGAACTCTCCTCTGCATCACCATTTCGA

CTTCCCTCATGGCGCTTGATTTTCTTTTCTTTTCCTTTTCCCCCCTCATCATGTACTCGG

AGGACACTGCAATTTATTTCTTTGTT

>Tb927.7.7140 | Trypanosoma brucei TREU927 | Vta1 like, putative | genomic | Tb927_07_v5.1 reverse | (geneCodeEnd+0 to geneEnd+0) | length=267

ACACCTACGCCACCACCGTCGCCCACGGTAACACCAGTGGTCTCTTTGCTTTGATTGTAA

GTGTACGGCAGAAAATCTTTCTGCAGTTGTTTGTGTTTCATCGGTGCTCTGTTTGTTTGA

TTTCTCCATGTTATTATCGGTGGTGGCAGGGGTGAATTAACCCCCAGCACGCCTCGTAAT

GTTTGATTCAAACGGGGGTGATCTTCAAGAAGAAGCACAAGAGGGAAAAACGCGAATATG

GAAAGGAAGGAGGAGTCAGGTGAAACG

>Tb927.3.3120 | Trypanosoma brucei TREU927 | hypothetical protein, conserved | genomic | Tb927_03_v5.1 reverse | (geneCodeEnd+0 to geneEnd+0) | length=267

AGCCATGCCACAACAAAGAGGGCGGTAGCCTCCGCAAGGCGCGAACATATCGGATCTGTA

TTTCTGCACCACACACATCTCTGTGTACAGGTTACCCTCTTTCTCTCTGACTGTGCCATT

CTTTGCAATACGCCAACTTTTCTTCTACCCCCCCTTTTTTTAAAAAAAAATATGACTTGG

CTACCTGTGCTGTGTTTAGACGCATACCGTACGCGGAAGTTCACTGACGCCGCACGCACC

CATATGTCGATGTAGATGTACGTGTAT

>Tb927.8.640 | Trypanosoma brucei TREU927 | Dpy-30 motif containing protein, putative | genomic | Tb927_08_v5.1 reverse | (geneCodeEnd+0 to geneEnd+0) | length=268

ATAAATTGCCGTGGCTGCTGTGTTGTGCCCAGTCTTACACTTTTCTTCCCTCCTTTTTTT

TCATTGATGTGCCTGTACTCTTCGTTGCCTGCAGACAGTTTTTTCTCTCTCCCTCTTCCT

TGTTTGATCTGTCAAATCCAATGACGCAAAAAAAAAACCACTTATGCGTTGTTGTTGTTC

CACGTCGGCATGAACCAGCGATGAGGAAAACACTCGCCCATCTTTTGAACGTGGTGTGCT

GCGCGGTTTGACTTGATCCCCTCACAGT

>Tb927.7.5030 | Trypanosoma brucei TREU927 | hypothetical protein, conserved | genomic | Tb927_07_v5.1 forward | (geneCodeEnd+0 to geneEnd+0) | length=268

ATGCGTGTACTTTTTTTCTTTTCGTGGTTGGGTACTGAGAGGTCATTCATTTAGTGGATG

TAAGTTTTTTTTCGTTTTGTCGAATTACGAGGTGAACCATTGTGTGCCTTCATGGTATGA

CGCCGTGGTGCCCTTGTTATGCGTTGATCTCCTCTAGAGCAGGGAAAGAAAGAATGATCC

ATGTACAGAAAGGTCGCTATGGGTGGGATATTCTGTTTGTTTCTGATTTCTGTTTATAGA

AAGCGTGTGCGTTTGTGTGATCGCAAAT

>Tb927.11.15220 | Trypanosoma brucei TREU927 | hypothetical protein, conserved | genomic | Tb927_11_v5.1 reverse | (geneCodeEnd+0 to geneEnd+0) | length=268

AGCAACGCGATGCGGAGCAAAAACAAGGAGGGAGGAGGAGGCAACGCACTAACATCTGTC

GGTGCTGTGTCTATGTAAACTATGACGCTATTTAGAATTGAGTACTACTGTTGTTAAAAG

GCGTTGGTAAGAGAGAAGGGGGGGGGGGTGGCGAGGAAAGACCCGCACGACACGTAAGCA

TATCTCCCACTGAAAGGATGATAGAGCTGACATGGAGAAGGGTGTTTCGTATGCTTATTT

CGTAGATCTTTACCTGTTTCTACAACCG

>Tb927.11.6520 | Trypanosoma brucei TREU927 | hypothetical protein, conserved | genomic | Tb927_11_v5.1 reverse | (geneCodeEnd+0 to geneEnd+0) | length=268

AGCTTTATTCCACAAGGCAAAAACCACAGGGGTGAAAGGGATACGTGTTTCCTTCACCTC

GTTGCTTTCGTGAAACAACGTTAGCGGGAACTTATGATATCGCTATGTTCGTCATCGTTT

TCCCCGTGCAGGCGTCGGTTCCCGACTTCCCACAACTGAGTTCCATCGAGGAACTATGTC

CATTTGCACAACTCGCGACACCTCCTCTCCTACGTATTCTGGTTTTGGGAACAAGTTGTT

GCGAGTGTTTCATTATTACCCACAACTG

>Tb927.11.8050 | Trypanosoma brucei TREU927 | Sas10/Utp3/C1D family/Sas10 C-terminal domain containing protein, putative | genomic | Tb927_11_v5.1 forward | (geneCodeEnd+0 to geneEnd+0) | length=269

GTGGTTGAAGAAGGAGTTCTGTCTAAGTGCGTTGTTTCGTACGGTGGCTTGGTACGGTAC

AAGCGTTGTGATCTTTGGGACGCACGGGAACTTCCGTGGTCTGCCCGGCGCTAATGTGCT

GTTCAGCGGTTACGTTTGGTTAAAAAAAATAATTATGCTGTGAATACCGGAGGACAATAA

GAAAAAACAAACCAAATCAGTAAGCAATAAACCCAAGTAAAATTAATTAAGACCGCGTCA

CCTGTGGGCATTATTACTACTGCTTGCTT

>Tb927.10.6530 | Trypanosoma brucei TREU927 | dolichyl-P-Man:GDP-Man5GlcNAc2-PP-dolichyl alpha-1,3-mannosyltransferase, putative (ALG3) | genomic | Tb927_10_v5.1 forward | (geneCodeEnd+0 to geneEnd+0) | length=270

GGCAACCGGAACAAGTGATCTTGGAAACAGTGGCACATGGGCGGAACGACATCACCGTGC

GCAACGCAAATCGTCGTTGGTGGACCGTTATCGTTCGCTGTTTCATCTTTGAACTGTCCA

GGTGGTTTCCGCTCGTCGTTTTGCTGTACTTCGTAATGCGATGATCGGCTGTAGTTCACC

TGAATGTTGTTAACACGCGGAGCGGTATGGCCTTGATAAGATGGCAGGCCTCACTACTCC

CTCTGATTTATAATTTCCATTTGGGCCATT

>Tb927.5.3020 | Trypanosoma brucei TREU927 | hypothetical protein, conserved | genomic | Tb927_05_v5.1 forward | (geneCodeEnd+0 to geneEnd+0) | length=270

GGCCGTCATCGCTGGCCCCCTTATTGTTGTGATGCTGAACTTTAATCGGATGGAAATACT

TATACAAATGGCCTTTGACCCCGCATCTTTTACAATTTTCCAAGCGCAAACTGACCCTTC

TTCCTCGTGGGTAAGTTACTGAAGTGTGGATGATTAACGACAGGGTATTCTTACGGCTCC

TGACTCGGCAATTTCAACATTACAAACTCTCTAGGACCTTCGTAAGCTGTTTGTGTTCGT

TTTCCCTCTTACCGTGCGACGATCTCTCAC

>Tb927.9.1390 | Trypanosoma brucei TREU927 | CSL zinc finger, putative | genomic | Tb927_09_v5.1 reverse | (geneCodeEnd+0 to geneEnd+0) | length=270

ATTGGAGAAGCCGGTGTTGACATCAGTACTCAACTGAGCAAGGGGATGTGTGATTTGCCG

GTTCAGGATGCCTCTTCCCCGAGATTAGCATGTGCCGTTCCATTATTTTCTCTTCCTTGA

GAATATAGTCGGTATTTAAAAACGTGAGACAATAATCACGGTGTTAAAATTGAGTGTAAG

TGGTAAAGTGGGGACCTAATTGCCTTCCTAAGGTGATGCTGGTTTACCCTTCTCCTCCCT

CCGGTTTGTAGTGCGTTATGGCAATACTTT

>Tb927.7.1520 | Trypanosoma brucei TREU927 | hypothetical protein, conserved | genomic | Tb927_07_v5.1 forward | (geneCodeEnd+0 to geneEnd+0) | length=271

GCGGGAAGAGTCGAGATGCCTTGAGGTGTTCATTCATCAATGAAAGCTATTGGTTCAGTT

GTGTTTCTCCCTTATTAATTTTTCGGTACGTATGCACCATTTACTATTTGTGTGTGAGGG

TAAAAAGGAGGGTGTGCAGGTGCTCTTTGCGCGCCGCGTACTGGTTATCGACGTTGACAG

TTCTCCCTTTCATTAAATATTGCTGTCGTCCACATAACGTTGGAGGTGCGTGTGTCTGTG

TTGTACACTGGACTCGTGGGAGAGGGACTGT

>Tb927.5.3970 | Trypanosoma brucei TREU927 | adenylate kinase, putative (ADKE) | genomic | Tb927_05_v5.1 forward | (geneCodeEnd+0 to geneEnd+0) | length=271

ATGTTGGCGCGTGAGGCAAGAAATAATGGTGAATTAAAGAACCGCGCAACGTTCTTTGGG

CTCTTGGTGGGAATACTCTCGAATGCACAAAGAAAAGGGAACATATGTATTGTAGAAGGA

TGGAAATGAAAGGAGGAAACAATGGATGTTTGCGGCGCCTGCAAGTCCCGCCAGAGACAT

GCAACCGCCTGCGGTGGATTTGGCCGCGCATGTCGGTTGGATATATTGTTTCTCTACCAT

CTCCCTTCCTAGCTTAGGTATTTGGTTGTGG

>Tb927.4.1600 | Trypanosoma brucei TREU927 | AAA domain containing protein, putative | genomic | Tb927_04_v5.1 reverse | (geneCodeEnd+0 to geneEnd+0) | length=271

AGCGCTGCAGTCACACCCTCCATAGCTGTGGCTGACTGACAGTGGGCAAGCAGTGATTGT

GAAGTGTGTGGGCTCGTCGTTGCAGAAACCCTTTGCGCCGCTTGACCCGTTCTGTTGTTT

TTTGCTCTGTTGTGGGTTTACCAGATGTTCCGGTTCTCATGTAATAATTTATCCGTTTTA

TCTTCTTTCATTACATTTGTGAAATGTGGGGAAGGGGGTACTGCAACTTACGGGATAACG

TAACGAGAGCCGGGCCGCAGTTTATTTTCCC

>Tb927.3.1780 | Trypanosoma brucei TREU927 | U6 snRNA-associated Sm-like protein LSm8p (TbLSm8) | genomic | Tb927_03_v5.1 reverse | (geneCodeEnd+0 to geneEnd+0) | length=272

GCGGGAGATGATTGTGAGTTCTGATGACGTTGCACTAATTCATCGGTCGTCGCTGCTCGA

GTTGGTGGGAATCACAACGCTGGTTAGGCTCATGTTCTCTGCTTTCTTTTTTTTTTTGTA

GTCAATTACATGTCCGCGCGGTGATTATTAGCTTGCATCAGAAGGAATATAGCGCATGCG

ACTGCTCTCATTTTGGTTTGTTGATAATACCCTATATCCATTAAAGACGTGACACATTCC

TCTTTTTGTTGGTTACTGTTGTTCTTGAGCTT

>Tb927.10.15870 | Trypanosoma brucei TREU927 | RNA binding protein, putative | genomic | Tb927_10_v5.1 forward | (geneCodeEnd+0 to geneEnd+0) | length=272

AACAGTGCGAAGTGAAGCACCTCGAAAGGAACATGGGGAAGTGGAACGAATGTTGCATAA

GCGTCGTACTGTCAAAAGAAACGGAAGTGGGAGACCCAAAGGGGGAATGCCAAAAAAAAG

TAGGCAACTGCTTCCCATCGATATTCGGTGCCTTTGGTTGCGGCACTCGCTGCAACGCCT

GCAGCTCTGCGGTTTAACTGCCAGGAGCAGGGCTACACCCTGACCTCTGCGTGCCCTCCC

TCACTTCTTGGCGCCTAATTGCGCTCTTCTTT

>Tb927.8.5260 | Trypanosoma brucei TREU927 | 60S ribosomal protein L39, putative | genomic | Tb927_08_v5.1 forward | (geneCodeEnd+0 to geneEnd+0) | length=273

AGTTGGGGTAATGTTAAGCTGTGGGCCAATCCATGAGGTGCGATTCGCGAAGTTTCCAGA

GTGCGGAGGAACAGATAAGATCCAAAAGAAAGTACTCGACGAACTTGTGCGTTCATATAC

ATGGAAAAGTCTCATGCACGCGTTGGACTACCCTTCATGTGTTTTTTCTCTTTTTTAATT

ATTTAATTTTTTATTGTCAGAAGATCGATTGTATGACCGTTGCCTCCATCATTAATTAAT

CTGACAAAAAGTAAAAATATATATATAAGCCAT

>Tb927.9.4960 | Trypanosoma brucei TREU927 | hypothetical protein, conserved | genomic | Tb927_09_v5.1 forward | (geneCodeEnd+0 to geneEnd+0) | length=274

AGTAATACGACCTCAATAGGGAATGACTATGGGTATGTGTAGCGTGGAGTGCCCTGCGTG

AGCGGGTTGTGAAATTTGGGGGTTGGTGGGAGAAATTTTTTTTTTTTATGGTCGAGCGAA

GGAAAGTTTAAACGAAAACGATCCAGTGCAAAGGGTAAAGAAAAGCAGGAATGCGAAGGA

ATGGAAGAAATATATTTTCGAAGCATTTATTATTATTATTATTTCCATATATCTTTATAT

TTGTCTTTGTTCCGCTACTGAAATGGTGAAACAG

>Tb927.11.3340 | Trypanosoma brucei TREU927 | RNA-binding protein 34, putative (RBP34) | genomic | Tb927_11_v5.1 forward | (geneCodeEnd+0 to geneEnd+0) | length=274

AAGTTTGCCGACGGTAAATGAAGGCGAAGAGGACGAAGCGCGGGGTCCTAATTTGTTTGT

TTGTTTTCCCCGATATCTCCGTTCCGTGTTGGAATTAACAAAAGAAAAATATGGCACCAT

GTGTGTACGCACTGCCTTTTAGATGGGGGGAGGTGGGTTGTGTGAGATGAGGTTATGGTT

AGGGTTAGGGTTGCGTTTTGTTAAAACCCTTTTACTCTTTCCTTCTCCCAACCTCACTTT

CTTCCTCTTTTAGGTCAGGTGTAGTTCCGTTACG

>Tb927.6.4590 | Trypanosoma brucei TREU927 | glutamyl-tRNA synthetase, putative | genomic | Tb927_06_v5.1 forward | (geneCodeEnd+0 to geneEnd+0) | length=275

GAGGGAGAAATTCAGCACAAACAGGGGGTTGGCTAATCTACCCATTGATAGTAATTAAAA

AAAAAGAAAGGACTGCGCGTATGCAACAAAAGACACACACCAAGACGATGCGAATGATTA

TAAGTTTTCCAATACATAAACATATATGTGTTATTGAAAAACCATATACTTGCGTGTGTG

GAGGCTTGCATGCACTTAAAAAAAAACTTATTGGTGAAGAAATCTAAGTTGTGTTAAGGT

TTAAGAAAATTTTGATGTATGTCGGTGATAAAAGC

>Tb927.11.16750 | Trypanosoma brucei TREU927 | hypothetical protein, conserved | genomic | Tb927_11_v5.1 reverse | (geneCodeEnd+0 to geneEnd+0) | length=276

ATGACGGGAACAATTTAATGGTGTGGTCACGCAATGAATTATTGTGGTGCGTGAGGTTCT

GTTTGATTGTTTTCATTAATGTCGTCAGGTTCCTGTTTTTTTTTCTTTCAAAAGGAAAAT

AATTGTTTCCACTTCTTTTTTTTTTTTTACCTTTTTCGTGTTACTATTCTTCTATCTGTG

TTCTGTTTCGTCTTAAATCGTGCGGTCGTCGAGTTGGGGTGGGTGTCAATTCTGCAGTTG

AAGATTGCTGTGGGATCTGATGTAGAGAAGAACCTC

>Tb927.2.6050 | Trypanosoma brucei TREU927 | beta prime COP protein | genomic | Tb927_02_v5.1 forward | (geneCodeEnd+0 to geneEnd+0) | length=276

ATTACGTAAATAATCGGCTTAAGATGGAGTGGTGGACCCGTTATTATTTTTTTTTCCCAA

TAGCGAGAGACGTCCCGTTTTTGTTTTTGTTTTTGCTTCTTCTTATACGGAAGGGAAGAT

TTTTCCTTTTTTTTTTTCAAAGAAGAAAGATGCAGGAAACCAGACAGTAAACACAAATAC

GGAGGAAAAAGAGCTGATATTTACGCATTTTGTTTTCTTTTCCCCCTCCTTCTGAGATTT

CCACTCCTTGGAGAGCAAGGCAGGGAATGGGAGGGG

>Tb927.11.8890 | Trypanosoma brucei TREU927 | DNA-dependent RNA polymerases, putative (RPC19) | genomic | Tb927_11_v5.1 forward | (geneCodeEnd+0 to geneEnd+0) | length=277

GACCGATGTAGCGGTGTTGCTGCTCTGTGCTGAGGGTGATGCTTCCACCCAAGGTGTTTT

CACTTTTAGCGGTAGCAGTGCTGGTGTCCGCATCATCGAAAAATATTTCCGGATTATGTC

GGTGGGCTTTGTTTTTCATGCATCCGGTTATATAATATTTGGATACGCACTCGGTGTAGA

AGCCCCAGTTGTGGTTTTCAAATAAATAAAGGAGTTTTGAATTCAATATCAAGATATGTT

CGCTACCGCAATGTGAGCTGGATCCTTTGAATAATCT

>Tb927.11.6590 | Trypanosoma brucei TREU927 | aminopeptidase, putative, metallo-peptidase, Clan MF, Family M17 | genomic | Tb927_11_v5.1 reverse | (geneCodeEnd+0 to geneEnd+0) | length=277

GCCACATGGCGTGCAAGTATCGGTTCCCGAATTTGGAAAATCGTTGTAGGGGAAACGAAT

GTGAATGAAGGGTCTTGACTGCCCATGTTGGAGATTTCTTCTACATGATTTCCTTAACCT

GCTCTCTTGGGGTTTGTGTAGGCGCGGATTGGGATGAGGTCGTGGAGGAAAAAGGGAGAA

CACATACACACACACTCAACCACCCACTCACAAGGGGAACTGCGGGAAAGGGGAAGGGAA

GGAAAATAAATACATGGTGAATTCTTCTTTGTTTTTG

>Tb927.1.1680 | Trypanosoma brucei TREU927 | Transcription elongation factor 1 domain-containing protein | genomic | Tb927_01_v5.1 forward | (geneCodeEnd+0 to geneEnd+0) | length=277

GGTTCATTGGAGCCATGCGTATGCATTTATACAACCCGCTCACAAGGAATTGGATATAAG

TTATGTGCCGCTCCTGGAAAGGATCTAATATCTTGAAAAAACTCCTGCACCATTTCATTT

AACGTTTACAGTTCCCGTGGATTAAGAGGCCTTTTCAGACAAAACGCCTGTGTTCGGGTA

CCAACGAATGCCTTCGCCTGTCCGCGGGGCCATCGGGTGCACATCCTTACCTCCTCACCT

GCCTGTACGTCTGTGTTTTACGTGAATGAAAAGGAGT

>Tb927.10.1280 | Trypanosoma brucei TREU927 | Domain of unknown function (DUF4499), putative | genomic | Tb927_10_v5.1 reverse | (geneCodeEnd+0 to geneEnd+0) | length=278

GTAGTAAATGAAAAAACAAAGGGCACGCGAGAATGATGTGTCGGGGAAGATGGTTGATCG

TATGAGTTCGGCCGGCTGGTCTTCACTTGACTACTCTTCAAACCCCGCCTTGATTATCTT

TTTCATTTCTTACCTTTTTAACTAGTTACAATATATACTTGTACTCAATTTCCTTCTGCT

TTTAGCTTCTTCCCATTCCGAGACACTGAACGCGTTTACAACAAGGGTCTGGGGAATTGC

TGCAAGCACGAAAGTAGGGAACTGGCGTATATGGGAAG

>Tb927.5.4400 | Trypanosoma brucei TREU927 | hypothetical protein, conserved | genomic | Tb927_05_v5.1 reverse | (geneCodeEnd+0 to geneEnd+0) | length=278

GATGTTTGGTTGCGCTTGCGTAGTCGAGCCCGATCTCAGTTGACATGCGGTATCACAACG

GTAAGTGTACATTCGCCAAAATATTCATGTAGATAGGTGGGAGATAGGTGTTTTATATTC

GCATGATGTGTGAAAGAAACGAGGAAGCACCCTTCGCAAGGGTTGGATGTATACGGTTGC

ATGTGTTGCCGTGTAGACATCGAACGTAGCATACTTGCGAGACAAAGCACAAGTATTCAG

AGCCGTCCGTTTGTATATGCTAAAGATGTGAACAATTC

>Tb927.3.1760 | Trypanosoma brucei TREU927 | chaperone protein DNAj, putative | genomic | Tb927_03_v5.1 reverse | (geneCodeEnd+0 to geneEnd+0) | length=278

AAAACCTAACGACGTTGTATTTTAGTTGTTTCGTTTTATTCGTTGTAATTTTCCACCCAC

CCAAGTCACTGGGTTCTTGTGTGACAATGATATGAGCGACGGGTGCAGAGTATTAGTTGA

AGATATGCTACACTGATTGTATCAAAAAAGAAAAAAAGAAAAACGATCACTGGCTCATTT

TTTTTCTTTTTCCTTTTTAAAAATTATTTTTGCTTCGTTAAAATAAACAAAAGCGTTAGC

GTATCCTTCAAAACGAGTTGCGGTGTTGATAATCATTG

>Tb927.11.5820 | Trypanosoma brucei TREU927 | Inhibitor of apoptosis-promoting Bax1, putative | genomic | Tb927_11_v5.1 forward | (geneCodeEnd+0 to geneEnd+0) | length=279

ATGTTGATGTGGTGGAGCACATGAGGCTCGAAATAACGTGTCTACAGATCTTGAAGAAAG

GAGAATAATAACGCTGAGCAAAGCATTTTCTTGCTTCTCTCTCTCCTTATACTTTTTTTT

TCTATCCGGGACCGGAATTTCTCTGCTTTTGATTTCTTTTGTTGCCTACTCCACATCTTT

TGCGCTCTCTTCGGTTTTCCCCAAATAGAATTTGAAGTTGCCTGTCTTTAGGGAGGAAGC

GGTGATGTCTAGGTGAGGAAGCGAACGACGCTAACACTG

>Tb927.10.4450 | Trypanosoma brucei TREU927 | stress-inducible protein STI1-like, putative | genomic | Tb927_10_v5.1 reverse | (geneCodeEnd+0 to geneEnd+0) | length=279

AATGCATGGCATGGAAGACTCATGGTCCCGTAGGCGGTGCGTACGCCACTCTCCGCTTCC

CTAACATTGGGTCTATGGCGCACACCACTGCAAGCCAGCGGAGGGGGGGGGGGGAGGGAC

GTATTTGCCATGGGAGCGCGTGTGGTGCAGTATGTGCTTCGTGCTAGTGCGTAGAGGGGT

TTAATAACTTTATTCCTATTTTACTGTTGTTTGTTTCATTATGTTTGCAGGCGCTCCATC

TGGCTCATGGACGCCCATGTTATGTTTCCTTGCTTCCTT

>Tb927.8.3530 | Trypanosoma brucei TREU927 | glycerol-3-phosphate dehydrogenase [NAD+], glycosomal | genomic | Tb927_08_v5.1 reverse | (geneCodeEnd+0 to geneEnd+0) | length=279

AAACACGATTACATTCGTGTGGGTGGTGTGGGTTTAGCAAAGTGGACGTGGGTTTCCTTT

CTTGTTATATGCCATATTTCAATAACAACAATAGGGAGTAAGCGGCAGTGCGCACCGAGG

TTGTAACCATATTGCGTTCCTTCCTCATTATGCGGCATCCTAACGGTTGAATCTAAGGTT

TCTTCAATTTTTTGCCCTTCTGAATCTTTTCTTTTTCGTTTCTCATTATTTTGATCCAAC

AATTCTAAAACGTGAATAGCCTTATAGGCCTGATGCTGG

>Tb927.7.880 | Trypanosoma brucei TREU927 | RNA-binding protein, putative (RPB25) | genomic | Tb927_07_v5.1 reverse | (geneCodeEnd+0 to geneEnd+0) | length=279

AGTTGTGAAAACGGAAGATACGCAGGTGCGTAAGTCCTTATTCTTTGAGGAAGAATGCTG

CTTTGTTGGGGAGAATGGAAGCTGTGGCACGGAGTTGTCGGAAGTAGTAGTGCTGTGGGG

GCTAAAGTTTCATGGGTTTCTTTCCAGTTTGTAATTGTCGGGGGAAAAAATACATCTCTG

CTTTTCATCGACTTTTTCCCCTTTTAATTTTTTGATTCCTGTGTTATTTCAACCGAACCA

AATCACTAAAACTTAGCGAGGGATATTTTTTTTTCCTCG

>Tb927.11.15500 | Trypanosoma brucei TREU927 | hypothetical protein, conserved | genomic | Tb927_11_v5.1 reverse | (geneCodeEnd+0 to geneEnd+0) | length=279

ACCGCAGTGGTGAGAACTTTTTAAGACCACCAATCTTTCATTTCCACAACAAGTTGTTGT

GCGGTCGCTGTTTTATTAGTTTCCCTTTAGCTCCCCTTTCGTGTAGCCTCGCACATATCG

TTTATCCAATTCAATGGAGCGCGTGGGCTCGCGGCCGCTTTTTCCTTACATCACCACCAC

CACCACCACCCTTTTCTGAAGGTTACATTCTTAACGCCAATTTGTATCAAATTCCGCGAA

CGGATGGAGAGACGGAAAGACCACTTGTCTATGTTCTAC

>Tb927.11.4480 | Trypanosoma brucei TREU927 | radial spoke protein RSP4/6, putative | genomic | Tb927_11_v5.1 forward | (geneCodeEnd+0 to geneEnd+0) | length=279

AATACACTAACGTTGTATAAGTTGGGCTGCACGTGAATCCTTGTAGGGAACTTGTCTCAT

GCAGGGGACGCTTGAAGGAAGGAATGAAGTGCGTACTGAAGAGGGAAGAAGTGGAAACTT

GAGACAAGCCTTCATCCCTCTCCCAGCATGCCCCCTTACAACGTCTAGAGAATGTAGACT

CGAACATTAGCAGCCTTGTTGGAGTTATTGTTTTTATTTTCGTAGACTGTTATGCCTCCA

TCTTACGTTAGTGCCTAACGTTATGTACAACATCGGATG

>Tb927.11.14460 | Trypanosoma brucei TREU927 | ADP-ribosylation factor GTPase activating protein 1, putative | genomic | Tb927_11_v5.1 forward | (geneCodeEnd+0 to geneEnd+0) | length=280

AAAATAGCCTGTTACATGGTAGTGTATACGACTATTTGTGAATATATGCCGCCTCTTTTT

CAACCCGAGTGAGCGGAGCATTGGAACAAGGTGTCTTGGTGTCGCTTAAGTCTCTATGTG

GGCAAGTTGTGTGTGCGTGTGTGGGGAGGGTTCCAGGTTGGTCTACTGTGCAAGTCGCAC

GCAAATTTTTATTCCTGGTTCTAATTTCGCAGCCTGCATAAGGGTAAGTGTCTCTGTTCG

TCGAAACTTATATGATTAGCTTTATGTACTGGGGTATACG

>Tb927.2.4090 | Trypanosoma brucei TREU927 | signal recognition particle receptor beta subunit, putative | genomic | Tb927_02_v5.1 reverse | (geneCodeEnd+0 to geneEnd+0) | length=281

ACTAATACTTCACGTATGTGTGGTACGTCCCTGTAGTGCCTCCTGGCGGGAGAAGTTTCC

CTTGTTGGCTCGCAGCATTCAAATGGAAGCGGAAGTGTACGCGAGCGGGGTGAGGAAGTT

AGAACATATAAAAAGAGGAGAGAGGAGGAAAATTTTGCGATGGGATTCCATGGTTACTGT

CACGTTTGTGGAACTGTTGCGCTTGTGTCTGTACATTCCTCTCTTTTTTGCGTTGTGTTT

TCTCACTTTCTCACCCAAAGTTGAAAATAAACTGTATTTTT

>Tb927.11.10530 | Trypanosoma brucei TREU927 | hypothetical protein, conserved | genomic | Tb927_11_v5.1 forward | (geneCodeEnd+0 to geneEnd+0) | length=281

GGTGTGGTTTTTCATCTAAACAAGTAAGTTCACTACTCAACTGAAGACAACGGTCTGTAA

ATATGTGGTGTGCCCACCGCAGACCTTGTATAGGTACGGTACATCAAATACAACGAACTC

AAGTTTACTTTTTGGTTTCTTTCTTTACAGGATTGTTCACATACACGTTGAGGAAAAGGC

CCTCTGGTTATTCCTCTAACTTTTTTTTTCAATTCAACCCATAGTATGTTGATTCCACAA

TCATTGTCTATCCCTTTTGTATGTTTGTATATTTTTAAGTG

>Tb927.5.1630 | Trypanosoma brucei TREU927 | hypothetical protein, conserved | genomic | Tb927_05_v5.1 forward | (geneCodeEnd+0 to geneEnd+0) | length=282

ATGATTCATCTGTGTGAAGGACTTTGTTTCCCGCTTTCACATTCAGTGCCCCACACGTAG

GTATGAGCTACACGTGCATAAATACCTCTAGAGAGGAGTGGCTAACGCTGACGTGCAGAA

GGTACGTGTGTGAAATGGATTTATTGGACAATCAAGTTACCGTGAGCCTGCCTGTTAACC

CGTAGTAGGTAGTTGCGGCTGCCACTGGCAGCAGAAGATCCTCCCACTTGCTGTTGTAAT

TGTGGTAGTATATTTTCTTTTCGCTGCAGCGTCATTTGCCTG

>Tb927.10.6820 | Trypanosoma brucei TREU927 | hypothetical protein, conserved | genomic | Tb927_10_v5.1 forward | (geneCodeEnd+0 to geneEnd+0) | length=282

ACTGTAGTGTTGAAAATTTGGACAAGTGAGTTTGATTGTGGAGTTCGCTGTGATACACTC

TTCGGAGAGAAGGCAGAGGATAATAGAGCAATTCCACACACTAGGTTGATTTGCCTGTGG

TGCAAACTATATGCCGTTGCTTTCTATTGACATTGTTCCCTACGAAGCACAATTTTTTTG

TCATTTCTTTATTATCTTTCATGTTTTGCTGCACTTTACTGCTGTCAAGCTATCGGGGGT

TTGAAACTATCCTTTGAGTTGGCCTTAGGTGGGAATATTCGG

>Tb927.11.9230 | Trypanosoma brucei TREU927 | hypothetical protein, conserved | genomic | Tb927_11_v5.1 forward | (geneCodeEnd+0 to geneEnd+0) | length=282

GCTCCTTAACGCACTTAGCGTCTGCCTGCGAGTCAACTCCGTCGGTGGGTGTTGTAAAGT

ACGCATCGGCTAAAAAAAGAAAGCCGGCAACGGTTAAGTATAAAAAACAGAAGGTGGACC

GGATTCACAGAGCGCTAACCCAAGAGTCATTATCGCTACAAAGCAGAGGAAGCGGGTGAA

TTGCGGTTAGGGAAGTAAAGAGGAACAGTACTATTTTTATTCTTTCTGCAAGGTGTGGGT

AGAATATTATCTGAACTGTCCAGTTCAGTTCGTGTGTCTTCG

>Tb927.9.14440 | Trypanosoma brucei TREU927 | hypothetical protein | genomic | Tb927_09_v5.1 reverse | (geneCodeEnd+0 to geneEnd+0) | length=283

ATCACGTAACCAAAGCGTTAAGTGCAACTGATTGGATGGCGATACAGGGGTCGTTTCCCA

ACCTTTTTCTATTACCATCGTAGTGTGAAAAATTGAGAAAGTGACCGCCGAATTGCCATC

CAGTCAACTGTATTTAGTACCGTCTCTCCTGCGTATTGTGCATCAGAGAGTGCGGTTTCT

CTGAACAGTAGTTTTTTTTTCCAAGTCATGGATTGCAGCAAGCTCCTTTTGGAGAAAAGA

GAACTGCTGCTCTTGTGCTGGAGGAGGGGATAATAATATTGAG

>Tb927.9.3780 | Trypanosoma brucei TREU927 | Uncharacterised protein family (UPF0041), putative | genomic | Tb927_09_v5.1 forward | (geneCodeEnd+0 to geneEnd+0) | length=283

AATATAGGGTGTGCTGACTTGCGTAGTGAACACCGACGGTTCTTTCACTATATAAACCTC

TAGGAATATAAGTTATCGTTTACATCCCTTTGTTGGGGCGACTCCCCCGTGAGAGGCAAA

AGAGTGCATAAAGTCAGGAGACAGTGTGACGAATATTGAGTGAGATGAGTGTCTATATCC

GACTTTCAGTGTTCCCTACCCATGCATGATTAAGACCGTAGCATCTGAGCATCTACCGTT

ATTAACTATGTGAAGATGGTTTGACGTATCCTTTGAGTGAAGC

>Tb927.7.1670 | Trypanosoma brucei TREU927 | Eukaryotic translation initiation factor 4E type 6 (eif4e6) | genomic | Tb927_07_v5.1 forward | (geneCodeEnd+0 to geneEnd+0) | length=283

GAAGTTTATTGGGTGAAGCACACCATACACACAACATGTAATGCTGTATCATATGTGACA

CTCATATGCAGGAAGTGAATCGTGTATGTGTGCACATGGATTCAGTGAAGGTATTACGGT

GAGCTGCGATGGTGATGGCGATGTGGCGGGGCGGAAAAAAAGGGAAGGATCAATGAAGTG

TGTTAGAAAGTGATGGAAACTGTATTCTGGACACAACAGACGAATTTCTGTTCGTCACAT

GAAAGGTGTGACCGATGTTGTTGGTGACGATCGTCTTCTTTCC

>Tb927.10.5050 | Trypanosoma brucei TREU927 | Mitochondrial ATP synthase epsilon chain, putative | genomic | Tb927_10_v5.1 forward | (geneCodeEnd+0 to geneEnd+0) | length=284

AGAACTCTTTGATCTCAGGAACGTAGCGGAGAACATCTGTTCATCATGTGTAGGGACGAA

GGAACTAGCGAACTGAAAATTAAAGCTGTTAGGGGGTGTTGAACAGTGCGGAACCTGGTT

GAGCCAAATTACAGGGGAGTAGTCCCCGCAAAGCGGGTCGTGGTTCGGTGCGTTGAGCTG

CTGATCGGCGTGCAGAGGTGTTTGTGGCATGTGGACAGTTATGGCGTTTTCGTTTTTGCA

CGCTTGCAATTTATTTCAACGTCGCCTGTGTTCTATCGTGGTAG

>Tb927.9.11310 | Trypanosoma brucei TREU927 | unspecified product | genomic | Tb927_09_v5.1 forward | (geneCodeEnd+0 to geneEnd+0) | length=284

AAGGAAGGAGGTACGGAGAAAGTGAAGTATATGTGTGTGCGGGGCGAAGAGTGAGAGGAG

GGGGAAAAAAATGACGACCACTCCCAACCAAACCAAGCCCCAGATACCGTTTGTGGATGG

GGAGTGCAGAGGAGGGGGGAGGGAGGAAAGAGCAAACAGATGAAGGAAAGGTAAGGATGT

GAAGGGGGTCAAAACCGCGGGATGGATGAGGAAGAATGAAGAGTGTGGTTATTATTTTTC

TTATGCCTCTTCTAGTGAAACTGACGAAGTAGCTCAGGTTTGCC

>Tb927.9.3290 | Trypanosoma brucei TREU927 | 4F5 protein family, putative | genomic | Tb927_09_v5.1 forward | (geneCodeEnd+0 to geneEnd+0) | length=284

AATATGATTTGTTGCGCGGGGCTTCCCATAGGACCCATGAAACTTGTCGACCTATTTGCT

AGATACCTGATGGTTTCCCTGTTTTCTGCCAGAACACAACATTCGTTGAGCCTTCAGTTG

AACACCCAATGGTTTCCGGACTGCATGCCAAATACAAGAAACTGGGGGTTGCGGTATGCC

GTGTCTCCCCTTTTTAAATATGAGTAATGAAGGACACGACTTGCATTAATAAAGGACACA

CTATCCTCCACGATTCTGAGAAGAGCAGTTCAAAAGGAGGGATT

>Tb927.1.4730 | Trypanosoma brucei TREU927 | Ribonuclease H2 non-catalytic subunit (Ylr154p-like), putative | genomic | Tb927_01_v5.1 forward | (geneCodeEnd+0 to geneEnd+0) | length=284

GGAATGTTGGGTGTTCAGGAGGAAGTCACGTGGCTCATTTTCTTTCATTTGCATTACTGG

TTCATGCGCTGCTGTTGGGTTATAGAATTAATGGCGGTGAAGGGATTCACACACATGCAG

ACACACGTATATATATATATATATATCACATTCCTACACTCACGTGTTATTTCACCAATT

ATGCTTCAATTGAGTGGATGGGTAGGATTAACTACCTTGTGGTTTATGTTGTTGTTGTTC

GAACGTATACCCTCAACAACAGGGCCCCAATACGAGGCATGTTT

>Tb927.6.4800 | Trypanosoma brucei TREU927 | hypothetical protein, conserved | genomic | Tb927_06_v5.1 forward | (geneCodeEnd+0 to geneEnd+0) | length=284

AGTCCGTATCGCGCCACATGTGTGCATAATCTACTGACTGTCTATCCATCTATCTATTCA

TCTCTTTAAGTAGAGACGTATGCAAGTGCATTGTTGAGCGCATCTCGGCTTCCTCTAATA

GAAGGACAGCAGCTTTGCGCCAAACAAGGTGATTGTTGTGGACTACATATCACAAGCAGA

CTAAGCATAAGTGATGCATGAGAGATGGGAGATGATGATGAAGGGATGGGGCTTCTGTTG

TGTACCCCCTCGTTATCTTCTTTCTCTTCTTTTAAAATTTTTTT

>Tb927.10.13960 | Trypanosoma brucei TREU927 | flagellar protein PF20 | genomic | Tb927_10_v5.1 reverse | (geneCodeEnd+0 to geneEnd+0) | length=284

AGTAAGAAATTAGAGTGTTATTCCCATATCCATGTACAATCCTATACGCCAACAGATGTT

CTGTGCTCACTTGTAGTGTGCAAAATAGATCCAACCCCAACTGTGAGTGGTGCTGGAAGG

TCGAAAGGCAAGGAGTGAAAGAGATTTCATTCACACATGGAAATATTTGGCCGATCATCA

TGTAGATTACCTCGAAATTGATTTTGAAGCAGGTGATGATACACATCGGGTGTTTAGGTT

TCCTCCGTAGGGTGATGTGCTCCACTTTGGTACCGTAAGTACTC

>Tb927.11.6700 | Trypanosoma brucei TREU927 | hypothetical protein, conserved | genomic | Tb927_11_v5.1 reverse | (geneCodeEnd+0 to geneEnd+0) | length=285

ATCGCGAAGGGGTACCGAGATAAGGATATGCTTAATGTGGTGGGGTTAGGGTTAGGGAAA

TATTTCCGCTGGGAAAAGAATAAATTTTCTTCCAGGAAGGGGCTGTCGTGAGGGTCCCCT

CTCCTACGCTAGTTTCCGCAAAGTAGTGTGCTGGTTGCGTGGTTTTAAAAAAGAAAAAAA

GCATTTCTTCTCACTTCCCCATTCCCCCTTGCTTCTGTTTTATAGGAAATTACGGAGTGT

TACAAATATCGTTATTCTTGTGGTAATCAGTACCGAGTACAAAAC

>Tb927.10.200 | Trypanosoma brucei TREU927 | vacuolar ATP synthase, putative | genomic | Tb927_10_v5.1 forward | (geneCodeEnd+0 to geneEnd+0) | length=285

AAGGCATCTCTGCTCTATCATCTTTTTTTTTGTTCGATGCCACGCTTTAGGGAGTTAAGA

TGGATGAGGGAAAAAGGAAAAGGATTTTTTTTCATGAAATTGTTTCCACGAAGGGAAAGA

TATATATATATATATACAATGAAAACTAGAAGAAAAAACTGATGCTGCGCCGGTGAGGTG

CCGAGGGTTGAAGAAAGGTAATGAATAAGGGCGAAGGGACAAAGTGAAAAAGGCGCTGCA

ACTCGAAGCATTCACCCGGGACCATCTTCTGCTTACGTAAGTAGT

>Tb927.4.1770 | Trypanosoma brucei TREU927 | hypothetical protein, conserved | genomic | Tb927_04_v5.1 reverse | (geneCodeEnd+0 to geneEnd+0) | length=285

AAGAAAAGGAAGGATCAAGTGAAGCTAAACGGTGCGAGCATTACGTAGGGGGGGGGATGC

GTGAGATGGGTTTGGAATCCGGTGAAACCATACTGAAGGGATGGAAGTACCTTCGTTGTG

GGCACGGAGTCCAAGGAGTATTTTTGCATCTTCCATTTTGGGCATTACCCGTACTCATTT

GTGCGCGCGGTACGTGTCTCCACAGACACGTATGTGCTCCTTTCAGCACTGCATCCTCAC

CATCACTATGAACATTTCGGTCCTCACTCTAAGCGCATAACCGCG

>Tb927.10.4310 | Trypanosoma brucei TREU927 | prohibitin 2, putative (PHB2) | genomic | Tb927_10_v5.1 forward | (geneCodeEnd+0 to geneEnd+0) | length=286

GAGGGCATCTTTAGGAACTTCGGTTTGCGCATGCGTTTGGTGAATCTTAGCGGAACTGCT

TCCACTCCTCTTAGCAATGAGTAGGTTGTCAATGACTCCTTTTGTCTTTCTTCGGCGTGC

GTGGTATTGTTTCGTATGGGTGGAGGCTGGATGGCGAGATCGGTAAAAACCTACCACACA

TCGTGGAAACTCTGTTAACAGCTAAAAGGGTTGTGTTTTTCTCACTTGTGGATGACGGGC

AAAGCAAACCAAAAACACTTTCAGTATTAACGACATTCATGCACTT

>Tb11.02.5105 | Trypanosoma brucei TREU927 | hypothetical protein, conserved (POMP15) | genomic | Tb927_11_v5.1 forward | (geneCodeEnd+0 to geneEnd+0) | length=286

AGTTCGTTGAGCAGTTGGGACACTTGGAATACACCTGAGCATCTGGAGAGACAGGCATGT

GTGTCCCGAACACAACGGATACTTGTCTACGTATCCTAGCGGCAAAGTAGGCGCACTTAT

GCTCAACGCATGAACTGTGTAAAATAGCGTTTTGTCCGCGTTCGAGCGTCTTTTTTCAGA

GATGCGGTAGGCCCTTTCAACGATCAACCATCCCCCCTCTATTTAGCAATGGGTGTGAGA

GAGTTGCTACCCACTTTTGTTTACCGTGTTCGTGCTCCATTGTTTT

>Tb927.8.2050 | Trypanosoma brucei TREU927 | GDP-mannose pyrophosphorylase | genomic | Tb927_08_v5.1 forward | (geneCodeEnd+0 to geneEnd+0) | length=286

AGGGCAGTGCGTGGAAAGTGGTACTTGCCTTTTCGAAAAGCTCCCTGAAGGCAAAGTTGT

CTCTGGATTAACACAAAAGACGACGCCGAAAATATTTACGTCTACTGCGCATCTTCTACA

TAGACTGTCACCCAATATTTTGGCTTTCCGTGAGAAGTGCTACCTGAGACGACCCCCGCT

GATCGTTTGGAAGCGGTTTCGGCTTTTACCCCTCCATTTCATAACATCCATTTCGTTGTC

GGGGGTTTTATCTTCCATCTTCTTCTTGTCCTCCCGAGCAATCCTT

>Tb927.7.7420 | Trypanosoma brucei TREU927 | ATP synthase alpha chain, mitochondrial precursor, ATP synthase F1, alpha subunit | genomic | Tb927_07_v5.1 reverse | (geneCodeEnd+0 to geneEnd+0) | length=288

GTTTAGCTAGCAGGTCTTTGTTAGTGAATGAGGTTAGCCTCCGGTAGCACTTTTATTTTC

ACTTTTAGGTATAAAACTCCAGAGAATTGTACACGTGGTGGTTTTTGGTTACGGACCGGA

TGAACACATGGACGGTCCTTTCTTTATGGTTAGTAAGGCGTTAACATTCTTTGATCATAT

GGTGGAATATATATCGCGGGAGGCGGTGTGTTTGGTGGTTGTTGTTGTTTCGTGTACTTA

CCTCTGAACAGTAGAGAAGTGCGTTTCGCTCTGTCGGCTTCGATGCTC

>Tb927.1.2985 | Trypanosoma brucei TREU927 | ER protein Pkr1, putative | genomic | Tb927_01_v5.1 forward | (geneCodeEnd+0 to geneEnd+0) | length=288

ACACACAAAAAGGGGGTGAGAAAGGACGGGGCTAGCAGGCACTAGGGCTCTTAAAGCGTT

TGTGGGAAAGCGACTGTTGGAATGTAAACGGTGCTAGGATCGATTCGGACATGTTTCCCC

ATGCGATACGAATCCTTTCGCCTCTTCGCTTGCGCTCATATGGTTTTTCCAACACCCGCA

CCACTGGCCTGTCCGGTTTTCAAGAGTATTTTTGCGCAAAGGGCAACAGAAATGGCAGCA

ACCCAAGAAACAACACAATCTCTTTTCTCATACTACCTGTTATTCCCT

>Tb927.6.3050 | Trypanosoma brucei TREU927 | aldehyde dehydrogenase family, putative | genomic | Tb927_06_v5.1 forward | (geneCodeEnd+0 to geneEnd+0) | length=288

AGGTTGGAGGAGTGTCAGCTTAATAATAATTTACATGAGAGACGAAACGATATATATATA

TATATATATATATATATATATTACGAAATAACTCTTTGTCACCATAATTAGCGCGACGAC

GTTCTTTCATTTTTTAGTCAATGCATCCGACTGTTCTGATACAGTTTACTTTAATCAATA

TATATCTTTTTTCGCTTTTTTCACAAATGTGTGTGTGCGCCTTAGAGAATTACCCATCCG

CTAAGCAAAATTAACTGTGAGTATATGCCACCGTGTCAGACGAGGTTG

>Tb927.10.6020 | Trypanosoma brucei TREU927 | hypothetical protein, conserved | genomic | Tb927_10_v5.1 reverse | (geneCodeEnd+0 to geneEnd+0) | length=289

AAGAGACACGCGCGAGGAGTGGCCGTGTCTAACTTCTCCGTGATCTTCACCTTTTTCGTT

TCTTGAAATACTCGTTAAGGGTGCCCTCGCCTCCGTTCCTGATTTCTATTCCTCATATAT

CCTATGCCCTCCCCTATAGACTCTTTCAGTTCATTTGCTGTATGGTGAAAAAGAGTGTTG

AGGAAACAAATGGAGGTCGCGGTGTGTGAAATCGTTCGCAATGTAAGGCTGCGTTACAGA

CGGAGGAATAATGGAACCACCAAACGCGATGCACGCAAGTGCGGGACGT

>Tb927.1.1030 | Trypanosoma brucei TREU927 | leucine-rich repeat protein (LRRP), putative | genomic | Tb927_01_v5.1 forward | (geneCodeEnd+0 to geneEnd+0) | length=289

AGCGAAACGCATTGCCGACACTGTGCCTCAGCTTCTCGGTGCGACAAAAGAAGTGGCCCA

GGTGTGCCGATGGAACGTCTTAAATTTTTCAAGTAGTACATCAGTTAAGTTTTACACGTA

GAATCTCTCTGTATTGTCGACAGACACTGCGTGTTTGCCTATCTCCTCATACCTTGCGTG

TTGCTAAGGATTGGTGATAACTTTTTCCCGCTACAACAAAAATATTTGTTTGTTAAAAAC

CTATAATCAGAACCATATTCTGCCGGTTAGTTGTATACCCACATTAGTG

>Tb927.11.6540 | Trypanosoma brucei TREU927 | hypothetical protein, conserved | genomic | Tb927_11_v5.1 reverse | (geneCodeEnd+0 to geneEnd+0) | length=290

ACAATGAAGTACCACTGGCATGACGATGGCGGCGCCTGCCGCCGAAACGAGGAGGAAAAC

ACAAGCCCCCCTTTTCGGGAAAAGGAAGAGAGGTTTCTCCACCTTCGATGCACCAGAGGG

GAGCGCCCGGGAATATGAAGCGACGGCGTTTGCTTATTCTTTTCCCACCGGGGACGGTCG

TACAGGTGCGTGTGGGCAGCACTTCACGATAAACTGATATCCGTTGCGTTAGACGTGGAT

CCTTACCTGTTCCTATTCTTTTTGGTCTTCAGTTAATTGTGAAGAATGGT

>Tb927.11.600 | Trypanosoma brucei TREU927 | hypothetical protein, conserved | genomic | Tb927_11_v5.1 reverse | (geneCodeEnd+0 to geneEnd+0) | length=291

GTAGGAAACAATTGGGCACCCCTCCCCTTCTCCCCTCCTTTCGGTCCGGGATGGTGGAAA

AGGGACACACTTGGGTGGGCGATGCCATAATAGGAGAATCGTAATGGTCTGAGTTGGTGT

AACACACTGTTCTTGTTGTTGCGAAATGTGTGGAGTAGTGGAAGAAGGAACATATTTATA

TATTTTTGTCTTTTCTTTTGTCGTTATGTCGTTTCAGCTGATGGTGTGCACGCACTTTAC

TTGTGCGCATCGCGTGGTCGTTACTACCTGACACAGGGTGTAGGAATGTGG

>Tb927.10.6080 | Trypanosoma brucei TREU927 | proteasome subunit beta type-5, putative, proteasome subunit beta type-5, putative | genomic | Tb927_10_v5.1 reverse | (geneCodeEnd+0 to geneEnd+0) | length=292

GGACACGACTCGTGAGGAGATGTCTGTGGGTCGTCGGTGTAGAAAGGGGGAGTTTTCGAG

GCAACCGGGAGGAGTATAGGGAGGAAAATAGTACCCTAATGGGATGTTTTGTGTGTGGAA

GGGGGTGGGGTGAATAAGCGGTGCCGTGAAGAAATGAAAGGAGCTGCAAGTCGGAAGGCA

CTTCTCCACACGACTGTTCCTGCGTTGATGAGAGGTGCATCTTATCACCGAATGCTTTCG

CAGAATGTCCCTGACTCCCATTTTTAAGGCCTCATCTGACTATGTGTTGAAG

>Tb927.8.5800 | Trypanosoma brucei TREU927 | hypothetical protein, conserved | genomic | Tb927_08_v5.1 forward | (geneCodeEnd+0 to geneEnd+0) | length=293

ATTGAGTCTGCAGAAGCCTCTGAGGTAAGTGGTGAGTGACCTGCAGATTTTTTTTTGTGA

GGTACGTACGCTGCAGATGCGCTGAGGACAATATCATCGCCTTCTCCTTTTTGGTATGCG

GAGGAGGGTCACTCAAGTTGAGGTTCATGTGAAGGGGAATGAAAATGTGTGCATGTGGAA

ACTTCTCTTTCTTGCTCATGCGTGTCTGTAAACTATTACAGCGATGACCGTACTCTTGCT

GATTGCTCAGCAACGTCGGTAGTGGGCACCTTGTTCCTCTGATATCATCCGCT

>Tb927.8.6460 | Trypanosoma brucei TREU927 | kinase binding protein CGI-121, putative | genomic | Tb927_08_v5.1 forward | (geneCodeEnd+0 to geneEnd+0) | length=294

AGAAGTTGTTGGGTTCTGTTGAAAAGTGCTAAAAGGTAGTGAGTGGCAGGGCGAAGCAAT

GGAAAGTGGGTCTAAAACCGAATTGTGGCGAGAAACGGTTCCATTGCGTGGCGGCAGAAA

AGAAGGAATACAAAAAAAAAATGATTGTGGGCACCTGCGACGGCTACTGCAACTGCCATG

GTCACTTTGATGCCTCTCGTCACTACAAGGCCGGTTGTCGAGTTGTGTTGTGCTGCCGGG

TCAGGACAAACGGAAAACGAATCGAGGAAAAGTTTCCGACCCCCTGTGTTTTTT

>Tb927.7.3140 | Trypanosoma brucei TREU927 | hypothetical protein, conserved | genomic | Tb927_07_v5.1 forward | (geneCodeEnd+0 to geneEnd+0) | length=294

ACCCCTCCTTCCCTCCTTCCACCCACCCGCTCATCCCTCAAAAAAAAAAAATGGTGATTT

TTTTTTTTTGAGCAGGGGGCGAATCTGTGGGAACGGAAAAAAAAAGGTAAGAAGAGTATT

CATTTTGTGGAAAAGAAATGGGCTGCCACGATACTCGGGCCATTGTTCCCCTCTCAGACA

CGCGACCACAGCTTTTTTTTCACCTCTGTTCACTATCATCAGTTTGTTATTTTGTCGAAC

GACAAGCGTTGTTTGTCACACATGCGCGACTATGGTTACTACTTTCCGACCTAG

>Tb927.10.4090 | Trypanosoma brucei TREU927 | hypothetical protein, conserved | genomic | Tb927_10_v5.1 reverse | (geneCodeEnd+0 to geneEnd+0) | length=294

AGGCGTATGTCGTCCATTCCCTTTTATTACTGTCAGTGATTGTTGTAACTTGCCGTTGCT

GTTTACGCTCCCCACAGTCGATATGCTGTTTGAAGTTATTTCCTCATTCGGTGGTGCTGG

AGGGACCTTGGACGGTGACCGTGTAAGTGATACTCCACTGCCACTCTAGGAGGTGGAGTA

TCGAGGTGAGTTCCATGTATGAGGAAGCTATCTGGAACGGTAGGGTGCCGTTATTATTTT

CCGAATACTAAAGAGGGCCAAAGATTGGGATAAAATATCATGGGACCACGACTG

>Tb927.6.3290 | Trypanosoma brucei TREU927 | intraflagellar transport protein 20 (IFT20) | genomic | Tb927_06_v5.1 forward | (geneCodeEnd+0 to geneEnd+0) | length=294

GCTGGACACAGTTAACATGGATAGAGTGATTATGTTTACGGGTGTATGTACGATGAAGGA

AAGGGAAAGGAAGGGATGACAGCAGCTAAAATATTATTTTATGTCACGTATTCCTCTCCC

CTTCCCATGTGCCCCTTGTCTCGTATGCTTCGGTTCACATCTGGGAGAACAAGGGGGGAA

GTGGAGGTAACATTAACGGTGAGTATAGATTTTTGTTTATGATTGCTTGATGGTTGATGC

GGGTAGTTCTTTTGTTTTTGGGGCTACTCATGCGATCTGTTTGTTCATATATAT

>Tb927.10.6670 | Trypanosoma brucei TREU927 | dynein light chain, putative | genomic | Tb927_10_v5.1 forward | (geneCodeEnd+0 to geneEnd+0) | length=295

ATCGACGAATGTATTTACTTGTTGAGTGTGCTCGCGTGATGATCGTTTGAAGTAGGCAAG

GCGTTCGCCTGCTTGACGTTATCCATCGGCTGCGGGATTTTTCTTTTCTTTTTTCTCATT

CTTTTTGACGCCGTTGCGTTCGGTGTTTTGGATGGACTTTGAACCAAGGGGATTAAACGG

ATTGCTAGAGTTAGAGAATCTTTCCCGTGCTGTACCACTTAAGTTACGCTTATACCTACT

AGGGACCTTACAGCCTTTGTCCCGTGGAGCATGTACCGTATACCGGAGAACAAGG

>Tb927.11.14870 | Trypanosoma brucei TREU927 | NAD+ synthase, putative | genomic | Tb927_11_v5.1 reverse | (geneCodeEnd+0 to geneEnd+0) | length=295

GGATAAACTTAAAAAAAAAATAACAGCAATACTAAACTTTGTTGGATGTTTCAGTTACCT

TTTACCTCCTGGAGGAGCTGAAAGGATTACTATAATAAGGTCTGTAGTATTGCATATTTT

GTGCTGCCTTCATGCATTGATGTCCGCTTGGGACGTTGAGCAGTTAAGCATCGTTTCCTC

ATTGCAACTGTAAGTTGTCTGTTATGTCTCTTTAAAAGGGGGAAAAGAGCTTCGTTACAC

CCGCATTTTATCCTCCCCTTCCAGTTTACTTTTTTTTTGTGTGTGTGTGTTTTCC

>Tb927.11.13870 | Trypanosoma brucei TREU927 | uncharacterized protein, PH0010 family/AmmeMemoRadiSam system protein A, putative | genomic | Tb927_11_v5.1 forward | (geneCodeEnd+0 to geneEnd+0) | length=296

AGTTATAACTGATTAGCGTGTTGATGCACTACAGATTGTAGCGGGACTCCGGTTCCGTTC

CTTCGTCTTACGTTTGCGCACACAACACTGACTGTGTTGGGGTGTGGGGTTGTATATCGG

CAGCTTCGTTCCTAAATCTTGGCCACACACCAAGGAAGGGAAACAAAATAGCTGCGTTAA

AGTTCTGCGATTTACACTCTCCTTTACTTTTCCCCTTGTTAGTCTACGTACGCGAGTCCT

CAAGAGTACAACATATGCAGAAGCGAGGCACCAGTCGTGCATTAGGGATGCTTTTG

>Tb927.10.3520 | Trypanosoma brucei TREU927 | protease regulatory ATPase subunit 4 (RPT4) | genomic | Tb927_10_v5.1 forward | (geneCodeEnd+0 to geneEnd+0) | length=296

GCTTCCGTTGGCGTCGTGACAGTGTGTTCTAAAGAGGAATGGTGTGCTCAACTGTAGAGA

AACGAGGTGCGGTGTCGGATGAGAAGAAACGAAAAACAAACAGGGGAACACATTAATGTG

CAGTCGTTTGGAACAGGAAGGAGGAAGAGCATGTGTCGTGTGTCGTGTGTTCAATGACTG

TGATGTGGGTGAAATGCAGGAAAAAACTCTGGAATTTCAATGGTGCTCCAAAGGGAAGCA

AGGGAGGAAGGGGATGTGCTTGTGGTTACGTAAATCGGTGGGATATTCGCATTGAG

>Tb927.11.1620 | Trypanosoma brucei TREU927 | hypothetical protein, conserved | genomic | Tb927_11_v5.1 reverse | (geneCodeEnd+0 to geneEnd+0) | length=297

AGCACGGAGATGCGTAGTGTCCGGGAATGAAGCGCTTGAATTTTCTTTTTTTTTTCCATT

TATTGTGCATGGGTTGTACTAAGACCTCGTACCGTGATTTCAAAGGATTTGGATGTTTGA

CGTGTGGCCCATGGCCATTTCAGCGTGAGGGTGAGTGATATATGTCGATAACGAGATTGT

GGAGGGGGGGGGTCTGTGATGAGTGGTAAATGTTTCAGCAGGGATTTTTTCCCATTTAGA

AGCGGCTACCATTTTCCCCGTTACCTCCGTTTCATATTATTTGTGTTTACAGTATTT

>Tb927.10.3120 | Trypanosoma brucei TREU927 | cytochrome c oxidase assembly protein, putative | genomic | Tb927_10_v5.1 forward | (geneCodeEnd+0 to geneEnd+0) | length=297

GCACATAAGCGACTACTTGTCCACATGGGGAAACTGAAGAGGCCTTAAAACCGCACATGT

TACGGAAAGACGAGGGCTGCATGGACTACCAGGCGGTTTCGTACGCTGTGTGAATGAGTT

GTAGTTGTCAGTCGCGGGTAGCTACCTGTGAAAAATGTCTCAGATTTTTTTTTTCCGACG

CGCGCCGCGTGTATTTTATCGTGTGTGGCGCAGGACGGTGTCGCCTACCACTTACCGGAC

GTTTATAAAGGTCAAATGCCTCCCTCACCCTCCGTCACATACGTGTATGTGCGTTCG

>Tb927.10.14760 | Trypanosoma brucei TREU927 | clathrin light chain, putative | genomic | Tb927_10_v5.1 reverse | (geneCodeEnd+0 to geneEnd+0) | length=298

GCGTGACCGTGACCACAATACCAATAAGGCTGTTTGTATGCACAAGAGTGAGTGTACGTA

CTCCAAAAATAAGTGGAAGCGAGGACGATTATGTGGCGCCCCCATTTTTTTGCTTAAGCA

CATCTGACTTGCTTCTTCTCTCGCCCCGCTCGTCCATGCAGGAAAGCAGAACTGAAAAAA

AGGAAGCGGGTGCTTCATGAAATATACACAAGCGCAATTTATCCTCCTTTTGTACCGGGT

CTGGTGACGCTGAATTACTATTACTTGCTGCTATTTCATCTATTGTCCGTCACTGCAT

>Tb927.10.14810 | Trypanosoma brucei TREU927 | Multiprotein-bridging factor 1, putative (mbf1) | genomic | Tb927_10_v5.1 reverse | (geneCodeEnd+0 to geneEnd+0) | length=298

AATATCCAACTCGTTTTACGACTATGTTCATGCGCCTATCACGCTCTGTTTCGATGTTCC

TGCATGTTATTCCGTTATTTACAAGTTATCATTGTTGCGAGTGAAATGTATGATGTTACG

GGAAGGGTGGCGACGATCAGGAGTGATATTTGCATGCGCGATTGTGAAGGTAGGGGAAAG

GGGAGCCGGCAGGGGTAGGGGATGTTTTTTTGTCCCTCCTAATTTACTTTTTTTCTATTT

GAATTCACAACACTGAAGCTGGCGTGGTTACTTCCTCGCAGCACGGGGTACGTTTGTC

>Tb927.7.4620 | Trypanosoma brucei TREU927 | hypothetical protein, conserved | genomic | Tb927_07_v5.1 reverse | (geneCodeEnd+0 to geneEnd+0) | length=298

AGACAGTGCTCACTGTCCACAGGCGGACCATGATTTTTACCGAATGGTGTATCTTCCTTT

TTCCACTCAGCTTGTAAGCGGGAAGGTTCGCTCTGTGTGACAAAGAAAAGACTGAAGGAC

GCATATAACCTGATACAGTTTCGCTTCGAAGGAATGTTATACACATGGGGCGCTCCTTCG

GTCTTGCACATTGTGTTTGGATTCTATCTGGCGTTTGACAGCAGCTACACGAGGTTCAGG

GTTACGACTGTGGGTGGGGCATCTATTTTCTTGTGTTTTTTTTTGTAACCTTACTAAG

>Tb927.10.14700 | Trypanosoma brucei TREU927 | hypothetical protein, conserved | genomic | Tb927_10_v5.1 reverse | (geneCodeEnd+0 to geneEnd+0) | length=300

ATCGTTTTCCAGTATGGGAGCTGGGGGCGCGGGTCCGCACGAGCAAGGGAGAGGGGAAAT

AGTAAAGGAATGGGGAGACGAAAGAGAAAAAGGCCAATGAAAGGAGTGAGGGAAGAATGT

TCCACCTTAAGGAGAGAAAAGTGATGGGCAATCCAAGGAATGAATCGAACGACGCGGGCG

TTTTACTTTCTTTTCCCTTTGCGGTTCCCGTTTTTTTTCTCTGTATATCTAATAAGAAAG

GTTCTTTACAACACCTCGCGGTTTACAGTGTTAATATATCTGTCGATTGGTTACAGCGAT

>Tb927.10.4990 | Trypanosoma brucei TREU927 | cdc2-related kinase 3, putative (CRK3) | genomic | Tb927_10_v5.1 forward | (geneCodeEnd+0 to geneEnd+0) | length=302

GATCATTATACATAATTAGACATCATTTGTTTTCGCTCGTTGTTCCAATCAGATTTTAGA

TGGCATGCTTTTTCTGTGCAGTTGAGAGCCCAGTAGAGCGTTTTACATGCCCATCCTCGC

CTAAGCAGGTATGTCTTATAAGAGGATGTGTCGGTCTTTGAGGGTCTTTTCCTTTCCTTA

AAAGCTGTTCTGGACGTATTTGTTACAGTAGATCAACCTGTGCGTTCTATCTTCGATACT

TTTTTCTGGAGGAAACAAGTACAATAACGAGGAGGGAAGAAAATAAAACTTGTTCTGTGC

CC

>Tb927.4.750 | Trypanosoma brucei TREU927 | 50S ribosomal protein L7Ae, putative | genomic | Tb927_04_v5.1 reverse | (geneCodeEnd+0 to geneEnd+0) | length=303

ATTACATGGGAGCGACCTTTTTATTCTGTTTGTTTGGACGCGTGCGTGTGGACTGGCAGG

GGGATGAATGACAACAAGGCGCGGGTGGTATTGAGAGGCAATGCGAAGAACTTTTTTTTT

TTTCTTAGATAAAAAGAAAGGAAAAGGGGAACGCAGTGCGAAGCAAAAAAAAAACAACAA

CTGCAATGAGTGAAGATATTGAACCAATAATGCAATGTTTTGTGTCTCATGGCATTTGAC

GAGTTTAAGTACCTAATGTTTACTGCAGCGGAATAAACTATACGTTCTTAAGCATATACG

CAT

>Tb927.7.4710 | Trypanosoma brucei TREU927 | 39S mitochondrial ribosomal protein L46, putative | genomic | Tb927_07_v5.1 reverse | (geneCodeEnd+0 to geneEnd+0) | length=304

AGACACCATGCATATGCATACGCGGCCAACAGGGAGAGCGTGTGTGCGTGTGCGGAGCGG

AGCAGTATGGGGGTCGTTATGTTATGTGGCACAGAGGGAATGAAAACGTGTGGTGTAAAT

TGTGCACACGGCGTGTTGTCACAAGTAGGCAAGCTACAACGTTGGATACTATGTTTTGTA

AGGTGTACAGCCTGTTGTTTAAAAGGGAACTTGGATTCCCTCTGACGTCTTTTTTCTCAC

CACTCATTTTCCCTCCTTGTCGCCATTGGTGTGAGGGGGTTGGTCGCACATTGATAAGTG

TTTT

>Tb927.3.5500 | Trypanosoma brucei TREU927 | RNA polymerase subunit, putative, DNA-directed RNA polymerase II subunit 3, putative (RPB3) | genomic | Tb927_03_v5.1 forward | (geneCodeEnd+0 to geneEnd+0) | length=305

AAAGACGTTGCGAAGTCTCACCACATGGGGGTACATATTCGCACGCACATCCGTACACTT

TTCGTGGTAACTCCTCTGCTTGTTTACTCATAATTCAAGCGTCGGGTTACCTTGCGATGC

TCCTGTTTCTACGTTAATGAGGATGTCGCCTTGAGTAAAATACCATGGGTTTAACTAATA

CAAACTTTCCTTGTAAATCATCTTTTTTTTTTCTTTGCTCTTTGTAGGTAGTAGTGGTCT

GGGTTTGGTGGGCAAGTACTCATTGGGAATCGTGGAGGGTAACAGAGAGAGAGAGAGTGC

TGGAT

>Tb927.11.14320 | Trypanosoma brucei TREU927 | hypothetical protein, conserved | genomic | Tb927_11_v5.1 forward | (geneCodeEnd+0 to geneEnd+0) | length=306

GTAAACATTGTGTGAGTATTGCTTCGTGTCACATTTGCTGTTGTGTAACGGGTGACAGAT

CTATGGTTCATTAGTTTTTTTTCCGTTTTGGTTCCTTTTATTGTTCAGGCACACGTGTTT

GTGTGCAGGTTTGTTGTGGGTACCAAATCGCGGAGCTAAGTGAGCGCGCTTGGCTTGGGA

GCCGCTATTTATTGTTTTTGTTATCGGTTACGGTATATTTGTTATTAGCAGTAGACGTAC

GCGAGCGGGGTGGGAAGAAAAACGTTGGATAGCAAATTTTTTAATGAGATTGTTTTCTCA

TGCGTG

>Tb927.8.5040 | Trypanosoma brucei TREU927 | EMG1/NEP1 methyltransferase, putative | genomic | Tb927_08_v5.1 forward | (geneCodeEnd+0 to geneEnd+0) | length=307

GCGTATTTGTATTTCCTCTACCTTCTTGTGTTTTCCATCGATTAACTGCTCCCCCTTTTG

TGCAGCCACTGCCTCATGGGGTGGGAGCGAGCGGTGATTTTGCGCGAAGGGAGCAAAATT

GCGCTTAGCGTGTAAATAAAAGAGTGCTGGTAACGTTTGTCGTAACCAAAAGTGTCAAAC

TTACACACAGGAACGCGTGCAATGCAAAGCGAAACGGTGAGGGTCGCGGAGTCTGAATTA

TCACCGGTGCCCGCCGCTGTGGAGGGGTTTGTGGTATCAAATAAAAGTAATATTACTTTC

GAATAAT

>Tb927.7.5010 | Trypanosoma brucei TREU927 | hypothetical protein, conserved | genomic | Tb927_07_v5.1 forward | (geneCodeEnd+0 to geneEnd+0) | length=308

AAGATAGATGCCTTTTTTGTTCATTGCTCTTCCATCTGATGTCGGAAACCTGTGAGGTGA

TTGGGTGAGATGTTTCCATCCTTCTCTGAAAATGCGTGGCCAAATATTTCTCCCATCCCT

CAGGTACGCATCTACGCCTGCCGCGGCCGTCGGATGTCAGCATGCCGTGGTGGTAGTGAG

GTTAGGGTTGTAACTGTCACCGATTCACGGCGGTGAAGGGGTCGATGCTTTCGTTATGTC

TCCTCTCATTCCTCCCCCGTACCGACATCCCACCGTATCGCTCTCCACCCCCTTGTTTTT

TGTTTAAT

>Tb927.11.8260 | Trypanosoma brucei TREU927 | carbonic anhydrase-like protein | genomic | Tb927_11_v5.1 forward | (geneCodeEnd+0 to geneEnd+0) | length=308

AATTGTTCCTCTGTGCGTTTGCTTTTGTGCCGAATGTGACGACTGAGTGAGTCAGGAAGT

TATGTGTGTATGTGCAGAGGGCGTTATAACTCTCTTCACACCAATGTCGGGTGGAGTGGG

TTGTGTGATATCTTGCTTCTCTTCTTTGTTGTCTTTCTCCTTAATTGTGCGGAGAGGCGC

GCGAAGAAATCGTTCCCCTGACATCTGTATCCCTACATCCTACACCATAAATACTGTCAA

ACGTGTATGGTGCCCAAACGCACTAACATTGTATTTAACGTCCCATGTTCAGAAGGGCGT

ATCACACG

>Tb927.11.1710 | Trypanosoma brucei TREU927 | mitochondrial RNA binding protein 1, guide RNA-binding protein of 21 kDa (gBP21) | genomic | Tb927_11_v5.1 reverse | (geneCodeEnd+0 to geneEnd+0) | length=309

ACGATATGAAGGAAGTGGGTGTGTAAATGAACGAATGAAAAGGAAAGGAAGGGAACAGAA

AGAAGAAAGAAGAAAAGGGAAAGGTTGAGGGATGAGGTCGTACGTATGTGTTTGTATTTT

TGTGTAGGATAGACAACAGATGTGTTGTTGGAAGGAATTGAGTACCAAGATGTTTGGTGC

AATGCAAGTGAGCACCTACCGTCTATTTGTTTGGTTTAGTGGTGTTGGGTTGAACCCGCT

TTGGAACTTCTTGCGTCTTCCTTCCGCATCCGACTTTTCCCCCCAACCCTTTAATCTCCT

TCAAGAAAG

>Tb927.11.10220 | Trypanosoma brucei TREU927 | hypothetical protein, conserved | genomic | Tb927_11_v5.1 forward | (geneCodeEnd+0 to geneEnd+0) | length=310

GTGGTGGTGTTTGCTGCTGCGGGGTGGTGTTTTTTGTGGCCTAGTTTCCCTAGACACGTA

CAGTAGAGTTAAATGGGCGCGCGCATTCGTTATTTTCCAGTGGAAGGCATGATGAGACAG

AAGAGAAGCAGTGGGTTCCCCTTCTTTTTGAGTCTGTTCGAAATGATGCGACTTTCTGAT

GTATTTTTGCTCTTCCACATTGTTTGGTGAAGAATGCCGAGGTACTCCGTTTTCTGTACC

CCGCGGTGGGCATCATATACCCAATTTCGAATTATGTTTCTTAGTTTTCGCATCTCCCCC

CACTTTCCAC

>Tb927.7.3520 | Trypanosoma brucei TREU927 | mitochondrial pyruvate carrier protein 2, putative | genomic | Tb927_07_v5.1 reverse | (geneCodeEnd+0 to geneEnd+0) | length=311

GTAAGGAATGGCATATATGAGTATCAGTGTGAGTGTGAGATGCGGTCATTTGTAATGCCC

GTAAAACAGGGAGAGAAGTGCGTACGAGTATTTTGGGGTATCGAGGAAAAGAAAGGAAGG

CGGTGAACGGCTCCTACCGTCTCGGGGAGTATATAAATTAGATTTAATGAATCGAAGGGA

AGTCGAAGGGAAACGAAGTTAACGCACGCGTATATTCGCGCGATTATGAAAAACGAATGT

TTGATATATCCATGCAGTTTTCGTTTTGAAGTGGGTCCACTACCGTACGACGTAAGTCAA

GTGAACGTAAC

>Tb927.10.5130 | Trypanosoma brucei TREU927 | Protein C21orf2 homolog, putative | genomic | Tb927_10_v5.1 forward | (geneCodeEnd+0 to geneEnd+0) | length=312

AGATACGCTACGCCACATATGGTTTGTTCTCGTAGCGCTCGCCCCCTTCTTGTCTTTGTT

GCTGTAAACGTTTGTGACCATGCGGTGTTTGGGGCGTGCGGTGCAAAGTGGGTTGAGCGT

GCACTGGTCAGTGACTACGGATATTCCACATTTGGTCTTTACTGTTCCCAGTGATTGACG

TGGAGGAAAAAGCGTGAGGTCGCTCCTTCCATGTGCTCCCTCTTTTTCTTTGCCCATGTG

CATCGAGTTATTTGCCCTCCTGTACTTCTCTCCTTCACGCGCGCAACCAAGCGATTACTT

TCACAACAACTG

>Tb927.7.3120 | Trypanosoma brucei TREU927 | small nuclear ribonucleoprotein SmD1, SmD-1 like small nuclear ribonucleoprotein (Sm-D1) | genomic | Tb927_07_v5.1 forward | (geneCodeEnd+0 to geneEnd+0) | length=313

AGTGTTCCTGCGGCCGAGGGACGGGACTCGTTTTTAAGGGAAAGAGCGGGAAAGTGAAGG

CACACACGAAGGCGCAAAGAGAGGAAGGGAGCGAGAGAATTCCTCTTTCCAGAGATGCGC

ACTGCAAAGGAACCCCTCCATTTCCTGTTTCTCTCCTCGTCGCCGCTGCCGTCATGTTCT

TTTTTTCGGTTTCTGGTACGTATCTATGACTCCGAGGGGAGCAGAAAAAAGAAAAACAAA

ACCGACCAACACTTTGGTGAGCTACTTTCTCATTACTTCGTTTGTGCCCCTGTGCTACTG

TTTGGCTTCTCCC

>Tb927.8.6390 | Trypanosoma brucei TREU927 | lysophospholipase, putative, alpha/beta hydrolase, putative (TbLysoPLA) | genomic | Tb927_08_v5.1 forward | (geneCodeEnd+0 to geneEnd+0) | length=313

ATTTTTGTGGAGTTGTAAGAAAGAGAGGTGAATGCCGGAAGCGGCTGCCACAATGTTGAG

GAAAGTAAATATGGCGCGTCACCTGAGAGAGAGAGAGAGAGAGGAAAGGGGGAAAAAGAA

AAGATAGAAAGAAAGGTCAGAAATGCAGGAAAATACAAAAATAAGGATGAGTTCACGAAT

GTCACTCCCAGAAGGTAACGTAACTATCGCCAACAGGTATTGCTAGTCCCGTTGGCAGGC

ATGAAATGAGTTTTATTTAACCAGAATGTTTGCTGTGGCTCATTATCACAGTCGTTTCTT

TGTTTAACTTTGG

>Tb927.11.10050 | Trypanosoma brucei TREU927 | hypothetical protein, conserved | genomic | Tb927_11_v5.1 forward | (geneCodeEnd+0 to geneEnd+0) | length=314

GCAGGACTTCTCGTCCCCTCCCTAACATTTATCTCTACTCGGGACTGGCGCGTCGTTGCA

TTCGCTTACGCCATTTTGTTTTTGTAGCTCATTATTTGGCAAGTATCCGGTAATGAAGGG

AGGGGGGTATGTTTCGTGTGTATTGTTGGTCGCAGTAAACTTATTCCGTTACGGGTTCTT

CTGCACCGTGGTGGAGTCAGTGATGATAGGGTTGTCGTTGCAAGGCGGTGTCTGTTGGCC

TGCTTTTTCCCTACACAAATGTGGCAACGGTTTAGTCCATACGAGGGACTTTTCGTTGGT

TATCGTAAGGGCAT

>Tb927.6.4790 | Trypanosoma brucei TREU927 | hypothetical protein, conserved | genomic | Tb927_06_v5.1 forward | (geneCodeEnd+0 to geneEnd+0) | length=314

AGGGGACATGTTTAACATACCGGCGACCAATGCTGCCGGAAAGATACCATATTTACACAC

ACACACACACACACGTATATATATATATATATATATATATATGCATATTGCTTTGGTGCT

AGTAGGGAGTTGTTGAGTTATGGTGGCTGGGACAAGGCAGAACAAACTTGCGAGGGGCGT

TTTACCGTGTGTGTGTGTGTTTGTGTGTGTCCACCACATAAATCCTATATGTTGTGTTGG

TTAGCCAACCTTCAGGACGATAGTTGTGTTCTACCTTGTGGTGTGATGATGGGGTTTGCA

CTTTCCTTTCTCGC

>Tb927.10.4260 | Trypanosoma brucei TREU927 | hypothetical protein, conserved | genomic | Tb927_10_v5.1 forward | (geneCodeEnd+0 to geneEnd+0) | length=315

AAAGCATGGCAAGGTTCTCCATCTTTTTTTCTTCAGTCTTTTGCTTGGAGGATGAATGAA

TGTGAATGGGAACGGATTGGTGTGTCAGTGGGTGTAGTCACCAAACTGTTTTGCTTTTCT

CACTTCCTTCCCCTTTTTATCTTGTTATGCCGATATGCATTCCTCTGTGCATAGTGCACC

ATTTGTTCCTTGCTTCCCAACCTTTCGCCGTGCAACCACGCGATGACGCTAACTTCAACA

CTACCGTGGGTCATGGCAAAGTGGTAACGAACAAAGGAGGAACGAAGTGAACCGAAAGTT

TATACATGATGATGG

>Tb927.10.7140 | Trypanosoma brucei TREU927 | membrane-bound acid phosphatase 2 (MBAP2) | genomic | Tb927_10_v5.1 forward | (geneCodeEnd+0 to geneEnd+0) | length=315

GTGCATTGTTGTTTTGTGTTTGTGTGTATGTGTTTCCGTTTGGGGATGACGGGGCATTTG

TAGACCAGGGAAGCCGCAGTATGATACTGGTATCTGACTGTTCTGATGCATGTGGTTTGT

CCGCTGCTTTCCTCCGTTTAGTCCGGCTCCGTTCGACCTCAAGCTCCTCATGCAAAGAAC

GTAACTGCCGAATACCGTAGACGCTTAGTTCCTCCGGTGGATGCAGTGTAAAAATCGGAC

CAACAACCAGTGATATAACAAATCCCCACCATCTTCCTCTGGGACTGGCCTGTTGTAAAT

AAGTTTGTTTGCGTG

>Tb927.9.12290 | Trypanosoma brucei TREU927 | Peroxin 19 | genomic | Tb927_09_v5.1 forward | (geneCodeEnd+0 to geneEnd+0) | length=315

GTGTCACTGGCTGGTTGAGCTTGAATAGGATTAAAAAGGAAGTAGCTGAAATGTTCATGA

CCGTCAAAGCGTCGTGTTTTTCATTCAAGTATGAGGGAGTAATAACATTGAAGGAAGTGT

CACATTCGGAAACCAGATGTAATTACGCGGTGCCGCTCCACGCCCCTGCTTTCTGTTTTA

CCGCTTTTCTTTCACTTCTGAGGTATGGGGTGTAGTAGTGGTGCGGTTGCATCTGCTGCG

TGTGTGTATGTACGAGTGTGTGAATTGGGGAGGCGGAAGCGAAGCCAAGTGACATTAGAT

CCTGCGAAGCGAACG

>Tb927.3.2310 | Trypanosoma brucei TREU927 | PACRGA | genomic | Tb927_03_v5.1 forward | (geneCodeEnd+0 to geneEnd+0) | length=315

AGCAGCGTGATGCCGGAGGAACGTCGAGAAGAAAGAATGAGAAAAGATGCGTTTTTTGAA

GTTAGAAAAGAAAGCTCACCCCAACTATTGGTTGATTTGTATTCAAAGGCCCCATGATGT

CCTCATGTAATTTAGATAAGCCTTTTGTTACATGCAGCTGAATACAGTGAAGAAAGCAGC

GGATGAATTCCAGGAAGGGGGCGCTCTTCTTCATTTACACCAAGCAGATAATTTTGTTTT

GTTTTGATAGAGTGGCTGCAAGGTGAAGCATACGGTGCTTACCACATAAACATGTACGAT

GTTCTCCATCAGGTG

>Tb927.7.2710 | Trypanosoma brucei TREU927 | NADH-cytochrome b5 reductase, putative | genomic | Tb927_07_v5.1 reverse | (geneCodeEnd+0 to geneEnd+0) | length=316

GTTTTGTCTTTCATACTGCAGTAGGTAGTCAGGATCTTCCAAATTATATATGGCATTCTT

CTCACAGATCTACCACTTACCACCATGCCGGTGACTTTAAGGGGGTACTTCAACTAGTAC

TTCATGTGAAAAGAAAAAAAAGAAAGAAATATAGTGGGCTGGGTTGGTAATCCACCCGTT

GTTTTTTCTGACTTATCGTGGCAGGAGAAAGGAGTGAGCGATTTGTGGGTACGTCACTAA

AAATAGTTGGTGAAGGATCAACGACGGTTACAGGGAAAGGTGTGTTGTCGCTGTAATATA

CACTTAGGAATACCGG

>Tb927.11.2940 | Trypanosoma brucei TREU927 | hypothetical protein, conserved | genomic | Tb927_11_v5.1 reverse | (geneCodeEnd+0 to geneEnd+0) | length=316

AAGTAAAAGGTTAATGCCGAGTAATTATATATACGTACTCACTCTATTCTAACTTTTTTC

ACCCTTCGCCGCACTCTGCTCCTGAGATCATATTTTGGGGGAACACCTGTCCGCTTGGGA

ACGTGTTGCAAACGGTAGCTCGGTTTCACCCCTTTACAATTTCTTTGTTCTCAACAGGGT

TAAACCGCATACACAAAGAGATAGCATATATCGAGAAAAAAGAAAATAGAAGGTAGTATT

TTTCGTGATACATTTAGTAGTAGCGTTAGGAAAGAGTTTGTACAGTTACTAACAGTGTAC

AGACATCAGCTTCATC

>Tb927.10.2890 | Trypanosoma brucei TREU927 | enolase | genomic | Tb927_10_v5.1 forward | (geneCodeEnd+0 to geneEnd+0) | length=316

ACGGTTATGGAAGCACTTCGCACTAAGTGACTCTTTTAAGCACTCCACAGGCCGTCTTGT

GCACCTGCAGTATCATATATTCTATTTGCCAGGTGTCAAGTTGTAGGGGGTGAGTGGGTG

CGGGAGTTTTTTCATTTGTTTGCTTGGGTGGTGAGAAAGGGAATATTATCTTTTTATCTT

TTTGTTTTTTCTTCTCGTAACTTTTCATGGATACGAAGGTAAGCCCGTGAAATGTAATAC

AGGTAATCTGAATGTAATACGGGTCAAATGAAAAATGGATAGTGAAATAAAAGGTGATTT

ACAAAAATAGGCTTTT

>Tb927.11.10420 | Trypanosoma brucei TREU927 | DNA-directed RNA polymerase I subunit, putative (RPA12) | genomic | Tb927_11_v5.1 forward | (geneCodeEnd+0 to geneEnd+0) | length=316

ACTTGTGAGCTTTCCTTTAGTAGCACATAAGTTGGAGGAAGGGGCACGCGGATGTGAGTG

TACTCCACGCTTCTTTACAAGTTTATAACATAAGGTAAACACGGCGTGCATGAGGATACT

CCAGCGTTTTGAGACACCGTTTAAAGCATGTGGCCCATAGCTCTATTTGCAACCTTTGCA

CAGCCATTACGTTGTGTAATGGGCCTTTGGACTTTTCGTTGTAGTCTTACAGGTCTACCC

TCGTTTGACCTTTGGCATTTGAGCGGGTTGTAAGTGTGCTCGGGATCTCGTCTCGTTAAT

GTGAGAGTTGATACAC

>Tb927.11.16600 | Trypanosoma brucei TREU927 | exosome-associated protein 2 (EAP2) | genomic | Tb927_11_v5.1 reverse | (geneCodeEnd+0 to geneEnd+0) | length=316

ATAGAGGAGGAAACGGGGGCGGTGCTACTGGAGAGCTAGAAGTGAATGAGGTATCTGTGC

TGGCAGGGCGCAGTGTGTTGTTCGGAGTATCACTTTCTTTGTGGCTCTCAACTCTAGGCC

GTTTATTCTTCAAGTGTTTCGAGTCATGTTTTTTTTTCATATGTGTTATGTCTGTGCGAT

TTCAAAGGATTCATATTGAACATCGACCTTCTCTCACCTCTCAATTGTTGTGACTCTAAA

CACCTGTGCAAGGGCAATTTCGTTGCGGTTGGGGTACAAATACCGCAAAGGGGACTGTGG

TTGCTCCAGCCCAATC

>Tb927.2.5160 | Trypanosoma brucei TREU927 | chaperone protein DNAj, putative | genomic | Tb927_02_v5.1 forward | (geneCodeEnd+0 to geneEnd+0) | length=316

GTAATTCCAAGCATGAAAAGGAAGAAAGAGGGGTTAAAAGGGAAGTTGTGCGAGATTTAA

GTATTGGATTCACGAGTCCCTACAATGCGAACCTTTTACTTCTAATCTGAATCATCTGGA

GAGATGTTCGTGAGAGCGATGACTGCCATCCAGAATGAAGTGTGGTCTCCCTCCTCCTTT

TGTATGTGGCGTTTCTCATCCTTGGAAGAAAGACATAGAGGAGAGATGGGAGCAAAGAAT

ATGCCCAACTTCTTATATTATTATTATTATTACTATTATTATTATTACTGTTTTCTGTTG

AGATTAAATTTCTACT

>Tb927.4.2840 | Trypanosoma brucei TREU927 | hypothetical protein, conserved | genomic | Tb927_04_v5.1 forward | (geneCodeEnd+0 to geneEnd+0) | length=317

GTTTCTTTAAGGTAAGTACCTACAGACGTATGAGACTTATGTATGTGTGTGCGTGTGTGT

CTTTTTGCCGTTGTTGTGCGGGTCTGACAGGATGAACGCAAAGAAAATTGAATGTCACAA

AAAAAATGTACAAATGAGAACAAGAGTGAGTTAGAGATAGAGGAGAAGATGAAAATGAAG

AGGGAAGAAAAAAGAAAAAGGGTTAAATATGTGGGGTCACTTCGAGTCATCAAATGATTT

CATTGCATATGCGTAAATGTATTTGAAACAATGGATATGTCTGATGTCTTGGAGAAGCGT

GTATGTGTACATATAGC

>Tb927.5.4100 | Trypanosoma brucei TREU927 | WD domain, G-beta repeat, putative | genomic | Tb927_05_v5.1 reverse | (geneCodeEnd+0 to geneEnd+0) | length=319

ACGGCGTGGATGAAGGGACTCGCTGTCCGTATTCGTCTCTGTGAGGATGTCTCTCCATTC

GTTCGACGTAAGGATCGACTAATTGTACAGGACTGTTCTGAAGGTGTTAACACCGCCGTT

TGTCGTACACAAGTACACAAATGTATGCAATTAACCGGGTAATTTAACGTTATGCGGGGT

CTGTTGCTGATTGTAGAGCGGAGGCGATGCACTGTTCATACCCGACATGGTGTTTAGGAG

CAGTTATTTATGTGTCGCGGTTGCCACAAGTGTGTTGTGCATTCTTCCGAGATTAATTGG

TGATCACGATCGATGCTTG

>Tb927.10.3290 | Trypanosoma brucei TREU927 | hypothetical protein, conserved | genomic | Tb927_10_v5.1 forward | (geneCodeEnd+0 to geneEnd+0) | length=319

ATTCTTATACGAGGTGCATGTGTTTCAGTTTTTCACAGGCATAATGCAGAGAAATACCTT

TGCCCACATCCCCAACAATTGTTGTTGTCGCAGGCGACGGCGGTGTGGAGACAAAAGTTC

TCCCACCATCTTTTTCACCTTTTACAATTATTCGTGACGCTCATGTGTGATGTGCGGTTG

TACACAGTTTCTCGTCGACGGATGTGGGTCCAACGTTTGAGTGTTGCGCGCACATGTGAG

TCCATGGAATAACAACGATGTCGTCTAAATATGGTGGAACTCAGTGCATATCTGAGTTAT

GTCTGCTCCACGGATTTCG

>Tb927.11.4330 | Trypanosoma brucei TREU927 | hypothetical protein, conserved | genomic | Tb927_11_v5.1 reverse | (geneCodeEnd+0 to geneEnd+0) | length=320

GGTTCGTCGTGCAGATACGAGGGATCGTTATATTCCCCAAATCAGCAGGATTTTTATTGG

CTTTTCAACATTTTGAATCAGGGTCTACGTGCTGTCCGTTGTTATGAAGGTGTATGTGCA

TGAGGTGCGCCCGCCAGAATTTAGTGTGTACTTCCTGCGTGGTCTCTTTTACTTTGTTTT

TACTTGTTTTTTTCCTTTACCTCTTGCGACCTGATCAACAGCGTCAAAATAAATACATTC

TGTTCTGGGCTCCGGGGATGGACACCGTACCGTTGGTGCTTCTTTTTGTTGATGGGGTGC

CTTTGCTACGGTATCAAGAG

>Tb927.7.1500 | Trypanosoma brucei TREU927 | nucleotide binding protein, putative (NBP 2) | genomic | Tb927_07_v5.1 forward | (geneCodeEnd+0 to geneEnd+0) | length=320

AGGGTTGTGGTGGTGGGGAGAGTGCAGAAGGCTGCTGTGAATGAAGGGATATCGGATATG

AGTATTAAGAGAGGTTTGTAAAGCGGAGTCTGCTGCAAGGATGGGCGTTGTGTTGTTTCC

TTATTTTTACCCAGCAAGCGCATCGTTAACTTTCTTTCTTCACTCAACCACACGTGTGTG

CATACGGTCAAAGTGGTAGCCTGGATTTGTACCCTATTGTGGCTTTCCCTCCTTGGATTG

AATAACTCGGCTGGGTCATGATAAGGCGTTTGCTTCCTATGGGAGGTGGGGGTTAGTTAC

CCTTCTCGAAGAACGTATGC

>Tb927.11.9460 | Trypanosoma brucei TREU927 | hypothetical protein, conserved | genomic | Tb927_11_v5.1 reverse | (geneCodeEnd+0 to geneEnd+0) | length=320

GAAATTTATTCCACCATCGCCACCTTTTAGATGTGCGTACGCGTGGAGCAATACTTTAGA

AGCACAAATTAAAGATGAAAGTGATTTCCATAATGGTGAAGCCATGAAAATGCACCGATT

TGTTCCCATAGAAGCACCGCGAGAAGTAAAGGATTTTAAAGGAACGACAACAAAGAAAAA

GAAACCTCCACTTGAGCGATGCCAAGTTAGAGAAGTTTCCCCCATAGAATATGATAACGG

CGGGAACTGCCACCACCAGGTAGTTTTTCCTTGGTATACTTTATTTTGAAACGCACCGTA

CATACATATATACATGTAAT

>Tb927.3.3990 | Trypanosoma brucei TREU927 | KREPB6 (KREPB6) | genomic | Tb927_03_v5.1 forward | (geneCodeEnd+0 to geneEnd+0) | length=320

AGGTAAAAAACAAGCGAACAAACAAAAAGTAAAACCATGGGCAAAAAGAAAAAGAAAAGA

AAAGGGTCCTGTTTTTGCTCTCTTTGTTGTTTATATCTGTGTGTTTGGGTTTTCTGTGGG

GTTTCAACTGGGCTTCCCGAGTGATGCCGTGCCATACGTTTTTTTTTCTGCTGCCTGTTC

GGCCCTGCCCATTTGTTTTTTTTTTTGCAATTCGAACCGTACAGTGGGAGAATGTGTCAC

TTCCGTATATGTTGGAGTTGCCTCTTCTAGCGTGATGTAGTTGGTTTTGTAACTGTGTGA

ATGCGCGCTTTTGAAACCGT

>Tb927.4.1090 | Trypanosoma brucei TREU927 | endosomal integral membrane protein, putative | genomic | Tb927_04_v5.1 reverse | (geneCodeEnd+0 to geneEnd+0) | length=320

AGAAACATGCGCAACGATAAAAATGCAGTGAATAGTTGGGTGTAGTTCTTACTTCCATTA

ACCCCCCCCCTTCTGTGAGGCCACTGTGCACGCTCGCAAGAAAGTTATTAAAGTTGCTCT

GGCACTGCGGTTCCGATTGAGTTCTGAAGTAAAAAGGGAGAGGAGAGTCTTTTTTTCCTT

TTTTTTCCTACTTAAGGACTCGGAGATACTGTTGTTTGCGTATGCGCATATTTACAAGTT

TTCTAGACTCCCTATCCCCCTTTCTTCCACGGATGTAGGTGCTGCGTGAAGTGGTGTTTT

GTATTTTGCCTATCCCCAAC

>Tb927.3.2680 | Trypanosoma brucei TREU927 | Ubiquitin-like domain containing protein, putative | genomic | Tb927_03_v5.1 reverse | (geneCodeEnd+0 to geneEnd+0) | length=321

ATGCATGAGCCTCGCAACACTCCCTGAAGCGAAGCGAAGAGAATTGAAATATTAATTCGC

AGCCGTGCCACCGCTGCAAAGCAACCAACCTTATGCAGCTCTCGTTGTTCTTATATGATT

GATTACTCTCCGTTTGTGAAACCTTATGCGCGGTGGTTCCCTGGAGAGCCCTGATGATGA

CGCCAACTGAGGGTCTTTTCTAGCGGCCGCTTTTCGTAGGGAACATGCACACGGTGAGGG

GTGATTTAAGTTGGAAATACGAATGGTAGCGGATATTCTGTTTTACTCCACATAGATATG

AGGCCGCGCGTATAAATAAAT

>Tb927.11.7790 | Trypanosoma brucei TREU927 | hypothetical protein, conserved | genomic | Tb927_11_v5.1 reverse | (geneCodeEnd+0 to geneEnd+0) | length=321

GCATCTACAAATGCACAGATCAAAGCATTGGTTCTCTGCCAGGAGTGAGGTATGATACGG

TGTTTTCTATCCTTCCATCTTTCGTTTCCCTCAGGGAGTATGTTCAATGCTTTATCCGCA

TCCAAGTTCACTTTTCCGGCTTCTGCTCTCGCTACATGAATATATATGTCTGTTTACTTT

TGATATATGCCAGACGACATCCGAAGCGGCTGTGAGGGATGAGACATATCGACATTCCTC

CCCCCAATCACCTGCCACTTATCACTTTTCACCATCAATATCTCAGCGCACTCGGTTCGT

TACACACCTCATGGTGCACTG

>Tb927.10.12240 | Trypanosoma brucei TREU927 | 3-ketoacyl-CoA reductase, putative | genomic | Tb927_10_v5.1 forward | (geneCodeEnd+0 to geneEnd+0) | length=322

ATTATCGAAATAACTTTTTTTTTTACAAAAAAAGTGGAAACACTAGGAGGAAAGGGGGGA

AACGGGATTGAGGGAAGGGTGTTTATTCAGTGACCGCCTTGCACACGTATATTACACGTG

TCTTCACAAATGAAAGGATTGGAGAGTATTTCAGGTGAGCGAACGTCTGCGTATTTTGTT

TTCGTCTCTTTTTGTAGTCTGCAACATAGGGATCATCTTACTCGTGGTAGAGTTTTTGTT

GGTTTTCCTGCGGGTCGCGAGTTGGCCCAAGAGACTCTATGATGATGAGTTAAGCAGGGG

CAATTTCGTGGATTGTTCTATG

>Tb927.7.2700 | Trypanosoma brucei TREU927 | NADH-cytochrome b5 reductase, putative (B5R) | genomic | Tb927_07_v5.1 reverse | (geneCodeEnd+0 to geneEnd+0) | length=322

AGATGTGACAGAAATTGAGCTTGACATTTCTACGAGTGCTGCCGAAAACTTCGACAAGCT

TGAGTGCACGGAGGAAGACATACCTTTACCAAATCAATAATTGTTTTGTATGACATACTT

AATGTTGTATGAAATGCCGCCTTTTGATCCGCGTTAGTGGCTTGTTTTAAGGTTGGCTGT

GGGCGCGCGAGTGGTTTTTCATCATAACTTTTTCTTTGTTATCGTTACCAAATGTATTTC

TCTTTTGTCTTGTGCACCCCTTATTTATTTTTCCCGTATTGATGGAATAAAACATTCGCG

TATATCGGTGCTTTTTCCCTTT

>Tb927.4.4600 | Trypanosoma brucei TREU927 | Mitochondrial ribosomal protein L51 / S25 / CI-B8 domain containing protein, putative | genomic | Tb927_04_v5.1 reverse | (geneCodeEnd+0 to geneEnd+0) | length=322

GTTTGTGACCGTTGTGGATGCACACCAGAAGTTGAAGGCACGTGAGTTGCCGCCCGAAAC

GATTGCGTTCCGGTTTGTGCTTCTGTACGGAGCAAGCACCTGTTCTGCATGCATTGTTGT

GTGGTGGAGTGCATCTTTTATCGATATTCACTGACCAGGCTACTGAGGTCTCAACTGCTG

CTACCCCAGTCGTATGTTGGGCACTCCTCCAATGCTGTTGAAACCTGGCGTCACGAGATT

TCCGATTGGGATTCTCTTTTCAACGAAGTATATGCGGCTGGACTCACCGCAGATCTTGCA

AATAATTCATTTTTTCTCTCAC

>Tb927.7.5520 | Trypanosoma brucei TREU927 | peptidyl-tRNA hydrolase, putative | genomic | Tb927_07_v5.1 forward | (geneCodeEnd+0 to geneEnd+0) | length=322

ATGGAGATGGTGGTGCGTTGTAGCCTTGAAATGTCTGGCCTTTTTGTATGTGTGGTTCTG

CTGCTTATGATTTCCCATCGACACGATTAGTTATCACTGCAGGTTGTCAGTCGCACTCTA

AACGAGTGAAAATTCACAGCTTGGGTGGTTGTTGCCGCTTTACCGTCCCCACAGCGACAT

CTCTAGAAGGTAAAACACACACACACACACATATATATATATATAACTCTGCGCATGATT

ATTCGACCGCTCGTTTGTGCTCATGGGGTCCGGTAAGGTGGGTCAGAAACTTCTTTAGTT

TTCACAAATTGTGGAACTGCTC

>Tb927.7.1440 | Trypanosoma brucei TREU927 | non-canonical poly (A) polymerase (ncPAP2) | genomic | Tb927_07_v5.1 forward | (geneCodeEnd+0 to geneEnd+0) | length=322

AAGAAAATGAAGAGACACGTTCAGTGGTTGAATTGTCGACGAAGGAGATTGTTGTATTCA

TGTGGGAATGAAGAACTCTTCCGTCCACGGTAACTGGATTCTTGGGAGCGTACTTGGTTC

CGGGGAAATGCGCTTTACCCTCCAAATGCAGACAAATGTCAACCATCTTCGGGTGCTACC

GAGAAAGAGGGAGCCACTATACTCTGTATGCGCGCATCTCCGTGTTACATCTTTTATGGC

ATGGATGGCTTCACTGCCTTAACTCGCTGTGCTTGGGTTGTGTTCACTCAGCTCCCACCT

TCGAGCTCCCCTAACGGTCGAT

>Tb927.10.10320 | Trypanosoma brucei TREU927 | Dynein light chain Tctex-type, putative | genomic | Tb927_10_v5.1 reverse | (geneCodeEnd+0 to geneEnd+0) | length=322

GCCTTCCCAAGAATCAATTACTCCATGGAAACGCCGCCATTTGTTGCTATAGGCATCACT

ACACCGAATAAAGGAAAGGTAAATGACCGGAGGGCGTTGGACCAACGCTGTGTCATGTTC

GGTCAGTTAGAGAAAGAAGGTGGCGACATAAACATCACGCCGTTGCGATAGGTAATCGGC

ATTAGGCTTGCGGATCCCCACCTCCACTTTTTTGTGATTCACATTTCTTTGGCCGTTGTG

TTCTAAAATCCCCCTTCTGTCCTCCGCATCCACCCCCTTTTTTTTCTCTTGCTCTTGTTT

TCCGTCATTCTCTGGGGATGTG

>Tb927.10.11200 | Trypanosoma brucei TREU927 | hypothetical protein, conserved | genomic | Tb927_10_v5.1 reverse | (geneCodeEnd+0 to geneEnd+0) | length=323

GCCGTTCCGTCCAAAAGGTGCAGCCGCAGTGGCTGTTTAAACCCTCCCCCCTGCTTACTG

CGACTTTCATTTTTTTTTTAAGAAAAAAAAGGAAAACCACTTGACTTTCGCGTTTTGCAC

AGGTATGATTGCGGGTTGATACCTCTTCCCAGCCGTGGCATCCCAAGTGTAACTCACTGG

TAAGTTGGATGATGGTTTGCGCAATCGACACTGGTGCCACTGCAGGCAGGTGCACAGGGC

TCACGCCCACATAAAATGGGCTGAAAGGAAGGTGAACACCCAGAGAATAAGGATGCATAA

GCATCTCCACGGCAGCCACCCGT

>Tb927.7.1680 | Trypanosoma brucei TREU927 | Uncharacterised conserved protein (DUF2305), putative | genomic | Tb927_07_v5.1 forward | (geneCodeEnd+0 to geneEnd+0) | length=324

AGGTACTGTTGGCATCGGTGTAATGTTTACAGTTAGTTAGTTAGATGAGCCTAAAATCTG

CTTAGACGACCATTTAAATGCGGTTGACTGAAAAATCAGAGGGATAAGCATCTTTGCGAT

GCGTTGAGTGCCGTTCACTTTTCACTATTTCAAGTAACGAAAGGGTTTGAAGGGTACCAC

TGATGCTCAGAAAGCTTCGCTTTCCCTCCCTTCTCTCGTGTTTCTTCAGGACGGATATTT

ATTTTGATATTTGTTGCGACCGCGATGTGAGAACCACTTGTTGGTTAGTACTTTCGTTAA

ATACTTCTCAGTATATATTTTTCT

>Tb927.10.3660 | Trypanosoma brucei TREU927 | aspartate aminotransferase | genomic | Tb927_10_v5.1 reverse | (geneCodeEnd+0 to geneEnd+0) | length=325

GAGGGGTGTGTCAAGTCGGATGGGTGATTGGGTTATGCCACTACTGTGGTTTTTATTATA

CTGTGTTTGTGTACATCGGCCCTTTTCCGGGTAATCAGATAGACCGTGCCTCAGTGCGTA

AGCATCGCTCCATGGCAAAATGTATGGCATGGGGGGATCATATACGGTTAGGGTTTGTAA

TCAATTCAAGGTATGTAGTTGTTGTTTATCATTTCCTTTTTGATTCGCATGTTCCTTCTG

TTCTGCTAAACAGTTGATGGAAGATATGGTGCATGCGTAAACTCACGGGAAATAAACAAA

GCGGGGTGGGAAGGGGGTAAACTTC

>Tb927.10.4880 | Trypanosoma brucei TREU927 | hypothetical protein, conserved | genomic | Tb927_10_v5.1 reverse | (geneCodeEnd+0 to geneEnd+0) | length=326

GATATCGAATAGGCAACATAGGGAATGGGGGGGGGGGATGGAGCGGAAGGTGACAATGTG

CATTTTTTTTTTCTCTTCACAGAAGAGCAATTAGCAACTGGGTGTCTAGATGTATGGTAG

GGGATAGTCATTTAATTATATGTTTTTGTTCCGTGGCGGTCCTTCGGGCTGTGTTCACTG

GTAATAATTGTGGAATCCACCTGGGTTTTTTTCTTGTGAACGCGCGTTATCTACTAGTAT

CTCTTCGTCTACCGTTTAGTTTTAACAACCATTTTTTCCTTTAACCCTTAGCCTCAACGG

CAGCAAGGTCAGCTAGCTGTACATAT

>Tb927.11.7550 | Trypanosoma brucei TREU927 | hypothetical protein, conserved | genomic | Tb927_11_v5.1 reverse | (geneCodeEnd+0 to geneEnd+0) | length=326

AAATGAACAACAACGACAGCAACAGCGACAGCGAAGTGAGTGGAAGGAAAGAGGGAAAAG

AAAGAAAGAGGAGCCGAAATAACGGCAAGAAAATCGAATGAAAAGGGCATGTTTGAGTTA

GTATACGCTAACACGCGTATGAATGTGTCTGTGGAAGTGACATAAATTCAGTGACTACGG

GCGGTAACGGGAAGGATAGAGGAGGGGAAAGACTAAAAGAAAAAAGTGACACAAGGGAGC

GGTTGAAAAGAAAAGAGTTGTGGAATTAGTTATTGGGACTTGTTTTAACGTGCACCAGCA

GGTGTGGACATCCTGAGGGAAGATTT

>Tb927.4.1270 | Trypanosoma brucei TREU927 | ruvB-like DNA helicase, putative | genomic | Tb927_04_v5.1 reverse | (geneCodeEnd+0 to geneEnd+0) | length=326

GAATATGAAAAAAGAAAAGAAGAGATGGAGAGAAAGTGGGATAACAAAAAGGGAAGAAGA

GCATGGAAGAAGGGGAAACTGTAGAGCAAATAAACAGAAGAAGCAGACTTCCCAGAAGCC

CGGAAACATGCGTGCGCCTAAGAAAGAGACTAAGTCTTACTTCTTTTTTTTTTTTACTAT

TTATACGGCCGCATGCAAAACTCTTGGAGAAGGAAGGAGGATGGTCTATTTCAAAGATTT

ATCAGGGGTGAACCACGCTAAGGTTAACACTAAGCTTTAAACTACGTATTCCATTCGCTG

GGAGTTACGAAAGAATATAAAATGAC

>Tb927.11.11460 | Trypanosoma brucei TREU927 | Mitochondrial import receptor subunit ATOM69, Archaic Translocase of outer membrane 69 kDa subunit (ATOM69) | genomic | Tb927_11_v5.1 forward | (geneCodeEnd+0 to geneEnd+0) | length=326

ATGATCAAGGAGAGGATAGGCATTTGTCGCACTGCCACTTTTCACATGCGCTATTGCCCT

CATAACATTGTGCATACCTGGTCTACCTGTTTGTACAAAGCGGGAGTGGGCGAGGAGGAT

ACTGGTGTTACCAGCGGCTGATCCCCCCCCCCCCCTACAACGGAGGCAAGGGGCAACGAC

AACAATCAGGGAGCATTTCCGCATCTCCCTCTTTTACCACCATCACTATCATCAGCGACA

ACATCACACTCATTCTAGCCTGACGCTGAGGAGTATGAATGATTGACGAAAGTCAAGGTA

GTGATAACCGGAGGGGGGTAGCAAAG

>Tb927.5.1620 | Trypanosoma brucei TREU927 | hypothetical protein, conserved | genomic | Tb927_05_v5.1 forward | (geneCodeEnd+0 to geneEnd+0) | length=327

AACAAAAATGGTTGTACCTCCATTGCAACTGTAACACCCCATGACTGAAGCGCCATGCAG

TTGCTTTTCATTCACTTCTTTTCTGTGCTCCATGTATATATATATATATATATATATATA

TATATATATATACATATATATATGTGTGTATGTGTGTGTGTGTGTGTATGAGTTACGTAT

TGTTGAACCGCATTTTGCTTTTCTTTGTGCTGAAGGGTGGAAGCGGCATTCGAATAATGA

GGTGGCGAGTAAGGGTGGACGGGTGCAAATGGTGTTTGGAAGGAGAAAAAAAAAACAGTG

TCTCTATTATCTGCCTCTACTATCTTT

>Tb927.8.1170 | Trypanosoma brucei TREU927 | hypothetical protein, conserved | genomic | Tb927_08_v5.1 reverse | (geneCodeEnd+0 to geneEnd+0) | length=328

GGTATGTACCTGAGAAGCAACTCTACAGTGAACAGCCTGTGGTCATTGGTGATAAATGAA

AGCGAAGGGGAGCAATAACAACAACCAAATATTACCAAGTAATGCGGAAGTGCATAACTG

CTTCTTCATGAGGATGAAGCAGCCGGACTGAAAGTAAGCGTAAGAGAAATTGTTGTGTGA

AGTAAGAAACAGGATAAGGTTAATGGTTTCATGGTGTGCCTGTGAACCGGCAGTTCGCAT

ACGTCTGTGCAAGGGGCCCCACTTCACCCTTCTGTACTTTCGGTGACGCGCAGGTAATAT

TAACGGAAGAGGCCGATTAGGGAATATT

>Tb927.10.1630 | Trypanosoma brucei TREU927 | atp-binding cassette sub-family e member 1 (ABCE1) | genomic | Tb927_10_v5.1 reverse | (geneCodeEnd+0 to geneEnd+0) | length=328

GGTATGGAAAGTGGATGCGTTGGGGTTACAATGATCTAAATGCCACCGTATTACGCTTTT

TCTGATTCGTTGTTGGAAGGGGGAGCGTGCGCACGTAGAGAGCTGCGTGGGGGTGAGGTA

GCGGAGGCAATGCCGCACGGATGGTCCGCGTATCAGAGCAAATAAGAATATTTTTAACGA

AAAACAAGCAACGGATGAATATCGGCACATGTACTTTTGTCCATTTCACGTTGTGGTGTT

CAATGCTGAGTAGGTGGAGTTAAGCACTAAAAGTTAAACTTAATTCGACTTAAGTGGGTT

ACGTGGCATAATAATGTACTTACTTTTG

>Tb927.4.2850 | Trypanosoma brucei TREU927 | hypothetical protein, conserved | genomic | Tb927_04_v5.1 forward | (geneCodeEnd+0 to geneEnd+0) | length=329

AGCATTCCGTGAAGGCGGGTGTGCGACTTGCTGCCGTTGGTTTTGGTGGCCGACACCAAG

ACGGGTATGAAAGTAGAAAAACAGAAAAGAAGAGATGCTAGAATGAATAACGTGGCGTCA

CACGGATATCTTAGCATTCCTTTGCAAACTTCTTCGACCAGAACAATTTCATGGCCGTGC

GTTGGTCTTTCACAGGAACAGAAAACTTCCCCCCCCCCCCCTCTCTCCAATGCAAACAAA

AAGAAAGAACACGCCTGGTTACTTTCTGCTAAAAGGTATGCACTTTCACCCGACATGGTG

CCGAATCGCTCAATGGTCGCACAGTAACC

>Tb927.11.3770 | Trypanosoma brucei TREU927 | Dpy-30 motif containing protein, putative | genomic | Tb927_11_v5.1 forward | (geneCodeEnd+0 to geneEnd+0) | length=330

GAGGGCGGTTGCAAGTGCTTTCATGAATGAAAGGAATGCGTTACTTTTTGTTGTAAAATG

TCCTCACACATTTGAGAGTGGGCAGACGATACGAAGATAGAGGTGGCGAGTGAAAGCAGT

GCGACATTATGGTGGTGGCGAATTTTTGGTTGTGTATTATTGTGTTGCTTATGTATGTGG

CGGCTGAAGGTACGAATGGTGAACCTTCGTAACACAATTCTTATGTTCTCTTGTTTCTGT

CCCATGTGCTTTTTTGTTTTGTGATTTGTTCTCCATCATGTATAGTTGCACTAGATGACA

CATGTGAGGAAGTACAAGTGGAACTGGGAT

>Tb927.1.1390 | Trypanosoma brucei TREU927 | hypothetical protein, conserved | genomic | Tb927_01_v5.1 forward | (geneCodeEnd+0 to geneEnd+0) | length=330

GTTACTGAATTCAGTGGCGCAGTTTATGCGGAATCACATATATATATATATATATATATA

TATATATATTGAAAAAAAAATAACAACAACAAAATGACGTCGTATGTGGTTGTTTAATCT

GGAAGGTGCCATCTTCTCCATGTGAGCGATATCTCGGTGGTAGATGTTTCACCTTTTGCA

AGTTACTGGGTTCGTGTTAAGTTACCGAGTCGTTTACATCCCGTCAAACCTTTTTATGCT

TATATCGTTTTGGCAGTGTGTGTGTGTATGTGTATGTGTGCGCAGTCTCCGTAGTCCATA

GGTTTGTAGTGCTCAGATAGCGCTTAACAT

>Tb927.3.5570 | Trypanosoma brucei TREU927 | syntaxin, putative | genomic | Tb927_03_v5.1 forward | (geneCodeEnd+0 to geneEnd+0) | length=330

AAGTCGCCATCACTTTCCCCTTGTGGTTTCCATTGTTGTTGTTGTTGTTGTTGTTGTTGT

GTTTGTTTTTTTTTTACCGTGTACAGGAGAGATTTCGGGATGTTTTTCATCGGAGCGATG

AAGTTTAGTCGTTGATGCTCTTCTCCGCTCTGCGCTAAAGGGAGGTGTCTGGTTGTTGTT

GCTGTTGTTGTTTTATAGCATGCAGTGAAAAGCTTAGGGCCACATCTTCACTCACGAGTT

CCACCATTCATTCGGCCTTTTTGTGTTGGTTATTAAATTTGGCGCCTCATTTATTTTCCT

CTTCCATTCTATCATATATGCTATTGCTTT

>Tb927.9.6320 | Trypanosoma brucei TREU927 | hypothetical protein, conserved | genomic | Tb927_09_v5.1 forward | (geneCodeEnd+0 to geneEnd+0) | length=330

ATCTCTATCTAGCAGTTGTGGGGGGAAATTGCTTACAGTTTCACTGATTGAAAGATTGCG

ACCGAATGGATAAAAACAGTTAATGTGGAATCATTTTTTTAGTGCCACTAAACGGTTGGA

AGGTGATCTGAAGAGAGCCTAGGCTGTTTCGGGAGTGCGCACTTGCTTGTTCCTTTGGTA

ATAGGGAACATTAATGCTTTTCTAAGAAGAATATTGTAATTGTCGTGGACACGTGGCCCT

TGGCACCTGCATATACTCTTCCCGACAATGCTCTACGGGGATCGCGTGGTTATTGTTTGA

TACGACGTAGCCCCTGCTCTACTCGACTTT

>Tb927.8.2030 | Trypanosoma brucei TREU927 | hypothetical protein, conserved | genomic | Tb927_08_v5.1 forward | (geneCodeEnd+0 to geneEnd+0) | length=331

GTGTAACTGTGCGCTCACCCTTCCCGTTTACTCTTTCGTATATTACTGTTATCGCACCTC

TTGAACCAGTGAAATGTGACCGCAACTTGAGGGGGGAGAGAGTAAAGTAGTGAGTAAAAG

GAAGAGATCAATTAGATGTGAACAGACTCACGCTGCCCAGCAGCCGCTGCTGGACACGTA

TTACTTCAGAGGGTGTAAAAAGTATTTTTATTTTTGTATGTGTAACCCCTTTGGTGTTTA

CCTTTTTTTTTAAAAAATGCTGCCCTCCTCGGCATTGCACTTCCGTTCGGATGCCACTCT

TTTGTCGGTTCCTGTTGTCCAGTAGCCTTTT

>Tb927.5.3950 | Trypanosoma brucei TREU927 | 2OG-Fe(II) oxygenase superfamily, putative | genomic | Tb927_05_v5.1 forward | (geneCodeEnd+0 to geneEnd+0) | length=331

AGCTAAGGAGCCGCTTCACTACTTTGTTTTATTTTTCGTTTCGGCGCGGCCGTCCGGGTG

GGGTACAAGATATATTGCTGGTAGCCCACCCTCACACGTTTTACAAAGCAAGTTTACGAG

TAACAATGGGTGGGAACCGCCCAAAAAATGGAAAGAAGACTTACATGCGGCGGGCGGAAA

GGGGGTTTTATGTTGTTTTTTTTTTTGCACTTTAGCGTGGTGTTAAGAGTCAATCAAGGG

GGTTCTGTGAACGGTTTTAACGAGAACATATTACAGCACTAAGACAAGTCTAAATAGTGA

ACCCCGTAAGTTTTTAAAGGACTGTTATTTG

>Tb927.4.1920 | Trypanosoma brucei TREU927 | GPI transamidase subunit 16, putative (GPI16) | genomic | Tb927_04_v5.1 reverse | (geneCodeEnd+0 to geneEnd+0) | length=333

AGTGAATACCGAAGACGAATGTTAGCGTGCAAGTGAAGGTGCGCACCTGTTTGCACATAG

TTTCGCATTTATCTAGTACATCTGTTTGGCCTCCTGTAGCGGGTGTTGTTGCATGTGATG

CTCCCTCTTTATGGTTGTTGGTTGGTTGGTTGGTGGCGTTGCCTGACGATAGAGATTCTC

TCCTTTCCTCCACTAACAGTGTTTTTCTCTTGCTAGCTCTTTTTGATATATTCAAAGGTG

AGATTCTCTAAGCTGGCGCTGTATCATCCCCCCCCTGTCGCTCTATTGGTGTATACCGGT

GTGTTTGTGTGTTGTGCAGGGCTTTGTCAGTTT

>Tb927.10.5830 | Trypanosoma brucei TREU927 | hypothetical protein, conserved | genomic | Tb927_10_v5.1 reverse | (geneCodeEnd+0 to geneEnd+0) | length=334

AGACATTCCTATTCGTTTATTTAACTTTGCTTGCTATAACAGCGGCAAATAACTATCACG

CTGGATAGACGTTGTCACAGATTCTCGCACCGTCGCTATTTCATATTGGTCTTTTACCTA

CTGCCGGAAAGCGACCTACTTAATTTTTTTTCTTTTCGTAGCTTCCGTGGTAAAAAGGTG

CATGTTCGTGTTACATCACTGATGGGGTAGTGATTTCTTCTTCCTTTCGTTTTCGAATTG

GATCTTATCAAAGTGACATCGTTACGGATCGCCACGGCTTAGCGGGGAATTTTAGTTCAC

ACATAATGTCGAAATTTTCTAACGATGACTATTT

>Tb927.9.7250 | Trypanosoma brucei TREU927 | hypothetical protein, conserved | genomic | Tb927_09_v5.1 forward | (geneCodeEnd+0 to geneEnd+0) | length=334

ATGAGGTAATACGTCCTCATTTTGCCACTTTTGTATATATATATATATGTTTCTCTCGTT

GGCTAGTGCTCGGCTTTTTTTATTCGCTGTGGCGTTGAATTGTAATTGAAGTGAACCTTT

AACATCGCGGTCGGTTAATCCAGAGATGGTCGTATAACAGAGGGAGATGTTACCCAAATA

ATTAAAAAGGAGGGCAGAAGGCTTATGAAGTTGAGCAGAAGTGTTGCGAGAACCTTCACT

GTTGTCCCATCTTTTTTGCCTCCCATCACAGGCGCCACTGGACGCTATAAGTGGTAGCGG

CCAACGCCATGAACTCCACCTCTTTGTTCTTTTT

>Tb927.1.3170 | Trypanosoma brucei TREU927 | CHORD, putative | genomic | Tb927_01_v5.1 forward | (geneCodeEnd+0 to geneEnd+0) | length=335

ACCCAGTTCACGATCGATGATACACTTGCCGTCTTTGAGAAGCTTCTGGTTGTGTTTGTG

TGTGTGTGCGTGCGTGCGTGCGTGTGTGTGTGTGTGTATATGCTTGTGAAGGAGCAAGCA

AATGACACAATGCCTCTGGAAACGACATCAAAAGGCTGATTGGTGGCTACTTGTTTTAGC

ATGGAGGGGATCACTTGTGTAAACGAAAGTTACAGTAACACATCTGATTAAAAAATGGCG

TGAACCCCAGGAAAGAAAGGAGGGATGCCCCTTAGCGTGTTAGTATTCCCGCCGGCCCTG

AAATGCATTCACGACTTTACTGAGTACTTTGCTGT

>Tb927.3.1680 | Trypanosoma brucei TREU927 | hypothetical protein, conserved | genomic | Tb927_03_v5.1 reverse | (geneCodeEnd+0 to geneEnd+0) | length=335

AATTCATCTTGTTTTAACTGAATGAAGCCAAAGGAGGCGCATGTGTGCGCGTGTAGGTGA

GAGGGAAATGAAGAAAAATAAAAAGGAGGATGAGGGAAGCGCTGAAAGTGTGCATTCATA

TGTGTGTACCGTACGAATGCAAACGCGGGCAAGGCAAAAGGAAACCGGGAGAGGGGGATA

TGTTCACTTTACCTATAACCTTCTTTTTAACCTCCTTGTTAGAAATATACGCATTTACAT

TCGCACATGCACATGCAAGCGATTCCTTTACCGCGTTGGTATGTTTTGTGTCATGTTTTT

ATCCTGTTGTTGTTGTTGTTTGCAATCACATGCGG

>Tb927.8.6230 | Trypanosoma brucei TREU927 | hypothetical protein, conserved | genomic | Tb927_08_v5.1 forward | (geneCodeEnd+0 to geneEnd+0) | length=336

GTAGAGATATTTGTACCCTTTCACCCCCTCTTCATGTATTATACTCAACGACAGTGGTAG

AAGAAAAACTGTTTCTCTCGACACCTACGCTGACAAAGTAGGGGATGTGGTGTGCCTTTG

GAATCATCCATGAAGAAAAGGTAGCGATACCGAAGTTAGGTAGAAGAGTTTCGGATATAT

ATTTTTTATTGTTGGTGGTGGTTCACTCTTTGTTCATCCCGTTTGTTCTCTTTCTCTACG

CTTCACTTCTGTACGATAGAGGATAAGCAGCTAGATAGCGCCAATCAGTGACATCGACAC

GTCACATACGGCACACGCGTAATCCTTGAAAAAAGT

>Tb927.11.2350 | Trypanosoma brucei TREU927 | hypothetical protein, conserved | genomic | Tb927_11_v5.1 forward | (geneCodeEnd+0 to geneEnd+0) | length=336

AAGGGCACGAGGAGGGGGGGGGGGAAACGTGCAGTGGTAGTAGTCGTGTCATTTTTGGTA

GTATCGATTAGGTTTCCGAGATGTATACATATTTTCAGGTATGCACTCATATGTTGAAAT

GCCGCGTAAACCACATGTGCATTCCTTGCTGGAGCTTCAATATCTAGCGAAGGAGCTGTG

TGGTATGGAGACACATTTTGATTCCTTCACCACACACGTAGCGTGCTGCACAAAGTCGCC

GGTGGTGTATGAGCTACGATTCCTTTCTCAGGGAAGTCGGACCATGAGACACGAGTGGAG

TTACTTATGATTTTGCTTGTATTTCCATTAGCCTCT

>Tb927.1.3830 | Trypanosoma brucei TREU927 | glucose-6-phosphate isomerase, glycosomal (PGI) | genomic | Tb927_01_v5.1 forward | (geneCodeEnd+0 to geneEnd+0) | length=337

AACAACCGACTGAAGAAAAATATTTATGTTGACTAATAGTTCATCAACTGACTTGGGGTT

AATTTTTTTTCCATTTGCTCCCCCTCTCTTTAAATCCTATTTTAACAAAAACGACGCAGA

CGATCAGTGAGCTACTCGACGCAGCAAAAACTTCCCTACTGCTTCCGTTCTTTCGAAGGG

AAATATATTGAATGCTTGCGCTGAGTATCTTCATATATTTTCTTTTCACTTATTTTCCTT

TATAAAATTTCGTTCCCACCTCTTATTGATGACCTTTTCCATCTCTGGATGGGATCGTTC

TTGGTGTTGGGTCTGTTAGCTGTTGTTCATGTGTGAC

>Tb927.9.9140 | Trypanosoma brucei TREU927 | hypothetical protein, conserved | genomic | Tb927_09_v5.1 reverse | (geneCodeEnd+0 to geneEnd+0) | length=338

ATTAAAGGCATCAGTTTTTTTGTTGTTTTCTTTTTGTCGCGTGCTTGGCCTCCGAGTTGT

AGAGGTACCATCCCCACGGGCACCTGTTCACATTGCGGGACCAACCCACAGATTCCTAAT

GTGCCGGGCACTCGGAGACTTTTGGAACATGTTTGTTTATCTATATGCTGATACGTTCAA

GACTGCGTATAGTGTTTTCATTGCTGTGTCAACCCCTCATATTTTGTGTCAGCCCTCTTG

CTGTGTTGTGTGGAGGTGGCTTGTTTTGCTTCTACCACCTTATTGTTCCTTTTTCAACCT

GTTGCTCACCTGATTTGTCTCACCAACACCATATATAG

>Tb927.6.3250 | Trypanosoma brucei TREU927 | C2H2 type zinc-finger (2 copies), putative | genomic | Tb927_06_v5.1 forward | (geneCodeEnd+0 to geneEnd+0) | length=339

ATATAATTTTTTTCGAGACGAGGCGGCGGGGTGATCAATAAGACATGGTGGTTAAGGAGA

AAAACAATAACAATAAAACATAACATGTACTTCAAATTTTAAGGCCTGGTCCCGCTATAG

GTGAGGAAAGAAAAATGAATATTGCTTCTTACCTCATTTGATCTTTCATATCGGTTTCAA

CTAGTCGACGAGTGCAGTTTTGGCAAAAACAATAAGCGAGGCAACAACAACAGCGGCAGC

GGCCAGTCGCCTTTATGGTCCCCATGCTCGTGCGGAGTGTATATCATCGGCAGTGGGCAG

GTCCGTAAGGAAGCAAAGCTTCAGAAGTGGAGTACACGG

>Tb927.6.2740 | Trypanosoma brucei TREU927 | pyridoxal kinase (pdxK) | genomic | Tb927_06_v5.1 forward | (geneCodeEnd+0 to geneEnd+0) | length=340

AGAATTAAATGCAGTCTGTCAAGTCCACCCTTTTTTCTCTCTGTTCTCTAAAGATAAATA

ATTCAGTTTTACGAGTTTGTCATGCTACGTGTTCTTCAGGTGTATTACGATATTGTGTTT

GATTTAAGAGAAGGATTTACTTTTATCAATATATGGTAGGGTAAATCGCATACAAAACGA

AGAAAAAGAAGAGGAATTCAGGTGCAAATGTGGACGCTTTTCTTTCTTGTTTTTGTGTTT

TAAACGCTATCCAATAAGGTTTTCTTCGGTATTATTACTTTTCCTTGATTTTTGTTATCT

TTATTACTAACACTTTTATTTTTCTTTTTGATCGTTGGTG

>Tb927.10.14360 | Trypanosoma brucei TREU927 | U2 small nuclear ribonucleoprotein 40K (U2_40K) | genomic | Tb927_10_v5.1 reverse | (geneCodeEnd+0 to geneEnd+0) | length=340

ACAGTCGCCGACTTGAGGTGTGTGGAGTTGTGGATGGTGTCCACGTGTTTTGGCTTACTT

GCAGTAGTGAGGAAAGCCACTGACCCCTGTGGGAATGTTGTTGAGAGAAATAACAAGATT

GTTTGTGTGTACGCGAGAGAAGGATTTGGGGAAGGGGGCCGTTACAGTCGGAGGTAAAAG

CGACAGTTGGGAAGTGGCGATTAAATAGACATCGTGGCGCTCCAATGGCACATGTCCCTG

AAAGATGGTATGGATGAAAAATGGTATGCTTGCCTATTTAAAGTCTGTTCTCTTCCTTCG

TTGCGGTCTATTTTCTTTCCAAGTTATTATAACAACAACT

>Tb927.10.8230 | Trypanosoma brucei TREU927 | bloodstream- specific protein 2 precursor, protein disulfide isomerase 2 (PDI2) | genomic | Tb927_10_v5.1 reverse | (geneCodeEnd+0 to geneEnd+0) | length=342

GCAAGATTAAGAAAACGTTTGGCCCAGTTTCAAATCCTTATCCACTTAGCTATTTTCGCT

TTTCGGCTCCGATGATGTGGGCAACAATGGTTTGCAAGTTGCCGGATGGAAGACAAAGTG

AAGAACCCCTTATATATGAATAGAAGGGGTCGTTGGCGGGGGTGGGTTATGGGGCTAACG

TAATGCCAGGTAATAGAGATGATAGTGGTAAAAGCGGGGTATCTTTAAGAAATTGAAATG

TCGGAAGGGAGGGGGGGGGGATAAATAACGGCGGAGGGTCGGGGGTGCTGCGCGCCTCTA

GAGTCCTACTCGCTGTAGATAATAGGTATATAAAAAGATTTG

>Tb927.11.2770 | Trypanosoma brucei TREU927 | hypothetical protein, conserved | genomic | Tb927_11_v5.1 reverse | (geneCodeEnd+0 to geneEnd+0) | length=342

ATGAGTGGGTGAGATTTGGGACAGAGAACACAAGAGGAAAGTCTGTGCGGTTGTGTGTGG

ATAAAGCTGCCTCCAGCAAGTCCTTTCGTGTGATCAAAGCAAAACCAAATACCTTTTGCC

CCCTCCTCATTTCTTTTAGGAGCAGGATCACCATATTGAATGTACAATGTGTTTGGTTGT

TTTTGTACCATTGGGACGATCTTTCTATCACCTCGTTATATATATATATATATATGTATT

TTTTGTTTGCAGAATGTTTTGCTTAGATATCTGCGTAGAGAGGCGCCAGAGAAGCAACAG

AGAAAAAAGTGGGCTGAGGCTCGTGTGTATGTTCGTCTAAGG

>Tb927.10.2240 | Trypanosoma brucei TREU927 | hypothetical protein, conserved | genomic | Tb927_10_v5.1 forward | (geneCodeEnd+0 to geneEnd+0) | length=342

GTTCTGAGAGGCACACACGTACATATTCGTGCCCACGTCTTAATCAAAGAACATAATGAA

GCGAGAAAGGATAAAATAAATGGGCGATGTAACGGACAGCCGTACGCTCCGTACCACCAA

GTGAAGGAAATGAGGCGGGCTAACAACTCCTCCGATTTTGCCTGATTTTCTCTTCCTCAT

TCTCGATGAAGATCAAATTCATTTTTTACTTTTGTGACCAGTAAACGTTTTTTTTGTTCT

TACCTTTCCGGTTTTGAAATAAACTTGGCAGGAGCGGCCATATCTTAGCAAGGTAGAGTG

AGGAGGAGAGAAAGAAAATGATGAGTTCACTTGTTGGCTGTG

>Tb927.7.5830 | Trypanosoma brucei TREU927 | hypothetical protein, conserved | genomic | Tb927_07_v5.1 forward | (geneCodeEnd+0 to geneEnd+0) | length=342

AGTGCACATGTTTGTTAGTTTGTTTGCTTTTGGAACTCTTGTGGAAACGGGCAAACTGTG

GAGCGGCGACGCGAAACCGCTCTTTCGTCACAGCGGAGTTGCCAATACCACCGGCGATGT

TTCTCTTCCTCCCTTTTTTTTTCTTCTTTGGCTTGTCCTCCCCTTTGCATGCAACACCTC

CTTTGCTTTCCATATTATTCTGTGCGCAGCAAACTCGTCGTTGTTGTTGCTCTTCTTCAC

TTTGCTCGAGCAAAATGCTGAAATGAGTGAGTGTTGGACCTTATAGAAGGCAAAAAGAAT

GAAAGGGAAAAGCACCTTTCGTGCACGTACGCCCATGCACTG

>Tb927.11.13770 | Trypanosoma brucei TREU927 | LEM3 (ligand-effect modulator 3) family / CDC50 family, putative | genomic | Tb927_11_v5.1 forward | (geneCodeEnd+0 to geneEnd+0) | length=343

GGTTGCGTGTCAAAAATTTGGCTGGTAATGTGTCGCGCTAGATCTTCAGTTAATTGTTTT

GTGCTTTCTATGTTGTCTTAATCCTTGGACTGCTACGTTGTATGTACCTATATATATGTA

TTTGGTACGTAGGTATTTAATTTTACTCCACCACATTCAGAGGTTTCTCCCTTTTTTGTC

TGCTGGAAGTTATTGGGAAGCTGTTTGAGCGCTTCATTCATGTTCACGATTTATCTTTTT

CTAAATGTTGCTTACCTTTACTTTGTTTGTCCAGAAGGATATGTCTTTCTGTCCCGCTGG

TCCCTCGTGTTGAGAGGAAAGAGGGTCTTCCCAATATGTGATC

>Tb927.8.3600 | Trypanosoma brucei TREU927 | hypothetical protein, conserved | genomic | Tb927_08_v5.1 reverse | (geneCodeEnd+0 to geneEnd+0) | length=344

ATATTCAAGTTTACATCACTAAAACTGACGCATCGCAGCAAGTGCCGCATAACGGCTAAG

GGTCGCTTTTTTTTTTTTTTACACACTCTGAAGATCCCTGTCCCTATGTTTTACATTCAT

CGTTTGTCGATTGGTTGGATAAGGGATGTGTAAGCATCAATGCAGTAAGATTCTTGAGAA

ACATGCCGTTGTGTAGACTGGTTGCACTAACGTTTGTTCATAGTTCCTTGCATTATCCTT

CTACTTCCCCTTAAATAAATGTATGCACATAAGCAGTGTTAAGCACCCTTAGTCATCCAT

AGGGGCTGTAGCTCCTGCTTTTCGGCTTTCGTCAGAGCATAGCG

>Tb927.4.2330 | Trypanosoma brucei TREU927 | hypothetical protein, conserved | genomic | Tb927_04_v5.1 forward | (geneCodeEnd+0 to geneEnd+0) | length=344

GGGCTCCAGTGAGTGGTAACATTATCGGAATTCTCAATGATTTCTCCCGCACAATCGGTG

TCCAACGTCTTCACTTTGAGAAAAGCAGGTGAGACCATGACGTTTTTCTTTTTATGCGAG

ACAATTATGGAAGGGGAGCTGGAAAAGAAAGAGAGAGTCGAAGTATTCTCAGAGTCTTTT

GTCCTTGTTTCCTTTCAGGCGTTTCACGCTTTATTTCTCCTCTTCCCCCCCCCCTGTTGT

GGATCACGTCACAAACTTATGCGTCACAAATGTAACGGGTCCGTCCTCACTGTATGTTGT

GCCTGTTGGTGAGGTGAGGCGAGGCGTAGTTGCGTGGTGTTTTG

>Tb927.11.9050 | Trypanosoma brucei TREU927 | p21-C-terminal region-binding protein, putative | genomic | Tb927_11_v5.1 forward | (geneCodeEnd+0 to geneEnd+0) | length=344

AGTTGACGTTGTCAAGGAATTGGGGCTCCTCCTCGATGGTTGAGCGGCCTTTGCGTTTCC

CATCCACTTATGTACGTGGGCGTGTGCCCGTCTGCTTCAACTAACGTCCCTTTTTTAAAA

AAATAGAATAGTGACGCGGCTTCAATATTCATACCGGAATATTGTGATTGACGAAGCGGC

CTTTGGATGTGCGAATGGCTTTCGCTGGGCGCTTGCGATTTGTATATTCCCCCCTCAGTC

ACTAACTCTCACAGTCAGGCGTGAATAAACAACAGGAGATTTGCCACCCATGCGAGCAAC

TAAGTGGGGAAAGAAGGGAGTTTGGATGCTTAACATTGATCGTG

>Tb927.10.15740 | Trypanosoma brucei TREU927 | hypothetical protein, conserved | genomic | Tb927_10_v5.1 forward | (geneCodeEnd+0 to geneEnd+0) | length=344

AAATATTGTCCCCTTGGTGGCCCAGTAACTTCAAACTAGTTCTGCTTTTTAATGGCGGAA

AGGAACGACAAACTCTTGTGGATTCCTCTTGCCTGGAGCCTCGATCAAGTGACAGAGCGT

CGTTGAAAAGGAGGAAACAAAGGGGAGGGCGGTTGAATCTTACATCTGGGATCTGCATTC

GATGCCATTTGTGGACCTCTGTTGAGCCATGCTTCGTTAGAGCCAACAAGGCCCAAAAGT

TGCAACCGTTACAGGAGACACGTGTATCATGTTGTGAATCGGTTTCGTATGATTGACCGC

GCTGGTGGCATAACGATTTCAGGTCCCCCTCTTTTATTATTCTT

>Tb927.5.2950 | Trypanosoma brucei TREU927 | Repeat of unknown function (DUF1126), putative | genomic | Tb927_05_v5.1 forward | (geneCodeEnd+0 to geneEnd+0) | length=345

GACTAATATATAAAGGTAGATAGGGTGAAAAGAAGGTCAATATGGTGATGCCACCTGACT

TGTTTGCGCCCAGAAGTGGTGCTGACTATGAGTACCAAGAATGTGGCGGTTGAGGAGATG

GTTGGTAACAAACTAGAAATGTATATTGCACGTGGCACGTTGTAGACAGCGTTACATCAA

TCATAATATGTAAATAATAGAGTGAATCGGAGGGGGAACGTGAGGACAGGCAGAGTTTTT

GTGGACGCACCGTAGATATTTCAACGTTATTTCTGCAGGACTGCCTATGTGCCTGGCGTG

GTTAGCTCATTGTAATATTGAGAATGCGTCTCCTGATCTTGCTCC

>Tb927.8.1990 | Trypanosoma brucei TREU927 | peroxidoxin (TRYP2) | genomic | Tb927_08_v5.1 forward | (geneCodeEnd+0 to geneEnd+0) | length=346

GGAAATAACATCTTTCTGGGGCACCGGTGTTGGGGGAACTCTTGTCTCACACGGGCATAA

ACTTACACAAGATTGAATATTTCTGTGTTAACTGTATCTTGGCTTGGCTATTCAAGCCAT

GGGTATGTAGTTTTGTTTTTGCCCCGGTCGCGGCCACCTACTGTGCCAGCTTGCTGGTGC

AGCTACGATGGGGAGGGAAAAAGGTGAAAAAAATACCCAGTGAGAGGTGACAGTTTTACA

GGAGCATAATACAACGCCCGTATATATAAATACGTTTTTTTTTTCTTTTATTGTGTGTGT

GTGTGTTGAGGTGTTGCTCTGAGCTGGGAAAGTGAGATGGGGGAAC

>Tb927.8.3470 | Trypanosoma brucei TREU927 | Vesicle transport v-SNARE protein N-terminus, putative | genomic | Tb927_08_v5.1 forward | (geneCodeEnd+0 to geneEnd+0) | length=346

ACATTGGTCGGCCTTGCTCTGCGTGGCGACAAATCTTTCTTATCAGTGGCCATATAGCCC

CGCACTATTAACCGACCCAGATGCTATGAGAACGAACCAAGCAACAAATGGGCTGCGGTG

CGTGCATTTGATGTGCCGTATTCGAGGGGTGGATTGACTGAGGGCACGTCGGAAGATGAT

AAAATTAGTAGTGTACTTGCTGTCTCCTTCTTGGTATCATTTGCGCGTGCTTTCTCTGTC

GCTTTCTTTCCCTCTCATATTTTATTCGTCGTGCTGGACATAAACTTCTCTCTCGTTGTA

ATGGAGTGGGAGGTGCCAGCAACTTTTAAACGCACGCGTCTAGCAC

>Tb927.10.15150 | Trypanosoma brucei TREU927 | DNA-directed RNA polymerase III subunit C11, putative (RPC11) | genomic | Tb927_10_v5.1 forward | (geneCodeEnd+0 to geneEnd+0) | length=346

GGTCGTCAGTTGCCACGTTAACACACTCCCTAACCCAACATTTAAACCCCGTGCAATGCA

CCTGCGGTTGCGGTTCTGTGGGTTGTAGGAACCGTTGCACCGGTGGATGTCCGACTAGAG

GTGTGTGCTTCCGTATTGGGGTACTCGTAACCGTACCTCATGGTTTTGAAAACTTGTTAA

CAGGTAGCGCCTGGGGTGACGGGGAGTAGAAGGTGGTATATTGTGGTGGCCGACCCCATG

CACTACATATTGGGAGAAGAGGCTTGTGATGGTGGGGACAAGGCCTAGTGGCAGAGGGGG

GTGCACAAGGGGCTGTTTGCCCGTTTACCGTTATATCCTCAGTTTT

>Tb927.10.10030 | Trypanosoma brucei TREU927 | hypothetical protein, conserved | genomic | Tb927_10_v5.1 forward | (geneCodeEnd+0 to geneEnd+0) | length=347

GTGATATGAAGTAAAAGGGAGTGCATGCGTCACTTTATTTTCATTCATCCACAGTTGTGT

TTGGATGGAAAGAGGCTATCGTGGTCGCGGCAAAGCTCTCCCCACGCCATTATGATAAGC

TTCTTCAAACTCTTTACCTTGTTCACACCGGCGGTCCATCACGTCCAAGAAAAAGAGTTA

AGCGACCACCCATGTTTCGTAAACTATACATTGTCACCTTCCTTTAACGACACGTACAGG

GGGAAATGTGGACGGGACACTGCATGGCCATGTGTAGGGTGTATGATCCCGTAGCTCACT

GCACTTCCTCACTTTACCAACTTTTTCTACCCTATAACGTCGTGTTT

>Tb927.4.5010 | Trypanosoma brucei TREU927 | calreticulin, putative | genomic | Tb927_04_v5.1 reverse | (geneCodeEnd+0 to geneEnd+0) | length=348

GAGCTCAACACACGTCCTTCATTAACATGTAAGTGAAAGTATAGTTCTTTCAACCGTTTA

TGTGGTCACGGCTTGTTCAGAGGAGTTTGTGAAGGGCGATGTCTCTCCGTGTTTTGTGTG

GTTGCTTGATATATTGCCTTGTGACATTTAAACCCATAAGACTTCAGTAAAACAGAGAAA

GGGGGAATGAAAGGTATCAAATGGGGAAAGAGAATTTCCACAGGGAGGGAAAAAAGCTAC

CAGGGTTTCCGAAAGGAGGGAAAAGAGGTGTGCTCATAAGGACGTGGTATGTAGAAGGGA

GAGAAGTACAGGAAGCGCATTTGAGGAGAAGGGTGACGAAGAATTCTA

>Tb927.8.1680 | Trypanosoma brucei TREU927 | kinetoplastid kinetochore protein 12 (kkt12) | genomic | Tb927_08_v5.1 reverse | (geneCodeEnd+0 to geneEnd+0) | length=349

AAGAGAAGTCAAGATATCAAAAAGAAGAAAAATGGCGATGATGGCTGGTTAAACGACTAA

CGCCTAGAAGTATGACGAGGAAATCGAAGAGACAGTATTAGAATGGGTGAAAAGGGTGGA

ACAACGCTAGATCAGCGCGAGAACCGCGAATTCACACATTCTCCCACCACTGAGTCCCAC

TTGTGGAGATGAGAGTGTATTAAAGGTAAGCAACAGAGGGAAGGGAAGGGTTCCTTCCCC

TCCCTCGGCTGTGGTTACTGCTCTGGGCTTTCTTACTTGCGTAACGCTTTTGCTTCTTCA

CTTTGTGTAAGGCCCCGGTAGGTAGATCGTGTTTCACCCTTGTGGATAG

>Tb927.10.9840 | Trypanosoma brucei TREU927 | chaperone protein DNAj, putative | genomic | Tb927_10_v5.1 forward | (geneCodeEnd+0 to geneEnd+0) | length=350

AGTCTACGCATTATTCTCAGCTAGCCGTGTGCATGCCACAACTACATCCGAAAGCGGTGT

ATCGATGACCATAGATTCTGGAGACTAACTTAGCTGTGTTGGTTTGGGAACTTCGCACTG

CGTGAACACTAATGCATTCAGTTTGGTCGAAACATTGTGACGGCACGCTAGTGACAACTA

CCTCAACATCACACTTCCAATAGAGTTAGTTTTTTTTTTCACTTTCTGACTTTTTTTTTA

TGTTGAGCCATCCCACAAGTGCACGCGTATTCGCGTATAGACGCTCTCATGAAAGCACAA

AGGCAGAGAGCGCGCTGCTGCAAGTGTCTACCGCTGTTTTTCTACTTTTC

>Tb927.11.13470 | Trypanosoma brucei TREU927 | PPPDE putative peptidase domain containing protein, putative | genomic | Tb927_11_v5.1 reverse | (geneCodeEnd+0 to geneEnd+0) | length=352

AAGCTGACGGAGGAGGTAACTAAGCGTCACCATGTGCTCTAATCCGTTCCCATTTAAAGA

CAACCAAGAGTGAAACGGTTCTGGGACCGCAAAAGGATCTCTTGGTTGGTTTTTTTTATC

TTTTTCTCCGGATTCGCGTGTTTGTGGAGAGAAGCCGAAGGGTAGGGAAATAGTTGGCGC

ATCATCCTGTCGGCACGCAGCAAATGTCTCCGGATTCATCTGTTGTTACAAGTTTCCCTC

CTTCCCCTCCCCCTGCTCTGGAACCAGAGGGGGCATAATCTTTCATTCAACAGGAGTTGC

ACCAGTGCAGATAGTGCTACTTAATCGCTATCGGTTTGGACGGCGGTTCCTG

>Tb927.7.6420 | Trypanosoma brucei TREU927 | hypothetical protein, conserved | genomic | Tb927_07_v5.1 reverse | (geneCodeEnd+0 to geneEnd+0) | length=353

GGCTGGAGAGGTTTCAGAATCTTTGCGTCTGAGTGAGGCATTAAATATTCGACATGAGGA

AAACAAGGTGCCGCGAATACGGCCGGAAAATCATTGCTTCAAATGTATTTATTTACTTGT

TTGTATTTATGTGAATTGTCTGAGTGATGAGTACAGAGACGGTTTCTCGTCTTCCAATCG

TCGACAGTTGTAGTGAAATGCATATTATGTGCGTAGGGATGAGTGCCGAGACGGGAAATT

AATTGTTAGGAGTAACTCGTTCGAAATATTTCGTAACAGGACGTAGAGGAAGGGTATCAG

TATTTTTTCCCCATTTGTTGTTAACCGTGATTTTTTTGTTTCCCATTTTGCTG

>Tb927.11.4900 | Trypanosoma brucei TREU927 | guanine nucleotide-binding beta subunit-like protein, WD40/YVTN repeat-like-containing protein | genomic | Tb927_11_v5.1 forward | (geneCodeEnd+0 to geneEnd+0) | length=354

ACAGAGGGTTCTAAAAAACAATCGGACAAAAAAGAAAAATAATCAAAACAAAATGATTTA

AAATATAAAAGAACATCTCTTTATTCCTCTTGCCTTTTTTGACCCTTCTAGTATCCTCCC

CCAACACCTACCTGCCCTTTCTTCCCTCGTTTCCACCCGTACACGTGTCCGTCAGTCGAG

CTTATGAATGAGTGTGCGATGGAAACTGATGCGGAGGGGTGGGGTTGGGGGGGAGGAGGG

AAGGGCCAACGCGTGTGAGTAGGGAGGGAATTGTTGCTGTAACTTTAACTTTCCACCAAA

CGATGTGTTAGTGAGCGAGTTGTGCATGGGAGTTGGAAACAGACAAAAACTATG

>Tb927.11.4880 | Trypanosoma brucei TREU927 | Calmodulin-binding, putative | genomic | Tb927_11_v5.1 forward | (geneCodeEnd+0 to geneEnd+0) | length=354

GAGATTAATTTTTTTTCTTTGACTACTCTCCTTTTCTCACCACAACTTACCGCATGTTTG

TTTGTTTTGCACTTTTTGCATGTGTGGAGGTGCTGAGGGAGAAGAGGGCGCACGGCCGTC

GGAGATAGAGGGCGAATAATTATGCGAAAGTGGAGGGTTGGTTGGCTTCTCATGTGCTCG

TGGCTAAGAACCGGTTTGCAGGAGCAGTTTAGTTTCGTGCTAGCGAGTCTACATACCCCC

AGTCTATTCCAGATTCCTCTTTCTTTTTCTTTTAGCGCCATCTCCGTCATCCGGCTACGT

AGATGTGTCAAGCAGTTTATGATATGTATAATTGTGTGCACCTCATCATTCTTG

>Tb927.8.4390 | Trypanosoma brucei TREU927 | Translation initiation factor eIF-2B subunit beta, putative (EIF2BB) | genomic | Tb927_08_v5.1 reverse | (geneCodeEnd+0 to geneEnd+0) | length=354

ATCGTGATGCTTGCGTTTGCTTAATGCTGTTGCTTTGCGGTAGCGGAGGCAACTTGGGGG

AGGGGGGGGGGGAAGAGTGATGATTTTGGAATATACACAGAGAGAAGATGGTAATGAACG

AACCGTGTGACTGTTGACTTGAAAGGGGGCCGGGGTGTTGATAGTCGGATGCAGTAGCGA

GACCATACTGGGGGTTCCCCTCGTTTTTTTCCAGTTTTCGCTCCATTATTTTTCCTCTCC

TATTGCGAGTATGCGGGAGAAAGGGTGATGAGTGTAACACATTTCTCAAGTATGCGGTGT

TAGCAGGCTGCACTCATTATTGAGGTTACTGTTTCTTGTTTCGTGACTTCCTGC

>Tb927.3.1600 | Trypanosoma brucei TREU927 | Tim10/DDP family zinc finger, putative | genomic | Tb927_03_v5.1 reverse | (geneCodeEnd+0 to geneEnd+0) | length=355

GTAAACGTCGTTAAGCATTTTGCCTTCAACGTTTGGCCTACACATATGTTTCTATGACTG

TACATTATATGAACTTGTGTTTTCGGCGAAAGCGTTCGGCGAAAGCTCCCGTACTTTTAT

GTAATCCCTGAGGTGCCGCATAACTTGCCCTGACCTTTTAATTTTTGGAGATGCTGCAGT

TACGCCGTGACGGTAGAGGCCCTTAAGCGCGCAAAGCATGAAGGGATGCTGAAAGAGAAC

GGGCGAAGATGATATAAATGGCTCTTGTCGTTGTTTTGTCTACCTCGGTTTGGTTTTGTG

TGTTTGTGTGTCAGAGAGAGAGAGAAGGGGAAAAGGAAGTTGTTACTGGGGAGCG

>Tb927.8.650 | Trypanosoma brucei TREU927 | cation-transporting ATPase, putative | genomic | Tb927_08_v5.1 reverse | (geneCodeEnd+0 to geneEnd+0) | length=355

AGGTTATTATGGTGCGGTAATTTTTTCTTGGCATTTGTTTGGCAGGTACTCTCACGCAGG

GTTGATGCTAGTACCGTAGGGTATCGCAAACACGGCGGGGAGAGAAGAATGCATTGGCAA

TGTTTGTGTGCATGTGTGTGTTTGTGTGTTGTACCCACGCGCCCAAATGATGAAATACAC

ATCAAAGGAGTAAGTATGGTTGACACATTTGTGTTGGCATTCGCATTATTTGAATGAGAG

ATTAAAAAAAACAGGAAAGAAGGAAAGAAAAGACGTCCTAACGCACATCGCATCGATGAT

GAAAGTGGAACCGTGTGCTGTTTGTGTAACGGATTAACTGATTCTCTTAACCCTT

>Tb927.10.6490 | Trypanosoma brucei TREU927 | KIAA1430 homologue, putative | genomic | Tb927_10_v5.1 forward | (geneCodeEnd+0 to geneEnd+0) | length=356

GTAGATAATGTTCTGCAGCCCCACTCTGCGTGCTGGCTGTTACTTCTTTGCAAGTACTTA

GTCGCCCACTTTTACCCCTCTCCCTTGGGCTGCGGCATCCTTTTTTTTTTTTTTAGCAGG

CTACCGCTCCACTTATGTATGTATTCTGTGAAGTTTACTAGAGACCCTGCATATTTTATC

CGTGTGGCGTCTCAAGTCGTGGCCCACCCGCCATATATGTGAGCACACACGCATATATAA

ATATATATATGAATCTGATTGTTTGTGAAATAGATGTGTCAACTACTGGGGAAAGAGCAA

CGACACAAACGGAGGAAACGCCACTCACTTAGCCGACGTTGTCTTTCTCGTGCCTT

>Tb927.9.6450 | Trypanosoma brucei TREU927 | peroxisomal membrane protein (Pex16), putative | genomic | Tb927_09_v5.1 forward | (geneCodeEnd+0 to geneEnd+0) | length=357

AATGCAAGGACGATCATGTGGAGGTTGGAAGGAGTCGCTACCTTGTATGCATACACAAAT

TTTAAACGAACAAAAGTAAATGAATGCATCTCTTTTTGGTGCTGAGTTGGGTGAAGAAGC

AATATGAAAAGATGATGTGACGGGGAAAGCTACCGGCAGCTCCGATAAGTGAGTGGGGTG

CTGTTCTAAGCAATGAGAAAGAAATGTAAGGAACAACTCGTGGCTATTCCCCCCCCCAGG

ACGTGGAGAATTCGAAATCCCTCTGTTTCATTCTATTTAAAATGCTAAACAGAAAGAGGG

TAGTAACTGCTTACACGCGTGGAGTTCTGCCTAACTACTGGGCATAGGCGCTGCAGT

>Tb927.7.7010 | Trypanosoma brucei TREU927 | hypothetical protein, conserved | genomic | Tb927_07_v5.1 reverse | (geneCodeEnd+0 to geneEnd+0) | length=358

GTGGCGTGGGAAGAAAATGAAAAGAAAAGAAAAGGAGGATGGAAGTTGCAGCGACGACGC

ACAAATGCATTTGGTGTGCAGAATTAGTGCTGGAGTTTACTCAATTGTTAGTTATGTTTC

CCATAACGTTGAAAGGGGCAACAGCATGGAGTGGGGGACCAGTAAGTGGAGCAGAAGGAA

GGTGAGTTTTATAGGGCCCCGTGCACAGTACATAACTCCTTTTACTTCAATGAGGTGAGC

GGATGGGCAGCTATAAAAGCCGAGCCGGTGATTTGAAACGTATCGTTGTTGGATGGAGAT

ATATTTATACGCTGGCAGATATTCATCTCCCTCCTTTTCGTGTGTGTTCTTCGTCTTT

>Tb927.10.4690 | Trypanosoma brucei TREU927 | dynein light chain, putative | genomic | Tb927_10_v5.1 reverse | (geneCodeEnd+0 to geneEnd+0) | length=358

GGATCAGCCGCACCTGTTTACTGGAGAATGTAGATATTGGCTGTACTTTCGGCCAGAGGA

TGGCGTTGGGAGGAGGGCAAGAGGACGGAGCAAAACAGCAGAAATGCCAAAATAGATGTT

CTGGATAGCTGCGCTGGGGGCGAATCAATAACGGACGCTCTGGTGTGTGATGCAAGTGGT

ACATCAGAACTTTTGAGAAAGGAGAACATGTGGTTTTACCCCTCCAGTGTTGGGCGCCCT

TCTTTCGTAAAGTACCTTTTGTTCCCTTACCAAAAGTAGTTCTAGGTAATGTGTTGTGAC

AACTGGGTTCCCTTCACGCTTTGGCCTATACTTTTCCGCTCCTGGCTCATTATCCTCG

>Tb927.10.15250 | Trypanosoma brucei TREU927 | paraflagellar rod component, putative (PFC15) | genomic | Tb927_10_v5.1 forward | (geneCodeEnd+0 to geneEnd+0) | length=358

AAAAAAAAAAAGAAAATAATAATGTATCAATGCAACAAGTAGATGGGAAAGAGAGCGGTG

ATGCGAAGTAATATCAAAAGAGAGATATAGGAAAGAAGGAGATGAGGGATTTGTGTATGT

GTGTGTGTGTGTGTAGAGTACGAAATATTGTCAACCAGGGGATGAGGTTTTAGGAAGAGG

TTAGGAATCACTGTTGCTGTTATTACTATTATCACCATTAATAGAATTATATGGGCGACC

CGGTACGCAGTGGCTACTCTTGTGCAAAGAAAAAAAAATGCAAATTCAATCTCGTCAGTT

CAAGGGAAGGAATAAACTCCCAGTTTATTGGCTGAAATAATTGAGTTGAGTGAGCAAG

>Tb927.6.1570 | Trypanosoma brucei TREU927 | 2-hydroxy-3-oxopropionate reductase, putative | genomic | Tb927_06_v5.1 reverse | (geneCodeEnd+0 to geneEnd+0) | length=359

ACAATCTCCCCAATCATCCTCAGATCGGTAACTGTTTGTCGTTTTCAACAAACGCATACG

CACACGCACATGGGCATTTTTCTTCTTTTATATTATTTTAATATACGAATTGAGGGACCT

GCAAGCAGGGGAGGGGGTAGTGCTAAAACGAAATTACATAATCGTTGTACTTTGGGGGGT

CCAAGGGAATAAGACTAACGACGAGAACGTTTGTGTGTACGTGTGTTCCTCCTGCTTTTG

TTTCACCTGGTGAACACGGTTCCAAGGGACTTTTTATTTGACGCTCCTTTTCCGTTCCCT

TTATCCCCACAGCGGGCATGGCAAGTGTGTGAGTGGGGTTCTGTCGGCCCCTCTTTTAC

>Tb927.10.4710 | Trypanosoma brucei TREU927 | 20S proteasome subunit, proteasome subunit beta type-2, putative (PSB4) | genomic | Tb927_10_v5.1 reverse | (geneCodeEnd+0 to geneEnd+0) | length=360

GAGAAATCAACTTCGCCATGCTTCGGATGGTGTCACTTGTTCCTTCGGCATGTATTTATC

TCTGCTTCGGGGGACTCCCCCCGCCTCCCGTTACCAGCTCCACACTTCGCCTTTCGTGTT

GTTTGCGTTGAAAAAGAAACGGAAAAGGAATTGAAGAAGGAGAGCGAAAGAAAGAACCAT

GGGGATTTTATGGCACGAGCAACGGCTAGGTGGGCGGCTGGTGAGGGAGTGAATTCAAAG

GTAAATACTTTCAGCTTTTTCCTTTTACGAGCGGCATTTTATTCCCTCCCGCGTGATGTC

GTCACAAAGAAGTGGCATGCAATGGGAAATGTGTCGCGAGTTTACCCTCTTCTTGTGTGT

>Tb927.5.4320 | Trypanosoma brucei TREU927 | zinc finger protein family member, putative (FIP1) | genomic | Tb927_05_v5.1 reverse | (geneCodeEnd+0 to geneEnd+0) | length=360

ACCGCCAGTTTACTTGAGTTAGAGGAACTCGTGTAAACGCGAGGGGATATACATTTGTGT

TCTCACACAGTGGCAACACTAACCAATAACTGCCTTGACTGTTGTGGCGAGGGTTGTTTT

TGTGCATTTATTACTACTTTCATTTATTACCGTGGCTGAATGGTGGAAGTCGGTGGACAT

GATATGAGAAGCAGTTGTAGGCTCAGTGTACGTCTTGTTATGGTGTGAAAGGGAGTGGAA

ATAATAAAATGAAGAAAAGTTTGGGGCAACTGTTGTTTCGTTATCACCCGCGTTGCGCTG

TTTTGTGTAACCTTCATGCCGATTCGTTCCCTGTTTTCTTTGCCACATTCTGTGTCCCTT

>Tb927.10.7190 | Trypanosoma brucei TREU927 | D-tyrosyl-tRNA deacylase, putative | genomic | Tb927_10_v5.1 reverse | (geneCodeEnd+0 to geneEnd+0) | length=360

ACACTCGTCAAGCGTCAGTACCGCGTGGTGCTTCCACAATCCTCCTGTCGCATGCTCAGC

AGATAAATATACCTCATATTCTCAGACTGATTGAAGAGCTGGCCGGATGGGGGTTGATAA

AAAGACTTTGCCTGCTAGGCTTCTCTTTGGGGATCGTTTCTTCCGGTGTTGGGGTTCGAC

GTGAGCCACATCGATTGGAATTCTCAGACATTTTTTGTGGTCCACGGTCAATTCTCCCTT

CAACTTGTTTTGTGTACGGCACCTTTGTTGCAAGTGTGGCAGTGCGGAGAAGCTTCAGTG

GCCTCAACGGTGGGGGAAGAAACGGATAAGGTATGTGCCTTAAAAGGGTGAGTTTGTTTG

>Tb927.9.13310 | Trypanosoma brucei TREU927 | Yos1-like, putative | genomic | Tb927_09_v5.1 reverse | (geneCodeEnd+0 to geneEnd+0) | length=361

AAAAGAAGGTGCAAGGCAAGCGAGAAGAGCGGGGAAGCGACAATGCGTCGGTATAATCAA

CAAGGTTTCCTGTTCAGTAGGGTTCCTCCGCTCCTACTGGCACGCTTTCAACGCGCAGAG

AGGCGGGGATAGTCACCGTATGCAAAGACCAGCGCATGTTATGCGGCACCTACTTTCACG

AAGTATTTCTCTGGGAAGTAACTTAAGTGTTTACCGCGGAAGGAGTTTGTGTCCGTTCTC

TCACGATTTTCCTACAGAGAAAAAGCACTGCTGTCTAAACCTCTCTATGTTCCAGAGGAA

ATCAAAAATGTTCCCCCGACTATCGCAGAGCTAGAGAAGATGCAATCCGATTGTTGTTGT

C

>Tb927.11.1900 | Trypanosoma brucei TREU927 | T-complex protein 1, beta subunit, putative | genomic | Tb927_11_v5.1 reverse | (geneCodeEnd+0 to geneEnd+0) | length=361

AAGCAAATGAGCAGTGTTGCCAAATGAAAAAGATTTTATTTATTAATCGTTACTAGCATT

TGTCGGCGAAGCATTCATAGGTAGAAGACGGGGGGTCAAAGGTATGCGAATGCGAAATGT

GTGTTGGAAACCGCGGGAGGACTCACCAACCCATCGCGTTTTACCGTGAAAAGTGGAGCT

GTACATTTCCGTCCCGTCATTTTGAGAACGTAACTTTAACACATAATTATAGATGGGGGA

AGCTTAATGGTAGGAGAGATATTGAAACTTAAGTGTGAGGGAAGCCAATATAAGAGAGGG

AAACAGAACCAGCGGAGTCGATTTTTTGTTGTTAAAAGGTTTTTTCTTTACACGTTTAGT

G

>Tb927.4.2740 | Trypanosoma brucei TREU927 | p25-alpha, putative | genomic | Tb927_04_v5.1 forward | (geneCodeEnd+0 to geneEnd+0) | length=363

AAACAAAAGAAATTGTAATATGTTATATTCATTTGATATATATATATATATATATATATA

TATTTGTGGTACTTGTAGGTGAAGGATGAAAAAGGGGATTACGGAAAAGAAACAAAGAAA

AAAGTAAAAGAAGAATAAGAATAAGAAGGGATGGAATAATTCTTTTTTTTAAGAAAAAAA

AAGAAAGAAAGGAAAAAATATGAGGGGGAGTGTGTGGGAGGACGGAAGGATGGAAAAAAA

AAAAGTCACAAGAAGTTTGAAAAAGAAGAAAAGAGAACGAAGTGAAGTGAAATGAATATG

AAATATAAATGCGGTGACTAGGAGAAAGGAAGAAGAAGAAAGTGAATTTTCGTCCCAAGA

TTG

>Tb927.10.4510 | Trypanosoma brucei TREU927 | PUB domain containing protein, putative | genomic | Tb927_10_v5.1 reverse | (geneCodeEnd+0 to geneEnd+0) | length=364

ACCGTCTTGGATTGGAACCTCTTTAGTTGCCTTTCATATAGTTTCGTCGAACTCCCGTTT

TCTTGATTCTAGTTTCACCTCACGTGCGAATGAGGCGCAACCTTGGGGTAACATTAAAAG

CGGCACCAGGAAAGAAAGTGAATTTTGAAGACGAAGACGAGGAGGAGGAGGAATCAGATA

AAGGGGGAGAGAAGCGCATATTGCAGAAGCACTGAAGGTAAGGACACAAGGTCAGCAGGA

AAAGAAAGTTAGGTGAAGAGGAAACTGCTCTCCCTGAGAGCAGGTGAGTGGAAGGTAACA

AATCAAAGAAAAGGCGGGCAGCAGGGTTGTTCTGCTTTCCTCCTTTCGTGTTTGTGCTGC

GTAT

>Tb927.11.3680 | Trypanosoma brucei TREU927 | calmodulin, putative | genomic | Tb927_11_v5.1 forward | (geneCodeEnd+0 to geneEnd+0) | length=364

GAGGGGGATGAGATAGGATGAGATGGAGTAAGTGCGTTTGTATTGTACAAGGGCGAAGTA

ATGGTCTTTTTTTCCCCCCCCCTTTGCTGCCACCGTCGTCGCTGGTGGGAGTGGCTGTAT

CCCATGTTGTGGTATCATCATCTCCTTTTAGATTCCGCCTCCCAGCGCTAGTGTGATATT

TTTATTTCGCCTTGAAAATGGCTCCGCCCGTCTCGCAACACCTCAGCGCAACTTTAGAGT

ACAAAGGAGAGAAAGAGTCGCACTACTTCTGTTTCTCCCCTTCCTCACTTCCATTCATAT

GTTTATGTGAGATTTTTACGGGCATCTGTGCACCTGCTTCGTAGTTCTTGCTTATACCTA

CTTT

>Tb927.8.4640 | Trypanosoma brucei TREU927 | flagellar protofilament ribbon protein, putative | genomic | Tb927_08_v5.1 reverse | (geneCodeEnd+0 to geneEnd+0) | length=365

GTCAACTAGAAGAATGAGGGGGAGGGGTGGCGTGCGCGCCGTTGGACACAGGGAATCTGT

GGCATTTCTTTTTGTCTAACTTCCACATTTCAACATTTGTGGTGGGTGCATGTCAATTTT

GAAGAAGCCTCTTTTTTTTGTTTCCAGTAAATAGAAGAAAAATGATATATTTATATATAT

ATGCGTATATGTACTGTGTATTCTTGCACGTAGAGGCGAAAACACACGAAGGAAGTGCAT

AAAAGGGGGAGAGAGCACGAGATGAACTTCTGTAAGTGTCGTTGTCGTGAGATGTATTCA

TCACTTAATCGTGAGTTTTTTGTATGCTGTGGGGTGCACTTGCCCCAATCGTCTTTTCTC

TGATG

>Tb927.6.2420 | Trypanosoma brucei TREU927 | p22 protein precursor | genomic | Tb927_06_v5.1 forward | (geneCodeEnd+0 to geneEnd+0) | length=366

ACGTAAAGAGGAGAGGAGGGGGGGGGGCGGGAGCACAACACAAAAAATAAAGAGAACTGT

GGAAAAAAAACAAGAAAGGGGTCTATAACTAGACTGCTCCAGTAAACATCTTTGGTTCCA

TTGTTGCTGGTGTTGCAAAGCTACATTTAATGAATTCCTGACTTTTCTGTTTGACTCTCT

TAGTTATTTTTGTTTGTGCGGAAATCATTTTTTTTTGTTTTTACTTCTCGGGTCGAAGGG

GCAGCAAAAGTACCGGCAGAAAGACGATATCGAAGATGAAAATGAAGGGGAGGGGGAGGG

GAGAACACGGCGGCGTGATGCTTGTGGTAGTGGACAGTGGAAGGACAAGGGAGGGGCGAT

GATGCG

>Tb927.11.3570 | Trypanosoma brucei TREU927 | aminopeptidase, putative, metallo-peptidase, Clan MA(E) Family M1 | genomic | Tb927_11_v5.1 forward | (geneCodeEnd+0 to geneEnd+0) | length=367

GCTTTTCTTTCGCCTTCATATACGTTATTACCATTAGAATTGGCTGTTGCTAAATTAGTG

GATGCAGCTGTTGGCTAAAATTAGGTGGGAAAATGAGTAAAAAACTTTAGGGATAGAAGT

GAATGTAGTAAAAGGTGGCTCAATCATGGGTAGGAAAGTAAAAACGAGTTGGATTTTGAT

GAAGGGGGAATATCGTCGAGGAATGGTCCATTCTTCCTCGTCGCGATGATTTCGTAGTGG

GCACGTGCCACAGTGCTTCTGGCGGGAGAGTGGGTTTGTCAGGAGGTGCGGCGATAAAGG

AATAGTTGTATTGGGTGTATACACGTATACTTGCACACTTGTTTCACACCAACGCTTCAT

CACTTTT

>Tb927.11.8750 | Trypanosoma brucei TREU927 | hypothetical protein, conserved | genomic | Tb927_11_v5.1 forward | (geneCodeEnd+0 to geneEnd+0) | length=367

ACAGAACCACGAACTTTTGCGCGGCAACGTTCCTGCAACTCTTGCCTCAAGTCGAGAGAG

GAGTTGAAAAAAAGAAAGGTGTGTGACACGTGATCTTTGGTCTTTCACGCCATGTGATGT

CAGGATCACGCTACCGTGGACAGAGGGAATAGACTTGAATGGTAGAACTCAGTTTTTAGC

TTTTCTCCCATTGTTCTTCGACACTTTTTTACGGCCGTCGGTAACCACTACAGTTTGAGT

CGCACCTGCTGTTTCTAACATAAAGGGGAGTTTTGCCCTACTTCTCAGTGTGTGGTGTTT

CAGCAAGTTGAAGAAGAAAGGGAAAGGATATCTCAGTTCTCCGGTTTGTGGGCCCTTCTA

AGAATTG

>Tb927.5.2940 | Trypanosoma brucei TREU927 | stress-induced protein sti1, putative | genomic | Tb927_05_v5.1 forward | (geneCodeEnd+0 to geneEnd+0) | length=367

GGTGAGTGGGAACGTGCGAAAGAAGCCCAGATGATGCCAGTGGGGTGGTGAAAAAAGACA

AAAAACCAATGCTGTCTGGTGCCCTCCCCCTCAACATCTGTGAGCGATGTGGACTCGTGC

CTTCTCAAGAACCACGCACCTCCTGCCAGGCGCTGCGGTGCCACGGAGTGCACTTTGTGG

TGGTTGTTTTACTTATCTGATAAGCATCAATATTATTATTATTATTACTATTATTATTAT

TATTACTGTTTGTGTCTTTCTTTCACCTTCTTTTTTATGAAGAGAAAGTTGAATGAACGA

ATAAGGGTGTGCCCGTTTTTTTGTTACCACAATTGACGTCATCACCCTTGTCGCCTGACT

CACACTG

>Tb927.1.2330 | Trypanosoma brucei TREU927 | beta tubulin | genomic | Tb927_01_v5.1 forward | (geneCodeEnd+0 to geneEnd+0) | length=368

GACGCGGACGGGGCATTTCCCGTTCGTCATTAGCAGTAGGTAATGAAGATGTTTGTTTCT

CGTCCCCTTTCTCCTTCGTCCTTCTGTCATTTTGTTCTTTTGTGTTTATGTTTTGTTGTT

TTTTTCTTTAATTTTTTTTTTCTTCCACGTTTGTGTACATCCGCGCGCCACTCTATTCAG

AGAGCCACGGATAGTAGAGGAGGTGGGAAGGGTATATGAGGGACACGCGTACCATGATGT

GGGATGTATTGGGGTCCCTGTCTGTCCTTACGTGACTATGTATGAACCGTCACGTGTAAG

ATGAGCTAGTGAGATCAACAGTACAACTCATCAACACGCCTTCTTCTCGTTAAATGTACA

CAATCTTG

>Tb927.4.1230 | Trypanosoma brucei TREU927 | hypothetical protein | genomic | Tb927_04_v5.1 reverse | (geneCodeEnd+0 to geneEnd+0) | length=370

AACGGCAGTTTAATCGACCAATTTGCTCTAATTTTTTCTTCCGCGAGCGTGAGCGCGCAC

ATACACACATGCACACACACACACACAATATATATACATATATATAGTTTGCACCCACCT

TGTGCCGATGTTGTGCTTGTGCATGCTCCGAAAAAAAGACTAAAACAGTATATGTGTGTA

TACAAACAGGTATAAATCTTATATCTTTTGCTTCAAATACATTTTTTCACCTATAAACTG

TTGTTTAGCCATTTGTACAGGCTAGATTTTGTACGTGAAATACAGTAGTATTTACTTTGA

GGAGGAGGGGGAGGAGGAAAAGGGAAGAAAAGAAAAAGAACATATACAATATCATAACTA

ACACGCGCAC

>Tb927.10.14750 | Trypanosoma brucei TREU927 | fibrillarin, putative | genomic | Tb927_10_v5.1 reverse | (geneCodeEnd+0 to geneEnd+0) | length=370

GGAATAGGAATGTGGACAAAAGGATGTAGGAAAGCCACGTTTGGTGGGAGAGGCTGCGCG

GGGAGCTCTTTGTCTGACTGTGTGATCTGAGATTATATGTTTGTGTTTCACGTTTCCTAT

GCCCGGGTTGGAGTGGGGAGGGGAAACGAACATTAAGAAGGGACCAGCGAATAACAGCGA

GAAAAACTATTATAAGGCAAGAGAAGAAAGATGAATATGCAACGGAAGTAAATGGAAGTT

TTCTGTATGAGTGGGGGAAAATAAAAGAATAATGAAATCATTTATATGATCGACGTTAAT

CATTTTACGTAATAACAGGGACGTAACTGACTGTGTGGTATGTGGGCGACTACATTGCGA

TTGTTTGCGC

>Tb927.6.4280 | Trypanosoma brucei TREU927 | glyceraldehyde 3-phosphate dehydrogenase, glycosomal (GAPDH) | genomic | Tb927_06_v5.1 reverse | (geneCodeEnd+0 to geneEnd+0) | length=371

AGTGATGTTCTCGCTCCCTACGCGCTTGGCGTGTGGTGCGGGTGTAAGAGCCCTGCATTT

AGCACTATGACCACCCGTACTCTTGTTTTGTAATGCACTTTGGTTTCGAATGACCAATTG

CTACGACAAACTGTCTTAGCAGACCAGATTAAGAATATATGTGTGACGGACAAGGAGGGA

TGGCAAAGCCCCTATGTGTAGAATGGAACATCCGATGAGCACTTTTGCGTCTTTGATATT

TAAGCAGTAATGTGTGGAGGGGGAGAGAATGCTACAAAACGTAAGAGATTTGATTTTTAT

GTACCTAAAGGAATAATATTCTAGGGTAGTGGGAGTAAGTGAAAATTGGGGAAAATATAT

GAATTGTGTGG

>Tb927.10.13130 | Trypanosoma brucei TREU927 | UTP-glucose-1-phosphate uridylyltransferase | genomic | Tb927_10_v5.1 reverse | (geneCodeEnd+0 to geneEnd+0) | length=371

GGTGATAAAACAGCTTACCTGTCACATCTAAAGAGAAAAGGGGGGGAGGTTAGGAAAGTG

TGGTACACACAAGTACTTGCATTATCTGATGCTGGGCATATGTGTTTACCACTGCTTGCA

GCGCTGACAACAGCTCTATGGCTGTTTTTTTTTTGTAGAGTACAAAACGGATTTATGGAG

TTTATTTTTCAATGTGAGGCTCACATTTGCCCTCTCTTGTCTGCGAACATATTTTTTTTA

GCAGCTTTAGCGATATCGATAACAGTGAAAATCCAATAAACTTTGGGAGTCGGAGGGAGG

AGGAGGAACCTTTCCTTCCGCTCCTTTTCCCCTTTTAACCCCACTTCGTTTACCTGTCTT

TGCATGCAGTT

>Tb927.9.9670 | Trypanosoma brucei TREU927 | proteasome alpha 1 subunit, putative, 20S proteasome subunit alpha-6, (putative) (TbPSA6) | genomic | Tb927_09_v5.1 reverse | (geneCodeEnd+0 to geneEnd+0) | length=373

GCTTGCCGCGTATGTTTTTGTACGCGTACATTCATGTGGTGAACTCTAGGTTCCCCAAGA

CTCCATGAAGGGGGTTTAGTGGAGGGAAAGGGAGAAGGCGAGGGGGCGGGATTGAACGTA

AACAGAGAAAGGGGGAGTGACATGTGTGGATGATCTTAGAACTAACTGCCGAGAAACGAA

CCGGTGCATGTCACAAGCGCTCATTTTTTTTTTCACGTTTTGTGGGGTGCTAACGTTTGA

AAGTTTATTTTTGTTTTACTATCTTTGCTCCTCCCCTTTTTATCTTCATTCTGGAGTGCA

TGGGTAATCGAGATGTGGCCGGGAATACTTCTGTGCCTTCTCTCCCCTTCTTGTCATCTT

TCTCCCTTTACTG

>Tb927.3.1260 | Trypanosoma brucei TREU927 | hypothetical protein, conserved | genomic | Tb927_03_v5.1 forward | (geneCodeEnd+0 to geneEnd+0) | length=374

GAGTTGGCCGCATTCAGAGCGTGAGGAAGTTGGAAAAGGGACAAGATTAGGTCTTTATGT

TCTACAAAACGTTTTCGTTGGAGTAGAATAGCACAAACTAGAAGAAAAAAGAAAAGAAAA

AAAGACGATACCCCGTGCTTTCGTGCTGTCATATTGGTGTTGGTTTGTTCCAACGAAGGT

TTTATGCCCACCTGTATCATTTCCTCCACGAGGTCTTCCTCTTTGTTTTTCCTTCCCCAC

TTTTTGATTGGCTCTTCCGGTTTTTACATTACCTCATGCGCACGTGGTGAAGTTTCCCAC

ACTTTGGAAAGGGAAAGGGGGAACCAATAGCGGAACAATACGTGTTTGTTTTCCCGTCTT

TCCCCCCAAATTTG

>Tb927.10.12700 | Trypanosoma brucei TREU927 | pyruvate dehydrogenase E1 alpha subunit, putative | genomic | Tb927_10_v5.1 reverse | (geneCodeEnd+0 to geneEnd+0) | length=375

AACGGCTGACCCAGTGCTATACAGTGAGGTCACTGGTAGCAGGGGATCGTCTAATATAGT

TTTTGAGCGTGCGGAAGCGGTAAGAAAGGAATCCCCAGATACCTCGTGTTATTCCGTCAC

TGGGCTGCCAAATTGCCACTTACACGATGAAAGCGCACCCACCTCAAACACAAAACAAGT

CACGGATGCATTCTTCCGATTCGTCAGCTATTCGGCGTGGCTCCACGCGCTCATGAAGAA

GGTGCTGGGGGCTCTCTTTAGGTAATGCACGGGTGAACGGGGAAATAGGCGAACGAGTTA

TCCTATCACTGAAGTGCGAGAAAGTGGGAAAAGATAACAGGAGATGAAAAGGTGATGTAA

CGTCAGGGAACGATT

>Tb927.7.2070 | Trypanosoma brucei TREU927 | heat shock protein DNAJ, putative | genomic | Tb927_07_v5.1 reverse | (geneCodeEnd+0 to geneEnd+0) | length=375

ATCGTCACCCCTCAACGCGAAGTAAAAAAGAAGGCAAGAAAGAAAGGAGAAAAGGGAGGG

GTAGCGGTAGCAGCAAAAACAGTAGCAAAACTTTTTAAAAAGTGACGAAAGGATTAGGGT

GTTTTAAATTATTTTACTGTGCATCGACGTAGTGCCTCTTTTCACTCGTGCGCTCCACTT

GCACCTTTTATGCTGCTGATGCTATCATTATTGTTATTATTGTCATTAAAAAAGTAAAAA

AAATAATAATAATATATAGGTAGGGAAGTGCGTGGAAAAGTTTAACCACAGTGAGTGGCA

CCGTCTTAAGTAGCCTCATTTCCTCGCAATTCGCTGAGAGGAAGGAATGGGGGGAGATTA

TGTTGCGCTGTTGCG

>Tb927.4.860 | Trypanosoma brucei TREU927 | hypothetical protein, conserved | genomic | Tb927_04_v5.1 reverse | (geneCodeEnd+0 to geneEnd+0) | length=376

ACGTTAGTGCGGAGGATTTGCATGTTTGTGTTTGTGTTGAGGAGAACTCAAACTTTTATT

TCAAGAAAAAAAGTTCGTAACTGTCTCGATTTTCTGTGACTGTTTGGCTGCCGGTGCCTG

TGTTTGTGTTTTTGTGCCTGTGTGTTGTAGTTTTGTCTGCCTACCAACACCATTCTTACT

GATTTTTTTGTTGGTGATGTCCATGTCCTCCATATGGGGAGCTAGAGAGATGTGTCGCTG

CTGCTGCTGTGCGCGTATTGGCAAAATGCTCCAAGCAAGCGCTTACACGTGCACTAGAGG

CTACGCATAACGTGCCTGATGTCACTCCCCTTTGTTGATGTTATAGTGCGAATTGAGGCA

GTTTGTTTCCTCTCCT

>Tb927.11.420 | Trypanosoma brucei TREU927 | Fcf2 pre-rRNA processing, putative | genomic | Tb927_11_v5.1 reverse | (geneCodeEnd+0 to geneEnd+0) | length=377

ATTTGTAAGCGTCAGTGCAACATGGAATTAACAACGTTGGGCCGTACCCATCAGCGTCCG

CGCCTCGCCATGGAGGTGTCCTCAAAGCACTTTTTTTTTTCTGTTTTACATTTCGCCAGT

CGTAATTTCCTCCGTCCCAATTTTATGGTTGTTCTTTAGTTGGGCTTACGCTGAAGGTGA

GCAGTGAGGCGGGGATTTATTGATTTGAAGAGGCGAGCCGCAGTGGTGAAAGAAAAACAC

GAAAGAGAGTCATAAAAGGTCATCGTGATGTGTTACCGTACGACCGCGCTGTACCGTGAA

TGGGGGAGGGTAAACTGAAACAGAATGTTATGGTTTGGTCTTTCCGTGTCTGTTCCTTTA

CCACTATTATTATTATT

>Tb927.11.6970 | Trypanosoma brucei TREU927 | NADH-cytochrome b5 reductase-like protein | genomic | Tb927_11_v5.1 reverse | (geneCodeEnd+0 to geneEnd+0) | length=378

ATTCGGTGCCGGATGCGTTCTACCTTCTAACATCGTTTGCGTTGGGTTGTTGCTGTTTTG

GGGGTGATTTGAAGAGGAGTAGAGTAGTTGGTCGTTTGATTCTTCCTAAAAGGTACGGGT

GTACTGCTTTTTCTCCCCCTCCCCCCCTTTTTTAAATACAGGTGTTTGTGATATATCGGT

CCATGCAGGTGCGGTGGGGCGAGTGAACTCTCGTATGCGTGTGTGCGTACTTGGAAGGGA

GAATTATGACCGCCAGTGAGGGTTCTAAATAGTGTCGCGACTGGGAACAGTCTCTGGCAT

TTCGTTTAGGGGTGGGTACATTACTTATTCTATAGCTCTTACGCTTTCGGCAGTACTCAG

GGACACATGTGCTATGGG

>Tb927.5.2810 | Trypanosoma brucei TREU927 | hypothetical protein, conserved | genomic | Tb927_05_v5.1 reverse | (geneCodeEnd+0 to geneEnd+0) | length=378

AAGTTATTCGTCACAGCCCCGGGCTGACTACAACTCGCTAGCACCGTCTTTTTTTTTTTT

TCACTCCTCGAATTGTTTCTCTGTGCTACGCCTCCATTACGTTCTACTTGTCACGTTGTG

TCGCCGTTTCGGTTGAGTGTTTTGTCGCTTTGCTACCGTTACTTTGTTGTTGTTGTTGAG

TGACGGCAGTGACGCAAGTGTAGTTCCTGTCCTTGAAGGTGAATCATACCCTGTGGTGTG

AAAGATGGCCGTGTGGCGGTTGTGCTCGAGCATATTGATTGCAGAAGTATCATGGCGTGG

GACTCGCAAAGTTGCCGGCATCATCCACCTCTAGACGTGGGGTGCGGGTGTTTTGAGCTG

TTTTCGGTGGTCATCGTC

>Tb927.2.1850 | Trypanosoma brucei TREU927 | Tetratricopeptide repeat, putative | genomic | Tb927_02_v5.1 forward | (geneCodeEnd+0 to geneEnd+0) | length=378

AAAACAACAACAACAAGAACAACAACAACAAGGAAGATAAGCGGCTTGCAAGCAGTGAAT

TCAAGAAGGGCCGTATATCACATGAAAGAGTAGAAAAACAGAGAAAAAAATATATACAAA

TGAACACACACACACACACGCACATATATATATATATATATATATATATATGACAAGGAA

TATGTAGAATTTTGGTCCCCCTTTTCACTCAAATACTCTTTTTTCTTTTTTTCTTTTTTC

TTCGCTTGAGCTAGCGTCCCCGCTACTTCTCGTTGTGGTATGTAGCATGTGTATTTGTTT

CCCTTCCAGTTTCCATCCGGTGGTTGTTTGTGCAGTCCGACAATTTCCCTGTGTGGTTAT

GTTCTTTTCTTCCAGTTG

>Tb927.11.11250 | Trypanosoma brucei TREU927 | cytosolic malate dehydrogenase (cMDH) | genomic | Tb927_11_v5.1 forward | (geneCodeEnd+0 to geneEnd+0) | length=379

GTTTATTTTGCTATTTTGCGGTGCTATATGAAACTAATGAGTATGATGTAATGGGGGGGG

GAAAAAGAGTGACGGTGACAGGATCAAACGAAAAGAAGGCATGTATGCGTATATATTGTA

ATCTGACTATCTATATTATGCAGGTACTCACCTCAGGGGGGAGAATATGGAAACCGGGAT

GATGCGGTGCCGTTGGTTGAAATGGATGTGTCCGATTCCCGTTGATTCTTTCTTTTGTAT

GGGTGATTTTGTTCAACTTTTGTTGGCGGAAGATGGCCCCCTCTTAACTAAAGGTTTCAC

TTCTCTTCGTTCGCGTCCTATTTATGTGATGGTGCTGTTCGTGACCCATTTCAATGAGGA

GGTTGAAGCAGTTTTAATG

>Tb927.11.5230 | Trypanosoma brucei TREU927 | hypothetical protein, conserved | genomic | Tb927_11_v5.1 forward | (geneCodeEnd+0 to geneEnd+0) | length=380

ATGGTGGTTTGTATTGGTTTCCCCCTTCTCTCTCTATCCTCCCCTCTTCTGTTTACTTTT

TAATTTTTATTTGATGCGTATTTGGTTGCACTTTGTGGGTCTCAGACTGCTCTCTGGTGG

TGGTGTTGGTGATTATGGTGTAATTGTTGTGTTTGATTCGGGTTGGACCCACTTGATTCC

ACGCATCGTGTATTCCTCTTTTTTTATATCCCTTTTCACATACCGGTGGTGGCAGGTGGA

GGAAGGCGCTTCGTATGCTTTTCCCTAATTGCCCTGACCAGTAACAATTAAAGAAGGAAA

CATCCCTGAAATTATGACAGCAGCGAATTGGTGTACGCCCTTTTTGTGGATTGCTGCAAA

GGTTTCCGTGACGTACTATT

>Tb927.8.2560 | Trypanosoma brucei TREU927 | spliceosomal U5 snRNP-specific protein, putative | genomic | Tb927_08_v5.1 forward | (geneCodeEnd+0 to geneEnd+0) | length=381

GTGCTGTCTTTAGTGTCGCCCATGTGTCATTAAGTTGCGCAAGTGGGAACTACAGTTGGG

GAGAGAATATTTTTCTTTCGTTTCCCTCATCACATATTTTCTTCTTCACCCACAACACCC

ACATACGTTCACGCACGTTATGACTTTAAAAAAAAAAACTAAGTATATGTAAACCGTTGT

TATTTTCTGCTGAAGAATTGTATGACACACGCCTTTCCCTACTTCTGTTGCCTCTCTCAG

ATTTTTTCACTTGCCCCAGGTAAACAGGTGGAGGGGAAGAGCATTACGTATTTCCTCCCA

AAGGTGTGGGGGTTAAGTTCAACATAACGTGGTACCGTTACACGTGAAAGAACGGAAACA

AATAAGGAGAAAAAGGAAAAG

>Tb927.7.840 | Trypanosoma brucei TREU927 | hypothetical protein, conserved | genomic | Tb927_07_v5.1 reverse | (geneCodeEnd+0 to geneEnd+0) | length=382

AGGTACCACGTAGGAAATGTGGAGAAGGTTGAATATGCTCATGAAGGAGTTTGTTAGGAA

TTTTGTTTGAGTTTTTTTTTTTAAATGTGCTTGTGTGTGTTCGTCTCTTTGCCTTTGTGT

GTGTATGTGAGTTCAGGATGGGGGGGACGATGAGGGAATGTGTGCGTTTTCGAGGAAAAG

GAACGATAAAAGGGGTATAGTGAGATGCTGTATTGATGTCAACCAGAATCTTGCTGTTAT

TGAAGCATCTGCGGTGCAGTGAAGGGTAGCGAACCCTGCGTTCCCCGTTCGGACAGCTTT

GTCTTAAGTTTTGAAGTAAATGGTGAAGATGTTTTCATTTTTCTCCAGTCTTCACAAAGT

TTTGACTAATACTCGGGGCGGC

>Tb927.4.600 | Trypanosoma brucei TREU927 | Alpha/beta hydrolase family, putative | genomic | Tb927_04_v5.1 reverse | (geneCodeEnd+0 to geneEnd+0) | length=382

GTTTTGTTTTTAAATGCAGCGTAACGGAGCAGCGGGGGCACCTAAAAAATGAAAAAAAGG

GGCCGTTTGTTATTACTATTGAAGGGAGGAAAAAGAATTGGAGCCATTCGTTGGACTTAC

TGTCTCAGGCTGTTTACATCGTTTGTCACACTTTTCCTTTTCTTTATCAGTTGCGTGTTC

TAAGGGATTGCCGTAAGGGACGTGTAAGGATTGTTTGTCCACTTTTTAAATTAATGCACT

CTTTCCCTATTGTTATTTTGTTGCGAATATTTTTTTCTTTTTGGTATTATTTCACTGTAG

CATCATCAAGGAACGTAATTGTCACAGCTGATACCGAAAGGTTATTGGAACGGAGGTGTA

GAAGGGGAGCGACACGTAAAAG

>Tb927.10.7710 | Trypanosoma brucei TREU927 | 40S ribosomal protein S8, putative | genomic | Tb927_10_v5.1 reverse | (geneCodeEnd+0 to geneEnd+0) | length=384

GTGTATTTTAATACGTCTGTGGCCTAACACGACGTGGGTAAGGAGAGGTAAACAAAATAA

AGTAGTGGTAGCAAGGGAAAGAAGGAAAGAGAGACGGCGTACTGTAAGTGTAAAAGACAA

CGAACGCAAGTGGTAAGCTGGAGTAAATTGGTTACGCGGAAGTCATCTGTAGATTAATAT

TTGTTGTTGTTACTGTTGTTTTTGTGTGATCAGTCCGTCACGGAACTCAATGGCGAATTC

CATCGCATGCGGACGTCTCTTCTCCCTCCCCCACCTACGCAATCCGTCGCGTGAGTGGTA

GCTGCTGTGGCGCAGTATTTTCGCTTAATCATATCCCACATGCGCGCATGCTGACATTTA

CATCCATGTAAGCGTCGATAACGT

>Tb927.6.4210 | Trypanosoma brucei TREU927 | aldehyde dehydrogenase, putative (ALDH) | genomic | Tb927_06_v5.1 reverse | (geneCodeEnd+0 to geneEnd+0) | length=385

AAGAGGTTGTATGTGTGTACGTTTGTTTATGGCTCCAATCTTAGCCCTTTTCTTTTTTTT

CTTTGTGCCTGCAGTAACAGTGAAATGAAAAGGAACCATTTTCTTTTGTTTAATGGGAAA

AGTGCAAGTTGGTGAATATTTTAATGATGTTGTTGGTATAACGGGGAAGGAATTAGGTAT

GAACCTTGTCTCCGCCATGTTGTTAACGTTTTTTTTTTGTCATTCATCTACCCCCTTTTT

GATTCGGCTTTATGTTCACAAGATTATTGGATGACATGAGAGAATGTGATGTGCCTGAAG

AGAAAGGCCCCCCATCTTTTTTTGTTTTTGTCTCTGGTCCATTTTCCTCAGCTTCCGGTG

CTTTTGTTTTCGTTCCTATGCGATC

>Tb927.5.4090 | Trypanosoma brucei TREU927 | hypothetical protein, conserved | genomic | Tb927_05_v5.1 reverse | (geneCodeEnd+0 to geneEnd+0) | length=386

AAAAAATCAACAGCGACACCAACACTGGAACGCTGGTGCCTCTTCATGAGTTTTTTCTTT

CGTGTCTCTGGCCTTGTTTGTATGGGACCGTCTGTTATGCTTGTGTCTCTCATTTTCTTT

CTTGCGGCATGCTCCGCCCTGGTGTGCGCTTTTGCGCTTGCTGCCGATGTGAAAAGTAGG

CCATTCCTTTTTCTCTTGAGTTTGTACATCAGTGGTAGCGGTCATTGCTTCTTAGCGTGA

TTTTGAACTGTTGCGAGAGTCGCGGTGGTTCGGCATCCTGAGAGCACTGAAGTGATAGAA

TGGGGAAGGTTTGCAGAGAGTAAGTGAAAAAAATGAAGTGTTCCGGTTTGTTCCAATCCT

CGTGCTCTATTCATTGACGTGCTTTT

>Tb927.4.2720 | Trypanosoma brucei TREU927 | ATP dependent DEAD-box helicase (RH) | genomic | Tb927_04_v5.1 forward | (geneCodeEnd+0 to geneEnd+0) | length=389

ATGGATGTCCATGAGGTTACCAGCACCATTTTATCTTCCAATTCTTCTGTGTGAGGAACG

TTCAATTTGCAAGGCTACCGGCGAATCCGAGCCTTAGGGATATTAAAAAATAAATGTACG

TTGGGCATGGAATTCTATGGTTGTGTGTAAAGATGGCTCGTCTCATTGCGGAAACTGATG

TGATAAGAATGATTCTTTTAGGTCGGGGGGGAGGCCGGGAGAAGTGGTTGATGATGATAT

CCCTCCTGTGAAAGTGCGGTCGTGTAATTGTGCGAATCAATAAACAACACTAATAGTGTT

CGCCATGATGTGTTCGACTTTTTTTTTCTTTCTTTCTTTTTTTCCTCTGCGCTATCATGA

ATTATGGTCGAACAGCTGCTGTACGGTTT

>Tb927.4.590 | Trypanosoma brucei TREU927 | PQQ-like domain/Protein of unknown function (DUF1620), putative | genomic | Tb927_04_v5.1 reverse | (geneCodeEnd+0 to geneEnd+0) | length=390

ATCCCGAGAGATATTGACTTCTCGGGATTAACATGTCGTCGCCTTGGTTGCGATGTGTGT

ATGTGTACGTTTTTCTGTTTTTGCTGAAAATTCCTGACGAGTCATTTCTGGGTGAAGGAA

AGCGAGAGAAAGTAGTGGTTGAGGTGTGGACTGTCTCTGTTAGTCAGCAGTGGCGCATGC

GAGCAGTTCGAAGGTTTTCTGGAGTGTGTACCGTGCGGGTTCGATGGAAGGGTGAAAGCG

GAGCCCTCCATTTGGGTGGAAAAGAAAGGAAGAGTCTGTTTCTGTGCGTGTGTGTATGTG

CAGCGTTAACTTAAGGAAAGGTTAAAGAGGACATTACCGTGAAGGCAGCGGGCGCAACTA

ACGGTGGATCGTCATTTTTTCTTTTTTAGT

>Tb927.4.5230 | Trypanosoma brucei TREU927 | hypothetical protein, conserved | genomic | Tb927_04_v5.1 reverse | (geneCodeEnd+0 to geneEnd+0) | length=390

GTGATAAGATGTGGTGTGCTCACATCTCTGTGCACCTATTACTTATTCATTCTTCCTTTT

GTTCGAGAGTTTGCAGTGGAAAAAACATCTGTTTATGATATATATATATATATATATATA

AGTACATATTTGTTTACGTTTCTTTTCTCCTCCATATATTGCACGCACTCGTGTTGGAGT

CACATCGATGTGTCCCATACCGTCAGTCACGTGTAATTGATTTATTTAATATATGGATTG

GTGTTTTTCAATTACTATTATTATTATTACTTTTACTACTATTACTTTTCTTTGGTTTTG

CTCTTTTTTCGTCTTCTGCCCCTCTTCACGCATCGACATCCGTTCCCAGAGACTCACTGA

GGTGTAAGTGGGCAACCGTCTCGGCACAGG

>Tb927.11.13390 | Trypanosoma brucei TREU927 | hypothetical protein, conserved | genomic | Tb927_11_v5.1 reverse | (geneCodeEnd+0 to geneEnd+0) | length=391

GGGGAAAGTCCCCCCTCCTCCCAACTACACACACGCGCGCGCATACACTCACCTTCACAA

CACCGTACGTAATGGAATTCTGATGGAGAAACTATAAAATTATGAGAAAGAGAGGGGAGA

GGCGTCGCGACAGCCACACGGGTGAGGGAGCGGATCATAGCCGCTGCAGTTCCTGCTCCC

TTCATATACGACTTGGTTGCTGTCAACAACCCAAACCGGTGAAGTGCTTGCGGAGTAAAA

TCGTATCACATTGAAGTTTCGATGTGGTAGGTGGCTTTTTACGTGTTCGTCGCTCCGTTT

GTTTGTTTGAAGGCGGCCTAACGTATTCTCTGTAGTCGTCGCTTGATGGATTCTTCCTTC

TGTGGCACTTCACATCTTATTCCACACGGAG

>Tb927.11.11320 | Trypanosoma brucei TREU927 | hypothetical protein, conserved | genomic | Tb927_11_v5.1 forward | (geneCodeEnd+0 to geneEnd+0) | length=391

ATGAGAATGAGATGAAGAAAGACAAGACGCAAAAACCGTCTCCATATAATAAACATAACA

GTACCTTCTAGAACATGTGAAGAGAGGGACACGCTACTTCAAGGGGTGCTGCGTTATTGT

TTAGCAGACCCAATACCATGACGATAGGACATCACGATAACCTTGAAACAAAACTTAACC

GACGACCTGTATAACAGCACACAATTAAAATGCAAAAAATAATCGGGACGACTGTCACTG

CTGTACAGTGCGAAACATCACTTAAACAACACCTTTTGCAACCCATAAAGTAGGAACAGA

GCAGTAAATGCGAAAGTACCATTAAAACATTACAAAAAACACTTTGGCAAGCAGATACTC

GTTTACCTCGCAGGTTTAACGGGGAAATTTC

>Tb927.3.2030 | Trypanosoma brucei TREU927 | acylphosphatase, putative | genomic | Tb927_03_v5.1 reverse | (geneCodeEnd+0 to geneEnd+0) | length=392

AAGTTATGGTTCACCACGACATGAGTTGCGTGTGCGCACGTACTTTTCTGTGTTATATTG

TGTATGTATTCATGAAGTGCGTTGTTGGTTGTTCTTGCTTTTTGTAGCGTGTAAAGAGCA

TGTGTGTGCGCACCGGTAGTTGTGACGAGGTTGATGTTGGTTTTCCCGCGGGATGGGATT

AATGTGGAAGTTGCTTGCACTGTTCCTTCTCTTTTTCCTTTTCCTTTTTGTTTTACGTTA

TTACTTTCTATACGTTTTGTGCTGCGTATGGAGGAGGGGAATTCTCCCCAGCTGCACATT

TGAGGTTAATGAGTTTCGCGTATGTGACAACCTGAAAAAGAGGAGGCAAGAAAGCCTCTC

CGAAGAGATGATTTTATCTATTGGCACGCAGG

>Tb927.3.4130 | Trypanosoma brucei TREU927 | hypothetical protein, conserved | genomic | Tb927_03_v5.1 reverse | (geneCodeEnd+0 to geneEnd+0) | length=393

AATCGCCTGGGCTTGATATGTTCGTGGGGACTAAGACACGGATGGAGTTGAGTGGCAGCA

ACGGTGGAGGAGAAGGGAAGAATGTGACTAGGGGTTACAACTGGGCGGAAGTGCGCATGA

CAAAAGGAGTTATGCATTTGGTTACGTCTGGACATGCAGGTGTTTCCCTTTCTACTTCCG

TGCATATGCATGCAGATACATAAGTATATGTACGTGCCTCTCCTTTTTTGTGCGGAGCGC

TGTCGCCTCCTTTGTTCTTGTTTGGGCTTCGACGTCAGGAAGAACTCTTTCGCTTTCCAG

CTGGGGCTGTTCTGGCTGTTGCTTGCCCCCGGAACACGTGGACGTAATGTGAGTGGCGCC

ACGTTGAGCTGTGTTCCTATTTTGCGGGGAGTC

>Tb927.7.2360 | Trypanosoma brucei TREU927 | N-acetyltransferase, putative | genomic | Tb927_07_v5.1 reverse | (geneCodeEnd+0 to geneEnd+0) | length=396

AGATTCTTTTAACATCTTTTTACGGAGAGGCGTGCAATCGCTCACGAGTTTACCTGGCAG

GCGCAGCCGTAGCGATAAGCTGATCCAGTTTAGGGATAGACCGTGAGACGTATGTACTGA

AGCGGAGGTGGAGCATGTCTGAGATTGAAAAGAGTATGAGTAATAGGGTGGAGTGTGGTT

TACTGAAAGAAAGGAAAAGGGAGGGGGTGGTGAGCACAATCAACCTGTTGACTTCTTTAT

TTTCCACGAGAAATCGATCGTCTCACTAGCGGTGGTGGTGTGTTGCGTGTAATGCCTTAC

ATGTAGATGGAGAATATCCTCGTGCGAACATGAGGGATTTCATGGTGGATGCTGCCATTC

GAGTTGAGGGAGGAGCCGAACAAAAAGTGAAACAAT

>Tb927.6.4480 | Trypanosoma brucei TREU927 | valyl-tRNA synthetase, putative (ValRS) | genomic | Tb927_06_v5.1 reverse | (geneCodeEnd+0 to geneEnd+0) | length=396

AGACGCCGGAGATGCAAACGCCTCAGTTGAGAACATGATATTTATTTAGGGAAACGAGGG

GAATGGATGGGGTATAATGAAGTCGGGTCTTCTTCAGGCACTGAGGGAGTCTGGTAAGTT

TAGTGTTTGACAGCTGGTGACATTAATAACCGGAGCGACTAGGATTACTCAGGGGACCAG

CATAACATATTTTCTCTTCTTCCTATTCATTTCTACACTGTTGTTGTATTACTGGTGAAA

ATTTCCCCTTTTAAAATGTTGACAGCTGAAAATCTAGCGTTTCGGGCAGGCCTTTCTTCC

CTGTATTCCTGATCCAATTGACTCTGATAATGACAACACCTGTTTCCTGCGATGAGCGCC

AATAAGAGTTTTACCCGATGAAAATTTCCTGAACTT

>Tb927.10.5070 | Trypanosoma brucei TREU927 | ribonuclease H, putative | genomic | Tb927_10_v5.1 forward | (geneCodeEnd+0 to geneEnd+0) | length=396

GACACCCCTGACGCATCCATCATTTCTTGGAAAGAAAGTAAAAGAAAACGAGAAGAAAAC

AACGTGCAGTGTATAAAGTTCTTGAAAACAGCAGCTTCTGCTGTCTTCACAGGACGGAAA

CACAACTGTCAAACACCTATGCAGGATGTCCCTCTGGTGTGTTTCATAGAAACAATTTTG

CGGCAGGAAATGTTCGATGAGGTGGAGGGACGAATATTTGTCCTTTTTGGTAATAACAAG

AGTTCTACACGCACAGGCATACGTAATACATCGGTGTGAAAAACAATCATTTGTTCAGTT

GGACGCTGTTTTGCGTACTAACTGGGTTGTTTTATCGTGTTTTTCTCCTATCCCAACATT

TTGGTGCGGAGACCGTCTTCACGTTTGATCAGACTC

>Tb927.7.6610 | Trypanosoma brucei TREU927 | hypothetical protein, conserved | genomic | Tb927_07_v5.1 reverse | (geneCodeEnd+0 to geneEnd+0) | length=399

AATGTTGGATAAGTCTTGAGTGTAGGGTGACAGGTGTGACTCCCCTGCATCGGTGCTTGT

CACCCCGTTTTCTTCTTCATGCGGCACTTCCGGTGGAGTGTAGCGACGAAAATATTTTTG

TTTTGATTATGTCATTTCTGTCTGGGGCTGCACTTTTCTTTTCTTTTCTCTTTTTTACCT

TTTTTGTCTGTTAATGCACTAAACGGCGCCGTTTGAGGAGTATCCCTATCTAGACAGCTG

CAGATCTGTGGTGATCCACCGTGCAGAGGCACGGGGATGAAAATCTAGTGCGCAGTGGCA

GGGGAGTCAGGGTGCGTGGCGCATGAAGTGCCCAAACTGCATTGTATCTGGTGGCCACCC

GTATATTTTCCGTTTTTCTTTTTTCCTTATCAATTATTC

>Tb927.3.3240 | Trypanosoma brucei TREU927 | hypothetical protein, conserved | genomic | Tb927_03_v5.1 reverse | (geneCodeEnd+0 to geneEnd+0) | length=401

ATGATGAAACAACATTGTCTGTACAGGAGAAGTGTTGCCTGTACGACCGTGCATTCGTGC

AGACAGTATTATGGAACAGATTCAGGGTAGTGAAGAAGAATAATTGCGGTGTTTCCTTTT

GAATATGACACATCGATTCCTCTCTCTAGACGAGGTGAAGATAATAGGACGTTGCATTGG

TGCTTCTCAAAGTATGGTAACTACCATATGATGGTGGCAGGCAGTGCTTGCTTTGTTGTT

AGTGTTTCGTGAATGCAGGAAACTTGGAGGGAAAGTGGTACCCGCTTATAACAGGGACGC

TATGCAACTTTCCCTCCTTTAAACGTTTTCTCTTTTTTTTCTTTTATCGCTCCTGCTAGC

GACATTGTCCCTCTACTTGTTGCTCTGAAGCACACCATAAT

>Tb927.9.11480 | Trypanosoma brucei TREU927 | hypothetical protein, conserved | genomic | Tb927_09_v5.1 forward | (geneCodeEnd+0 to geneEnd+0) | length=401

ATCGATTGCTGCGGTAGGAACAACCGCATAATTAGTGTGCTGAATATGGTGAGATCCCTG

TGAGAATAATGGTAAGGGCTCGTGGCGCCACTGATGTTTGGGTTGCTGCCGTTAGTGTTG

GATATCAACAACAGGATGTGGGACGAAGGAGTTAGTGAAACGAACAAGTCAAAGAAACAA

CGACTTCCCCCCCTCGGACATTGCGCATCTTTCTTTTCTTTGCTTTACTCAAACTCGAAC

AGACGAAATGAAAGCGATAACACGAAGGAGAAAGGATGGAACTAAGAGGAGGTAGGGTAA

CGTTTAGGCTAAAAGAAACAACGTGGCGAAGGGTTATGTGCTTCGTGAGGTATTGCTATA

ACGGATCCATTTATGGTCTTCTTTCTGTTCTGATCAGTGTT

>Tb927.11.7170 | Trypanosoma brucei TREU927 | seryl-tRNA synthetase | genomic | Tb927_11_v5.1 reverse | (geneCodeEnd+0 to geneEnd+0) | length=401

ACCGTAGCAATTACAAGCGGTTTACGTGTTTATACGAATAATCTATTTCTGCGGGTGTGA

GCGTGGGTCAGTAAGTGAAAATTAAATAATAGGGGTCTTCGGGGGCAGCGGTAGGGAAAC

GGGGGGGGGTGCCACATTTTGTTTCCTTTAGTTCCGTGACTAGTAGCATTGGGACCATTG

TATGCTGAGGGCCAGCCTGCGTCGAATGGTAGTTATGCACATTCGTGTAAGGGCACGGTT

GGTTGCCGTGTCAGGAGCCGTGACCATTTTGGCTACTGAGGGAGCTTACGACTGTTAGGC

CATCCTTACCTCACCTGTATTTGTTTTACAGCTCTACTAACTTTGAAATATATTTGCTCA

TGTTGTTGTTGTTTATCAATTCCTTCCGCAGGCACATGCAC

>Tb927.10.14180 | Trypanosoma brucei TREU927 | protein transport protein SEC13, putative | genomic | Tb927_10_v5.1 reverse | (geneCodeEnd+0 to geneEnd+0) | length=402

ACGACAACTTGTTTGAACAAGATATGGTGGAGAGATATTTACAACGGGAGGTCGGTTGGA

TGGGTGGGAGGAGAAGGGGAAATGAGGACTATGGGAAGAAGTGCGGAGGTAAGGCGGTTA

CCGCGTGGTGCATGGCAACAGCTGGTAGAGGGTTTTGATGGTCCTCCTGAGCACAGTTCA

CCCTCCTAGTTTTTTTGTCGGCATCATCTTGACCCTGTCAGTATAACCTTTTGCATTGCA

CCGCTCTCCAAGGAGGGATCATATACAAGCATCATAGTGGAAAGGGGGAAATCAGATCAG

AATAATCGAGAAAAAGAAGGCTGAAACTCCACTTGTTGTTTTCTTTCTTCCTTCCGTTTC

ACTCCTCTACGCACACGCATGCGCATGTGGGAATTCCTTGTT

>Tb927.1.3110 | Trypanosoma brucei TREU927 | soluble N-ethylmaleimide sensitive factor (NSF) attachment protein, putative | genomic | Tb927_01_v5.1 forward | (geneCodeEnd+0 to geneEnd+0) | length=403

GGGTGGAGTTCCACCCCTTGAAGTTGTGAGGAAGTATGGAAGGGGTCGATAGCAGGGGGA

CGGGCACCTCTTTTGCCATAGCTTTCTCAACAACTTCACTGGCACACCATTCTTCGACAG

AGTATTATTTTACAGTTGGCATTGCTACTTTTCTCTCCGTTTTTTTTTCCTGGCGGTAAT

CACGCCTTTTCGCTCTGCCCCGCCCCCCCCCCCCCTCTCCTTTGAAAACTGCTTGTAACG

CCGCCGTCCCGTGTCTTCAGCAGGAAGTACGAGTTAGCTCAAGCCTCAGGAAGAGCTAGT

TTTTTACTTTATACACCCAACTTACCACAACCAGGGAGAAAAAAACTAAAAGGACAAATG

TGGTAGATCGTCTTATTCCAGTGATGCGTGAAATAACCTTTCG

>Tb927.4.1780 | Trypanosoma brucei TREU927 | hypothetical protein, conserved | genomic | Tb927_04_v5.1 reverse | (geneCodeEnd+0 to geneEnd+0) | length=403

AGCTGGGAGAAACGATGAGCGGTGTGACGCCGTGTGGTAGCGGGAGCGGCATGTTAGGGT

CGACAAACTGGTGCATAGAGAACATCATATATCCATATGTGTGTATTCATGTCCATATTT

GTTTATTTTAATACAGTTGTGGAAAGCTCTGCTTCTGTATGGCGTGTGCGTGTTTTAATA

TTTTATGGAGAAATGATCCAACAGGGGTCCCACTTCGACCGGTGTCTGCACTTGATTCCC

ACTTTTTCTCCCCCTCCCCTCTTTATTTTCCTCTCCTTCTTCCCTTTTGGCACAGTATTA

TTGCTACCTTTCTGTATTCTCTTAACAGATGCTTATTTGCGTGCCTGATGCATTGCCGGT

CAGTTATTGAGACAGCTTCTGAGAGGTTTCCTCGCATTGGTAT

>Tb927.11.10040 | Trypanosoma brucei TREU927 | hypothetical protein, conserved | genomic | Tb927_11_v5.1 forward | (geneCodeEnd+0 to geneEnd+0) | length=404

ACAACACCTACGCACTTGACTTGGGAGCGGGCGGGCGTTGCCAAGTGAACGTGCCGTTGA

GTCGTACTGTGCTTTGGTTGTTTTGGAGGTAGCAGACACTGGTGAGATTCCCCTTTTATC

ATTACTACTATTATTATTATCATTATAATAATACATATATTATTGGTATTGACCTTTCTG

ACTCTTTGTTAAATACTGGTTCATTCGGGTTGATGTTCACTTGTTGGTTATTGATTGTGT

TTAGTGGCGTTTGAATGCTTTGACCCCCACGGAGGGGTAGTTGTAGTGTTGTTGTGCGTC

TCATGCACGAAGCAAGTTTCTCCATTAACAGAATTTGAAGAGTTTAAATGGTGTAAGTGG

ACACTGCAAGCACCGAATTGTTCTCTGCGAGTAGTGCTATTGTT

>Tb927.10.15220 | Trypanosoma brucei TREU927 | hypothetical protein, conserved | genomic | Tb927_10_v5.1 forward | (geneCodeEnd+0 to geneEnd+0) | length=406

ACGTCTTGCCGCATCCGCTTGAGCTACAGGTGGACGTGTGTGCCTTTGAGGAGCGTGGAA

AAAATTAAACGGGTGGCGGGGTAGAAGGTTCCTTCCGGCGCGCCGTCACTTTCGTGCAGT

TTCACGCTCAGTTTCAGTGGACGTTTCGTGCAGACATATGACTCGAACATTACAGAGAGG

GAAAAATGTAACACAGATTTATTAACTTCTAAATGCCTGCCGCAGGCAATGAATAGGAGT

ATGGGTAAATATCTTCGGACGCCTAGGCGCCGTGCCGTACGTTTTTGTATGGAGCGGTGG

AAGCGGCTGTTATTTATTGTTTGTCTTACATTATTCGTTGATTGCAACGTATTTACCGCA

CGTGTGTTTTGTCTTTGCTTCCAAAATGGGATTCACTAGGGAAGTG

>Tb927.1.3000 | Trypanosoma brucei TREU927 | amidohydrolase, putative | genomic | Tb927_01_v5.1 forward | (geneCodeEnd+0 to geneEnd+0) | length=406

ACGGTGGAAAGGGAATTTTTTGATCCGTTGCTTCCTGCGCTTGAGGTGCCATGCCTTGTC

ATAGCTCATTCGCGGGTCGCCGGTCGGTTGCGTCTCCGTTAACATGCTTCTGTTTTTCTT

TCTCTCTCTTATCACTTGGTGTATGTGCGGGCGTCGCCACTTTTCCCAACAACCTTAGCA

GCGTTTACAGTGATACTGGGGAACGGACGGGTAGGTGAAACAGTGGGTGAGCAACACACT

TTGTTTCGGAGCTACGCGGGTAAGCAATGAACAGGCGGGATACTCCTCGTTAAGTTCGGC

CCAACAGCTCCACGTCTACCACTCCCTCTTTTCTCGGAGCACGTCCCCGCGCGTGTACAT

GTTTACCCACGCCTCACTTGCGTACGCACGTCGTATTATCAGTGAT

>Tb927.9.9100 | Trypanosoma brucei TREU927 | hypothetical protein, conserved | genomic | Tb927_09_v5.1 reverse | (geneCodeEnd+0 to geneEnd+0) | length=407

AAGAGGGGGAAAAAAAGAAAAGAAAAGCGGAAATGAAGATGAAAAAAAAAGGGATATGGT

GGGATTAGGGAGTGAGACGGGGAGCCGGGTCTGCACGCCGTATCCATAAGAAAGGAACGG

GAACTCTATTCTGTGGGTAAGTGAGGAAAATGTACCCTCTTTCTTTCTGCTTTTTTTTGT

TGCAAGAAAAAAAAATCATGTAGGTGCGTGTTGCAGTAAGGGAATAGAGAAAAAGAGGGT

ACTTTTTTTTTTTGTGAAGTGACAGTCGGGGAACCCTTTTTGTTTATTTTCTTTCCTTTC

CTGTTTTCGACCACATCAATTGGCCTTTCTACCATTATTGTTACTGCAGTGCTGCAAAAC

AGCATTCAGTCCTGATGTTTAGCACTTCTTTAGATTGCGCCCTCTGT

>Tb927.9.9810 | Trypanosoma brucei TREU927 | hypothetical protein, conserved | genomic | Tb927_09_v5.1 reverse | (geneCodeEnd+0 to geneEnd+0) | length=407

GATTTTAGATGTTGTGGCGTGTTGATGGTTACAGGAGGAGGAGAGAACAAACATGACATG

GAATGAAGCAGCGCAAAGAGAATGAAGAGTTTGTCTATAGATGTGTGGGAGCGTGTAGAA

AGGGACGGAGGTGCTGACAGCCTAGTGCGCGTTGCTGCTCCCGGTGTAGCAGAGCGGTCA

GGAAAGGGTTGTGACAAAGCGTTAGTGCTGTCCGCACCAATAATCTGACTGTAAACAATT

AAAGGGGGTGGTATTTCATGTTTAGAGACAATGTAACGCGTTTTATCAGGTGTTTCCGTG

GTGTTCAGCTTTTCGACTTTTCTGTAGACTACAGCAGCAGCAAAAGGGGGTAAAAGTTGT

TTTTACAGGGTTCTAGTGGAGACGTTTGCAAATGATTATCCCTGCCC

>Tb927.11.5500 | Trypanosoma brucei TREU927 | Mitochondrial SSU ribosomal protein, putative (KRIPP1) | genomic | Tb927_11_v5.1 forward | (geneCodeEnd+0 to geneEnd+0) | length=408

ACGTCTTAAACAGTGCTAGTAGCGGTGAAGGTATTGTTGTGTAGAATTGGGAAGCAGGGG

GACGATAGCGGTGTCACACCGCTTGCCGTGTTGGTTGTGTGTATGAATCTGGACATAAGT

GAACTCCTAACGCAGAAACACCTGTTATTTGTTGACATTACCGTGAGTGTGGCTTTGGGT

TTGTCAGCGCACTGCTGAAAAAGGGGTGTCGACAGCTTGCGAGTATGTGTAATCAGCCTT

AACCGACTGTGATGGCGGGACCCCTTGCGGTGTGCGAAGAGAGTTGATATTAGCAGAAAC

GAAGCACAGGTTATGGTGCGACTGTGTGGTTGGTTACTTACTCTTGGGCTCGCCTCCTTC

GTTGGAGGAAGCGTGACGCTATCGACTCTGTTTTTAACGTTTTTTTTT

>Tb927.8.2300 | Trypanosoma brucei TREU927 | hypothetical protein, conserved | genomic | Tb927_08_v5.1 forward | (geneCodeEnd+0 to geneEnd+0) | length=408

GTTGCTCTGTGAGCTGCGCAAGCACCTAATGGTGAAAAGATGTTGCATCGTTGCCAGCAA

GAGTTTGGCGCACGAACCAACAGAATCTGGAAAAGGAGTAATACGTGCCACATGAGTTTC

GTGGTGGCGCGTCACCCCTTTTCCGTTGCCATAAGATCGTATCGTGTTGCCTGGATTGCT

CCCCCTCATATGAATTTGAGTGTTTCATTACACAGTGGTATCATCTGTTATTGCTATCGT

CGTAGTTTCTCACAACTTTAGATGTGCTCGTTTGAGGCGACAACCAAGTGCCTTGTGGGA

GGTTCTTCCATTTTTCCACCCCCCCACACGGAGTTGCGGCACAAGCTCATTGAAAGGGGT

GAAAGAAAAAAGGAAAGGAGAGGTACAGTGGAGGATAAAATTGTGTTT

>Tb927.9.6390 | Trypanosoma brucei TREU927 | hypothetical protein, conserved | genomic | Tb927_09_v5.1 forward | (geneCodeEnd+0 to geneEnd+0) | length=408

AGGAACATGTACAGGCGTCAAAGGGTTATGCATATAATAGTTATGTTAGGGGGCTGTGTA

CGATCAACAAACTTATGCTCATCAGGAGAGGATAGTGAAATCGTTTGTGGAAACCGACTG

CAACCTACGGAACCCACTGGCGGACGCAGAAGGGAGTTGTATGTGCGATGCTGAGCCGCG

CGTTTCTCATCTTTCCACATATTTTTAAAGAGAATATATACGATATGATGACGTTGCTTT

TCTCGTCGGTCCCATAGCTTTGTGATATCAAACCATGTTTCATTTCCCAGAGATGCTGAT

GGTGAAGTGGCCGCGAACAGCAAAGAAGCGTGAGTGGCGTGTGCTGATCGGCGGGTAATG

GCCCAGACATGGTCTGCCTCCAGCCATGCACTGTTTATTGGATCATGC

>Tb927.1.2760 | Trypanosoma brucei TREU927 | hypothetical protein | genomic | Tb927_01_v5.1 forward | (geneCodeEnd+0 to geneEnd+0) | length=409

AGAGGAAGGAATCGGGAGGGAAAAAAGAGGGGGAAAGTGAAGCGGTGACGTAAGGGGAGA

GATGACCCTGCACGTGCTTTGGCTGGTCGACTAATTGGGTTGGAGGGAAGAGGAATTAGG

AAGAAAAGGGTAAGGAAAGAGGATATACATGCACGAACCGTCTGGGAAGTGTGGCGCCTA

ATTATTTATTTTTAACCCATCCCCATTTCTTGTTGTCTTCTCAGTGGTTCTTAATTTTTA

TAACTCCCCTACGCACTGTGAAAGCACTCACATCCTTCTCCCCTCCCCCCTCATATCAGT

TGCCCTTTTTGTGCTTTTGGCATCACATGTCATGGTGCATATGTTCATCATATCCTCCGA

GTCCGCCTGCGCGGGTCCACTTAACGCATCTGTAACATAAAAAGCATAT

>Tb927.9.14430 | Trypanosoma brucei TREU927 | casein kinase II, putative | genomic | Tb927_09_v5.1 reverse | (geneCodeEnd+0 to geneEnd+0) | length=409

GGATTTTGCTGTGGATGTGGGAAGGAAATTGCCCAAGCTGCAATAAAGGGGGGGGGGCGA

AAGAGAACAGAGTGTAAGCACCTAAGCTATGAGAGAGCGGCGAAATGTTAAGTCGGTGGA

GATATAGCATTCGACGTACAACCGCGTAGGTATTGAGCAGGTGTGTCGAATTCTTTTTTT

TCCCCTTGTCCGTTGAGAAACCAGATAACGGAGTTGTGGGCTATATTTTTCTTTATTCAC

CTTTCTCTGTGTATATGGAGGCGAAGGAATTGAAGGAATGCACTTGCGGTGAAGTGTAAT

AAGAAATACGGAAGGGACCGCGCGGCAGTTGCGGTTGCGGTGCGACAAACATCGAACTTG

TTGTGTGTTCAGGTGTTGAGATATTTCACTTTCTCACTGTTTTCCAACG

>Tb927.10.7720 | Trypanosoma brucei TREU927 | hypothetical protein, conserved | genomic | Tb927_10_v5.1 reverse | (geneCodeEnd+0 to geneEnd+0) | length=410

AGTAATTGCCACGTCGGCTCTGTGTATGTTAAACGCAAGGCGGAAGCCATGCAAAGGGAC

GAAAACAGAACAGAAGGAGTGGAAAAACAAAACTTTAGTTCAAAACAGTGAAAGTTTTCG

AGCAAAAGGATCCCCGTGGAAACGTGGATTCTTTTTTTCGGAGAGGGAGAAGTGGAGGGA

CGAGGAGATGGGTGTGTGACAGTGGTAAGGGAGTACTTTCTTTCAGTGCGAAAGTGTGGC

AATCGTTTTCATGATAACTCGCCCCCTTCCCCCCCCCAAAAAAAGCATTTTCTCCTGCCA

TTTGTTTGCCCTCCTCCTTTTTCCCCCTCTCCTTTTTCTTCGTTTCTTTTACCTTGGCAT

TCTTTGTGTTGACAAACGAATATATCGTCGCCGTTGCGCAGATCTCTTTT

>Tb927.10.4570 | Trypanosoma brucei TREU927 | elongation factor 2 | genomic | Tb927_10_v5.1 reverse | (geneCodeEnd+0 to geneEnd+0) | length=411

GCATACCAGGACAATGTGCATAGGTGTTACTGTTTGGGCGGGAGGGCGATTTAAGCGCAC

ACCAACGTAAGCGGGGTAATCAATAGCACTGTTTTCTAACGTAAAAAAACCGAATGGAGA

AAGTGGCCAAAAATTAGGGCATATATGAAAAGTAAAGGTAATGGAAGTATAAAGGTGCAA

TCGAAGGGAGTAAGGGGTGCTAAGGAGAGTGCTTCCCTCGACGTACGGGCAAAGTTGCGG

GAAATTGGAAGTCCTGCACCAATACAAATAAAGTAAGTAAATATATTCAGATAATCTAAA

ATACAGAATGGACAGAGAGATAGATCAAGTCAAAGACAGAGGAAAGAAATCATTACTCCC

GAACATAAAGGACGTATATGTTGTTCCCATGGTCAGATGCATCACTGCAAC

>Tb927.9.12410 | Trypanosoma brucei TREU927 | unspecified product | genomic | Tb927_09_v5.1 forward | (geneCodeEnd+0 to geneEnd+0) | length=412

AACCCTCAATTCTTCTCAGTCATGGTGAATATGAAGTTGAGACTGAATGGTAAGAGGAGG

CATCCTTATTATTATTATTATTACTATTATTATTGTTTGTCTTATTTTATCGTCAAAGTT

GATACATATATCCGACAAGTCGCGTGTTTGTATATATATATATATGTGACAGTGTATTGA

CATCGCAATACGTGCTTTGATGAGTGTGTGCATCACATTCTCCTTTATCCCCTTTTCGTG

TAGCGAAAAACCATATATTCAGGCAGGCGTGTGGGGCGCAGAGAAAAAAGAAGTAAACTA

AAGTGACAGCGGTGAGCGAGTTGAAATGAGTTTGTAGTCCCCACTTTTTCGACCAGAGGG

AGAAAGAGAAACAGTTTTCGCAGTTTGTGATTTATTTTTGAGTTGCTTGTTG

>Tb927.11.5510 | Trypanosoma brucei TREU927 | dynein light chain p28, axonemal, putative | genomic | Tb927_11_v5.1 forward | (geneCodeEnd+0 to geneEnd+0) | length=414

AGAATAGAACAAACTGCTCAAGAAGTGAAGCATCATATGGTTGTTGGGTTGGTATGTCTT

CTGTTTGTGGCAGATATTGGTGTATCTGGCTTTTCCACCGTTGAGTTTGATATTGTAATG

TTACTTAACCCTTTCTCATGGTTTCTCCCTGCATCGTCGATCATCTGAAGGTTGCGTCTG

TGACGATGCACTTTGGTTGTCAACTACACTGTTCCTTTTCCACATTCTTTGGGAGATGAG

AAACCAGAAGCGACAATTGCGGAAGGGGAGTTAAGGAGAATGTTGGTGGAAACATTGTGA

AAAAAGCACTGTGTGTGAGAGAGAGGGCAAATGAATAGAATACTTGTGACTTTTTGTGTA

TGTTTGTGTATATGCGAAGGGAAGCAAGACAGAGAAAAGGAGCGTGCTCGATGT

>Tb927.10.1440 | Trypanosoma brucei TREU927 | hypothetical protein, conserved | genomic | Tb927_10_v5.1 reverse | (geneCodeEnd+0 to geneEnd+0) | length=414

AATTTCCGCGGGTGTTTGTCGGCTTTTCGATATAACTGTCAATGCGTTAGGGAGGAGATG

GATGAACGTGGTGTCATAGGATGGTGATGATTACTCCAGCAGATTCTGCGTATGCTATTA

TTTTTCGTTGCGATGATCTTGTAGCATATATATATATATATATGTGTGTGTGTGTGTAGG

TATAGGTGTAGGTGCGAACACGGATGCCGTGGGGCCCACTTAGCGTTCAAGTTCCTTCTC

TATCCGTTCACAGTGCGCTGTTGTTCTAGGAGTGGCCGGGGAAAAGTCTGTTACATAACG

TTGCACTGGTTATGTGTCAGCGAGTTATTTTGCCTAACACATTTTTCTCCATCCACCTTC

CACCTTTCTGATACTCACGTCTGTGTGTGCTTGCGGTGCTTTACCTTCCCCGTC

>Tb927.9.3760 | Trypanosoma brucei TREU927 | poly(A) export protein, putative (TbGLE2) | genomic | Tb927_09_v5.1 forward | (geneCodeEnd+0 to geneEnd+0) | length=415

GTTATTCTATTTTTCGAAGAATATGAAGCACCTATGGTTGTGAGATTGTTTAGTGGTAAT

AGTTATAATTTTCTTTTGTGTTCTTGTCACCGGTCAGTTCCGTTAGTGAGGAGTAGTTGC

GCTCGTACAGACCCGTTGGTAATTGTTGTGAATTTGGTGCTCAGGGCTCCATGCGGCACT

GGGGGCGAGGGCTCGTCATTCAGAGGATGTCGTATACTAAAGGGCAGGCAACGCTGTGCG

AGATCACAATCGTTTTAGCGATTCTTGTTGGAAGTATTTATGGAGTCTTATGACACTGGG

TATGGCTGGTATCATTCTGTGTTACCCTGTAGCCTACTGGTCGTTCATCATGGCCTACCT

TGTTGCCGTAGGCAGTACTAAATTTAAAAGAATTCCTTCGGCTGATAATATACTG

>Tb927.10.11000 | Trypanosoma brucei TREU927 | hypothetical protein, conserved | genomic | Tb927_10_v5.1 reverse | (geneCodeEnd+0 to geneEnd+0) | length=416

ATCTGTGGCACGTGGAACGGTGTTGCGGCACCGATCGCGGGACAACTTGCGATCTCGCTT

ACGTCCGTTACATGTTTATGTTCTCCCCTGTTCACTCAACTGAATTTTTTTTTCTTCGAG

GAAGTGCTTCTGCCGTCTGAGGTAAAGGAGGAAGCAAGTGGTCTCTCCTTAACTGTGCTC

AGGACGGTGAGAAAGCGAGCAGAAGAGTGTGGCTGTGGAGGTCTACGCTGCAGTTGGGGT

GGAGGGGTATAGCGGCGAGGAGAGGAATATTACACCACGAAATATTTCTTTGAGTATATC

AAGCAGTGTAGTGCAACTGCGTACGTGTGCACGTTGGATCGTTATCGGTTGAGCAACGAC

ACCCAACTGCCTAATTCGTTCACATGTTATTGCGCTGTTCGCGCAGGCCCCAGCTG

>Tb927.3.1840 | Trypanosoma brucei TREU927 | 3-oxo-5-alpha-steroid 4-dehydrogenase, putative | genomic | Tb927_03_v5.1 reverse | (geneCodeEnd+0 to geneEnd+0) | length=417

GATATATAAATATATATATATATATATATATATTGTGTTTGTGTGGACGCTACTCCGCAA

AAAGAAGAAAAGAAAATGCATTTTTATTGCAGGAAGTAAGAAAGGAATGAATGAATGATG

GAGGGGACGGGGGAGATGATACCCGTTACCGTTACCTTGAAGAAAAGTTGAATTTTCGTT

GGGGGCCGAAATTTGGATTAGTAGAGGTTTAAAATGGGGAGTGAACACCTCAGTACCGTG

AACAAGACAAAAAAAGAAATCACAACACGAGGGGTTATTTACAAATAAATATTATAATTC

TATGCAGGGTGAAAAGCAAAAACACACACACACACACACACACGTACACGAAAACAACAA

CGAGAAGAAAATGAGTGGTGTGATGTGAGGGAGTGAGATGGATAAAAAGAGGAAAAC

>Tb927.7.4440 | Trypanosoma brucei TREU927 | NAD dependent epimerase/dehydratase family, putative | genomic | Tb927_07_v5.1 reverse | (geneCodeEnd+0 to geneEnd+0) | length=417

ACAATGAGGGCTAATATGCTCAATGTGGGTTTTGCTGCTGACGAGCGTTGTGGATATGTA

CCATCATTGCTCACCATCACATCGGGTGCGAATGACTACACATTACAAATGGTTTCAACT

GAGAAACTTTTATAATCTCTGCAGTGGGTGACGCTTCCCGCCATCGGGGTTCCGCTGTCC

ATTTCCTCCTATCTATTATCTTTGTCTGCCAGCATGGTTTTCAGGAATTTTAGGAAGGAA

CGGTGCACAGGGTTGCCTTCGATAGCGTTTTTGGAACCTCATTTTTTTTTTTCTGGAGCC

GCCACGGACGGACTGTACCTGTAAAAGTAATAAGAAAACCGTTTACAGGACCTCACATCC

ACAGGTGGGGAAAGTTGTGAATGGTGTGTGGTAGGGTTGGCCTGAACGACCCAGCAG

>Tb927.7.1170 | Trypanosoma brucei TREU927 | RNA-binding protein, putative (Y14) | genomic | Tb927_07_v5.1 forward | (geneCodeEnd+0 to geneEnd+0) | length=418

GGCTTTCCCCATGCGCTTATACCACAATGGCTTCAGCGTATTTCTGTCTGTTGGTATTTG

TGGGATGTCGCGCCATCTCAGATTTCCCGTTATCTTTTCCAGCTGGACGATACCCAGTGT

GTGCTTCTTTACATCTTTTCTTCTTTCTTCTTTTAGTGGGCCATTCTTGTAACTGAAGGC

GGTGATGGTGGTGTGATAGTTTGCGAAGCGGCGTGTGCACGGAACAAGAACAAGCGAAGA

AGAGGAAACAACATCACGTGTTGGAAGTGCCACTGGGGCAAAGGGGCGTAGAGTTGGGGG

AATATTCAGGGAATCGGCCTGGAATAGCCTTCGTACTTCTCTTTGATTTGGTTGGGTGCC

GTGTTTTAAATTTATCTTTGGTCACCACTATTAGTGATCGAGGGTTGCGATCGGTTGG

>Tb927.5.1670 | Trypanosoma brucei TREU927 | hypothetical protein, conserved | genomic | Tb927_05_v5.1 forward | (geneCodeEnd+0 to geneEnd+0) | length=418

AGAGGTAAACTCCCTTTTGATTCCTTTCCTTTATACGTGCTTCGCCGTTACTGAAACCTA

TCGACGGCGGTGTGTACACGCCTGTGTGTGTGTGTGTGTGTGTGTGTCTGCTAAGATCCT

TGGCACTGCTAGTGACGACATTTGTTGGTTTTATCAGTGGAAATTTGTCTTCTTGCTATC

GTTACCATCGCTGCTCTTCTTTCACCTGTCGCCACTGTTTTTTTCCCCCTCTTGCCGGCA

CCCGTTTTCCAACTCGAATGCAAAAGGGTGTGGTGGTGGTGGTGCAGCATAGGTGATTGG

GCGCCAGCCGGGGCGTAAGGGCGTTACAGGCAATTTTTTTGTGCCTTTTGTTTCCCCTTT

CTTTTCCTGACATATGTGGTGGTTCACGCGTTACGATGCCCAGTAGAAAGGAAAATGG

>Tb927.3.2840 | Trypanosoma brucei TREU927 | inorganic pyrophosphatase, putative | genomic | Tb927_03_v5.1 reverse | (geneCodeEnd+0 to geneEnd+0) | length=419

AAGGTGTGACCGATTGCGTGTACTCGTAATGTGTTGTGTTATTATTTGTCGTCTTTAGGT

TGTATTGCATTTGATGTTCCCTAATCGTTTGAAGGTACGAAGCGACAAAAAAAAACGAAT

AATAATACGGTATCATATGTGGACTTGCAGCGCACTGACACTGTCACATTGGTCTACTTT

GGATTCCATTTTTGATGCTCATGCCTTCCAATTGAATTAACTCCATTTGTTTTTCTTTTG

TCCAACCTACTTTCAATCCTTCCTCATTGTCCCTTTCTTCTTTTTATTTTTCCCGCCTTC

GTCTATAACAAATTGAATTAACAATGTAATGAACGTTGTTTTACGACGGCTAAGATAACA

GAGGAAGGAAGGTTTGTTTTTATTGGAGATTTCGTCCCTCTGTTGTTAGTCCTTCCCTG

>Tb927.6.5050 | Trypanosoma brucei TREU927 | V-type ATPase, C subunit, putative | genomic | Tb927_06_v5.1 forward | (geneCodeEnd+0 to geneEnd+0) | length=420

AAGCGTGGAGGGGGAAAAAAAAAGAAAAAGGGAGGGAGCGAAGAAGAGGGAAAGAAAAGG

GTTTGATGGGAGGGATAAAAAATTAGAGAAAAGTTCAAAAAGGACAAAATAAAGAGGAAG

AGGAACCCCCTCACGGTATTGGAAAATGCCGTCCATACGGGTTTTCCCACCCCCGGTTTT

GGATGCCTTTCACAAATAACGTCAGAATACTTGAGTTCTTTTTTATTATATTTTACAACC

CCTGCTGTTTATCTTCCTTTCCTAACCCCGTCAATCACCTTTCTTTTTTTTTCTTCTTCT

GTTTTTTAAATCCACAATACACTCTGAATCTTACATTTTCTCCTCTTCTCTTTTCCAGGT

TCTTTTGCCTTCTGAGCCCTCCGTAGGGCTCCGCTGCAACAGGTACTCGACTACAACATT

>Tb927.10.6850 | Trypanosoma brucei TREU927 | Mitochondrial ribosomal protein S18, putative, mitochondrial RNA binding complex 1 subunit, mitochondrial edited mRNA stability factor 1 subunit, kinteoplast poly(A) polymerase complex 1 subunit (MRPS18) | genomic | Tb927_10_v5.1 forward | (geneCodeEnd+0 to geneEnd+0) | length=420

AAGGAGGTCTTATCCGCCTGTGTTTACACAGCTTTCGTCAAAACTCCTCCTATTTGATTA

TTTTCTGTTTGTTGAAATGATTACGTTTGGTGTCTTCATGCTGTATACCTATATATATAT

AAATATATATTCTTTTTATAGTGACTGCTCCGGCTCTGTCAATCGTATTTGCCCATTTTA

TTCTGACACACTGTTCTCTTTTTTTTTTTTCGTAGTGATGTAGTAGTGGTATTTATGCGA

AATTTTGACTTGTTGGGGGGACCCCTCATAGTAGCCACCACTGAATAATACACAGTACTC

CAAAGAGGAGTAGAGAAGTTCGTGAATGATGCACTTTCATCTGTAAAACAAGGTCCACTA

TCTCAAATATGTGAACGGAAGTACAAGTAGGGAGCAAATGTAGCTGACTATTGCGGCACC

>Tb927.10.15590 | Trypanosoma brucei TREU927 | macrophage infectivity potentiator, precursor, putative (MIP) | genomic | Tb927_10_v5.1 forward | (geneCodeEnd+0 to geneEnd+0) | length=422

ATTGGTTGATTATTGAGAGGAAGGGGGAAGGTAAAAGAATTTGCCGTGTATGATACTGTG

CGAGGGTGCATAAACGCGCTTGCTGGTAGTGTTCACCGGGGGATCAGAGTTGTTAGGGAG

TTAATGTGTGTTAATAGTGTTTGTTCCCGTTGGAAGAATTTTCACATATGTATGGGGGAA

AGGGAGCAAAATCAAGGCGGCACCGCCCGGGAGGCATCATCTCTACTGCTGTTTTTTGCA

TATCTATTGTGTTTGCCACCCTCCTACTCCTGTAAAGTAGGGGGTTGTATTCTACCGATG

TTAAAGCAGAGACAACGACAGAAAATGGCAAGGGCAATAACAACCAAAACAACCGTGATC

ATCCAAGTTGCAGGTACCAATAATGGAGACGATACCAAGTGCCTATCTTATCTCTTGTCA

CC

>Tb927.9.8200 | Trypanosoma brucei TREU927 | Pescadillo N-terminus/BRCA1 C Terminus (BRCT) domain containing protein, putative | genomic | Tb927_09_v5.1 reverse | (geneCodeEnd+0 to geneEnd+0) | length=422

AGAAAGCAGAGCGGAAGCCCGTCTTGCTTGCATTTATGGGGAATTCACGGTGGATAATTC

ACTCGGAGCGTAGTGTTTGACTCGTTTCTCAAGGTCTAGGTTGTGGTGTGTCGCTTCGCC

CTTGGTTTTTTTTTACTTCGGCTTCTTGCAGATCCTTCGCTTGGCCCACAACTCATCATT

CCGTAGCCACATTGTACTGTGAGTGGTAAGGAAAGAGAATCAATCATAAAACTGAATGTT

TCTGTCCGTCTCTTGTATTGATTTCTCTCGCATGTGCCGTTGCGCAAAAAGAGAGGGAGG

GTGAGGAGTTTACGGTTGTTTGAATCGGAGGGAAAAAAACGGTGAGGACAAGCGTTCGAT

GCGGTTTAAGATTAGTTAAAGTAATGTTCGAATTCCCGATAACGCCGCTTAACAGTCTTT

TT

>Tb927.4.3980 | Trypanosoma brucei TREU927 | chaperone protein DNAj, putative | genomic | Tb927_04_v5.1 reverse | (geneCodeEnd+0 to geneEnd+0) | length=422

GTAGGGCTTCAACCTTATACGGGTGTCACTTGCGTAGCAGTGTGTAATGTCTCTCAAACA

GTTTATTCCTCTCATTTTTGCGCTCGCACTTGTGGGCGCCCGTCCCTGCGTTAACACATG

TACTAAATACATAAATACGCATATACATATGCGCTTGTTTGCACCTGCTAAACAGTGTTT

GCTAGGTTTCTGTTTTATTCCCCCAAAGGCGGTTGCATGTCAGGTGTGAGAGGATGGGCT

TCCTTTAATTAAGGAAGCAGTAGACAGCAAAGGGGAACCCTAACTTAATTAAAAGGAGAG

GGAAAGGAAAGAGAAGAGTTGAGGGGGCACCCCCCCTCCCATTCGTTCAAAAATAAGGAT

TTAACTTTCTTTTCTTTTGCATTTTTACCAACGGACCTCTGACTCATGCTTCTCTTCGAC

CC

>Tb927.9.2980 | Trypanosoma brucei TREU927 | hypothetical protein, conserved | genomic | Tb927_09_v5.1 reverse | (geneCodeEnd+0 to geneEnd+0) | length=424

AGCGATGCAAGAAACATCTGCCTTTTAGAGGATAGAAAGTGACAAGATGAGCATGTGGGG

TTTGATTGTTGAGTGTGAGATGCCAAACTCCATGTACCAGGGAAGCATGAATGTATTTTT

TGTGAGCGCCTCCACACGCACACTCTAATTGATGTGGTTGAACACCAGACAGATGGTGTT

TGGCGTTGAATTTTGCATGTGAAGCTGTATAATTATCGGCGTTTTCACCTCCCGATCGAC

AACGTCTCATGCTTTTCCTTTTGTGCCAGCGTTGTAGTGAAGGGGGCCACTATGCCATGC

CCGTTTCCTACTGTAGGGAAGGATATGGTCCACACTGTTTTGTGCGGTGCCCCACTCGAC

CGCGGAAGGTAGAATAGAGGGAAAGCAGTTGGAAACGAGGAGAAGAAAAGAGAAGATACG

GTTG

>Tb927.11.3740 | Trypanosoma brucei TREU927 | proteasome regulatory ATPase subunit 2 (RPT2) | genomic | Tb927_11_v5.1 forward | (geneCodeEnd+0 to geneEnd+0) | length=425

GCGGCGTGGTCGGGAATGAGTTGTGTGGGAGGCGGGATGGGTGTAAAAGTCATGCAAAGC

ACAGGGAAAGGCCACAATTCCATATGTGTTTCGACTTTTCTAGTGGCCGCTCGTTCCCTT

TAAACTCGTTTCCCTCACTGTCCCGCCTACACCAAAGGTTGTGGAGTGCCTTTTCTCCCA

TAATCCCCTCATTTATTACTTCCCTCTGATGAATCTGACAGATGTTGAGTGAATACAGGA

CTGTGTGTGCGCATGTGTAGTTGGGTTATATATGCGTCCGCACCGTGAAGAGAAACGACG

AGACCGTTGATGTTTCTGGTGTCATGAAACGTACAAAATGATGATTTCCCCCCCTTTCAT

CTTCTGACACCTTGCGCAGTACATTTTCAGCGTTTCTATGCATATTACAAATATGTGGGG

CAAGT

>Tb927.8.4820 | Trypanosoma brucei TREU927 | eukaryotic translation initation factor 4 gamma, putative (eIF4G3) | genomic | Tb927_08_v5.1 reverse | (geneCodeEnd+0 to geneEnd+0) | length=426

ATGTGAGCGGATAGGGAACAGCGTAAGAAGGGTGAAGTTCGTTGTTGGCAGCACCTCTGG

TGGCGTCTCGCCGTCAGAGTGGGTGAAGGGGCCGCAGCGCGATTAAAGATGGAGGAGAGT

AATTCACCTGCTTTCACTAATGCATAAGGAAGAGACAAAGTTTAAATAACACCAGTGCGC

GATAACGTAAACGACGTCGGGACAAGAGAGAATAAGTCGAACGCAAATAAGCAGGGGAAA

CAGAGTGTGTAAGCGATGGCCCGCTAGGTACAGGGGAGTGCGGTCGTGTGACCTGTCGCT

AAGCAAATTTGATGTGGCCTATGTGGTCACGTTGACGCGTTGGTGTGAACGTTTCTGCGG

AATCCACTTTTGCTTTAGGATAGGAGGGAAACAATGTTAATGTTGTCTCGTGTACGTGCA

TGTGGT

>Tb927.3.2610 | Trypanosoma brucei TREU927 | GPI inositol deacylase 2 (GPIdeAc2) | genomic | Tb927_03_v5.1 reverse | (geneCodeEnd+0 to geneEnd+0) | length=427

AGAAAATAAAAGCGTGTATATTAACTTTGCTTTTGGAATCACGGCTCTCGTGTTCCTTTC

CTTTCCTTTTTGTTTTTTTCGTTTTCTTCCTCCTCTCTCCCTGTCTTTTTTTTTGTTGCT

GCTTGGTGGACTGGCGGCACACTGGGAGACAAACGACGCTGCAGTGGTGTCGTACGAGAC

TTTCGTAGTCGACCTCCAACCTGCGATGAAAATCCACACTTGTCTCGGCAGGAAGGCGTG

AGGGCCATAACTTTTTATAATATTGCGGCTACCCAATCCATGGTTGACGTTTGTCGTCTT

CTGTTTTTGTCGGTGTTGTTTCATTTTTCTTTTCTTGCCGCTCCTTTGCATTTCGCACGC

AGTTGTGTATGCTGCGACGGTCTCTCCTTCCCTGTGTTCGTGTGTTTGCTTATCCACGGC

ATACTTG

>Tb927.10.15760 | Trypanosoma brucei TREU927 | hypothetical protein, conserved | genomic | Tb927_10_v5.1 forward | (geneCodeEnd+0 to geneEnd+0) | length=429

AGTTTATCACTTGACAAGTATGGTAACAGTGATGAAGCGGACCGCAAAGAAACCATTGGA

AGGTGAAGAAGGAGCAGAGGCGGTAGCAAAAGGAGTGCGTAGGCATGTGAGTGGAATTTC

GATGTGCTGGACTAGCTTGATTTGATTTTTTGTTTTTTGTTTTTGTTTTTTTCCCTCCTC

TTGTTTGTCCTCCTCTGGTCACCCAGCAGACCTCTCGAACGGTCTCCTGCTCATCACGCA

AGAGGGTAAAAAGAAACGGGATGTGCAAATAAAAGCGTGAACAAGAAGGATGAAAGGGGG

GAAGCAAATGAGAAGTGAAGGGGAAAATGTATTTATAGTTTGACACTCACGTACACCCAT

ACAAAGGTACGTGATGCCAAAGCATCAGCGTATAACGTGAATGTCTGCTACACTGTCTTT

CCAAATGTC

>Tb927.10.12930 | Trypanosoma brucei TREU927 | hypothetical protein, conserved | genomic | Tb927_10_v5.1 reverse | (geneCodeEnd+0 to geneEnd+0) | length=429

AAAGACAGCGGTGGGCGAGAAGTGAACTCACGTGGGGGGGGGGGTCACGCGTGCACCAGG

TTACAGAGCCATTCTTGAATGGCTGTTGCAACGTGTGGGCGGTCGAGAGGGGGTTCTCTG

TTGTATAGTTATGCTTACGTAGGGATCGGCTTCGGTGGGGTTATCCCATAGTCTCTATTA

GAAGTAATAAAGTGGGAAGTGTTGCTACTACCACCCGTAAGATGGTAATTATCCCCACCG

CGGACCTTGTTCGGTTGCCGCGTGGAGCGAGTGTGGGTGTTACGGTTATTGGACCACGGT

TCCCAGACCCTAGCGCAGTGTGCATTCTGTTTTGATGGCCACCTGAAGAAAAACGATCGC

TGAGGAAGGAGATAAAATAGGGAGCGTGAGTAACGAAAGGAGAGATGCGCTATCGGAGAA

AGTGCAAGC

>Tb927.10.6030 | Trypanosoma brucei TREU927 | proteasome subunit alpha type-1, putative, 20s proteasome subunit, putative (PSA1) | genomic | Tb927_10_v5.1 reverse | (geneCodeEnd+0 to geneEnd+0) | length=430

ATGATGCATCGACCTGAATTAGCTCTTATTTCTATCACTTGGAGCGTTTGCTGGAGGAGG

AGGGGATGGTAGCAGTTCGAGGGTGTGGGGTGTGACGGGTGTTGTGGTTGTGTTCCCCCA

TCCCATCCCTTCCATTCCCTTCCCCTTCTCTCTTCCTTCCACTGCTTCAGTCTCGTTTCC

CCTTCCTTAGGTAATGTACTCGTACTCTGCTACTGTTCTTCTTACCGTTTCGTTTTCTCC

AAGCGCATCAGTTAGGCTGCCCCAGGCGCTTCTGCATTCGTCGTGGAAATATTCATATAT

GGAAATAACGGGGTTGTCGAAGGCGATGGAGACTTTTCAGGGGGTTAGAGGAAGTAAATA

CAACAACTGAAGGTAGCTTTGAGCCCCCTCAAGTGGGCAAAAGAAGTTTCGGGAGACTCA

TCTTGGCACT

>Tb927.11.7270 | Trypanosoma brucei TREU927 | proteasome beta 3 subunit, putative (PSB3) | genomic | Tb927_11_v5.1 forward | (geneCodeEnd+0 to geneEnd+0) | length=430

GGTGAAAATTAGTAAGACTTCCCAATCACTGTTGTGGGGCATGGTAGTGTAGATGACGGG

GAAAGGTATTTTTACATCTGTGCGGCGTCATATTTTCGCTGCCAGCGCTTGTCTTCCCTC

CCATCTGTTTCTTCTCACTTCGCGTGCTGGTGCCGTTTGTGCTAGCGCCTTATCACCACC

GGATCCTCACCTTGCCATACAGTGTTTCTTTGCAAACGAGGGTGGCCTTTCTTATATGTG

TGTTGGTTAGCTCGCCCTACCCCATTTTCCCCACCGTTCTAACCGTGAGCAAAGAGAGAG

ACATGAGAAAGTAAGAAAAAAAAAGAACATGTGATGTGTCAACCAACGGCGATATCGGCG

GTTGTCTTTTCCGAAAGGTGTCATTCCGTTGAAGAGGAATTCTTGTGGCAGAGGTGGCTG

ACGTAATTTG

>Tb927.11.1370 | Trypanosoma brucei TREU927 | glutaredoxin, putative | genomic | Tb927_11_v5.1 forward | (geneCodeEnd+0 to geneEnd+0) | length=431

AATTTGAGACAGTGAGGAGGATTTTTTTTAAAAAAAAAGAAGAGAAAAAAGAGAAAGGGG

GGATTACGTGAATATATATGCGGTTGCAACCCTCTGATTAATATAGTGCCAAACGAGAGG

TGTTTGCATATGTTGAGGGGGTAAACTACACACCCTTCCTTTATATACATCTCCTTCATC

CTTCACTTGATCGCTTTCATTTTTTTTATATTTTTGTTCCTGCTTATATTAGGGGTTATA

ATGCATTATGCATGATGTGTTCTGCTCATTTCATACACGAGGGAAGGGAAAACGGAAACG

GAAATGCAAGAACGGGAGAGAGTAGGAGCGAGTGAACTGACATCAATTCAATCGGGGGAA

GTGTTTAAAAACACCTCACCGGTATAACTTCCCCTATTGATACGTTACGCACGATGCTAT

TTCTGTTGTTG

>Tb927.11.8800 | Trypanosoma brucei TREU927 | hypothetical protein, conserved | genomic | Tb927_11_v5.1 forward | (geneCodeEnd+0 to geneEnd+0) | length=431

GAGGCGGGGAACAGTTCGTTTATGGTGCGTGTGACTGTGCACCGCATTCGTGTACGTTAA

AGGCGGAAGCAATTTTACCGGAGGTGGGAACAGAATCTGCTGTCGAGATGAGATCATCGA

ACGGGAGAGAGCATCAAAAAAATGTTTGACAACTTTATTTCCCAGCTTATGAGAAACACT

TGTTTGATTATGAGGTGTGTAACGCTTTTGTTTCCACTTTGCCTGTATTTTCAATCCTGC

TGTGCAACATTCGACGTGCACGACCGTAGAGGCACAGAGTAAGGGAGAAAGGATCTAGGG

AGGGGAAGATAGGGCGCCCCCTCCCGTGCTTACATCGTGTGCGTTAAAACCAGCCACCGA

GGTGGCAAAGGACCGCCTAACGGCAATTATTACATGATTCGATTGTTACGGCGGTCACGT

TTGGCATTGTC

>Tb927.7.6960 | Trypanosoma brucei TREU927 | ubiquitin-conjugating enzyme, putative, non-canonical ubiquiting-conjugating enzyme 1, putative | genomic | Tb927_07_v5.1 reverse | (geneCodeEnd+0 to geneEnd+0) | length=432

AGAAAGGAGAAAGGGTTTTGCAATAGCTCCCATAGTCACCTCCATTCGTGTCGGACCTGT

GCAGCGTCGCTAACACATTAATGGATCTACTCTTATCTGTCGAAGCCACTAATTCCAATT

AGTATCAGCAGGAGAGTAAGCAAAATGAAGAGAGTTTATATCTGTGTTGCTTTGCTTTTT

TTTTTTTTAATTATGCATTCCCTTATTGAGCGCCCACACCTCGTGGGGTCATTCACTTAC

ATATGGTAATTCACTGAAGCGGCAAAAAAGGAAAGAGCAGACGAAGGCGTGTGGAAGAAA

AGAAAAGTTGTAAGCAGAAACAATTGGAGGAACGAAGGTATGAGAAAAATTGTCACGCCT

GCTACAAGTGCAACCGGTTGTGAGTCGCCCAAGGGACTCCTTCACATATATACACGCCGT

TACTGCTCGCTG

>Tb927.10.15520 | Trypanosoma brucei TREU927 | signal recognition particle protein, putative | genomic | Tb927_10_v5.1 forward | (geneCodeEnd+0 to geneEnd+0) | length=432

GCGAACCCTCACCCTCCCTCCACGGGGAAACAGCAGTGGGGAAACTCAGGTAAAAAGCGT

AAACCCCGGAATTGTCACACAGTGTGGTGAAGGCATTGCTGTTACGGGAGTGTTGGAGTC

TTTGAGTCGTCGCATTAAAACATTCCATGACGTGGCTTTGTGTGTCTGCATTCAAATGGA

AATGAACTAAAATAAAACAAGTGTGGTAAGAGGAAGAAGCGGTGTGTTTATGAAGTGGGG

AATGAAGGAGCAGAGCGGAAGTGCGGAGGAATGAATGGAGCAACTTGCGTCTTCTTATAA

TCTTTTGTACTACCGTCACTTGCCAAACCTCGAATAACCATCTACAAGCGGAAGAAGAAA

AACGAAGCGGTTTAAGGGGCTCACACGGTAGTTTTTGACTTTATTCCGCATGGTGGTAGT

TTCGCTTTCCCT

>Tb927.8.3790 | Trypanosoma brucei TREU927 | paraflagellar rod component, putative (PFC2) | genomic | Tb927_08_v5.1 reverse | (geneCodeEnd+0 to geneEnd+0) | length=432

GATTTAAAAGGAAAAATAGAGAGGAATGTTAGCAGCAGACCGGATAGCACACACACACAC

ACACACACGCACACGCACACGTTCACTCCCTGTCCCCTGAAGATTTAACCAGCCCTCCGC

GAGACCTCATGGAGGTGCGGAAATGCAGAGATAATTGTGGGGGCAAAGGGCAACACCAGA

TTTTCAATGTAGATATGTGAGAAAGTGCTCGTAGCATTTTCAACAATTATGTGTACTTTT

ATGTCCTCAACTGAACATACTCGCCTGTAGCGCATGCGGGGAAGGAGGCTGTCTGGGAAA

TAGTGTGCAAAGCAGTTATTTCAAGGTTTGGGTAGCGGGAGGAAGAGGGTGAATATGAAG

AAGTGTTAACTTTGCTTTTTTGTTTCAACAGCTTTCCCTGCTTTCTTCTGTCCTGGTACT

ACCGCACTGCGC

>Tb927.11.4260 | Trypanosoma brucei TREU927 | Protein of unknown function, DUF607, putative | genomic | Tb927_11_v5.1 reverse | (geneCodeEnd+0 to geneEnd+0) | length=433

GTAAAAAAGAGATGTTGATCAGGGAAGTTCTTCGGTAGGCGCCCGTTAAAAACAAAAAAC

AACGTTCTTTCATCTAAATTAATTCTGATGCTTCACGGGGTTGATGTGCATGTGTTCGTG

CTTCTCGTGTTAATTTTGGTTTAAACTTACACTTATTGTATGCGCGTTGTGAAAATAACG

CCGTATACCAACGGAAGATGTGATTGTTTAGTTTCTTTCGCTTTGTGCGGCTGTGTGACT

GAGGTATATTATTGACCAGGAGAGTGATTATTTCCGCCCCATCCTTCTCATTGTTGCCGG

ACTATTGGATTCCCTCTTTGCAAATGGTATTTCACGCGTCTACGTCCTTGTTACCATGAC

TACTAGCTTCACGTGCGGGGCTACGCTCATGTATATATACTTAAGCACAAACGTTTCGTT

TACACCATTTTTT

>Tb927.11.11910 | Trypanosoma brucei TREU927 | hypothetical protein, conserved | genomic | Tb927_11_v5.1 forward | (geneCodeEnd+0 to geneEnd+0) | length=433

ACAGGTTATATCCCCTGACCTCTGATCCCGTGAGAGGTGAGCGTGCAAAGTACAAGGGGA

ATCTTCTGCAGCATGCATACAGGTGCTCGTTGAGTGTTGAGAATCTTTTCGAAAGCGCGG

CACGAAGTGAAGGCTTCTGATCGGTTGTTGTTAACTTTCCTCACGCTTGTGAGTACAGAC

CCACACTTTCTCCTTAAGATCATTTTTGTACCCCCTTTTTATTCATTCACTGGTTGGGGA

ACTCTTCAGATAGATCCCGCTATCTTCAAAGCCTAAAGTTAAAGGGGCTTGATCATAGAG

AGTGGGTAGCAGATCGTGAATGCAGGCTAGAAGAACGAGGAAGTTTCCCTCTTCTTCTGC

ATCGCAAGCGGATTGATTTGGTAGATAGCGGTTCATTGTTAAAGTTTCTAGCTTTGCCAC

TTTGTGGTCGAAG

>Tb927.11.250 | Trypanosoma brucei TREU927 | ubiquitin carboxyl-terminal hydrolase, putative, cysteine peptidase, Clan CA, family C12, putative | genomic | Tb927_11_v5.1 reverse | (geneCodeEnd+0 to geneEnd+0) | length=433

AGCCAGTGAACCAAATATGCGTTGGTGGGAGCCACTGTTCGTGTCTGCCGCGTTGCCTAC

GCATATGATGGCACCTGTGGTTTGATGGAAAGGTAGAGGTGGAGATGGTTACTCAAACCT

CCGTAGGGGTCGTCCAGATGGTGTTGGGAGTTTGTCAAAATTCTTAGAGGGATTTGGCCT

CAGCGTGGATAAATATTCATTCTTTAGTACACAATTGTGTCCCATGCGCTATTTCATGTG

AAGGATTTTGCTGGGCGTTGGCACGGTGGCTCCTGTTAGTGTTGCATCGCGGGGGTTACT

TATTGTTGTTCCTGTGATCATCATTATTATTACTATCATTACTGTTGTTTTTGTGTCCGG

CTTAACATTTATCAGTTTTTGGGGAGTTTGTTGGTGGCGAGAGGCATACCCAGTCATCAA

GCAACGCAAAAAC

>Tb927.7.570 | Trypanosoma brucei TREU927 | prefoldin, putative | genomic | Tb927_07_v5.1 reverse | (geneCodeEnd+0 to geneEnd+0) | length=435

ATGTGAGTGGCGTTGTGGTGAGGATTGTGACGAGAGGGGACGCTTTAAAACAAGGTGAGG

GACACAAAGTTTTGGGTTTTCTCACTGTGGCCGAATGCAGGTTAAATGAAAGAGGTAACT

GTAAGTGAGAAGAGTGGAGGGAATATAAAATATATTGAGAATCAGGTGGATGGACACATT

AAACAGGGACATACAGGGTGATTTTTTTTAAAACCCTTTTTCGAAGCGATGCATAGAATC

CCCTCTCTTTTTCTCATACTGTTCCTCCTATATGCACCTGCAGAAGTGTGTCTGGTCGCT

TCACTATGATGATAGACTAAACTACTGAAGTTTTGAAATGAGCCCTCGTCCACAAGTCGC

CCTGCCTCTACTCTTTTCTTTTTCTTTAAAAATCTCATCAGAGTGCTTCATCGATGTGCT

GTGCCCCATCACATC

>Tb927.3.1000 | Trypanosoma brucei TREU927 | frataxin-like, mitochondrial precursor, putative | genomic | Tb927_03_v5.1 forward | (geneCodeEnd+0 to geneEnd+0) | length=436

GTGTTGAACCGTTTGCGTGTGCATACGCGTGTATTGGGGCGTATGGCTGACCTGAGCGTC

GGGAGCAACCCAACGTAACGTGTGGACGCGTGGGTTCGTTTTAAAGATGGGTGTCGTTCC

CTCACGTACCGGGGCGAATGTGTGACAAGGGGAAAGGGAGAAAAAGTGATGTTGAACATT

ACATTTTGAGTGTTCGTTGATATGAGAGCGGATAGCGTGGCGTGGCGTGAATGGGTGGAT

GAACTGAACATCCATGTGTGAACAGTCGCCTCGGGAGAATGACGAGGAAAATTGTTGTTG

AATTCAAGACATAGTCCTGCGACAGCTTTGGTATCTGTTGAGTGTGTGTGAAGGTGGTTT

CAATTCATTTGCGTGAGCATGTATCGCGTCCTTGTTGTAGTTAATGTGTGATTGTTTGTT

TAATGGGAACGACAAG

>Tb927.10.5080 | Trypanosoma brucei TREU927 | GPI transamidase component Tta2 (TTA2) | genomic | Tb927_10_v5.1 forward | (geneCodeEnd+0 to geneEnd+0) | length=436

ATGACCTTTCCTCTCTTTTTTTTTTACTTCAAGTTCCTTGCCACTTTTTCATTTGTTGGT

CACCGGACCTCTTCGGTTCGTTTTTAATACCTTTTTGTTTTAACTTTGGAGGCGAGTGTG

GGCGGCTGCCGTGTATATGCGCCCTGTGGCTTCTCTGTGTTTGTCAGTGGTGGTTCTCAG

TAAAGAGGAGGTGACCTGTTGAAGTGGAAGCGGTATTCCTAATAGACTTCAACCGCCATT

AACACTGATATTCCCGGTTCGATATGCACTCAGACAATATTCTCACTGCTAAATACGCAC

AAGATTGTGGGGGAAAGAAGGTGGACACGTGTGCGTGTGTATGAGTATGGGGGAGTGTCG

CCGCATGCCAAGGGGTGGTGCCATGTGTGGCGAAAACGAAAGAAATAAGTGGAATAATAA

TGGTGAGAGGTAATTG

>Tb927.7.7330 | Trypanosoma brucei TREU927 | hypothetical protein, conserved | genomic | Tb927_07_v5.1 reverse | (geneCodeEnd+0 to geneEnd+0) | length=437

AGGGAAAGCCATGTCGATAGTGCCCGACATTTCCTCATTTATTTGTTTGTTTGTTTACGA

GTTCGTATGTTTGATCACCCGTCGGTCATGTCGAATACGTTAATCGGAGGTGCCGGAAAC

GGCAGAAGAAATGCGATGTGGCAACGTTAACATCTTCATGAAAAGGATGCATAGAAGGGA

AGAAAAGTGCGGACGAGTTGGGAATCAAAACCTCACGTCCTCCTTGCGCGGACAAGAGAA

AAAGAAAAAAGAAAAAAATTTCCACTGAGCTATACGTGCCTGAAGGATGAGCCAAGGGCT

TGCAACTCCATCAGCCCCCTAAAGTAGTTGATGCATCAGAGGAAGATGTGAAAAAGTAGC

AGAGGAGGCGTGAATTTTATGCGTACGTGTGCGTCTGTGCGTTCTGTTTCGCTGTGTTGC

CGTTGGGCCAACATTTG

>Tb927.11.11520 | Trypanosoma brucei TREU927 | glycosomal membrane protein (PEX11) | genomic | Tb927_11_v5.1 forward | (geneCodeEnd+0 to geneEnd+0) | length=438

GACAAGGGTCACAGGCTGCCAGCCGCCGGAAACTGGCTGCTGCGCGATGTGAAAAGGAGA

GAAGAAAAGTGAAAAAAACATAAAGGAAGAAAGGGTTATTGATAGCGGCAATACGCGTTC

ACGCCAATTTGCAAGTAGGTGCTAGGCACTGGAGGGGGTGAATAAACGGGTGAGAAAGCA

GCAAAGAGACAATGTTTATATATATATATATATATGTTCATCATCCCGAGTTGTGATGCA

ACGCCTAGAGGAAAGGATGAGGTGGAAGTGTCCGCTCGCATGCGCATATTTATTTACTCA

TTTTTTTTATAATTAACTTAAACAAGAGAAGGGATCCCCCGTTCATTCTCCAAATATATT

TGGAGTGGGGTCTGGTCATGAGAGAGTGCTTTTCTCACTGTTTTGTTCATTTTTCTTTAC

CCACTCTTGGCTGACTCC

>Tb927.8.1440 | Trypanosoma brucei TREU927 | maoC-like dehydratase, putative | genomic | Tb927_08_v5.1 forward | (geneCodeEnd+0 to geneEnd+0) | length=440

GCGTTTGACGTTAACGAAGAAAGGAGGTTGTGCCGTACCGTTTTGCATTTTCTCGTCTTC

TTGTTTTGGGAGCGTTGGGGGAGGCTGTGTGAGGGTTGTGACTGAGGCGATGAATGCGCA

TTCTAAGCAAACAAAAGGAAAAGGACAACTAAAAAGTTGAAACAATGGTGAGGTGGAGCG

GGGATAAAAAGACGACTTGTCAACACTTCTTGTCCATGCCGTTTCAATGTTCTTACTTGT

ATCCACGATCAGCTTCTGTATCCAGGGCAAACGCCCATCTAGTACGCGACCGATGGTGGA

CCCCACTTGAAAGGTGTGGGTATGGAACTCTAGGCGCTGTTGGAGAAAGGAAATTAAGGA

CGGAAAAAATAAGTGAGACCGAGCCGTAAATCATGAGCTCAGTAACTTGGGTATAGAGAC

TCTCCAAATTGAGAGGATTG

>Tb927.6.2000 | Trypanosoma brucei TREU927 | spliceosome-associated protein, putative | genomic | Tb927_06_v5.1 reverse | (geneCodeEnd+0 to geneEnd+0) | length=440

AGAGGCCGTGGCTGGGAAATAAAGCGATGAGCGTATGCGTTTGTATCACTTTTTGAAACA

GTGACGCAGGTGGTGCATTTGTGTGGGGTGGGGAAGGTGGGTGAATGTGCACTAGGGAAT

CGGGGGGGGGGAGTGGTGGTTTCGTATGCCTGCATCCTCATATATAATTTTTTTCTTTAT

GGGCTCCATTCGCTTTCCCCTATCCTTTCCCATTCTTGTGGTTCCACCGATTTATGTGGT

CGAAGATTAGAAGGGGGGGCGCAACCTTCGAAAAAATGTGAGGAATTGCATTCTTGATGC

TGCGGTTGCGTTAACGTACTCGTTACGTTTCCTAACGCCTGTCTTTTGAGTCCTTGCAGA

GTGTTTGCGCATAATAAATCTTATTTGTATAACGACAAAATGCTGTTGATGGAGAGATTG

GCATCTGATCCGCTCAATTT

>Tb927.9.15090 | Trypanosoma brucei TREU927 | cytosolic coat protein, putative | genomic | Tb927_09_v5.1 forward | (geneCodeEnd+0 to geneEnd+0) | length=441

ATTGGCCACGCCGATTGTTTCTGTGGTTACCTGACTGCGCAATCTTGTGCCTGCACGCGT

TGAGCGGAGAAGGGAGGAAATAAGGGTGGTCTTTCCCAGTGGGATGGTCCTGTGTATGAA

TGGGCACGCGTGAGGAAACTTACCAGTACCGACGTGAGCGTTGCGGTTTGGACAGGTTCC

CCCGTGCACAAAACGCAAATATTAATATATATGTTTATTTACATGTATACTCCCTCCTTC

GCAGTATGAGCGGGAAACAAGTGAACAACCGTGAGCCTGAAAAAGAACGCGGAAGAGGGA

AGTGTACGTTGAGAAACATACGACGAACCATTGACGAGAAGGAAAATGTGTAGGGGAAAG

GGGACGGAGGTAAAGCCAGTGTGACCCGGTGTTACGAGCGAGTTCAACTGCGTGATTTTT

GTTAATTGTGTGGGGCGCTGT

>Tb927.4.1040 | Trypanosoma brucei TREU927 | hypothetical protein, conserved | genomic | Tb927_04_v5.1 reverse | (geneCodeEnd+0 to geneEnd+0) | length=441

ATGGCAATGGCAGCGGGCGGCAACGCACGCATATATATATATATATATGCGCATGTGTAT

ACCCCAGTGCGTAATCTTCTTAGGAATAATAGGTGCTTTGATGCTCCTATGTATATGGTG

TGGATGGTCCACCTGCGGAATGTTGTTGTTACGCCGTGTGGAGGGAGGGAAGGGGCGAGG

AGAACTATCGGGTACAAGGGGGCTTCTTTTTCTTCACCTCTTTTTTTTTGTACATATTAA

TGTTTCCTTTTCCTGCTTTCGGTAGTCACTCACCCGCTCGATTCCTGTGTCCCTTTCTGT

ATAAATAATTCTCCTCTTTGTAGATGAATTTTACTCCTTGTGAGCAATCCTTCTTTCTTT

TTTTTTTCATTCTTCCACCCCTGATGATGCTTGTTTCTTTGTTGCGTTGTCACCCATGTA

CACCTTCGTATGTGTGACTAT

>Tb927.7.4660 | Trypanosoma brucei TREU927 | hypothetical protein, conserved | genomic | Tb927_07_v5.1 reverse | (geneCodeEnd+0 to geneEnd+0) | length=442

AACATTCCTTGTTTGGTGTGTTGGAATGGATTTCATTCGTGACGTATCCACGCCCTCGGA

CTTTGGGGGTTGATACTTTTTAATTTATTTTTCCAAGGCAACGTGTGCGAATATACGAGT

TGGCGACAAAGATTTAACTAATTATATCGAGCTAGAGTTTGTTACGTCCCGTTTCATCTC

CACTGTTCGTAAGCGTTTCTCTTCCCAGTTACTTTCTGGCGTTGGTACGGCGCCGCTGAT

GAGCACAGGAGGTGTCGGAGCAGTAGGTACGGGTACTTTGAAATGGGTATCAGTGTACAC

GGCACAAAGGTGGCTCACCTACTACGCCGCCATGAGCACACGCAGTAAGAGGTGGCGAGG

ATCCTTCTATGACAAAGGGGAAATAATGTCAGGTGTTGCGGGCTAAAGGGTGGCGTAATT

TGCACAACAAGTGTACATGTCG

>Tb927.10.12290 | Trypanosoma brucei TREU927 | UDP-Gal or UDP-GlcNAc-dependent glycosyltransferase, putative | genomic | Tb927_10_v5.1 reverse | (geneCodeEnd+0 to geneEnd+0) | length=443

AGTCAGCTGAGGAAGGACGTCGCGACTGTTCTGTAGTTTCGATGCCGTTTTCGCACGAAC

TAGCCGTGGGTTTTTGTGAAGCAACATGTAATCGACTGGACTGGATTGACGGAAAGTCGT

GGAGCAGTGCAAAAAGCAAGCAGCATCCTATCCCCGCTTCACCGGTTGCCGCACGGTTTG

CCTCCGGTTGTATTTTTGTTTACCGCTACGGATGCAAACGGGAACGAAGTTGGGGAAGCG

AGCCATGACACAGAACCGCCGGTTGTCGGCACCTTGTCGTCGCAGTCTAAACCCCCTGAG

CGTGTTGACACGCGCCACCCGCCCTTAGCGATACCGCTGGTGGACCTTCCCCCCCTCAAG

CATCCCCTAAAAGGAAAGAAAGGACAAATGTCCAATTACCGATAAGGTAGGAGCACGAAC

TGCAGAACCTCGTTAAACGGATG

>Tb927.2.5000 | Trypanosoma brucei TREU927 | hypothetical protein, conserved | genomic | Tb927_02_v5.1 reverse | (geneCodeEnd+0 to geneEnd+0) | length=444

AGGAATATATTTGTGTATACTCAGTGCTGTTGTTATCGTCATTTCGGTCTGTTTATATGT

TCGTACAGCCGTCAGCACGCTGCTAAACGAATAGAGTAGATAGATGCAAAAAGGACTTGC

ATGTGAACGCCAGTTCGTTACAAGGGGATAGAAGCAACAAAACGAAAACGGGTGGTGCCC

ACCTCTGGAGTCGGCGGAAAAGAGTGGTTCGTGTTAATACACGGAGTGCTTTTTTGAAAG

CCGTCACCATAGATATGTTCTTTGTACATAAGGGCGTGGTGTTGATCGTTATCTAGTTAT

ACCTTTCTTAATGTTTTGGACGCATGACCATGTCCCTCCATTCGGGGCACGAAACTATAG

ACAGCTGCAAAATCATAGTAGCCGATGAATTGTGATGGCCGACCGGAAGTGGAGCCTCAT

CTCGTTTCTGTTTTAATTTTTTAG

>Tb927.8.4380 | Trypanosoma brucei TREU927 | Archaic translocase of outer membrane 12 kDa subunit (ATOM12) | genomic | Tb927_08_v5.1 reverse | (geneCodeEnd+0 to geneEnd+0) | length=445

AATTTATTGATTAAGGGAGCCAGAGGAAGGGGGAAAAAAATATAGGAGAAGAGTATGTTG

AGGCCGATGAGCACCAAAAGAAGGAAGAGGGAAAGGGTAAATGAGAATCAAAGGCCCTGC

GGGAAAGTAACGAGGAATAGTTTTGCGGGGAGGGTGAAGCAGCAGGCGTCGATATCGTGA

GTATTGGATGTGAGTGGTGAGAGTTGTGGGCGAAAAATTCAAATGGTGGAGGAAACTAAT

TTTCTCAGGCACCAACTGGACGTGTAACGGGGTGTTGTGCTTTAAATCTTCTTCAGTACA

ACTTTTTTTTGTTGGGCACTTTCTTTGTCGCGAGCCATCGATTTTTAAAAAAAAACAGCT

TGTTTTCGTTTCCTCCGCTAAAATGCTAAACTTCCAGCGAACAAGTTGTTGTTATTATCA

CTAAGTTGTCGGTCCGCATCGTCTG

>Tb927.3.720 | Trypanosoma brucei TREU927 | zinc finger protein 3 (ZFP3) | genomic | Tb927_03_v5.1 reverse | (geneCodeEnd+0 to geneEnd+0) | length=445

ATGCAACGTTATTTTTGAGCTCTTACAACTGTTCCGTACGACTTGGGAAGAAATACACTT

GAAGGAAAAAAAAGAAAGAAAGAAAAGAAAGAAAGAAAGAAAGAAAGAAAGAGAGAAGCA

AAAAAAAAAAAAGAGAAAAATAAATGGCGGGTTGTCGTTGTGGGAAGTTCAGTTTTAGAA

ACACCGAAAGTTGTTCTTTCTTTTCTTTGTTTTTTTTACTTTTACTTTTACTTTTACTTT

TTTTTTCCTCTTTCATTCTTGAAATTTTTTTGTTTTTTTTTTGTTTTTTGTGTGATGCTT

CCTCCTTCTTTCATCAACAACAACAAAGATATACGATTTTGAGAAGAAAAAAAAAAGAAG

AAAAGAAAAAAGTACAGGGAGAAAAAAGTGAAAAAAAAAGGAAAGAAAAAAAAAGAAAAA

AAAATGGGAGAGGAAAGTTAAGTGG

>Tb927.11.14290 | Trypanosoma brucei TREU927 | vacuolar sorting protein, putative | genomic | Tb927_11_v5.1 forward | (geneCodeEnd+0 to geneEnd+0) | length=446

AGTACGGTTGGGTAAGTGAGGGAAGGGGAGGGGGAAAAAAAAAGATGAGCATTGAATTCT

CTACTCGCATTACCATGTGAATCCGTGGGTAAGCGGAAACTTCGCACGTATCTTCGAGTA

GGTGATATGTTCTACACTTCTGTAACGTGTTGTTCGACATCCTTTCCTTTTTTCCCTCCC

CTCTTTCCCTTTTATTGCTGATTGTAAGAGACTACCTTGTGGCTCAGTGGTGGTTAAGTA

CCGTTATCAAGCGAGTTTGCTTGATTCATTATGCTTCACAATTATGTGCCCTCCTGCAAT

TACAACTAAGAACCGCTGCGGTTGCGCGCCATTATTGCTTTAACTTTCTGTCGATTGGTC

GCGACGACATCAGTGAAGTGACAGGATGTCAGAAGAGTGTACACTCACTGAGGTGTGCGC

ATGCGGCCCGAAACTGAAGCGCTAAG

>Tb927.4.4620 | Trypanosoma brucei TREU927 | cytochrome oxidase subunit VIII (COXVIII) | genomic | Tb927_04_v5.1 reverse | (geneCodeEnd+0 to geneEnd+0) | length=447

ATGAAAGGGGTCCGAGCCGGTAAAACGCCAATGGTAGGGTTAGGAAAGATGCACGTAGTT

GTGAAAGGGAGCTATCGTGTACTATCGAAACTATTTTACGTACGTTCCATGCTTAGTAGT

TCATGTATAACTTTTATTCCTTGTTGTCTTTAAGTAGGTAGTCTTGTCTGTTCCCAGTCA

CCGTAAATGTGGATTATTTTTCTGTTTTTTACCTTATATTTCTAAAAAAATTCCAACGTT

CCTTATGGCACGGGTGTACTTCGTTGCAATGTTATATGTTGAGCACGGCAGGGTGCGGCA

AAACGCAAGGGAGTGGTAAAAATGTGAGTGACACTGTGGGTGTAAAGCACAAGCGTGGAC

CTGTTGCAGCCACAAGACGAGCGTTCGTTCTTTTAGCTGCATAGTGTAGGGGAAATAATT

TGCACAGAGTAAGTCAAGGGACGGTAG

>Tb927.8.570 | Trypanosoma brucei TREU927 | proteasome regulatory non-ATP-ase subunit 10 | genomic | Tb927_08_v5.1 reverse | (geneCodeEnd+0 to geneEnd+0) | length=447

ATGGGGTGTGGTGTTGAAAAGGAGAGGTGTGCCCACATGTTCCTCCAAAAGCCGCAAGGA

AAGACGTGTTTTCACGTACGTTAATCTCCCAAGCAAAAATATGTTTTGTCATTTAACCCT

GTGCTTTCCCCATCCTTGAAGGTCCGTGAAGGTGATCAACTCATCTCCATGCTTCCTTGC

TGCGATGCATATCCACAGAAGAGACGAGGCGAACAGATGGAAAGGGAAGAATGAAGACAA

AACCAAAGACACGAAGCAGGCGGAGGGAAACTAGCAACAGGATATGCACTGCACGAATCC

GATGATTTTCCGTGTTCCACTCGCGCGACCTTTTCTTCCACCGATTGGAAGTTGAGTGTT

TCTAAAATTCCTTTTTCTTTTCTGAGGCATATGGTTATAGGCGTTCTTTCTCCTTTGTCC

CTTTTCCCTATTGGCTACCGAATGTGC

>Tb927.3.2600 | Trypanosoma brucei TREU927 | ATP-dependent DEAD/H RNA helicase, putative | genomic | Tb927_03_v5.1 reverse | (geneCodeEnd+0 to geneEnd+0) | length=447

AGGGTTTGTCGGTCTTGTTTTTGTTTTTGTTTTAATTTTATCTATTTTTATTGACTAGTC

ATGGAAGTTGGGGAGCGACGAGAGTAGACGAGGGAAATACGCAAGGGGAAACTGAGGGAG

AAAGACAAGGGGGAATAAATGGAGGGAATGTGATTAGCGTCTTCCTCACTCCCACTGAAC

TGTGTTGGCCGTCGCATGCTGAAGATGATATTTGTATACGTGTGCGTGTGTGTATGTGTA

TGTGTGTCTCTGCGGGATGACACCTCTGCTGTCGTGCAAGCATAAATGAAGGGAGATGAT

AAAGGAATGATGATGATGTGAGAGAAGGTGAACGAGATAATGAGCGTACAAAGGTGTGGA

GCAGGCGGGAGTTTATTCGTGCACATTGAATGGGAGTGGTGAGAAATTCCTCGCCCCAAC

GATGGGTCTCCAAAGCACTTTGCTTTC

>Tb927.10.11990 | Trypanosoma brucei TREU927 | RNA-binding protein | genomic | Tb927_10_v5.1 forward | (geneCodeEnd+0 to geneEnd+0) | length=449

GCATACCTGTCCGGAGAAGTGAAGAAGAGAAAATAAGTAATACGTGTAACATTTTTTAAG

CGCTGTGGGGCCTCTCCCCATCTCCCCCTTTCTCTACCTCTATAAAAATAGGAGGAGAAA

CTGTGGTGTACTCTCCGTTGTGATCAAGTGATTTTTGACTTCCACGCACCATCATTTAGA

AGCGGCACTTTTCCCTTTTTTTTTTTGGTATTCCCTTCTTTTCAGCAACACGCACGTTGT

ATTAATTCGAACTCTCCGTTAGTGATTGTGTTCGCCTTCTGTGTTTACCAGTTGAAGAAG

TTGCTATATTTATTTATTGGTGTAAGTGCACTCCGCAGTGTGCCCTTGGCGGTGTGCTAC

ATATCGTGGGAGAGTTACGAAGGTCCGAAGAAAACATCTGGAAGGAGATCACAAAATAAA

AAAAGGGGGGGAGGTCAAAAGGAAGCTTG

>Tb927.9.2600 | Trypanosoma brucei TREU927 | hypothetical protein, conserved | genomic | Tb927_09_v5.1 reverse | (geneCodeEnd+0 to geneEnd+0) | length=449

AGTCACAACACTCACCGGGATATCGGTGACGTAGTCGCACTTTTTTGACCATTGATGTTT

TTATTTGCTCCACCGTCTTTGTTGTGTCGACTACGGGGTGTATTTCGCATACCGTTGCGA

AGTCTCACTGTTTCCTCCGCAAACTGTTATAAAGAAATTGCGCTTCGCGTGAGCTTGACT

GTTTTGCTACAACACCTGCACGCAAACTCCTGTCTCTCCTTCTCATTGTGATAAGACACT

ACTGTTTTGTTGTGCTATCTTCTTTATTCTAATTATTCTATTCCCTTTTTTCTTTCCGTC

TTACTTGCGTGGTTCCGTTGTATTATTGGCGGGAGAGTGAGGTAAGAGCCACAATGGTCA

TCGGCAAAGTAATATGTCAGTCGAAAGGTAAATGTATATCCGCCATTGCAGACAAACGGA

GCAGCAACACCAGCACAGGCAATGCCATC

>Tb927.10.770 | Trypanosoma brucei TREU927 | RF-1 domain containing protein, putative | genomic | Tb927_10_v5.1 forward | (geneCodeEnd+0 to geneEnd+0) | length=450

GATGGTATCGAATTACTAATATACATTGCGGTGCACACCTGGTCTACGAGGCTTTATTTC

AGTTAGCTAACTTTGCAGAGACAGTGATACACTTGTGTGCATGTGCAGAGCTAGTATGAA

GTCCTGATGGTCAGTCGAAATGAGATCTGCGCGGGCATATGTGCTCATGCACCGAGCAGA

TGAGGGAATGGTGCAAGGGGGAAATGCGTTCTGCAACCCAATGTGTATCTGTTTGCATGC

GCTTACGGGTCAGTGTTGGCACCGTTAGTACTGGGTGTTGTAACAACTGTTATCATCCTT

CAACCGCCTGGTGTTATGTACATCCCTCTCTTTTTCGTTTCGCTTCAGTCGTTGTTTTGC

GCCGCGCTTCAAAATTATGCCTGTACCCACCCTCATCCCCTCTGCCGTTACGGAGGAGTG

AGGAGCAGGAGGGGATCCTGTTGCTGTTCT

>Tb927.6.3920 | Trypanosoma brucei TREU927 | hypothetical protein, conserved | genomic | Tb927_06_v5.1 reverse | (geneCodeEnd+0 to geneEnd+0) | length=451

AATTACATATATATATATATGCAGAGAGATGTAGATACGGATTCCAAGTAAGGCTCTCCG

TTTTCCCGTCTGCTGGCTCCTCGTTAGACGTCTTTCGGTTTGCTCCAGTGTTTAGTAACG

AGAGCGGGACTTCAGGGAGAAAGAGGTCTATGGTGTTGTGCACGCTCCACTGTATCCGAA

CTCTCTCTCTCTCTCTTTTGGCAGTCCCCGTTTCTGTTTTCGTGGTGCGACTCCGCTATG

TACATCTTCGATTCCTTTGTTTACTCTTTGCACCAGTTTCATGACGTTTCTTTTTCATGT

GGTATAAGAGTTGTGTTTCTTGTGCACTTAACCATCTGTTCCTTTTTTTTTTTGACTGCT

GACGCTGTGCCTTTCTCCCTGTTACCTACATTTGTTTTCAAATACCTTCTTACTCTATTT

CCTTTTTATCGTTTACTTTGTTCGTTTCCTT

>Tb927.7.2290 | Trypanosoma brucei TREU927 | hypothetical protein, conserved | genomic | Tb927_07_v5.1 reverse | (geneCodeEnd+0 to geneEnd+0) | length=451

ATGGAGTGTACTGACTTCAAGTTATGTGACCTTGTGGAGCAACCCTCGTGTTTTTGTACG

TTAGGTTTCTTCCTTTTGTTACCTTTGCCTTTGTGAAAGACACACCAGTAGTGTCGAGGT

GTCCCCACTTTTCTTTTCTTTCCTGTCTCGTGGTGTCTGACTTTTTCAAGTTGCTCCTTA

ATGTCCCGTTTATTAACGATATTTTCCTGTACTGTGTGTATAACGTCAAGTGCCCAAATG

GCATAGGCGTGTGCTCGTGCATGCGCGCTTGTATATCTGTTCTCACCGACTATATATATA

TATATATATCCTATACTGTTTGCTTCAATTGAGTCGTTGCATATGCTCATTGTGCCTCCT

TTTCTTTCACCACAGTGACTTTCTCCCTCCGTACATTCTTGTTATCGTGCAGCCGAACTT

TTAACCAGGTGAAGCAAGTTAGTTGATTTGG

>Tb927.3.3760 | Trypanosoma brucei TREU927 | tryparedoxin 1b, putative (TXN1b) | genomic | Tb927_03_v5.1 forward | (geneCodeEnd+0 to geneEnd+0) | length=455

AGATGCCATGCTGTCTGTGAAGGAAGTGATAAGTATTGATGAAGTACGGTATGGTGTGGG

GCATCTGGTGTGGAAGCACTAGTTATTCGGGAGCAAGAAAATAAGTAAGTGAAAAGTACA

GAGCGAACAGGAGGGAGGGAATGAAAGTGAAAATGCTACGGAACTGAGCAGTAAGCGAAA

ATGGAAAATATGCTTTTGTGCCAGGGGAACATTTGTCATGCGGTCCTGCGTGTGTTTTTG

TCGGATATCTCGTCATCCATCTTGCGGAGTTTTGTTTATTGCTCTTTGTTTCGGGGTTGG

ATTCCTGTTGTGCAGGGGTCCTGTTCCTCACACTTTTGTCATTACATATGTTCTGACCGG

CACGCGCCATAATCTTCTGTGGCGTGGTTTGAATCGGTGTGGGGATATGAGCAGGAGCGG

TAAGATATTGAGATATGACGAAATATTGCCTTTAC

>Tb927.10.4840 | Trypanosoma brucei TREU927 | transcription factor IIa, putative (TFIIA-2) | genomic | Tb927_10_v5.1 reverse | (geneCodeEnd+0 to geneEnd+0) | length=455

ATTCTTTCGTTTGCAGCGATCGCGAAAATGTCGTTGCTATTAACTCGCTTAGTTTGCAAG

CAGCGAAGTTACCTTTGCGTCAAGTGAACCTGGCCGATCCTTCAGGTGCTTTGTGTTTGC

CTCTTTTTCTTGAAGCAATTCTTATCAGAGGGATGTTATTTATAATGGTTTCGTTAACGA

TAGTGTGGATGCAAAAGGACGGTCGTATTTTGCCTCGCCGTGTGCACCGCCGTCCTCTTC

TAGACCCCCCCCCAAAAAAAAAAGATAATAATAATAATAGTGGCGGTGTTGACGATACTC

CTCATATTCGGAAAATTCTGAGGTTATTCACCGCTAACATCTTGGTACATGCGTAATCAT

TACCGATAACGAGGCTAGGGTCGGCAGAATTACGTCCAGTAGTTTATTGGCCCATAAAGC

CCATACTTTTGCCCGCACAGATTGTTGATTCTTGC

>Tb927.11.6360 | Trypanosoma brucei TREU927 | 60S ribosomal protein L24, putative | genomic | Tb927_11_v5.1 reverse | (geneCodeEnd+0 to geneEnd+0) | length=456

AAGGGAGCGTGTGGTTAAGGATCCACCAATAATGATCACCTCTTTAGTTAATTTCGCACG

TTTCCCCTTTCTGTAGTACGCATAAATGTATGAATACATTTATATATTATCATAATTGTG

TGTTAATATCAGGTAAACAAACGGGGAGGAACCACCGGTGGAGTCAAAGCGATTCCAAAG

GGGCACCGCTGTGCAATAACATCACACTACGAAGCGACAGACAGCAACAACAGTAGTTGA

GTGGTATCGGGAAAAAGGGTGTATCCGCTGAAATCCCTAGTTTCTGAACAACTTAATTCG

TGTGGTAGTGAGTCGTGCTTTCTTTCAGTTTGTGGTATGCAAAGCACGGTGCTTGCAAGT

CGCTTCAACCAGTGTTATTGTGGTGGTGAAGTTGACTTCTCTTTTCCCTTAGTGTTCGCT

TAGTTTTTCGGTATCTCCCTTCACGCTTTCACCCTC

>Tb927.11.7350 | Trypanosoma brucei TREU927 | histone H2B variant, putative (H2BVAR) | genomic | Tb927_11_v5.1 forward | (geneCodeEnd+0 to geneEnd+0) | length=456

GAGCATATATTCAAGATACAAAGGAAATAGATCATTTTCCTTTCCTTCTTGTTTCCTCCC

TTCCTGTATTTGTGAGTTTTTTTTAATTTTCTGTTGATTACTCACCGCTTTTGTTGTTGT

CGTTGTCGCCCGTTTGGTTGCTCGAGATCATCATTTTTGTTTCCCACTTCGTGCCAGGAG

GTCTTACATTCCTTTTGTTGTTTTGTTATTTGCATTTCCCAACCCTGCCTCTCCACACCT

GCGAAAAGAAAAAAAAATGCACTGAAAAGGGAGGGGATTAGCCGTAACATTAGAAACATA

GACCGTGCAATGTTTGAGAAGGGCGCGGAAGGAGAGGAGGAACTGGCAGAAATGGGAAGA

AAAAGATTTCGAGCGGTTGTCTAAAGGGGTGGAAGAGGAGGGAAATACCGGCGTACTTAA

TGCAGCTGCACACGTATACGCCTATACATTTGTTGG

>Tb927.11.770 | Trypanosoma brucei TREU927 | hypothetical protein, conserved | genomic | Tb927_11_v5.1 forward | (geneCodeEnd+0 to geneEnd+0) | length=456

GCGGCTGTTTGTGTGGTGTACTGGGGCTCTTCAAAGTCTCCCTGCGGAGCGGGTTGGTGG

CGGGAACATACCGTTGGCAACCTGTTTCGGTCACTGTACTTTTTTTTTTTGGGGGGGGGG

AATGGGTGTTGACGAGGCTTCACACGTGTGGCAAAAGCCTACTTGCTCCCGCAGAGGGAG

ACCTGTGTAGTTCTTCACTTCTGCCTTACCGTACCCTCGCGTTCAAATGCGTCGTGGCCG

CTTAACATGCCTTTTCCTCCGTCTAACCCGTCTCATTTTGAGTTGGGGTGCACTGGAATC

CCACATACTGACGAACTTCGTTGTGGTTTATAGGCGTTTTGTCGAGGTTATTGCACCGCA

AAGTCCTATTGCCCAGTCACGGACCGACATGATCGAGTCGGATGGACTTTGGGAAATGCG

GGCTTCGTGTGCGGATCATCTTATGTGTGCCGTTTC

>Tb927.11.9070 | Trypanosoma brucei TREU927 | palmitoyl acyltransferase 4, putative | genomic | Tb927_11_v5.1 forward | (geneCodeEnd+0 to geneEnd+0) | length=456

AGGGTGGGCAATGTGCACCGTTGTCGAGGTATGGCACCCTCGAACTTCAACGCGGTTATT

TGGTGTTGCCTCAGTCAATACTTCATTGGGTTCCCTTTACACAAATGGGGCGCCGCCCTA

TGGACCTCAGTTGTTGAAGGTTACCTCGTTGAGGTGAGTCTCAGGGAAATACACGCGGAG

AGAACTGCAACGGCATGTCAGAAATCTCGGGGTCAAAAAGGGGAACCTCTCAAATGTTCG

CTGCACACTGCGGCTGCCATGACACACAGTGATATATACGACGATCATCATCCTTTATTT

GGGTAACGGCAAAAAAACTTACCGTGTTTTGTGGAGAGATCACAAGGTTTACCCCGATGC

CCTGGTGCGGACACACGATGAGGCAAAGGGCAGTGCGGCATATATATGTTACCCTTACTT

CCCTCCCCTAACCACCTATGTTTTTCCATTTAAAGG

>Tb927.11.8830 | Trypanosoma brucei TREU927 | hypothetical protein | genomic | Tb927_11_v5.1 forward | (geneCodeEnd+0 to geneEnd+0) | length=457

AATAGGGATCTCCTCCAATCTATCGAACGGCTGCGTCCTTTTTCCAGAAGAGTTGTATCT

GTTGATTTGCCCGTGGTGGAGATGGCAAGGGTGAGTTATATATGTAACTAGCATGTGGGG

TTGTACTATCCAAAGCCCATGTAGGTAGTCAATAAATGTGTACAGTTGGTTGTCGAGGTG

GGGGTGTTGCGTGCGTTGGTGAGGAGGGTGTGCTTTTTGTATGCATACAACGTATGCTTA

CCTCCCATCCACCACAACCACAACCTCCAACCAGTCAGAATGCTCGTTTTGTTCTTCGCT

TTTCACCGTATTCGGTACTGTTGCTTCTACCCAAATTTGAACACTTTGCTTTAAGTATTA

TCTTTTTTCTTTGTTTGCTCCGCCACGACAAGTCAACGACTGTGCCGCGACGGCGCCTTA

GTGGCGGCCCCTCCGTCGTTGCCCTTTTGCTCTGTAG

>Tb927.3.960 | Trypanosoma brucei TREU927 | protein transport protein Sec61 gamma subunit, putative | genomic | Tb927_03_v5.1 forward | (geneCodeEnd+0 to geneEnd+0) | length=459

AGCAGCATCTGGCGGGTCAAATTACAGAAACACCGCAGGCGGACGGACATATTTTGTTAT

GGCGTTACCTGTCAGGTACCATCACCTTATGGAGTAACTCCACCAGATGCACCCTTCCTC

TTTCTTCATTTGATTGGTTGTTGTTTTTCTTTTTTTTTCATATTTAAATATATTGTCGTT

TTATTGTTTCATTTTGAAGGGTGCTGTCATTAAGGAATTGCTGCTGGGAAAGAAGTAGAA

AAAGACAAAAAAATGAACAGAAGGGATGTGCCGAATAAAAGCTGTAGAATGGAGAAGTGA

TACTATGAGGTTTCTCGTTGTTTTTTTTTCTAAATTCCGTCATATGAAATAAGAGATTGC

GGCAATGCGTGTCGCGGGTTGCTCCAGCACACACTTGAAGGTGCGTTAGCATGTGCACCC

CCTCCCCCTCTATACGGAAACAGTAAGCAATTAAGCAAC

>Tb927.11.5390 | Trypanosoma brucei TREU927 | hypothetical protein, conserved | genomic | Tb927_11_v5.1 forward | (geneCodeEnd+0 to geneEnd+0) | length=459

GGATGCCGGGCATTTAGTTGGCATAGGCCTTTCCTTGGATATTTTCCTAAAGGCAAAGTG

CAGAAGGGGGTCATAGTGTGCCTAAATCGCTTGAGGGGTAAAGGACACCGGCAATTCGGC

GGTTGCTTTTGTTACTCGTTGTACCAGTTACATCATCAATTTTCTAATCATTCTGCGAAT

AGTCAAGGAGAAATACTGCTGTACGTACATTTTTTTAAACTTGTCTTTCACATGTATTAG

CACACGCGAATGCAGGTTTAAGTAGGCGATTCACTTTTCGTTGCGCACTTTCCTTCTTTT

TGTTAATTTCTATTGAGGTATGTTCTATCGTCAGTATCTTCTGCGTACGCTTGTGTGGTG

TTCGCTCCTCTTTCATATCCGTATCGGAAAAAAAAATAAGACAGAGGGAGAACATTATGC

CGAAATGCGAAAGGCAGGACTCCTTGATGTCTGGTGGTT

>Tb927.10.380 | Trypanosoma brucei TREU927 | mitochondrial RNA binding complex 1 subunit (PPR5) | genomic | Tb927_10_v5.1 forward | (geneCodeEnd+0 to geneEnd+0) | length=460

GTGAGGTGTGTTTGCTCTCTTACGGAGGGAAGGGGGGGGGGGGTTTCATGGGACGTTTTA

ACCAGGCGGTAGCGTTTTAGTTTGGAAGGATGCGAAATGGCAATGTGCTACAGCTCCTAA

GGTTATATTTTGTGCATGGATGTGCAATCTTGTGCCGTGTTTGGTCCCTTGGATTTCTTC

TCTTTTTGGTTGGTGGTATCGTTGCATGGACACATGCGCTTTTACTTTTTTTGTTACCTT

CAGTGCACGTAATGTACGTGGTCGATTCGCTTAAGCCATCCGCATGCGAATATGTATTAT

TGACTTACTGTTGTTTACCCTTCCTCTGGTTGCAAGATAATACTGTCAAAAGCTGCACGC

TGCTGTACCGTAAGCAATAGTGCTTTAGGAGGGCCGGTAAAAAGCAAAAAAAACAAAAAA

AAACAGTCCGTTATATATATAGAGAGAGGGAGAGGGGGAG

>Tb927.11.13020 | Trypanosoma brucei TREU927 | calmodulin | genomic | Tb927_11_v5.1 reverse | (geneCodeEnd+0 to geneEnd+0) | length=461

GTTCGTGTTTGCAGATGGTTGAGGTGAGCCTGGCTTCCTTCTCTACCTTTCCGTCATGTT

AAGTTTGTGTATTCGTTTGGGAAGAGATAAAATACTGGTATAAAACTCTTTTTGTGATAG

AAAATAGATAAGGGTGTTGGTGCTTCCCGATAAAGGGGGAGGAAACTGTTGAGGAGGTGC

GTGAAATAAAAGCAAAAGTGGCATAAGGTGTACATGATATGGGGCACTTAAGATTCTCAG

AAGGATTTGATACATGGCATCATATCAGTTTCGTACTGTCGAGGAGTACTGAGATTGCTC

TGAGAATTTTTGAATCCCTTTGACCTTTTTAACATTCTGATGTTACCGCGTTACCCGGAT

CTGTAACCTCACTCCTTCGGAACCATTTCATCCTTTATTTACATTTTTGTTATGTATATG

ACCTTTCCGGCTGTCATCAGTGAAAGGGACCTGTTTGTGTT

>Tb927.8.4580 | Trypanosoma brucei TREU927 | Tumour suppressor, Mitostatin, putative | genomic | Tb927_08_v5.1 reverse | (geneCodeEnd+0 to geneEnd+0) | length=463

GTTACATTTTTAACCGCAGGAACTTTTGTATTAATACGCGCATTATATTTTTGGTATTGC

CCTAGTCACTCTTTTTTCCCTTCTCCCCGAGTGTCTCTTCCGCTGCTACTTGATGCATTC

GTTCGACTTTACACACTGTTTTGTTTTTTTGCCTACACAGCTTCTAGGCCTCTCACGCTC

GTGTTTACTTTGTGGGTGAATGTGCCAATCGTTTGTTATTTACGTGGCGGTTAGAGTTGC

TTTATGTACATATACTCGTTGATGAATGAATGTGCTGCGGGGGCAGTGTGAGAGAACAGG

GAGAGGCACATCCATTTTACTCTTTTAGATGTATAATCTCTACTTACTCTTCTCTTTTTT

TTTCAGAAAAAGGAGACGGGGGACGTTTAGAAGCTGTTCGCGTGACCGAACGCATTACTT

GAGTACTCCCTCTACTGCTTTTCGTGAAGTGGGGCCTTATGAC

>Tb927.8.2100 | Trypanosoma brucei TREU927 | vacuolar ATP synthase 16 kDa proteolipid subunit, putative | genomic | Tb927_08_v5.1 forward | (geneCodeEnd+0 to geneEnd+0) | length=463

ACCAAGTTAGACGGAGCGAGGGCTAACGGAGAGAATAACCCCCCTAGGAAGAATGGGAAA

GAAAGAAAGAGAGAAAAGGTTGGATGAAGAATAGTTGCTCTGGTGCCAAACCATGCGACA

CAGAGTGGAGGGGATTTGAGTGGCGGGCTGAAGCACGAGAGGTAAAGTTCGTGAAAAGGA

TGACCAACAACTTGTGAGAATGTATCAATAAAGGTGTTGGTCCCGACAGAAGGTGAAAAA

ACTGGGTGTATCTGATTCGGGAACAGCACCGAAAGAAGGGGGAAATGCATTACTTTTGAC

AGGTTTCCGGGATGGTTAGAGGAAAACAACACTGTAGGAGAGTATTTTTGAGTGAATTCT

GCAAAGTGTGTCATCTTTGTTGGCATGACCGCGTGGCTGCCTGTTTCCCCGTCTGTATCT

ACTTTTTGTGTGCGTATGTGTACATGTGCTTTTCTTCACTCCT

>Tb927.8.7970 | Trypanosoma brucei TREU927 | hypothetical protein | genomic | Tb927_08_v5.1 forward | (geneCodeEnd+0 to geneEnd+0) | length=463

AAGGAATAAATGAATTGGCAACCACACGTGCGTGTATATGTGTGTGTACGTGTATGTCTG

TGCATCTTGTGTCTGTGTTTTCTTTTTTATCCTTTTTTATTTTACACTGATGCACGCTGT

TGAGAGGATATATGTAAAACAACAACAACAAAAAAAAAAAAAGGAAGATAAGGCTAGCAG

CAAACGAACAAACAAACAAGACGGAATCTTTCACTTTCCACACTTACCAGAGTCTCCGTG

TTTATCTCTATTTTGTGTATGTCTTACGTCCGTATGCCACTTTTCGGATTCTTGTTTTAC

GCTTCACTCACTCACGTCGCTTGCTTCTTTTTTTTTTTCTTCTTTTCCGTTCCTTTCGCT

GCTTTCCCGTTTTTGTTTTTAAAAATCTTACTTGTGTTTCTTTTTCCTGACTTTTTCCCG

TTTGCAGATTTTGCCGTATTGTTTTTATCCCCCAAGAATGAGG

>Tb927.11.2190 | Trypanosoma brucei TREU927 | Domain of unknown function (DUF543), putative | genomic | Tb927_11_v5.1 forward | (geneCodeEnd+0 to geneEnd+0) | length=464

AGAAAAGTGACGTATGAACGTTTCACATCCGAGTTTTGTTTGTAACTCCGATGTGTCGGA

CGCCAAACAGACTTATGGGGGTAATGCAAGGCGAAGGTCAGTGTGAAGAGGCCATTGAGC

AGGGGAGGGGGACGCACATGTTGAGTGGTGTGTGCGTGTGTGTGGAGTGAAGTAAAGGAA

TGCATAAACGGAAGAATATGATTCCCACCAACAATGCGCTCCAGAATGTTTTTTTGTTTT

TGCGTGTGTGTGACAATGAACGTGCCGAGTGGCATGGGGGTGTTTTACTTTTTAGTCCGC

GCGAAGGCAACTACCACCGCGAACAGCAATAGTCTCACCCCTCCTTTTCATTTTGTTACT

GTTCTTTGGTGTCGTTCCTGTGTGACCGATGCCTGCCTTGTATGCCGGTAATGTACACCG

CAGAGGGGGATGCAAAATGTGTTTTGACATTCTGTTAGTTAGGG

>Tb927.11.11120 | Trypanosoma brucei TREU927 | hypothetical protein, conserved | genomic | Tb927_11_v5.1 forward | (geneCodeEnd+0 to geneEnd+0) | length=465

GTGTCTGCGGAGGTGTGAGGCTTCTTTTTTTTTTTTTTTTGCCAGATAGTTGTTCTTTGC

CTGTATTCACTGGTGCGTACCCTCATTTCTTATAGTGACCCCCCTGTGGTATGGCCGTTG

TGCTGACGGTACCGCAAAACGAATTTGAAACGGCGTCACATAAAAGATACCGGGGACTGG

GGTGTTGTTGAGGTCCGGTGTTGGCTTAAGAGGGATCCGACCAATGGCGTCCTCTTTTTT

TTTTTTCTTTATTCATGGAGGAGATTACGTTCTGGTGTGCCGGTTGACGGTGTTTTGATG

TGGTGCTGTAGGTGGCGCACAGCGCTGCGTAGTTAGCGTTCGGTGGGTTGTAAGTAGTCC

ATGGATAAACTGTTGCGGTGTAGCATTGGGAAATGGAGGGCCTAAGCAGATGGCACCCTC

TAGGACTCATTAGTCCGTGGCCTTAACCGCTCTTCTTGCTCTCGT

>Tb927.10.11890 | Trypanosoma brucei TREU927 | hypothetical protein, conserved | genomic | Tb927_10_v5.1 forward | (geneCodeEnd+0 to geneEnd+0) | length=466

AGTAAAGGTGATGGTGATTTATTGTGAAAACAGAGACAATGCCGGTAGTGTGCGCAGAGG

AAGGCACACAAGAGGGGAGAAAAACTGGGCAAGGGGAAGTCATCTCATTTATCGTTTGTA

GTTGTTAATCAGCTTGCATTTGCTTTGTTACGCATGCAGTTGCATCCGGGAATCCTCCTC

CTGAGTCAAATGCAGTCAAGCAGGTACACACGGGGCAGCGAAGGTAATAGGGTGAAAATT

GTAACTGCCATCGTCTTTTTGTTGCTTACCATCATTTTTTTTTTTGTGGATGGAGGTCTG

CACTACTTCATGAGTGTTTGCACTGGCTCACATTGGCAAAAGGATGATTTTCCTTCCAAT

GCTTTCATTCTTTCCCTTTGAATCATCTCCTTCATTTGAGATCATCATTCCCCTTTCTTT

TACCGCTCCCTACACGCTCGTCCCACTGCCATCACTCCATATTCTG

>Tb927.8.5540 | Trypanosoma brucei TREU927 | Iron-sulfur assembly protein 1 (Isa1) | genomic | Tb927_08_v5.1 forward | (geneCodeEnd+0 to geneEnd+0) | length=466

AGCACAGTGTTACGTGTTACTTTTCTCACCCTCTCCTTGTTACCGTGTTTTTGCGTGTTG

GCCCGCGGTGTGCTGCCGACTGGTTCTTCCATGATGTGCATTGTTTCTTCTTTTCCTCTG

GTTGGTGTATCGTCTTTCTTTTGCGTCTTTGTACCAATTTTCTCATCTCTCCAGTTGAAG

TACAATTGCCACAGTGGCTCCCCCCAATGCTCGATTTTTTTTGTTTTATATATTTATTTG

TCATTCTTTACTTTTCGCTTCACGCGTCATTGTCAACAGCATACTTTTTTGTTCATTCAC

GGTCATGCAACCTTCTGGTTGTACAAATGTGGCGTCGTGAATAAGTAAATGTGTTACACC

TGCGCCCACTTTGGGTGTACGCGACGGGACTGCAGCACCTGTAGACGGATACAGGTGAGG

GAAAGAAGAGGCTATGAAGTGGAAGAGTTGACGTCCAAGTGTGTTT

>Tb927.10.13630 | Trypanosoma brucei TREU927 | glucosidase, putative | genomic | Tb927_10_v5.1 reverse | (geneCodeEnd+0 to geneEnd+0) | length=467

GGATGGCGAGGCCGGCGGGTTGCTGCCACATTTCTCTGGGTCTGATCTTGTTGCTCATGC

ACATCGGCTCTACACTCATCACTCTTTTCGTTTACCACCTCTGAACAGCAACAATGAAAG

GGGAGGTAGTTCGTCTGGGAAAGTGGTAGGCGTCGTGTTCTCATTCTATTTTACGTGACA

GCATGTCCTGTTAATCATACAGCAGCATACACTACTGACCCTATTTGAAGATACGTCGAG

GAGGAAAAAGTGAAAGGAAGCGGGAACACTATGCATCTAATCTCTTACCGGTATGAACCT

AGAAAACACTAAGGTCATAAATTCTTTGTGAATAATTGAACATGGGAAGGCCATGGGAGG

AGCAATAAACTCGCATGCACCAATTCTGAGAAGATGGGTGTGTTGGAGTCAGAGGGGGAC

GACAACGAATCGCCGTTGAAGAAGATGAGCCTAAGTATTTCAACGTC

>Tb927.10.12090 | Trypanosoma brucei TREU927 | RNA-binding protein, putative (RBP7A) | genomic | Tb927_10_v5.1 forward | (geneCodeEnd+0 to geneEnd+0) | length=469

ACTAGCTGAGGGAGTAGCTTATATTGTTAATATATGAATAGCAGTTGAGATATACCGCTA

ATGACTATGTACTTGGTTCTGGAATTTGACGATATATTTTCCCAATTGTAGCGCTTCTGC

TTCTGTGGTTGGATTTCTTATCTTATATTTGCGTGATATCACTATCTTAATCGTGAGGAG

GGCTAATCTTCCGGCGTTGAGATGGGTACTTGTGTTATCGATGCATGCTGCGGTTACTCC

TCACCAGTTGCTAACAAATAACACCGCGGAACAGAAACAATGATCGACCTTTTTCTTTCC

TCCTGTACATGGGAATTAGACTGCATGTAATGGGTTTAATGTTGAATCTTGTACGTGTCT

CTGTGTAGGAGGGCGGTGTTCGAAAGAGATTTGCTCCTCTAACAACACATATATGTGTTT

TTCTCGTTCTAAAATCTTTCTGTTGTGGTCTTTTCCACACCCCTGCGTT

>Tb927.7.5580 | Trypanosoma brucei TREU927 | cell cycle associated protein MOB1-B (MOB1B) | genomic | Tb927_07_v5.1 forward | (geneCodeEnd+0 to geneEnd+0) | length=469

AACAATTTGTGTGCGCGCCCTTGTGTTGGTGATTTTATTTCAATCTGCAGTGCTTCTCGA

GTGCGCTCCGAAGGTTGTTTACATCATCGAAGAGGGTCTCTGTGTTTTCGTTCCCTGTAT

TTCATGTTTATTCTGTGGTGATATAGGTTAAACATAGTGCTGGGCATTATTCTAAGAGGA

CGAGCTGTAGGTGACGTATATTGTAGCGGTTCCCGTGTGCTGTTTGGTGAGAAGGCAGTT

CCGATACATGCATACTATATGCATACATGTATTGGACTGCTGGCCGTCGTTGACGGGATC

CGTACGGGTTCCTTGTAACGATTACTTGAAGTGACTTAAATTCTCGTTAACAATTGGCGT

GTGTGGAGGAAGAGGGGGGAACGATGATATGCAGTGAGCCCGTGGCCCGTTCCGTTTGTG

TGGTGGCAGGACCTGTGCGTGTGCGTGTGTGTTGTATTCGGTGGACAGT

>Tb927.10.14790 | Trypanosoma brucei TREU927 | metallo-peptidase, Clan MG, Family M24 | genomic | Tb927_10_v5.1 reverse | (geneCodeEnd+0 to geneEnd+0) | length=470

GTGAAACGCCGTGTGTGTGTGTGTGATAAGACATCGCAGTGTCTTTTTTTTTTTTGTTAA

GGAAAGTATGAGAGAAACTGGGGAGCGAGAAAGATAGCTTTGATTCAGGAGCGGCTAGAA

GACGGATGAAGTACGCCGCTAAGCTACTGCCTTGCCCTTTTCCTCCCTTTCTTTTCCCTC

ATTTAGTCCACGCGAAAGGTCGATGGGACCGGAGTGCATGAACGGTGGGGAAGGGATTGT

AGAGGCGGAGGGAGGCGGTGTTGATGATGGTGGCATAGCAGAGCCTGGGATGGAGCGGGA

TGAGGATAGGAGTGCCACTGGTGCGACAAAGGAGGTGTTACGTACAATCGCCGGAACAAA

GAAAAAAAAGGGTAAATATAAGTAAAGAAAAGATATAATAAGGATAAAATGCCCGCGCGT

TTCTTAACAGCAATTTATTTTAAGGGAACTTCGTGAGAATCGCCTTTTCT

>Tb927.11.11010 | Trypanosoma brucei TREU927 | hypothetical protein, conserved | genomic | Tb927_11_v5.1 forward | (geneCodeEnd+0 to geneEnd+0) | length=470

AGTGAGCCCGGAGGGGCACATGCAGATGTACTGCCCTGGCCAAGCCTCCCACGCGCGTGT

TCCCCTTACGTTGTGGTCCCAGTGACCCCTTGTTTTCACTCACTTCCCCCCTCTGATGTT

TGTTCTTTTTTCGTTTGTTTGGTGTACTTTTCGTTCACACATATGTATTTACTATAACAC

AGGTGCGGAAACCCCCAAAGGAGGGGAAGGGGTTGATTCTCGGGGCTTCGTGCAACTACG

CAAACTAACGACGTGTCACCTCCTGCTCAATTCCTTCGCAACTTTTCACTCGACAAGGAA

AGACGGGCACGGTAACGCTTTTTTGATGGGCGAGACGTGTGGGCTAAAACTTGAAGATTG

AAAGGGGAAAGCAAGTTTATCTCCCAACTAAATGCAACTTGCCCCCAATGAATGCGTATG

TAGAAATTGGAGAAAGTGGCTAGAGGAAGAGTGAGAAAGGGGAATGAAGC

>Tb927.6.4500 | Trypanosoma brucei TREU927 | conserved repeat domain containing protein, putative | genomic | Tb927_06_v5.1 reverse | (geneCodeEnd+0 to geneEnd+0) | length=471

AAATAATTTGTCATTCTGACAGGTGCGATCTGCGCTTGTGAAGGACGACGCATTCAATTA

GGGTGCGCTCCACTTTGAAAGAGGCAATGTGACGGCCTGTACAGGGGGTGAAAGGAGGAA

TCGAGTAGGCAAGAAGGAAGTGAGGGTGAGGCCAAAAGGCATATAATGGATGGTCAGCTG

GCGCTTGTTCACTCATGTAACACGTGTCGTAGATCGTTCCTTGTATTGTGAGGAATTAAA

CGTAACCGAGGGTGCCTGATTCTTGCTATTTAAAGATAGAGTGTGTGATCAATGAACAGA

GCAAAAGAGGGGAAGACAAAGACTACGGTGCTGGTCAAGAAATGTCTTGCAAGTAACGTG

TTGATTTTAATGTGGAACAGCCTGATATCTGACAATGCGTAACCCTCTTGTGCGTTTATA

CCCTCCGTTAATATCTGCCACCTCTGCCTTTTTGCATGTGTTCAGCGTATG

>Tb927.10.12260 | Trypanosoma brucei TREU927 | cytosolic nonspecific dipeptidase, putative | genomic | Tb927_10_v5.1 forward | (geneCodeEnd+0 to geneEnd+0) | length=473

GTAGCAAATTGTCTTCAACGCTACCTCTCTCCTTCACCGGAGCGTTTGTTTATAATTTAC

CTTTCAAGGAAACCCCCACTGGCGCCATTACATATTGCACTAGTGGTATTATCGGTACCG

AGTGCTGAGCAGCGGAGTTTTTAACGTTCTGAGCAGAATCAGCCATGTAAGCCTTCAGGA

GGTCTAGCAGACAAAAAAACATGTCTTTCTTTTCCTTATTTGTTTTACGCATTTTTTTTA

TTTTGAAAATTTTCCGATGCGTTATGACTTATCTATTTTCTTTTTACATTTATATTTTGT

ATTTAGCGTTTTGGCACAAACACCAATTATTTTTGCGATGTTTGAGAGCGTAACATTTTC

CCCGCGTGCAATTACCACTGCCAGGCAATGGCGGTCGCAGGGGAGGGAAATGGAGTAGGG

GAAACATTTGCATTTCGGTCAGATAACATTTTCTAGACCGTCAGGTATTTTTC

>Tb927.2.4210 | Trypanosoma brucei TREU927 | glycosomal phosphoenolpyruvate carboxykinase (PEPCK) | genomic | Tb927_02_v5.1 reverse | (geneCodeEnd+0 to geneEnd+0) | length=474

ATATGTTAGGGGTATTGGCGTGTGCGGGTGTGTTCACCTACTCGATGTTTGAGGAGAAAT

ATGAATTGCACTAGCGTTTAAGTTGGGACATCGTTATTGACAAAGAACCAGTGCGTTAAT

CGTTTCGGGAGAGGAAGTGAAACTAATGTGATGTGCTGAAAGCGCAGCGGAGTAAAAAGG

AAACAACAAGGAAAAAATTATTAAAGTTTCCCTCTCTTTTCTCATTCTTTCCCCCCCCAT

TAACGACTACTCTTATGTATATCCGTATCAGTTAGTTGTTTTGTTCTATTTAAACTTTGT

TTGTTTGTTTGTTTGTTGTTGTTTTCTTTTTGTCATCGAGGGCAACTATTTTAGCGAGTG

CCAGTTGTATGCCAAGGAGTATTGAGCCCAACTGCGCGAGGGGGAGGGAATGGAAATGAG

CACATTAGATATTTATTGTGTTCCGTGCTCGGGCACGTTTCTCCAAAGAAGATT

>Tb927.10.4960 | Trypanosoma brucei TREU927 | hypothetical protein | genomic | Tb927_10_v5.1 reverse | (geneCodeEnd+0 to geneEnd+0) | length=474

ACCGCTTTTGCTCATTTTCTGGTTGCTATTGGTCGTGGGGATTTGTTGTGCAGGACGCGC

TTTGTGCGGGGGTTCCACCCATATGTTGATTCGTAAGGTGGTATAGAGCGGTATAGCATG

TGTATGTATGTAATGTTTGATGTGAGAAGCAATCAAATGGACTTTTTTTTTGCAACCTTG

TTCCTAGTTGTCTGTTGATTCCAGTGACATCCTACTGTTTATGTCTTTGTGAAGTGTGTC

TGTAAACAGGCTTATGGAGCAGTTTTTCTTTATTCTTCAGGACGATTGTGCGTGGGCATA

CTTCACCCATATGTCGTTGCTGCTCGCAGCGCGACACGGAGGCCCCGGACTCGCGTGGAC

CGCGATGACACTCCACGAAGGTGGTGTTGTGCGATAGGGTTTTGTTTTTGGCATCTATGT

TTCATAGCACTTGGTGGTTTTCGTACGCCTCTTTTCCCTCTCTGGCTTGCCCTG

>Tb927.11.4290 | Trypanosoma brucei TREU927 | 40S ribosomal protein S12 (RPS12) | genomic | Tb927_11_v5.1 reverse | (geneCodeEnd+0 to geneEnd+0) | length=475

ATGTAGTGAACTGTCGCGGCAGCCACATCAGCACTGGAGTTTGTAGGAGTTTGTGGCACA

TGAGTGAAGGAGAGAGTACCTCGGGAGTACGGGGGCGGGAGCTTCATGTTTGTGTTTGTA

TTGTTGAGGTGGTGGAGCATGTTGGAGAAAACGTTTTCCTATGTCCTCCATTACAGCTCG

TTCCATTTGGACGTGCCCCTCTTCTGAGCTTATTGATATTTTTCGTTGTTTAAACAATGA

AATTGATACTCCCTTTTTCCTTGTTGAACCCTCGATTCCTCTGTGATTTTGCTCTTCTTA

CTTGTCTGCCGACCTTTCTCTTTGAGGACGTCGATCTGTGGGAGGGAGACCATTTCTCTC

TTAAAATGGTTTACTTTATTATTTTATTCAGTGATATAAACGAAAAAGAAAACTACAACA

ACAAAAAACCAAAAGCTATCAGGAGTGACAAGGGTCTACGATGCATTACTTCAGT

>Tb927.7.2590 | Trypanosoma brucei TREU927 | prefoldin, putative | genomic | Tb927_07_v5.1 reverse | (geneCodeEnd+0 to geneEnd+0) | length=477

GTGGCACTCATCCTGTGTTGCCGGTGCCGGTTTTGTCTCCTCCTCGCCTCGCGCTCCGCC

TGTTTGTCATTACTGTTTTGTTATTGTCCGCATGGCGAGAAGAGACATAAGCGGGGGATA

ATATAAGCGTGTTAAAAAAGCAGTTAAGGGGGAGATGACCAAAGGTTAAGGGATACTTCA

GTGTTTAGTTGGGTTGGACTAGCATGTGTGGATGCTGTTATCACAACCTTTCATGCGGGA

ATGGGGTAGGGGACAAACCGAGGGTTGAGGGTGGCCGGAAAGCCGGGAGGAGGTGGGGGT

CGTTTCTCATAAATATTTCTGTATTTATGTTTTGACTTCCGGGTTTTTTTTTACGATCTT

TCCACTTCTTTGAGTACCTGAGAAAGGTAAAGAAGAGAGGCCAATGAATGTGCAACCCGC

AGTGGAATCTTTCTTTCTCCATACTACTACGACTCTGCACACATTTATTATCTCTTT

>Tb927.11.10100 | Trypanosoma brucei TREU927 | hypothetical protein, conserved | genomic | Tb927_11_v5.1 forward | (geneCodeEnd+0 to geneEnd+0) | length=478

AAGGCCGTTGGAAGTACGGTCCCGGGGTTCAGTTACCGTAGGTTCCACCTTTTCGCCAGT

GTTGTTTTATTCCTCTAACTCATAGCTGTGACCCTTCCACTGCTGATCCCATGATCTGAA

CCGTACGTATGGTGTTCTGTTGCTTGTGTAAGTAGAGGTAGTGGTAGCTGAAGTTTCTGC

GTTTGTGTATTGTCATTGTTTAGAATCGTCACGACTCCCACACATTTGGCGATTTGCTCC

ATACTTCGAAGAGGATGAAGAGAGGGGATAGCAGAGGGGGGGGGGGAAAGCGTGGAAGAA

GTCCAAAATCGCATCCGTATGTTTGCTGCGTAGTGAGAAGATCCGCCATTTAACCTGAGG

TTCGCACAAGGGTGCATTCGTGTATTAATGGTTGAGCTCAAATTGTGCACGACACTCCTC

ATCAGAGTCGTAGTTTCAGAATCGTCAATGGAAGCCGTTCGTTTGCTTGAACGAGAGG

>Tb927.7.2380 | Trypanosoma brucei TREU927 | hypothetical protein, conserved | genomic | Tb927_07_v5.1 reverse | (geneCodeEnd+0 to geneEnd+0) | length=479

AAGTGATACCCATTGCTTAACATTTTTTCCCCGTATCCGCGATACGAATACTGCATTGCC

GCTGGAATCTTTGGGTGCTGGGTATGCCTTGTGTTGTTCACTGCGCATCTGCGTGTGTTG

AAACGACATGCGGTGAGGCTGGTAGCACATACCCATGCCTCTGTGTAACACACCTACGTG

AGACACGATACATCAGAGGTTCTCATCATTCGGTGATGTTGTGCGAAGAGGTGTGCGCAC

CAGGTGAGTTGAAAGCACTGCAAGAAAACAGAGTGATGGGGACCCTAACTCCATGCAGCA

GTGTTTTGAAGCTAATTGATGTGTTGTGGGTAACATTAGTTAGAACTAAATGATAATGCA

CGGAATGGGAAGTAAACACGAAGATATTGAGATGCATACTTGCACCTGCCTTCGTTGCAA

ATAAAGGATGGCAAAGCACATATCGAGTTCCAGATGGCAGTATCCACAGAGTTTTAGTG

>Tb927.10.15230 | Trypanosoma brucei TREU927 | hypothetical protein, conserved | genomic | Tb927_10_v5.1 forward | (geneCodeEnd+0 to geneEnd+0) | length=479

GAAATATTTCACTGGGGAAAGTAAAAAGGGCGAAAACCCTTCCTTCTTTGATGGTCCCCC

CGTTCCTACTTTTAACTTTTAATTCCACGCTAGCATTTCCATTTCCGGAAGTGTCCTCGG

CCCCTGTTATTAGCACCGAGTGAAATTCAGGATAGGCAAGGAGTGTGCAGCGGTGACAAT

CGAGAACAACGAGGATGGGATGAACATGACGGGCATGCGGATCCGCGAAAATTGCTTATT

TATTCTTCTGTTTTCTAAGTCGGACTAGTGATTGAATCGAGGAGGGAAGAAAGGAAAAAA

GGGGGACGAGGGGAACCTACTTTTTTAGTGAGGTGACGATTCCATGGTACTGTTTTTTTT

TTCCTGTATTCATAGCGGTTCTAGAGCCCCTCCAACGGGGATACCAGTTCACAGCGGTTG

GGGTGGTCTAAGAGAGACCAAGCGCGTGTGCATTGATGCTTGTTTGTGTAACATACAGT

>Tb927.7.6600 | Trypanosoma brucei TREU927 | hypothetical protein, conserved | genomic | Tb927_07_v5.1 reverse | (geneCodeEnd+0 to geneEnd+0) | length=481

GAGCATGAGACAAGTTGAATGTAAGAAGGAGTCAACGGATTTTATTTGATTGGGAACACG

ACGATGGAAGATGTTGCTTCGTCTTGCTCAAGGCTACGTAGGTTGAGATGCTGCTTCGAT

GATAACAAGTTATTCAGCTGCGTGCTGTGGTTTTCCTTTATTGCTTTATAACATTAATAT

GCTTTACTTTTTGTTCTTTTTATTGAAGATATGAACAGATCGGCGACGTGCTCCGGCTTC

CGGATAAGCATGGGACATTTGGGTAGTGGAATGATGTATAGAAACTGCTTTAAATAGCGG

TGTGTGCGTGTGTGTGTGGACGGACGGATGGTTGATGTTAGCATGTTCCAACGTACCAAC

GTGTGACGGAGGGCATGCAACAATATGAATATCTACGTAATCGCATGCTAAGTAATGCTT

TGCTTGAAGTGACTTTTTGTCCCTTTTACAGTGTTGTGAGTGCTTTTAAATACCCAACGT

G

>Tb927.10.9870 | Trypanosoma brucei TREU927 | Protein of unknown function (DUF2870), putative | genomic | Tb927_10_v5.1 forward | (geneCodeEnd+0 to geneEnd+0) | length=483

AAATGTGAAGCAAGTACGTTTTTCTCCCCACGGGCACCTCGTATATTATTTCCATTATTA

TTATTATTGTTCCCAATGGTCTGCTCTTTCTTTTTTTTTTTTGTCCCCTACACTGCGAGG

TAGTGTGCTCGCATCTACGTATGCCTCCTCGTGAATGCAACGAAACAACATGAGAATGAA

CGAGGAGAGTGATAAACTTCATGTTAGATTGTTATAGAAACTTCTCAAGTGCGTGCGTGG

GGAGTACGTGTTGTACCTTGTCTATGCGCCACTGCTGTTGCGTTTTGTTCTCAGATCCCT

TATCCCAATATTTTCGCTGTTTGCACCCGCCGGTTTACATCGATGGGTCCGAGCACTGTG

TTTGTCGGTGCTCACCGCATGTGAAATACTTTCTCCTGCAGAGCGGATGCAACGCGTACT

CACACGCACAGGGTTATACATATATACACATATGCATATTTCCGTAGTGATAATAGCAAA

CAT

>Tb927.11.14310 | Trypanosoma brucei TREU927 | small nuclear ribonucleoprotein Sm-G (Sm-G) | genomic | Tb927_11_v5.1 forward | (geneCodeEnd+0 to geneEnd+0) | length=485

ATGGCAAATATATTTGCGAGTTCAAATGGTTGCCCTTTCTTTGTGCGTGTTTGGGGCGGG

GTTTGGTGTTTGGGGAACGATTATTCGTGTGTTTGTGTTTATGTTTGGCGAGGGGATGGG

GGTTAGACACAATGTGTGGAAGGAAGAAACTAAAGCAAGCAAATCGTGCGGTACTTCAGG

GGATTTTGAATGGTCACCGGCGCTTCAGTTTGATTTAGTGGAGTTGGTCCGCATTACCTT

TGGGGTTTCGCTAGATATGGGGTCTGTAGACCCATAAACAAGGATCAGCAGCGGTGGGTG

GAGTTGTTCCGAGTATACAAAAATGAAACGAAAACTCTGGTGTGCTGCACGACAAAAACG

TTCAGACTTGTTTTATCTTGCCGTCTTTTGCCTGGTGCGTTCCATCGTTTCAACGAGATG

GTCTTTTATAATCAATCGAATGGCTTCCACTACCGTAACCAAACACCTTTCCTTACACCA

CTGTT

>Tb927.9.5800 | Trypanosoma brucei TREU927 | hypothetical protein, conserved | genomic | Tb927_09_v5.1 forward | (geneCodeEnd+0 to geneEnd+0) | length=487

ACACCTCAGGTGCACAACATATATATATATATATATGTTTGTGCTAGTGTTACCGTGTTC

ATCAAGTTTATGTGCATGCAATTTGGTTAGTGGTTTACTTAGGAAGGGGACAACATTTTG

TACGCAACGTAAGTCGCTTCACTCCTTTGTGAGGGCCAAACCCCCACTCAGGATCTCGCA

ATGGCGTAAGTGAATAAAAACTGGCAATAACCGCTCTTGTTTAATTTTACTCTGATTCGC

ATCTGGACTTCCTCCCCTCTGCACCGTTTCTCTTATGACCGAGAGGTATGTGGTCAAGCA

CACAATTTTGTTAGCGCCATGTCCACAGTTACATTCTGTGTCCCGTATTTCTCCTTGAAC

TTCTGCTACTTTCCTCCTCTTGTGTTTTGGTACAGAAAAAGCAACTCATAGCTAGCAAGT

CAGTTTTTCACCAGCTAACTTCAAGTATTCGGCATATACCCATATCAAGGTAAAAAGGGA

GAAAAAG

>Tb927.9.9290 | Trypanosoma brucei TREU927 | Polyadenylate-binding protein 1 (Poly(A)-binding protein 1) (PABP 1) (PABP1) | genomic | Tb927_09_v5.1 reverse | (geneCodeEnd+0 to geneEnd+0) | length=489

AAGTTGATTTTGTGAGTGAAAGTAATATATGAAACGAATAAGCAAAAACAAAAGGTAAAA

GAGAGATGTGATAATCTGATTAAATGAAGTCGATATGAAACGTGCTCGGGGCTGTAGCGA

TTCAAAAAGCATTCCCTGTGTTATTAGTAGCGGACCGATAATTTATACACTGATGTTTTA

TCCCTCAATCACGTTGCCTAGAGCAGGGGAGATATTTGAGAGGAGGAAGAGGGATGGGCA

GAAGTGACTCGAAGGGAAGCGAAAAAGGAGAGAGAGAAACACTACCGAGAGGTAGTGAAG

AGGGAGAAATAATGCAAAGAGAGGTAGGGAGATTGGAGTACTACTGCTAAGTGGTACAGA

TAGCAAAGTTTGAACCCCCATCTTCATTTTAGCTTACGCTTCTTCCATGTTTCTCAAGCC

CCTTCTTTTCTTCACTTCAATTGCGCGTTTTATCGGTCCGCATTGTAAGTCGTGAAGTAT

CCTACTTGT

>Tb927.11.1330 | Trypanosoma brucei TREU927 | Stress responsive A/B Barrel Domain, putative | genomic | Tb927_11_v5.1 forward | (geneCodeEnd+0 to geneEnd+0) | length=489

ATAATTTGTCCCTTAGTGGTGCCAGGCATGAGGAAGAAGAGGGAGTGAAGGCACAAAAGG

CAAAACGAAACACAACACAACAAAGGGTTTTGTCCCCACTTCTGAGACACGAAATGCAGC

CTGCTTCTTCATTCATTCAATCACCTTAACTACATATATATATATGTGTTTGTGTGTGTG

TGTGTGTGTGTGTGTAGGTTTTCTTCCTTCAGCTCTTCATTTTATTTTGCAGGCGTTTAT

CGCGGTCATTTCTTCCCCCTCCCCTTTTCATCCTCCGGTGTGCGGCGTAAAAGGCAAGTA

AGCTGCAAGGAATGCAATTATCAACCGCTAACTTAAAGTGTTGGAGTGAGCCCGAGTGGT

TTCGTTTTGATATTGTTGGGGCGTAATGTTTGCGGTTGGTAATGAATAAAATGAAGTGAA

TGGGAAGAAGGTTTTATTATTTGAATTCCAGAGGGGATGGAGGAATAAATTATGGTTTTG

CTCATTTTC

>Tb927.7.760 | Trypanosoma brucei TREU927 | hypothetical protein, conserved | genomic | Tb927_07_v5.1 reverse | (geneCodeEnd+0 to geneEnd+0) | length=489

AAGCCGCTTCTGTTCAAAGGCCATGTTTGTGTTCGAGTATCGATTTGGTTAGCTCACCGC

CATTTTCCCTTTCATTTTTCATTTTTAAACTTCTAGAGGTTCGCTGCGAGGTGTCCGACA

GGTCAAGCGGTGAAAAAGACATGTGAATGGCGATAAATTGAATGAGATGAATGTACTTAG

AAAAAGCTACTTGTACTTTTCCCCAACCCCTTTCTCCTATGTTTGGGGATCGTGGCGGGG

TTCATTATGTAGATAAGAGGGGAGCCGGGAATGCAATGCGGATTAAGTTTGTAATGGGTA

AAAGGAGAGTAAGGGCAGAAGCGGACGAGGATAATATTTGTCTGCAAGGATTTCACTTTT

CTTCTTTTCGTCCCTTCATTCCCGTTTGTGATTTTCTCTTAGTTGCCTGTGTGGACTTGT

CCCATTGCCCTCACGATCGCCCGATCTTGTTTTGTATGCAATGCATATTGCATGATTTAT

AAAACAAAG

>Tb927.10.8790 | Trypanosoma brucei TREU927 | OTU-like cysteine protease, putative | genomic | Tb927_10_v5.1 forward | (geneCodeEnd+0 to geneEnd+0) | length=490

ATCTGGTATCCGTTTAGTGCTTTCACACTGACATTATATTTTTGTATTACCTAACTCTAT

ATGCGGTCTGTGAAGTTATGTGAACTCATTTGCAGCATGGCCGGTTAGATGTGCGTGGTT

TGATCGTGTTGAGGAGGGCACCACTTCCAAACAACACTGCGAAAGGCCTATCGTTTTGTC

ACACCTGTACCTTAACATGTTGTTTGTGTGTAGTGGTGCGGCGTGGGATTGTTTCGTTGG

CACGTTTTTTCCCCCATATTCCAGTAGGGAATGATGCAGTGGAGTGGGCGGTTTGGTCTA

ACATGTAAGTGGGGTTTGGATGAGTGCCGTGATGCTGATGCATGATCTGCCAATAACCCT

CCAGCAGATGTAGGGGACTACCGCGTGGAATTGGTGTTGCCATTTCACGTGTGAACTCGT

CTCATATTTTGCCTTGGTTGTCGTACGGTTGTGCCCTCTCCATCCCCTCTTGTTTGTTTG

TCTTACACTC

>Tb927.10.6880 | Trypanosoma brucei TREU927 | glyceraldehyde 3-phosphate dehydrogenase, cytosolic (GAP) | genomic | Tb927_10_v5.1 forward | (geneCodeEnd+0 to geneEnd+0) | length=493

AGAAAGTGGGCTTTTAGCCACTTCTTTTCTTTTTTGTTGCGTTTTACCCCCTACTCATCA

AGGTAAAATGCTCTCGCTTCAAACATTCAACCCGAAAGCAACCACGACGAGTGCCAATAG

GAGATTGGCCGGGGGAAGCAGGAGGTACTTATATCTTTTTCAATTCAACAAATTTATGGG

GATATAACGCGCAATTTGTGGAGTTCTGTGACACAATTAAAAGGGGTCAGTTTTTTTTGT

TTTTCTCTTGCATCAAACAATACCCTCCAGGAAAATTAAAGCGGCGGGAGATTATAGCTA

AGTGACTTCACAGAGGCACCAAAGGATGCCGTTAGCACAAAAAAACAGATTTCATATGTA

CTACATGTCACACGAAAAAAAAAGAGAGTTAATTTCAGGAGTAGTATACTAAAAGAGCAG

CCAAAAAACCAGAATAAAATGAGGAAACTTTTGATACAAACACAAGAAAAAGTTTGTACG

CCATTATTTGCTT

>Tb927.7.1780 | Trypanosoma brucei TREU927 | Adenine phosphoribosyltransferase, putative | genomic | Tb927_07_v5.1 forward | (geneCodeEnd+0 to geneEnd+0) | length=494

AGGAATATATTGGGCAGGTAACGCCCGTAACGGATCCTTAATGATGTTACAGGTATTATT

TTTAATTTGGGTAACAGAAGAATATATATATATATATAAAAGAGAGATAATCGAGGCAGT

GACATTATTTATATGTGTGATATCCAACAGCGTTGGTGGTCCACACGGAAAAAGACAGCG

GAGAAGAATCAGTGAAAGGAAGAGACATGGCGTTGAGTGCTTTTTTTTTTTCCTTCTTAC

GGGATGGGTATTTAAAAGGTGGTGCCTCATTTTTTGCGAGCAGTAGGCCTTGTTTTGTGT

CAAGTGCAGCATTTAGCATATTAGTTTACACATGACTACGGGGCGTTCTGGGAATAAGAA

TTCTCCTCGGGTTGGCACACCTGTAAATTTATATGTCGTACCTCCCCTCTATTGGCGTAT

CAATTTTTTTTCTCATTGCCTCCTGCATCCCTTCCCGCGTGTTCTTTTTTTTTTTTGCTG

CTTTTCGGTATTTC

>Tb927.10.1060 | Trypanosoma brucei TREU927 | T-complex protein 1, delta subunit, putative (TCP-1-delta) | genomic | Tb927_10_v5.1 reverse | (geneCodeEnd+0 to geneEnd+0) | length=495

AAAGGAGACGGAGAGAGGAGATAGATAAAGTGGTGAGTGCATTGGTGGAAAGGAACCGTG

AGACGAGAGCGGAGAAGCGGGACAGCATGCCTGATATGATGCCGGTGGGAAATTCAAATT

CTCATCGATGTCGGTGAACACTTTGTTGGCATGTGTATGCGGGTGCAACAAGCCTCTCGA
[truncated: 436,598 more chars]
