# Supplementary material for: Expression of the RNA-binding protein RBP10 promotes the bloodstream-form differentiation state in Trypanosoma brucei
Source: PLoS Pathog. 2017 Aug 11;13(8):e1006560. doi: 10.1371/journal.ppat.1006560 (PMC5568443; doi:10.1371/journal.ppat.1006560)
Supplement: S3 Text — The motif is in red, non-coding regions in lower case and VSG coding regions in upper case. Translations are below. (DOCX) [file ppat.1006560.s003.docx]

>NODE_135_length_4788_cov_21504.1_g129_i0 Bloodstream form 2098

ttataataagagcagtaataatagtaataataatgatgataatgataataataagagagtgttgtgaatgtgtatatacaaatattataataagagcggtaataataataatagtaataataatagtcatgatagtaataataataataggacagtgttgtgagtgtgtgtatatacgaatattatagaaaaaatagtgaaaacaaaatggcctggtaaatagaagaacgtaaaacaatggtaagtggtatgggagaacaagtaagctggtgggacaatgattgtgatagaaagataccgtgaggcatattattaagggacacacagaaaggcaaggagaaaggaatatagtatatgtgcaacttcaaataaaaatatgactttgtgaatgatcatgcaaaaggagttgatgggggaaattatggagagagagagagaatccaatgaagaggccaaatcgggacattagtattgtgcagaagcccctagtttagtggttaagtagagtgctcgtcgcagatagtacaggaatagatccccacgagcgccgagtatttagcttacactaaaaatgaatctgcatcaacgtggcgccagggttcggccccagaaaagaaatcgactggaaagccaaatgttccattcatgctgcggtcgcaacacaggatatgatgggaccgctttgtggggagcaccattacttagtgttccaccacggttcaccaccctgctcaggtagaggaaccaaaaatccctgagacaaccttcttccggggaggctgatggatatggtaaacatggttcatacgcaacgtggggggaatcaagcgggcgtcaaaacgagaccgctccactagacatggacacatgccgtcgctcacccgtgggtatcaattctcaaagaaaaacaaaagggttttagtgggtaggaacgccggtctccagcgttgagtcacacacactaccgcgtcctcgtggcgtggtgtgcgcaaacgcacttcctccttgcccgcggcttgaccggttgcgtgctcctgacggttgcgcgagcaaaaatcaaaaaaaatgtgcagaagcaggcgaaagtcaccaaaagaatgaaagaaagtacagacgaataaatgcgcaccaatacagcgcataatggaatgatataaggggggattgtgacccagagctcacacagaaattgagagactctgctgaggatatgatagtagtagctgtggtttatagtctaatggcggcgactagtgttaaatagttagtgaaaggggtaacaattcatatggtgatagagtgcttttggttagtggaaaatagagccgatcacagggaaatgtgatgggttaaagctaaatataagtcagggtgttttaagggaatgtgaagttgaagtctagaaacagtggcgttaaactcgttatggatgcgcaagcgacagcaaaaggaagctaaagggtcttcaacaccgtgaaatctctggtacatacaactcgaactgcttctttttttaattctcaccgcaccagaagaagagctcacttcgtttgcgtggcggctcaaaaactcagtagaaaccgacagtagaccggatgcaaaaaaagctaggagccggtaacacaaccggagtcactgcggttgtcctgcttcttttaacgtcgaacaagccgatagcagatgcagtggccgcaggggaaaacgcagcatccttcgaggttctctgcgatctaatagctctcgcaaagtcgcaaccagcaggtccaaagctggagagtggcagtacgtcagcctacgaagagctactgaaactcaattcaacactaagcccgaaggaatggacagacaaattcgtcgacaacgctaacgcaaaaacataccaccaacctggcccacccaaaataaccgagaccgggaactggaaagaactttggccagactgggtcaaggcggtggaagcagtagcaaaggagaaaaacatggacgaaattaaaaaatgaacctagccaagttaaacccacgccaactggagactacgacactaatcgtaagacaacttgcagaagaagcacgagaattaagaaaggagcgagcaggcctagccgaatacctaaaactcgccgacggcgacacaatccggaagcagctaaacaaagcagcatttggcgatgagacaaccgaccaagtaacgccgacagccgccaaagtttttacctcggcaagttaaacttacactgaggcgtgcagctcaacaggcgcagctcagaaggcaacagcggtaaccgccgcagctgcatgcgtgtgcgttaaagacaactgggacagcgaagctcaatattgcggcaaatcagccaaagtgacgacaacatggacaagcaccggcgccaccatacctgaaacagcagtgacggagccagcggcgttctgcaaagcaaaggcagcgccgacgttaacatccgactcactacagtcgaaactggaggccgtaacgcggttaatacgagttagcacaaacgccgcaatacttggcagcagcgaagccggcacttgtactgggaaggcagcagcgggagtttgcgtgtccgtgaccgacggggccgaagagaaatccacaggggccagcacattcgcttggggcgcgacgttgagagctctagcagtcaacctccgtcgcaaagaggaagcggcgaaaaaacccaggcagcaacacaagacatctttcagcgaccggatagcatggcgatgtgccaagcgctgacaaaaaggaggagaggaaatcggcaagactgtgtttgaagttagtggttaaatgttgaaaattcgcttcggttaatgcaaaagacgtgcgcacagaaaaaaaagctgacggtgtcgatgcggcgcctgccaacaaaaatggtgttacgaaaattgcgcgacaaaataacacatggacgacagacaacaggccaagacaaagccattagaactggcacttaacaaattgcgcatcttatttgccaacaaaagaattgactcatacgctgtggcatgctatagaggaaaaaaatacagtctataattgtaaaagtaaaaaatgagaatacaagcgcgctcatcaccaaaccgcaatctcaatactaaatcatagaaacagaagccaaagagggagagccactcatttccacccctaatactggcaATGGTCACCAAGGAGCGAAACGCAGCATTAAAAATTGTAATGTTAGTCGCTTCAGCACTGACACTACACCCACAACAAGCTCTAGCTCAGACCGCTGGTAGGCCCCTTGCAGATGTGGTAGCCAAAACTCTATGTACTTATTCAAAAACGGCCAAACGCCAGGCAGCAAACCTGGCGCAAACACTACAACGAGCCAGCTCAGCAGCAAAGCAATCCAGACAAGCGCAGCAGTTAGCGGCTTTAGCACTGGCCAAACTGCCAGACTACAAAGAAGCAGCCGCGACACTGTTAATTTACGCCACGCACAAAATACAAGACGCGCAAGCCAGCATCGAAAACTGGACAGGAGAGAATACTAAGCTAGTTGGCCAGGCGATGTATTCCTCAGGGAGAATCGACGAACTGATGTTGCTACTAGAAGGGCACCGAGAGGACGGCGCGAACGGACAGGACAAAACTTGCCTAGGCGCGGCCGCCGGCGGCAATACAGTAAATGAATTCGTCAAAACAGAATGCGACACGGAAAGCGGCCACAACATCGAGGCAGACAACTCAAACATAGGGCAAGCGGCAACGACTCTAAGCCAAGAAAGTACAGACCCAGAAGCCAGCGGAGGCGCAAGCTGCAAAATAACAGCAAACCTTGCCACTGACTACGACAGCCATGCGAATGAGTTACCGCTACTCGGCGGCCTGCTAACCATACACAACGCAGGCGGCTTCAAAACAGGACAAAGCTTGCAAACCGCAGCACCAACCAACAAGCTAATCAGCGCACTCAAAAATAAGGGCGCCGGTGTCGCAGCTAAACTGGCAACTGTAACGTCGGCAGCACCTACAAGCAAGCAGGAACTCAAAACACTACTGGCTTCGAAAGGGGAACGCGCCAAACTCCAAGCAGCCAACGACGAGTATAATAACTGGAAACCAGGCGCCAAGCCTGAGGACTTCGACGCCCACATCAAGAAAGTGTTCGGCGCAGAAGACGGCAAAGACAGCGCCTATGCCATTGCACTTGAAGGAATATCCATTGAGGTTCCCCTCGGAGGAGGACAAACACAAAACAAACAACTCTATTCCATGCAGCCAAAAGACCTAATGGCAGCTTTAATAGGAACGATAGCAGAACTCCAAACAGCCGCAGCAACCAAACCAGCATGCCCAGGCCATAAACAAACAACCACGGAAAGTGACGCCCTATGCAGTAAAATAAAGGATGCAAACGAATGCAACAGCAAGCCTTACTGCAGTTATAACGAAACCGCAGCTTATGGCGACAAAAAGTGCCAATTTAATGAAACAAAGGCCTCAAAAAATGGAGTCCCTGTAACGCAAACTCAAACTGTAGAACCTTCAACCAATCCAGAAAAGTGCAAAGGGAAAGAAGAGAAAGACTGCAAATCCCCGAATTGTAAATGGGAGGGCGAAACTTGCAAAGATTCCTCTATTCTAGTAAACAAGAAATTCGCCCTCAGCGTGGTTTCTGCCGCATTTGTGGCCTTGCTTTTTTAAttttccccctctttttcttgctaaaaattcttgctacttgaaaaattttcTGATATATTTTAACacgcaagttacccgaaaaaaaaaaaaaaaaa

Antat1.1

MVTKERNAALKIVMLVASAL

TLHPQQALAQTAGRPLADVV

AKTLCTYSKTAKRQAANLAQ

TLQRASSAAKQSRQAQQLAA

LALAKLPDYKEAAATLLIYA

THKIQDAQASIENWTGENTK

LVGQAMYSSGRIDELMLLLE

GHREDGANGQDKTCLGAAAG

GNTVNEFVKTECDTESGHNI

EADNSNIGQAATTLSQESTD

PEASGGASCKITANLATDYD

SHANELPLLGGLLTIHNAGG

FKTGQSLQTAAPTNKLISAL

KNKGAGVAAKLATVTSAAPT

SKQELKTLLASKGERAKLQA

ANDEYNNWKPGAKPEDFDAH

IKKVFGAEDGKDSAYAIALE

GISIEVPLGGGQTQNKQLYS

MQPKDLMAALIGTIAELQTA

AATKPACPGHKQTTTESDAL

CSKIKDANECNSKPYCSYNE

TAAYGDKKCQFNETKASKNG

VPVTQTQTVEPSTNPEKCKG

KEEKDCKSPNCKWEGETCKD

SSILVNKKFALSVVSAAFVA

LLF*

>NODE_4548_length_1629_cov_47.6499_g4147_i1 PC2A

agagccactcatttccacccctaatactggcaATGGTCACCAAGGAGCGAAACGCAGCATTAAAAATTGTAATGTTAGTCGCTTCAGCACTGACACTACACCCACAACAAGCTCTAGCTCAGACCGCTGGTAGGCCCCTTGCAGATGTGGTAGCCAAAACTCTATGTACTTATTCAAAAACGGCCAAACGCCAGGCAGCAAACCTGGCGCAAACACTACAACGAGCCAGCTCAGCAGCAAAGCAATCCAGACAAGCGCAGCAGTTAGCGGCTTTAGCACTGGCCAAACTGCCAGACTACAAAGAAGCAGCCGCGACACTGTTAATTTACGCCACGCACAAAATACAAGACGCGCAAGCCAGCATCGAAAACTGGACAGGAGAGAATACTAAGCTAGTTGGCCAGGCGATGTATTCCTCAGGGAGAATCGACGAACTGATGTTGCTACTAGAAGGGCACCGAGAGGACGGCGCGAACGGACAGGACAAAACTTGCCTAGGCGCGGCCGCCGGCGGCAATACAGTAAATGAATTCGTCAAAACAGAATGCGACACGGAAAGCGGCCACAACATCGAGGCAGACAACTCAAACATAGGGCAAGCGGCAACGACTCTAAGCCAAGAAAGTACAGACCCAGAAGCCAGCGGAGGCGCAAGCTGCAAAATAACAGCAAACCTTGCCACTGACTACGACAGCCATGCGAATGAGTTACCGCTACTCGGCGGCCTGCTAACCATACACAACGCAGGCGGCTTCAAAACAGGACAAAGCTTGCAAACCGCAGCACCAACCAACAAGCTAATCAGCGCACTCAAAAATAAGGGCGCCGGTGTCGCAGCTAAACTGGCAACTGTAACGTCGGCAGCACCTACAAGCAAGCAGGAACTCAAAACACTACTGGCTTCGAAAGGGGAACGCGCCAAACTCCAAGCAGCCAACGACGAGTATAATAACTGGAAACCAGGCGCCAAGCCTGAGGACTTCGACGCCCACATCAAGAAAGTGTTCGGCGCAGAAGACGGCAAAGACAGCGCCTATGCCATTGCACTTGAAGGAATATCCATTGAGGTTCCCCTCGGAGGAGGACAAACACAAAACAAACAACTCTATTCCATGCAGCCAAAAGACCTAATGGCAGCTTTAATAGGAACGATAGCAGAACTCCAAACAGCCGCAGCAACCAAACCAGCATGCCCAGGCCATAAACAAACAACCACGGAAAGTGACGCCCTATGCAGTAAAATAAAGGATGCAAACGAATGCAACAGCAAGCCTTACTGCAGTTATAACGAAACCGCAGCTTATGGCGACAAAAAGTGCCAATTTAATGAAACAAAGGCCTCAAAAAATGGAGTCCCTGTAACGCAAACTCAAACTGTAGAACCTTCAACCAATCCAGAAAAGTGCAAAGGGAAAGAAGAGAAAGACTGCAAATCCCCGAATTGTAAATGGGAGGGCGAAACTTGCAAAGATTCCTCTATTCTAGTAAACAAGAAATTCGCCCTCAGCGTGGTTTCTGCCGCATTTGTGGCCTTGCTTTTTTAAttttccccctctttttcttgctaaaaattcttgctacttgaaaaattttcTGATATATTTTAACacgcaagttacccgaaaaaaa

Antat1.1

MVTKERNAALKIVMLVASAL

TLHPQQALAQTAGRPLADVV

AKTLCTYSKTAKRQAANLAQ

TLQRASSAAKQSRQAQQLAA

LALAKLPDYKEAAATLLIYA

THKIQDAQASIENWTGENTK

LVGQAMYSSGRIDELMLLLE

GHREDGANGQDKTCLGAAAG

GNTVNEFVKTECDTESGHNI

EADNSNIGQAATTLSQESTD

PEASGGASCKITANLATDYD

SHANELPLLGGLLTIHNAGG

FKTGQSLQTAAPTNKLISAL

KNKGAGVAAKLATVTSAAPT

SKQELKTLLASKGERAKLQA

ANDEYNNWKPGAKPEDFDAH

IKKVFGAEDGKDSAYAIALE

GISIEVPLGGGQTQNKQLYS

MQPKDLMAALIGTIAELQTA

AATKPACPGHKQTTTESDAL

CSKIKDANECNSKPYCSYNE

TAAYGDKKCQFNETKASKNG

VPVTQTQTVEPSTNPEKCKG

KEEKDCKSPNCKWEGETCKD

SSILVNKKFALSVVSAAFVA

LLF*

>NODE_4391_length_1666_cov_76.897_g4147_i0 _PC2A

tatattggccaagggagcaatcaagtaccaacgtgaaaaatgtgccatgcaaaaatgtcgttaataagcacatgcgtcacacttgcggcacttctactatttcaaataaatcgaggggaagcagcagcgcacaccggcATGATCAAATCAGCATGGGAACCCATCTGCGGTATCAGCGAAGAACTTGGCCAAGTAGCTGGCGAAGCGTGGGCGGCGGCGGACACGATTCTCGAGAATGTTCACAAGTTCGAGCTAGCGGCCCTAAGAGCGGCAGTTTACTACGCCAAAAACGTCGCTTCACCAGAAGCTAAGAAAGCAGCTTTACTGCAAAATTACTATACGAACAAACAAGTAAGTCTAATAAATCAATACCGCTCATCGGCGCTGCAATCCCACTTACGGGCGGCGCAAACAAGCAGTTATCTAAAAGGCAGAATAGACGATTATCTAAGGTTGCTGGAACAAACGACAGACAGCAACAACAATTGCTTACTCCAAACAACCAGTGCGGAAACACCTGCTGCCAGACACGGCACACAACTTGAAAGCGCGGAGTGCGCATTGGACATTCCAAACATAGCTAAGAAACAAGCCCCAAGGGCCTATTTAACGGCAAGTGGCTACGGCAAAATTCACGAAGGAGCCGACGGCGGCAACACCATCCAGGCGGCAAGCGGGACCGACAAATGCCGACTGCTGTCAACCGACAACACAAACGGCTTCGCAAACTCAGCAAGCATAACAAAGGACATCAAAGCAATGGCAGGCTATCTTCACATCAAAGGCTCTGGGGCGGCAGCGCAATTTGAAACGCTGGAAAATCTGGCAGCAGGCGCGCCCGCAGACACCAAACCCTGGAAAGAGGCGTTAGCGGCACGCAATGGGATAGTAACGACCACCGAAGCAGCTTACAACAACGAAACAGTCGAACCCAGCAAACTGGCAAGTCTACAACACACAATTGACAAAGCGATGCTGCACACTGAAACTGCAACACCGCAACAAAGAAAAGATGAAGTAGCTAAAATACTAGGCGAAGATACAAAAGCTAAACTAGACGCAGCAGCGAATCTGATCAGCCAGGAGCAGATACCACAAGGTACAGCGGAACTTAAGACGCCCACCAAACTCGGGGACATAAATAACGCCGCCCAGCTGTTAGCACTCCTTTACCACTACCAGATTCAAGCTTTGCAAACGATCCTTGACCTGAAAACACAATTAGACACAGCAACCACAAAAAAAGACCCAAATACAGCAGAAGATACCTGCAACAAAATAACGGCGAAAATTGACTGCAACGCCAAGCCTTACTGCAGTTATAACGAAACCGCAGCTTATGGCGACAAAAAGTGCCAATTTAATGAAACAAAGGCCTCAAAAAATGGAGTCCCTGTAACGCAAACTCAAACTGTAGAACCTTCAACCAATCCAGAAAAGTGCAAAGGGAAAGAAGAGAAAGACTGCAAATCCCCGAATTGTAAATGGGAGGGCGAAACTTGCAAAGATTCCTCTATTCTAGTAAACAAGAAATTCGCCCTCAGCGTGGTTTCTGCCGCATTTGTGGCCTTGCTTTTTTAAttttccccctctttttcttgctaaaaattcttgctacttgaaaaattttcTGATATATTTTAACacgcaagttacccgaaaaaaa

VSG1125.211

MIKSAWEPICGISEELGQVA

GEAWAAADTILENVHKFELA

ALRAAVYYAKNVASPEAKKA

ALLQNYYTNKQVSLINQYRS

SALQSHLRAAQTSSYLKGRI

DDYLRLLEQTTDSNNNCLLQ

TTSAETPAARHGTQLESAEC

ALDIPNIAKKQAPRAYLTAS

GYGKIHEGADGGNTIQAASG

TDKCRLLSTDNTNGFANSAS

ITKDIKAMAGYLHIKGSGAA

AQFETLENLAAGAPADTKPW

KEALAARNGIVTTTEAAYNN

ETVEPSKLASLQHTIDKAML

HTETATPQQRKDEVAKILGE

DTKAKLDAAANLISQEQIPQ

GTAELKTPTKLGDINNAAQL

LALLYHYQIQALQTILDLKT

QLDTATTKKDPNTAEDTCNK

ITAKIDCNAKPYCSYNETAA

YGDKKCQFNETKASKNGVPV

TQTQTVEPSTNPEKCKGKEE

KDCKSPNCKWEGETCKDSSI

LVNKKFALSVVSAAFVALLF

*

>NODE_4471_length_1645_cov_42689.1_g4223_i0 PC-2A

cgtgggtggctagcaacaaggctaaagcatacactttacctctcattgtatctgtactatattgtaaggcaagcaaaaggcaaaaagcagccgaaagctaagcatctccacgcgccaatacagtcccgtgtttgcgagcgtcccggcccgcagcagcggcaccagccgtgcaacaaaaaatagtctttaaggcaacgctgttaagatcggcacagatacaATGAGAGGTAAAGTGTATGCTTTAGCCTTGTTGCTAGCCACCCACGACTCTGCAGAAGCAACTCCCAACGGCATAAAAGCAGGGGCGATAAACAGGCTTTGCTCATTGTCGGACAACCTGAAAGCCTACACAACATACACAAAAACAGCAATAGCAACGGCACTAACGGCAGTAAATGATGTACAGACTGTCAAGCAAAAGCTAATCCTCTTGGTTCTGCACACCAAGAAGCTCCCGACGCAAAACGCCCCACTCATTCTCAGTTTTATGGAGCGGACGATATCTCAAGCTATTTTGGACCTTAAACAAAATGCCCCAACGGCAGTGTTGGCGGCGGCGGCCGCAGCACTTAGCAGTGGCAGAATCGATGAGTACACTCAGCTACTTTACAACGCCAACACAAACCAGCCGGGCCTAGACAAGTACTGCGTCGCCAAAGATACTGGGGCGAGCAAAGCCGACCCAACAGACTTAGGCAGTTGCATAAGCGAAGGCAAGTGGAAAACCAAGATAACCGACGTGCCCAATACCGTGGCTCCAAGCTACGCAACAAGCGTCCCGAACCAAGCAGATGCGCAATCCGTAACTGCACACACCGGCCAAGACAACGGCTGCATGCTGCTTTCCTCCGCCAGCGGGGATGGCTTTGGTGTAACATCCCACGCGGGCAAGGATATACTAGTCATGGGGGGCATATTCAAACTAGGGACGACCGCCCTGGCGGCATCGCCTTTCAAGGATGTTTCGGCGACGGCGGCGCCTAACACACCGTTAGAACAGCTAAAAGCGATAGACCACAGCCAATTCCCAAAGATTGCTGGACCGGCAGTAACGATACTTAAGAAGCTAGCCAGCCTAGGCAAAGGTGCAGACTTCGATATACCCGACCAAACGATACAGAAGGCCGACTATGGACTCGGAGCTGAAGGCACCTTACAGATAGCCAGCAGCGATTTTAAGAAGATAAATACAGCGCTGCAGCAATTCGAGGCGAGCGCATCCGATGGCCTGCAAAGGTTGCTAACCCAGCTTCCCAGCGCTTTGCTTAGCCATGCGGCCATGAACTGCACCGTTCAATCATCAGCAGGCGGGGAACCGAAAACAACCGCGGATAGAAGTGACTGCAATTCGCACGCGGAGGAAGAAGCGTGCCGGAAAGCTAATTGCAACTTTGATGGCTCCAAAAAGCCCAAGTGCTTTGCTAAACCAAGTGAAAATCAAAGCGATAAGCAAGATGGAGAAAGCCGTAAAAAAGAGCAAAACGCCACAGGGAGCAATTCTTTTACAATTAAGAAGGCCCCTCTTTTGCTTGCATTTTTGCTGTTTTAAtttttttccccctcaactttttaagaaactttgctaaaaatttcttgctacttgaaaactttctgatatattttaacacctaaaaaaaaaaaaaaa

VSG1125.4862

MRGKVYALALLLATHDSAEA

TPNGIKAGAINRLCSLSDNL

KAYTTYTKTAIATALTAVND

VQTVKQKLILLVLHTKKLPT

QNAPLILSFMERTISQAILD

LKQNAPTAVLAAAAAALSSG

RIDEYTQLLYNANTNQPGLD

KYCVAKDTGASKADPTDLGS

CISEGKWKTKITDVPNTVAP

SYATSVPNQADAQSVTAHTG

QDNGCMLLSSASGDGFGVTS

HAGKDILVMGGIFKLGTTAL

AASPFKDVSATAAPNTPLEQ

LKAIDHSQFPKIAGPAVTIL

KKLASLGKGADFDIPDQTIQ

KADYGLGAEGTLQIASSDFK

KINTALQQFEASASDGLQRL

LTQLPSALLSHAAMNCTVQS

SAGGEPKTTADRSDCNSHAE

EEACRKANCNFDGSKKPKCF

AKPSENQSDKQDGESRKKEQ

NATGSNSFTIKKAPLLLAFL

LF*

>NODE_2790_length_2132_cov_3006.9_g2626_i0 PC_2A

aagcgaaaaataaggcacatagagtgcggcaggcccgcagcagtaaccttgggcatgaaactgcaaagttacatatgcttttgcgtgcgaaggtactatataggctcaaaagaaagccaaatttcacaaaaaaataaatgggtataacagaaacggtggcgctagccgttgtttcttatctgacggtggattgttaagtccaggtgtttaaaacgagaaccaacacagcaagcagaaagaaaataataagaaacaaagaagcaaagcaacaacaaataccacgccccctcaataagatccaaagccaaaactatacaatgcggaccatttgtggtcacggacttaaatgaagatattttctttaaaaaggtaaaacaaatgtttacaaaatagagcagacactggttgactgatgaagttgggcaacataagcaaaggaataaatgattagtgggcggatgtctgtggcactcgatttagcaaggtgtcgtcaaatttatattaaaattaagctgaatcatgccaaaattgtactagctatgcatgccgcctcctcagcacataagcataagctaataacccacaaaaccttacagataatattagaaaaagcaaaaaacggcagacATGAAGCAACACCTAGTTTTCGCTGCTGCTGCGGCGATAATCAGCTTAGCGCCGGCGGCGCACGTCGAAGCAGCAGCCGGCGATGCACTAAACCACGCAGCATGGTCCAAACTCTGCGACATAACGAGGGACCTAGATAACCTACCGTCGAACGAGCTAGCGTCGATAGCGGCCGAGCAAGTAAACGTTGGCGACCTGACGAAGCTTCAGCAACAGCTAGCGCTCTACAGAGTGCTAAATACCGACAAGGCGGCCACAACCGCAGAACAGGTGTTTGCAACCTTTTTATCAAGAAAGATAAGCGGCGCAGCGATAAGCGAGACTAAGCTAAAGGAGGCACTGCAAAGCACAGCAGATGCTGCTTTTTTGCACGGCCAATTAGCGGAGTGGCTAGCAACAGCCAGCTCTATCGGCGGCAACACGGCGGGCTGCCTAGGGGCGGCCGGCGGAGCCGACACAGCGGACAAAGCGACGATCACCCAGGCCCCGTACGCCTGCAAGCTGACAACGCAAATGGCAAGCGCCAAGCTAACGACGCCGGCAAGCATAGACGCCAACGGCTACACAGGCTTCACTGCGATACCAAACGTCGAAACAAGCAACGCCGTAACGCAAAAAAAGTGCGCATACTCCCAGCACGGCGCAAGCGGGCTGGGCGGAGCTCAGGACACGACAACCAGCATAGCATTCGCAGGTGGCGCATTCATCCTAACAGACAACGCCCTAACACGCAGCAACTGGGCAGCGACGTCTCAAGCGGGGACGCTCCCGCATGAAAAAGCTTTCAAAGCTCTGAAGAGGCAGCACAAAAGTCTGCCGAAAGCAAACTACACGAAAATAACTGATCTAAAGCAAGACAGCGACTTTAGAAGCGCCGCGATCGCGATAATATACGGGGATCAGGACAGTTCACAACTGGAAAACAAACTGACAGACTTGTTTGGCGCCAGCGACAACAGCTTTGAAGAAAAATTCTGGAAAAACGTTCGCGAAGCAAAAATAGACGGCGGAAAATTCGGCGCAAAAGAAGCGACAACCATCCAGGCCTTAGAAGACCCAATAAAACTCAGCAAAGCGTTTTATTATTACGCCAAGCAAGGGACGGAGCAGTTAAAAACACTGGAGGCGAGAGCGGCAGCAACCAACGACCTTCCGAAGCCAACGGAGGAACTTTGCAATGCGAAAAACGACGAGCCCAAAGCATGCAACGAAGCAACAGGTTGCCATTATGACGCTTCCAAAACAGAAGGGCCAAAATGTACTTTGAAAAAGGAATTAAAAGCTCAACTAGACAAAGCAAACCAAGAAACAGGAGGGAAAGATGGCAAACCCACAAACACCACAGGAAACAATTCGTTTCTTATTAGTAAGGCCCCTCTTTGGCTTGCGGTTTTGCTTTTTTAAattcccccctctttttttcttaaagaaaattttgctaaaattttttgctacttgaaaaaactttctgatatattttaacacgtaagttacccgaaaaaaag

VSG1125.4959

MKQHLVFAAAAAIISLAPAA

HVEAAAGDALNHAAWSKLCD

ITRDLDNLPSNELASIAAEQ

VNVGDLTKLQQQLALYRVLN

TDKAATTAEQVFATFLSRKI

SGAAISETKLKEALQSTADA

AFLHGQLAEWLATASSIGGN

TAGCLGAAGGADTADKATIT

QAPYACKLTTQMASAKLTTP

ASIDANGYTGFTAIPNVETS

NAVTQKKCAYSQHGASGLGG

AQDTTTSIAFAGGAFILTDN

ALTRSNWAATSQAGTLPHEK

AFKALKRQHKSLPKANYTKI

TDLKQDSDFRSAAIAIIYGD

QDSSQLENKLTDLFGASDNS

FEEKFWKNVREAKIDGGKFG

AKEATTIQALEDPIKLSKAF

YYYAKQGTEQLKTLEARAAA

TNDLPKPTEELCNAKNDEPK

ACNEATGCHYDASKTEGPKC

TLKKELKAQLDKANQETGGK

DGKPTNTTGNNSFLISKAPL

WLAVLLF*

>node_4379_length_1670_cov_1694.46_g4136_i0 PC_2A

tgaacatgtttgaagccggagaaaaaaccatacgcatgaccaaacaagtaccgaggcctcgggaagcactagcgctttttctggcggccttagcagcagcagcgattggctgtaacggcagcggacagacgggtcttaagaagtccgtatggacgcctatctgtaaaacgtcggaggaacttggcgagatagctggcgaagcgttacacgacgcaaactctatattagaacacgtgaaaaccATGCGAATCGCAGCGAAGCGAAGCGGAATATATGCAATCAAAAATATAGGCACAAACAAACAGCTGAAAGGAACTCTGCTTCAAGAATACTACGCCAGGAGAGCGACGGCAGCATTAAAGCGCTACAAAGACAAGGCAGCGCAGCTGCAACTCGATGCAACGGCGAAGGCAAGTTACCTAAAAGGCAGAGTCGACGAATACCTTAGTCTGCTGGAAACGGCAGCTCAGGGCAGCAACGCCTGTTTGTTGCAGGGGGACAGCGACGGCAGCCCAGCAGCAAGGAATTCAGACCGACTGCAAGGAGAAAAGTGCCGGCTAAAACCGCCAATAACACAGTCGGCGCAACGGGCGACAAAGACAATAGCTACAGAGGGCTTTACAGCGCTGCAAGCCAACAACGGCCAGGAAAGCAACGCCCAACCAGCGCAGTCAACAAAAAAATGCTTGCTCTTAAGCACCGTCGCTACCGAAGGATACGGAAATGGGGGCGCCGCAGCAAGCACGAACATCGAAGCGATGGCAGGTTATTTAACGATCCCCAACACCGATACCAAGCTCAGCCTAACGTCAGCAGCAACACTACACCAAGACGACAGCGGCAAACACCAGGCGTGGACAGAGGCGCACAAAGCGATTCAAGCACAAAGCAGCACAAACCACGGAGACTGCAAAAACGAAACCAACGAGGCGACAGACCGCACCGCGATAAAGCAGGCCATAACGCGGCTCTACGGCAACAAGCAGCCCGCAGATGACAGCATTGTTGGGGGCAAGCTAACACTGATACTAGGCGACAAAACATCCGCCAAACTAGCGGCCGCAGAAGACGAAGTAAACAAGGAAGAGATACCCGCAGGCATAGCAGGACGGCCAGCAGAAGCAACCCTCGGTCAAATAAACGACGAAGACGAGCTGACGAGCGTTCTAGCGTACTACACGACGGCAGCGTCGAAAAAAATCACAGACCTGGAACAAAAATTAAAAGACGCCATCGAAAAAACAGGCCAATCAAGTGCAGCAGACACTTGCAGTAAAATAGCAGATAAAAATGAGTGCAACAACAAGGCTTTCTGCAGTTATAATGAAAGTGCACCTGAGGGTGACAAAAAGTGCAAATTTAATGAAACAAAAGCAAAAGAAAAGGGTGTCCCTGCACCACAAACTCAAACGACAGGAGGTACAGAACCAACAACAGAGAAATGCAAAGGGAAATTGGAACCCGAATGCACCAAGGCTACTGATTGCAAATGGGATGGAAAAGAATGCAAAGATTCCTCTATTCTTGTAACCAAGAAATTCGCCCTCACTCTGATTTCTGCTGCATTTGTGGCCTTGCTTTTTTAAaacaaatttttcccccctatttttaaaaaattcttgctatttgaaaaactttctgatatattttaacacctaaaaccagccgaa

EATRO 1125.1654

MRIAAKRSGIYAIKNIGTNK

QLKGTLLQEYYARRATAALK

RYKDKAAQLQLDATAKASYL

KGRVDEYLSLLETAAQGSNA

CLLQGDSDGSPAARNSDRLQ

GEKCRLKPPITQSAQRATKT

IATEGFTALQANNGQESNAQ

PAQSTKKCLLLSTVATEGYG

NGGAAASTNIEAMAGYLTIP

NTDTKLSLTSAATLHQDDSG

KHQAWTEAHKAIQAQSSTNH

GDCKNETNEATDRTAIKQAI

TRLYGNKQPADDSIVGGKLT

LILGDKTSAKLAAAEDEVNK

EEIPAGIAGRPAEATLGQIN

DEDELTSVLAYYTTAASKKI

TDLEQKLKDAIEKTGQSSAA

DTCSKIADKNECNNKAFCSY

NESAPEGDKKCKFNETKAKE

KGVPAPQTQTTGGTEPTTEK

CKGKLEPECTKATDCKWDGK

ECKDSSILVTKKFALTLISA

AFVALLF*

>node_4928_length_1537_cov_930.609_g4653_i0 PC2_A

ttttctgtactatattgcaaggtagaagactaATGACTGAACTCACCACAGGCAAACTATTCCTTTTCTTGCTGCTCATTGCGTGCTGTGTCAGGTCCCAGGCGGTTCATGACACGAAGAAGCAGATTAAAAACGCTTGCGACAGCAGCGACCACATGAAGATCATCGCCACTCGGCTAGCCGGTGCGCTGGCCAGCCAAGCGGCGGAAATACGCACAGCCGAACTCAAGCAGGCAAAGTTAGCAGCCGCAGCCAGCGCGCAAAGCCGAACAGTTGCTCAACGGTTGGCCCCGGTTCTAGCAGCATACGGAACAGAGCTAAGCAAAGCGAAACAGGCGTTGTGGAGTGCTTTGCCGGCAGTCATAGCAGGCGCATCAGCAGCTTCTCAGCTGGCAGCAACGCAGTCGATGATAGCCGACGTCGCCAAGGTAGCGCTCGACGACGCTAACACACTGGCGGCGAACGGTTTTTTTACCGCCAATCAAGGCAAGCAATTAATACCGAAAACGAAGGCGACCAAGGATTCGGCCTGCTCGGCAACAACTACCGCCACCCGCGACAACGAAGATCGCGAACGCGACTACAGCCAAACAATAACGGCGCCGTTTTACTTTTTAACCGAGTCAGACGCCACAACTCAAATACAGGCGGGCCCGAGGCTATGTGGCGACGACGCGGCAGGTCAAGCGCCGTGCAGCGACGCAACTAACGCAGTAAACGGAGCCAACATAGGTATAAAAGGAGGACCGTTGTTGCAAACCAAGGCGGCGTCATATAGCAGGAGGATAGGCGACGGCAGCTACACAACAGCCTCTATACAAGCAGCCAACGCCATTCCCACAAAAGATTACGTCGAAGAGCAGCTGAGCAACATCAAAACCGCAGAAGATGCAATCAAAGCGTTAACATTCAAGGAGGCTTCAATAGGCAGCCACAACCTAAAGCAAAATCCAACATTCACGGCTGCGGTCGCAAAGATAATGCTTAAGCAAGACAAGCCGCCAACAACAGGCGCAGATCTAAACGCACTAACAGCAGTCATCAGGCAGCAATACGGCGCAGATGAAGGCGAGTACGCAGCAAAGGTTTGGAAGGTAGTTGATAGTACAACGGCTCAACACGCCGTCGATGGTCACGCCGCAACCGCCACGATAGGACAACTACCTTCACAACCGGCGCTAGCGATGTTGACAGCATATAACCTGGCTGGCAAGCCGCCAGCTTCACCAGTTTGTGTCGATGAGTCGCAACAAGTAGTAGATTCCAAAAAAACAAAAGAATGCAAGGAAGAAAAAGACAAGGATAAATGCAACGAAAAGAATGGGTGTGAATTTAAAGATGGAAAGTGTGAAGCTAGAGTAACAGAAACAGAAGCAGGAAAAACGGGTACTCAAAACACCACAGGAAGCAATTCTTTTGTCATTAACAAGGCCCCTCTTTTGCTTGCAGTTTTGATTTTTTAAtccccctccgtaaagaactttgctacttgaaaacctttctgatatattttaacacctaaaaaatcagccgaaaa

EATRO 1125.385

MTELTTGKLFLFLLLIACCV

RSQAVHDTKKQIKNACDSSD

HMKIIATRLAGALASQAAEI

RTAELKQAKLAAAASAQSRT

VAQRLAPVLAAYGTELSKAK

QALWSALPAVIAGASAASQL

AATQSMIADVAKVALDDANT

LAANGFFTANQGKQLIPKTK

ATKDSACSATTTATRDNEDR

ERDYSQTITAPFYFLTESDA

TTQIQAGPRLCGDDAAGQAP

CSDATNAVNGANIGIKGGPL

LQTKAASYSRRIGDGSYTTA

SIQAANAIPTKDYVEEQLSN

IKTAEDAIKALTFKEASIGS

HNLKQNPTFTAAVAKIMLKQ

DKPPTTGADLNALTAVIRQQ

YGADEGEYAAKVWKVVDSTT

AQHAVDGHAATATIGQLPSQ

PALAMLTAYNLAGKPPASPV

CVDESQQVVDSKKTKECKEE

KDKDKCNEKNGCEFKDGKCE

ARVTETEAGKTGTQNTTGSN

SFVINKAPLLLAVLIF*

>node_1279_length_1855_cov_529.777_g1210_i0 PC2_B

atagagaaaaggtttgattgactaatagcaaataaagataagagcgatgtaaacgaataaggcttgaaatgtggagtttaaaacaaaattcaaccggcgccttcgaactggctattataagcatttccttcctttcgcgacgcaaccagttcaaatctttctataacaactgaaacaacaactctttgccaaagcagaagcaacactgagaactccacagATGATAGGAAAAGCCTTTATTATTTTATCTTTACTTAACGAGCTGCCAAGCCCGACGGCAGCACAAGCGGCCGAAGGTGGTGCCCTCGGAAAAGACGTATGGCTACCTCTCGCTAAATTCACGGCGACGGCCGCGAAAATCCCAGGCAGGGCGGCAAAGCTGCTTCAAGACAGGTCGGCCCAAATAGTTAACCTTATGAAACTCCAAGTTCAGGCAGACATATGCCTCAACAAAGCAGCGTCAGAGGTGAGCGCACTTGGGTGGCAGGCGCTCGCTGTTGCAATAGCAGCAGACATCGGCAGCCTGCAAAGCTTGCAACAGCAGAGGAGTGAAGAGGCAATAGCGGCCGCGGCAGCTGCCGAATTCGCTCGGGGCCACGCAGCGGAATTCTTCAAAGTAGCTGCGGCAGTCCAAAGCGCCGCCAATAGCGGCTGCCTGACAACAAACAATAAAGGTGGCGCAGCCGGCAGCGTGATAAACGGATTCTCGACACTCGGCACCGCGGAGCAGCCAGCAATCGGTGCTACATCGACGGCTCACGTCGGCGACGACATAACGGCGATAACAACAACAGGGTTCAGCGACCTAGCAGCAACAGACGGCATACGCACCGACTCACTAACAGCGGACACAAACTGCGTTCTTTTCAAGGGAGGCAGCGCTGGACCGCTAACGACAGCAAACTTCGGCCAATCGATCCCTTTCGCAGGCGGCTATCTAACAAGGAACCCGACAGCCAACACAGCCAGCAGCGCCGACGGTACGGACTTTGTAACCAACCCCGGCGACGGCAAGATAGCAGGCATAAAAGTCTATAGGGACGCCCACGCCGCCGCAGCGAAAATACGCACAGCAGCAACCTTCGGCTCGAGCTTCACCGACTTCAAGAAGCTAGATCAGGCTAAGAAGTCAGTCCATTTGCGCACAGCAGTAAAAAACATAATTCTCGGCAAACCTGACGGATCCGTAGCCGACCTTTCCGACGAGATAGACACAAAGATAAACCAGGTATTCGGCGAAGACCAAGCAACATTCCACAGCAGGTTTTGGGATCAACTAACAAAAGTAAAAGTGGAAAAGGCGGCGAGTGGGCAAGACGAAACGACCCTCGATGCAATCACTTCTTTTGCAGCCTTAAGCCGAGCTCGGACTTATTACTCCACGAAAGTGATTAAAGGTTTGAGAGATAAGATATCCTCACTAGAAATTAAAAATTCCAAAACGGAAGTTAAAGTCACTGACGCCGACTGCAACAAACACCAATCAAAAGACAAATGCGCAGCCCCATGCAAATGGAACGAGAATACCACTGACATAAACAAAAAATGCTCATTAGATCCCGTAAAAGCGACAGAACAGCAAGCAGCCCAGACAGCAGGAGCAGGAGAAGGAGCTGCAGGAACAACAACAGATAAATGCAAAGATAAGAAAAAGGATGACTGCAAATCTCCGGACTGCAAATGGGAGGGTGAAACTTGCAAAGATTCCTCTATTCTCCTAAACAAACAATTCGCCCTAATGGTTTCTGCAGCCTTTGTGGCCTTGCTTTTTTAAttttttccccctctttttcttaaagaatttttgctactttaaaaacttctgatatattttaacacctaaaaccaaccgagc

MVSG5

MIGKAFIILSLLNELPSPTA

AQAAEGGALGKDVWLPLAKF

TATAAKIPGRAAKLLQDRSA

QIVNLMKLQVQADICLNKAA

SEVSALGWQALAVAIAADIG

SLQSLQQQRSEEAIAAAAAA

EFARGHAAEFFKVAAAVQSA

ANSGCLTTNNKGGAAGSVIN

GFSTLGTAEQPAIGATSTAH

VGDDITAITTTGFSDLAATD

GIRTDSLTADTNCVLFKGGS

AGPLTTANFGQSIPFAGGYL

TRNPTANTASSADGTDFVTN

PGDGKIAGIKVYRDAHAAAA

KIRTAATFGSSFTDFKKLDQ

AKKSVHLRTAVKNIILGKPD

GSVADLSDEIDTKINQVFGE

DQATFHSRFWDQLTKVKVEK

AASGQDETTLDAITSFAALS

RARTYYSTKVIKGLRDKISS

LEIKNSKTEVKVTDADCNKH

QSKDKCAAPCKWNENTTDIN

KKCSLDPVKATEQQAAQTAG

AGEGAAGTTTDKCKDKKKDD

CKSPDCKWEGETCKDSSILL

NKQFALMVSAAFVALLF*

>node_4566_length_1624_cov_261.857_g4310_i0, PC2_A

ctttttgtagactctccaacatccagcagaaaggaaagtaggaaATGATCACCGTATTATTACTCGTACTGCTTTTAGAATGCAGCATGCAAGTAAGGGCCCACTCAGCTGCCGGAGAGAATGCTGGTGCTTTTACGGCACTGTGCGGCCTAATAACTCTAGCCAAGTCACAACCAGCCACATTGCAAAAGCCATCCGAACTAAACAGCATAATAAACACAATTTCCGCCATAAATCTTACTGTGAACGACGATAGCTTCGGCGACAAAATCGATAAAGACAAAACCTGGGACAAAGCATCAGCGGAGTACAGGCAAACACTACCTGGCTGGGAACACAGACATGATGCATACGCACAGGCCAAAAAGCTACTAGCAGGCGATAACAAAGCAGCGTTCGAGCCGTGGAAGAAACACAAAGCCTCACCTGAGGTCAAGCAACAAGTAAACCTCATAGCTGAGCAAGCGTTTGACATCATAGCAGCAGCGTCAGCCGACATTCGGAACGTATTAGATACGCAGAAGACAATCGACAAGCAGAACAAGGCGCTCTACGGGACAGAAACTGCAAATGACGACACATACAAGGTAGGCACGGCAGCAGCCGGCAACACCCGGGCAAATGTATGTTCGCAGACCGGTGGCAGCCACGTAAGGAAGCCAGGGTTCAGTCTTGTACAAGATGCCCTTTGCTTGTGCGCCACAGGACCGGCAAGTGACGGGGCGCAGGGCAAAGCATGCTGCGAAGGCTGTGCAAAAGACGGTGGCGATGCGGAGGAACTGGCAGCAAACACAGACGCCAAAGACAAATGGCAAAAATTAGCGGCGGCCTGCGCTGCACTAAGTCTAAATGCCAAACTAACCCAACAGACTTTAGCAGCGGCAGCTGCAGCAATAGCAGGCCAGCTAACACACAAAGCTTCAACTCAAACAACAAGCAACAATGTACTAGGGAGCATTGCAGGCGCCGGTGCAGCAGGATGCACTGGCAACAACAATGGCGGGGGCGGAAAATGTGTCGTGTACGTCGACGGATTAACAACGGCGGGAAGCAACAAAGTCAATTGGTTAGTGGCGCTGCAGGAAGCGGCGAATCATGAGCATACGCGGGCTATAAGCGCCGATAACTTGCAAAGAGCGGTACTTCAGTTGACAGCTCTGAACAACAGTCTAGCCTTACTTCTGCACAGCCCCATAATGGAAGGAAGGAACAAACAACAGGAAGCACCAAAAGCGGGCTCCGCCAACGACGCCGCAGCCATAAAAGAGAAACAAGAAGAAGCCGAAAAAGACTGCAATAAAAAAGAAACAGAACAAGAATGCGCAAAACCGTGCAAATGGGATGGAGAAGCGAAATCACAAAATAAAAGGTGCACATTGAGTGACGAAGGCAAACAAGCAGCTAAAGAAGCAGAAAAACAGCCAGGGAATGATGGGAAAACGAACACAAACACCACAGGAAGCAATTCTTTTGTCATTGACAAGACTCCTCTTTTGTTTGCATTTTTGCTTTTTTAAaacaatttcccccctcaatttttcttttaaaaaattcttgctaaaaattcttgctatttgaaaactttctgatatattttaacacgcaagttacccgaaaaaaaaaa

EATRO 1125.313

MITVLLLVLLLECSMQVRAH

SAAGENAGAFTALCGLITLA

KSQPATLQKPSELNSIINTI

SAINLTVNDDSFGDKIDKDK

TWDKASAEYRQTLPGWEHRH

DAYAQAKKLLAGDNKAAFEP

WKKHKASPEVKQQVNLIAEQ

AFDIIAAASADIRNVLDTQK

TIDKQNKALYGTETANDDTY

KVGTAAAGNTRANVCSQTGG

SHVRKPGFSLVQDALCLCAT

GPASDGAQGKACCEGCAKDG

GDAEELAANTDAKDKWQKLA

AACAALSLNAKLTQQTLAAA

AAAIAGQLTHKASTQTTSNN

VLGSIAGAGAAGCTGNNNGG

GGKCVVYVDGLTTAGSNKVN

WLVALQEAANHEHTRAISAD

NLQRAVLQLTALNNSLALLL

HSPIMEGRNKQQEAPKAGSA

NDAAAIKEKQEEAEKDCNKK

ETEQECAKPCKWDGEAKSQN

KRCTLSDEGKQAAKEAEKQP

GNDGKTNTNTTGSNSFVIDK

TPLLFAFLLF*

>node_4129_length_1735_cov_242.596_g3902_i0 PC2_A

aagaaaaagatactgaaaattaaaggccccataaatgaaaagatgtcgtgctttatgttgacaaggcatttatgcgctttattttagttttttaacacgggctgcagaaagaagacaataaaaacccactaaagtaagaagaacacagcataacggaagagacagcacgaaaaacacagaccaggttagtccctttcgatatcttccaacactcaagatcaacaagataaatcaaataacggtagtacaacaATGAAAATATCAGCAGCGAGTGGCAAGGAGCCGATTCAAATAGCATTATTGTGCTGCCTTCTAACAGTATGGGAGGCTTCAGCGGCAAATGAAAATGCTGCAGCCTTTAAGGCTCTGTGCAGGATGTACAGGACACTGAAAGGCAAACTCCCACAAGCAGCGGCCGATCTAACCGACGTGGATTCGGAGATGAGACAACTAGAGAACTTAAAATTTAGCAGCCTTCTCTCGGCAGCCTACGACAACAGAACCTTTGGTGATGTTACAGACCCGGAACACTGGAGCCAACGCAAAAAAGAAATAGAAAGCCAAGGAGAAGCAGGAACAGAAGGCACATACAGGCGATACCCAGACTCCGAAGCAAAACAAGCAGCTCATAACAGGATTGTTATTTTCAACAGAAAGGCGAAGGAACTTAAGTCACGGCTGGACAGAATTAAGACAACACTGACCTTAGCAACGACCGACGCGGCGAACCATTTGAATGCGGCTTTGTACGGCGGAAACAAAACAAAGGACACCGACGATGGAGCTTTCGCAGCAAGGGCGGACGCTTGCATAGCCGCCGGCAACGCTGTAGGGAAGTCTCTGGTATCTGACTTTGCATGCGTCTGCGCGGCGACGACAGCGGATGACGTCTGTTGCAAGGGGTGCGCAAACACAAACTACGCAAATGCCGGGACAAATAACCCAAGCGACGCCAAAGCGGTATGGCAAGCAGTGAAAAGCAAATGCGAAAAGCTGCCAACCGTACAACAAGCGACAAAGGAAAACCTACAGGCAGCCCTAGAAAGTGTAATACAGGAAATAGGAGGGTTGTCGTCAAACGCCAATTCGCCTTACATGCTCGGAGGAGGTAGCGCAGGGACTTGTACCGGAGCGACAGATCATAACCAAGCCTGCGTGGACTACTCACAGAAACTTCAAGGAGGCGGAGCTGCAAGCATACCGTGGATGAAGCAAGTTACCGACGTAATCCGGAACATACGGTTAATCAACACAGCCTCAACAGAAGCACAAGCAGCCAAGGAAGCGATAAAGAACTTAGCAACTCTTGCCTGGACAACTTATGACCTAATTCCAGCAAAACCCGGCAACAACCAGCACGACGGGACAGAGCAGCAAGCGCAAGCAACCGCAGCAAACTCCAACCAAGGTGAAGAGTGCAACAAACAGCAATCGCGCGACAAATGCAATGATCCCTGCACGTGGAACGAAAATAGCAGCGACCGAACTAAACGATGCACATTGGACCCTGTCAAAGCGACCAAACAGCAGACCCAGGCAGCAGGAGCAGGAGATACACCTACAGGACCCAACACAAACACCACAGGAAACAATTCATTTATTATTAATAAAGCTCCTCTTTTGCTTGCAGTTTTGCTTTTCTAAaaatttcccccccaaattccccctctttttaaaaaactttgctacttgaaaaacttttgatatattttaacacgtaatttctgccgaaaaa

EATRO 1125.421

MKISAASGKEPIQIALLCCL

LTVWEASAANENAAAFKALC

RMYRTLKGKLPQAAADLTDV

DSEMRQLENLKFSSLLSAAY

DNRTFGDVTDPEHWSQRKKE

IESQGEAGTEGTYRRYPDSE

AKQAAHNRIVIFNRKAKELK

SRLDRIKTTLTLATTDAANH

LNAALYGGNKTKDTDDGAFA

ARADACIAAGNAVGKSLVSD

FACVCAATTADDVCCKGCAN

TNYANAGTNNPSDAKAVWQA

VKSKCEKLPTVQQATKENLQ

AALESVIQEIGGLSSNANSP

YMLGGGSAGTCTGATDHNQA

CVDYSQKLQGGGAASIPWMK

QVTDVIRNIRLINTASTEAQ

AAKEAIKNLATLAWTTYDLI

PAKPGNNQHDGTEQQAQATA

ANSNQGEECNKQQSRDKCND

PCTWNENSSDRTKRCTLDPV

KATKQQTQAAGAGDTPTGPN

TNTTGNNSFIINKAPLLLAV

LLF*

>node_973_length_2998_cov_1601.87_g912_i0 PC2_A

tctgaaagtttctcattaattccatgtgtttttatggtaaaattatcattttcacatgttgaagatgtaagaaactacatcgtttaaagatttgattcaatacttttatcagtgaaagtagttagttatatatatattcagagaacgcactggactttagcggagcaacaatgattattcacggaaaaaagcggcaaccttagcgcccactggtgcctatattttatttagaactctttgaagaatgcattgtttgcacccaagactctataatggctgtagaacgatgtcagatagaaaattaaactggtagaggccgtacaaatcatgtaaaatgataattatagcgggaattaatgacagacaggaagcggtggaaactcaagaaactgatgattgcactactctttgcagaaatgccaaaaaagcgtcaaatttttaaaaagtaaaaaaaagaaaaaggatgcataaaaacaaataaacgtcaataaatcttttaatttttcgcaacaaagagtaaaaacagtaaaattcagtggatgatacattcaataaaattggaaaatttattggagaaaagtaagcatttctattcttctagagtgaagcatgcaagtgtctgaaaatgcgcgtttaacaaattagaactggcgtgacttacaataactcaactgcatttaagctatcaggcaaacataacgaaggaaaaaccatttcccttgatgattagagcaccatagcgccttcccgtatcttcaaatttcaaaagaaagcaataattacaaacaaccctcgccgggaaatagtgtaaaaagaaggagtatatagtaaaatagaataaacaacaactccataatgcttagaatgtcgatatacaaagaaaggaacagacaacataggatatgaagccgaatcgactgcgcgagcaggggagataattgtaaatgagcataatatccgacgctggcaaacaccatacagactcgataaaaagccgcatttcgtacagaaatagtttaattcgcggccagaagtggtagctactatagtttttaatttgatggtggattaaaattttaagtgctaagtcgatgagtaacaaattcgtatactgtggtggttttttgatttaggtagagcatagagaaggtggagagaagattttaaaaaaacgaggggcagtgatgagttgaagtattgtaaataaatatatatatttaatttttatggcaaaggcagaaacagagcaaatacaaagactagagattttttttaaaaagctgcactaacacaccacctaacataaaaaactagtcaccatgattataacgaatttttcaagaaaatccaatgaatttaagagaaaaaattttccttcttatttcgacgccgccgaaatgccaacacagctactggaagaagtcaaaattagtaacgaaaacagcagccgcaactatttctccaaaaaacagccttttcctgacgctagaattggcaaacgcaaATGCCCAAGACATCAAGGCTATGCTCGCTAGGCTTTTTTGTCCTAGCGTTGCTTGCAGAAAGCTCACGTACGGCCACAACGTACACGATCTGCTCGACGCCGTGTAAATGCAGCAAACGACTCGGCAAAGCATCGACATTCTATGCTCAGAAGTTTGAAACCAACGTCGGCAAGCTTGTAAAGATGCAGACAGATTTAACAAAACTGCTTTTAGCGGCGACAGCAGCCGACGTAGCAACTGCAAAAACCGCTCTACCGACTCTGGCAGCGGCGGGAAAAGTAATTCAGGACTGCCAGGCAGCTGTCACAAGACAGCTTGCGGCACTGAAAACGGGCCTACCAAAGATAGCAAACGCCTCAGCGAAACTTGCAGCTCTAGCAAGACGGCAAGCCACAAAGACGACAGTCAAACTGACTCCAAAAGGCGGGACCAGCAACTACAAAGACACTTCGTTCGCGAACCCTCCCGTAGGCATAACCAGCGACGACAGCTGCGGCCATGAAACAGGCGACGGCGAAACCGACTACGACGACAACGAAGCAGACGAAAAAAATGCGATACTGGAACCCCAAGAATATCACACAGTAACTGTAACTTGCAGATCAACAGGCACAACAAACTGCAACAGCGCAGGACAAACGCAGGACGACGGCTACATCCAATTCGAGCTCACATCGGCCATAAAAGAAGAAACATCCAAGCCAACTACACGGTGGAATGCAGCCAACACCGACAGCGATGTAATAATTCACGGAGCGGTAAATGTGACGCAGGGAACCAAAGAGCCGACGGAAGCTGCCCTGAAGGAGCTCAAACAGGCAGCGCAAAACACGGCGTGCGACAAAAAGCTAACAGAATACGCAACAGTTTCAGCATCGCCTTTGTTCAGACGCCAGGCAATCCGTAGTTTGCTCAACCAAGACGCCGACGCACAGGATTTCACAACACCCCCAGACAAGCTAACCGGCGCCCTAACGGCAGCTTACGGCGCAGGCGGAATGGACTACAAAAAAAAGTTGTGGGAAGCCATAGACAACCTTAAACCAGCGATAACAAAGAACAAAGAGCGAGCCGAACTGGACATTAAAGAAAACACACCTCTTGAGCAACTCACTGAAGCCCTTGCACGGCAGATAGGCGAAGCTAACTCGAAGGCATCACAAACAACTAAAAATAACAAAAATGCAAACGATCCAAGCAAGTCAGATGCAGCAGACAAAAAAGAGGAAAAGAAAGACGGGGATCAAAAAGACGAGGAATGCAAAGCCACTGAAGAAGGTAAATGTGACAAGACAAAATGCGATTGGAACGCTGAGAAGAAAGAGTGCAAAGTTAAGGAGGGCGCGGCTGTTATTTCTGCTGTAATTAAAGCCCCTCTTTTGCTTGCGTTTTTGCTTTTCTAAttccccctctttttcttgctaaaatttccttgctataatttttgctacttgaaaactttctgatatattttaacacgcaagttacccgag

EATRO 1125.2523

MPKTSRLCSLGFFVLALLAE

SSRTATTYTICSTPCKCSKR

LGKASTFYAQKFETNVGKLV

KMQTDLTKLLLAATAADVAT

AKTALPTLAAAGKVIQDCQA

AVTRQLAALKTGLPKIANAS

AKLAALARRQATKTTVKLTP

KGGTSNYKDTSFANPPVGIT

SDDSCGHETGDGETDYDDNE

ADEKNAILEPQEYHTVTVTC

RSTGTTNCNSAGQTQDDGYI

QFELTSAIKEETSKPTTRWN

AANTDSDVIIHGAVNVTQGT

KEPTEAALKELKQAAQNTAC

DKKLTEYATVSASPLFRRQA

IRSLLNQDADAQDFTTPPDK

LTGALTAAYGAGGMDYKKKL

WEAIDNLKPAITKNKERAEL

DIKENTPLEQLTEALARQIG

EANSKASQTTKNNKNANDPS

KSDAADKKEEKKDGDQKDEE

CKATEEGKCDKTKCDWNAEK

KECKVKEGAAVISAVIKAPL

LLAFLLF*

>node_4581_length_1620_cov_98.6767_g4324_i0 PC2_A

gatagccttcggccaaaagtaagtcatctaacccaccactgtcaacaagggacaccatctATGAGACAAGCTATCCTAGTTTCCGCATTAGCAACGCTATACCTATCAAACCAAGTGAAAGGAGCAGCAAACGACAACGCTAGGCCTTATAACGTACTTTGCGCAATTTTAAATGTCGCCACAGCGAAACCTGACGTTGACACGACTGACTACAGCGCTAAGATCTCAGAAGAATTGGAAATGGTACGACACCTCAACATGTCAGTAAGCGACGACGGTTTTTTCAATCAAGATTTTAAAACGCCAAACACAGACAGAGACGCAAAAGAACCATGGAAAAGCAACAAAGCGGCTTGGGAGAAGAGTAAAAACCTAGTAGAGGCAGGTGAAACCAAGTTTCACGGTATAAAAATTACACGAAAACTCGCCAGCCACGAACGAGCGGTCGCTGCCGCAATTGCAGACGAAACAGCAAGCACGATTCAACAGTTGCAGAAGACGCTCAAATCCACAAAAACAACAGCAGACGTTACTGCTGAACTCAACAAAGCGATCTACGGCGCAAATGGTGCTCTGGACAAATCAGGCGATACGACGTTCGTAACACAAAGCGGCGCCGGCTGCGGAGGCGGGGGTCAGGGCGCCTCAAAAGCAGGCATTTCTCTAGCTAATGACATGGTTTGCCTGTGCTCAAACACGAATGGGCAAAACACTGCATGCACCGGAGCAGCAATCAACTCGGATCTGAAATACGACACCAGCGCCAATGCCGCGACAGCATTCACAAAACTCAGAGAAAAATGCCCCATGCATATTAACCTAAAAGCAACAGCCGCAGGATTACGAAACGCAATAACAAGTTTCATAGGAACTTTAAAAGGGAGCGCCAAAGCGACACAAGCAGGCAACACAATCCTAGGCTATGCTGACGCAGACACCTGCAACGGCGGAGCCAACGCGGACTGTGTACTCTACAAAACAGCGGAAGCTGGGAAGCCACTAAATGTACAATGGCTAACTCATTTATCAACAGCGGCAGATATTCTAGAGACAATCAAAAGCGAACAAGAGCACAATAAACAGCTGCTAAGCAGTGCCAGGGCGCTAGTAACGGGAGTGCTCAGCAGCTACATTCAGGCAGACAGACCAAGGCCAGACACCGTGTCACAGCGAACCATCAACGAACGAAGTACCAACCCATCAACGCCGCCAATCTGCGCCACCCACACAAGCAAGGAAGATTGCAAACCACCCTGCAAATGGAATGTGAATGCCACTGACAAAACTAAAAAATGCTCGTTAGACCCTAAAAAAGCAGCAGAACAACAAGCAACCCAAGCAGGAACAGGAGAGGAAACTGCAGGAGCAAACTCTGAAGGCAAAAAGTGCTCCGACAAGAAAAAAGAGGGAGATTGTACTGGAAATTGCAAATGGGATGGAAAAGAATGCAAAGATTCCTCTATTCTAGTAAACAAACAATTCGCCCTCAGCATGGTTTCTGCTGCATTTGTGGCCTTGCTTTTCTAAaattttccccctctcaatttttctttctgaaatttgctaaaaaattcttgctacttgaaaaactttctgatatattttaacacctaaaaaaaa

EATRO 1125.2564

MRQAILVSALATLYLSNQVK

GAANDNARPYNVLCAILNVA

TAKPDVDTTDYSAKISEELE

MVRHLNMSVSDDGFFNQDFK

TPNTDRDAKEPWKSNKAAWE

KSKNLVEAGETKFHGIKITR

KLASHERAVAAAIADETAST

IQQLQKTLKSTKTTADVTAE

LNKAIYGANGALDKSGDTTF

VTQSGAGCGGGGQGASKAGI

SLANDMVCLCSNTNGQNTAC

TGAAINSDLKYDTSANAATA

FTKLREKCPMHINLKATAAG

LRNAITSFIGTLKGSAKATQ

AGNTILGYADADTCNGGANA

DCVLYKTAEAGKPLNVQWLT

HLSTAADILETIKSEQEHNK

QLLSSARALVTGVLSSYIQA

DRPRPDTVSQRTINERSTNP

STPPICATHTSKEDCKPPCK

WNVNATDKTKKCSLDPKKAA

EQQATQAGTGEETAGANSEG

KKCSDKKKEGDCTGNCKWDG

KECKDSSILVNKQFALSMVS

AAFVALLF*

>node_4442_length_1653_cov_27.607_g4197_i0 PC2_A

gcgcatgctATGTTTCTGTTTTCCTTAACGCCCTTCGCTGCGTTTGCCGCGCAGGACAACGCTAAAGAATACAGAGATATGTGTGCAATCCTCAAACTGCTAACGCAAAAGATTCCAGCTACTCAATCAAAAGACAGCGGCGACAAAGCGGTGGCTGCCATTACTCCGACGGCCAAAATGGAAGCGATATACGCCAACATCGTCCTTCTTAATTTAACGGTGGCGCCAGACACCGTACTAGCTGTACTGAGTGATAAGGCGAAATACAAAGACGGAAAAACAGTAAAAGCAAATGCCGAAGTAAAAGATGTATTTACTGACATCGACGAGACCACAATTGACCTGATGTTCTCCCAGGCACAGAAGATAACGGCGTCGGCGGCAAACAAAGACTTCACTCAGAAATATGGAATACCTATCGACTTAGAAACAAGAGAGCGACTGCGGCCGACAGTGGCGGCGCTAGCAAACAAGGCGGCAAAATTTTGGCGCAGATTACTCGCGCTCGGAATCGAAGACAATGAACTAAGGACTCAGACACGGAAACTCATGCTGAAGGCGCTGTACGGCCAAGCTTACTTGACCAAGCACGGTGCATCTATCACAGCCGACGGGGAGGCGCCAGCATTAGACCAAAACGAATTTTCCTGGACCGCCTCGGCAAGCCGTGACGGCAACTGCAAGGACGCCGATGGACAGGCCGGTAAAGCAGGCCATTGCCTGGCACAAGACATGGTTTGCTTATGCGTCGCCGGGCACAACAGCAATAACCAGTACTGCTCAAGGCAACAAACAGCTAGCGACGACTATTCAGGCACAAGAGCAACAAAGGCAACAGCCTTAGCCAGCTTCAACAAGCTAGCAGCAGCTTGCGGCGACGGGCAAGAAGAGAAAACCAGAGAGTTGTCTGGGCCAGCACTGGCTCAAGCAGTAGCAGTACTAACAGCGAACTTCGGCACCAACTGGGTAAGTCAAGCTTCGCTAGGAGATGCAACGGGGACCGGGAGCGACAAAAGCGGCATCCTTGGTGTTTACAGTGTCGCCGGCGGCACAAAAGTTGATTGCAGTGCAACTACCGGGAGCCCACTCGCCGGCGGCGGCAAAGGCATTTGCATAAACTACAACGCGTTGCTTGCAGGCGGCAAAGGCATACCCTGGATAGATACCGTGCTGGAAGCCGCCAGCAACCTAAACAAAATTTATCAGGATAGCGTCGACCAGATGGGCATCGTCAAATCGGCGGAAGCCGTAGAAAGTCAGATAGAAAACCTACTTTTGATGAGAAATTTATTAACACAAGCCAGCACAAAAGATGTACAGCAAAAAGTGAAGAAACCAACAGTAGAGGAACTCAATAAATGCAAATCTGCTGCAAACAAAACAGTAGAAGGATGTTCAGCTATTGACTGCGAATATGACTCAGAAAAAAACGAGTGCAGACCTAAAAAAGGAACAGAAACCACAGCAACAGGCCCAGGAGAAAGAACTACACCCGCGGATGGCAAAGCAAACAACACCGTAAGCGATTCTTTACTCATTAAAACTTCCCCTCTTTGGCTTGCATTTTTGCTGTTTTAAttttcccactcaaattccctctccctttaaaaactttgctacttgaaaactttttgatatattttaacacctgaa

EATRO 1125.84

MFLFSLTPFAAFAAQDNAKE

YRDMCAILKLLTQKIPATQS

KDSGDKAVAAITPTAKMEAI

YANIVLLNLTVAPDTVLAVL

SDKAKYKDGKTVKANAEVKD

VFTDIDETTIDLMFSQAQKI

TASAANKDFTQKYGIPIDLE

TRERLRPTVAALANKAAKFW

RRLLALGIEDNELRTQTRKL

MLKALYGQAYLTKHGASITA

DGEAPALDQNEFSWTASASR

DGNCKDADGQAGKAGHCLAQ

DMVCLCVAGHNSNNQYCSRQ

QTASDDYSGTRATKATALAS

FNKLAAACGDGQEEKTRELS

GPALAQAVAVLTANFGTNWV

SQASLGDATGTGSDKSGILG

VYSVAGGTKVDCSATTGSPL

AGGGKGICINYNALLAGGKG

IPWIDTVLEAASNLNKIYQD

SVDQMGIVKSAEAVESQIEN

LLLMRNLLTQASTKDVQQKV

KKPTVEELNKCKSAANKTVE

GCSAIDCEYDSEKNECRPKK

GTETTATGPGERTTPADGKA

NNTVSDSLLIKTSPLWLAFL

LF*

>node_4884_length_1548_cov_16.1909_g4611_i0 PC2_A

cagcggcagtgaagctgcaaagaacagacgtaactttggagggaccaccagaccaaccaacggtgatacaaaacctgaatctgctaacgttgcactcatccaactttacaaacaaaacttacctaacctatgacactgttcagaagtggagcgaaaaaaaagccgaattcgccggtgcacaaagcaaaacagcagatggcgattacgtggtgaaaccagattcagaagcaaaagctgttggtcacaacagactcgcgcgtatacttaaacatgccaataacgtgtatcaaacggcacaagacgccaataatcagttaaaccagcgcatcacagacgtcaacaatcacctaacaagcgcaatatatggcatcaataaaactgatgacaactcggaagcaccatatgacacgaggcaaaacgcttgcgccgcagcaggcaatgcaatcgggaaatcgcttttatcggaaATGGTGTGTATTTGCAGTGGCAGTTCCGCCGCAAGCGTGTGCTGCACCACATGCGGAAATGCGCTCTACACAAACGCGGTCAACAACAACCCGAACAATGCCAAAACAGCGGCAGCAATCACCCTCGCAATCTGCAAAAAGAAGCCAAAACCGAAATTGTCGGTAGCCACAATAGCGTCCAGCGTCAATCTTTGGCTCAGCACCATAGGCAACAAGCAAACTGACACCAATGGATACCTAAGACTCGGCGGCGGCGCGACGGCCGTCAACTGCGAGGGGGGCAGCGACGCAAACACAGCATGCACGGACTACACAACTGTACTAGCCTCTGGAGACATAACCAAGGTGCCGTGGGTAAAGCGACTACTTCAGGCCGAAGCGGCACTAATACAGGCAGACAAAGAAGCAGCAGTGCTAACAGCAGCCAAAACGACTCTAAGTCAGCTTACAATCCAGGCGTGGACTGTCTATGATAGCATACCATCGACGAGCATCGATGCTAAACCAACCCAGGCACAAAAGACAACGACAGGCCACCAGATAGAATGCGACAAACACACTGCTAAAACCGCAGCAGAATGCAAAAGCCTTGGCTGCGACCATGACGAAAAGGAAAACAAGTGCAAACCCAAACCAGGAGCAGAAACCGCAGCAGCAGGAACAGGAGAGGGAGGATCAGACCCTAACCGTAGCCAGTATACGGACACAGAAAAATGCAAAAAGGTTCCAGGCAAACCAAAAAATGGAAAGAAAGCTGTTTGTGGGTGGATTGAAGGTAAATTCCAGGATTCTAGTTTTCTAGTAGACAAACAATTCGCCCTCATGTTTTCTGCTTTTGTGGCATTGCTTTTCTAAaagaaatttcccccatcttttaaaaacttttttccttccgaaatttgatatattttaacatgtaaattcgactgagagattattattattgttggatgcctggggatgttattttttgttgatggcttgaaaattttctccctttttactttttttttacttttttaggattagttttttaaaaggctttagggatagaacttatatttttttctgcgtctttcat

EATRO 1125.383

MVCICSGSSAASVCCTTCGN

ALYTNAVNNNPNNAKTAAAI

TLAICKKKPKPKLSVATIAS

SVNLWLSTIGNKQTDTNGYL

RLGGGATAVNCEGGSDANTA

CTDYTTVLASGDITKVPWVK

RLLQAEAALIQADKEAAVLT

AAKTTLSQLTIQAWTVYDSI

PSTSIDAKPTQAQKTTTGHQ

IECDKHTAKTAAECKSLGCD

HDEKENKCKPKPGAETAAAG

TGEGGSDPNRSQYTDTEKCK

KVPGKPKNGKKAVCGWIEGK

FQDSSFLVDKQFALMFSAFV

ALLF*

>node_5189_length_1464_cov_15.5295_g4903_i0 PC2_A

gacattagactgtaagaaaacatccttgtgcacgcccacacaaacATGGCCGTTGCCAACGTCCTAGCAATTGCTTCAGCGGTCCTGTTTCTGACGACAATACTAGAGAAAACTTCAGCAACACACATGGGAGTGCTCAAAACAGACCTTGACAAAGCGTGCGAGCTAGCAACCGAACTAAAGGCGACCTCAGCCCACGTGCAGTTCATGTTAAACAAACAGACAAACGAAGAAGAACAACTACGAGATCTGGCAGACGACCTAGCTGCAATAACTCAAAATGCAGCACCGGAAAGCAGAACTTGGATAGTGAAACTCGCGGTCTTTGCACGAAACCAAGAACGACAAACGCGACATGTAATCGCACATCAAACACTGGAGGCGATCAAGGTTGTAGCCAAATCGGCAACGTATGCCGGCAGAATAGACGAAACAGCAAAACTGCTGTTGCAGCCGAAAACCGGCTCAACCTCGATGTGCGTTAGCAATGCAGCAGCCTTCGGCGGAATTAACCCAGCAACAGCCAACACTATCAAGTGTTTCAGCAAAGCAAACGCCGAATATGATGAGCCAGACGAACAGCCAACCAAAAAGGCGTTAGCAGGCAAAGCGAAGTTTGAAGCAATCAAGCAAGGCAACGCAGGAGTAACAAGCAGAGGAACACACTGCATTATCTTACACAGCGGCGCCGGCAGCGGCTTCAGGACGACTGAGGGTGAAATCGCTATGATGGCCTGGCTCATCAAACAGTCAACGGCAGTGGAAACACCGCAAACGTGGCAGGGCGGCTCTAGCAACCTAGGAACTGTCGGCAAACCTTTCACGGATATCGAAACAAAGCTAAGCACGTTCAACAGTCAAATAGCATCCCATGAAGCACTAAACACCGCTATACTCAAAATAAGTCAAGATAAAGACGCCCAGGCTGAACCATTAGAAATACCAATAGGCAGCCTAGGCAACGGCCAACCTGAAACAGCAATAACAATACCGGCAGCGGAACTAACTTCTATAAAAAAAACTCCTGTCCAGTACCGGGAGAAGCACGAAGCTACTCCTTTAGAGACACAAAGACTCAATTTCTTTACCAAGCAGCTTGAAATTAACAGAACGGCTTGTGAAATTGGGAGCAAAGCATCTGCAGCGAAGTGTCCAATCCAACAAAGTGACAACAGCAAAATAAACTGCGCGGACTTTTCACAGGAGAAGTGCGAAGGCGACTGCGAATGGGACAAAAAGGACGGAAAGTGCAAACTAACAGACAAGGCTCAGCAACAGGCGGAAAAAGCAAACCAAGAAACAGGAGGGACAGATGGGAAAAACACAAACACCACAGGAAGTAGTTCTTTTCTCATTAGCAAGGCCCCTCTTTGGCTTGCATTTTTGCTTTTTTAAtttttaacccctctttttaaaattttccttgctaaaaatttttgctatttgatatattttaacacgtaa

EATRO 1125.514

MAVANVLAIASAVLFLTTIL

EKTSATHMGVLKTDLDKACE

LATELKATSAHVQFMLNKQT

NEEEQLRDLADDLAAITQNA

APESRTWIVKLAVFARNQER

QTRHVIAHQTLEAIKVVAKS

ATYAGRIDETAKLLLQPKTG

STSMCVSNAAAFGGINPATA

NTIKCFSKANAEYDEPDEQP

TKKALAGKAKFEAIKQGNAG

VTSRGTHCIILHSGAGSGFR

TTEGEIAMMAWLIKQSTAVE

TPQTWQGGSSNLGTVGKPFT

DIETKLSTFNSQIASHEALN

TAILKISQDKDAQAEPLEIP

IGSLGNGQPETAITIPAAEL

TSIKKTPVQYREKHEATPLE

TQRLNFFTKQLEINRTACEI

GSKASAAKCPIQQSDNSKIN

CADFSQEKCEGDCEWDKKDG

KCKLTDKAQQQAEKANQETG

GTDGKNTNTTGSSSFLISKA

PLWLAFLLF*

>node_5344_length_1428_cov_13.209_g5053_i0 PC2_A

caactatatcagtagcgacaacaatcgaatattcccatgcagcggtaggagcggcgctaaaacaaagcgtcgcgaagcaaATGTGCGACTACAGCAAACAGGCAAAGGGACAGCACCCGCATCTAAAAGCAAAACTAGCGACAGCCAAAAATAACGCCGCAGCAGCATTCAAAGGCCACCGACAGCAGTCGCTAGCAATGCTGATAGTTCCGCAACATAGCGCGGCAAGCCAAATACTAGCAGCATACGCCGCCAGCCTAATGGCCGAGAGCCTAGGACACATCGAGCAAGCGGCTACAACAGCTGTGACAGCAGTGGCACAAGCTAGCTACAGCGCTGGACGCACGGACGAACTTCTAAAGCTGCTAAAAAGCATGGAGGACGGCTCATCAAACGACAAGTGGTGCATAGCCACGACCGGCGGAGCCGCCAAAGCAACCAACAACCCAACAGAAGGGTGCGAAGCACAAATCAGCGAAGCGAATACCGAAGCAGCGACGAACCTCGGCACAACAGCCAAAACCGTATTCGGCGACAACGGGCACACAATAGCAGAAGGCAACAACGTATGCTACCTCACAGCCAATCTAGCAACTTACGCAGACACCGCCGTAAGCCTGCTAGACGGGGCGATCAAAATACAAACCGCAGGCGACATAGGCAACAGCCAGAAGTTCGAGACAAACAGCCTACAAGCTCCCTTCATAAAGCCCATTGCCGACAACTACGACGAGATCGGAGAAGACTTACAAAAATCGGCAGACAAGATGCCCACAACCGACGCTGAACTACTCAATTTCCTAAAACAGTACAAAACGAATAACAAACTTAAAGAGGCGGCGGGCAAAGTCAATAACTGGGACTCGAACAAACCTGACGGCGAAAAAGCAGAATATCTCAAAACGATATTTGGACTTAACGAAGCCGGTACTGAAAGTGAATTCGTTACTGCTCTAAAAGCGACAAAGCGGACTGTAAAAACAGGAAAATCAACCAGTGCTGAAACGCCAATACTAAAGATGACCGACGACCAACTAAGAGAAGCAACAGAAGCCGCACTATCAGAGCTTAAACTAACAGCGACCCAAAACACTAACTGTCCGACGAAACAGCTAGCAGCAAAAGCAGAAGGTCAGAAAGACTGTACCAAAAATACGAAAAAGATGGACTGCAAAGACGGCGATGGTTGCAAATGGACTAACGAGGACGAAGAAACAGGAAGCCACTGCAAAGCTAAAAACGATGGAGAGGGAGTAAAAACAGAGAATGAAGGAACAACAACCACAAGCGCCACAGGGAACAATTCTTTTGTCATTAAAACTTCCCCTCTTTTGCTTGCAGTTTTGCTTTTCTAAaacaaattttcctcctcaatttttaaaaagaattttcctacttgaaaaactttctgatatattttaacacgcg

EATRO 1125.4710

MCDYSKQAKGQHPHLKAKLA

TAKNNAAAAFKGHRQQSLAM

LIVPQHSAASQILAAYAASL

MAESLGHIEQAATTAVTAVA

QASYSAGRTDELLKLLKSME

DGSSNDKWCIATTGGAAKAT

NNPTEGCEAQISEANTEAAT

NLGTTAKTVFGDNGHTIAEG

NNVCYLTANLATYADTAVSL

LDGAIKIQTAGDIGNSQKFE

TNSLQAPFIKPIADNYDEIG

EDLQKSADKMPTTDAELLNF

LKQYKTNNKLKEAAGKVNNW

DSNKPDGEKAEYLKTIFGLN

EAGTESEFVTALKATKRTVK

TGKSTSAETPILKMTDDQLR

EATEAALSELKLTATQNTNC

PTKQLAAKAEGQKDCTKNTK

KMDCKDGDGCKWTNEDEETG

SHCKAKNDGEGVKTENEGTT

TTSATGNNSFVIKTSPLLLA

VLLF*

>node_4607_length_1614_cov_8.87043_g4350_i0 PC2_A

aaacctgcacagtctgtacaaactgacagagcaaaccgcagATGCAAGGTAAGCCAACAATAACAACATCGAAACTGCTAGCTTTGGTAGTATTAGCGGCAGGGTGTACACTGAGTCATAACGCATCAGCGGCGCCGGCGGACGAAGGAACAAACAGTCAAGTCTTTGCTCTTCTCTGTGAGCTAGTAAGGGCGGCAGCAACGACCACTCCGAAGCAGCCAGCAGCGCAGATCAATGCACAAACCAAGAAAACAGCAACTCTAATTAAGCTGCTCATCGCCGACAGCACCGTAATATCGCAGCTAGCCCACACTGACGACGCTGACAGCATAATAGCAAAGGCCGGCTCGAAAGCGACCGAAATGTGCGGTGGAACCAACAGGGAAGACTGTATCAACGCGGCAAACCATCTGAGGGAAATAAAGAACAAGCAGGAGGCAACGCTCATAGCGAAACTGAGCGAACCGTCACTTATCCGACAAAGACTAGCCGAGACAGCGACGAAACTGACAAAACTGATAGAAGACTCGGAGAAAAACATGACGTGCACCAACGGTTTTTCGTACGAGAACATCATGAACAAAGCGTTGATCGGCAAACAGCAAGGCGGCAACAATTTCAGATTACACATGCCGGGAACCAACAGGCAAACTGCCTGTGGGCAAGGCCCAAATACGGCGGGGGCAACGGCGGGCATGTCGATTGCGGACGACTTGCTTTGTCTTTGCGCGTCTGAAACAGGTCACGAAAATAACAAAGGCTGCGACAGTGACAGCGGCGAAGCGGTAGACTTCAGCCAACCACATGATAGCCAGGGGACCGAGTGGGAGCGCCTGCACAGCTTATGTATCAAAAGAACGCCCCATAACATAGCCAAAACGGGCAGTGACCTGCGTAATTTACTAGGCAAAGTAAAGCAAGCTATTGCGGCACCCCAAGCCAACGACAAGAAAAACGGCTACCTAGGGACCCTAAAAGACGGAGGAACCGCGGGCAACTGCGACGGGACCAATACAGCAGGAAGGGGCGCATGCGCTTTCTATGGCAAGACAACAGACGCAGCTGCCGGACCAGCATGGCTCAAAACGGTCGAAGAAGCAGCTGACTGCCTAGATCACCTAAAGCAACAACAGCAATCAGCATTACTACGACAACAAGAGATACAGCTACTTAACAACAGCCTAACAGACCTGCTGCACTTACACAGCAATGCAGCCGGATCCAAGCCGGGCGCTAAGGAGGAGCCAAGTCTGCGCTCCGATGGGATACAGATAAGCGACGCCACTCGGAGGTGCGCAGCAGCTGAAGATAGCAAAGACGAATGTGACAAACTGGCGAAGCATGGCTGCGTTTATAACCAGCAAGGAGATAGTGGAAAAAAGTGTACATTAAAGCCTGAAGCACAAGCACAACTCGAAAAGTCAAGCCAAGAAACAGAAAGCAAAACTGGATCCACAAACACCACACAAAGCAGCAATTCTTTTGTCATTAAAACTTCCCCTCTTTTGCTTGCAGTTTTGCTTTTTTAAaacaatttttccccctcaaattcccctctcttttcaaaaatcttccttgctacttgaaacctttctgatatattttaacaccttt

EATRO 1125.276

MQGKPTITTSKLLALVVLAA

GCTLSHNASAAPADEGTNSQ

VFALLCELVRAAATTTPKQP

AAQINAQTKKTATLIKLLIA

DSTVISQLAHTDDADSIIAK

AGSKATEMCGGTNREDCINA

ANHLREIKNKQEATLIAKLS

EPSLIRQRLAETATKLTKLI

EDSEKNMTCTNGFSYENIMN

KALIGKQQGGNNFRLHMPGT

NRQTACGQGPNTAGATAGMS

IADDLLCLCASETGHENNKG

CDSDSGEAVDFSQPHDSQGT

EWERLHSLCIKRTPHNIAKT

GSDLRNLLGKVKQAIAAPQA

NDKKNGYLGTLKDGGTAGNC

DGTNTAGRGACAFYGKTTDA

AAGPAWLKTVEEAADCLDHL

KQQQQSALLRQQEIQLLNNS

LTDLLHLHSNAAGSKPGAKE

EPSLRSDGIQISDATRRCAA

AEDSKDECDKLAKHGCVYNQ

QGDSGKKCTLKPEAQAQLEK

SSQETESKTGSTNTTQSSNS

FVIKTSPLLLAVLLF*

>node_4140_length_1662_cov_12.2458_g3956_i0 BS_2098

taggccttATGGCGTTATCCGTGCTCGTGCAATCAGCGCTGGCGACCGGAAACGCGGGCAAGGCGTTAACGCACGGCACTTGGACGCCCATATGTGAGATGACAACGAATCTAGCAGCGGTCTTCAACGAACAGTCACAGAGCCTAGCGCAGAGCTTAGAAAACAGTGAAGACAATCGAAAGCTCGCTCTTCAACTAAGCATTTACGCCTCCCAGGCGGAACCGGCCAAGAGGCTGAAGCTTTTACCACTAATAGCGGGCATAGCGGCCAAAACGGCAATGCCAACTGACACAACCACTCAAGGCGTTAAAATGGCACCGGCAGCAGCGCAAGCTACTTCTTTCGTAAGGGGACGCCTAGCCGAATTCATCTCGATCGCAGCGGACAGCTACTCAACCTCAGGCACACACGGCTGCCTGGAGAAAATCGCAGGCACAACAACAGTAGAAGGGGCAACCACACTGGCGCAATGTGCACTAGCAACCGACACAACAGGCAAAGTACATCCAAAAACATATAGCGGCGACCCAGACAAGCTATTTAAACCAAGCACAAGCGACCATGCAGCAGCGGCCCGGATGGCCGGCACGCCCACATGCAACATCTTTAAAACCGCGGCAGGCAGTCTCGTAGAAACAGCCAGCAACCACAAGGATATAACCATGGGAGCCGGGTACCTGACACTAATACAAAGCGACGACTCGATCAAACACGCACCCTTCGGGACGTTCGACGCCGCAGACGCAGGGACGACACCAAAAGACATGCACGCAGCGCAGGTCGCGCAGAACAACTGGAGAACAAGCGGCCTTTTAACTGCCGCAAAAAAGATCGACCCAGACAGCGCCGATATAACGACATCCACAGAGTTCAAAAGAGCCGTCAAAATTTTTTTCTTAAAACAAGCAGGACATTACGACGCGAAAACAGACCAAACAACGGTAGACGCCGCCATCAAAGACCATTACGCCACCGACAAAGACCTCAGCCTCAAAAAAGTATGGAAGGAGATGCAAGCAGCGACGATTCCCAAAGAGCTGGTAAGCCCAACAGCTCAAGAAGACCCAAAGCTAATTTCTCAGACTAATACCAAGCAATTGGAGTTAATACTCCTTCACTACCAAGCAAAAGCGGCATTTGAGCTACTTGAACAGCAAGACAAAATTAAAAATTTTGCAACAAAGTCAGTAACCGACGACAGAATCTCACAGACTCAGAAAGAGTGTGCAGCACACCACGCGGACAAAAATGCCTGCGATTCCAAGGATTTCTGTACTTATGATGAAACAGAGAGTACAGATAAGAAGTGGAAATACAATGCCACCAAAGCCACTAAAAACGGCGTCCCTGTAACCTAAactcaaactgcaggatcaactgacgagggtgtaaaatgctccgatcataaagaccaggcgacctgtgagaaggccaatgaaggtaagactaccaaagtttgtggctggaaaggagaaaatactgatggatctgacaaatcaggttataaatgccatgattacagttttctagtaaacaatcaattcgtcctcagcgtggtttctgctgcctttgtggccttgcttttctaaaacaaatttttcccctctctttttattaaaaaaatttttgataaattcttgctatttgatatattttaacacc

EATRO 1125.4040

MALSVLVQSALATGNAGKAL

THGTWTPICEMTTNLAAVFN

EQSQSLAQSLENSEDNRKLA

LQLSIYASQAEPAKRLKLLP

LIAGIAAKTAMPTDTTTQGV

KMAPAAAQATSFVRGRLAEF

ISIAADSYSTSGTHGCLEKI

AGTTTVEGATTLAQCALATD

TTGKVHPKTYSGDPDKLFKP

STSDHAAAARMAGTPTCNIF

KTAAGSLVETASNHKDITMG

AGYLTLIQSDDSIKHAPFGT

FDAADAGTTPKDMHAAQVAQ

NNWRTSGLLTAAKKIDPDSA

DITTSTEFKRAVKIFFLKQA

GHYDAKTDQTTVDAAIKDHY

ATDKDLSLKKVWKEMQAATI

PKELVSPTAQEDPKLISQTN

TKQLELILLHYQAKAAFELL

EQQDKIKNFATKSVTDDRIS

QTQKECAAHHADKNACDSKD

FCTYDETESTDKKWKYNATK

ATKNGVPVT*

>node_501_length_4594_cov_8749.46_g485_i0 BSNdiff

agagtgttgtgagtgtgtgtatacgaatattataataagagcagtaataataatactaataataataataatagtgataataataggagagtattgtgagtgtgtgtatacgtatattataataagagcagcaataataatactaataataataataatagtaataataataattggagagtgctgtgagtgtgtgcatacgaatattataataagagcggtaataatcataataataatgatgataataatagtaggagagtgttgtgagtgtgtatatatacgaatattataataagagtagtaacaataataataggagagtgtttgtgagtgtgtatatacaaatattataattaggtgattctgaaacttgaatgtagtggtaaaatataagcacttaaaacattagaaggggtaaaattaaagtaatagagtgagtaaagaaataagtacgaggaattgggaaagcatgaggaattttaaaagaagagcatgaaatgaagtaaatagaatatgcgtagtgccaaacaaaaatatgattatgggaatcactatacacttgatgaaatgggggatatgaaatagacaaaaagagtctagtgtacattccaattagagccgttacattatacacagaaacagcggaaaaagtatataacgaaggtaagaaaacacaaaatacaacagttgattaaacgtcagaaaaatagtaatttttaaaatgtatacaggaagaggtcagtagttacccttatttcacacagaaatggatggctgctattgcaaagatgactgtaacaagaacattaagagaagtaattaatgaaactattgttatgaaagtcttataatcaaaaatgatgttgggactgtcatgacagtaaactagtgatgttcttagtacatctgaaccccatattgtacatgtatactacaattaccatagcaattaataaaaacagcaatgtatagaagacacggaaacagctgaatgcgtgtagtacgctgcagcagctgtgactcaagctaacgtttataacaaaacacgcaaatgcatacacatatgttgcctttagatattgagcatacacccttaaacagatgtatcatagaggtatcaaataatttcttatacatgtgtaggaaaggattcatcggctaagtatcttagtggttcaaccgacttctcttgttaaactcttttttcctcatagtagtttttcaaaatttaaaggtttttttgcgtaaagatatttgttatcttgagatctttgctattactcttgtcactggtattacttctggcgtatttaagcttaataactattcaaaataataacacaaatgaaacaacaaatataacgcataataagcataagaagccaaatgtgtttgtgtttctagttcaaactcatacaacaaaaagatcctgtaagctaatattgagtgcgaaactgagccatatttaaattattacaattgcgatgggaggtttgtataatcatgttaagaggcctagatgggttctgaaatttttagaaaaacagaatgcaagtgatgaggatattgtaattcatttggatggatctgatgttatagttagtgatggggagaagtataacaatgtagtaaaatattttatacaaaatactgctgaaaatgaagagaagtttagtgtagatgttatttacaaagaagatgcagagagatgttattggttttttaattttagtcacagaaaataagagtataagaagtcacctcacttactaaagtcacttcctagtggaaaacagtatcttagtggcagtgataaaatagcaagagtttgggaaaattataaatttaatatgcatttgggaattattaaatggaagagaagaattgtagtcagatcagataatttacaaacctttattaatttggagtgcaaatcaggaagaagctgttggacagagatttgttttgcggagagggataattgggtgtggttatgaggagagatttttatactattccacgcagtggggtgattggagaagctctattcatttattttccagttcagccaatttcgtggaaagagaaagcgaatttaattgttagaaatcatacagagcacggtcaggtggtataaagagttgaaaatgtggaagtatggttcggtggacttgaataaaattcgatcttatgttctaaaaaaagagtaatcgaatattgaatatggacaggttaatggtcatgtagtaaagaagaaaatttgtttaaaacgaacaaattgtaggaagcggcagaacaataaagtaacgataacctcgtgtaggcacggcgaaaagttataatatcacttaaacacaaagcctgtcaccgcattttttaagcataagtggaaaacctgaacgacgtatccagctgtgaagctacacagacagccacctagcgcaaactgaagtgacacaaaagtagtgccacaaatagcagtaaataaagaaaaaggcatgaatATGTTAATTATTAGATTTCTTGCTCTGTGTATACTATTAAGGAATGCACATGCTGCAAAGATCCAACACGCAGCTGAATTCGATGCTCTATGCAACTTGTTCACATTAAAAGATGCGATCGCACCGTCAGTTGCCGACGAACAAATTGCAACTTTCTCGGACCTAAGAACGCCAATCTACAACCTCAATGTATCAGTAGCAGATGAAGCCTTCCTAAAAGATGCGAATGGAAAATACAAGGTTCTGATGCAAGGGGCCGATGACGCGGCAAAACTAAGAGCGTGTCAACAGCACATAACAGAGATAATAAACTCAAAACATGGGCCTGAAAACAAAAATATATATGCCAAGCTCTCAAACCAAGCACAGAAAGCATCAGCTCGAGCGATCATAACAGACCTGCTCAAGGAGGCAGACAAACTTGGCAACAAATACGAATAAccgcactcgataaacgagcacaaccaaataacgtgttattttagcttctagtgaaacaagcctaattcagtattctacttttatgccgaggtaccaaagactgggtaaaaaataacaaacctacagctagccaagtacactcaacacaaaagcaATGTTGCCACCTGTATTTGCGGCGTCTTTATACCTAGCTTTATTATCACCAACCGCACACGCGGCAGCAGGCGACAATATCAACGAATTTACAGTAATGTGCAAGCTCTTTACGGCCTGCGAACAAGGCATCTCAGCAAACGCCGCCGCCACACCGAACTCACTTGACGACGAACTGGAAACAATGATTGCAATAAACTTGACAGCAGCAGACAAGTCGTTTTTTGAGCGAAACTTCGATACCAACACAACACACGATACAGATGGAAACTACACAAAATACAAGACAACCTGGGCAGCGTTAAAAAAAGATCTAGGATCGAAAAAGATCGGGACCTTTGACCTCAAATTAACGAGACTCCCAGACAGCGCGCTAAGGGGCTATGTTCAACGGGCAGCCTTGGCCGCCATCGCAGCGTTAACATCAGCCAAAGACCAGCTAAGCAAAGGCCCCACTTCACAAACCATAAACAACAAACTCAAAGAGGTACTGTACGGCGACAAACAAGCAGCAGCTTCAGCAGATGCAGACAAGACATTTGGGAGCACGCTAGCAGCAGCCTGCGGAAACAACGGCGCTGCGGAGAACAAAGTAGGGATTTCCCTAAGCAACGATTTAGTGTGTCTATGCGGCGGCGGCAACACCGCAATAGCTGCATGCGCAGGCACCGGCGCAACCCTAGCCGGCGGACAAGCATTCGCTGCAGGCAGCCAAACAGGGACCCCGGCACTAACCGATGTGCGAGCAAAATGCCACGACAAGAACAAAGGACCGACATCAGGCACAGAGCTGCAGGCTCTTCTCACAACATTCGAATCCATGATCGGCGCGCGCGACGGCACAACAAGCGGCGCCGAAGCAAACTACGGCAAAGGCGCAGCTGCAACTTGCAACGCCGGCGACGGCAACAGCTGCGTCAACTATAAATACCAACTAGGAGCGGCGGACAGGGGCATACCATGGGTGAAGAAACTAAAATCTGCCATAGCGGACATAAAAGCTCTCAACACAAAAATCGCCGAAGTAAAACAGGTACAAACCAAAGTCAAAAACATGGAAGAGAAGTTACTTCTGACGTACATGGCAGCGCAAGCCATAATAACTGACTCGACTAAAACGCAGCAAAAAGAAGCGCCAACCAGCGCGGCAAACCCAAGGACACAAACAGCCATATGCAAACCACAAAACAAAACCCCAGCAGAATGCCCAAGCCAACACTGCGACTATAATGAAAAGGCCACGGATGGTAACAAATGCAAAGCTAAACCAGGAACAGAAACTGCAGCAGCAGGAACAGGAGAACCACCTGCAGGAACCAGCACAAACAACACAGGAACAACAATTCTTTTGTCATTAAAAAGGCCCCTCTTTTGCTTGCATTTTTGCTTTTCTAAAAGAAAATTTTCCCCCTCTTTTTCTTCCTTGCTAAAATTTTTGCTACTTGAAAAACTTTCTGATATATTTTAAcacctttaaattccccgaaaaaaaa

EATRO 1125.224

(ORF may be extended - error affecting the termination codon?

MLPPVFAASLYLALLSPTAH

AAAGDNINEFTVMCKLFTAC

EQGISANAAATPNSLDDELE

TMIAINLTAADKSFFERNFD

TNTTHDTDGNYTKYKTTWAA

LKKDLGSKKIGTFDLKLTRL

PDSALRGYVQRAALAAIAAL

TSAKDQLSKGPTSQTINNKL

KEVLYGDKQAAASADADKTF

GSTLAAACGNNGAAENKVGI

SLSNDLVCLCGGGNTAIAAC

AGTGATLAGGQAFAAGSQTG

TPALTDVRAKCHDKNKGPTS

GTELQALLTTFESMIGARDG

TTSGAEANYGKGAAATCNAG

DGNSCVNYKYQLGAADRGIP

WVKKLKSAIADIKALNTKIA

EVKQVQTKVKNMEEKLLLTY

MAAQAIITDSTKTQQKEAPT

SAANPRTQTAICKPQNKTPA

ECPSQHCDYNEKATDGNKCK

AKPGTETAAAGTGEPPAGTS

TNNTGTTILLSLKRPLFCLH

FCFSKRKFSPSFSSLLKFLL

LEKLSDIF*

>node_7963_length_799_cov_3.88038_g7534_i0 BSNdiff

aaacgcatacaaagctatcgcgacgatagaaacagcagaatacgtaaacagcacggcggcgccatcaagccgcccttgtctaaagcaagtaattaagctcacgcatttagacaaaacaaaaacctccgatggcgatgttaagcaagcagtccaaagctggataggcggcgacgacgacaagaaaataaaagagatcgaagcggcggtagacaaggaggagataccgaaagatatagcccacttatcacagccaacagtcctcagcagcataacttcaacagaacaactcaacgcgatactttaccattaccaaaatatagcactgcaaaagatcgtcaagttgcaggaggagttatccgaggcacagaacaaaaaagatccaaaaacagcagcagacatttgcaataaaattaaggacgcaacagaatgcaacaacaagcctttttgcacctataacacaacagaaactgacgaaaataaaaagtgcaaatttgatgtgacaaaagcctctaaaaacggggtccccgtaacacaagcccaaactggaggaggaactggaggatcccaaactacttcagaatgcaaagatagacagcagaaagattgcactgaaaattgcaaatgggaaggcgaaacttgcaaagattcctctattctagtaaacaagcaattcgccctcagcatggtttctgctgcttttgtggccttgctgttttaaaaatttccctcctctttttaaaattttccttcctaaataattcttgctacttgaaaaacttttgatatattttaacacgta

(longest ORF is 163 nt)
