# Supplementary material for: Expression of the RNA-binding protein RBP10 promotes the bloodstream-form differentiation state in Trypanosoma brucei
Source: PLoS Pathog. 2017 Aug 11;13(8):e1006560. doi: 10.1371/journal.ppat.1006560 (PMC5568443; doi:10.1371/journal.ppat.1006560)
Supplement: S1 Fig — A. LambdaN-RBP10 expression in bloodstream forms: Western blot showing inducible (24h) expression of full length lambdaN-RBP10 or six different fragments (F1-6) of RBP10 fused with lambdaN peptide on the N-terminus and a myc tag on the C-terminus. Samples from two independent clones are shown. B. Characterisation of bloodstream-form trypanosomes with a sequence encoding an N-terminal tandem affinity purification tag integrated in frame with the RBP10 coding region. The upper panel is a western blot showing the presence or absence of TAP-RBP10 and native RBP10, with genotypes above the lanes. The lower panel is a cumulative growth plot for the cell line used in the interactome experiments. C. Scatter plot showing average reads per million for bound vs. unbound RNAs. The most prominent enriched spots are labelled, with functional designations where available. "AT" = putative adenosine transporter. This plot does not allow for variations between samples, so some labelled mRNAs may not be in the final enriched list. One spot that was clearly less in the bound fraction is also labelled. The plot shows that many mRNAs were enriched in the bound fraction but only one (labelled) was more than 4-fold depleted. D. Analysis of binding of individual mRNAs to RBP10. The transcripts were split into groups based on the ratios of bound/unbound reads per million reads; the number of different open reading frames in each class is on the y axis. E. RNAi cell line: Western blots showing the time course of RBP10 decrease in Lister 427 bloodstream forms after tetracycline addition F. RBP10-myc expression in procyclic forms: Western blots showing the time course of expression of RBP10-myc in Lister 427 procyclic forms after tetracycline addition. (PDF) [file ppat.1006560.s005.pdf]

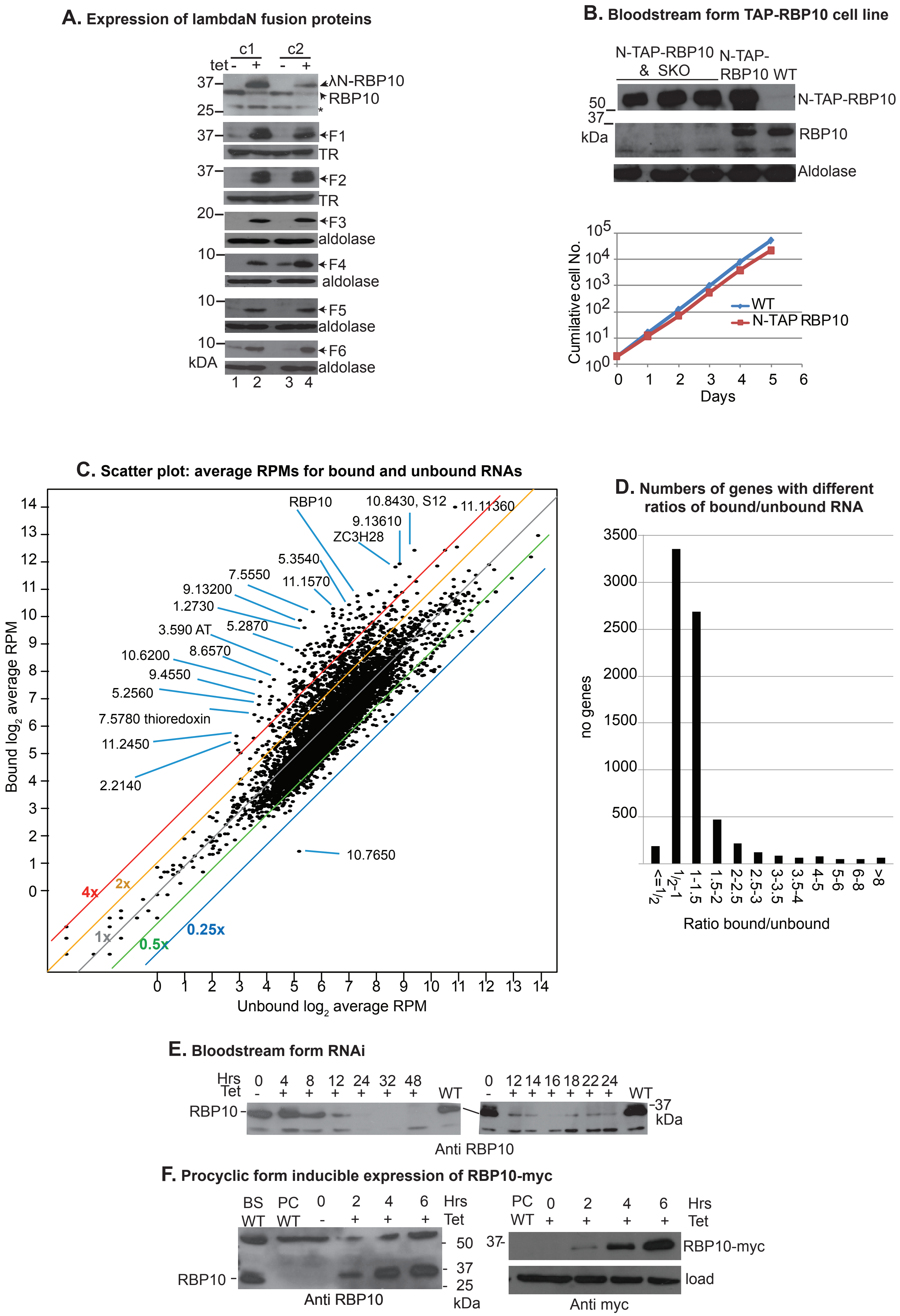
A. Expression of lambdaN fusion proteins
c1
c2
tet - + - +
37
25
λN-RBP10
RBP10
*
F1
TR
F2
TR
20
F3
aldolase
10
F4
aldolase
F5
aldolase
F6
aldolase
kDA
1 2 3 4
B. Bloodstream form TAP-RBP10 cell line
N-TAP-RBP10 & SKO
N-TAP-RBP10
WT
50
37
kDa
N-TAP-RBP10
RBP10
Aldolase
Cumilative cell No.
Days
WT
N-TAP RBP10
C. Scatter plot: average RPMs for bound and unbound RNAs
Bound log2 average RPM
Unbound log2 average RPM
RBP10
10.8430, S12
11.11360
9.13610
ZC3H28
5.3540
7.5550
11.1570
9.13200
1.2730
3.590 AT
5.2870
10.6200
8.6570
9.4550
5.2560
7.5780 thioredoxin
11.2450
2.2140
10.7650
4x
2x
1x
0.5x
0.25x
D. Numbers of genes with different ratios of bound/unbound RNA
no genes
<=1/2
1/2-1
1-1.5
1.5-2
2-2.5
2.5-3
3-3.5
3.5-4
4-5
5-6
6-8
>8
Ratio bound/unbound
E. Bloodstream form RNAi
Hrs 0 4 8 12 24 32 48
Tet - + + + + + +
WT
0 12 14 16 18 22 24
- + + + + + +
WT
RBP10
-37
kDa
Anti RBP10
F. Procyclic form inducible expression of RBP10-myc
BS WT
PC WT
0 2 4 6 Hrs
- + + + Tet
50
37
25
kDa
RBP10
Anti RBP10
PC WT
0 2 4 6 Hrs
+ + + + Tet
37
RBP10-myc
load
Anti myc
