# Supplementary material for: Expression of the RNA-binding protein RBP10 promotes the bloodstream-form differentiation state in Trypanosoma brucei
Source: PLoS Pathog. 2017 Aug 11;13(8):e1006560. doi: 10.1371/journal.ppat.1006560 (PMC5568443; doi:10.1371/journal.ppat.1006560)
Supplement: S2 Fig — Sequence alignment was done with the Megalign Pro application of DNAStar. using MUSCLE [131], except that a single gap was introduced to make the last four amino acids from T. brucei and T. congolense match T. vivax. The sequence IDs and colour code are given at the top of page 1. The RNP1 and RNP2 motifs from the RRM are indicated (red), as are mapped phosphorylation sites (outlined text). A purple bar show the position of the C-terminal region characterised in Fig 1. An evolutionary tree with uncorrected pairwise distances is at the bottom of page 2. (PDF) [file ppat.1006560.s006.pdf]

A. Sequence alignment

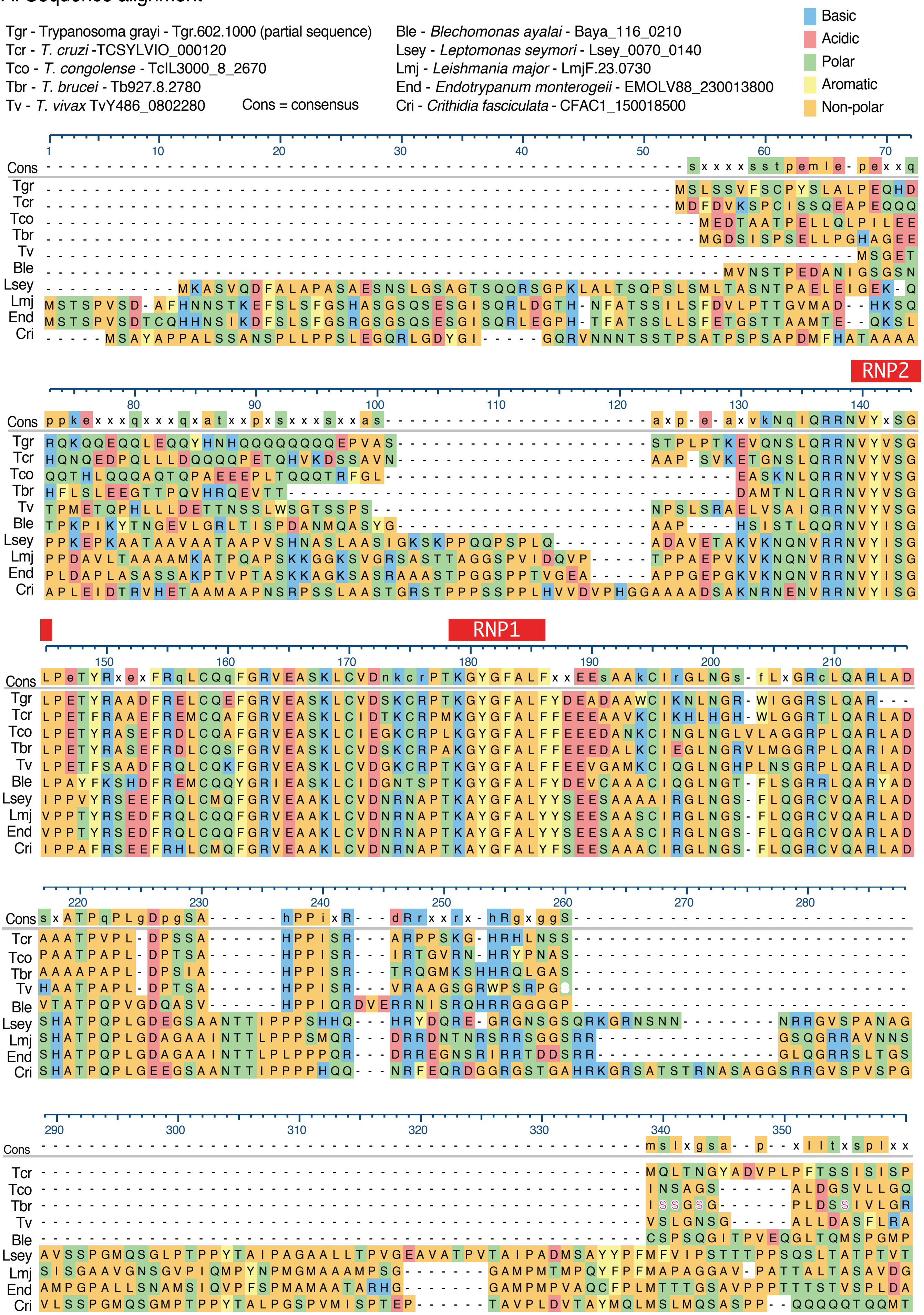

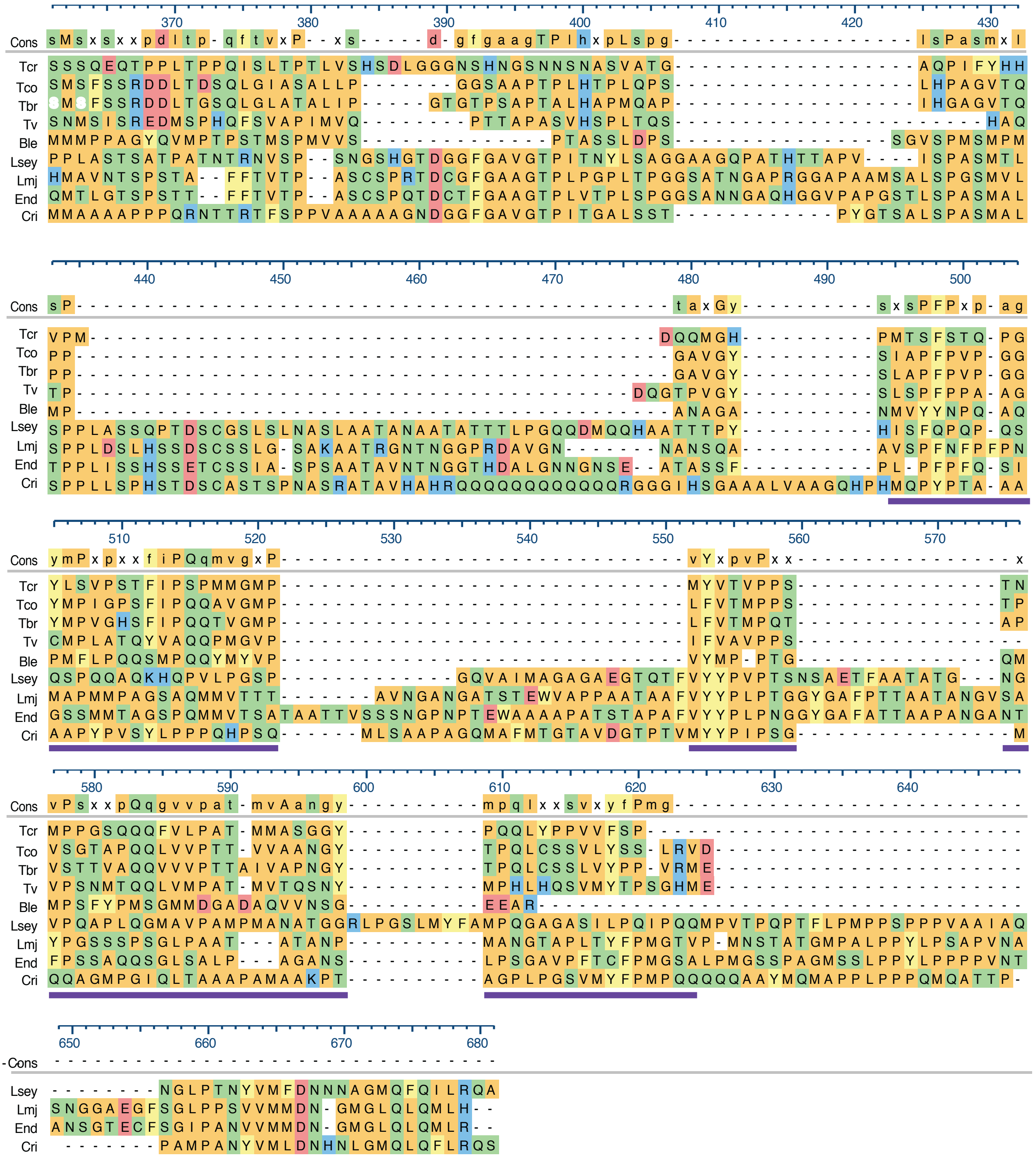
370 380 390 400 410 420 430
Cons sMsxsxxpdltp-qftvxP--xs-----d-gfgaagTPlhxpLspg------------------lsPasmxl
Tcr SSSQEQTPPLTPPQISLTPTLVSHSDLGGGNSHNGSNNSNASVATG------------------AQPIFYHH
Tco SMSFSSRDDLTDSQLGIASALLP------GGSAAPTPLHTPLQPS------------------LHPAGVTQ
Tbr SMSFSSRDDLTGSQLGLATALIP-----GTGTPSAPTALHAPMQAP------------------IHGAGVTQ
Tv SNMSISREDMSPHQFSVAPIMVQ--------PTTAPASVHSPLTQS---------------------HAQ
Ble MMMPPAGYQVMPTPSTMSPMVVS-------------PTASSLDPS-----------------SGVSPMSMPM
Lsey PPLASTSATPATNTRNVSP--SNGSHGTDGGFGAVGTPITNYLSAGGAAGQPATHTTAPV----ISPASMTL
Lmj HMAVNTSPSTA--FFTVTP--ASCSPRTDCGFGAAGTPLPGPLTPGGSATNGAPRGGAPAAMSALSPGSMVL
End QMTLGTSPSTT--FFTVTP--ASCSPQTDCTFGAAGTPLVTPLSPGGSANNGAQHGGVPAPGSTLSPASMAL
Cri MMAAAAPPPQRNTTRTFSPPVAAAAAGNDGGFGAVGTPITGALSST-------------PYGTSALSPASMAL
440 450 460 470 480 490 500
Cons sP---taxGy----------sxsPFPxp-ag
Tcr VPM--DQQMGH----------PMTSFSTQ-PG
Tco PP--GAVGY----------SIAPFPVP-GG
Tbr PP--GAVGY----------SLAPFPVP-GG
Tv TP--DQGTPVGY----------SLSPFPPA-AG
Ble MP--ANAGA----------NMVYYNPQ-AQ
Lsey SPPLASSQPTDSCGSLSLNASLAATANAATATTTLPGQQDMQQHAATTTPY----------HISFQPQP-QS
Lmj SPPLDSLHSSDSCSSLG-SAKAATRGNTNGGPRDAVGN-------NANSQA----------AVSPFNFPFPN
End TPPLISSHSSETCSSIA-SPSAATAVNTNGGTHDALGNNGNSE--ATASSF----------PL-PFPFQ-SI
Cri SPPLLSPHSTDSCASTSPNASRATAVHAHRQQQQQQQQQQQQRGGGIHSGAAALVAAGQHPHMQPYPTA-AA
510 520 530 540 550 560 570
Cons ymPxpxxfiPQqmvgxP-------------------------------vYxpvPxx----------------x
Tcr YLSVPSTFIPSPMMGMP-------------------------------MYVTVPPS---------------TN
Tco YMPIGPSFIPQQAVGMP-------------------------------LFVTMPPS---------------TP
Tbr YMPVGHSFIPQQTVGMP-------------------------------LFVTMPQT---------------AP
Tv CMPLATQYVAQQPMGVP-------------------------------IFVAVPPS-----------------
Ble PMFLPQQSMPQQYMYVP-------------------------------VYMP-PTG---------------QM
Lsey QSPQQAQKHQPVLPGSP----------GQVAIMAGAGAEGTQTFVYYPVPTSNSAETFAATATG---NG
Lmj MAPMMPAGSAQMMVTTT-------AVNGANGATSTEWVAPPAATAAFVYYPLPTGGYGAFPTTAATANGVSA
End GSSMMTAGSPQMMVTSATAATTVSSSNGPNPTEWAAAAPATSTAPAFVYYPLPNGGYGAFATTAAPANGANT
Cri AAPYPVSYLPPPQHPSQ------MLSAAPAGQMAFMTGTAVDGTPTVMYYPIPSG----------------M
580 590 600 610 620 630 640
Cons vPsxxpQqgvvpat-mvAangy-----------mpqlxxsvxyfPmg---------------------------
Tcr MPPGSQQQFVLPAT-MMASGGY-----------PQQLYPPVVFSP-----------------------------
Tco VSGTAPQQLVVPTT-VVAANGY-----------TPQLCSSVLYSS-LRVD-------------------------
Tbr VSTTVAQQVVVPTTAIVAPNGY-----------TPQLCSSLVYPP-VRME-------------------------
Tv VPSNMTQQLVMPAT-MVTQSNY-----------MPHLHQSVMYTPSGHME-------------------------
Ble MPSFYPMSGMMDGADAQVVNSG-----------EEAR--------------------------------------
Lsey VPQAPLQGMAVPAMPMANATGGRLPGSLMYFAMPQGAGASILPQIPQQMPVTPQPTFLPMPPSPPPVAAIAQ
Lmj YPGSSSPSGLPAAT---ATANP-----------MANGTAPLTYFPMGTVP-MNSTATGMPALPPYLPSAPVNA
End FPSSAQQSGLSALP---AGANS-----------LPSGAVPFTCFPMGSALPMGSSPAGMSSLPPYLPPPPVNT
Cri QQAGMPGIQLTAAAPAMAAKPT-----------AGPLPGSVMYFPMPQQQQQAAYMQMAPPLPPPQMQATTP-
650 660 670 680
Cons ----------------------------------
Lsey --------NGLPTNYVMFDNNNAGMQFQILRQA
Lmj SNGGAEGFSGLPPSVVMMDN-GMGLQLQMLH--
End ANSGTECFSGIPANVVMMDN-GMGLQLQMLR--
Cri -------PAMPANYVMLDNHNLGMQLQFLRQS

B. Tree

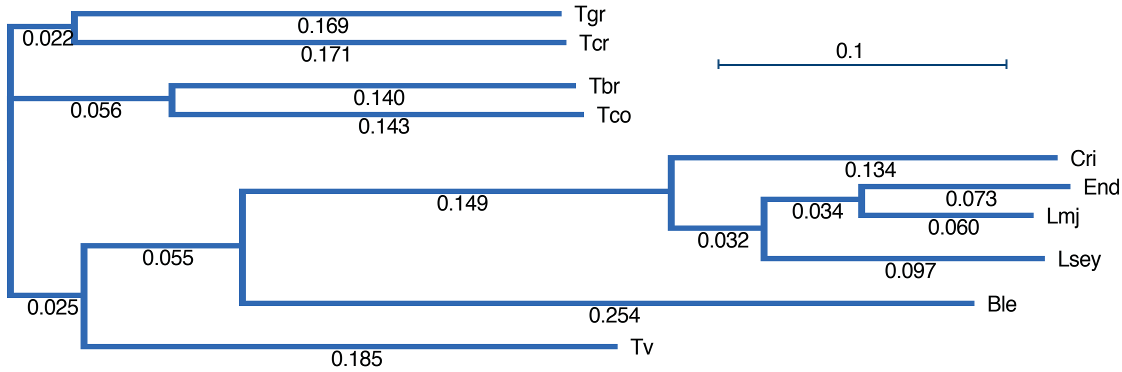
Tgr
Tcr
0.169
0.171
0.022
Tbr
Tco
0.140
0.143
0.056
0.1
Cri
End
Lmj
Lsey
Ble
Tv
0.134
0.073
0.060
0.034
0.032
0.097
0.149
0.055
0.254
0.025
0.185
