# Supplementary figures and images for: Expression of the RNA-binding protein RBP10 promotes the bloodstream-form differentiation state in Trypanosoma brucei
Source: PLoS Pathog. 2017 Aug 11;13(8):e1006560. doi: 10.1371/journal.ppat.1006560 (PMC5568443; doi:10.1371/journal.ppat.1006560)

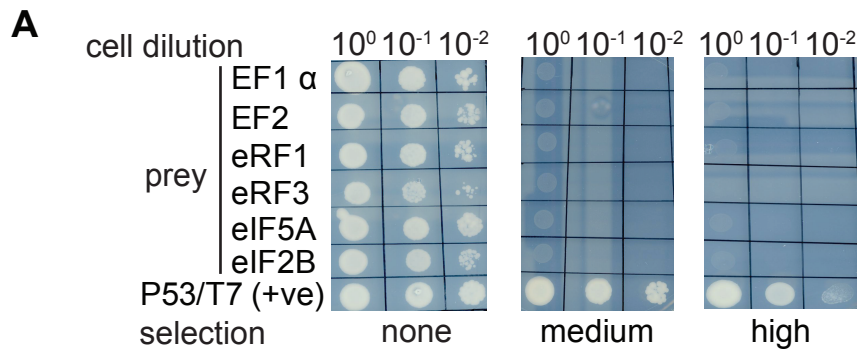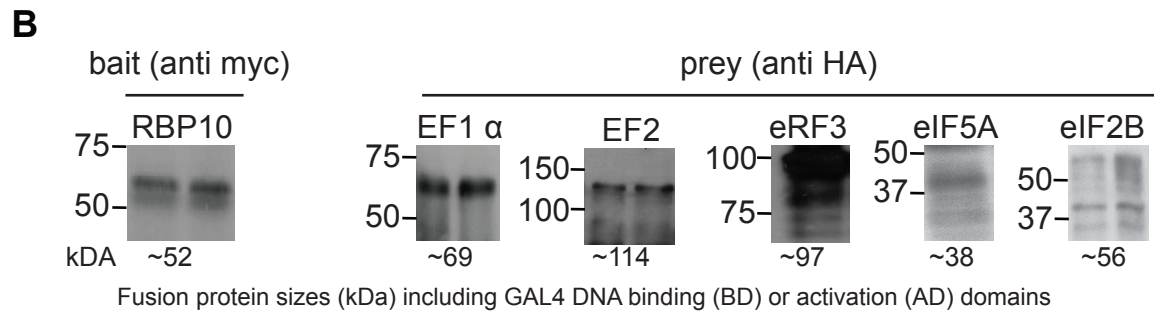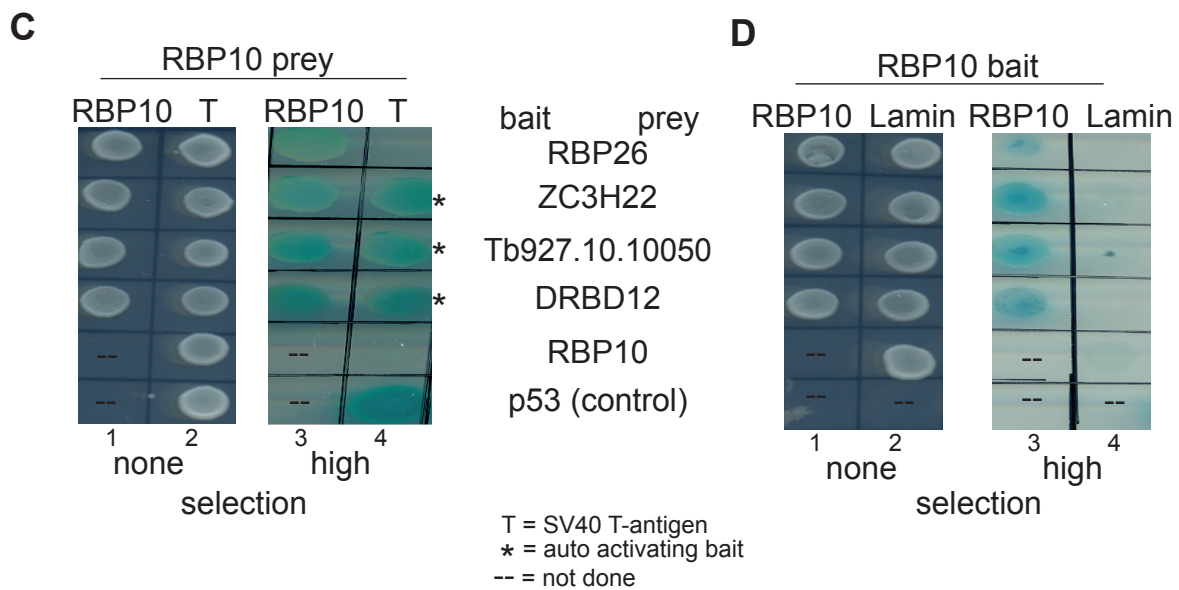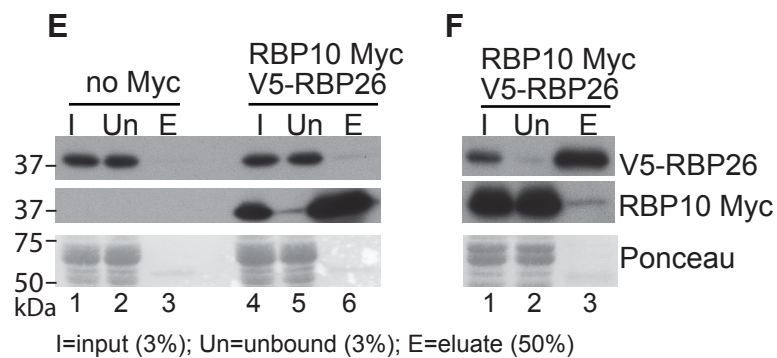

Supplement: S3 Fig — A, B: Pairwise yeast two-hybrid screen between RBP10 and translation factors. A) Plates showing interactions between RBP10 as bait and the translation factors EF1 α, EF2, eRF1 eRF3, eIF5A, eIF2B as the prey. Cells diluted 1:10 or 1:100 were grown on medium and high stringency nutrient selection media. P53 and SV40 T-antigen interaction served as positive control. B) Bait and prey proteins expression were detected using anti-myc and anti-HA antibodies respectively. C, D: Confirmation of the genome wide yeast two-hybrid data using full-length prey inserts. C) Plates showing pairwise interactions of RBP10 as prey and RBP26, ZC3H22, Tb927.10.10050, DRBD12 as baits. As a negative control SV40 T-antigen (T) was used as prey (lanes 2 & 4); the asterisk (*) highlights the auto activating baits. P53/T interaction served as positive control. The cells were selected on quadruple dropout medium (QDO); blue colonies highlight addition confirmation by alpha-galactosidase assay. D) Reciprocal interaction. As in (A), but using RBP10 as bait. Screening using Lamin (lanes 2 & 4) as bait served as a negative control. E, F: Co-immunoprecipitation of RBP26 with RBP10 (and vice-versa). E) Co-immunoprecipitation with α-myc beads using extracts from cells expressing V5-RBP26, with (lanes 4–6) or without (lanes 1–3) additional expression of RBP10 myc. The precipitated proteins were detected by Western blotting using anti-myc, and anti-V5 antibodies. Ponceau staining served as a loading control. I: input, Un: unbound (both 3% of the lysate) and E: eluate (50% of the boiled beads in sample buffer). F) Reciprocal co-immunoprecipitation using α-V5 beads. (PDF) [file ppat.1006560.s007.pdf]

**A**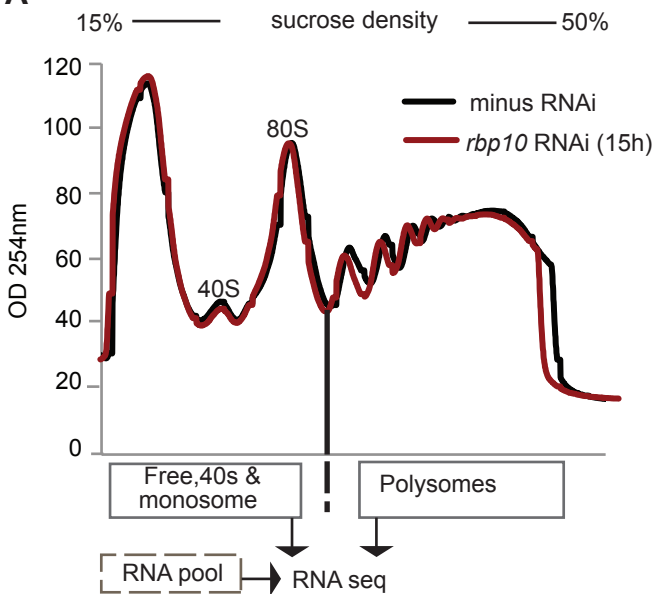**B**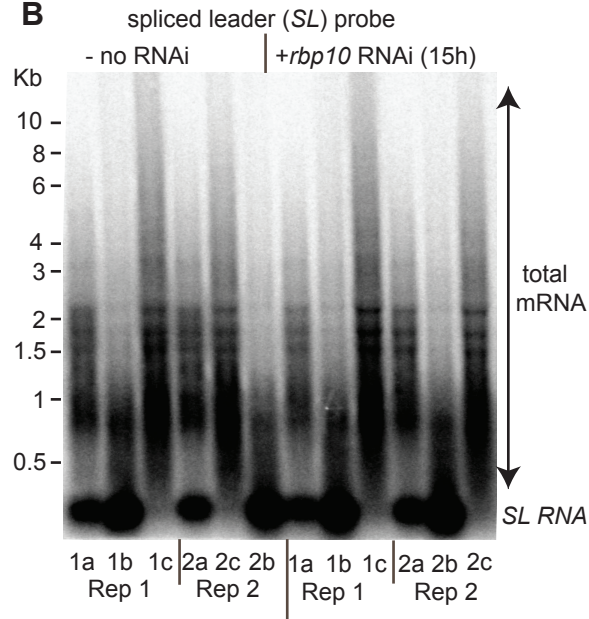**C**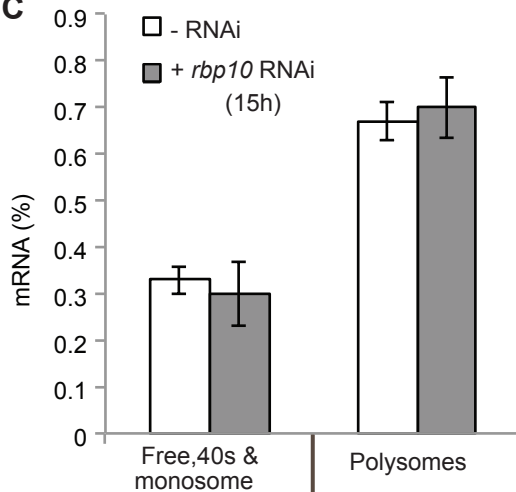

a = input  
b = subunits & monosome  
c = polysomes

Supplement: S4 Fig — A. Typical sucrose gradient profiles of samples. B. Total mRNA in the sucrose gradient fractions, detected using the spliced leader probe. C. Average quantitation of the spliced leader signal. (PDF) [file ppat.1006560.s008.pdf]

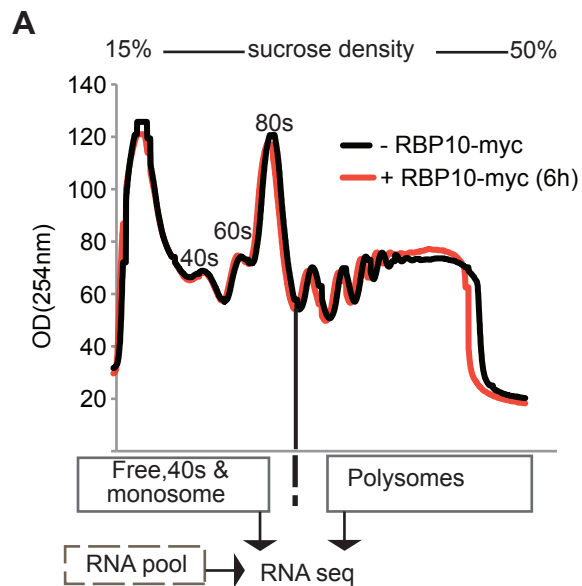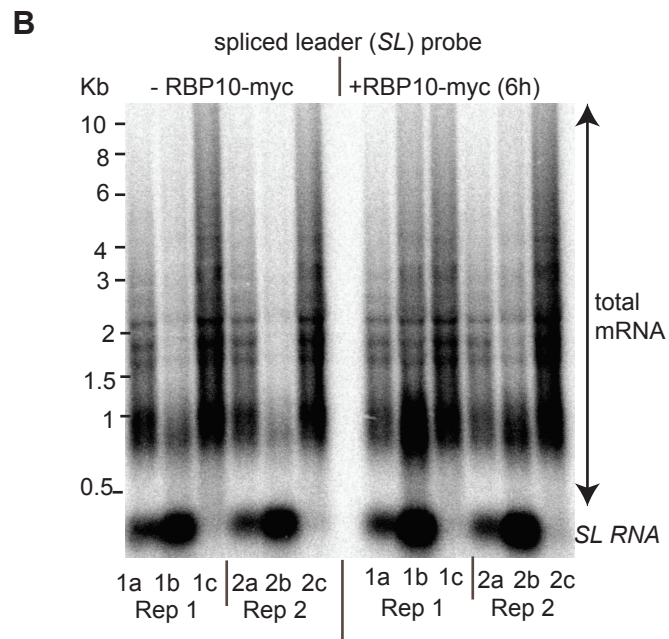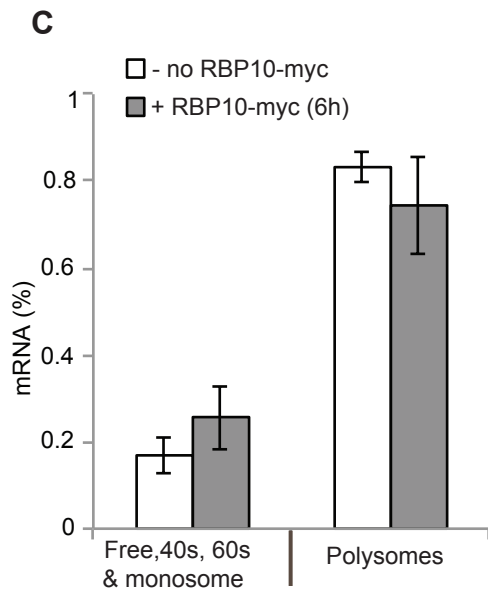

a = input

b = subunits & monosome

c = polysomes

Supplement: S5 Fig — A. Typical Sucrose gradient profiles of samples. B. Total mRNA in the sucrose gradient fractions, detected using the spliced leader probe, C. Average quantitation of the spliced leader signal. (PDF) [file ppat.1006560.s009.pdf]

**A Summary**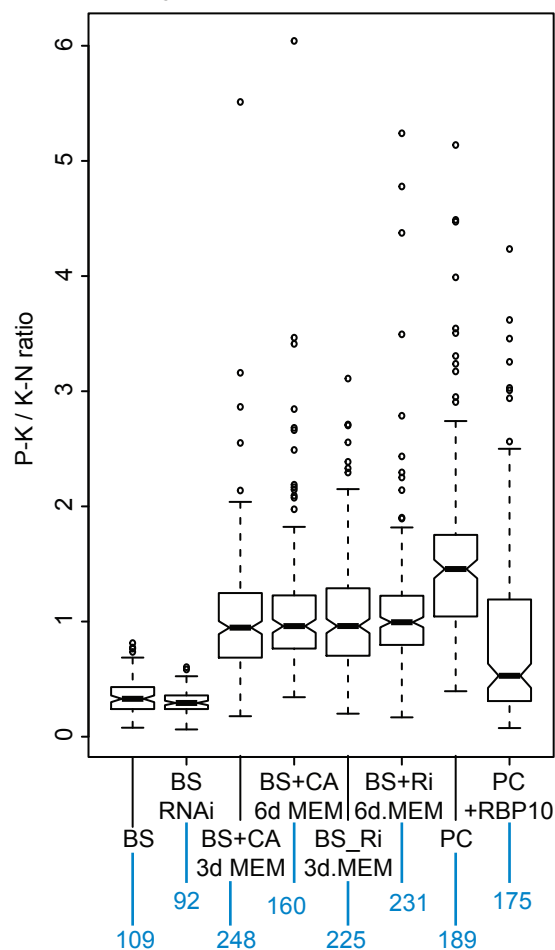**B**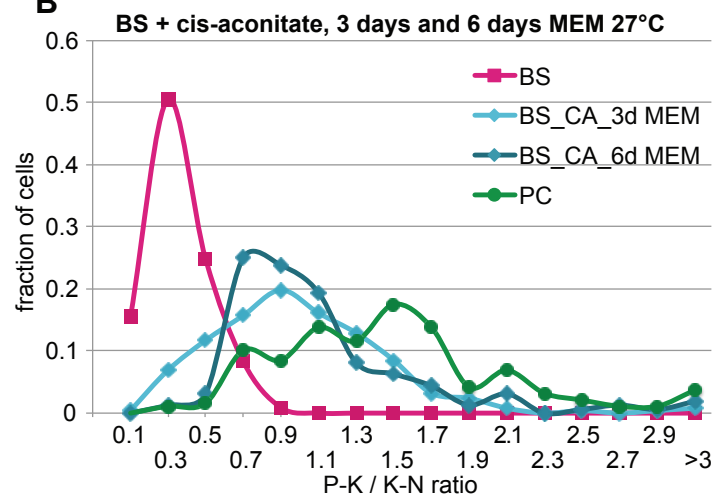**C**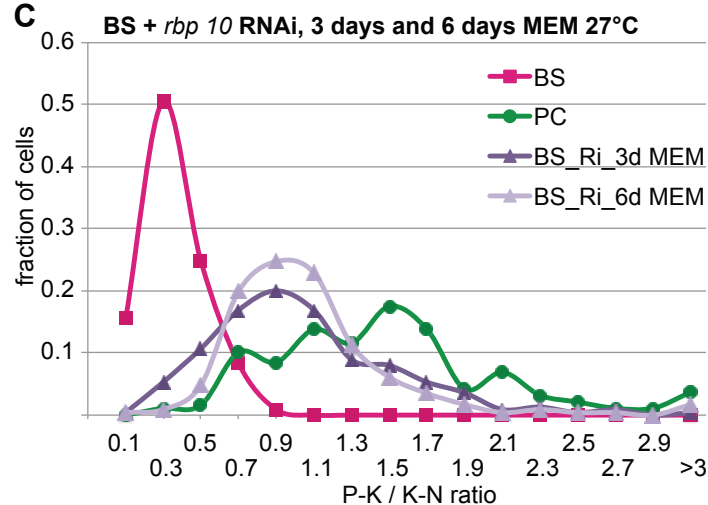**D**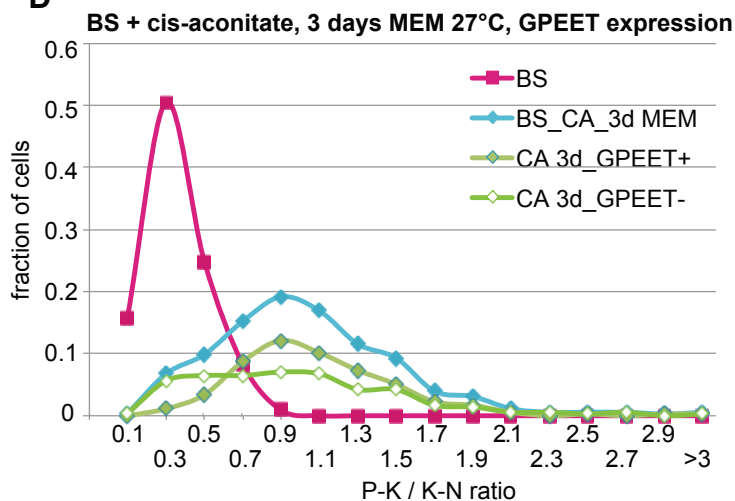**E**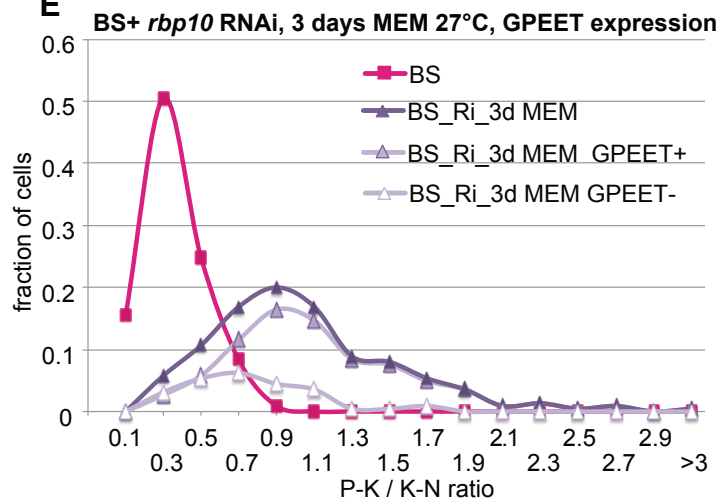**F**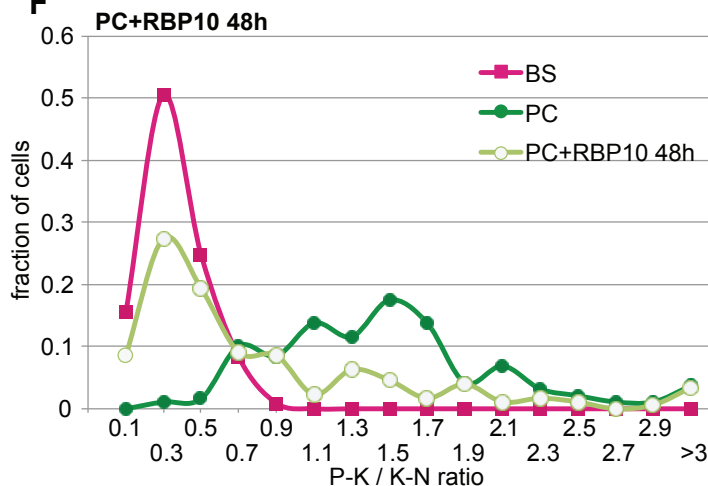**G**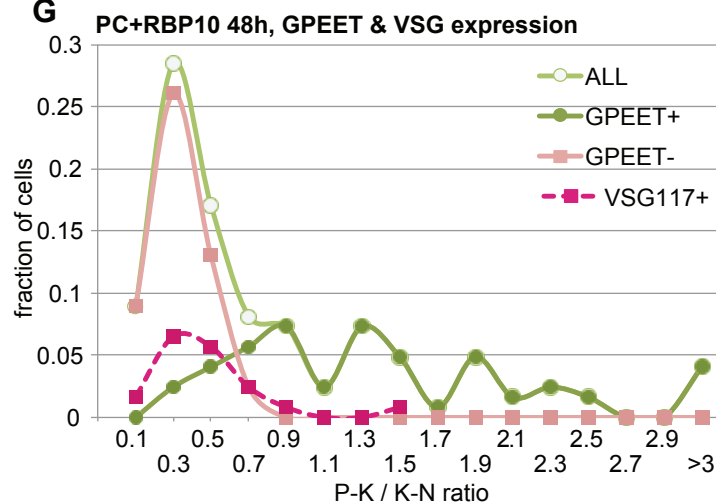

Supplement: S8 Fig — Cells were treated as indicated, fixed, and stained for DNA (cyan), EP-procyclin, phopho-GPEET procyclin, or VSG. (magenta). The posterior-kinetoplast and kinetoplast-nucleus distances were measured in Image J and the ratios calculated. A. Summary of all results. This is the same as Fig 5H, but all of the outliers are included, and the numbers of cells analysed are below each lane. BS—normal bloodstream forms; PC- normal procyclic forms; CA - 17h cis-aconitate pretreatment; RNAi or Ri; 17h rbp10 induction; +RBP10—induced expression or RBP10 for 2 days. B. Cell distributions after cis-aconitate-induced differentiation. The x axis shows the P-K/K-N ration, and y-axis shows the percentage of cells with that ratio. Controls are bloodstream forms (magenta) and established procyclic forms (green). The other lines are for 3 and 6 days after transfer to procyclic medium (MEM). C. As B, but with transfer after 17h rbp10 RNAi. D. Phospho-GPEET expression 3 days after cis-aconitate-stimulated differentiation. The cells indicated with the cyan line (BS_CA_3d MEM) were subdivided into GPEET positive and GPEET negative. E. Phospho-GPEET expression 3 days after RNAi-stimulated differentiation; other details as in D. F. Cell distributions in procyclic forms after induced expression of RBP10. G. As (F), but also showing staining with anti-phospho-GPEET and anti-VSG117. Cells with no GPEET have bloodstream-form kinetoplast positions, and a subset of them cross-reacts with anti-VSG117 antibodies. The remainder may express VSGs that do not cross react with the anti-117 antibody. (Note that the plotted "GPEET negative" parasites will include VSG117-positive cells, because the counts were derived from different images. Counting of images of double-stained smears was avoided because of potential bleed from very high signals). (PDF) [file ppat.1006560.s012.pdf]

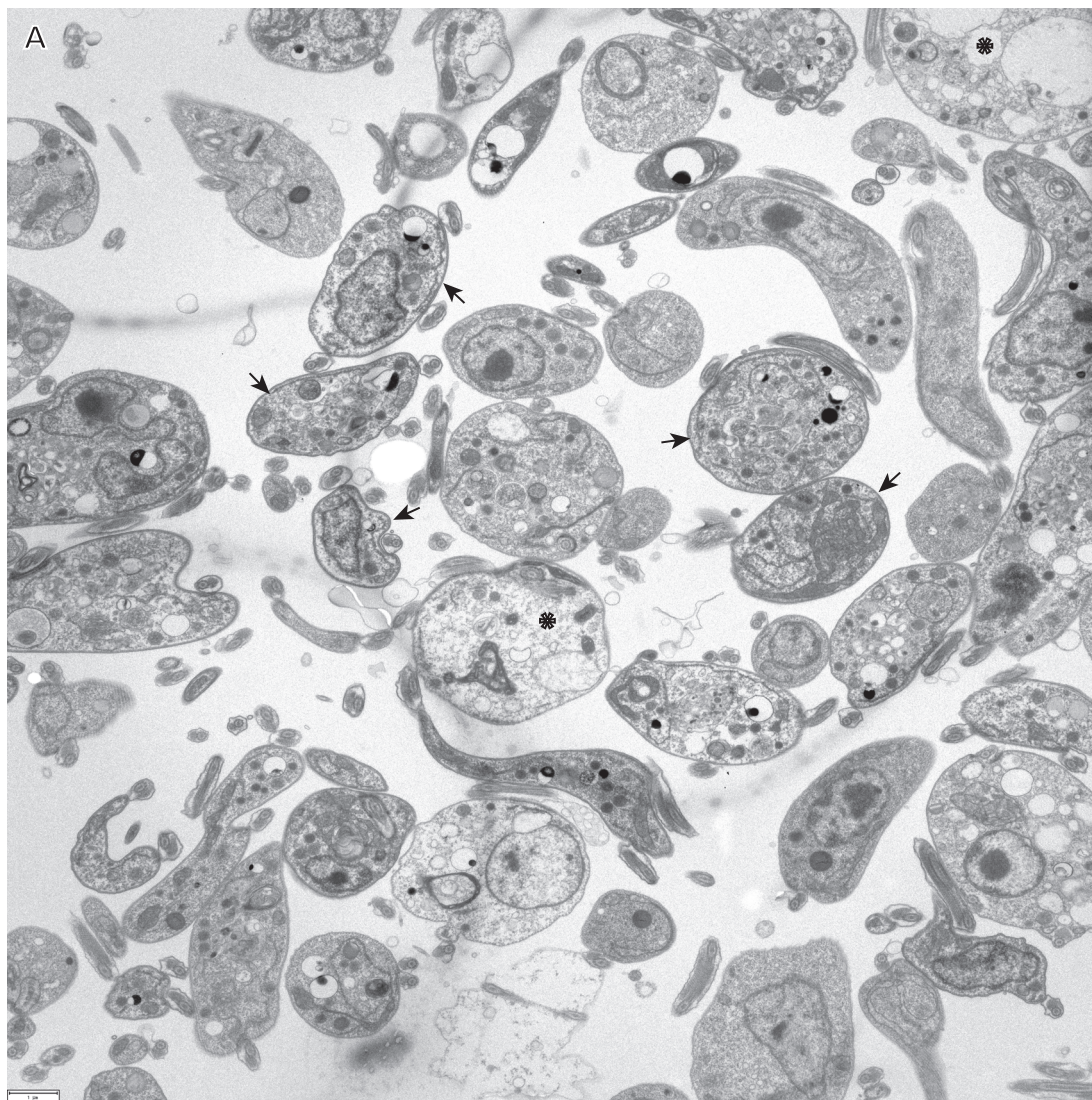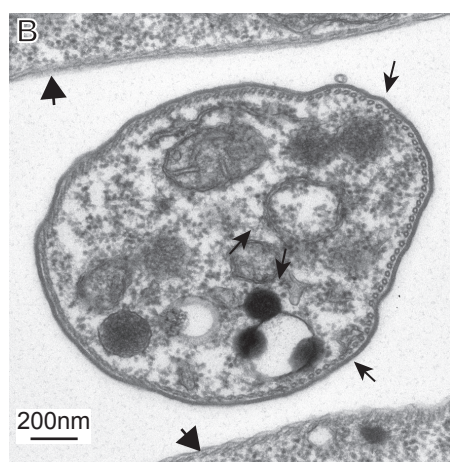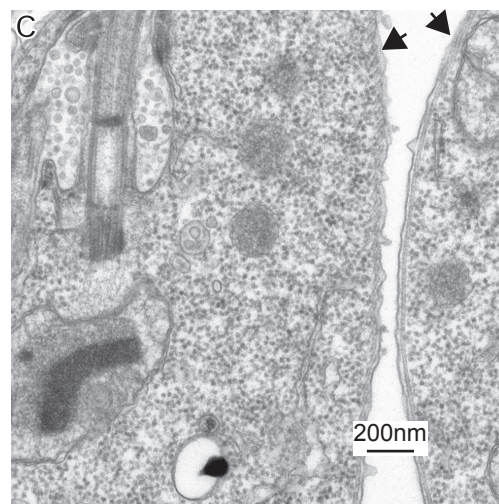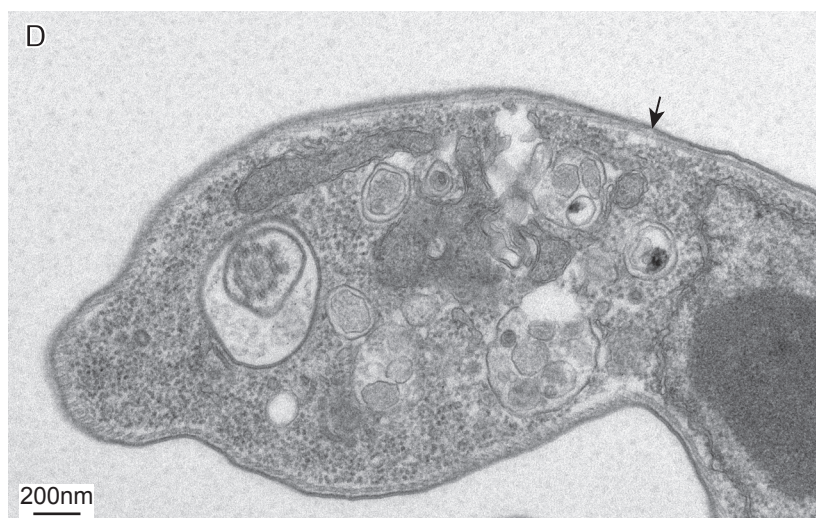

Supplement: S9 Fig — A. A procyclic trypanosome culture 48h after induction of expression of RBP10 expression. VSG coats are quite difficult to distinguish at this magnification but possible candidates are indicated by thin arrows. The asterisks indicate degenerating cells. In some cases plasma membranes look slightly thickened, these could be cells that are in the process of acquiring a coat. Higher-resolution images were used for quantitation; the images here have reduced resolution in order to decrease the file size. B. Close-up of cells with (thin arrows) and without (thick arrows) VSG coats from a different field. C. Procyclic forms from a normally-growing culture. These cells seem to have membrane blebs. D. A bloodstream-form trypanosome from a normal culture. (PDF) [file ppat.1006560.s013.pdf]
