# Supplementary material for: Expression of the RNA-binding protein RBP10 promotes the bloodstream-form differentiation state in Trypanosoma brucei
Source: PLoS Pathog. 2017 Aug 11;13(8):e1006560. doi: 10.1371/journal.ppat.1006560 (PMC5568443; doi:10.1371/journal.ppat.1006560)
Supplement: S6 Fig — A. Scatter plot comparing the effect of rbp10 RNAi on total RNA (x axis) with the effect on polysomal RNA (y axis) for mRNAs that were at least 3x enriched according to DeSeq; all P-values were less than 8E-5. The black line is the regression line and the red and green lines show the 95% confidence limits for the data. The blue shadow encloses total mRNAs that were less than 1.5x affected, and the pink shadow encloses polysomal RNAs that were less than 1.5x affected. The cyan line is perfect correlation. The box beneath the graph lists relevant TritrypDB accession numbers. The gene numbers on the plot are also accession numbers, with "Tb927." removed. B. As (B), but here the y axis shows the effect of RNAi on the percentage of the mRNA in polysomes. (PDF) [file ppat.1006560.s010.pdf]

A

B

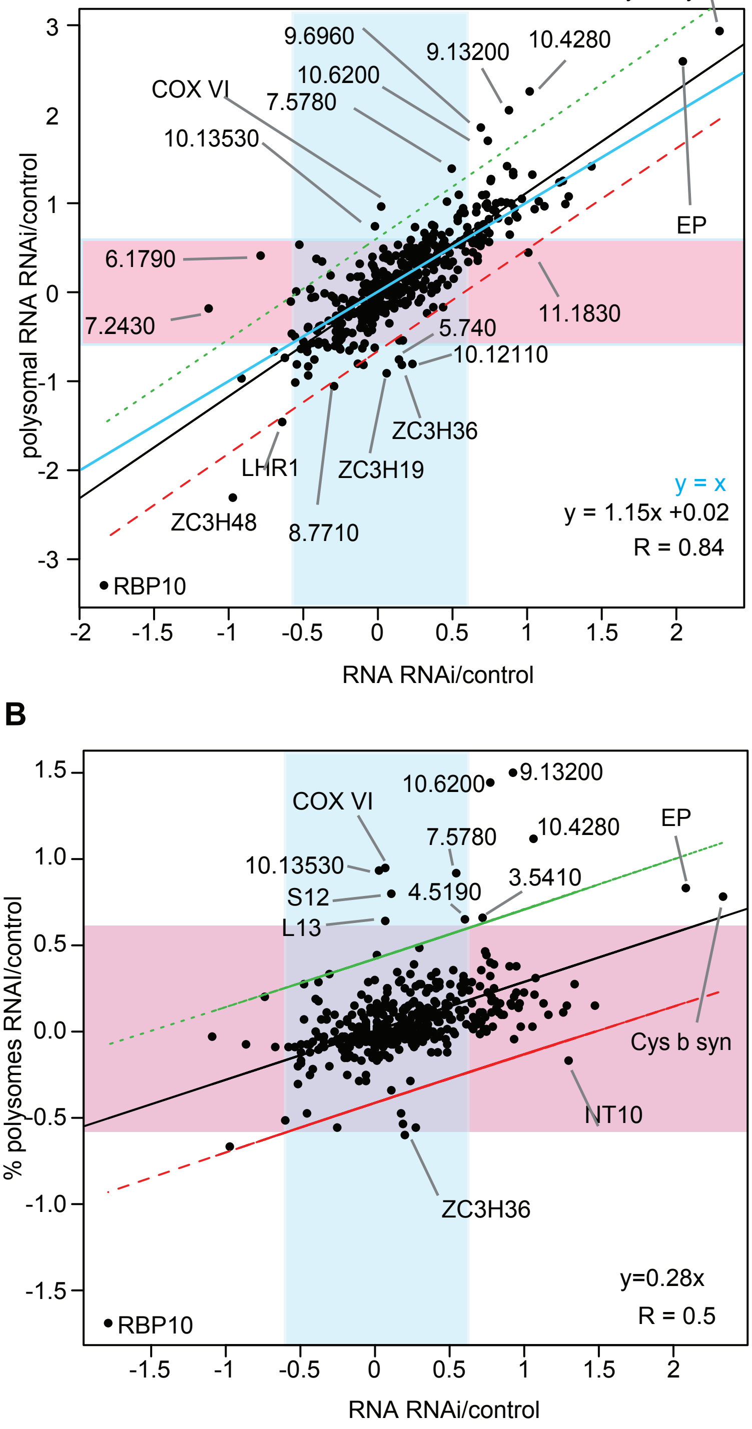

Tb927.10.10250	EP procyclin
Tb11.02.5400	cystathionine beta-synthase
Tb927.10.280	Cytochrome oxidase subunit VI (COXVI)
Tb927.10.4280	Cytochrome bd ubiquinol oxidase subuni
Tb927.10.12760	ZC3H36
Tb927.9.7470	purine nucleoside transporter NT10
Tb927.3.3310	60S ribosomal protein L13
Tb927.10.8430	40S ribosomal protein S12
Tb927.9.10280	ZC3H48
Tb927.7.2580	ZC3H19
Tb927.8.6010	Heme uptake protein LHR1
