# Supplementary material for: Expression of the RNA-binding protein RBP10 promotes the bloodstream-form differentiation state in Trypanosoma brucei
Source: PLoS Pathog. 2017 Aug 11;13(8):e1006560. doi: 10.1371/journal.ppat.1006560 (PMC5568443; doi:10.1371/journal.ppat.1006560)
Supplement: S7 Fig — Cells were fixed with fomaldehyde, permeabilized with triton x-100, and stained using a polyclonal antibody to phosphglycerate kinase (PGK). The grey-scale panels show PGK alone and the differential interference contrast panels show DNA in cyan and PGK in magenta. A. Procyclic forms. B. Bloodstream forms. C. Bloodstream forms after incubation with cis aconitate for 17h at 27°C. D. Bloodstream forms with 17h rbp10 RNAi followed by culture for 3 days under procyclic-form culture conditions. The selection shows one procyclic-like trypanosome (left) and one which still has bloodstream-form morphology (right). E. Procyclic forms after 2 days induction of expression of RBP10-myc. Cells with terminal kinetoplasts are labelled "b" and cells with more procyclic morphology are labelled "p". Cells that appear to be dividing are indicated with asterisks. A very abnormal cell is indicated with "?". Cells if unclear status are not labelled. (PDF) [file ppat.1006560.s011.pdf]

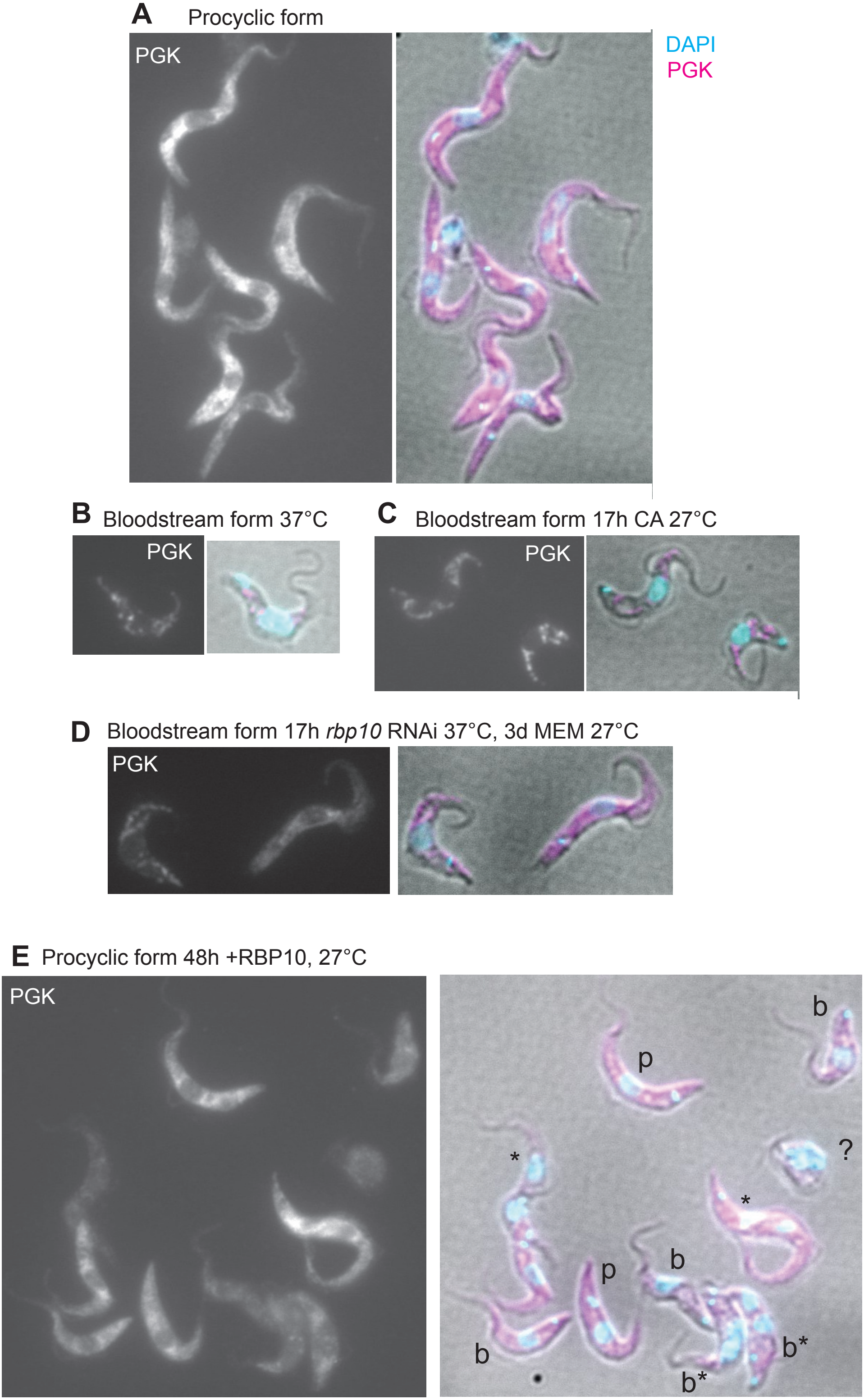
A Procyclic form
PGK
DAPI
PGK
B Bloodstream form 37°C
PGK
C Bloodstream form 17h CA 27°C
PGK
D Bloodstream form 17h *rbp10* RNAi 37°C, 3d MEM 27°C
PGK
E Procyclic form 48h +RBP10, 27°C
PGK
b
p
?
*
*
b
p
b
b*
b*
